# Determinants and perception of health insurance participation among healthcare providers in Nigeria: A mixed-methods study

**DOI:** 10.1371/journal.pone.0255206

**Published:** 2021-08-04

**Authors:** Hezekiah Olayinka Shobiye, Ibironke Dada, Njide Ndili, Emmanuella Zamba, Frank Feeley, Tobias Rinke de Wit

**Affiliations:** 1 John F. Kennedy School of Government, Harvard University, Cambridge, MA, United States of America; 2 Department of Global Health, Boston University School of Public Health, Boston, MA, United States of America; 3 PharmAccess Foundation, Lagos, Nigeria; 4 Lagos State Health Management Agency, Lagos, Nigeria; 5 PharmAccess Group and Joep Lange Institute, Amsterdam, Netherlands; 6 Amsterdam Institute of Global Health and Development, Amsterdam, Netherlands; International Medical University, MALAYSIA

## Abstract

**Background:**

To accelerate universal health coverage, Nigeria’s National Health Insurance Scheme (NHIS) decentralized the implementation of government health insurance to the individual states in 2014. Lagos is one of the states that passed a State Health Insurance Scheme into law, in order to expand the benefits of health insurance beyond the few residents enrolled in community-based health insurance programs, commercial private health insurance plans or the NHIS. Public and private healthcare providers are a critical component of the Lagos State Health Scheme (LSHS) rollout. This study explored the determinants and perception of provider participation in health insurance programs including the LSHS.

**Methods:**

This study used a mixed-methods cross sectional design. Quantitative data were collected from 60 healthcare facilities representatively sampled from 6 Local Government Areas in Lagos state. For the qualitative data, providers were interviewed using structured questionnaires on selected characteristics of each health facility in addition to the managers’ opinions about the challenges and benefits of insurance participation, capacity pressure, resource availability and financial management consequences.

**Results:**

A higher proportion of provider facilities participating in insurance relative to non-participating facilities were larger with mid to (very) high patient volume, workforce, and longer years of operation. In addition, a greater proportion of private facilities compared to public facilities participated in insurance. Furthermore, a higher proportion of secondary and tertiary facilities relative to primary facilities participated in insurance. Lastly, increase in patient volume and revenue were motivating factors for provider facilities to participate in insurance, while low tariffs, delay and denial of payments, and patients’ unrealistic expectations were mentioned as inhibiting factors.

**Conclusion:**

For the Lagos state and other government insurance schemes in developing countries to be successful, effective contracting and quality assurance of healthcare providers are essential. The health facilities indicated that these would require adequate and regular provider payment, investments in infrastructure upgrades and educating the public about insurance benefit plans and service expectations.

## Introduction

Nigeria’s National Health Insurance Scheme (NHIS), established in 1999 but operational since 2005, has been faced with numerous challenges including the failure to mandate enrollment for the entire population and the consequent lack of adoption at the state government level [1]. Thus, only about 5 million Nigerians, representing 3% of the population, have insurance coverage through the NHIS and these are mostly members of the formal sector, particularly federal civil servants [2]. To that end, in 2014, the NHIS decentralized the implementation of the country’s social health insurance program to the states in the quest to accelerate progress towards universal health coverage (UHC) [3].

In 2015, Lagos state was the first of the 36 states and Federal Capital Territory to pass the State-Based Health Insurance Scheme (SHIS) into law [4]. The Lagos State Health Scheme (LSHS) was designed as a mandatory health insurance program to enroll all Lagos state residents and reduce the financial burden of obtaining care while improving access to quality care.

Public and private healthcare providers are crucial to the rollout and success of this insurance program. Their ability to cooperate and participate in insurance importantly determines the extent and quality of care that population members would access [5]. However, there is little understanding in Nigeria and in many other low- and middle-income countries (LMICs) on how to effectively engage providers, what type of providers join or choose not to join an insurance program, and why they do so [6].

Prior to the passage of the LSHS, Lagos state rolled out three community-based health insurance (CBHI) schemes, which altogether enrolled close to 40,000 community members [7]. A program assessment done in 2010 reported some positive outcomes relating to quality of maternal and neonatal services and patient satisfaction [8]. However, the assessment also noted high turnover rate among participating members and healthcare providers. In addition to the CBHI, Lagos state also has residents registered with the NHIS and private health insurance plans through Health Maintenance Organizations (HMOs) [9]. For the NHIS enrollees in Lagos, actual numbers are unknown, although they are few, mostly formal sector workers in the federal civil service, and many registered patients have increasingly complained about the poor quality of care received [9]. In the commercial market, the number of Lagos residents with private health insurance is also unknown, though it is accessible primarily to the wealthy and for some employees largely through employer benefits [10]. In a nationwide poll, about 2% of Nigerians have private health insurance, many of which live in Lagos [11].

HMOs are a major part of health insurance in Nigeria [12]. For the NHIS, they serve as an intermediary between the providers and the insurance scheme [13]. HMOs receive payments from the NHIS and are supposed to disburse funds to providers based on the volume of insured patients in the health facility: capitation for primary care and fee-for-service (FFS) for quantities of secondary level care services provided to an insured patient [3]. For private insurance, the HMOs collect premium from individuals, companies or groups and negotiate with facilities to provide services to the individuals at an agreed service rate [12]. According to the NHIS online directory, there are about 60 HMOs accredited nationwide by the NHIS [14].

On the provider side, there have been complaints in the public media suggesting widespread dissatisfaction about the low tariffs, delayed payments, increased administrative burden and the losses incurred from participating in government and private insurance plans [15]. Some, as a result, have discontinued participation or have not been motivated to participate in any insurance program. For example, a study in 2014, examined the uptake of NHIS among 180 private healthcare providers in Lagos state and found that only 61% of the respondents accepted NHIS patients [16]. In addition, half of the respondents were dissatisfied with the operations of the scheme citing reasons such as inability to reimburse payment for services and subsequent losses that were incurred.

### Theoretical framework

The two-sided economic theory by Sloan and colleagues can be used in the analysis of health insurance markets and provider response to insurance [17]. This theory suggests two markets in which providers sell their services. The first is the private market where providers are the price setters, determining the amount to charge for services offered to each patient paying out of pocket or via private insurance. The other is the public market, where providers are price takers, accepting the fee offered by a public program like the Lagos State Health Scheme without charging additional fees to the patients.

**Fig 1:**
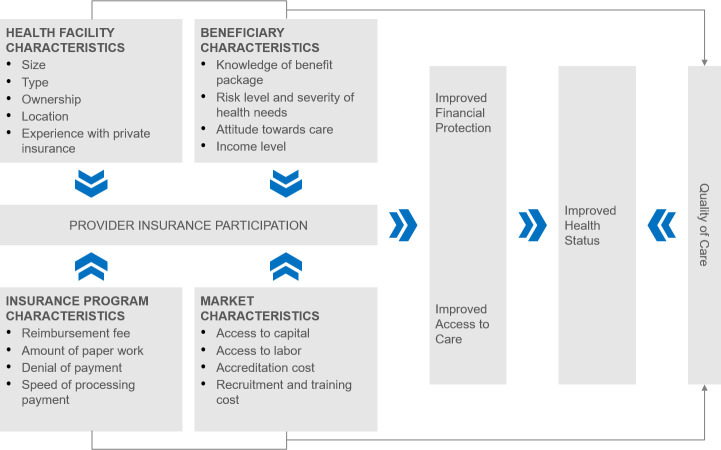
Theoretical framework for provider participation in insurance programs. Source: Authors’ original concept.

Providers can act as imperfect agents of patients, desire to maximize profit, and thus, prefer to serve patients in a market where they can generate more income [18]. This suggests that the extent of provider participation in any program would be determined by their assessment of the benefits and costs of participating in the program. Studies done in countries like the United States have identified several factors that may influence a provider’s assessment of the benefits and costs of participating in an insurance program [5, 19–25]. For this paper, each factor has been categorized under one of four characteristics as described in Fig 1: insurance program characteristics, beneficiary characteristics, health facility characteristics and the market characteristics.

Under the insurance program characteristics, the reimbursement fee, amount of paperwork, speed of processing payment, inappropriate claims reductions and denials can influence provider participation [26]. For example, studies have shown that providers respond better to insurance programs that have high reimbursement fees, lower amount of paper work, short processing time for reimbursements and lower probability of reducing and denying claims [5, 19, 22, 24, 25].

Under the beneficiary characteristics, the difficulty of patients defined by health need or risk level of beneficiaries, ability of patients to understand insurance benefit package and income level of patients may influence provider participation in an insurance program [26]. Providers may prefer to have low-risk patients since they tend to utilize less care compared to high-risk patients. In addition, patients who understand the insurance benefit package are less likely to have a poor attitude towards their providers, have unrealistic expectations of the care they receive and are likely to be more compliant with their treatments and medications [19–21]. Furthermore, providers may prefer patients with high income who can pay out of pocket and recover any insurance benefits later. This payment process eliminates any administrative burden on the provider in filing and collecting insurance claims [26].

The characteristics of a health facility may also influence provider participation in an insurance program [26]. For example, providers who offer care as specialists may be less likely to participate in an insurance program because of their advanced training and need to command higher fees compared to providers who are generalists and offer care at the primary level. In addition, the location of the health facility may influence provider participation. Health facilities located in a low-income area, rural area, areas with few health facilities and a low concentration of beneficiaries may be more likely to participate in an insurance program [19, 21]. Furthermore, the size of the health facility may influence provider participation. Health facilities with high number of beds and high volume of patients capable of paying out-of-pocket may not be incentivized to participate in a government insurance program. Similarly, health facilities which accept and have a high volume of privately insured patients may not be willing to participate in a government insurance program [19–23, 25].

Finally, the characteristics of the market that affect the productivity of a provider such as the ease for providers to access capital and labor in the market, and the associated costs of accreditation, recruiting and training of staff, may influence provider participation in an insurance program [26]. For example, the easier it is for a provider to access capital, the more likely it can improve its infrastructure, recruit and train staff needed, achieve accreditation, and increase revenue from additional insured patients. It is more likely in the end that it would participate in an insurance program [21, 25].

Using the theoretical framework as a guide, this study aims to answer three main research questions. First, what are the characteristics of provider facilities that do, and do not, participate in insurance programs? Second, what are the drivers and barriers to provider participation in insurance? Lastly, what opportunities and challenges do facility managers anticipate from the rollout of the LSHS? The findings from this study will provide an evidence-based guide to Lagos state and governments of many other low- and middle-income economies on how to contract with providers and expand their participation in health insurance scheme in a phased manner.

## Methods

### Study setting

**Fig 2:**
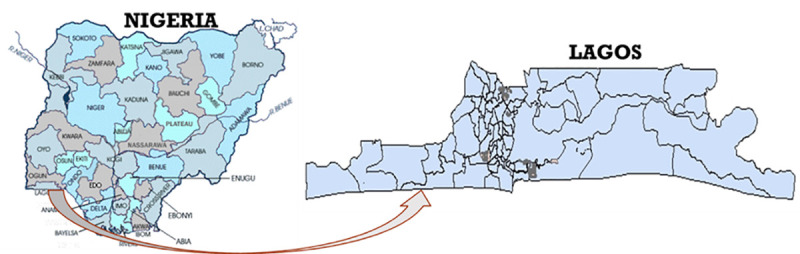
Map showing Lagos state in Nigeria.

This study was conducted in Lagos state, the economic and business hub of Nigeria with an approximate population size of 24 million people. As shown in Fig 2, it is administratively divided into 20 Local Government Areas (LGAs) [9]. Relative to other states in the country, it has one of the highest numbers of health facilities. According to the Healthcare Facilities Monitoring and Accreditation Agency (HEFAMAA) in 2017, there are 26 General Hospitals and 256 Primary Health Centers managed by the state government, and over 2,800 Private Hospitals, Specialist Clinics and Laboratories in Lagos [27].

### Sample size and selection of health facilities

Health facilities for this study were sampled from the health facilities in the database of the Lagos State Healthcare Facilities Monitoring and Accreditation Agency (HEFAMAA) [28]. The database contained 3,376 health facilities categorized into public and private facilities including faith-based, laboratories, diagnostic centers, dental clinics, physiotherapy centers, special clinics for eyes and skin, and company-supported facilities. This list of facilities was combined with a list of NHIS Lagos accredited primary and secondary facilities accessed online from NHIS’s website. See S1 and S2 Appendices. Within this combined list, facilities categorized as Company, Army, Navy, Air force or Police-owned were removed as they were not relevant for the general population. In addition, dental clinics, standalone laboratories, special clinics that only provided services for the eyes, bones, skin, ears, nose or throat were removed from the list. Furthermore, duplicates and facilities with no information on location were removed. Following this process, 2,726 health facilities remained in the database.

LGAs were selected to get a range of population and health financing characteristics. From the 20 LGAs in Lagos state, 6 LGAs were purposively selected for the study. The purposive selection considered the differences in the size of the LGAs, urbanization, number of health facilities, likelihood of insurance participation, and income level of residents. These are Alimosho, Ibeju-Lekki, Oshodi-Isolo, Eti-Osa, Mushin and Ikeja LGAs. From the 6 LGAs, there were 1,142 health facilities in the combined database. In each LGA, health facilities were selected using a multi-stage stratified sampling technique [29]. Strata were created according to ownership, facility type, and NHIS participation status. From each stratum, health facilities were randomly selected, and in cases where there was only one facility available in the stratum, that facility was automatically selected. In some other cases and for some LGAs without a facility available for a stratum, no facility was selected. As a result, there was a variation in the number of facilities selected per LGA, ranging from a minimum of 8 to a maximum of 13 facilities. Furthermore, in case provider facilities initially selected did not respond, secondary options were drawn from the database.

In the end, 60 facilities were selected. This number represented 5% of the total number of health facilities in the six LGAs. The rationale for this sampling size was based on several considerations. First, the sample size took into consideration the objective of the data collection exercise, the timeframe, and resources available. The sample size is also consistent with what is adequate in the literature [30]. As a result, the sample size was large enough to gather substantial and relevant responses from the providers that were interviewed [31–33].

### Tools for data collection

Data collection and interviews for all the facilities were conducted between December 2017 and February 2018. Quantitative and qualitative data were collected at the selected health facilities to get an understanding of the facility’s capacity and perception of insurance participation. For the quantitative part, information was collected on the health facility characteristics including number of years of operation, workforce, HMO affiliation, bed size, service volume, and finances. However, not every manager was transparent with sharing the facility’s financial information. There was also no feasible way to double check the reliability of all the financial information collected even though some facility managers gave us access directly to their financial statements through their account officers. As a result, we have omitted the financial variable from our analysis and results. For the qualitative part, 60 health facility managers were interviewed to get their perspectives on insurance participation, capacity pressure, resource availability and the Lagos State Health Scheme. The average length of each interview was one hour. The interviews were conducted in English Language, the official language of communication.

The principal investigator (HOS) supported by two trained research assistants (RAs) conducted the field data collection. The RAs were both knowledgeable about healthcare delivery and survey administration, and both had a master’s degree. Both RAs were trained by the principal investigator on health research ethics, the procedure for data collection, and administration of informed consent. To ensure standardization in the data collection process, the principal investigator and RAs conducted the first two data collection and interviews together. Following the first two visits, each RA was randomly allocated to collect data from health facilities in each LGA.

The survey instruments for both quantitative and qualitative were paper-based tools. See S3 Appendix. The questions were derived from a review of the literature on insurance participation and discussions with healthcare providers and health financing experts. Prior to data collection, the questionnaire was pretested for feedback on clarity, ease of completion and inclusion of the most relevant issues to insurance participation. This pretest was conducted in six health facilities in Lagos. Based on the feedback from the pretest, minor changes were made to the questionnaire.

### Data management and analysis

We used a convergent parallel mixed-methods design to collect, analyze and interpret both quantitative and qualitative data [34]. Following data collection, the responses from the provider questionnaire were transcribed from paper and uploaded into an electronic database system. From there, they were screened and converted into Microsoft Excel. The quantitative part of the data was analyzed using Excel spreadsheet and Stata Statistical Software while the qualitative part of the data was coded and analyzed using NVivo Software.

To analyze the quantitative data, descriptive statistics such as percentages, mean and median were used to summarize the demographic characteristics of the health facility managers. Similar descriptive statistics were used to describe the general characteristics of the health facilities and to examine any difference in insurance participation status based on location, ownership, level, type, volume of services provided, bed size, workforce, years of operation, and HMO affiliation.

To understand the volume of service delivery in each facility, data on number of inpatients, outpatients, deliveries, and laboratory tests were collected for the years 2015 and 2016. Some of the facilities did not offer inpatient, delivery or laboratory services. However, all the facilities offered outpatient services. As a result, the number of outpatient services for each facility in 2016 was recoded and a new category of service delivery volume from very low to very high was created. Facilities with annual outpatient services less than or equal to 5,000 at the end of 2016 was labelled “very low” volume, less than or equal to 15,000 as “low” volume, less than or equal to 50,000 as “mid” volume, less than or equal to 100,000 as “high” volume and greater than 100,000 as “very high” volume.

For the qualitative data, framework analysis, a qualitative research method that allows for theme-based analysis and is better suited for analyzing the specific questions facility managers were asked was used for the data analysis [35]. In the end, results from the qualitative data analysis were triangulated with those from the quantitative analysis for a holistic picture, and to generate relevant policy recommendations.

The research protocol was approved by the Institutional Review Boards at the Nigerian Institute of Medical Research and Boston University Medical School. All respondents provided written informed consent.

## Results

### Demographic characteristics

Table 1 summarizes the demographic characteristics of the 60 facility managers that were interviewed. Based on highest qualifications, 25 had a medical degree, including those who had completed advanced medical training in a particular specialty. 13 had a master’s degree (11 of which obtained a medical degree before pursuing a master’s degree), 13 had a bachelor’s degree, 7 had a diploma, 1 had a PhD and another a high school certificate. In terms of profession, 36 of the managers were medical doctors, 15 were nurses/midwives and 9 were non-medical professionals. Non-medical professionals included mostly hospital or insurance administrators, followed by community health workers and then a mechanical engineer. In terms of gender, slightly more of the managers interviewed were male [32] than female [28]. The average age of the managers was 50 and average number of years spent as a manager was 10.

**Table 1:** Demographic characteristics of facility managers.

	Freq (n)		Percentage (%)	
**Qualifications**				
High School Certificate	1		2	
Diploma	7		12	
Bachelors	13		22	
Medical Degree	25		42	
Masters	13		22	
PhD	1		2	
**Profession**				
Medical Doctor	36		60	
Nurse/Midwife	15		25	
Non-medical professional	9		15	
**Gender**				
Female	28		47	
Male	32		53	
	**Min**	**Max**	**Mean**	**Median**
**Age**	27	82	50	51
**Years spent as a manager**	0.3	36	10	5

### Characteristics of provider facilities that do, and do not, participate in insurance programs

Table 2 describes the characteristics of the health facilities. Out of the 60 health facilities, 39 were privately owned, 19 were public facilities and 2 were faith-based facilities. In addition, 13 of the facilities were health clinics or posts, 10 were maternity homes, 27 were medical centers, 9 were specialist hospitals and 1, a teaching hospital. In terms of location, 33 of the facilities were located in an urban area, 23 in a peri-urban area, while 4 of the facilities were located in a rural area. 31 out of the 60 facilities provided care at the secondary level, though these facilities also provided primary care services (Higher level health facilities–secondary and tertiary–also provide primary care and receive primary care capitation under the National Health Insurance Scheme); 25 operated only at the primary care level while 4 of the facilities provided tertiary and lower levels of care. Each one of the facilities had at least one bed, with 35 of them having 10 beds or less, 14 between 11 to 20 beds and 11 facilities with greater than 20 beds. 24 of the facilities had been in operation for over 20 years, 17 between 10 and 20 years, 13 between 5 and 10 years, and 6 facilities between 1 and 5 years.

**Table 2:** Health facility characteristics by insurance participation.

	Accepts Insurance, n (%)	No Insurance, n (%)	Total
**Total**	**36 (60)**	**24 (40)**	**60**
**LGA**			
Alimosho	9 (69)	4 (31)	13
Eti-Osa	7 (70)	3 (30)	10
Ibeju Lekki	6 (75)	2 (25)	8
Ikeja	5 (50)	5 (50)	10
Mushin	5 (56)	4 (44)	9
Oshodi/Isolo	6 (60)	4 (40)	10
**OWNERSHIP**			
Faith-based	1 (50)	1 (50)	2
Private	26 (67)	13 (33)	39
Public	9 (47)	10 (53)	19
**TYPE**			
Health Clinic/Post	3 (23)	10 (77)	13
Maternity Home	3 (30)	7 (70)	10
Medical Center/Hospital	20 (74)	7 (26)	27
Specialist Hospital	9 (100)	0 (0)	9
Teaching Hospital	1 (100)	0 (0)	1
**LEVEL**			
Primary	8 (32)	17 (68)	25
Secondary	24 (77)	7 (23)	31
Tertiary	4 (100)	0 (0)	4
**LOCATION**			
Urban	19 (58)	14 (42)	33
Peri-Urban	14 (61)	9 (39)	23
Rural	3 (75)	1 (25)	4
**YEARS IN OPERATION**			
1–5 Years	2 (33)	4 (67)	6
5.1–10 Years	4 (31)	9 (69)	13
10.1–20 Years	12 (71)	5 (29)	17
> 20 Years	18 (75)	6 (25)	24
**BED SIZE**			
10 Beds and less	13 (37)	22 (63)	35
11–20 Beds	12 (86)	2 (14)	14
Greater than 20 Beds	11 (100)	0 (0)	11
**SERVICE VOLUME**			
Very low	19 (48)	21 (52)	40
Low	8 (73)	3 (27)	11
Mid	4 (100)	0 (0)	4
High	2 (100)	0 (0)	2
Very high	3 (100)	0 (0)	3

In all, 36 facilities accepted one or more type of health insurance: private health insurance (PHI), national health insurance scheme (NHIS) or community-based health insurance (CBHI). Based on facility ownership, 1 out of the 2 faith-based facilities, 26 out of 39 private facilities, and 9 out of 19 public facilities participated in an insurance program. Over half (20) of the facilities accepting insurance are medical centers or hospitals, representing 74% of all medical centers/hospitals sampled. All of the 9 specialist hospitals and the 1 teaching hospital participated in an insurance program. 77% of all health clinics/posts and 70% of all maternity homes visited did not accept insurance. 8 out of 25 facilities providing only primary care accepted insurance, while 24 out of 31 at the secondary level, and all the facilities [4] at the tertiary level accepted insurance.

In terms of location, 19 (58%) of the facilities in the urban area, 14 (61%) of the peri-urban facilities and 3 (75%) of the rural facilities visited accepted one or more type of insurance. Per years of operation, 18 (75%) of the facilities that have been in operation for more than 20 years participated in an insurance program, with the proportion of participating facilities decreased as the years of operation reduced. Only 2 (33%) of the facilities between 1 and 5 years in operation participated in an insurance program. All the facilities with more than 20 beds, participated in an insurance program, while 12 (86%) and 13 (37%) of facilities with 11 to 20 beds and 10 beds and less, participated in an insurance program. Similarly, for service delivery volume, all facilities with mid, high and very high service volume participated in an insurance program, while only 8 (73%) and 19 (48%) of the facilities with low and very low service volume respectively participated in an insurance program.

In Table 3, on average, facilities that accepted insurance have a larger workforce than facilities not participating in any insurance program. In private facilities, on average there were 3 times more doctors, two times more nurses and midwives, and 5 times more non-medical workers in the facilities accepting health insurance compared to those without health insurance. In the public facilities, on average, there were 60 times more doctors, 28 times more nurses and midwives, and 14 times more non-medical workers in the facilities accepting health insurance compared to those who do not. The wide difference between the average and median number of workers observed in the public facilities is due to the presence of a large teaching hospital. By facility type, the teaching hospital on the average has more health workforce than any other type of hospital: the specialist hospitals, medical centers, health clinics and maternity homes.

**Table 3:** Workforce–full time and part time, by facility ownership and insurance status.

Type of Health Workforce	Faith-based (n = 2)		Private (n = 39)				Public (n = 19)			
	No insurance (n = 1)	Insurance (n = 1)	No insurance (n = 13)		Insurance (n = 26)		No insurance (n = 10)		Insurance (n = 9)	
	Mean	Mean	Mean	Median	Mean	Median	Mean	Median	Mean	Median
Medical Doctor	3	4	2.3	2	6.8	4.5	1.4	1	83.6	29
Nurse/Midwife	6	10	3.2	3	7.3	5.5	4.6	3	128.8	62
Auxiliary Nurse	5	0	2.8	2	3.5	3	0	0	0	0
Community Health Worker	2	4	0.5	0	1	1	3.5	2	1.1	0
Pharmacist	0	1	0	0	0.3	0	0.1	0	12.3	11
Pharmacist Technician	2	1	0	0	1.2	1	0.9	1	6.3	5
Pharmacist Assistant	1	0	0.1	0	0.2	0	0.1	0	0.9	0
Laboratory Technician	0	2	0.2	0	1.3	1	1	0.5	4.6	3
Laboratory Scientist	1	2	0	0	0.9	0.5	0.2	0	9	5
Non-medical & Others	4	13	2.8	3	14	8.5	10.9	6.5	149	86

In terms of HMO affiliation, the maximum number of HMOs dealing with a single facility was 58 while the average number was 18 and the minimum was 0. Among the public facilities, the average number of HMOs was 12, while it was 20 for the private facilities. There were no HMOs dealing with health clinics/posts and only 1 with the maternity homes. For the medical centers/hospitals, the average number of HMOs was 16, with a minimum of 0 and a maximum of 52, while for the specialist hospitals, the average number of HMOs was 32, with a minimum of 10 and a maximum of 58. In the teaching hospital, there were 33 affiliated HMOs. See Table 4.

**Table 4:** Distribution of HMOs according to facility ownership and type.

	Min	Max	Mean	Median	Freq (n)
All facilities accepting insurance	0	58	18	14	36
**Ownership**					
Public	0	33	12	5	9
Private	0	58	20	16	26
Faith-based	2	2	2	2	1
**Type**					
Health Clinic/Post	0	0	0	0	3
Maternity Home	0	1	0	0	3
Medical Center/Hospital	0	52	16	12	20
Specialist Hospital	10	58	32	29	9
Teaching Hospital	33	33	33	33	1

### Drivers and barriers to provider participation in insurance

On the drivers to provider participation, the analysis of the data revealed facility-level and population-level themes associated with insurance benefits. At the facility level, majority of the managers described the top benefit of insurance participation as increase in patient volume. This was followed by an increase in revenue and an increased opportunity to upgrade facility infrastructure.

“*The health facility is sure of revenue at the end of the month*. *Health facility can plan and budget ahead for consumables because of constant inflow*… *Increase in client flow helps to ensure that the hospital is in profit; helped with the infrastructural upgrade in 2016*.*” (Private Facility Manager)*

*“Increase in number of patients, increase in revenue, gives room for increase in hospital facility.” (Public Facility Manager)*

Population level themes included public and private facility managers’ perception that insurance benefits the communities by making care more accessible and affordable to patients, and consequently improving the lives of community members who would have not been able to access care in the first place.

“*Most important benefit is the facility being able to impact the lives of patients positively by rendering quality care to patients*…*” (Private Facility Manager)*

*“The health insurance scheme is beneficial to the people: it is affordable, cheap and available. People can access care anytime. The benefits allow a father, mother and 4 children to access care.” (Public Facility Manager)*

For the barriers, the recurring themes were associated with the insurance policies, characteristics of the beneficiaries and market influences. The challenge most frequently reported by facility managers was related to the beneficiaries, particularly their unrealistic expectations and poor knowledge of insurance plans.

“*This place is a rural area*, *and so some people don’t understand the health insurance scheme*. *We need mobilization to make people understand the scheme*…*” (Public Facility Manager)*

*“Patients are unwilling to pay for insurance because they did not access care, even among the educated ones that you think would understand the whole policy.” (Public Facility Manager)*

Issues related to the insurance policy followed this and were more common among private than public facilities, with the greatest concern being low tariffs, followed by the delay in processing claims and payments. Other complaints included poor attitude of HMOs, increased paperwork and denial of payments.

“*Capitation fees for patients was too small*, *so we didn’t take them so we took more of companies who could pay*.*" (Private Facility Manager)*

“*The tariff plans can be unfair*, *HMOs are stubborn*, *we commenced plans with the HMO*, *and they have not paid us in 3 months after treating more than 250 patients*.*” (Private Facility Manager)*

*“Sometimes we have provided care and the HMO says it is not covered by them in their plans; slashing of tariffs following submission of claims; challenging to have to document all the processes and paperwork” (Private Facility Manager)*

“*The HMO over promises and we then look like the devil to some of our patients*. *They will not give us code for some services to offer*. *A lot of discrepancies between what the insurance offers and the patients’ expectations*. *As a result*, *there are some things that we cannot do*.*” (Public Facility Manager)*

In addition to the benefits and challenges of insurance participation, facility managers were asked about their perception of profitability from government and private insurance. Concerning the profitability of government insurance, facility managers reacted differently. Among private facilities, over half accepting government insurance felt that government insurance was not profitable. All these facilities have low or very low service volumes. The remaining private facilities felt that government insurance had contributed minimally or adequately to the facility’s profit. All of these were secondary level facilities but with a mix of service volumes from high to very low. For public facilities, majority stated that government insurance had contributed either minimally or adequately to the facility’s revenue. Most of these facilities have service volumes ranging from mid to high levels and were either secondary or tertiary care facilities. Only one public facility manager stated that government insurance had not contributed to the facility’s revenue.

With regards to the profitability of private insurance, all the private facilities felt that the private insurance had contributed minimally or adequately to the health facility’s bottom line profit. Out of the two public facilities accepting private insurance, one stated that it had adequately contributed to the facilities revenue while the other reported no contribution from private insurance to facility’s surplus.

#### Opportunities and challenges that facility managers anticipate from the LSHS

Facility managers were asked about their anticipated opportunities from the LSHS. The key emerging themes can be divided into facility and population level themed opportunities. The top 3 opportunities stated by facility managers included increase in patient volume, increase in revenue and opportunity to invest in facility upgrade. These are benefits at the facility level similar to the top 3 benefits of insurance participation described earlier by facility managers already participating in an insurance program. The population level theme center around facility managers’ anticipating an opportunity to improve access to free care for Lagos residents and a consequent improvement in the community’s health.

In terms of perceived challenges of the LSHS, the emerging themes were generally similar to the challenges with existing insurance programs and can be divided into 3 levels: facility, patient or program policy issues. At the facility level, the facility managers were mostly concerned about monetary rewards and capacity issues. As a result, they largely highlighted issues related to low reimbursement rates, delay in claims processing and payment, low volume of patients and the likelihood for the scheme not to be profitable as potential challenges with the LSHS. In terms of capacity, they described issues related to inadequate workforce, particularly the shortage of available staff skilled in handling insurance claims. Lack of infrastructure and sufficient reliable power supply were also concerns that were reported.

At the policy level, the facility managers highlighted potential challenges like the lack of regulation and transparency in the handling of funds, need for political support at the local government level and inefficient distribution and allocation of enrollees. Lastly, at the patient level, the recurring themes were related to the poor understanding of the scheme and its benefits, unwillingness to participate in the scheme, and the unreasonable cost of premium for the patients.

When the manager’s responses were analyzed by facility ownership, private facilities were mostly concerned with monetary related challenges, while public facilities mostly highlighted capacity related challenges. As shown in Table 5, the top 2 challenges private facilities envisage from the scheme were low reimbursement fees and delay in claims processing and payment. Conversely, the top two challenges for public facilities were inadequate workforce and the lack of infrastructure, drugs and commodities. In addition, at the policy level, the private facility managers were concerned about the role of HMOs in the administration of the scheme, inefficient distribution of enrollees, and politicizing of the scheme. Policy concerns among public facility managers were mostly focused on the funding flow and political support for the insurance scheme at the LGA level. This was very common among facility managers at the primary care level. Primary health facility managers highlighted the current flow of funds from the local government council to the facility as a potential challenge in the expansion and provision of services when the LSHS kicks off eventually. The existing funding flow allows the public authority managing the facility to collect the insurance proceeds, which may not be remitted to the facility to enable it to increase resources to treat additional patients.

“*…If the current allocation system continues the way it is*, *it’ll be a challenge*. *But the Commissioner [for Health] promised that the money will go directly to the facility*. *The local government may not be happy with it*, *so we are not sure how this will operate*.*” (Public Facility Manager)*

**Table 5:** LSHS top challenges perceived by private and public facility managers.

	**Emerging themes for private facility managers**
1	Low reimbursement fees and potential for scheme not to be profitable
2	Delay in claims processing and payment
3	Lack of regulation and transparency in the handling of funds
4	Poor understanding of insurance plans and expectations
5	Inadequate funding of the scheme
6	Low volume of patients
7	Inefficient distribution and allocation of enrollees
8	Unwillingness to participate in the scheme
9	Unfavorable government politics with the scheme
10	HMOs’ role in the administration of the scheme
	**Emerging themes for public facility managers**
1	Inadequate workforce including training
2	Lack of infrastructure, drugs and commodities
3	Lack of power supply
4	Lack of autonomy and decision making
5	Lack of regulation and transparency in the handling of funds
6	Political support at the local government level
7	Low reimbursement fees
8	Poor understanding of insurance plans and expectations
9	Unwillingness to participate in the scheme
10	Unfavorable government politics with the scheme

Similarly, the facility managers highlighted as a potential unintended consequence of the scheme, a reduction in financial support from the LGA especially for services such as community outreach that would not be covered by the insurance scheme. The facility managers maintained that the state government intends to make a deduction from the LGA’s budget because of increased insurance collections, and this may disincentivize local government officials from providing continued support to the PHCs in the LGAs.

“*Lagos state government plans to take money from source*, *meaning that they will be taking money directly from the LGA*. *It is going to affect services*. *There are services that are already free and there are other services like community outreaches that will not be under the health insurance*, *and the LGAs will no longer support these services since the state government is already deducting money from them*. *There will be malalignment between the health department and other departments in the LGA*. *As a result*, *lack of political will could worsen and if the capitation should go directly to them [LGA]*, *we will be worse off and there will be a stand still*.*” (Public Facility Manager)*

## Discussion

This study examined the characteristics of providers that participated in insurance programs, the factors that influenced their participation and the expectations of facility managers from the Lagos State Health Scheme.

### Characteristics of provider facilities that do or do not participate in insurance

We observed that the number of years of operation, size, type, and ownership of facilities matter in insurance participation. A higher proportion of provider facilities participating in insurance relative to non-participating facilities were larger with mid to (very) high patient volume, workforce, and longer years of operation. In addition, a higher proportion of medical centers, specialist and teaching hospitals participated in insurance compared to health clinics and maternity homes. This could be because medical centers, specialist and teaching hospitals typically have higher service volume and workforce to accommodate any increase in the volume of patients associated with insurance participation than the health clinics and maternity homes, which are generally smaller in size and capacity. This could also be because the large facilities have the administrative capacity and the sophistication to understand the advantages of capitation paid for primary care.

Conversely, the size and capacity of a facility could also be a key factor that HMOs consider before reaching out to the facility to join their insurance plans. Larger facilities are more likely than smaller facilities to have the capacity to train the workforce needed for insurance work, which could mean less contracting burden for the HMOs. In general, HMOs were more likely to deal with the medical centers, specialist and teaching hospitals than health clinics and maternity homes.

In terms of ownership, a greater proportion of private facilities compared to public facilities participated in insurance. This could be because private facilities enjoy higher levels of autonomy in decision making about operations than public facilities. However, for the few public facilities participating, nearly all were large facilities with mid to very high volume of service delivery. The smaller public facilities were less likely to participate in insurance.

Looking at insurance participation from the facility level, we observed that a higher proportion of facilities at the secondary and tertiary levels participated in an insurance program than primary level facilities. This was not surprising since most of the large facilities operated at the secondary and tertiary levels of care. However, the lack of participation at the primary level raises a wider concern for the role of primary care in achieving universal health coverage. If the primary facilities are not fully equipped to provide the first line of services that community members need, it means the population either would be unable to receive timely preventive care or would visit the secondary level facilities to receive primary care, overcrowding and overburdening these facilities, while the primary facilities remain underutilized.

### Drivers and barriers of insurance participation

Regarding the drivers and barriers to insurance participation, we observed that high reimbursement fee, low amount of paperwork, short processing time for reimbursements and lower probability of claim reduction and denial were all motivating factors for facilities to join an insurance program. Conversely, at the patient level, patients poor understanding of their insurance plans and unrealistic expectations when they show up for care, as perceived by the facilities were barriers to insurance participation. Our findings are supported by many other studies that have examined determinants of provider participation in insurance programs [5, 20, 36–41].

From the results of this study, it is also clear that increase in patient volume, which could lead to increase in revenue, and additional cash to manage the operations of the facility and invest in facility upgrade is a key driver for facility insurance participation. Maximizing profit is also a key driver for private facilities and not for the public facilities. Private insurance was perceived to be more profitable than government insurance. This could be because the providers in the private market are price setters and are able to determine the amount charged to patients paying out of pocket or negotiate with HMOs based on their assessment of the cost and benefits, the amount charged to insured patients. On the other hand, in the public insurance market, providers are price takers, they do not have control over the amount charged to patients and the reimbursement received is well below the fees they charge. These findings therefore align with the two-sided economic theory by Sloan and colleagues that suggests that providers like every other firm in a dual market would gravitate towards the market that offers more benefits before considering the other [17].

To that end, if providers perceive government insurance to be less profitable, it may have an unintended consequence on provider contracting and improved access to care. In the case of the LSHS, this could mean that providers may choose not to participate in the scheme, or when they do participate, ignore LSHS enrollees, while spending more time providing care to patients paying out of pocket or via private insurance. This may eventually lead to patients perceiving the quality of care received through the LSHS to be poor and discouraging future registration with the scheme.

Ghana’s national health insurance program currently faced a similar challenge. In a study assessing the achievements and challenges of the national health insurance scheme in Ghana, many patients obtaining care through the national insurance program perceived their care to be poorer than patients who paid for care via private means [42]. These patients reported their prescribed drugs to be of lower quality, and that patients who paid out of pocket were prioritized over them to receive care. They also reported experiencing negative attitudes from health workers, who generally perceived patients accessing care through the government’s insurance program as poor. As a result, some beneficiaries of the national scheme in Ghana failed to renew their memberships [42]. In Nigeria also, a study assessing the quality of care received by diabetic patients in public facilities under Nigeria’s NHIS program found that those with health insurance perceived the quality of care received to be worse, even though it was not, and in spite of the fact that they paid less out of pocket compared to those without insurance receiving same care [43]. To ensure that this does not happen with the LSHS, the government must be ready to guarantee providers adequate and prompt payment of their reimbursements.

### Expectations from the Lagos State Health Scheme

The facility managers anticipate an increased volume of patients, revenue and opportunity to invest in facility upgrade as some of the benefits that the facilities would get from the Lagos State Health Scheme. However, they also anticipate, especially the private facilities, that low reimbursement fees without a guarantee of high patient volumes would be a major challenge to participate in the scheme.

There is also a big capacity issue for the public facilities: inadequate infrastructure, shortage of skilled health workers, dearth of drugs and supplies, and poor access to finance for infrastructure upgrade that could pose a challenge to the successful implementation and scale-up of the scheme across the state. Many public facility managers lack the management and financial autonomy to address these capacity gaps in their facilities. These findings about the weak capacity at the public facilities and their managers’ lack of financial autonomy is consistent with the Nigerian study described earlier assessing the quality of care received by diabetic patients in public facilities under Nigeria’s NHIS program [43]. The study found that inadequate supply of health workers, lack of equipment, shortage of drugs and the inability to manage funds to keep drugs in stock when used, were barriers to delivering quality care to patients in the public facilities. As a result, many of the patients receiving care under the scheme perceived the quality of their care to be poor [43]. To prevent this from happening in the LSHS, the Lagos state government should invest in timely infrastructure upgrade, recruitment of health workers and adequate supply of drugs and commodities for the public facilities. It would also need to ensure that public facility managers have increased management and financial autonomy to run the operations and improve the quality of care delivered in the facilities.

In the public facilities also, facility managers for the primary health centers are concerned about the lack of transparency in the flow of funds and the possibility of a weak buy-in of the LSHS by some officials at the local government level. Primary health facilities are typically funded with fixed budgetary allocation from the local government, which facility managers do not have financial autonomy over. As a result, the facility managers at the primary care level are mostly worried that they may not receive the revenue earned from capitation if the payments go directly to the LGAs or Local Council Development Areas (LCDAs) instead of the primary health centers. These facility managers are also worried about the potential withdrawal of funding support from the LGAs/LCDAs for programs such as community outreaches and public health education programs that would not be covered under the state insurance program. The Lagos state government will need to pay particular attention to these issues since the success of the LSHS and the attainment of universal health coverage will be impossible to achieve if the population members do not have access to quality care at the primary health level.

Finally, the LSHS should consider the associated administrative burden for providers that would need to work with multiple HMOs (if HMOs are being considered in the roll out of the scheme). The HMO model has been beneficial to the NHIS as a non-governmental system for paying capitations, approving secondary care, and a non-governmental system to process claims for that care. This model is valuable since some of the personnel needed to run an efficient claims payment system may be unavailable in the civil service system. Thus, for efficient claims processing, we would suggest that Lagos state considers selecting one HMO through a competitive process to perform this duty. However, we understand that one HMO across the state may create a monopoly, and as a result, we would suggest having one HMO in charge of each of the five senatorial zones in Lagos and for government to contract with each HMO for a maximum of 4 years, after which a competitive selection process would commence again. This is similar to what is done in the United States with the Medicare program, where private healthcare insurers compete to handle claims for exclusive geographic regions [44, 45]. In the end, the LSHS would get the advantage of private sector claims processing expertise without unduly complicating the provider’s administrative duties by forcing them to deal with multiple payers.

## Study limitations

This study has some potential limitations. First, only six LGAs were purposively selected for the study. This may limit the generalizability and transferability of the findings from the study. However, to improve the validity of the findings, the six LGAs were selected to reflect many of the differences in population size, income level, urbanization and insurance participation across the state. Secondly, some providers, particularly the large public teaching and general hospitals were purposively selected and may have overestimated some of the findings for the public facilities. However, given their large capacities and insurance participation insights, it was imperative to include them in the sample frame. Lastly, the sample size for this study was relatively small, and as a result, it was impossible to run a multivariable regression analysis to examine the statistical significance of the quantitative findings.

## Conclusion

This study used the two-sided economic theory by Sloan and colleagues to assess the determinants of provider participation in insurance. This theory hypothesizes that there are two markets, public and private, where providers sell their services, and that providers would naturally gravitate towards the market that offers more financial benefits. From this study, it is clear that the perceived benefits of participating in insurance in the private market currently outweigh its perceived costs, particularly for the big facilities, whereas this is likely not the case for the public insurance market. This is evident from the results showing that providers generally perceived private insurance to be more profitable than government insurance even though government insurance paid faster and more regularly. This could be, as explained by the two-sided economic theory, that providers in the private market are price setters and as a result can negotiate with HMOs the amount charged to insured patients. On the other hand, in the public insurance market, providers are price takers and do not have control over the amount charged to patients.

Therefore, the key to success of the LSHS and other public insurance programs in low-and-middle income countries lies in the government’s ability to effectively manage and finance the scheme, and contract with healthcare providers. In its contracting with providers, governments must create policies that would ensure a reduction in the costs and an improvement in the benefits of insurance participation for the providers. We have suggested a model through alternating and potentially competitive time-limited contracts with HMOs that can help Lagos state and governments of other developing economies to achieve this and accelerate its progress towards universal health coverage. We have also found that smaller primary care facilities will need additional help to provide the level of resources required for insurance accreditation and to manage the process of collecting insurance payments. It is also vital that public facilities receive the funds earned through additional services provided to insured patients, and that public subsidies for outreach and other “uncovered” services are maintained.

## Supporting information

S1 Appendix List of NHIS accredited primary provider facilities. Obtained online from NHIS website, October 2017.
(PDF)

S2 Appendix List of NHIS accredited secondary provider facilities. Obtained online from NHIS website, October 2017.
(PDF)

S3 Appendix Questionnaire and interview guide. (PDF)

S1 File Minimal dataset for reproduction of results. Dataset containing variables used for analysis in Excel format.
(XLSX)

## References

[1] Onoka CA, Onwujekwe OE, Uzochukwu BS, Ezumah NN. Promoting universal financial protection: constraints and enabling factors in scaling-up coverage with social health insurance in Nigeria. Health Res Policy Syst. 2013 Jun 13;11(1):20. doi: 10.1186/1478-4505-11-20 23764306 PMC3686590

[2] Okpani AI, Abimbola S. Operationalizing universal health coverage in Nigeria through social health insurance. Niger Med J J Niger Med Assoc. 2015;56(5):305–10. doi: 10.4103/0300-1652.170382 26778879 PMC4698843

[3] Ogundeji YK, Ohiri K, Agidani A. A checklist for designing health insurance programmes–a proposed guidelines for Nigerian states. Health Res Policy Syst. 2019 Aug 22;17(1):81. doi: 10.1186/s12961-019-0480-8 31438972 PMC6704650

[4] Lagos to kickstart Health Insurance Scheme–Lagos State Government [Internet]. [cited 2017 Apr 25]. Available from: http://lagosstate.gov.ng/blog/2017/04/20/lagos-to-kick-start-health-insurance-scheme/

[5] Garner DD, Liao WC, Sharpe TR. Factors Affecting Physician Participation in a State Medicaid Program. Med Care. 1979;17(1):43–58. doi: 10.1097/00005650-197901000-00004 366292

[6] Etiaba E, Onwujekwe O, Honda A, Ibe O, Uzochukwu B, Hanson K. Strategic purchasing for universal health coverage: examining the purchaser–provider relationship within a social health insurance scheme in Nigeria. BMJ Glob Health. 2018 Oct 1;3(5):e000917. doi: 10.1136/bmjgh-2018-000917 30483406 PMC6231103

[7] Lagos State Health Insurance Scheme–Ministry of Health [Internet]. [cited 2017 Apr 25]. Available from: http://health.lagosstate.gov.ng/lagos-state-health-insurance-scheme/

[8] Improving Financial Access to Maternal, Newborn and Child Health Services for the Poor in Nigeria. Community Based Health Insurance Brief. 2011. [Internet]. [cited 2017 May 15]. Available from: https://www.healthpolicyproject.com/pubs/97_communitybasedhealthinsurance.pdf

[9] Lagos Household Survey Report. Lagos Bureau of Statistics; Ministry of Economic Planning and Budget. 2014. [Internet]. [cited 2017 Apr 26]. Available from: http://mepb.lagosstate.gov.ng/wp-content/uploads/sites/29/2017/01/HOUSEHOLD-SURVEY-2013-REPORT.pdf

[10] International Finance Corporation. The Business of Health in Africa: Partnering with the Private Sector to Improve People’s Lives. Washington (District of Columbia): International Finance Corporation. 2007.

[11] Health Insurance Coverage For Nigerians Still Abysmal; An Urgent Call For New Strategy. [Internet]. NOI Polls. 2019 [cited 2020 Jul 8]. Available from: https://noi-polls.com/fourteen-years-after-the-establishment-of-nhis-about-90-percent-of-nigerians-still-do-not-have-health-insurance-cover/

[12] Obikeze E, Onwujekwe O. The roles of health maintenance organizations in the implementation of a social health insurance scheme in Enugu, Southeast Nigeria: a mixed-method investigation. Int J Equity Health. 2020 Mar 12;19(1):33. doi: 10.1186/s12939-020-1146-4 32164725 PMC7068878

[13] Onoka CA, Hanson K, Mills A. Growth of health maintenance organisations in Nigeria and the potential for a role in promoting universal coverage efforts. Soc Sci Med. 2016 Aug 1;162:11–20. doi: 10.1016/j.socscimed.2016.06.018 27322911

[14] HMO Contacts–National Health Insurance Scheme [Internet]. [cited 2020 Jun 26]. Available from: https://www.nhis.gov.ng/hmo-contacts/

[15] Sick health insurance scheme leaves patients helpless [Internet]. [cited 2017 May 15]. Available from: http://en.africatime.com/articles/sick-health-insurance-scheme-leaves-patients-helpless

[16] Christina CP, Latifat TT, Collins NF, Olatunbosun AT. National health insurance scheme: How receptive are the private healthcare practitioners in a local government area of Lagos state. Niger Med J J Niger Med Assoc. 2014;55(6):512–6. 10.4103/0300-1652.144712 PMC4262851 25538373

[17] Sloan FA. Private physicians and public programs. Lexington, Mass.: Lexington, Mass.: Lexington Books; 1978.

[18] Nguyen H. The principal-agent problems in health care: evidence from prescribing patterns of private providers in Vietnam. Health Policy Plan. 2011 Jul 1;26(suppl_1):i53–62. doi: 10.1093/heapol/czr028 21729918

[19] Sloan FA, Steinwald B. Physician Participation in Health Insurance Plans: Evidence on Blue Shield. J Hum Resour. 1978;13(2):237–63. 670695

[20] Sloan F, Mitchell J, Cromwell J. Physician Participation in State Medicaid Programs. J Hum Resour. 1978;13:211–45. 363938

[21] Hill DB. Physician Participation in Health Care Programs. Policy Stud J Urbana Ill. 1981 Summer;9(7):1092–6.

[22] Davidson SM, Perloff JD, Kletke PR, Schiff DW, Connelly JP. Full and Limited Medicaid Participation Among Pediatricians. Pediatrics. 1983 Oct 1;72(4):552–9. 6351008

[23] Perloff JD, Kletke PR, Fossett JW, Banks S. Medicaid Participation among Urban Primary Care Physicians. Med Care. 1997;35(2):142–57. doi: 10.1097/00005650-199702000-00005 9017952

[24] Brunt CS, Jensen GA. Payment generosity and physician acceptance of Medicare and Medicaid patients. Int J Health Care Finance Econ. 2014 Dec 1;14(4):289–310. doi: 10.1007/s10754-014-9152-y 25005072

[25] Long SK. Physicians May Need More Than Higher Reimbursements To Expand Medicaid Participation: Findings From Washington State. Health Aff (Millwood). 2013 Sep 1;32(9):1560–7. doi: 10.1377/hlthaff.2012.1010 24019360

[26] Folland S, Goodman AC, Stano M. The economics of health and health care. 7th ed. Upper Saddle River, N.J: Pearson; 2013. 602 p.

[27] Lagos and Equitable Healthcare Services–Lagos State Government [Internet]. [cited 2018 Apr 2]. Available from: https://lagosstate.gov.ng/blog/2017/07/05/lagos-and-equitable-healthcare-services/

[28] Lagos State Health Facility Monitoring and Accreditation Agency (HEFAMAA) Directory [Internet]. [cited 2021 May 8]. Available from: https://hefamaa.lagosstate.gov.ng/new/hefamaa-directory/

[29] Quinn Patton M. Qualitative research and evaluation methods. 2002. 3rd edition, Thousand Oaks, CA: Sage [Internet]. SAGE Publications Inc. Available from: https://us.sagepub.com/en-us/nam/qualitative-research-evaluation-methods/book232962

[30] Baker SE, Edwards R. National Centre for Research Methods review paper: How many qualitative interviews is enough? Expert voices and early career reflections on sampling and cases in qualitative research. National Centre for Research Methods, Southampton, UK. [Internet]. [cited 2018 Jun 11]. Available from: http://eprints.ncrm.ac.uk/2273/4/how_many_interviews.pdf

[31] Onwuegbuzie AJ, Dr NLL, Onwuegbuzie AJ, Leech NL. Onwuegbuzie AJ, & Leech NL. Sampling Designs in Qualitative Research: Making the Sampling Process More Public. The Qualitative Report, 2007;12(2), 238–254.

[32] Onwuegbuzie A, Collins K. Onwuegbuzie AJ, Collins KM. A typology of mixed methods sampling designs in social science research. The qualitative report. 2007;12(2):281–316. Qual Rep. 2007 Jun 1;12(2):281–316.

[33] Ritchie J, Lewis J, Nicholls CM, Ormston R, editors. Qualitative research practice: A guide for social science students and researchers. Sage; 2013 Nov 1. [Internet]. [cited 2018 Jun 21]. Available from: http://www.sxf.uevora.pt/wp-content/uploads/2013/03/Ritchie_2003.pdf

[34] Creswell JW, Clark VLP. Designing and Conducting Mixed Methods Research. SAGE Publications; 2017. 521 p.

[35] Srivastava, A. & Thomson, S. B. (2009). Framework Analysis: A Qualitative Methodology for Applied Policy Research. JOAAG, Vol. 4. No. 2 [Internet]. [cited 2018 Apr 2]. Available from: http://research.apc.org/images/a/ad/Framework_analysis.pdf

[36] Cunningham PJ, O’Malley AS. Do Reimbursement Delays Discourage Medicaid Participation By Physicians? Health Aff Chevy Chase. 2009;W17–28. doi: 10.1377/hlthaff.28.1.w17 19017679

[37] Berman S, Dolins J, Tang S, Yudkowsky B. Factors That Influence the Willingness of Private Primary Care Pediatricians to Accept More Medicaid Patients. Pediatrics. 2002 Aug 1;110(2):239–48. doi: 10.1542/peds.110.2.239 12165573

[38] Yudkowsky BK, Cartland JDC, Flint SS. Pediatrician Participation in Medicaid: 1978 to 1989. Pediatrics. 1990 Apr 1;85(4):567–77. 2179849

[39] Hadley J. Physician participation in Medicaid: evidence from California. Health Serv Res. 1979;14(4):266–80. 393659 PMC1072124

[40] Mitchell JB. Physician Participation in Medicaid Revisited. Med Care. 1991;29(7):645–53. doi: 10.1097/00005650-199107000-00004 2072769

[41] Perloff JD, Kletke P, Fossett JW. Which physicians limit their Medicaid participation, and why. Health Serv Res. 1995 Apr;30(1 Pt 1):7–26. 7721586 PMC1070348

[42] Teye JK, Arhin AA, Anamzoya AS. Achievements and Challenges of the National Health Insurance Scheme in Ghana. Curr Polit Econ Afr Hauppauge. 2015;8(3):487–511.

[43] Okoro CS. Assessing the Quality of Care Received by Diabetes Patients under the Nigeria NHIS. Does Enrollment in Health Insurance Matter. Doctoral Dissertation, Boston University. 2017. Available from: https://open.bu.edu/handle/2144/23376

[44] Foote SB. The Impact of the Medicare Modernization Act’s Contracts Reform on Fee-for-Service Medicare Symposium: Medicare after the Medicare Modernization Act. St Louis Univ J Health Law Policy. 2007 2008;1:67–78.

[45] U.S. Centers for Medicare and Medicaid Services. What is a Medicare Administrative Contractor (MAC) and What do they do? [Internet]. 2017 [cited 2018 Jul 11]. Available from: https://www.cms.gov/Medicare/Medicare-Contracting/Medicare-Administrative-Contractors/What-is-a-MAC.html

