# Supplementary material for: Determinants and perception of health insurance participation among healthcare providers in Nigeria: A mixed-methods study
Source: PLoS One. 2021 Aug 4;16(8):e0255206. doi: 10.1371/journal.pone.0255206 (PMC8336839; doi:10.1371/journal.pone.0255206)
Supplement: S1 Appendix — Obtained online from NHIS website, October 2017. (PDF) [file pone.0255206.s001.pdf]

ACTIVE PRIMARY HCP AS AT JULY 19TH 2017

	HCP CODE	HCP NAME	ADDRESS
1	AB/0001	Sancta Maria Specialist & Mat.	22 Constitutition Crescent Aba
2	AB/0003	Janet Memorial Hospital	245 Aba-Owerri Road, Aba
3	AB/0004	Abia State University Teaching Hospital	Aba
4	AB/0005	Life Care Clinics Ltd	8, Ezinkwu Street, Aba
5	AB/0006	New Era Specialist Hospital	213/215 Ezikiwe Road, Aba
6	AB/0007	John Okorie Memorial Hospital	12-14 Akabuogu Street Off P.H. Road, Aba
7	AB/0013	Delta Hospital Ltd.	78, Faulks Road
8	AB/0014	Federal Medical Centre, Umuahia	Umuahia, Abia State
9	AB/0015	Obioma Hospital & Maternity	21, School Road, Umuahia
10	AB/0016	Priscillia Memorial Hospital	32, Bunny Street
11	AB/0018	General Hospital, Ama-Achara	Ama-Achara, Umuahia
12	AB/0019	Mendel Hospital & Diagnostic Centre Limited	20 Tenant Road, Abia
13	AB/0020	Clehansan Hospital & Maternity	17 Osusu Rd, Aba.
14	AB/0022	Alpha Inland Hospital	36 Glass Industry Road, Aba
15	AB/0023	Todac Clinic Ltd.	59/61 Okigwe Road,Aba
16	AB/0024	Living Word Hospital	5/7 Umuocham Street, Aba
17	AB/0025	Living Word Hospital	117 Ikot Ekpene Road, Aba
18	AB/0027	Ebemma Hospital	3rd Avenue, Ubani Estate Ogbor Hill, Aba
19	AB/0028	Horstman Hospital	32, Okigwe Road, Aba
20	AB/0030	Princess Mary Specialist Hospital	45, New Umuahia Road Ogbor Hill, Aba
21	AB/0031	Austin Grace Hospital	16, Okigwe Road, Aba
22	AB/0034	St. Paul Hospital	6-12 St. Paul's Road, Umunggasi, Aba
23	AB/0035	Seven Days Adventist Hospital	Umuoba Road, Aba
24	AB/0036	New Lead Hospital & Mat. Ltd	10, Oblagu Ave/By 160 Faulks Road, Aba.
25	AB/0038	Hammersmith Medical Centre	Plots 3 Amauzkwu Layout, Umauhia
26	AB/0039	St. Vincent's Hospital	30 Azikwe Road Umuahia
27	AB/0041	New Era Hospital & Maternity	90b Oji River Street Umuzukwu Layout
28	AB/0090	DSS Clinic	Umuahia, Abia State
29	AB/0093	Police Hospital, Umuahia	Abia State Police Command, Abia State
30	AB/0094	Sick bay NNLS Owerenta	Abia, Abia State
31	AB/0095	The Nazareth Clinics	57 Pound Road, Aba. Abia State
32	AB/0097	Isaac Okwuonu Memorial Hospital	155 Ikot-Ekpene Road, Ogbor Hill, Aba.
33	AB/0098	Spring Clinic	18 Scotland Crescent, Aba
34	AB/0104	National Root Crops Research Institute Staff Clinic, Umudike	Umudike, Umuahia
35	AB/0107	Healing Cross Hospital	Kilometre 2 Bende Road, Umuahia, Abia State
36	AB/0110	Aloma specialist hospital	9(108) aba road, umuahia

37	AB/0111	Best care specialist hospital	6, madukwe close off Ahuanya Av. Umungasi, Aba
38	AB/0112	Divine grace missionary hospital	49, Azikwe road, Aba
39	AB/0113	El-Shaddai hospital	40 AHOADA STREET, UMUAHIA
40	AB/0115	GLORY OF GOD HOSPITAL	OWAZA TOWN, ABIA STATE
41	AB/0116	GROUP MEDICAL PRACTICE	1,EMUCHAY CLOSE OFF AZUKA DRIVE, OGBO HILL ABA
42	AB/0117	Harvey Clinic	14, HOWELL''''S CRESCENT, ABA
43	AB/0118	MARGARET HOSPITAL	45 FINBARR'S ROAD, UMUAHIA
44	AB/0120	REGENTS CROSS SPEC. HOSPITAL	106, IKOT EKPENE ROAD, ABA
45	AB/0123	SUNSHINE HOSPITAL	KM1 IKOT EKPENE ROAD, UMUAHIA
46	AB/0145	Michael Okpara University of Agriculture Medical Centre	Umudike,
47	AB/0149	J Medi - Care Hospital	73 Bonny Street Umuahia
48	AB/0150	Chukwuebuka Hospital	50 School Road, Umuahia
49	AB/0153	Ngozi Hospital	2 Isuochi Close, Umuahia
50	AB/0170	MC Hospital	59, Omuma Road, Aba
51	AB/0172	Uzondu Polyclinic	8 Ofali Agwu Road Amaekpu Ohafia,Abia State
52	AB/0173	Miracle Hospital	5 Ekpem Street, 200 Obohia Road,Aba, Abia State
53	AB/0174	General Hospital Ohafia	Ohafia Abia State
54	AB/0175	Daughters Of Mary Mother Of Mercy Hospital	AhiaekeNdume, UmuahiaAbia State
55	AB/0177	Blessed Haven Hospital	50,Omoba Road, Ogbor Hill, Aba, Abia State
56	AB/0179	Impact Hospital &Maternity	59B Ukaegbu Road OgborHills,AbaAbia State
57	AB/0181	Cottage Hospital Azumiri	Azumiri, Abia State
58	AB/0182	Nigerian Christian Hospital Nlagu	Km 18,Aba-Ekot-Ekpene Road Aba Abia State
59	AB/0184	Life Essential &Maternity	32, Uyo Street, by Okigwe road, Umuahia
60	AB/0185	Goodness & Mercy Christian Hospital	121,Faulks Road Opp UBA PLC Abia State
61	AB/0186	Police Hospital	Bendel Road Umuahia Abia State
62	AB/0187	Standard Hospital And Maternity	43, Abdulrahma Kanu Street Off 129 Continental Rd (Former MCC Road) Aba
63	AB/0188	Shammah Christian Hospital Maternity & Lab	4, Ogwo road, Aba Abia State
64	AB/0190	Uzondu Polyclinic	8 Ofali Agwu Road Amaekpu Ohafia,Abia State
65	AB/0192	Shammah Christian Hospital Maternity & Lab	4 Ogwo Road Off Ohanku Road, Aba. Abia State.
66	AB/0193	Standard Hospital And Maternity	43, Abdulrahman Kanu Street Off 129 Continental Road (Former MCC Road), Aba. Abia State.

67	AB/0194	Madonna Catholic Hospital	Ohokobe Afaraukwu Aba Road, Umuahia. Abia State
68	AB/0197	Pink Rose Hospital	4 Enyiukwu Road Extension/172 Aba Road (Stanpol Junction) Umuahia Abia State
69	AB/0199	Nissi Foundation Hospital & Maternity	56 Omuoba Road, Ogbor Hill, Aba Abia State
70	AB/0200	Amara Jane Hospital & Maternity	Umuana Gate 2,The Ndume , Umuahia Abia State
71	AB/0201	Dominion Hospital & Maternity	9A Sunny Lane, off Azuka Drive, Ogbor Hill, Aba Abia State
72	AB/0202	Christ De King Health Services Ltd	9 Orie Ngodo, Isuochi. Abia State
73	AB/0203	Calif Specialist Hospital	25/27 Chief Adiukwu Street, By Dip Gold Filling Station, Off Enugu –PH Express Way, Aba Abia State
74	AB/0204	143 Battalion MRS	Ovim Barracks, Ovim Abia State
75	AB/0211	Embassy Specialist & maternity Care Ltd.	No. 59c Ayaba Umueze Road, Opp. ABSUTH, second gate, Aba.
76	AB/0213	All Saints Hospital	No. 26 Item street Umuahia.
77	AB/0214	St. Anthony Hospital Ltd.	No. 52-80 Etche Road, Aba.
78	AB/0216	Emschel Clinics and Maternity	No. 2 Scotland crescent, Aba.
79	AD/0002	General Hospital, Ganye	Ganye, Adamawa State
80	AD/0006	Cottage Hospital, Mayo-Belwa	Mayo-Belwa
81	AD/0011	Beekay Clinic & Maternity	Maiha Road, Mubi
82	AD/0013	General Hospital, Numan	Numan
83	AD/0015	Cottage Hospital, Song	Song
84	AD/0017	Specialist Hospital, Yola	Yola
85	AD/0018	Barka Clinic & Maternity	Uba Town
86	AD/0019	Triumph Medical Clinic	Mubi Road, Jimeta
87	AD/0020	Matco Clinic	Army Barracks Road, Malamre, Yola
88	AD/0021	Galbose Clinic	Mubi Road, Yola
89	AD/0022	Mahmud Clinic	Jimeta, Yola
90	AD/0023	Dawau Clinic Ltd.	Jimeta Yola
91	AD/0024	Peace Hospital	No. 2 Luggere Street, Beside St. Theresa''''s Cathedral, Yola, Adamawa State
92	AD/0025	Adamawa Hospital	Abuja Road, Yola Town
93	AD/0027	Sauki Medical Clinic	Bauchi Street, Off Mustapha Moh''D Way, Jimeta, Yola
94	AD/0028	The Freedom Polyclinic	9b Hospital Road, Jimeta
95	AD/0029	Galbose Clinic & Maternity	22 Atiki Abubakar Way, Jimeta, Yola
96	AD/0030	Jimeta Clinics & Maternity	Bishop Street, Jimeta, Yola
97	AD/0048	Federal Medical Centre	Yola
98	AD/0049	Arhyel Medical Centre	Yola Bye-Pass
99	AD/0050	Noma Clinic & Maternity	Gombi Town
100	AD/0051	Arhyel-Royal Hospital	Numan Road, Jimeta
101	AD/0052	General Hospital, Mubi	Mubi

102	AD/0053	Newlife Hospital	Sabon Layi, Mubi
103	AD/0054	General Hospital, Garkida	Garkida
104	AD/0055	Cottage Hospital, Hong	Hong
105	AD/0056	Cottage Hospital, Guyuk	Guyuk
106	AD/0057	Cottage Hospital, Borrong Demsa	Borrong Demsa
107	AD/0076	75 STG Medical Centre	NAF Station, Yola
108	AD/0077	General Hospital, Michika	Michika, Adamawa State
109	AD/0078	Cottage Hospital, Fufore	Fufore, Adamawa State
110	AD/0081	23 Bde MC Yola	Yola, Adamawa State
111	AD/0082	232 TK Bn MRS, Yola	Yola, Adamawa State
112	AD/0083	Federal College of Education, College Clinic	Jimeta, Yola Road, Adamawa State
113	AD/0084	Federal Polytechnic Clinic, Mubi	Barama Ward, Mubi
114	AD/0085	MercyLand Clinics & Maternity	Mubi Bye-pass Jimeta, Yola
115	AD/0096	DSS Clinic	Yola, Adamawa State
116	AD/0097	Police Clinics, Adamawa	Adamawa State
117	AD/0098	Federal University of Tech. Medical Centre, Yola	Yola, Adamawa State
118	AD/0101	COTTAGE HOSPITAL, GULEK	GULEK
119	AD/0102	DEMBELE NURSING & MAT. HOME	GAYA, MUBI
120	AD/0104	OPTIMUM CLINIC	100 MOH''''''''D MUSTAPHA WAY, JIMETA
121	AD/0105	UBIS CLINIC	10 MAIHA RD, MUBI
122	AD/0116	Trust Medical Centre	Anguwan Faransa, Gombi
123	AD/0118	The New Boshang Clinic and maternity Ltd	Karewa GRA, Jimeta-Yola
124	AD/0120	Adamawa State University Health Centre	Adamawa State University, Mubi
125	AD/0121	Valli Medical Clinic	22 Mallamre, Opp FCE First Gate, Along Aliyu Way, Jimeta
126	AD/0124	Kowa Clinic	Near New Market, Mayo Belwa
127	AD/0125	Da'ama Specialist Hospital	# 70/72 Atiku Abubakar Road, Yola
128	AD/0126	American University Of Nigeria Clinic.	Lamido Zubairu Way, Yola By-Pass, Adamawa State.
129	AD/0127	Nigerian Law School Clinic	Nigerian Law School, Yola Campus Adamawa
130	AD/0128	Bamaiyi Hospital And Clinic	123, Atiku Abubakar Road, Jimeta-Yola, Adamawa state
131	AD/0129	Meddy Private Clinic	Opposite School Of Nursing Yola, Adamawa State.
132	AD/0130	Graceland Clinic & Maternity	2 City Hotel Road, Numan
133	AD/0131	Medical and Health Department,	Adamawa State Polytechnic, Yola, Adamawa State
134	AD/0132	Valada Hospital & Maternity	Army Barracks Road Yola Adamawa State
135	AK/0001	Samaritan Clinic	No. 7 Ekong Uko Street, Eket
136	AK/0002	Immanuel General Hospital	Eket, Akwa Ibom
137	AK/0007	General Hospital, Ikot Ekpene	Ikot Ekpene, Akwa Ibom
138	AK/0010	General Hospital, Iquita-Oron	Iquita-Oron
139	AK/0011	State Staff Clinic	Secretariat Complex, Abak Rd., Uyo

140	AK/0012	University Of Uyo Teaching Hospital, Uyo	Uyo, Akwa Ibom
141	AK/0013	Mfon Abasi Medical Centre	Uyo, Akwa Ibom
142	AK/0014	Uwana Family Hospital	Uyo, Akwa Ibom
143	AK/0015	Ibom Clinic/Hospital	Uyo, Akwa Ibom
144	AK/0016	Lifecare Hospital	Uyo, Akwa Ibom
145	AK/0017	Uwakmfon Specialist Clinics & Hospital	27 Nwaniba Road, Uyo, Akwa Ibom State
146	AK/0030	NAF Medical Centre	Air Force Comp Sec. Sch., Uyo
147	AK/0032	6 Bn MRS Abak	Abak, A/Ibom State
148	AK/0033	Sick bay	FOB Ibaka, Akwa Ibom State
149	AK/0034	Mainland Clinic/Hospital	2 Andy-Umana Lane, Off Aka Road, Uyo, Akwa Ibom
150	AK/0035	Majesty Hospital & Eye Clinics	47 Wellinton Bassey Akwa Ibom
151	AK/0037	Mount Olive Clinic & Maternity	8 Ebonny Essien Street, off Ikot Ekpene Rd, Uyo, A/Ibom State
152	AK/0043	DSS Clinic	Uyo, Akwa Ibom State
153	AK/0044	Police Clinics, Akwa Ibom	Akwa Ibom State
154	AK/0045	University of Uyo Health Centre, Uyo	Uyo, A/Ibom State
155	AK/0046	All Saints Medical Centre	1-5 Independent Avenue, Abak, Akwa Ibom
156	AK/0047	Alma clinics	15, Afia street, uyo, Akwa-ibom
157	AK/0048	Dammy memorial hospital	34, ukana offot street, uyo, Akwa-ibom
158	AK/0049	Dan Abia specialist clinic	11b, Idoro road, off itam junction, uyo,Akwa-ibom
159	AK/0050	Domingo hospital	Plot 9, unit E, Ben Udo Street, Ewet Housing estate, uyo
160	AK/0051	Ekop specialist clinic	181, F Ewet Housing Estate, Uyo
161	AK/0053	Gateway hospital	15, okon essien lane, uyo
162	AK/0054	Good shepherd hospital	75, eket oron road, Eket
163	AK/0055	Iha foundation medical center	Eket Annex, Ekpeyu street, Ikot ebok Eket
164	AK/0056	Kaizo specialist hospital	10, market road,Afaha offot, off Abak road, Uyo
165	AK/0057	kUFRE ABASI CLINICS & MAT.	31/34, EKPO OBOT STREET, UYO
166	AK/0058	Mercy hospital	95 hospital road, Abak, Ak/Ibom
167	AK/0059	St. Athanasius hospital	1, ufeh street,F.H.estate , Abak road, Uyo
168	AK/0060	Ubong Abasi specialist clinic	5, clement Isong street, FHA estate, Abak road, Uyo
169	AK/0062	Uwem foundation clinic	8b Etienam street, oron, AK/IBOM
170	AK/0082	Our Lady of Lourdes Infirmary	22 Ekpanya Street Uyo
171	AK/0083	Eduwem Clinic	Ntan Akpan Udom,Ibiano-Ibom, Akwa Ibom State
172	AK/0084	Etuknwa Specialist Clinic	120,Udo-Umana Street, Uyo Akwa Ibom State
173	AK/0085	Dunamis Clinic And Maternity	25 A,Afia Etid Street, Off Udo Umana Street, Uyo Akwa Ibom State

174	AK/0087	Jeconiah Specialist Hospital	Off 308,Oron Road, Uyo Akwa Ibom State
175	AK/0088	Manafen Group Hospital	114,Oron Road, Oron Akwa Ibom State
176	AK/0089	Our Lady Of Lourdes Infirmary	22,Ekpanya Street, Uyo Akwa Ibom State
177	AK/0090	Petalice Medical Centre LTD	No 5/7 Marickson Hospital Way Akwa Ibom State
178	AK/0091	Police Clinic Ikot Ekpene	Ikot Ekpene Akwa Ibom State
179	AK/0092	Premier Medical Services	3,High Tension Line,Edet Akpan Avenue, Uyo Akwa Ibom State
180	AK/0093	Queen Esther Specialist Hospital	Ediye Samuel Street,Itiam Etoi, Uyo Akwa Ibom State
181	AK/0094	Sifon Clinic	No 1,Uteng Lane, Uyo Akwa Ibom State
182	AK/0095	St. Lukes Hospital	Anua P.M.B. 3, Uyo, Akwa Ibom State
183	AK/0096	St. Patrick`s Clinic &Maternity	15,Nsie Street Off Eyetong Road, Oron Akwa Ibom State
184	AK/0097	Staphender Specialist klinic	3,Ibokette Street Off Aka Road, Uyo Akwa Ibom State
185	AK/0099	Nissi Specialist Hospital	Plot 42,Line H, Ewet Housing Estate, Uyo Akwa Ibom State
186	AK/0105	Dyme Hospital & Maternity Ltd	10,Edem Urua Street, Uyo, Akwa-Ibom State
187	AK/0106	Trio Medical Centre & Maternity Ltd	Plot 1,Unit M, Ewet Housing Estate, Uyo
188	AK/0107	Coray Specialist Clinic & Maternity	8, Ibiam street, Uyo. Akwa Ibom state
189	AK/0109	RecoveryHill Nig. Ltd.	3, Idim Edoho Street, Iko Ekwa, Eket, Akwa Ibom State.
190	AK/0110	The Bridge Clinic Ltd	111B, Umuahia Road, Ikot Ekpene. Akwa Ibom state.
191	AK/0114	Cozar Specialist Hospital Ltd	45, Afia Nsit Road, Eket, Akwa Ibom State.
192	AK/0115	San Dominique Hospital	Plot 1, Unit M, Ewet Housing Estate,Uyo. Akwa Ibom state.
193	AN/0002	General Hospital, Awka	Awka, Anambra State
194	AN/0003	Harmony Hospital	No. 5 Oraogbu Street, Awka, Anambra State.
195	AN/0004	Beacon Hospital	12. Umudora Street, Awka, Anambra State.
196	AN/0005	Divine Hospital & Maternity	9 Ken Okoli Street, Off Works Road, Awka, Anambra State.
197	AN/0006	Silgrey Royal Hospital & Maternity	Ikegwuonu Close, Opp. Mission Rd. Awka, Anambra State.
198	AN/0008	Nnamdi Azikiwe University Teaching Hospital	Nnewi, Anambra State
199	AN/0012	General Hospital Onitsha	Hospital Road, Onitsha, Anambra State.

200	AN/0015	Menax Hospital	Onowu Anatogu, St. Woliwo Layout Onitsha,
201	AN/0016	Rush Green Maternity Hosp. & Clinic	64, Ugwunobamkpa Road, Near Isokwe Police Post, Onitsha.
202	AN/0017	New Hope Medical Centre	80, Modebe Avenue, Odoakpu, Onitsha, Anambra State.
203	AN/0018	Venik Specalist Hospital	No. 1 Olo Street, Omagba Layout Onitsha, Anambra State.
204	AN/0022	St. Lukes Hospital	7 Onwudiwe Street Onitsha, Anambra State.
205	AN/0027	Mezie Hospital & Maternity	103b Upper New Market, Onitsha, Anambra
206	AN/0028	Intensive Care Hospital	36, Oguta Road, Onitsha, Anambra State.
207	AN/0029	Ace Specialist Hospital	73b Limca Road/1umuoji Street Onitsha, Anambra State.
208	AN/0030	Obiwanne Hospital & Maternity	102 Limka Road, Nkpor Anambra State.
209	AN/0031	St. Annes Hospital & Maternity	7, Enugu Road, Nkpor, Anambra State.
210	AN/0032	Abazie Hospital(Formerly Head Bridge Hospital)	2 Abazie Lane, Off Our Lady''s Road Nkpor, Anambra State.
211	AN/0033	The Children's Hospital	11 Uli Street, Nnewinchi, Nnewi, Anambra State
212	AN/0082	302 FAR MRS Onitsha	Onitsha, Anambra State
213	AN/0083	14 FER MRS Onitsha	Onitsha, Anambra State
214	AN/0084	Cottage Hospital/Comprehensive Health Centre	Anambra State Police Command, Anambra State
215	AN/0085	Christ the King Hospital	Off Nkwura Street, Awka, Anambra State
216	AN/0086	Holy Rosary Specialist Hospital	Waterside, Along Bank of the Niger, Onitsha, Anambra State
217	AN/0087	Ifebi Medical Centre	16A, Igwebuike School Road, Awka, Anambra State
218	AN/0088	Beluchukwu Memorial Hospital	Nkwo, Enugu-Ukwu, Anambra State
219	AN/0089	Chukwunonso Hospital & Maternity	Nanka, Orumba, Anambra State.
220	AN/0090	Chukwunonso Hospital & Maternity	Oko, Ekwulobin
221	AN/0091	St. Charles Borromeo Hospital	Off Limca Road, Onitsha, Anambra State
222	AN/0092	St. Augustines Hospital & Maternity	Oko, Ekwulobia, Anambra State
223	AN/0095	Ugochukwu Hospital & Maternity	No. 3 Obinna Street, Nkpor, Anambra State
224	AN/0096	Chimaobi Hospital & Maternity	No. 6 Agba Str., Nnewichi, Nnewi, Anambra State
225	AN/0097	The Light Specialist Hospital	No. 40, Eme Court Rd., Umudim, Nnewi, Anambra State
226	AN/0098	Life Specialist Hospital	No. 7 Ikemba Drive, Umudim, Nnewi, Anambra State

227	AN/0099	Chimex Specialist Hospital	No. 87 Ezemewi Road, Nnewichi, Nnewi, Anambra State
228	AN/0100	King David Hospital	Agulu, Anambra State
229	AN/0123	Sefton Specialist Medical Centre	5, Onubuya Street, off Court Road, Akwa
230	AN/0124	Federal College of Education (Technical)	Umunze, Anambra State
231	AN/0127	DSS Clinic	Awka, Anambra State
232	AN/0128	Federal Polytechnic Med. Centre, Okoh	Okoh, Enugu State
233	AN/0129	St. Catherines Hospital & Maternity	Umumze, Anambra State
234	AN/0131	AWKA MEDICAL CENTER	HOUSE3,ROAD4,UDOKA HOUSING ESTATE,ANAMBRA STATE
235	AN/0132	CHIBUZOR HOSPITAL AND MAT.	34,ORLU ROAD, EKWULOBIA,ANAMBRA
236	AN/0133	CROWN HOSPITAL	UMUOJI ROAD,MKPOR, ANAMBRA STATE
237	AN/0134	DELTA HOSPITAL AND MAT.	21 ZIK AVENUE, FEGGE, ONITSHA
238	AN/0135	Dionye Memorial Hospital	OKPO VILLAGE,EKWULOBIA, ANAMBRA
239	AN/0136	GOD'S ESTATE SPEC. HOSPITAL	36, MOORE STREET, ODOAKPU
240	AN/0137	GOZIE SPECIALIST HOSPITAL	10A NEW CEMETARY ROAD, ONITSHA
241	AN/0138	IMMACULATE HEART HOSPITAL AND MAT.	OLD ENUGU-ONITSHA
242	AN/0140	Nnamdi Azikwe University Medical Centre	AWKA
243	AN/0142	PARAGON HOSPITAL AND MAT.	7, BISHOP SHANAHAM STREET, FEGGE, ONITSHA
244	AN/0144	TRINITY HOSPITAL	50 AMIKWO ROAD, AWKA
245	AN/0171	Apex Medical Centre	Igbo-Ukwu
246	AN/0173	Pioneer Int. Spec Hospital	Plot 193 Owele Ebo Estate, Onitsha
247	AN/0175	R N Afunogo Memorial Hospital	4B St Faith Road, Awka
248	AN/0177	Chidera Hospital	12 Metun Road Umuanuka, Otolo Nnewi
249	AN/0178	Urban Hospital & Maternity	Enugu/Onitsha Express Way
250	AN/0184	St Catherine Hospital & Maternity	Umunze Orumba
251	AN/0188	Divine Care Specialist Hospital & Maternity	77 Igwe Orizu Road, Otolo Nnewi
252	AN/0191	Bex Memorial Hospital	Km 1 Owerri Road, Onitsha
253	AN/0193	Iyienu Hospital	Iyienu Hospital Road, Ogidi
254	AN/0195	St Victoria Specialist Hospital & Maternity	61 Orlu Ekwulobia
255	AN/0198	Crest Specialist Clinic	20 Enweana Street B/D Cat Kwata, Awka
256	AN/0202	Infant Jesus Hospital & Children Clinic	1 Emenike Crescent, Oremohu Street, Awada
257	AN/0203	Bethsaida Specialist Hospital	Plot CL6, Pond Street Housing Estate, Feggae, Onitsha
258	AN/0205	Ogechukwu Hospital	26 Orlu Road, Ekwulobia
259	AN/0206	First Hospital & Maternity	Near St Michael Church, Nawfia
260	AN/0213	Chidera Specialist Hospital	Nkwo Uga, Uga
261	AN/0214	Our Lady Of Lourdes Hospital	Ihiala, Anambra State
262	AN/0215	St. Mary’s Hospital & Maternity	Agba Village, Ekwulobia, Anambra State.

263	AN/0216	His Glory Hospital & Maternity	96 Arthur Eze Avenue, near Tracy Hotel, Awka
264	AN/0217	Kanayo Specialist Hospital & Maternity	17 Enugu Road, Onitsha
265	AN/0219	Messiah Foundation Hospital	20 Awka Road, Ekwulobia
266	AN/0221	Mata Christi Hospital	73 Limka Road Nkpor
267	AN/0224	Obioma Specialist Hospital & maternity	19 Onwukwe Street Abunor Nnewichi,Nnewi Anambra State
268	AN/0225	St. Helen`s Specialist Hospital	Agbou Estate Behind Kenneth Dike Sec Sch Off Obinagu, Amawbia Awka Anambra State
269	AN/0226	Toronto Hospital limited	2,Upper Niger Bridge Onitsha Anambra State
270	AN/0227	Izunna Hospital And Maternity LTD.	Ezinalo Village Ifite Awka, Awka South Anambra State
271	AN/0228	Harmony Specialist Hospital &Maternity	5 Orogbu Awka Anambra State
272	AN/0229	Dayspring Cottage Hospital	Little-Wood Estate Umuota, Obosi Anambra State
273	AN/0232	All Saints Hospital & Maternity	Agulu (Nkitaku Village) Anambra State
274	AN/0233	Chukwunonso Hospital & Maternity	Nnobi Anambra State
275	AN/0234	Okpala Hospital And Maternity	Enugwu-ukwu Near Ezi-Elias Anambra State
276	AN/0235	Anambra State University Teaching Hospital	Awka Amaku Anambra State
277	AN/0236	Rock Foundation Hospital & Maternity	5,Igwebuike Road Awka Anambra State
278	AN/0237	Isaac Chira Memorial Hospital	Awkuzu Anambra State
279	AN/0238	Cynvic Specialist Hospital	General Hospital Road Agba Ekwulobia Anambra State
280	AN/0239	Madueke Memorial Hospital And Maternity	13,Sunny Dan Street Nkpor Anambra State
281	AN/0240	Our Lady Of Lourdes Hospital	Ihiala Anambra State
282	AN/0241	Uchenna Hospital & Maternity.	116, Onitsha Old Road Nnewi, Nnewichi-Nnewi Anambra State
283	AN/0242	Madonna Maternity Hospital	No. 2, Emma Nnaemeka Street Awka Anambra State
284	AN/0243	Prime Specialist Hospital	5, Nwufo Meniru Street Beside Bishop Crother Seminary, Awka Anambra State
285	AN/0244	Zion Hospital	Opp. General Hospital Enugwu Ukwu Anambra State
286	AN/0246	Lumen Christ Specialist Hospital	17, Atani Road Km 1 Iyiowa Odekpe Anambra State
287	AN/0247	Nnewi Diocesan Hospital	Igwe Orizu Road Otolo, Nnewi Anambra State
288	AN/0248	First Foundation Hospital & Mat	Ummunachi Anambra State
289	AN/0249	Bethsaida Specialist Hospital	48b Nnobi Road Opp. Nnewi High School, Nnewi Anambra State
290	AN/0250	Police Clinic/IGP M.D. Abubakar Medical Centre	CPS Onitsha Anambra State

291	AN/0253	Beke Memorial Hospital	64 F.G Onyiuke Avenue, Nimo Anambra State
292	AN/0254	Obioma Hospital	5/7 Anuka Road Otolo, Nnewi Anambra State.
293	AN/0255	Mercy Specialist Hospital And Maternity	4A New American Road, Onitcha, Anambra State
294	AN/0256	St. Joseph’s Hospital	Adazi Nnukwu, Anambra State
295	AN/0257	Regina Caeli Specialist Hospital	17, Regina Caeli Road, Awka Anambra State
296	AN/0258	Aguata Diocesan Hospital	Umunze, Anambra State
297	AN/0259	Rice Clinic	Rise- Gopat Building Agulu-Nnobi Road Adazi-Ani, Anambra State
298	AN/0260	Ezinne Hospital & Children Welfare Centre	1A Oboli Lane, DMGS Round About Onisha, Anambra State
299	AN/0261	Kandudi Specialist Hospital	Oye Market, Achina Anambra State
300	AN/0263	Dike Medical Centre	Ebenebe Anambra State
301	AN/0264	Kwenas Clinic & Maternity	Afor Ufuma Anambra State
302	AN/0265	Nwanja Allied Hospital	No 1 Modebe Avenue, Odoakpu Onitsha, Anambra State
303	AN/0267	Edozie Hospital & Maternity	5, Omanukwue Crescent, Obieze Village, Ifite Duru, Anambra State
304	AN/0268	Obijackson Hospital	No 2 Madonna University Road, Okija, Anambra State
305	AN/0269	All Hallow Hospital & Maternity	24B Court Road, Onisha Anambra State
306	AN/0273	Assumpta Clinic & Maternity	Charles Modozie Street, off Commissioners Quarters Road, Ifite Awka Anambra State
307	AN/0274	Aguata Diocesan Hospital Igboukwu	Divine Grace Villa 2 Rd 3 Abuja Estate Awka, Anambra State
308	AN/0275	Madonna Specialist Hospital Nig. Ltd	Divine Grace Villa 2 Rd 3 Abuja Estate Awka, Anambra State
309	BA/0001	Peoples Clinic Ltd.	Aminu Street Bauchi, Bauchi
310	BA/0002	Mai Jama'A Clinic	Wunti Street, Bauchi
311	BA/0003	Under Five (5) Health Care	Jahun Road, Bauchi
312	BA/0004	Urban Mat. & Child Welfare Clinic	Kofan Ran Bauchi, Bauchi
313	BA/0005	Reemee Medicare Nigeria Limited	6, Rimi Road, Gra Bauchi
314	BA/0006	Abubakar Tafawa Balewa University Teaching Hospital	Bauchi, Bauchi State
315	BA/0018	Shifa'A Medical Clinic	Sule Katagum Road, Azare
316	BA/0020	Mainiima Consultant Clinic - Azare	Abdulkadir Street Azare, Bauchi State
317	BA/0023	Niima Consultant Hospital - Bauchi	Niima Close, Off Airport Rd. Bauchi
318	BA/0024	DSS Clinic	Bauchi, Bauchi State
319	BA/0025	Federal Medical Centre - Azare	P.M.B. 005, Azare, Bauchi State.
320	BA/0026	Bauchi State Specialist Hospital	Nassarawa Jahun Road, Bauchi
321	BA/0027	33 AB MRS Bauchi	Bauchi, Bauchi State

322	BA/0028	211 TK Bn MRS, Bauchi	Bauchi, Bauchi State
323	BA/0029	301 SPAR GS Bauchi	Bauchi State
324	BA/0030	ACCS MIR Bauchi	Bauchi, Bauchi State
325	BA/0032	Bauchi State Police Clinic	Bauchi, Bauchi State
326	BA/0033	Abubakar Tafawa Balewa Health Centre	Bauchi, Bauchi State
327	BA/0035	RAILWAY HOSPITAL, BAUCHI	RAILWAY COMPOUND
328	BA/0036	PAHLYCON CLINICS	SABUWAN KASUWA RAILWAY ROAD, BAUCHI
329	BA/0037	Federal Polytechnic medical center, bauchi	BAUCHI
330	BA/0040	GENERAL HOSPITAL NINGI	NINGI- BAUCHI STATE
331	BA/0041	GENERAL HOSPITAL MISAU	MISAU- BAUCHI STATE
332	BA/0042	GENERAL HOSPITAL SHIRA	SHIRA - BAUCHI STATE
333	BA/0043	GENERAL HOSPITAL TORO	TORO - BAUCHI STATE
334	BA/0044	GENERAL HOSPITAL TAFAWA BALEWA	TAFAWA BALEWA - BAUCHI STATE
335	BA/0045	GENERAL HOSPITAL KATAGUM	KATAGUM - BAUCHI STATE
336	BA/0046	GENERAL HOSPITAL DARAZO	DARAZO - BAUCHI STATE
337	BA/0047	GENERAL HOSPITAL DASS	DASS - BAUCHI STATE
338	BA/0048	GENERAL HOSPITAL JAMA'ARE	JAMA''''ARE - BAUCHI STATE
339	BA/0049	GENERAL HOSPITAL GAMAWA	GAMAWA - BAUCHI STATE
340	BA/0050	GENERAL HOSPITAL ALKALERI	ALKALERI - BAUCHI STATE
341	BA/0051	GENERAL HOSPITAL ITAS	ITAS - BAUCHI STATE
342	BA/0053	General Hospital Bayara	Bauchi
343	BA/0054	Model Primary Health Centre, Yelwan Duguri	Yelwan Duguri
344	BA/0055	Ibrahim Bako Primary Health Centre	Ibrahim Bako Road
345	BA/0056	Family Planning Clinic, Kofar Wase	Kofar Wase
346	BA/0057	Model Primary Health Centre, Nasaru Village	Nasaru Village
347	BA/0058	Model Primary Health Centre, Tsangaya Village	Tsangaya Village
348	BA/0059	Model Primary Health Centre, Ganjuwa	Mia Village Ganjuwa
349	BA/0060	Model Primary Health Centre, Dambam	Jalam Village, Dambam
350	BA/0061	Model Primary Health Centre, Yayu	Yayu Village
351	BA/0062	Model Primary Health Centre, Zaki	Murmur Village
352	BA/0063	Model Primary Health Centre, Giade	Zabi Village, Giade
353	BA/0064	Model Primary Health Centre, Shira	Faggo Village
354	BA/0065	Urban Maternity, Azare	Azare Village Katagum
355	BA/0066	Yahaya Clinic, Jama'are	Federal Low Cost, Talba Road, Jama''''''''are
356	BA/0067	Alwadata Consultant Clinic	7 KWANI CLOSE FADAMI CLOSE MADA BAUCHI
357	BA/0069	Abubakar Tatari Polytechnic Medical Centre	Office of The Registrar, Wuntin Dada, Jos Road, Bauchi
358	BA/0070	General Hospital, Kafi Madaki	Kafi Madaki
359	BA/0071	General Hospital, Burra	Burra
360	BA/0072	General Hospital, Boto	Boto
361	BA/0076	Gama Clinics	Daba Makaho Street, Azare
362	BA/0083	Albishir Clinic & Maternity	2 Bununu Road, Behind Police Outpost, GRA Bauchi
363	BA/0084	Medical Centre College of Education	College of Education, Azare

364	BA/0086	Shifa Royal Hospital	Bauchi, Bauchi State
365	BA/0087	Ardo Standard Medicare Hospital	NO 42, S/Abdulkadir Street Azare, Katagum LGA, Bauchi State
366	BA/0088	Al-Ameen Hospital LTD	2, Nassarawa Jahun , Bauchi, Bauchi State.
367	BA/0089	Wadhum & Wayo Memorial Clinic &Maternity	144, Ungwan Taskani, Yelwa Tudun Bauchi, Bauchi State.
368	BA/0090	Apple Clinic And Maternity	No 3, Lame Close, off Sunday Awoniyi St, New GRA, Bauchi, Bauchi State
369	BA/0092	National Fistula Clinic	Ningi, Bauchi State.
370	BN/0003	TBT Hospital	91, Cape Doanes Way, Gboko
371	BN/0004	Myom Hospital	General Hospital Road, Gboko
372	BN/0007	Baki Hospital	Opp. Base Garden GRA, Gboko
373	BN/0012	Federal Medical Centre - Makurdi	Makurdi, Benue State
374	BN/0015	Madonna Health Service Ltd.	(Madonna Hospital) New Bridge Road, Behind Union Bank Mkd
375	BN/0016	Jolua Clinic & Maternity	No.9 Maccathy Close, Makurdi
376	BN/0017	Lobi Hospital	High Level, Makurdi
377	BN/0018	Bishop Murray Medical Centre	Close To St. Theresa Church, High Level Makurdi
378	BN/0019	Sandra Hospital	24, J.S. Tarka Road, Opp. Maltino Filling Station, Makurdi
379	BN/0020	Immaculate Conception Hospital	Walla Mayo, High Level, Makurdi
380	BN/0021	Judita Hospital	No.4, Gondo Road, Makurdi
381	BN/0023	Family Practice Hospital	4, Landar Close Ankpa Quarters, North Bank, Makurdi
382	BN/0024	Homa Hospital	Hudco Quarters, High Level , Makurdi
383	BN/0025	Hemko Hospital	Kanshio, Makurdi
384	BN/0026	El-Shaddai Specialist Hospital	Plot 2319, Kanshio Makurdi
385	BN/0028	Pampas Hospital & Maternity	27, Iyorchia Ayu Road, Wurukum Makurdi
386	BN/0029	Queen's Clinic	16, Railway Close, High Level Makurdi
387	BN/0030	Grace Cottage Hospital	7, David Mark Bye Pass, Makurdi
388	BN/0052	St. Daniel's Hospital	Behind Elipi Motor Park, Otukpo
389	BN/0053	Abo Clinic & Maternity	Along Enugu Expressway, Otukpa Branch, Otukpa
390	BN/0054	Salem Hospital	G.S.S Road, Otukpo
391	BN/0059	45 Nigerian Airforce Hospital	Air Force Base, Makurdi, Benue State
392	BN/0060	Federal Staff Clinic	Federal Secretariat, Makurdi, Benue State
393	BN/0061	72 Para Bn MRS Makurdi	Makurdi, Benue State
394	BN/0063	NASME MIR Makurdi	Makurdi, Benue State
395	BN/0064	Cottage Hospital/Comprehensive Health Centre	Benue State Police Command, Makurdi, Benue State
396	BN/0065	Benonil Hospital	7, Zion Str Wurukum Makurdi
397	BN/0066	Benue Women Clinic	35, Modern Market Rd Makurdi

398	BN/0067	Kings Cross Clinic	20, Badagry Str High Level Makurdi
399	BN/0068	Rahama Clinic & Maternity	12, Brewery Qtrs Opp Benue State University Medical School Gboko Rd Makurdi
400	BN/0069	Multicare Hospital	Ogbaji Jonah Str Behind LG Commission Makurdi
401	BN/0080	University of Agriculture, Makurdi	Makurdi, Benue State
402	BN/0084	DSS Clinic	Makurdi, Benue State
403	BN/0091	winners hospital	10, J.S Tarka road, Makurdi
404	BN/0096	OTABO HOSPITAL	Effoyo Edumoga, Benue state
405	BN/0097	New Era clinic	11, Agbo street, Sabon Gari, Oturkpo
406	BN/0098	Mount Zion Gate Hospital Ltd	38, Ijami Road, Otukpo
407	BN/0109	Clinic of Divine Love	33 Old Oturkpo Road, Makurdi
408	BN/0111	Ihotu Clinic & Maternity	63 Upo Road Oturkpo
409	BN/0114	St Mary's Hospital	Ichama Road, Okpoga
410	BN/0116	Alpha Hospital & Maternity	Efoga-Edumoga, Along Old Oturkpo Road
411	BN/0122	Powaren Hospital	9, J.S. Tarka way, Gboko, Benue state
412	BN/0123	Lord is Saviour Clinic & Maternity	34B, Mkar Road, Gboko town, Benue State.
413	BN/0125	Healing Hand Specialist Hospital	71, Adikpo Street, Makurdi, Benue State.
414	BN/0126	Nation’s Hospital &Eye Clinic	Ankpa Qtrs Extension, Behind BEM hotel, Makurdi, Benue State.
415	BN/0128	Hope Hospital	2, Akure Street, Wadata, Makurdi
416	BN/0129	Kings Park Polyclinic	Karanga Akanya Street, Off Abu King Shuluwa Road
417	BN/0130	Pishon Women Specialist Clinic	12, Silas Ebute Street, Off Modern Market Road, Makurdi, Benue State.
418	BN/0131	Delight Specialist Clinic &Maternity	No 3, Gilga Street, Beside Benue Investment Building, Otukpo Road, Benue State.
419	BN/0133	Dora Specialist Hospital	Off High Court Road, North Bank, Makurdi, Benue State.
420	BN/0134	Benue State University Health Services Directorate	Benue State University , Makurdi, Benue State.
421	BN/0136	St. Monica’s Hospital	Opp. St. Anne’s Church, Off Adikpo-Vandeikya Road Adikpo Benue State
422	BN/0137	NKST Hospital	Mkar- Gboko , Benue State.
423	BN/0139	Gracely Hospital And Medical Services.	NO. 5 Adzaage Hinden Street, Behind Fed. Pay office Makurdi Benue State
424	BN/0141	Adoose Specialist Hospital	No. 12 Keghem Malu Street New GRA Makurdi Benue State.

425	BN/0142	Ushakaa Hospital	Behind Civil Service Commission Old Otukpo Road, Makurdi Benue State
426	BN/0144	Service Assurance Specialist Hospital (S. A. S. H).	Beside Adonai Service Station Opp. Modern Market Junction, Manor Quaters Makurdi, Benue State.
427	BN/0145	N. K. S. T Health Clinic.	Achia, Usambe Ikurau-ya, Kwande, Benue State
428	BN/0146	Otia Hospital.	1, Kano Street Otukpo, Benue State
429	BN/0147	Oyare Hospital And Maternity	No. 4, Chief Omakpo Avenue Otukpo, Benue State.
430	BN/0148	Adiyama Hospital	62, SEC Road Oju, Benue State.
431	BN/0150	Vineyard Health Centre.	Km. 16, Makurdi- Aliade Rd. Opp. NNPC Depot, Makurdi, Benue State.
432	BN/0153	Amazing Grace Hospital.	No. 16 Uke Wende Street High Level, Makurdi Benue State.
433	BN/0154	St. Vincent Hospital.	Aliade, Benue State
434	BN/0155	First Fertility Hospital Ltd.	Plot BNB 5827, Behind Tile Ankpa Ward Benue State
435	BN/0156	N K S T Hospital, Zaki Biam	Off Wukari Road Zaki Biam, Benue State.
436	BN/0157	N K S T Hospital, Mbaakon.	N K S T Hospital, Mbaakon
437	BN/0158	Myom Hospital	No. 28, General Hospital Road Gbako, Benue State
438	BN/0159	Victory Hospital	Off General Hospital Gboko,Benue State.
439	BN/0160	Wurukum Specialist Clinic	Lortyom. Beegu Close, Off Abu King Shuluma Rd. Akpehe Near Police & Division Makurdi, Benue State.
440	BN/0162	Benue State University Teaching Hospital.	BSUTH Outreach Station, Benue State
441	BN/0163	Benue State University Teaching Hospital.	BSUTH Gboko rd. Behind Tile Ankpa Ward Makurdi, Benue State.
442	BN/0167	St. Vincent Hospital Aliade	Aliade-Otukpo Road Benue State
443	BN/0169	St. Gregory Hospital Ikpayongo	Ikpayongo Benue State
444	BN/0170	General Hospital North Bank	North Bank ,Markudi Benue State
445	BN/0172	Gilead Dental Services	No.6 Kwararafa Quaters Markudi Benue State
446	BN/0176	General Hospital Buruku	Buruku Benue State
447	BN/0178	General Hospital Naka	Along Adoka Road,Naka Benue State
448	BN/0179	Fr. Mathias Healthcare Unit	St. Theresa Catholic Church, Naka, Benue State
449	BO/0001	General Hospital, Askira/Uba	Askira Uba, Lassa Town

450	BO/0002	General Hospital, Bama	Bama Gulumba Road, Bama Town
451	BO/0003	General Hospital, Biu	Along Yola Road, Biu
452	BO/0005	General Hospital, Gwoza	Gwoza Town, Maiduguri
453	BO/0007	University Of Maiduguri Teaching Hospital	Along Bama Rd., Maiduguri
454	BO/0009	General Hospital, Kukawa	Along Kukawa-Damasak Road, Kukawa
455	BO/0011	Taimakon Jama'A Clinic & Mat.	Maiduguri, Borno
456	BO/0012	Borno Medical Clinic	No.8, Sir Kashim Ibrahim Way
457	BO/0013	New Foundation Hospital Limited	No. 340, Sabon Lamba Street, Galadima Junction, Maiduguri
458	BO/0014	State Specialist Hospital	Along Shehu Laminu Way
459	BO/0015	Nursing Home, Maiduguri	Behind Nhis Zonal Office, Off Shehu Laminu Way, Maiduguri
460	BO/0016	Ayamsu Memorial Medical Centre	17 Bukar Bolori Street, Kumshe Ward Maiduguri
461	BO/0017	Kanem Hospital & Maternity	152, Tafawa Balewa Street, Maiduguri
462	BO/0018	Nakowa Specialist Hospital	Along Govt. House Road, Maiduguri
463	BO/0019	Zaman Clinic & Annex	326, Waziri Kyari Drive, Gamboru Ward, Maiduguri
464	BO/0028	General Hospital Monguno	Along Maiduguri-Baga Expressway, Maiduguri
465	BO/0029	General Hospital, Ngala	Ngala
466	BO/0030	Federal Neuropshychiatry Hospital	Off, Baga Road, Fed. Low Cost, Maiduguri
467	BO/0047	DSS Clinic	Maiduguri, Borno State
468	BO/0048	204 WG Medical Centre	NAF Station, Maiduguri
469	BO/0051	City Medical Centre	Galadima Ward, Borno State
470	BO/0053	333 AR MRS Maiduguri	Maiduguri, Borno State
471	BO/0054	21 Bde MC Maiduguri	Maiduguri, Borno State
472	BO/0055	212 Bn MRS, Maiduguri	Maiduguri, Borno State
473	BO/0056	331 AR MRS Biu	Borno, Borno State
474	BO/0057	202 Bn MRS Bama	Bama, Borno State
475	BO/0058	243 Bn MRS Monguno	Monguno, Borno State
476	BO/0059	231 TK Bn MRS Biu	Biu, Borno State
477	BO/0060	Police Hospital Alkome	Maiduguri, Borno State
478	BO/0061	Cottage Hospital/Comprehensive Health Centre	Police Training School Gwoza, Borno State
479	BO/0062	Alkome Polyclinic	Baga Road, Maiduguri, Borno State
480	BO/0063	Sunni Hospital	Abba Ganaram, Kashim Ibrahim Way, Maiduguri, Borno State
481	BO/0065	Kwatam Medical Clinic	Baga Road, Maiduguri, Borno State
482	BO/0066	Gospel Mission to Nigeria	G260 Galdimare, Biu, Gombe State
483	BO/0067	Hirku Clinic & Maternity	Damaturu Road, Biu, Borno State
484	BO/0092	Abbott Clinic & Maternity	Damaturu Road, Biu, Borno State

485	BO/0093	University of Maiduguri Staff Clinic	Bama Road, Maiduguri, Borno State
486	BO/0095	Nig Arabic Language Village Hospital	Ngalla Borno
487	BO/0096	General Hospital Damboa	Damboa, Borno State
488	BO/0097	General Hospital Marama	Hawul, Borno State
489	BO/0098	General Hospital Dikwa	Dikwa, Borno State
490	BO/0099	General Hospital Kwaya Kusar	Kwaya Kusar, Borno State
491	BO/0100	General Hospital Shani	Shani, Borno State
492	BO/0101	General Hospital Konduga	Konduga, Borno State
493	BO/0102	General Hospital Damasak	Damasak, Borno State
494	BO/0103	General Hospital Gubio	Gubio, Borno State
495	BO/0104	General Hospital Nganzi	Nganzi, Borno State
496	BO/0105	General Hospital Marte	Marte, Borno State
497	BO/0106	General Hospital Ngoshe	Ngoshe, Borno State
498	BO/0107	General Hospital Benisheik	Benisheik, Borno State
499	BO/0108	General Hospital Kirawa	Kirawa, Gwoza, Borno State
500	BO/0109	General Hospital Magumeri	Magumeri, Borno State
501	BO/0110	General Hospital Askira	Uba Askira, Borno State
502	BO/0111	DOMINION MATERNITY HOME	OFF NAF BASE, BULUNKUTU
503	BO/0113	UNITYY CLINICS & MAT.	OLD GRA, NEAR GOVT HOUSE, MAIDUGURI
504	BO/0138	Police College Clinic	Maiduguri
505	BO/0140	Horama Hospital & Maternity	Off Lagos Street, Maiduguri
506	BO/0141	Police College Clinic	Maiduguri, Borno
507	BO/0142	Royal Specialist Hospital	Opp Gombole Road, Maiduguri
508	BO/0143	Amir Medical Clinic	Tashan Damboa Maiduguri
509	BO/0144	Mustashfa Specialist Hospital	Damboa-Biu Road Behind Indimis House, Maiduguri
510	BO/0146	Heman Hospital & Diagnostic Centre	New GRA, Opp, Bama rd motor park, Maiduguri
511	BO/0147	New Ultra Metroplitan Clinic & Maternity	1, Chez Coan Junction, FNPH Rd, Maiduguri
512	BO/0148	26 Task Force Brigade Medical Centre	Gwoza Borno State
513	BO/0149	7 Division Hospital	Maimalari Military Cantonment, Maiduguri.
514	BO/0150	Alheri Hospital	Bolori Market Road, Maiduguri
515	BO/0151	Umar Shehu Ultramodern Hospital	Jos Road, Bulunkutu
516	BO/0152	Falala Specialist Medical Clinic	Mando Road, Polo GRA, Maiduguri
517	BY/0002	Asueifa New Life Clinic	9, Asueifa hospital street, Bay bridge, Yenizue-Gene , Yenagoa
518	BY/0003	Kuro Specialist Hospital	16, Otiotio road, Yenizue-Gene, Yenagoa
519	BY/0004	Crest Consultant Hospital and Fertility CenterClinic	Yenagoa
520	BY/0006	Odi General Hospital	Odi
521	BY/0007	St. Peter's Hospital	Amarata, Yenogoa
522	BY/0008	Everly Medical Centre	Yenegoa Opp. Boat Yard
523	BY/0009	Palen Hospital	Ekeki Yenogoa

524	BY/0010	Mieye Medical Centre	630 Chief Melford Okilo Road, (Before Commisioner's Estate junction Opolo Yenagoa
525	BY/0011	Opuere Mat. & Nursing Home	Commisioners Estate, Opolo, Yenagoa
526	BY/0012	Tobis Clinic	KM 14, Melford Okilo Road, Akenfa Yenogoa
527	BY/0013	New Uchenna Hospital	Imgbi Road Amerata Yenagoa
528	BY/0016	Our Saviour's Clinic	Kisma
529	BY/0017	Alfa Clinic	Off Imiringi Road, (Before Mechanic Village) Yenogoa
530	BY/0056	DSS Clinic	Yenegoa, Bayelsa State
531	BY/0057	Federal Medical Centre, Yenagoa	Yenagoa, Bayelsa State
532	BY/0058	Trinidex (Trinity) Medical Centre	1 Didie Hotel Road (1st Upstair by the Left) Yenagua
533	BY/0060	Police Clinics, Bayelsa	Bayelsa State
534	BY/0061	Gloryland-inri med. Center	7 DSP Alameseigha Express way by Hospital Junction, Yenagoa
535	BY/0065	Federal Polytechnic Ekowe Clinic	Federal Polytechnic, Ekowe
536	BY/0066	Federal University Medical Centre	Otuoke
537	BY/0067	Yenogoa Hospital & Maternity	Yenogoa, Bayelsa
538	BY/0068	Kokobo Specialist Clinc	Mbiama- Yennagoa Road, Akenfua Bayelsa State.
539	BY/0069	New Survivor`s Hospital.	New Survivor`s Hospital Road, Igbogene Bayelsa State.
540	BY/0070	Ripus Hospital LTD	No. 35 Otitio Road, Yenagoa Bayelsa State.
541	CR/0001	General Hospital,Mary Sclessor Avenue	Mary Sclessor Avenue, Calabar
542	CR/0003	Hannah Foundation Clinic & Trauma Centre	13 Akim Close, Housing Estate, Calabar
543	CR/0004	Faith Foundation Specialist Clinic	57 Ndidem Isang, Isong Road, Calabar
544	CR/0005	Peace Medical Centre	17 Efut Street, Calabar
545	CR/0006	City Clinic	101 Ndidem Isang, Isong Road, Calabar
546	CR/0007	Bakor Medical Centre	141, Ndidem Isand, Isong Road, Calabar
547	CR/0008	Mevon Specialist Clinic	30 Akim Road, Calabar
548	CR/0009	Goldie Clinic	137, Goldie Street, Calabar
549	CR/0010	Efkam Clinic	94 Ndidem Usang Iso Road, Calabar
550	CR/0011	Madonna Specialist Hospital Nig. Ltd.	Divine Grace Villa, 57, Murray Street, Calabar
551	CR/0012	Canaan Medical Centre	20b Iso Oqua, Big Qua Town,Calabar

552	CR/0013	Ideal Medical Centre	30 Mbukpa Road
553	CR/0014	Mambo Clinic & Maternity	12 Atamunu Street
554	CR/0032	Melrose Hospital	5 Melrose Avenue (Enim Okpa) F/C. Ikom
555	CR/0033	Benson Clinic & Maternity	Opp. Motor Park, Four Corners, Ikom
556	CR/0039	Sacred Heart Catholic Hospital	Ranch Road, Obudu
557	CR/0041	Monaiya Catholic Hospital & Maternity	Igoli, Ogoja
558	CR/0042	General Hospital, Igoli	Abakpa Road, Igoli, Ogoja
559	CR/0043	Santa Maria Clinics	11 Erokoro Street, Abakpa, Ogoja
560	CR/0046	Danex Medical Centre	5 Danex Road Edenkokol, Ijiman, Ugep
561	CR/0048	Comprehensive Health Centre	(Poly Clinics) Ooba Okpoma, Yala
562	CR/0049	Offoboche Specialist Hospital & Maternity	Ijaja Estate, Okuku, Yala
563	CR/0050	Lutheran Hospital & Maternity	Abakaliki Road, Yahe, Yala
564	CR/0051	County Specialist Hospital	18 Calabar Road, Ikom
565	CR/0052	Mount Zion Medical Centre	28b Yellow Duke Street, Calabar
566	CR/0056	NAF Medical Centre	NAF Station, Calabar
567	CR/0058	University of Calabar Teaching Hospital	Moore Road, Calabar, Cross River State.
568	CR/0061	13 Bde MC Calabar	Calabar, Cross River State
569	CR/0062	146 Bn MRS, Calabar	Calabar, Cross River State
570	CR/0063	245 Recce Bn MRS, Ikom	Ikom, Cross River State
571	CR/0064	130 Bn MRS Ogoja	Ogoja, Cross River State
572	CR/0065	341 AR MRS Ogoja	Ogoja, Cross River State
573	CR/0066	University of Calabar Health Centre	Calabar, Cross River State
574	CR/0068	Navy Medical Centre	Calabar, Cross River State
575	CR/0069	Dynasty Medical Centre	73 Ediim-Otop Street, Calabar, Cross River State
576	CR/0070	Nigerian Navy Medical Clinic	7, Club Road, Calabar, Cross River State
577	CR/0071	Immanuel Infirmary	No. 1 Assemblies of God Rd., Army Junction, Calabar
578	CR/0072	Obudu Clinic Ltd	105-108 Ogoja Road, Obudu, Cross River State
579	CR/0073	Peoples Specialist Clinic	48 Akim Road, Calabar
580	CR/0074	Bassey Enon Medical Centre	11B Yellow Duke Street, Calabar, C/River State
581	CR/0075	Ikpeme Medical Centre	18/20 Ambo Street, Calabar, Cross River State

582	CR/0076	Victoria Itam Hospital	8A Ediba Lane, Big Qua Town, Calabar, C/River State
583	CR/0083	DSS Clinic	Calabar, Cross River State
584	CR/0084	Police Clinics, Cross River	Cross River State
585	CR/0085	Police Children School Clinic, Calabar	Cross River State
586	CR/0086	Amazing Grace Specialist Clinic	45, Ikot Uduak Street, Off MCC Road, Calabar, C/River State
587	CR/0087	Heritage Specialist Clinic	64, Ekpo Abasi Street, Calabar, Cross River State
588	CR/0088	Testimony Medical Resource	58, Atakpa Street, Calabar, Cross River State
589	CR/0089	Mutanda Medical Centre	9, Edgerley Road, Calabar, Cross River State
590	CR/0090	Pathfinder Clinic	30 Atamunu Street, Calabar, Cross River State
591	CR/0091	St. Joseph Hospital	Ikot Ene Ekpabuyo, Cross River State
592	CR/0092	Comprehensive Health Centre (UCTH Annex)	Okoyong, Calabar, C/River State
593	CR/0093	General Hospital Akamkpa	Akamkpa, C/River State
594	CR/0094	General Hospital Ugep	Obol Ubi Ujong Avenue, Ugep
595	CR/0095	General Hospital Obubra	Obubra, C/River State
596	CR/0096	Holy Family Catholic Hospital	Beside Holy Family Parish, Ikom, C/River State
597	CR/0097	Ranch Medical Centre	Obudu Cattle Resort, Obanliku, C/River State
598	CR/0098	Eja Memorial Joint Hospital	Itigidi, Abi LGA, C/River State
599	CR/0099	Union Medical Centre, Calabar	104 Ndidem Island, Isong Road, Calabar, C/River State
600	CR/0100	Cottage Hospital Akpet Central	Akpet Central, Calabar, C/River State
601	CR/0101	Ultimate Medical & Consultant	Plot 328 Unit B Effanga, Mkpa State Housing Estate, Calabar
602	CR/0123	Group Specialist Clinic	Ndidem Usang Iso Road, Calabar
603	CR/0124	Mission Hill Clinic & Maternity	8, Miles, Calabar
604	CR/0125	Ussy Medicals Consulting Room	no. 3 Baney Ntui Street, Isong Inyang Akampa.
605	CR/0126	First Foundation Clinic	Ikom
606	CR/0127	Obim Medical Centre	Ikom
607	CR/0128	Ekana Medical Centre	Ogada, Obubra
608	CR/0129	Simba Clinic and Maternity	Mkpani Junction
609	CR/0130	Nkem Clinic & Maternity	94, Ikom - Calabar Road, Ugep.
610	CR/0131	Mercy Clinic & Maternity	No. 1 Ministry of Works Road, Igoli, Ogoja
611	CR/0132	Egozi Clinic	Magistrate Road, Ugep, Yakurr

612	CR/0133	Awukams Clinic	No.7 Awukams Crescent off Ogoja Road, Ikom.
613	CR/0134	Kusaki Medical Services (ABEK)	Ikom
614	CR/0135	Integrity Clinic	Behind Dapani Hotel, Ikom
615	CR/0136	Jody Clinic	Okundi, Boki LGA
616	CR/0137	Ubi Medical Consult	24 Old Market Road Ijom - Ugep
617	CR/0138	Pace Clinic	96 Mcc Road Calabar
618	CR/0139	St Peter Hospital	Idum-Mbube Ogoja
619	CR/0142	NNSS Sick Bay	Calabar
620	CR/0144	Bassey Enun Specialist Clinic Annex	Bakpan Biase Cross River State.
621	CR/0145	Alice Effanga Okon Specialist Clinic	No.4 Ekenglwatt Street, Calabar South, Cross River State
622	CR/0146	General Hospital Bekwara	Abochichi Cross River State
623	CR/0147	Progress Clinic And Maternity	No.4, Edim Ekong Oqua, Off Eta Agbor, Cross River State.
624	CR/0148	Luksana Foundation Medical Centre	1,Ntol Mgbeje Street, Igoli, Ogoja Cross River State
625	CR/0149	Calabar Women and Children Hospital	164/165 Murtala Mohammed Way Calabar
626	CR/0152	Luciama Memorial Specialist Hospital	No.10 Luciama Hospital Road, Off Water Intake Road-Ikot Effanga, Calabar Cross River State.
627	CR/0153	Angels Clinic	No.10 Ikom-Calabar Road Ugep Cross River State
628	CR/0154	Nigeria Customs Service Medical Centre	Nigeria Customs Service, Opp. Airport, Calabar Cross River State
629	CR/0155	Bakor Hospital Ltd	No.7,Hospital Road, Nde 3,Ikom Cross River State
630	CR/0158	Mary Mother Of Mercy Specialist Clinic	14, Ukpong Archibong Street, Essien Town, Calabar Cross River State.
631	DT/0001	May Flower Clinic & Maternity	54 Nnebisi Road, Off Cable Point Asaba
632	DT/0002	Good Samaritan Hospital	3 Good Samaritan Rd, Cable Point-Asaba
633	DT/0004	Crest Clinics	30, Ezenei Avenue, Asaba
634	DT/0012	Oghor Specialist Clinic & Hospital	Izobo Street, Effurum
635	DT/0014	Caboc Dental Clinic	Plot 540, 12th Ddpa, Airport Road, Effurum
636	DT/0023	Sapele Clinic	181, New Ogorode Road
637	DT/0027	Kwofia Clinic And Maternity	3, Kwofia Close, Po Box 35, Ovwian, Delta St.
638	DT/0029	Ufor Hospital	19, Uloho Avenue Off Isoko Rd Ughelli
639	DT/0032	Erada Clinic And Maternity	9, Marioghae Str., Atamakolomi Layout,Off Udu Rd, Enerhen, W
640	DT/0033	Estate Specialist Hospital	47, Onofegbara Str., Polokor Market, Warri

641	DT/0035	Twins Clinic/Maternity Annexe	No. 1, Twins Clinic Drive, Off Deco Rd., Warri
642	DT/0036	Fountains Clinic And Maternity	1, Enughe Str., Off Ogborikoko Road, Warri
643	DT/0037	Elohor Clinic And Maternity	No. 200 Old Ughelli-Warri Road, Oviri-Agbarho, Delta State
644	DT/0038	Deko Specialist Clinic	1, S. O. Agbowu Str .,By Efejuku Str, Off Deco Road, Warri
645	DT/0039	Winrose Clinic Limited	Enerhen Road, Effurum
646	DT/0060	Federal Medical Centre Asaba	Asaba
647	DT/0063	Sage Clinic	29 Okere/Ugborikoko Road Okumagba Layout, Warri
648	DT/0064	Central Hospital Warri	1 Mabiaku RD NPA, Warri, Delta State
649	DT/0065	Capitol Hill Clinic	2 Omamofe Silo Str Warri
650	DT/0066	Agbawus Medical Centre	3, Ogbo Drive, Ekurede, Warri
651	DT/0068	Goodnews Hospital	Omatar Street, Off Refinery Road by WOPOT Company, Ekpan, Warri, Delta State
652	DT/0069	Etefia Clinic	25, Oruhworun Road, Po Box 12, Ovwian, Warri
653	DT/0070	Veenell Hospital	Okumagba Avenue, Warri
654	DT/0083	DSS Clinic	Asaba, Delta State
655	DT/0084	Central Hospital Ughelli	Ughelli, Delta State
656	DT/0088	Humanity Hospital	No. 3 Enere Road, Warri, Delta State
657	DT/0090	Westend Hospital	2, 25th Street, Airport Road, Warri, Delta State
658	DT/0092	Ame Specialist Hospital	15, Okumagba Avenue, Warri, Delta State
659	DT/0093	93 Bn MRS, Warri	Warri, Delta State
660	DT/0094	Navy Medical Centre	Nigerian Naval Engineering College, Naval Base, Ogorode, Sapele Delta State
661	DT/0095	Sick bay NNS DELTA	Warri, Delta State
662	DT/0096	Ebony Clinic & Maternity	109 Okumagba Avenue, By Ojabugbe Junction Warri, Delta State
663	DT/0098	Radio Road Clinic & Maternity	9 Radio Road, Off Lower Erejinwa Road, Warri, Delta State
664	DT/0099	Ogana Memorial Clinic & Maternity	33, Ginuwa Road by Ogboru Junction, Delta State
665	DT/0100	St. Georges Specialist Clinic & Maternity	4, Idoma Street (Opp. Palm Grove Motel Gate), Warri, Delta State
666	DT/0101	Vertimon Medical Centre	3 Merogun Srt, Warri, Delta State
667	DT/0102	Day Spring Clinic	Plot 57C 14th Street, D.D.P.A., Effurun, Warri, Delta State
668	DT/0103	Lily Clinic/Hospital	6 Brisbe Street Off Deco Rd, Warri Delta
669	DT/0111	Petroleum Training Institute (Hospital)	Effurun, Delta State.

670	DT/0112	Police Clinics, Delta	Delta State
671	DT/0113	Camp Clinic	Steel Town 2, Ohioworun, Delta State
672	DT/0114	Fairview Hospital	1A, Akpu Street, Okumagba Avenue, Warri, Delta
673	DT/0115	GN-Children's & Gen. Med. Cli	3, Enakeyarhe-Ukuwere Street, DDPA, Ugborikoko, Effuru
674	DT/0116	Group Christian Hospital	Inside Mosheshe Estate, off Airport Road, Effurun
675	DT/0118	Kings Clinic	100, Okere Road, Warri
676	DT/0119	Kowa Clinic Ughelli	15, Isoko Road, Delta State
677	DT/0120	Lonia Clinic & Maternity	143 Ekete-DSC Express Way, Ovwian, Warri
678	DT/0121	Madona Clinics	60, Enerhen Road, Delta
679	DT/0124	Pearl Clinic	Plot 621, Nienogha Avenue, Delta
680	DT/0125	Regal Clinic & Maternity	226, Jakpa Road, Effurun, Delta State
681	DT/0126	Rideno Clinic	26b, Aragba Road, Orhuwhorun
682	DT/0127	Solar Clinic	1, Erhie Street, off Giwa Amu Road, Warri
683	DT/0129	St. Joseph Catholic Hospital	142, Nnebisi Road, Delta
684	DT/0130	St. Louis Clinic	4, Okundolor Street, off Ogbunu Road, Warri
685	DT/0131	Obule Medical Centre	115, Okpe Road, Sapele
686	DT/0132	Viva Clinic	6, Okere Ogborikoko Road, Okumagba layout
687	DT/0133	Winners Clinic	3, Tom Bra Close (off airport road, by DDPA, Effurun
688	DT/0149	B.U.Clinics & Hospital LTD	26 Kotie Street, Warri
689	DT/0152	Assumption Clinic & Maternity	8 Idiaregbe Street, Okumagba Layout, PO Box 137, Warri
690	DT/0154	St Patricks Specialist Clinic	131 Effurun / Warri Road
691	DT/0155	Assumption Clinic & Maternity	8 Idiaregbe Street, Okumagba Layout, Warri
692	DT/0157	Solar Clinic	1 Erhie Street Off Giwa Avenue Amu Road
693	DT/0163	Shekinah Medical Centre Limited	Opp. Heros Of Faith Church, Aro Rd Off Ughelli-Patani RD, Ughelli, Delta state
694	DT/0164	Marilo Clinic and Maternity	1 Celestial Church Road, off Ezenei Avenue, Asaba
695	DT/0165	Island Clinic & Maternity	100,Okere Ugborkoko Road, Okumagba Layout ,Warri, Delta State
696	DT/0166	Okparavero Memorial Hospital	No 159,Sapele rd, Okirighwre, Sapele, Delta State
697	DT/0167	First-Rank Hospital LTD	27,Ejewor Street, Behind Gateway To Heaven Ministry 24/26 Arubaye St.Okumagba Layout, Warri, Delta State

698	DT/0168	Shepherd Hospital And Fertility Center	100B Edjeba RD, Warri, Delta State
699	DT/0169	Rapha Specialist Children and General Hospital	
700	DT/0170	St. Andrew`s Hospital	39,Robert Road, Warri Delta State
701	DT/0171	Mudiaga Faith Medical Center	5,Ogbe Avenue Opp.
702	DT/0175	Abieyuwa Spec.Clinic	16 Edeyan Str.Masoje Junction Off PTI Road, Effurun Delta State
703	DT/0176	Onoruvie Memorial Clinic	17A Onoruvie Street Ozoro(Former Uvie Clinic)Delta State
704	DT/0178	Glorious Star Hospital LTD	Salubi Layout, Opp. Orodje Grammer School, Sapele, Delta State
705	DT/0180	Liberal Clinic And Diagnostic Centre	187,Sapele-Warri RD, Opp. Zenith Bank Sapele Delta State
706	DT/0181	Numa Clinic And Maternity	No 4, Numa Street Warri, Off Lower Ere-Jiwa,Warri Delta State
707	DT/0182	Purity Specialist Hospital	2 Eru Street By Agbaiho/DSC Roundabout Ebrumade,Effurun Delta State
708	DT/0183	Anioma Hospital	No 13,Memeh Street, Boji Boji,Owa,Agbor, Delta State
709	DT/0185	St. Rebecca Specialist Hospital	44 Denis Osadebay Way Asaba, Delta State
710	DT/0186	Federal University Of Petroleum Resources Health Centre	Ugbomro Effurun, Delta State
711	DT/0189	3-H Clinic & Maternity	7 Visah Street Off Urhiomwun Rd Ovwian, Delta State
712	DT/0190	James Clinic And Maternity Services	No 259,Sapele/Warri RD, Sapele, Delta State
713	DT/0191	Vicah Clinic & Hospital	6 Maduku Street Okumagba Layout Warri, Delta State
714	DT/0193	St.John The Baptist Catholic Hospital And Maternity	Alika Street , Boji-Boji Owa ,Delta State
715	DT/0195	Divine Grace Hospital Limited	Km 2 Refinery Road, By Mcaiver Hotel, Effurun Delta State
716	DT/0200	Healing Streams Clinic And Maternity	No 2, Plot 29,DDPA Estate, Igbudu,Warri Delta State
717	DT/0201	St. Luke Hospital Asaba	Opp Delta Broadcasting Office Asaba, Delta State
718	DT/0202	El-comfort Hospital	1,Fear God Street, BONSASC, Asaba, Delta Street
719	DT/0203	Lana Hospital	48, Atufe Road, Off Oke Road, Sapele Delta State.
720	DT/0205	Macap Clinic & Maternity	Egbokodo Road, By Amanoristsewor Junction Ubeji, Warri, Delta State
721	DT/0207	Shabach Hospitals Ltd	200 PTI Road, By Sunny Eru Motors, Effurun, Delta State

722	DT/0208	Twin City Hospital & Diagnostic Centre	99, Osadebey Way, Asaba Delta State
723	DT/0209	Group Christian Medical Hospital	3, Shirlet Onyemene Street, Okpanam, Asaba Delta State
724	DT/0210	Kenet Group Medical Services	14, Nwaezeapu Street, Off Nnebisi Road, Asaba Delta State
725	DT/0212	Komfort Consult & Surgery	5, Debe Crescent, Okpanam, GRA, Asaba, Delta State.
726	DT/0213	Queen Of Apostles Catholic Hospital	Obiaruku, Delta State
727	DT/0214	Catholic Hospital Abbi	Ogbezi Road, Abbi. Delta State
728	EB/0001	Mercy Hospital	26, Obi Street Abakaliki
729	EB/0002	St. Joseph's Hospital And Maternity	Arikpe Street Abakaliki
730	EB/0003	St. Theresa's Hospital & Mate	18 Jameni Street Abakaliki
731	EB/0004	Christ The King Hospital	85, Afikpo Road, Abakaliki
732	EB/0007	Federal Teaching Hospital, Abakaliki	Opposite Police Barracks
733	EB/0008	Holy Trinity Hosp. And Maternity	Abakaliki
734	EB/0027	Afikpo Medical Centre	Eke Market Road
735	EB/0030	Presbylterian Joint Hospital	Uburu Ohaukwu Local Govt. Area
736	EB/0032	DSS Clinic	Abakaliki, Ebonyi State
737	EB/0034	Child Health Consultant Clinic	6, Afikpo Street, Abakaliki, Ebonyi State
738	EB/0037	Felix Memorial Hospital	Abakaliki, Ebonyi State
739	EB/0039	Cant MRS Abakaliki	Abakaliki, Ebonyi State
740	EB/0040	Felysussy Memorial Hospital	12 Onitsha Str Abakaliki
741	EB/0042	Police Clinics, Ebonyi	Ebonyi State
742	EB/0043	Medical Centre Akanu Ibiam Federal Polytechnic, Unwana	Ibeyi, Ebonyi State
743	EB/0051	Kanmelu Hospital	5 Ekwulimi Street, Abakaliki
744	EB/0052	Ericon Hospital	45B Enugu Road, Abakaliki
745	EB/0053	Grace Hospital	4 Gunning Road, Abakaliki
746	EB/0056	Mater Misericodiae Hospital	Afikpo North LGA
747	EB/0058	Federal University Ndufu-Alike Ikwo Medical Centre	Federal University Ndufu-Alike Ikwo, Ebonyi State
748	EB/0060	El- Shaddai Specialist Hospital	44 Ogoja Rd. Abakaliki, Ebonyi State.
749	ED/0001	General Hospital, Afuze	Afuze
750	ED/0002	General Hospital, Igarra	Igarra, Akoko-Edo
751	ED/0003	Government Hospital	Ibillo
752	ED/0004	District Hospital, Akoko-Edo	Uneme-Osu
753	ED/0009	Psychiatric Hospital	Uselu
754	ED/0010	Ise Clinic & Maternity	37a Textile Mill Road, Benin
755	ED/0011	Mount Gilead Hospital	Off Uselu, Lagos Road, Benin City
756	ED/0015	District Hospital, Irrua	Usugbenu-Irrua
757	ED/0016	Irrua Specialist Hospital	Irrua
758	ED/0021	Central Hospital, Uromi	Uromi
759	ED/0024	General Hospital, Ubiaja	Ubiaja
760	ED/0025	District Hospital, Ewohinmi	Ewohimi
761	ED/0026	General Hospital, Iruekpen	Iruekpen, Along Benin Auchi Road
762	ED/0027	General Hospital, Ekpoma	Ekpoma

763	ED/0032	General Hospital, Anegbette	Anegbette
764	ED/0033	General Hospital, Agenebode	Agenebode
765	ED/0035	General Hospital, Agbede	Agbede
766	ED/0036	Central Hospital, Auchi	Auchi
767	ED/0037	Fate Medical Centre	99 Jatt/Auchi Junction
768	ED/0041	General Hospital, Igueben	Igueben
769	ED/0043	Narrow Way Clinic	No.9, S. Ahahor Street, Off Benin-Auchi Expressway, Aduwawa
770	ED/0045	Cina Well Point Hospital	16b Oviasogie Street, Theresa Cath. Church Road, New Benin
771	ED/0046	Oreste Medical Centre	58 Akenzua Way, Benin City
772	ED/0047	Owen Clinic	Upper Mission Road, Benin
773	ED/0049	Ekpen Hospital	Off Agho Street, Off Ekwenwan Road
774	ED/0050	Central Hospital, Benin	Along Sapele Road, Near Ring Road, Benin
775	ED/0055	General Hospital, Orhionmwom	Abudu Town, Orhionmwom
776	ED/0057	Government Hospital, Urhonigbe	Urhonigbe Along Agbor Abraka Road.
777	ED/0061	Igbinedion Hospital & Med. Research	Okada Wonderland
778	ED/0062	District Hospital, Ekiadolor	Ekiadolor
779	ED/0065	General Hospital, Iguobazuwa	Iguobazuwa Town, Hq Of Ovia South-West, LGA
780	ED/0066	Government Hospital, Usen	Usen
781	ED/0072	Government Hospital, Uzeba	Uzeba
782	ED/0076	District Hospital, Uhunmwode-Egba	Egba
783	ED/0077	Cottage Hospital, Obayanto	Obayantor, Along Sapele Road, New Benin Owena River Basin De
784	ED/0079	District Hospital, Apana	Apana
785	ED/0080	General Hospital, Fugar	Fugar
786	ED/0082	St. Margaret's Hospital & Maternity	No.1, St. Margaret's Hospital Drive, Upper Sakpoba Road, Ben
787	ED/0084	Azuwa Hospital	2nd West East, Benin City
788	ED/0085	Modic Medical Centre	No. 6 Akenzue Street, Ogbe, Benin City
789	ED/0086	Aiwuyor Memorial Clinic	30a, Uwa Street, Off 2nd East Circular Road, Benin City
790	ED/0087	Urban Medical Centre	Warrake
791	ED/0088	Safe Haven Hospital	No 11 Erhumuse, Ehaikpen Street
792	ED/0089	Ihenyen Medical Centre	No. 22, Ogbefun Street, Off College Road, Benin City
793	ED/0090	Time Hospital	3,2, Agbonma Lane, Off Ekwenwa Road, Benin City
794	ED/0091	God's Victory Clinic & Maternity	15 Obayuwuna Street, Off Siluko Road, Benin City
795	ED/0092	Government Hospital, Igbanke	Igbanke Along Uromi Road
796	ED/0114	Iyare Clinic	10, Second Adokpolor Street, Off Med. Stores Ltd, Benin
797	ED/0117	Madonna Clinic	Ibillo

798	ED/0118	University Of Benin Teaching Hospital Ugbowo	Benin City
799	ED/0119	DSS Clinic	Benin, Edo State
800	ED/0120	Military Hospital, Benin	Benin City, Edo State
801	ED/0121	81 AMG Medical Centre	NAF Station, Benin
802	ED/0125	4 Bde MC Benin	Benin, Edo State
803	ED/0126	NASEME MIR Auchi	Auchi, Edo State
804	ED/0127	University of Benin Health Centre	Benin, Edo State
805	ED/0129	Cottage Hospital/Comprehensive Health Centre	Edo State Police Command, Edo State
806	ED/0131	St. Mary Magdalene Hospital	8 Isibor Street off 3rd East Corator Rd., Off Murtala Mohd Way, Benin City
807	ED/0132	Niger Clinic	1 Ogbemudia Street, Behind Maria Filling Station Benin City
808	ED/0133	Ayunko Clinic & Maternity	39 Fed. Govt. College Rd., Ibillo
809	ED/0134	Hope Clinic & Maternity	No. 1 Whytehouse Street, Jatau, Auchi, Edo State
810	ED/0135	Faith Mediplex	Igiwa-Amadi/Airport Road GRA, Benin City, Edo State
811	ED/0137	Shalom Clinic & Maternity	6, Irhinmwiangbon Street, along Coolers Street, Off 1st Power Line, Useh Qtrs, Benin City
812	ED/0138	Osula Royal Hospital	No.5 Idahosa Lane, Oredo LGA
813	ED/0139	The Rock Hospital	39 Imuetinyan Street, Off Ihama Rd, G.R.A Benin Edo State
814	ED/0142	Our Medical Centre	No. 2 Oregbeni Street, Ikpoba Hill, Ikoba, Benin City
815	ED/0143	Lahor Research Lab. & Medical Centre	121 Old Benin, Agbor Road, Benin City
816	ED/0144	St. Rapheal Hospital	12, Adesua Road, Oredo LGA, Benin City
817	ED/0145	Aniso Specialist Medical Center	No. 11 2nd Fed. Road, Off 4th Srt Road or Off Benin Technical Coal Rd, Uselu Ltd Benin City
818	ED/0146	Lio-la Cross Hospital	1 Upper Wisdom Street Off 2nd East Road Benin
819	ED/0164	National Institute of Oil Palm Research, Edo	Ovia North LGA, Edo State
820	ED/0165	Akemekhai Medical Centre	3, Oyerekhau Lane, Jattu, Esako West, Auchi, Edo State
821	ED/0167	Auchi Polytechnic Medical Centre	Auchi, Edo State
822	ED/0168	Hope Hospital & Maternity	42, Costain Road, Benin, Edo State
823	ED/0170	Radiance Clinic & Maternity	Plot 194, Country Home Hotel Road, off Sapele Road, Benin City
824	ED/0171	Ogbeide Gen. Practice Clinic	58, Uwa Str., off 2nd East Circular Road, Benin.
825	ED/0172	Morat Clinic	5, Akugbe Str., Upper Siluko Road, Benin

826	ED/0174	Christway Medical Centre	31, Uwa Str., Off Murtala Mohammed Way, Benin
827	ED/0175	Royal Crown Clinic and Maternity	7, Okhuowaruyi Street, Benin
828	ED/0176	Hope Medical Centre, Igbueben	7 Suleman Street off sapele road, Benin City
829	ED/0177	Berta Medical Centre	New Upper Mission RoadExtension, Aduwawa, Benin
830	ED/0178	Gift Medical Clinic	6a, Iyamu Str., off Erie Str., Benin
831	ED/0179	Echos Hospital	5, Benin Technical College Road, Benin
832	ED/0180	Dekins First Trinity Medical Centre	1, Bieni Avenue, off court road, Abudu, Benin
833	ED/0181	Chisi Cottage Clinic	PCU Quarters, by Asoro Hills, Ekenwa
834	ED/0182	Bamby Hospital	147, Siluko Road, Benin
835	ED/0183	St. Luke's Clinic & Maternity	29, Ihumudumu Road, Ekpoma, Edo State
836	ED/0184	Irowa Medical Centre	Jos-Bazuaye Est. off Sapele Road, Benin, Edo State
837	ED/0214	S & T MRS, Benin	Benin
838	ED/0215	195 BN MRS, Agenebode	Agenebode
839	ED/0217	St Philomina Catholic Hospital	23 Dawson Road, off Mission Road, Benin City
840	ED/0218	Stella Obasanjo Women & Children Hospital	Country Home, Hotel Road, Benin City
841	ED/0219	Christ Medical Centre	18B Ogbesasa Street, Off Sapele Road, Off Sokponba Road, PO Box 3096, Benin
842	ED/0222	Irele Ifijeh Hospital Limited	22 Lucky Igbinedion Way, Ikpoba Hill, Benin City
843	ED/0223	Ovbioise Hospital	2 Isibor Aigbontaen Street, Aduwawa, Benin City
844	ED/0225	Vinda Medical Clinic	10A First Uwa Lane, Opp. Adesuwa RD Junction, Off Sapele RD, Benin City Edo State
845	ED/0228	Aimiuwu Hospital	36 Eghosa Street Off Erhumwunse street, Benin Edo State
846	ED/0230	City Hospital Auchi	36 Eghosa Street Off Erhumwunse street, Benin Edo State
847	ED/0231	Flowell Pharma Nig.Ltd	69b Sapele RD.
848	ED/0232	Quality Medical Centre	25, Erinmwiogha Street, Opp. Benin Tech. Coll. Road, Benin City Edo State
849	ED/0233	Life Medical Centre	34 Guobadia Street, Off Ugbor Rd, G.R.A, Benin City Edo State.
850	ED/0235	Enofe Medical Centre	5 Ajayi Street Off Okpagha Rd,Eyaen,Edo State
851	ED/0237	Aigbogun Clinic And Maternity	52,Old Benin Asaba Road,

852	ED/0238	Bode Medical Center	New Auchi Road, Upland Agenebode Edo State
853	ED/0239	Bazuaye Hospital	34 Costain Rd, New Benin Edo State
854	ED/0241	Fountain Of Life Specialist Hospital	3B Ogbesasa Street, Off Sapele Road Edo State
855	ED/0242	Edi International Hospital	No. 1,Coronation Drive, Off Adeyan Avenue ,G.R.A Benin City Edo State
856	ED/0244	Abe Medical Clinic And Maternity	17 Aruosa St, Off Sokponba Rd, Benin City Edo State.
857	ED/0248	Total Health Medical Services Center	20, Ayo-Idiaghe Street,Isiohor,Benin Edo State
858	ED/0249	Standard Medical Clinic And Maternity	No. 14,Eyeye St,Off Uwasota, Ugbowo,Benin City, Edo State
859	ED/0251	General Hospital Sabogidda-Ora	General Hospital Sabogidda-Ora Owan West Edo State
860	ED/0252	Amanfoh Nursing And Maternity Home	20,Old Ukoni Road ,Uromi ,Edo State
861	ED/0253	Ebony Medical Centre	59 Uwasota Rd, Ugbowo Benin City Edo State
862	ED/0254	Aipoh Medical Centre	6,Obayagbona Street, by Spirit & Life Bible Church(Joromi Road)Off Ekenwa Road, Benin Edo State
863	ED/0255	Bethel Faith Medical Centre	Plot 11 and 12,Jos Bazuaye Layout, Erediawa Street, Off Ekenwan Road, Benin City,Edo State
864	ED/0258	Suyi Hospital	No. 110/112, MM Way, Benin City Edo State.
865	ED/0260	Isda Hospital	4,Aghavbere Street, Off Agbonkina Street Off Benin/Abraka Road, Idogbo Community, Benin Edo State
866	ED/0261	Police Hospital Benin G.R.A	Plot 12,Dennis Osadebey Avenue, Benin Edo State
867	ED/0262	Akugbe Medical Center	No. 9,Omokaro St. Off Upper Sokponba RD ,Benin City Edo State
868	ED/0263	Faith Dome Medical Centre	No. 3,Itama Street, Eguare Ekopma Edo State
869	ED/0264	Eromosele Medical Centre	Ujoelen Road Ekpoma
870	ED/0265	Enoma Medical Center	3,Hospital Road, Off Old Benin Agbor Road, Ogbeson, Benin Edo State
871	ED/0267	Tona Hospital	No. 2,Ighodalo Street, Off Benin-Agbor Road ,Ikpoba Hill, Benin City, Edo State
872	ED/0268	Ropheka Specialist Clinic & Maternity	22,Upper Ewekan Road,

873	ED/0269	Okougha Medical Center	Behind Aoma (UZEBBA) Market Uzebba Owan
874	ED/0270	Halpha Specialist Hospital	1,Akhiobanke Street, G.R.A, Benin Edo State
875	ED/0271	Unique Hospital And Maternity.	64,Uwarrake Road, Opp. Uwarrake Motor Park, Auchi Edo State
876	ED/0272	Gims Clinic LTD	12 Edebiri Street ,Ogbe Quarters, Benin City Edo State
877	ED/0273	Mega Clinic And Maternity	271 Upper Siluko RD ,Beside Noma Petrol, Ogida, Benin City Edo state
878	ED/0275	Eguavon Medical Centre	No 5,Abia,Iruekpen,Ekpoma,Edo State
879	ED/0276	Oriaifo Medical Center	No 1,Oriaifo Close, Uromi-Illushi Road, Uromi Edo State
880	ED/0278	Eseohe Medical Center	No. 2,Police Barrack RD ,Off Benin Auchi Express Way, Beside Mousco Petrol Station ,Ekpoma
881	ED/0279	Halpha Specialist Hospital	1, Akhiobanke Street, G.R.A, Benin
882	ED/0280	Jimon Medical Center	152, Upper Owina Street Evbotubu Qtrs, Benin Edo State.
883	EK/0001	St.Gregory Hospital	KM 2 Basiri Iyin Road, Ado Ekiti
884	EK/0002	Olumorin Specialist Hospital	Ado-Ekiti
885	EK/0003	Ayobola Hospital	22,Ekute Quarters Ado-Ekiti
886	EK/0004	Ade-Tade Hospital	11, Okebola Street, Ado Ekiti
887	EK/0005	Orire Hospital Complex	1, Orire Lane, Box 944, Ado Ekiti
888	EK/0006	University Teaching Hospital, Ado Ekiti	Ado Ekiti
889	EK/0007	Federal Teaching Hospital	Ido Ekiti
890	EK/0008	Alafia Hospital	Off State Housing Estate Rd., Off Adebayo Road, Ado Ekiti
891	EK/0009	Trinity Medical Centre	Km 3, Iwokoro Rd., Ado Ekiti
892	EK/0010	Supreme Faith Hospital	Ado
893	EK/0011	Joe-Jane Medical Centre	27 Dallimore Street, Ado Ekiti
894	EK/0012	Divine Citadel Hospital	Coca Cola Bus Stop, Ajibosun Street, Ado Ekiti
895	EK/0027	DSS Clinic	Ado-Ekiti, Ekiti State
896	EK/0028	Comprehensive Health Centre	Okesa, Ado Ekiti, Ekiti State
897	EK/0029	Adetoyin Hospital	No. L16 Onigari, Secretariat Rd., Ado Ekiti
898	EK/0031	Cottage Hospital, Ado-Ekiti	Ekiti State
899	EK/0032	Federal Polytechnic Health Centre	Ado Ekiti, Ekiti State
900	EK/0033	VICTORY HOSPITAL	2 AWEDELE STR, ADO-EKITI
901	EK/0034	STATE SPECIALIST HOSPITAL	IKOLE-EKITI
902	EK/0035	UNIVERSITY OF ADO EKITI HEALTH CENTRE	MAIN CAMPUS
903	EK/0036	PRAISE THE LORD MEDICAL CENTRE	7 ISATO STR, IRONA ADO EKITI
904	EK/0037	OGUNTOYE HOSPITAL	136 ILAWE RD, ADO EKITI
905	EK/0038	Endoscopy De Gilead Specialist Hospital	1, Gilead Lane, NTA Road, off Ilawe Road, Ado-Ekiti

906	EK/0045	Folasade Clinic, Usi Ekiti	Irede Estate, Usi Ekiti
907	EK/0047	Maria Assumptia Catholic Hospital	Ireje Street, Ikere Road, Ado Ekiti
908	EK/0048	Afolabi Hospital	Afolabi Hospital Close off Opopogbooro, Behind Mojere Mkt
909	EK/0052	Sanya Clinic	12 Bamgboye Street, Off Ikere Road, Ado Ekiti
910	EK/0057	Ademolawe Memorial Hospital	Ikole Ekiti, Ekiti State
911	EK/0058	Liberty Hospital	Block 14 Plot5 Ekiti State Housing Corporation, Ado Ekiti, Ekiti State.
912	EK/0059	Queens Care Specialist Hospital	1 Unity Avenue, UM Iyin Road, Ado Ekiti, Ekiti State.
913	EK/0060	Itunuoluwa Medical Centre	Gbahun Alore Street Ekute Quarters, Ado Street Ekute Quarters, Ado Ekiti, Ekiti State.
914	EK/0061	Potter Family Care Clinic	114 Okela Housing Road Ado Ekiti, Ekiti State
915	EK/0062	Olives Specialist Clinics & Fertility Centre.	Plot 5 Tiniola Avenue Ekiti Housing Estate Ado Ekiti, Ekiti State.
916	EK/0063	Eden Life Hospital	Opp. CAC Comprehensive High Umem Avenue Ilawe Road, Ado Ekiti, Ekiti State.
917	EK/0064	Maternal Child Specialist	Adebayo Road Ado Ekiti, Ekiti State.
918	EK/0065	College Of Education Health Center	Ikere Ekiti, Ekiti State
919	EK/0066	Fola-Mayowa Clinics & Maternity Home	41, Ekute Street, Ado Ekiti, Ekiti State.
920	EK/0067	Unique Consultant Hospital	Adebayo, Ado Ekiti, Ekiti State
921	EK/0068	Shekinah Group Medical Centre	Federal Polytechnic Road Ado- Ekiti, Ekiti State
922	EK/0070	Federal University Health Centre, Oye-Ekiti	Oye-Ekiti
923	EK/0072	Kristal Medical Centre	No 1, Florence Adedoja Street, Oke Ila Housing Estate, GRA, Ado Ekiti, Ekiti State
924	EK/0074	Heirs Specialist Hospital, Oye Equity	Madam Aluko's House, Irare Estate, Oye Ekiti, Ekiti State.
925	EK/0075	Adebayo Memorial Hospital	Adebayo Street, off Amvion Road, Otun Ekiti, Ekiti State.
926	EN/0002	St. Patricks Hospital & Maternity	8, Owerri Road, Asata, Enugu
927	EN/0006	Christ The King Hospital	No. 18 Emeka Abalu Ave. Phase 6, Trans Ekulu, Enugu
928	EN/0007	Peenok Medical Centre	24, Zik Avenue, Uwani, Enugu
929	EN/0008	Dr. Nlogha Okeke Memorial Foundation, Enugu.	30, Amigbo Lane Uwani, Enugu
930	EN/0009	Jeno Hospital Ltd.	23 Nnamdi Azikiwe Avenue, Trans-Ekulu, Enugu

931	EN/0011	Michael Obayi Memorial Hospital	49/51, Emmanuel Obayi Avenue, Anyanaeleigbo Achara, Nsukka
932	EN/0012	Cimac Hospital Ltd.	91, Agbani Road, Enugu.
933	EN/0013	Ristela Hospital & Maternity	3, Ristela Close, Independence Layout, Off Residential Road, E
934	EN/0015	Our Saviour Medical Centre	9th Mile
935	EN/0016	Ntasiobi Hospital,	Enugu
936	EN/0017	Akulue Memorial Hospital	16/18 Akulue Road, Onuiyi, Nsukka, Enugu
937	EN/0018	Unity Ngwo Hospital & Mat.	Ngwo, Enugu, Enugu State
938	EN/0019	Benem Hospital	Ngwo, Enugu, Enugu State
939	EN/0024	Life Foundation Hospital	3, Agu Street, Achara Layout, Enugu
940	EN/0025	Ibezim Medical Clinics	56, Ufuma Street, Achara Layout, Enugu
941	EN/0026	Kenechukwu Specialist Hospital & Maternity	94, Nike Road, Abakpa, Enugu State
942	EN/0027	Julius Ezenyirioha Memorial Hospital & Maternity	32, Nike Road, Abakpa Nike, Enugu.
943	EN/0028	Chinwendu Hospital & Maternity	29, Imo River St. Housing Estate, Abakpa, Enugu
944	EN/0029	St. Leo's Specialist Hospital	Nike Lake Road, Abakpa, Enugu
945	EN/0030	Orient Hospital	Agbani Town, Agbani, Enugu
946	EN/0031	Mother Of Mercy Hospital & Mat.	Ihe, Awgu Lga, Enugu State
947	EN/0032	St. Clara's Maternity Hospital	Umonandiogu, Ezeogu Lga, Enugu
948	EN/0034	The Merken Hospital Centre	Amechi Road, Awkunanaw, Enugu
949	EN/0037	Royal Hospital	3 Aria Road, Gra, Enugu
950	EN/0042	Annuciation Hospital	Emene, Enugu.
951	EN/0044	ESUT Teaching Hospital, Parklane	G R A, Enugu
952	EN/0046	Orakwue Hospital	16 Affa Street, Uwani, Enugu
953	EN/0102	University Of Nigeria Teaching Hosp., Enugu	Enugu
954	EN/0103	82 Div Hospital	Enugu, Enugu State
955	EN/0104	337 BSG NAF Medical Centre	NAF Base, Enugu
956	EN/0105	Ubabuike Hospital	Enugu
957	EN/0107	Medical Post (AFCSC)	Enugu
958	EN/0109	103 Bn MRS Enugu	Enugu, Enugu State
959	EN/0110	Cottage Hospital/Comprehensive Health Centre, Oji River	Police College,Oji River, Enugu
960	EN/0111	Cottage Hospital/Comprehensive Health Centre, Police Detecti	Police Detective College, Enugu
961	EN/0112	Cottage Hospital/Comprehensive Health Centre	Enugu State Police Command, Enugu.
962	EN/0113	National Orthopaedic Hospital	Enugu, Enugu State
963	EN/0115	Mayo Hospital	19, College Road, Ogui New Layout, Enugu
964	EN/0116	UNN Medical Centre, Enugu	UNEC, Enugu Campus
965	EN/0117	Nigeria Law School Medical Centre	Agani Town, Enugu
966	EN/0118	All Saints Medical Centre	7 Edemani Road, Nsukka

967	EN/0119	University of Nigeria, Nsukka Medical Centre	Nsukka, Enugu State
968	EN/0164	Mother of Christ Specialist Hospital	Ogui, Enugu State
969	EN/0169	DSS Clinic	Enugu, Enugu State
970	EN/0170	Police Clinics, Enugu	Enugu, Enugu State
971	EN/0171	Federal College of Education, Eha-Amufu	Eha-Amufu, Enugu State
972	EN/0175	THE GOOD SHEPHERD SPECIALIST HOSPITAL	CHRIST CHURCH STREET, UWANI
973	EN/0177	ALPHA SPECIALIST CLINIC	55 NIKE ROAD,ABAKPA, ENUGU
974	EN/0179	CHINEMELU HOSPITAL	88,OGURGURU ROAD,ODENIGBO,NSUKKA
975	EN/0180	NSUKKA MEDICAL CLINIC	7, NRU STREET,UGWU-NKWO, NSUKKA
976	EN/0190	Nigerian Railway Clinic. Enugu	Railway Compound, Western Avenue, Enugu
977	EN/0195	Renaissance Hospital	Isuja, Nsukka
978	EN/0197	St Bridget's Specialist Hospi	12 John Okoro Street Emene
979	EN/0198	Semino Clinic	8 Onuayo Street Enugu
980	EN/0200	Daberechi Hospital & Maternity	22 College Road, Abakpa, Enugu
981	EN/0201	Chigozie Hospital & Maternity	11 Ekwulu Lane, Emene
982	EN/0202	Elohim Hospital and Maternity	12 Eke Obinagu, Enugu
983	EN/0203	NAF Sick Bay	Agbani, Enugu
984	EN/0205	Royal Cross Hospital & Maternity	14 TTC Road Nsukka
985	EN/0206	The Good Shepherd Hospital	8/10 Iheagu Road NRU Village, Nsukka
986	EN/0213	St Raphael's Hospital & Mater	Ibagwa-Aka, Nsukka
987	EN/0216	St Mary's Hospital and Maternity	16 Nwodo Close, GRA, Enugu
988	EN/0217	Bishop Shanahan Hospital	46 Enugu Road, Nsukka
989	EN/0219	Nwa-Ossai Foundation Hospital	20, Enugu road, Orba, Nsukka
990	EN/0221	Divine Mercy Hospital& Maternity	32 Orba Road, Nsukka, Enugu State
991	EN/0225	Kings Court Hospital &Maternity	28,Edenwu street New Haven, Enugu State
992	EN/0226	Hosanna Hospital &Maternity	Nsude,Udi Local Government Area, Enugu state
993	EN/0230	Ekwomibe Memorial Hospital	Ngwo, Enugu State
994	EN/0231	Faith Foundation Hospital	27B,Ogurugo Road Nsukka,Enugu State
995	EN/0235	Mount Arafat Hospital	59,Aku Road Nsukka,Enugu State
996	EN/0236	Izundu Specialist Medical Centre	Izundu Medical Avenue New Site,Oji River Enugu, Enugu State
997	EN/0237	Summit Hospital &Maternity	Nsukka Road Obollo Afor,Enugu State
998	EN/0238	Tendercare Hospital & Maternity	NO.5B Ezeji Close Ugboezechi,Abakpa,Enugu State
999	EN/0242	Jideofor Hospital	Ikpenwa Flat N.A. Quarters Agwu,Enugu State
1000	EN/0244	Enugu State Uni Of Science &Tech (ESUT) Medical Centre	Agbani, Enugu State
1001	EN/0246	St. Gabriel Hospital(Specialist)	2, fourth Avenue Trans-Ekulu Enugu state

1002	EN/0247	Mapol 3 Clinic Enugu	Agbani Road Awkwnawnaw Enugu State
1003	EN/0248	Calix Healthcare Ltd	10A Hill View Road, ESBS,
1004	EN/0251	Freedom Fertility Centre	No 6 Hill View Avenue, Independent Layout, Enugu, Enugu State
1005	EN/0252	Potters Master’s Touch Hospital	4 Diara Drive, Golf View Layout, Enugu-Onitsha Express Way, Phase V1, Trans Ekulu. Enugu State
1006	EN/0253	Keme Hospital	21 Egbo Nnaji Street, Achara Layout, Enugu, Enugu State
1007	EN/0254	Christian Miracle Centre	3 Akutu Crescent, , Independent Lay Out, Enugu State
1008	EN/0255	Holy Family Hospital	Old Obollo Afor Road, Ohom, Orba Enugu State
1009	EN/0256	Choice Medical Centre	1C Monrovia Street, New Heaven, Enugu State
1010	EN/0257	Marcus Nnadi Memorial Hospital	16 University Road, Obollor Afor Enugu State
1011	EN/0258	Chukwuemeka Hospital & Maternity	Ulanya, Ovoko, Obollo-Nsukka Road, Nsukka Enugu State
1012	EN/0259	Bridge View Clinic Ltd	2 Nwodo Street ,Off Ekulu Avenue GRA, Enugu State
1013	EN/0260	St. Anthony’s Hospital	5 Urban Girls Road, Obechara, Nsukka Enugu State
1014	FCT/0001	Wildot Clinic	Plot 903 Obafemi Awolowo Way, Utako District
1015	FCT/0002	Arewa Specialist Hospital & Diagnostic Centre	Plot 645, Alex Ekwueme Street, Jabi District, Abuja
1016	FCT/0004	Gwarinpa General Hospital	Gwarinpa,Abuja
1017	FCT/0005	Sybron Medical Centre	25, Mungo Park close, opp Havista Hotel, Asokoro, Abuja
1018	FCT/0008	Pan-Raf Hospital Ltd.	Nyanya Area C, Abuja
1019	FCT/0010	Bismol Clinic & Maternity	Plot 8, Area F, Nyanya, Abuja
1020	FCT/0011	St. Patricks, Clinic & Mat.	Area F, Beside Police Station, Nyanya Abuja
1021	FCT/0012	Jalingo Medical Clinic	Plot 53, Phase Ii, Opp. Area A, Nyanya Abuja
1022	FCT/0013	Alfad Specialist Medical Centre	Kuje, FCT, Abuja.
1023	FCT/0014	Primary Health Clinic, Karonmagaji	Karonmajigi, Abuja
1024	FCT/0015	Midland Clinic	Karu-Site By Bus Stop, Karu, Abuja
1025	FCT/0016	Divine Mercy Hospital & Diagnostic Centre Ltd	Plot 93, Phase 2/1 Gado Nasko Street, Kubwa, Abuja
1026	FCT/0017	The Comforter Clinic & Diag. Centre	Plot 1233, 1 (R) 4 Road by AMAC International Market behind CBN Quarters Lugbe FHA
1027	FCT/0036	St. Georges Hospital & Maternity	No. 18 3rd Avenue Gwarimpa Ii \,Abuja

1028	FCT/0037	Unity Clinic	Plot D1 Phase Ii Site One Gado Nasko Road, Kubwa
1029	FCT/0038	Sumit Hospital	Bwari
1030	FCT/0039	Express Hospital	Nysc Road, Kubwa, Abuja
1031	FCT/0040	Kings Care Hospital Ltd - Kubwa	7, Christmas Road, Behind Police Affairs, Kubwa Abuja
1032	FCT/0043	Kubwa Clinic & Maternity	Plot159b Kubwa, Behind Channel 8 Restaurant, Kubwa, Abuja
1033	FCT/0044	Pison Hospital	3553 Gado Nasko Road by MTN office, Phase lV, Kubwa
1034	FCT/0045	Miracle Seed Clinic & Maternity	40b Dasuki Street, Phase Ll, Site 2, Kubwa, Abuja
1035	FCT/0046	Grace Of God Specialist Med. Centre	Phrase 2, Site 2, Kubwa, Abuja
1036	FCT/0047	Kubwa General Hospital	Phase 4 Opp. Gss, Kubwa, Abuja
1037	FCT/0049	St. Vincent Clinic & Maternity	Daughtrer''s Of Charity, Biyazhin Road, Kubwa
1038	FCT/0050	Royal Lords Hospital, Clinics & Maternity Ltd	Plot 107, Zone 4, Dutse Alhaji, Abuja
1039	FCT/0073	Primary Health Centre, Gwagwalada	Gwagwalada
1040	FCT/0086	Rhema Foundation Hospital	Rhema Fountain, Kwali, Abuja
1041	FCT/0087	Standard Care Hospital & Maternity	T-Junction Opp. Min. Of Finance, Phase 1, FHA, Lugbe, Airport Road, Abuja
1042	FCT/0091	Maitama District Hospital	Maitama Abuja
1043	FCT/0092	Asokoro District Hospital	Asokoro, Abuja
1044	FCT/0093	Wuse District Hospital	Wuse, Abuja
1045	FCT/0095	Asher Hospital & Maternity	Plot 1197, Kataamkpe Cadastral Zone B07, Opp NICON Junction, Berger-Kubwa Express way
1046	FCT/0097	Sauki Private Hospital	Plot 90 Zone 6 Yaounde Crescent Wuse
1047	FCT/0098	Leblon Clinic	Opposite Gloryland Nursery & Primary Sch., Behind Gwarinpa Sec. School, Life Camp, Abuja
1048	FCT/0099	General Hospital, Nyanya	Nyanya, Abuja
1049	FCT/0101	Al-Bama Clinics & Maternity	No. 35 Royal Palm Street, Fed. Housing Estate, Nyanya
1050	FCT/0102	Our Lady Of Fatima Catholic Hospital	Ushafa Rd., Bwari, Abuja
1051	FCT/0103	Kwali General Hospital	Off Abaji/Lokoja Road, Near Area Council Secretariat, Kwali,
1052	FCT/0104	Abaji General Hospital	Abaji, Abuja
1053	FCT/0105	General Hospital - Karshi	Karshi, Abuja
1054	FCT/0106	Alliance Hospital and Services Ltd	Plot 801, No. 5 Malumfashi Close off Emeka Anyaoku Street, Garki Area 11
1055	FCT/0107	Ruz Medical Centre	Ubiaja Crescent, Garki, Abuja
1056	FCT/0108	Rouz Hospital & Maternity Limited	Apo, Legislative Qtrs, Phase II
1057	FCT/0109	Pyramid Medical Centre	Plot 1438 Garki Ii Opp. Nia Cadastral Zone A3 Nnamdi Azikwe

1058	FCT/0110	Amana Medical Centre	No. 5, Ilorin Street, off Ogbomosho Street, Area 8, Abuja
1059	FCT/0111	Fereprod. Medical Centre	Off Ahmadu Bello Way Behind Nepa Sub-Station-2 Cbn Area Ii,
1060	FCT/0112	Primary Health Centre, Karu	Karu, Abuja
1061	FCT/0113	Alpha-Zed Medical Clinic Limited	Angwar-Gwari, Karu, FCT
1062	FCT/0114	Kuje Primary Health Centre	Kuje Near Local Govt. Office Kuje, Abuja
1063	FCT/0115	Jilf Clinic Ent.	Plot D 231 Crescent By 232 Road Kado Estate
1064	FCT/0116	Ebony Hospital	House No. 76, 3rd Avenue, Gwarinpa Housing Estate, Abuja
1065	FCT/0120	Limi Hospital & Mat., Ltd.	Plot 541, Behind Icpc, Central Area, Abuja
1066	FCT/0122	National Assembly Clinic	Three Arm Zone, Central Area, Abuja
1067	FCT/0123	State House Clinic	(Presidency) Three Arm Zone, Central Area, Abuja
1068	FCT/0124	Woman Development Centre, Staff Clinic	Woman Development Centre, Central Area, Abuja
1069	FCT/0127	Sami-Wadata Clinics Ltd.	Plot 766 Bukcanan Crescent, Off Aminu Kano Crescent, Wuse II
1070	FCT/0128	Iduna Specialist Hospital	Plot 1200 Ndjamena Crescent, Wuse Ii
1071	FCT/0131	Mercy Specialist Hospital & Diag. Centre	Off Aminu Kano Crescent, Plot 619c Zone A7, Wuse II, Abuja
1072	FCT/0132	Kings Care Hospital - Wuse	Wuse Zone 4, Abuja
1073	FCT/0133	Capital Hospital	15, Oran Street, by Bank PHB, Off Olusegun Obasanjo way, Abuja
1074	FCT/0136	CRI Medi Clinics	Opp. Peniel Apartment, Off Adetokunbo Ademola Crescent, Wuse
1075	FCT/0138	Queens Clinics	Plot 225, Cotonou Crescent, Wuse Zone 6, Abuja
1076	FCT/0139	Bio-Royal Hospital & Maternity Ltd.	No. 190, Okene/Jebba Close, Garki
1077	FCT/0140	INEC Staff Clinic	Area 10, Inec Office, Garki
1078	FCT/0141	Holy Trinity Hospital Ltd.	27, Oro Ago Crescent, off Muhammadu Buhari Way, Garki II, Abuja
1079	FCT/0143	Diamond Medical Centre Ltd.	Plot 42, Beside Mr. Bigs By Model Pri. Sch. Gark Ii
1080	FCT/0145	Savannah Hospitals Ltd.	Behind City Rock Hotel, Mararaba, Off Nyanya abuja, Km 42, Keffi Road, Mararaba
1081	FCT/0146	Crescent Clinic	Off Oyo Street, Area 2 Section I, Garki
1082	FCT/0147	Dara Medical Clinics	Plot 202, Bacita Close, Off Plateau Street, Area 2 , Garki

1083	FCT/0149	Ministry Of Defence Staff Clinic	Ship House, Olusegun Obasanjo Way, Area 10, Garki
1084	FCT/0150	Kinectic Hospital Limited	Ahoada Close, off Emeka Anyaoku street, Area Ii, Garki
1085	FCT/0151	Alibu Medical Centre	Area F, Nyanya Abuja
1086	FCT/0152	Lafiak Hospital	Toto Road, Abaji
1087	FCT/0153	Gwagwalada Clinic & Maternity	Kutunku Along Frcn Road, Abuja
1088	FCT/0154	Jerab Hospitals	Plot 145, Kutunku Near Radio House, Gwagwalada, Abuja
1089	FCT/0155	Sliver Fountain Medical Centre	Kukwala District, Opp. Kuchingoro, Along Airport Road, Abuja
1090	FCT/0156	Rapha Hospital And Maternity	Phase Iii, Near Giwa Hotel, Jikwoyi, Abuja
1091	FCT/0157	Arewa Clinic & Maternity - Karu	Plot F14, By Nepa Office, Karu
1092	FCT/0158	Nisa Premier Hospital	Plot 618, Alex Ekweme Way, Jabi Abuja
1093	FCT/0159	Abuja Clinic - Maitama	Maitama, Abuja
1094	FCT/0330	National Hospital Abuja	Central Area
1095	FCT/0333	Albert Horsfall Med. Centre	NIA Hq, off Muritala Mohammed way, near Danata & Sawoe, Garki
1096	FCT/0334	Defence Intelligence Agency	Asokoro
1097	FCT/0335	Revenue Mobilization & Fiscal	210 Tafawa Balewa Way, Central Area, Garki
1098	FCT/0336	Living Stream Specialist Hospi	43/45, Street A, Phase I, off University Rd, Gwagwalada
1099	FCT/0337	Angelic Care Hospital & Mat.	19, Ngwa close off Funmilayo Ransome Kuti Road, Garki Area 3, Abuja
1100	FCT/0340	DSS Medical Centre.	VGC, Opposite National Military Cemetary, Airport Road, Abuja
1101	FCT/0343	Nigeria Customs Service Clinics - Wuse	Wuse Zone 3, Abuja
1102	FCT/0344	Nigeria Customs Service Med. Centre - Karu	Karu Customs Barracks, Abuja
1103	FCT/0345	Guards Brigade Med. Centre	Yakubu Gowon Barracks, Abuja
1104	FCT/0346	108 Nigerian Air Force Hospital Abuja	108 Nigerian Air Force Hospital Abuja
1105	FCT/0347	Cornerstone Specialist Hospital	Plot 1397, Abidjan Street, Zone 3, Wuse, Abuja
1106	FCT/0350	City Hospital	Wuse Zone 5, Abuja
1107	FCT/0357	Bwari General Hospital	Kawu Road, Bwari, FCT, ABuja
1108	FCT/0359	Abuja Cliniic, Garki	Area 3, Garki, Abuja
1109	FCT/0360	Abuja Cliniic - Karu	Karu, Abuja
1110	FCT/0362	G & S Specialist Clinic	8B Rhine Street (Opp. IBB Way), Maitama
1111	FCT/0366	Saffron Hospital Ltd.	1 KM, Left Off Checkpoint, Abuja-Keffi Expressway, Phase IV, Nyanya, FCT
1112	FCT/0370	Medical Centre (Council of Legal Education) Nigerian Law Sch	Nigerian Law School, Bwari, P.M.B. 170, Garki, Abuja

1113	FCT/0371	4 Bn MRS Abuja	Abuja
1114	FCT/0372	NWC MIR Abuja	Abuja
1115	FCT/0374	Force Headquarters Hospital	Force Headquarters, Abuja
1116	FCT/0376	Sick bay NHQ	NN HQ, Abuja
1117	FCT/0377	DHQ MRS	Mogadishu Cantonment, Abuja
1118	FCT/0378	Horrizons Medical Centre	Plot 777 Bouake Street Wuse Zone 6, Abuja
1119	FCT/0379	Bepos Clinic & Maternity	10, Faskari Street Area 3 Garki Abuja
1120	FCT/0380	ICPC Staff Clinic	Plot 802 Constitution Ave Central Area Abuja
1121	FCT/0381	National Commission for Colleges of Education (NCCE) Staff C	NCCE Management Staff Quarters Mabushi Wuse II Abuja
1122	FCT/0382	Kefat Medical Centre	No 5 Damaturu Close, Behind Mr.Biggs, FHA, Gwarinpa
1123	FCT/0385	Well Point Clinic	House 1, Road 45, Off 1st Ave, Gwarinpa
1124	FCT/0386	The Crown Hospital Ltd.	Behind Police quarters, Gwagwa, Abuja
1125	FCT/0387	Ronella Specialist Hospital	51 Crescent Road, Flat 3, Phase IV, Kubwa, Abuja
1126	FCT/0389	Rainbow Clinic & Maternity	Plot 218, Gudu District Apo Qtrs Garki, Abuja
1127	FCT/0390	Federal Staff Hospital - Jabi	Airport Road, Jabi
1128	FCT/0391	Everlasting Care Hospital Limited	Plot 643 Gimbia Street, Area II, Garki, Abuja
1129	FCT/0392	Micheal Cross Specialist Hospital	Plot FG Church Road, Karu Site, Karu, Abuja
1130	FCT/0393	Convenant Clinic & Maternity	Anygba Street Opp NTA Area 11 Garki
1131	FCT/0394	St Mary Catholic Hospital, Gwagwalada	No 1 FRCN Road, Opposite Police Station Gwagwalada
1132	FCT/0395	Precious Clinic & Maternity	House 57, 1st Avenue FHA, Lugbe, Abuja
1133	FCT/0396	Kutunku Alheri Clinic & Maternity Ltd	Along New Millenium Hotel, P.O Box, 7, New Kutun-Gwagwalada
1134	FCT/0397	Abuja Unity Hospital & Maternity, Lugbe	FHA Phase I Lugbe
1135	FCT/0398	Agnes Maternity Home	Opp Old Block Industries Near Over Head Water Tank Lugbe
1136	FCT/0399	Sophy Hospital & Maternity	Jukwoyi-Karshi Rd, Jukwoyi, Phase II
1137	FCT/0400	St Luke''s Clinic & Lab	Plot 838 FHA 2, Karu
1138	FCT/0425	Kuje General Hospital	Kuje, Abuja.
1139	FCT/0454	Kela Clinics & Maternity Ltd.	Beside Lutheran Church, Opp. Phase II Primary Sch., Along Keffi Road, Nyanya, Abuja
1140	FCT/0472	Dr. Hassans Clinic/Diagnostic Centre	9 Njamena Street, off Aminu Kano Crescent, Wuse II, Abuja
1141	FCT/0476	St. Peters Catholic Hospital	Abuja-Lokoja Road, Abaji, Abuja

1142	FCT/0477	City International Clinics	Kabayi, Mararaba, Nasarawa State
1143	FCT/0478	University of Abuja Teaching Hospital, Gwagwalada	Abuja
1144	FCT/0479	Conita Clinic & Maternity, Dagiri	Beside Dagiri Primary School, Gwagwalada, Abuja
1145	FCT/0481	N - Foundation Hospital	No. 2 Amesi Street, Behind Redeemed Church of God, Kuje, Abuja
1146	FCT/0482	Maraba Clinic & Maternity	Behind Bank of the North, Gwagwalada, Abuja
1147	FCT/0483	Mumeen Medical Centre	Wodi Shopping Village, Behind Old Motor Park (NARTO Motor Park) Gwagwalada, Abuja
1148	FCT/0484	DSS Clinic, Bwari	(ISS), Usuma Dam - Bwari, FCT, Abuja
1149	FCT/0485	National War College, Ushafa Camp Clinic	Ushafa, NWC Camp, Bwari LGA, Abuja
1150	FCT/0486	Federal Road Safety Commission Medical Centre	Plot 18 Cotonou Crescent, Wuse Zone 6, Abuja
1151	FCT/0488	National Centre for Women Development Staff Clinic	Central Area, Garki
1152	FCT/0489	Roma Clinic & Maternity	12B Subeiru Crescent, Gbazango, Kubwa, Abuja
1153	FCT/0490	J. Ugonma Hospital Ltd.	153 Extension, Old Maitama, Kubwa
1154	FCT/0491	Freedom Hospital & Health Consultants	405 Phase III Road, Kubwa
1155	FCT/0492	Alpha Clinic	332 Alpha Clinic Zone 1, Dutse Alhaji, Abuja
1156	FCT/0493	Gideon Legacy Hospital Limited	Along Nitel Road, Zone 5, Dutse Alhaji, Abuja
1157	FCT/0494	Divine Winner Medical Center	First Gate Along Police Station Road, Dutse Alhaji, Abuja
1158	FCT/0495	Allied Surgery	Off Specalist Hospital Road, Behind Charismatic Revival Church, Gwagwalada
1159	FCT/0498	NIPRID Staff Clinic	NIPRID, Idu, Abuja
1160	FCT/0499	Faith Medical Centre	Old Karimo Along Dantata Road, Abuja
1161	FCT/0500	JEDONEC Clinic & Maternity	9, Abdulkadir Babagba Street, Works & Housing Phase II, Gwarinpa, Abuja
1162	FCT/0502	Quality Care Clinic	Dawaki Extension, Behind News Engineering, off Gwarinpa Estate, Abuja
1163	FCT/0503	Accolade Medical Clinic Limited	Flat 5 Block 1 Kujama Street, behind Shopping Center, Area 2, Garki Abuja
1164	FCT/0506	Rachel Hospital	Plot 23, Onitsha Street, Off Gimbiya, Garki, Abuja

1165	FCT/0507	N & S Clinic & Maternity	Area C Junction, Nyanya, Abuja
1166	FCT/0508	PAAFAG Hospital & Medical Diagnosis Centre	FHA Lugbe FHA Estate, Lugbe, Abuja
1167	FCT/0510	Northland Medical Centre	Okata House Off Old Karu Road, Nyanya, Abuja
1168	FCT/0511	Bwari Medical Centre	Bwari, Abuja
1169	FCT/0512	Bethel Dental Clinic	Nwora Plaza, Plot 1103 Aminu Kano Crescent, Wuse II, Abuja
1170	FCT/0542	Capital Doctors Clinics & Maternity	ECWA Church Road, Pasali, Kuje, Abuja
1171	FCT/0543	Diamond Crest Hospital Ltd	Opp. Fed. High Court, Zuma, Abuja
1172	FCT/0546	Garki Hospital	Area 8, Garki, Abuja
1173	FCT/0549	Allison Clinic & Maternity	Off Prison Road, Pasali, Kuje, FCT
1174	FCT/0550	Hillcrest Clinics	19 Orau Street Off Nouakchott Street Wuse Zone 1
1175	FCT/0552	Dave Vivian Hospital and Maternity	Behind Shining Star Nursery & Primary School Kubwa
1176	FCT/0554	Hi-Fi Clinics	40 Suez Crescent, Ibrahim Abacha Estate Wuse Zone 4
1177	FCT/0556	Fill Medical Centre	Plot 9 Gado Nasco Road, Phase ll site ll Kubwa
1178	FCT/0559	Police Hospital	Behind Area 1 Shopping Complex, Area 1 Garki
1179	FCT/0562	Allison Clinic & Maternity	Off Prison Road, Pasali, Kuje
1180	FCT/0563	Fortress Hospital	8 Gender Close, Off Gender Street, Wuse Zone 1
1181	FCT/0566	St George's Clinic	Sector F Corner Shop, Federal Housing Estate, Airport Road, Lugbe
1182	FCT/0568	University of Abuja Medical Centre, Abuja	GWAGWALADA LGA
1183	FCT/0571	Kapital Hospital and Maternity Ltd	29 Yaounde Street, Zone 6, Wuse
1184	FCT/0573	Kelina Medical Center	116, 3rd Avenue, Gwarinpa Estate
1185	FCT/0575	Clinic Care Medical Services	35 Nouakchott Street, Wuse Zone 1
1186	FCT/0582	AHQ Clinic	Abuja
1187	FCT/0583	NAF Sick Bay	Abuja
1188	FCT/0584	Sisters of Nativity Hospital	Jikwoyi Phase 1, Behind Asaka Cement
1189	FCT/0586	Pogma Clinic & Maternity	Behind Aso Savings and Loans, Jikwoyi Phase 3
1190	FCT/0589	Living Rock Hospital	Behind High Court, Zuba
1191	FCT/0590	Promesa Medical Centre	House 57, 321 Road off 3rd Avenue, Gwarinpa
1192	FCT/0593	Lifeway Medical Centre	60 Harper Street, Wuse Zone 7
1193	FCT/0595	Kubwa Muslim Community Hospital (KMC)	Phase 4, Opposite Jumat Mosque Kubwa
1194	FCT/0596	Jikwoyi Medical Centre	3 Esu Jetta Road, Phase 1, Jikwoyi
1195	FCT/0597	Wamakko Hospital and Maternity	Plot 490 Cadastral Zone C2 Gwarinpa 1

1196	FCT/0598	Crystal Paediatric Clinic Limited	1, Ikot Ekpene street, opposite National Assembly Clinic, Area 11, Garki
1197	FCT/0599	AJ Lifetime Hospital and Maternity Limited	1 Korinya Street off Gado Nasco, Kubwa
1198	FCT/0600	Royal Specialist Hospital	2 Kukawa Street, off Gimbiya Street, Area 11, Garki
1199	FCT/0601	Tolbert Specialists Hospitals Ltd	3 Substation Close off Constitution Avenue, Gaduwa Estate
1200	FCT/0603	RF Hospital	Zone A, Relocation Estate, Gidan Mangoro
1201	FCT/0604	Jethrone Hospital and Diagnostic Centre	Plot 44, NYSC Road, Gbazango
1202	FCT/0605	Well Care Clinics	Opp. Abattoir, FRCN Road, Gwagwalada
1203	FCT/0607	Kenony Clinic Limited	Plot B144 Adisa Close 2/2 ?Off Dasuki Road, Kubwa
1204	FCT/0608	Peacecare Clinic	46 3rd Avenue, Opp. Zenith Bank, Gwarinpa
1205	FCT/0609	View Point Hospital	4 Sunday Awoniyi Street, 3rd Avenue, near MIB Plaza, Gwarinpa
1206	FCT/0610	Rubochi General Hospital	Rubochi Town
1207	FCT/0611	Samto Medical Services	4th/2nd Avenue, Supreme Court Quarters, Karu
1208	FCT/0612	Family Care Multiclinics	9, Lord Lugard Street, Asokoro
1209	FCT/0613	Primecare Hospitals	Plot 75, Road 321 off 3rd Avenue by Haman Maiduguri Crescent, Gwarinpa
1210	FCT/0614	Wellington Clinics Limited	10 Vanem Street, off Euphrates Street, Maitama
1211	FCT/0615	Vintage Medical Centre	F Road, CITEC Estate, Mbora District
1212	FCT/0618	Henry Akingba Medical Center	Prisons Village, Bill Clinton Drive, along Airport Road
1213	FCT/0619	Medimax Hospital	11, 612 Road (7th Avenue) Gwarinpa
1214	FCT/0622	Socu Hospital	Ungwan Dogo Street, off Transformer Bus Stop, Ngwa Dogo, Orozo
1215	FCT/0623	Nizamiye Hospital Limited	Plot 113 Sector S, Cadastral Zone, Life Camp
1216	FCT/0624	Micelo Clinics	33/35 Obafemi Awolowo Street, Phase 1, Trademore Estate via VON, Lugbe
1217	FCT/0625	Laura Hospital	Kogo, Bwari
1218	FCT/0628	First Call Hospital & Diagnostic Center	Plot 167, Cadastral Zone B09, Beside Lakeview Homes, Phase 2, Kado-Karshi along New GwarinpaBanex Express Road

1219	FCT/0631	Aviation Medical Clinic	Nnamdi Azikwe Airport
1220	FCT/0632	Camp Medical Centre	Regional Market, Dei-Dei, Beside First Bank
1221	FCT/0635	Lifeway Trinity Clinic & Maternity	Plot 6, House 2, Behind Deeper Life Bible Church, Phase 4, Jikwoyi
1222	FCT/0636	Vinette Hospital & Maternity	14, Gindiri Street, Garki 2
1223	FCT/0638	EFAB Medical Centre	28, 2nd Avenue (Adjacent to Mosque) RFAB City Estate, Mbora 1, Life Camp
1224	FCT/0639	Zenith Medical and Kidney Centre	28 Ubiaja Crescent, Garki 2
1225	FCT/0644	Massan Health Clinic	Road 1, Flat 16, Near Ajis Prime Resort, Lugbe F.H.A, Abuja
1226	FCT/0645	Open Healthcare Ltd	11, Ademola Awosika Road, Kubwa Ext. 3, off Bwari-Dutse Expressway, Abuja
1227	FCT/0650	Salama Infirmary Hospital and Maternity	No 24, 64 crescent, After Chaley Boy Boulevard, Gwarinpa
1228	FCT/0651	Chivar Clinics and Urology Center	30, Agadez street, off Aminu Kano crescent, Wuse 2, Abuja
1229	FCT/0652	Zikora Hospital	Polt 16A, End of 69 road (Off 6th Avenue - Galadima Estate) Gwarinpa, Abuja
1230	FCT/0653	Primus International Super Specialist Hospital	Karu New Extension, Behind Customs Quarters, Karu, Abuja
1231	FCT/0654	Irefa Hospital Ltd	Opp Ave Maria Boy's College, Nyanya, Phase IV, Abuja FCT
1232	FCT/0655	Penthouse Clinics Ltd	Plot 170, Kado Karimu Rd, after Kado Fish Market, Abuja – FCT.
1233	FCT/0656	AT- Taqwa Clinic & Maternity Ltd	Plot 653, Zone 303, Adjacent Family Worship Centre, Wuye District, Abuja-FCT.
1234	FCT/0657	Fountain sides Hospital	Plot 73A 1st Avenue opp. Bonny B Corner Shop Gwarimpa Abuja
1235	FCT/0658	Cedacrest Hospital	Plot 195 Apo-Dutse way off. Oladipo Diya way, by Amina Court Estate, Gudu Abuja
1236	FCT/0659	Cornelian Maternity & Rural Healthcare Centre	Holy Child Lane, Opposite Loyola Jesuit Maingate, Gidan Mangoro, Abuja-FCT.
1237	FCT/0660	Icon Eye Clinic	Suite B16, Shakir Plaza, by Assemblies of God, Area 11, Garki, Abuja, FCT
1238	FCT/0661	Samrock Hospital	Plot 1013-1014 Army Post Service Scheme, Kurudu
1239	FCT/0662	Gilgal Hospital	Plot 1013-1014 Army Post Service Scheme, Kurudu, FCT
1240	FCT/0663	Zaman Clinic & Maternity	25 Road 21B, Babangida Rd. FHA, Lugbe, Abuja

1241	FCT/0665	Vic-James Hospital	Plot 211, Cadastral Zone 07-05, Gbazango Extension, Kubwa, Abuja
1242	FCT/0667	Nigerian Security and Civil Defence Corps Medical Center	Sauka Academy (Beside Immigration Passport Office), off Airport Road, Abuja.
1243	FCT/0668	Life Point Medical Center	Plot 64, Moses Majekodummi Crescent, Utako District, Abuja
1244	FCT/0670	Ila Universal Hospital	Plot MFI, AAI Layout, Along FUNTAJ International School, Kuje
1245	FCT/0672	Marie Stopes Clinic	Plot 233 FHA Estate, After Corner Shop, (Opposite Custom Clinic), Phase 1, Karu-Site, FCT
1246	FCT/0673	Modern Health Hospital	Plot 506, Excellence and Friends Street, Off Arab Road, Kubuwa Extension 2, After Dunamis Church, Kubuwa, Abuja
1247	FCT/0674	Louis-Pastuer Hospital LTD	813C, 64 Crescent, Gwarinpa 2 Estate, Map Global Junction, Abuja
1248	FCT/0676	LeahJada-Joseph Medical Centre	Plot 7, Chris Chukwukelu Street, Behind Central Mosque, Gwagwalada, F.C.T
1249	FCT/0678	Healthland Hospital and Diagnostic Centre	Plot 12, Sauki Extension, Off FCMB, Kuje, F.C.T
1250	FCT/0679	Jehova Adonai Eagle Hospital	314, Street B, Phase 1, Gwagwalada, F.C.T
1251	FCT/0680	Joyland Medical Centre & Children Hospital	Main Close, Valencia Garden Estate, Dakwo District, F.C.T
1252	FCT/0681	The Central Clinic	1, Sakete Close, Kampala Street, Wuse II, Abuja
1253	FCT/0685	LeahJada-Joseph Medical Centre	Plot 7, Chris Chukwukelu Street, Behind Central Mosque, Gwagwalada, F.C.T
1254	FCT/0688	Maida Hospital	94, 43rd Crescent, Off 4th Avenue, Gwarinpa, Abuja
1255	FCT/0690	Nigeria Atomic Energy Commission Staff Clinic	SHETCO Complex, Sheda, F.C.T
1256	FCT/0695	Guinea Savannah Medical Center	Communal Center, NNPC Housing Estate Area 2,Garki
1257	FCT/0697	MD Abubakar Hospital	Police Baracks, Dei-Dei
1258	FCT/0698	Police Cantonment Clinic	Area 11, Garki Abuja
1259	FCT/0699	De Rose Of Sharon Hospital & Maternity Jikwoyi Abuja Limited	Abuja
1260	FCT/0702	Happy Eye Vision Clinic.	40 Suarez Crescent, Ibrahim Abacha Estate Wuse Zone 4 Abuja FCT.

1261	FCT/0704	White dove Hospital	AA3 Quarters Extension, New Enal International School, Kuje F.C.T
1262	FCT/0705	Standard care Medical Centre	Ishaya Ikwo Ibrahim Road, Navy Estate Junction, Karshi, Abuja F.C.T
1263	FCT/0707	Martin Luther- Obama Specialist Hospital.	51, Yellow House, Loyola Street, Opp. Loyola Jesuit College Staff Gate, Gidan Mangoro, Abuja F.C.T
1264	FCT/0711	Modelcare Hospital	5, Jaba Close Off Dunukofia Str. By FCDA Minister`s Gate, Garki Area 11, Abuja F.C.T
1265	FCT/0712	Vecad Clinic Ltd	4 Umuahia Close, Off Emeka Anyaoku Str. Garki Area 11 F.C.T
1266	FCT/0714	First Hospital And Maternity LTD	Jikwoyi- Karshi Express Way, Opp. Old Timber Shed, Jikwoyi, Abuja F.C.T
1267	FCT/0716	Freedomscan Medical Centre	Area B, Last Road, Nyanya, Abuja F.C.T
1268	FCT/0718	Hammersmith Medical Centre	No 10, Justice Okeke Street, Trademore Estate Phase 3, Lugbe, Abuja F.C.T
1269	FCT/0722	Fountain Trust Medical & Diagnostic Centre Ltd	No. 6 Pastor Enoch Adeboye Street Off 24 Crescent, 2nd Avenue Gwarimpa Estate, Abuja F.C.T
1270	FCT/0724	Jedam Spring Hospital Limited	Plot 528, Family Along Funtaj Rd. Kuje F.C.T
1271	FCT/0725	Community Hospital & Diagnostic Centre	Plot 342 Kubwa Extension 111 Dutse Alhaji Abuja F.C.T
1272	FCT/0726	Kodesh Hospital LTD	Close 5, Phase2, Army Housing Estate, Kurudu, Abuja. F.C.T
1273	FCT/0728	Yalen Medical Services Nigeria Limited	Yalen Medical Services Nigeria Limited
1274	FCT/0730	Menovas Consultants Clinic	Plot 53 Road B, Bentell Villa Estate Gaduwa District, Abuja F.C.T
1275	FCT/0731	Yabisam Hospital	2 Hidu Road Angwan – Tiv, Tudun Wada Behind FHA Lugbe, Abuja FCT
1276	FCT/0732	Jodi Medical Center	2 Almurie Omanze Street, Off Ladoke Akintola Boulevard, Garki 2, Abuja F.C.T
1277	FCT/0734	Life Medical Spa And Specialist Centre.	8,Justice Mohammed Bello Street Asokoro Abuja F.C.T
1278	FCT/0739	Premier Heart Hospital and Clinics	45 Fourth Avenue F.H.A Gwarimpa Abuja
1279	FCT/0742	Tahira Hospital	Block D2 F.C.D.A Estate Kubwa. Abuja F.C,T

1280	GM/0001	General Hospital Billiri	Along Yola Road,Gombe
1281	GM/0002	Federal Teaching Hospital, Gombe	Gombe
1282	GM/0003	Doma Hospital	5. Commercial Area,Gombe
1283	GM/0004	Miyelti Hospital	A.Y. Guest House, Dukku Road
1284	GM/0005	Yarma Memorial Hospital	Buba Shongo Quarters,Gombe
1285	GM/0006	Bajoga General Hospital	Funakaye Lga
1286	GM/0007	Dadin Kowa Primary Health Centre	Gombe
1287	GM/0009	Musaba Medical Centre	Dukku Road
1288	GM/0010	Hamdala Specialist Clinic	Near Pantami Stadium,Gombe
1289	GM/0011	Salem Medical Centre	Pantami
1290	GM/0012	Metro Consultants	Federal Low Cost Housing Estate, along Goodluck Jonathan rd, near All Saint College, Gombe
1291	GM/0013	Sabana Specialist Hospital Ltd.	Federal Lowcost, Gombe
1292	GM/0014	Specialist Hospital, Jekadafari	Jekadafari
1293	GM/0025	General Hospital, Kaltungo	Kaltungo,Along Yola Road, Gombe
1294	GM/0026	Kumo General Hospital	Kumo L.G.A
1295	GM/0028	Bimma Med. Centre	Fed. Lowcost, Gombe
1296	GM/0036	General Hospital Dukku	Dukku Town
1297	GM/0037	General Hospital Zambuk	Zambuk Town
1298	GM/0045	DSS Clinic	Gombe, Gombe State
1299	GM/0046	Police Clinics, Gombe	Gombe State
1300	GM/0047	Yambu Dok Clinic	Billiri, Gombe State
1301	GM/0060	Panda Maternity Clinic	Panda
1302	GM/0067	Gadawo Health Clinic	Gadawo
1303	GM/0068	Jabba Health Clinic	Jabba
1304	GM/0069	Kalshingi Health Clinic	Kalshingi
1305	GM/0070	Tabra Maternity Clinic	Tabra
1306	GM/0071	Akko Health Clinic	Akko
1307	GM/0072	Zongomari Health Clinic	Zongomari
1308	GM/0073	Bula Health Clinic	Bula
1309	GM/0074	Lawanti Health Clinic	Lawanti
1310	GM/0075	Shongo Health Clinic	Shongo
1311	GM/0076	Tumu Health Clinic	Tumu
1312	GM/0077	Pindiga Health Clinic	Pindiga
1313	GM/0078	Chilo Family Support Maternity Clinic	Chilo
1314	GM/0079	Yarima Shehu Maternity Clinic	Akko
1315	GM/0080	Goma Health Clinic	Goma
1316	GM/0081	Tukulma Maternity Clinic	Tukulma
1317	GM/0082	Maiganga Maternity Clinic	Maiganga
1318	GM/0083	Kundullum Dispensary	Kundullum
1319	GM/0084	Garko Dispensary	Garko
1320	GM/0085	Kaltanga Dispensary	Kaltanga
1321	GM/0086	Piyau Dispensary	Piyau
1322	GM/0087	Zabin Kani Dispensary	Zabin-Kani
1323	GM/0088	Kembu Dispensary	Kembu
1324	GM/0089	Wurodole Dispensary	Wurodole
1325	GM/0090	Badara Dispensary	Badara
1326	GM/0091	Tulmi Dispensary	Tulmi

1327	GM/0092	Gokaru Dispensary	Gokaru
1328	GM/0093	Lombo Dispensary	Lombo
1329	GM/0094	Tambie/Yolo Dispensary	Tambie
1330	GM/0095	Birnin Bolawa Primary Health Clinic	Birnin Bolawa
1331	GM/0096	Birnin Fulani Primary Health Clinic	Birnin Fulani
1332	GM/0097	Barwo Nassarawa Primary Health Clinic	Barwo Nassarawa
1333	GM/0098	Barwo Windi Primary Health Clinic	Barwo Windi
1334	GM/0099	Jigawa Primary Health Clinic	Jigawa
1335	GM/0100	Nafada Primary Health Clinic	Nafada East
1336	GM/0101	Nada General Hospital	Nafada East
1337	GM/0102	Sundingo Dispensary	Birnin Bolawa
1338	GM/0103	Kiyayo Dispensary	Birnin Fulani
1339	GM/0104	Madaki Lamu Dispensary	Birnin Fulani
1340	GM/0105	Guduku Dispensary	Guduku
1341	GM/0106	Dindele Dispensary	Jigawa
1342	GM/0107	Jolie Dispensary	Jigawa
1343	GM/0108	Shole Health Clinic	Nafada East
1344	GM/0109	Nyalkam Dispensary	Nafada East
1345	GM/0110	Primary Health Centre Filiya	Filiya
1346	GM/0111	Kulishin Maternity Clinic	Kulishin
1347	GM/0112	Burak Maternity Clinic	Burak
1348	GM/0113	Bangunji Maternity Clinic	Bangunji
1349	GM/0114	Karel Maternity Clinic	Karel
1350	GM/0115	Lapan Maternity Clinic	Lapan
1351	GM/0116	Gwandum Maternity Clinic	Gwandum
1352	GM/0117	Model Maternity Clinic	Shongom
1353	GM/0118	Lalapido Maternity Clinic	Lalapido
1354	GM/0119	Lashikoltok Maternity Clinic	Lashikoltok
1355	GM/0120	Kalaku Health Centre	Kalaku
1356	GM/0121	Kushi Maternity Clinic	Kushi
1357	GM/0122	Amkolom Health Clinic	Amkolom
1358	GM/0123	Torro Health Clinic	Torro
1359	GM/0124	Majidadi Maternity Clinic	Majidadi
1360	GM/0125	Popandi Health Clinic	Popandi
1361	GM/0126	Pamadu Health Clinic	Pamadu
1362	GM/0127	Labarya Maternity Clinic	Labarya
1363	GM/0128	Lasassap Health Clinic	Lasassap
1364	GM/0129	Jauro Sajo Community Health Clinic	Shongom
1365	GM/0130	Lapandimtai Health Clinic	Shongom
1366	GM/0131	Lasanjang Health Clinic	Shongom
1367	GM/0132	Labekke Health Clinic	Shongom
1368	GM/0133	Tedmuzu Health Clinic	Shongom
1369	GM/0134	301 AR (GS) MRS	Gombe
1370	GM/0137	Madi Memorial Clinic and Maternity	Tunfure Investment Estate, Gombe
1371	GM/0138	Jewel Specialist Hospital	Tunfure, Behind Baranbu Filling Station, Gombe, Gombe State
1372	GM/0139	Madina Specialist Eye & medical Center Ltd.	Madina Specialist & Medical Centre Limited

1373	GM/0140	Police Clinic	Inside Police Barracks, Billiri Gombe State
1374	GM/0141	Kumo Medical Clinic	Along Gombe-Yola Road, Kumo, Gombe state
1375	GM/0142	Federal University Staff Clinic	Kashere, Gombe State
1376	GM/0143	General Hospital Kashere	Kashere, Gombe State
1377	IM/0001	General Hospital Abor	Abor Mbaise
1378	IM/0002	Corpus Christi(Monice Memorial) Hospital	Amuzi Mbaise
1379	IM/0004	Jasman Hospital Ltd.	Udo Ezinihitte Mbaise, Imo
1380	IM/0005	Obizie People's Medical Centr	Obizi Ezinihite, Mbaise, Imo State
1381	IM/0006	Bishop Okoye Memorial Hospital	Inyishi, Ikeduru, Owerri, Imo State
1382	IM/0009	General Hospital, Okigwe	Okigwe, Imo State
1383	IM/0013	Our Lady Of Mercy Hospital & Mat.	102/104 Royce Road, Owerri
1384	IM/0015	First Concepts Hospital	105, Ikenegbu Layout, Owerri
1385	IM/0017	Angboso Specialist Hospital	Plot P6 Work Layout, Owerri
1386	IM/0018	Umezuruike Hospital	21 Umezuruike Street, Owerri
1387	IM/0019	Total Care Medical Centre	148/150 Tetilow Road, Owerri
1388	IM/0020	St. John's Hospital	9 Anokun Str. Owerri
1389	IM/0022	Ngozi Hospital & Maternity	Plot 72, Okigwe Road Layout, Owerri.
1390	IM/0023	The Chapel Grd Spec. Hospital & Maternity Ltd.	Plot 620 Works Layout, Owerri
1391	IM/0024	Ezem Medical Centre	4 Osuji Street, Owerri
1392	IM/0026	Queen Juilana . Hospital & Maternity	7, Ironsi Street, Owerri, Imo
1393	IM/0029	St. Elizabeth Hospital	10 Govt. House/Shell Camp Road, Owerri, Imo State
1394	IM/0030	Akaraugo Hospital & Maternity	3, Egbukole Street, Ikenegbu, Imo State
1395	IM/0031	Life Spring Specialist Hospital	Plot 49, Okigwe Road Layout, Owerri, Imo State
1396	IM/0032	Jone Medical Centre	Plot 189 Ikenegbu Layout Extension, through Chukwuma Nwoha Street, Owerri, Imo Sate
1397	IM/0033	Salvation Hospital & Maternity	38, Mcc/Uratta Road, Owerri, Imo State
1398	IM/0034	Ikenegbu Hospital & Maternity	Plot 11 & 13 Amadi Crescent, Okigwe Road Layout, Owerri, Imo
1399	IM/0035	St. David's Hospital	14, Mbari Street, Ikenegbu, Owerri, Imo State
1400	IM/0060	New Cross Hospital Ltd.	5, New Cross Hospital Street, Ugwuorji, Owerri

1401	IM/0061	Aladinma Hospital	Transfiguration Road, Owerri, Imo State
1402	IM/0066	Uchenna Hospital & Maternity	10/12 Eniweruzoh Crescent Owerri
1403	IM/0067	Chinyere Clinic & Maternity	14b, Onumonu Str., Owerri
1404	IM/0068	Christiana Specialist Hospital	Egbu/Emuoba Rd., Owerri
1405	IM/0070	Six C. Specialist Clinic	28/30 Orlu Road, Imo State
1406	IM/0071	St. Joseph Hospital	9, Anokun Street, Owerri
1407	IM/0072	St. Luke's Hospital	No 2 Ihioma Road Orlu, Imo State
1408	IM/0073	Capital Health Hospital	44 Oyima Street,Owerri, Imo State
1409	IM/0090	New Life Maternity Home	Ikechukwu Estate, Nkworji-Uratta, Owerri
1410	IM/0091	Ivory Clinic	Okigwe, Imo State
1411	IM/0092	Imo State University Teaching Hospital	Orlu, Imo State
1412	IM/0093	Imo State Specialist Hospital, Owerri	New Owerri, Imo State
1413	IM/0094	Federal Medical Centre, Owerri	Owerri, Imo State
1414	IM/0095	34 AB MRS, Owerri	Owerri, Imo State
1415	IM/0096	342 AR MRS, Owerri	Owerri, Imo State
1416	IM/0097	Holy Rosary Hospital	Owerri-Umuahia Rd, Nkwogwe Mbaise
1417	IM/0098	Solid Rock Hospital & Maternity	No 1 Solid Rock Hospital Road Opp. NNPC Filling Station Cristal Rd. Owerri
1418	IM/0100	Uchendu Hospital	98 Douglas Rd. Owerri
1419	IM/0112	Federal Polytechnic Medical Centre, Nekede	Nekede, Owerri, Imo State
1420	IM/0115	Federal University of Technology Medical Centre	Ihiagwa, Owerri, Imo State
1421	IM/0118	Amanda Hospital	Plot 412/415 Works Layout, Owerri, Imo State
1422	IM/0121	DSS Clinic	Owerri, Imo State
1423	IM/0122	Police Hospital, Imo	Owerri, Imo State
1424	IM/0124	International Christian Hospitals	11 WAAST AVENUE, IKENEGBU
1425	IM/0125	EBUBEDIKE MEMORIAL HOSPITAL	71, OPARANOZIE STR. OWERRI

1426	IM/0128	CHRISTIAN MEDICAL CENTER	NWAORIEUBI
1427	IM/0129	ETITI MEDICAL CENTER	Etiti Isinweke Ihitte Owerri
1428	IM/0130	ROSANNA MEDICAL CENTER	UMUKOTO NEKEDE
1429	IM/0148	Holy Family Hospital and Maternity	94 Ikenegbu Layout, Owerri
1430	IM/0168	New Amex Specialist Hospital	Plot 989 Amakohia, Owerri
1431	IM/0170	New Creation Medical Consultant	19 Obazu Road, Nworji Mbieri, Owerri
1432	IM/0173	Imo State University Teaching Hospital Primary Health Centre	Orlu
1433	IM/0189	New Annex Specialist Hospital	Plot 989, Amakohia, Owerri
1434	IM/0191	St. Joseph’s Clinic & Maternity	Enyiogugu, Aboh Mbaise, Imo State
1435	IM/0193	Edozie Clinic & Maternity	W.W Acholonu Memorial Mission, Awaka, Imo State
1436	IM/0194	Bettina Medical Centre	11 Old Okigwe Road, Owerri
1437	IM/0195	Mother of Mercy Hospital	Umuna Orlu
1438	IM/0196	Alvan Ikoku Medical Center	Owerri
1439	IM/0197	Vaden Specialist Clinic and Maternity	4B, Solid Rock road, off World Bank Road, Owerri
1440	IM/0201	1st Royal Care Hospital Ltd	Off Port Harcourt Road, Behind Ebere Link Filling Station, New Owerri, Imo State
1441	IM/0204	Steps of Faith Hospital	No. 9, Mere Street, Owerri, Imo State
1442	IM/0206	Alpha Clinic & Maternity	23, Nwafor Street, Orji Uratta, Owerri, Imo State
1443	IM/0207	Floxy Pan-Opticum Eye Specialist Hospital	464, Tony Okeke Street, Prefab, Owerri, Imo State
1444	IM/0208	FamilyCare Specialist Clinics	New Owerri, Imo State
1445	IM/0210	Geoffrey Memorial Healthcare	No. 43, Okigwe Road, Owerri, Imo State
1446	IM/0211	Umuowa Cottage Hospital & Maternity	Imo Airport Road Junction, Owerri, Imo State
1447	IM/0212	Salvation Hospital and Maternity	Nwaorienkpu, Ekwe, Imo State
1448	IM/0214	Frank Medical Centre	Eke Nguru Mbaise, Imo State
1449	IM/0215	Digoe Hospital	5,Orlu Road Owerri Imo State
1450	IM/0217	Digoe Hospital	5,Orlu Road Owerri Imo State
1451	IM/0218	St. Damian`s Hospital	Okporo Orlu, Imo State

1452	IM/0219	Zennia Hospital & Maternity	No 1 Road 17, Federal Housing Umuguma, Owerri Imo State
1453	IM/0221	Mark of Glory Specialist Hospital	1st Bus Stop, Emeremadu Street, Owerri Imo State
1454	IM/0223	Crown Medical Centre	Nnarambia Ahiazu Mbaise, Imo State
1455	IM/0224	Austin Graces Hospital	14 Eni Njoku Street, Ikenegbu Layout, Owerri Imo State
1456	IM/0225	Ave Maria Medical Centre	Ogbe, Ahiara Imo State
1457	IM/0226	Owerri Sickbay	Road 24, Federal Housing Estate, Umuguma, Owerri, Imo State
1458	IM/0228	Blessing of The Lord Hospital	Site & Services, World Bank, New Owerri Imo State
1459	IM/0229	Eastern Summit Specialist Clinics &Maternity	37 Orlu Road, Amakaohia, Owerri Imo State
1460	IM/0230	Hossana Hospital and Maternity	Eziama, Logara, Ngor-Okpala Imo State
1461	JG/0001	General Hospital, Dutse	Dutse, Jigawa State
1462	JG/0002	Jahun General Hospital	Jahun, Jigawa State
1463	JG/0003	General Hospital, Hadejia	Hadejia, Jigawa State
1464	JG/0005	General Hospital, Gumel	Gumel, Jigawa State
1465	JG/0006	General Hospital, Kazaure	Kazaure, Jigawa State
1466	JG/0007	Zainab Memorial Hospital	Kazaure, Jigawa State
1467	JG/0008	General Hospital, Ringim	Ringim, Jigawa State
1468	JG/0009	Federal Medical Centre, Birnin Kudu	Brnin Kudu, Jigawa State
1469	JG/0017	DSS Clinic	Dutse, Jigawa State
1470	JG/0019	Police Clinics, Jigawa	Jigawa State
1471	JG/0024	General Hospital Babura	Babura
1472	JG/0026	Federal University Dutse Medical Clinic	Federal University Campus
1473	JG/0027	Rasheed Shekoni Specialist Hospital	Dutse, Jigawa
1474	JG/0028	Gunduma Health System Council, Dutse General Hospital	Kiyawa Road, Dutse, Jigawa State
1475	JG/0029	Albarka Clinics	Beside Inuwa Dutse Estate, Jigawa State
1476	JG/0034	General Hospital, Kafin Hausa	Kafin Hausa
1477	KB/0001	General Hospital Argungu	Argungu
1478	KB/0003	Federal Medical Center - Birnin Kebbi	Birnin Kebbi
1479	KB/0008	Sir Yahaya Memorial Hospital	Ahmadu Bello Way, Birnin Kebbi
1480	KB/0020	General Hospital, Yauri	Yauri
1481	KB/0021	1 Bn MRS Birnin Kebbi	Birnin Kebbi, Kebbi State
1482	KB/0022	223 Lt Tk Bn MRS Zuru	Zuru, Kebbi State
1483	KB/0023	Godiya Hospital	Bye-Pass Rd Wasarawa, Birinin Kebbi
1484	KB/0024	DSS Clinic	Birnin Kebbi, Kebbi State
1485	KB/0025	Police Clinics, Kebbi	Kebbi State
1486	KB/0026	Nakowa Clinic Ltd	Sokoto - Kontagora Road, Yauri, Kebbi State
1487	KB/0027	Martha Bamaiyi General Hospital Zuru	Zuru Bye-Pass Behind First Bank, Zuru, Kebbi State
1488	KB/0037	Waziri Umar Federal Ploytechnic	Gwandu Gwaji Road, Kebbi state

1489	KB/0038	D D G Medical Centre	Behind OANDO Filling Station, Birnin Kebbi, Kebbi State.
1490	KB/0039	University Clinic (F.U.B.K)	Federal University Birnin Kebbi, Kebbi State
1491	KD/0005	Air Force Medical Centre Mando	Kaduna
1492	KD/0008	Abdul-Azeez Memorial Hospital Clinic & Maternity	D.B. 10, Kabala West Road, Near Amaboni Hotal Tudun Wada,Kad
1493	KD/0010	Giwa Hospital And Specialist Clinic	2. Giwa Road Abakpa Kaduna
1494	KD/0012	Alba Clinic & Medical Centre	25, Constitution Road, Kaduna
1495	KD/0013	Sefa Specialist Hospital	3, College Road, Kaduna
1496	KD/0014	Covenant Hospital & Maternity	Au 6, Benin Street Kaduna.
1497	KD/0015	Belmont Specialist Hospital	S.S. 5 Nasarawa Road, Kaduna
1498	KD/0016	Jowako Hospital	F 15 Jos Road, Kaduna
1499	KD/0017	Kagoro Sheshan Clinic	Bb11 & 12 Nupe Road, Kaduna
1500	KD/0018	Chasel Hospital	15, Isa Kaita Road, Kaduna
1501	KD/0019	Garkuwa Specialist Hospital	1a Sultan Road, Off Isa Kaita Road, Kaduna
1502	KD/0020	Iyali Hospital & Maternity	7, Kagoro Close, Off Gwari Crescent, Ungwar Rimi Gra, Kaduna
1503	KD/0021	Rimi Clinics & Maternity Nig. Ltd.	24, Kubaka Road, Off Ja Abdulkadir Road, Ungwan Rimi, Kaduna
1504	KD/0022	Salem Hospital Clinics & Mat.	B4 School Road, Ungwar Rimi, Kaduna
1505	KD/0026	Eagle Hospital, Kaduna	No. 3, Kujama Street/Gwari Road, Sao tasha, kaduna
1506	KD/0028	Barau Dikko Teaching Hospital, Kaduna	Barau Dikko Teaching Hospital, Kaduna
1507	KD/0029	Jinya Specialist Hospital	7, Bida Road Kaduna
1508	KD/0030	Kabala Hospital	F 1a Market Road, Kaduna
1509	KD/0076	Salamatu Hospital & Maternity	2, Bimin Yero Road, Off Kabala West, Road, Kaduna
1510	KD/0078	Fountain Hospital & Maternity	Ap 13 Community Bank Road, Prp Bus Stop/Gamagira, Tudun Wada
1511	KD/0080	Shehu Kangiwa Medical Centre	(Sick Bay), Kaduna Polytechnic, T/Wada Kaduna
1512	KD/0081	Yusuf Dantsoho Hospital	T/Wada, Kaduna
1513	KD/0082	Maneks Hospital Ltd.	A1 Block 5, Makera Road, Kakuri Kaduna
1514	KD/0083	Amina Hospital Ltd.	Gwari Avenue, Kachia Road, Kaduna
1515	KD/0084	Ishaku Hospital	Gwari Avenue, Kachia Road, Kaduna
1516	KD/0085	Giltoe Aris Specialist Hospital	17/19 Maiduwa Road, Near Barnawa Market, Kaduna
1517	KD/0086	C Jay Medical Centre	Near Dambo Int''''L College, No 13, Kubani Crescent, Barnawa Kd
1518	KD/0087	Gwamna Awan Hospital	Nasarawa, Kaduna

1519	KD/0088	Sabon-Tasha Bengola Hospital	Opp. Command Sec. School, Ung. S
1520	KD/0089	44 Armed Forces Reference Hospital	Kaduna
1521	KD/0090	Federal Neuropsychiatric Hospital	Barnawa, Kaduna
1522	KD/0111	Tim Unity Specialist Hospital	Bz 104, Ogori Road, Off Kabala Road, Sardunana Crescent Kad
1523	KD/0112	Almadina Clinic	No. 28 Queen Elizabeth Road, Gra Zairia
1524	KD/0113	Ladiya Hospital	64, Benin Street, Zaira
1525	KD/0123	Salama InfIrmary Hosp. & Maternity	Sokoto Road, Kwangla, Zaira
1526	KD/0124	St. Luke's Anglican Hospital	Wusasa Zaira
1527	KD/0126	Hajia Gambo Sawaba Gen. Hospital	Kofar-Gayan, Zaria
1528	KD/0148	Sultan Clinics	Nm 12b, Constitution Rd., Kaduna
1529	KD/0149	Ahmadu Bello University Health Services (Main Campus)	Samaru Zaria
1530	KD/0151	DSS Clinic	Kaduna, Kaduna State
1531	KD/0152	1 Div Hospital	Kaduna, Kaduna State
1532	KD/0153	Nigerian Defence Academy	Kawo, Kaduna
1533	KD/0154	Infantry Centre & Sch. Armed Forces CSC Med. Centre	Jaji, Kaduna
1534	KD/0155	Depot Nigerian Army/Nig. Army Military School Med. Centre	Chindit Barracks, Zaria, Kaduna
1535	KD/0157	National Eye Centre	Mando Road, Off Nnamdi Azikiwe Way, Kaduna
1536	KD/0160	General Hospital, Giwa	Giwa, Kaduna State
1537	KD/0162	St Patrick Ibrahim Yakowa Memorial Hospital, Kafanchan	Kafanchan, Kaduna State.
1538	KD/0163	Savannah Poly Clinic	Samaru, Zaria.
1539	KD/0165	Moufay Clinic	11A College Road, Kurmashi, Kaduna
1540	KD/0170	Mends Specialist Hospital & Aviation Medical Centre	5, Abba Kyari Close, Ungwan Rimi GRA, Kaduna
1541	KD/0171	Crystal Clinic & Maternity	M2 Poly Road, T/Wada, Kaduna
1542	KD/0173	312 AR MRS, Kaduna	Kaduna, Kaduna State
1543	KD/0174	29 Bn MRS, Kaduna	Kaduna, Kaduna State
1544	KD/0175	7 Bn MRS, Jaji	Jaji, Kaduna State
1545	KD/0176	11 FER MRS, Zaria	Zaria, Kaduna State
1546	KD/0182	Abi Clinics & Hospital	BZ 104 Ogori Road, Sardauna Crescent, Kaduna
1547	KD/0183	Cottage Hospital/Comprehensive Health Centre	Police College, Kaduna
1548	KD/0187	Monarch Specialist Hospital	N/k 12, Arochukwu Road, Kaduna
1549	KD/0188	Dana Clinic	Old Airport Road, Mando, Kaduna
1550	KD/0189	Prime Specialist Hospital	Az 16 Bakori Road, Kaduna
1551	KD/0190	Anna-Kitch Medical Centre	15, Wusasa Road, Zaria
1552	KD/0192	New Era Hospital & Maternity	8, Funtua Street, Kafanchan
1553	KD/0193	Multi Clinic Nig. Ltd.	26A Yahaya Road, Unguwa Rimi G.R.A., Kaduna
1554	KD/0194	McRoyal Hospital	Sabon Gari, Kachia

1555	KD/0195	Oxford Hospital	38, Airforce Road, Samaru RD, Makera, Kaduna
1556	KD/0196	Restoration Hospital Inc.	AL 1, Wushishi RD, BU Lagos St., Kaduna
1557	KD/0197	Hitaf Specialist	D% Garbajar Abdulkadir Rd, Off Rimi drive, U/Rimi, Kaduna
1558	KD/0199	General Hospital Makarfi	Makarfi Town
1559	KD/0200	St Gerard Catholic Hospital	Kakuri Kaduna
1560	KD/0201	National Teachers Institute Staff Clinic	Km 5 Kaduna-Zaria Expressway, Kaduna
1561	KD/0223	A B U Teaching Hospital, Shika	Shika, Zaria, Kaduna State
1562	KD/0226	Biba Hospital	AE 7, Matazu Close, off Rigachukwu Road, T/Wada, Kaduna
1563	KD/0228	Al Mansur Specialist Hospital	No. 7A Wurno Road Opposite KSMC, Kaduna
1564	KD/0229	Police Clinics, Kaduna	Kaduna State
1565	KD/0230	Jibrin Mai-Gwari Hospital	Birnin Gwari, Kaduna
1566	KD/0231	National Board for Tech. Education (NBTE), Medical Centre	Plot B Bida Road, Kaduna
1567	KD/0235	Ag-Lima County Hospital & Maternity	No. 16, College Road Sabon Tasha, Kaduna
1568	KD/0236	Barka Specialist Hospital	AU5 Gamagira Road, Barkin Ruwa, U/Sanusi, Kaduna
1569	KD/0238	Grace Memorial Specialist Hospital	Dan-Magaji New Market Road, Zaria, Kaduna
1570	KD/0239	Horeb Specialist Hospital	4 Force Road Maraba Estate, Kaduna
1571	KD/0240	Jema'a Hospital	90 Sarkin Pawa Road, Samaru, Zaria, Kaduna
1572	KD/0241	Jicon Hospital & Specialist Clinic	No. 4 Danwaire Street, Kaduna
1573	KD/0242	Maraba Clinic and Maternity	Shehu Idris Street Kabama Medium Housing Estate, Zaria Kaduna
1574	KD/0243	Mashi Hospital & Specialist Clinics	17 Sabo-Line Kurmin-Mashi, Kaduna
1575	KD/0244	Mayfair Clinic Nigeria Ltd.	8 Crescent Rd Sabon Gari, Zaria
1576	KD/0247	Outfit Hospital Ltd	Marafa Close Off Independence Road, Opp ASD City Mall. Kaduna
1577	KD/0248	Rakiya Memorial Hospital & Maternity Inc.	JJ 9, Ibadan Street, Kaduna
1578	KD/0249	Saby Hospital	HI Kagoma Road, Kaduna
1579	KD/0250	Saidu Dange Railway Zaria	Sokoto Road Railway Hospital, Zaria
1580	KD/0251	Senda Poly Clinic	37, Dawaki Bassa Road, Kakuri, Kaduna
1581	KD/0252	Skills Specialist Clinic	No. 16 Kurmi Avenue, Kabala Costain, Kaduna
1582	KD/0253	Sovanel Orthopaedic Clinic & Med. Clinic	Abubakar Kigo Rd, New Extension, Kaduna

1583	KD/0254	Wilbasun Hospital & Maternity	No. MB28 Massallaci Road, Narayi, Kaduna
1584	KD/0280	Diamond Specialist Hospital	4 MM Lawan Jaafaru Isa Road, GRA, Off Isa Kaita Road
1585	KD/0281	Nigerian Railway Hospital	Railway Compound, Kafanchan, Kaduna State
1586	KD/0282	Chaha Eye Hospital & General Clinics	42B Jabi Road off Alkali Road GRA Kaduna
1587	KD/0284	Zaria Clinic & Medical Centre	16 Hospital Road T/Wada, Zaria
1588	KD/0288	Royal Medical Centre	Opp. Kurmin Marshi Police Station, Kaduna
1589	KD/0290	Calvary Clinic & Maternity	2 Kano Street, By Hospital Road, Kafanchan
1590	KD/0294	Salvation Hospital & Maternity	2B Algbria Crescent, Barnawa, Kaduna
1591	KD/0296	Fomwan Hospital	28 Musana Road, Malali, Kaduna
1592	KD/0298	Musaka Specialist Hospital	10B Nagwamatse Road, U/Rimi, Kaduna
1593	KD/0308	Albarka Hospital	BZ170, Offa Road by Sardauna Crescent, Kaduna
1594	KD/0309	St Louis Hospital	Zonkwa Kaduna Road, Zangon Kataf
1595	KD/0314	Command and Staff College Clinic	Jaji
1596	KD/0315	Acada MRS	Aganu Kachia
1597	KD/0316	Arty Training School MRS	Kachia
1598	KD/0318	Federal College of Education Medical Centre	Kongo, Zaria
1599	KD/0319	Dabo Mohammed Lere Memorial Hospital	Along Jos Road, Saminaka
1600	KD/0321	Defence Industry Corporation of Nigeria, MRS	Ahmadu Bello Way, Kaduna
1601	KD/0327	Muslim Specialist Hospital	Wusasa, Zaria
1602	KD/0328	Aero Medical Centre	Nigeria College of Aviation Technology, Zaria
1603	KD/0329	Mai Jedah Memorial Hospital	K12 Kakuri road, Kawo Kaduna
1604	KD/0330	Aloaye Clinic and Maternity	4 Gaskiya Road, Zaria
1605	KD/0331	Trinity Nursing and Maternity Home	Behind JV Hotel Ungwan Boro
1606	KD/0332	Faith Nursing and Maternity	7, Magaji Close, Ungwan Pama
1607	KD/0333	Harmony Hospital	38 Nuhu Aliyu Crescent, Barnawa Layout, Kaduna
1608	KD/0334	Dami Clinic Maternity	2, Denge Road, New Afaka
1609	KD/0335	Brightway Medical Clinic	1 Narayi Road, Mountain Road, Kudenda
1610	KD/0336	Jodeb Hospital and Maternity	5, Yoruba Road, Kaduna
1611	KD/0338	Badarawa Hospital	2 Kamsalem Road, near L.E.A Primary School, Kaduna
1612	KD/0340	Crystal Clinic and Maternity	6 Makera Road, Opp, Kaduna South LGA Secretariat, Kakuri
1613	KD/0341	Pal Hospital	15, River Road, Behind Diamond Bank, GRA, Zaria

1614	KD/0342	Dallof Special;ist Hospital Limitred	136 Bayajida Roadf, Angwa Rimi, GRA Kaduna
1615	KD/0344	Nat. Inst. for Chemica Tech Medical centre	Basawa, Zaria
1616	KD/0346	Dialogue Sepcialist Clinics Ltd.	#1, Kukan Road, U/Dosa, Kaduna
1617	KD/0347	ChrysoryteHospital Nig. Ltd	Kamazao Road by Ex-Service quaters, by Refinery junction, Mahutta, kaduna
1618	KD/0348	National Institute for Chemical Technology	Medical Center, Basawa, Zaria,
1619	KD/0349	Elite international Hospital	# 1, Chikaji Road, Opp MTD Police, Zaria
1620	KD/0350	Makemvic Hospital Ltd	#59, Gwar Road (KASUPDA), off Post office, opp Step by Step Hotel, Sabon Tasha, Kaduna
1621	KD/0351	Terra Medical Center	# 5, Alh nasiru Idi close, Hanwa new extension, Zaria
1622	KD/0352	Family - Care Hospital and Maternity	#54, King Hassan (Bourmedine) Rd, Narayi High cost, Banawa Kaduna
1623	KD/0353	Mummy Maternity and Nursing Home	Kwangila, Zaria
1624	KD/0354	Al-Madinat Specialist Hospital	MM1 Sabon Birni Road, By Nassarawa Road along Kawo New Extension
1625	KD/0355	Bawa Memorial Medical Centre	#100, Hayin Mallam, Zango, Zaria
1626	KD/0358	Major Ibrahim B Abdullahi (MIBA) Memorial Hospital	# 21D, Aminu Kano road, Sabo Gari Zaria
1627	KD/0359	Rural Hospital Pambeguwa	Along Saminaka Road, Pambeguwa, Kaduna
1628	KD/0360	Mallam Sidi Shehu Dalibi Memorial Health Center	14, Hospital Rd, K/Gayan Low Cost Housing Estate, Gidan Isa Maigari, Zaria City
1629	KD/0362	Dan Giwa Memorial Hospital	By Noruin Guest House, Maraban Rido kaduna, Kaduna State
1630	KD/0364	Bio Clinics & Hospital	9/15 Mozambique Road, Barnawa Kaduna, Kaduna State
1631	KD/0366	St. Martins De-orres Hospial	Catholic Diocese of Zaria, P.O. Box 90, Wusasa, Zaria, Kaduna State.
1632	KD/0367	Godiya Hospital	XP 34, Kabala Road by Express bye pass, Tudun Wada, Kaduna State.
1633	KD/0370	Ema Hospital &Maternity Ltd	DD 6 Kabba Road, Kaduna, Kaduna State.
1634	KD/0371	Nuhu Bamalli Polytechnic	Zaria, Kaduna State.
1635	KD/0372	Fisabil Hospital	22, NIMASA Road, off Katuru Road, From Rabah Road, Badarawa, Kaduna, Kaduna State
1636	KD/0377	Gemine Hospital & Maternity	NB 6, Barnawa Dan Warri Street, Kaduna State
1637	KD/0379	Belside Hospital	No. 5 Kanta Road, Kaduna, Kaduna State

1638	KD/0380	Garden City Specialist Hospital	No.2 Sultan Road Ungwa Rimi, Kaduna State
1639	KD/0383	Adonai Rapha Medical	5, Auza Street Barnawa, Kaduna State.
1640	KD/0386	Al- Muniff Hospital	13AB, Abba Road U/Rimi Kaduna, Kaduna State
1641	KD/0388	Medical Center Kaduna State University	Tafawa Balewa Way, Kaduna State
1642	KD/0389	Sultan Hospital Nig Ltd	AH1, Makarfi Road, Rigasa New Ext. Kaduna State
1643	KD/0391	NNPC Industrial Hospital Kaduna	Km 16, Kachia Road, NNPC Housing Estate Kaduna State
1644	KD/0395	Women’s Health International	52, Hanwa GRA Zaria, Kaduna State.
1645	KD/0396	AP Smart Hospital	Gwagwada Street, Trikania, Kaduna State.
1646	KD/0397	National Ear Care Center	3, Golf Course Road, Kaduna State
1647	KD/0399	El Barau Medical and Diagnostic Centre	Benin Street, Sabon Gari, Zaria, Kaduna State
1648	KD/0400	MSK Specialist Hospital	10 Kangiwa/Makarfi Road, Rigasa Kaduna, Kaduna State
1649	KG/0001	ASCL Medical Centre, Ajaokuta	Ajaokuta Steel Co. Ltd., Kogi State
1650	KG/0005	General Hospital Dekina	Dekina
1651	KG/0007	Maternal & Child Welfare, Dekina	Dekina
1652	KG/0009	General Hospital, Idah	Idah
1653	KG/0012	Niger Hospital	
1654	KG/0015	General Hospital, Mopa	Mopa
1655	KG/0017	General Hospital, Okene	Okene
1656	KG/0019	Ecwa Hospital	Mission Road Egbe
1657	KG/0020	Federal Medical Center Lokoja	Lokoja
1658	KG/0021	General Hospital, Kabba	Kabba
1659	KG/0022	General Hospital, Isanlu	Isanlu
1660	KG/0023	General Hospital, Ankpa	Ankpa
1661	KG/0025	Ali Omeza Hospital	Lokoja
1662	KG/0026	Comprehensive Health Centre	Isanlu Town
1663	KG/0028	Comprehensie Health Clinic	Idofin
1664	KG/0030	Specialist Hospital, Obangede	Obangede
1665	KG/0031	Cottage Clinic	Odo Ere
1666	KG/0032	Femi Hospital	Edumo-Bunu
1667	KG/0033	General Hospital, Ayetoro Gbede	Ayetoro Gbede
1668	KG/0034	Township Clinic & Maternity	Ajaokuta
1669	KG/0035	Maria Gorrete Hospital	Ayingba
1670	KG/0036	General Hospital, Iyamoye	Iyamoye
1671	KG/0045	Cant MRS Lokoja	Lokoja, Kogi State
1672	KG/0046	Federal College Of Education	Federal College Of Education Okene
1673	KG/0047	Grimard Catholic Hospital	Grimard Catholic Hospital Anyiagba

1674	KG/0048	Duro Soleye Hospital	B+B Ajaokuta
1675	KG/0050	Welfare Clinic & Maternity	GRA Okene
1676	KG/0051	Odoma Clinic & Maternity	Odoma Clinic Idah
1677	KG/0060	DSS Clinic	Lokoja, Kogi State
1678	KG/0061	Federal Polytechnic Medical Centre	Idah - Ajaka Road, Kogi State
1679	KG/0062	Fed. College of Education Medical Centre, Okene	Okene, Kogi State
1680	KG/0064	poly hospital and mat.home	janet ekundayo road, Lokoja
1681	KG/0065	Laronk clinic and maternity	Ajaokuta
1682	KG/0066	Comprehensive Health Center(UITH branch)	Ihima
1683	KG/0071	Oyiba Hospital	5 Enyidudu Street, Woziomi Okene
1684	KG/0072	Oyiza Hospital	Okene Road, Okene
1685	KG/0074	Police Clinic	Police State Command, Lokoja
1686	KG/0080	Federal University Lokoja Medical Centre	Lokoja
1687	KG/0081	Oyiza Hospital	Along Okene Road, Adavi Town, Kogi State.
1688	KG/0082	Good Shepherd Medical Centre	No 10, Egume /Ankpa Road, Anyigba, Kogi State.
1689	KG/0084	Kogi State Specialist Hospital	Along Okene-Kabba Road
1690	KG/0086	National Iron Ore Mining Company Limited Medical Centre	National Iron Ore Mining Company Limited, Itakpe
1691	KG/0089	Acheka Medical Centre	Ojielanyi Ankpa, Kogi State.
1692	KG/0090	Liberty Medical Clinic	Road 12, ASCO Camp, Ajaokuta, Kogi State.
1693	KG/0091	St. Margaret 's Clinic	Behind Dabras Hotel, Ebogogo Rd, G.R.A. Okene, Kogi
1694	KG/0092	Covenant Care Clinic & Maternity	Flat 91G Ondo Estate (SAE), Ajaokuta, Kogi State.
1695	KG/0093	General Hospital Ugwolawo	Ugwolawo, Ofu LGA
1696	KG/0095	Peace Hospital	No 1, Peace Crescent, Off Old Egume Rd, Anyigba, Kogi State.
1697	KG/0096	Zenith Specialist Hospital	Olunle Street, Near Army Signal, Lokoja, Kogi Stat
1698	KG/0097	Victory Memorial Medical Centre	Opposite Federal College of Education, Otite, Kogi State.
1699	KG/0098	Comprehensive Healthcare Centre, Onyedega	Behind Police Station, Onyedega Kogi State.
1700	KG/0099	S & A Hospital	Ajetachi Anyigba, Kogi State
1701	KG/0100	Nagazi clinic & Maternity	Behind Nagazi new market, Nagazi Okene. Kogi state
1702	KG/0102	The Ray of light Hospital	25, ogugu road, okpo, Kogi state
1703	KG/0103	Bethel Hospital	Ochogobi lay out, Ankpa, Kogi state
1704	KG/0104	Hillcrest Clinic And Maternity	Opposite Dunamis church, along Ganaja road, lokoja, Kogi state
1705	KG/0105	Relief Clinic And Maternity	2A lafia street, okene, Kogi state
1706	KG/0106	Rehoboth Specialist Hospital	2nd gate, phase II, lokongoma, lokoja, Kogi state

1707	KG/0107	Neighbour Multicare Hospital And Women Welfare	Omata street, Anyigba, Kogi state
1708	KG/0108	Neighbour Multicare Hospital And Women Welfare	Omata street, Anyigba, Kogi state
1709	KG/0109	Immanuel Clinic	No 12, Ajaka Road, Idah, Kogi State
1710	KG/0111	Concord Hospital	1st 200 unit estate, opposite total filling station, Gadumo, Lokoja, Kogi state
1711	KG/0113	Helping Hands Women Hospital	Behind T-Square Hotel, Zone 8 Roundabout, Lokoja
1712	KN/0015	Sir Mohammed Sanusi Specialist Hosp.	Yan Kaba, Kano
1713	KN/0026	Khadijat Memorial Hospital	No. 20 Katsina Road, Off Hajj Camp Road, Opp Customs Barrack
1714	KN/0028	Mos-Metro Clinic	Sabon Gari, Kano
1715	KN/0029	Unicare Clinic & Maternity	Katsina Road , Kano
1716	KN/0030	Topcare Specialist Medical Centre	No; 118 Hadejia Rd , Kano
1717	KN/0031	Ivory Clinic & Maternity	Aminu Kano, Kano
1718	KN/0032	Premier Clinics	Gyadi Gyadi, Kano
1719	KN/0034	Saymays Specialist Hospital Ltd	Zoo Road, Kano
1720	KN/0035	International Clinic & Hospital Ltd.	40, Niger Street, Airport Road, Kano
1721	KN/0036	Medicus Clinics	No. 2 Beirut Road, Kano
1722	KN/0037	Al-Ammeen Medical Centre	Maiduguri Road, Kano
1723	KN/0038	Nakowa Clinics	No. 9 Airport Road, Kano, Barewa Clinics & Maternity, 11, Za
1724	KN/0041	Warshi Hospital	Dakata, Kano
1725	KN/0042	Zaks Clinic and Maternity Ltd.	Opp. Corner Dankura, Behind PDP HQ. Zoo Road, Kano
1726	KN/0043	Global Clinic	No. 1, 33rd Link, Off New Court road, Gyadi-Gyadi, Kano
1727	KN/0044	Classic Clinic Ltd.	1a Abbass Road, Kano
1728	KN/0046	Ideal Hospital	Sabon Gari, Kano
1729	KN/0070	Barewa Clinic & Maternity	11, Zaria Road, Kano.
1730	KN/0071	DSS Clinic	Kano, Kano State
1731	KN/0072	Nigeria Customs Service Med. Centre	Katsina Rd, Customs Barracks, Kano
1732	KN/0073	Armed Forces Spec. Hospital	2, Ashton Road, Kano
1733	KN/0074	349 Nigerian Airforce Hospital, Kano	NAF Base, Kano
1734	KN/0075	General Hospital, Gwarzo	P.O. BOX 10911 Kano,
1735	KN/0076	Murtala Mohammed Spec. Hospital	Kano City, Kano State
1736	KN/0077	General Hospital, Wudil	Wudil, Kano, Kano State
1737	KN/0078	Mohammed Abdullahi Wase Spec. Hospital	Nasarawa, Kano, Kano State
1738	KN/0079	General Hospital, Danbatta	Danbata, Kano, Kano State
1739	KN/0081	Sheikh Mohammed Jida Gen. Hospital	Sabon Gari, Kano, Kano State
1740	KN/0082	Aminu Kano Teaching Hospital	Gyadi Gyadi, Kano, Kano State
1741	KN/0085	3 Bde MC, Kano	Kano, Kano State
1742	KN/0086	Bayero University Health Centre	Kano, Kano State
1743	KN/0088	5 Bn MRS Janguza	Janguza, Kano, Kano State

1744	KN/0089	Cottage Hospital/Comprehensive Health Centre	Police Academy, Wudil, Kano
1745	KN/0090	Cottage Hospital/Comprehensive Health Centre	Kano State Police Command, Kano State
1746	KN/0091	National Orthopaedic Hospital, Kano	Kano, Kano
1747	KN/0094	Godiya Clinics amd Materinity	Plot 177/177 Na''''ibawa Zaria Road Kano
1748	KN/0095	Standard Special Hospital	8A Lamido Crescent Nassarawa Kano
1749	KN/0096	Cordial Hospital & Maternity	68 Sarkin Yaki Nomansland Kano
1750	KN/0098	Excellence Clinic Maternity Ltd	32 Tarauni Gardens Maiduguri Road Kano
1751	KN/0099	Triumph Clinic And Maternity Limited	106, New Hospital Road Extension Hausawa Kano
1752	KN/0108	Good Pasture Clinic	6, Citta Avenue, Noman''''''''s Land, Kano
1753	KN/0109	Lafiya Surgery	52, Ogoja Street, Sabon Gari, Kano
1754	KN/0110	General Hospital, Tudun Wada	Tudun Wada LGA, Kano State
1755	KN/0111	General Hospital, Bichi	Bichi, Kano State
1756	KN/0112	Federal College of Educ. (Technical) Staff Clinic, Bichi	Bichi, Kano State
1757	KN/0113	Fed. College of Educ. Staff Clinic, Gwale	Gwale, Kano State
1758	KN/0115	Al Noury Specialist Hospital	No 1 Iyaka Road off Zaria Road, Kano
1759	KN/0119	Modula Clinic & Maternity	59, New Road, Sabon Gari, Kano
1760	KN/0120	Nigerian Law School Medical Centre	Tiga Road, Off Zaria - Kano Express Road, Kano
1761	KN/0122	Vintage Clinic	28 Sharada GRA, Kano
1762	KN/0135	Pinnacle Specialist Hospital, Kano	Plot 11-12 Hausawa Quarters, Kano
1763	KN/0137	Good Posture Clinic	6 Citta Avenue, Nomanland, Kano
1764	KN/0144	Sharada Industrial Clinic	60 Ahmed Nuhu Street, Close to Corner Freedom Radio, Kano
1765	KN/0145	General Hospital, Minjibir	Minjibir
1766	KN/0147	Alpha Clinic & Maternity	5B, Sarkin Yaki Street by Airport Road, Opp Oando Filling Station, Kano
1767	KN/0148	City Medical Clinic	104 Aminu Kano Way, Goron Dutse, Kano
1768	KN/0149	Amity Hospital & Medical Services Ltd	10, 13 Link off court Rd, Hausawa, Kano State
1769	KN/0150	Sumaila General Hospital	Kano
1770	KN/0152	Gaya General Hospital	25, Constitution Rd, Kano
1771	KN/0153	Yadakunya Leprosy Hospital	Zone 1, Municipal, P.M.B 3247, kano
1772	KN/0154	Wisdom Hospital	2, Abdullahi Bayero Street, Beside Nasarawa Hosp, Kano State

1773	KN/0155	NOL International Hospital and Diagnosis Centre Limited	1A Sultan Road Nassarawa GRA, Kano
1774	KN/0156	Al Farma Ultra Modern Hospital and Diagnostic Centre	7A Katsina Road Opp. Federal Secretariat, Kano
1775	KN/0157	Mariya Sanusi Maternity Hospital	Kano State
1776	KN/0159	Dawakin Kudu General	Dawakin Kudu, Kano
1777	KN/0160	Tunik Hospital	Nomansland, Kano, Kano State.
1778	KN/0162	Kanya Clinics & Hosp	Panshekara, Kano, Kano State.
1779	KN/0163	Police Children School Clinic, Shanono	
1780	KN/0164	Gezawa General Hospital	Kano State
1781	KN/0165	Infectious Diseases Hospital.	No. 1, Abuja Rd/France Rd Kano
1782	KN/0166	Waziri Shehu Gidado General Hospital	Bachirawa Katsina Road, Kano State
1783	KN/0170	Sumar Specialist Clinics	Airport Road, Behind Kabo Holdings By Kriss Nursery & Primary School, Kano, Kano State.
1784	KN/0171	Greenland Clinics And Maternity Ltd	No. 49a Sarki Yaki Street Nomansland Kano State.
1785	KN/0172	Prime Specialist Hospital	19 Lamodo Crescent Kano, Kano State
1786	KN/0173	Sabo Bakin Zuwo Maternity Hospital	Jakara , Kano State
1787	KN/0176	Mai Akoko Clinic	No. 96 Naibawa Yanlemo, Kano, Kano State
1788	KN/0177	Oasis Clinic Maternity	Ungwu Uku U- Turn Behind Matrix Filling Station, Opposite Zenith Bank, Kano, Kano State.
1789	KN/0178	City Clinic & Maternity	No. 149 Daurawa Quarters, Maiduguri Road, Kano, Kano State.
1790	KN/0180	Karaye Comprehensive Healthcare Centre	Karaye, Kano State.
1791	KN/0181	Northwest University Teaching Hospital	Off Zaria Road Kano
1792	KN/0182	Al-Ihsan Expert Medical and Diagnostic Centre	312,Tudu Fulani, Darmanawa Qtrs. Kano
1793	KN/0183	Best Choice Clinic and Maternity	488, Hospital Road, Sabon Gandu, Kano, Kano State
1794	KT/0001	Bakori Clinic & Maternity	1, Raba Road, Bakori, behind Sharia Court, Bakori, Katsina State
1795	KT/0002	Primary Health Care Center, Bakori	Bakori District
1796	KT/0003	Federal Government Girls College Clinic	F.G.C. Bakorii
1797	KT/0004	Primary Health Center, Dandume	Dandume
1798	KT/0005	General Hospital, Daura	Daura
1799	KT/0006	Federal Govt. College Daura Clinic	F.G.C Daura
1800	KT/0007	Bawo Clinic	Mallamawa Quarters Daura
1801	KT/0009	General Hospital, Dutsinma	Dutsinma
1802	KT/0012	General Hospital Funtua	Funtua
1803	KT/0013	Khadijat Medical Center	No. 53 Katsina Road Funtua
1804	KT/0014	Bistit Hospital	No. 36 Katsina Road
1805	KT/0015	Hajara Med. & Ultrasound Centre	Birnin-Gwari-Lagos Road, Dandume, Katsina

1806	KT/0016	Nakowa Hospital & Ultra Sound Center	Sokoto Road, Funtua
1807	KT/0019	General Hospital Kankara	Health Service Mgt. Board
1808	KT/0020	General Hospital, Kankiya	Kankiya
1809	KT/0021	Federal Medical Center, Katsina	Murtala Mohammed Way
1810	KT/0024	Federal College Of Education Staff Clinic	Katsina
1811	KT/0025	Hassan Usman Katsina Staff Clinic	Katsina
1812	KT/0033	General Hospital Kurfi	Health Service Mgt. Board
1813	KT/0035	Malumfashi Clinic & Maternity	Malumfashi
1814	KT/0036	General Hospital Mani	Mani-Mashi Road Katsina
1815	KT/0038	Primary Health Care Center Mashi	Mashi
1816	KT/0039	Katsina General Hospital	Katsina, Katsina State
1817	KT/0041	DSS Clinic	Katsina, Katsina State
1818	KT/0042	General Hospital, Malufashi	Malufashi, Katsina State
1819	KT/0044	35 Bn MRS, Katsina	Katsina, Katsina State
1820	KT/0046	Ibrahim Coomassie Modern Police Hospital	Katsina State Police Command, Katsina
1821	KT/0047	New Millenium Hospital	110, Ahmadu Bello Way, Katsina
1822	KT/0055	New Funtua Clinic	3 Jabir Roundabout, Zaria Road, Funtua
1823	KT/0061	Jibia Clinic	Tundun Wada Area, Jibia Town
1824	KT/0062	Daura Clinics & Medical Centre	# 29, Zango Road, Daura, Katsina
1825	KT/0064	Abdullahi Diko Inde Customs Clinic	Lema Jibril road, Customs House, Katsina
1826	KT/0065	Amfani Clinic & Mat Home	15, funtua crescent, New Layout Katsina
1827	KT/0066	University Clinic	Federal University, Take- off Campus, federal University Dutsinma, Katsina State
1828	KT/0067	Maternal and Children Hospital	Kafur Road. Malumfashi
1829	KT/0068	K Dara Specialist Clinic Limited	Gidan Dawa
1830	KT/0069	Umaru Musa Yar'adua University Health Centre	Dustinma Road, Katsina
1831	KT/0070	Turai Umar Yar'Adua Maternity Children Hospital	Gidan Dawa off Hassan Usman Katsina Road, Katsina
1832	KT/0072	Albarka Clinic & Maternity	Ali Cinema Malumfashi, (Tinglin Area) Katsina State
1833	KT/0073	General Hospital Musawa	Hospital Rd, Shaishawu Musawa, Katsina
1834	KT/0075	St Vincent Catholic Church	Along Funtua Road, Gidan Dawa, Malumfachi, Katsina State
1835	KT/0076	Alheri Clinic	Lawrence Onoja Road, Kofar Kaura, New Layout katsina, Katsina State
1836	KT/0078	AJS Ultra Modern Clinic And Diagnostic Center	No. 37 Daura Street K- Kaura Layout Katsina, Katsina State
1837	KT/0079	National Obstetric Fistula Center	Babbar Ruga Katsina, Katsina State.
1838	KT/0081	Labmina Maternal And Child.	Kofar Kaura New Lay Out Katsina, Katsina State

1839	KT/0082	Katsina State Orthopaedic & Speciality Hospital	Dutsinma Road, Katsina
1840	KT/0083	General Hospital Jibia	Hayin-gada Jibia LGA
1841	KW/0001	Temitope Hospital	Sabo Line Isale, Off Amilegbe Road, Ilorin
1842	KW/0002	Ola Olu Hospital	Opp. International Tobacco Coy. Garrage Offa Road, Iiorin
1843	KW/0004	Ilorin Clinics & Maternity Hospital	163, Alimi Road, Isale Alore/Abata, Ologe, Ilorin
1844	KW/0005	Eyitayo Hospital	No. 40, Ojo-Iya Road, Ilorin
1845	KW/0006	Oorelope Hospital	No. 30, Opo-Malu Road, Ilorin, Kwara State
1846	KW/0007	Life Care Hospital	No. 55 Gaa-Akanbi Road, Ilorin
1847	KW/0012	Olalomi Hospital - Ilorin	Stadium Road, Ilorin
1848	KW/0013	Cottage Hospital	Ajase Ipo, Ilorin
1849	KW/0014	Abiye Maternity Hospital	Ganmo, Ilorin
1850	KW/0015	Omolola Hospital	Opp. Govt. Sec. School, Along Jebba Road, Ilorin.
1851	KW/0019	Fogofolu Hospital & Mat. Centre	No.12 Shofoluew Street, Sabo-Oke, Ilorin
1852	KW/0020	Sobi Specialist Hospital	Alagbado Okelele, Ilorin
1853	KW/0024	Olarewaju Hospital	Oro Ago Close Ilorin
1854	KW/0025	Wale Clinic & Maternity	Offa.
1855	KW/0027	University Teaching Hospital Ilorin	Ilorin, Lagos Road, Ilorin
1856	KW/0028	Delniks Hospital	Oja Iya Off University Road, Iiorin
1857	KW/0029	Oyin Folorunsho	Tanke Bubu Off University Road, Iiorin
1858	KW/0030	Femis Hospital	Plot 7, Femis Close, off New Yidi Road, Ilorin
1859	KW/0031	Aisat Memorial Hospital	Off. Jaigbade Residence, Itamerin, Ilorin
1860	KW/0032	General Hospital, Omuaran	Omuaran
1861	KW/0033	Jebba Specialist Hospital	Jebba
1862	KW/0035	Airport Clinic	Eyenkorin Road, Ilorin
1863	KW/0036	General Hospital, Pategi	Pategi, Ilorin
1864	KW/0037	Abanise Lolu Hospital	Oke Agodi Aromaradu Adeta, Ilorin
1865	KW/0038	Kiddiz Clinic	Eastern Reservoirs, Olorunshogo, Ilorin
1866	KW/0039	Sadiku Hospital	16, Abdulwahab Folawuyo, Unity Road, Ilorin
1867	KW/0041	Tobi Hospital	Opp. Stadium Shopping Complex, Ilorin
1868	KW/0042	Sabo-Oke Medical Centre	12,Fufu/Okafor Street, Sabo-Oke, Ilorin
1869	KW/0044	Adeyemo Hospital	Omu-aran, Kwara State
1870	KW/0045	Central Clinic	Lafiaji Road, Tsaraji, Kware State
1871	KW/0046	Tolulope Clinic & Maternity	Off Abdullahi Mo''Hd Road, Tanke Iiewe, Ilorin
1872	KW/0048	Anchormed Hospital	University Road, Tanke, Ilorin

1873	KW/0049	Ela Memorial Medical Centre	Near Basin Gate, Ilorin
1874	KW/0050	ECWA Hospital	Omu-Aran
1875	KW/0051	Surulere Medical Centre	Off Taiwo Road, Ilorin
1876	KW/0052	Kosemani Hospital	Emirs Road, Ilorin
1877	KW/0054	General Hospital, Erin-Iie	Erin-Iie
1878	KW/0063	Ola-Olu Hospital	Opposite Int. Tobacco Coy, Garrage Offa Road, Ilorin
1879	KW/0085	DSS Clinic	Ilorin, Kwara State
1880	KW/0086	227 WG Medical Centre	NAF Station, Ilorin, Kwara State
1881	KW/0087	Specialist Hospital, Offa	Offa Town, Kwara State
1882	KW/0089	Ajisafe Hospital	GRA Road, Agana Area, Omuaran, Kwara State
1883	KW/0091	Garin Alimi Hospital	283, Umaru Saro Road, Ilorin
1884	KW/0093	222 LTK BN MRS, Ilorin	Ilorin, Kwara State
1885	KW/0094	University of Ilorin Health Centre	Ilorin, Kwara State
1886	KW/0097	Cottage Hospital/Comprehensive Health Centre	Kwara State Police Command, Ilorin, Kwara State
1887	KW/0100	Amao Megida Hospital	No 16 Sokoto Rd, Sabo-Oke, Ilorin
1888	KW/0101	Sarfam Medical Centre	No 34 Fate Rd, Ilorin
1889	KW/0102	Ore Ofe Clinic & Maternity	Isale-Maliki Street, Balogun Funlani Ilorin
1890	KW/0104	Oluseyi Hospital	Ajase Ipo
1891	KW/0106	Shallom Clinic & Maternity	Bode-Saadu
1892	KW/0107	Olufolarin Okoro Memorial Hospital	No 78, Agbo Oba Rd Ilorin
1893	KW/0109	Rolakim Hospital	Opposite St Clares Grammar School, Offa
1894	KW/0110	Olalomi Hospital - Offa	Irra Rd offa
1895	KW/0111	Royal Care Hospital	No 1 Ponyan Rd NNPC Pipeline Gaa-Akanbi Ilorin
1896	KW/0113	General Hospital Lafiagi	General Hospital Lafiagi
1897	KW/0114	General Hospital Kiama	General Hospital Kiama
1898	KW/0136	Federal Polytechnic Medical Centre, Offa	Offa, Kwara State
1899	KW/0137	NEW ERA HOSPITAL	OSI
1900	KW/0138	CIVIL SERVICE HOSPITAL	ILOFA ROAD, ILORIN
1901	KW/0139	General Hospital, Share	Share
1902	KW/0140	UITH COMPREHENSIVE HEALTH CENTRE	ESIE
1903	KW/0141	ADEWOLE COTTAGE HOSPITAL	ADEWOLE RD, ALONG YEBMOT HOTEL, ILORIN
1904	KW/0144	OLUTAYO CLINIC & MATERNITY	SANGO, ILORIN
1905	KW/0145	IRETI CLINIC & MAT.	24A COCA-COLA RD, OFF UNITY RD, ILORIN
1906	KW/0146	OMOSEBI HOSPITAL	GAA AKAMBI ILORIN
1907	KW/0147	CHILDREN SPECIALIST HOSP.	CENTRE GBORO
1908	KW/0148	AJIKOBI COTTAGE HOSP.	OKEKE, ILORIN
1909	KW/0162	Ibukun Faith Clinic & Maternity	Olorunsogo Danialu Upper Gua Akanbi Road, Ilorin
1910	KW/0163	St Brendan's Catholic Mission	Ahmadu Bello Way, Bacita
1911	KW/0164	Ogo-Oluwa Hospital	Ahmadu Bello Way, Bacita
1912	KW/0168	NNSHS Sick Bay	Offa
1913	KW/0169	Ayoade Hospital	Olorunsogo, Gaa Akanbi, Ilorin

1914	KW/0170	Ona Ara Hospital	7, Agunbiade Street, Sango, Ilorin
1915	KW/0171	Cottage Hospital	Lade
1916	KW/0173	Olupelu Clinic and Maternity	Itaamo via Pakata, Ilorin
1917	KW/0174	Yegun Clinic and Maternity	1, Hospital Road, Pategi
1918	KW/0175	Ifeolu Clinic and Maternity	Gaa Akanbi Roundabout, Ilorin
1919	KW/0176	Crescent Gold Crown Hospital	Behind Mark Petrol Station, Tanke, Ilorin
1920	KW/0177	22 BDE MC, Ilorin	22 Brigade, Sobi Barracks, Ilorin, Kwara state
1921	KW/0178	Oko Medical Centre	Idofin Road, Oko
1922	KW/0179	Balm Hospital	296 Bishop Street, GRA Ilorin
1923	KW/0180	Hauwa Memorial Hospital	Gure Town
1924	KW/0181	Folorunsho Hospital	Oke Odo, Erinle, Kwara State
1925	KW/0182	Olalomi Hospital	Along Ira Road, Offa, Kwara State
1926	KW/0184	Arewa Clinic & Maternity	87 Alimi (Kaiama) Road, Popo Igbona Area, Ilorin, Kwara State
1927	KW/0185	Al Mubeen Clinic and Maternity	5, Aiyetoro Gbede Lane, Off Sodiq Sulyman Road, Beside Deeper Life Church, Fate, Ilorin, Kwara State
1928	KW/0186	Al Barka Hospital	Ajase Ipo Road, Olunlade Area Ilorin, Kwara State
1929	KW/0187	Emmanuel Hospital	Km 3, JEBBA-ILORIN Road, Jebba, Kwara State
1930	KW/0188	An-Nusra Hospital	Along Tasso Road, Yanri, Kwara State
1931	KW/0189	Ilera Layo Medical Centre	Aboto Oja, Abota Oja, Kwara State
1932	KW/0190	Landmark University Medical Centre	Landmark University, Omuaran, Kwara State
1933	KW/0191	Notre Dame Medical Centre	Along Kabba Kajola Road, Amoyo Town, Kwara State
1934	KW/0192	Nakowa Medical Centre	12 Kafanchan Rd, Adjacent God Grace Nursery & Primary School, Kulende Estate Phase II, Ilorin, Kwara State
1935	KW/0193	Rapha Clinic & Maternity	Patigi, Kwara State
1936	KW/0194	Springsway Clinic & Maternity	Opposite Ministry of Social Development, Juvenile Remand Home, Oko-Era, Kwara State
1937	KW/0195	St. Cyprian Catholic Clinic & Maternity	Omolabi Street, Off Iraa Road, Offa, Kwara State
1938	KW/0196	Oyebamiji Central Hospital	20, Kontangora Road, Off Taiwo Road (Beside Ajoke Nur./Pri. School), Ilorin, Kwara State
1939	KW/0197	Tim Hospital	3, Olukade Close, off Abikan Street, Isale-Aluko, Ilorin, Kwara State

1940	KW/0198	Yusjib Industrial Medicare	35, Ajase Ipo Road, (opposite Oando filling station, offa garage), Ilorin, Kwara State
1941	KW/0199	Midland Fertility Center	12 Kano Road, Adewole Estate, Ilorin, Kwara State
1942	KW/0200	Housewife Clinic & Maternity	Majeobaje Street, Lamodi Area, Offa, Kwara State
1943	KW/0201	Khadijat Memorial Hospital	Annex 1, Kulende Housing Estate Junction, Beside, Imam Petroleum, Sango, Ilorin, Kwara State
1944	KW/0202	Omolewa Hospital	Gaa Odota, Ilorin, Kwara State
1945	KW/0203	Onome Clinic and Maternity	1a, Ijakadi Street, off Secretariat Roas, Orita Merin, Offa, Kwara State
1946	KW/0204	Life Fount Hospita	University Road, Beside Overcomer's Church, Tanke, Ilorin Kwara State
1947	KW/0205	E-Sqaure Clinic and Maternity	Olayinka House, Opposite Musco Filling Station, Oke-Ola, Oro, Kwara State
1948	KW/0207	CHL Healthcare Services	Comprehensive Heealthcare, Lamodi Area, Offa, Kwara State
1949	KW/0208	Adualere Tosho Clinic Maternity	No. 3, Adualere Near Goye Church, Ilorin.
1950	KW/0209	Group Medical Centre	Pipeline Road, Tanke Ilorin, Kwara State.
1951	KW/0210	Iyanu- Olu Clinic And Maternity	No. 1 Ilupeju Street, Off Basin Road Ilorin, Kwara State.
1952	KW/0212	Akorede Clinic And Maternity	Opp. Ilorin International Airport Egbejila Junction, Ilorin, Kwara State
1953	KW/0213	Akanji Memorial Hospital	136 Sobi Road Ilorin, Kwara State
1954	KW/0214	General Hospital Ilorin	Abdulazeez Attah Road Ilorin, Kwara State
1955	KW/0215	Dayspring Medical Centre	No. 97, University Road, Tanke Ilorin, Kwara State
1956	KW/0216	First Bisdem Hospital	No. 3 John Oyewole Street, Off Eastern Reservior, Tanke Ilorin, Kwara State
1957	KW/0217	Adeyi Hospital	214 Oba Mama Road Ilorin, Kwara State
1958	KW/0218	Alafia Tayo Hospital	Tinu Street, Akerebiata Ilorin, Kwara State
1959	KW/0219	Mimtaz Hospital	Apalara Area, Behind Police Post Ilorin, Kwara State
1960	LA/0001	Prince & Princess Hospital, Egbeda	Plot 75. 12th Road Gowon Estate Egbeda, Lagos
1961	LA/0002	Talent Specialist Hospital	Plot 440 4th Avenue, Gowon Estate, Egbeda, Lagos

1962	LA/0004	Barbinton Medical Centre, ketu	No. 9 Samuel Anibaba Street Iyana School Bus Stop Ketu Lagos
1963	LA/0006	St.Claver Hospital	Plot 780, Somide Odujirin Avenue, near Skye Bank, Omole Phase II, Berger Area, Lagos
1964	LA/0007	Deji Clinic	No. 56, Agbado Road, Toko Taya Bus Stop, Iju-Ishaga, Lagos
1965	LA/0010	Osuntuyi Medical Center, iju_ishaga	255 Iju Rd, Balogun B/Stop, Iju-Ishaga
1966	LA/0012	General Hospital, Orile-Agege	Orile-Agege
1967	LA/0013	St. Thomas's Hosp. Ifako-Agege	17, Awoni Murphy Street, Itako, Agege, Lagos.
1968	LA/0014	Betta Hospitals Ltd.	9-11 Coker Street, Ipaja Road, Agege, Lagos
1969	LA/0016	All Souls Infirmary Hospital & Mat. Centre	No. 8 Church Street, Elere, Agege, Lagos.
1970	LA/0017	Mucas Hospital	19 Ogun Str., Adeolu Bus Stop, Dopemu, Agege, Lagos.
1971	LA/0018	Masol Hospital Clinic & Mat. Home	8, Kola Akinlade Str., off Akinsegun Rd., Jesu Oseun, Abule Egba, Lagos
1972	LA/0036	Ajeromi General Hospital	Awodi-Ora Ajegunle
1973	LA/0037	Prince & Princess Hospital	191 Ojo Road, Ligoli Bus Stop, Ajegunle, Lagos.
1974	LA/0038	All Soul's Clinics Ltd	No. 235 Ojo Road, Ajegunle, Lagos
1975	LA/0041	Convenant Conception Hospital	No. 9 Illiasu Road Ikotun-Egbe Lagos
1976	LA/0042	Mount Sina'I Hospital - Egbe	105, Isolo Road , Egbe Lagos
1977	LA/0044	Elf Foundation Mother & Child Clinic	Alimosho L.G. Ikotun Lagos
1978	LA/0045	Broad Hospital & Materinty	17 Fehintola Street , Off Isijola Street Ikotun Lagos
1979	LA/0046	Zenith Medical Centre	1. Adeyefa Street. Off Abeokuta Exp. Iyana-Paja
1980	LA/0047	Veta Hospital Ltd.	1, Veta Close, Araromi Bus Stop Abeoukuta Expressway Iyana-Paja Lagos
1981	LA/0057	Shepherd Specialist Hospital	2nd Avenue 23 Road, T Close House 38, Festac , Lagos
1982	LA/0059	First Faith Med. Centre Ltd.	2, Kekere Ekun Str., Orile Iganmu, Lagos.
1983	LA/0060	Grayma Medical Centre	27, Amai Street, Off Alafia Bus Stop, Along Lagos-Badagry expressway(Km 3), Lagos.
1984	LA/0061	Praise Medical Centre, Festac.	3rd Avenue O'' Close House 2, Festac Town, Lagos.
1985	LA/0062	St. Anthony's Medical Centre	19 Badagry Expressway, Festac Town, Lagos
1986	LA/0063	Hova's Place Hospital & Clinic	House 23, T Close, 5th Avenue, Festac Town, Lagos

1987	LA/0065	Palm Beach Foundation Hospital Ltd.	157 Old Ojo Road, Amuwo Odofin, Festac Town, Lagos
1988	LA/0079	La Saplenza Hospital Ltd.	2nd Ave, 21 Road D Close House 15 Festac Town Lagos
1989	LA/0080	St. Jude's Hospital	21 Road, B Close, House 3, Festac Town
1990	LA/0081	Royal Oak Hospital	21 Rd C Close House 24 Festac Town, Lagos
1991	LA/0082	Ituah Hospital Ltd.	512 Road J. Close Festac Town Lagos
1992	LA/0083	God's Power Clinic Hosp. & Mat.	321 Road C. Close House 7. Festac Town Lagos
1993	LA/0085	Emel Hospital	21 Road Z Close,Festac Town
1994	LA/0086	AMC Hospital	House 19, Dubar Road, Mile 2, Lagos
1995	LA/0087	Cedar Group Hospital	208 Road, D Close, FHA, Festac Town, Lagos
1996	LA/0088	Lagoon Hospital, Apapa	8 Marine Road , Apapa Lagos
1997	LA/0090	Dako Medical Centre (Hosp. & Mat. Home)	225b Kirikiri Rd., Trinity Bus Stop, Olodi, Apapa, Lagos.
1998	LA/0093	Abbey Medical Centre	74 Idewu Street, Olodi, Apapa, Lagos
1999	LA/0094	Sonex Clinics Ltd. (Hosp. & Mat.)	5b Emordi Street, Olodi, Apapa, Lagos.
2000	LA/0096	Ostard Hospital & Mat. Home	161, Idewu Street, Olodi, Apapa, Lagos.
2001	LA/0097	Faleti Medical Centre	204, Kirikiri Road, Trinity B/Stop, Olodi, Apapa, Lagos.
2002	LA/0099	Iduna Specialist Hospital	1a, Takorodi Road, Gra, Apapa, Lagos.
2003	LA/0100	Heda Hospital Group	No 2A, Princess Aina jegede close, Ajao Estate, Apapa, Lagos.
2004	LA/0101	Christ Medical Centre	28 Randle Road, Apapa, Lagos
2005	LA/0102	Hilton Hospital	2 Berger Street, Olodi, Apapa, Lagos.
2006	LA/0111	General Hospital, Badagry	Badagry
2007	LA/0112	Grace-Land Medical Center	147, Badagry Expressway
2008	LA/0125	General Hospital, Epe	Epe
2009	LA/0134	Doren Specialist Hospital	1 Kemfat Road , Thomas Village, lekki.
2010	LA/0135	Kings Hospital	39 Mashalashi Street off Keffi Road, Obalende, Ikoyi
2011	LA/0136	Gold Cross Hospital	17B Bourdillon Road, Ikoyi
2012	LA/0138	Cosmoderm Medical Centre	202, Awolowo Road, Folomo, Ikoyi, Lagos.
2013	LA/0143	J-Rapha Hospital	40 Ado Lamgbasa Road, Ajah,
2014	LA/0144	Shalom Hospital Of Int'L Check Up Ltd.	Off Admirally Way, Off Wale Ariyo Street, Lekki, Lagos.
2015	LA/0145	Germaine Health Centre	Km 63 Lagos/Epe Expressway, Lekki, Lagos.

2016	LA/0146	Magbon Alade Medical Centre	1, Okeowo Ave, Magbon Alade Town, Lekki, Lagos
2017	LA/0147	The Peninsular Hospital	Km 23, Lagos Expressway, Ikota, Lekki, Lagos
2018	LA/0148	Havilah Hospital	Havilah Bus Stop, Ajah - Adoh Road Opp. Dredging Site, Ajah
2019	LA/0150	Udeco Medical Centre	Plot 39, 36 Udeco Medical Road off Chevron Drive, Chevy Estate, Lekki
2020	LA/0151	The Royal Infirmary Hospital	Badore Road, Off Ajah - Addo Road
2021	LA/0164	Olatunbosun Convalescent & Mat. Home	2, Olatunbosun Close, Egbatedo Obadeyi B/Stop Ijaye
2022	LA/0165	Uptown Medical Centre	60 Agbado Road, Ijaiye Ojokoro near Bukinsheen Int''l School
2023	LA/0170	The Shepered Medical Center (For Children)	Ikeja-Lagos
2024	LA/0173	Yima Clinic	No. 107 Ogudu Rd, Ojota
2025	LA/0174	Eko Hospital, Ikeja	31, Mobolaji Bank, Anthony Way Ikeja
2026	LA/0175	Allison Memorial Clinc	Plot 330 Adeyemo Akakpo St. Omole
2027	LA/0176	Bodet Medicare & Maternity	6/8 Odewale St. Alausa, Ikeja
2028	LA/0177	Brafus Specialist Hospital	1b Ajayi Road, Ogba
2029	LA/0178	Eziekel Medical Centre	55 Oduduwa Crescent
2030	LA/0179	St. Leo's Catholic Clinic	C/O Catholic Church,10 Toyin Str. Ikeja
2031	LA/0181	Life Support Medical Center	1, Oba Docemo Street G.R.A Ikeja, Lagos.
2032	LA/0182	St. Ives Medical Centre	90 Opebi Rd. Ikeja
2033	LA/0183	Foly Clinic	3 Julius Showunmi Str. Shogunle
2034	LA/0184	Fasanmi Clinic And Maternity	5/7 Olori Str. Shogunle Ikeja
2035	LA/0186	Ikeja Medical Centre	11 Ogun Modede Street Off, Allen Avenue , Ikeja
2036	LA/0187	Holy Trinity Hospital	1108 Awolowo Way Ikeja Lagos
2037	LA/0189	County Hospital Ltd.	41-45 Isheri Road Ogba Ikeja Lagos
2038	LA/0190	Motayo Hospital Limited	3. Owodumi Street, Off Toyin Street Ikeja Lagos
2039	LA/0191	CRI Medi Clinics - Ikeja	Ikeja Opearations Hq Ngb Building 144 Oba Akran Avenue Ikeja
2040	LA/0192	Queens Specialist Hospital Ltd	3B CMD Road, Magodo GRA
2041	LA/0194	Hugo Medical Centre	3, Johnson Street, Off Awolowo Way, Ikeja, Lagos
2042	LA/0196	Triumph Medical Centre	32, Unity Road, Ikeja, Lagos.
2043	LA/0198	June-One Clinic	52, Opebi Road, Ikeja, Lagos
2044	LA/0200	Shoreman Medical Centre	31, Olowu Street, Ikeja, Lagos
2045	LA/0201	Estate Clinic & Holy Trinity Hospital	110b Awolowo Way, Ikeja, Lagos

2046	LA/0203	Ebenezer-Thomas Clinic	34a, Aba Johnson Cresc. Off Adeniyi Jones Adekunle Village,
2047	LA/0237	New-Gate Hospital	59 Ikorodu
2048	LA/0238	General Hospital, Ikorodu	Ikorodu, Lagos
2049	LA/0239	Ikorodu Hospital & Clinic.	9, Muniratu Alojo Street,Off Lagos Road By Afribank,Ikorodu
2050	LA/0240	Idera Hospital Group	Ebute Metta Branch, Off Ojogbe Bus Stop, Ikorodu, Lagos.
2051	LA/0251	Deji Clinic Ltd.	19 Demurin Street, Catop Chicken George Ketu Lagos
2052	LA/0252	Ayo Clinic	No. 8 Alapere Street , Ketu Lagos
2053	LA/0253	Zion Medical Centre	No. 6 Visa Omotunde Street Off Elebiju Road Ketu Lagos
2054	LA/0254	Gloria Hospital	9, Oladeinde Str., Anthony Village
2055	LA/0256	Life Fountain Medical Clinic	45, Oyedele Ogunniyi Str. Anthony Village
2056	LA/0258	Royal Rapha Clinic	4, First Avenue Maryland
2057	LA/0259	Folarin Fountain Hospital & Maternity Home	7, Emmanuel street, Maryland, Jesu Oyingbo complex, behind Mobil/Mr Biggs, Ikeja.
2058	LA/0265	Med-In Specialist Hospital	1, Osugbo Street Ogudu,Kosofe
2059	LA/0266	Mount Sinai Special. Clinic	19a Millitary Str. Onikan Lagos
2060	LA/0267	Avon Medical Services Limited	3rd Floor, West Wing City Hall, Catholic Mission Street, Lagos
2061	LA/0268	Longing Medical Centre	1 Josepha Close, Off Ogundeji Oguntona St, By Ajala Bus Stop, Ijaiye Ojokoro
2062	LA/0269	Good Faith Clinic	19, Ojo-Giwa Street, Off Idumagbo Avenue, Lagos.
2063	LA/0270	Ajike-Sanda Memorial Medical Centre	54, Adeniji Adele Street, Lagos.
2064	LA/0274	St. Stephen's Hospital	17/19 Glover Street, Ebute-Metta
2065	LA/0275	St. Paul's Hospital & Maternity Centre	71a Ondo Street (East), Ebute Metta
2066	LA/0277	Mount Sinai Hospital - Ebute Metta	177, Borno Way, Ebute Metta
2067	LA/0279	Federal Medical Centre - Ebute Metta	PMB 1097, Ebute-Metta
2068	LA/0281	Lagos Mainland Hospital	Abule-Oja, Yaba
2069	LA/0282	St. Stevens Hospital Limited	16, Majekodunmi Street, Maboju-Shogunle
2070	LA/0283	Nigerian Railway Hospital	Ebute Metta,
2071	LA/0285	Mt. Sinai Hospital	177 Borno Way, Ebute Metta
2072	LA/0287	Lobel Hospital	No. 1b Kufeju Street, Alagomeji, Yaba, Lagos
2073	LA/0288	St. Luke's Hospital	18 Commercial Avenue, Sabo, Yaba, Lagos.
2074	LA/0289	Psychiatric Hospital(Community/Staff Clinic)	Yaba, Lagos
2075	LA/0290	Noble Hospital & Maternity Centre	340, Borno Way, Alagomeje, Yaba, Lagos.
2076	LA/0291	Marien Clinic	7, Montgomery Road, Yaba, Lagos

2077	LA/0305	Ebenezer Specialist Hospital	355 Agege Motor Road Challenge B/Stop, Mushin
2078	LA/0306	Ultima Medical Hospital	3. Cappa Avenue Palmgrove Estate Mushin Lagos
2079	LA/0307	Lumico Medical Centre	8 Folario Street Mushin Lagos
2080	LA/0308	Nusamin Clinc & Maternity Hospital	1A & 1B Akintola Close Behind LUTH, Mushin Lagos
2081	LA/0309	St. Claire Hospital	44 Alh. Mush Street Papa Ajao Mushin Lagos
2082	LA/0310	Mount Sinai Hospital - Mushin	32 Olanubi Street, Papa Ajao. Mushin Lagos
2083	LA/0311	Hillstar Clinics	98 Palm Avenue Papa Ajao, Mushin Lagos
2084	LA/0312	AK Oyekan Hospital	2. Bishop St. Moshalashi Idi-Oro. Mushin Lagos
2085	LA/0313	May Clinics Limited	Doxology House, 24/26 Sadiku Street Ilasamaja, Off Isolo Express
2086	LA/0314	Summit Hospital & Maternity Home	5 Summit Close, Off Mosalashi B/Stop
2087	LA/0316	First Shadrach's Hospital & Mat. Home	8, Assoc. Avenue, Illupeju, Lagos.
2088	LA/0318	Model Medical Centre	3, Wilmer Str. Off Town Planning Way, Illupeju, Lagos.
2089	LA/0320	Iyalode Bisoye Tejuoso Hospital	18-20 Ilupeju Bye-Pass Road, Ilupeju, Lagos.
2090	LA/0322	May Hospital Ilupeju	20, Sura Mogaji Street, Off Coker Road, Illupeju, Lagos.
2091	LA/0323	Ufuoma Specialist Hospital & Mat.	23, Obalodu Street, Ilupeju Estate, Lagos.
2092	LA/0325	Tabernacle Clinics	15, Ibadan Street, Ilasamaja, Lagos
2093	LA/0332	Kiladejo Hospital & Specialist Clinics	15ppl Road Off Ppl Bus Stop Badagry Expressway Lagos
2094	LA/0333	Fellowship Clinics	16,Dada Street,Off Ojo Alaba Int''l Mkt. Road,Beside Ojo LG. Secretariat, Ojo, Lagos
2095	LA/0334	Sabo Crown Hospital & Maternity Home	1 Dr Chinwuba Anameje Street, beside Sabo-Oniba Primary Sch. Ojo-Igbede Road
2096	LA/0335	La Saplenza Hospital & Maternity Home.	14,Adeyemi Street, Off Olojo Drive, Ojo
2097	LA/0337	Appleyard Medical Clinic	Off Km 34, Lagos Badagry, Express Way
2098	LA/0338	Ibijola Medical Centre	31 Badagry Exp/Way Lag.
2099	LA/0339	Femola Hospital	106, Olola Ring Road, Ijanikin
2100	LA/0349	Prime Clinic	2. Sunkanmi Awoyungbo Str. Ewu-Tutun Mofoluku Oshodi
2101	LA/0351	Dolu Hospital	7.Sunmola Abayomi Str. Motoluku Lagos

2102	LA/0353	Edmac Medical Centre	21 Samuel Street, Mafoloku Oshodi Lagos
2103	LA/0354	Divine -Grace Medical Centre	3 Sobogun Rofa Street, Mafoloku-Oshodi
2104	LA/0355	Holy Saviour Hosp. Clinic & Mat. Home	45/47 Mofoluku Road Garage Bus Stop, Oshodi
2105	LA/0357	Geo Medical Centre	12 Subairu Street, Ladipo Bus Stop, Sogunle
2106	LA/0358	One Life Hospital Limited	485 Agege Motor Road, Bolade, Oshodi
2107	LA/0359	St. Emmanuel Clinics & Hospital	2,Bola Ademuwon Street,Behind Aswani Market,Isolo
2108	LA/0360	Alpha Peace Medical Centre	Kareem Street,Isolo Way,Isolo
2109	LA/0361	St. Raphael's Hospital Ltd.	9,Ago Okota Palace Way,Okota - Layout, Isolo Okota
2110	LA/0362	Kahn Specialist Hospital	30, Babs Aregbe Street, Off Dr Fasheun Street, Okota
2111	LA/0363	Bose Specialist Hospital	7,Ilori Street, Ire-Akari Estate, Isolo
2112	LA/0365	Mount Pisgah Hospital Ltd.	140 Isolo Road, Egbe, Lagos State
2113	LA/0366	Boyle Medical Centre	1b Oyewole Way(Off Olufemi Peters) Ire Akari Estate,Isolo
2114	LA/0367	Lives Fountain Medicare	28, Godwin Omonua Street, Ire-Akari Estate, Isolo
2115	LA/0368	Bissalam Hospital Complex	4, Modupe Shita Street, Off Liasu Road, Egbe
2116	LA/0370	General Hospital, Isolo	Isolo, Lagos
2117	LA/0371	Optimal Medical Centre,Oshodi	5a Adeyemo Street Off 7/8 B/Stop Mafoloku,
2118	LA/0372	Labson Specialist Hospital & Mat. Home	83 Bangbose Street, Campos Square, Lagos Island
2119	LA/0374	CRI Mediclinic Lekki Vgc	Plot 232 Road 1 Vgc Lekki Epc Expressway Lagos
2120	LA/0376	Adonai Medical Centre	No. 36 Adekunle Kuye Street, Lagos.
2121	LA/0378	Akinbolade Specialist Hospital	66, Badagry Expressway, Ojo, Lagos
2122	LA/0379	Nuklius Specialist Clinic	104, Lewis Street, Obalende, Lagos.
2123	LA/0380	Cyva Clinics Ltd	14, Cyva Crescent, Finger Estate, Satellite Town, Lagos.
2124	LA/0400	Patela Hospital	46 Johnson Street, Yaba, Lagos.
2125	LA/0401	Solbi Continental Hospital Ltd.	3, Fola Agoro Str., Off Abule Ijesha Str., Shomolu
2126	LA/0402	Medol Clinic	54,Adaranyo Street,Famous B/Stop,(Opp Pedro Pri.Sch.)Shomolu
2127	LA/0403	General Hospital Gbagada	Gbagada
2128	LA/0404	Jon-Ken Hospital	61-65 Street, Finbirr Rd, Akoka

2129	LA/0408	Obanle Aje Hospital & Mat. Home	No. 24 Braimoh Street, Obanikoro, Lagos
2130	LA/0416	Jon-dental Clinic	20, Shobande St., Akoka Yaba
2131	LA/0419	Olukemi Hospital & Maternity	3, Silifatu Akinsanya Str. Off Omilani Str. Ijeshatedo
2132	LA/0421	Providence Hospital	Plot 32 Western Avenue Alaka, Stadium, Lagos
2133	LA/0422	Patmos Clinic	346 Herbert Macauly Way Yaba
2134	LA/0426	Providence Hospital	2B Maduike Street, off Raymond Njoku Street, SW Ikoyi, Lagos, Lagos.
2135	LA/0428	Rotfol Medical Centre	2, Gbaja Mark Flats, Surulere, Lagos.
2136	LA/0429	Olaniba Mem. Spec. Hospital	10a Olatunde Onimole Street, Surulere, Lagos
2137	LA/0431	Isioma Hospital	28, Molusi Avenue Ikate, Surulere, Lagos
2138	LA/0432	Zolace Clinic	18 Olukole Street, Off Ogunlana Drive, Surulere, Lagos.
2139	LA/0435	Mercy Thomas Oredugba Medical Centre	8, Mogaji Street, Aguda, Surulere, Lagos
2140	LA/0438	St. Luke's Medical Centre	61 Teniola Street off Adeshina Street, by Airways Link Road, Ijeshatedo, Surulere
2141	LA/0440	A.B. Specialist Hospital Group	87, Randle Avenue, Surulere, Lagos
2142	LA/0441	The Comforter Hospital	20 Alh. Jinadu Street, Surulere, Lagos.
2143	LA/0442	First Cross Hospital	No. 2 Oshopishan Street, Surulere, Lagos.
2144	LA/0443	Toki Medical Centre	No. 3 Sabiu Ajose Crescent, Adeniran Ogunsaya, Surulere, Lag
2145	LA/0444	Smith Medical Centre	3 Eniasoro Beyioku Street, Surulere
2146	LA/0447	Model Specialist Hospital	5 Tafawa Balewa Crescent Off Adeniran Ogunsanya
2147	LA/0448	First Dominican Hospital Ltd	27, Alhaji Masha Road, Surulere, Lagos.
2148	LA/0450	Mezonel Hospital	1 Adetola Street, Aguda-Surulere, Lagos.
2149	LA/0452	Alexander Memorial Hospital	79, Oduduwa Street, Ikate, Surulere, Lagos
2150	LA/0454	Prime Hospital	40, Adelabu Street, Surulere, Lagos.
2151	LA/0455	Robertson Med. Centre	1-5 James Robertson Street, Surulere, Lagos
2152	LA/0456	Uwemedimo Hospital Ltd	19 Market Street, Ijesha - Surulere, Lagos
2153	LA/0457	New day Specialist Clinic Ltd.,	2, Ladele Street, Off Nnobi Street, Ikate, Surulere, Lagos

2154	LA/0458	Shalom Hospital Ltd.	4a, Mosumola Street, Aguda, Surulere, Lagos.
2155	LA/0459	Remilekun Memorial Medical Centre	29A Itire Road, Surulere, Lagos
2156	LA/0460	Topaz Hospital	12, Adeniyi Street, By Municipal Library, Lagos
2157	LA/0492	Living Spring Hospital & Mat. Home	49, Alafia Avenue Off Afolabi Obe Street Ori-Oke B/Stop
2158	LA/0502	BCL Clinic	10, MacDonald Road, Ikoyi, Lagos
2159	LA/0503	SSA Clinic	Lagos
2160	LA/0507	68 Nigerian Army Ref. Hosp.	Myhoung Barracks, Yaba, Lagos
2161	LA/0508	Military Hospital or Creek Hospital	Awolowo Road, Ikoyi, Lagos
2162	LA/0509	81 Division Hospital	Dodan Barracks, Ikoyi, Lagos
2163	LA/0510	445 Nigerian Airforce Hospital	Sam Ewang Air Force Base, Ikeja
2164	LA/0511	Nigerian Airforce Medical Centre - Onikan	12 King George V Road, Onikan, Lagos
2165	LA/0512	403 CMD Medical Centre	NAF Station, Shasha, Lagos
2166	LA/0524	Lagoon Clinic	11A, Idejo Street, V/Island, Lagos
2167	LA/0526	Lagoon Hospital, Ikeja	97/101 Obafemi Awolowo Way, Ikeja.
2168	LA/0528	Finnih Medical Centre	42, Oduduwa Crescent G.R.A, Ikeja.
2169	LA/0529	Kupa Medical Centre Ltd	4, Lateef Salami Street, Off M/M In""tl Airport Rd. Ajao Estate, Lagos.
2170	LA/0533	Delta Crown Hospital & Mat. Home	14, Ajayi Street, Ketu, Lagos.
2171	LA/0535	Osuntuyi Medical Centre, Obanikoro	6, Alh. Salisu Street, Obanikoro, Lagos.
2172	LA/0539	Crystal Specialist Hospital	148, Akowonjo Road, Dopemu, Lagos.
2173	LA/0541	Faith City Hospital, Ajao -estate	16, Asa-Afariogun Street, Off Osolo Way, Ajao Estate, Lagos.
2174	LA/0543	Faith City Hospital, V/I	2B, Oko-Awo Close, V/Island, Lagos
2175	LA/0545	R-Jolad Hospital	1, Akinyede Street, By New Garage, Bariga, Lagos.
2176	LA/0549	Heals Specialist Hospital	40B, Godwin Omonua Street, Ireakari Estate.
2177	LA/0552	Healing Cross Hospital Ltd	34, Apena Street, Okota Isolo, Lagos
2178	LA/0553	Eko Hospital, Surulere	Surulere, Lagos
2179	LA/0554	Krown Hospital & Maternity Centre	11, Alhaji Sekoni Street, Off Alimosho Rd, Iyana-Ipaja, Lagos.
2180	LA/0557	Legus Specialist Hospital	34 Akorohunfayo Street, Ikorodu Rd., Igbobi, Lagos
2181	LA/0560	Fabma Hospital	13, Yaya Abatan Street, Ijaiye Ogba, Ifako, Ijaiye, Lagos
2182	LA/0561	Lagos Univ. Teach. Hosp.	Idiaraba, Lagos
2183	LA/0562	Lagos Island Maternity Hosp.	Broad Street, Lagos
2184	LA/0563	Lagos State University Teach. Hospital	Ikeja, Lagos State
2185	LA/0564	Lagos State General Hospital	Broad Street, Lagos.

2186	LA/0566	Elegbeleye Specialist Hospital	1, Elegbeleye Street, Ikosi-Ketu, Lagos
2187	LA/0569	Alheri Hospital	10, Ojodu Abiodun Road, Off Kosoko Road, Berger B/Stop, Ojodu, Lagos
2188	LA/0578	149 Bn MRS Ojo	Ojo, Lagos State
2189	LA/0579	174 Bn MRS Ikorodu	Ikorodu, Lagos
2190	LA/0580	65 Bn MRS Victoria Island	V/Island, Lagos
2191	LA/0582	242 Recce Bn MRS, Badagry	Badagry, Lagos
2192	LA/0583	56 Signal Command MRS, Mile 2	Mile 2, Lagos
2193	LA/0584	NASFA MRS, Apapa	Apapa, Lagos
2194	LA/0585	NASFA MIR Apapa	Apapa, Lagos State
2195	LA/0586	University of Lagos Health Centre	Yaba, Lagos State
2196	LA/0588	Police Hospital, Falomo	Ikoyi, Lagos
2197	LA/0589	Cottage Hospital/Comprehensive Health Centre	Police College, Ikeja, Lagos State
2198	LA/0590	Cottage Hospital/Comprehensive Health Centre	Police Lagos State Police Command
2199	LA/0591	Nigerian Navy Reference Hospital	Navy Town, Ojo, Lagos
2200	LA/0592	Navy Medical Centre	Dockyard, Victoria Island, Lagos
2201	LA/0593	Obisessan Navy Medical Centre	Mobil Road, Apapa, Lagos
2202	LA/0594	Sick bay NNS BEECROFT	Lagos, Lagos State
2203	LA/0597	Sick bay Gowon Estate	Gowon Estate, Lagos.
2204	LA/0599	Ladi-lak Medical Centre	No 53 IGY Ologbin street, Ladi-lak B/Stop, Bariga, Lagos
2205	LA/0600	Ultimate Charry Spec. Medical Centre	15B Admirality Way, Lekki Phase I, Victoria Island, Lagos
2206	LA/0601	Samaria Hospital	No 17 Dabo Bashorun street off Ali Dada Street, Okota, Lagos
2207	LA/0602	Watershed Hospital	No 3 Dele Orisabiyi street off Ago palace way, Okota, Lagos
2208	LA/0604	St Annes Infirmary (Queens Clinic)	5A Dauda Lane, Off Moshood Abiola Way, Ebute Metta
2209	LA/0606	Vones Medical Centre	9 Barikisu St Onike Yaba
2210	LA/0607	Omol Medical Centre and Maternity Home Limited	No 57 Shyllon street, Palm Grove, Lagos
2211	LA/0608	Springtime Medical Centre	22, Olusesi Str., Opp. Chemron 2nd Gate by the Conservation Centre, Lekki Pennisula
2212	LA/0611	T & S Hospital	169 Agege Motor Road Olosha B/S Mushin
2213	LA/0612	Good Health Medical Centre	No 19 Oludegun street, Ire-Akari estate, Isolo
2214	LA/0613	Labi Hospital & Maternity	13 Oziegbe Street, Ilupeju
2215	LA/0615	Twins Convalescent Health Centre	No 37/39 Karonwe street Abule Egba Ifako
2216	LA/0616	Continental Hospital and Maternity Home	11 Ago-Owu St Off Kayode St Onipanu

2217	LA/0617	Aniyun hospital	No 3 Femi Aderibigbe, close off Diya street Beside Total filling station, Ifako
2218	LA/0618	Safe-way Hospital & Maternity	Km 46 Lagos-Epe Express Road, Sangotedo, Lagos
2219	LA/0620	Moyub Hospital	No 1 Hajia Halima Mohammed ,close off Taiwo street ,Okota
2220	LA/0621	Divine Medical Centre	16, Akanbi Danmole Street, off Ribadu Rd., SW., Ikoyi, Lagos
2221	LA/0622	Barnes Hospital	Plot 34B Oniru Road, Dideolu Estate, Victoria Island
2222	LA/0623	Bola Hospital And Maternity Home	30 Akiti Avenue Off Okota Road
2223	LA/0626	Ago Medical Centre	No 6 Oyinlola close, By Arewa B/stop Ago palace way, Okota
2224	LA/0627	Best Hope Hospital Limited	40 Alaromi St Off Layi Oyekanmi St Mushin
2225	LA/0629	Apex-Care Hospital	42 Kujore St Ojota
2226	LA/0630	Ameso Specialist Hospital	7 Jumat Olukoya Street Off Ogudu Rd Ojota
2227	LA/0631	Lifecare Hospital And Maternity	5 Segun Omolale street, off Balogun b/stop Ago palace way ,Okota
2228	LA/0632	Tmac Specialist Hospital	14, Berkeley Street, off King George Street, Onikan, Lagos Island
2229	LA/0633	New Alpha Medical centre	36, Abusatu Str., Off Lagos Abeokuta, Exp. Way, Araromi, Adeolu B/Staop, Agege, Lagos
2230	LA/0634	Pinecrest Specialist Hospital	83 Makinde street, Mofoluku, Oshodi
2231	LA/0635	Aruna Ogun Memorial Specialist Hospital	2/4 Bamimosu St Ipakodo-Ikorodu
2232	LA/0636	Geo-marie Hospital	35, AdePegbu Street, Abule-Egba, Lagos
2233	LA/0637	Heda Hospital	2A, Princess Aina close, Ajao Estate ,Oshodi
2234	LA/0638	Evangel Hospital	106, Abeokuta Expressway, Awori B/Stop, Abute-Egba, Lagos
2235	LA/0639	Donas Hospital and Maternity	67 Owulade Avenue Irawo Bus/Stop Owode
2236	LA/0640	Balda Hospital & Maternity	No 31, Itsekiri Street, Iju-Ajuwon, Lagos
2237	LA/0643	Ayodele Medical Centre	23/25, Jonathan Coker Rd, Off Fagba Junction, Iju Agege
2238	LA/0644	First Line Hospital	2nd Avenue House 14 By 207 Road Junction, Festac Town
2239	LA/0645	Petros foundation Hospital	20 Seinde Callisto Crescent Charity, Oshodi
2240	LA/0646	Ancilla Catholic Hospital	3, Muyiwa Olojo Street, Iju, Lagos
2241	LA/0647	Jobi Clinic & Maternity Hospital	3/5 Adeyeri Owuyo St Ikorodu

2242	LA/0648	Omni Medical Centre	18, Boyle''s Street, Onikan, Lagos Island, Lagos
2243	LA/0649	Motolani Medical Centre	13, Alfa-Nla Street, Agege, Lagos
2244	LA/0650	Esiri Specialist Hospital	5 Alashe Close Ojogbe Ikorodu
2245	LA/0651	T&S Hospital, Apapa	26 Bombay Crescent Apapa
2246	LA/0652	Christiana Clinic	8 Ogunyemu street, Pedro, Bariga
2247	LA/0653	Estate Model Clinic	Blk 77, Flat 4, Ijaiye Low Cost Estate, Agege, Lagos
2248	LA/0654	Unita Hospital	63, Oduduwa, Street, by College B/Stop, Ikotun, Lagos
2249	LA/0655	Subol Hospital	16/17, Oba Amusa Avenue, Off Ikotun Rd, Idimu-Lagos
2250	LA/0656	Shammah Hospital	5, Epe Street Off Iludun Street Amukoko Lagos
2251	LA/0657	Solik Hospital	1a, Abeokuta Street, Off Okunola B/Stop, Egbeda, Lagos
2252	LA/0659	Bethesda Family Clinic and Maternity	207 Road, A Close House 6. Festac Town Lagos
2253	LA/0661	Hanoba Medical Centre	23, Boyle''s Street, Onikan, Lagos
2254	LA/0662	Ajayi Medical Center	50 Ekulu Street Ikorodu
2255	LA/0663	Shefi Hospital	7-9 Ogunlana Street Off Akowonjo RD Egbena
2256	LA/0666	Setfone Hospital Limited	23 Church Str Abule Taylor B/Stop Off Abeokuta Exp Way
2257	LA/0667	Natafod Consultants Hospital	104 Cemetry Road, Mosafejo, NEPA B/Stop, Amukoko, Lagos
2258	LA/0668	High Rocks Hospital	38 Afariogun street, Oshodi
2259	LA/0670	Adesola Clinic	2 Onabola street Pedro Bariga
2260	LA/0671	Ropheka Hospital Ltd	6 Dosu Ogundele Street Miccom Bus/Stop Akowonjo Lagos
2261	LA/0672	Capes Hospital Ltd	16a Alubarika street, Bariga
2262	LA/0673	Promise Medical Center Ltd	112 Dopemu Rd Ayinla B/Stop Dopemu Lagos
2263	LA/0674	Primex Hospital	58 Igando Rd Unity Bus/Stop Ikotun-Egbe Lagos
2264	LA/0675	Bonne Sante Health Service Ltd.	3rd Avenue, Festac, 321 Road, C Close, House No. 8, Festac Town, Lagos
2265	LA/0677	Okiki Clinic & Maternity	18 Ola Sheu Str Iyana Ipaja by Alimosho Bus/Stop
2266	LA/0679	Mobonike-T Medical Centre	26, Surulere 1 Bus Stop Dopemu Lagos
2267	LA/0680	Majoroh Medical Center	6 Olofin Street Apapa Lagos
2268	LA/0682	Jericho Clinic	30 Ayodele street, Mafoluku ,Lagos
2269	LA/0683	Light Hospital	15 Olumide Onanubi Str Off Folarin Str Alimosho/Off Jimoh B/S Akowonjo
2270	LA/0684	Somito Specialist Hospital	Plot 937, 13th , Road First Avenue Festac Town Lagos

2271	LA/0686	Plato Hospital And Maternity Home	641 Agege Motor Road, Shogunle, Lagos
2272	LA/0687	Hamkad Hospital	39 Olawale Cole Street U-turn B/Stop Abule Egba
2273	LA/0688	G. Estate Clinic	34 Rd A Close Plot 311 Gowon Estate Ipaja Lagos
2274	LA/0689	Beebat Medical Center	2 Jimoh Ojora Street Off Ojo Road Bus Stop , Ajegunle Apapa Lagos
2275	LA/0690	DaySpring Hospital & Maternity	1 Popoola Street Off Isijola Street Ikotun Lagos
2276	LA/0693	Ericon Clinic and Maternity	8 Ladipo Close, Olodi Apapa Lagos
2277	LA/0694	The Ark Hospital and Maternity	8 Ogun Abewenla Str Off Ayobo Rd Oja B/Stop Ipaja
2278	LA/0697	Adenike Fadeyibi Memorial Hospital	20 Akowonjo Rd, Egbeda, Akowonjo Lagos
2279	LA/0699	Golden Cross Infirmary Hospital and Maternity Home Ltd.	22 Road Festac Town Lagos
2280	LA/0700	Goodwill Family Hospital	84 Oluwa Street Ladega Bus Stop Olodi Apapa Lagos
2281	LA/0701	Saseun Hospital And Maternity	172 Kiri-Kiri Road Apapa
2282	LA/0702	ST. James Clinic Hospital And Maternity	69, Wilmer Crescent, Cocnut Bus stop, Olodi
2283	LA/0703	Gateway Clinic & Maternity	211, Muyibi Str, Off Peoples B/Stop, Olodi Apapa, Lagos
2284	LA/0704	Christ Foundation Clinic	68 Alhaja Sinatu Ajayi Road, Morogbo Agbara, Lagos
2285	LA/0705	Elphy Hospital	6, Task Force Rd, By PPL B/S, Okokomaiko, Lagos
2286	LA/0706	Onyems-B Hospital	16, Oba-Dauda Str., Ojoo Alaba, Lagos
2287	LA/0708	Bonne Sante Hospital	12, Ahl Sulaiman Str. Opp Ojoo Military Cantonment, Ojo, Lagos
2288	LA/0709	Tin Can Hospital	3, Kudaisi Str. Off Tin Can Exp. Olodi, Apapa
2289	LA/0710	Makal Medical Centre	42, Alh Balogun Str, Kuje, Anuwo Off Old Ojo Rd, Lagos
2290	LA/0711	Patol Medical Centre	48, Swamp Str. Off Kirikiri Rd, Olodi, Apapa
2291	LA/0713	De-New Aiyetoro Medical Centre	3, Samson Str, Ibasa-Nla Aiyetoro, Near Ijanikin Ojo LGA Badagry Exp. Way, Lagos
2292	LA/0714	The Great Physcian Hospital Ltd	3, Niyi Adebule Str, New Nepa B/S Badagry
2293	LA/0715	Ola-Oki Medical Centre	Opp Iberoko Low cost B/S Ibereko, Badagry
2294	LA/0716	General Hospital Apapa	16, Ibikunle Akintoye Str, Off Randle Rd, Apapa
2295	LA/0718	St. Louis Medical Centre	3, Ado Avenue, Apapa, Lagos
2296	LA/0719	C R I Medi Clinic	42A Issac John Street GRA Ikeja

2297	LA/0720	Unity Hospital ,Ikeja.	138 Obafemi Awolowo Way Ikeja
2298	LA/0721	Adefemi Hospital	49 Seriki Aro, Avenu Off Obafemi Awolowo Way Lagos
2299	LA/0722	Devine Hospital	8 Lawal Street by Oregun Bus Stop, Oregun, Ikeja
2300	LA/0723	The Duke Medical	26 Bamishele Street Off Allen Avenue Ikeja
2301	LA/0724	Isalu Hospital	10 Wempco Road, Off Lateef Jakande Road, Ogba, Ikeja
2302	LA/0725	Omolewa Medical Centre	10B Idowu Akinola Off Olufemi Ojo, Oluwura Isheri, Lagos
2303	LA/0726	RCCG Life Centre Hospital & Maternity	9 Amore Street Off Toyin Street, Ikeja
2304	LA/0727	Idowu Specialist Hospital	10A Olaniji Street, Off Kudirat Abiola Way, Julie Estate, Oregun
2305	LA/0729	White House Clinic	S. Ola Bangbosi Street Off Toyin
2306	LA/0730	The Valley Surgical and Medical Clinics	5 Olatayo Alao Street River valley Estate Ojodu, Lagos
2307	LA/0731	Solid Rock Hospital	Plote 108 Isheri Road Ojodu
2308	LA/0732	Sharon Height Medical Centre	13 Amore Street Off Toyin Street Ikeja
2309	LA/0733	Mother & Child Hospital	39A, Adeniyi Jones Avenue Ikeja
2310	LA/0734	Life Care Clinic & Maternity	12, Alh. Tokumbo Ali Street Off Toyin Street Ikeja
2311	LA/0735	Jibola Hospital & Maternity	22 Comfort Adeniyi Street Mende, Maryland, Lagos
2312	LA/0736	German Friendship	20, Amore Street Off Toyin Street
2313	LA/0737	G & S Medical & Dental Hospital	15 Muyibi Street, Oveira Ogba, Lagos
2314	LA/0738	Dominion Medical Consultant	7 Ade Ojo Street Wemabod Estate, Ikeja, Lagos
2315	LA/0739	Balmgilead Hospital	49, Kayode Street Off Iyaiye Rd. Ogba
2316	LA/0740	Amazing Grace Medical Centre	PF 910, Omole Phase 1 Omotade Crescent Agidibi Rd. IKeja
2317	LA/0741	Utibe Abasi Hospital - Surulere	1 Akinsulire Street Aguda
2318	LA/0742	Standard Medical Centre	18, Agbonyin Street Off Adelebun Street Aguda
2319	LA/0743	St. Charles Hospital	59, Ayilara Street
2320	LA/0745	Parkande Specialist Hospital	13, Rasaq Balogun Street Off Adetola Street Off Adeniran Ogunsanya
2321	LA/0748	Jalupon EState Hospital	63 Bode Thomas Street Lagos
2322	LA/0750	Fountain Hospital Ltd.	B Daramola Lane Off Adesina Street Ijesha-tedo
2323	LA/0752	Primus Hospital	272 Ijesha Rd. Ijesha-tedo
2324	LA/0753	Bimtom Hospital	205 Ijesha Rd. Ijesha-tedo
2325	LA/0754	Life Link Clinic	1B Amosu Street Off Bode Thomas Street By UBA

2326	LA/0791	The Health Arena	13 Strachan Str Off Igbosere Rd Opp Lapal House Lagos
2327	LA/0827	Shasha Medical Centre	15, Oyebola Street, off Ajako B/Stop, Shasha, Alimosho, Lagos
2328	LA/0834	Gold Cross Hospital	18 Iwaya Road, Onikan, Lagos
2329	LA/0858	Jeffis Specialist Hospital	9 Adegbola Street, Alakuku, Lagos
2330	LA/0861	Lex Medical Centre	5 Rahat Alabi Street, Iyana Meiran B/Stop, Ijaiye, Lagos
2331	LA/0919	Gideon Salvation Hospital & Maternity Centre	3 Oyediran Str. Oke-Ira Ogbu
2332	LA/0920	Fannimed Hospital	Block 210, Lily Road, LSDPC Med. Estate, Phase IV, Oba-Oguniyi Rd, Ogba, Lagos
2333	LA/0921	Centre for Management Development, Lagos	Shangisha, Lagos
2334	LA/0922	Utibe Abasi Hospital - Victoria Island	897B Balarabe Musa Crescent Victoria Island Lagos
2335	LA/0930	Treasuregold Hospital	116B, Ago Palace Way, Last Bus Stop, Okota, Isolo,
2336	LA/0932	Fafolu Medical Centre	22, Fafolu Street, Olorunshogo, Lagos
2337	LA/0934	Ascon Clinic	Badagry, Lagos
2338	LA/0954	Good faith Clinic	No. 1 Akindelu Street, off Folawoyo Bankole Street, Ikate, Surulere, Lagos
2339	LA/0955	DSS Clinic	Shangisa, Lagos State
2340	LA/0961	9 Bde MC Ikeja	Ikeja Lagos
2341	LA/0964	Solad Medical Centre	No 8 Maria Ipaja - Lagos
2342	LA/0965	Obitoks Hospital	2 Gideon Adeniran Street oke Odo Lagos
2343	LA/0967	Adeolu hospital	4-6, Oluwole Street, Akoka
2344	LA/0971	adeshina hospital	29. daudu street, off church b/stop, oshodi
2345	LA/0972	AGAPE MEDICAL CENTER	16, OGUNBIYI ILO STREET, OLUDE B/STOP, IPAJA
2346	LA/0973	AL-GUDUS HOSPITAL	Temitope House, 48A Market Street, Abiodun Bus Stop, Shomolu
2347	LA/0974	ANNA MARIA HOSPITAL	19A DA SILVA STR. OFF BISOLA DUROSINMI DRIVE, LEKKI PHASE 1
2348	LA/0975	APEX CARE CLINIC	50/52 BROAD STREET, BOOKSHOP HOUSE
2349	LA/0976	AYOMIDE MEDICAL CLINIC	11, IGBEHIN ADUN STREET, OSHODI
2350	LA/0977	BALM CLINICS	2/3 GODMON ESTATE, OKOTA
2351	LA/0978	BARBINTO CLINICS	25, OJOMO AMAKA AVENUE, OWODE AJEGUNLE, OFF IKORODU ROAD.
2352	LA/0979	BAYO OGUNRO MEMORIAL HOSPITAL	158 KIRIKIRI ROAD OLODI APAPA

2353	LA/0980	BEE HIVE NURSING HOME	64 ADEWOLAN STREET
2354	LA/0981	BIO BATAM HOSPITAL	53 TIJANI STREET IYANA IPAJA OFF LAGOS ABEOKUTA EX/WAY, AGEGE
2355	LA/0982	BRODIE MENDS MEMORIAL HOSPITAL	8, KUGBUYI STREET MUSHIN
2356	LA/0983	BVM HOSPITAL	103, MUYIBI STREET BY WILMER B/STOP
2357	LA/0984	Charis Medical Center	4, OWOLABI BALOGUN STREET OFF FOLAGORO, SHOMOLU
2358	LA/0985	CITIZEN MEDICAL CENTER	86 NORMAN WILLIAMS STREET S/W IKOYI
2359	LA/0988	CROWNRISE HOSPITAL	34, BASHIR SHEKONE ROAD, OFF EGBE OKE AFA, JAKANDE ISOLO
2360	LA/0989	CRUSAINTS HOSPITAL	54, ADESIYAN STREEET, ILUPEJU
2361	LA/0990	Leme De-Hope Clinic	5A KAYODE-ADEBANJI STREET , OKE AFA EJIGBO
2362	LA/0991	DUNIA HOSPITAL	47 OLD OKO OBA RD. AGEGE
2363	LA/0992	F.C.E AKOKA	AKOKA YABA
2364	LA/0993	FIRST CITY HOSPITAL, OBALENDE	11, NOJEEM MAYEGUN RD, OBALENDE
2365	LA/0994	FIRST CITY HOSPITAL, LEKKI	OSAPA LONDON LEKKI PENINSULA
2366	LA/0995	FIRST FAITH MEDICAL CENTRE, SURULERE	5, KARIMU COKER ORILE IGANMU
2367	LA/0996	FOLU CLINIC & MAT.	516, IKORODU ROAD, KETU
2368	LA/0998	GOODTIDINGS HOSPITAL	37/39 FASORO STREET, SURULERE
2369	LA/0999	GRACE SHIELD HOSPITAL & MAT	1, TOBOWOLA STREET,OFF ODEMUYIWA ROAD
2370	LA/1001	HOLINESS HOSPITAL	3, MODUPEOLA STREET,OFF AKEJA ROADBEHIND MUSLIM COLL. ILE IWE B/STOP
2371	LA/1002	HOLY FAMILY CATHOLIC MEDICAL CENTER	22 RD FESTAC TOWN
2372	LA/1003	IKOTUN NURSING HOME AND CONV. CLINIC	24, ABOGUN LOKO STR IJEGUN ROAD, IKOYUN
2373	LA/1004	IMPERIAL MEDICAL CENTER	65 BRICKFIELD ROAD, EBUTE METTA WEST
2374	LA/1005	JACOBSON NURSING HOME	26, OLUFEMI SOKAN STR. PAKO-AGUDA
2375	LA/1006	JIBOLU CLINIC	12, AYILARA STR.
2376	LA/1007	JLT SPECIALIST CLINIC & SKIN CENTER	43A KAKAWA STREET, LAGOS
2377	LA/1008	KRANZ SPECIALIST CLINIC & MAT. HOME	9, UNITY ROAD OFF TOYIN STREET, IKEJA
2378	LA/1009	LIBRA SPECIALIST HOSPITAL	1, ONISEMO STREET,OFF MARSHA SURULERE
2379	LA/1010	LIFE CARE CLINIC AND MATERNITY, OSHODI	44 AROWOJOBE STR. MAFOLUKU
2380	LA/1011	life line children hospital	133 OGUNLANA DRIVE SURULERE
2381	LA/1013	living faith spec. hospital	37 modele str off ojuelegba
2382	LA/1014	longe medical center	126, olusegun osoba road, agbado crossing

2383	LA/1015	lota medical center	1 mojisola abass street off salvation str powerline b/stop, isheri-olofin
2384	LA/1016	magdalene hospital	5, araoti street yaba
2385	LA/1018	Managed Health Care Services	16 Obokun Street off Coker Road, Ilupeju
2386	LA/1019	Manifel Medical Center	192 ijegun road, ikotun
2387	LA/1021	mount sinai hospital,ikeja	21, ajao road,ikeja
2388	LA/1022	mount sinai hospital,surulere	30, falolu street, surulere
2389	LA/1023	National Orthopaedic Hospital	120/124 ikorodu rd.
2390	LA/1024	Fegbemi New Dimension Hospital and Maternity Home	20b brown str. oshodi
2391	LA/1025	new grace land hospital	1 & 2 grace land close off adeyemi apena str.
2392	LA/1026	olu-adeyemi hospital	off nusiratu str, isolo
2393	LA/1027	onaolapo victory clinic	4, amoshe str, meiran ojokoro
2394	LA/1028	optimal medical center,ogba	10, alimi ogunyemi str. off college road by cinema b/stop, ogba
2395	LA/1029	Optimal Specialist Hospital	9 Gbaja Street Surulere
2396	LA/1030	PACIFIC HOSPITAL	314 IJU WATER WORKS , IFAKO
2397	LA/1031	OSBON CLINIC AND MAT.	9, ADENIYI ADEYELE STR, ILE EPO B/STOP, EJIGBO
2398	LA/1033	PRAISE MEDICAL CENTER	12, MARINE ROAD,APAPA
2399	LA/1034	QUINTA HOSPITAL	182, KIRIKIRI ROAD, OLODI APAPA
2400	LA/1035	REGINA MUNDI CATHOLIC HOSPITAL MUSHIN	142-144 AGEGE MOTOR ROAD, MUSHIN
2401	LA/1036	REM YEMS HEALTH CENTER	3 KOLLINTON CLOSE ALAGBADO
2402	LA/1037	RIB WAY POLY CLINIC	24, LUTHER STR. OFF BAMGBOSE
2403	LA/1038	SANOMENS MEDICAL CENTER	8, ISOLO/EJIGBO ROAD, OKE AFA ISOLO
2404	LA/1039	ROYAL SALVATION HOSPITAL	9, MARQUIS STREET, MASALLACHI B/STOP, SHASHA , AKOWONJO
2405	LA/1040	SHALOM HOSPITAL	OYERO STREET,ADURA B/STOP,LAGOS-ABEOKUTA EX/WAY, ALAGBADO
2406	LA/1041	SHALOM OLUWOLE CLINIC	157, IJEBU ROAD,IKOTUN
2407	LA/1042	SILVAN CLINIC	42/44 OKOTA ROAD, ISOLO
2408	LA/1043	SOLIC CLINIC	1A ABEOKUTA STR. OFF IPONLE B/STOP, EGBEDA
2409	LA/1044	SONAEN MEDICAL CENTER	10 GKS(AGBABIAKA) AGO
2410	LA/1045	ST. CHARLES CLINIC	36/60 OSHS DRIVE OFF KIRIKIRI ROAD, OLODI APAPA
2411	LA/1046	ST. CLEMENT HOSPITAL	321 RD. G-CLOSE 3RD AVENUE FESTAC TOWN
2412	LA/1047	ST. LOUIS MEDICAL CENTER	7A/9A ABIBU OKI MARINA
2413	LA/1048	ST. MICHAELS CLINIC	KM 974, LAGOS-ABEOKUTA EX/WAY, ALAKUKO B/STOP, ALAGBADO

2414	LA/1050	ST.KIZITO (PHC)	ILASAN HOUSING ESTATE JAKANDE, LEKKI
2415	LA/1051	ST.MILLA HOPSITAL	19B LADIPO OLUWOLE STR. APAPA G.R.A
2416	LA/1052	STEPHENS HOSPITAL & MAT HOME	138, OLD OJO RD. AGBOJU AMUWO PAKO B/STOP
2417	LA/1053	STEPHENS HOSPITAL	4 OYEWUMI CLOSE OFF FALOLU STR. SURULERE
2418	LA/1054	General Hospital, Surulere	Randle Avenue
2419	LA/1055	TAI OWORU MEMORIAL HOSPITAL	12 EMMANUEL STREET, PALM GROOVE
2420	LA/1056	TOYEK HOSPITAL	479, IKORODU RD. KETU B/STOP
2421	LA/1057	IHE DOCTORS CLINIC	7A EGUNJOBI STREET BY SALAMI B/STOP, SHASHA
2422	LA/1058	TROPICAL MEDICAL CLINIC	BLK 9, PLOT 99, LSPDC LAYOUT
2423	LA/1059	UPLIFT MEDICALCENTER	15, ADMIRALTY WAY, LEKKI PHASE 1
2424	LA/1060	VICTORY CONVALESCENT CENTER	22 ADETORO STREET, IKOTUN
2425	LA/1061	Vineyard Hospital Limited	4 Gbemi Ogundeyi Street, Idimu, Lagos
2426	LA/1062	UNITY HOSPITAL, Surulere	81, OJUELEGBA ROAD, SURULERE
2427	LA/1093	Noble Medicals (African X-ray House)	140, Okota Road,College b/stop,okota
2428	LA/1186	Maritol medical center	67 sam shonibare street, off ogunlana drive
2429	LA/1189	Redeemed Medical Centre	Elias Close, Okokomaiko, Lagos
2430	LA/1196	Tower Hospital	30, Olorun Alaba St.,Akuwonjo Alimosho Lagos
2431	LA/1197	AFRIMED SPECIALIST HOSPITAL	1 Williams Street, off Diya Street, Sawmill, Gbagada Lagos.
2432	LA/1199	Folabi Medical Center	78 Oworonsoki Road, Labule Bus Stop, Lagos
2433	LA/1201	Jim-Sam Hospital & Maternity	22 Gaskiya College Road, Ijora Badia, Apapa, Lagos
2434	LA/1204	Bestcare Hospital	2A Keffi Street, South-West Ikoyi
2435	LA/1205	Skylight Clinic	27 Ibari/Old Otta Road, Moshalashi B/Stop, Alagbado
2436	LA/1207	Calvery Grace Hospital	Lagos Badagry Express Way, Iyana Iba B/Stop, Opp. LASU, Ojo
2437	LA/1208	Ajifat Medical Centre	17 Shoga Street, Mosafejo, Amukoko
2438	LA/1209	City Medicare Clinic	36 Jimoh Ojora Street, Ajegunle
2439	LA/1210	Faith Nursing Services	32 Duro Adewale Street, Off Wazobia B/Stop, Abaranje, Ikotun
2440	LA/1211	Merit Medical Centre	25 Wowo Street Olodi Apapa
2441	LA/1213	Jacob's Memorial Hospital, Specialist Clinic & Matern	82 Old Ewu Road Oshodi
2442	LA/1215	Eprah Medical Clinic	228 Ikotun Road, Alhaja B/Stop, Idimu

2443	LA/1216	Jibol Medical Clinic	119 Tolu Road, Olodi-Apapa
2444	LA/1218	Lade Hospital	17 Olatunji Ige Street Ketu
2445	LA/1219	Landay Clinic	29 Kofo Abayomi Street, Apapa
2446	LA/1224	Jim Sim Hospital & Maternity	22 College Road, Ijora Badia, Ajao Estate
2447	LA/1245	Inland Specialist Hospital	11 Bola Owodunni Street, Behind Eskay Filling Station, Alapere, Ketu
2448	LA/1257	Holy Rosary Medical Centre	321 Road A Close House 18, Festac Town
2449	LA/1259	Duro Soleye Hospital	34 Allen Avenue, Ikeja
2450	LA/1260	Yombo Hospital and Maternity Home	23 Oluyombo Street, Ikosi, Ketu
2451	LA/1261	Fellowship Hospital Ltd	9 Oliyide Street, Off Unity Road Ikeja
2452	LA/1262	Nigerian Army Signals MRS	2 Marine Road, Apapa
2453	LA/1263	NNS Qurra Sick Bay	Apapa Lagos
2454	LA/1264	Armed Forces Resettlement Centre MRS	Lagos
2455	LA/1265	Nigerian Army School of Medical Sciences, Ojo	Ojo, Lagos
2456	LA/1268	NASS Medical Centre	Apapa Lagos
2457	LA/1269	15 FER	Topo Badagry
2458	LA/1270	Santa Maria Hospital	10, Santa Maria Street, Egan Igando
2459	LA/1275	Jaiyeola clinic	4 Bankole Street by Famous Bust Stop, Pedro, Shomolu, Bariga
2460	LA/1276	Tonajib Hospital	46 Maria Ibironke Street, Ikotun, Lagos
2461	LA/1278	Tinu Hospital	86 Mushin Road, Isolo
2462	LA/1283	A. B. 10 Hospital	2 Adeleke Odunuga Close, Harmony Estate, Ifako-Ijaye
2463	LA/1285	Morak Medical Nigeria Ltd	22 Adelakun Street, Off Hostel Bus Stop, Egbe
2464	LA/1286	Yaba College of Technology Medical Centre	Herbert Macaulay Way, Yaba
2465	LA/1288	Omosola Hospital	5 Ige Street Iyana Ipaja Roundabout
2466	LA/1289	St Anthony's of Padua's Medical centre	40 Cardoso Street, Awodiora-Ajegunle
2467	LA/1290	Angel Hospital	20 Ecwa Church Road, Coker Village, Orile Iganmu
2468	LA/1291	Ethiope Clinic Limited	6 Alhaji Kadiri Salami Street, Olodi Apapa
2469	LA/1292	Ancel Hospital	44 Kemberi Road, Okoko-Ojo, Lagos
2470	LA/1293	Ore-Ofe Hospital	KM 28 Badagry Express Way, Ojo
2471	LA/1294	Joedan Hospital	3 Lagos Badagry Express Way Orile Iganmu, Lagos
2472	LA/1295	Ademola Hospital	1 Omowale Street, Cele Bus Stop, Ijanikin-Ojo

2473	LA/1296	Ola Iya Specialist Hospital Ltd	29 akinhanmi street, Off Fashoro Street, Surulere
2474	LA/1297	Funto Hospital	131 Ijesha Road, Itire, Surulere
2475	LA/1298	Ore-Ofe Clinic	2 Aguntashola Street Ijeshatedo
2476	LA/1299	Immanuel (M) Hospital	19 Adebayo Mokuolu Street, Anthony Village, Lagos
2477	LA/1301	Divine Blessing Hospital	1 Aruna Ayuba Close, Akesan, Lagos
2478	LA/1302	San Pedro Specialist Medical Centre	4 Eddy Onugha Drive, Okota
2479	LA/1303	Tolulope Medical Centre	53, Temitope Street. Ilasamaja, Lagos
2480	LA/1304	St Mary's Specialist Hospital	Plot 458 Titilayo Adedoyin Street, Omole Phase 1, Ikeja
2481	LA/1305	Eternal Life Hospital	1 Ashimowu Abeo Street, Sari Iganmu, Lagos
2482	LA/1306	Rock Medical Centre	9 Iju Road, Agege
2483	LA/1307	Kunlade Hospital	7 Opeki Road, Olude Ipaja, Lagos
2484	LA/1308	The Green Pastures Hospital	7 Afolabi Obe Street, Ori Oke, Ejigbo
2485	LA/1310	Fajip Hospital	43 Agbonyin Avenue, Surulere
2486	LA/1311	Blue Cross Hospital	48 Ijaiye Road, Ogba
2487	LA/1313	P & G Medical Centre	29 Sanni Labode Street, New Oko-Oba, Lagos
2488	LA/1315	Tolu Medical Centre	25 Amodu Tijani Street, Tolu Bus Stop, Olodi Apapa
2489	LA/1319	Grand Medical Centre	20 Kayode Street, Onipanu, Lagos
2490	LA/1320	Urban Medical Centre	16 Baiyewu Close, Off Ajayi Road, Ogba
2491	LA/1321	City of Hope Medical Centre	80 Bale Ayetoro street Ajegunle
2492	LA/1322	Adeb Hospital	5 Ogunshipe Crescent, Coker, Orile Iganmu, Lagos
2493	LA/1323	Rikky Hospital	193/197 Ojo Road Ajegunle
2494	LA/1324	Syban Medical Clinic	1 Ogunderu Street, Sari Iganmu, Lagos
2495	LA/1325	Ajangbadi Central Hospital	1 Adeola Street Salami Bus Stop, Ajangbadi, Lagos
2496	LA/1326	Ilogbo Central Hospital	175 Ilogbo Road Ajangbadi
2497	LA/1327	Biomass Nigeria Limited	3 Old Isheri Ijaye Road Aguda
2498	LA/1329	Ajara Medical Centre	1 Ajara Medical Centre Street, Topo, Badagry
2499	LA/1331	Bee-Hive Nursing Home	64 Adetola Street, Surulere
2500	LA/1332	Celian Hospital	231 Kirikiri Road, Olodi Apapa
2501	LA/1333	Dialyzer Medical Centre	60, Arowojobe Street, Oseni Bus Stop, Oshodi
2502	LA/1334	Molayo Medical Centre	20 Akinyele Street, Oko Oba
2503	LA/1335	Nigeria French Language Clinic	Nigeria French Village, Badagry
2504	LA/1337	T J Dennis Specialist Hospital	3/5 Olaitan Street off Nnobi Street, Kilo Junction, Surulere
2505	LA/1338	Tonez Hospital	32 James Robertson Street, Surulere

2506	LA/1339	Bankole Medical Centre	3 Ajigbotinu Street, Agege
2507	LA/1341	Jocon Davids Hospitals	6B, Adebajo Street, Asa Estate, Soluji, Gbagada
2508	LA/1344	Tare's Clinic	11 Shiro Street, Fadeyi
2509	LA/1345	Lydia Nursing Home and Maternity	42 Ogunsola Street, Aguda, Ogba
2510	LA/1347	Good Seed Specialist Hospital	1/3 Asa Afariogun Road, Ajao Estate
2511	LA/1349	Polus Medical Clinic	192 Iju Road, Londo B/Stop, Iju
2512	LA/1350	Mary-Anne Medical Centre	62 Akanro Street, Ilasamaja, Mushin
2513	LA/1354	St Patricks Medical Center	19 Adams Street, Oworonshoki
2514	LA/1355	St Anthony's Hospital	7 Fadiya Street, Ketu, Lagos
2515	LA/1356	Eromon Specialist Hospital	47 Ganiyat Street, Monkey Village, Mazamaza, near Navy gate
2516	LA/1357	Bey Clinic	2 Alhaji Shittu Street, Aguda, Surulere
2517	LA/1359	Royal Specialist Hospital	11 Olubunmi Alonge Street, Pako, Aguda
2518	LA/1360	New World Clinic	5 Bankole Street, Ilasamaja, Isolo
2519	LA/1361	Aishat Specialist Hospital Ltd	26 Fagbenro Street, Idi-Araba
2520	LA/1362	De-Best Maternity & Convalescent Home	29 Ijaoye Street, Jibowu, Yaba
2521	LA/1363	Sentinel Hospital	1 Benson Akinyele Street, Okota
2522	LA/1365	Rally Hospital	13 Fadiya Street Off Demurin, Ketu
2523	LA/1366	Prime Healthgate Medical	9, Capital Road, Agege
2524	LA/1367	Framag Specialist Hospital	7 Regina Omolara Street, off Opebi Road
2525	LA/1368	House of Care Hospital	1 Ajayi Street off Ogundana Street, Allen Avenue, Ikeja
2526	LA/1369	Olive Medicare Hospital & Maternity	6 Gholus Close, Dopemu Roundabout
2527	LA/1370	Royan Hospital	7 Aina Street Ojodu
2528	LA/1371	Mublyn Clinic & Maternity	424 Ikorodu Road, Ojota
2529	LA/1372	Acme Medical Centre	10 Akinwunmi Street Alagomeji
2530	LA/1373	Bernice Clinic & Maternity	No. 5 Fadare Street, Off Adedoyin Street Kosofe, Mile 12, Ketu, Lagos
2531	LA/1374	Rayine Hospital	15 Ashibejoye Street, Apapa
2532	LA/1375	New Capstone Specialist Hospital	2 Ilamoye Street off Adeshina Street, Ijeshatedo
2533	LA/1376	Glorious Mercy Clinic	14 Akintunde Williams
2534	LA/1377	G & T Nursing & Maternity Home	Block 50, Plot 13, 2 Idowu Babafemi Street, Off AIT Station, Alagbado, Lagos
2535	LA/1378	Holyfield Nursing Home	23 Ose-Olorun, off AIT Road, Alagbado
2536	LA/1379	New Merit Hospital Ltd	103 Ibari Road, Alagbado
2537	LA/1380	Jones Specialist Hospital	54 Isolo Road, Egbe

2538	LA/1381	Our Lady Hospital	Double Star Iyana Iheri Bus Stop, Jakande Isolo
2539	LA/1382	Di-Imose Convalescent Home	49 Oshodi Road, Oshodi
2540	LA/1384	New City Clinic & Maternity	19, Agbelekale Street, Oshodi, Lagos.
2541	LA/1385	Peniel Specialist Clinic	11 Ikorodu Road, Jibowu
2542	LA/1386	El-Bethel Hospital	323, Old Ojo Road, Oluti Bus Stop
2543	LA/1387	First City Diagnosis	Plot 3, Jerry Iriabe Street, Lekki Phase 1
2544	LA/1388	Basis Hospital	2 Ogunromi Street Idimu
2545	LA/1389	Rhowil Totalcare Medical Centre	Plot 3b, Block 7, Site G, Satellite Town
2546	LA/1390	Safehands Medical Centre	53 Old Ojo Road, Opposite Festac 1st Gate, Amuwo
2547	LA/1391	DFO Medical Clinic	1 Banji Adewole Lane, Pako, Akoka
2548	LA/1392	De-Bajo Hospital	15 Bajulaye Road Somolu
2549	LA/1393	Nimbus Medical Centre	6 Kola Iyaomolere Street, By Omotayo Banwo Street, Ogudu, Ori-Oke Ojota
2550	LA/1394	God's Convenant Clinic	14 Oworo Road, Pako B/Stop, Oworonsoki
2551	LA/1395	St Danie's Health Centre	14 Oduduwa Street Car Wash B/Stop, Oworosoki
2552	LA/1396	Sikeoye Hope Clinic	64 Karimu Street, Ojuelegba
2553	LA/1397	Adebayo Ogunsanya Memorial Hospital Limited	8 Eniasoro Beyioku Street, Off Ajao Road, Surulere
2554	LA/1398	His Mercy Hospital	17 Adeyemi Street Off Chemist B/Stop, Orile Iganmu
2555	LA/1399	First Graceland Hospital	KM 42, Lekki-Epe Express Way, Abijoh Aja
2556	LA/1400	Rolawe Medical Centre	3 Bajomo Close, Rolawe House, Shella Estate, Iju Ishaga
2557	LA/1401	Molabi Hospital & Maternity Home	21, Rotimi Omotosho Street, Isheri Osun
2558	LA/1402	Maciland Medical Centre	51 Lafunke Street, Papa B/Stop, Ijegun Ikotun
2559	LA/1403	Emis Hospital	33 Banjo Street off Laisu Road, Egbe
2560	LA/1404	Mount Carmel Hospital	6 Mount Carmel Avenue, Owutu, Ikorodu
2561	LA/1405	Mayfair Medical Centre	30/32 Adenle Street, Oke Koto, Agege
2562	LA/1406	First Cross Nursing & Maternity	14 Dairo Street Off Oluwalogbon, Ketu
2563	LA/1407	Rekky Hospital	24 Abolere Street, Pero B/Stop, Ogba Road, Agege
2564	LA/1408	Bedkal Divine Hospital	9-11 Adeshina Avenue, Marcy Hill Estate, Akins B/Stop off Addo Road, Ajah

2565	LA/1409	John Stephens Medical Clinic & Maternity	2 Jimoh Balogun Street, Off CMD Road, Ikosi, Ketu
2566	LA/1410	Inland Specialist Hospital	153, Ogudu Road GRA, Ojota
2567	LA/1411	Adetunji Memorial Hospital	4 Olayemi Street, Mazamaza
2568	LA/1412	Holifield Specialist Hospital	Plot 260, 12 Road, Festac Town
2569	LA/1413	Fineday Hospital	57 Demurin Street, Ketu
2570	LA/1414	Grace Springs Hospital	Oba Adetona, Ilupeju
2571	LA/1415	El-Shaddai Hospital & Laboratory Ltd	KM 4 LASU Road, Iyana School B/Stop, Iba, Ojo
2572	LA/1416	Cossy Clinic	Block 5, Flat 3, Godmon Estate, Okota
2573	LA/1429	Grace Fountain Medical Centre	11 Ileogbo Street, off Agbe Ward, Abule Egba
2574	LA/1430	The Point Specialist Hospital	10 Akerele Road, Surulere, Lagos
2575	LA/1431	Jomark Hospital & Maternity Home	27 Alaba Road, Alaba-Oro
2576	LA/1432	Kenny Medical Centre	190 Kirikiri Road, Osondu B/Stop, Olodi Apapa
2577	LA/1433	B-Rock Convalscent and Maternity	3/7 Olaiya Street off Igando Road, Mosalasi Bus Stop, Ikotun
2578	LA/1434	Aviation Medical Clinics Headquarters (FAAN)	Murtala Mohammed Way, Domestic Airport, Ikeja
2579	LA/1436	Bob Specialist Hospitals	22 Olugbede Street, off Aeor Road, Egbeda Estate
2580	LA/1438	Aqua Libra Clinic and Maternity	1 Ogunsola Street, Iju-Ishaga
2581	LA/1439	Samacs Clinics	24, Shekoni Street, Coker Village , Iganmu
2582	LA/1440	Britannia Hospital	Block 91, Plot 13 Aliu Animashaun Avenue, Lekki
2583	LA/1442	Bolakunmi-Idowu Memorial Medical Center	5 Segun Ishola Street, Owuwu, Ikorodu
2584	LA/1443	Nobility Hospital	30 Godwin Omonwa Street, Ire-Akari Estate, Isolo
2585	LA/1444	DLW Medical Centre	35, Shiaba Street off Ogba Road, Ikeja
2586	LA/1446	Golden Victory Way Hospital Ltd	49b, Shasha, Lagos State
2587	LA/1447	St. Monica's Hospital & Maternity Centre	3, Tolanikawo Akanbi Crescent, Ayobo, Lagos, Lagos state
2588	LA/1448	Ohbee Hospital	1, Kudaki Road, Hostel Bus stop, Egbe, Lagos, Lagos state
2589	LA/1449	Rivet Specialist Hospital	2/3 Cobham Street, Off Olutosin Ajayi Street, Ajao Estate, Lagos State
2590	LA/1450	Hamaab Medical Centre	18, Odutayo street, off Nnobi Street, Masha - kilo, Surulere, Lagos State
2591	LA/1451	Ambassador Medical Clinics	38/44 Mashalashi street off St. Gregory Road, Obalende, Ikoyi, Lagos State

2592	LA/1452	El- Femik Hospital	13B, Alhaji Salami street off Brown road, Aguda, Surulere, Lagos State
2593	LA/1453	Strategic Insight Healthcare, The Hospital Ltd	21, Raji Oladimeji Crescent, Off CMD Road, Opp Lagos State Gov. Secretariat Alausa, Magodo Phase 2, Lagos State
2594	LA/1455	Chinaza Specialist Hospital	2, Hakeem Onitiri Street, Off Ali Dada Str,Off Ago Palace Way, Okota, Lagos State
2595	LA/1456	Rosescan Hospital	
2596	LA/1457	Chironna Medical Centre	21, Adebola Street, (Off Adeniran Ogunsanya Str) Surulere, Lagos State
2597	LA/1458	King Solomon Hospital	4b Bola Crescent, Anthony Village, Somolu ,Lagos State
2598	LA/1462	Able God Hospital and Maternity Home	52, Ijesha Road, Opp Mobil Petrol Station, Adedeji, B/stop, ijeshatedo Lagos State
2599	LA/1463	Crest Hospital	156/160 Isuti Road, Oremeji B/Stop, Egan, Lagos State
2600	LA/1464	Rally Hospital	9, Ibadan Street,Off Illupeju Road, Illupeju, Lagos State
2601	LA/1467	Pharcept Hospital	Plot 74a, Block K, Olajomehin Road, Home of Grace B/Stop, Agbede Meeting, Ikorodu, Lagos State
2602	LA/1468	Progressive Cottage Hospital	334 Adeyemo Akapo Str, Omole Estate, Phase 1, LCD, Ojodu Lagos State
2603	LA/1469	Vintage Hospital	53, Isuti road, Egan Lagos State
2604	LA/1471	Life Anchor Hospital	Anchor Alpha House, 17A Bamisile Street off Bameke Shasha, Lagos State
2605	LA/1472	St. Catherine of Siena Medical Centre	45/49 Old Ewu Road, Mafoluku, Oshodi, Lagos State
2606	LA/1473	Uque Zion Hospital	26, Ile Eja road okerube, Ikotun, Lagos State
2607	LA/1475	Standard Lifecare Clinic	5, Paul Street, Off Sanni Balogun,Off Egba Road, New Oko Oba, Abule Egba Lagos State
2608	LA/1477	We Care Hospital	38, Oguntona Crescent, Ggbagada, Lagos State
2609	LA/1478	Layole & Sons Hospital Ltd	89, Oyemekun Road, Off College road, Ifako/Ijaiye, Lagos State
2610	LA/1481	Almaz clinics LTD	13,Kewulere sreet, near St Hildas Catholic Church Obadore, Alimosho, Lagos State
2611	LA/1482	J. A. Lashman Hospital Ltd	9, Adedosu Street, Off Akilo road, Ogba, Lagos State

2612	LA/1483	Caring Trust Medical Clinic	59, Jubrila Street, Johnson Bus Stop, Ijeshatedo, Lagos State
2613	LA/1486	Samom Clinic	5, Anu Oluwapo Street, Off Adetola Bus stop, Ijaiye, Ojokoro, Lagos State
2614	LA/1487	Kuba Hospital	45, Cemetrt Street, Ebute Metta, Lagos State
2615	LA/1488	Blossom Hospital & Maternity	No 8, Martins Street, Junction Bus stop, Igboelerin, Iba LCDA, Lagos State
2616	LA/1489	Patience Clinic	51, Ariyo St Off Olojo Drive mile 10 B/Stop Ira Qtrs Ojo, Lagos State
2617	LA/1493	Mary The Queen Hospital & Diagnostic Center	29, Babalola Street, Akowonjo, Shasha, Lagos State
2618	LA/1494	Renaissance Medical	12, Adeola Odeku Victoria Island, Lagos State
2619	LA/1496	Neolife clinic	5, Olakunle street, Ajegunle, Ikorodu road, Lagos State
2620	LA/1497	B.A. Clinic	1, Ademola Close, Maya, Ikorodu, Lagos State
2621	LA/1498	Malomo medical center and Maternity home	26/28 Runsewe Estate Avenue, Lagos/Abeokuta express way, Ahmadiyyah area, Lagos State
2622	LA/1499	Deseret International Hospital	23, Oduduwa Crescent, GRA, Ikeja, Lagos State
2623	LA/1500	Deelacs Medical Center	11, Magbon Road, Magbon End, Badagry expressway, Lagos State
2624	LA/1501	Ever- Life Hospital	3, Peter Edoso Street, Ijanikin, Lagos State
2625	LA/1502	Dolu Medical center	5, Sunday saidi street, Okunola, Alimosho, Lagos State
2626	LA/1503	El- Dunamis Medical Centre	6 King Solomon St, Ologuro B/Stop, Akesan Alimosho, Lagos State
2627	LA/1508	Dominion Specialist clinics & laboratory services	18, Doyin Omololu street, Alapere, Ketu, Lagos State
2628	LA/1509	Oak Hospital	191, Lagos road, Ikorodu, Lagos State
2629	LA/1511	Afrique Medical clinic	H17, K close, off 3rd avenue, Gowon estate, Egbeda-lagos, Lagos State
2630	LA/1512	Moye Hospital	8, Gbadamosi Str, Yakoyo, Ojodu, Berger, Lagos State
2631	LA/1513	Dr Abimbola Awoniyi Memorial Hospital	183 Bamgbose street Lagos Island, Lagos State
2632	LA/1514	Eze Udo Medical Centre	2, Olowojeunjeje street, Ajegunle Apapa, Lagos State
2633	LA/1516	Femi Hospital	41 Bale Street Olodi Apapa, Lagos State

2634	LA/1517	Trinity Clinic & Maternity	No 6, Oyedele Str. By Trinity Clinic Bus Stop, Off Mobil Filling Station Off Igbo-Elerin Road, Lagos State
2635	LA/1518	Mount Zion Convalescent Home	11 Sunmonu Str. Off Ogba Rd, Agege, Lagos State
2636	LA/1519	Adeoti Group Nursing Home	106 Ipaja Rd, Sofunde House, Agege, Lagos State
2637	LA/1520	Alalade Memorial Hospital	No 67, Oshodi- Abeokuta Express Road, Dopemu, Ikeja, Lagos State
2638	LA/1521	Adebayo Living Tower Hospital Ltd	8, Adebayo Ojajuni Street, Imeke, Badagry, Lagos State
2639	LA/1522	Araba medical center	122, Ekoro- Agbelekale road, Big Joy B/stop, Abule Egba, Lagos State
2640	LA/1523	Grace of God Hospital LTD	3, Abubakar Street, off Pipeline junction, Ekoro Abule-Egba, Lagos State
2641	LA/1524	St. Victoria hospital	97, Capitol road, Agege by pass,Lagos State
2642	LA/1526	Temitope Nursing Clinic & Mat. Home	1 Yisa street Solanke Bus stop Aboru Iyana Ipaja, Lagos
2643	LA/1527	Blue Sky Hospital	20, Orelope street, Egbeda, Lagos State
2644	LA/1528	Royal Apex Hospital Ltd	230 Ojo Igbede Rd, Ajangbadi-Ilemba House, Lagos State
2645	LA/1530	Sight Centre Ltd	11 Olubi Str, Opp Town Hall Gate, Ita Elewa, Ikorodu, Lagos State
2646	LA/1532	Christ City of Peace Clinic and Maternity Home	4, Yisa Bello Close, Behind Eduland School, Bakery Bus stop, Egbeda-Lagos State
2647	LA/1535	Hopewell Hospital Ltd	3, Badiru Kassim Street, Ebute, Ikorodu, Lagos State
2648	LA/1536	Seriki Abimbola memorial hopital	1, seriki abimbola street, by Old NEPA, badagry, Lagos State
2649	LA/1537	Dental Plus Ltd	No. 2 Deji Odunuga Street Off Adebayo Mokuolu Street, Anthony village, Lagos State
2650	LA/1538	Muskat hospital	9, abbi street off rasaki bus stop, ilemba awori, shibiri road, Lagos State
2651	LA/1539	Lifegate Specialist Hospital	2B, Abiodun Jagun Str., Ogba , Ikeja,Lagos State
2652	LA/1540	Anthony cardinal okogie's medical center	1, akinsegun street, Oko Oba, Agege,Lagos State
2653	LA/1541	Bolutife Nursing Homel	7, Vero Acha street, Jankara, Ijaiye-Ojokoro B/Stop, Lagos State

2654	LA/1546	Salem specialist hospital	23/24 Omotoye estate road, off Ipaja road, Orile Agege, Lagos State
2655	LA/1547	The Family Clinic	25, Ijesha Road, surulere, Lagos State
2656	LA/1549	Cedar Clinics	No 19, Folarin st, off Ladipo st, Mushin, Lagos State
2657	LA/1550	Locket Medicals Ltd	47, Ariyo St, Ira Quarters Ojo Alaba, Lagos State
2658	LA/1554	Onwusikawa children's medical center	20, Ogunbela Avenue, AGO PALACE, Okota, Lagos State
2659	LA/1555	Al- Sadiq Memorial Convalescent Home	21, Ayisat Road, Mile 12, Ketu, Lagos State
2660	LA/1556	Sacred Heart Clinic	26A Pelewura Crescent, Apapa, Lagos State
2661	LA/1557	Mary Ade specialist hospital	2, Tijani Ashogbon street, ilaje b/stop, Bariga, Lagos State
2662	LA/1560	life Fount medical center	103 Mosalasi Road, Egan, Igando, Lagos State
2663	LA/1561	Holy Fill Hospital	137, Isawo road, Owotu, Agric, Ikorodu, Lagos State
2664	LA/1562	Care Giver hospital	20 Torikoh Street opp Coca cola Depot, Torikoh badagry, Lagos State
2665	LA/1563	West Care Specialist Hospital	32, Samuel Street Vulcanizer bus stop Egbeda, Akowonjo, Lagos State
2666	LA/1564	Efan hospital & maternity	7/9 Alh. Yusuf Adebayo street, coconut b/stop, Olodi Apapa, Lagos State
2667	LA/1565	Precious gift nursing services	1b, isolo road, Egbe (Cele b/stop), beside Egbe bridge hotel, Egbe, Lagos State
2668	LA/1566	D. Lawrence Clinic	50, Ikorodu road, Jibowo, Lagos State
2669	LA/1567	Mater ChristI Clinic	8A, Bishop Okogie St, Off Bayo Oyewole St, Balogun Bus stop, Ago Palace Way, Okota Isolo, Lagos State
2670	LA/1568	St. Nicholas Hospital	58 Campbell Street Lagos Island, Lagos State
2671	LA/1569	Royale Medical Centre	No 1 Piriye Ayubele Street Iyana School, Iyana sch B/stop Iba, Lagos State
2672	LA/1570	Olufunmlayo medical center	40/42 Mba street, Ajegunle, Lagos State
2673	LA/1572	Bolutife Hospital	26, Eweje street, Mafoluku, Oshodi, Lagos State

2674	LA/1576	Goodness Medical Centre	151, Shibiri Road,Opp Otto- Awori Local Council Dev. Area Office, Shibiri - Lagos, Lagos State
2675	LA/1577	Krobon clinic	1, Famuyiwa street, Mongoro Agege off Sebiotimo street behind Oando petroleum, Lagos State
2676	LA/1579	THH Healthcare	Plot 1073 4th Avenue, 42 Road Junction, Festac Town, Lagos State
2677	LA/1580	The Humana Hospital	9, Asabi Cole Str Off Lateef Jakande RD Agidingbi, Ikeja, Lagos State
2678	LA/1581	Ahmadiya Muslim Hospital	39, Payne crescent, Apapa, Lagos State
2679	LA/1582	Adunmo memorial hospital	8, Folasade Adetunji street off akibu adeniji street, jankara-ijaye , Ojokoro, Lagos State
2680	LA/1583	Ketu_L_Medical services	26/28, faloye street, ketu, ijaniki, Lagos State
2681	LA/1584	God's goal hospital	118, olojo drive, ojo-alaba int'l makt, Lagos State
2682	LA/1586	Deetab Hospital	21, Bakare Faro Street, Safejo Amukoko, Lagos State
2683	LA/1587	Crystal eye consult	140, Bode Thomas, surulere, Lagos State
2684	LA/1590	Adedeinbo medical center	25, Layori soetan, oremeji round about bus stop, Ifako Bariga, Lagos State
2685	LA/1591	St. Raphael Divine Mercy Specialist Hospital	Boge Town, Ijede Road, Ikorodu, Lagos State
2686	LA/1592	Madek hospital & maternity home	19, association crescent, Abimbola Awoliyi estat, new Oko-Oba, Abule Egba, Lagos State
2687	LA/1593	Rolayo Medical Centre	No 5, Sule Mayo Street, Ikorodu, Lagos State
2688	LA/1594	Ogunmodede memorial hospital & mat. Home	12, asabi oloyede street off abiola adeyemi street off igando road, ikotun, Lagos State
2689	LA/1595	El-Rhema vision Hospital	Plot 129B, D-Close, 3rd avenue, opp. Microfinance bank, Gowon estate, Egbeda, Lagos State
2690	LA/1596	Golden Victory way Hospital LTD	49B, Shasha, Lagos State
2691	LA/1597	ABS MEDICARE CENTER	5, OLA BADMUS STREET OFF MEIRAN COMMAND ROAD, OJA B/STOP, Ajasa via Ipaja, Lagos State
2692	LA/1598	Hallmark clinical and children diagnostic lab	4 Aradula Close Sabo-Oniba, btw peoples and church bus stops, off Alaba int'l mkt, Lagos State

2693	LA/1599	Ife-Oluwa Clinic Limited	12, Turner Eradiri street, off Ojo road bus stop/Jimoh Ojora street, ajegunle, Apapa, Lagos State
2694	LA/1600	De right source specialist hospital	7a abigi Close off Irepodun Street, off Budland str, Akiode, Lagos State
2695	LA/1601	Funbi medical center	plot 10, Kolawole Ogun close off mosalasi b/stop off old Otta road, Alagbado, Lagos State
2696	LA/1602	New gate Hospital Ltd	84,Ogba Isherin Road Opp Millenium School, Ojodu, Lagos State
2697	LA/1603	Tokayo Medical Center	1, Olaleye street, Ikosi ketu, Lagos State
2698	LA/1604	Oladapo medical center	KT Petrol Station Complex, Beside First Bank , Omitoro, Ijede RD, Lagos State
2699	LA/1605	Teslon Pharmacy Nig Ltd	204, lagos Rd, Agric B/S Ikorodu, Lagos State
2700	LA/1608	Iwalewa Conv. Health Centre	20 Erelu Danisa Str. Off Adeshina Str, Ijeshatedo, Lagos State
2701	LA/1610	Dee Tee & Kay maternity Home & Clinic	24, Ondo str. Oke-Ira, Ojodu, Ikeja, Lagos State
2702	LA/1611	Cottage Medicals	45, Maye Street, Off Montgomery Road, Yaba, Lagos State
2703	LA/1613	Magodo Specialist Hospital LIMITED	10, Jaiye Oyedotun street, Magodo GRA, Lagos State
2704	LA/1614	Tofaan's Hospital	15 ,Asipa road, amule, bus stop, ayobo, Lagos State
2705	LA/1617	Sparkview Hospital	20A Wilmer Road, Isheri-Oke Ojodu Beger Lagos State
2706	LA/1618	Limad Hospital	No. 30, Abusi Edumare Street, Ajasa Command, Lagos State
2707	LA/1619	Ibirinade Coker Memorial Hospital	45/47, Karimu Street, off Ojuelegba Road, Surulere, Lagos State
2708	LA/1620	Leye clinic & maternity home	4, salako street, Alpere, ketu, Lagos State
2709	LA/1621	Morayo clinic	No 1 Morayo lane, iworo-ajido road; imeke-badagry, Lagos State
2710	LA/1622	Toluwalope Maternity Centre and Clinic.	1, Toluwalope Close, km 32, Lagos/Badagry Expressway, Lagos State
2711	LA/1623	Hopeland hospital & maternity	
2712	LA/1624	Treasure Hospital	1 Chief Obidegwu st Unity Estate off Iba rd, Iba, Lagos State
2713	LA/1626	Alimosho General Hospital	LASU/Isheri road, Igando, Lagos State

2714	LA/1627	Diamond Hospital	7, Owhin street, off 78, Palm Avenue, Papa Ajao, Mushin, Lagos State
2715	LA/1629	Hero medical center	8, Igunnu street by oja bus stop, orile iganmu lagos, Lagos State
2716	LA/1630	Akinola Medical Centre	5 Progress College Road New Oko Oba Agege, Lagos State
2717	LA/1632	Croabay Medical Centre	115, Ikotun-Idimu Road, Foursquare Busstop, Idimu, Lagos State
2718	LA/1633	Mofeayo Nursing & Maternity Home	2 Abimbola street, Egan, Off Modina Rd, Alimosho L.G.A. Lagos State
2719	LA/1637	Jomark Hospital & Maternity Home	54, community road off ago palace way, Ago Okota, Lagos State
2720	LA/1638	De- Solidrock Maternity and Healthcare Centre	22, Cele road, Oluwatedo Estate by Ile-Iwe Bus stop Isashi, Off Badagry Express way Lagos State
2721	LA/1642	Mac Toye Hospital	205, Ijegun road, ijegun ikotun, Lagos State
2722	LA/1643	Babalola hospital and Maternity	10, osungboye street, Akilo Agege, Lagos State
2723	LA/1645	Cradle medical center	26, Dopemu road, Agege, Lagos State
2724	LA/1647	O & S Hospitals	13 Ahme street, Masha Surulere, Lagos State
2725	LA/1648	Fuja Medical Centre	23 Elegbata St. Apongbon, Lagos Island, Lagos State
2726	LA/1649	Fran Andrews Clinic	No 74, Ijesha Road, Ijeshatedo, Surulere, Lagos State
2727	LA/1653	St. Jacob Nursing Home	15, Kamoru Adeyemi Street, Idimu, Lagos State
2728	LA/1654	p-AFONJA NURSING SERVICES	5, OLU-ODUSISI Street, Ologunye b/stop, along Igando road, ikotun, Lagos State
2729	LA/1655	D-Shallom Healthcare Center	32, Amusa Street, Igando, Lagos State
2730	LA/1656	De-Best maternity & convalescent home	10, Olatunji Oyeleke Avenue, Omitoro Bus stop, Ijede road, Lagos State
2731	LA/1657	St. Joseph Clinic	St. Joseph Catholic Church, Kinkiri Apapa, Lagos State
2732	LA/1658	True Care Model Hospital Ltd	19, AdeyemiApena Street,Papa, Epe. Behind The General Hospital, Epe Lagos State
2733	LA/1659	Odyras hospital	198, association avenue, ikotun, Lagos State
2734	LA/1660	Bidems Victory Hospital	8, Simco Umemmeku Street, Off Isawo, Agric, Ikorod, Lagos State

2735	LA/1661	Nigeria Customs service Medical Centre,	Nigeria Customs Service Medical Centre, Seme Badagri Lagos State
2736	LA/1662	Mojol Hospital Laboratory	20, Church Street, Sasa, Bammake, Lagos State
2737	LA/1663	Josek Medical Centre	3 Ifesowapo st Okeira Ogba Ikeja, Lagos State
2738	LA/1665	Ife-Oluwani Nursing Home	6, Aina street, Ahmadiyya, Ojokoro, ifako Ijaiye, Lagos State
2739	LA/1666	Remsol Convalescent Nursing Home	12 Safejo Close, Amukoko, Apapa, Lagos State
2740	LA/1667	New merit hospitals limited	29.Oladoje street,Gengeto,Okooba, Agege Lagos State
2741	LA/1668	Golden care Hosp.	235, old ojo road, opposite Agboju market, by Lion junction, Lagos State
2742	LA/1670	Prestige medical center	6a, Aina Obembe Street, Oluwaga Ipaja, Lagos State
2743	LA/1672	INTON HOSPITAL	6, ADENEKAN SALAKO STREET, OFF IJAYE ROAD BY CATERPILLAR B/STOP, OGBA, Lagos State
2744	LA/1674	Ewushu Hospital	6, borokini dada close , Ikorodu, Lagos State
2745	LA/1675	First Mainland hospital	66, Sanni Balogun street, New Oko Oba Agege, Lagos State
2746	LA/1677	General Hospital, Mushin	48, oliyide street, Mushin, Lagos State
2747	LA/1678	Amodu adesola memorial hospital	5, AANIBABA STREET, Ikorodu, Lagos State
2748	LA/1679	New Ikeja Hospital and Maternity Home	15, Gbajobi street off Obafemi awolowo way, Ikeja, Lagos State
2749	LA/1681	Rabboni specialist Hospital	7, Eyiowuawi street, Pedro Lagos Lagos State
2750	LA/1682	The Vine Medical Centre	115 New Ipaja Rd, Akin Ogun- Ipaja, Lagos State
2751	LA/1685	New Divine Grace Nursing Home	60 Abamishe Street off Cele Busstop Agodo Egbe, Lagos State
2752	LA/1687	General Hospital Ibeju Lekki	lagos road, Akodo, Lagos State
2753	LA/1688	Swift eye care	9, Amodu Ojikutu str. Off Bishop Oluwole str V/I, Lagos State
2754	LA/1689	His Stripes Specialist Hospital	19/22 Savage St. Orile- Iganmu, Surulere, Lagos State
2755	LA/1690	AS- Salam Convalescent Centre	Km 34, Lekki- Epe Express Way, Lakowe, Ibeju- Lekki, Lagos State
2756	LA/1692	O-Jibol Medical Centre	119,Tolu Road, Olodi, Apapa, Lagos State
2757	LA/1695	Lucy Memorial Hospital	62, Ago Palace Way, By Skye Bank, Okota, Lagos State
2758	LA/1696	Sanctuary Hospitals Ltd	59, Bajulaiye Road, Shomolu, Lagos State

2759	LA/1697	Mayflower Clinics	1, Ibari Str, Ifako, Agege, Lagos, Lagos State
2760	LA/1699	Mediservices Community Hospital	245 Ipaja Road, Ijaja, Lagos State
2761	LA/1704	Adekite Clinic	5, Otubu Est, Ijegun, Benina Satellite Town, Lagos
2762	LA/1705	Emmanuelle Medical Services	22, Idimu Road, Ikotun, Lagos
2763	LA/1706	Regency Specialist Hospital	32, Ajayi Road, Off Yaya Abatan Road, Ogba Lagos State.
2764	LA/1707	New St. Louis Hospital & Maternity Centre	1, Oiriero Street, By Ogooluwa Bus Stop, Ishashi Town, Ojo Lagos.
2765	LA/1715	Rehoboth Pharmaceuticals Limited	No. 12 IsholaAnigbajumo Street, Off Afaraogun Street, Lagos
2766	LA/1718	Ife Oluwa Nursing Home	6, Aina Street, Ahmadiyya, Ojokoro, Ifako Ijaiye Lagos State.
2767	LA/1719	Blooming care Hospital	30. Baale Animashahun Road Alakuko.
2768	LA/1721	Gods Of Kindomlife Specialist Hospital And Maternity Home.	No. 9 Ogundipe Street, Checking Point B/Stop, Off Badagry Expressway, Lagos State.
2769	LA/1723	Eboa Medical Center	48, Yakubu Street, Off Jimoh Balogun Street, Cele Bus Stop, Ikosi Ketu Lagos State
2770	LA/1725	Healing Virtues Hospital	21, Odubakin Street, By Abule- Egun Bus- Stop, Baruwa, Ipaja, Lagos State
2771	LA/1726	Renue Clinic	5th Avenue, R Close, House 1, Festac Town, Lagos State
2772	LA/1728	Life Spring Hospital	No. 20, Adeboye Gbadamosi Street (Formally Misibau Mojeeb Street), Ago, Okota, Lagos State.
2773	LA/1731	Outreach Women And Children Hospital	4th Avenue By 3rd Avenue Junction, Festac Town Lagos State
2774	LA/1732	Life Pillar Clinic And Maternity Home	1, Solomon Abiodun Street, Rainbow Bus Stop, Ijegemo Lagos State.
2775	LA/1734	Adebambo Hospital	38, Muri- Ojora Street, Amukoko, Lagos State
2776	LA/1735	Safejo Medical Centre	14, Safejo Road, Safejo, Amukoko Ajegunle, Lagos State
2777	LA/1740	Omoju Medical Centre & Maternity	14, Olaijbe Street, Berruwa, Ipaje, Lagos State.
2778	LA/1743	Yomi Nursing And Maternity Home	7, Olorunosebi Street, Off Oluwalogbon Street, Ketu Lagos State.
2779	LA/1744	Yomi Nursing And Maternity Home	7, Olorunosebi Street, Off Oluwalogbon Street, Ketu Lagos State.
2780	LA/1745	Lakefront Hospital	3, Lakefront Street, Off Agidi Road, Alapere- Ketu Lagos State

2781	LA/1746	Peniel Noble Maternity	No. 30, Adegboyega, Agiliti Mile 12, Ketu, Lagos State.
2782	LA/1747	Oar Medical Centre	No. 18, Musa Eromon Street, Igoke Estate, Oke- Odo, Lagos State.
2783	LA/1748	New Covenant Hospital & Maternity	No. 1, Adetola Street, By Conoil Filling Station, Aguda Surulere Lagos State
2784	LA/1749	Flokel Specialist Hospital	12, Okota Road, Isolo, Lagos State
2785	LA/1750	De Shiloh Convalescent Home	2, Oyefesobi Street, Ikosi Ketu, Lagos State
2786	LA/1751	De- Immaculate Maternity Home	Chief Owolowo Street, Adamo Ikorodu, Lagos state
2787	LA/1752	Glad Tidings Medical Centre	No. 1, Old 3, Church Street, Off Gbadamosi Opesa Street, Opeki B/Stop, Ipaja, Lagos State
2788	LA/1753	Akanbi Memorial Hospital	27, Yetunde Kuforji Street, I.B.T.C B/Stop Ayobo Lagos State
2789	LA/1754	Vineyard Nursing Home	13, Kareem Street, Shibiri, Off Ajamgbadi, Ojo, Lagos State
2790	LA/1755	Peterhoff Specialist Hospital	4, Ganiyu Osebaby Street, Off Dr. Fasheun Avenue, Ago, Lagos State.
2791	LA/1757	Ovansa Nursing Home (Annex)	2, Sholanle Street, Odo- Eran, Itire, Lagos State
2792	LA/1758	Ovansa Nursing And Maternity Home	24, Oremeji Street, Ijeshatedo, Surulere, Lagos State
2793	LA/1759	Louise Med Hospital Limited	Plot 14, Blk. 95, Omorinre Johnson Street, Lekki Phase 1, Lagos State
2794	LA/1760	Riverside Diagnostic Hospital Limited	Plot 1397e Block 63a, Shalom Road, Off Raji Rasaki Road, Near Apple Junction, Amuwo Odofin, Lagos State
2795	LA/1761	Synergy Medicals	27,Kujore Street, Ojota, Lagos State
2796	LA/1762	Bronila Medical Services Limited	Oshota Bus Stop By Elepe, Ijede Road Ikorodu, Lagos State.
2797	LA/1763	The Excellence Hospital (Omots Medical) Services Ltd.	5, Alogba Street, Off Owode- Ibeshe Road, By Voice Of Nigeria, Ebute, Ikorodu, Lagos State.
2798	LA/1764	New Nigeria Hospital	34, Dillion Street, Behind Kirikiri Medium Prison, Kirikiri Town Lagos State.
2799	LA/1766	Golden Mother Maternity Home	27, College Road, Opp. Ifako Ijaiye General Hospital Lagos State
2800	LA/1769	St. Julius Hospital	No. 17, Adekunbi Street, off Toyin Street, Ikeja, Lagos

2801	LA/1771	Ibijola Medical Centre,	Ibiye Off Km 34, Badagry Express Way, Ibiye Town, Olorunda LCDA Lagos State
2802	LA/1772	Godwin Nursing Home	11, Owokoniran Street, Idimu, Lagos State
2803	LA/1773	Faith Nursing Home	41B, Segun Awowowo Street, Ejigbo, Lagos State
2804	LA/1774	Jon Fem Hospital	52, Iseyin Street, Off Oyewole Street, Palmgroove Kupetu, Palmgroove, Lagos State.
2805	LA/1775	NNPC Clinic	No. 1B, Muri Okunola Street, Lagos State
2806	LA/1776	Ajowa Nursing Home	12, Emmanuel Daodu St, Araromi Bus-Stop, Alimosho,Iyana Ipaja, Lagos State.
2807	LA/1777	Jola Oluwa Clinic Centre	33, Kayode Koyejo Street, Off Liasu Rd. Idimu Lagos State
2808	LA/1780	A Harmony Clinic & Maternity Home	10, Ekundayo Road, Opp. Holy Child Primary School, Badagry, Lagos State
2809	LA/1781	Star Hospital & Maternity	5, Oremeji Street Off Lawanson Road, Itire, Surulere Lagos State
2810	LA/1782	Sherriff Clinic/Hospital	1, Gbadamosi Eletu Street, Osapa London, Eti- Osa West Lagos State
2811	LA/1783	Pike Medical Centre	63B, Okesuna Street, Island, Lagos State
2812	LA/1784	Avon Medical Services LTD	8, Adedamola Ojomo Close, Off Bode Thomas, Surulere, Lagos State
2813	LA/1785	Owutu Clinic And Hospital	2 Ayorinde Kadejo Street, Mart-k Bus Stop, Owutu, Ikorodu Lagos State
2814	LA/1786	Oyinlola Clinic	25, Ajiboye Street, Alapere-Ketu Lagos State
2815	LA/1787	Isuti Medical Centre	No. 16, Steve Igbasan Street, Off Isuti Rd. Egan, Lagos State
2816	LA/1788	Kingswill Specialist Hospital	3, Ayinuola Close/18b Rafiu Babatunde Road, By Apple Junction Amuwo- Odofin Lagos State
2817	LA/1789	Harris –Shibiri Medical Center	65 Ekunpa-Ajewamiwa Road, Whitehouse Busstop, Ojo Lagos State
2818	LA/1790	Emabal Clinic & Maternity Centre	44b Aiyetoro Street, Aguda, Lagos State
2819	LA/1791	Sure Hope Clinic	1, Osibanjo Close, Aso Rock B/Stop, Igbo- Olomu, Off Isawo Road, Agric, Ikorodu, Lagos State

2820	LA/1792	Dele Hospital	15, Ajasa Lamberu Street, Off Obafemi-Awolowo Road, Oke Ota-Ona, Ikorodu, Lagos State
2821	LA/1793	St. Williams Hospital	16, Adeeko Olusanya Street, Federal Site Services Scheme, Abule Odu Saliat B/Stop, Lagos State
2822	LA/1794	Bee Hess Hospital	155, Akowonjo Road, Akowonjo , Lagos State
2823	LA/1796	Lifesource Hospital	Plot 10, Jajo Estate, After Bethel School, Along Mowon-La Road, Ijede LCDA, Ikorodu, Lagos State
2824	LA/1797	Godbless Medical Centre	15, Camp Davies Road, Orisumibare, Ayobo Lagos
2825	LA/1800	Quantum Medicare	No.28, Ajanaku Street, off Salvation Road, off Opebi Road, Ikeja. Lagos State
2826	LA/1801	Theolad Hospital	51, Jagunmolu Street Temlie Bus Stop By Oando Filling Station, Bariga Lagos State
2827	LA/1802	Al-Afiat Convalescent/ Maternity Home	27, Aina Road, Agiliti, Mile 12, Ketu, Lagos State
2828	LA/1803	Mercy Stripes Specialist Hospital	30, Philip Taiwo Street, Coker Estate, Orisunbare, Shasha Lagos Lagos State
2829	LA/1805	Stevans Medical Centre	17,Ori-Okuta Road, off Isawo Road, Agric, Ikorodu, Lagos
2830	LA/1807	Theo Hospital	25, Davies Street, Ketu, Lagos State
2831	LA/1808	Ketu-L-Medical Services (Annex)	Ketu Medical Centre, opp. AP Petrol Station, Abule B/Stop, off Iyana Era B/Stop, off Lagos –Badagary Exp. Way, Ijanikin, Lagos State
2832	LA/1809	Owolola Bethel Health Care Services	2, Semiu Tiamyu Street, Hilltop Estate, Pipeline Aboru, Lagos State
2833	LA/1810	Dandy Medical Centre	No.3,Olayiwola Street, New Oko-Oba, Abule-Egba, Lagos State
2834	LA/1811	Davestar Hospital	No.1, J.K. Adewunmi Osun St. By Aristo Bus Stop, off Ijegun Road, Isheri Osun, Alimosho Lagos State
2835	LA/1812	Pakal Medical & Hospital LTD	28,Community Rd Obadore, off Lasu-Isheri Express Road, Alimosho Lagos State
2836	LA/1813	Macedonia Specialist Hospital	28,Ijaiye Road, Ogba
2837	LA/1816	De-Immaculate Clinic	Ijede Road, Church Bus -Stop, Itamaga, Ikorodu, Lagos State

2838	LA/1818	Boye Convalescent And Maternity Home	No 4, Kasumu Street, Shangisha/Magodo, Lagos, Lagos State
2839	LA/1821	Kola Christwealth Hospital	No 40b, Ire-Akari Estate Road, Isolo, Lagos, Lagos State
2840	LA/1822	Our Lady and St. Francis Catholic Hospital	Along Super Flamingo Street, Toga-Zanmu Village, Lagos/Badagry Expressway, Lagos State
2841	LA/1826	Makog Hospital	3, Agbaje Salami Street, Off Cynergy Hotel, Free Trade Zone Road, Magbon-Alade, Ibeju-Lekki, Lagos State
2842	LA/1828	Mike-Bethel Hospital	2, Odunsi Street, Somolu Odunsi Bus Stop, Bariga, Lagos State
2843	LA/1829	Theolad Hospital	51,Jagunmolu Street, Temple Bus Stop, By Oando Filling Station, Bariga, Lagos State
2844	LA/1830	Luli Maternity Home	Plot 70, Agbolade-Ogunniyi Street, Oworonsoki, Lagos State
2845	LA/1831	Westcare Hospital	31, Ifoshi Road, Opposite Garisalam Central Mosque
2846	LA/1832	Divine Favours Maternity Home	27, Kareem Oyelade Street, Papa Ashafa, Agege, Lagos State
2847	LA/1834	Shanu Medical Centre	8, Dada Apena Close, Behind GT Bank, Ikorodu Road, Ikorodu, Lagos State
2848	LA/1835	Healing Oak Medical And Healthcare Centre	65, Dada Street Off Omomo, off Ibari, off Iju Road, Ifako-Ijaiye, Lagos State
2849	LA/1836	Samas Hospital	24, Aina Obembe Street, Off Oluwaga Bus-Stop, Ipaja-Lagos, Lagos State
2850	LA/1837	IFPF Hospital	374, Ikorodu Road, Maryland, Lagos State
2851	LA/1839	St. Edward Specialist Hospital & Cardiac Centre	4, Dolapo Oshonaike Street Off Ado Road, Ajah, Lekki, Lagos State
2852	LA/1840	Mustard Seed Maternity Home	4, Abayomi Street, Lawanson, Surulere, Lagos, Lagos State
2853	LA/1842	Boot Hospitals	47b James Robertson/ Joseph Shyngle Close, Surulere, Lagos State
2854	LA/1846	David & Daniel Nursing Home	Ologunfe Road, End Of Ologunfe By Ologunfe Bus-Stop, Awoyaya, Ibeju-Lekki, Lagos State
2855	NG/0002	General Hospital, Agaie	Agaie, Minna
2856	NG/0003	Federal Medical Centre, Bida	Bida.
2857	NG/0004	Maraba Hospital - Bida	Minna Road. Bida
2858	NG/0009	Fem-Universal Pharmacy	ABK House, BCC Road, Bida

2859	NG/0012	Taimako Hospital	Se 158 Sabon Gari, Minna
2860	NG/0013	General Hosipital, Minna.	Hospital Road, Minna.
2861	NG/0014	IBB Specialist Hospital	Biko Road, Chanchangi, Minna
2862	NG/0015	Mustapha Hospital & Maternity	No. 1 Lafia Street, "F" Layout, Off Bosso Road, Minna.
2863	NG/0016	Bay Specialist Hospital	Plot 33, Bay Clinic Road, Tunga, Minna
2864	NG/0017	Imani Specialist Hospital	Minna.
2865	NG/0018	The Savannah Medical Consultancy, Bay Clinic Road	David Mark Road, after INEC Office, Opposite FMO Agric
2866	NG/0020	Standard Hospital	Old Airport Quarters, Minna
2867	NG/0021	Unity Clinic	Bida Road, Kpakungu, Minna
2868	NG/0022	Alheri Specialist Hospital	Minna
2869	NG/0023	Kowa Clinic	Bosso, Minna
2870	NG/0024	General Hospital, Mokwa	Mokwa
2871	NG/0025	Care Specialist Hospital	Beside Custom Office, Minna
2872	NG/0047	Basic Health Clini	Doko
2873	NG/0048	Goje Medical Centre	Kontangora
2874	NG/0049	General Hospital, Kontangora	Kontangora
2875	NG/0053	Rural Hospital, Lapai	Lapai
2876	NG/0059	Base Medical Centre, Suleja	Suleiman Barau Road, Opp. Rimeview Hotel, Suleja
2877	NG/0060	Citizen Hospital	Suleja
2878	NG/0061	Fary Group Clinic	105, Suleiman Barau Road, Suleja
2879	NG/0062	General Hospital, Suleja	Suleja
2880	NG/0063	Berith Specialist Hospital	Kwamba Area, Minna Road, Suleja
2881	NG/0064	Maraba Hospital - Suleija	Maganda, Off Suleiman, Barau Road
2882	NG/0065	Suzan Memorial Clinic and Maternity	Uphill Suleja Near Suleja Club,G.R.A.
2883	NG/0066	Delight Hospital	Plot 66, Suleiman Barau Road, Suleja
2884	NG/0081	Rural Health Hospital, Wushishi	Wushishi
2885	NG/0082	Comprehensive Health Care Centre Zungeru, Niger State	Comprehensive Healthcare center, Zungeru
2886	NG/0085	General Hospital, Kutigi	Kutigi
2887	NG/0086	General Hosiptal, Tungan Magajia	Tungan Magajia
2888	NG/0087	Rural Hospital, Nasco Road	Nasco Road
2889	NG/0088	General Hospital, New Bussa	New Bussa
2890	NG/0091	NAF Medical Centre	NAF Station, Minna
2891	NG/0092	99 AWS Medical Centre	NAF Base, Kainji
2892	NG/0093	Top Medical Centre	Plot 4 Paiko Road, Tunga, Minna, Niger State
2893	NG/0094	General Hospital, Kagara	Kagara, Niger State
2894	NG/0098	31 AB MC, Minna	Minna, Niger State
2895	NG/0099	311 AR MRS Kontagora	Kontagora, Niger State
2896	NG/0100	313 AR MRS, Minna	Minna, Niger State
2897	NG/0101	221 LTK Bn MRS Kainji	Kainji, Niger State

2898	NG/0102	Wasiha Medical Centre	Plot 10B Off Niiteco Road, Tunga, Minna
2899	NG/0103	Gilead Medical clinic & Maternity	SW 416 K/Wari Rd, Minna
2900	NG/0104	Bida Clinic	Opposite Central Market, Bida
2901	NG/0105	Great Faith Medical Clinic	Tunga Low Cost, Minna
2902	NG/0106	Masol Hospital & Maternity	Off Our Lady Of Fatima Church, Niteco Road, Tunga, Minna
2903	NG/0107	Liberty Hospital	Opposite 1st Bank Plc, Minna Road, Suleja
2904	NG/0109	Suleja Hospital Ltd	11, Hassan Dallalu Road, Opp. City Gate Chemist, Suleja.
2905	NG/0110	Gaskiya Clinic	Victory Road, Suleja, Niger State
2906	NG/0111	Bisi Clinic & Maternity	SW 16, Benin Street, Minna, Niger State
2907	NG/0126	Royal Care Hospital	Randan Ruwa Bosso Road, Minna
2908	NG/0127	DSS Clinic	Minna, Niger State
2909	NG/0129	Police Clinic, Minna	Minna, Niger State
2910	NG/0130	Federal Polytechnic Bida Medical Centre	Bida, Niger State
2911	NG/0131	Federal College of Education Medical Centre, Kontagora	Kontagora, Niger State
2912	NG/0132	Fed. Polytechnic Medical Centre, Kontagora	Kontagora, Niger State
2913	NG/0134	SOLACE HOSPITAL	HASSAN DALHATU ROAD, SULEJA
2914	NG/0135	SOKOJI HOSPITAL	POLY ROAD, BIDA
2915	NG/0136	RAILWAY INDUSTRIAL CLINIC	RAILWAY COMPOUND MINNA
2916	NG/0137	NASSARA CLINIC AND MAT	131, MAIGARI GODABE ROAD, GRA, KONTAGORA
2917	NG/0139	LUCAS HOSPITAL	BESIDE ASSEMBLY OF GOD CHURCH MADALA
2918	NG/0140	KAINJI MEDICAL CENTER	48 KAINJI ROAD, PO Box 171, New Bussa
2919	NG/0141	Federal College Of Education Med.center	KONTAGORA
2920	NG/0143	faith clinic and maternity	kotaworo area, bida
2921	NG/0144	Al-AZEEZ MEDICAL CENTER	OPP MADENGE STATION, LAGOS-KADUNA ROAD, KONTAGORA
2922	NG/0157	Supreme Hospital	Behind Christ Apostolic Church, Rafin Sanyi, Suleja
2923	NG/0164	Rural Hospital, Kuta	Gwada Road, Kuta
2924	NG/0167	Rural Hospital, Gurara	Hospital Road, Gawu-Babangida
2925	NG/0168	General Hospital	Hospital Road, Kafin-Koro
2926	NG/0169	Rural Hospital, Bida	Minna Road, Bida
2927	NG/0171	Rhema Medical Centre	Near Kontagora General Hospital
2928	NG/0172	Lokoto Health Clinic	Chanchaga
2929	NG/0174	Shakwata Health Clinic	Chanchaga
2930	NG/0175	Maternal & Child Health. Maitumbi	Maitumbi
2931	NG/0176	Maternal & Child Health. Gwari Road	Gwari Road
2932	NG/0177	Maternal & Child Health. Old Airport Road	Chanchaga
2933	NG/0178	Maternal & Child Health. Tudun Wada	Tunga
2934	NG/0179	Passi Health Clinic	Passi
2935	NG/0180	Taiyi (A) Health Clinic	Taiyi

2936	NG/0181	Taiyi (B) Health Clinic	Taiyi
2937	NG/0182	Primary Health Clinic, Dabbo	Dabbo
2938	NG/0183	PPFN Clinic Chanchaga	Chanchaga
2939	NG/0184	PPFN Clinic Barkin Sale	Chanchaga
2940	NG/0186	FSP Medica Outfit, Limawa	Chanchaga
2941	NG/0187	Primary Health Clinic, Dutse Kura Hausa	Chanchaga
2942	NG/0188	Primary Health Clinic, Dutse Kura Gwari	Chanchaga
2943	NG/0189	School Health Clinic, Bosso Road	Chanchaga
2944	NG/0190	Maternal & Child Health Centre, Gulu	Lapai
2945	NG/0191	Health Clinic, Eddo	Lapai
2946	NG/0193	Basic Health Clinic, Gulu	Lapai
2947	NG/0194	Primary Health Clinic, Evuti	Lapai
2948	NG/0195	Health Post, Gbedako	Lapai
2949	NG/0196	Health Clinic, Zago	Lapai
2950	NG/0197	Health Post, Chepa	Lapai
2951	NG/0198	Pelemi Health Post	Pelemi Village
2952	NG/0199	Health Clinic, Gupa	Lapai
2953	NG/0200	Basic Health Clinic, Muye	Lapai
2954	NG/0201	Health Post, Egba	Lapai
2955	NG/0202	Health Post, Yelwa	Lapai
2956	NG/0203	Health Post, Gbage	Lapai
2957	NG/0205	Health Post, Kpada	Lapai
2958	NG/0206	Health Post, Sokun	Lapai
2959	NG/0207	Health Clinic, Ebbo	Lapai
2960	NG/0208	Health Post, Bazhi	Lapai
2961	NG/0209	Baka Health Post, Baka	Lapai
2962	NG/0210	Comprehensive Health Centre, Lapai	Lapai
2963	NG/0211	Basic Health Centre, Agwara	Opposite LGA Secretariat, Agwara
2964	NG/0212	Maternal & Child Health Clinic, Agwara	Agwara
2965	NG/0213	Health Centre, Papiri Gajere	Papiri Gajere Village
2966	NG/0214	Kwana Dispensary	Agwara
2967	NG/0215	Papiri Dispensary	Papiri
2968	NG/0216	Health Post, Kokolo	Kokolo Town
2969	NG/0217	Basic Health Centre Rofia	Rofia Village
2970	NG/0218	Mission Dispensary, Agwara	Agwara
2971	NG/0219	Gallah Dispensary, Gallah	Gallah Town
2972	NG/0220	Kamala Dispensary	Kamala
2973	NG/0221	Zabo Health Clinic, Zabo	Lapai
2974	NG/0222	Model Primary Health Care Centre, Shaku	Lapai
2975	NG/0223	Health Clinic, Saminaka	Lapai
2976	NG/0224	Maternal & Child Health Clinic, Lapai	Lapai
2977	NG/0225	Town Dispensary	Gaba/Kudu
2978	NG/0228	Health Post, Takalafiya	Lapai
2979	NG/0229	Dangana Health Post	Lapai
2980	NG/0230	Zolegi Health Post	Lapai
2981	NG/0231	Etsugi Health Post	Lapai
2982	NG/0232	Zwafu Health Post	Lapai
2983	NG/0233	Gabi Health Post	Lapai
2984	NG/0234	Gbacidan Health Post	Lapai
2985	NG/0235	Shaku Kuku Model Health Clinic	Shaku

2986	NG/0236	Dapugi Health Post	Dapugi
2987	NG/0237	Mayaki Health Clinic	Mayaki Village
2988	NG/0238	Kpashimi Health Clinic	Kpashimi Village
2989	NG/0239	Zabbo Health Post	Zabbo Village
2990	NG/0240	Eddo Health Post	Eddo Village
2991	NG/0241	Evitu Health Post	Evitu Village
2992	NG/0242	Ekkan Health Post	Ekkan Village
2993	NG/0243	Tunga Magaji Health Centre	Agwara
2994	NG/0244	Tunga Kade Health Centre	Agwara
2995	NG/0245	Hikiyan Health Centre	Agwara
2996	NG/0246	Muhatu Health Clinic	Agwara
2997	NG/0247	Adahe Health Centre	Agwara
2998	NG/0248	Mago Health Centre	Agwara
2999	NG/0249	Zuma Barracks MRS	Suleja
3000	NG/0252	Spring Valley Hospital	Behind Sarafina along White Dove School/Redeemed Church, adjacent Supreme School Suleja, Niger State
3001	NG/0253	Federal College of Wildlife Management Medical Centre	Federal College of Wildlife Management, New Bussa, Niger State.
3002	NG/0255	Basic Health Clinic, Agwara	Agwara, Niger State
3003	NG/0256	St. John the Baptist dispensary and PHC Papiri	Papiri, Niger State
3004	NG/0257	New Bethel Hospital & Maternity	Bosso Low Cost Senior Staff Qtrs
3005	NG/0258	31 AB MC Cantonment	Military Cantonment, Minna, Niger State
3006	NG/0259	Hope Clinic & Maternity	Along Gofanti Road, Wawa, Minna, Niger State
3007	NG/0260	Rainbow Clinic & Maternity	IBB University Road, P.O.Box 87 Lapai, Niger State
3008	NG/0261	Kontagora Clinic	Lagos-Kano Road, Kontagora
3009	NG/0263	Shehu Aliyu Usman Shagari General Hospital	Nasko, Niger State
3010	NG/0264	Olive Goodnews Clinic and Maternity (OGCM)	4TH ECWA Church Road, Sauka-Kahuta, Minna, Niger State
3011	NG/0265	Abdulsalam Abubakar General Hospital	Gulu, Niger State
3012	NG/0266	King Salem Hospital & Maternity	No. 3 Fasuwm Street, PDP Qtrs, Behind ABS Guest Inn, Suleja, Niger State
3013	NG/0267	Yetu Hospital	Zungeru Road, Lemu, Niger State
3014	NG/0268	Lafia Medics & Clinics	Alhaji Bokani Shopping Complex BCGA, Kontagora, Niger State
3015	NG/0269	Divine Mercy Hospital and Maternity	Dutsen Kura Gwari-Minna, Niger State
3016	NG/0270	Adalchi Clinics & Maternity	2,Jantaboway, Lapai, Niger State
3017	NG/0271	Umaru Musa Yar Adua Memorial Hospital	Abuja-Kaduna Road, Sabon Wuse, Niger State
3018	NG/0272	Bazza Jasim Clinic	

3019	NG/0273	National Institute for Fresh Water Fisheries Research	New Bussa, Niger State
3020	NG/0274	Sallam Hospital and Maternity	No. 10 Justus Sadiq, Angwan Magaji Road, Suleja, Niger State
3021	NG/0275	Teekay Hospital	5,Musa Kwajafa Street, off Yaro College Road, Suleja, Niger State
3022	NG/0277	Police Secondary School Clinic	Police Secondary School, Shano, Minna, Niger State
3023	NG/0278	Garkiya Children Specialist Clinic, Minna, Niger State	Bosso, Minna Niger State
3024	NG/0279	Zuma Total Care Hospital	Suleja, Niger State
3025	NG/0281	Charles Health Care Limited	No 2, Aliyu Sardauna Street, Between Old NTA Office And Union Bank PLC, Suleja, Niger State.
3026	NG/0282	Holy Ghost Dominion Healing Hospital.	No. 1, Peace Avenue, Paulosa Suleja, Niger State
3027	NG/0283	Berith Specialist Hospital	Plot 16087, Minna Road, Kwamba Layout, Suleja, Niger State.
3028	NG/0284	Faejoc Rapha Clinic LTD	Karfe Town, Old Bwari Road, Behind Zuma Barrack, Suleja Niger State
3029	NG/0287	Optimal Family Medical Center	3, Jubilee Road, Suleja Niger State.
3030	NG/0288	Yetu Specialist Hospital.	Yetu Specialist Hospital.
3031	NW/0001	May Day Specialist Hospital & Maternity	Esu Karu Road, opp Skye Bank Mararaba
3032	NW/0003	Federal Medical Centre, keffi	Keffi
3033	NW/0004	Adonai Hospital	2, Adonai Close Off Old Karu Road, Mararaba
3034	NW/0005	Blessed Trinity Hospital Ltd.	5, Criss Park Street, Mararaba
3035	NW/0006	General Hospital, Keffi	Keffi
3036	NW/0007	Henad Medical Centre	Dogo Road
3037	NW/0008	General Hospital, Uke	Uke
3038	NW/0009	General Hospital, Akwanga	Akwanga
3039	NW/0010	E.R.C.C. Medical Centre	Keffi Road Akwanga
3040	NW/0011	General Hospital, Nasarawa Eggon	Nasarawa Eggon
3041	NW/0012	General Hospital, Keana	Keana
3042	NW/0013	General Hospital, Awe	Awe, Nasarawa State
3043	NW/0014	General Hospital, Obi	Obi, Nasarawa State
3044	NW/0015	General Hospital,Wamba	Wamba, Nasarawa State
3045	NW/0016	Dalhatu Araf (Specialist Hospital)	Nasarawa
3046	NW/0017	Kowa Hospital	Jos Road, Lafia, Nasarawa State
3047	NW/0018	Sauki Hospital	Jos Road, Lafia, Nasarawa State
3048	NW/0047	His Mercy Hospital	Aso Rd., By NEPA Transformer, Mararaba
3049	NW/0048	Karu Hospitals Limited	Behind Building Material Market, Mararaba
3050	NW/0050	Nagari Allah Magani Clinic & Maternity	Behind Chief Magistrate Court, Keffi.

3051	NW/0051	177 Bn MRS Keffi	Keffi
3052	NW/0052	Maraba Guruku Medical Center	Mararaba Int. Mkt Mararaba
3053	NW/0054	Alpha Hospital	36 Makama Dogo Rd Nassara Tooto
3054	NW/0055	Nissi Clinic & Maternity	Area 1 Masaka Near Balami Filling Station, Nasarawa State
3055	NW/0065	St. Anthony's Clinic	13th Elisha Darehovt Street Opp., Midland Plaza,
3056	NW/0066	DSS Clinic	Lafia Nasarawa State
3057	NW/0067	General Hospital, Toto	Toto, Nasarawa State
3058	NW/0068	General Hospital, Garaku	Nasarawa State
3059	NW/0069	General Hospital, Nasarawa	Nasarawa State
3060	NW/0070	Fed. Polytechnic Nasarawa Medical Centre	Nasarawa, Nasarawa State
3061	NW/0071	SHUKURA MEDICAL CENTER	OPP KOFAR HOUSE, HAUSA PRY. SCH. KEFFI
3062	NW/0073	ROYAL HOSPITAL	KEFFI-ABUJA ROAD, BEHIND GWAZA HOTEL, AKWANGA
3063	NW/0074	OUR LADY OF APOSTLES HOSPITAL	OLA HOSPITAL, AKWANGA
3064	NW/0076	MISAN CLINIC AND MAT.	ADO, KARU
3065	NW/0077	M&D HOSPITAL	PLOT 6565 JOS ROAD, LAFIA
3066	NW/0080	DEHI HOSPITAL AND MAT.	49, UNGWA MAKAMA-DOGO ROAD,NASSARAWA
3067	NW/0085	Kings Care Hospital	Kings Care Close Mararaba
3068	NW/0090	New Metro Clinic & Maternity	20 Dr Kash Street, Behind St John Catholic, Mararaba
3069	NW/0092	Fountainhead Medical Centre	22 Jos Road, NIPDC Estate, Off Abacha Road Mararaba
3070	NW/0093	Nasarawa State University Health Centre	Keffi
3071	NW/0095	Police Clinic	Nasarawa
3072	NW/0097	Nasarawa Medical Centre	Along Toto-Abaji Road Nasarawa
3073	NW/0098	Livia Shammah Hospitals Limited	Opp. MRS Filling Station, beside Zimara Int. School, Abuja-Keffi Exp, Road Mararaba.
3074	NW/0102	Total Care Hospital	35, Abacha Rd, Mararaba, Nasarawa State
3075	NW/0104	Pijag Clinic & Maternity	No 17, Base 2, Aku Village, Mararaba, Nasarawa State.
3076	NW/0105	Vicar Clinic	Near Deeper Life, Kabayi Mararaba, Nasarawa State
3077	NW/0106	Kyari Lafiya Clinic & Maternity Ltd	No 10, Philip Dada Street, One Man Village, Nasarawa State.
3078	NW/0107	General Hospital Doma	Agbashi Road, Doma, Nasarawa State.
3079	NW/0109	Alkawari Hospital &Maternity Ltd	Km 18 Keffi Express Way, Nyanya Gwandara, Nasarawa State.
3080	NW/0112	Graceland Specialist Hospital	Kuchikau 1, Harafoam Street, Kuchikau, Nasarawa State.
3081	NW/0113	Masaka Central Hospital	100 royal college road, immediately after royal college, Masaka, Nasarawa State.

3082	NW/0114	Ettal Clinic &Maternity	Behind Alafam Filling Station by Primary Healthcare, Masaka, Nasarawa State.
3083	NW/0115	Tafa Clinic & Maternity	No 46, Old Karu Road, Mararaba, Nasarawa State
3084	NW/0116	Olivet Medical Center	Behind Police Barracks, National Supply, Lafia, Nasarawa State
3085	NW/0118	Agu Hospital	Jos Road, Opp Skye Bank, Lafia, Nasarawa State.
3086	NW/0119	Edither Choice Hospital	1 Mohammed Mana Street Off Abacha Road,
3087	NW/0120	Diamond Clinic & Maternity	No.4 Tudun Gwandara Opposite Emmanuel Baptist Church Lafia Nasarawa State
3088	NW/0121	Oshyeg'Ba Josh Medical Centre Lafia	Behind Ministry Of Justice, Off Shendam Road, Lafia Nasarawa State
3089	NW/0122	Al-Nur Clinic & Maternity Home	Al-Nur Clinic & Maternity Home
3090	NW/0123	Anointed Clinic & Maternity	Behind Abdul Abdul Filling Station, By Ronkus International School, Mararaba Nasarawa State
3091	NW/0124	Bold Step Clinic & Maternity	Behind RCCG Rafin Kwara, Masaka.Nasarawa State
3092	NW/0125	Shabu-Crystal Health Hospital	Akurba Road, Shabu Lafia North Nasarawa State
3093	NW/0126	Neman Clinic & Maternity	Kagbaru Road, Before Capital Gate Way Hotel Ado .Nasarawa State
3094	NW/0128	Oshyeg'Ba Josh Medical Centre Lafia	Behind Ministry Of Justice, Off Shendam Road, Lafia Nasarawa State
3095	NW/0129	Al-Nur Clinic & Maternity Home	Tudun Wada Keffi, Nasarawa State
3096	NW/0135	Rayyan Hospital	Adjacent Keffi L.G.A. Nasarawa State
3097	NW/0136	San Health Project Lafia East	Newtaal Road Along Lafia East Primary School Lafia
3098	OD/0001	State Specialist Hospital,Ikare	Ikare Akoko
3099	OD/0002	Terebo Omowetan Hospital	Beside Muslim Primary School Ese Quarters, Ikare Akoko
3100	OD/0004	Epinmi Basic Health Centre	Epinmi, Ondo State
3101	OD/0005	Sijuwade Specialist Hospital	10,SIJUWADE BAA-SEGUN RD, AKURE
3102	OD/0006	St. David's Hospital	OBA ADESIDA RD.AKURE
3103	OD/0007	St. John & Mary Hospital	2 Adebayo Onilane Isikan Allase Akure
3104	OD/0008	Ade-Tade Hospital	5-7 Ade-Tade Str. Illesa Garrage, P.O.Box 3200, Akure
3105	OD/0009	Joe-Jane Medicine Centre	Okejebu Rd. Akure
3106	OD/0010	Abitoye Hospital	101, Ijoka Road, Akure

3107	OD/0011	State Specialist Hospital, Akure	Akure
3108	OD/0015	State Specialist Hospital,Okitipupa	Okitipupa
3109	OD/0018	Federal Medical Centre - Owo	Owo
3110	OD/0021	State Specialist Hospital,Ondo	Ondo
3111	OD/0022	Oludare Hospital	Fanibi Layout, Akure
3112	OD/0045	Adeyemi College Of Education Health Centre	Ondo State
3113	OD/0049	323 AR MRS, Akure	Akure, Ondo State
3114	OD/0050	Sick bay	FOB Igbokoda, Ondo State
3115	OD/0052	Liberty Hospital & Maternity	Oluwatunyi Quarters off Ijoko Road ,Akure
3116	OD/0053	First Mercy Specialist Hospital	19c Gbogi street, off Osa Adesida Road, Akure
3117	OD/0054	MOMAAK Specialist Hospital	3 Lafe Inn way, Okuta Eleri
3118	OD/0055	J & E Fatunla Hospital	11/13 Arisoyin layout Oke Arotitun (By IBIPRESS), Akure
3119	OD/0056	Christ Hospital	5 Adeyemi College Road, Ondo
3120	OD/0057	City Specialist Hospital	56 Oke Arata street, Igbo-Oliki, Akure, Ondo State
3121	OD/0058	Adedewe Okunriboye Hospital	Opposite Celestial Church, Ondo Road Bye pass, Akure
3122	OD/0059	Adelabu Hospital & Maternity Home	106 Sobe Road Ifon
3123	OD/0060	Anthony Adeleke Memorial Hospital	Okitipupa L. G. A, Ondo
3124	OD/0061	Laju Hospital	Road 7 Fumbi Fagun crescent, Ondo
3125	OD/0062	Ogunsusi Memorial Hospital	1 Zion Estate, Ile-oluji
3126	OD/0063	Emmanuel Clinic	2 Towobola street, Akinjagunla
3127	OD/0064	Fujah Specialist Hospital	22, Adetutu Street, Opp. Isikan Market, Akure, Ondo State
3128	OD/0073	DSS Clinic	Akure, Ondo State
3129	OD/0074	Police Clinics, Ondo	Ondo State
3130	OD/0075	Police Children School Clinic, Ondo	Ondo State
3131	OD/0076	Federal University of Technology Health Centre	Akure, Ondo State
3132	OD/0078	MIRACLE HOSPITAL	17A IWEALEWA STREET, AKURE
3133	OD/0081	BABALOLA NINEWELLS SPEC. HOSPITAL	46, ABITOYE STREET,OSHOKOTI LAYOUT,AKURE
3134	OD/0082	ARIF MEDICAL CENTER	BEHIND SABO MAIN MARKET ORE
3135	OD/0083	SHEKINAH HOSPITALS	BEHIND OLD OWO GARAGE, OFF EL-SHADDAI RD. AKURE
3136	OD/0084	OKE ROYAL HOSPITAL	CJO-8, OLORUNSHOGO STREET,IKARE-AKOKO
3137	OD/0085	AJIBOYE HOSPITAL	78, ADEMULEGU ROAD, ONDO
3138	OD/0086	INLAND MEDICAL CENTER	2, OLANIPEKUN LANE,BEHIND GEN. HOSPITAL, IKARE-AKOKO
3139	OD/0087	ROTSAM CLINIC	FUTA AREA, AKURE
3140	OD/0088	BABALOLA MEDICAL CENTER	21, HOSPITAL RD, ONDO TOWN
3141	OD/0101	19 BN MRS, Okitipupa	Okitipupa
3142	OD/0103	Ndubuisi Hospital & Maternity	Epetimehin Close, Off Brig Ademulegun Road, Ondo

3143	OD/0104	Wole Ayo Hospital Ltd	4 Olorunsogo Street off Brigadier Ademulegun Road, Ondo
3144	OD/0105	Kharis Medical Centre	2 Ondo Bye-Pass JUT, Oyeme Road
3145	OD/0108	Sckye Hospital Ltd	83B Oba Adesida Road, Akure
3146	OD/0109	Gloryfield Hospital	Ondo Road, Ore
3147	OD/0113	General Hospital, Ile Oluji	Ile Oluji, Ondo State
3148	OD/0114	General Hospital	Opp Anglican Grammar School, Oke-Ogbe, Iju-Itaogbolu, Ondo State
3149	OD/0115	Olatunde Medical Centre	Adelae Crescent, off Agunloye Street, Ondo Rd, Akure
3150	OD/0116	Simion Medical Centre	22, Lafa way, off Ilesa rd, Akure, Ondo State
3151	OD/0118	General Hospital Igbokoda	Omomira Street, Igbokoda
3152	OD/0119	National Institute for Educational Planning & Admin Health C	KLM 4, Laje Road, Ondo
3153	OD/0120	Opeyemi Medical Clinics Limited	Sagamu-Benin Express Way, Opp Ade Super Petroleum Ore
3154	OD/0121	Obanoyen Clinic Hospital	53, Irugbe street, Ijebu Owo, Ondo state
3155	OD/0122	Hopeland Specialist Medical Center	1, Arikawe Street, Akure, Ondo state
3156	OD/0123	The Catholic Hospital	Off Adeyemi college of Education rd, Oke-odunwo, Ondo
3157	OD/0124	Omololu Hospital	2, Akinjo Street, Oshokoti Layout, Oke-Ijebu, Akure
3158	OD/0125	St Louis Catholic Hospital	Owo Hospital Rd, Oke Ogun area, Owo, Ondo State
3159	OD/0126	Rainbow Medical center	9, Joy street, Ore, Ondo State
3160	OD/0127	Victory Specialist Hospital	4, Victory Street, Ibitayo Estate, Ondo
3161	OD/0128	Ibukun Olu Ayo Hospital	Off Ikoya Rd, Maryland Hotel, Okitipupa, Ondo
3162	OD/0129	Akintan Memorila Hospital	L/016A, Owolabo, Street, Ikare, Akoko, Ondo
3163	OD/0130	Ola Jesu Medical Clinic	King Emmanuel Rd, Ore, Ondo
3164	OD/0131	Owoyemi Specialist Hospital	5, Oshinle road, Akure-ondo, Olu Kayode roundabout, Akure, Ondo State.
3165	OD/0132	Joladet Clinic	9, Araromi Street, Owo, Ondo State.
3166	OD/0133	Dec Medical Clinic	No 4, Grace Ibitola Street, Ore, Ondo State.
3167	OD/0134	Mercy Land Medical Center	177, Ondo-Ore road, Ondo
3168	OD/0135	Rufus Giwa Polytechnic Health Center	Rufus Giwa Polytechnic Health Center
3169	OD/0143	Elizade University Medical Centre	Wuraola Adeojo Avenue, Ilara Mokin Ondo State

3170	OD/0144	Police Clinic	Owo Area Command Ijebu Owo Ondo State
3171	OD/0145	Police Clinic	Yaba Ondo
3172	OD/0146	Hope Hospital & Maternity	5, Owa Palace Road, Odode Idanre Ondo State
3173	OD/0147	New Day Clinic & Maternity	Opp. OSRC Gate Ijare Road, Akure Ondo State
3174	OD/0149	Goshenland Specialist Hospital & Fertility Centre	Block A. Plot 35 Aaye Community Oda Road. Akure Ondo State
3175	OD/0150	Midas Clinic	65,Oke-Ijebu Street Akure Ondo State
3176	OD/0151	Paramount Specialist Hospital & Fertility Centre	22 Sydney Emmanuel Crescent, Valentino Street, Ondo State
3177	OD/0154	Mao Hospital Limited	13, Gbangbalogun Street, Akure Ondo State
3178	OD/0155	Kaymas Medical Centre	Newtown, Oke-Aro, Akure Ondo State
3179	OD/0156	Miteda Specialist Hospital	Plot 5,Laoye Adegoke Road, Alagbaka, Akure Ondo State
3180	OD/0157	Blue Print Specialist Clinic	3, Falodun Avenue Oke-Ogun, Owo Ondo State
3181	OD/0158	Pima Hospital & Maternity	Off Okitipupa Road, Ore Ondo State
3182	OG/0001	Federal Medical Centre, Abeokuta	Bisi Onabanjo Way, Idi-Aba, Abeokuta, Ogun State
3183	OG/0002	Mercy Group Clinics	Back Of Uba Building Pansheke, Ibara, Abeokuta
3184	OG/0004	Femtob Specialist Hospital	No. 5 Obileye Street, Gra, Ijebu-Ode
3185	OG/0005	Egba Medical Centre	Ishabu, Abeokuta
3186	OG/0006	Oba Ademola Maternity Hospital	Ijemo, Abeokuta
3187	OG/0007	Neuropsychiatric Hospital	Aro, Abeokuta, Ogun State
3188	OG/0009	Kingsmith Hospital & Maternity	12 Ojodu Road, Akute, Ogun State
3189	OG/0026	State Hospital, Ijebu-Ode	Ijebu-Ode
3190	OG/0027	State Hospital, Abeokuta	Abeokuta, Ogun State
3191	OG/0031	Hetta Medical Centre	15, Martina Road, Sango, Ota
3192	OG/0032	Larion Medical Centre	8, Laz Ukwuoma Close, Morogbo Agbara, Lagos.
3193	OG/0034	Rophe Medical Centre	23, Anambra Crescent, Agbara Estate, Ogun State.
3194	OG/0036	Medicare Clinic	105, Idi-Iroko Road, Otta, Ogun State.
3195	OG/0039	35 AB MC Abeokuta	Abeokuta, Ogun State
3196	OG/0040	321 AR MRS, Abeokuta	Abeokuta, Ogun State
3197	OG/0041	12 FER MRS, Ijebu-Ode	Ijebu-Ode, Ogun State
3198	OG/0042	ESR MRS Owode	Ogun, Ogun State
3199	OG/0043	192 Bn MRS Owode	Owode, Ogun State
3200	OG/0044	Sick Post Music School	Otta, Ogun State
3201	OG/0045	Sick bay NNSS, Abeokuta	Abeokuta, Ogun State

3202	OG/0047	Olutunu Clinics	Agosa, Idiroko
3203	OG/0048	Tobiloba Clinic & Maternity	1 Tobiloba Way off Kuforiji-olubi Estate, Adigbe, Abeokuta
3204	OG/0049	Headstone Medical Centre	103 Idiroko Road, Owode Yewa
3205	OG/0050	Adura Majekodunmi Hospital	Akomoje Iberekodo Abeokuta
3206	OG/0051	Fame Medical Centre	No 48 joju Rd Sango Ota
3207	OG/0052	Mayowa Integrated Medical Services Ltd, Idi-Iroko	1, ALAWODE WAY, IDI-IROKO
3208	OG/0053	Central Specialist Hospital	133 Idiroko Rd Ota
3209	OG/0054	Bomi Clinics	No 5 Ibi Kunle Str Sango Ota
3210	OG/0055	Twins Specialist Clinic	2 Gate GRA Ota
3211	OG/0056	Hassan Medical Hospital	2 Baale Str Iloye Sango Ota
3212	OG/0057	Ayotola Specialist Hospital	3 Suraju Odunuga Street, Off Baruwa Str, Sagamu
3213	OG/0058	Victoria Memorial Hospital	Morogbo Agbara
3214	OG/0059	Lafiya Medical Center	No 6 Olanode Street Ayetoro Garage, Abeokuta
3215	OG/0060	Soremekun Memorial Hospital	127 Lagos-Abeokuta Rd, Vespa B/Stop, Ifo
3216	OG/0061	Agboola Hospital Ltd	19 Olori Rd Baale Aknosi Town, Ajuwon Iju
3217	OG/0062	Agbara Medical centre	1B Adigbogbo Str Jide, Agbara
3218	OG/0063	Alpha Clinic	3 Catholic Church Str., Ajegunle Olororo Aiyegbami, Sagamu
3219	OG/0064	Comprehensive Medical Centre	12 Lisabi Elite Road Idi-Aba
3220	OG/0065	Owokoniran Memorial Hospital	3 Owokoniran Street, Makun Station, Sagamu
3221	OG/0066	Federal College Of Education Health Centre Abeokuta	Abeoukuta
3222	OG/0067	Bisoyegboye Medical Centre	No 1B Taiwo Str, Makun, Sagamu
3223	OG/0068	Lanik Medical Centre	No 5 Imoru Str Ijebu-Ode
3224	OG/0069	Naval Sick Bay	Abeokuta
3225	OG/0070	Itunu Hospital	48 Tinubu Street Itezko Abeoukuta
3226	OG/0072	Akin-Olugbade Hospital	16 Iyalode Street Abeokuta
3227	OG/0073	Faramed Clinics	37 Akinwunmi Street Joji Rd. Ota
3228	OG/0074	Krownfit Medical Centre	Km 7 Idiroko Road Sango ,Ota
3229	OG/0075	Ariset Medical Centre and Ventures Ltd	6 Akunleyon Street Ota
3230	OG/0076	Rubee Medical Centre Ltd	Km 38 Abeokuta Motor Road, Sango, Ota
3231	OG/0077	Oluwalogbon Medical Center	Ifelodun Qtrs. Atan Ota
3232	OG/0078	Mulib Hospital	57 Ijoko Motor Road, Sango Ota
3233	OG/0079	First Line Clinics Hospital and Maternity	Km 2 Idiroko Rd Ota
3234	OG/0080	Ibukun Clinics	1 Agunloye Street, Otta
3235	OG/0081	Kolade Specialist Hospital	68 Idiroko Road, Sango Ota
3236	OG/0082	Ota Specialist Hospital Ltd	67 Idiroko Road, Ota
3237	OG/0102	DSS Clinic	Abeokuta, Ogun State
3238	OG/0103	Police Clinics, Abeokuta	Eleweran
3239	OG/0104	Federal Polytechnic Medical Centre	Ilaro, Ogun State

3240	OG/0105	University of Agric. Health Centre , Abeokuta	Abeokuta, Ogun State
3241	OG/0107	Diamond Cross Med. Centre	36 Okejigbo Rd, Iporo Ake, Ogun State
3242	OG/0109	Gbekelolu Medical Centre	3 Gbeke Opalami Street, Makun Sagamu, Ogun State
3243	OG/0110	Holy Cross Hospital	Ijaye Oja Aro Abeokuta, Ogun State
3244	OG/0111	Jesus is Love & Power Hospital	St. Mary''s Catholic Church Area Alaja, Ifo
3245	OG/0112	Medytop Specialist Clinic	3 Ejimo Alebiosu Str, Isale Ijagba Sagamu
3246	OG/0113	New Day Specialist Hospital	7 Sode Street, Akarigbo Sabo Sagamu, Ogun State
3247	OG/0114	Omonayajo Health Foundation Hospital	23 MKO Abiola Way Olorunsogo Oke Ola, Abeokuta, Ogun State
3248	OG/0115	Ore-Ofe Clinic	88A Ejirin Rd Ijebu-Ode, Ogun State
3249	OG/0116	Sacred Heart Hospital	Lantoro Abeokuta, Ogun State
3250	OG/0129	Hossana Specialist Hospital	Oja Odan Road, Ilaro
3251	OG/0131	Korede Hospital Ltd	17 Onikolobo Road, Ibara, Abeokuta
3252	OG/0133	Joseph Specialist Hospital	5, Surulere Street off Lalubu Street, Oke Ilewo, Abeokuta
3253	OG/0136	Overcomers Specialist Hospital	1 New Koregun Street, Behind Mouka Foam, Town Planning Area, Ilishan
3254	OG/0140	Lead Hospital	6 Fashola Street off Pipeline Road, Behind Mountain of Fire Church, Asore-Ajuwon
3255	OG/0141	Aladewura Clinic	33A Kuto Road, Abeokuta
3256	OG/0142	Ore Ofe Elshaddai Clinic	6 Isope Road off Ejirin Road, Texaco Junction, Ijebu Ode
3257	OG/0144	Olabisi Onabanjo University Teaching Hospital Shagamu	Shagamu, Ogun State
3258	OG/0146	Shalom Hospital & Maternity Home	71 Ilugun Road, Mokola, Abeokuta
3259	OG/0149	Adeyemi Medical Centre	4 Muri Abiola Street, Agbado Station
3260	OG/0151	Anuolu Poly Clinic	25 Sokabi Street, Isara Remo
3261	OG/0152	Lafia Kowa Specialist Hospital	12 Ejirin Road, Ijebu Ode
3262	OG/0156	Olu-Ola Specialist Hospital	12B Odo-Egbo Street, Ijebu Ode
3263	OG/0158	Akobi Specialist Hospital	Plot 5, Blk vw, Molipa Community layout, Ijebu-Ode
3264	OG/0160	Cornerstone Hospital	Beside Akute Grammar School, Akute
3265	OG/0161	Azygus Hospital & Maternity	12 Adekunle Adebayo Avenue, Ilupeju Estate, Agbado
3266	OG/0164	Michael & Alice Memorial Hospital	Plot 2, Block 31, Kemta Estate, Ajebo Road, Abeokuta

3267	OG/0165	Bisted Medical Clinic & Maternity	KM2 Abeokuta-Lagos Road, Beside Target Petrol Station
3268	OG/0166	Shaje Hospital	OOUTH Annex, Quarry Road, Shaje, Abeokuta
3269	OG/0167	St Shilo Medical Centre	35 Olujimi Fashola Street off Ilo Awele Road, Toll Gate, Ota
3270	OG/0168	Mamre Medical Centre	13 Ijoko Road, Arifanla, Akute
3271	OG/0170	Healing Spring Medical Centre	23 Ilishan Road, Iperu
3272	OG/0171	P.R Peterson Medical Centre	6 Adewale Adetokunbo Street, along NTA Road, Ijebu-Ode
3273	OG/0172	Bode Medical Centre	1 Odebunmi Street, Akute
3274	OG/0174	Amazing Grace Hospital	7 Iloro Street, Off New Road, Ijebu-Ode
3275	OG/0176	First Alpha Medical Centre	Kuforiji Olubi Drive, Adigbe, Abeokuta
3276	OG/0178	Redemption Hospital	Opp Deeper Life Camp, off Major Thompson, Sabo, Sagamu
3277	OG/0179	Abilak Medical Centre	Techno B/Stop, opp Access School, Ifo
3278	OG/0180	J-Pillar Clinic and Maternity	8 Iyana Iyesi Junction, Iyana Iyesi, Ota
3279	OG/0181	Beachland Specialist Hospital	Plot 10 Alfirdaus Estate, Arepo, Off Lagos-Abeokuta Express Way
3280	OG/0185	Surgicare Consult	Psalm 23, Oba Oluwa Road, GRA Housing Estate, Ibara, Abeokuta
3281	OG/0186	O.G.M Hospital	#5, Adenle str, off Akute-Ijoko Rd, Ogun State
3282	OG/0187	Strong Tower Hospital	Oke Aro Road, Agbado
3283	OG/0188	Babcock University Medical Centre	Ilisan Remo
3284	OG/0189	Otunba Tunwase National Paediatric Centre	Shagamu-Benin Express Way, Ijebu-Ode
3285	OG/0190	Ogunlade Hospital	35 Olukokun Street, Ijebu Ode
3286	OG/0191	Queen's Hospital	Poly Exp Road, Ilaro
3287	OG/0192	Shirish Clinic	102 Joju Road Otta
3288	OG/0193	St. Joseph Catholic Hospital	Oke-Agbo, Ijebu Igbo
3289	OG/0195	Comfort Clinic and Maternity	3, Ganiyu Ayinla str, off Celetial ave, Ereko qtrs, Ibafo
3290	OG/0196	Chirst The Saviour Medical Centre & Maternity	12, Pipeline rd, off Ojodu-Akute rd, Alagbole, Ogun state
3291	OG/0197	Chiers Medical Centre	2, Mabel Alake Adebiyi Close, Off Abeokuta Sports Club road, GRA, Ibara, Ogun State
3292	OG/0199	Goodluck Hopsital & Maternity Home Ltd	3, The Light Church Close, Mercy Estate, Asokore-Akute
3293	OG/0200	St Albert Great Clinic	Most Pure Heart of Mary Catholic Church Compound, Esure, Ijebu Mushin, Ogun State
3294	OG/0201	First Contact Hospital and Maternity	33B, Kuto Road, Behind Yemi-Ola Executive Stores, Abeokuta, Ogun State

3295	OG/0202	Abisola Medical Center	3, Akinolugbade Rd, Ita Iyalode, Abeokuta
3296	OG/0203	Ro'ona Hospital	7, Bonojo Street, Ijebu- Ode, Ogun State.
3297	OG/0204	Peace of God Medical Centre	75, Olusegun Osoba Rd, Oke-Ilewo Street (opp Access bank) Oke Ilewo, Abeokuta, Ogun State.
3298	OG/0205	Heritage Hospital Services	102A, Opeoluwa Street, Ita Oshin, Ogun State
3299	OG/0206	Brims Hospital	4, Muraina Soyode Street, behind Olub filling station, Ayetoro road Abeokuta, Ogun State
3300	OG/0208	Rapha Clinic & Maternity Center	42, Oguinsanya Street, Ali-Isiba Street, Sango-Ota, Ogun State
3301	OG/0209	State Hospital Ota, Ogun State	Ota, Ogun State
3302	OG/0210	Kaytuns Clinics	2, Ahmed Kayode Avenue, Arinko Housing Estate Sango Otta Ogun State.
3303	OG/0211	The Real Hope Hospital & Diagnostic Services LTD.	KM 3, Shagamu Road, Ita Oluwa Bus Stop, Ogijo, Ogun State
3304	OG/0212	Bliss Medical Centre	1, Jolasun Street Jolasco B/Stop Akute, Ogun State.
3305	OG/0213	Twins Specialist Clinic	Plot 11, Gunwa Ola Street GRA Extension, Sagamu, Ogun State
3306	OG/0214	Ishwot Consulting Hospital	1 Janet Jibowu Street, Ita- Oshin Behind Old Nepa Office Aro Ogun State.
3307	OG/0215	Samsteve Hospital	17, Ojodu Road By Mechanic B/Stop, Alagbole, Ogun State
3308	OG/0216	By- Faith Medical Center.	5, Aong Oba Adeniyi Sonariwo Primary School Road, Sagamu
3309	OG/0217	Ire-Ayo Hospital Ltd.	Itako-Ijebu Ltd, Ogun State
3310	OG/0218	Precious Gift Clinic & Maternity	18, Adekunle Fajuyi road, Fowosedo, Abeokuta, Ogun State
3311	OG/0219	Iyaniwura Specilaist Hospital	I Olujobi Falana close, Behind AP filling station, Mowe town, Sagamu, Ogun state
3312	OG/0220	God's Hope Hospital Limited	3/8, Apene Street, off Adigbe road, Adigbe, Abeokuta, Ogun State
3313	OG/0221	Idera Health Services Ltd	Blk A Plot Araromi CTCU Ltd Estate, off old Sgamu/Abeokuta road, OgunState
3314	OG/0223	Glory Medical Centre	15, Nuruddeen Oshodi Street, Kwara Qtrs Ibafo.
3315	OG/0224	Toyek Clinic & Maternity	Off Falana Street, Mowe,
3316	OG/0227	Alless Trinity Hospital	B, Taiwo Ogundeyi Street, Bode Olude Elega, Abeokuta Ogun State

3317	OG/0228	Broadland Hospital Service	Gasline, Ibafo Road Magboro, Ogun State
3318	OG/0229	Peace Han Clinic & Maternity Home	Km 11, Idiroko Road, Opp. Winners Canaanland, Ota, by May & Baker Ogun State
3319	OG/0232	Mike Medics Hospital	Aduba Estate, Akinbo Off Ijoko Road ,Akute Ogunn State
3320	OG/0233	St. Michael-Israel Specialist Hospital LTD	50,Awujale Street, Itale, Ijebu-Ode Ogun State
3321	OG/0234	Access Medical Centre	Behind PHCN Substation ,Ijeun Lukosi Road, Off Abiola Way, Abeokuta Ogun State
3322	OG/0236	Pillars Specialist Clinic	136,Akarigbo Road,Ijoku, Opp.First Bank Plc
3323	OG/0237	Mcpherson University Health Centre	Km 96, Lagos-Ibadan Expressway,Senki-Sotayo, Village Ogun State
3324	OG/0238	Concise Health Care LTD	7 Temidayo Crescent ,Off Adejumo Street ,Aro Lambo, Matogun Ogun State
3325	OG/0239	De-Green pearl Hospital	7A,Funsho Ogunmeyi Close, Off Igodo Road,Lagos-Ibadan Expressway Ogun State
3326	OG/0240	Ojugbele Specialist Hospital	105,Idiroko Road, Oju-Ore Ogun State
3327	OG/0241	State Hospital Isara-Remo	Oke-Oja, Isara-Remo,Ogun State
3328	OG/0242	True Vine Medical Centre	Ogun State Housing Corporation Estate (Old Estate) Sabo-Itanrin Road,Ijebu-Ode Ogun State
3329	OG/0244	Jon-Fem Hospital LTD and Maternity Home	63, Akeja Street, Off Anglican Road, Ilo Ifako Sango-Ota, Ogun State
3330	OG/0245	Life Builders Hospital	91,Igbusi Road Off Ilogbo Road, Iyana Ilogbo Bustop,Lagos-Abeokuta Expressway, Ogun State
3331	OG/0246	Angels & Eagle Specialist Hospital	9b Sikiru Jimoh Street, Beside Wemby Suites, GRA, Ibara, Abeokuta Ogun State
3332	OG/0247	Patricia Memorial Hospital	89,GAA Sabo Lafenwa, Abeokuta, Ogun State
3333	OG/0248	Abundant Life Hospital Ltd	1,Idahosa Street, Temidire Estate (Olopomeji Area),Off Igbodi Road, Off Adesan Road, Mowe, Ogun State
3334	OG/0250	Joybells Hospital	8,Illupeju Street, Off Jogbe Street, Ijebu-Ode Ogun State
3335	OG/0251	Odo- Esa Pry. Health Centre	Upper Olisa Street by General Hospital, Ijebu-Ode, Ogun State

3336	OG/0252	The Unique Great Shepherd Specialist Hospital	1,Olujide Somolu Rd., Opposite Fire Service HQRS., Onikoko, Abeokuta, Ogun State
3337	OG/0253	Emmanuelle Medical Services	Idera Quarters Besides NUD Primary School, Owode Egba Ogun State
3338	OG/0254	Shodes B Clinic & Maternity Home	61,Ishasi Road, Akute Ogun State
3339	OG/0255	Grace Medical Enterprises Nig. Ltd	1,Rafatu Alake Street, Off Olusegun Osoba Road, Agbado-Crossing Ogun State
3340	OG/0256	Bisayo Hospital	4,Lugboro Street, Car Wash, Adatan, Abeokuta Ogun State
3341	OG/0257	Maciland Medical Centre	Isunba-Lafenwa Adeleye-Aparadija Rd. Ogun State
3342	OG/0258	O & A Medical Centre	3, Asero Housing Estate,Asero Abeokuta. Ogun State
3343	OG/0259	3-Tees Medical Centre	10,Adebiyi Street, Off Messiah Praise School,Tipper Garage, Onibudu Estate,Akute Ogun State
3344	OG/0260	TC Specialist Hospital Ltd	5,Mosunmola Ashaye Street Makun, Sagamu,Ogun State
3345	OG/0261	Bode-Olude Hospital	HSE 2,Plot B,Block c, Popoola Family Estate,Bode-Olude, Abeokuta Ogun State
3346	OG/0262	Bells University of Technology Health Centre	Km 8, Idiroko Road, Benja Village Ogun State
3347	OG/0263	Bliss Hospital	127,Akarigbo Street,Ijoku, Sagamu,Ogun State
3348	OS/0003	Hope Medical Centre & Mat. Home	Oyan Road, Ila-Orangun
3349	OS/0004	Olunife Hospital	Ile Ife
3350	OS/0009	Olive Medical Centre	Ilesha
3351	OS/0010	Mojisola Medical Centre	No.5 Bibilari Avenue,Oke-Omi Iru, Osogbo Rd, Ilesha
3352	OS/0014	Klm Muslim Hospital	Feesu Road, Iwo
3353	OS/0016	Ibukun Olu Hospital	Obaagun, Osogbo
3354	OS/0018	Biket Medical Centre	Off Kuyi Square, Osogbo-Ikirun Road
3355	OS/0019	Adebare Specialist Hospital	Gbongan Road Osogbo
3356	OS/0021	Ladoke Akintola Teaching Hospital	Osogbo
3357	OS/0022	Abake Medical Centre	Odiolowo, Osogbo
3358	OS/0023	Alpha P-Hospital	24/26 Ayedun Street, Osogbo
3359	OS/0037	Oroki Hospital	No. 32, Ayetoro Street, Old Ikirun Rd Osogbo
3360	OS/0040	Obafemi Awolowo Teaching Hospital	Ile-Ife/Ilesha
3361	OS/0041	Adenle Memorial Hospital	Ilesha
3362	OS/0050	Obafemi Awolowo University Health Centre	Ile-Ife, Osun State
3363	OS/0052	ECC MRS, Ede, Osun State	ECC Mrs Ede, Osun State
3364	OS/0053	Onward Specialist Hospital	Agunbelewo Ilobu Road, Oshogbo

3365	OS/0054	Modupe Medical Centre	Oke Ola Apomu,Near Ayedaade Grammar School, Ikire, Osun State
3366	OS/0057	DSS Clinic	Osogbo, Osun State
3367	OS/0058	Police Clinics, Osun	Osun State
3368	OS/0059	Federal Polytechnic Medical Centre, Ede	Ede North, Osun State
3369	OS/0061	Seventh Day Adventist Hospital	LAGERE RD,ILE-IFE
3370	OS/0062	Our Lady of Fatima Catholic Hospital (Jaleyemi)	ODI-OLOWO,OSOGBO
3371	OS/0063	TITILOPE MEDICAL CENTRE	92A MORE OPP POLICE BARRACKS ILE IFE
3372	OS/0065	AL-AMEEN MEDICAL CENTRE	OKE-BOLA , GBONGAN
3373	OS/0066	Ronbay Hospital & Maternity	TALAFIA IMAM STR, BEHIND FED. POLY EDE
3374	OS/0067	Life Fountain Specialist Hospital	6 OLAFARE BIOBAKU STR. PARAKIN
3375	OS/0068	St. Victoria Specialist Hospital	OPP NTA MOKURO RD, ILE-IFE
3376	OS/0069	GBONJUBOLA MEMORIAL HOSP.	2185 CAC IGBONNA OSOGBO
3377	OS/0070	EBENEZER SPECIALIST HOSP.	17 STATION RD, OSOGBO
3378	OS/0071	BALM OF GILEAD SPEC. HOSPITAL	OKE-OPO GRA ILESHA
3379	OS/0072	St. Jude's Hospital	OFF FATIMA COLLEGE RD, LAAKOSIN IKIRE
3380	OS/0088	Our Lady of Lourdes Catholic Hospital & Maternity	Parde Road, Ipetumodu
3381	OS/0089	Anu Oluwa Hospital	Off Council Road, Isale Imole Area, Ilobu
3382	OS/0091	Morning Star Medical Centre	10, Oyetunde Street, Alekuwodo, Osogbo
3383	OS/0092	Apex Medical Centre	133 Ibadan Road, Ile Ife
3384	OS/0094	Oke-Ola Catholic Hospital	Oke-Ola Apomu, Isokan District
3385	OS/0095	Labab Hospital	8 Station Road, Inisha
3386	OS/0096	Bowen University Health Centre	Iwo
3387	OS/0098	Demal Clinic	# 30, Kola Adetunji Street, Oke Ijetu Ilesa Garage Area, Oshogbo
3388	OS/0099	Living Hope Hospital	Parakin obalufe Layout, Osun state
3389	OS/0100	Anu-Olu Hospital and Maternity	1, Alh Wahab Salami Str, Agip Area, Ede
3390	OS/0101	Life care Nursing & Maternity Home	Talafia Immam Street, Ede, Osun state.
3391	OS/0103	Spring Hope Specialist Hospital	Spring House, Old Ikirun Road, Oshogbo, Osun State
3392	OS/0104	Victory Hospital	1, Olumodan Compound, Iyalode street, Off FEESU road, Iwo, Osun State
3393	OS/0105	Okoye-Kola Hospital	20, Hospital Road, Isale Agunjin Ikirun, Osun State.
3394	OS/0106	Emmanuel Medical Centre	8 Deborah Oladogun Street Oshogbo

3395	OS/0107	Ayomide Women's Health Specialist Hospital	30 Powerline, Ikirun Road, Osogbo, Osun State.
3396	OS/0108	Hamstring Specialist Hospital &Diagnostic Centre	Isiah Okunola Street, off Okinni Grammer School, Okinni, Oshogbo
3397	OS/0109	Titilope Medical Centre	Jaleoyemi Street, Off Station Road, Ikirun, Osun State
3398	OS/0110	Victory Point Hospital	Ojoda New Town, Ile- idande, Osogbo, Osun State.
3399	OS/0111	Ultimate Specialist Hospital	No. 179 Fajuyi Road, Ile- Ife Osun State.
3400	OS/0112	Oloye Hospital	Gbongan Road, Beside GTBank, Ogo-Oluwa Area, Osogbo Osun State
3401	OS/0113	Living Hope Medical Centre	No. 26 Fola Aliu Street, Boripe Community, Rinsayo Area, Osogbo Osun State.
3402	OS/0114	Gold- Royal Hospital	Ojoyin Street, Beside De- Treasure Hotel, Ladere, Ile- Ife Osun State.
3403	OS/0115	Iyiola Hospital	Back Of Meritan Petrol Station, Off Ilesha Road, Ife Osun State.
3404	OS/0116	Shifa Medical Centre	No. 2 Old Osogbo Road, Odo- Amo A rea, Ikirun Osun State
3405	OS/0117	Abitop Medical Centre	Opposite Phase 2, OAUTHC Ile- Ife Osun State.
3406	OS/0118	Dunafort Hospital & Laboratory Services	Tinumola Area, Osogbo Osun State
3407	OS/0119	Goodness & Mercy Specialist Hospital	Christ life Centre, Ofatedo, Osogbo Osun State.
3408	OS/0120	Crystal Cross Specialist Hospital, Osogbo	Off Total Filling Station, After FRSC, Gbongan Road, Osogbo Osun State.
3409	OS/0121	Emmanuel Specialist’s Hospital	E27 Obayemi Street, Agborogboro Layout, Oranfe, Ilesha Road, Ile Ife Osun State.
3410	OS/0122	Hope Nursing And Maternity	Arulogun Area, Ede Osun State
3411	OS/0124	Hope Nursing And Maternity	Arulogun Area, Ede Osun State
3412	OS/0125	Jola-Olu Specialist Hospital	Agunbelewo Ilobu Road, P.O. Box 979, Osogbo Osun State.
3413	OS/0126	Zion Glory Hospital	Lane 1, Bolajoko Estate, Woru Area, Agunbelewo, Osogbo Osun State.
3414	OS/0129	Mercy Medical And Pathological Centre.	10 New Oba Road, Oke- Oniti, Osogbo Osun State
3415	OS/0130	Alpha Hospital & Maternity Home	9 Adetoro Street, Near Moricaz Mosque, Ede Osun State
3416	OS/0131	Amos- Alayo Hospital And Maternity	Surulere Area , Ikirun Osun State
3417	OS/0132	Olanrewaju Medical Centre	Jagun Aregba Compound,Okuku
3418	OS/0133	Alaafia-Oluwa Hospital And Maternity.	

3419	OS/0134	Osun State University	Oke-baale, Osogbo
3420	OS/0135	Mannabat Medical Centre	Opp. Old Tajudeen School, Off Sawmill Road, Ido-osun
3421	OS/0136	Olanrewaju Medical Centre	Jagun Aregba Compound, Okuku Osun State
3422	OS/0137	Aalafia–Oluwa Hospital And Maternity	Plot 5, Aladesanmi Street, Dada Estate, Osogbo Osun State
3423	OS/0138	Osun State University	Oke-baale, Osogbo Osun State
3424	OS/0139	BWB Hospital	No.5 orisumbare Street, Kasmo Area, Oke-baale,Osogbo Osun State
3425	OS/0140	Rehoboth Ear, Nose and Throat Hospital	Tajudeen Suleiman Street, Zone3, Behind M-Oyetunji Filling Station, Capital
3426	OS/0141	Oke-baale Central Hospital	Opp. Okebaale Police Station, Okebaale, Osogbo Osun State
3427	OS/0142	Safe Parturition (SP) Specialist Hospital	6, Abiodun Adeshina Street, Behind GOF Schools, Osogbo Osun State
3428	OY/0001	University College Hospital	Ibadan
3429	OY/0003	Tobi Medical Centre Ltd.	57/453 Felele Layout, Ibadan
3430	OY/0004	Molly Specialist Hospital	18, Iwo Road Idiape Junction, Ibadan
3431	OY/0005	Badejoko Memorial Hospital	Oremeji Mokola Opp. Premier, Hotel Junction, Ibadan
3432	OY/0006	Welfare Hospital Ltd.	Old Ife Road Newgbagi, Market Area, Ibadan
3433	OY/0007	Gold Cross Medical Clinic	36, Oje-Adeoye-Oje Road, Yemetu, Ibadan
3434	OY/0008	J. Rapha Hospital	No. 6, Aare Avenue New Bodija, Ibadan
3435	OY/0009	Adeoye Memorial Specialist Hospital	Nw/ 4/185 George Street, Ibadan
3436	OY/0010	Mobolaji Hospital	43, Obafemi Awolowo Way, Oke-Bola, Ibadan
3437	OY/0011	Idi-Ape Medical Centre	Plot 2 & 6, Afolabi Layout Idi-Ape, Ibadan
3438	OY/0012	Eyiolawi (Iyolawi) Memorial Hospital	Opp. Islamic High School, Orita Bashanu, Ibadan
3439	OY/0013	St. Martinas Hospital Ltd.	NW6/309 Akintola Road, Ekotedo, Ibadan, Oyo State
3440	OY/0014	Lanark Specialist Hospital	Sw9/1423, State Hospital Road, Ring Road Area, Ibadan
3441	OY/0015	Christus Special Hospital	Zion Building, Kayode Obenbe, Crescent, New Ire Road, Ibadan
3442	OY/0028	Lade Hospital & Maternity	SW 1171b Agege, Ibadan
3443	OY/0029	Oluwaseyi Hospital	SW9/337 Abeokuta Road, Ibadan
3444	OY/0030	J.V.M. Hospital	No. 26, Imalefalafia Street, Oke-Ado, Ibadan
3445	OY/0031	The Vine Hospital & Maternity Centre	Block 10 Plot 24, Alafin Avenue, Oluyole Extension, Ibadan

3446	OY/0032	Oke-Ado Hospital	SW7/217, Ibukun Olu Street, Oke Ado, Ibadan
3447	OY/0033	Kings Hospital	3a Sodeinde Close, Anfanni, Ring Road, Ibadan
3448	OY/0034	Trinity Hospital (Nig) Ltd.	84, Fajuyi Road Ekotedo Adamasingga, Ibadan, Oyo State
3449	OY/0035	Femi Specialist Hospital	7, Bcos Road Basorun, Ibadan
3450	OY/0036	Teju Specialist Hospital Annex	Oyo-Ogbomosho Road,Ibadan
3451	OY/0042	Kejide Specialist Hospital	Sw 9/704a Elewure Str., Challenge, Ibadan
3452	OY/0043	Mak Clinic	By Alma Guest House Junction, Opp. Nitel New Bodija, Ibadan
3453	OY/0044	Alafia Hospital	Mokola, Ibadan
3454	OY/0051	Teju Specialist Hospital	6 Alh. Animasaun Street, Ring Road, Ibadan
3455	OY/0052	Toun Memorial Specialist Hospital	Km 1/2 New Life Road, Ibadan
3456	OY/0063	Dorji Hospital	14, Avenue New Bodija, Ibadan
3457	OY/0064	Dorji Hospital & Maternity	Abidokun Close Police Molete, Ibadan
3458	OY/0065	Molly Specialist Hospital	No.6, American Quarters Yidi, Ibadan
3459	OY/0066	Metropolitan Specialist Hospital & Mat.	Ibadan,
3460	OY/0067	Lad Medical Centre	1 Lad Hospital Road, Beside Police Barracks, Orita Challenge, Ibadan
3461	OY/0068	Al-Hayyu Hospital	Saw-Mill Ife Road, Ibadan
3462	OY/0069	Saint Christopher's Hospital	10, Okeremi Street, Off Shobande Ave. Oremeji, Ibadan
3463	OY/0070	Asvon Hospital	Km 11/4 Opp. New Gbagi Market, New Life Road, Ibadan
3464	OY/0071	New Bodija Hospital Group	3, Ayoola Drive, New Bodija, Ibadan
3465	OY/0072	Lafia Hospital	Sw 9/960 Abeokuta Road, Apata, Ibadan
3466	OY/0073	Fadebi Memorial Hospital	Km 7, Iwo Road (Oki), Ibadan
3467	OY/0074	Modupe Hospital	Via Akinloye Oje Area, Ibadan
3468	OY/0075	Medical Practitioners Services	Mokola Roundabout, Ibadan
3469	OY/0076	Imperial Hospital	Yemetu, Ibadan
3470	OY/0077	Beta-Life Hospital	14, Popoola Yemoja Street, Off Awolowo Avenue, Oke Ado, Ibadan
3471	OY/0078	Highland Specialist Hospital	The House Of Hearing, Adeoyo Hospital Road, Yemetu, Ibadan
3472	OY/0079	St. Mary's Catholic General Hospital, Eleta, Ibadan	Eleta, Ibadan, Oyo State
3473	OY/0080	Dominic Hospital & Maternity	7 Up Bus-Stop Monatani, Agodi, Ibadan
3474	OY/0081	Skyline Specialist Clinic	14, Olalekan Alabi Close, Off Abayomi Bus Stop, Iwo Road, Ibadan, Oyo State

3475	OY/0083	Baptist Hospital	Oyo, Ibadan
3476	OY/0084	Fiditi General Hospital	Yemoja Area, Fiditi
3477	OY/0085	Florence Hospital	Near Co-Operative Building, Oja-Igbo, Ogbomosho
3478	OY/0086	Oyin Specialist Hospital	Airport Road, Ibadan
3479	OY/0164	DSS Clinic	Ibadan, Oyo State
3480	OY/0165	2 Div Hospital	Adekunle Fajuyi Cantonment, Ibadan
3481	OY/0166	NAF Medical Centre	NAF Air Force Comp Sec. Sch. Ibadan
3482	OY/0167	Muslim Hospital	Afote Road, Shaki
3483	OY/0172	Ireti Oluwa Hospital	Kajola Saw-Mill, Apake, Ogbomosho
3484	OY/0173	General Hospital, Iseyin	Iseyin, Oyo State.
3485	OY/0174	Our Lady Catholic Hospital	Iseyin, Oyo State.
3486	OY/0175	Adeoyo Maternity Hospital	Yemetu Road, Ibadan.
3487	OY/0176	Ring Road Hospital	Ring Road, Ibadan
3488	OY/0177	Jerico General Hospital	Magazine Road, Jerico, Ibadan.
3489	OY/0178	State Hospital, Oyo	Oyo, Oyo State
3490	OY/0179	State Hospital, Ogbomoso	Ogbomosho, Oyo State.
3491	OY/0180	Oni Memorial Children Hospital	Ring Road, Ibadan
3492	OY/0182	State Hospital, Saki	Saki, Sango Road, Ibadan, Oyo State
3493	OY/0183	Baptist Medical Centre, Saki	Ajegunle Area, Saki, Oyo State
3494	OY/0184	Jerico Nursing Home	Magazine Road, Ibadan.
3495	OY/0186	244 Recce Bn MRS Shaki	Shaki, Oyo State
3496	OY/0187	81 Bn MRS, Ibadan	Ibadan, Oyo State
3497	OY/0188	Cottage Hospital/Comprehensive Health Centre	Oyo Police Command, Ibadan, Oyo State
3498	OY/0189	Queen's Hospital	38, Iya Olobe St. Dugbe Eketedo Ibadan
3499	OY/0190	St Lucia Hospital	Oke-Ado Ibadan
3500	OY/0191	Unity Medical Centre	31 Eleyele Rd Ibadan
3501	OY/0192	St. Joseph Medical Centre	No 27 Akintola Rd Ekotedo Ibadan
3502	OY/0193	Full House Spec. Dermatological Hospital	37, Adeniran Onyulola Ave, Ring Rd Ibadan
3503	OY/0194	Jokotola Memorial Infirmary Hospital	Sw8/1073 Imalefalafia St Oke Ado Ibadan
3504	OY/0195	Anu Oluwa Specialist Hospital	19 Bluegate Avenue, Oluyole Estate Extension, Ibadan
3505	OY/0196	Joy Hospital & Maternity Centre	32 Morgan St. Oke-Ado Ibadan
3506	OY/0197	Fajimi Memorial Hospital	Aba Eja, Tinuoye, off Army Barrack Rd Behind Total Petrol Station Ojoo
3507	OY/0198	Mommoh Memorial Hospital	Idi-Ope Oyo
3508	OY/0199	New Day Hospital	Behind Hosanna Joe Restuarant Off Ibadan-Lagos Express Rd Owode-Academy Area, Ibadan
3509	OY/0200	Faith Foundation Hospital	Okeho Rd Iseyin Oyo State

3510	OY/0201	Faith Medical Hospital	No. 40 Nitel Rd, Ogbomosho Oyo State
3511	OY/0202	Shekinah Medical Centre	No.2 Bamgboshe Street Owode Oyo
3512	OY/0203	Victory Medical Centre	House 4, Road 120,80,20, NW7 Poly-Eleyele Rd, Mosafejo B/Stop Ibadan
3513	OY/0205	Olayonu Medical Centre Hospital & Maternity Services	Ole Agbala Masia Area Ogbomosho, Oyo State
3514	OY/0206	Shalom Medical Centre	Sabo Area Ogbomosho
3515	OY/0207	Oluwaseun Clinic & Maternity	Toye Ajagun Area NNPC Pipeline Okonlawo L/out Apata Ibadan
3516	OY/0208	Modupe Hospital	Powerline Onikokoro Akobo Jurin, Ibadan
3517	OY/0210	Divine Favour Hospital	Iyana Church, Along Isebo Rd, Jinarere Bus stop, Ibadan
3518	OY/0211	Zoe Specialist Mission Hospital	No.4 Adeogun Str., Oluyele Estate, Ibadan
3519	OY/0212	Continental Medical Centre	S7/998A Engineer Adebajo Oshinbowale Close, Challenge Ibadan
3520	OY/0213	NAF Detachment Medical Centre	Ibadan, Oyo State
3521	OY/0242	Bowen University Teaching Hospital, Ogbomoso	Ogbomosho, Oyo State
3522	OY/0243	New World Specialist Hospital	No. 7, Omona Yajo Close, Behind Children Home Sch. College Cresc., Molete Area, Ibadan
3523	OY/0245	Bisol Medical Centre	Ilora, Beside Immanuel Baptist Church Mafojubaye Street, Ilora
3524	OY/0246	Ayo Hospital	Premium House Annex, Magazine Road, Jerico, Ibadan
3525	OY/0249	Ayo Hospital	Behind St. Lucia Hospital, Oke-Ado, Ibadan
3526	OY/0250	Catholic Hospital, Oluyoro	Oluyoro, Ibadan, Oyo State
3527	OY/0251	Bethel Specialist Hospital	30, Oyo Road, Coca/Oremeji Area Ajibade Bus Stop, Ibadan
3528	OY/0252	University of Ibadan Health Services (Jaja Health Clinic)	Ibadan, Oyo State
3529	OY/0253	Fed. College of Educ. Special Medical Centre	Oyo State
3530	OY/0256	Abiola Memorial Hospital	N4/790 Yemetu, Ibadan
3531	OY/0257	Aggrey Hospital	92 Yemetu Adeoyo Rd, Yemetu-Oja Bus Stop, Ibadan
3532	OY/0258	Alawaye Hospital & Nursing Home Ltd	7, Alawaye Hospital Road, Iwo Road, Ibadan
3533	OY/0259	Alms Hospital	Esuruoso Layout, Sharp Corner, Apata, Ibadan
3534	OY/0260	Ariyo Medical Centre	57/57B Oba Abimbola Rd, Felele, Ibadan

3535	OY/0261	Ariyo Medical Centre Ltd.	7 Irepodun Str Agbowo Bodija (Near the Market), Ibadan
3536	OY/0262	Christus Specialist Hospital	Olive House Blk N6/53 Fajuyi Rd, Ibadan
3537	OY/0263	Banby Specialist Hospital	Plot 11, Blk XLII, Akobo Estate, Olorunda-Abaa Rd, Ibadan
3538	OY/0264	Comfort Medical Centre	10, Lakanmi Close to Toatle Garden, Ibadan
3539	OY/0265	SOLAAK SPECIALIST CLINICS	11 SODEHINDE STR, OFF ANFANI RD, RING RD, IBADAN
3540	OY/0268	DOCTORS POLYCLINIC	12 AKINTOLA STR, AGBOWO U.I IBADAN
3541	OY/0269	JOLAMADE SPECIALIST HOSPITAL	PLOT C2-C3 SALAMI SOMADE ESTATE,BASORUN, IBADAN
3542	OY/0270	IBADAN CENTRAL HOSPITAL	OSOSAMI RD, OFF RING RD,IBADAN
3543	OY/0271	FORTUNE HOSPITAL & DENTAL CENTRE	32 MKO ABIOLA WAY , RING RD, IBADAN, ANFANI ROUNDABOUT
3544	OY/0272	HERITAGE COMPREHENSIVE HOSPITAL	57 ANFANI RD, RING RD, IBADAN
3545	OY/0273	VICTORY HOSPITAL	SW 8/949F ADE- OLUYOYE STR, OSOANMI, OKE ADO
3546	OY/0275	MERCYLAND HOSPITAL & DENTAL	OPP. NEW GBAGI MARKET NEW IFE RD,IBADAN
3547	OY/0276	EBENEZER CLINIC (HOSP. & MAT. HOME)	3/5 ONI LANE, OFF YEMETU ADEOYO RD,IBADAN
3548	OY/0277	JOLALEKE SPEC. HOSPITAL	12 ALONG FODASIS RD, OPP. NEW ADEOYO HOSPITAL. IBADAN
3549	OY/0278	Shalom Group Medical Centre	Aboderin Close, Agbaje Orita Challenge, Ibadan
3550	OY/0279	CROWN HOSPITAL	SW 9/1147A AGBAJE B/STOP, ORITA CHALLENGE,IBADAN
3551	OY/0280	HOPE MEDICAL CENTRE	Yemetu - Adeoyo Bere Road
3552	OY/0281	FELEWA HOSPITAL	SW8/442A COLLEGE CRESC, OKE ADO IBADAN
3553	OY/0282	GRACE HOSPITAL & MATERNITY	1, AROTAYO STR, AROMETA BUS-STOP, SANGO/POLY ROAD, ELEYELE, IBADAN
3554	OY/0283	HOSSANA SPEC. HOSPITAL	95AB SURULERE STR, ABGOWO U.I. IBADAN
3555	OY/0284	LAFIA HOSPITAL	60B FAGBOUN RD, OKEBLA. IBADAN
3556	OY/0285	Peamak Hospital & Maternity Centre	Alalubosa Road Oyo
3557	OY/0286	PROVIDENCE POLYCLINIC	OLAOGUN OLD IFE RD, IBADAN
3558	OY/0287	ROYAL CLINIC & MAT. NIG. LTD	58 OYO RD, VETERINARY B/STOP MOKOLA, IBADAN
3559	OY/0288	Santhos Hospital	Odedele Odeleke Street, Challenge, Ibadan
3560	OY/0289	ST. GEORGE'S NURSING HOME	57 OBAFEMI AWOLOWO RD, OKEBOLA . IBADAN

3561	OY/0290	ST.Jacob's Msission Hospital	Road 26, Owode Housing Estate, Beside Tafo Event Center, Along Abeokuta Road, Apata, Ibadan
3562	OY/0291	ST.VINCENT MEDICAL CENTRE	34 AARE AVENUE , NEW BODIJA ESTATE,IBADAN
3563	OY/0292	TZ HOSPITAL & MATERNITY	AGBAKIN LAYOUT BEHIND MR BIGGS IWO RD,IBADAN
3564	OY/0343	Nigerian Railway Hospital	Dugbe, Ibadan, Oyo State
3565	OY/0344	CORNERSTONE MEDICAL CENTRE	N6A/816B Loco Street, Sango, Ibadan
3566	OY/0345	Chrisbo Medical Centre	House 11, Plot 1, Phase III, Bashorun Estate, Akobo, Ibadan
3567	OY/0348	Foremost Base Hospital Limited	1 Rufai Street N6A/958 Coca Cola Area, Ibadan
3568	OY/0350	St Rita's Clinic & Maternity Home	9 Maboreje Street, Iwo Road, Ibadan
3569	OY/0354	St Patric's Hospital	107/780 Idi Ope
3570	OY/0357	St Marello Catholic Hospital	Opere Village, Ibadan
3571	OY/0358	Restoration Hospital	12, Alagbangba Street, Off Ashi Road Ibadan
3572	OY/0360	KAY KAY SPECIALIST	PLOT 15 BLK 10 ORITA BASHERUN ESTATE,AKOBO IBADAN LAGELU L.G.A
3573	OY/0361	Iye Hospital Ltd	22 Osibodu Layout Lagos/Ibadan Express Road, Ibadan
3574	OY/0363	Samron Medical Centre	62 Adebiyi Street, Joyce - B Road off Ring Road Ibadan
3575	OY/0365	First Life Hospital	5 Molusi Street Molete, Ibadan
3576	OY/0366	St Dominic Catholic Hospital	Ogungbade Village, New Ife Road, Ibadan
3577	OY/0367	Hamdala Hospital & Maternity	Transformer Bus Stop, Agbowo, UI, Ibadan
3578	OY/0368	Abayomi Hospital	Idi-Oro, Behind California Hotel, Ogbomosho
3579	OY/0369	Laduntan Hospital	Okegbade Road, Ogbomosho
3580	OY/0372	Immaculate Hospital	23 Odutola Road Off Ososami Road, Oke Ado, Ibadan
3581	OY/0376	Odinjo Primary Health Centre	Ibadan
3582	OY/0378	Mapo Primary Health Centre	Ibadan
3583	OY/0379	Oranyan Primary Health Centre	Ibadan
3584	OY/0381	Molete Primary Health Centre	Ibadan
3585	OY/0383	Oke Olola Primary Health Centre	Oyo
3586	OY/0384	Koso Primary Health Centre	Oyo
3587	OY/0385	Otefon Primary Health Centre	Oyo
3588	OY/0386	Igbonla Primary Health Centre	Oyo
3589	OY/0387	Ibrahim Taiwo Primary Health Centre	Ibrahim Taiwo Way, Ogbomoso
3590	OY/0388	Okeelerin Primary Health Centre	Ogbomoso
3591	OY/0390	Isale Oyo Primary Health Centre	Oyo
3592	OY/0391	Ilowagbade Oyo Primary Health Centre	Oyo
3593	OY/0392	Oke Oroki Oyo Primary Health Centre	Oyo

3594	OY/0393	Muslim Ummah Hospital	KM 5 Kiamo Road Kisi
3595	OY/0395	Sunday Adewusi Jagun Primary Health Centre	Ogbomoso
3596	OY/0396	Tarra Health Centre	Ogbomoso
3597	OY/0397	Ikose Health Centre	Ogbomoso
3598	OY/0398	Masifa-Aguodo Health Post	Ogbomoso
3599	OY/0399	Benbow Medical Centre	Gbelekale Junction, Arein Road, Aremon, Ibadan
3600	OY/0402	Anu Oluwa Hospital	Opp The Blood of Jesus Apostolic Church, Oke Itunu, Mokola Ibadan
3601	OY/0403	Al-Amaq Medical Centre	Oke-Bola Street, Ibadan
3602	OY/0405	Beta Life Hospital	6 Adegbite Street, Agbowo, University of Ibadan
3603	OY/0406	Camaria Specialist Hospital	13 Camaria Road, Oluwa Bashorun, Ibadan
3604	OY/0408	Elyon Sepcialist Clinic	72 Iwo Road, Ibadan
3605	OY/0409	Nasrullahi Medical Centre	53/27, Labo Area, Ibadan
3606	OY/0410	Beatrice Medical Centre	16 Bode Kumapaiji Street, Off Iwo Road, Ibadan
3607	OY/0411	Lagelu Medical Centre	Plot 19, Block 40, Oluyole Estate Extension, Ibadan
3608	OY/0413	Christ Hope Hospital and Maternity	59 Ijaye Road, Moniya, Ibadan
3609	OY/0414	Ola Oluwa Medical	SW8/6333, Isale Ososami, Ibadan
3610	OY/0415	Durojaiye Memorial Hospital	7 Animashaun Street near Muslim Grammar School, Odinjo, Ibadan
3611	OY/0417	Sunnydale Hospital	1 Main Avenue, Alalubosa, GRA, Ibadan
3612	OY/0419	Mosaq Ogo-Oluwa Hospital	No. 242 Aiyepe Area, Ibadan
3613	OY/0420	Jobi Memorial Hospital	Opp St Cyprian Catholic Church, Oke Offa, Ibadan
3614	OY/0423	Omotayo Maternity Home	SW8/517A Oluyole Street, Oke Ado Ibadan
3615	OY/0424	Lily Hospital and Maternity	Beside People of Faith Gospel Church, Providence, off Aro Meta B/Stop Eleyele Rd, Ibadan
3616	OY/0425	Oluwayomi Hospital	3 Oyeniwe Street, Olorombo, Bashorun, Ibadan
3617	OY/0426	Ayofunmi Hospital and Maternity	43 Polytechnic Road, Sango, Ibadan
3618	OY/0429	Vine Branch Maternity and Infant Welfare Centre	Plot 4, Block 5, Road 302 Agodi GRA, Ibadan
3619	OY/0433	Saanu Memorial Hospital	Ijokodo Agbaje Road, Ibadan
3620	OY/0434	Aramed Medical Centre	KM 15, Lagos-Ibadan Expressway, Ibadan
3621	OY/0436	Adeoto Memorial Hospital	Popo Bus Stop, Akobo Ojurin, Ibadan
3622	OY/0437	Premier Hospital	SW5/265 Atere Compound, Itamaya Ibadan

3623	OY/0439	Hallel Specialist Hospital	# 11, Ilupeju Estate, Akobo-ojurin, Ibadan.
3624	OY/0440	Evangel Med Center	Dejo Dairo Street, Adex B/Stop, Monotan, Ibadan
3625	OY/0441	Oroki Med Centre	Oke Oroki, Oyo
3626	OY/0442	Mayokun Hospital	Ajangba Estate Kisi
3627	OY/0446	St Anne's Specialist Hospital	#9, Moremi Rd, off Aare Avenue, Bodija, Ibadan
3628	OY/0447	Odun Lakin Specialist Hospital	Plot 18, & 19, Block 5B, Adewumi Layout, Afani Rd, Ibadan
3629	OY/0448	Cocoa Research Institute Of Nigeria Health Centre	Idi -Ayunre, km 14, Ibadan - Ijebu Ode road, Oyo state
3630	OY/0451	Ebimacc Maternity & Health Care Clinic	Odo-Ona, Elewe, Academy road, off Orita challenge, Ibadan, Oyo state
3631	OY/0453	Oluwafemi Specialist Hospital	Gbogun Owode, Apata, Ibadan, Oyo State
3632	OY/0454	Shiloh Medical Centre & Maternity	Lane 17, Ajokukan Ave, Elebu, Oluyole Ext. Ibadan, Oyo State
3633	OY/0455	God’s Faithfulness Hospital	77, Apanpa Layout, Imalefalafi Street, Behind Odutola Tire Factory, Ibadan, Oyo State
3634	OY/0456	Castle Hospital & Maternity Center	45, Adeyi Avenue, Old Bodija, Ibadan, Oyo State
3635	OY/0457	Abraham & Maria Hospital & Diagnostic Centre	1 Emmanuel Adewale Avenue, Off Arola – Ayegun Road, Ibadan, Oyo State
3636	OY/0458	Baptist Cottage Hospital, Omi-Adio	Oke-Oloro Street, Omi-Adio, Oyo State
3637	OY/0459	Olayinka Maternity Health Center	Plot 10,Coker Layout, Idi-Ishin, Jericho Quaters (Aka St.Florence Clinic, Agbeni), Ibadan,Oyo State
3638	OY/0460	Segun Adeniyi Memorial Health Clinic& Maternity Home	17, Adedayo Street Kuola, Apata, Ibadan, Oyo State
3639	OY/0461	Naomi Medical Centre	3, Araromi Lane, Beside Daily Times Office, Oke Padre, Ibadan, Oyo State
3640	OY/0462	The Horizon Hospital	23, Odutola Street, Behind Conac Optical Imalefalafia,Oke-Ado, Ibadan,Oyo State
3641	OY/0463	God’s Faithfulness Hospital	77, Apampa Layout, Imalefalafi Street, Behind OdutolaTyre Factory,Ibadan, Oyo State
3642	OY/0464	Molete Diagnostic & Clinical Services Limited	270,Obafemi Awolowo Way, Molete, Oyo State
3643	OY/0465	Best Care Hospital	1, Alhaji Adeniran Street, Oke Itunnu, Mokola, Ibadan, Oyo State
3644	OY/0466	Royal Crown Specialist Hospital	Plot 7, Phase II Akobo, Ibadan, Oyo State

3645	OY/0467	Oniwumi Medical centre	Abayomi Street, Off Abayomi Bus Stop, Iwo Road Ibadan, Oyo State
3646	OY/0468	Police Clinic, Agodi	Agodi
3647	OY/0470	Police Clinic, Ogbomoso	Ogbomoso
3648	OY/0471	Police Clinic, Challenge	
3649	OY/0474	Lead City University	Lead City University Near Toll Gate, Ibadan Oyo State
3650	OY/0475	College Medical Centre	Federal College Of Animal Health And Production Technology Ibadan, Oyo State
3651	OY/0479	Zion Hospital & Maternity	Oke Ayo Premises, Beside Oando Filling Station, Ayaganku Expressway, Ibadan Oyo State.
3652	OY/0480	Glory Medical Centre	Adjacent Overcomer School, Mabolaje, Oyo Oyo State.
3653	OY/0483	The Issachar Medical Centre	11 Super Guest House Road, Aba Alfa (Ogundele Estate), Ibadan Oyo State
3654	OY/0485	Redeemer's Health Centre	37 Awolowo Avenue Old Bodija, Ibadan Oyo State
3655	OY/0487	Bronx Hospital Ltd	6 Oba Oyewusi Street, Off Tunde Lakanmi Crescent, Off Osuntokun Avenue, Old Bodija, Ibadan. Oyo State.
3656	OY/0488	Ramoth-Gilead Hospital	44, Liberty Stadium Road, Ibadan Oyo State.
3657	OY/0489	Alaafia Tayo Clinic And Maternity	Plot4 Alagba Layout Olomi Ibadan Oyo State.
3658	OY/0490	Police Clinic Oyo	Atiba Police Barracks, Akunlemu
3659	OY/0491	Montana Medical Centre	No. 7 Kajegbo Str. Opp. Chicken Republic Elewura Challenge Ibadan Oyo State
3660	OY/0493	Agugu Central Hospital	No. 12 Agugu Oluyoro Road Ibadan Oyo State
3661	OY/0494	Safepath Hospital	No. 3 Adejumo Str. Behind Zenith Bank Challenge Ibadan Oyo State
3662	OY/0496	St. Macrina Hospital Ltd	Swb/21 College Crescent Molete Challenge Ibadan Oyo State
3663	OY/0497	The Rock Medical Services	21 Excellent Avenue Alabidun Airport Road Alaika Ibadan Oyo State
3664	OY/0499	Hermon Pharmcare Nig. Ltd.	1 Irepodun Close, Off Ososami New Adeoyo Round About Ring Road Ibadan Oyo State
3665	OY/0500	Zenith Hospital	Opp. Airmark Filling Station, Oluyole Ext., Ibadan Oyo State
3666	OY/0501	Ogo Oluwa Welfare Medical Centre.	Opp. Baptist High Sch. Kajola, Ogbomoso Oyo State.
3667	OY/0502	Delight Hospital & Fertility Centre	No. 1, Emmanuel Close, Elewura, Ring road, Ibadan Oyo State

3668	OY/0503	King’s Way International Hospital	1 Adenuga Str. Kogi, New Bodija Ibadan Oyo State
3669	OY/0504	God’s Knot Hospital	7, Ologuneru Rd. Iyana Ekerin Beside Police Station Ibadan Oyo State
3670	OY/0507	Al-Safura Medical Diagnostic	Near Kadupe Community Bank Sanngo, Saki Oyo State
3671	OY/0511	Jubilee Hospital	Samonda Market Complex, Sango Behind Oyo State Trade Fair Complex, Ibadan Oyo State.
3672	OY/0512	Grace & Favour Specialist Hospital LTD	Apa-Baale Bus Stop Off Love Of Christ Global Church, Oyo State.
3673	OY/0513	Moviv Specialist Hopital Limited	Adetoun Area, Off Eruwa Road Ologuneru Eleyele, Ibadan Oyo State.
3674	OY/0516	Ayokemi Hospital & ultrasound centre	Adex Bus/Stop Behind filling Station, Monatan Ibadan, Oyo State.
3675	OY/0518	Ladoke Akintola University of Technology Teaching Hospital	Ogbomoso, Oyo State
3676	OY/0520	Ladoke Akintola University Of Technology	Ilorin Road Ogbomoso Oyo State
3677	OY/0523	Popular Hospital	34,Arigidi Street, Old Bodija, Ibadan Oyo State
3678	OY/0524	Sure Hope Clinic	Carpenter Bus Stop, Ologuneru Eruwa Road Ibadan, Oyo State
3679	OY/0530	Joy Hospital & Maternity Centre	32, Adeyinka Morgan Street, Oke-Ado, Ibadan
3680	OY/0532	The family specialist Hospital	15, Water Reservoir Road, Oluwala Basorun, Ibadan
3681	PL/0001	General Hospital, Barkin-Ladi	Barkin-Ladi
3682	PL/0003	Cottage Hospital, Bassa	Bassa
3683	PL/0005	Cottage Hospital, Bokkos	Bokkos
3684	PL/0007	Eldin Specialist Hospital	12 Dogon Dutse Road, Jos
3685	PL/0008	New Era Clinic	Jk 27 Chollon Street, Off Akpata
3686	PL/0009	Sauki Clinic & Hospital	Joseph Gomwalk Road
3687	PL/0010	Kauna Hospital	35 Ahmadu Bello Way, On Rwang-Pam
3688	PL/0011	General Hospital, Langtang	Langtang, Jos
3689	PL/0014	Chillas Specialist Hospital	20 Zaria Bye Pass, Jos
3690	PL/0015	General Hospital, Pankshin	Pankshin
3691	PL/0018	Alpha Medical Centre	7, Delimi Street, Jos
3692	PL/0019	Adoose Specialist Hospital	25, Ibrahim Dasuki Street
3693	PL/0020	Comprehensive Health Centre	Dadin Kowa
3694	PL/0022	General Hospital, Shendam	Shendam
3695	PL/0023	Gwash Specialist Hospital	New Abuja, Opp Summary Hotel, Dadin Kowa, Jos South LGA
3696	PL/0057	3 Div Hospital	Maxwel Khobe Cantonment, Jos
3697	PL/0058	463 Nigerian Airforce Hospital	NAF Station, Jos
3698	PL/0059	Federal Staff Clinic	Federal Secretariat, Jos, Plateau State

3699	PL/0061	332 AR MRS Shendan	Shendan, Plateau State
3700	PL/0064	University of Jos Health Centre	Jos, Plateau State
3701	PL/0066	Cottage Hospital/Comprehensive Health Centre	Police Staff College, Jos
3702	PL/0067	Matanmi Hospital	89 Masallachin Juma''a Jos
3703	PL/0068	Memorial Medical Centre (Dakwak)	Special Site Pankshin
3704	PL/0069	May Hospital	Yelwa Rd Box 181 Shendam
3705	PL/0070	Nissi Dominus Medical Centre	Jaktu Estate Mangu
3706	PL/0071	Helpang Medical Centre	Helpang Barikin Ladi
3707	PL/0072	Samantha Lucil Hospital	16, Enugu-Agidi street Jos
3708	PL/0073	Bene Clinic	Yelwa Road Box 204 Shendam
3709	PL/0075	Our Lady Of Apostle Hospital (O. L. A.)	1 Zaria By pass Jos
3710	PL/0076	Solat Women Hospital	44 Kashim Ibrahim Street Jos
3711	PL/0077	Bukuru Specialist Hospital	Gyel Gyero Road Jos
3712	PL/0078	Federal Lowcost Clinic And Maternity	Federal Lowcost Miango Road Jos
3713	PL/0079	Nassarawa Medical Centre	4B Dogon Dutse Jos
3714	PL/0080	Lawrak Hospital	21, Temple Road Off Miango Road Jos
3715	PL/0100	Jos University Teaching Hospital	Murtala Mohammed Way, Jos
3716	PL/0102	Plateau Specialist Hospital	Jos, Plateau State
3717	PL/0103	DSS Clinic	Jos, Plateau State
3718	PL/0104	Police Clinic, Jos	Jos, Plateau State
3719	PL/0105	Fed. College of Education, Pankshin	Pankshin, Plateau State
3720	PL/0106	National Inst. for Policy & Strategic Studies Medical Centre	Jos South, Jos, Plateau State
3721	PL/0107	Ola Hospital & Orphanage Zawan	Ola Hospital, Zawan, Plateau State
3722	PL/0108	Hwolshe Medical Centre	Tudun Wada Ring Road, P O Box 8103, Anglo, Jos.
3723	PL/0112	Virgill Mem Catholic Health Centre	Namu P O Box 10 Shendam, Jos
3724	PL/0113	Federal College of Education Staff Clinic	FCE, PMB 27, Jos, Plateau State
3725	PL/0115	Aminci Hospital	Bwandang, Bukuru, Jos
3726	PL/0116	Focus Hospital	No. 7 NEPA Close, Tudun Wada Layout, Jos
3727	PL/0117	Jenvak Hospital	22, T/Wada Ring Road, Jos
3728	PL/0120	Ultima Clinic & Maternity	15, Fudawa Ring Road, Yan-Trailer, Jos
3729	PL/0121	Vom Christian Hospital	Vom, Plateau State
3730	PL/0122	NIPSS Staff Clinic	Kuru, Jos
3731	PL/0124	Skane Radio-Diagnostic Centre Nig. Ltd	32B Ibrahim Taiwo Road, Jos, Opp. Plateau Hospital, Jos
3732	PL/0139	M & B Medical Centre	Dorowa Village, Bukuru
3733	PL/0141	Tadam Medical Centre	41 Yakubu Gowon Way, Old Airport Road
3734	PL/0143	Ginza Medical Centre	38A, Tafawa Balewa Street, Jos
3735	PL/0148	Mandela Klinik	Beside Police Station K/Vom
3736	PL/0150	Bingham University Teaching Hospital, Jos	23 Zaria By Pass, Jos
3737	PL/0153	Graceville Royal Hospitals	23T Unity Lane, Dadin Kowa, Jos
3738	PL/0157	Gaccon Hospital &Maternity	2 Alheri Police Station, Jos, Plateau State.

3739	PL/0158	Dee Medical Centre	Plot BP 1669/21 Bukuru Express way, Off Kugiya Road, Jos, Plateau State.
3740	PL/0159	Fertile Ground Hospital	Plot 1234, Zaramaganda, Jos, Adjacent Golden Bread, Plateau State.
3741	PL/0161	Marysol Specialist Hospital	Kwang Delimi, Off Rayfield Resort Road, Jos, Plateau State.
3742	PL/0162	Rayfield Medical Service LTD	30, Raphael Davon Street, Rayfield Jos, Plateau State.
3743	PL/0163	Adventist Health International	Seventh Day Adventist Hospital, Jengre, Plateau State.
3744	PL/0164	Oasis Medical Centre Limited	No. 47, Yakubu Gowon Way, Jos.
3745	PL/0165	Mashiah Foundation Clinic	Behind JMDB Qtrs Tundu Wada, Jos, Plateau State.
3746	PL/0166	Cottage Hospital Dyeroil	Dyeroil, Plateau State.
3747	PL/0167	Parkview Family Medicine Royal Hospital	By Solomon Lar Amusement Park, Domkat Bali Way, Jos, Plateau State
3748	PL/0168	Kauna Health Clinic & Maternity	Opp Tudun Wada Police Station, Jos, Plateau State.
3749	PL/0169	Hope Hill Specialist Medical Centre	No. 7C, Hospital Place, Opp. Four Square Gospel Church, Along Green Valley Hotel, Jos
3750	PL/0171	Al Noor Clinic & Maternity	5, Madara Junction Angwan Rogo Jos Plateau State
3751	PL/0172	Abnira Medical Centre	Plot 10078 Hospital place, behind Plateau Hospital, Jos
3752	PL/0173	Cottage Hospital, Kwalla	P.O Box 644, Kwalla Qua’anpan, Jos
3753	PL/0174	General Hospital	Mangu,Pankshin Road
3754	PL/0175	Wase Cottage Hopital	Wase Town, Garkawa Rd
3755	PL/0176	General Hospital	Angware
3756	PL/0178	General Hospital, Dengi	Jarmai Rd, Dengi
3757	PL/0179	General Hospital, Tunkus	Tunkus Jarmai Rd,Dengi
3758	PL/0180	General Hospital Jarmai Rd, Tunkus	Tunkus
3759	RV/0001	Olivet Clinic	18 Ede Street, Ogbunabali Street
3760	RV/0002	Olunwa Medical Centre	4 Amadi Ama Road, Trans-Amadi Layout (Rain-Bow Town)
3761	RV/0003	Maryland Health Care	No.1 Igwuruta Road, Rumuokurushi
3762	RV/0004	Springs Hospital	458, Ikwere Road by Kala B/Stop, Mile 5, Diobu
3763	RV/0005	Sophia Clinic	27 Old Aba Road, Rumuogba, Port Harcourt
3764	RV/0006	Ropheka Specialist Clinic	39, Elelenwo Street, Rumueme, P/Harcourt
3765	RV/0007	Anchor Hospital	km 15 P/H - Aba Expressway

3766	RV/0008	Princess Medical Centre	No.7 National Supply Road, Canco/Bewac Junction, Trans-Amadi Industrial Layout, P/Harcourt
3767	RV/0009	St. Jude	40 Elelenwo Road (Old Refinery Road) Oil Mill Junction, Rumukwurushi, PH
3768	RV/0010	New Orlins Clinic	9 Orazi Road, Rumola
3769	RV/0011	Harley Clinic	21B Akaninwor Road, Oroazi, Port Harcourt
3770	RV/0012	Fanny Royal Hospital	53 Market Road
3771	RV/0013	Family Medical Centre	39 Elelewon Road, Rumuokwurushi
3772	RV/0014	Tim-Grace Clinic & Maternity	25 Elelewon Road, Off Oil Mil Junction
3773	RV/0015	Eddy Medical Centre	4, Ndoki Lane, Behind 53 Market Road, Rumuomasi, P/H
3774	RV/0016	Queens Clinic	38 Rumuolumini Rd Wimpy Junction
3775	RV/0017	Ebony Hospital	27 Old Aba Road, Rumuogba -Odio
3776	RV/0019	Siya Medical Centre	112, Old Aba Road, Rumuobiakani, Port Harcourt
3777	RV/0020	St. Jude's Medical Clinic & Maternity	4, Bimkol Crescent, G.R.A. Phase Iii
3778	RV/0037	Fanny Royal Hospital	4, Bimkol Crescent G.R.A. Phase III
3779	RV/0038	Aggrey Clinic Ltd.	107 Aggrey Road Rivers
3780	RV/0039	Odessa Med. Services Ltd.	5 Royal Paragon Hotel Avenue, Iwofe Road, Rivers
3781	RV/0040	Meridian Hospitals	21, Ibokwe Street, D.Line
3782	RV/0042	Ponyx Clinic	2, Chuku Olunda Street, Off Anon Lodge, by Waterline Junction, Rumukalagbor
3783	RV/0043	Hossana Clinic/ Maternity	33, Abel Jumbo St. Mile 2, P/Harcourt
3784	RV/0044	Destiny Hospital & Maternity	38, Ihediohama Diobu Mile 2, P/Harcourt
3785	RV/0045	Calvary Clinic	7a, Haardy U. Street, Orazi, P/Harcourt
3786	RV/0046	Mercy Clinic & Mat.	No.1b Mercy Crescent, G.R.A. Phase 4, Behind Liberation Sati
3787	RV/0047	University Of Port Harcourt Teaching Hospital	Port Harcourt
3788	RV/0048	Braithwaite Memorial Hospital	Port Harcourt
3789	RV/0049	Ashford And Patrice Clinic Ltd.	38 Manila Pepple D. Line, Ph, Rivers State.
3790	RV/0050	Providence Clinic & Maternity	11 Railway Close, D-Line Ph.
3791	RV/0051	Vita Medical Centre	27, Nsukka Street, Mile1 Diobu,
3792	RV/0052	Seaside Specialist Surgery	89, VictorialStreet

3793	RV/0054	Nobsams Clinics	44 Trans Amadi Industrial Layout, Oginigba
3794	RV/0079	Christ The King Hospital	145 Old Aba/P.H Road, Mbano Camp, Oyigbo, Rivers State
3795	RV/0080	St. Mary's Hospital & Maternity	5, Estate Road, Woji
3796	RV/0081	Teme Hospital Ltd	10, New Hospital Lane
3797	RV/0082	Triumph Hospital	2, Estate Road Off Aba Road, Rumuogba
3798	RV/0084	Kez Clinic	16, Chief Wopara Street, Rumuomasi
3799	RV/0085	Empire Specialist Clinic & Trauma	19, Echinwo, Elekahia
3800	RV/0086	Alma Hospital	15, Wani Street, Oroworukwo
3801	RV/0087	Acron Medical Consultants	1, Acron Drive, Behind Plot 28, Stadium Rd, GRA Phase IV, Port Harcourt
3802	RV/0088	First Rivers Hospital	250a Ph/Aba Expressway
3803	RV/0089	Sonabel Medical Centre	14 Owabie Road, Mile 4 Rumueme
3804	RV/0090	New Mile One Hospital	15 Emenike Street, Mile 1 Diobu
3805	RV/0091	Valentine Hospitals	Plot B2, 0pp. 20 Forces Av. Old Gra
3806	RV/0092	Rivon Clinic	Aba/Ph Expressway, Genesis Junction
3807	RV/0093	St. Catherines Specialist Clinic	17b Ahiamakara Road, By Nwaya Junction, Trans-Amadi
3808	RV/0094	Pristine Med. Consultant	7 Nwviuke Rd., Off 125 Okporo Rd., Rumuogba
3809	RV/0095	Sapiens Clinic	2a Mini Ezekwu Street, Off Okporo Rd., Rumuogba
3810	RV/0096	St. Patrick Hospital	3 Ogbunabali Street
3811	RV/0097	Alphonso Hospital	Amadi Bus Stop, Along Refinery Rd., Elelenwon
3812	RV/0098	Mount Hermon Medical Centre	Plot 12 NTA/Choba Road near NTA Station, Port Harcourt
3813	RV/0099	Morning Star Hospital	19 Isiokpo Street, D/Line
3814	RV/0100	Caprin Hospital	4, Igwuruta Road, Eneka, Port Harcourt
3815	RV/0101	Woji Cottage Hospital	Accon Road, Woji Town, Rivers State
3816	RV/0102	Palmers Hospital & Mat. Home	5/7 Agip Road Rumueme
3817	RV/0103	Cresthill Medical Centre	2a Uyo Street Rumueomasi
3818	RV/0104	St. Martins Hospital	21 Stadium Road Port Harcourt
3819	RV/0106	Halten Clinic	11, Agudama Street, D/Line
3820	RV/0108	Carmel Clinic	3, Eleworks Drive, Rumuibekwe
3821	RV/0110	Ansett Diagnostic Hospital Limited	9, New Layout Drive, Oginigba Town, Trans-Amadi Industrial Layout
3822	RV/0209	Hopeville Specialist Hospital	Elijbolo-Eneka Road, Off New Airport Road, Eliozu
3823	RV/0210	Pamo Clinics & Hospital Ltd.	300 Aba Rd., Rumuomasi

3824	RV/0211	Bosom Hospital	6, Akaniwo St, Off Eligbam Rd.
3825	RV/0212	Cumi Medical Centre	3, Endless Road, Off Elitor Rd, Woji
3826	RV/0213	Marina Hospital	16 Akwa St, Off 35 Aba Rd, By Savannah Bank
3827	RV/0214	Lifetime Medical Centre	1A Worukwo St, Waterline, off Olusegun Obasanjo Rd.
3828	RV/0215	Immanuel Clinic	4, Nkanwa Lane, off NTA Rd Rumnokuta
3829	RV/0216	Jeconiah Children's Clinic	4, Okoroji Lane, D/Line
3830	RV/0218	Nigeria Customs Service Med. Centre	Nigeria Customs Service Medical Centre Port Harcourt-1,Rivers State
3831	RV/0219	Nigeria Customs Service Clinic	Onne Port II, Port Harcourt
3832	RV/0220	Military Hospital, P/Harcourt	Port Harcourt, Rivers State
3833	RV/0221	97 SOG Medical Centre	NAF Base Port Harcourt
3834	RV/0224	Garrison Clinics	10, Udom Street, D-Line, Port Harcourt.
3835	RV/0226	Castle Clinics	7B Shell Location Road, Rumuadaolu, Off 42 Rumuola Rd., P/H
3836	RV/0233	2 Bde MC Port Harcourt	Port Harcourt, Rivers State
3837	RV/0234	2 Bn MRS Port Harcourt	Port Harcourt, Rivers State
3838	RV/0235	73 Bn MRS Elele	Elele, Rivers State
3839	RV/0236	343 AR MRS Elele	Elele, Rivers State
3840	RV/0237	Cottage Hospital/Comprehensive Health Centre	Rivers State Police Command, Rivers State
3841	RV/0238	Navy Medical Centre - Borokin	Borokin, Port Harcourt, Rivers State
3842	RV/0239	Navy Medical Centre - Onne	Onne, Port Harcourt, Rivers State
3843	RV/0240	Sick bay NNS PATHFINDER	Port Harcourt, Rivers State
3844	RV/0241	Precious Life Medical Centre	4 Okorodo street D/line
3845	RV/0242	Sondel Clinic And Maternity	Plot 12 Phase 1 Borikiri Residential layout
3846	RV/0243	St Michael's Clinic	4th Street, Block 4 Elekaha Housing Estate
3847	RV/0244	Divine Grace Clinic/Maternity	21 Elijiji Road, Off Woji Road , P/Harcourt
3848	RV/0245	Udo PolyClinics	9/10 Chief Ejim''''s Street Rumubiakani P/H
3849	RV/0246	Datta Medical Centre	21A Woji Road, Off Old Aba Road, P/H
3850	RV/0247	Higgwe Memorial Hospital	644 Ikwerre Road Rumuokoro P/H
3851	RV/0249	Jehovah Raphas's Clinic	7 Elelenwo Street, Off market Road, Rumuomasi
3852	RV/0250	Eli Johnson Specialist Hospital	13 Bori Road, Rumuibekwe Housing Estate, P/H
3853	RV/0251	Health Fountains Hospital	NTA/Uniport Road , Ozuoba, P/H
3854	RV/0252	The Riverside Clinic	21 Harbour Road , P/H
3855	RV/0253	Summit Clinic	17 Omoku Street, D/line, P/H

3856	RV/0254	Daily Spring Hospital Ltd	11 Rumuigbo Street, Old GRA, P/H
3857	RV/0255	Silvia Specialist Clinic/Maternity	6 Circular Road, Phase 2 Presidential Estate
3858	RV/0256	El- Bene Hospital	5 El Bene Close, Off Ada George Road Rumuchiorlu Street, by Open Doors Church Junction, Rumueme, Mile 4, P/H
3859	RV/0257	Seiyefa Clinic	11 Harold Wilson Drive , Borikiri, P/H
3860	RV/0258	Heart Health Medical Services	14 Omoku Street, D/line, P/H
3861	RV/0260	Hope Clinics	3/4 Wokogoloma Street, Waterline, P/H
3862	RV/0261	Krisany Medical Services	5B Asinobi/Agudama Street, D/line, P/H
3863	RV/0262	Metroplitan Clinic	2nd Avenue, Federal Housing Estate Mile 4, Agip, Rumueme
3864	RV/0263	Atinu Critical Care Hospital	Old Refinery Road , P/H
3865	RV/0264	Sylverlene Clinic And Maternity	80D Igwuruta Road ,East/West Road Junction Opp Tank, Rumukwurushi
3866	RV/0265	Promise Land Clinic	4 Bende Street, Rumuomas, i P/H
3867	RV/0267	Salem Hospital	16 Location Road, Oyigbo, P/H
3868	RV/0350	DSS Clinic	Port Harcourt, Rivers State
3869	RV/0351	CRI Medi Clinic	Plot 80A Second Avenue, Along Dr. Peter Odili Street, P/Harcourt
3870	RV/0352	Omoku Clinic	Fed. Col. of Education Technical, River State
3871	RV/0353	Bethsaida Clinic & Maternity	21, Pipeline Satelite Town, Oyibo, Rivers State
3872	RV/0354	Gbeye Clinic & Maternity	16 Ahoada road Omoku
3873	RV/0355	Gbeye Clinic & Maternity	36 Umuzhi street, Ahoada
3874	RV/0356	Life Care Medical Centre	3 Azikiwe street Mile 11 Diobu P/ Harcourt
3875	RV/0357	Life Clinics Medical Centre	15 Uyo street, Mile 1 Diobu P/ Harcourt
3876	RV/0358	Pamax Hospital Ltd	Isiokpo
3877	RV/0359	Rehoboth Specialist Hospital	2 Winners'''' way off Afam str,D-line P/Harcourt
3878	RV/0360	Ridcol Clinic & Maternity	26 Emekuku str, D- line P/Harcourt
3879	RV/0361	TRUVINE SPECIALIST CLINIC	6 OHIAMINI RD, BEHIND COLL OF ARTS & SCI OFF R/OLA P/HARCOURT
3880	RV/0372	Family Care Clinic	15 Potts Johnson Street, Port Harcourt
3881	RV/0376	SHAMAKOP HOSPITAL & MEDICAL CONSULTANTS	5B ABEOKUTA STREET D LINE PORT HARCOURT

3882	RV/0377	Nigerian Navy Ship Yard	Port Harcourt
3883	RV/0378	The Shield Clinics	Plot 4 Close D, Peace Estate, Trans Woji Road, Port Harcourt
3884	RV/0381	Adanta Children`s Hospital	44 Evo Road GRA Phase2, Port Harcourt Rivers State
3885	RV/0382	Aviation Medical Clinics	Port Harcourt Airport, Port Harcourt River State
3886	RV/0383	Nedal Specialist Clinic	Rumuekini/Rumuosi Junction-Along East West Road Akpor Rivers State
3887	RV/0385	Railway Clinic	NRC ,Port Harcourt Rivers State
3888	RV/0386	Harryland Medical Centre	No 4,St.Mark`s Rd ,Off U.B.E Junction, Borikiri Sandfill Area, Port Harcourt, River State
3889	RV/0387	University of Port Harcourt (Lulu Briggs Health Centre)	University of Port Harcourt
3890	RV/0388	Life Line Clinic	30 Rumunduru RD, Eliowhani Kingdom ,Port Harcourt Rivers State
3891	RV/0389	Paragon Clinics And Imaging	96 Stadium Road, Port Harcourt River State
3892	RV/0390	Noble Medical Consultants &Fertility Hospital	No.1 Bloom breed School Road Mgbuoba Opp. NTA Gate Rivers State
3893	RV/0391	Health Of The Sick Hospital	65/67 Nkpogu RD, Trans Amadi, Port Harcourt River State
3894	RV/0392	Kendox Medical Services	No 7,Moses Chinda Avenue, Off 194 Elelenwo Port Harcourt Rivers State
3895	RV/0393	St. Mary`s Clinic	Lane 2,Block 4,Aggrey Rd Housing Est. Port Harcourt River State
3896	RV/0394	Goshen Clinic And Maternity	Close 8,No 8, Off Circular Road Elekahia Housing Estate, Port Harcourt
3897	RV/0395	Jose Vital Hospital Limited	Plot 19,East West RD, By Rumuokoro Near St. Jude Catholic Church, Port Harcourt
3898	RV/0396	Foundation Clinic	No 1,Foundation Clinic Off Palace Road Omoku Rivers State
3899	RV/0397	Corban Medical LTD	Echichinwo RD/Elekaha Estate Opp. Gov`t.
3900	RV/0398	Danferd Specialist Clinic	246,Ada George Road, Opposite Romans Filling Station By Okuton Junction River State
3901	RV/0399	Gold Trust Clinics	2,Chief Benson Street Off Rumuola Road, Port Harcourt Rivers State
3902	RV/0400	Shammar Christian Hospital	Plot 16 Block 33 Woji Estate, Port Harcourt

3903	RV/0401	Chiji-Man Specialist Hospital	10,Ovim Street, Mile 3 Diobu, Port Harcourt River State
3904	RV/0402	Atlantic Medical Center	76c Emekuku Street/Line, Port Harcourt, Rivers State
3905	RV/0403	Spring Rose Hospital	No.22 Rumuagbola Road Port Harcourt River State
3906	RV/0404	El-Joe Dam Hospital	Off Shell Location Road, Mgbuoba, By Four Square Gospel Church Port Harcourt Rivers State
3907	RV/0405	Krystal Clinics	8 Jetty Road Abuloma Rivers State
3908	RV/0406	St. Anthony`s J.C Clinic	42,School Road Elelenwo Girls Secondary School),Elelenwo, Port Harcourt
3909	RV/0407	Life Forte Specialist Hospital	3,Oluwosu Street, Off Ude Road Rumuollumeni, Port Harcourt Rivers State
3910	RV/0408	Hilton Clinics	No 2 Ejekwu Willie Close, Opp. Silver Spoon Hotel Ada George By Wimpey Road, Mile 4,Port Harcourt Rivers State
3911	RV/0409	Pearl Clinics & Maternity	4,Manilla Pepple Street, D/Line, Port Harcourt River State
3912	RV/0410	Okpeden Medical Center	12 Omoku Road, Ahoada Rivers State
3913	RV/0411	Phenix Clinics	21 Nnewi (Kolo) Street Off Abakiliki Road By Educational B/Stop Mile 1 Diobu, Port Harcourt Rivers State
3914	RV/0412	Ada Clinic	Plot 64B Circular Road, Federal Housing Estate, Trans Amadi Port Harcourt Rivers State
3915	RV/0413	Divine Grace Hospital LTD	28B,Seaside Rd, Oyigbo, Port Harcourt Rivers State
3916	RV/0414	Health Wise Hospital	No 3,Benin Street-Line Off 27 Aba Road By Oando Filling Station, Port Harcourt Rivers State
3917	RV/0424	Grace And Mercy Clinic LTD	25B, Ilom Street, Off Woji Rd., Port Harcourt, Rivers State
3918	RV/0427	Cedar Trust Model Clinics & Maternity	33,Eneka-Igbo-Etchee Road,Off 1st Market R/About, Enaka Port-Harcourt Rivers State
3919	RV/0428	Military Hospital Port-Harcourt	Aba Road Port-Harcourt, Rivers state
3920	RV/0429	Phoenix Medical Consult	3, Igboukwu Close, By 1st Artillery Bus Stop ,Aba Road, Port- Harcourt
3921	RV/0430	Cova Care Ltd	8, Main Street, Off Farm Road 2, Off G.U Ake Road, Eliozu. Rivers State

3922	RV/0431	Sophike Medical Centre	Plot 9, Road 7, Federal Housing Estate, Off Peter Odili Road, Ports-Harcourt. Rivers State
3923	RV/0433	Wulzi Specialist Hospital	117, Airport Road, Igwuruta- Ali, Greater Port-Harcourt, Rivers State.
3924	RV/0434	Ell-Specialist Hospitals Limited	7, Umuechem Street, D/Line, Port-Harcourt. Rivers State
3925	RV/0435	Calvary Clinic	10, Miniokpobiri Street, Okpooro Road, By Mama B Junction, Port –
3926	RV/0436	Gbeye Hospital & Maternity	4, Destiny Street, Of Eliopranwo Road, Off Ada George Road, Port- Harcourt. Rivers State
3927	RV/0437	Mina's Urological/Specialist Hospital Ltd	House 25, Road 6, Freedom Estate, Rukpokwu, Port-Harcourt. Rivers State
3928	RV/0438	Nigerian Navy Basic Training School- Sick Bay	Nigerian Basic Training School, Onne, Rivers State
3929	RV/0439	Nimley Clinic	17, Churchill Road, Creek Road Extension, Port-Harcourt. Rivers State
3930	RV/0440	Bennod International Hospital Limited	28 Ada George Road, Port-Harcourt -. Rivers State
3931	RV/0441	Channel Clinic & Hospital Limited	10, Hospital Road, Bonny, Rivers State
3932	RV/0443	Magnum Nuvon Medical Centre Ltd	18, Canon Ndu Street, Elelenwo, Rivers State
3933	RV/0446	Federal Polytechnic Of Oil and Gas Health Centre Bonny	Abalamable Town, Bonny Island, Rivers State
3934	RV/0447	Eastlans Specialist Hospital Limited	4, Worlu Street, Rumuigbo, Port-Harcourt, Rivers State
3935	RV/0451	Abundant Health Specialist Ltd	9, Aleruchi Close, Off No.5 Ada George Road, Port-Harcourt Rivers State
3936	RV/0452	Greencare Medical Consultants Ltd	15, East West Worlu Road (Baba), Off Obiwali Road, Rumigbo, Port-Harcourt , Rivers State
3937	RV/0453	Save A Life Mission Hospital	38,Uyo St. Off Stadium Road, Rumuomasi, Port-Harcourt, Rivers State
3938	RV/0454	Richimok Hospital Limited	102,Nta/Choba Road, Port-Harcourt, Rivers State
3939	RV/0456	Ofure Specialist Clinic	Plot 65,Okporo Rd, Behind Eco bank, Off Artillery, Port-Harcourt, Rivers State
3940	RV/0457	Westhill Medical Services Ltd	4,Wogu Street ,Off Omoku Rd, D/Line Port-Harcourt Rivers State
3941	RV/0458	St. Vincent Hospital	1a,St.Vincent Close, Ogbogoro Road, Rumuoluneni, Port-Harcourt, Rivers State

3942	RV/0459	Althahaus Medical Centre	122c,General Driyai Street, GRA, Port-Harcourt, Rivers State
3943	RV/0460	Bethesda Family Hospital	Plot 18 (Opp. Apostolic Army Church) Eastern By-Pass, Port-Harcourt, Rivers State
3944	RV/0461	Beracca Eye Clinic Ltd	9,Rumuogba Estate, Rumuogba, Port-Harcourt ,Rivers State
3945	RV/0462	Ultimate Specialist Clinic & Hospital	67,Royal Avenue, Off Odili/Okuru/Abuloma Link Road, Port-Harcourt, Rivers State
3946	RV/0464	International Trauma & Critical Care Centre Ltd	11, Emenike Street, Mile One Port-Harcourt, Rivers State
3947	RV/0466	NNPC Medical Services	NNPC-Eleme/Moscow Road, Port-Harcourt Rivers State
3948	SO/0002	Sokoto Clinic	Sokoto
3949	SO/0004	Shepherd Specialist Clinic	No. 32 Zaria Rd. Ung. Rogo Area
3950	SO/0005	Sahel Specialist Hospital	Sultan Abubakar Rd. Sokoto
3951	SO/0006	Specialist Hospital	Abdullahi Fodiyo Road, Sokoto
3952	SO/0007	Hussaini Medical Center	Emir Yahaya Rd. Sokoto
3953	SO/0008	Lafiya Clinic & Health Services	25, Shuni Road, Mararaba, Sokoto
3954	SO/0009	Karaye Hospital	Ali Akilu Rd. Sokoto
3955	SO/0010	Usman Danfodio University Teaching Hospital	Behind Standard Trust Bank, Kano Rd. Sokoto
3956	SO/0011	Al-Fijir Specialist Medical Center	No. 32 Zaria Road, Unguwan Rogo Area
3957	SO/0031	1 Bde MC, Sokoto	Sokoto, Sokoto State
3958	SO/0033	Free Hand Specialist Hospital	31, Shuni Rd, Sokoto
3959	SO/0037	Usman Danfodio University Health Services	University main campus, Sokoto
3960	SO/0038	DSS Clinic	Sokoto, Sokoto State
3961	SO/0039	Hamdala Medical Centre	47 Emir Yahaya Road, Sokoto
3962	SO/0040	Liberty Clinic	11, Gummi Road, Hajiya Halima Estate, Sokoto
3963	SO/0041	Police Clinic	Sokoto
3964	SO/0042	Al-Umma Hospital	Aliyu Gida Road, Sokoto South
3965	SO/0043	General Hospital, Kebbe	Kebbe
3966	SO/0044	Primary Health Care Centre, Kuchi	Kebbe
3967	SO/0045	Primary Health Clinic, Sabon Birnin	Sabon Birnin
3968	SO/0046	Sangerawa Dispensary	Sabon Birnin
3969	SO/0047	Tsamaye Dispensary	Sabon Birnin
3970	SO/0048	Unguwar Lalle Upgrade Dispensary	Sabon Birnin
3971	SO/0049	Laginge Dispensary	Sabon Birnin
3972	SO/0050	Makuwana Dispensary	Sabon Birnin
3973	SO/0051	Teke Dispensary	Sabon Birnin
3974	SO/0052	Kwatsal Dispensary	Sabon Birnin
3975	SO/0053	Gatawa Model Primary Health Clinic	Sabon Birnin
3976	SO/0054	Kalgo Dispensary	Sabon Birnin
3977	SO/0055	Tara Dispensary	Sabon Birnin
3978	SO/0056	Kunawa Dispensary	Sabon Birnin
3979	SO/0057	Magarau Dispensary	Sabon Birnin

3980	SO/0058	Tagirke Dispensary	Sabon Birnin
3981	SO/0059	Rambadawa Dispensary	Sabon Birnin
3982	SO/0060	Kyara Dispensary	Sabon Birnin
3983	SO/0061	Bachaka Dispensary	Sabon Birnin
3984	SO/0062	Dankura Dispensary	Sabon Birnin
3985	SO/0063	Dama Dispensary	Sabon Birnin
3986	SO/0064	Kalage Dispensary	Sabon Birnin
3987	SO/0065	Yarbulutu Dispensary	Sabon Birnin
3988	SO/0066	Kiratatawa Dispensary	Sabon Birnin
3989	SO/0067	Garinidi Dispensary	Sabon Birnin
3990	SO/0068	Karuwa Dispensary	Sabon Birnin
3991	SO/0069	Burkusima Dispensary	Sabon Birnin
3992	SO/0070	Gangara Dispensary	Sabon Birnin
3993	SO/0071	Magira Dispensary	Sabon Birnin
3994	SO/0072	An-Nisha Clinic	2, Garba Mohammed Rd, opp School of Works, Runjin Sabo, Sokoto
3995	SO/0073	Ubandoma Soecialist Clinic	# 24, Besse Road, Rujin Sambo Area, Sokoto
3996	SO/0075	State Specialist Hospital	Sultan Abubakar Road, Sokoto, Sokoto State
3997	SO/0077	Zefafi Women Specialist Hospital	No. 1 Maikahon Karo Road Beside Giginya Stadium Sokoto, Sokoto State
3998	SO/0078	Iman Hospital	No. 13 Dankoro Road Runjin Sambo Sokoto, Sokoto State
3999	SO/0079	Wali Bako Specialist Medical Center	2 Dange Road, Behind GGC, Sokoto
4000	SO/0080	Caliphate Multispecialty Hospital	No. 2 Nagwamatse Road, Sokoto, Sokoto State.
4001	SO/0082	Alheri Medical And Consultancy Clinics	Federal Housing Estate Sokoto, Sokoto State.
4002	TR/0001	Jinya Medical Centre	3c Gombe Road, Jalingo
4003	TR/0003	Biyama Hospital	Donga Road, Wukari
4004	TR/0004	El-Duniya Clinic and Convalescent Limited	14, Borno Road, Near State Low Cost, Jalingo
4005	TR/0005	Gateway Hospital	No. 3c Gombi Road, Near State Low Cost, Jalingo
4006	TR/0006	Courage Hospital, Mayo Renowo Crescent	Mayo Renowo Crescent, Behind State Legislative Qtrs,Jalingo
4007	TR/0007	General Hospital, Wukari	Wukari
4008	TR/0008	Gongola Hospital	Old Bantaje Road, Wukari
4009	TR/0009	Federal Medical Centre - Jalingo	No. 29 Idris Makeri Street, Jalingo
4010	TR/0010	Rafu Clinic	Katsina Ala Road, Wukari
4011	TR/0011	Bethel Hospital	Ibi Road, GRA, Wukari
4012	TR/0012	First Referral Hospital	Mutum Biyu
4013	TR/0013	General Hospital, Zing	Bitaka Road, Zing
4014	TR/0014	Abdul Medical Centre	No. 5 Donga Road, Jalingo
4015	TR/0025	3 Bn MRS Takum	Takum, Taraba State
4016	TR/0026	Rapha Hospital	Katsina-ala Road, Takum

4017	TR/0028	Jonas clinics & Maternity	No 1, Old Garage Round-about, Takum
4018	TR/0029	Niger Clinic	Opp water Board, Yakum
4019	TR/0034	DSS Clinic	Jalingo, Taraba State
4020	TR/0035	Police Clinics, Taraba	Taraba State
4021	TR/0037	General Hospital, Bali	Bali, Taraba State
4022	TR/0038	General Hospital, Gembu	Gembu, Taraba State
4023	TR/0039	20 BN MRS, Jalingo	Serti
4024	TR/0041	Totus Hospital & Maternity Limited	Along Jalingo/Wukari Road near UBA, Mutum Biyu
4025	TR/0043	Kwararafa Hospital & Maternity	8, After Zion Church, Along Air Strip, Wukari
4026	TR/0044	Danny's Wellness Clinics Ltd	Presidential Lodge Area, Specialist Hospital Road, Jalingo
4027	TR/0045	Taraba Specialist Hospital	1, Jolly Nyame Way, Off By-Pass, Jalingo Taraba state
4028	TR/0046	Sauki Hospital & Maternity	NO. 8, Near Zion Baptist Church Sabon Gari, Jalingo, Taraba State.
4029	TR/0047	First Referral Hospital.	Serti Taraba State
4030	TR/0048	University Health Services Centre	Federal University Wukari, Taraba State
4031	TR/0049	First Nasarawo Hospital	Sintali B Jalingo
4032	TR/0050	Kwenas Clinic & Maternity	Near Old Garage, Wukari
4033	TR/0051	Federal Polytechnic Bali Medical Centre	Federal Polytechnic Bali
4034	TR/0052	College Of Agriculture, Jalingo Medical Hospital	College Of Agriculture Jalingo, Taraba State
4035	TR/0054	Maitala Trust Medical Services Ltd	Off Former Jankada Hospital Jalingo Taraba State
4036	TR/0055	Waritoma - Rahila Hospital	Opp. Shagari Low Cost, Along Takum Road, Wukari Taraba State
4037	TR/0056	Goggoji Memorial Clinic	Behind Federal Science T. College Jalingo, Taraba State
4038	TR/0057	Taraba State Polytechnic Campus Clinic	Jalingo-Wukari Road, Jalingo, Taraba State.
4039	TR/0058	General Hospital Takum	Takum, Taraba State
4040	TR/0059	Zahra Clinic	AdjacentMastala Adamawa Street Jalingo, Taraba State.
4041	YB/0001	Bade Medical Clinic	Sabon Gari Ward, Gashua
4042	YB/0002	General Hospital, Gashua	Gashua,Along Nguru Rd., Gashua
4043	YB/0004	General Sani Abacha Specialist Hospital	Along Gujba Rd., Damaturu
4044	YB/0005	Yobe Medical Center	No.13 Gashua Road, Damaturu
4045	YB/0006	Borno Medical Center	Along Gashua Road, Damaturu
4046	YB/0007	Dr(Mrs) Maryam Abacha Fsp	Gashua Rd., Damaturu
4047	YB/0013	General Hospital Dapchi	Dapchi, Ajari Ward
4048	YB/0016	General Hospital Buni-Yadi	Along Biu Road, Buni-Yadi
4049	YB/0017	Federal Medical Center, Nguru	Along Gashua Rd.
4050	YB/0019	General Hospital Potiskum	Along Hospital Road
4051	YB/0020	Ajiko Medical Center	Along Gashua Rd
4052	YB/0021	General Hospital, Geidam	Kaigama Road, Geidam

4053	YB/0022	Potiskum Medical Clinic	No. 166 Mohammed Idriss Way, Potiskum
4054	YB/0026	241 Recce Bn MRS Nguru	Nguru, Yobe State
4055	YB/0030	DSS Clinic	Damaturu, Yobe State
4056	YB/0031	Police Clinics, Yobe	Yobe State
4057	YB/0032	Federal College Of Education, Potiskum	Potiskum, Yobe State
4058	YB/0033	Federal Polytechnic Clinic	Damaturu, Yobe State
4059	YB/0039	Eva Clinic & Maternity	160 Mohammed Idris Way, Potiskum
4060	YB/0040	City Medical Centre	Zango Ward, Damaturu
4061	YB/0042	Afua Medical Clinic & Maternity	Boni Yadi along Biu Road
4062	YB/0044	General Hospital Damagum	Potiskum Road Adjacent Fune LGA Secretariat, Damagun, Yobe State
4063	ZF/0001	General Hospital, Anka	Anka
4064	ZF/0002	General Hospital, Bakura	Bakura
4065	ZF/0004	Polyclinics & Maternity	Hospital Zaria Road, Samaru
4066	ZF/0005	Gusau General Hospital - Gusau	Mortage Area, Gusau
4067	ZF/0006	Daula Hospital & Mat. Home	K/Namoda Road, Lebin-Lebin
4068	ZF/0007	Women & Children Specialist Hospital	Gusau
4069	ZF/0008	Arewa Hospital	Sani Abacha Way
4070	ZF/0009	Gusau Medical Clinic	Opp. Govt. House
4071	ZF/0017	General Hospital, Kaura Namoda	Kaura Namoda
4072	ZF/0018	General Hospital, Maru	Maru
4073	ZF/0019	General Hospital, Talata Mafara	Talata Mafara
4074	ZF/0020	Tsafe General Hospital	Hospital Tsafe
4075	ZF/0021	Federal Medical Centre Gusau	Gusau
4076	ZF/0025	DSS Clinic	Gusau, Zamfara State
4077	ZF/0026	Police Clinics, Zamfara	Zamfara State
4078	ZF/0027	Federal Polytechnic, Kaura Namoda	Kaura Namoda, Zamfara State
4079	ZF/0028	1 BAD	Gusau
4080	ZF/0029	Yariman Bakura Specialist Hospital	Kawa Namoda Road, Tudun Wada, Gusau, Zamfara State
4081	ZF/0030	Haske Family Hospital Gusau	Zaria Road, Along Gusau Hotel Gusau, Zamfara State
4082	ZF/0031	Hilal Specialist Hospital	12, Olusegun Obasanjo Drive Gusau, Zamfara State
4083	ZF/0032	Federal University Gusau Medical Center	Federal University Gusau Campus, Zamfara State

LGA	STATE
Aba South	Abia
Aba South	Abia
Aba South	Abia
Aba South	Abia
Aba South	Abia
Aba South	Abia
Aba South	Abia
Aba North	Abia
Aba North	Abia
Aba North	Abia
Umuahia South	Abia
Aba South	Abia
Aba South	Abia
Obingwa	Abia
Aba North	Abia
Aba South	Abia
Obingwa	Abia
Aba North	Abia
Aba North	Abia
Aba North	Abia
Aba North	Abia
Aba South	Abia
Aba North	Abia
Aba South	Abia
Umuahia North	Abia
Umuahia North	Abia
Aba South	Abia
Aba North	Abia
Aba North	Abia
Aba North	Abia
Aba North	Abia
Aba South	Abia
Aba South	Abia
Umuahia South	Abia
Umuahia North	Abia
Umuahia North	Abia

Osisioma	Abia
Aba North	Abia
Umuahia North	Abia
Ukwa West	Abia
Aba North	Abia
Aba South	Abia
Umuahia North	Abia
Aba North	Abia
Umuahia North	Abia
Umuahia South	Abia
Umuahia North	Abia
Umuahia North	Abia
Umuahia North	Abia
Aba South	Abia
Ohafia	Abia
Aba South	Abia
Ohafia	Abia
Aba North	Abia
Aba North	Abia
Aba North	Abia
Ukwa East	Abia
Obingwa	Abia
Umuahia North	Abia
Aba North	Abia
Umuahia North	Abia
Osisioma	Abia
Aba South	Abia
Ohafia	Abia
Aba South	Abia
Osisioma	Abia

Umuahia North	Abia
Umuahia South	Abia
Aba North	Abia
Umuahia North	Abia
Aba North	Abia
Umu - Nneochi	Abia
Osisioma	Abia
Isuikwuato	Abia
Osisioma	Abia
Umuahia North	Abia
Aba South	Abia
Aba South	Abia
Ganye	Adamawa
Mayo - Belwa	Adamawa
Mubi South	Adamawa
Numan	Adamawa
Song	Adamawa
Yola North	Adamawa
Yola North	Adamawa
Yola North	Adamawa
Yola North	Adamawa
Yola North	Adamawa
Yola North	Adamawa
Yola North	Adamawa
Yola North	Adamawa
Yola North	Adamawa
Yola North	Adamawa
Yola North	Adamawa
Yola North	Adamawa
Yola North	Adamawa
Yola South	Adamawa
Yola South	Adamawa
Gombi	Adamawa
Yola South	Adamawa
Mubi South	Adamawa

Mubi North	Adamawa
Gombi	Adamawa
Hong	Adamawa
Guyuk	Adamawa
Demsa	Adamawa
Yola North	Adamawa
Michika	Adamawa
Fufore	Adamawa
Yola North	Adamawa
Yola South	Adamawa
Yola North	Adamawa
Mubi North	Adamawa
Yola North	Adamawa
Yola North	Adamawa
Yola North	Adamawa
Yola North	Adamawa
Madagali	Adamawa
Mubi South	Adamawa
Yola North	Adamawa
Mubi South	Adamawa
Gombi	Adamawa
Yola North	Adamawa
Mubi North	Adamawa
Yola North	Adamawa
Mayo - Belwa	Adamawa
Yola North	Adamawa
Yola North	Adamawa
Yola South	Adamawa
Yola South	Adamawa
Yola South	Adamawa
Numan	Adamawa
Yola North	Adamawa
Yola North	Adamawa
Eket	Akwa Ibom
Eket	Akwa Ibom
Ikot Ekpene	Akwa Ibom
Oron	Akwa Ibom
Uyo	Akwa Ibom

Uyo	Akwa Ibom
Uyo	Akwa Ibom
Uyo	Akwa Ibom
Uyo	Akwa Ibom
Uyo	Akwa Ibom
Uyo	Akwa Ibom
Uyo	Akwa Ibom
Abak	Akwa Ibom
Uyo	Akwa Ibom
Uyo	Akwa Ibom
Uyo	Akwa Ibom
Uyo	Akwa Ibom
Uyo	Akwa Ibom
Uyo	Akwa Ibom
Uyo	Akwa Ibom
Abak	Akwa Ibom
Uyo	Akwa Ibom
Uyo	Akwa Ibom
Uyo	Akwa Ibom
Uyo	Akwa Ibom
Uyo	Akwa Ibom
Uyo	Akwa Ibom
Eket	Akwa Ibom
Eket	Akwa Ibom
Uyo	Akwa Ibom
Uyo	Akwa Ibom
Abak	Akwa Ibom
Uyo	Akwa Ibom
Uyo	Akwa Ibom
Oron	Akwa Ibom
Uyo	Akwa Ibom
Ibiono Ibom	Akwa Ibom
Uyo	Akwa Ibom
Uyo	Akwa Ibom

Uyo	Akwa Ibom
Oron	Akwa Ibom
Uyo	Akwa Ibom
Uyo	Akwa Ibom
Ikot Ekpene	Akwa Ibom
Uyo	Akwa Ibom
Uyo	Akwa Ibom
Uyo	Akwa Ibom
Uyo	Akwa Ibom
Oron	Akwa Ibom
Uyo	Akwa Ibom
Uyo	Akwa Ibom
Uyo	Akwa Ibom
Essien Udim	Akwa Ibom
Uyo	Akwa Ibom
Eket	Akwa Ibom
Ikot Ekpene	Akwa Ibom
Eket	Akwa Ibom
Uyo	Akwa Ibom
Awka South	Anambra
Awka South	Anambra
Awka South	Anambra
Awka South	Anambra
Awka South	Anambra
Nnewi North	Anambra
Onitsha-North	Anambra

Onitsha-North	Anambra
Onitsha-North	Anambra
Onitsha-North	Anambra
Onitsha-North	Anambra
Onitsha -South	Anambra
Onitsha -South	Anambra
Onitsha -South	Anambra
Onitsha -South	Anambra
Idemili North	Anambra
Idemili North	Anambra
Idemili North	Anambra
Nnewi North	Anambra
Onitsha -South	Anambra
Onitsha-North	Anambra
Anambra East	Anambra
Awka South	Anambra
Onitsha-North	Anambra
Awka South	Anambra
Njikoka	Anambra
Orumba North	Anambra
Aguata	Anambra
Onitsha-North	Anambra
Orumba North	Anambra
Idemili North	Anambra
Nnewi North	Anambra
Nnewi North	Anambra
Nnewi North	Anambra

Nnewi North	Anambra
Anaocha	Anambra
Awka North	Anambra
Orumba South	Anambra
Awka North	Anambra
Orumba North	Anambra
Orumba South	Anambra
Awka South	Anambra
Aguata	Anambra
Idemili North	Anambra
Onitsha -South	Anambra
Aguata	Anambra
Onitsha -South	Anambra
Onitsha-North	Anambra
Idemili North	Anambra
Awka South	Anambra
Onitsha -South	Anambra
Awka South	Anambra
Aguata	Anambra
Onitsha -South	Anambra
Awka South	Anambra
Nnewi North	Anambra
Idemili North	Anambra
Orumba South	Anambra
Nnewi North	Anambra
Onitsha-North	Anambra
Idemili North	Anambra
Aguata	Anambra
Awka South	Anambra
Idemili North	Anambra
Onitsha -South	Anambra
Aguata	Anambra
Njikoka	Anambra
Aguata	Anambra
Ihala	Anambra
Aguata	Anambra

Awka North	Anambra
Onitsha -South	Anambra
Aguata	Anambra
Idemili North	Anambra
Nnewi North	Anambra
Awka South	Anambra
Onitsha -South	Anambra
Awka South	Anambra
Awka South	Anambra
Idemili-South	Anambra
Anaocha	Anambra
Aguata	Anambra
Njikoka	Anambra
Awka South	Anambra
Awka South	Anambra
Oyi	Anambra
Aguata	Anambra
Idemili North	Anambra
Ihala	Anambra
Nnewi North	Anambra
Awka South	Anambra
Awka South	Anambra
Njikoka	Anambra
Ogbaru	Anambra
Ogbaru	Anambra
Dunukofia	Anambra
Awka South	Anambra
Onitsha -South	Anambra

Njikoka	Anambra
Nnewi North	Anambra
Onitsha-North	Anambra
Anaocha	Anambra
Awka South	Anambra
Orumba South	Anambra
Anaocha	Anambra
Onitsha-North	Anambra
Aguata	Anambra
Awka North	Anambra
Orumba North	Anambra
Onitsha -South	Anambra
Dunukofia	Anambra
Ihala	Anambra
Onitsha-North	Anambra
Awka South	Anambra
Awka South	Anambra
Awka South	Anambra
Bauchi	Bauchi
Bauchi	Bauchi
Bauchi	Bauchi
Bauchi	Bauchi
Bauchi	Bauchi
Bauchi	Bauchi
Katagum	Bauchi
Katagum	Bauchi
Bauchi	Bauchi
Bauchi	Bauchi
Katagum	Bauchi
Bauchi	Bauchi
Bauchi	Bauchi

Bauchi	Bauchi
Bauchi	Bauchi
Bauchi	Bauchi
Bauchi	Bauchi
Bauchi	Bauchi
Bauchi	Bauchi
Bauchi	Bauchi
Bauchi	Bauchi
Ningi	Bauchi
Misau	Bauchi
Shira	Bauchi
Toro	Bauchi
Tafawa Balewa	Bauchi
Katagum	Bauchi
Darazo	Bauchi
Dass	Bauchi
Jama'Are	Bauchi
Gamawa	Bauchi
Alkaleri	Bauchi
Itas/Gadau	Bauchi
Bauchi	Bauchi
Alkaleri	Bauchi
Bauchi	Bauchi
Bauchi	Bauchi
Ningi	Bauchi
Ningi	Bauchi
Ganjuwa	Bauchi
Dambam	Bauchi
Yayu	Bauchi
Zaki	Bauchi
Giade	Bauchi
Shira	Bauchi
Katagum	Bauchi
Jama'Are	Bauchi
Bauchi	Bauchi
Bauchi	Bauchi
Ganjuwa	Bauchi
Ningi	Bauchi
Tafawa Balewa	Bauchi
Katagum	Bauchi
Bauchi	Bauchi
Katagum	Bauchi

Bauchi	Bauchi
Katagum	Bauchi
Bauchi	Bauchi
Bauchi	Bauchi
Bauchi	Bauchi
Ningi	Bauchi
Gboko	Benue
Gboko	Benue
Gboko	Benue
Makurdi	Benue
Makurdi	Benue
Makurdi	Benue
Makurdi	Benue
Makurdi	Benue
Makurdi	Benue
Makurdi	Benue
Makurdi	Benue
Makurdi	Benue
Makurdi	Benue
Makurdi	Benue
Makurdi	Benue
Makurdi	Benue
Makurdi	Benue
Makurdi	Benue
Otukpo	Benue
Ogbadibo	Benue
Otukpo	Benue
Makurdi	Benue
Makurdi	Benue
Makurdi	Benue
Makurdi	Benue
Makurdi	Benue
Makurdi	Benue
Makurdi	Benue

Makurdi	Benue
Makurdi	Benue
Makurdi	Benue
Makurdi	Benue
Makurdi	Benue
Makurdi	Benue
Okpokwu	Benue
Otukpo	Benue
Otukpo	Benue
Makurdi	Benue
Otukpo	Benue
Okpokwu	Benue
Okpokwu	Benue
Gboko	Benue
Gboko	Benue
Makurdi	Benue
Makurdi	Benue
Makurdi	Benue
Makurdi	Benue
Makurdi	Benue
Makurdi	Benue
Makurdi	Benue
Makurdi	Benue
Kwande	Benue
Gboko	Benue
Makurdi	Benue
Makurdi	Benue

Makurdi	Benue
Makurdi	Benue
Kwande	Benue
Otukpo	Benue
Otukpo	Benue
Oju	Benue
Makurdi	Benue
Makurdi	Benue
Gwer East	Benue
Makurdi	Benue
Ukum	Benue
Vandeikya	Benue
Gboko	Benue
Gboko	Benue
Makurdi	Benue
Makurdi	Benue
Makurdi	Benue
Gwer East	Benue
Gwer East	Benue
Makurdi	Benue
Makurdi	Benue
Buruku	Benue
Gwer West	Benue
Gwer West	Benue
Abadam	Borno

Bama	Borno
Biu	Borno
Gwoza	Borno
Maiduguri M. C.	Borno
Kukawa	Borno
Maiduguri M. C.	Borno
Maiduguri M. C.	Borno
Maiduguri M. C.	Borno
Maiduguri M. C.	Borno
Maiduguri M. C.	Borno
Maiduguri M. C.	Borno
Maiduguri M. C.	Borno
Maiduguri M. C.	Borno
Maiduguri M. C.	Borno
Monguno	Borno
Ngala	Borno
Maiduguri M. C.	Borno
Maiduguri M. C.	Borno
Maiduguri M. C.	Borno
Maiduguri M. C.	Borno
Maiduguri M. C.	Borno
Maiduguri M. C.	Borno
Maiduguri M. C.	Borno
Biu	Borno
Bama	Borno
Monguno	Borno
Biu	Borno
Maiduguri M. C.	Borno
Gwoza	Borno
Maiduguri M. C.	Borno
Maiduguri M. C.	Borno
Maiduguri M. C.	Borno
Biu	Borno
Biu	Borno
Biu	Borno

Maiduguri M. C.	Borno
Ngala	Borno
Damboa	Borno
Hawul	Borno
Dikwa	Borno
Kwaya / Kusar	Borno
Shani	Borno
Konduga	Borno
Mobbar	Borno
Gubio	Borno
Nganzai	Borno
Marte	Borno
Gwoza	Borno
Bama	Borno
Gwoza	Borno
Magumeri	Borno
Askira / Uba	Borno
Jere	Borno
Maiduguri M. C.	Borno
Maiduguri M. C.	Borno
Maiduguri M. C.	Borno
Maiduguri M. C.	Borno
Jere	Borno
Maiduguri M. C.	Borno
Maiduguri M. C.	Borno
Maiduguri M. C.	Borno
Maiduguri M. C.	Borno
Gwoza	Borno
Jere	Borno
Maiduguri M. C.	Borno
Jere	Borno
Maiduguri M. C.	Borno
Yenagoa	Bayelsa
Yenagoa	Bayelsa
Yenagoa	Bayelsa
Ekeremor	Bayelsa
Yenagoa	Bayelsa
Yenagoa	Bayelsa
Yenagoa	Bayelsa

Yenagoa	Bayelsa
Yenagoa	Bayelsa
Yenagoa	Bayelsa
Yenagoa	Bayelsa
Yenagoa	Bayelsa
Yenagoa	Bayelsa
Yenagoa	Bayelsa
Yenagoa	Bayelsa
Yenagoa	Bayelsa
Yenagoa	Bayelsa
Yenagoa	Bayelsa
Southern Ijaw	Bayelsa
Ogbia	Bayelsa
Yenagoa	Bayelsa
Yenagoa	Bayelsa
Yenagoa	Bayelsa
Yenagoa	Bayelsa
Calabar Municipality	Cross River
Calabar Municipality	Cross River
Calabar Municipality	Cross River
Calabar Municipality	Cross River
Calabar Municipality	Cross River
Calabar Municipality	Cross River
Calabar Municipality	Cross River
Calabar Municipality	Cross River
Calabar Municipality	Cross River
Calabar Municipality	Cross River
Calabar Municipality	Cross River

Calabar Municipality	Cross River
Calabar Municipality	Cross River
Ikom	Cross River
Ikom	Cross River
Obudu	Cross River
Ogoja	Cross River
Ogoja	Cross River
Ogoja	Cross River
Yakurr	Cross River
Yala	Cross River
Yala	Cross River
Yala	Cross River
Ikom	Cross River
Calabar Municipality	Cross River
Calabar Municipality	Cross River
Calabar Municipality	Cross River
Calabar Municipality	Cross River
Calabar Municipality	Cross River
Ikom	Cross River
Ogoja	Cross River
Ogoja	Cross River
Calabar Municipality	Cross River
Calabar Municipality	Cross River
Calabar Municipality	Cross River
Calabar Municipality	Cross River
Calabar Municipality	Cross River
Obudu	Cross River
Calabar Municipality	Cross River
Calabar Municipality	Cross River
Calabar Municipality	Cross River

Calabar Municipality	Cross River
Calabar Municipality	Cross River
Calabar Municipality	Cross River
Calabar Municipality	Cross River
Calabar Municipality	Cross River
Calabar South	Cross River
Calabar South	Cross River
Calabar South	Cross River
Calabar South	Cross River
Akpabuyo	Cross River
Odukpani	Cross River
Akamkpa	Cross River
Yakurr	Cross River
Obubra	Cross River
Ikom	Cross River
Obanliku	Cross River
Abi	Cross River
Calabar Municipality	Cross River
Biase	Cross River
Calabar Municipality	Cross River
Calabar Municipality	Cross River
Calabar Municipality	Cross River
Akamkpa	Cross River
Ikom	Cross River
Ikom	Cross River
Obubra	Cross River
Yakurr	Cross River
Yakurr	Cross River
Ogoja	Cross River
Yakurr	Cross River

Ikom	Cross River
Ikom	Cross River
Ikom	Cross River
Boki	Cross River
Yakurr	Cross River
Calabar Municipality	Cross River
Ogoja	Cross River
Calabar Municipality	Cross River
Biase	Cross River
Calabar South	Cross River
Bekwarra	Cross River
Calabar Municipality	Cross River
Ogoja	Cross River
Calabar Municipality	Cross River
Calabar Municipality	Cross River
Yakurr	Cross River
Calabar Municipality	Cross River
Ikom	Cross River
Calabar Municipality	Cross River
Uvwie	Delta
Uvwie	Delta
Uvwie	Delta
Uvwie	Delta
Uvwie	Delta
Sapele	Delta
Udu	Delta
Ughelli North	Delta
Warri North	Delta
Warri North	Delta

Warri North	Delta
Warri North	Delta
Warri North	Delta
Warri North	Delta
Warri North	Delta
Uvwie	Delta
Warri North	Delta
Warri North	Delta
Warri North	Delta
Warri North	Delta
Warri North	Delta
Warri North	Delta
Warri North	Delta
Uvwie	Delta
Ughelli North	Delta
Warri North	Delta
Uvwie	Delta
Warri North	Delta
Warri North	Delta
Sapele	Delta
Warri North	Delta
Warri South	Delta
Warri South	Delta
Warri South	Delta
Warri South	Delta
Warri South	Delta
Uvwie	Delta
Warri North	Delta
Warri North	Delta

Warri South	Delta
Udu	Delta
Warri South	Delta
Uvwie	Delta
Uvwie	Delta
Warri South	Delta
Ughelli North	Delta
Udu	Delta
Uvwie	Delta
Uvwie	Delta
Uvwie	Delta
Udu	Delta
Warri South	Delta
Oshimili - South	Delta
Warri South	Delta
Sapele	Delta
Warri South	Delta
Uvwie	Delta
Warri South	Delta
Warri South	Delta
Uvwie	Delta
Warri North	Delta
Warri South	Delta
Ughelli North	Delta
Uvwie	Delta
Uvwie	Delta
Sapele	Delta
Warri South	Delta

Warri South	Delta
Warri North	Delta
Warri South	Delta
Uvwie	Delta
Uvwie	Delta
Isoko North	Delta
Sapele	Delta
Sapele	Delta
Warri South	Delta
Uvwie	Delta
Ika North- East	Delta
Oshimili - North	Delta
Uvwie	Delta
Udu	Delta
Sapele	Delta
Warri South	Delta
Ika North- East	Delta
Uvwie	Delta
Warri South	Delta
Oshimili - South	Delta
Oshimili - South	Delta
Sapele	Delta
Warri North	Delta
Warri North	Delta

Aniocha - South	Delta
Aniocha - South	Delta
Aniocha - South	Delta
Aniocha - South	Delta
Warri North	Delta
Aniocha North	Delta
Abakaliki	Ebonyi
Abakaliki	Ebonyi
Abakaliki	Ebonyi
Abakaliki	Ebonyi
Abakaliki	Ebonyi
Abakaliki	Ebonyi
Afikpo North	Ebonyi
Ohaukwu	Ebonyi
Abakaliki	Ebonyi
Abakaliki	Ebonyi
Abakaliki	Ebonyi
Abakaliki	Ebonyi
Abakaliki	Ebonyi
Ebonyi	Ebonyi
Afikpo South	Ebonyi
Abakaliki	Ebonyi
Abakaliki	Ebonyi
Abakaliki	Ebonyi
Afikpo North	Ebonyi
Ikwo	Ebonyi
Abakaliki	Ebonyi
Esan West	Edo
Akoko Edo	Edo
Akoko Edo	Edo
Akoko Edo	Edo
Egor	Edo
Egor	Edo
Egor	Edo
Esan Central	Edo
Esan Central	Edo
Esan North East	Edo
Esan South East	Edo
Esan South East	Edo
Esan West	Edo
Esan West	Edo

Etsako Central	Edo
Etsako East	Edo
Etsako West	Edo
Etsako West	Edo
Etsako West	Edo
Igueben	Edo
Ikpoba/Okha	Edo
Oredo	Edo
Oredo	Edo
Oredo	Edo
Oredo	Edo
Oredo	Edo
Orhionmwon	Edo
Orhionmwon	Edo
Ovia North East	Edo
Ovia North East	Edo
Ovia South West	Edo
Ovia South West	Edo
Owan West	Edo
Uhunmwode	Edo
Orhionmwon	Edo
Etsako Central	Edo
Etsako Central	Edo
Ikpoba/Okha	Edo
Oredo	Edo
Oredo	Edo
Oredo	Edo
Owan West	Edo
Oredo	Edo
Oredo	Edo
Oredo	Edo
Oredo	Edo
Esan North East	Edo
Oredo	Edo
Akoko Edo	Edo

Egor	Edo
Oredo	Edo
Oredo	Edo
Oredo	Edo
Oredo	Edo
Etsako West	Edo
Oredo	Edo
Esan Central	Edo
Owan East	Edo
Ikpoba/Okha	Edo
Akoko Edo	Edo
Etsako West	Edo
Oredo	Edo
Oredo	Edo
Oredo	Edo
Oredo	Edo
Ikpoba/Okha	Edo
Ikpoba/Okha	Edo
Oredo	Edo
Egor	Edo
Esan North East	Edo
Ovia North East	Edo
Etsako West	Edo
Etsako East	Edo
Oredo	Edo
Oredo	Edo
Oredo	Edo
Egor	Edo

Oredo	Edo
Ikpoba/Okha	Edo
Igueben	Edo
Ikpoba/Okha	Edo
Oredo	Edo
Egor	Edo
Orhionmwon	Edo
Oredo	Edo
Egor	Edo
Esan West	Edo
Oredo	Edo
Oredo	Edo
Etsako East	Edo
Oredo	Edo
Oredo	Edo
Oredo	Edo
Ikpoba/Okha	Edo
Ikpoba/Okha	Edo
Oredo	Edo
Oredo	Edo
Etsako Central	Edo
Oredo	Edo
Egor	Edo
Egor	Edo
Ikpoba/Okha	Edo
Orhionmwon	Edo

Etsako West	Edo
Oredo	Edo
Oredo	Edo
Oredo	Edo
Oredo	Edo
Egor	Edo
Egor	Edo
Owan West	Edo
Esan North East	Edo
Egor	Edo
Oredo	Edo
Oredo	Edo
Oredo	Edo
Egor	Edo
Oredo	Edo
Oredo	Edo
Oredo	Edo
Esan West	Edo
Ikpoba/Okha	Edo
Ikpoba/Okha	Edo
Oredo	Edo

Owan West	Edo
Oredo	Edo
Etsako East	Edo
Oredo	Edo
Oredo	Edo
Esan West	Edo
Esan North East	Edo
Esan West	Edo
Oredo	Edo
Oredo	Edo
Ado Ekiti	Ekiti
Ado Ekiti	Ekiti
Ado Ekiti	Ekiti
Ado Ekiti	Ekiti
Ado Ekiti	Ekiti
Ado Ekiti	Ekiti
Ido / Osi	Ekiti
Ado Ekiti	Ekiti
Ado Ekiti	Ekiti
Ado Ekiti	Ekiti
Ado Ekiti	Ekiti
Ado Ekiti	Ekiti
Ado Ekiti	Ekiti
Ado Ekiti	Ekiti
Ado Ekiti	Ekiti
Ado Ekiti	Ekiti
Ado Ekiti	Ekiti
Ado Ekiti	Ekiti
Ikole	Ekiti
Ado Ekiti	Ekiti
Ado Ekiti	Ekiti
Ado Ekiti	Ekiti
Ado Ekiti	Ekiti

Ido / Osi	Ekiti
Ado Ekiti	Ekiti
Ado Ekiti	Ekiti
Ado Ekiti	Ekiti
Ikole	Ekiti
Ado Ekiti	Ekiti
Ado Ekiti	Ekiti
Ado Ekiti	Ekiti
Ado Ekiti	Ekiti
Ado Ekiti	Ekiti
Ado Ekiti	Ekiti
Ado Ekiti	Ekiti
Ikere	Ekiti
Ado Ekiti	Ekiti
Ado Ekiti	Ekiti
Ado Ekiti	Ekiti
Oye	Ekiti
Ado Ekiti	Ekiti
Oye	Ekiti
Moba	Ekiti
Enugu East	Enugu
Enugu East	Enugu
Enugu South	Enugu
Enugu South	Enugu
Enugu East	Enugu

Nsukka	Enugu
Enugu South	Enugu
Enugu East	Enugu
Udi	Enugu
Enugu South	Enugu
Nsukka	Enugu
Udi	Enugu
Udi	Enugu
Enugu South	Enugu
Enugu South	Enugu
Enugu East	Enugu
Enugu East	Enugu
Enugu East	Enugu
Enugu East	Enugu
Enugu South	Enugu
Awgu	Enugu
Ezeagu	Enugu
Enugu South	Enugu
Enugu East	Enugu
Aninri	Enugu
Enugu East	Enugu
Enugu South	Enugu
Enugu North	Enugu
Aninri	Enugu
Enugu East	Enugu
Enugu East	Enugu
Enugu East	Enugu
Enugu North	Enugu
Oji-River	Enugu
Enugu North	Enugu
Enugu South	Enugu
Enugu East	Enugu
Enugu South	Enugu
Enugu North	Enugu
Nkanu West	Enugu
Nsukka	Enugu

Nsukka	Enugu
Enugu North	Enugu
Enugu North	Enugu
Enugu South	Enugu
Enugu North	Enugu
Enugu South	Enugu
Enugu East	Enugu
Nsukka	Enugu
Nsukka	Enugu
Enugu North	Enugu
Nsukka	Enugu
Enugu South	Enugu
Enugu East	Enugu
Enugu East	Enugu
Enugu South	Enugu
Enugu East	Enugu
Nkanu East	Enugu
Nsukka	Enugu
Nsukka	Enugu
Nsukka	Enugu
Enugu North	Enugu
Nsukka	Enugu
Nsukka	Enugu
Nsukka	Enugu
Enugu North	Enugu
Udi	Enugu
Udi	Enugu
Nsukka	Enugu
Nsukka	Enugu
Oji-River	Enugu
Udenu	Enugu
Enugu East	Enugu
Awgu	Enugu
Nkanu West	Enugu
Enugu East	Enugu

Enugu East	Enugu
Enugu North	Enugu
Enugu North	Enugu
Enugu East	Enugu
Enugu South	Enugu
Enugu North	Enugu
Udenu	Enugu
Enugu North	Enugu
Udenu	Enugu
Igbo Eze North	Enugu
Enugu North	Enugu
Nsukka	Enugu
Municipal	FCT
Municipal	FCT
Municipal	FCT
Municipal	FCT
Municipal	FCT
Municipal	FCT
Municipal	FCT
Municipal	FCT
Kuje	FCT
Municipal	FCT
Municipal	FCT
Municipal	FCT
Municipal	FCT
Municipal	FCT

Bwari	FCT
Bwari	FCT
Bwari	FCT
Bwari	FCT
Bwari	FCT
Municipal	FCT
Bwari	FCT
Bwari	FCT
Bwari	FCT
Bwari	FCT
Bwari	FCT
Gwagwalada	FCT
Kwali	FCT
Municipal	FCT
Municipal	FCT
Municipal	FCT
Municipal	FCT
Municipal	FCT
Municipal	FCT
Municipal	FCT
Municipal	FCT
Municipal	FCT
Bwari	FCT
Kwali	FCT
Abaji	FCT
Municipal	FCT
Municipal	FCT
Municipal	FCT
Municipal	FCT
Municipal	FCT

Municipal	FCT
Municipal	FCT
Municipal	FCT
Municipal	FCT
Kuje	FCT
Municipal	FCT
Municipal	FCT
Municipal	FCT
Municipal	FCT
Municipal	FCT
Municipal	FCT
Municipal	FCT
Municipal	FCT
Municipal	FCT
Municipal	FCT
Municipal	FCT
Municipal	FCT
Municipal	FCT
Municipal	FCT
Municipal	FCT
Municipal	FCT
Municipal	FCT
Municipal	FCT
Municipal	FCT
Municipal	FCT

Municipal	FCT
Municipal	FCT
Municipal	FCT
Abaji	FCT
Gwagwalada	FCT
Gwagwalada	FCT
Municipal	FCT
Municipal	FCT
Municipal	FCT
Municipal	FCT
Municipal	FCT
Municipal	FCT
Municipal	FCT
Municipal	FCT
Municipal	FCT
Gwagwalada	FCT
Municipal	FCT
Municipal	FCT
Municipal	FCT
Municipal	FCT
Municipal	FCT
Municipal	FCT
Municipal	FCT
Municipal	FCT
Bwari	FCT
Municipal	FCT
Municipal	FCT
Municipal	FCT
Municipal	FCT
Bwari	FCT

Municipal	FCT
Municipal	FCT
Municipal	FCT
Municipal	FCT
Municipal	FCT
Municipal	FCT
Municipal	FCT
Municipal	FCT
Municipal	FCT
Municipal	FCT
Municipal	FCT
Municipal	FCT
Bwari	FCT
Municipal	FCT
Municipal	FCT
Municipal	FCT
Municipal	FCT
Municipal	FCT
Gwagwalada	FCT
Municipal	FCT
Gwagwalada	FCT
Municipal	FCT
Municipal	FCT
Municipal	FCT
Municipal	FCT
Kuje	FCT
Municipal	FCT
Municipal	FCT
Abaji	FCT

Municipal	FCT
Gwagwalada	FCT
Gwagwalada	FCT
Kuje	FCT
Gwagwalada	FCT
Gwagwalada	FCT
Bwari	FCT
Bwari	FCT
Municipal	FCT
Municipal	FCT
Bwari	FCT
Bwari	FCT
Bwari	FCT
Bwari	FCT
Bwari	FCT
Bwari	FCT
Gwagwalada	FCT
Municipal	FCT
Municipal	FCT
Municipal	FCT
Bwari	FCT
Municipal	FCT
Municipal	FCT

Municipal	FCT
Municipal	FCT
Municipal	FCT
Bwari	FCT
Municipal	FCT
Kuje	FCT
Gwagwalada	FCT
Municipal	FCT
Kuje	FCT
Municipal	FCT
Bwari	FCT
Municipal	FCT
Bwari	FCT
Municipal	FCT
Kuje	FCT
Municipal	FCT
Municipal	FCT
Gwagwalada	FCT
Municipal	FCT
Municipal	FCT
Municipal	FCT
Municipal	FCT
Municipal	FCT
Municipal	FCT
Municipal	FCT
Gwagwalada	FCT
Municipal	FCT
Municipal	FCT
Bwari	FCT
Municipal	FCT
Municipal	FCT

Municipal	FCT
Bwari	FCT
Municipal	FCT
Municipal	FCT
Municipal	FCT
Bwari	FCT
Gwagwalada	FCT
Bwari	FCT
Municipal	FCT
Municipal	FCT
Municipal	FCT
Municipal	FCT
Municipal	FCT
Municipal	FCT
Municipal	FCT
Municipal	FCT
Municipal	FCT
Municipal	FCT
Municipal	FCT
Municipal	FCT
Municipal	FCT
Bwari	FCT
Municipal	FCT

Municipal	FCT
Municipal	FCT
Municipal	FCT
Municipal	FCT
Municipal	FCT
Municipal	FCT
Municipal	FCT
Municipal	FCT
Municipal	FCT
Municipal	FCT
Municipal	FCT
Municipal	FCT
Municipal	FCT
Municipal	FCT
Municipal	FCT
Municipal	FCT
Municipal	FCT
Municipal	FCT
Municipal	FCT
Municipal	FCT
Municipal	FCT
Municipal	FCT

Bwari	FCT
Municipal	FCT
Municipal	FCT
Kuje	FCT
Municipal	FCT
Bwari	FCT
Municipal	FCT
Gwagwalada	FCT
Kuje	FCT
Gwagwalada	FCT
Municipal	FCT
Municipal	FCT
Gwagwalada	FCT
Municipal	FCT
Kwali	FCT
Municipal	FCT
Bwari	FCT
Municipal	FCT
Municipal	FCT
Municipal	FCT

Kuje	FCT
Municipal	FCT
Municipal	FCT
Municipal	FCT
Municipal	FCT
Municipal	FCT
Municipal	FCT
Municipal	FCT
Municipal	FCT
Kuje	FCT
Bwari	FCT
Municipal	FCT
Municipal	FCT
Municipal	FCT
Municipal	FCT
Municipal	FCT
Municipal	FCT
Municipal	FCT
Municipal	FCT

Billiri	Gombe
Gombe	Gombe
Gombe	Gombe
Gombe	Gombe
Gombe	Gombe
Gombe	Gombe
Gombe	Gombe
Gombe	Gombe
Gombe	Gombe
Gombe	Gombe
Gombe	Gombe
Gombe	Gombe
Gombe	Gombe
Kaltungo	Gombe
Akko	Gombe
Gombe	Gombe
Dukku	Gombe
Yalmaltu/ Deba	Gombe
Gombe	Gombe
Gombe	Gombe
Billiri	Gombe
Akko	Gombe
Akko	Gombe
Akko	Gombe
Akko	Gombe
Akko	Gombe
Akko	Gombe
Akko	Gombe
Akko	Gombe
Akko	Gombe
Akko	Gombe
Akko	Gombe
Akko	Gombe
Akko	Gombe
Akko	Gombe
Akko	Gombe
Akko	Gombe
Akko	Gombe
Akko	Gombe
Akko	Gombe
Akko	Gombe
Akko	Gombe
Akko	Gombe
Akko	Gombe
Akko	Gombe
Akko	Gombe
Akko	Gombe
Akko	Gombe

Akko	Gombe
Akko	Gombe
Akko	Gombe
Nafada	Gombe
Nafada	Gombe
Nafada	Gombe
Nafada	Gombe
Nafada	Gombe
Nafada	Gombe
Nafada	Gombe
Nafada	Gombe
Nafada	Gombe
Nafada	Gombe
Nafada	Gombe
Nafada	Gombe
Nafada	Gombe
Nafada	Gombe
Nafada	Gombe
Shongom	Gombe
Shongom	Gombe
Shongom	Gombe
Shongom	Gombe
Shongom	Gombe
Shongom	Gombe
Shongom	Gombe
Shongom	Gombe
Shongom	Gombe
Shongom	Gombe
Shongom	Gombe
Shongom	Gombe
Shongom	Gombe
Shongom	Gombe
Shongom	Gombe
Shongom	Gombe
Shongom	Gombe
Shongom	Gombe
Shongom	Gombe
Shongom	Gombe
Shongom	Gombe
Shongom	Gombe
Shongom	Gombe
Shongom	Gombe
Gombe	Gombe
Akko	Gombe
Akko	Gombe
Gombe	Gombe

Billiri	Gombe
Akko	Gombe
Akko	Gombe
Akko	Gombe
Aboh Mbaise	Imo
Ahiazu Mbaise	Imo
Ezinihitte Mbaise	Imo
Ezinihitte Mbaise	Imo
Ikeduru (Iho)	Imo
Okigwe (Okigwe)	Imo
Owerri West (Umuguma)	Imo
Owerri Urban (Owerri)	Imo
Owerri Urban (Owerri)	Imo
Owerri Urban (Owerri)	Imo
Owerri Urban (Owerri)	Imo
Owerri Urban (Owerri)	Imo
Owerri Urban (Owerri)	Imo
Owerri Urban (Owerri)	Imo
Owerri Urban (Owerri)	Imo
Owerri Urban (Owerri)	Imo
Owerri Urban (Owerri)	Imo
Owerri Urban (Owerri)	Imo
Owerri Urban (Owerri)	Imo
Owerri Urban (Owerri)	Imo
Owerri Urban (Owerri)	Imo
Owerri Urban (Owerri)	Imo
Owerri Urban (Owerri)	Imo
Owerri North (Orie Uratta)	Imo

Owerri North (Orie Uratta)	Imo
Owerri West (Umuguma)	Imo
Owerri Urban (Owerri)	Imo
Owerri Urban (Owerri)	Imo
Owerri Urban (Owerri)	Imo
Owerri Urban (Owerri)	Imo
Mbaitoli (Nwaorieubi)	Imo
Owerri Urban (Owerri)	Imo
Owerri North (Orie Uratta)	Imo
Okigwe (Okigwe)	Imo
Orlu	Imo
Owerri North (Orie Uratta)	Imo
Owerri Urban (Owerri)	Imo
Owerri Urban (Owerri)	Imo
Owerri Urban (Owerri)	Imo
Aboh Mbaise	Imo
Owerri Urban (Owerri)	Imo
Owerri Urban (Owerri)	Imo
Owerri West (Umuguma)	Imo
Owerri West (Umuguma)	Imo
Owerri North (Orie Uratta)	Imo
Owerri Urban (Owerri)	Imo
Owerri Urban (Owerri)	Imo
Owerri Urban (Owerri)	Imo
Owerri Urban (Owerri)	Imo

Mbaitoli (Nwaorieubi)	Imo
Ihitte/Uboma (Isinweke)	Imo
Owerri West (Umuguma)	Imo
Owerri Urban (Owerri)	Imo
Owerri North (Orie Uratta)	Imo
Owerri North (Orie Uratta)	Imo
Orlu	Imo
Owerri North (Orie Uratta)	Imo
Aboh Mbaise	Imo
Owerri North (Orie Uratta)	Imo
Owerri Urban (Owerri)	Imo
Orlu	Imo
Owerri Urban (Owerri)	Imo
Owerri Urban (Owerri)	Imo
Owerri West (Umuguma)	Imo
Owerri Urban (Owerri)	Imo
Owerri North (Orie Uratta)	Imo
Owerri Urban (Owerri)	Imo
Owerri West (Umuguma)	Imo
Owerri Urban (Owerri)	Imo
Ngor Okpala (Umuneke)	Imo
Isu (Umundugba)	Imo
Aboh Mbaise	Imo
Owerri North (Orie Uratta)	Imo
Owerri North (Orie Uratta)	Imo
Orlu	Imo

Owerri West (Umuguma)	Imo
Owerri West (Umuguma)	Imo
Ahiazu Mbaise	Imo
Owerri Urban (Owerri)	Imo
Ahiazu Mbaise	Imo
Owerri West (Umuguma)	Imo
Owerri West (Umuguma)	Imo
Owerri North (Orie Uratta)	Imo
Ngor Okpala (Umuneke)	Imo
Dutse	Jigawa
Jahun	Jigawa
Hadejia	Jigawa
Gumel	Jigawa
Kazaure	Jigawa
Kazaure	Jigawa
Ringim	Jigawa
Birnin Kudu	Jigawa
Dutse	Jigawa
Dutse	Jigawa
Babura	Jigawa
Dutse	Jigawa
Dutse	Jigawa
Dutse	Jigawa
Dutse	Jigawa
Kafin Hausa	Jigawa
Argungu	Kebbi
Birnin Kebbi	Kebbi
Birnin Kebbi	Kebbi
Yauri	Kebbi
Birnin Kebbi	Kebbi
Zuru	Kebbi
Birnin Kebbi	Kebbi
Birnin Kebbi	Kebbi
Birnin Kebbi	Kebbi
Yauri	Kebbi
Zuru	Kebbi
Birnin Kebbi	Kebbi

Birnin Kebbi	Kebbi
Birnin Kebbi	Kebbi
Igabi	Kaduna
Kaduna South	Kaduna
Kaduna North	Kaduna
Kaduna North	Kaduna
Kaduna North	Kaduna
Kaduna North	Kaduna
Kaduna North	Kaduna
Kaduna North	Kaduna
Kaduna North	Kaduna
Kaduna North	Kaduna
Kaduna North	Kaduna
Kaduna North	Kaduna
Kaduna North	Kaduna
Kaduna North	Kaduna
Kaduna North	Kaduna
Kaduna North	Kaduna
Kaduna North	Kaduna
Kaduna North	Kaduna
Kaduna South	Kaduna
Kaduna South	Kaduna
Kaduna South	Kaduna
Kaduna South	Kaduna
Kaduna South	Kaduna
Kaduna South	Kaduna
Kaduna South	Kaduna
Kaduna South	Kaduna
Kaduna South	Kaduna
Kaduna South	Kaduna

Kaduna South	Kaduna
Kaduna South	Kaduna
Kaduna South	Kaduna
Kaduna North	Kaduna
Sabon Gari	Kaduna
Sabon Gari	Kaduna
Zaria	Kaduna
Zaria	Kaduna
Zaria	Kaduna
Kaduna North	Kaduna
Sabon Gari	Kaduna
Kaduna North	Kaduna
Kaduna North	Kaduna
Kaduna North	Kaduna
Kaduna North	Kaduna
Zaria	Kaduna
Igabi	Kaduna
Giwa	Kaduna
Jema'a	Kaduna
Zaria	Kaduna
Kaduna South	Kaduna
Kaduna North	Kaduna
Kaduna South	Kaduna
Kaduna North	Kaduna
Kaduna North	Kaduna
Kaduna North	Kaduna
Zaria	Kaduna
Kaduna North	Kaduna
Kaduna North	Kaduna
Kaduna North	Kaduna
Igabi	Kaduna
Kaduna North	Kaduna
Zaria	Kaduna
Jema'a	Kaduna
Kaduna North	Kaduna
Kachia	Kaduna

Kaduna South	Kaduna
Kaduna South	Kaduna
Kaduna North	Kaduna
Makarfi	Kaduna
Kaduna South	Kaduna
Igabi	Kaduna
Sabon Gari	Kaduna
Kaduna South	Kaduna
Kaduna North	Kaduna
Kaduna North	Kaduna
Birnin Gwari	Kaduna
Kaduna North	Kaduna
Chikun	Kaduna
Kaduna South	Kaduna
Zaria	Kaduna
Kaduna North	Kaduna
Sabon Gari	Kaduna
Kaduna South	Kaduna
Zaria	Kaduna
Kaduna South	Kaduna
Zaria	Kaduna
Kaduna North	Kaduna
Kaduna North	Kaduna
Kaduna South	Kaduna
Sabon Gari	Kaduna
Kaduna South	Kaduna
Kaduna North	Kaduna
Kaduna North	Kaduna

Chikun	Kaduna
Kaduna North	Kaduna
Jaba	Kaduna
Kaduna North	Kaduna
Zaria	Kaduna
Kaduna North	Kaduna
Jema'a	Kaduna
Kaduna South	Kaduna
Kaduna North	Kaduna
Kaduna North	Kaduna
Kaduna North	Kaduna
Zangon Kataf	Kaduna
Igabi	Kaduna
Kachia	Kaduna
Kachia	Kaduna
Zaria	Kaduna
Lere	Kaduna
Kaduna North	Kaduna
Sabon Gari	Kaduna
Sabon Gari	Kaduna
Kaduna North	Kaduna
Zaria	Kaduna
Chikun	Kaduna
Chikun	Kaduna
Kaduna North	Kaduna
Igabi	Kaduna
Chikun	Kaduna
Kaduna North	Kaduna
Kaduna North	Kaduna
Kaduna South	Kaduna
Sabon Gari	Kaduna

Kaduna North	Kaduna
Sabon Gari	Kaduna
Kaduna North	Kaduna
Chikun	Kaduna
Sabon Gari	Kaduna
Sabon Gari	Kaduna
Kaduna South	Kaduna
Zaria	Kaduna
Kaduna South	Kaduna
Kaduna South	Kaduna
Kaduna North	Kaduna
Zaria	Kaduna
Zaria	Kaduna
Kubau	Kaduna
Zaria	Kaduna
Kaduna South	Kaduna
Kaduna South	Kaduna
Zaria	Kaduna
Igabi	Kaduna
Kaduna North	Kaduna
Zaria	Kaduna
Kaduna North	Kaduna
Kaduna South	Kaduna
Kaduna North	Kaduna

Kaduna South	Kaduna
Kaduna South	Kaduna
Kaduna North	Kaduna
Kaduna North	Kaduna
Igabi	Kaduna
Kaduna South	Kaduna
Zaria	Kaduna
Kaduna South	Kaduna
Kaduna North	Kaduna
Sabon Gari	Kaduna
Kaduna North	Kaduna
Ajaokuta	Kogi
Dekina	Kogi
Dekina	Kogi
Idah	Kogi
Lokoja	Kogi
Mopa Moro	Kogi
Okene	Kogi
Yagba West	Kogi
Lokoja	Kogi
Kabba/Bunu	Kogi
Yagba East	Kogi
Ankpa	Kogi
Lokoja	Kogi
Yagba East	Kogi
Yagba East	Kogi
Kabba/Bunu	Kogi
Yagba West	Kogi
Kabba/Bunu	Kogi
Ijumu	Kogi
Ajaokuta	Kogi
Dekina	Kogi
Kabba/Bunu	Kogi
Lokoja	Kogi
Okene	Kogi
Dekina	Kogi

Ajaokuta	Kogi
Okene	Kogi
Idah	Kogi
Lokoja	Kogi
Idah	Kogi
Okene	Kogi
Lokoja	Kogi
Ajaokuta	Kogi
Okehi	Kogi
Okene	Kogi
Okene	Kogi
Lokoja	Kogi
Lokoja	Kogi
Adavi	Kogi
Dekina	Kogi
Lokoja	Kogi
Okehi	Kogi
Ankpa	Kogi
Ajaokuta	Kogi
Adavi	Kogi
Ajaokuta	Kogi
Ofu	Kogi
Dekina	Kogi
Lokoja	Kogi
Okehi	Kogi
Ibaji	Kogi
Dekina	Kogi
Okene	Kogi
Olamaboro	Kogi
Ankpa	Kogi
Lokoja	Kogi
Okene	Kogi
Lokoja	Kogi

Dekina	Kogi
Dekina	Kogi
Idah	Kogi
Lokoja	Kogi
Lokoja	Kogi
Nasarawa	Kano
Fagge	Kano
Fagge	Kano
Fagge	Kano
Nasarawa	Kano
Kano Municipal	Kano
Tarauni	Kano
Kano Municipal	Kano
Nasarawa	Kano
Kano Municipal	Kano
Tarauni	Kano
Nasarawa	Kano
Tarauni	Kano
Kano Municipal	Kano
Kano Municipal	Kano
Nasarawa	Kano
Fagge	Kano
Kumbotso	Kano
Kano Municipal	Kano
Kano Municipal	Kano
Kano Municipal	Kano
Kano Municipal	Kano
Gwarzo	Kano
Kano Municipal	Kano
Wudil	Kano
Nasarawa	Kano
Danbata	Kano
Fagge	Kano
Tarauni	Kano
Kano Municipal	Kano
Ungogo	Kano
Dala	Kano

Wudil	Kano
Kano Municipal	Kano
Dala	Kano
Tarauni	Kano
Nasarawa	Kano
Fagge	Kano
Tarauni	Kano
Tarauni	Kano
Fagge	Kano
Fagge	Kano
Tudun Wada	Kano
Bichi	Kano
Bichi	Kano
Gwale	Kano
Tarauni	Kano
Fagge	Kano
Bebeji	Kano
Nasarawa	Kano
Kumbotso	Kano
Ado Odo-Ota	Ogun
Kano Municipal	Kano
Minjibir	Kano
Fagge	Kano
Dala	Kano
Tarauni	Kano
Sumaila	Kano
Gaya	Kano
Kano Municipal	Kano
Nasarawa	Kano

Tarauni	Kano
Fagge	Kano
Dala	Kano
Dawaki Kudu	Kano
Fagge	Kano
Kumbotso	Kano
Shanono	Kano
Gezawa	Kano
Fagge	Kano
Fagge	Kano
Nasarawa	Kano
Nasarawa	Kano
Nasarawa	Kano
Dala	Kano
Kumbotso	Kano
Kumbotso	Kano
Tarauni	Kano
Karaye	Kano
Gwale	Kano
Kumbotso	Kano
Kumbotso	Kano
Bakori	Katsina
Bakori	Katsina
Bakori	Katsina
Dandume	Katsina
Daura	Katsina
Daura	Katsina
Daura	Katsina
Dutsin-Ma	Katsina
Funtua	Katsina
Funtua	Katsina
Funtua	Katsina
Dandume	Katsina

Funtua	Katsina
Kankara	Katsina
Kankia	Katsina
Katsina	Katsina
Katsina	Katsina
Katsina	Katsina
Kurfi	Katsina
Malufashi	Katsina
Mani	Katsina
Mashi	Katsina
Katsina	Katsina
Katsina	Katsina
Malufashi	Katsina
Malufashi	Katsina
Katsina	Katsina
Katsina	Katsina
Funtua	Katsina
Jibia	Katsina
Daura	Katsina
Katsina	Katsina
Katsina	Katsina
Dutsin-Ma	Katsina
Malufashi	Katsina
Katsina	Katsina
Katsina	Katsina
Katsina	Katsina
Malufashi	Katsina
Musawa	Katsina
Malufashi	Katsina
Katsina	Katsina
Katsina	Katsina
Katsina	Katsina
Katsina	Katsina

Katsina	Katsina
Jibia	Katsina
Ilorin East	Kwara
Ilorin East	Kwara
Ilorin East	Kwara
Ilorin East	Kwara
Ilorin East	Kwara
Ilorin East	Kwara
Ilorin-West	Kwara
Ilorin-West	Kwara
Ilorin-West	Kwara
Ilorin East	Kwara
Ilorin East	Kwara
Ilorin East	Kwara
Ilorin-South	Kwara
Offa	Kwara
Ilorin East	Kwara
Ilorin-South	Kwara
Ilorin East	Kwara
Ilorin East	Kwara
Ilorin East	Kwara
Irepodun	Kwara
Moro	Kwara
Ilorin East	Kwara
Patigi	Kwara
Ilorin East	Kwara
Ilorin East	Kwara
Ilorin-South	Kwara
Ilorin East	Kwara
Ilorin East	Kwara
Irepodun	Kwara
Ilorin East	Kwara
Ilorin East	Kwara
Ilorin East	Kwara

Ilorin-South	Kwara
Irepodun	Kwara
Ilorin East	Kwara
Ilorin East	Kwara
Oyun	Kwara
Ilorin-South	Kwara
Ilorin East	Kwara
Ilorin East	Kwara
Offa	Kwara
Irepodun	Kwara
Ilorin-South	Kwara
Ilorin-South	Kwara
Ilorin-South	Kwara
Ilorin-West	Kwara
Ilorin East	Kwara
Ilorin East	Kwara
Ilorin-West	Kwara
Irepodun	Kwara
Moro	Kwara
Ilorin-West	Kwara
Offa	Kwara
Offa	Kwara
Ilorin-South	Kwara
Edu	Kwara
Kaiama	Kwara
Offa	Kwara
Ekiti	Kwara
Ilorin-South	Kwara
Irepodun	Kwara
Irepodun	Kwara
Ilorin-West	Kwara
Ilorin East	Kwara
Ilorin-West	Kwara
Ilorin-South	Kwara
Ilorin-West	Kwara
Ilorin-West	Kwara
Ilorin East	Kwara
Edu	Kwara
Edu	Kwara
Offa	Kwara
Ilorin-South	Kwara

Ilorin East	Kwara
Patigi	Kwara
Ilorin-West	Kwara
Patigi	Kwara
Ilorin-South	Kwara
Ilorin-South	Kwara
Ilorin East	Kwara
Irepodun	Kwara
Ilorin-South	Kwara
Baruten	Kwara
Oyun	Kwara
Offa	Kwara
Ilorin-West	Kwara
Ilorin-South	Kwara
Ilorin-South	Kwara
Moro	Kwara
Baruten	Kwara
Asa	Kwara
Irepodun	Kwara
Ifelodun	Kwara
Ilorin East	Kwara
Patigi	Kwara
Ilorin-West	Kwara
Offa	Kwara
Ilorin-West	Kwara
Ilorin-West	Kwara

Ilorin-South	Kwara
Ilorin-West	Kwara
Offa	Kwara
Ilorin East	Kwara
Ilorin-West	Kwara
Offa	Kwara
Offa	Kwara
Irepodun	Kwara
Offa	Kwara
Ilorin-West	Kwara
Ilorin-South	Kwara
Ilorin-South	Kwara
Ilorin-West	Kwara
Ilorin East	Kwara
Ilorin-West	Kwara
Ilorin-South	Kwara
Ilorin-South	Kwara
Ilorin-West	Kwara
Ilorin East	Kwara
Ilorin-West	Kwara
Agege	Lagos
Agege	Lagos

Agege	Lagos
Agege	Lagos
Agege	Lagos
Agege	Lagos
Agege	Lagos
Agege	Lagos
Agege	Lagos
Agege	Lagos
Agege	Lagos
Agege	Lagos
Ajeromi/Ifelodun	Lagos
Ajeromi/Ifelodun	Lagos
Ajeromi/Ifelodun	Lagos
Alimosho	Lagos
Alimosho	Lagos
Alimosho	Lagos
Alimosho	Lagos
Alimosho	Lagos
Alimosho	Lagos
Amuwo-Odofin	Lagos
Amuwo-Odofin	Lagos
Amuwo-Odofin	Lagos
Amuwo-Odofin	Lagos
Amuwo-Odofin	Lagos
Amuwo-Odofin	Lagos

Amuwo-Odofin	Lagos
Amuwo-Odofin	Lagos
Amuwo-Odofin	Lagos
Amuwo-Odofin	Lagos
Amuwo-Odofin	Lagos
Amuwo-Odofin	Lagos
Amuwo-Odofin	Lagos
Amuwo-Odofin	Lagos
Amuwo-Odofin	Lagos
Apapa	Lagos
Apapa	Lagos
Apapa	Lagos
Apapa	Lagos
Apapa	Lagos
Apapa	Lagos
Apapa	Lagos
Apapa	Lagos
Apapa	Lagos
Apapa	Lagos
Badagry	Lagos
Ojo	Lagos
Epe	Lagos
Eti-Osa	Lagos
Eti-Osa	Lagos
Eti-Osa	Lagos
Eti-Osa	Lagos
Eti-Osa	Lagos
Eti-Osa	Lagos
Eti-Osa	Lagos

Eti-Osa	Lagos
Eti-Osa	Lagos
Eti-Osa	Lagos
Eti-Osa	Lagos
Eti-Osa	Lagos
Ifako-Ijaye	Lagos
Ifako-Ijaye	Lagos
Ikeja	Lagos
Ikeja	Lagos
Ikeja	Lagos
Ikeja	Lagos
Ikeja	Lagos
Ikeja	Lagos
Ikeja	Lagos
Ikeja	Lagos
Ikeja	Lagos
Ikeja	Lagos
Ikeja	Lagos
Ikeja	Lagos
Ikeja	Lagos
Ikeja	Lagos
Ikeja	Lagos
Ikeja	Lagos
Ikeja	Lagos
Ikeja	Lagos
Ikeja	Lagos
Ikeja	Lagos
Ikeja	Lagos
Ikeja	Lagos
Ikeja	Lagos

Ikeja	Lagos
Ikorodu	Lagos
Ikorodu	Lagos
Ikorodu	Lagos
Ikorodu	Lagos
Kosofe	Lagos
Kosofe	Lagos
Kosofe	Lagos
Kosofe	Lagos
Kosofe	Lagos
Kosofe	Lagos
Kosofe	Lagos
Kosofe	Lagos
Lagos Island	Lagos
Lagos Island	Lagos
Ifako-Ijaye	Lagos
Lagos Island	Lagos
Lagos Island	Lagos
Lagos Mainland	Lagos
Lagos Mainland	Lagos
Lagos Mainland	Lagos
Lagos Mainland	Lagos
Lagos Mainland	Lagos
Lagos Mainland	Lagos
Lagos Mainland	Lagos
Lagos Mainland	Lagos
Lagos Mainland	Lagos
Lagos Mainland	Lagos
Lagos Mainland	Lagos
Lagos Mainland	Lagos
Lagos Mainland	Lagos

Mushin	Lagos
Mushin	Lagos
Mushin	Lagos
Mushin	Lagos
Mushin	Lagos
Mushin	Lagos
Mushin	Lagos
Mushin	Lagos
Mushin	Lagos
Mushin	Lagos
Mushin	Lagos
Mushin	Lagos
Mushin	Lagos
Mushin	Lagos
Mushin	Lagos
Mushin	Lagos
Ojo	Lagos
Ojo	Lagos
Ojo	Lagos
Ojo	Lagos
Ojo	Lagos
Ojo	Lagos
Ojo	Lagos
Oshodi/Isolo	Lagos
Oshodi/Isolo	Lagos

Oshodi/Isolo	Lagos
Oshodi/Isolo	Lagos
Oshodi/Isolo	Lagos
Oshodi/Isolo	Lagos
Oshodi/Isolo	Lagos
Oshodi/Isolo	Lagos
Oshodi/Isolo	Lagos
Oshodi/Isolo	Lagos
Oshodi/Isolo	Lagos
Oshodi/Isolo	Lagos
Agege	Lagos
Oshodi/Isolo	Lagos
Oshodi/Isolo	Lagos
Oshodi/Isolo	Lagos
Oshodi/Isolo	Lagos
Oshodi/Isolo	Lagos
Oshodi/Isolo	Lagos
Ibeju/Lekki	Lagos
Surulere	Lagos
Ojo	Lagos
Oshodi/Isolo	Lagos
Amuwo-Odofin	Lagos
Somolu	Lagos
Somolu	Lagos
Somolu	Lagos
Somolu	Lagos
Somolu	Lagos

Somolu	Lagos
Lagos Mainland	Lagos
Surulere	Lagos
Surulere	Lagos
Surulere	Lagos
Eti-Osa	Lagos
Surulere	Lagos
Surulere	Lagos
Surulere	Lagos
Surulere	Lagos
Surulere	Lagos
Surulere	Lagos
Surulere	Lagos
Surulere	Lagos
Surulere	Lagos
Surulere	Lagos
Surulere	Lagos
Surulere	Lagos
Surulere	Lagos
Surulere	Lagos
Surulere	Lagos
Surulere	Lagos
Surulere	Lagos
Surulere	Lagos
Surulere	Lagos

Surulere	Lagos
Surulere	Lagos
Surulere	Lagos
Oshodi/Isolo	Lagos
Eti-Osa	Lagos
Lagos Mainland	Lagos
Lagos Mainland	Lagos
Eti-Osa	Lagos
Eti-Osa	Lagos
Ikeja	Lagos
Lagos Island	Lagos
Lagos Mainland	Lagos
Eti-Osa	Lagos
Ikeja	Lagos
Ikeja	Lagos
Oshodi/Isolo	Lagos
Kosofe	Lagos
Mushin	Lagos
Agege	Lagos
Oshodi/Isolo	Lagos
Eti-Osa	Lagos
Kosofe	Lagos
Oshodi/Isolo	Lagos
Oshodi/Isolo	Lagos
Surulere	Lagos
Alimosho	Lagos
Mushin	Lagos
Ifako-Ijaye	Lagos
Mushin	Lagos
Eti-Osa	Lagos
Ikeja	Lagos
Lagos Island	Lagos

Kosofe	Lagos
Ikeja	Lagos
Ojo	Lagos
Ikorodu	Lagos
Eti-Osa	Lagos
Badagry	Lagos
Apapa	Lagos
Lagos Mainland	Lagos
Lagos Mainland	Lagos
Lagos Mainland	Lagos
Eti-Osa	Lagos
Ikeja	Lagos
Agege	Lagos
Ojo	Lagos
Eti-Osa	Lagos
Apapa	Lagos
Lagos Mainland	Lagos
Lagos Mainland	Lagos
Somolu	Lagos
Eti-Osa	Lagos
Oshodi/Isolo	Lagos
Oshodi/Isolo	Lagos
Lagos Mainland	Lagos
Lagos Mainland	Lagos
Mushin	Lagos
Eti-Osa	Lagos
Mushin	Lagos
Oshodi/Isolo	Lagos
Mushin	Lagos
Ifako-Ijaye	Lagos
Mushin	Lagos

Kosofe	Lagos
Eti-Osa	Lagos
Oshodi/Isolo	Lagos
Eti-Osa	Lagos
Eti-Osa	Lagos
Oshodi/Isolo	Lagos
Oshodi/Isolo	Lagos
Mushin	Lagos
Kosofe	Lagos
Kosofe	Lagos
Oshodi/Isolo	Lagos
Lagos Island	Lagos
Agege	Lagos
Oshodi/Isolo	Lagos
Ikorodu	Lagos
Agege	Lagos
Oshodi/Isolo	Lagos
Agege	Lagos
Kosofe	Lagos
Agege	Lagos
Agege	Lagos
Amuwo-Odofin	Lagos
Oshodi/Isolo	Lagos
Agege	Lagos
Ikorodu	Lagos

Lagos Island	Lagos
Agege	Lagos
Ikorodu	Lagos
Apapa	Lagos
Somolu	Lagos
Agege	Lagos
Alimosho	Lagos
Alimosho	Lagos
Ajeromi/Ifelodun	Lagos
Alimosho	Lagos
Amuwo-Odofin	Lagos
Eti-Osa	Lagos
Ikorodu	Lagos
Alimosho	Lagos
Alimosho	Lagos
Ajeromi/Ifelodun	Lagos
Oshodi/Isolo	Lagos
Somolu	Lagos
Alimosho	Lagos
Somolu	Lagos
Alimosho	Lagos
Alimosho	Lagos
Amuwo-Odofin	Lagos
Alimosho	Lagos
Agege	Lagos
Apapa	Lagos
Oshodi/Isolo	Lagos
Alimosho	Lagos
Amuwo-Odofin	Lagos

Oshodi/Isolo	Lagos
Alimosho	Lagos
Alimosho	Lagos
Ajeromi/Ifelodun	Lagos
Alimosho	Lagos
Apapa	Lagos
Alimosho	Lagos
Alimosho	Lagos
Amuwo-Odofin	Lagos
Apapa	Lagos
Surulere	Lagos
Apapa	Lagos
Apapa	Lagos
Ojo	Lagos
Ojo	Lagos
Ojo	Lagos
Ojo	Lagos
Apapa	Lagos
Amuwo-Odofin	Lagos
Apapa	Lagos
Ojo	Lagos
Badagry	Lagos
Badagry	Lagos
Apapa	Lagos
Apapa	Lagos
Ikeja	Lagos

Ikeja	Lagos
Ikeja	Lagos
Ikeja	Lagos
Ikeja	Lagos
Ikeja	Lagos
Kosofe	Lagos
Ikeja	Lagos
Ikeja	Lagos
Ikeja	Lagos
Ikeja	Lagos
Ikeja	Lagos
Ikeja	Lagos
Ikeja	Lagos
Ikeja	Lagos
Kosofe	Lagos
Ikeja	Lagos
Ikeja	Lagos
Ikeja	Lagos
Ikeja	Lagos
Ikeja	Lagos
Surulere	Lagos
Ikeja	Lagos
Surulere	Lagos
Surulere	Lagos
Surulere	Lagos
Surulere	Lagos
Surulere	Lagos
Surulere	Lagos
Surulere	Lagos

Lagos Island	Lagos
Alimosho	Lagos
Lagos Island	Lagos
Alimosho	Lagos
Alimosho	Lagos
Ikeja	Lagos
Ikeja	Lagos
Kosofe	Lagos
Lagos Island	Lagos
Oshodi/Isolo	Lagos
Mushin	Lagos
Badagry	Lagos
Surulere	Lagos
Lagos Mainland	Lagos
Ikeja	Lagos
Alimosho	Lagos
Alimosho	Lagos
Somolu	Lagos
Oshodi/Isolo	Lagos
Alimosho	Lagos
Somolu	Lagos
Lagos Island	Lagos
Lagos Island	Lagos
Oshodi/Isolo	Lagos
Oshodi/Isolo	Lagos
Kosofe	Lagos
Ajeromi/Ifelodun	Lagos

Surulere	Lagos
Agege	Lagos
Mushin	Lagos
Ajeromi/Ifelodun	Lagos
Somolu	Lagos
Eti-Osa	Lagos
Mushin	Lagos
Mushin	Lagos
Kosofe	Lagos
Agege	Lagos
Somolu	Lagos
Eti-Osa	Lagos
Eti-Osa	Lagos
Surulere	Lagos
Kosofe	Lagos
Surulere	Lagos
Alimosho	Lagos
Alimosho	Lagos
Amuwo-Odofin	Lagos
Alimosho	Lagos
Lagos Mainland	Lagos
Surulere	Lagos
Surulere	Lagos
Lagos Island	Lagos
Ikeja	Lagos
Surulere	Lagos
Oshodi/Isolo	Lagos
Surulere	Lagos
Surulere	Lagos
Ifako-Ijaye	Lagos

Alimosho	Lagos
Lagos Mainland	Lagos
Mushin	Lagos
Alimosho	Lagos
Ikeja	Lagos
Surulere	Lagos
Somolu	Lagos
Oshodi/Isolo	Lagos
Epe	Lagos
Oshodi/Isolo	Lagos
Alimosho	Lagos
Ifako-Ijaye	Lagos
Surulere	Lagos
Ifako-Ijaye	Lagos
Ifako-Ijaye	Lagos
Apapa	Lagos
Ajeromi/Ifelodun	Lagos
Mushin	Lagos
Ifako-Ijaye	Lagos
Lagos Island	Lagos
Oshodi/Isolo	Lagos
Alimosho	Lagos
Ifako-Ijaye	Lagos
Alimosho	Lagos
Oshodi/Isolo	Lagos
Alimosho	Lagos
Oshodi/Isolo	Lagos
Ajeromi/Ifelodun	Lagos
Amuwo-Odofin	Lagos
Lagos Island	Lagos
Alimosho	Lagos

Eti-Osa	Lagos
Apapa	Lagos
Amuwo-Odofin	Lagos
Surulere	Lagos
Surulere	Lagos
Somolu	Lagos
Ifako-Ijaye	Lagos
Alimosho	Lagos
Oshodi/Isolo	Lagos
Eti-Osa	Lagos
Alimosho	Lagos
Alimosho	Lagos
Surulere	Lagos
Oshodi/Isolo	Lagos
Surulere	Lagos
Ojo	Lagos
Alimosho	Lagos
Somolu	Lagos
Kosofe	Lagos
Apapa	Lagos
Lagos Island	Lagos
Ifako-Ijaye	Lagos
Ojo	Lagos
Ajeromi/Ifelodun	Lagos
Ajeromi/Ifelodun	Lagos
Alimosho	Lagos
Ajeromi/Ifelodun	Lagos
Oshodi/Isolo	Lagos
Alimosho	Lagos

Ajeromi/Ifelodun	Lagos
Kosofe	Lagos
Apapa	Lagos
Amuwo-Odofin	Lagos
Kosofe	Lagos
Amuwo-Odofin	Lagos
Ikeja	Lagos
Kosofe	Lagos
Ikeja	Lagos
Apapa	Lagos
Apapa	Lagos
Oshodi/Isolo	Lagos
Ojo	Lagos
Apapa	Lagos
Badagry	Lagos
Alimosho	Lagos
Somolu	Lagos
Alimosho	Lagos
Oshodi/Isolo	Lagos
Ifako-Ijaye	Lagos
Alimosho	Lagos
Lagos Mainland	Lagos
Alimosho	Lagos
Ajeromi/Ifelodun	Lagos
Surulere	Lagos
Ajeromi/Ifelodun	Lagos
Ojo	Lagos
Ojo	Lagos
Surulere	Lagos
Ojo	Lagos

Surulere	Lagos
Surulere	Lagos
Abakaliki	Ebonyi
Kosofe	Lagos
Alimosho	Lagos
Oshodi/Isolo	Lagos
Mushin	Lagos
Ikeja	Lagos
Apapa	Lagos
Agege	Lagos
Alimosho	Lagos
Alimosho	Lagos
Surulere	Lagos
Ifako-Ijaye	Lagos
Ifako-Ijaye	Lagos
Apapa	Lagos
Mushin	Lagos
Ikeja	Lagos
Ajeromi/Ifelodun	Lagos
Abakaliki	Ebonyi
Ajeromi/Ifelodun	Lagos
Apapa	Lagos
Ojo	Lagos
Ojo	Lagos
Ikeja	Lagos
Badagry	Lagos
Surulere	Lagos
Ajeromi/Ifelodun	Lagos
Oshodi/Isolo	Lagos
Agege	Lagos
Badagry	Lagos
Surulere	Lagos
Abakaliki	Ebonyi

Agege	Lagos
Somolu	Lagos
Somolu	Lagos
Ifako-Ijaye	Lagos
Oshodi/Isolo	Lagos
Ifako-Ijaye	Lagos
Mushin	Lagos
Somolu	Lagos
Kosofe	Lagos
Amuwo-Odofin	Lagos
Surulere	Lagos
Surulere	Lagos
Oshodi/Isolo	Lagos
Mushin	Lagos
Lagos Mainland	Lagos
Oshodi/Isolo	Lagos
Kosofe	Lagos
Agege	Lagos
Ikeja	Lagos
Ikeja	Lagos
Alimosho	Lagos
Ikeja	Lagos
Kosofe	Lagos
Lagos Mainland	Lagos
Kosofe	Lagos
Ajeromi/Ifelodun	Lagos
Surulere	Lagos
Agege	Lagos
Alimosho	Lagos
Alimosho	Lagos
Alimosho	Lagos
Alimosho	Lagos

Oshodi/Isolo	Lagos
Oshodi/Isolo	Lagos
Oshodi/Isolo	Lagos
Lagos Mainland	Lagos
Amuwo-Odofin	Lagos
Eti-Osa	Lagos
Alimosho	Lagos
Amuwo-Odofin	Lagos
Amuwo-Odofin	Lagos
Somolu	Lagos
Somolu	Lagos
Kosofe	Lagos
Kosofe	Lagos
Kosofe	Lagos
Surulere	Lagos
Abakaliki	Ebonyi
Ajeromi/Ifelodun	Lagos
Ibeju/Lekki	Lagos
Ifako-Ijaye	Lagos
Alimosho	Lagos
Alimosho	Lagos
Alimosho	Lagos
Ikorodu	Lagos
Agege	Lagos
Kosofe	Lagos
Agege	Lagos
Eti-Osa	Lagos

Kosofe	Lagos
Kosofe	Lagos
Ojo	Lagos
Amuwo-Odofin	Lagos
Kosofe	Lagos
Mushin	Lagos
Ojo	Lagos
Oshodi/Isolo	Lagos
Ifako-Ijaye	Lagos
Surulere	Lagos
Ajeromi/Ifelodun	Lagos
Apapa	Lagos
Alimosho	Lagos
Ikeja	Lagos
Alimosho	Lagos
Agege	Lagos
Apapa	Lagos
Eti-Osa	Lagos
Ikorodu	Lagos
Oshodi/Isolo	Lagos
Ikeja	Lagos
Alimosho	Lagos
Alimosho	Lagos
Alimosho	Lagos
Oshodi/Isolo	Lagos
Surulere	Lagos
Eti-Osa	Lagos

Surulere	Lagos
Kosofe	Lagos
Oshodi/Isolo	Lagos
0	Ikeja
Surulere	Lagos
Kosofe	Lagos
Surulere	Lagos
Alimosho	Lagos
Mushin	Lagos
Ikorodu	Lagos
Ikeja	Lagos
Alimosho	Lagos
Alimosho	Lagos
Oshodi/Isolo	Lagos
Alimosho	Lagos
Ifako-Ijaye	Lagos
Kosofe	Lagos
Ifako-Ijaye	Lagos
Alimosho	Lagos
Ikeja	Lagos

Surulere	Lagos
Ifako-Ijaye	Lagos
Lagos Mainland	Lagos
Ojo	Lagos
Ojo	Lagos
Alimosho	Lagos
Eti-Osa	Lagos
Kosofe	Lagos
Ikorodu	Lagos
Ifako-Ijaye	Lagos
Ikeja	Lagos
Badagry	Lagos
Ojo	Lagos
Alimosho	Lagos
Alimosho	Lagos
Kosofe	Lagos
Ikorodu	Lagos
Alimosho	Lagos
Ikeja	Lagos
Lagos Island	Lagos
Apapa	Lagos
Apapa	Lagos

Ojo	Lagos
Agege	Lagos
Agege	Lagos
Ikeja	Lagos
Badagry	Lagos
Alimosho	Lagos
Alimosho	Lagos
Alimosho	Lagos
Alimosho	Lagos
Alimosho	Lagos
Ojo	Lagos
Ikorodu	Lagos
Alimosho	Lagos
Ikorodu	Lagos
Badagry	Lagos
Kosofe	Lagos
Ojo	Lagos
Ikeja	Lagos
Agege	Lagos
Oshodi/Isolo	Lagos

Agege	Lagos
Surulere	Lagos
Mushin	Lagos
Ojo	Lagos
Oshodi/Isolo	Lagos
Kosofe	Lagos
Apapa	Lagos
Somolu	Lagos
Alimosho	Lagos
Ikorodu	Lagos
Badagry	Lagos
Alimosho	Lagos
Apapa	Lagos
Alimosho	Lagos
Mushin	Lagos
Oshodi/Isolo	Lagos
Lagos Island	Lagos
Ojo	Lagos
Ajeromi/Ifelodun	Lagos
Oshodi/Isolo	Lagos

Ojo	Lagos
Agege	Lagos
Amuwo-Odofin	Lagos
Ikeja	Lagos
Ajeromi/Ifelodun	Lagos
Ifako-Ijaye	Lagos
Ojo	Lagos
Ojo	Lagos
Ajeromi/Ifelodun	Lagos
Surulere	Lagos
Kosofe	Lagos
Ikorodu	Lagos
Ifako-Ijaye	Lagos
Ikorodu	Lagos
Alimosho	Lagos
Alimosho	Lagos
Alimosho	Lagos
Oshodi/Isolo	Lagos
Ojo	Lagos

Ajeromi/Ifelodun	Lagos
Ikeja	Lagos
Alimosho	Lagos
Ikeja	Lagos
Kosofe	Lagos
Ikorodu	Lagos
Ikorodu	Lagos
Mushin	Lagos
Ikeja	Lagos
Lagos Mainland	Lagos
Kosofe	Lagos
Alimosho	Lagos
Ikeja	Lagos
Alimosho	Lagos
Surulere	Lagos
Kosofe	Lagos
Badagry	Lagos
Badagry	Lagos
Amuwo-Odofin	Lagos
Ojo	Lagos
Alimosho	Lagos

Mushin	Lagos
Surulere	Lagos
Agege	Lagos
Alimosho	Lagos
Alimosho	Lagos
Oshodi/Isolo	Lagos
Ojo	Lagos
Alimosho	Lagos
Agege	Lagos
Agege	Lagos
Surulere	Lagos
Lagos Island	Lagos
Surulere	Lagos
Alimosho	Lagos
Alimosho	Lagos
Alimosho	Lagos
Ikorodu	Lagos
Amuwo-Odofin	Lagos
Epe	Lagos
Alimosho	Lagos
Ikorodu	Lagos

Badagry	Lagos
Alimosho	Lagos
Ifako-Ijaye	Lagos
Ifako-Ijaye	Lagos
Ajeromi/Ifelodun	Lagos
Agege	Lagos
Amuwo-Odofin	Lagos
Alimosho	Lagos
Ifako-Ijaye	Lagos
Ikorodu	Lagos
Ifako-Ijaye	Lagos
Mushin	Lagos
Ikorodu	Lagos
Ikeja	Lagos
Somolu	Lagos
Alimosho	Lagos
Alimosho	Lagos
Ibeju/Lekki	Lagos
Eti-Osa	Lagos
Surulere	Lagos
Agege	Lagos
Badagry	Lagos
Ikeja	Lagos
Oshodi/Isolo	Lagos

Alimosho	Lagos
Alimosho	Lagos
Amuwo-Odofin	Lagos
Alimosho	Lagos
Ikeja	Lagos
Ojo	Lagos
Oshodi/Isolo	Lagos
Ifako-Ijaye	Lagos
Ifako-Ijaye	Lagos
Badagry	Lagos
Kosofe	Lagos
Alimosho	Lagos
Amuwo-Odofin	Lagos
Oshodi/Isolo	Lagos
Amuwo-Odofin	Lagos
Ojo	Lagos
Ajeromi/Ifelodun	Lagos
Ajeromi/Ifelodun	Lagos
Alimosho	Lagos
Kosofe	Lagos
Kosofe	Lagos
Kosofe	Lagos

Kosofe	Lagos
Alimosho	Lagos
Surulere	Lagos
Oshodi/Isolo	Lagos
Kosofe	Lagos
Ikorodu	Lagos
Alimosho	Lagos
Alimosho	Lagos
Ojo	Lagos
Oshodi/Isolo	Lagos
Mushin	Lagos
Surulere	Lagos
Eti-Osa	Lagos
Amuwo-Odofin	Lagos
Kosofe	Lagos
Ikorodu	Lagos
Ikorodu	Lagos
Amuwo-Odofin	Lagos
Ifako-Ijaye	Lagos
Ikeja	Lagos

Badagry	Lagos
Alimosho	Lagos
Oshodi/Isolo	Lagos
Mushin	Lagos
Eti-Osa	Lagos
Alimosho	Lagos
Alimosho	Lagos
Badagry	Lagos
Surulere	Lagos
Eti-Osa	Lagos
Lagos Island	Lagos
Surulere	Lagos
Ikorodu	Lagos
Kosofe	Lagos
Alimosho	Lagos
Amuwo-Odofin	Lagos
Ojo	Lagos
Surulere	Lagos
Ikorodu	Lagos

Ikorodu	Lagos
Alimosho	Lagos
Alimosho	Lagos
Ikorodu	Lagos
Alimosho	Lagos
Ikeja	Lagos
Somolu	Lagos
Kosofe	Lagos
Alimosho	Lagos
Ikorodu	Lagos
Kosofe	Lagos
Ojo	Lagos
Alimosho	Lagos
Ifako-Ijaye	Lagos
Alimosho	Lagos
Alimosho	Lagos
Ifako-Ijaye	Lagos
Ikorodu	Lagos

Kosofe	Lagos
Oshodi/Isolo	Lagos
Badagry	Lagos
Ibeju/Lekki	Lagos
Somolu	Lagos
Somolu	Lagos
Kosofe	Lagos
Oshodi/Isolo	Lagos
Agege	Lagos
Ikorodu	Lagos
Ifako-Ijaye	Lagos
Alimosho	Lagos
Kosofe	Lagos
Eti-Osa	Lagos
Surulere	Lagos
Surulere	Lagos
Ibeju/Lekki	Lagos
Agaie	Niger
Bida	Niger
Bida	Niger
Bida	Niger

Bosso	Niger
Chanchaga	Niger
Chanchaga	Niger
Chanchaga	Niger
Chanchaga	Niger
Chanchaga	Niger
Chanchaga	Niger
Chanchaga	Niger
Chanchaga	Niger
Chanchaga	Niger
Chanchaga	Niger
Mokwa	Niger
Chanchaga	Niger
Chanchaga	Niger
Kontagora	Niger
Kontagora	Niger
Lapai	Niger
Suleja	Niger
Suleja	Niger
Suleja	Niger
Suleja	Niger
Suleja	Niger
Suleja	Niger
Suleja	Niger
Suleja	Niger
Wushishi	Niger
Wushishi	Niger
Gbako	Niger
Mariga	Niger
Magama	Niger
Bosso	Niger
Chanchaga	Niger
Bida	Niger
Chanchaga	Niger
Gurara	Niger
Chanchaga	Niger
Kontagora	Niger
Lapai	Niger
Agaie	Niger

Chanchaga	Niger
Chanchaga	Niger
Bida	Niger
Chanchaga	Niger
Chanchaga	Niger
Suleja	Niger
Suleja	Niger
Suleja	Niger
Chanchaga	Niger
Bosso	Niger
Bosso	Niger
Chanchaga	Niger
Bida	Niger
Kontagora	Niger
Kontagora	Niger
Suleja	Niger
Bida	Niger
Chanchaga	Niger
Kontagora	Niger
Suleja	Niger
Borgu	Niger
Kontagora	Niger
Bida	Niger
Kontagora	Niger
Suleja	Niger
Shiroro	Niger
Gurara	Niger
Paikoro	Niger
Bida	Niger
Kontagora	Niger
Chanchaga	Niger
Chanchaga	Niger
Chanchaga	Niger
Chanchaga	Niger
Chanchaga	Niger
Chanchaga	Niger
Chanchaga	Niger
Chanchaga	Niger

Chanchaga	Niger
Chanchaga	Niger
Chanchaga	Niger
Chanchaga	Niger
Chanchaga	Niger
Chanchaga	Niger
Chanchaga	Niger
Chanchaga	Niger
Lapai	Niger
Lapai	Niger
Lapai	Niger
Lapai	Niger
Lapai	Niger
Lapai	Niger
Lapai	Niger
Lapai	Niger
Lapai	Niger
Lapai	Niger
Lapai	Niger
Lapai	Niger
Lapai	Niger
Lapai	Niger
Lapai	Niger
Lapai	Niger
Lapai	Niger
Lapai	Niger
Lapai	Niger
Agwara	Niger
Agwara	Niger
Agwara	Niger
Agwara	Niger
Agwara	Niger
Agwara	Niger
Agwara	Niger
Agwara	Niger
Agwara	Niger
Agwara	Niger
Lapai	Niger
Lapai	Niger
Lapai	Niger
Lapai	Niger
Lapai	Niger
Lapai	Niger
Lapai	Niger
Lapai	Niger
Lapai	Niger
Lapai	Niger
Lapai	Niger
Lapai	Niger
Lapai	Niger

Lapai	Niger
Lapai	Niger
Lapai	Niger
Lapai	Niger
Lapai	Niger
Lapai	Niger
Lapai	Niger
Agwara	Niger
Agwara	Niger
Agwara	Niger
Agwara	Niger
Agwara	Niger
Agwara	Niger
Suleja	Niger
Suleja	Niger
Borgu	Niger
Agwara	Niger
Agwara	Niger
Bosso	Niger
Chanchaga	Niger
Borgu	Niger
Lapai	Niger
Kontagora	Niger
Kontagora	Niger
Chanchaga	Niger
Lapai	Niger
Suleja	Niger
Gbako	Niger
Kontagora	Niger
Chanchaga	Niger
Lapai	Niger
Tafa	Niger
Chanchaga	Niger

Borgu	Niger
Suleja	Niger
Suleja	Niger
Chanchaga	Niger
Bosso	Niger
Suleja	Niger
Suleja	Niger
Suleja	Niger
Suleja	Niger
Suleja	Niger
Suleja	Niger
Bida	Niger
Karu	Nasarawa
Keffi	Nasarawa
Karu	Nasarawa
Karu	Nasarawa
Keffi	Nasarawa
Lafia	Nasarawa
Karu	Nasarawa
Akwanga	Nasarawa
Akwanga	Nasarawa
Nasarawa Eggon	Nasarawa
Keana	Nasarawa
Awe	Nasarawa
Obi	Nasarawa
Wamba	Nasarawa
Lafia	Nasarawa
Lafia	Nasarawa
Lafia	Nasarawa
Karu	Nasarawa
Karu	Nasarawa
Keffi	Nasarawa

Keffi	Nasarawa
Karu	Nasarawa
Nasarawa	Nasarawa
Karu	Nasarawa
Karu	Nasarawa
Lafia	Nasarawa
Toto	Nasarawa
Nasarawa	Nasarawa
Nasarawa	Nasarawa
Nasarawa	Nasarawa
Keffi	Nasarawa
Akwanga	Nasarawa
Akwanga	Nasarawa
Karu	Nasarawa
Lafia	Nasarawa
Karu	Nasarawa
Karu	Nasarawa
Karu	Nasarawa
Karu	Nasarawa
Keffi	Nasarawa
Lafia	Nasarawa
Nasarawa	Nasarawa
Karu	Nasarawa
Karu	Nasarawa
Karu	Nasarawa
Karu	Nasarawa
Karu	Nasarawa
Doma	Nasarawa
Karu	Nasarawa
Karu	Nasarawa
Karu	Nasarawa

Karu	Nasarawa
Karu	Nasarawa
Lafia	Nasarawa
Lafia	Nasarawa
Karu	Nasarawa
Lafia	Nasarawa
Lafia	Nasarawa
Keffi	Nasarawa
Nasarawa	Nasarawa
Karu	Nasarawa
Lafia	Nasarawa
Karu	Nasarawa
Lafia	Nasarawa
Keffi	Nasarawa
Keffi	Nasarawa
Lafia	Nasarawa
Akure South	Ondo
Akoko North East	Ondo
Akoko South East	Ondo
Akure North	Ondo
Akure North	Ondo
Akure North	Ondo
Akure North	Ondo
Akure North	Ondo
Akure North	Ondo

Akure North	Ondo
Okitipupa	Ondo
Owo	Ondo
Akure North	Ondo
Akure North	Ondo
Akure North	Ondo
Akure South	Ondo
Akure North	Ondo
Akure South	Ondo
Akure South	Ondo
Akure South	Ondo
Akure South	Ondo
Ondo West	Ondo
Akure South	Ondo
Akure South	Ondo
Ose	Ondo
Okitipupa	Ondo
Ondo West	Ondo
Ileoluji/Okeigbo	Ondo
Ondo West	Ondo
Akure South	Ondo
Akure South	Ondo
Ondo West	Ondo
Ondo West	Ondo
Akure North	Ondo
Akure South	Ondo
Akure South	Ondo
Odigbo	Ondo
Akure South	Ondo
Akoko North East	Ondo
Ondo West	Ondo
Akoko North East	Ondo
Akure South	Ondo
Ondo West	Ondo
Okitipupa	Ondo
Ondo West	Ondo

Ondo West	Ondo
Akure South	Ondo
Akure South	Ondo
Odigbo	Ondo
Ileoluji/Okeigbo	Ondo
Akure North	Ondo
Akure South	Ondo
Akure South	Ondo
Ilaje	Ondo
Ondo West	Ondo
Odigbo	Ondo
Owo	Ondo
Akure South	Ondo
Ondo West	Ondo
Akure South	Ondo
Owo	Ondo
Odigbo	Ondo
Ondo West	Ondo
Okitipupa	Ondo
Akoko South West	Ondo
Odigbo	Ondo
Akure South	Ondo
Owo	Ondo
Odigbo	Ondo
Ondo West	Ondo
Owo	Ondo
Ifedore	Ondo

Owo	Ondo
Ondo West	Ondo
Idanre	Ondo
Akure South	Ondo
Akure South	Ondo
Akure South	Ondo
Ondo West	Ondo
Akure South	Ondo
Akure South	Ondo
Akure South	Ondo
Owo	Ondo
Odigbo	Ondo
Abeokuta South	Ogun
Abeokuta South	Ogun
Ijebu Ode	Ogun
Abeokuta South	Ogun
Abeokuta South	Ogun
Abeokuta North	Ogun
Ifo	Ogun
Ijebu Ode	Ogun
Abeokuta South	Ogun
Ado Odo-Ota	Ogun
Ado Odo-Ota	Ogun
Ado Odo-Ota	Ogun
Ado Odo-Ota	Ogun
Abeokuta North	Ogun
Abeokuta North	Ogun
Ijebu North	Ogun
Egbado North	Ogun
Egbado North	Ogun
Abeokuta North	Ogun
Abeokuta South	Ogun

Ipokia	Ogun
Abeokuta North	Ogun
Egbado South	Ogun
Abeokuta North	Ogun
Ado Odo-Ota	Ogun
Ipokia	Ogun
Ado Odo-Ota	Ogun
Ado Odo-Ota	Ogun
Ado Odo-Ota	Ogun
Ado Odo-Ota	Ogun
Sagamu	Ogun
Ado Odo-Ota	Ogun
Abeokuta South	Ogun
Ifo	Ogun
Ifo	Ogun
Ado Odo-Ota	Ogun
Sagamu	Ogun
Abeokuta South	Ogun
Sagamu	Ogun
Odeda	Ogun
Sagamu	Ogun
Ijebu Ode	Ogun
Obafemi/Owode	Ogun
Abeokuta South	Ogun
Abeokuta North	Ogun
Ado Odo-Ota	Ogun
Ado Odo-Ota	Ogun
Ado Odo-Ota	Ogun
Ado Odo-Ota	Ogun
Ado Odo-Ota	Ogun
Ado Odo-Ota	Ogun
Ado Odo-Ota	Ogun
Ado Odo-Ota	Ogun
Ado Odo-Ota	Ogun
Ado Odo-Ota	Ogun
Abeokuta North	Ogun
Sagamu	Ogun
Egbado South	Ogun

Abeokuta North	Ogun
Abeokuta South	Ogun
Sagamu	Ogun
Abeokuta South	Ogun
Ifo	Ogun
Sagamu	Ogun
Sagamu	Ogun
Abeokuta South	Ogun
Ijebu Ode	Ogun
Abeokuta South	Ogun
Egbado South	Ogun
Abeokuta South	Ogun
Abeokuta South	Ogun
Ikenne	Ogun
Ifo	Ogun
Abeokuta South	Ogun
Ijebu Ode	Ogun
Sagamu	Ogun
Abeokuta North	Ogun
Ifo	Ogun
Remo North	Ogun
Ijebu Ode	Ogun
Ijebu Ode	Ogun
Ijebu Ode	Ogun
Ifo	Ogun
Ifo	Ogun
Obafemi/Owode	Ogun

Abeokuta North	Ogun
Abeokuta South	Ogun
Ado Odo-Ota	Ogun
Ifo	Ogun
Ijebu Ode	Ogun
Ijebu Ode	Ogun
Ifo	Ogun
Ijebu Ode	Ogun
Abeokuta South	Ogun
Sagamu	Ogun
Ifo	Ogun
Ado Odo-Ota	Ogun
Obafemi/Owode	Ogun
Abeokuta South	Ogun
Ifo	Ogun
Ifo	Ogun
Ikenne	Ogun
Odogbolu	Ogun
Ijebu Ode	Ogun
Egbado South	Ogun
Ado Odo-Ota	Ogun
Ijebu North	Ogun
Obafemi/Owode	Ogun
Ifo	Ogun
Abeokuta South	Ogun
Ifo	Ogun
Ijebu East	Ogun
Abeokuta South	Ogun

Abeokuta North	Ogun
Ijebu Ode	Ogun
Abeokuta North	Ogun
Abeokuta North	Ogun
Abeokuta North	Ogun
Ado Odo-Ota	Ogun
Ado Odo-Ota	Ogun
Ado Odo-Ota	Ogun
Sagamu	Ogun
Ifo	Ogun
Sagamu	Ogun
Abeokuta North	Ogun
Ifo	Ogun
Sagamu	Ogun
Ijebu Ode	Ogun
Abeokuta North	Ogun
Sagamu	Ogun
Abeokuta South	Ogun
Sagamu	Ogun
Obafemi/Owode	Ogun
Obafemi/Owode	Ogun
Abeokuta North	Ogun

Obafemi/Owode	Ogun
Ado Odo-Ota	Ogun
Ifo	Ogun
Ijebu Ode	Ogun
Abeokuta South	Ogun
Sagamu	Ogun
Obafemi/Owode	Ogun
Ifo	Ogun
Obafemi/Owode	Ogun
Ado Odo-Ota	Ogun
Remo North	Ogun
Ijebu Ode	Ogun
Ado Odo-Ota	Ogun
Ifo	Ogun
Abeokuta South	Ogun
Abeokuta North	Ogun
Obafemi/Owode	Ogun
Ijebu Ode	Ogun
Ijebu Ode	Ogun

Obafemi/Owode	Ogun
Obafemi/Owode	Ogun
Ifo	Ogun
Ifo	Ogun
Abeokuta South	Ogun
Ado Odo-Ota	Ogun
Abeokuta South	Ogun
Ifo	Ogun
Sagamu	Ogun
Abeokuta South	Ogun
Ado Odo-Ota	Ogun
Sagamu	Ogun
Ila	Osun
Ife Central	Osun
Ilesa East	Osun
Ilesa East	Osun
Iwo	Osun
Ifelodun	Osun
Olorunda	Osun
Osogbo	Osun
Osogbo	Osun
Osogbo	Osun
Olorunda	Osun
Olorunda	Osun
Ife Central	Osun
Ilesa East	Osun
Ife Central	Osun
Ede South	Osun
Olorunda	Osun

Isokan	Osun
Osogbo	Osun
Ede South	Osun
Ede North	Osun
Ife Central	Osun
Osogbo	Osun
Ife East	Osun
Ayedaade	Osun
Ede South	Osun
Ife Central	Osun
Ife Central	Osun
Olorunda	Osun
Osogbo	Osun
Ilesa East	Osun
Irewole	Osun
Ife North	Osun
Olorunda	Osun
Osogbo	Osun
Ife Central	Osun
Isokan	Osun
Odo-Otin	Osun
Iwo	Osun
Osogbo	Osun
Ife Central	Osun
Ede South	Osun
Ede North	Osun
Olorunda	Osun
Iwo	Osun
Ifelodun	Osun
Osogbo	Osun

Osogbo	Osun
Egbedore	Osun
Ifelodun	Osun
Olorunda	Osun
Ife Central	Osun
Osogbo	Osun
Egbedore	Osun
Ife North	Osun
Ife East	Osun
Ifelodun	Osun
Ife East	Osun
Osogbo	Osun
Egbedore	Osun
Osogbo	Osun
Ife East	Osun
Ede South	Osun
Ede South	Osun
Egbedore	Osun
Egbedore	Osun
Olorunda	Osun
Ede South	Osun
Ifelodun	Osun
Odo-Otin	Osun
Egbedore	Osun

Osogbo	Osun
Egbedore	Osun
Odo-Otin	Osun
Egbedore	Osun
Osogbo	Osun
Osogbo	Osun
Osogbo	Osun
Osogbo	Osun
Osogbo	Osun
Ibadan North	Oyo
Ibadan North	Oyo
Ibadan North	Oyo
Ibadan North	Oyo
Ibadan North	Oyo
Ibadan North	Oyo
Ibadan North	Oyo
Ibadan North	Oyo
Ibadan North	Oyo
Ibadan North	Oyo
Ibadan North	Oyo
Ibadan North	Oyo
Ibadan North East	Oyo
Ibadan North	Oyo
Ibadan South West	Oyo
Ibadan South West	Oyo
Ibadan South West	Oyo
Ibadan South West	Oyo

Ibadan South West	Oyo
Ibadan South West	Oyo
Ibadan South West	Oyo
Ibadan South West	Oyo
Ibadan South West	Oyo
Ibadan North	Oyo
Ibadan North	Oyo
Ibadan North	Oyo
Ibadan South-East	Oyo
Ibadan South-East	Oyo
Ibadan North	Oyo
Ibadan South West	Oyo
Ibadan South West	Oyo
Ibadan North	Oyo
Ibadan South West	Oyo
Ibadan North East	Oyo
Ibadan North	Oyo
Ibadan North East	Oyo
Ibadan North	Oyo
Ibadan South West	Oyo
Ibadan North East	Oyo
Ibadan North East	Oyo
Ibadan North	Oyo
Ibadan North	Oyo
Ibadan South West	Oyo
Ibadan North	Oyo
Ibadan South West	Oyo
Ibadan North East	Oyo
Ibadan North	Oyo

Oyo West	Oyo
Afijio	Oyo
Ogbomoso South	Oyo
Ibadan North	Oyo
Ibadan South West	Oyo
Ibadan North	Oyo
Ibadan North	Oyo
Saki West	Oyo
Ogbomoso North	Oyo
Iseyin	Oyo
Iseyin	Oyo
Ibadan North	Oyo
Ibadan South West	Oyo
Ibadan North	Oyo
Oyo West	Oyo
Ogbomoso South	Oyo
Ibadan South West	Oyo
Saki West	Oyo
Saki East	Oyo
Ibadan South-East	Oyo
Saki West	Oyo
Ibadan North	Oyo
Ibadan North	Oyo
Ibadan North West	Oyo
Ibadan South West	Oyo
Ibadan North	Oyo
Ibadan North East	Oyo
Ibadan South West	Oyo
Ibadan South West	Oyo
Ibadan South West	Oyo
Ibadan South West	Oyo
Akinyele	Oyo
Oyo West	Oyo
Ibadan South-East	Oyo
Iseyin	Oyo

Ogbomoso North	Oyo
Oyo East	Oyo
Ibadan North West	Oyo
Ogbomoso North	Oyo
Ogbomoso North	Oyo
Ido	Oyo
Lagelu	Oyo
Egbeda	Oyo
Ibadan North West	Oyo
Ibadan North West	Oyo
Ibadan North	Oyo
Ogbomoso North	Oyo
Ibadan South West	Oyo
Afijio	Oyo
Ibadan North	Oyo
Ibadan South West	Oyo
Ibadan North East	Oyo
Ibadan North	Oyo
Ibadan North	Oyo
Oyo West	Oyo
Ibadan North	Oyo
Ibadan North	Oyo
Ibadan North West	Oyo
Ido	Oyo
Ibadan South-East	Oyo

Ibadan North West	Oyo
Ibadan North West	Oyo
Lagelu	Oyo
Ibadan North	Oyo
Ibadan South West	Oyo
Ibadan North	Oyo
Ibadan North East	Oyo
Ibadan South West	Oyo
Ibadan South West	Oyo
Ibadan South West	Oyo
Ibadan North East	Oyo
Egbeda	Oyo
Ibadan North	Oyo
Ibadan South West	Oyo
Oluyole	Oyo
Oluyole	Oyo
Akinyele	Oyo
Ibadan South West	Oyo
Ibadan North West	Oyo
Ibadan North	Oyo
Ibadan South West	Oyo
Oyo West	Oyo
Egbeda	Oyo
Ibadan North	Oyo
Ibadan South-East	Oyo
Ibadan South West	Oyo

Ido	Oyo
Ibadan North	Oyo
Ibadan North East	Oyo
Ibadan South West	Oyo
Ibadan North	Oyo
Lagelu	Oyo
Ibadan North	Oyo
Ibadan North East	Oyo
Ibadan North	Oyo
Oluyole	Oyo
Ibadan North	Oyo
Lagelu	Oyo
Ibadan South West	Oyo
Ibadan South West	Oyo
Ibadan North	Oyo
Egbeda	Oyo
Akinyele	Oyo
Ogbomoso South	Oyo
Ogbomoso South	Oyo
Ibadan South West	Oyo
Ibadan South-East	Oyo
Ibadan South-East	Oyo
Ibadan South-East	Oyo
Ibadan South-East	Oyo
Atiba	Oyo
Atiba	Oyo
Atiba	Oyo
Atiba	Oyo
Ogbomoso North	Oyo
Ogbomoso North	Oyo
Atiba	Oyo
Atiba	Oyo
Atiba	Oyo

Irepo	Oyo
Ogbomoso North	Oyo
Ogbomoso North	Oyo
Ogbomoso North	Oyo
Ogbomoso North	Oyo
Ibadan North East	Oyo
Ibadan North	Oyo
Ibadan North	Oyo
Ibadan North	Oyo
Ibadan North	Oyo
Ibadan North East	Oyo
Ibadan South-East	Oyo
Ibadan North	Oyo
Oluyole	Oyo
Akinyele	Oyo
Ibadan South-East	Oyo
Ibadan South-East	Oyo
Ibadan South West	Oyo
Egbeda	Oyo
Ibadan North East	Oyo
Ibadan South-East	Oyo
Ibadan North	Oyo
Ibadan North	Oyo
Ibadan North	Oyo
Ibadan North East	Oyo
Ido	Oyo
Oluyole	Oyo
Lagelu	Oyo
Ibadan South West	Oyo

Lagelu	Oyo
Lagelu	Oyo
Atiba	Oyo
Irepo	Oyo
Ibadan North	Oyo
Ibadan South West	Oyo
Oluyole	Oyo
Oluyole	Oyo
Ido	Oyo
Ido	Oyo
Ibadan South West	Oyo
Ibadan North	Oyo
Ido	Oyo
Ido	Oyo
Ibadan North West	Oyo
Ido	Oyo
Ibadan North	Oyo
Ibadan South West	Oyo
Ibadan South West	Oyo
Ibadan South West	Oyo
Ibadan North West	Oyo
Lagelu	Oyo

Ibadan North East	Oyo
Ibadan North	Oyo
Ogbomoso North	Oyo
Ibadan North East	Oyo
Oluyole	Oyo
Ibadan South West	Oyo
Ibadan South West	Oyo
Oyo East	Oyo
Akinyele	Oyo
Ibadan North	Oyo
Ibadan North	Oyo
Ibadan South West	Oyo
Oluyole	Oyo
Atiba	Oyo
Ibadan South West	Oyo
Ibadan North	Oyo
Ibadan South West	Oyo
Ibadan South West	Oyo
Egbeda	Oyo
Ibadan South West	Oyo
Ibadan South West	Oyo
Ogbomoso South	Oyo
Ibadan South West	Oyo

Ibadan North	Oyo
Ido	Oyo
Saki West	Oyo
Ido	Oyo
Egbeda	Oyo
Ido	Oyo
Lagelu	Oyo
Ogbomoso North	Oyo
Ogbomoso North	Oyo
Ibadan North	Oyo
Ido	Oyo
Ibadan South-East	Oyo
Ibadan North	Oyo
Barikin Ladi	Plateau
Bassa	Plateau
Bokkos	Plateau
Jos North	Plateau
Jos North	Plateau
Jos North	Plateau
Jos North	Plateau
Jos North	Plateau
Pankshin	Plateau
Pankshin	Plateau
Jos North	Plateau
Jos North	Plateau
Barikin Ladi	Plateau
Shendam	Plateau
Jos North	Plateau
Jos North	Plateau
Jos North	Plateau
Jos North	Plateau

Shendam	Plateau
Jos North	Plateau
Jos North	Plateau
Jos North	Plateau
Pankshin	Plateau
Shendam	Plateau
Mangu	Plateau
Barikin Ladi	Plateau
Jos North	Plateau
Shendam	Plateau
Jos North	Plateau
Jos North	Plateau
Jos South	Plateau
Jos South	Plateau
Jos North	Plateau
Jos South	Plateau
Jos North	Plateau
Jos North	Plateau
Jos North	Plateau
Jos South	Plateau
Pankshin	Plateau
Jos South	Plateau
Jos North	Plateau
Jos South	Plateau
Qua'An Pan	Plateau
Pankshin	Plateau
Jos South	Plateau
Jos North	Plateau
Jos North	Plateau
Jos North	Plateau
Jos South	Plateau
Jos South	Plateau
Jos North	Plateau
Jos South	Plateau
Jos South	Plateau
Jos North	Plateau
Jos South	Plateau
Jos North	Plateau
Jos South	Plateau
Jos North	Plateau

Jos South	Plateau
Jos South	Plateau
Jos South	Plateau
Jos South	Plateau
Bassa	Kogi
Jos South	Plateau
Jos North	Plateau
Pankshin	Plateau
Jos South	Plateau
Jos South	Plateau
Jos North	Plateau
Langtang North	Plateau
Jos North	Plateau
Qua'An Pan	Plateau
Mangu	Plateau
Wase	Plateau
Jos East	Plateau
Kanam	Plateau
Mikang	Plateau
Mikang	Plateau
Port Harcourt	Rivers
Port Harcourt	Rivers
Port Harcourt	Rivers
Obio/Akpor	Rivers
Obio/Akpor	Rivers
Port Harcourt	Rivers
Obio/Akpor	Rivers

Obio/Akpor	Rivers
Port Harcourt	Rivers
Obio/Akpor	Rivers
Obio/Akpor	Rivers
Obio/Akpor	Rivers
Obio/Akpor	Rivers
Obio/Akpor	Rivers
Obio/Akpor	Rivers
Obio/Akpor	Rivers
Obio/Akpor	Rivers
Obio/Akpor	Rivers
Obio/Akpor	Rivers
Obio/Akpor	Rivers
Port Harcourt	Rivers
Port Harcourt	Rivers
Port Harcourt	Rivers
Obio/Akpor	Rivers
Port Harcourt	Rivers
Port Harcourt	Rivers
Port Harcourt	Rivers
Port Harcourt	Rivers
Port Harcourt	Rivers
Port Harcourt	Rivers
Port Harcourt	Rivers
Port Harcourt	Rivers
Port Harcourt	Rivers
Port Harcourt	Rivers

Port Harcourt	Rivers
Port Harcourt	Rivers
Obio/Akpor	Rivers
Port Harcourt	Rivers
Obio/Akpor	Rivers
Obio/Akpor	Rivers
Obio/Akpor	Rivers
Port Harcourt	Rivers
Port Harcourt	Rivers
Obio/Akpor	Rivers
Port Harcourt	Rivers
Port Harcourt	Rivers
Port Harcourt	Rivers
Obio/Akpor	Rivers
Port Harcourt	Rivers
Obio/Akpor	Rivers
Obio/Akpor	Rivers
Port Harcourt	Rivers
Obio/Akpor	Rivers
Port Harcourt	Rivers
Port Harcourt	Rivers
Obio/Akpor	Rivers
Port Harcourt	Rivers
Obio/Akpor	Rivers
Obio/Akpor	Rivers
Obio/Akpor	Rivers
Port Harcourt	Rivers
Obio/Akpor	Rivers
Port Harcourt	Rivers
Obio/Akpor	Rivers
Obio/Akpor	Rivers

Obio/Akpor	Rivers
Obio/Akpor	Rivers
Obio/Akpor	Rivers
Port Harcourt	Rivers
Port Harcourt	Rivers
Port Harcourt	Rivers
Port Harcourt	Rivers
Port Harcourt	Rivers
Port Harcourt	Rivers
Port Harcourt	Rivers
Port Harcourt	Rivers
Obio/Akpor	Rivers
Port Harcourt	Rivers
Port Harcourt	Rivers
Port Harcourt	Rivers
Port Harcourt	Rivers
Port Harcourt	Rivers
Port Harcourt	Rivers
Port Harcourt	Rivers
Port Harcourt	Rivers
Port Harcourt	Rivers
Port Harcourt	Rivers
Port Harcourt	Rivers
Obio/Akpor	Rivers
Obio/Akpor	Rivers
Obio/Akpor	Rivers
Obio/Akpor	Rivers
Obio/Akpor	Rivers
Obio/Akpor	Rivers
Obio/Akpor	Rivers
Port Harcourt	Rivers
Port Harcourt	Rivers

Port Harcourt	Rivers
Port Harcourt	Rivers
Port Harcourt	Rivers
Port Harcourt	Rivers
Port Harcourt	Rivers
Port Harcourt	Rivers
Port Harcourt	Rivers
Port Harcourt	Rivers
Obio/Akpor	Rivers
Obio/Akpor	Rivers
Obio/Akpor	Rivers
Oyigbo	Rivers
Port Harcourt	Rivers
Port Harcourt	Rivers
Port Harcourt	Rivers
Oyigbo	Rivers
Ogba/Egbema/Ndoni	Rivers
Ogba/Egbema/Ndoni	Rivers
Port Harcourt	Rivers
Port Harcourt	Rivers
Ikwerre	Rivers
Port Harcourt	Rivers
Port Harcourt	Rivers
Obio/Akpor	Rivers
Port Harcourt	Rivers
Port Harcourt	Rivers

Port Harcourt	Rivers
Obio/Akpor	Rivers
Port Harcourt	Rivers
Port Harcourt	Rivers
Obio/Akpor	Rivers
Port Harcourt	Rivers
Port Harcourt	Rivers
Obio/Akpor	Rivers
Obio/Akpor	Rivers
Obio/Akpor	Rivers
Obio/Akpor	Rivers
Port Harcourt	Rivers
Obio/Akpor	Rivers
Port Harcourt	Rivers
Obio/Akpor	Rivers
Obio/Akpor	Rivers
Ogba/Egbema/Ndoni	Rivers
Obio/Akpor	Rivers
Obio/Akpor	Rivers
Obio/Akpor	Rivers
Obio/Akpor	Rivers

Port Harcourt	Rivers
Port Harcourt	Rivers
Obio/Akpor	Rivers
Obio/Akpor	Rivers
Port Harcourt	Rivers
Obio/Akpor	Rivers
Obio/Akpor	Rivers
Obio/Akpor	Rivers
Obio/Akpor	Rivers
Ahoada East	Rivers
Port Harcourt	Rivers
Port Harcourt	Rivers
Oyigbo	Rivers
Port Harcourt	Rivers
Obio/Akpor	Rivers
Obio/Akpor	Rivers
Obio/Akpor	Rivers
Obio/Akpor	Rivers
Obio/Akpor	Rivers

Obio/Akpor	Rivers
Obio/Akpor	Rivers
Obio/Akpor	Rivers
Obio/Akpor	Rivers
Obio/Akpor	Rivers
Obio/Akpor	Rivers
Obio/Akpor	Rivers
Obio/Akpor	Rivers
Obio/Akpor	Rivers
Obio/Akpor	Rivers
Obio/Akpor	Rivers
Bonny	Rivers
Obio/Akpor	Rivers
Obio/Akpor	Rivers
Obio/Akpor	Rivers
Obio/Akpor	Rivers
Obio/Akpor	Rivers
Obio/Akpor	Rivers
Obio/Akpor	Rivers
Obio/Akpor	Rivers

Obio/Akpor	Rivers
Obio/Akpor	Rivers
Obio/Akpor	Rivers
Port Harcourt	Rivers
Port Harcourt	Rivers
Tai	Rivers
Sokoto South	Sokoto
Sokoto South	Sokoto
Sokoto South	Sokoto
Sokoto South	Sokoto
Sokoto South	Sokoto
Sokoto South	Sokoto
Sokoto South	Sokoto
Sokoto South	Sokoto
Sokoto South	Sokoto
Sokoto North	Sokoto
Sokoto North	Sokoto
Wamakko	Sokoto
Sokoto North	Sokoto
Sokoto South	Sokoto
Sokoto South	Sokoto
Sokoto North	Sokoto
Sokoto South	Sokoto
Kebbe	Sokoto
Kebbe	Sokoto
S/Birni	Sokoto
S/Birni	Sokoto
S/Birni	Sokoto
S/Birni	Sokoto
S/Birni	Sokoto
S/Birni	Sokoto
S/Birni	Sokoto
S/Birni	Sokoto
S/Birni	Sokoto
S/Birni	Sokoto
S/Birni	Sokoto
S/Birni	Sokoto
S/Birni	Sokoto

S/Birni	Sokoto
S/Birni	Sokoto
S/Birni	Sokoto
S/Birni	Sokoto
S/Birni	Sokoto
S/Birni	Sokoto
S/Birni	Sokoto
S/Birni	Sokoto
S/Birni	Sokoto
S/Birni	Sokoto
S/Birni	Sokoto
S/Birni	Sokoto
S/Birni	Sokoto
S/Birni	Sokoto
Sokoto North	Sokoto
Sokoto North	Sokoto
Sokoto South	Sokoto
Sokoto South	Sokoto
Sokoto North	Sokoto
Sokoto South	Sokoto
Sokoto South	Sokoto
Wamakko	Sokoto
Jalingo	Taraba
Wukari	Taraba
Jalingo	Taraba
Jalingo	Taraba
Jalingo	Taraba
Wukari	Taraba
Wukari	Taraba
Jalingo	Taraba
Wukari	Taraba
Wukari	Taraba
Jalingo	Taraba
Zing	Taraba
Jalingo	Taraba
Takum	Taraba
Takum	Taraba

Takum	Taraba
Takum	Taraba
Jalingo	Taraba
Jalingo	Taraba
Bali	Taraba
Sardauna	Taraba
Jalingo	Taraba
Gassol	Taraba
Wukari	Taraba
Jalingo	Taraba
Jalingo	Taraba
Jalingo	Taraba
Gashaka	Taraba
Wukari	Taraba
Jalingo	Taraba
Wukari	Taraba
Bali	Taraba
Jalingo	Taraba
Jalingo	Taraba
Wukari	Taraba
Jalingo	Taraba
Jalingo	Taraba
Takum	Taraba
Jalingo	Taraba
Bade	Yobe
Bade	Yobe
Damaturu	Yobe
Damaturu	Yobe
Damaturu	Yobe
Damaturu	Yobe
Damaturu	Yobe
Gujba	Yobe
Nguru	Yobe
Potiskum	Yobe
Damaturu	Yobe
Geidam	Yobe

Geidam	Yobe
Nguru	Yobe
Damaturu	Yobe
Damaturu	Yobe
Potiskum	Yobe
Damaturu	Yobe
Potiskum	Yobe
Damaturu	Yobe
Gujba	Yobe
Fune	Yobe
Anka	Zamfara
Bakura	Zamfara
Gusau	Zamfara
Gusau	Zamfara
Gusau	Zamfara
Gusau	Zamfara
Gusau	Zamfara
Gusau	Zamfara
Kaura Namoda	Zamfara
Maru	Zamfara
Talata Mafara	Zamfara
Tsafe	Zamfara
Gusau	Zamfara
Gusau	Zamfara
Gusau	Zamfara
Kaura Namoda	Zamfara
Gusau	Zamfara
Gusau	Zamfara
Gusau	Zamfara
Gusau	Zamfara
Bungudu	Zamfara
