# Supplementary material for: Determinants and perception of health insurance participation among healthcare providers in Nigeria: A mixed-methods study
Source: PLoS One. 2021 Aug 4;16(8):e0255206. doi: 10.1371/journal.pone.0255206 (PMC8336839; doi:10.1371/journal.pone.0255206)
Supplement: S2 Appendix — Obtained online from NHIS website, October 2017. (PDF) [file pone.0255206.s002.pdf]

ACTIVE SECONDARY HCP AS AT JULY 19TH 2017

	HCP CODE	SEC HCP CODE	HCP NAME	SERVICE TYPE	ADDRES	LGA	STATE
1	AB/0001	AB/0001/S/3	Sancta Maria Specialist & Mat.		22 Constitutition Crescent Aba	Aba South	Abia
2	AB/0004	AB/0004/S/3	Abia State University Teaching Hospital		Aba	Aba South	Abia
3	AB/0004	AB/0004/S/7	Abia State University Teaching Hospital		Aba	Aba South	Abia
4	AB/0004	AB/0004/S/4	Abia State University Teaching Hospital		Aba	Aba South	Abia
5	AB/0004	AB/0004/S/6	Abia State University Teaching Hospital		Aba	Aba South	Abia
6	AB/0004	AB/0004/S/5	Abia State University Teaching Hospital		Aba	Aba South	Abia
7	AB/0004	AB/0004/S/2	Abia State University Teaching Hospital		Aba	Aba South	Abia
8	AB/0004	AB/0004/S/1	Abia State University Teaching Hospital		Aba	Aba South	Abia
9	AB/0005	AB/0005/S/5	Life Care Clinics Ltd		8, Ezinkwu Street, Aba	Aba South	Abia
10	AB/0005	AB/0005/S/3	Life Care Clinics Ltd		8, Ezinkwu Street, Aba	Aba South	Abia
11	AB/0005	AB/0005/S/1	Life Care Clinics Ltd		8, Ezinkwu Street, Aba	Aba South	Abia
12	AB/0006	AB/0006/S/1	New Era Specialist Hospital		213/215 Ezikiwe Road, Aba	Aba South	Abia
13	AB/0006	AB/0006/S/8	New Era Specialist Hospital		213/215 Ezikiwe Road, Aba	Aba South	Abia
14	AB/0006	AB/0006/S/2	New Era Specialist Hospital		213/215 Ezikiwe Road, Aba	Aba South	Abia
15	AB/0006	AB/0006/S/7	New Era Specialist Hospital		213/215 Ezikiwe Road, Aba	Aba South	Abia
16	AB/0006	AB/0006/S/5	New Era Specialist Hospital		213/215 Ezikiwe Road, Aba	Aba South	Abia
17	AB/0006	AB/0006/S/3	New Era Specialist Hospital		213/215 Ezikiwe Road, Aba	Aba South	Abia
18	AB/0014	AB/0014/S/3	Federal Medical Centre, Umuahia		Umuahia, Abia State	Aba North	Abia
19	AB/0014	AB/0014/S/4	Federal Medical Centre, Umuahia		Umuahia, Abia State	Aba North	Abia
20	AB/0014	AB/0014/S/7	Federal Medical Centre, Umuahia		Umuahia, Abia State	Aba North	Abia
21	AB/0014	AB/0014/S/1	Federal Medical Centre, Umuahia		Umuahia, Abia State	Aba North	Abia
22	AB/0014	AB/0014/S/2	Federal Medical Centre, Umuahia		Umuahia, Abia State	Aba North	Abia
23	AB/0014	AB/0014/S/5	Federal Medical Centre, Umuahia		Umuahia, Abia State	Aba North	Abia
24	AB/0014	AB/0014/S/6	Federal Medical Centre, Umuahia		Umuahia, Abia State	Aba North	Abia
25	AB/0014	AB/0014/S/8	Federal Medical Centre, Umuahia		Umuahia, Abia State	Aba North	Abia
26	AB/0014	AB/0014/S/15	Federal Medical Centre, Umuahia		Umuahia, Abia State	Aba North	Abia
27	AB/0019	AB/0019/S/6	Mendel Hospital & Diagnostic Centre Limited		20 Tenant Road, Abia	Aba South	Abia
28	AB/0019	AB/0019/S/5	Mendel Hospital & Diagnostic Centre Limited		20 Tenant Road, Abia	Aba South	Abia
29	AB/0019	AB/0019/S/3	Mendel Hospital & Diagnostic Centre Limited		20 Tenant Road, Abia	Aba South	Abia
30	AB/0020	AB/0020/S/3	Clehansan Hospital & Maternity		17 Osusu Rd, Aba.	Aba South	Abia
31	AB/0022	AB/0022/S/6	Alpha Inland Hospital		36 Glass Industry Road, Aba	Obingwa	Abia
32	AB/0023	AB/0023/S/3	Todac Clinic Ltd.		59/61 Okigwe Road,Aba	Aba North	Abia
33	AB/0023	AB/0023/S/5	Todac Clinic Ltd.		59/61 Okigwe Road,Aba	Aba North	Abia
34	AB/0023	AB/0023/S/1	Todac Clinic Ltd.		59/61 Okigwe Road,Aba	Aba North	Abia
35	AB/0023	AB/0023/S/14	Todac Clinic Ltd.		59/61 Okigwe Road,Aba	Aba North	Abia
36	AB/0023	AB/0023/S/6	Todac Clinic Ltd.		59/61 Okigwe Road,Aba	Aba North	Abia
37	AB/0024	AB/0024/S/4	Living Word Hospital		5/7 Umuocham Street, Aba	Aba South	Abia
38	AB/0024	AB/0024/S/6	Living Word Hospital		5/7 Umuocham Street, Aba	Aba South	Abia
39	AB/0024	AB/0024/S/3	Living Word Hospital		5/7 Umuocham Street, Aba	Aba South	Abia
40	AB/0024	AB/0024/S/2	Living Word Hospital		5/7 Umuocham Street, Aba	Aba South	Abia
41	AB/0024	AB/0024/S/1	Living Word Hospital		5/7 Umuocham Street, Aba	Aba South	Abia
42	AB/0024	AB/0024/S/7	Living Word Hospital		5/7 Umuocham Street, Aba	Aba South	Abia

43	AB/0025	AB/0025/S/5	Living Word Hospital		117 Ikot Ekpene Road, Aba	Obingwa	Abia
44	AB/0025	AB/0025/S/10	Living Word Hospital		117 Ikot Ekpene Road, Aba	Obingwa	Abia
45	AB/0025	AB/0025/S/6	Living Word Hospital		117 Ikot Ekpene Road, Aba	Obingwa	Abia
46	AB/0025	AB/0025/S/3	Living Word Hospital		117 Ikot Ekpene Road, Aba	Obingwa	Abia
47	AB/0028	AB/0028/S/1	Horstman Hospital		32, Okigwe Road, Aba	Aba North	Abia
48	AB/0028	AB/0028/S/7	Horstman Hospital		32, Okigwe Road, Aba	Aba North	Abia
49	AB/0028	AB/0028/S/11	Horstman Hospital		32, Okigwe Road, Aba	Aba North	Abia
50	AB/0028	AB/0028/S/10	Horstman Hospital		32, Okigwe Road, Aba	Aba North	Abia
51	AB/0031	AB/0031/S/1	Austin Grace Hospital	-	16, Okigwe Road, Aba	Aba North	Abia
52	AB/0031	AB/0031/S/2	Austin Grace Hospital	-	16, Okigwe Road, Aba	Aba North	Abia
53	AB/0034	AB/0034/S/1	St. Paul Hospital		6-12 St. Paul's Road, Umunggasi, Aba	Aba South	Abia
54	AB/0034	AB/0034/S/5	St. Paul Hospital		6-12 St. Paul's Road, Umunggasi, Aba	Aba South	Abia
55	AB/0035	AB/0035/S/4	Seven Days Adventist Hospital		Umuoba Road, Aba	Aba North	Abia
56	AB/0038	AB/0038/S/2	Hammersmith Medical Centre		Plots 3 Amauzkwu Layout, Umauhia	Umuahia North	Abia
57	AB/0041	AB/0041/S/3	New Era Hospital & Maternity		90b Oji River Street Umuzukwu Layout	Aba South	Abia
58	AB/0059	AB/0059/S/4	Bencare Pharmacy Nig. Ltd.	Pharmacy	26 Faulks Road, Aba	Aba South	Abia
59	AB/0060	AB/0060/S/4	Banthas Pharmacy & Stores Ltd.	Pharmacy	93, Okigwe Road, Aba	Aba North	Abia
60	AB/0062	AB/0062/S/4	Ceeje Pharmacy	Pharmacy	Mbalse Owerri Road, Aba	Aba South	Abia
61	AB/0064	AB/0064/S/4	Elegance Pharmacy	Pharmacy	37 Jubilee Road, Aba	Aba North	Abia
62	AB/0065	AB/0065/S/4	Cinta Pharmacy	Pharmacy	1 Okigwe Road, Aba	Aba South	Abia
63	AB/0067	AB/0067/S/4	Diamond Pharmacy Chemist Ltd.	Pharmacy	2 Umule Street Off Faulk Road, Aba	Aba South	Abia
64	AB/0069	AB/0069/S/4	Fatiwa Pharmacy Ltd.	Pharmacy	62, Aba Owo Road, Aba	Aba North	Abia
65	AB/0071	AB/0071/S/4	Rufus Obi Chemist Ltd.	Pharmacy	Rochem House 140 Aba-Owerri Road, Aba	Aba South	Abia
66	AB/0073	AB/0073/S/4	Em-Chemists Ltd.	Pharmacy	79 Aba -Owerri Road, Aba	Aba South	Abia
67	AB/0084	AB/0084/S/5	Streamlight Biomedical Lab.	Laboratory	49C Umuahia Road, Aba	Aba North	Abia
68	AB/0085	AB/0085/S/5	Steve Fort Medical Lab. Services	Laboratory	30 Jubilee Road, Aba	Aba North	Abia
69	AB/0086	AB/0086/S/5	Excellence Medical Diag. Lab.	Laboratory	1 Okigwe Road, Aba	Aba South	Abia
70	AB/0087	AB/0087/S/5	Taag Medical Laboratory	Laboratory	2 Bende Road, Umuahia	Umuahia North	Abia
71	AB/0088	AB/0088/S/5	Gardenia Laboratory Nig. Ltd.	Laboratory	2 Ohafia Street, Umauahia	Umuahia North	Abia
72	AB/0093	AB/0093/S/5	Police Hospital, Umuahia		Abia State Police Command, Abia State	Aba North	Abia
73	AB/0093	AB/0093/S/4	Police Hospital, Umuahia		Abia State Police Command, Abia State	Aba North	Abia
74	AB/0100	AB/0100/S/1	St. Anthonys Hospital Ltd.	Gen. surgery, Laboratory, Radiology, Orthopaedics, Ultrasound	62/80 Etshe Road, Aba, Abia State	Aba North	Abia
75	AB/0100	AB/0100/S/14	St. Anthonys Hospital Ltd.	Gen. surgery, Laboratory, Radiology, Orthopaedics, Ultrasound	62/80 Etshe Road, Aba, Abia State	Aba North	Abia
76	AB/0100	AB/0100/S/10	St. Anthonys Hospital Ltd.	Gen. surgery, Laboratory, Radiology, Orthopaedics, Ultrasound	62/80 Etshe Road, Aba, Abia State	Aba North	Abia
77	AB/0100	AB/0100/S/7	St. Anthonys Hospital Ltd.	Gen. surgery, Laboratory, Radiology, Orthopaedics, Ultrasound	62/80 Etshe Road, Aba, Abia State	Aba North	Abia
78	AB/0100	AB/0100/S/5	St. Anthonys Hospital Ltd.	Gen. surgery, Laboratory, Radiology, Orthopaedics, Ultrasound	62/80 Etshe Road, Aba, Abia State	Aba North	Abia
79	AB/0101	AB/0101/S/3	The Nasareth Clinics	O&G	57 Pound Road, Aba	Aba North	Abia
80	AB/0102	AB/0102/S/5	New Convenant Med. Lab. & Blood Bank	Laboratory	8 Okigwe Road, Aba, Abia State	Aba South	Abia
81	AB/0103	AB/0103/S/4	Adamor Nig. Ltd	Pharmacy	44 Okigwe Road, Aba, Abia State	Aba South	Abia

82	AB/0104	AB/0104/S/4	National Root Crops Research Institute Staff Clinic, Umudike		Umudike, Umuahia	Umuahia South	Abia
83	AB/0107	AB/0107/S/1	Healing Cross Hospital		Kilometre 2 Bende Road, Umuahia, Abia State	Umuahia North	Abia
84	AB/0107	AB/0107/S/5	Healing Cross Hospital		Kilometre 2 Bende Road, Umuahia, Abia State	Umuahia North	Abia
85	AB/0110	AB/0110/S/2	Aloma specialist hospital		9(108) aba road, umuahia	Umuahia North	Abia
86	AB/0126	AB/0126/S/3	BESTCARE SPEC. HOSPITAL & MATERNITY	O & G	6 MADUKWE CLOSE OFF AHUNANYA AVE; UMUNGASI ABA	Osisioma	Abia
87	AB/0128	AB/0128/S/3	DENNIS BLESSING HOSPITAL	O & G	15 OHAFIA STR, UMUAHIA	Umuahia North	Abia
88	AB/0129	AB/0129/S/4	ENKAL PHARMACY	Pharmacy	AHABA JUNCTION	Isuikwuato	Abia
89	AB/0139	AB/0139/S/3	ST. ANTHONY's HOSPITAL	O & G, Dental, OPTHALMOLOGY	62-80 ETCHE RD, ABA	Aba South	Abia
90	AB/0139	AB/0139/S/8	ST. ANTHONY's HOSPITAL	O & G, Dental, OPTHALMOLOGY	62-80 ETCHE RD, ABA	Aba South	Abia
91	AB/0139	AB/0139/S/15	ST. ANTHONY's HOSPITAL	O & G, Dental, OPTHALMOLOGY	62-80 ETCHE RD, ABA	Aba South	Abia
92	AB/0145	AB/0145/S/5	Michael Okpara University of Agriculture Medical Centre		Umudike,	Umuahia South	Abia
93	AB/0145	AB/0145/S/11	Michael Okpara University of Agriculture Medical Centre		Umudike,	Umuahia South	Abia
94	AB/0145	AB/0145/S/15	Michael Okpara University of Agriculture Medical Centre		Umudike,	Umuahia South	Abia
95	AB/0145	AB/0145/S/4	Michael Okpara University of Agriculture Medical Centre		Umudike,	Umuahia South	Abia
96	AB/0145	AB/0145/S/7	Michael Okpara University of Agriculture Medical Centre		Umudike,	Umuahia South	Abia
97	AB/0150	AB/0150/S/2	Chukwuebuka Hospital		50 School Road, Umuahia	Umuahia North	Abia
98	AB/0152	AB/0152/S/3	The Nazareth Clinics	O&G, Paediatrics	57 Pound Road, Aba. Abia State	Aba South	Abia
99	AB/0152	AB/0152/S/6	The Nazareth Clinics	O&G, Paediatrics	57 Pound Road, Aba. Abia State	Aba South	Abia
100	AB/0153	AB/0153/S/1	Ngozi Hospital	General Surgery	2 Isuochi Close, Umuahia	Umuahia North	Abia
101	AB/0171	AB/0171/S/2	Madonna Catholic Hospital		Ohokobe Afaraukwu, Aba Road, Umuahia, Abia State	Umuahia North	Abia
102	AB/0171	AB/0171/S/4	Madonna Catholic Hospital		Ohokobe Afaraukwu, Aba Road, Umuahia, Abia State	Umuahia North	Abia
103	AB/0171	AB/0171/S/3	Madonna Catholic Hospital		Ohokobe Afaraukwu, Aba Road, Umuahia, Abia State	Umuahia North	Abia
104	AB/0171	AB/0171/S/1	Madonna Catholic Hospital		Ohokobe Afaraukwu, Aba Road, Umuahia, Abia State	Umuahia North	Abia
105	AB/0171	AB/0171/S/5	Madonna Catholic Hospital		Ohokobe Afaraukwu, Aba Road, Umuahia, Abia State	Umuahia North	Abia
106	AB/0176	AB/0176/S/2	Chukwuebuka Hospital		50,School Road Umuahia, Abia State	Umuahia North	Abia
107	AB/0178	AB/0178/S/13	Global Eye Clinic		8-12 Umuka Road Umungasi,AbaAbia State	Osisioma	Abia
108	AB/0179	AB/0179/S/3	Impact Hospital &Maternity		59B Ukaegbu Road OgborHills,AbaAbia State	Aba North	Abia
109	AB/0181	AB/0181/S/1	Cottage Hospital Azumiri		Azumiri, Abia State	Ukwa East	Abia
110	AB/0182	AB/0182/S/5	Nigerian Christian Hospital Nlagu		Km 18,Aba-Ekot-Ekpene Road Aba Abia State	Obingwa	Abia
111	AB/0182	AB/0182/S/2	Nigerian Christian Hospital Nlagu		Km 18,Aba-Ekot-Ekpene Road Aba Abia State	Obingwa	Abia
112	AB/0182	AB/0182/S/6	Nigerian Christian Hospital Nlagu		Km 18,Aba-Ekot-Ekpene Road Aba Abia State	Obingwa	Abia
113	AB/0182	AB/0182/S/3	Nigerian Christian Hospital Nlagu		Km 18,Aba-Ekot-Ekpene Road Aba Abia State	Obingwa	Abia
114	AB/0182	AB/0182/S/15	Nigerian Christian Hospital Nlagu		Km 18,Aba-Ekot-Ekpene Road Aba Abia State	Obingwa	Abia
115	AB/0182	AB/0182/S/1	Nigerian Christian Hospital Nlagu		Km 18,Aba-Ekot-Ekpene Road Aba Abia State	Obingwa	Abia
116	AB/0182	AB/0182/S/7	Nigerian Christian Hospital Nlagu		Km 18,Aba-Ekot-Ekpene Road Aba Abia State	Obingwa	Abia

117	AB/0184	AB/0184/S/5	Life Essential &Maternity		32, Uyo Street, by Okigwe road, Umuahia	Umuahia North	Abia
118	AB/0186	AB/0186/S/5	Police Hospital		Bendel Road Umuahia Abia State	Umuahia North	Abia
119	AB/0186	AB/0186/S/4	Police Hospital		Bendel Road Umuahia Abia State	Umuahia North	Abia
120	AB/0189	AB/0189/S/15	Federal Medical Centre		Umuahia Abia State	Umuahia North	Abia
121	AB/0189	AB/0189/S/8	Federal Medical Centre		Umuahia Abia State	Umuahia North	Abia
122	AB/0190	AB/0190/S/3	Uzondu Polyclinic		8 Ofali Agwu Road Amaekpu Ohafia,Abia State	Ohafia	Abia
123	AB/0191	AB/0191/S/13	Exxelli Vision Eye Clinic		6 Aba Road Umuahia Abia State	Umuahia North	Abia
124	AB/0196	AB/0196/S/4	Seven Days Adventist Hospital		1 New Umuahia Road Aba, Abia State	Aba North	Abia
125	AB/0198	AB/0198/S/5	Quickhealth Specialist Clinic		2 Nwadinobi Street, off BCA Road, Umuahia Abia State	Aba North	Abia
126	AB/0201	AB/0201/S/13	Dominion Hospital & Maternity		9A Sunny Lane, off Azuka Drive, Ogbor Hill, Aba Abia State	Aba North	Abia
127	AB/0202	AB/0202/S/4	Christ De King Health Services Ltd		9 Orie Ngodo, Isuochi. Abia State	Umu - Nneochi	Abia
128	AB/0202	AB/0202/S/5	Christ De King Health Services Ltd		9 Orie Ngodo, Isuochi. Abia State	Umu - Nneochi	Abia
129	AB/0203	AB/0203/S/5	Calif Specialist Hospital		25/27 Chief Adiukwu Street, By Dip Gold Filling Station, Off Enugu –PH Express Way, Aba Abia State	Osisioma	Abia
130	AB/0203	AB/0203/S/3	Calif Specialist Hospital		25/27 Chief Adiukwu Street, By Dip Gold Filling Station, Off Enugu –PH Express Way, Aba Abia State	Osisioma	Abia
131	AB/0208	AB/0208/S/5	Quickhealth Specialist Clinic		2 Nwadinobi Street, off BCA Road, Umuahia Abia State	Umuahia North	Abia
132	AB/0211	AB/0211/S/15	Embassy Specialist & maternity Care Ltd.		No. 59c Ayaba Umueze Road, Opp. ABSUTH, second gate, Aba.	Osisioma	Abia
133	AB/0212	AB/0212/S/13	Parker Eye Clinic.		No. 30 Macaulay street, off Azikiwe road, Umuahia.	Umuahia North	Abia
134	AB/0213	AB/0213/S/15	All Saints Hospital		No. 26 Item street Umuahia.	Umuahia North	Abia
135	AB/0214	AB/0214/S/15	St. Anthony Hospital Ltd.		No. 52-80 Etche Road, Aba.	Aba South	Abia
136	AB/0214	AB/0214/S/8	St. Anthony Hospital Ltd.		No. 52-80 Etche Road, Aba.	Aba South	Abia
137	AB/0214	AB/0214/S/4	St. Anthony Hospital Ltd.		No. 52-80 Etche Road, Aba.	Aba South	Abia
138	AB/0214	AB/0214/S/5	St. Anthony Hospital Ltd.		No. 52-80 Etche Road, Aba.	Aba South	Abia
139	AB/0214	AB/0214/S/1	St. Anthony Hospital Ltd.		No. 52-80 Etche Road, Aba.	Aba South	Abia
140	AB/0214	AB/0214/S/7	St. Anthony Hospital Ltd.		No. 52-80 Etche Road, Aba.	Aba South	Abia
141	AB/0215	AB/0215/S/15	Pamel Eze Global Foundation{PEGF}		Umuosu Umuna Nsulu Ngwa	Isiala Ngwa North	Abia
142	AB/0217	AB/0217/S/1	Samcon Friends Mission Hospital and Motherless Babies Home.		Mgboko Ohokobe	Obingwa	Abia
143	AB/0217	AB/0217/S/3	Samcon Friends Mission Hospital and Motherless Babies Home.		Mgboko Ohokobe	Obingwa	Abia
144	AD/0002	AD/0002/S/4	General Hospital, Ganye		Ganye, Adamawa State	Ganye	Adamawa
145	AD/0002	AD/0002/S/2	General Hospital, Ganye		Ganye, Adamawa State	Ganye	Adamawa
146	AD/0002	AD/0002/S/8	General Hospital, Ganye		Ganye, Adamawa State	Ganye	Adamawa
147	AD/0002	AD/0002/S/3	General Hospital, Ganye		Ganye, Adamawa State	Ganye	Adamawa
148	AD/0002	AD/0002/S/7	General Hospital, Ganye		Ganye, Adamawa State	Ganye	Adamawa
149	AD/0002	AD/0002/S/6	General Hospital, Ganye		Ganye, Adamawa State	Ganye	Adamawa
150	AD/0002	AD/0002/S/1	General Hospital, Ganye		Ganye, Adamawa State	Ganye	Adamawa
151	AD/0002	AD/0002/S/15	General Hospital, Ganye		Ganye, Adamawa State	Ganye	Adamawa
152	AD/0006	AD/0006/S/5	Cottage Hospital, Mayo-Belwa		Mayo-Belwa	Mayo - Belwa	Adamawa
153	AD/0006	AD/0006/S/2	Cottage Hospital, Mayo-Belwa		Mayo-Belwa	Mayo - Belwa	Adamawa

154	AD/0006	AD/0006/S/1	Cottage Hospital, Mayo-Belwa		Mayo-Belwa	Mayo - Belwa	Adamawa
155	AD/0013	AD/0013/S/4	General Hospital, Numan		Numan	Numan	Adamawa
156	AD/0013	AD/0013/S/7	General Hospital, Numan		Numan	Numan	Adamawa
157	AD/0013	AD/0013/S/8	General Hospital, Numan		Numan	Numan	Adamawa
158	AD/0013	AD/0013/S/1	General Hospital, Numan		Numan	Numan	Adamawa
159	AD/0013	AD/0013/S/2	General Hospital, Numan		Numan	Numan	Adamawa
160	AD/0015	AD/0015/S/1	Cottage Hospital, Song		Song	Song	Adamawa
161	AD/0015	AD/0015/S/2	Cottage Hospital, Song		Song	Song	Adamawa
162	AD/0015	AD/0015/S/5	Cottage Hospital, Song		Song	Song	Adamawa
163	AD/0017	AD/0017/S/6	Specialist Hospital, Yola		Yola	Yola North	Adamawa
164	AD/0017	AD/0017/S/11	Specialist Hospital, Yola		Yola	Yola North	Adamawa
165	AD/0017	AD/0017/S/9	Specialist Hospital, Yola		Yola	Yola North	Adamawa
166	AD/0017	AD/0017/S/10	Specialist Hospital, Yola		Yola	Yola North	Adamawa
167	AD/0017	AD/0017/S/3	Specialist Hospital, Yola		Yola	Yola North	Adamawa
168	AD/0017	AD/0017/S/8	Specialist Hospital, Yola		Yola	Yola North	Adamawa
169	AD/0017	AD/0017/S/1	Specialist Hospital, Yola		Yola	Yola North	Adamawa
170	AD/0017	AD/0017/S/7	Specialist Hospital, Yola		Yola	Yola North	Adamawa
171	AD/0017	AD/0017/S/15	Specialist Hospital, Yola		Yola	Yola North	Adamawa
172	AD/0017	AD/0017/S/2	Specialist Hospital, Yola		Yola	Yola North	Adamawa
173	AD/0017	AD/0017/S/4	Specialist Hospital, Yola		Yola	Yola North	Adamawa
174	AD/0017	AD/0017/S/5	Specialist Hospital, Yola		Yola	Yola North	Adamawa
175	AD/0019	AD/0019/S/1	Triumph Medical Clinic		Mubi Road, Jimeta	Yola North	Adamawa
176	AD/0019	AD/0019/S/7	Triumph Medical Clinic		Mubi Road, Jimeta	Yola North	Adamawa
177	AD/0019	AD/0019/S/3	Triumph Medical Clinic		Mubi Road, Jimeta	Yola North	Adamawa
178	AD/0019	AD/0019/S/2	Triumph Medical Clinic		Mubi Road, Jimeta	Yola North	Adamawa
179	AD/0019	AD/0019/S/5	Triumph Medical Clinic		Mubi Road, Jimeta	Yola North	Adamawa
180	AD/0020	AD/0020/S/2	Matco Clinic		Army Barracks Road, Malamre, Yola	Yola North	Adamawa
181	AD/0020	AD/0020/S/4	Matco Clinic		Army Barracks Road, Malamre, Yola	Yola North	Adamawa
182	AD/0020	AD/0020/S/5	Matco Clinic		Army Barracks Road, Malamre, Yola	Yola North	Adamawa
183	AD/0020	AD/0020/S/7	Matco Clinic		Army Barracks Road, Malamre, Yola	Yola North	Adamawa
184	AD/0020	AD/0020/S/1	Matco Clinic		Army Barracks Road, Malamre, Yola	Yola North	Adamawa
185	AD/0022	AD/0022/S/7	Mahmud Clinic		Jimeta, Yola	Yola North	Adamawa
186	AD/0022	AD/0022/S/5	Mahmud Clinic		Jimeta, Yola	Yola North	Adamawa
187	AD/0022	AD/0022/S/1	Mahmud Clinic		Jimeta, Yola	Yola North	Adamawa
188	AD/0022	AD/0022/S/2	Mahmud Clinic		Jimeta, Yola	Yola North	Adamawa
189	AD/0023	AD/0023/S/2	Dawau Clinic Ltd.		Jimeta Yola	Yola North	Adamawa
190	AD/0023	AD/0023/S/1	Dawau Clinic Ltd.		Jimeta Yola	Yola North	Adamawa
191	AD/0023	AD/0023/S/5	Dawau Clinic Ltd.		Jimeta Yola	Yola North	Adamawa
192	AD/0024	AD/0024/S/3	Peace Hospital		No. 2 Luggere Street, Beside St. Theresa''''s Cathedral, Yola, Adamawa State	Yola North	Adamawa
193	AD/0024	AD/0024/S/13	Peace Hospital		No. 2 Luggere Street, Beside St. Theresa''''s Cathedral, Yola, Adamawa State	Yola North	Adamawa
194	AD/0024	AD/0024/S/4	Peace Hospital		No. 2 Luggere Street, Beside St. Theresa''''s Cathedral, Yola, Adamawa State	Yola North	Adamawa
195	AD/0024	AD/0024/S/2	Peace Hospital		No. 2 Luggere Street, Beside St. Theresa''''s Cathedral, Yola, Adamawa State	Yola North	Adamawa
196	AD/0024	AD/0024/S/7	Peace Hospital		No. 2 Luggere Street, Beside St. Theresa''''s Cathedral, Yola, Adamawa State	Yola North	Adamawa

197	AD/0024	AD/0024/S/10	Peace Hospital		No. 2 Luggere Street, Beside St. Theresa''''s Cathedral, Yola, Adamawa State	Yola North	Adamawa
198	AD/0024	AD/0024/S/5	Peace Hospital		No. 2 Luggere Street, Beside St. Theresa''''s Cathedral, Yola, Adamawa State	Yola North	Adamawa
199	AD/0024	AD/0024/S/1	Peace Hospital		No. 2 Luggere Street, Beside St. Theresa''''s Cathedral, Yola, Adamawa State	Yola North	Adamawa
200	AD/0025	AD/0025/S/1	Adamawa Hospital		Abuja Road, Yola Town	Yola North	Adamawa
201	AD/0025	AD/0025/S/2	Adamawa Hospital		Abuja Road, Yola Town	Yola North	Adamawa
202	AD/0025	AD/0025/S/5	Adamawa Hospital		Abuja Road, Yola Town	Yola North	Adamawa
203	AD/0027	AD/0027/S/4	Sauki Medical Clinic		Bauchi Street, Off Mustapha Moh''D Way, Jimeta, Yola	Yola North	Adamawa
204	AD/0027	AD/0027/S/1	Sauki Medical Clinic		Bauchi Street, Off Mustapha Moh''D Way, Jimeta, Yola	Yola North	Adamawa
205	AD/0027	AD/0027/S/5	Sauki Medical Clinic		Bauchi Street, Off Mustapha Moh''D Way, Jimeta, Yola	Yola North	Adamawa
206	AD/0027	AD/0027/S/3	Sauki Medical Clinic		Bauchi Street, Off Mustapha Moh''D Way, Jimeta, Yola	Yola North	Adamawa
207	AD/0027	AD/0027/S/7	Sauki Medical Clinic		Bauchi Street, Off Mustapha Moh''D Way, Jimeta, Yola	Yola North	Adamawa
208	AD/0027	AD/0027/S/2	Sauki Medical Clinic		Bauchi Street, Off Mustapha Moh''D Way, Jimeta, Yola	Yola North	Adamawa
209	AD/0027	AD/0027/S/6	Sauki Medical Clinic		Bauchi Street, Off Mustapha Moh''D Way, Jimeta, Yola	Yola North	Adamawa
210	AD/0028	AD/0028/S/2	The Freedom Polyclinic		9b Hospital Road, Jimeta	Yola North	Adamawa
211	AD/0028	AD/0028/S/5	The Freedom Polyclinic		9b Hospital Road, Jimeta	Yola North	Adamawa
212	AD/0029	AD/0029/S/2	Galbose Clinic & Maternity		22 Atiki Abubakar Way, Jimeta, Yola	Yola North	Adamawa
213	AD/0029	AD/0029/S/5	Galbose Clinic & Maternity		22 Atiki Abubakar Way, Jimeta, Yola	Yola North	Adamawa
214	AD/0029	AD/0029/S/3	Galbose Clinic & Maternity		22 Atiki Abubakar Way, Jimeta, Yola	Yola North	Adamawa
215	AD/0029	AD/0029/S/4	Galbose Clinic & Maternity		22 Atiki Abubakar Way, Jimeta, Yola	Yola North	Adamawa
216	AD/0029	AD/0029/S/7	Galbose Clinic & Maternity		22 Atiki Abubakar Way, Jimeta, Yola	Yola North	Adamawa
217	AD/0029	AD/0029/S/6	Galbose Clinic & Maternity		22 Atiki Abubakar Way, Jimeta, Yola	Yola North	Adamawa
218	AD/0029	AD/0029/S/1	Galbose Clinic & Maternity		22 Atiki Abubakar Way, Jimeta, Yola	Yola North	Adamawa
219	AD/0030	AD/0030/S/2	Jimeta Clinics & Maternity		Bishop Street, Jimeta, Yola	Yola North	Adamawa
220	AD/0030	AD/0030/S/1	Jimeta Clinics & Maternity		Bishop Street, Jimeta, Yola	Yola North	Adamawa
221	AD/0030	AD/0030/S/3	Jimeta Clinics & Maternity		Bishop Street, Jimeta, Yola	Yola North	Adamawa
222	AD/0030	AD/0030/S/5	Jimeta Clinics & Maternity		Bishop Street, Jimeta, Yola	Yola North	Adamawa
223	AD/0030	AD/0030/S/7	Jimeta Clinics & Maternity		Bishop Street, Jimeta, Yola	Yola North	Adamawa
224	AD/0041	AD/0041/S/5	Springfield Hospital	Gen. Med. Practice, Gen. Surgery, Laboratory	Airport Road, Jimeta	Yola North	Adamawa
225	AD/0041	AD/0041/S	Springfield Hospital	Gen. Med. Practice, Gen. Surgery, Laboratory	Airport Road, Jimeta	Yola North	Adamawa
226	AD/0041	AD/0041/S/2	Springfield Hospital	Gen. Med. Practice, Gen. Surgery, Laboratory	Airport Road, Jimeta	Yola North	Adamawa
227	AD/0051	AD/0051/S/2	Arhyel-Royal Hospital		Numan Road, Jimeta	Yola South	Adamawa
228	AD/0051	AD/0051/S/5	Arhyel-Royal Hospital		Numan Road, Jimeta	Yola South	Adamawa
229	AD/0052	AD/0052/S/15	General Hospital, Mubi		Mubi	Mubi South	Adamawa
230	AD/0052	AD/0052/S/5	General Hospital, Mubi		Mubi	Mubi South	Adamawa
231	AD/0052	AD/0052/S/4	General Hospital, Mubi		Mubi	Mubi South	Adamawa
232	AD/0052	AD/0052/S/7	General Hospital, Mubi		Mubi	Mubi South	Adamawa
233	AD/0052	AD/0052/S/8	General Hospital, Mubi		Mubi	Mubi South	Adamawa

234	AD/0052	AD/0052/S/2	General Hospital, Mubi		Mubi	Mubi South	Adamawa
235	AD/0052	AD/0052/S/1	General Hospital, Mubi		Mubi	Mubi South	Adamawa
236	AD/0053	AD/0053/S/4	Newlife Hospital	Surgery, O&G, Laboratory, Radiology and Pharmacy	Sabon Layi, Mubi	Mubi North	Adamawa
237	AD/0053	AD/0053/S/7	Newlife Hospital	Surgery, O&G, Laboratory, Radiology and Pharmacy	Sabon Layi, Mubi	Mubi North	Adamawa
238	AD/0053	AD/0053/S/1	Newlife Hospital	Surgery, O&G, Laboratory, Radiology and Pharmacy	Sabon Layi, Mubi	Mubi North	Adamawa
239	AD/0053	AD/0053/S/2	Newlife Hospital	Surgery, O&G, Laboratory, Radiology and Pharmacy	Sabon Layi, Mubi	Mubi North	Adamawa
240	AD/0053	AD/0053/S/3	Newlife Hospital	Surgery, O&G, Laboratory, Radiology and Pharmacy	Sabon Layi, Mubi	Mubi North	Adamawa
241	AD/0053	AD/0053/S/5	Newlife Hospital	Surgery, O&G, Laboratory, Radiology and Pharmacy	Sabon Layi, Mubi	Mubi North	Adamawa
242	AD/0054	AD/0054/S/2	General Hospital, Garkida		Garkida	Gombi	Adamawa
243	AD/0054	AD/0054/S/4	General Hospital, Garkida		Garkida	Gombi	Adamawa
244	AD/0054	AD/0054/S/7	General Hospital, Garkida		Garkida	Gombi	Adamawa
245	AD/0054	AD/0054/S/15	General Hospital, Garkida		Garkida	Gombi	Adamawa
246	AD/0054	AD/0054/S/1	General Hospital, Garkida		Garkida	Gombi	Adamawa
247	AD/0054	AD/0054/S/8	General Hospital, Garkida		Garkida	Gombi	Adamawa
248	AD/0054	AD/0054/S/5	General Hospital, Garkida		Garkida	Gombi	Adamawa
249	AD/0055	AD/0055/S/5	Cottage Hospital, Hong		Hong	Hong	Adamawa
250	AD/0055	AD/0055/S/1	Cottage Hospital, Hong		Hong	Hong	Adamawa
251	AD/0055	AD/0055/S/2	Cottage Hospital, Hong		Hong	Hong	Adamawa
252	AD/0056	AD/0056/S/1	Cottage Hospital, Guyuk		Guyuk	Guyuk	Adamawa
253	AD/0056	AD/0056/S/2	Cottage Hospital, Guyuk		Guyuk	Guyuk	Adamawa
254	AD/0056	AD/0056/S/5	Cottage Hospital, Guyuk		Guyuk	Guyuk	Adamawa
255	AD/0077	AD/0077/S/6	General Hospital, Michika		Michika, Adamawa State	Michika	Adamawa
256	AD/0077	AD/0077/S/2	General Hospital, Michika		Michika, Adamawa State	Michika	Adamawa
257	AD/0077	AD/0077/S/3	General Hospital, Michika		Michika, Adamawa State	Michika	Adamawa
258	AD/0077	AD/0077/S/1	General Hospital, Michika		Michika, Adamawa State	Michika	Adamawa
259	AD/0077	AD/0077/S/4	General Hospital, Michika		Michika, Adamawa State	Michika	Adamawa
260	AD/0077	AD/0077/S/5	General Hospital, Michika		Michika, Adamawa State	Michika	Adamawa
261	AD/0077	AD/0077/S/15	General Hospital, Michika		Michika, Adamawa State	Michika	Adamawa
262	AD/0077	AD/0077/S/8	General Hospital, Michika		Michika, Adamawa State	Michika	Adamawa
263	AD/0077	AD/0077/S/7	General Hospital, Michika		Michika, Adamawa State	Michika	Adamawa
264	AD/0078	AD/0078/S/1	Cottage Hospital, Fufore		Fufore, Adamawa State	Fufore	Adamawa
265	AD/0078	AD/0078/S/2	Cottage Hospital, Fufore		Fufore, Adamawa State	Fufore	Adamawa
266	AD/0078	AD/0078/S/5	Cottage Hospital, Fufore		Fufore, Adamawa State	Fufore	Adamawa
267	AD/0083	AD/0083/S/5	Federal College of Education, College Clinic		Jimeta, Yola Road, Adamawa State	Yola North	Adamawa
268	AD/0084	AD/0084/S/4	Federal Polytechnic Clinic, Mubi		Barama Ward, Mubi	Mubi North	Adamawa
269	AD/0084	AD/0084/S/5	Federal Polytechnic Clinic, Mubi		Barama Ward, Mubi	Mubi North	Adamawa
270	AD/0104	AD/0104/S/5	OPTIMUM CLINIC		100 MOH""""D MUSTAPHA WAY, JIMETA	Yola North	Adamawa
271	AD/0116	AD/0116/S/3	Trust Medical Centre		Anguwan Faransa, Gombi	Gombi	Adamawa

272	AD/0116	AD/0116/S/6	Trust Medical Centre		Anguwan Faransa, Gombi	Gombi	Adamawa
273	AD/0116	AD/0116/S/7	Trust Medical Centre		Anguwan Faransa, Gombi	Gombi	Adamawa
274	AD/0116	AD/0116/S/5	Trust Medical Centre		Anguwan Faransa, Gombi	Gombi	Adamawa
275	AD/0116	AD/0116/S/1	Trust Medical Centre		Anguwan Faransa, Gombi	Gombi	Adamawa
276	AD/0116	AD/0116/S/2	Trust Medical Centre		Anguwan Faransa, Gombi	Gombi	Adamawa
277	AD/0118	AD/0118/S/7	The New Boshang Clinic and maternity Ltd		Karewa GRA, Jimeta-Yola	Yola North	Adamawa
278	AD/0118	AD/0118/S/3	The New Boshang Clinic and maternity Ltd		Karewa GRA, Jimeta-Yola	Yola North	Adamawa
279	AD/0118	AD/0118/S/1	The New Boshang Clinic and maternity Ltd		Karewa GRA, Jimeta-Yola	Yola North	Adamawa
280	AD/0118	AD/0118/S/6	The New Boshang Clinic and maternity Ltd		Karewa GRA, Jimeta-Yola	Yola North	Adamawa
281	AD/0121	AD/0121/S/5	Valli Medical Clinic		22 Mallamre, Opp FCE First Gate, Along Aliyu Way, Jimeta	Yola North	Adamawa
282	AD/0121	AD/0121/S/2	Valli Medical Clinic		22 Mallamre, Opp FCE First Gate, Along Aliyu Way, Jimeta	Yola North	Adamawa
283	AD/0121	AD/0121/S/3	Valli Medical Clinic		22 Mallamre, Opp FCE First Gate, Along Aliyu Way, Jimeta	Yola North	Adamawa
284	AD/0121	AD/0121/S/1	Valli Medical Clinic		22 Mallamre, Opp FCE First Gate, Along Aliyu Way, Jimeta	Yola North	Adamawa
285	AD/0121	AD/0121/S/6	Valli Medical Clinic		22 Mallamre, Opp FCE First Gate, Along Aliyu Way, Jimeta	Yola North	Adamawa
286	AD/0125	AD/0125/S/6	Da'ama Specialist Hospital		# 70/72 Atiku Abubakar Road, Yola	Yola North	Adamawa
287	AD/0125	AD/0125/S/5	Da'ama Specialist Hospital		# 70/72 Atiku Abubakar Road, Yola	Yola North	Adamawa
288	AD/0125	AD/0125/S/2	Da'ama Specialist Hospital		# 70/72 Atiku Abubakar Road, Yola	Yola North	Adamawa
289	AD/0125	AD/0125/S/1	Da'ama Specialist Hospital		# 70/72 Atiku Abubakar Road, Yola	Yola North	Adamawa
290	AD/0126	AD/0126/S/5	American University Of Nigeria Clinic.		Lamido Zubairu Way, Yola By-Pass, Adamawa State.	Yola North	Adamawa
291	AD/0132	AD/0132/S/4	Valada Hospital & Maternity		Army Barracks Road Yola Adamawa State	Yola North	Adamawa
292	AD/0132	AD/0132/S/5	Valada Hospital & Maternity		Army Barracks Road Yola Adamawa State	Yola North	Adamawa
293	AK/0001	AK/0001/S/4	Samaritan Clinic		No. 7 Ekong Uko Street, Eket	Eket	Akwa Ibom
294	AK/0001	AK/0001/S/5	Samaritan Clinic		No. 7 Ekong Uko Street, Eket	Eket	Akwa Ibom
295	AK/0001	AK/0001/S/7	Samaritan Clinic		No. 7 Ekong Uko Street, Eket	Eket	Akwa Ibom
296	AK/0002	AK/0002/S/3	Immanuel General Hospital	-	Eket, Akwa Ibom	Eket	Akwa Ibom
297	AK/0002	AK/0002/S/13	Immanuel General Hospital	-	Eket, Akwa Ibom	Eket	Akwa Ibom
298	AK/0002	AK/0002/S/4	Immanuel General Hospital	-	Eket, Akwa Ibom	Eket	Akwa Ibom
299	AK/0002	AK/0002/S/14	Immanuel General Hospital	-	Eket, Akwa Ibom	Eket	Akwa Ibom
300	AK/0002	AK/0002/S/5	Immanuel General Hospital	-	Eket, Akwa Ibom	Eket	Akwa Ibom
301	AK/0007	AK/0007/S/5	General Hospital, Ikot Ekpene		Ikot Ekpene, Akwa Ibom	Ikot Ekpene	Akwa Ibom
302	AK/0007	AK/0007/S/2	General Hospital, Ikot Ekpene		Ikot Ekpene, Akwa Ibom	Ikot Ekpene	Akwa Ibom
303	AK/0007	AK/0007/S/4	General Hospital, Ikot Ekpene		Ikot Ekpene, Akwa Ibom	Ikot Ekpene	Akwa Ibom
304	AK/0007	AK/0007/S/7	General Hospital, Ikot Ekpene		Ikot Ekpene, Akwa Ibom	Ikot Ekpene	Akwa Ibom
305	AK/0007	AK/0007/S/8	General Hospital, Ikot Ekpene		Ikot Ekpene, Akwa Ibom	Ikot Ekpene	Akwa Ibom
306	AK/0009	AK/0009/S/12	Dominion Specialist Hospital	ENT	4, Ibong Road, Ikot Epene	Ikot Ekpene	Akwa Ibom
307	AK/0010	AK/0010/S/4	General Hospital, Iquita-Oron		Iquita-Oron	Oron	Akwa Ibom
308	AK/0010	AK/0010/S/7	General Hospital, Iquita-Oron		Iquita-Oron	Oron	Akwa Ibom
309	AK/0012	AK/0012/S/7	University Of Uyo Teaching Hospital, Uyo		Uyo, Akwa Ibom	Uyo	Akwa Ibom
310	AK/0012	AK/0012/S/10	University Of Uyo Teaching Hospital, Uyo		Uyo, Akwa Ibom	Uyo	Akwa Ibom

311	AK/0012	AK/0012/S/6	University Of Uyo Teaching Hospital, Uyo		Uyo, Akwa Ibom	Uyo	Akwa Ibom
312	AK/0012	AK/0012/S/3	University Of Uyo Teaching Hospital, Uyo		Uyo, Akwa Ibom	Uyo	Akwa Ibom
313	AK/0012	AK/0012/S/4	University Of Uyo Teaching Hospital, Uyo		Uyo, Akwa Ibom	Uyo	Akwa Ibom
314	AK/0012	AK/0012/S/1	University Of Uyo Teaching Hospital, Uyo		Uyo, Akwa Ibom	Uyo	Akwa Ibom
315	AK/0012	AK/0012/S/2	University Of Uyo Teaching Hospital, Uyo		Uyo, Akwa Ibom	Uyo	Akwa Ibom
316	AK/0012	AK/0012/S/5	University Of Uyo Teaching Hospital, Uyo		Uyo, Akwa Ibom	Uyo	Akwa Ibom
317	AK/0013	AK/0013/S/13	Mfon Abasi Medical Centre		Uyo, Akwa Ibom	Uyo	Akwa Ibom
318	AK/0013	AK/0013/S/3	Mfon Abasi Medical Centre		Uyo, Akwa Ibom	Uyo	Akwa Ibom
319	AK/0013	AK/0013/S/2	Mfon Abasi Medical Centre		Uyo, Akwa Ibom	Uyo	Akwa Ibom
320	AK/0013	AK/0013/S/1	Mfon Abasi Medical Centre		Uyo, Akwa Ibom	Uyo	Akwa Ibom
321	AK/0017	AK/0017/S/1	Uwakmfon Specialist Clinics & Hospital		27 Nwaniba Road, Uyo, Akwa Ibom State	Uyo	Akwa Ibom
322	AK/0017	AK/0017/S/2	Uwakmfon Specialist Clinics & Hospital		27 Nwaniba Road, Uyo, Akwa Ibom State	Uyo	Akwa Ibom
323	AK/0017	AK/0017/S/5	Uwakmfon Specialist Clinics & Hospital		27 Nwaniba Road, Uyo, Akwa Ibom State	Uyo	Akwa Ibom
324	AK/0018	AK/0018/S/4	Beemas Pharmacy	Pharmacy	75 Aka Road	Uyo	Akwa Ibom
325	AK/0019	AK/0019/S/15	Ceejay Vision Centre	Ophthalmology	Uyo	Uyo	Akwa Ibom
326	AK/0021	AK/0021/S/4	Base Pharmacy	Pharmacy	44, Abak Road, Uyo	Uyo	Akwa Ibom
327	AK/0023	AK/0023/S/4	One Stop Pharmacy	Pharmacy	17, Nwaniba Street, Uyo	Uyo	Akwa Ibom
328	AK/0027	AK/0027/S/4	Frank-Chimex Pharmacy	Pharmacy	44, Aka road, Uyo	Uyo	Akwa Ibom
329	AK/0028	AK/0028/S/5	Central Laboratory	Laboratory	Uyo	Uyo	Akwa Ibom
330	AK/0034	AK/0034/S/3	Mainland Clinic/Hospital		2 Andy-Umana Lane, Off Aka Road, Uyo, Akwa Ibom	Uyo	Akwa Ibom
331	AK/0035	AK/0035/S/15	Majesty Hospital & Eye Clinics		47 Wellinton Bassey Akwa Ibom	Uyo	Akwa Ibom
332	AK/0035	AK/0035/S/5	Majesty Hospital & Eye Clinics		47 Wellinton Bassey Akwa Ibom	Uyo	Akwa Ibom
333	AK/0037	AK/0037/S/1	Mount Olive Clinic & Maternity		8 Ebonny Essien Street, off Ikot Ekpene Rd, Uyo, A/Ibom State	Uyo	Akwa Ibom
334	AK/0037	AK/0037/S/3	Mount Olive Clinic & Maternity		8 Ebonny Essien Street, off Ikot Ekpene Rd, Uyo, A/Ibom State	Uyo	Akwa Ibom
335	AK/0040	AK/0040/S/5	Royal Pathology	Laboratory	3 Essien Nton Road, Ikot Ekpene	Ikot Ekpene	Akwa Ibom
336	AK/0045	AK/0045/S/5	University of Uyo Health Centre, Uyo		Uyo, A/Ibom State	Uyo	Akwa Ibom
337	AK/0045	AK/0045/S/1	University of Uyo Health Centre, Uyo		Uyo, A/Ibom State	Uyo	Akwa Ibom
338	AK/0045	AK/0045/S/4	University of Uyo Health Centre, Uyo		Uyo, A/Ibom State	Uyo	Akwa Ibom
339	AK/0046	AK/0046/S/1	All Saints Medical Centre		1-5 Independent Avenue, Abak, Akwa Ibom	Abak	Akwa Ibom
340	AK/0048	AK/0048/S/6	Dammy memorial hospital		34, ukana offot street, uyo, Akwa-ibom	Uyo	Akwa Ibom
341	AK/0048	AK/0048/S/2	Dammy memorial hospital		34, ukana offot street, uyo, Akwa-ibom	Uyo	Akwa Ibom
342	AK/0048	AK/0048/S/1	Dammy memorial hospital		34, ukana offot street, uyo, Akwa-ibom	Uyo	Akwa Ibom
343	AK/0048	AK/0048/S/3	Dammy memorial hospital		34, ukana offot street, uyo, Akwa-ibom	Uyo	Akwa Ibom
344	AK/0049	AK/0049/S/1	Dan Abia specialist clinic		11b, Idoro road, off itam junction, uyo,Akwa-ibom	Uyo	Akwa Ibom
345	AK/0055	AK/0055/S/3	Iha foundation medical center		Eket Annex, Ekpeyu street, Ikot ebok Eket	Eket	Akwa Ibom
346	AK/0056	AK/0056/S/3	Kaizo specialist hospital		10, market road,Afaha offot, off Abak road, Uyo	Uyo	Akwa Ibom
347	AK/0056	AK/0056/S/2	Kaizo specialist hospital		10, market road,Afaha offot, off Abak road, Uyo	Uyo	Akwa Ibom
348	AK/0056	AK/0056/S/1	Kaizo specialist hospital		10, market road,Afaha offot, off Abak road, Uyo	Uyo	Akwa Ibom
349	AK/0058	AK/0058/S/15	Mercy hospital		95 hospital road, Abak, Ak/Ibom	Abak	Akwa Ibom
350	AK/0058	AK/0058/S/3	Mercy hospital		95 hospital road, Abak, Ak/Ibom	Abak	Akwa Ibom

351	AK/0058	AK/0058/S/7	Mercy hospital		95 hospital road, Abak, Ak/Ibom	Abak	Akwa Ibom
352	AK/0058	AK/0058/S/1	Mercy hospital		95 hospital road, Abak, Ak/Ibom	Abak	Akwa Ibom
353	AK/0058	AK/0058/S/5	Mercy hospital		95 hospital road, Abak, Ak/Ibom	Abak	Akwa Ibom
354	AK/0058	AK/0058/S/4	Mercy hospital		95 hospital road, Abak, Ak/Ibom	Abak	Akwa Ibom
355	AK/0059	AK/0059/S/3	St. Athanasius hospital		1, ufeh street,F.H.estate , Abak road, Uyo	Uyo	Akwa Ibom
356	AK/0059	AK/0059/S/6	St. Athanasius hospital		1, ufeh street,F.H.estate , Abak road, Uyo	Uyo	Akwa Ibom
357	AK/0059	AK/0059/S/2	St. Athanasius hospital		1, ufeh street,F.H.estate , Abak road, Uyo	Uyo	Akwa Ibom
358	AK/0059	AK/0059/S/1	St. Athanasius hospital		1, ufeh street,F.H.estate , Abak road, Uyo	Uyo	Akwa Ibom
359	AK/0060	AK/0060/S/1	Ubong Abasi specialist clinic		5, clement Isong street, FHA estate, Abak road, Uyo	Uyo	Akwa Ibom
360	AK/0060	AK/0060/S/3	Ubong Abasi specialist clinic		5, clement Isong street, FHA estate, Abak road, Uyo	Uyo	Akwa Ibom
361	AK/0062	AK/0062/S/3	Uwem foundation clinic		8b Etienam street, oron, AK/IBOM	Oron	Akwa Ibom
362	AK/0066	AK/0066/S/1	DOMINGO HOSPITAL	SURGERY	Plot 9, unit E, Ben Udo Street, Ewet Housing estate, uyo	Urue Offong Oruko	Akwa Ibom
363	AK/0069	AK/0069/S/5	JIL LABORATORY	Laboratory	145, ORON ROAD, AK/IBOM	Uyo	Akwa Ibom
364	AK/0071	AK/0071/S/4	M&I PHARMACY	Pharmacy	139G UNIT A,ISEMIN AVENUE,EWET HOUSING ESTATE,UYO	Uyo	Akwa Ibom
365	AK/0073	AK/0073/S/3	MFON ABASI MEDICAL CENTER	Internal medicine, Optometry ,O&G, Surgery,	10 GIBBS STREET, UYO	Uyo	Akwa Ibom
366	AK/0073	AK/0073/S/1	MFON ABASI MEDICAL CENTER	Internal medicine, Optometry ,O&G, Surgery,	10 GIBBS STREET, UYO	Uyo	Akwa Ibom
367	AK/0073	AK/0073/S/2	MFON ABASI MEDICAL CENTER	Internal medicine, Optometry ,O&G, Surgery,	10 GIBBS STREET, UYO	Uyo	Akwa Ibom
368	AK/0073	AK/0073/S/13	MFON ABASI MEDICAL CENTER	Internal medicine, Optometry ,O&G, Surgery,	10 GIBBS STREET, UYO	Uyo	Akwa Ibom
369	AK/0076	AK/0076/S/5	ULTIMATE MEDICAL DIAGNOSTICS	Laboratory, Radiology	15, IBB AVENUE, UYO	Uyo	Akwa Ibom
370	AK/0076	AK/0076/S/7	ULTIMATE MEDICAL DIAGNOSTICS	Laboratory, Radiology	15, IBB AVENUE, UYO	Uyo	Akwa Ibom
371	AK/0078	AK/0078/S/5	UNIC DIAGNOSTIC LABORATORY	Laboratory	10 GIBBS STREET, UYO	Uyo	Akwa Ibom
372	AK/0080	AK/0080/S/13	Canon Eye Clinic & Optometric Services Ltd	Optometry	89 Aka Road	Uyo	Akwa Ibom
373	AK/0082	AK/0082/S/3	Our Lady of Lourdes Infirmary	O&G	22 Ekpanya Street Uyo	Uyo	Akwa Ibom
374	AK/0082	AK/0082/S/1	Our Lady of Lourdes Infirmary	O&G	22 Ekpanya Street Uyo	Uyo	Akwa Ibom
375	AK/0084	AK/0084/S/1	Etuknwa Specialist Clinic		120,Udo-Umana Street, Uyo Akwa Ibom State	Uyo	Akwa Ibom
376	AK/0085	AK/0085/S/14	Dunamis Clinic And Maternity		25 A,Afia Etid Street, Off Udo Umana Street, Uyo Akwa Ibom State	Uyo	Akwa Ibom
377	AK/0087	AK/0087/S/3	Jeconiah Specialist Hospital		Off 308,Oron Road, Uyo Akwa Ibom State	Uyo	Akwa Ibom
378	AK/0087	AK/0087/S/6	Jeconiah Specialist Hospital		Off 308,Oron Road, Uyo Akwa Ibom State	Uyo	Akwa Ibom
379	AK/0087	AK/0087/S/1	Jeconiah Specialist Hospital		Off 308,Oron Road, Uyo Akwa Ibom State	Uyo	Akwa Ibom
380	AK/0090	AK/0090/S/6	Petalice Medical Centre LTD		No 5/7 Marickson Hospital Way Akwa Ibom State	Uyo	Akwa Ibom
381	AK/0090	AK/0090/S/3	Petalice Medical Centre LTD		No 5/7 Marickson Hospital Way Akwa Ibom State	Uyo	Akwa Ibom
382	AK/0090	AK/0090/S/2	Petalice Medical Centre LTD		No 5/7 Marickson Hospital Way Akwa Ibom State	Uyo	Akwa Ibom
383	AK/0090	AK/0090/S/1	Petalice Medical Centre LTD		No 5/7 Marickson Hospital Way Akwa Ibom State	Uyo	Akwa Ibom
384	AK/0092	AK/0092/S/3	Premier Medical Services		3,High Tension Line,Edet Akpan Avenue, Uyo Akwa Ibom State	Uyo	Akwa Ibom
385	AK/0092	AK/0092/S/7	Premier Medical Services		3,High Tension Line,Edet Akpan Avenue, Uyo Akwa Ibom State	Uyo	Akwa Ibom

386	AK/0092	AK/0092/S/6	Premier Medical Services		3,High Tension Line,Edet Akpan Avenue, Uyo Akwa Ibom State	Uyo	Akwa Ibom
387	AK/0093	AK/0093/S/6	Queen Esther Specialist Hospital		Ediye Samuel Street,Itiam Etoi, Uyo Akwa Ibom State	Uyo	Akwa Ibom
388	AK/0095	AK/0095/S/3	St. Lukes Hospital		Anua P.M.B. 3, Uyo, Akwa Ibom State	Uyo	Akwa Ibom
389	AK/0095	AK/0095/S/7	St. Lukes Hospital		Anua P.M.B. 3, Uyo, Akwa Ibom State	Uyo	Akwa Ibom
390	AK/0095	AK/0095/S/11	St. Lukes Hospital		Anua P.M.B. 3, Uyo, Akwa Ibom State	Uyo	Akwa Ibom
391	AK/0095	AK/0095/S/2	St. Lukes Hospital		Anua P.M.B. 3, Uyo, Akwa Ibom State	Uyo	Akwa Ibom
392	AK/0095	AK/0095/S/1	St. Lukes Hospital		Anua P.M.B. 3, Uyo, Akwa Ibom State	Uyo	Akwa Ibom
393	AK/0095	AK/0095/S/4	St. Lukes Hospital		Anua P.M.B. 3, Uyo, Akwa Ibom State	Uyo	Akwa Ibom
394	AK/0095	AK/0095/S/5	St. Lukes Hospital		Anua P.M.B. 3, Uyo, Akwa Ibom State	Uyo	Akwa Ibom
395	AK/0095	AK/0095/S/6	St. Lukes Hospital		Anua P.M.B. 3, Uyo, Akwa Ibom State	Uyo	Akwa Ibom
396	AK/0095	AK/0095/S/8	St. Lukes Hospital		Anua P.M.B. 3, Uyo, Akwa Ibom State	Uyo	Akwa Ibom
397	AK/0097	AK/0097/S/1	Staphender Specialist klinic		3,Ibokette Street Off Aka Road, Uyo Akwa Ibom State	Uyo	Akwa Ibom
398	AK/0099	AK/0099/S/3	Nissi Specialist Hospital		Plot 42,Line H, Ewet Housing Estate, Uyo Akwa Ibom State	Uyo	Akwa Ibom
399	AK/0102	AK/0102/S/14	Full Care Ultrasound Diagnostic Center		Full Care Ultrasound Diagnostic Center	Uyo	Akwa Ibom
400	AK/0105	AK/0105/S/5	Dyme Hospital & Maternity Ltd		10,Edem Urua Street, Uyo, Akwa-Ibom State	Uyo	Akwa Ibom
401	AK/0105	AK/0105/S/3	Dyme Hospital & Maternity Ltd		10,Edem Urua Street, Uyo, Akwa-Ibom State	Uyo	Akwa Ibom
402	AK/0107	AK/0107/S/14	Coray Specialist Clinic & Maternity		8, Ibiam street, Uyo. Akwa Ibom state	Uyo	Akwa Ibom
403	AK/0107	AK/0107/S/3	Coray Specialist Clinic & Maternity		8, Ibiam street, Uyo. Akwa Ibom state	Uyo	Akwa Ibom
404	AK/0108	AK/0108/S/8	Winners Dental		56, Nepa line, Uyo. Akwa Ibom State.	Uyo	Akwa Ibom
405	AK/0111	AK/0111/S/14	Zoe Consultants Hospital		33, Enwe street, Uyo. Akwa Ibom state.	Uyo	Akwa Ibom
406	AK/0111	AK/0111/S/1	Zoe Consultants Hospital		33, Enwe street, Uyo. Akwa Ibom state.	Uyo	Akwa Ibom
407	AK/0111	AK/0111/S/3	Zoe Consultants Hospital		33, Enwe street, Uyo. Akwa Ibom state.	Uyo	Akwa Ibom
408	AK/0111	AK/0111/S/2	Zoe Consultants Hospital		33, Enwe street, Uyo. Akwa Ibom state.	Uyo	Akwa Ibom
409	AK/0112	AK/0112/S/13	Nathel Optical Services.		65, Iboko Street, Uyo. Akwa Ibom State.	Uyo	Akwa Ibom
410	AK/0113	AK/0113/S/8	Ark Dental and Maxillofacial Clinic Ltd		32, Ikot Ebido Street, opp. Winners Chapel, Uyo, Akwa Ibom state.	Uyo	Akwa Ibom
411	AK/0115	AK/0115/S/2	San Dominique Hospital		Plot 1, Unit M, Ewet Housing Estate,Uyo. Akwa Ibom state.	Uyo	Akwa Ibom
412	AK/0115	AK/0115/S/3	San Dominique Hospital		Plot 1, Unit M, Ewet Housing Estate,Uyo. Akwa Ibom state.	Uyo	Akwa Ibom
413	AK/0115	AK/0115/S/5	San Dominique Hospital		Plot 1, Unit M, Ewet Housing Estate,Uyo. Akwa Ibom state.	Uyo	Akwa Ibom
414	AK/0115	AK/0115/S/11	San Dominique Hospital		Plot 1, Unit M, Ewet Housing Estate,Uyo. Akwa Ibom state.	Uyo	Akwa Ibom
415	AK/0115	AK/0115/S/15	San Dominique Hospital		Plot 1, Unit M, Ewet Housing Estate,Uyo. Akwa Ibom state.	Uyo	Akwa Ibom
416	AK/0116	AK/0116/S/3	Good Health Specialist Clinics Ltd		26, Udobio Street, Uyo, Akwa Ibom State.	Uyo	Akwa Ibom
417	AN/0003	AN/0003/S/1	Harmony Hospital		No. 5 Oraogbu Street, Awka, Anambra State.	Awka South	Anambra
418	AN/0004	AN/0004/S/1	Beacon Hospital		12. Umudora Street, Awka, Anambra State.	Awka South	Anambra
419	AN/0004	AN/0004/S/3	Beacon Hospital		12. Umudora Street, Awka, Anambra State.	Awka South	Anambra
420	AN/0004	AN/0004/S/2	Beacon Hospital		12. Umudora Street, Awka, Anambra State.	Awka South	Anambra
421	AN/0008	AN/0008/S/1	Nnamdi Azikiwe University Teaching Hospital		Nnewi, Anambra State	Nnewi North	Anambra
422	AN/0008	AN/0008/S/2	Nnamdi Azikiwe University Teaching Hospital		Nnewi, Anambra State	Nnewi North	Anambra
423	AN/0008	AN/0008/S/4	Nnamdi Azikiwe University Teaching Hospital		Nnewi, Anambra State	Nnewi North	Anambra

424	AN/0008	AN/0008/S/6	Nnamdi Azikiwe University Teaching Hospital		Nnewi, Anambra State	Nnewi North	Anambra
425	AN/0008	AN/0008/S/11	Nnamdi Azikiwe University Teaching Hospital		Nnewi, Anambra State	Nnewi North	Anambra
426	AN/0008	AN/0008/S/15	Nnamdi Azikiwe University Teaching Hospital		Nnewi, Anambra State	Nnewi North	Anambra
427	AN/0008	AN/0008/S/14	Nnamdi Azikiwe University Teaching Hospital		Nnewi, Anambra State	Nnewi North	Anambra
428	AN/0008	AN/0008/S/12	Nnamdi Azikiwe University Teaching Hospital		Nnewi, Anambra State	Nnewi North	Anambra
429	AN/0008	AN/0008/S/7	Nnamdi Azikiwe University Teaching Hospital		Nnewi, Anambra State	Nnewi North	Anambra
430	AN/0008	AN/0008/S/5	Nnamdi Azikiwe University Teaching Hospital		Nnewi, Anambra State	Nnewi North	Anambra
431	AN/0008	AN/0008/S/3	Nnamdi Azikiwe University Teaching Hospital		Nnewi, Anambra State	Nnewi North	Anambra
432	AN/0012	AN/0012/S/4	General Hospital Onitsha		Hospital Road, Onitsha, Anambra State.	Onitsha-North	Anambra
433	AN/0012	AN/0012/S/15	General Hospital Onitsha		Hospital Road, Onitsha, Anambra State.	Onitsha-North	Anambra
434	AN/0012	AN/0012/S/6	General Hospital Onitsha		Hospital Road, Onitsha, Anambra State.	Onitsha-North	Anambra
435	AN/0012	AN/0012/S/5	General Hospital Onitsha		Hospital Road, Onitsha, Anambra State.	Onitsha-North	Anambra
436	AN/0012	AN/0012/S/3	General Hospital Onitsha		Hospital Road, Onitsha, Anambra State.	Onitsha-North	Anambra
437	AN/0012	AN/0012/S/1	General Hospital Onitsha		Hospital Road, Onitsha, Anambra State.	Onitsha-North	Anambra
438	AN/0015	AN/0015/S/1	Menax Hospital		Onowu Anatogu, St. Woliwo Layout Onitsha,	Onitsha-North	Anambra
439	AN/0015	AN/0015/S/3	Menax Hospital		Onowu Anatogu, St. Woliwo Layout Onitsha,	Onitsha-North	Anambra
440	AN/0016	AN/0016/S/14	Rush Green Maternity Hosp. & Clinic		64, Ugwunobamkpa Road, Near Isokwe Police Post, Onitsha.	Onitsha-North	Anambra
441	AN/0017	AN/0017/S/5	New Hope Medical Centre		80, Modebe Avenue, Odoakpu, Onitsha, Anambra State.	Onitsha-North	Anambra
442	AN/0017	AN/0017/S/7	New Hope Medical Centre		80, Modebe Avenue, Odoakpu, Onitsha, Anambra State.	Onitsha-North	Anambra
443	AN/0018	AN/0018/S/3	Venik Specalist Hospital		No. 1 Olo Street, Omagba Layout Onitsha, Anambra State.	Onitsha-North	Anambra
444	AN/0018	AN/0018/S/14	Venik Specalist Hospital		No. 1 Olo Street, Omagba Layout Onitsha, Anambra State.	Onitsha-North	Anambra
445	AN/0027	AN/0027/S/3	Mezie Hospital & Maternity		103b Upper New Market, Onitsha, Anambra	Onitsha -South	Anambra
446	AN/0029	AN/0029/S/1	Ace Specialist Hospital		73b Limca Road/1umuoji Street Onitsha, Anambra State.	Onitsha -South	Anambra
447	AN/0041	AN/0041/S/15	Nosco Eye Hospital	Ophthalmology	152 Niger Bridge, Approach, Onitsha, Anambra State	Onitsha-North	Anambra
448	AN/0042	AN/0042/S/6	Prime Concept Consultants Children's Specialist Hosp.	Paediatrics	36, Obosi Road, Nkpor, Anambra State.	Idemili North	Anambra
449	AN/0043	AN/0043/S/4	Junno Pharm. Ltd.	Pharmacy	Nnobi Road Otolo-Nnewi Anambra State.	Nnewi North	Anambra
450	AN/0044	AN/0044/S/4	Rams Gate Pharm	Pharmacy	117P/Harcourt Rd, Fegge, Onitsha, Anambra State.	Onitsha-North	Anambra
451	AN/0045	AN/0045/S/4	Paxs Pharm. Ltd.	Pharmacy	No. 80 Awka Road, Onitsha, Anambra State	Onitsha-North	Anambra
452	AN/0046	AN/0046/S/4	Canif Pharm. Ltd.	Pharmacy	100 Awka Road, Onitsha, Anambra State.	Onitsha-North	Anambra
453	AN/0047	AN/0047/S/4	Pitasen Pharmacy Ltd.	Pharmacy	58. Afani Road, Iyiowo Odekpe, Anambra	Ogbaru	Anambra
454	AN/0048	AN/0048/S/4	God,s Care Pharmaceutical Ltd.	Pharmacy	13, Ihembosi Street, Off, PH Rd., Woliwo Layout, Anambra Sta	Onitsha-North	Anambra
455	AN/0050	AN/0050/S/4	Samino Pharmacy	Pharmacy	30, Nwaziki Avenue, Awada, Onitsha, Anambra State	Onitsha-North	Anambra
456	AN/0052	AN/0052/S/4	Arimans Pharmacy	Pharmacy	1 Court Road, Onitsha, Anambra	Onitsha-North	Anambra
457	AN/0053	AN/0053/S/4	Karibu Pharmaceuticals Ltd.	Pharmacy	8A, Upper New Market Road, Onitsha, Anambra State.	Onitsha-North	Anambra
458	AN/0054	AN/0054/S/4	Wisdom Pharmacy Ltd.	Pharmacy	136, Oguta Road, Onitsha, Anambra State.	Onitsha-North	Anambra

459	AN/0056	AN/0056/S/4	Hallywood Chemists	Pharmacy	38, Oguta Road, Onitsha, Anambra State	Onitsha-North	Anambra
460	AN/0060	AN/0060/S/4	Crispa Solar Pharm. Ltd.	Pharmacy	3, Ogbunike Street,Nkpor Ago, Onitsha, Anambra State.	Idemili North	Anambra
461	AN/0061	AN/0061/S/4	Paxs Pharmaceuticals Ltd.,	Pharmacy	2, Market Road, Nkpor Junction Onitsha, Anambra	Idemili North	Anambra
462	AN/0062	AN/0062/S/4	Selar Pharmacy Ltd.	Pharmacy	101 Onitsha Road, Nnewi, Anambra State.	Nnewi North	Anambra
463	AN/0065	AN/0065/S/4	Lillimeg Pharmacy	Pharmacy	378, Azikiwe Avenue, Awka, Anambra State	Anaocha	Anambra
464	AN/0066	AN/0066/S/4	Georgie Chemist	Pharmacy	69, Ziks Avenue, Awka, Anambra State.	Onitsha-North	Anambra
465	AN/0067	AN/0067/S	Wikason Pharmacuticals	Pharmacy	1 Nwagu Junction, Nwagu, Agulu, Anambra State.	Onitsha-North	Anambra
466	AN/0068	AN/0068/S/5	Chuks Medical Diagnostic Lab.	Laboratory	8A Bishop Onyeabor Street Inland Town, Onitsha,	Onitsha-North	Anambra
467	AN/0070	AN/0070/S/5	Fezi Med. Laboratory	Laboratory	62A Old Market Road, Onitsha, Anambra	Onitsha-North	Anambra
468	AN/0071	AN/0071/S/5	Lisom Reference Laboratory	Laboratory	46 Old Market Road, Onitsha, Anambra State	Onitsha-North	Anambra
469	AN/0073	AN/0073/S/5	Gem Medical Laboratories	Laboratory	3A Tasia Road, Onitsha, Anambra State.	Onitsha-North	Anambra
470	AN/0074	AN/0074/S/5	Olive Medical Laboratory	Laboratory	51 Old Market Road, Onitsha, Anambra State	Onitsha-North	Anambra
471	AN/0075	AN/0075/S/5	Dozie Medical Laboratory	Laboratory	14, Awka Road, Onitsha, Anambra State	Idemili North	Anambra
472	AN/0077	AN/0077/S/5	Chuks Med. Lab.	Laboratory	48 Owerri, Road, Nnewi, Onitsha	Nnewi South	Anambra
473	AN/0080	AN/0080/S/5	Glanson Medical Laboratory	Laboratory	Awka, Anambra State	Awka North	Anambra
474	AN/0086	AN/0086/S/4	Holy Rosary Specialist Hospital		Waterside, Along Bank of the Niger, Onitsha, Anambra State	Onitsha-North	Anambra
475	AN/0086	AN/0086/S/3	Holy Rosary Specialist Hospital		Waterside, Along Bank of the Niger, Onitsha, Anambra State	Onitsha-North	Anambra
476	AN/0086	AN/0086/S/5	Holy Rosary Specialist Hospital		Waterside, Along Bank of the Niger, Onitsha, Anambra State	Onitsha-North	Anambra
477	AN/0086	AN/0086/S/2	Holy Rosary Specialist Hospital		Waterside, Along Bank of the Niger, Onitsha, Anambra State	Onitsha-North	Anambra
478	AN/0086	AN/0086/S/14	Holy Rosary Specialist Hospital		Waterside, Along Bank of the Niger, Onitsha, Anambra State	Onitsha-North	Anambra
479	AN/0087	AN/0087/S/5	Ifebi Medical Centre		16A, Igwebuike School Road, Awka, Anambra State	Awka South	Anambra
480	AN/0087	AN/0087/S/2	Ifebi Medical Centre		16A, Igwebuike School Road, Awka, Anambra State	Awka South	Anambra
481	AN/0087	AN/0087/S/7	Ifebi Medical Centre		16A, Igwebuike School Road, Awka, Anambra State	Awka South	Anambra
482	AN/0087	AN/0087/S/14	Ifebi Medical Centre		16A, Igwebuike School Road, Awka, Anambra State	Awka South	Anambra
483	AN/0088	AN/0088/S/14	Beluchukwu Memorial Hospital		Nkwo, Enugu-Ukwu, Anambra State	Njikoka	Anambra
484	AN/0088	AN/0088/S/5	Beluchukwu Memorial Hospital		Nkwo, Enugu-Ukwu, Anambra State	Njikoka	Anambra
485	AN/0088	AN/0088/S/7	Beluchukwu Memorial Hospital		Nkwo, Enugu-Ukwu, Anambra State	Njikoka	Anambra
486	AN/0091	AN/0091/S/11	St. Charles Borromeo Hospital		Off Limca Road, Onitsha, Anambra State	Onitsha-North	Anambra
487	AN/0091	AN/0091/S/4	St. Charles Borromeo Hospital		Off Limca Road, Onitsha, Anambra State	Onitsha-North	Anambra
488	AN/0091	AN/0091/S/5	St. Charles Borromeo Hospital		Off Limca Road, Onitsha, Anambra State	Onitsha-North	Anambra
489	AN/0091	AN/0091/S/1	St. Charles Borromeo Hospital		Off Limca Road, Onitsha, Anambra State	Onitsha-North	Anambra
490	AN/0091	AN/0091/S/6	St. Charles Borromeo Hospital		Off Limca Road, Onitsha, Anambra State	Onitsha-North	Anambra
491	AN/0097	AN/0097/S/3	The Light Specialist Hospital		No. 40, Eme Court Rd., Umudim, Nnewi, Anambra State	Nnewi North	Anambra
492	AN/0099	AN/0099/S/1	Chimex Specialist Hospital		No. 87 Ezemewi Road, Nnewichi, Nnewi, Anambra State	Nnewi North	Anambra
493	AN/0099	AN/0099/S/5	Chimex Specialist Hospital		No. 87 Ezemewi Road, Nnewichi, Nnewi, Anambra State	Nnewi North	Anambra
494	AN/0101	AN/0101/S/4	Zion Noel Pharmacy Ltd	Pharmacy	No 5 Oranye Street Off Old mkt Rd Onitsha	Onitsha-North	Anambra
495	AN/0102	AN/0102/S/4	Gauze Pharmacy	Pharmacy	No 10 Oko Road Ekuloba Aguata	Aguata	Anambra

496	AN/0103	AN/0103/S/11	St Charles Borromeo Hospital	General Surgery, O&G, Int. medicine, Paediatrics, Physiotherapy, Pharmacy, Laboratory	Off Limca Road Onitsha Anambra St	Onitsha-North	Anambra
497	AN/0103	AN/0103/S/6	St Charles Borromeo Hospital	General Surgery, O&G, Int. medicine, Paediatrics, Physiotherapy, Pharmacy, Laboratory	Off Limca Road Onitsha Anambra St	Onitsha-North	Anambra
498	AN/0103	AN/0103/S/1	St Charles Borromeo Hospital	General Surgery, O&G, Int. medicine, Paediatrics, Physiotherapy, Pharmacy, Laboratory	Off Limca Road Onitsha Anambra St	Onitsha-North	Anambra
499	AN/0103	AN/0103/S/3	St Charles Borromeo Hospital	General Surgery, O&G, Int. medicine, Paediatrics, Physiotherapy, Pharmacy, Laboratory	Off Limca Road Onitsha Anambra St	Onitsha-North	Anambra
500	AN/0103	AN/0103/S/5	St Charles Borromeo Hospital	General Surgery, O&G, Int. medicine, Paediatrics, Physiotherapy, Pharmacy, Laboratory	Off Limca Road Onitsha Anambra St	Onitsha-North	Anambra
501	AN/0103	AN/0103/S/4	St Charles Borromeo Hospital	General Surgery, O&G, Int. medicine, Paediatrics, Physiotherapy, Pharmacy, Laboratory	Off Limca Road Onitsha Anambra St	Onitsha-North	Anambra
502	AN/0103	AN/0103/S/2	St Charles Borromeo Hospital	General Surgery, O&G, Int. medicine, Paediatrics, Physiotherapy, Pharmacy, Laboratory	Off Limca Road Onitsha Anambra St	Onitsha-North	Anambra
503	AN/0105	AN/0105/S/5	Queen's Laboratory	Laboratory	129 P/H Road Fegge, Onitsha	Onitsha -South	Anambra
504	AN/0119	AN/0119/S/3	The Life Specialist Hospital	O&G, Laboratory	No. 7, Ikemba Drive, Umudim, Nnewi, Anambra State	Nnewi North	Anambra
505	AN/0119	AN/0119/S/5	The Life Specialist Hospital	O&G, Laboratory	No. 7, Ikemba Drive, Umudim, Nnewi, Anambra State	Nnewi North	Anambra
506	AN/0123	AN/0123/S/1	Sefton Specialist Medical Centre		5, Onubuya Street, off Court Road, Akwa	Awka North	Anambra
507	AN/0123	AN/0123/S/5	Sefton Specialist Medical Centre		5, Onubuya Street, off Court Road, Akwa	Awka North	Anambra
508	AN/0123	AN/0123/S/7	Sefton Specialist Medical Centre		5, Onubuya Street, off Court Road, Akwa	Awka North	Anambra
509	AN/0124	AN/0124/S/5	Federal College of Education (Technical)	Primary, Pharmacy, Laboratory	Umunze, Anambra State	Orumba South	Anambra
510	AN/0124	AN/0124/S/4	Federal College of Education (Technical)	Primary, Pharmacy, Laboratory	Umunze, Anambra State	Orumba South	Anambra
511	AN/0133	AN/0133/S/7	CROWN HOSPITAL		UMUOJI ROAD,MKPOR, ANAMBRA STATE	Idemili North	Anambra
512	AN/0133	AN/0133/S/5	CROWN HOSPITAL		UMUOJI ROAD,MKPOR, ANAMBRA STATE	Idemili North	Anambra
513	AN/0135	AN/0135/S/5	Dionye Memorial Hospital		OKPO VILLAGE,EKWULOBIA, ANAMBRA	Aguata	Anambra
514	AN/0138	AN/0138/S/7	IMMACULATE HEART HOSPITAL AND MAT.		OLD ENUGU-ONITSHA	Idemili North	Anambra
515	AN/0138	AN/0138/S/3	IMMACULATE HEART HOSPITAL AND MAT.		OLD ENUGU-ONITSHA	Idemili North	Anambra
516	AN/0138	AN/0138/S/1	IMMACULATE HEART HOSPITAL AND MAT.		OLD ENUGU-ONITSHA	Idemili North	Anambra
517	AN/0138	AN/0138/S/5	IMMACULATE HEART HOSPITAL AND MAT.		OLD ENUGU-ONITSHA	Idemili North	Anambra
518	AN/0140	AN/0140/S/5	Nnamdi Azikwe University Medical Centre		AWKA	Awka South	Anambra
519	AN/0140	AN/0140/S/1	Nnamdi Azikwe University Medical Centre		AWKA	Awka South	Anambra

520	AN/0140	AN/0140/S/4	Nnamdi Azikwe University Medical Centre		AWKA	Awka South	Anambra
521	AN/0142	AN/0142/S/5	PARAGON HOSPITAL AND MAT.		7, BISHOP SHANAHAM STREET, FEGGE, ONITSHA	Onitsha -South	Anambra
522	AN/0147	AN/0147/S/14	BEACON HOSPITAL & MATERNITY	PAEDIATRICS	10 BEACON HOSPITAL STR, AWKA	Awka South	Anambra
523	AN/0147	AN/0147/S/5	BEACON HOSPITAL & MATERNITY	PAEDIATRICS	10 BEACON HOSPITAL STR, AWKA	Awka South	Anambra
524	AN/0147	AN/0147/S/6	BEACON HOSPITAL & MATERNITY	PAEDIATRICS	10 BEACON HOSPITAL STR, AWKA	Awka South	Anambra
525	AN/0149	AN/0149/S/4	ESKAY-NEZ	PHARMACY	114C AWKA RD, ONITSHA	Onitsha-North	Anambra
526	AN/0154	AN/0154/S/4	JONACO PHARMACY STORES LTD	PHARMACY	45 OGUATA RD, ONITSHA	Onitsha-North	Anambra
527	AN/0159	AN/0159/S	NEW HOPE HOSPITAL & MAT.	RADIOLOGY	26, UMUNNA STR,ONITSHA	Onitsha -South	Anambra
528	AN/0163	AN/0163/S/4	OMED PHARMACY	PHARMACY	47, MODEBE AV, ONITSHA	Onitsha -South	Anambra
529	AN/0165	AN/0165/S/7	PIONEER X- RAY	RADIOLOGY	5 ONWUBUYA STR, OFF COURT RD, AWKA	Awka South	Anambra
530	AN/0166	AN/0166/S/5	PRIME CONCEP CON. CHILD SPEC. HOSPITAL	LABORATORY	36, OBOSI RD	Idemili North	Anambra
531	AN/0171	AN/0171/S/1	Apex Medical Centre		Igbo-Ukwu	Aguata	Anambra
532	AN/0171	AN/0171/S/7	Apex Medical Centre		Igbo-Ukwu	Aguata	Anambra
533	AN/0171	AN/0171/S/3	Apex Medical Centre		Igbo-Ukwu	Aguata	Anambra
534	AN/0171	AN/0171/S/5	Apex Medical Centre		Igbo-Ukwu	Aguata	Anambra
535	AN/0173	AN/0173/S/3	Pioneer Int. Spec Hospital		Plot 193 Owele Ebo Estate, Onitsha	Onitsha -South	Anambra
536	AN/0173	AN/0173/S/5	Pioneer Int. Spec Hospital		Plot 193 Owele Ebo Estate, Onitsha	Onitsha -South	Anambra
537	AN/0182	AN/0182/S/5	Nkem Medical Laboratory	Laboratory	Umunze Orumba	Orumba South	Anambra
538	AN/0188	AN/0188/S/3	Divine Care Specialist Hospital & Maternity		77 Igwe Orizu Road, Otolo Nnewi	Nnewi North	Anambra
539	AN/0191	AN/0191/S/7	Bex Memorial Hospital		Km 1 Owerri Road, Onitsha	Onitsha-North	Anambra
540	AN/0191	AN/0191/S/1	Bex Memorial Hospital		Km 1 Owerri Road, Onitsha	Onitsha-North	Anambra
541	AN/0191	AN/0191/S/4	Bex Memorial Hospital		Km 1 Owerri Road, Onitsha	Onitsha-North	Anambra
542	AN/0191	AN/0191/S/3	Bex Memorial Hospital		Km 1 Owerri Road, Onitsha	Onitsha-North	Anambra
543	AN/0191	AN/0191/S/5	Bex Memorial Hospital		Km 1 Owerri Road, Onitsha	Onitsha-North	Anambra
544	AN/0191	AN/0191/S/2	Bex Memorial Hospital		Km 1 Owerri Road, Onitsha	Onitsha-North	Anambra
545	AN/0192	AN/0192/S/13	Faith Eye Clinic	Optometry	40B Modebe Avenue, Onitsha	Onitsha -South	Anambra
546	AN/0195	AN/0195/S/1	St Victoria Specialist Hospital & Maternity		61 Orlu Ekwulobia	Aguata	Anambra
547	AN/0195	AN/0195/S/2	St Victoria Specialist Hospital & Maternity		61 Orlu Ekwulobia	Aguata	Anambra
548	AN/0195	AN/0195/S/3	St Victoria Specialist Hospital & Maternity		61 Orlu Ekwulobia	Aguata	Anambra
549	AN/0195	AN/0195/S/6	St Victoria Specialist Hospital & Maternity		61 Orlu Ekwulobia	Aguata	Anambra
550	AN/0197	AN/0197/S/11	Omega Physiotherapy	Physiotherapy	46 Old Hospital Road, Onitsha	Onitsha -South	Anambra
551	AN/0203	AN/0203/S/3	Bethsaida Specialist Hospital		Plot CL6, Pond Street Housing Estate, Feggae, Onitsha	Onitsha -South	Anambra
552	AN/0214	AN/0214/S/5	Our Lady Of Lourdes Hospital		Ihiala, Anambra State	Ihala	Anambra
553	AN/0214	AN/0214/S/1	Our Lady Of Lourdes Hospital		Ihiala, Anambra State	Ihala	Anambra
554	AN/0214	AN/0214/S/3	Our Lady Of Lourdes Hospital		Ihiala, Anambra State	Ihala	Anambra
555	AN/0214	AN/0214/S/2	Our Lady Of Lourdes Hospital		Ihiala, Anambra State	Ihala	Anambra
556	AN/0214	AN/0214/S/6	Our Lady Of Lourdes Hospital		Ihiala, Anambra State	Ihala	Anambra
557	AN/0214	AN/0214/S/10	Our Lady Of Lourdes Hospital		Ihiala, Anambra State	Ihala	Anambra
558	AN/0215	AN/0215/S/5	St. Mary's Hospital & Maternity		Agba Village, Ekwulobia, Anambra State.	Aguata	Anambra
559	AN/0216	AN/0216/S/5	His Glory Hospital & Maternity		96 Arthur Eze Avenue, near Tracy Hotel, Awka	Awka North	Anambra
560	AN/0217	AN/0217/S/5	Kanayo Specialist Hospital & Maternity	Laboratory, O & G	17 Enugu Road, Onitsha	Onitsha -South	Anambra

561	AN/0217	AN/0217/S/3	Kanayo Specialist Hospital & Maternity	Laboratory, O & G	17 Enugu Road, Onitsha	Onitsha -South	Anambra
562	AN/0224	AN/0224/S/3	Obioma Specialist Hospital & maternity		19 Onwukwe Street Abunor Nnewichi,Nnewi Anambra State	Nnewi North	Anambra
563	AN/0225	AN/0225/S/3	St. Helen`s Specialist Hospital		Agbou Estate Behind Kenneth Dike Sec Sch Off Obinagu, Amawbia Awka Anambra State	Awka South	Anambra
564	AN/0226	AN/0226/S/7	Toronto Hospital limited		2,Upper Niger Bridge Onitsha Anambra State	Onitsha -South	Anambra
565	AN/0226	AN/0226/S/5	Toronto Hospital limited		2,Upper Niger Bridge Onitsha Anambra State	Onitsha -South	Anambra
566	AN/0226	AN/0226/S/15	Toronto Hospital limited		2,Upper Niger Bridge Onitsha Anambra State	Onitsha -South	Anambra
567	AN/0230	AN/0230/S/2	Okoye Specialist Hospital		Barr Ajekwe Close Off 25 Emma	Awka South	Anambra
568	AN/0231	AN/0231/S/14	Raginaceali Hospital		Raginaceali Road Awka Anambra State	Awka South	Anambra
569	AN/0231	AN/0231/S/5	Raginaceali Hospital		Raginaceali Road Awka Anambra State	Awka South	Anambra
570	AN/0231	AN/0231/S/12	Raginaceali Hospital		Raginaceali Road Awka Anambra State	Awka South	Anambra
571	AN/0231	AN/0231/S/2	Raginaceali Hospital		Raginaceali Road Awka Anambra State	Awka South	Anambra
572	AN/0231	AN/0231/S/18	Raginaceali Hospital		Raginaceali Road Awka Anambra State	Awka South	Anambra
573	AN/0231	AN/0231/S/3	Raginaceali Hospital		Raginaceali Road Awka Anambra State	Awka South	Anambra
574	AN/0231	AN/0231/S/11	Raginaceali Hospital		Raginaceali Road Awka Anambra State	Awka South	Anambra
575	AN/0234	AN/0234/S/5	Okpala Hospital And Maternity		Enugwu-ukwu Near Ezi-Elias Anambra State	Njikoka	Anambra
576	AN/0235	AN/0235/S/3	Anambra State University Teaching Hospital		Awka Amaku Anambra State	Awka South	Anambra
577	AN/0235	AN/0235/S/10	Anambra State University Teaching Hospital		Awka Amaku Anambra State	Awka South	Anambra
578	AN/0235	AN/0235/S/15	Anambra State University Teaching Hospital		Awka Amaku Anambra State	Awka South	Anambra
579	AN/0235	AN/0235/S/4	Anambra State University Teaching Hospital		Awka Amaku Anambra State	Awka South	Anambra
580	AN/0235	AN/0235/S/7	Anambra State University Teaching Hospital		Awka Amaku Anambra State	Awka South	Anambra
581	AN/0235	AN/0235/S/2	Anambra State University Teaching Hospital		Awka Amaku Anambra State	Awka South	Anambra
582	AN/0235	AN/0235/S/6	Anambra State University Teaching Hospital		Awka Amaku Anambra State	Awka South	Anambra
583	AN/0235	AN/0235/S/8	Anambra State University Teaching Hospital		Awka Amaku Anambra State	Awka South	Anambra
584	AN/0235	AN/0235/S/12	Anambra State University Teaching Hospital		Awka Amaku Anambra State	Awka South	Anambra
585	AN/0235	AN/0235/S/11	Anambra State University Teaching Hospital		Awka Amaku Anambra State	Awka South	Anambra
586	AN/0235	AN/0235/S/5	Anambra State University Teaching Hospital		Awka Amaku Anambra State	Awka South	Anambra
587	AN/0235	AN/0235/S/18	Anambra State University Teaching Hospital		Awka Amaku Anambra State	Awka South	Anambra
588	AN/0237	AN/0237/S/5	Isaac Chira Memorial Hospital		Awkuzu Anambra State	Oyi	Anambra
589	AN/0238	AN/0238/S/3	Cynvic Specialist Hospital		General Hospital Road Agba Ekwulobia Anambra State	Aguata	Anambra
590	AN/0238	AN/0238/S/1	Cynvic Specialist Hospital		General Hospital Road Agba Ekwulobia Anambra State	Aguata	Anambra
591	AN/0240	AN/0240/S/5	Our Lady Of Lourdes Hospital		Ihiala Anambra State	Ihala	Anambra
592	AN/0240	AN/0240/S/3	Our Lady Of Lourdes Hospital		Ihiala Anambra State	Ihala	Anambra
593	AN/0240	AN/0240/S/4	Our Lady Of Lourdes Hospital		Ihiala Anambra State	Ihala	Anambra
594	AN/0240	AN/0240/S/10	Our Lady Of Lourdes Hospital		Ihiala Anambra State	Ihala	Anambra
595	AN/0240	AN/0240/S/1	Our Lady Of Lourdes Hospital		Ihiala Anambra State	Ihala	Anambra

596	AN/0243	AN/0243/S/6	Prime Specialist Hospital		5, Nwufo Meniru Street Beside Bishop Crother Seminary, Awka Anambra State	Awka South	Anambra
597	AN/0245	AN/0245/S/11	Landmark Physiotherapy		Behind Anaedo Social Club Hqt Nnewi Anambra State	Nnewi North	Anambra
598	AN/0247	AN/0247/S/2	Nnewi Diocesan Hospital		Igwe Orizu Road Otolo, Nnewi Anambra State	Ogbaru	Anambra
599	AN/0249	AN/0249/S/3	Bethsaida Specialist Hospital		48b Nnobi Road Opp. Nnewi High School, Nnewi Anambra State	Awka South	Anambra
600	AN/0252	AN/0252/S/5	Safecheck Laboratories Limited		7No 74 F.G. Onyiuke Avenue, Nimo. Anambra State.	Njikoka	Anambra
601	AN/0253	AN/0253/S/1	Beke Memorial Hospital		64 F.G Onyiuke Avenue, Nimo Anambra State	Njikoka	Anambra
602	AN/0253	AN/0253/S/3	Beke Memorial Hospital		64 F.G Onyiuke Avenue, Nimo Anambra State	Njikoka	Anambra
603	AN/0253	AN/0253/S/2	Beke Memorial Hospital		64 F.G Onyiuke Avenue, Nimo Anambra State	Njikoka	Anambra
604	AN/0253	AN/0253/S/6	Beke Memorial Hospital		64 F.G Onyiuke Avenue, Nimo Anambra State	Njikoka	Anambra
605	AN/0254	AN/0254/S/3	Obioma Hospital		5/7 Anuka Road Otolo, Nnewi Anambra State.	Nnewi North	Anambra
606	AN/0255	AN/0255/S/3	Mercy Specialist Hospital And Maternity		4A New American Road, Onitcha, Anambra State	Onitsha-North	Anambra
607	AN/0255	AN/0255/S/1	Mercy Specialist Hospital And Maternity		4A New American Road, Onitcha, Anambra State	Onitsha-North	Anambra
608	AN/0255	AN/0255/S/6	Mercy Specialist Hospital And Maternity		4A New American Road, Onitcha, Anambra State	Onitsha-North	Anambra
609	AN/0255	AN/0255/S/2	Mercy Specialist Hospital And Maternity		4A New American Road, Onitcha, Anambra State	Onitsha-North	Anambra
610	AN/0256	AN/0256/S/6	St. Joseph's Hospital		Adazi Nnukwu, Anambra State	Anaocha	Anambra
611	AN/0256	AN/0256/S/2	St. Joseph's Hospital		Adazi Nnukwu, Anambra State	Anaocha	Anambra
612	AN/0256	AN/0256/S/3	St. Joseph's Hospital		Adazi Nnukwu, Anambra State	Anaocha	Anambra
613	AN/0256	AN/0256/S/5	St. Joseph's Hospital		Adazi Nnukwu, Anambra State	Anaocha	Anambra
614	AN/0256	AN/0256/S/1	St. Joseph's Hospital		Adazi Nnukwu, Anambra State	Anaocha	Anambra
615	AN/0256	AN/0256/S/10	St. Joseph's Hospital		Adazi Nnukwu, Anambra State	Anaocha	Anambra
616	AN/0256	AN/0256/S/4	St. Joseph's Hospital		Adazi Nnukwu, Anambra State	Anaocha	Anambra
617	AN/0257	AN/0257/S/10	Regina Caeli Specialist Hospital		17, Regina Caeli Road, Awka Anambra State	Awka South	Anambra
618	AN/0257	AN/0257/S/7	Regina Caeli Specialist Hospital		17, Regina Caeli Road, Awka Anambra State	Awka South	Anambra
619	AN/0257	AN/0257/S/1	Regina Caeli Specialist Hospital		17, Regina Caeli Road, Awka Anambra State	Awka South	Anambra
620	AN/0259	AN/0259/S/11	Rice Clinic		Rise- Gopat Building Agulu-Nnobi Road Adazi-Ani, Anambra State	Anaocha	Anambra
621	AN/0259	AN/0259/S/5	Rice Clinic		Rise- Gopat Building Agulu-Nnobi Road Adazi-Ani, Anambra State	Anaocha	Anambra
622	AN/0259	AN/0259/S/7	Rice Clinic		Rise- Gopat Building Agulu-Nnobi Road Adazi-Ani, Anambra State	Anaocha	Anambra
623	AN/0259	AN/0259/S/13	Rice Clinic		Rise- Gopat Building Agulu-Nnobi Road Adazi-Ani, Anambra State	Anaocha	Anambra
624	AN/0259	AN/0259/S/4	Rice Clinic		Rise- Gopat Building Agulu-Nnobi Road Adazi-Ani, Anambra State	Anaocha	Anambra
625	AN/0259	AN/0259/S/3	Rice Clinic		Rise- Gopat Building Agulu-Nnobi Road Adazi-Ani, Anambra State	Anaocha	Anambra
626	AN/0260	AN/0260/S/6	Ezinne Hospital & Children Welfare Centre		1A Oboli Lane, DMGS Round About Onisha, Anambra State	Onitsha-North	Anambra
627	AN/0261	AN/0261/S/1	Kandudi Specialist Hospital		Oye Market, Achina Anambra State	Aguata	Anambra
628	AN/0261	AN/0261/S/3	Kandudi Specialist Hospital		Oye Market, Achina Anambra State	Aguata	Anambra
629	AN/0262	AN/0262/S/5	Bright Medical Laboratory		Oye Market Achina, Anambra State	Aguata	Anambra
630	AN/0263	AN/0263/S/14	Dike Medical Centre		Ebenebe Anambra State	Awka North	Anambra
631	AN/0266	AN/0266/S/4	Joez Pharmacy		81, Zik Avenue Awka, Anambra State	Awka South	Anambra

632	AN/0267	AN/0267/S/1	Edozie Hospital & Maternity		5, Omanukwue Crescent, Obieze Village, Ifite Duru, Anambra State	Dunukofia	Anambra
633	AN/0267	AN/0267/S/14	Edozie Hospital & Maternity		5, Omanukwue Crescent, Obieze Village, Ifite Duru, Anambra State	Dunukofia	Anambra
634	AN/0267	AN/0267/S/3	Edozie Hospital & Maternity		5, Omanukwue Crescent, Obieze Village, Ifite Duru, Anambra State	Dunukofia	Anambra
635	AN/0267	AN/0267/S/2	Edozie Hospital & Maternity		5, Omanukwue Crescent, Obieze Village, Ifite Duru, Anambra State	Dunukofia	Anambra
636	AN/0268	AN/0268/S/6	Obijackson Hospital		No 2 Madonna University Road, Okija, Anambra State	Ihala	Anambra
637	AN/0268	AN/0268/S/5	Obijackson Hospital		No 2 Madonna University Road, Okija, Anambra State	Ihala	Anambra
638	AN/0268	AN/0268/S/4	Obijackson Hospital		No 2 Madonna University Road, Okija, Anambra State	Ihala	Anambra
639	AN/0268	AN/0268/S/3	Obijackson Hospital		No 2 Madonna University Road, Okija, Anambra State	Ihala	Anambra
640	AN/0270	AN/0270/S/13	Hepzibah Eye Clinic		40 Court Road, Awka Anambra State	Awka South	Anambra
641	AN/0271	AN/0271/S/5	Sun Medical Diagnostic		General Hospital ,Awka Road, Onitsha Anambra State	Onitsha-North	Anambra
642	AN/0271	AN/0271/S/7	Sun Medical Diagnostic		General Hospital ,Awka Road, Onitsha Anambra State	Onitsha-North	Anambra
643	AN/0274	AN/0274/S/5	Aguata Diocesan Hospital Igboukwu		Divine Grace Villa 2 Rd 3 Abuja Estate Awka, Anambra State	Awka South	Anambra
644	AN/0274	AN/0274/S/3	Aguata Diocesan Hospital Igboukwu		Divine Grace Villa 2 Rd 3 Abuja Estate Awka, Anambra State	Awka South	Anambra
645	AN/0276	AN/0276/S/5	Ennembo Biomedical Diagnostic Laboratory		23,Ugwunabankpa Rd, Onitsha Anambra State	Onitsha-North	Anambra
646	BA/0002	BA/0002/S/3	Mai Jama'A Clinic		Wunti Street, Bauchi	Bauchi	Bauchi
647	BA/0002	BA/0002/S/1	Mai Jama'A Clinic		Wunti Street, Bauchi	Bauchi	Bauchi
648	BA/0006	BA/0006/S/8	Abubakar Tafawa Balewa University Teaching Hospital	O & G, Gen. Surg., Paediatrics, ENT, Pharm., Radiology, Physiotherapy, Dental	Bauchi, Bauchi State	Bauchi	Bauchi
649	BA/0006	BA/0006/S/1	Abubakar Tafawa Balewa University Teaching Hospital	O & G, Gen. Surg., Paediatrics, ENT, Pharm., Radiology, Physiotherapy, Dental	Bauchi, Bauchi State	Bauchi	Bauchi
650	BA/0006	BA/0006/S/12	Abubakar Tafawa Balewa University Teaching Hospital	O & G, Gen. Surg., Paediatrics, ENT, Pharm., Radiology, Physiotherapy, Dental	Bauchi, Bauchi State	Bauchi	Bauchi
651	BA/0006	BA/0006/S/3	Abubakar Tafawa Balewa University Teaching Hospital	O & G, Gen. Surg., Paediatrics, ENT, Pharm., Radiology, Physiotherapy, Dental	Bauchi, Bauchi State	Bauchi	Bauchi
652	BA/0006	BA/0006/S/6	Abubakar Tafawa Balewa University Teaching Hospital	O & G, Gen. Surg., Paediatrics, ENT, Pharm., Radiology, Physiotherapy, Dental	Bauchi, Bauchi State	Bauchi	Bauchi
653	BA/0006	BA/0006/S/7	Abubakar Tafawa Balewa University Teaching Hospital	O & G, Gen. Surg., Paediatrics, ENT, Pharm., Radiology, Physiotherapy, Dental	Bauchi, Bauchi State	Bauchi	Bauchi

654	BA/0006	BA/0006/S/11	Abubakar Tafawa Balewa University Teaching Hospital	O & G, Gen. Surg., Paediatrics, ENT, Pharm., Radiology, Physiotherapy, Dental	Bauchi, Bauchi State	Bauchi	Bauchi
655	BA/0006	BA/0006/S/4	Abubakar Tafawa Balewa University Teaching Hospital	O & G, Gen. Surg., Paediatrics, ENT, Pharm., Radiology, Physiotherapy, Dental	Bauchi, Bauchi State	Bauchi	Bauchi
656	BA/0010	BA/0010/S/4	Sahelian Pharm. Ltd.,	Pharmacy	No. 6, Yandoka Road, P.M.B 2505, Bauchi	Bauchi	Bauchi
657	BA/0012	BA/0012/S/4	Tinma Pharmaceutical Chemist	Pharmacy	C1 Kobi Street, Bauchi	Bauchi	Bauchi
658	BA/0014	BA/0014/S/5	Lisa Laboratory	Laboratory	No. 1, Boni Haruna Street, Bauchi.	Bauchi	Bauchi
659	BA/0015	BA/0015/S/5	Gaskiya Laboratory	Laboratory	Ran Road, Bauchi	Bauchi	Bauchi
660	BA/0016	BA/0016/S/4	Nana Rich Med. Laboratory	Laboratory	Auwal Shopping Complex, Johun Road, Bauchi	Bauchi	Bauchi
661	BA/0017	BA/0017/S/4	Safara Pharm. Ltd	Pharmacy	No. 5, Hospital Road, Bauchi	Bauchi	Bauchi
662	BA/0023	BA/0023/S/3	Niima Consultant Hospital - Bauchi		Niima Close, Off Airport Rd. Bauchi	Bauchi	Bauchi
663	BA/0023	BA/0023/S/5	Niima Consultant Hospital - Bauchi		Niima Close, Off Airport Rd. Bauchi	Bauchi	Bauchi
664	BA/0023	BA/0023/S/4	Niima Consultant Hospital - Bauchi		Niima Close, Off Airport Rd. Bauchi	Bauchi	Bauchi
665	BA/0023	BA/0023/S/7	Niima Consultant Hospital - Bauchi		Niima Close, Off Airport Rd. Bauchi	Bauchi	Bauchi
666	BA/0023	BA/0023/S/6	Niima Consultant Hospital - Bauchi		Niima Close, Off Airport Rd. Bauchi	Bauchi	Bauchi
667	BA/0023	BA/0023/S/1	Niima Consultant Hospital - Bauchi		Niima Close, Off Airport Rd. Bauchi	Bauchi	Bauchi
668	BA/0023	BA/0023/S/8	Niima Consultant Hospital - Bauchi		Niima Close, Off Airport Rd. Bauchi	Bauchi	Bauchi
669	BA/0023	BA/0023/S/2	Niima Consultant Hospital - Bauchi		Niima Close, Off Airport Rd. Bauchi	Bauchi	Bauchi
670	BA/0025	BA/0025/S/15	Federal Medical Centre - Azare		P.M.B. 005, Azare, Bauchi State.	Katagum	Bauchi
671	BA/0025	BA/0025/S/12	Federal Medical Centre - Azare		P.M.B. 005, Azare, Bauchi State.	Katagum	Bauchi
672	BA/0025	BA/0025/S/3	Federal Medical Centre - Azare		P.M.B. 005, Azare, Bauchi State.	Katagum	Bauchi
673	BA/0025	BA/0025/S/4	Federal Medical Centre - Azare		P.M.B. 005, Azare, Bauchi State.	Katagum	Bauchi
674	BA/0025	BA/0025/S/6	Federal Medical Centre - Azare		P.M.B. 005, Azare, Bauchi State.	Katagum	Bauchi
675	BA/0025	BA/0025/S/7	Federal Medical Centre - Azare		P.M.B. 005, Azare, Bauchi State.	Katagum	Bauchi
676	BA/0025	BA/0025/S/5	Federal Medical Centre - Azare		P.M.B. 005, Azare, Bauchi State.	Katagum	Bauchi
677	BA/0025	BA/0025/S/1	Federal Medical Centre - Azare		P.M.B. 005, Azare, Bauchi State.	Katagum	Bauchi
678	BA/0027	BA/0027/S/8	33 AB MRS Bauchi		Bauchi, Bauchi State	Bauchi	Bauchi
679	BA/0027	BA/0027/S/5	33 AB MRS Bauchi		Bauchi, Bauchi State	Bauchi	Bauchi
680	BA/0033	BA/0033/S/4	Abubakar Tafawa Balewa Health Centre		Bauchi, Bauchi State	Bauchi	Bauchi
681	BA/0033	BA/0033/S/1	Abubakar Tafawa Balewa Health Centre		Bauchi, Bauchi State	Bauchi	Bauchi
682	BA/0067	BA/0067/S/6	Alwadata Consultant Clinic	PRIMARY	7 KWANI CLOSE FADAMI CLOSE MADA BAUCHI	Bauchi	Bauchi
683	BA/0073	BA/0073/S/4	NEEBEN PHARMACY LIMITED		B 295 WUNTI STREET- BAUCHI STATE	Itas/Gadau	Bauchi
684	BA/0074	BA/0074/S/8	Ubani Dental Clinic	Dental	1 Boni Haruna Street, Bauchi	Bauchi	Bauchi
685	BA/0077	BA/0077/S/4	Zamani Pharmaceutical Chemist	Pharmacy	Along Federal Lowcost/Railway Road	Bauchi	Bauchi
686	BA/0080	BA/0080/S/4	Yankari Pharmacy	Pharmacy	203 Railway Road, Bauchi	Bauchi	Bauchi
687	BA/0081	BA/0081/S/4	Frontier Pharmaceutical Ltd	Pharmacy	17 & 18 Basma Plaza, Yelwan Makaranta, Bauchi	Bauchi	Bauchi
688	BA/0091	BA/0091/S/15	Makkah Specialist Eye Hospital		Old Ministry of Works, Kobi Street Bauchi	Bauchi	Bauchi
689	BA/0091	BA/0091/S/13	Makkah Specialist Eye Hospital		Old Ministry of Works, Kobi Street Bauchi	Bauchi	Bauchi
690	BA/0092	BA/0092/S/4	National Fistula Clinic		Ningi, Bauchi State.	Ningi	Bauchi
691	BA/0093	BA/0093/S/4	Idyllic Pharmacare Nig. Limited.		A558, Ajiya Adamu Road, Bauchi, Bauchi State.	Bauchi	Bauchi

692	BA/0094	BA/0094/S/11	Ages Physiotherapy & Rehabilitation Clinic		5, Maiduguri Bye-Pass, Bauchi, Bauchi State	Bauchi	Bauchi
693	BA/0095	BA/0095/S/4	Allianz Preston Lue Pharmacy		No B288 Wunti Street Bauchi, Bauchi State.	Bauchi	Bauchi
694	BN/0003	BN/0003/S/5	TBT Hospital		91, Cape Doanes Way, Gboko	Gboko	Benue
695	BN/0011	BN/0011/S/7	General Hospital	Laboratory, Pharmacy, Radiology	Katsina-Ala	Katsina-Ala	Benue
696	BN/0012	BN/0012/S/3	Federal Medical Centre - Makurdi		Makurdi, Benue State	Makurdi	Benue
697	BN/0012	BN/0012/S/6	Federal Medical Centre - Makurdi		Makurdi, Benue State	Makurdi	Benue
698	BN/0012	BN/0012/S/15	Federal Medical Centre - Makurdi		Makurdi, Benue State	Makurdi	Benue
699	BN/0012	BN/0012/S/1	Federal Medical Centre - Makurdi		Makurdi, Benue State	Makurdi	Benue
700	BN/0015	BN/0015/S/5	Madonna Health Service Ltd.		(Madonna Hospital) New Bridge Road, Behind Union Bank Mkd	Makurdi	Benue
701	BN/0015	BN/0015/S/7	Madonna Health Service Ltd.		(Madonna Hospital) New Bridge Road, Behind Union Bank Mkd	Makurdi	Benue
702	BN/0015	BN/0015/S/1	Madonna Health Service Ltd.		(Madonna Hospital) New Bridge Road, Behind Union Bank Mkd	Makurdi	Benue
703	BN/0015	BN/0015/S/3	Madonna Health Service Ltd.		(Madonna Hospital) New Bridge Road, Behind Union Bank Mkd	Makurdi	Benue
704	BN/0015	BN/0015/S/6	Madonna Health Service Ltd.		(Madonna Hospital) New Bridge Road, Behind Union Bank Mkd	Makurdi	Benue
705	BN/0015	BN/0015/S/2	Madonna Health Service Ltd.		(Madonna Hospital) New Bridge Road, Behind Union Bank Mkd	Makurdi	Benue
706	BN/0018	BN/0018/S/4	Bishop Murray Medical Centre		Close To St. Theresa Church, High Level Makurdi	Makurdi	Benue
707	BN/0019	BN/0019/S/2	Sandra Hospital		24, J.S. Tarka Road, Opp. Maltino Filling Station, Makurdi	Makurdi	Benue
708	BN/0020	BN/0020/S/7	Immaculate Conception Hospital		Walla Mayo, High Level, Makurdi	Makurdi	Benue
709	BN/0020	BN/0020/S/1	Immaculate Conception Hospital		Walla Mayo, High Level, Makurdi	Makurdi	Benue
710	BN/0020	BN/0020/S/2	Immaculate Conception Hospital		Walla Mayo, High Level, Makurdi	Makurdi	Benue
711	BN/0020	BN/0020/S/10	Immaculate Conception Hospital		Walla Mayo, High Level, Makurdi	Makurdi	Benue
712	BN/0020	BN/0020/S/5	Immaculate Conception Hospital		Walla Mayo, High Level, Makurdi	Makurdi	Benue
713	BN/0030	BN/0030/S/6	Grace Cottage Hospital		7, David Mark Bye Pass, Makurdi	Makurdi	Benue
714	BN/0030	BN/0030/S/3	Grace Cottage Hospital		7, David Mark Bye Pass, Makurdi	Makurdi	Benue
715	BN/0030	BN/0030/S/1	Grace Cottage Hospital		7, David Mark Bye Pass, Makurdi	Makurdi	Benue
716	BN/0030	BN/0030/S/2	Grace Cottage Hospital		7, David Mark Bye Pass, Makurdi	Makurdi	Benue
717	BN/0031	BN/0031/S/4	Federal Medical Centre	O&G, Paediatrics, Gen. Surgery, Ophthalmology, Dentistry, Physiotherapy, Radiology, laboratory,pharmacy &internal medicine	Makurdi, Benue State	Makurdi	Benue
718	BN/0031	BN/0031/S/5	Federal Medical Centre	O&G, Paediatrics, Gen. Surgery, Ophthalmology, Dentistry, Physiotherapy, Radiology, laboratory,pharmacy &internal medicine	Makurdi, Benue State	Makurdi	Benue
719	BN/0031	BN/0031/S/7	Federal Medical Centre	O&G, Paediatrics, Gen. Surgery, Ophthalmology, Dentistry, Physiotherapy, Radiology, laboratory,pharmacy &internal medicine	Makurdi, Benue State	Makurdi	Benue

720	BN/0031	BN/0031/S/11	Federal Medical Centre	O&G, Paediatrics, Gen. Surgery, Ophthalmology, Dentistry, Physiotherapy, Radiology, laboratory,pharmacy &internal medicine	Makurdi, Benue State	Makurdi	Benue
721	BN/0031	BN/0031/S/8	Federal Medical Centre	O&G, Paediatrics, Gen. Surgery, Ophthalmology, Dentistry, Physiotherapy, Radiology, laboratory,pharmacy &internal medicine	Makurdi, Benue State	Makurdi	Benue
722	BN/0031	BN/0031/S/2	Federal Medical Centre	O&G, Paediatrics, Gen. Surgery, Ophthalmology, Dentistry, Physiotherapy, Radiology, laboratory,pharmacy &internal medicine	Makurdi, Benue State	Makurdi	Benue
723	BN/0033	BN/0033/S/4	Desmon Pharmacy & Allied	Pharmacy	Ventures 51, Railway Bye Pass, High Level Makurdi	Makurdi	Benue
724	BN/0035	BN/0035/S/4	Vical Pharmacy	Pharmacy	6, Ali Akilu Road, Makurdi	Makurdi	Benue
725	BN/0039	BN/0039/S/5	Baz Medical Laboratory	Laboratory	7, Zion Street, Makurdi	Makurdi	Benue
726	BN/0040	BN/0040/S/5	Compass Medical Lab.	Laboratory	27, Iyochia Ayu Street, Makurdi	Makurdi	Benue
727	BN/0041	BN/0041/S/7	Hospital of Immaculate Conception	Physiotherapy, Radiology, Orthopaedic Surgery, Gen. Medicine, Gen. Surgery, Laboratory	Makurdi	Makurdi	Benue
728	BN/0041	BN/0041/S/10	Hospital of Immaculate Conception	Physiotherapy, Radiology, Orthopaedic Surgery, Gen. Medicine, Gen. Surgery, Laboratory	Makurdi	Makurdi	Benue
729	BN/0041	BN/0041/S/2	Hospital of Immaculate Conception	Physiotherapy, Radiology, Orthopaedic Surgery, Gen. Medicine, Gen. Surgery, Laboratory	Makurdi	Makurdi	Benue
730	BN/0041	BN/0041/S/5	Hospital of Immaculate Conception	Physiotherapy, Radiology, Orthopaedic Surgery, Gen. Medicine, Gen. Surgery, Laboratory	Makurdi	Makurdi	Benue
731	BN/0041	BN/0041/S/11	Hospital of Immaculate Conception	Physiotherapy, Radiology, Orthopaedic Surgery, Gen. Medicine, Gen. Surgery, Laboratory	Makurdi	Makurdi	Benue
732	BN/0041	BN/0041/S/1	Hospital of Immaculate Conception	Physiotherapy, Radiology, Orthopaedic Surgery, Gen. Medicine, Gen. Surgery, Laboratory	Makurdi	Makurdi	Benue
733	BN/0042	BN/0042/S/7	El-Shaddai Specialist Hospital	Gen. Medicine, Radiology, O & G, Laboratory, Surgery	Plot 2319, Kanshio, Makurdi	Makurdi	Benue
734	BN/0042	BN/0042/S/1	El-Shaddai Specialist Hospital	Gen. Medicine, Radiology, O & G, Laboratory, Surgery	Plot 2319, Kanshio, Makurdi	Makurdi	Benue

735	BN/0042	BN/0042/S/5	El-Shaddai Specialist Hospital	Gen. Medicine, Radiology, O & G, Laboratory, Surgery	Plot 2319, Kanshio, Makurdi	Makurdi	Benue
736	BN/0042	BN/0042/S/3	El-Shaddai Specialist Hospital	Gen. Medicine, Radiology, O & G, Laboratory, Surgery	Plot 2319, Kanshio, Makurdi	Makurdi	Benue
737	BN/0044	BN/0044/S/1	Bishop Murrah Medical Centre	Gen. Medicine, Paediatrics, O & G, Surgery, Radialogy, Laboratory	Close to St. Theresa Church, High Level, Makurdi	Makurdi	Benue
738	BN/0044	BN/0044/S/5	Bishop Murrah Medical Centre	Gen. Medicine, Paediatrics, O & G, Surgery, Radialogy, Laboratory	Close to St. Theresa Church, High Level, Makurdi	Makurdi	Benue
739	BN/0044	BN/0044/S/3	Bishop Murrah Medical Centre	Gen. Medicine, Paediatrics, O & G, Surgery, Radialogy, Laboratory	Close to St. Theresa Church, High Level, Makurdi	Makurdi	Benue
740	BN/0044	BN/0044/S/7	Bishop Murrah Medical Centre	Gen. Medicine, Paediatrics, O & G, Surgery, Radialogy, Laboratory	Close to St. Theresa Church, High Level, Makurdi	Makurdi	Benue
741	BN/0045	BN/0045/S/4	Josac Pharm. Ltd.	Pharmacy	No 5, Railway By-Pass, Makurdi	Makurdi	Benue
742	BN/0047	BN/0047/S/4	Ad-tophin Pharmaceuticals Ltd.	Pharmacy	Gboko Road, High Level, Makurdi	Makurdi	Benue
743	BN/0052	BN/0052/S/3	St. Daniel's Hospital		Behind Elipi Motor Park, Otukpo	Otukpo	Benue
744	BN/0052	BN/0052/S/2	St. Daniel's Hospital		Behind Elipi Motor Park, Otukpo	Otukpo	Benue
745	BN/0055	BN/0055/S/2	General Hospital,	Laboratory, General Surgery, Gen. Medicine, Dentistry, Pharmacy	Otukpo	Otukpo	Benue
746	BN/0055	BN/0055/S/1	General Hospital,	Laboratory, General Surgery, Gen. Medicine, Dentistry, Pharmacy	Otukpo	Otukpo	Benue
747	BN/0056	BN/0056/S/4	Mosdan Pharmacy	Pharmacy	Federal Road, Otukpo	Otukpo	Benue
748	BN/0059	BN/0059/S/1	45 Nigerian Airforce Hospital		Air Force Base, Makurdi, Benue State	Makurdi	Benue
749	BN/0059	BN/0059/S/2	45 Nigerian Airforce Hospital		Air Force Base, Makurdi, Benue State	Makurdi	Benue
750	BN/0059	BN/0059/S/8	45 Nigerian Airforce Hospital		Air Force Base, Makurdi, Benue State	Makurdi	Benue
751	BN/0059	BN/0059/S/11	45 Nigerian Airforce Hospital		Air Force Base, Makurdi, Benue State	Makurdi	Benue
752	BN/0059	BN/0059/S/7	45 Nigerian Airforce Hospital		Air Force Base, Makurdi, Benue State	Makurdi	Benue
753	BN/0059	BN/0059/S/6	45 Nigerian Airforce Hospital		Air Force Base, Makurdi, Benue State	Makurdi	Benue
754	BN/0059	BN/0059/S/5	45 Nigerian Airforce Hospital		Air Force Base, Makurdi, Benue State	Makurdi	Benue
755	BN/0059	BN/0059/S/4	45 Nigerian Airforce Hospital		Air Force Base, Makurdi, Benue State	Makurdi	Benue
756	BN/0059	BN/0059/S/15	45 Nigerian Airforce Hospital		Air Force Base, Makurdi, Benue State	Makurdi	Benue
757	BN/0059	BN/0059/S/3	45 Nigerian Airforce Hospital		Air Force Base, Makurdi, Benue State	Makurdi	Benue
758	BN/0060	BN/0060/S/4	Federal Staff Clinic		Federal Secretariat, Makurdi, Benue State	Makurdi	Benue
759	BN/0064	BN/0064/S/4	Cottage Hospital/Comprehensive Health Centre		Benue State Police Command, Makurdi, Benue State	Makurdi	Benue
760	BN/0064	BN/0064/S/1	Cottage Hospital/Comprehensive Health Centre		Benue State Police Command, Makurdi, Benue State	Makurdi	Benue
761	BN/0066	BN/0066/S/5	Benue Women Clinic	Primary	35, Modern Market Rd Makurdi	Makurdi	Benue
762	BN/0066	BN/0066/S/3	Benue Women Clinic	Primary	35, Modern Market Rd Makurdi	Makurdi	Benue
763	BN/0070	BN/0070/S/4	Med-Pearle Pharmacy & stores	Pharmacy	54 Iyorchia Ayu Rd wurukum Makurdi	Makurdi	Benue
764	BN/0071	BN/0071/S/4	Nativity Pharmacy Ltd Diocese Of Makurdi	Pharmacy	1 Front Of Federal Medical centre, Makurdi	Makurdi	Benue
765	BN/0073	BN/0073/S	Tosem Specialist Giagnostic Laboratory	Laboratory	33 Old Oturkpo Rd High Level Makurdi	Makurdi	Benue

766	BN/0076	BN/0076/S/4	Federal Staff Clinic Makurdi	Pharmacy	Federal Secretariat Complex Makurdi	Makurdi	Benue
767	BN/0078	BN/0078/S	Immanuel Medical centre/Eye Specialist Clinic	Ophthalmology	39, Modern Market Rd Makurdi	Makurdi	Benue
768	BN/0080	BN/0080/S/4	University of Agriculture, Makurdi		Makurdi, Benue State	Makurdi	Benue
769	BN/0080	BN/0080/S/5	University of Agriculture, Makurdi		Makurdi, Benue State	Makurdi	Benue
770	BN/0082	BN/0082/S/9	OK Specialist Hospital	Psychiatry	Along Modem Market Road, Makurdi, Benue State	Makurdi	Benue
771	BN/0083	BN/0083/S/1	Jonado Pharmacy	Pharmacy	Km 7 Otukpo Rd Kanshio Near Seminary Makurdi	Makurdi	Benue
772	BN/0102	BN/0102/S/4	st. Daniel's	O&G,int. medicine,surgery, pharmacy, laboratory	5, ochelifu street, behind elipi motor park, oturkpo	Otukpo	Benue
773	BN/0102	BN/0102/S/14	st. Daniel's	O&G,int. medicine,surgery, pharmacy, laboratory	5, ochelifu street, behind elipi motor park, oturkpo	Otukpo	Benue
774	BN/0102	BN/0102/S/1	st. Daniel's	O&G,int. medicine,surgery, pharmacy, laboratory	5, ochelifu street, behind elipi motor park, oturkpo	Otukpo	Benue
775	BN/0102	BN/0102/S/7	st. Daniel's	O&G,int. medicine,surgery, pharmacy, laboratory	5, ochelifu street, behind elipi motor park, oturkpo	Otukpo	Benue
776	BN/0102	BN/0102/S/5	st. Daniel's	O&G,int. medicine,surgery, pharmacy, laboratory	5, ochelifu street, behind elipi motor park, oturkpo	Otukpo	Benue
777	BN/0102	BN/0102/S/2	st. Daniel's	O&G,int. medicine,surgery, pharmacy, laboratory	5, ochelifu street, behind elipi motor park, oturkpo	Otukpo	Benue
778	BN/0102	BN/0102/S/3	st. Daniel's	O&G,int. medicine,surgery, pharmacy, laboratory	5, ochelifu street, behind elipi motor park, oturkpo	Otukpo	Benue
779	BN/0103	BN/0103/S/4	HAVRON PHARMACY	Pharmacy	116,BENUE CRESCENT, WADATA, MAKURDI	Makurdi	Benue
780	BN/0106	BN/0106/S/4	BARNEY PHARMACY	PHARMACY	12 COLLEGE CRESCENT,MAKURDI	Makurdi	Benue
781	BN/0112	BN/0112/S/4	Ezim Pharmacy	Pharmacy	37 Jerecho Road, Oturkpo	Otukpo	Benue
782	BN/0122	BN/0122/S/2	Powaren Hospital		9, J.S. Tarka way, Gboko, Benue state	Gboko	Benue
783	BN/0122	BN/0122/S/10	Powaren Hospital		9, J.S. Tarka way, Gboko, Benue state	Gboko	Benue
784	BN/0122	BN/0122/S/3	Powaren Hospital		9, J.S. Tarka way, Gboko, Benue state	Gboko	Benue
785	BN/0124	BN/0124/S/15	Wurukum Specialist Clinic		19, Konshisha Street, High Level, Makurdi, Benue State.	Makurdi	Benue
786	BN/0127	BN/0127/S/4	Jaywhite Pharmacy & Stores Ltd		Suite A10/A11 Royal Shopping Mall, No 7 Kashim Ibrahim Road, Old GRA, Makurdi, Benue State.	Makurdi	Benue
787	BN/0129	BN/0129/S/10	Kings Park Polyclinic		Karanga Akanya Street, Off Abu King Shuluwa Road	Makurdi	Benue
788	BN/0129	BN/0129/S/1	Kings Park Polyclinic		Karanga Akanya Street, Off Abu King Shuluwa Road	Makurdi	Benue
789	BN/0132	BN/0132/S/5	Delight Diagnostic &Research Laboratory		17, Old Otukpo Road, Makurdi, Benue State.	Otukpo	Benue
790	BN/0133	BN/0133/S/1	Dora Specialist Hospital		Off High Court Road, North Bank, Makurdi, Benue State.	Makurdi	Benue
791	BN/0137	BN/0137/S/4	NKST Hospital		Mkar- Gboko , Benue State.	Gboko	Benue
792	BN/0137	BN/0137/S/5	NKST Hospital		Mkar- Gboko , Benue State.	Gboko	Benue
793	BN/0138	BN/0138/S/10	NKST Rehabilitation Hospital		Mkar- Gboko , Benue State.	Gboko	Benue
794	BN/0138	BN/0138/S/5	NKST Rehabilitation Hospital		Mkar- Gboko , Benue State.	Gboko	Benue
795	BN/0138	BN/0138/S/11	NKST Rehabilitation Hospital		Mkar- Gboko , Benue State.	Gboko	Benue
796	BN/0138	BN/0138/S/4	NKST Rehabilitation Hospital		Mkar- Gboko , Benue State.	Gboko	Benue
797	BN/0141	BN/0141/S/2	Adoose Specialist Hospital		No. 12 Keghem Malu Street New GRA Makurdi Benue State.	Makurdi	Benue

798	BN/0141	BN/0141/S/15	Adoose Specialist Hospital		No. 12 Keghem Malu Street New GRA Makurdi Benue State.	Makurdi	Benue
799	BN/0143	BN/0143/S/5	Hope Hospital.		No. 2 Akure Street Wadata, Benue State.	Makurdi	Benue
800	BN/0149	BN/0149/S/5	General Hospital, Oju.		Secondary Health Care Facility Oju, Benue State.	Oju	Benue
801	BN/0149	BN/0149/S/4	General Hospital, Oju.		Secondary Health Care Facility Oju, Benue State.	Oju	Benue
802	BN/0152	BN/0152/S/4	Lord Is Saviour Clinic And Maternity.		No. 34B Mkar Rd. Gboko, Benue State.	Gboko	Benue
803	BN/0152	BN/0152/S/5	Lord Is Saviour Clinic And Maternity.		No. 34B Mkar Rd. Gboko, Benue State.	Gboko	Benue
804	BN/0152	BN/0152/S/1	Lord Is Saviour Clinic And Maternity.		No. 34B Mkar Rd. Gboko, Benue State.	Gboko	Benue
805	BN/0154	BN/0154/S/5	St. Vincent Hospital.		Aliade, Benue State	Gwer East	Benue
806	BN/0154	BN/0154/S/4	St. Vincent Hospital.		Aliade, Benue State	Gwer East	Benue
807	BN/0155	BN/0155/S/1	First Fertility Hospital Ltd.		Plot BNB 5827, Behind Tile Ankpa Ward Benue State	Makurdi	Benue
808	BN/0155	BN/0155/S/5	First Fertility Hospital Ltd.		Plot BNB 5827, Behind Tile Ankpa Ward Benue State	Makurdi	Benue
809	BN/0155	BN/0155/S/4	First Fertility Hospital Ltd.		Plot BNB 5827, Behind Tile Ankpa Ward Benue State	Makurdi	Benue
810	BN/0155	BN/0155/S/3	First Fertility Hospital Ltd.		Plot BNB 5827, Behind Tile Ankpa Ward Benue State	Makurdi	Benue
811	BN/0157	BN/0157/S/4	N K S T Hospital, Mbaakon.		N K S T Hospital, Mbaakon	Vandeikya	Benue
812	BN/0157	BN/0157/S/5	N K S T Hospital, Mbaakon.		N K S T Hospital, Mbaakon	Vandeikya	Benue
813	BN/0158	BN/0158/S/1	Myom Hospital		No. 28, General Hospital Road Gbako, Benue State	Gboko	Benue
814	BN/0158	BN/0158/S/5	Myom Hospital		No. 28, General Hospital Road Gbako, Benue State	Gboko	Benue
815	BN/0158	BN/0158/S/4	Myom Hospital		No. 28, General Hospital Road Gbako, Benue State	Gboko	Benue
816	BN/0158	BN/0158/S/3	Myom Hospital		No. 28, General Hospital Road Gbako, Benue State	Gboko	Benue
817	BN/0161	BN/0161/S/4	General Hospital, Adikpo		Kwande Benue State	Kwande	Benue
818	BN/0161	BN/0161/S/5	General Hospital, Adikpo		Kwande Benue State	Kwande	Benue
819	BN/0163	BN/0163/S/10	Benue State University Teaching Hospital.		BSUTH Gboko rd. Behind Tile Ankpa Ward Makurdi, Benue State.	Makurdi	Benue
820	BN/0163	BN/0163/S/4	Benue State University Teaching Hospital.		BSUTH Gboko rd. Behind Tile Ankpa Ward Makurdi, Benue State.	Makurdi	Benue
821	BN/0163	BN/0163/S/5	Benue State University Teaching Hospital.		BSUTH Gboko rd. Behind Tile Ankpa Ward Makurdi, Benue State.	Makurdi	Benue
822	BN/0163	BN/0163/S/8	Benue State University Teaching Hospital.		BSUTH Gboko rd. Behind Tile Ankpa Ward Makurdi, Benue State.	Makurdi	Benue
823	BN/0163	BN/0163/S/12	Benue State University Teaching Hospital.		BSUTH Gboko rd. Behind Tile Ankpa Ward Makurdi, Benue State.	Makurdi	Benue
824	BN/0163	BN/0163/S/6	Benue State University Teaching Hospital.		BSUTH Gboko rd. Behind Tile Ankpa Ward Makurdi, Benue State.	Makurdi	Benue
825	BN/0163	BN/0163/S/15	Benue State University Teaching Hospital.		BSUTH Gboko rd. Behind Tile Ankpa Ward Makurdi, Benue State.	Makurdi	Benue
826	BN/0163	BN/0163/S/13	Benue State University Teaching Hospital.		BSUTH Gboko rd. Behind Tile Ankpa Ward Makurdi, Benue State.	Makurdi	Benue
827	BN/0163	BN/0163/S/11	Benue State University Teaching Hospital.		BSUTH Gboko rd. Behind Tile Ankpa Ward Makurdi, Benue State.	Makurdi	Benue
828	BN/0163	BN/0163/S/9	Benue State University Teaching Hospital.		BSUTH Gboko rd. Behind Tile Ankpa Ward Makurdi, Benue State.	Makurdi	Benue
829	BN/0163	BN/0163/S/14	Benue State University Teaching Hospital.		BSUTH Gboko rd. Behind Tile Ankpa Ward Makurdi, Benue State.	Makurdi	Benue
830	BN/0163	BN/0163/S/1	Benue State University Teaching Hospital.		BSUTH Gboko rd. Behind Tile Ankpa Ward Makurdi, Benue State.	Makurdi	Benue
831	BN/0163	BN/0163/S/2	Benue State University Teaching Hospital.		BSUTH Gboko rd. Behind Tile Ankpa Ward Makurdi, Benue State.	Makurdi	Benue
832	BN/0163	BN/0163/S/7	Benue State University Teaching Hospital.		BSUTH Gboko rd. Behind Tile Ankpa Ward Makurdi, Benue State.	Makurdi	Benue
833	BN/0163	BN/0163/S/3	Benue State University Teaching Hospital.		BSUTH Gboko rd. Behind Tile Ankpa Ward Makurdi, Benue State.	Makurdi	Benue

834	BN/0167	BN/0167/S/4	St. Vincent Hospital Aliade		Aliade-Otukpo Road Benue State	Gwer East	Benue
835	BN/0167	BN/0167/S/5	St. Vincent Hospital Aliade		Aliade-Otukpo Road Benue State	Gwer East	Benue
836	BN/0170	BN/0170/S/4	General Hospital North Bank		North Bank ,Markudi Benue State	Makurdi	Benue
837	BN/0172	BN/0172/S/8	Gilead Dental Services	Dentistry	No.6 Kwararafa Quaters Markudi Benue State	Makurdi	Benue
838	BN/0174	BN/0174/S/11	Glory Physiotherapy		No. 27 Inikpi Street, High Level Markudi Benue State	Makurdi	Benue
839	BN/0175	BN/0175/S/8	Cool Smile Dental Clinic		No.87,Ankpa Road Markudi Benue State	Makurdi	Benue
840	BN/0177	BN/0177/S/5	Family Support Clinic		Kwararafa Quaters Markudi Benue State	Makurdi	Benue
841	BO/0002	BO/0002/S/4	General Hospital, Bama		Bama Gulumba Road, Bama Town	Bama	Borno
842	BO/0002	BO/0002/S/5	General Hospital, Bama		Bama Gulumba Road, Bama Town	Bama	Borno
843	BO/0003	BO/0003/S/8	General Hospital, Biu		Along Yola Road, Biu	Biu	Borno
844	BO/0003	BO/0003/S/3	General Hospital, Biu		Along Yola Road, Biu	Biu	Borno
845	BO/0003	BO/0003/S/4	General Hospital, Biu		Along Yola Road, Biu	Biu	Borno
846	BO/0003	BO/0003/S/5	General Hospital, Biu		Along Yola Road, Biu	Biu	Borno
847	BO/0003	BO/0003/S/2	General Hospital, Biu		Along Yola Road, Biu	Biu	Borno
848	BO/0003	BO/0003/S/6	General Hospital, Biu		Along Yola Road, Biu	Biu	Borno
849	BO/0003	BO/0003/S/1	General Hospital, Biu		Along Yola Road, Biu	Biu	Borno
850	BO/0005	BO/0005/S/5	General Hospital, Gwoza		Gwoza Town, Maiduguri	Gwoza	Borno
851	BO/0005	BO/0005/S/4	General Hospital, Gwoza		Gwoza Town, Maiduguri	Gwoza	Borno
852	BO/0005	BO/0005/S/1	General Hospital, Gwoza		Gwoza Town, Maiduguri	Gwoza	Borno
853	BO/0005	BO/0005/S/2	General Hospital, Gwoza		Gwoza Town, Maiduguri	Gwoza	Borno
854	BO/0007	BO/0007/S/15	University Of Maiduguri Teaching Hospital		Along Bama Rd., Maiduguri	Maiduguri M. C.	Borno
855	BO/0007	BO/0007/S/6	University Of Maiduguri Teaching Hospital		Along Bama Rd., Maiduguri	Maiduguri M. C.	Borno
856	BO/0007	BO/0007/S/1	University Of Maiduguri Teaching Hospital		Along Bama Rd., Maiduguri	Maiduguri M. C.	Borno
857	BO/0007	BO/0007/S/3	University Of Maiduguri Teaching Hospital		Along Bama Rd., Maiduguri	Maiduguri M. C.	Borno
858	BO/0007	BO/0007/S/5	University Of Maiduguri Teaching Hospital		Along Bama Rd., Maiduguri	Maiduguri M. C.	Borno
859	BO/0007	BO/0007/S/2	University Of Maiduguri Teaching Hospital		Along Bama Rd., Maiduguri	Maiduguri M. C.	Borno
860	BO/0007	BO/0007/S/4	University Of Maiduguri Teaching Hospital		Along Bama Rd., Maiduguri	Maiduguri M. C.	Borno
861	BO/0007	BO/0007/S/10	University Of Maiduguri Teaching Hospital		Along Bama Rd., Maiduguri	Maiduguri M. C.	Borno
862	BO/0007	BO/0007/S/11	University Of Maiduguri Teaching Hospital		Along Bama Rd., Maiduguri	Maiduguri M. C.	Borno
863	BO/0007	BO/0007/S/7	University Of Maiduguri Teaching Hospital		Along Bama Rd., Maiduguri	Maiduguri M. C.	Borno
864	BO/0007	BO/0007/S/8	University Of Maiduguri Teaching Hospital		Along Bama Rd., Maiduguri	Maiduguri M. C.	Borno
865	BO/0009	BO/0009/S/6	General Hospital, Kukawa		Along Kukawa-Damasak Road, Kukawa	Kukawa	Borno
866	BO/0009	BO/0009/S/2	General Hospital, Kukawa		Along Kukawa-Damasak Road, Kukawa	Kukawa	Borno
867	BO/0012	BO/0012/S/3	Borno Medical Clinic		No.8, Sir Kashim Ibrahim Way	Maiduguri M. C.	Borno
868	BO/0012	BO/0012/S/5	Borno Medical Clinic		No.8, Sir Kashim Ibrahim Way	Maiduguri M. C.	Borno
869	BO/0012	BO/0012/S/4	Borno Medical Clinic		No.8, Sir Kashim Ibrahim Way	Maiduguri M. C.	Borno
870	BO/0012	BO/0012/S/1	Borno Medical Clinic		No.8, Sir Kashim Ibrahim Way	Maiduguri M. C.	Borno
871	BO/0012	BO/0012/S/2	Borno Medical Clinic		No.8, Sir Kashim Ibrahim Way	Maiduguri M. C.	Borno
872	BO/0012	BO/0012/S/7	Borno Medical Clinic		No.8, Sir Kashim Ibrahim Way	Maiduguri M. C.	Borno

873	BO/0014	BO/0014/S/2	State Specialist Hospital		Along Shehu Laminu Way	Maiduguri M. C.	Borno
874	BO/0014	BO/0014/S/12	State Specialist Hospital		Along Shehu Laminu Way	Maiduguri M. C.	Borno
875	BO/0014	BO/0014/S/1	State Specialist Hospital		Along Shehu Laminu Way	Maiduguri M. C.	Borno
876	BO/0014	BO/0014/S/4	State Specialist Hospital		Along Shehu Laminu Way	Maiduguri M. C.	Borno
877	BO/0014	BO/0014/S/6	State Specialist Hospital		Along Shehu Laminu Way	Maiduguri M. C.	Borno
878	BO/0014	BO/0014/S/3	State Specialist Hospital		Along Shehu Laminu Way	Maiduguri M. C.	Borno
879	BO/0014	BO/0014/S/11	State Specialist Hospital		Along Shehu Laminu Way	Maiduguri M. C.	Borno
880	BO/0014	BO/0014/S/5	State Specialist Hospital		Along Shehu Laminu Way	Maiduguri M. C.	Borno
881	BO/0015	BO/0015/S/4	Nursing Home, Maiduguri		Behind Nhis Zonal Office, Off Shehu Laminu Way, Maiduguri	Maiduguri M. C.	Borno
882	BO/0015	BO/0015/S/5	Nursing Home, Maiduguri		Behind Nhis Zonal Office, Off Shehu Laminu Way, Maiduguri	Maiduguri M. C.	Borno
883	BO/0016	BO/0016/S/8	Ayamsu Memorial Medical Centre		17 Bukar Bolori Street, Kumshe Ward Maiduguri	Maiduguri M. C.	Borno
884	BO/0016	BO/0016/S/5	Ayamsu Memorial Medical Centre		17 Bukar Bolori Street, Kumshe Ward Maiduguri	Maiduguri M. C.	Borno
885	BO/0017	BO/0017/S/7	Kanem Hospital & Maternity		152, Tafawa Balewa Street, Maiduguri	Maiduguri M. C.	Borno
886	BO/0017	BO/0017/S/1	Kanem Hospital & Maternity		152, Tafawa Balewa Street, Maiduguri	Maiduguri M. C.	Borno
887	BO/0017	BO/0017/S/2	Kanem Hospital & Maternity		152, Tafawa Balewa Street, Maiduguri	Maiduguri M. C.	Borno
888	BO/0017	BO/0017/S/6	Kanem Hospital & Maternity		152, Tafawa Balewa Street, Maiduguri	Maiduguri M. C.	Borno
889	BO/0017	BO/0017/S/4	Kanem Hospital & Maternity		152, Tafawa Balewa Street, Maiduguri	Maiduguri M. C.	Borno
890	BO/0017	BO/0017/S/3	Kanem Hospital & Maternity		152, Tafawa Balewa Street, Maiduguri	Maiduguri M. C.	Borno
891	BO/0017	BO/0017/S/5	Kanem Hospital & Maternity		152, Tafawa Balewa Street, Maiduguri	Maiduguri M. C.	Borno
892	BO/0018	BO/0018/S/3	Nakowa Specialist Hospital		Along Govt. House Road, Maiduguri	Maiduguri M. C.	Borno
893	BO/0018	BO/0018/S/7	Nakowa Specialist Hospital		Along Govt. House Road, Maiduguri	Maiduguri M. C.	Borno
894	BO/0018	BO/0018/S/5	Nakowa Specialist Hospital		Along Govt. House Road, Maiduguri	Maiduguri M. C.	Borno
895	BO/0019	BO/0019/S/3	Zaman Clinic & Annex		326, Waziri Kyari Drive, Gamboru Ward, Maiduguri	Maiduguri M. C.	Borno
896	BO/0019	BO/0019/S/1	Zaman Clinic & Annex		326, Waziri Kyari Drive, Gamboru Ward, Maiduguri	Maiduguri M. C.	Borno
897	BO/0019	BO/0019/S/2	Zaman Clinic & Annex		326, Waziri Kyari Drive, Gamboru Ward, Maiduguri	Maiduguri M. C.	Borno
898	BO/0019	BO/0019/S/5	Zaman Clinic & Annex		326, Waziri Kyari Drive, Gamboru Ward, Maiduguri	Maiduguri M. C.	Borno
899	BO/0028	BO/0028/S/4	General Hospital Monguno		Along Maiduguri-Baga Expressway, Maiduguri	Monguno	Borno
900	BO/0028	BO/0028/S/3	General Hospital Monguno		Along Maiduguri-Baga Expressway, Maiduguri	Monguno	Borno
901	BO/0028	BO/0028/S/1	General Hospital Monguno		Along Maiduguri-Baga Expressway, Maiduguri	Monguno	Borno
902	BO/0028	BO/0028/S/5	General Hospital Monguno		Along Maiduguri-Baga Expressway, Maiduguri	Monguno	Borno
903	BO/0028	BO/0028/S/2	General Hospital Monguno		Along Maiduguri-Baga Expressway, Maiduguri	Monguno	Borno
904	BO/0028	BO/0028/S/6	General Hospital Monguno		Along Maiduguri-Baga Expressway, Maiduguri	Monguno	Borno
905	BO/0030	BO/0030/S/5	Federal Neuropshychiatry Hospital		Off, Baga Road, Fed. Low Cost, Maiduguri	Maiduguri M. C.	Borno
906	BO/0030	BO/0030/S/4	Federal Neuropshychiatry Hospital		Off, Baga Road, Fed. Low Cost, Maiduguri	Maiduguri M. C.	Borno
907	BO/0030	BO/0030/S/9	Federal Neuropshychiatry Hospital		Off, Baga Road, Fed. Low Cost, Maiduguri	Maiduguri M. C.	Borno
908	BO/0030	BO/0030/S/7	Federal Neuropshychiatry Hospital		Off, Baga Road, Fed. Low Cost, Maiduguri	Maiduguri M. C.	Borno
909	BO/0030	BO/0030/S/14	Federal Neuropshychiatry Hospital		Off, Baga Road, Fed. Low Cost, Maiduguri	Maiduguri M. C.	Borno
910	BO/0051	BO/0051/S/7	City Medical Centre		Galadima Ward, Borno State	Maiduguri M. C.	Borno
911	BO/0051	BO/0051/S/6	City Medical Centre		Galadima Ward, Borno State	Maiduguri M. C.	Borno

912	BO/0051	BO/0051/S/10	City Medical Centre		Galadima Ward, Borno State	Maiduguri M. C.	Borno
913	BO/0051	BO/0051/S/1	City Medical Centre		Galadima Ward, Borno State	Maiduguri M. C.	Borno
914	BO/0051	BO/0051/S/5	City Medical Centre		Galadima Ward, Borno State	Maiduguri M. C.	Borno
915	BO/0051	BO/0051/S/3	City Medical Centre		Galadima Ward, Borno State	Maiduguri M. C.	Borno
916	BO/0062	BO/0062/S/3	Alkome Polyclinic		Baga Road, Maiduguri, Borno State	Maiduguri M. C.	Borno
917	BO/0063	BO/0063/S/3	Sunni Hospital		Abba Ganaram, Kashim Ibrahim Way, Maiduguri, Borno State	Maiduguri M. C.	Borno
918	BO/0063	BO/0063/S/4	Sunni Hospital		Abba Ganaram, Kashim Ibrahim Way, Maiduguri, Borno State	Maiduguri M. C.	Borno
919	BO/0063	BO/0063/S/5	Sunni Hospital		Abba Ganaram, Kashim Ibrahim Way, Maiduguri, Borno State	Maiduguri M. C.	Borno
920	BO/0065	BO/0065/S/1	Kwatam Medical Clinic		Baga Road, Maiduguri, Borno State	Maiduguri M. C.	Borno
921	BO/0065	BO/0065/S/6	Kwatam Medical Clinic		Baga Road, Maiduguri, Borno State	Maiduguri M. C.	Borno
922	BO/0065	BO/0065/S/5	Kwatam Medical Clinic		Baga Road, Maiduguri, Borno State	Maiduguri M. C.	Borno
923	BO/0065	BO/0065/S/3	Kwatam Medical Clinic		Baga Road, Maiduguri, Borno State	Maiduguri M. C.	Borno
924	BO/0065	BO/0065/S/2	Kwatam Medical Clinic		Baga Road, Maiduguri, Borno State	Maiduguri M. C.	Borno
925	BO/0066	BO/0066/S/5	Gospel Mission to Nigeria		G260 Galdimare, Biu, Gombe State	Biu	Borno
926	BO/0067	BO/0067/S/5	Hirku Clinic & Maternity		Damaturu Road, Biu, Borno State	Biu	Borno
927	BO/0092	BO/0092/S/1	Abbott Clinic & Maternity		Damaturu Road, Biu, Borno State	Biu	Borno
928	BO/0092	BO/0092/S/5	Abbott Clinic & Maternity		Damaturu Road, Biu, Borno State	Biu	Borno
929	BO/0092	BO/0092/S/7	Abbott Clinic & Maternity		Damaturu Road, Biu, Borno State	Biu	Borno
930	BO/0093	BO/0093/S/4	University of Maiduguri Staff Clinic		Bama Road, Maiduguri, Borno State	Maiduguri M. C.	Borno
931	BO/0093	BO/0093/S/5	University of Maiduguri Staff Clinic		Bama Road, Maiduguri, Borno State	Maiduguri M. C.	Borno
932	BO/0095	BO/0095/S/5	Nig Arabic Language Village Hospital		Ngalla Borno	Ngala	Borno
933	BO/0095	BO/0095/S/4	Nig Arabic Language Village Hospital		Ngalla Borno	Ngala	Borno
934	BO/0113	BO/0113/S/5	UNITYY CLINICS & MAT.		OLD GRA, NEAR GOVT HOUSE, MAIDUGURI	Maiduguri M. C.	Borno
935	BO/0140	BO/0140/S/6	Horama Hospital & Maternity		Off Lagos Street, Maiduguri	Maiduguri M. C.	Borno
936	BO/0140	BO/0140/S/14	Horama Hospital & Maternity		Off Lagos Street, Maiduguri	Maiduguri M. C.	Borno
937	BO/0140	BO/0140/S/3	Horama Hospital & Maternity		Off Lagos Street, Maiduguri	Maiduguri M. C.	Borno
938	BO/0142	BO/0142/S/1	Royal Specialist Hospital		Opp Gombole Road, Maiduguri	Jere	Borno
939	BO/0142	BO/0142/S/2	Royal Specialist Hospital		Opp Gombole Road, Maiduguri	Jere	Borno
940	BO/0142	BO/0142/S/3	Royal Specialist Hospital		Opp Gombole Road, Maiduguri	Jere	Borno
941	BO/0142	BO/0142/S/6	Royal Specialist Hospital		Opp Gombole Road, Maiduguri	Jere	Borno
942	BO/0144	BO/0144/S/3	Mustashfa Specialist Hospital		Damboa-Biu Road Behind Indimis House, Maiduguri	Maiduguri M. C.	Borno
943	BO/0144	BO/0144/S/7	Mustashfa Specialist Hospital		Damboa-Biu Road Behind Indimis House, Maiduguri	Maiduguri M. C.	Borno
944	BO/0144	BO/0144/S/2	Mustashfa Specialist Hospital		Damboa-Biu Road Behind Indimis House, Maiduguri	Maiduguri M. C.	Borno
945	BO/0144	BO/0144/S/1	Mustashfa Specialist Hospital		Damboa-Biu Road Behind Indimis House, Maiduguri	Maiduguri M. C.	Borno
946	BO/0145	BO/0145/S/8	Amand Dental Hospital		Damboa-Biu Road, Maiduguri	Maiduguri M. C.	Borno
947	BO/0146	BO/0146/S/2	Heman Hospital & Diagnostic Centre		New GRA, Opp, Bama rd motor park, Maiduguri	Maiduguri M. C.	Borno
948	BO/0146	BO/0146/S/6	Heman Hospital & Diagnostic Centre		New GRA, Opp, Bama rd motor park, Maiduguri	Maiduguri M. C.	Borno
949	BO/0146	BO/0146/S/5	Heman Hospital & Diagnostic Centre		New GRA, Opp, Bama rd motor park, Maiduguri	Maiduguri M. C.	Borno
950	BO/0146	BO/0146/S/1	Heman Hospital & Diagnostic Centre		New GRA, Opp, Bama rd motor park, Maiduguri	Maiduguri M. C.	Borno
951	BO/0146	BO/0146/S/3	Heman Hospital & Diagnostic Centre		New GRA, Opp, Bama rd motor park, Maiduguri	Maiduguri M. C.	Borno

952	BO/0147	BO/0147/S/5	New Ultra Metroplitan Clinic & Maternity		1, Chez Coan Junction, FNPH Rd, Maiduguri	Maiduguri M. C.	Borno
953	BO/0147	BO/0147/S/14	New Ultra Metroplitan Clinic & Maternity		1, Chez Coan Junction, FNPH Rd, Maiduguri	Maiduguri M. C.	Borno
954	BO/0149	BO/0149/S/11	7 Division Hospital		Maimalari Military Cantonment, Maiduguri.	Jere	Borno
955	BO/0149	BO/0149/S/8	7 Division Hospital		Maimalari Military Cantonment, Maiduguri.	Jere	Borno
956	BO/0149	BO/0149/S/5	7 Division Hospital		Maimalari Military Cantonment, Maiduguri.	Jere	Borno
957	BO/0149	BO/0149/S/1	7 Division Hospital		Maimalari Military Cantonment, Maiduguri.	Jere	Borno
958	BO/0149	BO/0149/S/10	7 Division Hospital		Maimalari Military Cantonment, Maiduguri.	Jere	Borno
959	BO/0149	BO/0149/S/14	7 Division Hospital		Maimalari Military Cantonment, Maiduguri.	Jere	Borno
960	BO/0149	BO/0149/S/2	7 Division Hospital		Maimalari Military Cantonment, Maiduguri.	Jere	Borno
961	BO/0149	BO/0149/S/4	7 Division Hospital		Maimalari Military Cantonment, Maiduguri.	Jere	Borno
962	BO/0149	BO/0149/S/7	7 Division Hospital		Maimalari Military Cantonment, Maiduguri.	Jere	Borno
963	BO/0150	BO/0150/S/5	Alheri Hospital		Bolori Market Road, Maiduguri	Maiduguri M. C.	Borno
964	BO/0151	BO/0151/S/6	Umar Shehu Ultramodern Hospital		Jos Road, Bulunkutu	Jere	Borno
965	BO/0151	BO/0151/S/2	Umar Shehu Ultramodern Hospital		Jos Road, Bulunkutu	Jere	Borno
966	BO/0151	BO/0151/S/4	Umar Shehu Ultramodern Hospital		Jos Road, Bulunkutu	Jere	Borno
967	BO/0151	BO/0151/S/5	Umar Shehu Ultramodern Hospital		Jos Road, Bulunkutu	Jere	Borno
968	BO/0151	BO/0151/S/3	Umar Shehu Ultramodern Hospital		Jos Road, Bulunkutu	Jere	Borno
969	BO/0151	BO/0151/S/14	Umar Shehu Ultramodern Hospital		Jos Road, Bulunkutu	Jere	Borno
970	BO/0151	BO/0151/S/1	Umar Shehu Ultramodern Hospital		Jos Road, Bulunkutu	Jere	Borno
971	BO/0152	BO/0152/S/3	Falala Specialist Medical Clinic		Mando Road, Polo GRA, Maiduguri	Maiduguri M. C.	Borno
972	BO/0152	BO/0152/S/1	Falala Specialist Medical Clinic		Mando Road, Polo GRA, Maiduguri	Maiduguri M. C.	Borno
973	BO/0154	BO/0154/S/11	Alpine Specialist Hospital		10 Tafawa Street, GRA, Maiduguri	Maiduguri M. C.	Borno
974	BO/0154	BO/0154/S/5	Alpine Specialist Hospital		10 Tafawa Street, GRA, Maiduguri	Maiduguri M. C.	Borno
975	BY/0002	BY/0002/S/1	Asueifa New Life Clinic		9, Asueifa hospital street, Bay bridge, Yenizue-Gene , Yenagoa	Yenagoa	Bayelsa
976	BY/0003	BY/0003/S/1	Kuro Specialist Hospital		16, Otiotio road, Yenizue-Gene, Yenagoa	Yenagoa	Bayelsa
977	BY/0004	BY/0004/S/3	Crest Consultant Hospital and Fertility CenterClinic		Yenagoa	Yenagoa	Bayelsa
978	BY/0004	BY/0004/S/5	Crest Consultant Hospital and Fertility CenterClinic		Yenagoa	Yenagoa	Bayelsa
979	BY/0006	BY/0006/S/3	Odi General Hospital		Odi	Ekeremor	Bayelsa
980	BY/0006	BY/0006/S/2	Odi General Hospital		Odi	Ekeremor	Bayelsa
981	BY/0007	BY/0007/S/3	St. Peter's Hospital		Amarata, Yenogoa	Yenagoa	Bayelsa
982	BY/0007	BY/0007/S/1	St. Peter's Hospital		Amarata, Yenogoa	Yenagoa	Bayelsa
983	BY/0007	BY/0007/S/5	St. Peter's Hospital		Amarata, Yenogoa	Yenagoa	Bayelsa
984	BY/0008	BY/0008/S/1	Everly Medical Centre		Yenegoa Opp. Boat Yard	Yenagoa	Bayelsa
985	BY/0008	BY/0008/S/3	Everly Medical Centre		Yenegoa Opp. Boat Yard	Yenagoa	Bayelsa
986	BY/0009	BY/0009/S/2	Palen Hospital		Ekeki Yenogoa	Yenagoa	Bayelsa
987	BY/0009	BY/0009/S/1	Palen Hospital		Ekeki Yenogoa	Yenagoa	Bayelsa
988	BY/0010	BY/0010/S/2	Mieye Medical Centre		630 Chief Melford Okilo Road, (Before Commisioner's Estate junction Opolo Yenagoa	Yenagoa	Bayelsa
989	BY/0010	BY/0010/S/1	Mieye Medical Centre		630 Chief Melford Okilo Road, (Before Commisioner's Estate junction Opolo Yenagoa	Yenagoa	Bayelsa

990	BY/0013	BY/0013/S/2	New Uchenna Hospital		Imgbi Road Amerata Yenagoa	Yenagoa	Bayelsa
991	BY/0013	BY/0013/S/5	New Uchenna Hospital		Imgbi Road Amerata Yenagoa	Yenagoa	Bayelsa
992	BY/0021	BY/0021/S/8	General Hospital	Obst. & Gynae, Internal Medicine, Paediatrics, General Surgery, Radiology, Dentistry	Okolobiri	Yenagoa	Bayelsa
993	BY/0021	BY/0021/S/1	General Hospital	Obst. & Gynae, Internal Medicine, Paediatrics, General Surgery, Radiology, Dentistry	Okolobiri	Yenagoa	Bayelsa
994	BY/0021	BY/0021/S/6	General Hospital	Obst. & Gynae, Internal Medicine, Paediatrics, General Surgery, Radiology, Dentistry	Okolobiri	Yenagoa	Bayelsa
995	BY/0021	BY/0021/S/2	General Hospital	Obst. & Gynae, Internal Medicine, Paediatrics, General Surgery, Radiology, Dentistry	Okolobiri	Yenagoa	Bayelsa
996	BY/0021	BY/0021/S	General Hospital	Obst. & Gynae, Internal Medicine, Paediatrics, General Surgery, Radiology, Dentistry	Okolobiri	Yenagoa	Bayelsa
997	BY/0021	BY/0021/S/7	General Hospital	Obst. & Gynae, Internal Medicine, Paediatrics, General Surgery, Radiology, Dentistry	Okolobiri	Yenagoa	Bayelsa
998	BY/0022	BY/0022/S/2	Asueifai New Life Clinic	General Medical Practice	Onopa, Yenagoa	Yenagoa	Bayelsa
999	BY/0024	BY/0024/S/4	Edyee Pharmacy Chemist Ltd.	Pharmacy	109, Mbiama/Yenagoa Road Ovom, Yenagoa	Yenagoa	Bayelsa
1000	BY/0026	BY/0026/S/4	Capstone Pharmacy & Stores Ltd.	Pharmacy	No.58 Jetty Road Yenagoa	Yenagoa	Bayelsa
1001	BY/0027	BY/0027/S/4	Mangrove Pharmacy	Pharmacy	No. 216 Mbiama Road	Yenagoa	Bayelsa
1002	BY/0036	BY/0036/S/2	Obis Clinic	Internal Medicine	Opp. Bycas, Agudama Epia, Yenogoa	Yenagoa	Bayelsa
1003	BY/0039	BY/0039/S/5	Glory Land Diagnostic Centre	Radiology, Laboratory	DSP Way, Yenogoa	Yenagoa	Bayelsa
1004	BY/0039	BY/0039/S/7	Glory Land Diagnostic Centre	Radiology, Laboratory	DSP Way, Yenogoa	Yenagoa	Bayelsa
1005	BY/0040	BY/0040/S	May Dental Hospital	Dentistry	Kpasia Yenogoa	Yenagoa	Bayelsa
1006	BY/0041	BY/0041/S/4	Cynflak Pharmacy Chemist	Pharmacy	196 Yenagoa, Mbiama Road Bayelsa	Yenagoa	Bayelsa
1007	BY/0042	BY/0042/S/4	Hilek Pharmacy	Pharmacy	3/4 Julius Berger Junction Yenogoa	Yenagoa	Bayelsa
1008	BY/0043	BY/0043/S/4	Efosa Pharmacy	Pharmacy	Ekeki, Yenogoa	Yenagoa	Bayelsa
1009	BY/0044	BY/0044/S/4	Jeffson Pharmacy	Pharmacy	Driefallin Road Yenogoa	Yenagoa	Bayelsa
1010	BY/0045	BY/0045/S/4	Okmoore Pharmacy	Pharmacy	Ekeki Police Station Yenogoa	Yenagoa	Bayelsa
1011	BY/0050	BY/0050/S	Geotech Med. Laboratory	Laboratory	Yenogoa,	Yenagoa	Bayelsa
1012	BY/0051	BY/0051/S/5	Meditech Lab.c/o Solomon Chief Lab.	Laboratory	Federal Medical Centre Yenogoa	Yenagoa	Bayelsa
1013	BY/0053	BY/0053/S/5	Creda Medical Laboratory	Laboratory	DSP Way, Ovanal, Yenogoa	Yenagoa	Bayelsa
1014	BY/0057	BY/0057/S/1	Federal Medical Centre, Yenagoa		Yenagoa, Bayelsa State	Yenagoa	Bayelsa
1015	BY/0057	BY/0057/S/3	Federal Medical Centre, Yenagoa		Yenagoa, Bayelsa State	Yenagoa	Bayelsa
1016	BY/0057	BY/0057/S/7	Federal Medical Centre, Yenagoa		Yenagoa, Bayelsa State	Yenagoa	Bayelsa
1017	BY/0057	BY/0057/S/6	Federal Medical Centre, Yenagoa		Yenagoa, Bayelsa State	Yenagoa	Bayelsa
1018	BY/0057	BY/0057/S/5	Federal Medical Centre, Yenagoa		Yenagoa, Bayelsa State	Yenagoa	Bayelsa

1019	BY/0057	BY/0057/S/13	Federal Medical Centre, Yenagoa		Yenagoa, Bayelsa State	Yenagoa	Bayelsa
1020	BY/0057	BY/0057/S/15	Federal Medical Centre, Yenagoa		Yenagoa, Bayelsa State	Yenagoa	Bayelsa
1021	BY/0057	BY/0057/S/11	Federal Medical Centre, Yenagoa		Yenagoa, Bayelsa State	Yenagoa	Bayelsa
1022	BY/0057	BY/0057/S/8	Federal Medical Centre, Yenagoa		Yenagoa, Bayelsa State	Yenagoa	Bayelsa
1023	BY/0057	BY/0057/S/4	Federal Medical Centre, Yenagoa		Yenagoa, Bayelsa State	Yenagoa	Bayelsa
1024	BY/0057	BY/0057/S/2	Federal Medical Centre, Yenagoa		Yenagoa, Bayelsa State	Yenagoa	Bayelsa
1025	BY/0061	BY/0061/S/7	Gloryland-inri med. Center		7 DSP Alameseigha Express way by Hospital Junction, Yenagoa	Yenagoa	Bayelsa
1026	BY/0061	BY/0061/S/10	Gloryland-inri med. Center		7 DSP Alameseigha Express way by Hospital Junction, Yenagoa	Yenagoa	Bayelsa
1027	BY/0061	BY/0061/S/5	Gloryland-inri med. Center		7 DSP Alameseigha Express way by Hospital Junction, Yenagoa	Yenagoa	Bayelsa
1028	BY/0064	BY/0064/S/4	Touch Good Pharmacy	Pharmacy	20, Imgbi Road, Amara, Yenagoa, Bayelsa State	Yenagoa	Bayelsa
1029	BY/0067	BY/0067/S/8	Yenogoa Hospital & Maternity		Yenogoa, Bayelsa	Yenagoa	Bayelsa
1030	BY/0067	BY/0067/S/4	Yenogoa Hospital & Maternity		Yenogoa, Bayelsa	Yenagoa	Bayelsa
1031	BY/0067	BY/0067/S/7	Yenogoa Hospital & Maternity		Yenogoa, Bayelsa	Yenagoa	Bayelsa
1032	BY/0067	BY/0067/S/3	Yenogoa Hospital & Maternity		Yenogoa, Bayelsa	Yenagoa	Bayelsa
1033	BY/0067	BY/0067/S/6	Yenogoa Hospital & Maternity		Yenogoa, Bayelsa	Yenagoa	Bayelsa
1034	BY/0067	BY/0067/S/2	Yenogoa Hospital & Maternity		Yenogoa, Bayelsa	Yenagoa	Bayelsa
1035	BY/0067	BY/0067/S/5	Yenogoa Hospital & Maternity		Yenogoa, Bayelsa	Yenagoa	Bayelsa
1036	BY/0067	BY/0067/S/1	Yenogoa Hospital & Maternity		Yenogoa, Bayelsa	Yenagoa	Bayelsa
1037	CR/0001	CR/0001/S/5	General Hospital,Mary Sclessor Avenue		Mary Sclessor Avenue, Calabar	Calabar Municipality	Cross River
1038	CR/0001	CR/0001/S/1	General Hospital,Mary Sclessor Avenue		Mary Sclessor Avenue, Calabar	Calabar Municipality	Cross River
1039	CR/0001	CR/0001/S/4	General Hospital,Mary Sclessor Avenue		Mary Sclessor Avenue, Calabar	Calabar Municipality	Cross River
1040	CR/0003	CR/0003/S/11	Hannah Foundation Clinic & Trauma Centre		13 Akim Close, Housing Estate, Calabar	Calabar Municipality	Cross River
1041	CR/0003	CR/0003/S/2	Hannah Foundation Clinic & Trauma Centre		13 Akim Close, Housing Estate, Calabar	Calabar Municipality	Cross River
1042	CR/0003	CR/0003/S/10	Hannah Foundation Clinic & Trauma Centre		13 Akim Close, Housing Estate, Calabar	Calabar Municipality	Cross River
1043	CR/0003	CR/0003/S/1	Hannah Foundation Clinic & Trauma Centre		13 Akim Close, Housing Estate, Calabar	Calabar Municipality	Cross River
1044	CR/0004	CR/0004/S/1	Faith Foundation Specialist Clinic		57 Ndidem Isang, Isong Road, Calabar	Calabar Municipality	Cross River
1045	CR/0004	CR/0004/S/6	Faith Foundation Specialist Clinic		57 Ndidem Isang, Isong Road, Calabar	Calabar Municipality	Cross River
1046	CR/0004	CR/0004/S/3	Faith Foundation Specialist Clinic		57 Ndidem Isang, Isong Road, Calabar	Calabar Municipality	Cross River
1047	CR/0007	CR/0007/S/2	Bakor Medical Centre		141, Ndidem Isand, Isong Road, Calabar	Calabar Municipality	Cross River
1048	CR/0007	CR/0007/S/1	Bakor Medical Centre		141, Ndidem Isand, Isong Road, Calabar	Calabar Municipality	Cross River
1049	CR/0007	CR/0007/S/5	Bakor Medical Centre		141, Ndidem Isand, Isong Road, Calabar	Calabar Municipality	Cross River
1050	CR/0007	CR/0007/S/6	Bakor Medical Centre		141, Ndidem Isand, Isong Road, Calabar	Calabar Municipality	Cross River
1051	CR/0007	CR/0007/S/3	Bakor Medical Centre		141, Ndidem Isand, Isong Road, Calabar	Calabar Municipality	Cross River
1052	CR/0008	CR/0008/S/2	Mevon Specialist Clinic	-	30 Akim Road, Calabar	Calabar Municipality	Cross River
1053	CR/0008	CR/0008/S/1	Mevon Specialist Clinic	-	30 Akim Road, Calabar	Calabar Municipality	Cross River
1054	CR/0008	CR/0008/S/6	Mevon Specialist Clinic	-	30 Akim Road, Calabar	Calabar Municipality	Cross River
1055	CR/0008	CR/0008/S/5	Mevon Specialist Clinic	-	30 Akim Road, Calabar	Calabar Municipality	Cross River
1056	CR/0008	CR/0008/S/3	Mevon Specialist Clinic	-	30 Akim Road, Calabar	Calabar Municipality	Cross River
1057	CR/0012	CR/0012/S/3	Canaan Medical Centre		20b Iso Oqua, Big Qua Town,Calabar	Calabar Municipality	Cross River
1058	CR/0020	CR/0020/S/4	Joel Manuel & Mayfair Pharmacy	Pharmacy	78 Ekpo Abasi Street, Calabar	Calabar South	Cross River

1059	CR/0021	CR/0021/S/4	Kamel Pharmacy Ltd.	Pharmacy	90b Goldie Street, Calabar	Calabar South	Cross River
1060	CR/0024	CR/0024/S/4	Fovic Medical Diag. & Analytical Centre	Laboraotory	87, Ekpo Abasi Street, Calabar South	Calabar South	Cross River
1061	CR/0027	CR/0027/S/4	Bez Pharmacy	Pharmacy	10 White House Street, Calabar	Calabar South	Cross River
1062	CR/0028	CR/0028/S/4	Andy Pharmacy	Pharmacy	28 Ndidem Usang Isong Road, Calabar	Calabar Municipality	Cross River
1063	CR/0032	CR/0032/S/5	Melrose Hospital		5 Melrose Avenue (Enim Okpa) F/C. Ikom	Ikom	Cross River
1064	CR/0034	CR/0034/S/4	Mekason Pharmacy Ltd.	Pharmacy	2 Ogoja Road, Ikom	Ikom	Cross River
1065	CR/0038	CR/0038/S/4	Zodiac Pharmacy	Pharmacy	17 Okim Osabor Street, Ikom	Ikom	Cross River
1066	CR/0039	CR/0039/S/4	Sacred Heart Catholic Hospital		Ranch Road, Obudu	Obudu	Cross River
1067	CR/0039	CR/0039/S/5	Sacred Heart Catholic Hospital		Ranch Road, Obudu	Obudu	Cross River
1068	CR/0041	CR/0041/S/5	Monaiya Catholic Hospital & Maternity		Igoli, Ogoja	Ogoja	Cross River
1069	CR/0041	CR/0041/S/4	Monaiya Catholic Hospital & Maternity		Igoli, Ogoja	Ogoja	Cross River
1070	CR/0041	CR/0041/S/7	Monaiya Catholic Hospital & Maternity		Igoli, Ogoja	Ogoja	Cross River
1071	CR/0041	CR/0041/S/11	Monaiya Catholic Hospital & Maternity		Igoli, Ogoja	Ogoja	Cross River
1072	CR/0042	CR/0042/S/4	General Hospital, Igoli		Abakpa Road, Igoli, Ogoja	Ogoja	Cross River
1073	CR/0042	CR/0042/S/1	General Hospital, Igoli		Abakpa Road, Igoli, Ogoja	Ogoja	Cross River
1074	CR/0042	CR/0042/S/5	General Hospital, Igoli		Abakpa Road, Igoli, Ogoja	Ogoja	Cross River
1075	CR/0051	CR/0051/S/1	County Specialist Hospital		18 Calabar Road, Ikom	Ikom	Cross River
1076	CR/0051	CR/0051/S/5	County Specialist Hospital		18 Calabar Road, Ikom	Ikom	Cross River
1077	CR/0051	CR/0051/S/3	County Specialist Hospital		18 Calabar Road, Ikom	Ikom	Cross River
1078	CR/0051	CR/0051/S/7	County Specialist Hospital		18 Calabar Road, Ikom	Ikom	Cross River
1079	CR/0052	CR/0052/S/4	Mount Zion Medical Centre		28b Yellow Duke Street, Calabar	Calabar Municipality	Cross River
1080	CR/0058	CR/0058/S/11	University of Calabar Teaching Hospital		Moore Road, Calabar, Cross River State.	Calabar Municipality	Cross River
1081	CR/0058	CR/0058/S/6	University of Calabar Teaching Hospital		Moore Road, Calabar, Cross River State.	Calabar Municipality	Cross River
1082	CR/0058	CR/0058/S/15	University of Calabar Teaching Hospital		Moore Road, Calabar, Cross River State.	Calabar Municipality	Cross River
1083	CR/0058	CR/0058/S/4	University of Calabar Teaching Hospital		Moore Road, Calabar, Cross River State.	Calabar Municipality	Cross River
1084	CR/0058	CR/0058/S/5	University of Calabar Teaching Hospital		Moore Road, Calabar, Cross River State.	Calabar Municipality	Cross River
1085	CR/0058	CR/0058/S/3	University of Calabar Teaching Hospital		Moore Road, Calabar, Cross River State.	Calabar Municipality	Cross River
1086	CR/0058	CR/0058/S/1	University of Calabar Teaching Hospital		Moore Road, Calabar, Cross River State.	Calabar Municipality	Cross River
1087	CR/0058	CR/0058/S/2	University of Calabar Teaching Hospital		Moore Road, Calabar, Cross River State.	Calabar Municipality	Cross River
1088	CR/0066	CR/0066/S/4	University of Calabar Health Centre		Calabar, Cross River State	Calabar Municipality	Cross River
1089	CR/0066	CR/0066/S/5	University of Calabar Health Centre		Calabar, Cross River State	Calabar Municipality	Cross River
1090	CR/0070	CR/0070/S/3	Nigerian Navy Medical Clinic		7, Club Road, Calabar, Cross River State	Calabar Municipality	Cross River
1091	CR/0070	CR/0070/S/5	Nigerian Navy Medical Clinic		7, Club Road, Calabar, Cross River State	Calabar Municipality	Cross River
1092	CR/0070	CR/0070/S/1	Nigerian Navy Medical Clinic		7, Club Road, Calabar, Cross River State	Calabar Municipality	Cross River
1093	CR/0070	CR/0070/S/4	Nigerian Navy Medical Clinic		7, Club Road, Calabar, Cross River State	Calabar Municipality	Cross River
1094	CR/0071	CR/0071/S/3	Immanuel Infirmary		No. 1 Assemblies of God Rd., Army Junction, Calabar	Calabar Municipality	Cross River
1095	CR/0072	CR/0072/S/5	Obudu Clinic Ltd		105-108 Ogoja Road, Obudu, Cross River State	Obudu	Cross River

1096	CR/0072	CR/0072/S/1	Obudu Clinic Ltd		105-108 Ogoja Road, Obudu, Cross River State	Obudu	Cross River
1097	CR/0073	CR/0073/S/1	Peoples Specialist Clinic		48 Akim Road, Calabar	Calabar Municipality	Cross River
1098	CR/0074	CR/0074/S/1	Bassey Enon Medical Centre		11B Yellow Duke Street, Calabar, C/River State	Calabar Municipality	Cross River
1099	CR/0075	CR/0075/S/1	Ikpeme Medical Centre		18/20 Ambo Street, Calabar, Cross River State	Calabar Municipality	Cross River
1100	CR/0076	CR/0076/S/3	Victoria Itam Hospital		8A Ediba Lane, Big Qua Town, Calabar, C/River State	Calabar Municipality	Cross River
1101	CR/0076	CR/0076/S/6	Victoria Itam Hospital		8A Ediba Lane, Big Qua Town, Calabar, C/River State	Calabar Municipality	Cross River
1102	CR/0084	CR/0084/S/5	Police Clinics, Cross River		Cross River State	Calabar Municipality	Cross River
1103	CR/0084	CR/0084/S/4	Police Clinics, Cross River		Cross River State	Calabar Municipality	Cross River
1104	CR/0084	CR/0084/S/1	Police Clinics, Cross River		Cross River State	Calabar Municipality	Cross River
1105	CR/0084	CR/0084/S/3	Police Clinics, Cross River		Cross River State	Calabar Municipality	Cross River
1106	CR/0086	CR/0086/S/3	Amazing Grace Specialist Clinic		45, Ikot Uduak Street, Off MCC Road, Calabar, C/River State	Calabar Municipality	Cross River
1107	CR/0086	CR/0086/S/6	Amazing Grace Specialist Clinic		45, Ikot Uduak Street, Off MCC Road, Calabar, C/River State	Calabar Municipality	Cross River
1108	CR/0087	CR/0087/S/2	Heritage Specialist Clinic		64, Ekpo Abasi Street, Calabar, Cross River State	Calabar South	Cross River
1109	CR/0088	CR/0088/S/1	Testimony Medical Resource		58, Atakpa Street, Calabar, Cross River State	Calabar South	Cross River
1110	CR/0091	CR/0091/S/5	St. Joseph Hospital		Ikot Ene Ekpabuyo, Cross River State	Akpabuyo	Cross River
1111	CR/0092	CR/0092/S/5	Comprehensive Health Centre (UCTH Annex)		Okoyong, Calabar, C/River State	Odukpani	Cross River
1112	CR/0093	CR/0093/S/5	General Hospital Akamkpa		Akamkpa, C/River State	Akamkpa	Cross River
1113	CR/0094	CR/0094/S/4	General Hospital Ugep		Obol Ubi Ujong Avenue, Ugep	Yakurr	Cross River
1114	CR/0094	CR/0094/S/5	General Hospital Ugep		Obol Ubi Ujong Avenue, Ugep	Yakurr	Cross River
1115	CR/0095	CR/0095/S/5	General Hospital Obubra		Obubra, C/River State	Obubra	Cross River
1116	CR/0096	CR/0096/S/5	Holy Family Catholic Hospital		Beside Holy Family Parish, Ikom, C/River State	Ikom	Cross River
1117	CR/0096	CR/0096/S/7	Holy Family Catholic Hospital		Beside Holy Family Parish, Ikom, C/River State	Ikom	Cross River
1118	CR/0096	CR/0096/S/4	Holy Family Catholic Hospital		Beside Holy Family Parish, Ikom, C/River State	Ikom	Cross River
1119	CR/0098	CR/0098/S/5	Eja Memorial Joint Hospital		Itigidi, Abi LGA, C/River State	Abi	Cross River
1120	CR/0099	CR/0099/S/6	Union Medical Centre, Calabar		104 Ndidem Island, Isong Road, Calabar, C/River State	Calabar Municipality	Cross River
1121	CR/0101	CR/0101/S/3	Ultimate Medical & Consultant		Plot 328 Unit B Effanga, Mkpa State Housing Estate, Calabar	Calabar Municipality	Cross River
1122	CR/0101	CR/0101/S/8	Ultimate Medical & Consultant		Plot 328 Unit B Effanga, Mkpa State Housing Estate, Calabar	Calabar Municipality	Cross River
1123	CR/0101	CR/0101/S/1	Ultimate Medical & Consultant		Plot 328 Unit B Effanga, Mkpa State Housing Estate, Calabar	Calabar Municipality	Cross River
1124	CR/0101	CR/0101/S/2	Ultimate Medical & Consultant		Plot 328 Unit B Effanga, Mkpa State Housing Estate, Calabar	Calabar Municipality	Cross River
1125	CR/0106	CR/0106/S/5	Federal Psychiatric Hospital Calabar	Laboratory, Psychiatrics, Pharmacy	Calabar, Cross River State	Calabar South	Cross River
1126	CR/0106	CR/0106/S/4	Federal Psychiatric Hospital Calabar	Laboratory, Psychiatrics, Pharmacy	Calabar, Cross River State	Calabar South	Cross River
1127	CR/0106	CR/0106/S/9	Federal Psychiatric Hospital Calabar	Laboratory, Psychiatrics, Pharmacy	Calabar, Cross River State	Calabar South	Cross River
1128	CR/0108	CR/0108/S/15	Cross River State Eye Care Programme	Ophthalmology	C/o College of Health Tech. Mary Slessor Avenue, Calabar	Calabar South	Cross River
1129	CR/0109	CR/0109/S/8	Govt. Dental/Maxillofacial Centre	Dental	Moore Road, Calabar	Calabar South	Cross River

1130	CR/0112	CR/0112/S/5	Dr. Lawrence Henshew Mem. Hospital (form IDH)	Laboratory, Pharmacy	New Edgerly Road, Calabar	Calabar South	Cross River
1131	CR/0112	CR/0112/S/4	Dr. Lawrence Henshew Mem. Hospital (form IDH)	Laboratory, Pharmacy	New Edgerly Road, Calabar	Calabar South	Cross River
1132	CR/0140	CR/0140/S/15	Zerah Eye Centre	Opthamology	101 Ndidem Isang Isong Calabar	Calabar Municipality	Cross River
1133	CR/0143	CR/0143/S/13	Mego Vision Clinic		82, Mayne Avenue, Calabar, Cross River State.	Calabar Municipality	Cross River
1134	CR/0145	CR/0145/S/1	Alice Effanga Okon Specialist Clinic		No.4 Ekenglwatt Street, Calabar South, Cross River State	Calabar South	Cross River
1135	CR/0149	CR/0149/S/11	Calabar Women and Children Hospital		164/165 Murtala Mohammed Way Calabar	Calabar Municipality	Cross River
1136	CR/0149	CR/0149/S/7	Calabar Women and Children Hospital		164/165 Murtala Mohammed Way Calabar	Calabar Municipality	Cross River
1137	CR/0149	CR/0149/S/6	Calabar Women and Children Hospital		164/165 Murtala Mohammed Way Calabar	Calabar Municipality	Cross River
1138	CR/0149	CR/0149/S/4	Calabar Women and Children Hospital		164/165 Murtala Mohammed Way Calabar	Calabar Municipality	Cross River
1139	CR/0149	CR/0149/S/5	Calabar Women and Children Hospital		164/165 Murtala Mohammed Way Calabar	Calabar Municipality	Cross River
1140	CR/0149	CR/0149/S/1	Calabar Women and Children Hospital		164/165 Murtala Mohammed Way Calabar	Calabar Municipality	Cross River
1141	CR/0149	CR/0149/S/3	Calabar Women and Children Hospital		164/165 Murtala Mohammed Way Calabar	Calabar Municipality	Cross River
1142	CR/0151	CR/0151/S/13	Benita Eye Clinic		10,Mayna Avenue, Calabar Cross River State	Calabar South	Cross River
1143	CR/0152	CR/0152/S/6	Luciama Memorial Specialist Hospital		No.10 Luciama Hospital Road, Off Water Intake Road-Ikot Effanga, Calabar Cross River State.	Calabar Municipality	Cross River
1144	CR/0152	CR/0152/S/15	Luciama Memorial Specialist Hospital		No.10 Luciama Hospital Road, Off Water Intake Road-Ikot Effanga, Calabar Cross River State.	Calabar Municipality	Cross River
1145	CR/0152	CR/0152/S/5	Luciama Memorial Specialist Hospital		No.10 Luciama Hospital Road, Off Water Intake Road-Ikot Effanga, Calabar Cross River State.	Calabar Municipality	Cross River
1146	CR/0152	CR/0152/S/3	Luciama Memorial Specialist Hospital		No.10 Luciama Hospital Road, Off Water Intake Road-Ikot Effanga, Calabar Cross River State.	Calabar Municipality	Cross River
1147	CR/0152	CR/0152/S/1	Luciama Memorial Specialist Hospital		No.10 Luciama Hospital Road, Off Water Intake Road-Ikot Effanga, Calabar Cross River State.	Calabar Municipality	Cross River
1148	CR/0154	CR/0154/S/4	Nigeria Customs Service Medical Centre		Nigeria Customs Service, Opp. Airport, Calabar Cross River State	Calabar Municipality	Cross River
1149	CR/0155	CR/0155/S/5	Bakor Hospital Ltd		No.7,Hospital Road, Nde 3,Ikom Cross River State	Ikom	Cross River
1150	CR/0155	CR/0155/S/6	Bakor Hospital Ltd		No.7,Hospital Road, Nde 3,Ikom Cross River State	Ikom	Cross River
1151	CR/0155	CR/0155/S/1	Bakor Hospital Ltd		No.7,Hospital Road, Nde 3,Ikom Cross River State	Ikom	Cross River
1152	CR/0155	CR/0155/S/3	Bakor Hospital Ltd		No.7,Hospital Road, Nde 3,Ikom Cross River State	Ikom	Cross River
1153	CR/0155	CR/0155/S/2	Bakor Hospital Ltd		No.7,Hospital Road, Nde 3,Ikom Cross River State	Ikom	Cross River
1154	CR/0155	CR/0155/S/7	Bakor Hospital Ltd		No.7,Hospital Road, Nde 3,Ikom Cross River State	Ikom	Cross River
1155	CR/0158	CR/0158/S/3	Mary Mother Of Mercy Specialist Clinic		14, Ukpong Archibong Street, Essien Town, Calabar Cross River State.	Calabar Municipality	Cross River
1156	DT/0002	DT/0002/S/1	Good Samaritan Hospital		3 Good Samaritan Rd, Cable Point-Asaba	Uvwie	Delta
1157	DT/0005	DT/0005/S/8	Blue Cross Specialist Dental Clinic	Dental	21, Nnebisi Road, Former NEPA Commercial office, Asaba	Uvwie	Delta
1158	DT/0010	DT/0010/S/5	Reference Diagnostic Medical Lab.	Laboratory	134, Nnebisi Road, Asaba	Oshimili - South	Delta
1159	DT/0012	DT/0012/S/1	Oghor Specialist Clinic & Hospital		Izobo Street, Effurum	Uvwie	Delta
1160	DT/0014	DT/0014/S/8	Caboc Dental Clinic		Plot 540, 12th Ddpa, Airport Road, Effurum	Uvwie	Delta
1161	DT/0019	DT/0019/S/4	Supamed Pharmacy	Pharmacy	94, Jakpa Road, Effurum	Uvwie	Delta
1162	DT/0020	DT/0020/S/4	Arhomed Pharm. Ltd.	Pharmacy	30A, 1st Urhusi Street Eku	Ethiope East	Delta
1163	DT/0026	DT/0026/S/4	Hosanna Pharm. Ltd.	Pharmacy	14 Market Raod Sapele	Sapele	Delta

1164	DT/0027	DT/0027/S/5	Kwofia Clinic And Maternity		3, Kwofia Close, Po Box 35, Ovwian, Delta St.	Udu	Delta
1165	DT/0029	DT/0029/S/1	Ufor Hospital		19, Uloho Avenue Off Isoko Rd Ughelli	Ughelli North	Delta
1166	DT/0029	DT/0029/S/7	Ufor Hospital		19, Uloho Avenue Off Isoko Rd Ughelli	Ughelli North	Delta
1167	DT/0029	DT/0029/S/5	Ufor Hospital		19, Uloho Avenue Off Isoko Rd Ughelli	Ughelli North	Delta
1168	DT/0033	DT/0033/S/5	Estate Specialist Hospital		47, Onofegbara Str., Polokor Market, Warri	Warri North	Delta
1169	DT/0033	DT/0033/S/1	Estate Specialist Hospital		47, Onofegbara Str., Polokor Market, Warri	Warri North	Delta
1170	DT/0033	DT/0033/S/4	Estate Specialist Hospital		47, Onofegbara Str., Polokor Market, Warri	Warri North	Delta
1171	DT/0039	DT/0039/S/2	Winrose Clinic Limited		Enerhen Road, Effurum	Warri North	Delta
1172	DT/0044	DT/0044/S/4	Pharmserve Nig. Ltd.	Pharmacy	129 Ajarnimogbo Road Warri	Warri South	Delta
1173	DT/0045	DT/0045/S/4	Greso Pharm. Ltd.	Pharmacy	27 NNPC Housing Complex Rd. Ekpan-Warri	Uvwie	Delta
1174	DT/0046	DT/0046/S/4	Rosa Mystica Pharmacy Ltd.	Pharmacy	110, Warri Sapele Road Warri	Warri South	Delta
1175	DT/0047	DT/0047/S/4	SF Dimentional Services Ltd. (Pharm)	Pharmacy	15 Robert Road Warri	Warri North	Delta
1176	DT/0048	DT/0048/S/4	Bowspring Industries Ltd.(Pharm)	Pharmacy	17, Ogunu Road Warri	Warri North	Delta
1177	DT/0049	DT/0049/S/4	Herltez-Nig Ltd. (Pharm)	Pharmacy	81, Okere Ugborikoko Road Warri	Warri North	Delta
1178	DT/0050	DT/0050/S/4	Medvic (Nig.) Ltd. (Pharm.)	Pharmacy	128 Warri/Sapele Road Opp. Old Leventis Warri	Warri South	Delta
1179	DT/0054	DT/0054/S/4	Osco Pharmacy	Pharmacy	No. 43, Isoko Road, Ughelli	Uvwie	Delta
1180	DT/0056	DT/0056/S/4	Excelsior Pharmacy Ltd.	Pharmacy	N0. 15 Emeblren Street, Off FSS Okumogba Layout Warri	Warri South	Delta
1181	DT/0057	DT/0057/S/4	Keesom Pharm. & stores	Pharmacy	No. 9 Odion Road. Warri	Warri South	Delta
1182	DT/0060	DT/0060/S/8	Federal Medical Centre Asaba		Asaba	Uvwie	Delta
1183	DT/0060	DT/0060/S/3	Federal Medical Centre Asaba		Asaba	Uvwie	Delta
1184	DT/0060	DT/0060/S/4	Federal Medical Centre Asaba		Asaba	Uvwie	Delta
1185	DT/0060	DT/0060/S/15	Federal Medical Centre Asaba		Asaba	Uvwie	Delta
1186	DT/0060	DT/0060/S/1	Federal Medical Centre Asaba		Asaba	Uvwie	Delta
1187	DT/0060	DT/0060/S/5	Federal Medical Centre Asaba		Asaba	Uvwie	Delta
1188	DT/0060	DT/0060/S/10	Federal Medical Centre Asaba		Asaba	Uvwie	Delta
1189	DT/0060	DT/0060/S/7	Federal Medical Centre Asaba		Asaba	Uvwie	Delta
1190	DT/0064	DT/0064/S/6	Central Hospital Warri		1 Mabiaku RD NPA, Warri, Delta State	Warri North	Delta
1191	DT/0064	DT/0064/S/1	Central Hospital Warri		1 Mabiaku RD NPA, Warri, Delta State	Warri North	Delta
1192	DT/0064	DT/0064/S/10	Central Hospital Warri		1 Mabiaku RD NPA, Warri, Delta State	Warri North	Delta
1193	DT/0064	DT/0064/S/15	Central Hospital Warri		1 Mabiaku RD NPA, Warri, Delta State	Warri North	Delta
1194	DT/0064	DT/0064/S/8	Central Hospital Warri		1 Mabiaku RD NPA, Warri, Delta State	Warri North	Delta
1195	DT/0064	DT/0064/S/2	Central Hospital Warri		1 Mabiaku RD NPA, Warri, Delta State	Warri North	Delta
1196	DT/0065	DT/0065/S/5	Capitol Hill Clinic		2 Omamofe Silo Str Warri	Warri North	Delta
1197	DT/0065	DT/0065/S/1	Capitol Hill Clinic		2 Omamofe Silo Str Warri	Warri North	Delta
1198	DT/0065	DT/0065/S/7	Capitol Hill Clinic		2 Omamofe Silo Str Warri	Warri North	Delta
1199	DT/0066	DT/0066/S/5	Agbawus Medical Centre		3, Ogbo Drive, Ekurede, Warri	Warri North	Delta
1200	DT/0066	DT/0066/S/3	Agbawus Medical Centre		3, Ogbo Drive, Ekurede, Warri	Warri North	Delta
1201	DT/0070	DT/0070/S/4	Veenell Hospital		Okumagba Avenue, Warri	Warri North	Delta
1202	DT/0070	DT/0070/S/7	Veenell Hospital		Okumagba Avenue, Warri	Warri North	Delta
1203	DT/0073	DT/0073/S/12	Mass Specialist Clinic	ENT	No. 13 Effurun/Sapele Road Enerhen Junction, Effurun	Uvwie	Delta
1204	DT/0075	DT/0075/S/4	Mr. Anglese Hilary	Pharmacy	C/o Spendour Pharm. 1, Ugborikoko Road Off Airport Rd	Uvwie	Delta
1205	DT/0077	DT/0077/S/4	Peter Ughwubruisi Chemists Ltd.	Pharmacy	43, Market Road Sapele	Sapele	Delta
1206	DT/0078	DT/0078/S/4	Amen Pharmacy	Pharmacy	8 adeola Road Ugbeyiyi Sapele	Sapele	Delta

1207	DT/0080	DT/0080/S/4	Dubindelu Pharm. Industries Nig. Ltd.	Pharmacy	62, Nnebisi Road Asaba	Uvwie	Delta
1208	DT/0084	DT/0084/S/1	Central Hospital Ughelli		Ughelli, Delta State	Ughelli North	Delta
1209	DT/0084	DT/0084/S/7	Central Hospital Ughelli		Ughelli, Delta State	Ughelli North	Delta
1210	DT/0084	DT/0084/S/10	Central Hospital Ughelli		Ughelli, Delta State	Ughelli North	Delta
1211	DT/0084	DT/0084/S/8	Central Hospital Ughelli		Ughelli, Delta State	Ughelli North	Delta
1212	DT/0085	DT/0085/S/4	Dieveta Pharmacy	Pharmacy	50, Iwereko Road, Ughelli, Delta State	Ughelli North	Delta
1213	DT/0087	DT/0087/S/4	Fedunique (Nig.) Ltd.	Pharmacy	7, NNPC Housing Complex Rd., Edjeba, (Opp. C.O.E.), Warri.	Warri South	Delta
1214	DT/0088	DT/0088/S/11	Humanity Hospital		No. 3 Enere Road, Warri, Delta State	Warri North	Delta
1215	DT/0088	DT/0088/S/5	Humanity Hospital		No. 3 Enere Road, Warri, Delta State	Warri North	Delta
1216	DT/0088	DT/0088/S/4	Humanity Hospital		No. 3 Enere Road, Warri, Delta State	Warri North	Delta
1217	DT/0088	DT/0088/S/2	Humanity Hospital		No. 3 Enere Road, Warri, Delta State	Warri North	Delta
1218	DT/0090	DT/0090/S/12	Westend Hospital		2, 25th Street, Airport Road, Warri, Delta State	Uvwie	Delta
1219	DT/0090	DT/0090/S/7	Westend Hospital		2, 25th Street, Airport Road, Warri, Delta State	Uvwie	Delta
1220	DT/0090	DT/0090/S/5	Westend Hospital		2, 25th Street, Airport Road, Warri, Delta State	Uvwie	Delta
1221	DT/0090	DT/0090/S/4	Westend Hospital		2, 25th Street, Airport Road, Warri, Delta State	Uvwie	Delta
1222	DT/0092	DT/0092/S/3	Ame Specialist Hospital		15, Okumagba Avenue, Warri, Delta State	Warri North	Delta
1223	DT/0094	DT/0094/S/11	Navy Medical Centre		Nigerian Naval Engineering College, Naval Base, Ogorode, Sapele Delta State	Sapele	Delta
1224	DT/0094	DT/0094/S/4	Navy Medical Centre		Nigerian Naval Engineering College, Naval Base, Ogorode, Sapele Delta State	Sapele	Delta
1225	DT/0094	DT/0094/S/8	Navy Medical Centre		Nigerian Naval Engineering College, Naval Base, Ogorode, Sapele Delta State	Sapele	Delta
1226	DT/0094	DT/0094/S/7	Navy Medical Centre		Nigerian Naval Engineering College, Naval Base, Ogorode, Sapele Delta State	Sapele	Delta
1227	DT/0094	DT/0094/S/3	Navy Medical Centre		Nigerian Naval Engineering College, Naval Base, Ogorode, Sapele Delta State	Sapele	Delta
1228	DT/0094	DT/0094/S/2	Navy Medical Centre		Nigerian Naval Engineering College, Naval Base, Ogorode, Sapele Delta State	Sapele	Delta
1229	DT/0094	DT/0094/S/1	Navy Medical Centre		Nigerian Naval Engineering College, Naval Base, Ogorode, Sapele Delta State	Sapele	Delta
1230	DT/0094	DT/0094/S/5	Navy Medical Centre		Nigerian Naval Engineering College, Naval Base, Ogorode, Sapele Delta State	Sapele	Delta
1231	DT/0096	DT/0096/S/5	Ebony Clinic & Maternity		109 Okumagba Avenue, By Ojabugbe Junction Warri, Delta State	Warri South	Delta
1232	DT/0098	DT/0098/S/5	Radio Road Clinic & Maternity		9 Radio Road, Off Lower Erejinwa Road, Warri, Delta State	Warri South	Delta
1233	DT/0099	DT/0099/S/5	Ogana Memorial Clinic & Maternity		33, Ginuwa Road by Ogboru Junction, Delta State	Warri South	Delta
1234	DT/0100	DT/0100/S/5	St. Georges Specialist Clinic & Maternity		4, Idoma Street (Opp. Palm Grove Motel Gate), Warri, Delta State	Warri South	Delta
1235	DT/0101	DT/0101/S/5	Vertimon Medical Centre		3 Merogun Srt, Warri, Delta State	Warri South	Delta
1236	DT/0101	DT/0101/S/6	Vertimon Medical Centre		3 Merogun Srt, Warri, Delta State	Warri South	Delta
1237	DT/0101	DT/0101/S/14	Vertimon Medical Centre		3 Merogun Srt, Warri, Delta State	Warri South	Delta
1238	DT/0101	DT/0101/S/1	Vertimon Medical Centre		3 Merogun Srt, Warri, Delta State	Warri South	Delta
1239	DT/0103	DT/0103/S/5	Lily Clinic/Hospital		6 Brisbe Street Off Deco Rd, Warri Delta	Warri North	Delta
1240	DT/0103	DT/0103/S/7	Lily Clinic/Hospital		6 Brisbe Street Off Deco Rd, Warri Delta	Warri North	Delta

1241	DT/0103	DT/0103/S/1	Lily Clinic/Hospital		6 Brisbe Street Off Deco Rd, Warri Delta	Warri North	Delta
1242	DT/0103	DT/0103/S/14	Lily Clinic/Hospital		6 Brisbe Street Off Deco Rd, Warri Delta	Warri North	Delta
1243	DT/0103	DT/0103/S/6	Lily Clinic/Hospital		6 Brisbe Street Off Deco Rd, Warri Delta	Warri North	Delta
1244	DT/0103	DT/0103/S/4	Lily Clinic/Hospital		6 Brisbe Street Off Deco Rd, Warri Delta	Warri North	Delta
1245	DT/0103	DT/0103/S/2	Lily Clinic/Hospital		6 Brisbe Street Off Deco Rd, Warri Delta	Warri North	Delta
1246	DT/0103	DT/0103/S/3	Lily Clinic/Hospital		6 Brisbe Street Off Deco Rd, Warri Delta	Warri North	Delta
1247	DT/0115	DT/0115/S/6	GN-Children's & Gen. Med. Cli		3, Enakeyarhe-Ukuwere Street, DDPA, Ugborikoko, Effuru	Uvwie	Delta
1248	DT/0116	DT/0116/S/14	Group Christian Hospital		Inside Mosheshe Estate, off Airport Road, Effurun	Uvwie	Delta
1249	DT/0116	DT/0116/S/3	Group Christian Hospital		Inside Mosheshe Estate, off Airport Road, Effurun	Uvwie	Delta
1250	DT/0116	DT/0116/S/5	Group Christian Hospital		Inside Mosheshe Estate, off Airport Road, Effurun	Uvwie	Delta
1251	DT/0116	DT/0116/S/4	Group Christian Hospital		Inside Mosheshe Estate, off Airport Road, Effurun	Uvwie	Delta
1252	DT/0116	DT/0116/S/1	Group Christian Hospital		Inside Mosheshe Estate, off Airport Road, Effurun	Uvwie	Delta
1253	DT/0120	DT/0120/S/1	Lonia Clinic & Maternity		143 Ekete-DSC Express Way, Ovwian, Warri	Udu	Delta
1254	DT/0120	DT/0120/S/6	Lonia Clinic & Maternity		143 Ekete-DSC Express Way, Ovwian, Warri	Udu	Delta
1255	DT/0120	DT/0120/S/8	Lonia Clinic & Maternity		143 Ekete-DSC Express Way, Ovwian, Warri	Udu	Delta
1256	DT/0120	DT/0120/S/7	Lonia Clinic & Maternity		143 Ekete-DSC Express Way, Ovwian, Warri	Udu	Delta
1257	DT/0120	DT/0120/S/10	Lonia Clinic & Maternity		143 Ekete-DSC Express Way, Ovwian, Warri	Udu	Delta
1258	DT/0120	DT/0120/S/15	Lonia Clinic & Maternity		143 Ekete-DSC Express Way, Ovwian, Warri	Udu	Delta
1259	DT/0120	DT/0120/S/2	Lonia Clinic & Maternity		143 Ekete-DSC Express Way, Ovwian, Warri	Udu	Delta
1260	DT/0120	DT/0120/S/3	Lonia Clinic & Maternity		143 Ekete-DSC Express Way, Ovwian, Warri	Udu	Delta
1261	DT/0120	DT/0120/S/5	Lonia Clinic & Maternity		143 Ekete-DSC Express Way, Ovwian, Warri	Udu	Delta
1262	DT/0125	DT/0125/S/2	Regal Clinic & Maternity		226, Jakpa Road, Effurun, Delta State	Uvwie	Delta
1263	DT/0125	DT/0125/S/3	Regal Clinic & Maternity		226, Jakpa Road, Effurun, Delta State	Uvwie	Delta
1264	DT/0125	DT/0125/S/14	Regal Clinic & Maternity		226, Jakpa Road, Effurun, Delta State	Uvwie	Delta
1265	DT/0125	DT/0125/S/5	Regal Clinic & Maternity		226, Jakpa Road, Effurun, Delta State	Uvwie	Delta
1266	DT/0125	DT/0125/S/1	Regal Clinic & Maternity		226, Jakpa Road, Effurun, Delta State	Uvwie	Delta
1267	DT/0129	DT/0129/S/5	St. Joseph Catholic Hospital		142, Nnebisi Road, Delta	Oshimili - South	Delta
1268	DT/0130	DT/0130/S/3	St. Louis Clinic		4, Okundolor Street, off Ogbunu Road, Warri	Warri South	Delta
1269	DT/0131	DT/0131/S/5	Obule Medical Centre		115, Okpe Road, Sapele	Sapele	Delta
1270	DT/0131	DT/0131/S/1	Obule Medical Centre		115, Okpe Road, Sapele	Sapele	Delta
1271	DT/0140	DT/0140/S/5	Medichem Laboratories	Laboratory	1, Ejemudario Street, Warri, Delta State	Warri South	Delta
1272	DT/0141	DT/0141/S/4	Holymarks Pharmacy	Pharmacy	3, Ajomo Avenue, Delta State	Uvwie	Delta
1273	DT/0149	DT/0149/S/5	B.U.Clinics & Hospital LTD		26 Kotie Street, Warri	Warri South	Delta
1274	DT/0149	DT/0149/S/3	B.U.Clinics & Hospital LTD		26 Kotie Street, Warri	Warri South	Delta
1275	DT/0149	DT/0149/S/6	B.U.Clinics & Hospital LTD		26 Kotie Street, Warri	Warri South	Delta
1276	DT/0149	DT/0149/S/2	B.U.Clinics & Hospital LTD		26 Kotie Street, Warri	Warri South	Delta
1277	DT/0149	DT/0149/S/10	B.U.Clinics & Hospital LTD		26 Kotie Street, Warri	Warri South	Delta
1278	DT/0151	DT/0151/S/1	St Rebecca's Specialist Hospital	Surgery	11 Federal College of Education Technical Road Asaba	Uvwie	Delta
1279	DT/0152	DT/0152/S/5	Assumption Clinic & Maternity		8 Idiaregbe Street, Okumagba Layout, PO Box 137, Warri	Warri South	Delta
1280	DT/0152	DT/0152/S/3	Assumption Clinic & Maternity		8 Idiaregbe Street, Okumagba Layout, PO Box 137, Warri	Warri South	Delta
1281	DT/0156	DT/0156/S/1	Syracuse Clinic	General Surgery	12, Willie Street off Deco Road, Warri	Warri North	Delta
1282	DT/0160	DT/0160/S/3	Goshen Medical Centre, Asaba	O&G, General Surgery	8 Agu Road, Umuagu, Asaba	Oshimili - South	Delta
1283	DT/0160	DT/0160/S/1	Goshen Medical Centre, Asaba	O&G, General Surgery	8 Agu Road, Umuagu, Asaba	Oshimili - South	Delta

1284	DT/0162	DT/0162/S/13	Hellyncom Eye Clinic		285 Warri/Sapele Road by First Marine Gate, Warri	Uvwie	Delta
1285	DT/0163	DT/0163/S/5	Shekinah Medical Centre Limited		Opp. Heros Of Faith Church, Aro Rd Off Ughelli-Patani RD, Ughelli, Delta state	Ughelli North	Delta
1286	DT/0163	DT/0163/S/3	Shekinah Medical Centre Limited		Opp. Heros Of Faith Church, Aro Rd Off Ughelli-Patani RD, Ughelli, Delta state	Ughelli North	Delta
1287	DT/0165	DT/0165/S/3	Island Clinic & Maternity		100,Okere Ugborkoko Road, Okumagba Layout ,Warri, Delta State	Uvwie	Delta
1288	DT/0166	DT/0166/S/15	Okparavero Memorial Hospital		No 159,Sapele rd, Okirighwre, Sapele, Delta State	Sapele	Delta
1289	DT/0166	DT/0166/S/3	Okparavero Memorial Hospital		No 159,Sapele rd, Okirighwre, Sapele, Delta State	Sapele	Delta
1290	DT/0166	DT/0166/S/10	Okparavero Memorial Hospital		No 159,Sapele rd, Okirighwre, Sapele, Delta State	Sapele	Delta
1291	DT/0166	DT/0166/S/6	Okparavero Memorial Hospital		No 159,Sapele rd, Okirighwre, Sapele, Delta State	Sapele	Delta
1292	DT/0166	DT/0166/S/5	Okparavero Memorial Hospital		No 159,Sapele rd, Okirighwre, Sapele, Delta State	Sapele	Delta
1293	DT/0167	DT/0167/S/1	First-Rank Hospital LTD		27,Ejewor Street, Behind Gateway To Heaven Ministry 24/26 Arubaye St.Okumagba Layout, Warri, Delta State	Warri South	Delta
1294	DT/0168	DT/0168/S/3	Shepherd Hospital And Fertility Center		100B Edjeba RD, Warri, Delta State	Warri South	Delta
1295	DT/0168	DT/0168/S/7	Shepherd Hospital And Fertility Center		100B Edjeba RD, Warri, Delta State	Warri South	Delta
1296	DT/0169	DT/0169/S/1	Rapha Specialist Children and General Hospital			Warri North	Delta
1297	DT/0169	DT/0169/S/6	Rapha Specialist Children and General Hospital			Warri North	Delta
1298	DT/0169	DT/0169/S/10	Rapha Specialist Children and General Hospital			Warri North	Delta
1299	DT/0170	DT/0170/S/5	St. Andrew`s Hospital		39,Robert Road, Warri Delta State	Warri South	Delta
1300	DT/0173	DT/0173/S/13	Aubaine Eye Care Inc.		24 Airport RD, White Idegbe Estate, Ugborikoko,Effurun,Warri, Delta State	Uvwie	Delta
1301	DT/0187	DT/0187/S/11	Humanity Hospital		3 Humanity Hospital Close Off Enerhen Road, Effurun Delta State	Ughelli North	Delta
1302	DT/0188	DT/0188/S/4	Concord-Bay Pharmacy		173,Effurun/Sapele Road, Warri, Delta State	Uvwie	Delta
1303	DT/0192	DT/0192/S/15	El-Shaddai Specialist Eye Hospital		6 Ebenuwa Street, Asaba. Delta State	Oshimili - South	Delta
1304	DT/0198	DT/0198/S/13	Isoks Vision Limited		N Stateo 3,Dennis Osadebe RD,Asaba Delta	Oshimili - North	Delta
1305	DT/0201	DT/0201/S/8	St. Luke Hospital Asaba		Opp Delta Broadcasting Office Asaba, Delta State	Oshimili - South	Delta
1306	DT/0201	DT/0201/S/1	St. Luke Hospital Asaba		Opp Delta Broadcasting Office Asaba, Delta State	Oshimili - South	Delta
1307	DT/0201	DT/0201/S/2	St. Luke Hospital Asaba		Opp Delta Broadcasting Office Asaba, Delta State	Oshimili - South	Delta
1308	DT/0201	DT/0201/S/5	St. Luke Hospital Asaba		Opp Delta Broadcasting Office Asaba, Delta State	Oshimili - South	Delta
1309	DT/0201	DT/0201/S/7	St. Luke Hospital Asaba		Opp Delta Broadcasting Office Asaba, Delta State	Oshimili - South	Delta
1310	DT/0201	DT/0201/S/4	St. Luke Hospital Asaba		Opp Delta Broadcasting Office Asaba, Delta State	Oshimili - South	Delta
1311	DT/0201	DT/0201/S/3	St. Luke Hospital Asaba		Opp Delta Broadcasting Office Asaba, Delta State	Oshimili - South	Delta
1312	DT/0203	DT/0203/S/2	Lana Hospital		48, Atufe Road, Off Oke Road, Sapele Delta State.	Sapele	Delta
1313	DT/0204	DT/0204/S/13	Simply Vision Express Ltd		7. Anwai Road, Asaba, Delta State.	Aniocha - South	Delta
1314	DT/0206	DT/0206/S/8	AUV5 Dental Clinic		2, Mmemli Onya Street, Off Jarret Street, Asaba, Delta State	Aniocha North	Delta
1315	DT/0207	DT/0207/S/3	Shabach Hospitals Ltd		200 PTI Road, By Sunny Eru Motors, Effurun, Delta State	Warri North	Delta
1316	DT/0207	DT/0207/S/1	Shabach Hospitals Ltd		200 PTI Road, By Sunny Eru Motors, Effurun, Delta State	Warri North	Delta

1317	DT/0208	DT/0208/S/11	Twin City Hospital & Diagnostic Centre		99, Osadebey Way, Asaba Delta State	Aniocha - South	Delta
1318	DT/0208	DT/0208/S/5	Twin City Hospital & Diagnostic Centre		99, Osadebey Way, Asaba Delta State	Aniocha - South	Delta
1319	DT/0208	DT/0208/S/1	Twin City Hospital & Diagnostic Centre		99, Osadebey Way, Asaba Delta State	Aniocha - South	Delta
1320	DT/0208	DT/0208/S/2	Twin City Hospital & Diagnostic Centre		99, Osadebey Way, Asaba Delta State	Aniocha - South	Delta
1321	DT/0210	DT/0210/S/3	Kenet Group Medical Services		14, Nwaezeapu Street, Off Nnebisi Road, Asaba Delta State	Aniocha - South	Delta
1322	DT/0210	DT/0210/S/1	Kenet Group Medical Services		14, Nwaezeapu Street, Off Nnebisi Road, Asaba Delta State	Aniocha - South	Delta
1323	DT/0210	DT/0210/S/6	Kenet Group Medical Services		14, Nwaezeapu Street, Off Nnebisi Road, Asaba Delta State	Aniocha - South	Delta
1324	EB/0004	EB/0004/S/1	Christ The King Hospital		85, Afikpo Road, Abakaliki	Abakaliki	Ebonyi
1325	EB/0007	EB/0007/S/6	Federal Teaching Hospital, Abakaliki		Opposite Police Barracks	Abakaliki	Ebonyi
1326	EB/0007	EB/0007/S/1	Federal Teaching Hospital, Abakaliki		Opposite Police Barracks	Abakaliki	Ebonyi
1327	EB/0007	EB/0007/S/3	Federal Teaching Hospital, Abakaliki		Opposite Police Barracks	Abakaliki	Ebonyi
1328	EB/0007	EB/0007/S/2	Federal Teaching Hospital, Abakaliki		Opposite Police Barracks	Abakaliki	Ebonyi
1329	EB/0007	EB/0007/S/5	Federal Teaching Hospital, Abakaliki		Opposite Police Barracks	Abakaliki	Ebonyi
1330	EB/0007	EB/0007/S/7	Federal Teaching Hospital, Abakaliki		Opposite Police Barracks	Abakaliki	Ebonyi
1331	EB/0007	EB/0007/S/4	Federal Teaching Hospital, Abakaliki		Opposite Police Barracks	Abakaliki	Ebonyi
1332	EB/0017	EB/0017/S/15	Niger Optical Center	Ophthalmology	Abakaliki	Abakaliki	Ebonyi
1333	EB/0020	EB/0020/S/4	Ekoh Drugs Co. Ltd.	Pharmacy	4B Ogoja Rd Abakaliki	Abakaliki	Ebonyi
1334	EB/0021	EB/0021/S/4	Ceno Pharmacy	Pharmacy	5 Zik Avenue Abakaliki	Abakaliki	Ebonyi
1335	EB/0022	EB/0022/S/4	Sun Pharmacy	Pharmacy	23 Afikpo St. Abakaliki	Abakaliki	Ebonyi
1336	EB/0023	EB/0023/S/4	Godal Pharmacy	Pharmacy	Off Water Works Rd, Abakaliki	Aba North	Abia
1337	EB/0024	EB/0024/S/5	Asia Specialist Laboratory	Laboratory	4C Ogoja Rd,. Abakaliki	Abakaliki	Ebonyi
1338	EB/0025	EB/0025/S/5	Eston Medical Laboratory	Laboratory	12 Gunny Rd., Abakaliki	Aba North	Abia
1339	EB/0029	EB/0029/S/4	New Good Health Pharmacy	Pharmacy	Eke Market Rd. Afikpo	Afikpo North	Ebonyi
1340	EB/0031	EB/0031/S/2	General Hospital	Gen. med., Gen. surgery, Paediatrics, Pharmacy, Laboratory	Ezzamgbo	Ohaukwu	Ebonyi
1341	EB/0031	EB/0031/S/6	General Hospital	Gen. med., Gen. surgery, Paediatrics, Pharmacy, Laboratory	Ezzamgbo	Ohaukwu	Ebonyi
1342	EB/0031	EB/0031/S/4	General Hospital	Gen. med., Gen. surgery, Paediatrics, Pharmacy, Laboratory	Ezzamgbo	Ohaukwu	Ebonyi
1343	EB/0031	EB/0031/S/5	General Hospital	Gen. med., Gen. surgery, Paediatrics, Pharmacy, Laboratory	Ezzamgbo	Ohaukwu	Ebonyi
1344	EB/0031	EB/0031/S/1	General Hospital	Gen. med., Gen. surgery, Paediatrics, Pharmacy, Laboratory	Ezzamgbo	Ohaukwu	Ebonyi
1345	EB/0041	EB/0041/S/4	Bishan Pharmacy	Pharmacy	1A Water Works Road, Abakaliki, Ebonyi State	Abakaliki	Ebonyi
1346	EB/0043	EB/0043/S/5	Medical Centre Akanu Ibiam Federal Polytechnic, Unwana		Ibeyi, Ebonyi State	Afikpo South	Ebonyi
1347	EB/0043	EB/0043/S/4	Medical Centre Akanu Ibiam Federal Polytechnic, Unwana		Ibeyi, Ebonyi State	Afikpo South	Ebonyi
1348	EB/0047	EB/0047/S/4	Alcord Pharmacy	Pharmacy	29, Akinkenwa, Abakaliki	Abakaliki	Ebonyi
1349	EB/0049	EB/0049/S/4	GABIO PHARMACY	Pharmacy	9, OBIRI, ABAKALIKI	Abakaliki	Ebonyi
1350	EB/0050	EB/0050/S/4	Elka Pharmacy Co Nig Ltd	Pharmacy	19 Brakembry/ Lagos Street, Abakaliki	Abakaliki	Ebonyi
1351	EB/0056	EB/0056/S/1	Mater Misericodiae Hospital		Afikpo North LGA	Afikpo North	Ebonyi
1352	EB/0056	EB/0056/S/2	Mater Misericodiae Hospital		Afikpo North LGA	Afikpo North	Ebonyi
1353	EB/0056	EB/0056/S/3	Mater Misericodiae Hospital		Afikpo North LGA	Afikpo North	Ebonyi

1354	EB/0056	EB/0056/S/6	Mater Misericodiae Hospital		Afikpo North LGA	Afikpo North	Ebonyi
1355	EB/0056	EB/0056/S/4	Mater Misericodiae Hospital		Afikpo North LGA	Afikpo North	Ebonyi
1356	EB/0056	EB/0056/S/5	Mater Misericodiae Hospital		Afikpo North LGA	Afikpo North	Ebonyi
1357	EB/0056	EB/0056/S/10	Mater Misericodiae Hospital		Afikpo North LGA	Afikpo North	Ebonyi
1358	EB/0057	EB/0057/S/4	New Good Health Pharmacy		1, Amachi Road, Afikpo, Ebonyi State	Afikpo South	Ebonyi
1359	EB/0059	EB/0059/S/7	Mater Misericordia		Afikpo Ebonyi State	Afikpo North	Ebonyi
1360	EB/0060	EB/0060/S/1	El- Shaddai Specialist Hospital		44 Ogoja Rd. Abakaliki, Ebonyi State.	Abakaliki	Ebonyi
1361	EB/0063	EB/0063/S/4	Midtown Pharmacy		13 Zik Avenue Abakaliki	Abakaliki	Ebonyi
1362	ED/0001	ED/0001/S/4	General Hospital, Afuze		Afuze	Esan West	Edo
1363	ED/0001	ED/0001/S/5	General Hospital, Afuze		Afuze	Esan West	Edo
1364	ED/0002	ED/0002/S/2	General Hospital, Igarra		Igarra, Akoko-Edo	Akoko Edo	Edo
1365	ED/0002	ED/0002/S/4	General Hospital, Igarra		Igarra, Akoko-Edo	Akoko Edo	Edo
1366	ED/0002	ED/0002/S/1	General Hospital, Igarra		Igarra, Akoko-Edo	Akoko Edo	Edo
1367	ED/0002	ED/0002/S/5	General Hospital, Igarra		Igarra, Akoko-Edo	Akoko Edo	Edo
1368	ED/0002	ED/0002/S/6	General Hospital, Igarra		Igarra, Akoko-Edo	Akoko Edo	Edo
1369	ED/0002	ED/0002/S/3	General Hospital, Igarra		Igarra, Akoko-Edo	Akoko Edo	Edo
1370	ED/0003	ED/0003/S/5	Government Hospital		Ibillo	Akoko Edo	Edo
1371	ED/0003	ED/0003/S/4	Government Hospital		Ibillo	Akoko Edo	Edo
1372	ED/0004	ED/0004/S/2	District Hospital, Akoko-Edo		Uneme-Osu	Akoko Edo	Edo
1373	ED/0004	ED/0004/S/1	District Hospital, Akoko-Edo		Uneme-Osu	Akoko Edo	Edo
1374	ED/0009	ED/0009/S/9	Psychiatric Hospital		Uselu	Egor	Edo
1375	ED/0009	ED/0009/S/2	Psychiatric Hospital		Uselu	Egor	Edo
1376	ED/0009	ED/0009/S/1	Psychiatric Hospital		Uselu	Egor	Edo
1377	ED/0011	ED/0011/S/1	Mount Gilead Hospital		Off Uselu, Lagos Road, Benin City	Egor	Edo
1378	ED/0011	ED/0011/S/6	Mount Gilead Hospital		Off Uselu, Lagos Road, Benin City	Egor	Edo
1379	ED/0011	ED/0011/S/5	Mount Gilead Hospital		Off Uselu, Lagos Road, Benin City	Egor	Edo
1380	ED/0011	ED/0011/S/2	Mount Gilead Hospital		Off Uselu, Lagos Road, Benin City	Egor	Edo
1381	ED/0011	ED/0011/S/11	Mount Gilead Hospital		Off Uselu, Lagos Road, Benin City	Egor	Edo
1382	ED/0011	ED/0011/S/4	Mount Gilead Hospital		Off Uselu, Lagos Road, Benin City	Egor	Edo
1383	ED/0011	ED/0011/S/3	Mount Gilead Hospital		Off Uselu, Lagos Road, Benin City	Egor	Edo
1384	ED/0013	ED/0013/S	St. Mary Hospital	Pharmacy, Laboratory	62 Textile Mill Road	Egor	Edo
1385	ED/0013	ED/0013/S/5	St. Mary Hospital	Pharmacy, Laboratory	62 Textile Mill Road	Egor	Edo
1386	ED/0015	ED/0015/S/2	District Hospital, Irrua		Usugbenu-Irrua	Esan Central	Edo
1387	ED/0016	ED/0016/S/11	Irrua Specialist Hospital		Irrua	Esan Central	Edo
1388	ED/0016	ED/0016/S/6	Irrua Specialist Hospital		Irrua	Esan Central	Edo
1389	ED/0016	ED/0016/S/8	Irrua Specialist Hospital		Irrua	Esan Central	Edo
1390	ED/0016	ED/0016/S/2	Irrua Specialist Hospital		Irrua	Esan Central	Edo
1391	ED/0016	ED/0016/S/3	Irrua Specialist Hospital		Irrua	Esan Central	Edo
1392	ED/0016	ED/0016/S/1	Irrua Specialist Hospital		Irrua	Esan Central	Edo
1393	ED/0016	ED/0016/S/10	Irrua Specialist Hospital		Irrua	Esan Central	Edo
1394	ED/0021	ED/0021/S/6	Central Hospital, Uromi		Uromi	Esan North East	Edo
1395	ED/0021	ED/0021/S/7	Central Hospital, Uromi		Uromi	Esan North East	Edo
1396	ED/0021	ED/0021/S/1	Central Hospital, Uromi		Uromi	Esan North East	Edo
1397	ED/0021	ED/0021/S/3	Central Hospital, Uromi		Uromi	Esan North East	Edo
1398	ED/0021	ED/0021/S/8	Central Hospital, Uromi		Uromi	Esan North East	Edo
1399	ED/0021	ED/0021/S/2	Central Hospital, Uromi		Uromi	Esan North East	Edo
1400	ED/0024	ED/0024/S/2	General Hospital, Ubiaja		Ubiaja	Esan South East	Edo

1401	ED/0025	ED/0025/S/1	District Hospital, Ewohinmi		Ewohimi	Esan South East	Edo
1402	ED/0025	ED/0025/S/2	District Hospital, Ewohinmi		Ewohimi	Esan South East	Edo
1403	ED/0026	ED/0026/S/2	General Hospital, Iruekpen		Iruekpen, Along Benin Auchi Road	Esan West	Edo
1404	ED/0026	ED/0026/S/1	General Hospital, Iruekpen		Iruekpen, Along Benin Auchi Road	Esan West	Edo
1405	ED/0026	ED/0026/S/6	General Hospital, Iruekpen		Iruekpen, Along Benin Auchi Road	Esan West	Edo
1406	ED/0027	ED/0027/S/2	General Hospital, Ekpoma		Ekpoma	Esan West	Edo
1407	ED/0027	ED/0027/S/1	General Hospital, Ekpoma		Ekpoma	Esan West	Edo
1408	ED/0033	ED/0033/S/3	General Hospital, Agenebode		Agenebode	Etsako East	Edo
1409	ED/0033	ED/0033/S/10	General Hospital, Agenebode		Agenebode	Etsako East	Edo
1410	ED/0033	ED/0033/S/6	General Hospital, Agenebode		Agenebode	Etsako East	Edo
1411	ED/0033	ED/0033/S/4	General Hospital, Agenebode		Agenebode	Etsako East	Edo
1412	ED/0033	ED/0033/S/2	General Hospital, Agenebode		Agenebode	Etsako East	Edo
1413	ED/0033	ED/0033/S/1	General Hospital, Agenebode		Agenebode	Etsako East	Edo
1414	ED/0033	ED/0033/S/5	General Hospital, Agenebode		Agenebode	Etsako East	Edo
1415	ED/0035	ED/0035/S/2	General Hospital, Agbede		Agbede	Etsako West	Edo
1416	ED/0035	ED/0035/S/6	General Hospital, Agbede		Agbede	Etsako West	Edo
1417	ED/0035	ED/0035/S/5	General Hospital, Agbede		Agbede	Etsako West	Edo
1418	ED/0035	ED/0035/S/1	General Hospital, Agbede		Agbede	Etsako West	Edo
1419	ED/0035	ED/0035/S/4	General Hospital, Agbede		Agbede	Etsako West	Edo
1420	ED/0036	ED/0036/S/11	Central Hospital, Auchi		Auchi	Etsako West	Edo
1421	ED/0036	ED/0036/S/3	Central Hospital, Auchi		Auchi	Etsako West	Edo
1422	ED/0036	ED/0036/S/10	Central Hospital, Auchi		Auchi	Etsako West	Edo
1423	ED/0036	ED/0036/S/8	Central Hospital, Auchi		Auchi	Etsako West	Edo
1424	ED/0036	ED/0036/S/2	Central Hospital, Auchi		Auchi	Etsako West	Edo
1425	ED/0036	ED/0036/S/6	Central Hospital, Auchi		Auchi	Etsako West	Edo
1426	ED/0036	ED/0036/S/1	Central Hospital, Auchi		Auchi	Etsako West	Edo
1427	ED/0041	ED/0041/S/1	General Hospital, Igueben		Igueben	Igueben	Edo
1428	ED/0041	ED/0041/S/2	General Hospital, Igueben		Igueben	Igueben	Edo
1429	ED/0041	ED/0041/S/6	General Hospital, Igueben		Igueben	Igueben	Edo
1430	ED/0043	ED/0043/S/5	Narrow Way Clinic		No.9, S. Ahahor Street, Off Benin-Auchi Expressway, Aduwawa	Ikpoba/Okha	Edo
1431	ED/0049	ED/0049/S/5	Ekpen Hospital		Off Agho Street, Off Ekwenwan Road	Oredo	Edo
1432	ED/0049	ED/0049/S/4	Ekpen Hospital		Off Agho Street, Off Ekwenwan Road	Oredo	Edo
1433	ED/0050	ED/0050/S/8	Central Hospital, Benin		Along Sapele Road, Near Ring Road, Benin	Oredo	Edo
1434	ED/0050	ED/0050/S/4	Central Hospital, Benin		Along Sapele Road, Near Ring Road, Benin	Oredo	Edo
1435	ED/0050	ED/0050/S/1	Central Hospital, Benin		Along Sapele Road, Near Ring Road, Benin	Oredo	Edo
1436	ED/0050	ED/0050/S/2	Central Hospital, Benin		Along Sapele Road, Near Ring Road, Benin	Oredo	Edo
1437	ED/0050	ED/0050/S/5	Central Hospital, Benin		Along Sapele Road, Near Ring Road, Benin	Oredo	Edo
1438	ED/0050	ED/0050/S/15	Central Hospital, Benin		Along Sapele Road, Near Ring Road, Benin	Oredo	Edo
1439	ED/0050	ED/0050/S/6	Central Hospital, Benin		Along Sapele Road, Near Ring Road, Benin	Oredo	Edo
1440	ED/0050	ED/0050/S/3	Central Hospital, Benin		Along Sapele Road, Near Ring Road, Benin	Oredo	Edo
1441	ED/0055	ED/0055/S/5	General Hospital, Orhionmwom		Abudu Town, Orhionmwom	Orhionmwon	Edo
1442	ED/0055	ED/0055/S/4	General Hospital, Orhionmwom		Abudu Town, Orhionmwom	Orhionmwon	Edo
1443	ED/0055	ED/0055/S/3	General Hospital, Orhionmwom		Abudu Town, Orhionmwom	Orhionmwon	Edo
1444	ED/0055	ED/0055/S/6	General Hospital, Orhionmwom		Abudu Town, Orhionmwom	Orhionmwon	Edo

1445	ED/0055	ED/0055/S/1	General Hospital, Orhionmwom		Abudu Town, Orhionmwom	Orhionmwon	Edo
1446	ED/0055	ED/0055/S/2	General Hospital, Orhionmwom		Abudu Town, Orhionmwom	Orhionmwon	Edo
1447	ED/0057	ED/0057/S/2	Government Hospital, Urhonigbe		Urhonigbe Along Agbor Abraka Road.	Orhionmwon	Edo
1448	ED/0057	ED/0057/S/6	Government Hospital, Urhonigbe		Urhonigbe Along Agbor Abraka Road.	Orhionmwon	Edo
1449	ED/0057	ED/0057/S/1	Government Hospital, Urhonigbe		Urhonigbe Along Agbor Abraka Road.	Orhionmwon	Edo
1450	ED/0057	ED/0057/S/5	Government Hospital, Urhonigbe		Urhonigbe Along Agbor Abraka Road.	Orhionmwon	Edo
1451	ED/0057	ED/0057/S/4	Government Hospital, Urhonigbe		Urhonigbe Along Agbor Abraka Road.	Orhionmwon	Edo
1452	ED/0061	ED/0061/S/4	Igbinedion Hospital & Med. Research		Okada Wonderland	Ovia North East	Edo
1453	ED/0061	ED/0061/S/5	Igbinedion Hospital & Med. Research		Okada Wonderland	Ovia North East	Edo
1454	ED/0062	ED/0062/S/2	District Hospital, Ekiadolor		Ekiadolor	Ovia North East	Edo
1455	ED/0062	ED/0062/S/1	District Hospital, Ekiadolor		Ekiadolor	Ovia North East	Edo
1456	ED/0065	ED/0065/S/5	General Hospital, Iguobazuwa		Iguobazuwa Town, Hq Of Ovia South-West, LGA	Ovia South West	Edo
1457	ED/0065	ED/0065/S/2	General Hospital, Iguobazuwa		Iguobazuwa Town, Hq Of Ovia South-West, LGA	Ovia South West	Edo
1458	ED/0065	ED/0065/S/1	General Hospital, Iguobazuwa		Iguobazuwa Town, Hq Of Ovia South-West, LGA	Ovia South West	Edo
1459	ED/0065	ED/0065/S/4	General Hospital, Iguobazuwa		Iguobazuwa Town, Hq Of Ovia South-West, LGA	Ovia South West	Edo
1460	ED/0065	ED/0065/S/6	General Hospital, Iguobazuwa		Iguobazuwa Town, Hq Of Ovia South-West, LGA	Ovia South West	Edo
1461	ED/0066	ED/0066/S/4	Government Hospital, Usen		Usen	Ovia South West	Edo
1462	ED/0066	ED/0066/S/5	Government Hospital, Usen		Usen	Ovia South West	Edo
1463	ED/0066	ED/0066/S/1	Government Hospital, Usen		Usen	Ovia South West	Edo
1464	ED/0066	ED/0066/S/6	Government Hospital, Usen		Usen	Ovia South West	Edo
1465	ED/0066	ED/0066/S/2	Government Hospital, Usen		Usen	Ovia South West	Edo
1466	ED/0074	ED/0074/S/2	Government Hospital	Gen. Medicine, Gen. Surgery, Paediatric, Laboratory, Pharmacy	Uzebba	Owan West	Edo
1467	ED/0074	ED/0074/S/1	Government Hospital	Gen. Medicine, Gen. Surgery, Paediatric, Laboratory, Pharmacy	Uzebba	Owan West	Edo
1468	ED/0074	ED/0074/S/6	Government Hospital	Gen. Medicine, Gen. Surgery, Paediatric, Laboratory, Pharmacy	Uzebba	Owan West	Edo
1469	ED/0076	ED/0076/S/4	District Hospital, Uhunmwode-Egba		Egba	Uhunmwode	Edo
1470	ED/0076	ED/0076/S/2	District Hospital, Uhunmwode-Egba		Egba	Uhunmwode	Edo
1471	ED/0076	ED/0076/S/5	District Hospital, Uhunmwode-Egba		Egba	Uhunmwode	Edo
1472	ED/0076	ED/0076/S/1	District Hospital, Uhunmwode-Egba		Egba	Uhunmwode	Edo
1473	ED/0076	ED/0076/S/6	District Hospital, Uhunmwode-Egba		Egba	Uhunmwode	Edo
1474	ED/0077	ED/0077/S/1	Cottage Hospital, Obayanto		Obayantor, Along Sapele Road, New Benin Owena River Basin De	Orhionmwon	Edo
1475	ED/0077	ED/0077/S/2	Cottage Hospital, Obayanto		Obayantor, Along Sapele Road, New Benin Owena River Basin De	Orhionmwon	Edo
1476	ED/0080	ED/0080/S/10	General Hospital, Fugar		Fugar	Etsako Central	Edo
1477	ED/0080	ED/0080/S/4	General Hospital, Fugar		Fugar	Etsako Central	Edo
1478	ED/0080	ED/0080/S/3	General Hospital, Fugar		Fugar	Etsako Central	Edo
1479	ED/0080	ED/0080/S/5	General Hospital, Fugar		Fugar	Etsako Central	Edo
1480	ED/0080	ED/0080/S/6	General Hospital, Fugar		Fugar	Etsako Central	Edo
1481	ED/0080	ED/0080/S/1	General Hospital, Fugar		Fugar	Etsako Central	Edo
1482	ED/0084	ED/0084/S/11	Azuwa Hospital		2nd West East, Benin City	Oredo	Edo
1483	ED/0084	ED/0084/S/5	Azuwa Hospital		2nd West East, Benin City	Oredo	Edo

1484	ED/0084	ED/0084/S/15	Azuwa Hospital		2nd West East, Benin City	Oredo	Edo
1485	ED/0084	ED/0084/S/2	Azuwa Hospital		2nd West East, Benin City	Oredo	Edo
1486	ED/0087	ED/0087/S/5	Urban Medical Centre		Warrake	Owan West	Edo
1487	ED/0089	ED/0089/S/4	Ihenyen Medical Centre		No. 22, Ogbefun Street, Off College Road, Benin City	Oredo	Edo
1488	ED/0090	ED/0090/S/3	Time Hospital		3,2, Agbonma Lane, Off Ekwenwa Road, Benin City	Oredo	Edo
1489	ED/0090	ED/0090/S/5	Time Hospital		3,2, Agbonma Lane, Off Ekwenwa Road, Benin City	Oredo	Edo
1490	ED/0090	ED/0090/S/4	Time Hospital		3,2, Agbonma Lane, Off Ekwenwa Road, Benin City	Oredo	Edo
1491	ED/0096	ED/0096/S/4	V. J. Essex Pharm	Pharmacy	138 Ekenuwan Road, Benin	Oredo	Edo
1492	ED/0097	ED/0097/S/4	Vamak Pharmacy	Pharmacy	66 Igbe Road, Auchi	Etsako West	Edo
1493	ED/0103	ED/0103/S/11	Fortune Physiotherapy Clinic	Physiotherapy	Ire Street, Off Wire Road, or I.C.E. Road, Benin City	Oredo	Edo
1494	ED/0104	ED/0104/S	Horatio Dental Clinic	Dental	No. 8, Akenzua Road, Off Plymouth, Benin City	Oredo	Edo
1495	ED/0108	ED/0108/S/4	Viviski Pharmaceuticals Ltd.	Pharmacy	Benin City	Oredo	Edo
1496	ED/0109	ED/0109/S/4	Coka Chemist Ltd.	Pharmacy	Benin City	Oredo	Edo
1497	ED/0111	ED/0111/S/5	O.P.R.I. Medical Laboratory Services	Laboratory	No. 31, Ikpoba Hill Road, Benin City	Oredo	Edo
1498	ED/0118	ED/0118/S/7	University Of Benin Teaching Hospital Ugbowo		Benin City	Egor	Edo
1499	ED/0118	ED/0118/S/15	University Of Benin Teaching Hospital Ugbowo		Benin City	Egor	Edo
1500	ED/0118	ED/0118/S/1	University Of Benin Teaching Hospital Ugbowo		Benin City	Egor	Edo
1501	ED/0118	ED/0118/S/11	University Of Benin Teaching Hospital Ugbowo		Benin City	Egor	Edo
1502	ED/0118	ED/0118/S/6	University Of Benin Teaching Hospital Ugbowo		Benin City	Egor	Edo
1503	ED/0118	ED/0118/S/4	University Of Benin Teaching Hospital Ugbowo		Benin City	Egor	Edo
1504	ED/0118	ED/0118/S/8	University Of Benin Teaching Hospital Ugbowo		Benin City	Egor	Edo
1505	ED/0118	ED/0118/S/2	University Of Benin Teaching Hospital Ugbowo		Benin City	Egor	Edo
1506	ED/0118	ED/0118/S/3	University Of Benin Teaching Hospital Ugbowo		Benin City	Egor	Edo
1507	ED/0125	ED/0125/S/8	4 Bde MC Benin		Benin, Edo State	Oredo	Edo
1508	ED/0127	ED/0127/S/4	University of Benin Health Centre		Benin, Edo State	Oredo	Edo
1509	ED/0127	ED/0127/S/5	University of Benin Health Centre		Benin, Edo State	Oredo	Edo
1510	ED/0133	ED/0133/S/14	Ayunko Clinic & Maternity		39 Fed. Govt. College Rd., Ibillo	Akoko Edo	Edo
1511	ED/0134	ED/0134/S/14	Hope Clinic & Maternity		No. 1 Whytehouse Street, Jatau, Auchi, Edo State	Etsako West	Edo
1512	ED/0135	ED/0135/S/7	Faith Mediplex		Igiwa-Amadi/Airport Road GRA, Benin City, Edo State	Oredo	Edo
1513	ED/0135	ED/0135/S/3	Faith Mediplex		Igiwa-Amadi/Airport Road GRA, Benin City, Edo State	Oredo	Edo
1514	ED/0135	ED/0135/S/4	Faith Mediplex		Igiwa-Amadi/Airport Road GRA, Benin City, Edo State	Oredo	Edo
1515	ED/0135	ED/0135/S/6	Faith Mediplex		Igiwa-Amadi/Airport Road GRA, Benin City, Edo State	Oredo	Edo
1516	ED/0135	ED/0135/S/8	Faith Mediplex		Igiwa-Amadi/Airport Road GRA, Benin City, Edo State	Oredo	Edo
1517	ED/0135	ED/0135/S/5	Faith Mediplex		Igiwa-Amadi/Airport Road GRA, Benin City, Edo State	Oredo	Edo
1518	ED/0135	ED/0135/S/2	Faith Mediplex		Igiwa-Amadi/Airport Road GRA, Benin City, Edo State	Oredo	Edo

1519	ED/0135	ED/0135/S/1	Faith Mediplex		Igiwa-Amadi/Airport Road GRA, Benin City, Edo State	Oredo	Edo
1520	ED/0138	ED/0138/S/3	Osula Royal Hospital	Primary	No.5 Idahosa Lane, Oredo LGA	Oredo	Edo
1521	ED/0139	ED/0139/S/1	The Rock Hospital	Primary	39 Imuetinyan Street, Off Ihama Rd, G.R.A Benin Edo State	Oredo	Edo
1522	ED/0139	ED/0139/S/6	The Rock Hospital	Primary	39 Imuetinyan Street, Off Ihama Rd, G.R.A Benin Edo State	Oredo	Edo
1523	ED/0139	ED/0139/S/3	The Rock Hospital	Primary	39 Imuetinyan Street, Off Ihama Rd, G.R.A Benin Edo State	Oredo	Edo
1524	ED/0142	ED/0142/S/1	Our Medical Centre		No. 2 Oregbeni Street, Ikpoba Hill, Ikoba, Benin City	Ikpoba/Okha	Edo
1525	ED/0143	ED/0143/S/10	Lahor Research Lab. & Medical Centre		121 Old Benin, Agbor Road, Benin City	Ikpoba/Okha	Edo
1526	ED/0143	ED/0143/S/5	Lahor Research Lab. & Medical Centre		121 Old Benin, Agbor Road, Benin City	Ikpoba/Okha	Edo
1527	ED/0144	ED/0144/S/6	St. Rapheal Hospital		12, Adesua Road, Oredo LGA, Benin City	Oredo	Edo
1528	ED/0144	ED/0144/S/15	St. Rapheal Hospital		12, Adesua Road, Oredo LGA, Benin City	Oredo	Edo
1529	ED/0145	ED/0145/S/5	Aniso Specialist Medical Center		No. 11 2nd Fed. Road, Off 4th Srt Road or Off Benin Technical Coal Rd, Uselu Ltd Benin City	Egor	Edo
1530	ED/0145	ED/0145/S/2	Aniso Specialist Medical Center		No. 11 2nd Fed. Road, Off 4th Srt Road or Off Benin Technical Coal Rd, Uselu Ltd Benin City	Egor	Edo
1531	ED/0147	ED/0147/S/4	Cystius Pharmacy	Pharmacy	8, Uwasota Road, Ugbowo, Benin City, Edo State	Oredo	Edo
1532	ED/0149	ED/0149/S/8	Tomsek Dental Clinic	Dental	54B, Saponba Road, Benin City, Edo State	Oredo	Edo
1533	ED/0156	ED/0156/S/5	Day Jay Medical Diagnostic Laboratory	Laboratory	150 Upper Mission Road, Benin City, Edo State	Oredo	Edo
1534	ED/0157	ED/0157/S	Stalwart Eye Centre	Ophthalmology	Mechatronic Plaza, 23 Ihama Road, GRA, Benin City, Edo State	Oredo	Edo
1535	ED/0158	ED/0158/S/8	Gil Gal Dental Clinic	Dental	No.7 Ihama Road GRA, Benin City, Edo State	Oredo	Edo
1536	ED/0161	ED/0161/S/1	Alges Pharmacy Ltd	Pharmacy	24 A Ugbowo Lagos Road	Oredo	Edo
1537	ED/0165	ED/0165/S/3	Akemekhai Medical Centre		3, Oyerekhau Lane, Jattu, Esako West, Auchi, Edo State	Etsako West	Edo
1538	ED/0165	ED/0165/S/1	Akemekhai Medical Centre		3, Oyerekhau Lane, Jattu, Esako West, Auchi, Edo State	Etsako West	Edo
1539	ED/0165	ED/0165/S/14	Akemekhai Medical Centre		3, Oyerekhau Lane, Jattu, Esako West, Auchi, Edo State	Etsako West	Edo
1540	ED/0167	ED/0167/S/4	Auchi Polytechnic Medical Centre		Auchi, Edo State	Etsako East	Edo
1541	ED/0168	ED/0168/S/1	Hope Hospital & Maternity		42, Costain Road, Benin, Edo State	Oredo	Edo
1542	ED/0171	ED/0171/S/1	Ogbeide Gen. Practice Clinic		58, Uwa Str., off 2nd East Circular Road, Benin.	Oredo	Edo
1543	ED/0171	ED/0171/S/2	Ogbeide Gen. Practice Clinic		58, Uwa Str., off 2nd East Circular Road, Benin.	Oredo	Edo
1544	ED/0176	ED/0176/S/3	Hope Medical Centre, Igbueben		7 Suleman Street off sapele road, Benin City	Igueben	Edo
1545	ED/0178	ED/0178/S/3	Gift Medical Clinic		6a, Iyamu Str., off Erie Str., Benin	Oredo	Edo
1546	ED/0179	ED/0179/S/1	Echos Hospital		5, Benin Technical College Road, Benin	Egor	Edo
1547	ED/0179	ED/0179/S/15	Echos Hospital		5, Benin Technical College Road, Benin	Egor	Edo
1548	ED/0184	ED/0184/S/3	Irowa Medical Centre		Jos-Bazuaye Est. off Sapele Road, Benin, Edo State	Oredo	Edo
1549	ED/0184	ED/0184/S/5	Irowa Medical Centre		Jos-Bazuaye Est. off Sapele Road, Benin, Edo State	Oredo	Edo
1550	ED/0188	ED/0188/S/11	Cedan Physiotherapy Centre	Physiotherapy	12, Aiwerioba Avenue, Off Akenzua Street, Benin	Oredo	Edo
1551	ED/0190	ED/0190/S/4	Elint Pharmacy	Pharmacy	59, Ugbowo-Lagos Road, Benin	Egor	Edo
1552	ED/0192	ED/0192/S/4	Goodness Pharmacy	Pharmacy	270b, Ugbowo-Lagos Road, Benin	Egor	Edo
1553	ED/0195	ED/0195/S/4	JIMAH YUSUF PHARMACEUTICAL	Pharmacy	58, OTARU RD,AUCHI	Etsako West	Edo
1554	ED/0199	ED/0199/S/5	NEW GEN MEDICAL DIAGNOSTICS	Laboratory	41,PLYMOUTH RD,BENIN	Oredo	Edo

1555	ED/0202	ED/0202/S/4	OSKAJAY NIG. LTD	Pharmacy	88,FORESTRY ROAD,BENIN	Oredo	Edo
1556	ED/0203	ED/0203/S/5	SALAMI MEDICAL LABORATORY	Laboratory	52 MISSION ROAD, BENIN	Oredo	Edo
1557	ED/0205	ED/0205/S/4	SYL-WRIGHT CHEMISTS	PHARMACY	1, BOUNDARY ROAD,BENIN	Oredo	Edo
1558	ED/0207	ED/0207/S/3	Edi International Hospital	O&G	Coronation Drive Off GRA Benin	Oredo	Edo
1559	ED/0208	ED/0208/S/5	Orbiose Hospital	Laboratory	2 Isibor Agbontan Street, Off Benin/Auchi Road Aduwawa	Ikpoba/Okha	Edo
1560	ED/0209	ED/0209/S/15	Mayo's Eye Clinic	Opthamology	32 Utoinwan Street, New Benin	Oredo	Edo
1561	ED/0211	ED/0211/S/15	Prime Optical and Eye Clinic	Opthamology	41 Airport Road, Benin	Oredo	Edo
1562	ED/0217	ED/0217/S/13	St Philomina Catholic Hospital		23 Dawson Road, off Mission Road, Benin City	Oredo	Edo
1563	ED/0217	ED/0217/S/1	St Philomina Catholic Hospital		23 Dawson Road, off Mission Road, Benin City	Oredo	Edo
1564	ED/0217	ED/0217/S/2	St Philomina Catholic Hospital		23 Dawson Road, off Mission Road, Benin City	Oredo	Edo
1565	ED/0217	ED/0217/S/3	St Philomina Catholic Hospital		23 Dawson Road, off Mission Road, Benin City	Oredo	Edo
1566	ED/0217	ED/0217/S/4	St Philomina Catholic Hospital		23 Dawson Road, off Mission Road, Benin City	Oredo	Edo
1567	ED/0217	ED/0217/S/5	St Philomina Catholic Hospital		23 Dawson Road, off Mission Road, Benin City	Oredo	Edo
1568	ED/0217	ED/0217/S/6	St Philomina Catholic Hospital		23 Dawson Road, off Mission Road, Benin City	Oredo	Edo
1569	ED/0217	ED/0217/S/7	St Philomina Catholic Hospital		23 Dawson Road, off Mission Road, Benin City	Oredo	Edo
1570	ED/0218	ED/0218/S/6	Stella Obasanjo Women & Children Hospital		Country Home, Hotel Road, Benin City	Oredo	Edo
1571	ED/0218	ED/0218/S/8	Stella Obasanjo Women & Children Hospital		Country Home, Hotel Road, Benin City	Oredo	Edo
1572	ED/0218	ED/0218/S/1	Stella Obasanjo Women & Children Hospital		Country Home, Hotel Road, Benin City	Oredo	Edo
1573	ED/0218	ED/0218/S/2	Stella Obasanjo Women & Children Hospital		Country Home, Hotel Road, Benin City	Oredo	Edo
1574	ED/0218	ED/0218/S/7	Stella Obasanjo Women & Children Hospital		Country Home, Hotel Road, Benin City	Oredo	Edo
1575	ED/0218	ED/0218/S/15	Stella Obasanjo Women & Children Hospital		Country Home, Hotel Road, Benin City	Oredo	Edo
1576	ED/0218	ED/0218/S/12	Stella Obasanjo Women & Children Hospital		Country Home, Hotel Road, Benin City	Oredo	Edo
1577	ED/0218	ED/0218/S/5	Stella Obasanjo Women & Children Hospital		Country Home, Hotel Road, Benin City	Oredo	Edo
1578	ED/0218	ED/0218/S/4	Stella Obasanjo Women & Children Hospital		Country Home, Hotel Road, Benin City	Oredo	Edo
1579	ED/0218	ED/0218/S/3	Stella Obasanjo Women & Children Hospital		Country Home, Hotel Road, Benin City	Oredo	Edo
1580	ED/0220	ED/0220/S/4	Ladith Pharmaceutical Ltd		No. 77,Airport Road, Benin Edo State	Oredo	Edo
1581	ED/0221	ED/0221/S/4	Ladith Pharmaceutical co. Ltd		59,Ore-Oghene Near Olihia MKT Benin Edo State	Oredo	Edo
1582	ED/0223	ED/0223/S/5	Ovbioise Hospital		2 Isibor Aigbontaen Street, Aduwawa, Benin City	Ikpoba/Okha	Edo
1583	ED/0224	ED/0224/S/15	Uwa Eye Clinic		8, Igun Street, Off. Sakpomba RD.	Oredo	Edo
1584	ED/0227	ED/0227/S/2	Ukponmwan Foundation Hospital		53 Idahosa Street, Benin City, Edo State	Oredo	Edo
1585	ED/0227	ED/0227/S/15	Ukponmwan Foundation Hospital		53 Idahosa Street, Benin City, Edo State	Oredo	Edo
1586	ED/0231	ED/0231/S/4	Flowell Pharma Nig.Ltd		69b Sapele RD.	Oredo	Edo
1587	ED/0233	ED/0233/S/3	Life Medical Centre		34 Guobadia Street, Off Ugbor Rd, G.R.A, Benin City Edo State.	Egor	Edo
1588	ED/0240	ED/0240/S/3	St. Camillus Hospital		Uromi Edo State	Esan North East	Edo
1589	ED/0240	ED/0240/S/5	St. Camillus Hospital		Uromi Edo State	Esan North East	Edo
1590	ED/0240	ED/0240/S/4	St. Camillus Hospital		Uromi Edo State	Esan North East	Edo
1591	ED/0241	ED/0241/S/3	Fountain Of Life Specialist Hospital		3B Ogbesasa Street, Off Sapele Road Edo State	Oredo	Edo
1592	ED/0242	ED/0242/S/4	Edi International Hospital		No. 1,Coronation Drive, Off Adeyan Avenue ,G.R.A Benin City Edo State	Oredo	Edo

1593	ED/0242	ED/0242/S/5	Edi International Hospital		No. 1,Coronation Drive, Off Adeyan Avenue ,G.R.A Benin City Edo State	Oredo	Edo
1594	ED/0248	ED/0248/S/1	Total Health Medical Services Center		20, Ayo-Idiaghe Street,Isiohor,Benin Edo State	Egor	Edo
1595	ED/0253	ED/0253/S/1	Ebony Medical Centre		59 Uwasota Rd, Ugbowo Benin City Edo State	Egor	Edo
1596	ED/0253	ED/0253/S/3	Ebony Medical Centre		59 Uwasota Rd, Ugbowo Benin City Edo State	Egor	Edo
1597	ED/0253	ED/0253/S/6	Ebony Medical Centre		59 Uwasota Rd, Ugbowo Benin City Edo State	Egor	Edo
1598	ED/0257	ED/0257/S/8	Ubani Dental Clinic		77 Momodu Ajayi Road	Akoko Edo	Edo
1599	ED/0259	ED/0259/S/4	Veras Pharmacy		Veras Plaza,2-Abuja Express Way, Ibillo ,Edo State	Akoko Edo	Edo
1600	ED/0261	ED/0261/S/3	Police Hospital Benin G.R.A		Plot 12,Dennis Osadebey Avenue, Benin Edo State	Oredo	Edo
1601	ED/0261	ED/0261/S/5	Police Hospital Benin G.R.A		Plot 12,Dennis Osadebey Avenue, Benin Edo State	Oredo	Edo
1602	ED/0261	ED/0261/S/7	Police Hospital Benin G.R.A		Plot 12,Dennis Osadebey Avenue, Benin Edo State	Oredo	Edo
1603	ED/0264	ED/0264/S/3	Eromosele Medical Centre		Ujoelen Road Ekpoma	Esan West	Edo
1604	ED/0270	ED/0270/S/2	Halpha Specialist Hospital		1,Akhiobanke Street, G.R.A, Benin Edo State	Oredo	Edo
1605	ED/0272	ED/0272/S/3	Gims Clinic LTD		12 Edebiri Street ,Ogbe Quarters, Benin City Edo State	Oredo	Edo
1606	ED/0272	ED/0272/S/1	Gims Clinic LTD		12 Edebiri Street ,Ogbe Quarters, Benin City Edo State	Oredo	Edo
1607	ED/0275	ED/0275/S/3	Eguavon Medical Centre		No 5,Abia,Iruekpen,Ekpoma,Edo State	Esan West	Edo
1608	ED/0275	ED/0275/S/5	Eguavon Medical Centre		No 5,Abia,Iruekpen,Ekpoma,Edo State	Esan West	Edo
1609	ED/0276	ED/0276/S/5	Oriaifo Medical Center		No 1,Oriaifo Close, Uromi-Illushi Road, Uromi Edo State	Esan North East	Edo
1610	ED/0276	ED/0276/S/3	Oriaifo Medical Center		No 1,Oriaifo Close, Uromi-Illushi Road, Uromi Edo State	Esan North East	Edo
1611	ED/0278	ED/0278/S/5	Eseohe Medical Center		No. 2,Police Barrack RD ,Off Benin Auchi Express Way, Beside Mousco Petrol Station ,Ekpoma	Esan West	Edo
1612	ED/0278	ED/0278/S/6	Eseohe Medical Center		No. 2,Police Barrack RD ,Off Benin Auchi Express Way, Beside Mousco Petrol Station ,Ekpoma	Esan West	Edo
1613	ED/0278	ED/0278/S/3	Eseohe Medical Center		No. 2,Police Barrack RD ,Off Benin Auchi Express Way, Beside Mousco Petrol Station ,Ekpoma	Esan West	Edo
1614	ED/0278	ED/0278/S/1	Eseohe Medical Center		No. 2,Police Barrack RD ,Off Benin Auchi Express Way, Beside Mousco Petrol Station ,Ekpoma	Esan West	Edo
1615	ED/0279	ED/0279/S/2	Halpha Specialist Hospital		1, Akhiobanke Street, G.R.A, Benin	Oredo	Edo
1616	ED/0280	ED/0280/S/2	Jimon Medical Center		152, Upper Owina Street Evbotubu Qtrs, Benin Edo State.	Oredo	Edo
1617	EK/0001	EK/0001/S/5	St.Gregory Hospital	Laboratory, USS, O & G, Pharmacy, General Surgery	KM 2 Basiri Iyin Road, Ado Ekiti	Ado Ekiti	Ekiti
1618	EK/0001	EK/0001/S/14	St.Gregory Hospital	Laboratory, USS, O & G, Pharmacy, General Surgery	KM 2 Basiri Iyin Road, Ado Ekiti	Ado Ekiti	Ekiti
1619	EK/0001	EK/0001/S/4	St.Gregory Hospital	Laboratory, USS, O & G, Pharmacy, General Surgery	KM 2 Basiri Iyin Road, Ado Ekiti	Ado Ekiti	Ekiti
1620	EK/0001	EK/0001/S/3	St.Gregory Hospital	Laboratory, USS, O & G, Pharmacy, General Surgery	KM 2 Basiri Iyin Road, Ado Ekiti	Ado Ekiti	Ekiti
1621	EK/0001	EK/0001/S/1	St.Gregory Hospital	Laboratory, USS, O & G, Pharmacy, General Surgery	KM 2 Basiri Iyin Road, Ado Ekiti	Ado Ekiti	Ekiti
1622	EK/0002	EK/0002/S/5	Olumorin Specialist Hospital		Ado-Ekiti	Ado Ekiti	Ekiti
1623	EK/0002	EK/0002/S/14	Olumorin Specialist Hospital		Ado-Ekiti	Ado Ekiti	Ekiti

1624	EK/0002	EK/0002/S/3	Olumorin Specialist Hospital		Ado-Ekiti	Ado Ekiti	Ekiti
1625	EK/0004	EK/0004/S/14	Ade-Tade Hospital		11, Okebola Street, Ado Ekiti	Ado Ekiti	Ekiti
1626	EK/0004	EK/0004/S/5	Ade-Tade Hospital		11, Okebola Street, Ado Ekiti	Ado Ekiti	Ekiti
1627	EK/0005	EK/0005/S/5	Orire Hospital Complex		1, Orire Lane, Box 944, Ado Ekiti	Ado Ekiti	Ekiti
1628	EK/0006	EK/0006/S/6	University Teaching Hospital, Ado Ekiti		Ado Ekiti	Ado Ekiti	Ekiti
1629	EK/0006	EK/0006/S/11	University Teaching Hospital, Ado Ekiti		Ado Ekiti	Ado Ekiti	Ekiti
1630	EK/0006	EK/0006/S/12	University Teaching Hospital, Ado Ekiti		Ado Ekiti	Ado Ekiti	Ekiti
1631	EK/0006	EK/0006/S/7	University Teaching Hospital, Ado Ekiti		Ado Ekiti	Ado Ekiti	Ekiti
1632	EK/0006	EK/0006/S/4	University Teaching Hospital, Ado Ekiti		Ado Ekiti	Ado Ekiti	Ekiti
1633	EK/0006	EK/0006/S/3	University Teaching Hospital, Ado Ekiti		Ado Ekiti	Ado Ekiti	Ekiti
1634	EK/0006	EK/0006/S/2	University Teaching Hospital, Ado Ekiti		Ado Ekiti	Ado Ekiti	Ekiti
1635	EK/0006	EK/0006/S/10	University Teaching Hospital, Ado Ekiti		Ado Ekiti	Ado Ekiti	Ekiti
1636	EK/0006	EK/0006/S/5	University Teaching Hospital, Ado Ekiti		Ado Ekiti	Ado Ekiti	Ekiti
1637	EK/0006	EK/0006/S/1	University Teaching Hospital, Ado Ekiti		Ado Ekiti	Ado Ekiti	Ekiti
1638	EK/0007	EK/0007/S/12	Federal Teaching Hospital		Ido Ekiti	Ido / Osi	Ekiti
1639	EK/0007	EK/0007/S/11	Federal Teaching Hospital		Ido Ekiti	Ido / Osi	Ekiti
1640	EK/0007	EK/0007/S/3	Federal Teaching Hospital		Ido Ekiti	Ido / Osi	Ekiti
1641	EK/0007	EK/0007/S/9	Federal Teaching Hospital		Ido Ekiti	Ido / Osi	Ekiti
1642	EK/0007	EK/0007/S/2	Federal Teaching Hospital		Ido Ekiti	Ido / Osi	Ekiti
1643	EK/0007	EK/0007/S/4	Federal Teaching Hospital		Ido Ekiti	Ido / Osi	Ekiti
1644	EK/0007	EK/0007/S/15	Federal Teaching Hospital		Ido Ekiti	Ido / Osi	Ekiti
1645	EK/0007	EK/0007/S/8	Federal Teaching Hospital		Ido Ekiti	Ido / Osi	Ekiti
1646	EK/0007	EK/0007/S/7	Federal Teaching Hospital		Ido Ekiti	Ido / Osi	Ekiti
1647	EK/0007	EK/0007/S/5	Federal Teaching Hospital		Ido Ekiti	Ido / Osi	Ekiti
1648	EK/0007	EK/0007/S/10	Federal Teaching Hospital		Ido Ekiti	Ido / Osi	Ekiti
1649	EK/0007	EK/0007/S/6	Federal Teaching Hospital		Ido Ekiti	Ido / Osi	Ekiti
1650	EK/0011	EK/0011/S/5	Joe-Jane Medical Centre		27 Dallimore Street, Ado Ekiti	Ado Ekiti	Ekiti
1651	EK/0013	EK/0013/S/4	State Specialist Hospital	Pharmacy, Laboratory	Ijero-Ekiti	Ijero	Ekiti
1652	EK/0013	EK/0013/S/5	State Specialist Hospital	Pharmacy, Laboratory	Ijero-Ekiti	Ijero	Ekiti
1653	EK/0014	EK/0014/S/5	State Specialist Hospital	Pharmacy, Laboratory	Ikere-Ekiti	Ikere	Ekiti
1654	EK/0014	EK/0014/S/4	State Specialist Hospital	Pharmacy, Laboratory	Ikere-Ekiti	Ikere	Ekiti
1655	EK/0016	EK/0016/S/10	State Specialist Hospital	Pharmacy, Laboratory, Orthopaedic, Internal Medicine	Ado	Ado Ekiti	Ekiti
1656	EK/0016	EK/0016/S/4	State Specialist Hospital	Pharmacy, Laboratory, Orthopaedic, Internal Medicine	Ado	Ado Ekiti	Ekiti
1657	EK/0016	EK/0016/S/2	State Specialist Hospital	Pharmacy, Laboratory, Orthopaedic, Internal Medicine	Ado	Ado Ekiti	Ekiti
1658	EK/0016	EK/0016/S/5	State Specialist Hospital	Pharmacy, Laboratory, Orthopaedic, Internal Medicine	Ado	Ado Ekiti	Ekiti
1659	EK/0017	EK/0017/S/5	State Specialist Hospital	Pharmacy, Laboratory	Ikere	Ikere	Ekiti
1660	EK/0017	EK/0017/S/4	State Specialist Hospital	Pharmacy, Laboratory	Ikere	Ikere	Ekiti
1661	EK/0018	EK/0018/S/4	Federal Medical Centre, Ido-Ekiti	Pharmacy, Laboratory, Radiology	Ido-Ekiti	Ado Ekiti	Ekiti

1662	EK/0018	EK/0018/S/7	Federal Medical Centre, Ido-Ekiti	Pharmacy, Laboratory, Radiology	Ido-Ekiti	Ado Ekiti	Ekiti
1663	EK/0018	EK/0018/S/5	Federal Medical Centre, Ido-Ekiti	Pharmacy, Laboratory, Radiology	Ido-Ekiti	Ado Ekiti	Ekiti
1664	EK/0019	EK/0019/S/7	State Specialist Hospital	Pharmacy, Laboratory, Radiology, Physiotherapy	Ikole Ekiti	Ikole	Ekiti
1665	EK/0020	EK/0020/S/4	Lanre & Lanre Pharmacy	Pharmacy	No.10 Akin Akeju Shopping Complex Opp. State	Ado Ekiti	Ekiti
1666	EK/0023	EK/0023/S/4	Julie Pharmacy	Pharmacy	Ado	Ado Ekiti	Ekiti
1667	EK/0024	EK/0024/S/5	Grace Medical Laboratories	Laboratory	Ado	Ado Ekiti	Ekiti
1668	EK/0029	EK/0029/S/14	Adetoyin Hospital		No. L16 Onigari, Secretariat Rd., Ado Ekiti	Ado Ekiti	Ekiti
1669	EK/0032	EK/0032/S/5	Federal Polytechnic Health Centre	Primary Provider	Ado Ekiti, Ekiti State	Ado Ekiti	Ekiti
1670	EK/0033	EK/0033/S/1	VICTORY HOSPITAL		2 AWEDELE STR, ADO-EKITI	Ado Ekiti	Ekiti
1671	EK/0034	EK/0034/S/1	STATE SPECIALIST HOSPITAL	O & G, Pharmacy, General Surgery	IKOLE-EKITI	Ikole	Ekiti
1672	EK/0035	EK/0035/S/4	UNIVERSITY OF ADO EKITI HEALTH CENTRE		MAIN CAMPUS	Ado Ekiti	Ekiti
1673	EK/0035	EK/0035/S/5	UNIVERSITY OF ADO EKITI HEALTH CENTRE		MAIN CAMPUS	Ado Ekiti	Ekiti
1674	EK/0038	EK/0038/S/2	Endoscopy De Gilead Specialist Hospital		1, Gilead Lane, NTA Road, off Ilawe Road, Ado-Ekiti	Ado Ekiti	Ekiti
1675	EK/0047	EK/0047/S/3	Maria Assumptia Catholic Hospital		Ireje Street, Ikere Road, Ado Ekiti	Ado Ekiti	Ekiti
1676	EK/0055	EK/0055/S/6	Maternal Child Specialist Clinic		Adebayo Estate , Former UNAD Health Centre, Iwokoro, Ado Ekiti, Ekiti State.	Ado Ekiti	Ekiti
1677	EK/0055	EK/0055/S/7	Maternal Child Specialist Clinic		Adebayo Estate , Former UNAD Health Centre, Iwokoro, Ado Ekiti, Ekiti State.	Ado Ekiti	Ekiti
1678	EK/0055	EK/0055/S/15	Maternal Child Specialist Clinic		Adebayo Estate , Former UNAD Health Centre, Iwokoro, Ado Ekiti, Ekiti State.	Ado Ekiti	Ekiti
1679	EK/0055	EK/0055/S/1	Maternal Child Specialist Clinic		Adebayo Estate , Former UNAD Health Centre, Iwokoro, Ado Ekiti, Ekiti State.	Ado Ekiti	Ekiti
1680	EK/0055	EK/0055/S/3	Maternal Child Specialist Clinic		Adebayo Estate , Former UNAD Health Centre, Iwokoro, Ado Ekiti, Ekiti State.	Ado Ekiti	Ekiti
1681	EK/0059	EK/0059/S/3	Queens Care Specialist Hospital		1 Unity Avenue, UM Iyin Road, Ado Ekiti, Ekiti State.	Ado Ekiti	Ekiti
1682	EK/0060	EK/0060/S/1	Itunuoluwa Medical Centre		Gbahun Alore Street Ekute Quarters, Ado Street Ekute Quarters, Ado Ekiti, Ekiti State.	Ado Ekiti	Ekiti
1683	EK/0061	EK/0061/S/3	Potter Family Care Clinic		114 Okela Housing Road Ado Ekiti, Ekiti State	Ado Ekiti	Ekiti
1684	EK/0062	EK/0062/S/3	Olives Specialist Clinics & Fertility Centre.		Plot 5 Tiniola Avenue Ekiti Housing Estate Ado Ekiti, Ekiti State.	Ado Ekiti	Ekiti
1685	EK/0063	EK/0063/S/3	Eden Life Hospital		Opp. CAC Comprehensive High Umem Avenue Ilawe Road, Ado Ekiti, Ekiti State.	Ado Ekiti	Ekiti
1686	EK/0064	EK/0064/S/5	Maternal Child Specialist		Adebayo Road Ado Ekiti, Ekiti State.	Ado Ekiti	Ekiti
1687	EK/0067	EK/0067/S/3	Unique Consultant Hospital		Adebayo, Ado Ekiti, Ekiti State	Ado Ekiti	Ekiti
1688	EK/0069	EK/0069/S/10	Grenfield Specialist Hospital and Diagnostic Centre		Surulere street, off Ora Road, Ido Ekiti, Ekiti State	Ido / Osi	Ekiti
1689	EK/0071	EK/0071/S/1	Dominion Specialist Medical and Diagnostic Centre		No. 4, Nova Road, Adebayo, Ado Ekiti, Ekiti State.	Ado Ekiti	Ekiti
1690	EK/0071	EK/0071/S/6	Dominion Specialist Medical and Diagnostic Centre		No. 4, Nova Road, Adebayo, Ado Ekiti, Ekiti State.	Ado Ekiti	Ekiti
1691	EK/0071	EK/0071/S/3	Dominion Specialist Medical and Diagnostic Centre		No. 4, Nova Road, Adebayo, Ado Ekiti, Ekiti State.	Ado Ekiti	Ekiti

1692	EK/0074	EK/0074/S/2	Heirs Specialist Hospital, Oye Equity		Madam Aluko's House, Irare Estate, Oye Ekiti, Ekiti State.	Oye	Ekiti
1693	EN/0002	EN/0002/S/7	St. Patricks Hospital & Maternity		8, Owerri Road, Asata, Enugu	Enugu East	Enugu
1694	EN/0002	EN/0002/S/3	St. Patricks Hospital & Maternity		8, Owerri Road, Asata, Enugu	Enugu East	Enugu
1695	EN/0006	EN/0006/S/2	Christ The King Hospital		No. 18 Emeka Abalu Ave. Phase 6, Trans Ekulu, Enugu	Enugu East	Enugu
1696	EN/0006	EN/0006/S/1	Christ The King Hospital		No. 18 Emeka Abalu Ave. Phase 6, Trans Ekulu, Enugu	Enugu East	Enugu
1697	EN/0007	EN/0007/S/2	Peenok Medical Centre		24, Zik Avenue, Uwani, Enugu	Enugu South	Enugu
1698	EN/0007	EN/0007/S/3	Peenok Medical Centre		24, Zik Avenue, Uwani, Enugu	Enugu South	Enugu
1699	EN/0008	EN/0008/S/6	Dr. Nlogha Okeke Memorial Foundation, Enugu.		30, Amigbo Lane Uwani, Enugu	Enugu South	Enugu
1700	EN/0008	EN/0008/S/14	Dr. Nlogha Okeke Memorial Foundation, Enugu.		30, Amigbo Lane Uwani, Enugu	Enugu South	Enugu
1701	EN/0008	EN/0008/S/15	Dr. Nlogha Okeke Memorial Foundation, Enugu.		30, Amigbo Lane Uwani, Enugu	Enugu South	Enugu
1702	EN/0008	EN/0008/S/8	Dr. Nlogha Okeke Memorial Foundation, Enugu.		30, Amigbo Lane Uwani, Enugu	Enugu South	Enugu
1703	EN/0008	EN/0008/S/2	Dr. Nlogha Okeke Memorial Foundation, Enugu.		30, Amigbo Lane Uwani, Enugu	Enugu South	Enugu
1704	EN/0008	EN/0008/S/3	Dr. Nlogha Okeke Memorial Foundation, Enugu.		30, Amigbo Lane Uwani, Enugu	Enugu South	Enugu
1705	EN/0008	EN/0008/S/1	Dr. Nlogha Okeke Memorial Foundation, Enugu.		30, Amigbo Lane Uwani, Enugu	Enugu South	Enugu
1706	EN/0008	EN/0008/S/4	Dr. Nlogha Okeke Memorial Foundation, Enugu.		30, Amigbo Lane Uwani, Enugu	Enugu South	Enugu
1707	EN/0008	EN/0008/S/5	Dr. Nlogha Okeke Memorial Foundation, Enugu.		30, Amigbo Lane Uwani, Enugu	Enugu South	Enugu
1708	EN/0009	EN/0009/S/3	Jeno Hospital Ltd.		23 Nnamdi Azikiwe Avenue, Trans-Ekulu, Enugu	Enugu East	Enugu
1709	EN/0013	EN/0013/S/3	Ristela Hospital & Maternity		3, Ristela Close, Independence Layout, Off Residential Road, E	Enugu East	Enugu
1710	EN/0016	EN/0016/S/15	Ntasiobi Hospital,		Enugu	Enugu South	Enugu
1711	EN/0016	EN/0016/S/2	Ntasiobi Hospital,		Enugu	Enugu South	Enugu
1712	EN/0016	EN/0016/S/10	Ntasiobi Hospital,		Enugu	Enugu South	Enugu
1713	EN/0016	EN/0016/S/6	Ntasiobi Hospital,		Enugu	Enugu South	Enugu
1714	EN/0016	EN/0016/S/4	Ntasiobi Hospital,		Enugu	Enugu South	Enugu
1715	EN/0016	EN/0016/S/8	Ntasiobi Hospital,		Enugu	Enugu South	Enugu
1716	EN/0016	EN/0016/S/14	Ntasiobi Hospital,		Enugu	Enugu South	Enugu
1717	EN/0016	EN/0016/S/1	Ntasiobi Hospital,		Enugu	Enugu South	Enugu
1718	EN/0016	EN/0016/S/3	Ntasiobi Hospital,		Enugu	Enugu South	Enugu
1719	EN/0016	EN/0016/S/5	Ntasiobi Hospital,		Enugu	Enugu South	Enugu
1720	EN/0017	EN/0017/S/4	Akulue Memorial Hospital		16/18 Akulue Road, Onuiyi, Nsukka, Enugu	Nsukka	Enugu
1721	EN/0018	EN/0018/S/2	Unity Ngwo Hospital & Mat.		Ngwo, Enugu, Enugu State	Udi	Enugu
1722	EN/0018	EN/0018/S/3	Unity Ngwo Hospital & Mat.		Ngwo, Enugu, Enugu State	Udi	Enugu
1723	EN/0025	EN/0025/S/3	Ibezim Medical Clinics		56, Ufuma Street, Achara Layout, Enugu	Enugu South	Enugu
1724	EN/0026	EN/0026/S/3	Kenechukwu Specialist Hospital & Maternity		94, Nike Road, Abakpa, Enugu State	Enugu East	Enugu
1725	EN/0026	EN/0026/S/5	Kenechukwu Specialist Hospital & Maternity		94, Nike Road, Abakpa, Enugu State	Enugu East	Enugu
1726	EN/0027	EN/0027/S/3	Julius Ezenyirioha Memorial Hospital & Maternity		32, Nike Road, Abakpa Nike, Enugu.	Enugu East	Enugu
1727	EN/0027	EN/0027/S/5	Julius Ezenyirioha Memorial Hospital & Maternity		32, Nike Road, Abakpa Nike, Enugu.	Enugu East	Enugu
1728	EN/0029	EN/0029/S/1	St. Leo's Specialist Hospital		Nike Lake Road, Abakpa, Enugu	Enugu East	Enugu
1729	EN/0034	EN/0034/S/1	The Merken Hospital Centre		Amechi Road, Awkunanaw, Enugu	Enugu South	Enugu
1730	EN/0034	EN/0034/S/3	The Merken Hospital Centre		Amechi Road, Awkunanaw, Enugu	Enugu South	Enugu
1731	EN/0037	EN/0037/S/1	Royal Hospital		3 Aria Road, Gra, Enugu	Enugu East	Enugu
1732	EN/0037	EN/0037/S/2	Royal Hospital		3 Aria Road, Gra, Enugu	Enugu East	Enugu
1733	EN/0037	EN/0037/S/5	Royal Hospital		3 Aria Road, Gra, Enugu	Enugu East	Enugu

1734	EN/0042	EN/0042/S/4	Annuciation Hospital		Emene, Enugu.	Aninri	Enugu
1735	EN/0042	EN/0042/S/1	Annuciation Hospital		Emene, Enugu.	Aninri	Enugu
1736	EN/0042	EN/0042/S/2	Annuciation Hospital		Emene, Enugu.	Aninri	Enugu
1737	EN/0042	EN/0042/S/3	Annuciation Hospital		Emene, Enugu.	Aninri	Enugu
1738	EN/0042	EN/0042/S/15	Annuciation Hospital		Emene, Enugu.	Aninri	Enugu
1739	EN/0042	EN/0042/S/14	Annuciation Hospital		Emene, Enugu.	Aninri	Enugu
1740	EN/0042	EN/0042/S/12	Annuciation Hospital		Emene, Enugu.	Aninri	Enugu
1741	EN/0042	EN/0042/S/6	Annuciation Hospital		Emene, Enugu.	Aninri	Enugu
1742	EN/0042	EN/0042/S/8	Annuciation Hospital		Emene, Enugu.	Aninri	Enugu
1743	EN/0042	EN/0042/S/5	Annuciation Hospital		Emene, Enugu.	Aninri	Enugu
1744	EN/0044	EN/0044/S/13	ESUT Teaching Hospital, Parklane		G R A, Enugu	Enugu East	Enugu
1745	EN/0044	EN/0044/S/15	ESUT Teaching Hospital, Parklane		G R A, Enugu	Enugu East	Enugu
1746	EN/0044	EN/0044/S/5	ESUT Teaching Hospital, Parklane		G R A, Enugu	Enugu East	Enugu
1747	EN/0044	EN/0044/S/4	ESUT Teaching Hospital, Parklane		G R A, Enugu	Enugu East	Enugu
1748	EN/0044	EN/0044/S/11	ESUT Teaching Hospital, Parklane		G R A, Enugu	Enugu East	Enugu
1749	EN/0044	EN/0044/S/3	ESUT Teaching Hospital, Parklane		G R A, Enugu	Enugu East	Enugu
1750	EN/0044	EN/0044/S/6	ESUT Teaching Hospital, Parklane		G R A, Enugu	Enugu East	Enugu
1751	EN/0044	EN/0044/S/7	ESUT Teaching Hospital, Parklane		G R A, Enugu	Enugu East	Enugu
1752	EN/0044	EN/0044/S/12	ESUT Teaching Hospital, Parklane		G R A, Enugu	Enugu East	Enugu
1753	EN/0044	EN/0044/S/1	ESUT Teaching Hospital, Parklane		G R A, Enugu	Enugu East	Enugu
1754	EN/0044	EN/0044/S/2	ESUT Teaching Hospital, Parklane		G R A, Enugu	Enugu East	Enugu
1755	EN/0044	EN/0044/S/10	ESUT Teaching Hospital, Parklane		G R A, Enugu	Enugu East	Enugu
1756	EN/0046	EN/0046/S/5	Orakwue Hospital		16 Affa Street, Uwani, Enugu	Enugu South	Enugu
1757	EN/0046	EN/0046/S/7	Orakwue Hospital		16 Affa Street, Uwani, Enugu	Enugu South	Enugu
1758	EN/0046	EN/0046/S/2	Orakwue Hospital		16 Affa Street, Uwani, Enugu	Enugu South	Enugu
1759	EN/0053	EN/0053/S	St. Leo's Hospital	General surgery, O & G	57, Nike Lake Road (Opp. Amazing Love Church)	Enugu East	Enugu
1760	EN/0053	EN/0053/S/3	St. Leo's Hospital	General surgery, O & G	57, Nike Lake Road (Opp. Amazing Love Church)	Enugu East	Enugu
1761	EN/0057	EN/0057/S/11	Parklane Specialist Hospital	Otolaryngology, Dentist, Dermatology, Physiotherapy, Orthopaedic, Radiology, Paediatrics, O&G, Int. med., Gen. surg., Urology, Pharm., Lab., Ophthalmology, Diabetes, Nutrition, Optometry	Enugu, Enugu State	Aninri	Enugu
1762	EN/0057	EN/0057/S/10	Parklane Specialist Hospital	Otolaryngology, Dentist, Dermatology, Physiotherapy, Orthopaedic, Radiology, Paediatrics, O&G, Int. med., Gen. surg., Urology, Pharm., Lab., Ophthalmology, Diabetes, Nutrition, Optometry	Enugu, Enugu State	Aninri	Enugu

1763	EN/0057	EN/0057/S/7	Parklane Specialist Hospital	Otolaryngology, Dentist, Dermatology, Physiotherapy, Orthopaedic, Radiology, Paediatrics, O&G, Int. med., Gen. surg., Urology, Pharm., Lab., Ophthalmology, Diabetes, Nutrition, Optometry	Enugu, Enugu State	Aninri	Enugu
1764	EN/0057	EN/0057/S/6	Parklane Specialist Hospital	Otolaryngology, Dentist, Dermatology, Physiotherapy, Orthopaedic, Radiology, Paediatrics, O&G, Int. med., Gen. surg., Urology, Pharm., Lab., Ophthalmology, Diabetes, Nutrition, Optometry	Enugu, Enugu State	Aninri	Enugu
1765	EN/0057	EN/0057/S/3	Parklane Specialist Hospital	Otolaryngology, Dentist, Dermatology, Physiotherapy, Orthopaedic, Radiology, Paediatrics, O&G, Int. med., Gen. surg., Urology, Pharm., Lab., Ophthalmology, Diabetes, Nutrition, Optometry	Enugu, Enugu State	Aninri	Enugu
1766	EN/0057	EN/0057/S/2	Parklane Specialist Hospital	Otolaryngology, Dentist, Dermatology, Physiotherapy, Orthopaedic, Radiology, Paediatrics, O&G, Int. med., Gen. surg., Urology, Pharm., Lab., Ophthalmology, Diabetes, Nutrition, Optometry	Enugu, Enugu State	Aninri	Enugu
1767	EN/0057	EN/0057/S/1	Parklane Specialist Hospital	Otolaryngology, Dentist, Dermatology, Physiotherapy, Orthopaedic, Radiology, Paediatrics, O&G, Int. med., Gen. surg., Urology, Pharm., Lab., Ophthalmology, Diabetes, Nutrition, Optometry	Enugu, Enugu State	Aninri	Enugu
1768	EN/0057	EN/0057/S/4	Parklane Specialist Hospital	Otolaryngology, Dentist, Dermatology, Physiotherapy, Orthopaedic, Radiology, Paediatrics, O&G, Int. med., Gen. surg., Urology, Pharm., Lab., Ophthalmology, Diabetes, Nutrition, Optometry	Enugu, Enugu State	Aninri	Enugu

1769	EN/0057	EN/0057/S/5	Parklane Specialist Hospital	Otolaryngology, Dentist, Dermatology, Physiotherapy, Orthopaedic, Radiology, Paediatrics, O&G, Int. med., Gen. surg., Urology, Pharm., Lab., Ophthalmology, Diabetes, Nutrition, Optometry	Enugu, Enugu State	Aninri	Enugu
1770	EN/0057	EN/0057/S/15	Parklane Specialist Hospital	Otolaryngology, Dentist, Dermatology, Physiotherapy, Orthopaedic, Radiology, Paediatrics, O&G, Int. med., Gen. surg., Urology, Pharm., Lab., Ophthalmology, Diabetes, Nutrition, Optometry	Enugu, Enugu State	Aninri	Enugu
1771	EN/0057	EN/0057/S/8	Parklane Specialist Hospital	Otolaryngology, Dentist, Dermatology, Physiotherapy, Orthopaedic, Radiology, Paediatrics, O&G, Int. med., Gen. surg., Urology, Pharm., Lab., Ophthalmology, Diabetes, Nutrition, Optometry	Enugu, Enugu State	Aninri	Enugu
1772	EN/0057	EN/0057/S/13	Parklane Specialist Hospital	Otolaryngology, Dentist, Dermatology, Physiotherapy, Orthopaedic, Radiology, Paediatrics, O&G, Int. med., Gen. surg., Urology, Pharm., Lab., Ophthalmology, Diabetes, Nutrition, Optometry	Enugu, Enugu State	Aninri	Enugu
1773	EN/0057	EN/0057/S	Parklane Specialist Hospital	Otolaryngology, Dentist, Dermatology, Physiotherapy, Orthopaedic, Radiology, Paediatrics, O&G, Int. med., Gen. surg., Urology, Pharm., Lab., Ophthalmology, Diabetes, Nutrition, Optometry	Enugu, Enugu State	Aninri	Enugu
1774	EN/0068	EN/0068/S/3	Hanlett Patterson Medical Clinic	O & G	West Road, 6 Trans-Ekulu Road Enugu, Enugu State	Aninri	Enugu
1775	EN/0073	EN/0073/S/5	National Orthopaedic Hospital	Orthopaedics, Burns & Plastics, Physiotherapy, Radiology,Laboratory, Pharmacy, Traumatology, Surgery, Prosthetics/orthortics	Enugu	Enugu East	Enugu
1776	EN/0078	EN/0078/S/4	IVY Pharm.	Pharmacy	NAGPP, 44, Chime Road	Aninri	Enugu
1777	EN/0079	EN/0079/S/4	Daisy Pharmacy Ltd.	Pharmacy	49, Zik Avenue, Enugu	Aninri	Enugu

1778	EN/0081	EN/0081/S/4	Ndu Pharmacy/Dunix Ltd.	Pharmacy	147, Zik Avenue, Uwani, Enugu	Enugu South	Enugu
1779	EN/0084	EN/0084/S	KOV Pharmacy	Pharmacy	57, Nike Road, Abakpa, Enugu	Enugu East	Enugu
1780	EN/0085	EN/0085/S/4	Elofex Pharmacy	Pharmacy	Tracas Road, Nsukka, Enugu State	Abeokuta South	Ogun
1781	EN/0092	EN/0092/S/4	Annox Nig. Ltd. Pharm. & Stores	Pharmacy	1B UNTH Road, Enugu	Enugu North	Enugu
1782	EN/0099	EN/0099/S/5	St. Lukkis Lab.	Laboratory	79 Nike Road, Nike Abakpa, Enugu	Enugu East	Enugu
1783	EN/0100	EN/0100/S	Peenok Med. Centre	Laboratory	24, Zik Ave. Uwani Enugu	Enugu South	Enugu
1784	EN/0102	EN/0102/S/12	University Of Nigeria Teaching Hosp., Enugu		Enugu	Enugu North	Enugu
1785	EN/0102	EN/0102/S/3	University Of Nigeria Teaching Hosp., Enugu		Enugu	Enugu North	Enugu
1786	EN/0102	EN/0102/S/2	University Of Nigeria Teaching Hosp., Enugu		Enugu	Enugu North	Enugu
1787	EN/0102	EN/0102/S/15	University Of Nigeria Teaching Hosp., Enugu		Enugu	Enugu North	Enugu
1788	EN/0102	EN/0102/S/14	University Of Nigeria Teaching Hosp., Enugu		Enugu	Enugu North	Enugu
1789	EN/0102	EN/0102/S/8	University Of Nigeria Teaching Hosp., Enugu		Enugu	Enugu North	Enugu
1790	EN/0102	EN/0102/S/11	University Of Nigeria Teaching Hosp., Enugu		Enugu	Enugu North	Enugu
1791	EN/0102	EN/0102/S/10	University Of Nigeria Teaching Hosp., Enugu		Enugu	Enugu North	Enugu
1792	EN/0102	EN/0102/S/4	University Of Nigeria Teaching Hosp., Enugu		Enugu	Enugu North	Enugu
1793	EN/0102	EN/0102/S/5	University Of Nigeria Teaching Hosp., Enugu		Enugu	Enugu North	Enugu
1794	EN/0111	EN/0111/S/2	Cottage Hospital/Comprehensive Health Centre, Police Detecti		Police Detective College, Enugu	Enugu North	Enugu
1795	EN/0111	EN/0111/S/5	Cottage Hospital/Comprehensive Health Centre, Police Detecti		Police Detective College, Enugu	Enugu North	Enugu
1796	EN/0113	EN/0113/S/4	National Orthopaedic Hospital		Enugu, Enugu State	Enugu East	Enugu
1797	EN/0113	EN/0113/S/10	National Orthopaedic Hospital		Enugu, Enugu State	Enugu East	Enugu
1798	EN/0113	EN/0113/S/1	National Orthopaedic Hospital		Enugu, Enugu State	Enugu East	Enugu
1799	EN/0113	EN/0113/S/11	National Orthopaedic Hospital		Enugu, Enugu State	Enugu East	Enugu
1800	EN/0113	EN/0113/S/7	National Orthopaedic Hospital		Enugu, Enugu State	Enugu East	Enugu
1801	EN/0116	EN/0116/S/4	UNN Medical Centre, Enugu		UNEC, Enugu Campus	Enugu North	Enugu
1802	EN/0117	EN/0117/S/4	Nigeria Law School Medical Centre		Agani Town, Enugu	Nkanu West	Enugu
1803	EN/0118	EN/0118/S/14	All Saints Medical Centre		7 Edemani Road, Nsukka	Nsukka	Enugu
1804	EN/0118	EN/0118/S/1	All Saints Medical Centre		7 Edemani Road, Nsukka	Nsukka	Enugu
1805	EN/0118	EN/0118/S/5	All Saints Medical Centre		7 Edemani Road, Nsukka	Nsukka	Enugu
1806	EN/0119	EN/0119/S/4	University of Nigeria, Nsukka Medical Centre		Nsukka, Enugu State	Nsukka	Enugu
1807	EN/0119	EN/0119/S/2	University of Nigeria, Nsukka Medical Centre		Nsukka, Enugu State	Nsukka	Enugu
1808	EN/0119	EN/0119/S/14	University of Nigeria, Nsukka Medical Centre		Nsukka, Enugu State	Nsukka	Enugu
1809	EN/0119	EN/0119/S/3	University of Nigeria, Nsukka Medical Centre		Nsukka, Enugu State	Nsukka	Enugu
1810	EN/0119	EN/0119/S/5	University of Nigeria, Nsukka Medical Centre		Nsukka, Enugu State	Nsukka	Enugu

1811	EN/0122	EN/0122/S/5	Edn Diagnostic Laboratory	Laboratory	135 Agbani Road, Uwani, Enugu	Enugu South	Enugu
1812	EN/0126	EN/0126/S/7	UNN Medical Centre	Radiodiagnostic/Ultrasonography,Physiotherapy,Internal Medicine,O&G,Laboratory,Pharmacy	Nsukka	Nsukka	Enugu
1813	EN/0126	EN/0126/S/11	UNN Medical Centre	Radiodiagnostic/Ultrasonography,Physiotherapy,Internal Medicine,O&G,Laboratory,Pharmacy	Nsukka	Nsukka	Enugu
1814	EN/0126	EN/0126/S/5	UNN Medical Centre	Radiodiagnostic/Ultrasonography,Physiotherapy,Internal Medicine,O&G,Laboratory,Pharmacy	Nsukka	Nsukka	Enugu
1815	EN/0126	EN/0126/S/4	UNN Medical Centre	Radiodiagnostic/Ultrasonography,Physiotherapy,Internal Medicine,O&G,Laboratory,Pharmacy	Nsukka	Nsukka	Enugu
1816	EN/0126	EN/0126/S/3	UNN Medical Centre	Radiodiagnostic/Ultrasonography,Physiotherapy,Internal Medicine,O&G,Laboratory,Pharmacy	Nsukka	Nsukka	Enugu
1817	EN/0126	EN/0126/S/2	UNN Medical Centre	Radiodiagnostic/Ultrasonography,Physiotherapy,Internal Medicine,O&G,Laboratory,Pharmacy	Nsukka	Nsukka	Enugu
1818	EN/0127	EN/0127/S/4	Braunx Pharmaceuticals	Pharmacy	No 138 Upper Chime Ave New Haven Enugu	Enugu South	Enugu
1819	EN/0128	EN/0128/S/4	Deco Pharmacy Ltd	Pharmacy	No 29 Edinburgh Rd Ogui New Layout Enugu	Enugu South	Enugu
1820	EN/0131	EN/0131/S/4	Afrique Concept Ltd Pharmaceuticals	Pharmacy	No 26 Okpara Ave Enugu	Enugu North	Enugu
1821	EN/0132	EN/0132/S/4	Royale Pharmacy	Pharmacy	S1/S2 Dhamija Road Trans Ekulu Enugu	Enugu East	Enugu
1822	EN/0133	EN/0133/S/4	Uncle Ben Pharmacy Ltd	Pharmacy	No 55 Amuri Road Abakpa Enugu	Enugu East	Enugu
1823	EN/0137	EN/0137/S/4	Gredel Pharmacy	Pharmacy	No 28 UNTH Road Enugu	Enugu North	Enugu
1824	EN/0141	EN/0141/S/7	All Saints Medical Centre	Radio-Diagnostic/Ultrasonograph,General Surgery,Laboratory	2 Ogurugu Road, Odenigbo, Nsukka	Nsukka	Enugu
1825	EN/0141	EN/0141/S/1	All Saints Medical Centre	Radio-Diagnostic/Ultrasonograph,General Surgery,Laboratory	2 Ogurugu Road, Odenigbo, Nsukka	Nsukka	Enugu
1826	EN/0143	EN/0143/S/1	Trans Ekulu Hospital	General Surgery	No 21 Nome drive Trans Ekulu	Enugu East	Enugu
1827	EN/0148	EN/0148/S/4	Modern Pharmacy	Pharmacy	No 1 Nome Drive Trans Ekulu Enugu	Enugu East	Enugu
1828	EN/0150	EN/0150/S/5	St Patricks Hospital	Paediatrics,Laboratory,Pharmacy,Internal Medicine	8 Owerri Road, Asata, Enugu	Enugu South	Enugu
1829	EN/0150	EN/0150/S/2	St Patricks Hospital	Paediatrics,Laboratory,Pharmacy,Internal Medicine	8 Owerri Road, Asata, Enugu	Enugu South	Enugu
1830	EN/0150	EN/0150/S/4	St Patricks Hospital	Paediatrics,Laboratory,Pharmacy,Internal Medicine	8 Owerri Road, Asata, Enugu	Enugu South	Enugu
1831	EN/0150	EN/0150/S/6	St Patricks Hospital	Paediatrics,Laboratory,Pharmacy,Internal Medicine	8 Owerri Road, Asata, Enugu	Enugu South	Enugu
1832	EN/0155	EN/0155/S/5	EM Medical Lab. Services	Laboratory	135, Agbani Road, Enugu State	Enugu North	Enugu

1833	EN/0159	EN/0159/S/4	Godini Pharmacy	Pharmacy	No. 2B UNTH Road, Enugu	Enugu North	Enugu
1834	EN/0160	EN/0160/S/7	Niger Foundation Hospital	Laboratory, Pharmacy, Radiodiagnostic/Ultrasonography	off Presidential Road, Enugu State	Enugu East	Enugu
1835	EN/0160	EN/0160/S/4	Niger Foundation Hospital	Laboratory, Pharmacy, Radiodiagnostic/Ultrasonography	off Presidential Road, Enugu State	Enugu East	Enugu
1836	EN/0160	EN/0160/S/5	Niger Foundation Hospital	Laboratory, Pharmacy, Radiodiagnostic/Ultrasonography	off Presidential Road, Enugu State	Enugu East	Enugu
1837	EN/0161	EN/0161/S/4	Goldern Pharmacy	Pharmacy	31 Nike Road, Abakpa, Enugu	Enugu East	Enugu
1838	EN/0162	EN/0162/S/4	Klinimed Pharmaceuticals	Pharmacy	14 College Road, Abakpa, Enugu	Enugu East	Enugu
1839	EN/0164	EN/0164/S/6	Mother of Christ Specialist Hospital		Ogui, Enugu State	Enugu North	Enugu
1840	EN/0164	EN/0164/S/7	Mother of Christ Specialist Hospital		Ogui, Enugu State	Enugu North	Enugu
1841	EN/0164	EN/0164/S/2	Mother of Christ Specialist Hospital		Ogui, Enugu State	Enugu North	Enugu
1842	EN/0164	EN/0164/S/5	Mother of Christ Specialist Hospital		Ogui, Enugu State	Enugu North	Enugu
1843	EN/0164	EN/0164/S/1	Mother of Christ Specialist Hospital		Ogui, Enugu State	Enugu North	Enugu
1844	EN/0164	EN/0164/S/3	Mother of Christ Specialist Hospital		Ogui, Enugu State	Enugu North	Enugu
1845	EN/0171	EN/0171/S/4	Federal College of Education, Eha-Amufu		Eha-Amufu, Enugu State	Enugu North	Enugu
1846	EN/0175	EN/0175/S/1	THE GOOD SHEPHERD SPECIALIST HOSPITAL		CHRIST CHURCH STREET, UWANI	Enugu South	Enugu
1847	EN/0175	EN/0175/S/2	THE GOOD SHEPHERD SPECIALIST HOSPITAL		CHRIST CHURCH STREET, UWANI	Enugu South	Enugu
1848	EN/0175	EN/0175/S/3	THE GOOD SHEPHERD SPECIALIST HOSPITAL		CHRIST CHURCH STREET, UWANI	Enugu South	Enugu
1849	EN/0175	EN/0175/S/4	THE GOOD SHEPHERD SPECIALIST HOSPITAL		CHRIST CHURCH STREET, UWANI	Enugu South	Enugu
1850	EN/0175	EN/0175/S/6	THE GOOD SHEPHERD SPECIALIST HOSPITAL		CHRIST CHURCH STREET, UWANI	Enugu South	Enugu
1851	EN/0175	EN/0175/S/5	THE GOOD SHEPHERD SPECIALIST HOSPITAL		CHRIST CHURCH STREET, UWANI	Enugu South	Enugu
1852	EN/0190	EN/0190/S/7	Nigerian Railway Clinic. Enugu		Railway Compound, Western Avenue, Enugu	Enugu North	Enugu
1853	EN/0192	EN/0192/S/8	Samaks Maxilofacial & Dental Clinic	Dental	77 Zik Avenue, Uwani, Enugu	Enugu North	Enugu
1854	EN/0196	EN/0196/S/4	Adonai Stelmon Medical Laboratory	Pharmacy	63 Chime Avenue, New Haven Enugu	Enugu South	Enugu
1855	EN/0198	EN/0198/S/3	Semino Clinic	O&G, Laboratory	8 Onuayo Street Enugu	Enugu East	Enugu
1856	EN/0198	EN/0198/S/5	Semino Clinic	O&G, Laboratory	8 Onuayo Street Enugu	Enugu East	Enugu
1857	EN/0201	EN/0201/S/3	Chigozie Hospital & Maternity		11 Ekwulu Lane, Emene	Enugu South	Enugu
1858	EN/0208	EN/0208/S/6	Chidike Children Specialist Clinic		57 Nike Abakpa, Nike, Enugu	Enugu East	Enugu
1859	EN/0210	EN/0210/S/8	First Dental Consultant	Dental	184 Ogui Road, Lagos Street, Asata, Enugu	Enugu North	Enugu
1860	EN/0215	EN/0215/S/8	Dentaserve Dental Clinic	Dental	20 Ekulu Avenue, GRA, Enugu	Enugu North	Enugu
1861	EN/0216	EN/0216/S/15	St Mary's Hospital and Maternity		16 Nwodo Close, GRA, Enugu	Enugu North	Enugu
1862	EN/0216	EN/0216/S/6	St Mary's Hospital and Maternity		16 Nwodo Close, GRA, Enugu	Enugu North	Enugu
1863	EN/0216	EN/0216/S/5	St Mary's Hospital and Maternity		16 Nwodo Close, GRA, Enugu	Enugu North	Enugu
1864	EN/0216	EN/0216/S/3	St Mary's Hospital and Maternity		16 Nwodo Close, GRA, Enugu	Enugu North	Enugu
1865	EN/0216	EN/0216/S/2	St Mary's Hospital and Maternity		16 Nwodo Close, GRA, Enugu	Enugu North	Enugu
1866	EN/0216	EN/0216/S/1	St Mary's Hospital and Maternity		16 Nwodo Close, GRA, Enugu	Enugu North	Enugu

1867	EN/0217	EN/0217/S/4	Bishop Shanahan Hospital		46 Enugu Road, Nsukka	Nsukka	Enugu
1868	EN/0217	EN/0217/S/5	Bishop Shanahan Hospital		46 Enugu Road, Nsukka	Nsukka	Enugu
1869	EN/0217	EN/0217/S/1	Bishop Shanahan Hospital		46 Enugu Road, Nsukka	Nsukka	Enugu
1870	EN/0217	EN/0217/S/2	Bishop Shanahan Hospital		46 Enugu Road, Nsukka	Nsukka	Enugu
1871	EN/0217	EN/0217/S/3	Bishop Shanahan Hospital		46 Enugu Road, Nsukka	Nsukka	Enugu
1872	EN/0218	EN/0218/S/9	Federal Neuropsychiatric Hospital		Chime Avenue, New Haven, PMB 01181, Enugu	Enugu North	Enugu
1873	EN/0218	EN/0218/S/4	Federal Neuropsychiatric Hospital		Chime Avenue, New Haven, PMB 01181, Enugu	Enugu North	Enugu
1874	EN/0218	EN/0218/S/5	Federal Neuropsychiatric Hospital		Chime Avenue, New Haven, PMB 01181, Enugu	Enugu North	Enugu
1875	EN/0223	EN/0223/S/7	Trisa Clinic and Radiological Services		C4 presidential road (former Golden Gate Independence layout Enugu.	Enugu South	Enugu
1876	EN/0224	EN/0224/S/7	San Bernadine Clinic		10,Carter Street Ogue,Enugu State	Enugu North	Enugu
1877	EN/0227	EN/0227/S/5	Lamb Of God Medical Lab		191,Chime Avenue, Enugu State	Enugu North	Enugu
1878	EN/0228	EN/0228/S/18	Alpha Specialist Hospital		NO.6/8 Third Avenue Trans-Ekulu,Enugu State	Enugu East	Enugu
1879	EN/0229	EN/0229/S/15	Enugu Eye Clinics		1,Anyaegbunam Street Uwani,Enugu State	Enugu South	Enugu
1880	EN/0231	EN/0231/S/5	Faith Foundation Hospital		27B,Ogurugo Road Nsukka,Enugu State	Nsukka	Enugu
1881	EN/0231	EN/0231/S/4	Faith Foundation Hospital		27B,Ogurugo Road Nsukka,Enugu State	Nsukka	Enugu
1882	EN/0232	EN/0232/S/10	City Clinics		10,Enweani Street,Opp New Haven Junction By Abakaliki Express Way, City Layout, New Haven, Enugu State	Enugu East	Enugu
1883	EN/0233	EN/0233/S/6	Prime Life Medical		30A,Emeka Ebilla Lane Idaw River, Enugu State	Enugu South	Enugu
1884	EN/0234	EN/0234/S/4	Mark Twain Nig.Ltd		1,Unth Road, Enugu State	Enugu North	Enugu
1885	EN/0238	EN/0238/S/6	Tendercare Hospital & Maternity		NO.5B Ezeji Close Ugboezechi,Abakpa,Enugu State	Enugu East	Enugu
1886	EN/0239	EN/0239/S/13	Springfield Eyecare Centre		200 Agbani Road,Enugu State	Enugu South	Enugu
1887	EN/0240	EN/0240/S/15	The Eye Specialist Hospital		11,Church View Ekulu West GRA,Enugu state	Enugu East	Enugu
1888	EN/0241	EN/0241/S/5	Noble Laboratory Services		6,Oceanic Avenue Off Presedential Road,Nkpor,Enugu State	Enugu South	Enugu
1889	EN/0242	EN/0242/S/3	Jideofor Hospital		Ikpenwa Flat N.A. Quarters Agwu,Enugu State	Awgu	Enugu
1890	EN/0242	EN/0242/S/1	Jideofor Hospital		Ikpenwa Flat N.A. Quarters Agwu,Enugu State	Awgu	Enugu
1891	EN/0243	EN/0243/S/4	Renhoks Pharmaceuticals		83,Park Avenue GRA, Enugu State	Enugu North	Enugu
1892	EN/0244	EN/0244/S/5	Enugu State Uni Of Science &Tech (ESUT) Medical Centre		Agbani, Enugu State	Nkanu West	Enugu
1893	EN/0245	EN/0245/S/4	Laddan Pharmacy		15/19 Atani Street Abakpa Enugu State	Enugu East	Enugu
1894	EN/0248	EN/0248/S/5	Calix Healthcare Ltd		10A Hill View Road, ESBS,	Enugu North	Enugu
1895	EN/0248	EN/0248/S/4	Calix Healthcare Ltd		10A Hill View Road, ESBS,	Enugu North	Enugu
1896	EN/0248	EN/0248/S/6	Calix Healthcare Ltd		10A Hill View Road, ESBS,	Enugu North	Enugu
1897	EN/0249	EN/0249/S/15	The Eye Specialist Hospital		282 Enugu Road, Ugwunkwo, Nsukka Enugu State	Nsukka	Enugu
1898	EN/0250	EN/0250/S/3	Freedom Fertility Centre		No 6 Hill View Avenue, Independent Layout, Enugu, Enugu State	Enugu North	Enugu
1899	EN/0250	EN/0250/S/14	Freedom Fertility Centre		No 6 Hill View Avenue, Independent Layout, Enugu, Enugu State	Enugu North	Enugu
1900	EN/0250	EN/0250/S/6	Freedom Fertility Centre		No 6 Hill View Avenue, Independent Layout, Enugu, Enugu State	Enugu North	Enugu
1901	EN/0250	EN/0250/S/5	Freedom Fertility Centre		No 6 Hill View Avenue, Independent Layout, Enugu, Enugu State	Enugu North	Enugu

1902	EN/0250	EN/0250/S/4	Freedom Fertility Centre		No 6 Hill View Avenue, Independent Layout, Enugu, Enugu State	Enugu North	Enugu
1903	EN/0250	EN/0250/S/2	Freedom Fertility Centre		No 6 Hill View Avenue, Independent Layout, Enugu, Enugu State	Enugu North	Enugu
1904	EN/0250	EN/0250/S/1	Freedom Fertility Centre		No 6 Hill View Avenue, Independent Layout, Enugu, Enugu State	Enugu North	Enugu
1905	EN/0251	EN/0251/S/6	Freedom Fertility Centre		No 6 Hill View Avenue, Independent Layout, Enugu, Enugu State	Enugu North	Enugu
1906	EN/0251	EN/0251/S/4	Freedom Fertility Centre		No 6 Hill View Avenue, Independent Layout, Enugu, Enugu State	Enugu North	Enugu
1907	EN/0251	EN/0251/S/2	Freedom Fertility Centre		No 6 Hill View Avenue, Independent Layout, Enugu, Enugu State	Enugu North	Enugu
1908	EN/0251	EN/0251/S/1	Freedom Fertility Centre		No 6 Hill View Avenue, Independent Layout, Enugu, Enugu State	Enugu North	Enugu
1909	EN/0251	EN/0251/S/14	Freedom Fertility Centre		No 6 Hill View Avenue, Independent Layout, Enugu, Enugu State	Enugu North	Enugu
1910	EN/0251	EN/0251/S/5	Freedom Fertility Centre		No 6 Hill View Avenue, Independent Layout, Enugu, Enugu State	Enugu North	Enugu
1911	EN/0251	EN/0251/S/3	Freedom Fertility Centre		No 6 Hill View Avenue, Independent Layout, Enugu, Enugu State	Enugu North	Enugu
1912	EN/0254	EN/0254/S/3	Christian Miracle Centre		3 Akutu Crescent, , Independent Lay Out, Enugu State	Enugu North	Enugu
1913	EN/0256	EN/0256/S/7	Choice Medical Centre		1C Monrovia Street, New Heaven, Enugu State	Enugu North	Enugu
1914	EN/0256	EN/0256/S/3	Choice Medical Centre		1C Monrovia Street, New Heaven, Enugu State	Enugu North	Enugu
1915	EN/0261	EN/0261/S/8	Kappa Dental Services		6B Amawbia Close, New Heaven, Enugu, Enugu State	Enugu North	Enugu
1916	EN/0263	EN/0263/S/7	Champion Diagnostics		85 Nike Road, By Liberty Bus Stop Abakpa, Enugu State	Awgu	Enugu
1917	EN/0264	EN/0264/S/6	Favoured Child Clinics		4 Jerry-Ugwu, Avenue, New Heaven Extension, Enugu State	Enugu North	Enugu
1918	EN/0265	EN/0265/S/5	Mc Chucks Laboratory Services		2 Ohafia/Nnaji Street, By Zik Avenue, Enugu, Enugu State	Enugu South	Enugu
1919	FCT/0002	FCT/0002/S/5	Arewa Specialist Hospital & Diagnostic Centre		Plot 645, Alex Ekwueme Street, Jabi District, Abuja	Municipal	FCT
1920	FCT/0002	FCT/0002/S/3	Arewa Specialist Hospital & Diagnostic Centre		Plot 645, Alex Ekwueme Street, Jabi District, Abuja	Municipal	FCT
1921	FCT/0002	FCT/0002/S/6	Arewa Specialist Hospital & Diagnostic Centre		Plot 645, Alex Ekwueme Street, Jabi District, Abuja	Municipal	FCT
1922	FCT/0002	FCT/0002/S/2	Arewa Specialist Hospital & Diagnostic Centre		Plot 645, Alex Ekwueme Street, Jabi District, Abuja	Municipal	FCT
1923	FCT/0002	FCT/0002/S/1	Arewa Specialist Hospital & Diagnostic Centre		Plot 645, Alex Ekwueme Street, Jabi District, Abuja	Municipal	FCT
1924	FCT/0004	FCT/0004/S/2	Gwarinpa General Hospital		Gwarinpa,Abuja	Municipal	FCT
1925	FCT/0004	FCT/0004/S/6	Gwarinpa General Hospital		Gwarinpa,Abuja	Municipal	FCT
1926	FCT/0004	FCT/0004/S/5	Gwarinpa General Hospital		Gwarinpa,Abuja	Municipal	FCT
1927	FCT/0004	FCT/0004/S/3	Gwarinpa General Hospital		Gwarinpa,Abuja	Municipal	FCT
1928	FCT/0004	FCT/0004/S/4	Gwarinpa General Hospital		Gwarinpa,Abuja	Municipal	FCT
1929	FCT/0005	FCT/0005/S/1	Sybron Medical Centre		25, Mungo Park close, opp Havista Hotel, Asokoro, Abuja	Municipal	FCT
1930	FCT/0005	FCT/0005/S/5	Sybron Medical Centre		25, Mungo Park close, opp Havista Hotel, Asokoro, Abuja	Municipal	FCT
1931	FCT/0005	FCT/0005/S/3	Sybron Medical Centre		25, Mungo Park close, opp Havista Hotel, Asokoro, Abuja	Municipal	FCT
1932	FCT/0008	FCT/0008/S/1	Pan-Raf Hospital Ltd.		Nyanya Area C, Abuja	Municipal	FCT
1933	FCT/0008	FCT/0008/S/3	Pan-Raf Hospital Ltd.		Nyanya Area C, Abuja	Municipal	FCT
1934	FCT/0008	FCT/0008/S/5	Pan-Raf Hospital Ltd.		Nyanya Area C, Abuja	Municipal	FCT

1935	FCT/0008	FCT/0008/S/6	Pan-Raf Hospital Ltd.		Nyanya Area C, Abuja	Municipal	FCT
1936	FCT/0008	FCT/0008/S/8	Pan-Raf Hospital Ltd.		Nyanya Area C, Abuja	Municipal	FCT
1937	FCT/0008	FCT/0008/S/13	Pan-Raf Hospital Ltd.		Nyanya Area C, Abuja	Municipal	FCT
1938	FCT/0008	FCT/0008/S/15	Pan-Raf Hospital Ltd.		Nyanya Area C, Abuja	Municipal	FCT
1939	FCT/0008	FCT/0008/S/10	Pan-Raf Hospital Ltd.		Nyanya Area C, Abuja	Municipal	FCT
1940	FCT/0008	FCT/0008/S/2	Pan-Raf Hospital Ltd.		Nyanya Area C, Abuja	Municipal	FCT
1941	FCT/0014	FCT/0014/S/5	Primary Health Clinic, Karonmagaji		Karonmajigi, Abuja	Municipal	FCT
1942	FCT/0017	FCT/0017/S/14	The Comforter Clinic & Diag. Centre		Plot 1233, 1 (R) 4 Road by AMAC International Market behind CBN Quarters Lugbe FHA	Municipal	FCT
1943	FCT/0018	FCT/0018/S/4	Al Fama Pharmacy	Pharmacy	New FCDA Life Camp, Gwaprinpa, Abuja	Municipal	FCT
1944	FCT/0020	FCT/0020/S/4	Nolad Pharm. & Allied Co. Ltd.	Pharmacy	Plot 278 Aminu Kano Crescent (Opposite Mr. Biggs) Wuse II, Abuja	Municipal	FCT
1945	FCT/0022	FCT/0022/S/4	Richneworld Pharmacy Ltd.	Pharmacy	2nd Avenue, Off Foreign Affairs Qtrs, Gwarinpa	Municipal	FCT
1946	FCT/0024	FCT/0024/S/5	Alpha-Royal Medical Ltd.	Laboratory	Suite A Mobil Filling Station complex, Karu Site	Municipal	FCT
1947	FCT/0028	FCT/0028/S/5	Goodman Medical Diag. Lab.	Laboratory	7 Cornershop, Area A, Nyanya	Municipal	FCT
1948	FCT/0030	FCT/0030/S/4	Boniks pharmacy	Pharmacy	Shop 5, Gwarinpa Estate, By FHA Home Office, 3rd Avenue	Municipal	FCT
1949	FCT/0031	FCT/0031/S/4	Hephzibah Pharmacy Nig. Ltd.	Pharmacy	Shop 3&4, Goodson Plaza, Behind Tony Anthony Lane, Gwarinpa	Municipal	FCT
1950	FCT/0040	FCT/0040/S/1	Kings Care Hospital Ltd - Kubwa		7, Christmas Road, Behind Police Affairs, Kubwa Abuja	Bwari	FCT
1951	FCT/0040	FCT/0040/S/5	Kings Care Hospital Ltd - Kubwa		7, Christmas Road, Behind Police Affairs, Kubwa Abuja	Bwari	FCT
1952	FCT/0040	FCT/0040/S/3	Kings Care Hospital Ltd - Kubwa		7, Christmas Road, Behind Police Affairs, Kubwa Abuja	Bwari	FCT
1953	FCT/0044	FCT/0044/S/5	Pison Hospital		3553 Gado Nasko Road by MTN office, Phase lV, Kubwa	Municipal	FCT
1954	FCT/0044	FCT/0044/S/7	Pison Hospital		3553 Gado Nasko Road by MTN office, Phase lV, Kubwa	Municipal	FCT
1955	FCT/0044	FCT/0044/S/3	Pison Hospital		3553 Gado Nasko Road by MTN office, Phase lV, Kubwa	Municipal	FCT
1956	FCT/0046	FCT/0046/S/1	Grace Of God Specialist Med. Centre		Phrase 2, Site 2, Kubwa, Abuja	Bwari	FCT
1957	FCT/0046	FCT/0046/S/5	Grace Of God Specialist Med. Centre		Phrase 2, Site 2, Kubwa, Abuja	Bwari	FCT
1958	FCT/0047	FCT/0047/S/13	Kubwa General Hospital		Phase 4 Opp. Gss, Kubwa, Abuja	Bwari	FCT
1959	FCT/0047	FCT/0047/S/4	Kubwa General Hospital		Phase 4 Opp. Gss, Kubwa, Abuja	Bwari	FCT
1960	FCT/0047	FCT/0047/S/5	Kubwa General Hospital		Phase 4 Opp. Gss, Kubwa, Abuja	Bwari	FCT
1961	FCT/0049	FCT/0049/S/3	St. Vincent Clinic & Maternity		Daughtrer''s Of Charity, Biyazhin Road, Kubwa	Bwari	FCT
1962	FCT/0049	FCT/0049/S/4	St. Vincent Clinic & Maternity		Daughtrer''s Of Charity, Biyazhin Road, Kubwa	Bwari	FCT
1963	FCT/0049	FCT/0049/S/5	St. Vincent Clinic & Maternity		Daughtrer''s Of Charity, Biyazhin Road, Kubwa	Bwari	FCT
1964	FCT/0050	FCT/0050/S/4	Royal Lords Hospital, Clinics & Maternity Ltd		Plot 107, Zone 4, Dutse Alhaji, Abuja	Bwari	FCT
1965	FCT/0050	FCT/0050/S/5	Royal Lords Hospital, Clinics & Maternity Ltd		Plot 107, Zone 4, Dutse Alhaji, Abuja	Bwari	FCT
1966	FCT/0050	FCT/0050/S/14	Royal Lords Hospital, Clinics & Maternity Ltd		Plot 107, Zone 4, Dutse Alhaji, Abuja	Bwari	FCT
1967	FCT/0055	FCT/0055/S/4	Nassara Pharmacy Chem. & Store	Pharmacy	18/19 Sultan Dasuki Way Fha, Kubwa	Bwari	FCT
1968	FCT/0062	FCT/0062/S/5	Kavron Medical Laboratory	Laboratory	Ushafa Road, Bwari	Bwari	FCT
1969	FCT/0065	FCT/0065/S/4	Anes Pharmacy & Stores Ltd.	Pharmacy	34, Shagari Road Opp. Jamb Office, Bwari	Bwari	FCT
1970	FCT/0070	FCT/0070/S/4	Novac Pharmacy	Pharmacy	Southern Dasuki/Gado Nasco, Traffic Junction, Phase 2/2, Kubwa.	Bwari	FCT

1971	FCT/0075	FCT/0075/S/5	Oxmed Laboratory	Laboratory	172 Civil Centre, Gwagwalada	Gwagwalada	FCT
1972	FCT/0078	FCT/0078/S/5	Peak Medical Centre	Laboratory	Gwagwalada	Gwagwalada	FCT
1973	FCT/0079	FCT/0079/S/4	Agestones Pharmaceuticals	Pharmacy	Plot 7, Phase 1 Shopping Centre, Gwagwalada	Gwagwalada	FCT
1974	FCT/0080	FCT/0080/S/1	Acme-Joe Pharmacy	Pharmacy	18 Secretariat Road, Phase 1, Gwagwalada	Gwagwalada	FCT
1975	FCT/0081	FCT/0081/S/6	Skylark Pharmacy	Pharmacy	9 Phase III Shopping complex, Off specialist Hosp., Gwagwalada	Gwagwalada	FCT
1976	FCT/0082	FCT/0082/S/4	Atma Rime Pharm. Nig. Ltd.	Pharmacy	12B, University Road, Phase II, Gwagwalada	Gwagwalada	FCT
1977	FCT/0085	FCT/0085/S/12	Adkan Specialist Clinic	Urology, ENT	Block D, 23 FMW&H Estate, Behind Kontangora Estate, Gwagwalada	Gwagwalada	FCT
1978	FCT/0085	FCT/0085/S/4	Adkan Specialist Clinic	Urology, ENT	Block D, 23 FMW&H Estate, Behind Kontangora Estate, Gwagwalada	Gwagwalada	FCT
1979	FCT/0086	FCT/0086/S/5	Rhema Foundation Hospital		Rhema Fountain, Kwali, Abuja	Kwali	FCT
1980	FCT/0091	FCT/0091/S/3	Maitama District Hospital		Maitama Abuja	Municipal	FCT
1981	FCT/0091	FCT/0091/S/4	Maitama District Hospital		Maitama Abuja	Municipal	FCT
1982	FCT/0091	FCT/0091/S/5	Maitama District Hospital		Maitama Abuja	Municipal	FCT
1983	FCT/0091	FCT/0091/S/2	Maitama District Hospital		Maitama Abuja	Municipal	FCT
1984	FCT/0091	FCT/0091/S/1	Maitama District Hospital		Maitama Abuja	Municipal	FCT
1985	FCT/0092	FCT/0092/S/2	Asokoro District Hospital		Asokoro, Abuja	Municipal	FCT
1986	FCT/0092	FCT/0092/S/5	Asokoro District Hospital		Asokoro, Abuja	Municipal	FCT
1987	FCT/0092	FCT/0092/S/4	Asokoro District Hospital		Asokoro, Abuja	Municipal	FCT
1988	FCT/0092	FCT/0092/S/6	Asokoro District Hospital		Asokoro, Abuja	Municipal	FCT
1989	FCT/0092	FCT/0092/S/3	Asokoro District Hospital		Asokoro, Abuja	Municipal	FCT
1990	FCT/0092	FCT/0092/S/1	Asokoro District Hospital		Asokoro, Abuja	Municipal	FCT
1991	FCT/0092	FCT/0092/S/8	Asokoro District Hospital		Asokoro, Abuja	Municipal	FCT
1992	FCT/0092	FCT/0092/S/11	Asokoro District Hospital		Asokoro, Abuja	Municipal	FCT
1993	FCT/0093	FCT/0093/S/1	Wuse District Hospital		Wuse, Abuja	Municipal	FCT
1994	FCT/0093	FCT/0093/S/2	Wuse District Hospital		Wuse, Abuja	Municipal	FCT
1995	FCT/0093	FCT/0093/S/4	Wuse District Hospital		Wuse, Abuja	Municipal	FCT
1996	FCT/0093	FCT/0093/S/12	Wuse District Hospital		Wuse, Abuja	Municipal	FCT
1997	FCT/0093	FCT/0093/S/11	Wuse District Hospital		Wuse, Abuja	Municipal	FCT
1998	FCT/0093	FCT/0093/S/6	Wuse District Hospital		Wuse, Abuja	Municipal	FCT
1999	FCT/0093	FCT/0093/S/3	Wuse District Hospital		Wuse, Abuja	Municipal	FCT
2000	FCT/0093	FCT/0093/S/5	Wuse District Hospital		Wuse, Abuja	Municipal	FCT
2001	FCT/0095	FCT/0095/S/5	Asher Hospital & Maternity		Plot 1197, Kataamkpe Cadastral Zone B07, Opp NICON Junction, Berger-Kubwa Express way	Municipal	FCT
2002	FCT/0095	FCT/0095/S/11	Asher Hospital & Maternity		Plot 1197, Kataamkpe Cadastral Zone B07, Opp NICON Junction, Berger-Kubwa Express way	Municipal	FCT
2003	FCT/0095	FCT/0095/S/1	Asher Hospital & Maternity		Plot 1197, Kataamkpe Cadastral Zone B07, Opp NICON Junction, Berger-Kubwa Express way	Municipal	FCT
2004	FCT/0095	FCT/0095/S/3	Asher Hospital & Maternity		Plot 1197, Kataamkpe Cadastral Zone B07, Opp NICON Junction, Berger-Kubwa Express way	Municipal	FCT
2005	FCT/0095	FCT/0095/S/7	Asher Hospital & Maternity		Plot 1197, Kataamkpe Cadastral Zone B07, Opp NICON Junction, Berger-Kubwa Express way	Municipal	FCT
2006	FCT/0095	FCT/0095/S/8	Asher Hospital & Maternity		Plot 1197, Kataamkpe Cadastral Zone B07, Opp NICON Junction, Berger-Kubwa Express way	Municipal	FCT

2007	FCT/0095	FCT/0095/S/2	Asher Hospital & Maternity		Plot 1197, Kataamkpe Cadastral Zone B07, Opp NICON Junction, Berger-Kubwa Express way	Municipal	FCT
2008	FCT/0095	FCT/0095/S/15	Asher Hospital & Maternity		Plot 1197, Kataamkpe Cadastral Zone B07, Opp NICON Junction, Berger-Kubwa Express way	Municipal	FCT
2009	FCT/0097	FCT/0097/S/12	Sauki Private Hospital		Plot 90 Zone 6 Yaounde Crescent Wuse	Municipal	FCT
2010	FCT/0097	FCT/0097/S/10	Sauki Private Hospital		Plot 90 Zone 6 Yaounde Crescent Wuse	Municipal	FCT
2011	FCT/0097	FCT/0097/S/6	Sauki Private Hospital		Plot 90 Zone 6 Yaounde Crescent Wuse	Municipal	FCT
2012	FCT/0097	FCT/0097/S/1	Sauki Private Hospital		Plot 90 Zone 6 Yaounde Crescent Wuse	Municipal	FCT
2013	FCT/0097	FCT/0097/S/3	Sauki Private Hospital		Plot 90 Zone 6 Yaounde Crescent Wuse	Municipal	FCT
2014	FCT/0097	FCT/0097/S/14	Sauki Private Hospital		Plot 90 Zone 6 Yaounde Crescent Wuse	Municipal	FCT
2015	FCT/0097	FCT/0097/S/5	Sauki Private Hospital		Plot 90 Zone 6 Yaounde Crescent Wuse	Municipal	FCT
2016	FCT/0097	FCT/0097/S/4	Sauki Private Hospital		Plot 90 Zone 6 Yaounde Crescent Wuse	Municipal	FCT
2017	FCT/0097	FCT/0097/S/11	Sauki Private Hospital		Plot 90 Zone 6 Yaounde Crescent Wuse	Municipal	FCT
2018	FCT/0097	FCT/0097/S/15	Sauki Private Hospital		Plot 90 Zone 6 Yaounde Crescent Wuse	Municipal	FCT
2019	FCT/0097	FCT/0097/S/8	Sauki Private Hospital		Plot 90 Zone 6 Yaounde Crescent Wuse	Municipal	FCT
2020	FCT/0097	FCT/0097/S/7	Sauki Private Hospital		Plot 90 Zone 6 Yaounde Crescent Wuse	Municipal	FCT
2021	FCT/0097	FCT/0097/S/2	Sauki Private Hospital		Plot 90 Zone 6 Yaounde Crescent Wuse	Municipal	FCT
2022	FCT/0099	FCT/0099/S/3	General Hospital, Nyanya		Nyanya, Abuja	Municipal	FCT
2023	FCT/0099	FCT/0099/S/1	General Hospital, Nyanya		Nyanya, Abuja	Municipal	FCT
2024	FCT/0099	FCT/0099/S/6	General Hospital, Nyanya		Nyanya, Abuja	Municipal	FCT
2025	FCT/0099	FCT/0099/S/4	General Hospital, Nyanya		Nyanya, Abuja	Municipal	FCT
2026	FCT/0102	FCT/0102/S/5	Our Lady Of Fatima Catholic Hospital		Ushafa Rd., Bwari, Abuja	Bwari	FCT
2027	FCT/0103	FCT/0103/S/1	Kwali General Hospital		Off Abaji/Lokoja Road, Near Area Council Secretariat, Kwali,	Kwali	FCT
2028	FCT/0103	FCT/0103/S/3	Kwali General Hospital		Off Abaji/Lokoja Road, Near Area Council Secretariat, Kwali,	Kwali	FCT
2029	FCT/0103	FCT/0103/S/5	Kwali General Hospital		Off Abaji/Lokoja Road, Near Area Council Secretariat, Kwali,	Kwali	FCT
2030	FCT/0103	FCT/0103/S/4	Kwali General Hospital		Off Abaji/Lokoja Road, Near Area Council Secretariat, Kwali,	Kwali	FCT
2031	FCT/0105	FCT/0105/S/5	General Hospital - Karshi		Karshi, Abuja	Municipal	FCT
2032	FCT/0105	FCT/0105/S/4	General Hospital - Karshi		Karshi, Abuja	Municipal	FCT
2033	FCT/0105	FCT/0105/S/8	General Hospital - Karshi		Karshi, Abuja	Municipal	FCT
2034	FCT/0105	FCT/0105/S/3	General Hospital - Karshi		Karshi, Abuja	Municipal	FCT
2035	FCT/0106	FCT/0106/S/10	Alliance Hospital and Services Ltd		Plot 801, No. 5 Malumfashi Close off Emeka Anyaoku Street, Garki Area 11	Municipal	FCT
2036	FCT/0106	FCT/0106/S/2	Alliance Hospital and Services Ltd		Plot 801, No. 5 Malumfashi Close off Emeka Anyaoku Street, Garki Area 11	Municipal	FCT
2037	FCT/0106	FCT/0106/S/4	Alliance Hospital and Services Ltd		Plot 801, No. 5 Malumfashi Close off Emeka Anyaoku Street, Garki Area 11	Municipal	FCT
2038	FCT/0106	FCT/0106/S/3	Alliance Hospital and Services Ltd		Plot 801, No. 5 Malumfashi Close off Emeka Anyaoku Street, Garki Area 11	Municipal	FCT
2039	FCT/0106	FCT/0106/S/1	Alliance Hospital and Services Ltd		Plot 801, No. 5 Malumfashi Close off Emeka Anyaoku Street, Garki Area 11	Municipal	FCT
2040	FCT/0106	FCT/0106/S/6	Alliance Hospital and Services Ltd		Plot 801, No. 5 Malumfashi Close off Emeka Anyaoku Street, Garki Area 11	Municipal	FCT

2041	FCT/0106	FCT/0106/S/5	Alliance Hospital and Services Ltd		Plot 801, No. 5 Malumfashi Close off Emeka Anyaoku Street, Garki Area 11	Municipal	FCT
2042	FCT/0107	FCT/0107/S/7	Ruz Medical Centre		Ubiaja Crescent, Garki, Abuja	Municipal	FCT
2043	FCT/0107	FCT/0107/S/5	Ruz Medical Centre		Ubiaja Crescent, Garki, Abuja	Municipal	FCT
2044	FCT/0108	FCT/0108/S/1	Rouz Hospital & Maternity Limited		Apo, Legislative Qtrs, Phase II	Municipal	FCT
2045	FCT/0108	FCT/0108/S/6	Rouz Hospital & Maternity Limited		Apo, Legislative Qtrs, Phase II	Municipal	FCT
2046	FCT/0108	FCT/0108/S/5	Rouz Hospital & Maternity Limited		Apo, Legislative Qtrs, Phase II	Municipal	FCT
2047	FCT/0108	FCT/0108/S/3	Rouz Hospital & Maternity Limited		Apo, Legislative Qtrs, Phase II	Municipal	FCT
2048	FCT/0109	FCT/0109/S/5	Pyramid Medical Centre		Plot 1438 Garki Ii Opp. Nia Cadastral Zone A3 Nnamdi Azikwe	Municipal	FCT
2049	FCT/0110	FCT/0110/S/5	Amana Medical Centre		No. 5, Ilorin Street, off Ogbomosho Street, Area 8, Abuja	Municipal	FCT
2050	FCT/0110	FCT/0110/S/3	Amana Medical Centre		No. 5, Ilorin Street, off Ogbomosho Street, Area 8, Abuja	Municipal	FCT
2051	FCT/0110	FCT/0110/S/6	Amana Medical Centre		No. 5, Ilorin Street, off Ogbomosho Street, Area 8, Abuja	Municipal	FCT
2052	FCT/0110	FCT/0110/S/1	Amana Medical Centre		No. 5, Ilorin Street, off Ogbomosho Street, Area 8, Abuja	Municipal	FCT
2053	FCT/0110	FCT/0110/S/2	Amana Medical Centre		No. 5, Ilorin Street, off Ogbomosho Street, Area 8, Abuja	Municipal	FCT
2054	FCT/0110	FCT/0110/S/4	Amana Medical Centre		No. 5, Ilorin Street, off Ogbomosho Street, Area 8, Abuja	Municipal	FCT
2055	FCT/0111	FCT/0111/S/4	Fereprod. Medical Centre		Off Ahmadu Bello Way Behind Nepa Sub-Station-2 Cbn Area Ii,	Municipal	FCT
2056	FCT/0111	FCT/0111/S/2	Fereprod. Medical Centre		Off Ahmadu Bello Way Behind Nepa Sub-Station-2 Cbn Area Ii,	Municipal	FCT
2057	FCT/0111	FCT/0111/S/5	Fereprod. Medical Centre		Off Ahmadu Bello Way Behind Nepa Sub-Station-2 Cbn Area Ii,	Municipal	FCT
2058	FCT/0111	FCT/0111/S/1	Fereprod. Medical Centre		Off Ahmadu Bello Way Behind Nepa Sub-Station-2 Cbn Area Ii,	Municipal	FCT
2059	FCT/0111	FCT/0111/S/12	Fereprod. Medical Centre		Off Ahmadu Bello Way Behind Nepa Sub-Station-2 Cbn Area Ii,	Municipal	FCT
2060	FCT/0111	FCT/0111/S/10	Fereprod. Medical Centre		Off Ahmadu Bello Way Behind Nepa Sub-Station-2 Cbn Area Ii,	Municipal	FCT
2061	FCT/0111	FCT/0111/S/3	Fereprod. Medical Centre		Off Ahmadu Bello Way Behind Nepa Sub-Station-2 Cbn Area Ii,	Municipal	FCT
2062	FCT/0111	FCT/0111/S/6	Fereprod. Medical Centre		Off Ahmadu Bello Way Behind Nepa Sub-Station-2 Cbn Area Ii,	Municipal	FCT
2063	FCT/0115	FCT/0115/S/5	Jilf Clinic Ent.		Plot D 231 Crescent By 232 Road Kado Estate	Municipal	FCT
2064	FCT/0120	FCT/0120/S/3	Limi Hospital & Mat., Ltd.		Plot 541, Behind Icpc, Central Area, Abuja	Municipal	FCT
2065	FCT/0120	FCT/0120/S/1	Limi Hospital & Mat., Ltd.		Plot 541, Behind Icpc, Central Area, Abuja	Municipal	FCT
2066	FCT/0120	FCT/0120/S/7	Limi Hospital & Mat., Ltd.		Plot 541, Behind Icpc, Central Area, Abuja	Municipal	FCT
2067	FCT/0120	FCT/0120/S/6	Limi Hospital & Mat., Ltd.		Plot 541, Behind Icpc, Central Area, Abuja	Municipal	FCT
2068	FCT/0120	FCT/0120/S/5	Limi Hospital & Mat., Ltd.		Plot 541, Behind Icpc, Central Area, Abuja	Municipal	FCT
2069	FCT/0120	FCT/0120/S/8	Limi Hospital & Mat., Ltd.		Plot 541, Behind Icpc, Central Area, Abuja	Municipal	FCT
2070	FCT/0120	FCT/0120/S/4	Limi Hospital & Mat., Ltd.		Plot 541, Behind Icpc, Central Area, Abuja	Municipal	FCT
2071	FCT/0120	FCT/0120/S/2	Limi Hospital & Mat., Ltd.		Plot 541, Behind Icpc, Central Area, Abuja	Municipal	FCT
2072	FCT/0122	FCT/0122/S/4	National Assembly Clinic		Three Arm Zone, Central Area, Abuja	Municipal	FCT
2073	FCT/0123	FCT/0123/S/1	State House Clinic		(Presidency) Three Arm Zone, Central Area, Abuja	Municipal	FCT
2074	FCT/0123	FCT/0123/S/5	State House Clinic		(Presidency) Three Arm Zone, Central Area, Abuja	Municipal	FCT
2075	FCT/0123	FCT/0123/S/4	State House Clinic		(Presidency) Three Arm Zone, Central Area, Abuja	Municipal	FCT

2076	FCT/0123	FCT/0123/S/6	State House Clinic		(Presidency) Three Arm Zone, Central Area, Abuja	Municipal	FCT
2077	FCT/0123	FCT/0123/S/11	State House Clinic		(Presidency) Three Arm Zone, Central Area, Abuja	Municipal	FCT
2078	FCT/0123	FCT/0123/S/15	State House Clinic		(Presidency) Three Arm Zone, Central Area, Abuja	Municipal	FCT
2079	FCT/0123	FCT/0123/S/2	State House Clinic		(Presidency) Three Arm Zone, Central Area, Abuja	Municipal	FCT
2080	FCT/0123	FCT/0123/S/8	State House Clinic		(Presidency) Three Arm Zone, Central Area, Abuja	Municipal	FCT
2081	FCT/0123	FCT/0123/S/3	State House Clinic		(Presidency) Three Arm Zone, Central Area, Abuja	Municipal	FCT
2082	FCT/0123	FCT/0123/S/7	State House Clinic		(Presidency) Three Arm Zone, Central Area, Abuja	Municipal	FCT
2083	FCT/0127	FCT/0127/S/1	Sami-Wadata Clinics Ltd.		Plot 766 Bukcanan Crescent, Off Aminu Kano Crescent, Wuse II	Municipal	FCT
2084	FCT/0127	FCT/0127/S/3	Sami-Wadata Clinics Ltd.		Plot 766 Bukcanan Crescent, Off Aminu Kano Crescent, Wuse II	Municipal	FCT
2085	FCT/0127	FCT/0127/S/5	Sami-Wadata Clinics Ltd.		Plot 766 Bukcanan Crescent, Off Aminu Kano Crescent, Wuse II	Municipal	FCT
2086	FCT/0127	FCT/0127/S/12	Sami-Wadata Clinics Ltd.		Plot 766 Bukcanan Crescent, Off Aminu Kano Crescent, Wuse II	Municipal	FCT
2087	FCT/0128	FCT/0128/S/5	Iduna Specialist Hospital		Plot 1200 Ndjamena Crescent, Wuse Ii	Municipal	FCT
2088	FCT/0128	FCT/0128/S/2	Iduna Specialist Hospital		Plot 1200 Ndjamena Crescent, Wuse Ii	Municipal	FCT
2089	FCT/0131	FCT/0131/S/3	Mercy Specialist Hospital & Diag. Centre		Off Aminu Kano Crescent, Plot 619c Zone A7, Wuse II, Abuja	Municipal	FCT
2090	FCT/0131	FCT/0131/S/2	Mercy Specialist Hospital & Diag. Centre		Off Aminu Kano Crescent, Plot 619c Zone A7, Wuse II, Abuja	Municipal	FCT
2091	FCT/0131	FCT/0131/S/4	Mercy Specialist Hospital & Diag. Centre		Off Aminu Kano Crescent, Plot 619c Zone A7, Wuse II, Abuja	Municipal	FCT
2092	FCT/0131	FCT/0131/S/1	Mercy Specialist Hospital & Diag. Centre		Off Aminu Kano Crescent, Plot 619c Zone A7, Wuse II, Abuja	Municipal	FCT
2093	FCT/0131	FCT/0131/S/6	Mercy Specialist Hospital & Diag. Centre		Off Aminu Kano Crescent, Plot 619c Zone A7, Wuse II, Abuja	Municipal	FCT
2094	FCT/0131	FCT/0131/S/5	Mercy Specialist Hospital & Diag. Centre		Off Aminu Kano Crescent, Plot 619c Zone A7, Wuse II, Abuja	Municipal	FCT
2095	FCT/0132	FCT/0132/S/8	Kings Care Hospital - Wuse		Wuse Zone 4, Abuja	Municipal	FCT
2096	FCT/0132	FCT/0132/S/10	Kings Care Hospital - Wuse		Wuse Zone 4, Abuja	Municipal	FCT
2097	FCT/0132	FCT/0132/S/6	Kings Care Hospital - Wuse		Wuse Zone 4, Abuja	Municipal	FCT
2098	FCT/0132	FCT/0132/S/15	Kings Care Hospital - Wuse		Wuse Zone 4, Abuja	Municipal	FCT
2099	FCT/0132	FCT/0132/S/11	Kings Care Hospital - Wuse		Wuse Zone 4, Abuja	Municipal	FCT
2100	FCT/0132	FCT/0132/S/1	Kings Care Hospital - Wuse		Wuse Zone 4, Abuja	Municipal	FCT
2101	FCT/0132	FCT/0132/S/3	Kings Care Hospital - Wuse		Wuse Zone 4, Abuja	Municipal	FCT
2102	FCT/0133	FCT/0133/S/8	Capital Hospital		15, Oran Street, by Bank PHB, Off Olusegun Obasanjo way, Abuja	Municipal	FCT
2103	FCT/0133	FCT/0133/S/5	Capital Hospital		15, Oran Street, by Bank PHB, Off Olusegun Obasanjo way, Abuja	Municipal	FCT
2104	FCT/0133	FCT/0133/S/15	Capital Hospital		15, Oran Street, by Bank PHB, Off Olusegun Obasanjo way, Abuja	Municipal	FCT
2105	FCT/0138	FCT/0138/S/5	Queens Clinics		Plot 225, Cotonou Crescent, Wuse Zone 6, Abuja	Municipal	FCT
2106	FCT/0138	FCT/0138/S/10	Queens Clinics		Plot 225, Cotonou Crescent, Wuse Zone 6, Abuja	Municipal	FCT
2107	FCT/0139	FCT/0139/S/5	Bio-Royal Hospital & Maternity Ltd.		No. 190, Okene/Jebba Close, Garki	Municipal	FCT
2108	FCT/0139	FCT/0139/S/6	Bio-Royal Hospital & Maternity Ltd.		No. 190, Okene/Jebba Close, Garki	Municipal	FCT
2109	FCT/0139	FCT/0139/S/3	Bio-Royal Hospital & Maternity Ltd.		No. 190, Okene/Jebba Close, Garki	Municipal	FCT

2110	FCT/0139	FCT/0139/S/1	Bio-Royal Hospital & Maternity Ltd.		No. 190, Okene/Jebba Close, Garki	Municipal	FCT
2111	FCT/0141	FCT/0141/S/8	Holy Trinity Hospital Ltd.		27, Oro Ago Crescent, off Muhammadu Buhari Way, Garki II, Abuja	Municipal	FCT
2112	FCT/0141	FCT/0141/S/5	Holy Trinity Hospital Ltd.		27, Oro Ago Crescent, off Muhammadu Buhari Way, Garki II, Abuja	Municipal	FCT
2113	FCT/0141	FCT/0141/S/2	Holy Trinity Hospital Ltd.		27, Oro Ago Crescent, off Muhammadu Buhari Way, Garki II, Abuja	Municipal	FCT
2114	FCT/0141	FCT/0141/S/1	Holy Trinity Hospital Ltd.		27, Oro Ago Crescent, off Muhammadu Buhari Way, Garki II, Abuja	Municipal	FCT
2115	FCT/0141	FCT/0141/S/7	Holy Trinity Hospital Ltd.		27, Oro Ago Crescent, off Muhammadu Buhari Way, Garki II, Abuja	Municipal	FCT
2116	FCT/0143	FCT/0143/S/5	Diamond Medical Centre Ltd.		Plot 42, Beside Mr. Bigs By Model Pri. Sch. Gark Ii	Municipal	FCT
2117	FCT/0143	FCT/0143/S/3	Diamond Medical Centre Ltd.		Plot 42, Beside Mr. Bigs By Model Pri. Sch. Gark Ii	Municipal	FCT
2118	FCT/0147	FCT/0147/S/3	Dara Medical Clinics		Plot 202, Bacita Close, Off Plateau Street, Area 2 , Garki	Municipal	FCT
2119	FCT/0147	FCT/0147/S/6	Dara Medical Clinics		Plot 202, Bacita Close, Off Plateau Street, Area 2 , Garki	Municipal	FCT
2120	FCT/0147	FCT/0147/S/14	Dara Medical Clinics		Plot 202, Bacita Close, Off Plateau Street, Area 2 , Garki	Municipal	FCT
2121	FCT/0147	FCT/0147/S/1	Dara Medical Clinics		Plot 202, Bacita Close, Off Plateau Street, Area 2 , Garki	Municipal	FCT
2122	FCT/0147	FCT/0147/S/7	Dara Medical Clinics		Plot 202, Bacita Close, Off Plateau Street, Area 2 , Garki	Municipal	FCT
2123	FCT/0149	FCT/0149/S/15	Ministry Of Defence Staff Clinic		Ship House, Olusegun Obasanjo Way, Area 10, Garki	Municipal	FCT
2124	FCT/0149	FCT/0149/S/5	Ministry Of Defence Staff Clinic		Ship House, Olusegun Obasanjo Way, Area 10, Garki	Municipal	FCT
2125	FCT/0149	FCT/0149/S/3	Ministry Of Defence Staff Clinic		Ship House, Olusegun Obasanjo Way, Area 10, Garki	Municipal	FCT
2126	FCT/0150	FCT/0150/S/7	Kinectic Hospital Limited		Ahoada Close, off Emeka Anyaoku street, Area Ii, Garki	Municipal	FCT
2127	FCT/0150	FCT/0150/S/5	Kinectic Hospital Limited		Ahoada Close, off Emeka Anyaoku street, Area Ii, Garki	Municipal	FCT
2128	FCT/0150	FCT/0150/S/8	Kinectic Hospital Limited		Ahoada Close, off Emeka Anyaoku street, Area Ii, Garki	Municipal	FCT
2129	FCT/0152	FCT/0152/S/14	Lafiak Hospital		Toto Road, Abaji	Abaji	FCT
2130	FCT/0152	FCT/0152/S/1	Lafiak Hospital		Toto Road, Abaji	Abaji	FCT
2131	FCT/0153	FCT/0153/S/1	Gwagwalada Clinic & Maternity		Kutunku Along Frcn Road, Abuja	Gwagwalada	FCT
2132	FCT/0153	FCT/0153/S/5	Gwagwalada Clinic & Maternity		Kutunku Along Frcn Road, Abuja	Gwagwalada	FCT
2133	FCT/0153	FCT/0153/S/3	Gwagwalada Clinic & Maternity		Kutunku Along Frcn Road, Abuja	Gwagwalada	FCT
2134	FCT/0154	FCT/0154/S/2	Jerab Hospitals		Plot 145, Kutunku Near Radio House, Gwagwalada, Abuja	Gwagwalada	FCT
2135	FCT/0154	FCT/0154/S/5	Jerab Hospitals		Plot 145, Kutunku Near Radio House, Gwagwalada, Abuja	Gwagwalada	FCT
2136	FCT/0154	FCT/0154/S/3	Jerab Hospitals		Plot 145, Kutunku Near Radio House, Gwagwalada, Abuja	Gwagwalada	FCT
2137	FCT/0154	FCT/0154/S/6	Jerab Hospitals		Plot 145, Kutunku Near Radio House, Gwagwalada, Abuja	Gwagwalada	FCT
2138	FCT/0154	FCT/0154/S/1	Jerab Hospitals		Plot 145, Kutunku Near Radio House, Gwagwalada, Abuja	Gwagwalada	FCT
2139	FCT/0154	FCT/0154/S/7	Jerab Hospitals		Plot 145, Kutunku Near Radio House, Gwagwalada, Abuja	Gwagwalada	FCT
2140	FCT/0155	FCT/0155/S/1	Sliver Fountain Medical Centre		Kukwala District, Opp. Kuchingoro, Along Airport Road, Abuja	Municipal	FCT
2141	FCT/0155	FCT/0155/S/5	Sliver Fountain Medical Centre		Kukwala District, Opp. Kuchingoro, Along Airport Road, Abuja	Municipal	FCT
2142	FCT/0156	FCT/0156/S/1	Rapha Hospital And Maternity		Phase Iii, Near Giwa Hotel, Jikwoyi, Abuja	Municipal	FCT
2143	FCT/0156	FCT/0156/S/5	Rapha Hospital And Maternity		Phase Iii, Near Giwa Hotel, Jikwoyi, Abuja	Municipal	FCT

2144	FCT/0156	FCT/0156/S/6	Rapha Hospital And Maternity		Phase Iii, Near Giwa Hotel, Jikwoyi, Abuja	Municipal	FCT
2145	FCT/0156	FCT/0156/S/3	Rapha Hospital And Maternity		Phase Iii, Near Giwa Hotel, Jikwoyi, Abuja	Municipal	FCT
2146	FCT/0158	FCT/0158/S/6	Nisa Premier Hospital		Plot 618, Alex Ekweme Way, Jabi Abuja	Municipal	FCT
2147	FCT/0158	FCT/0158/S/1	Nisa Premier Hospital		Plot 618, Alex Ekweme Way, Jabi Abuja	Municipal	FCT
2148	FCT/0158	FCT/0158/S/3	Nisa Premier Hospital		Plot 618, Alex Ekweme Way, Jabi Abuja	Municipal	FCT
2149	FCT/0158	FCT/0158/S/5	Nisa Premier Hospital		Plot 618, Alex Ekweme Way, Jabi Abuja	Municipal	FCT
2150	FCT/0159	FCT/0159/S/15	Abuja Clinic - Maitama		Maitama, Abuja	Municipal	FCT
2151	FCT/0159	FCT/0159/S/5	Abuja Clinic - Maitama		Maitama, Abuja	Municipal	FCT
2152	FCT/0159	FCT/0159/S/6	Abuja Clinic - Maitama		Maitama, Abuja	Municipal	FCT
2153	FCT/0159	FCT/0159/S/4	Abuja Clinic - Maitama		Maitama, Abuja	Municipal	FCT
2154	FCT/0159	FCT/0159/S/11	Abuja Clinic - Maitama		Maitama, Abuja	Municipal	FCT
2155	FCT/0159	FCT/0159/S/10	Abuja Clinic - Maitama		Maitama, Abuja	Municipal	FCT
2156	FCT/0159	FCT/0159/S/8	Abuja Clinic - Maitama		Maitama, Abuja	Municipal	FCT
2157	FCT/0159	FCT/0159/S/7	Abuja Clinic - Maitama		Maitama, Abuja	Municipal	FCT
2158	FCT/0159	FCT/0159/S/2	Abuja Clinic - Maitama		Maitama, Abuja	Municipal	FCT
2159	FCT/0165	FCT/0165/S/4	Bamak Pharmacy Ltd.	Pharmacy	Suite 5 Sopping Complex Zone 3, Wuse	Municipal	FCT
2160	FCT/0169	FCT/0169/S/4	Welcare Pharmacy Nig. Ltd.	Pharmacy	590a Adekunbo Ademola Crescent, Wuse II	Municipal	FCT
2161	FCT/0170	FCT/0170/S/4	Opi Pharmacy & Stores Ltd.,	Pharmacy	Sky Memorial Complex, Plot 578 Michael Okpara Way, Zone 5	Municipal	FCT
2162	FCT/0172	FCT/0172/S/4	Lakeside Pharmacy Ltd.	Pharmacy	Suite Shariff Shopping Complex Kade Str. Wuse II, Off Aminu	Municipal	FCT
2163	FCT/0178	FCT/0178/S/4	Total Trust Pharmacy Ltd.	Pharmacy	Capital Plaza Old Karu Raod, Nyanya	Municipal	FCT
2164	FCT/0179	FCT/0179/S/4	Universal Gaskiya Pharmacy	Pharmacy	Sop 50/51 KN Comlpex Nyanya Market Rd. Abuja	Municipal	FCT
2165	FCT/0180	FCT/0180/S/4	Comprehensive Health Centre	Pharmacy, Laboratory	Karshi, Abuja	Municipal	FCT
2166	FCT/0180	FCT/0180/S/5	Comprehensive Health Centre	Pharmacy, Laboratory	Karshi, Abuja	Municipal	FCT
2167	FCT/0183	FCT/0183/S/4	Besio Pharmacy Ltd.	Pharmacy	45 Hillside Area A, Nyanya	Municipal	FCT
2168	FCT/0188	FCT/0188/S/4	Redemption Pharmacy Ltd.	Pharmacy	Shop 25, Neighbouthood Shopping Complex, F.H.A. Lugbe	Municipal	FCT
2169	FCT/0189	FCT/0189/S/4	Newlife Pharmacy Nig. Ltd.	Pharmacy	Shop G3, Fatima Plaza, Plot 2142, Mambolo Street, Opp. Catholic Church, Wuse Zone 2, Abuja	Municipal	FCT
2170	FCT/0193	FCT/0193/S/4	Deluxe Pharmacy & Stores	Pharmacy	11, Peshe Road, Karu	Municipal	FCT
2171	FCT/0194	FCT/0194/S/4	Biogenes Pharmacy	Pharmacy	Conershop Opp. Custom Barracks, Karu	Municipal	FCT
2172	FCT/0195	FCT/0195/S/5	Abba Medical Laboratories	Laboratory	UTC Complex, Suite FF 14, Area 10	Municipal	FCT
2173	FCT/0198	FCT/0198/S/5	Sauki Medical Diagnostic Centre	Laboratory	Shop 6, Neigbourhood Centre, Wuse Zone 3	Municipal	FCT
2174	FCT/0201	FCT/0201/S/5	Biosensors Medical Laboratory	Laboratory	Suite 212, 411 Plaza, Near Big Bite, Adetokunbo Ademola Crescent.	Municipal	FCT
2175	FCT/0202	FCT/0202/S/5	Solo Medical Laboratory Services	Laboratory	466, Lobito Crescent, Wuse II, Abuja.	Municipal	FCT
2176	FCT/0203	FCT/0203/S/5	Mek Medical Daignostic Laboratory	Laboratory	Suite D6, Abuja Shopping Mall, Wuse Zone 3	Municipal	FCT
2177	FCT/0207	FCT/0207/S/4	Abson Pharmacy & Stores	Pharmacy	Suite 9, Peoples Shopping Centre, Plot 1263, Jere Street, Garki	Municipal	FCT
2178	FCT/0208	FCT/0208/S	Regal Dental Clinic	Dental	Plot 202, Bacita Close, Off Plateau Street, Area 2, Section	Municipal	FCT
2179	FCT/0211	FCT/0211/S/4	Medixal Nig. Ltd.	Pharmacy	Shop 10 Zone B N/hood Shopping Centre, Apo	Municipal	FCT
2180	FCT/0216	FCT/0216/S/6	Maitama District Hospital	Internal Medicine, Surgery, O & G, Paediatrics, Pharmacy, Laboratory	Maitama, Abuja	Municipal	FCT

2181	FCT/0227	FCT/0227/S/4	Alpha Pharmacy	Pharmacy	Victory Plaza, Plot 1490 Gimbiya Street, Area 11, Garki, Abuja	Municipal	FCT
2182	FCT/0229	FCT/0229/S/4	De-peror Pharmacy & Stores Limited	Pharmacy	Syndicate Plaza, Area 11, Garki, Abuja	Municipal	FCT
2183	FCT/0230	FCT/0230/S/4	Mc Sofi Pharmacy Ltd.	Pharmacy	Plot 1330 Jere Str. Off Funmilayo Ranson Kuti Way, Garki II	Municipal	FCT
2184	FCT/0231	FCT/0231/S/4	Ril Pharmacy & Stores Ltd.	Pharmacy	Suite 516 Labbo Abubakar Shopping Complex 12 Crescent, Kado	Municipal	FCT
2185	FCT/0234	FCT/0234/S/4	Zagbayi Pharmacy Ltd.	Pharmacy	Plot 855 Tafawa Balawa Way Area 11	Municipal	FCT
2186	FCT/0236	FCT/0236/S/4	Health Harbour Pharmacy,	Pharmacy	12/14 Maitama Sule Street, Opp. O.A.U. Qtrs., Asokoro	Municipal	FCT
2187	FCT/0238	FCT/0238/S/4	Giems Forture Ltd. Pharmacy Div.	Pharmacy	Plot D 232 Road By 231 Crescent Kado Housing Estate.	Municipal	FCT
2188	FCT/0240	FCT/0240/S/4	Patigah Pharmacy Ltd.EffurunOvwie	Pharmacy	Plot 2149 Novakcott Str. Wuse Zone 1	Municipal	FCT
2189	FCT/0241	FCT/0241/S/5	Winners Medical Diag. & Herbal Centre	Laboratory	Plot 139, Kadonger Close, Garki II Opp. Cbn.	Municipal	FCT
2190	FCT/0242	FCT/0242/S/5	Adonai Laboratory	Laboratory	Area 7, (Behind Arts & Culture) Garki, Abuja	Municipal	FCT
2191	FCT/0258	FCT/0258/S/4	Beta Pharm Ltd.	Pharmacy	Area 2, Shopping Complex	Municipal	FCT
2192	FCT/0259	FCT/0259/S/4	Aldak Pharmacy	Pharmacy	Abuja Shopping Complex, Area 10, Garki	Municipal	FCT
2193	FCT/0263	FCT/0263/S/4	Zion Pharmacy	Pharmacy	Plot 51, Ubiaja Cres. Opp CBN Snr., Staff quarters, Garki II, Abuja.	Municipal	FCT
2194	FCT/0266	FCT/0266/S/4	Aners Pharmaceuticals & Co.	Pharmacy	Suite 3 & 4, Beside Plaza, Off NEPA Camp Road, Apo, Area 1	Municipal	FCT
2195	FCT/0269	FCT/0269/S/4	Fhidu Pharmacy Ltd.	Pharmacy	Jikwoyi Phase I, After Ashaka Cement,	Municipal	FCT
2196	FCT/0275	FCT/0275/S/15	Clear View Optics Ltd.	Ophthalmology	NICON Hilton Hotel, Maitama, Abuja	Municipal	FCT
2197	FCT/0285	FCT/0285/S/8	Calens Dental Clinics	Dental	Suite A9, Poly Plaza Near Ap Plaza, Wuse II, Abuja	Municipal	FCT
2198	FCT/0296	FCT/0296/S/15	Nuns Eye Centre	Ophthalmology	Ahmadu Bello Way, Plot 1249, Opp. Standard Trust Bank.	Municipal	FCT
2199	FCT/0302	FCT/0302/S/5	Tayodek Diagnostics	Radiology, Laboratory	Plot 211M, Garki Cornershops, Garki, Abuja	Municipal	FCT
2200	FCT/0302	FCT/0302/S/7	Tayodek Diagnostics	Radiology, Laboratory	Plot 211M, Garki Cornershops, Garki, Abuja	Municipal	FCT
2201	FCT/0310	FCT/0310/S/4	Becan Pharmacy Ltd.	Pharmacy	739, Sheriff Plaza, Aminu Kano Crescent, Wuse II, Abuja	Municipal	FCT
2202	FCT/0311	FCT/0311/S/4	Rhosag Pharmacy	Pharmacy	Fabdal Shopping Complex, Suite 105, Wuse Zone 4, Abuja	Municipal	FCT
2203	FCT/0312	FCT/0312/S	Pharmamedics Pharmacy Ltd.	Pharmacy	Shop 20, Wuse Neighbourhood Centre, Zone 3, Abuja	Municipal	FCT
2204	FCT/0313	FCT/0313/S	Junox ABC Nig. Ltd.,	Pharmacy	Shop E7, Emab Plaza 751 Aminu Kano Crescent, Wuse II, Abuja	Municipal	FCT
2205	FCT/0316	FCT/0316/S/4	C.O.D.Chemists	Pharmacy	Wuse Shopping Centre Abuja	Municipal	FCT
2206	FCT/0318	FCT/0318/S/4	Lawcas Pharmacy	Pharmacy	Shop 5 & 10 Plot 59011 Adetokumbo Ademola Cret Wuse II Abuja	Municipal	FCT
2207	FCT/0319	FCT/0319/S/4	Agape Pharmacy	Pharmacy	Sheraton Hotel Wuse Zone 4, Abuja	Municipal	FCT
2208	FCT/0320	FCT/0320/S/4	Lifewares Pharmacy	Pharmacy	Corner Shop 43 Mambilo Wuse Zone 2 Abuja	Municipal	FCT
2209	FCT/0321	FCT/0321/S/4	Skylark Pharmacy & Chem. Co. Ltd	Pharmacy	D9/10 Sky Memorial Complex Wuse Zone 5 Abuja	Municipal	FCT
2210	FCT/0322	FCT/0322/S/4	Paxs Pharmacy	Pharmacy	Poly Plaza By Ap Plaza Wuse II Abuja	Municipal	FCT
2211	FCT/0323	FCT/0323/S/4	Eutrix Pharmaceuticals Ltd	Pharmacy	5, Ladi Kwali Street, Wuse Zone 4 Abuja	Municipal	FCT
2212	FCT/0324	FCT/0324/S/4	Ace Pharmaceutical Co. Ltd	Pharmacy	Finance Qtrs, Complex Plot 817 Wuye District Abuja	Municipal	FCT
2213	FCT/0330	FCT/0330/S/4	National Hospital Abuja		Central Area	Municipal	FCT
2214	FCT/0330	FCT/0330/S/1	National Hospital Abuja		Central Area	Municipal	FCT
2215	FCT/0330	FCT/0330/S/10	National Hospital Abuja		Central Area	Municipal	FCT

2216	FCT/0330	FCT/0330/S/12	National Hospital Abuja		Central Area	Municipal	FCT
2217	FCT/0330	FCT/0330/S/3	National Hospital Abuja		Central Area	Municipal	FCT
2218	FCT/0330	FCT/0330/S/6	National Hospital Abuja		Central Area	Municipal	FCT
2219	FCT/0330	FCT/0330/S/11	National Hospital Abuja		Central Area	Municipal	FCT
2220	FCT/0330	FCT/0330/S/15	National Hospital Abuja		Central Area	Municipal	FCT
2221	FCT/0330	FCT/0330/S/5	National Hospital Abuja		Central Area	Municipal	FCT
2222	FCT/0333	FCT/0333/S/5	Albert Horsfall Med. Centre		NIA Hq, off Muritala Mohammed way, near Danata & Sawoe, Garki	Municipal	FCT
2223	FCT/0333	FCT/0333/S/14	Albert Horsfall Med. Centre		NIA Hq, off Muritala Mohammed way, near Danata & Sawoe, Garki	Municipal	FCT
2224	FCT/0333	FCT/0333/S/4	Albert Horsfall Med. Centre		NIA Hq, off Muritala Mohammed way, near Danata & Sawoe, Garki	Municipal	FCT
2225	FCT/0333	FCT/0333/S/8	Albert Horsfall Med. Centre		NIA Hq, off Muritala Mohammed way, near Danata & Sawoe, Garki	Municipal	FCT
2226	FCT/0334	FCT/0334/S/4	Defence Intelligence Agency		Asokoro	Municipal	FCT
2227	FCT/0334	FCT/0334/S/5	Defence Intelligence Agency		Asokoro	Municipal	FCT
2228	FCT/0335	FCT/0335/S/5	Revenue Mobilization & Fiscal		210 Tafawa Balewa Way, Central Area, Garki	Municipal	FCT
2229	FCT/0336	FCT/0336/S/6	Living Stream Specialist Hospi		43/45, Street A, Phase I, off University Rd, Gwagwalada	Gwagwalada	FCT
2230	FCT/0336	FCT/0336/S/14	Living Stream Specialist Hospi		43/45, Street A, Phase I, off University Rd, Gwagwalada	Gwagwalada	FCT
2231	FCT/0336	FCT/0336/S/3	Living Stream Specialist Hospi		43/45, Street A, Phase I, off University Rd, Gwagwalada	Gwagwalada	FCT
2232	FCT/0336	FCT/0336/S/5	Living Stream Specialist Hospi		43/45, Street A, Phase I, off University Rd, Gwagwalada	Gwagwalada	FCT
2233	FCT/0337	FCT/0337/S/6	Angelic Care Hospital & Mat.		19, Ngwa close off Funmilayo Ransome Kuti Road, Garki Area 3, Abuja	Municipal	FCT
2234	FCT/0337	FCT/0337/S/3	Angelic Care Hospital & Mat.		19, Ngwa close off Funmilayo Ransome Kuti Road, Garki Area 3, Abuja	Municipal	FCT
2235	FCT/0337	FCT/0337/S/5	Angelic Care Hospital & Mat.		19, Ngwa close off Funmilayo Ransome Kuti Road, Garki Area 3, Abuja	Municipal	FCT
2236	FCT/0340	FCT/0340/S/4	DSS Medical Centre.		VGC, Opposite National Military Cemetary, Airport Road, Abuja	Municipal	FCT
2237	FCT/0340	FCT/0340/S/6	DSS Medical Centre.		VGC, Opposite National Military Cemetary, Airport Road, Abuja	Municipal	FCT
2238	FCT/0340	FCT/0340/S/3	DSS Medical Centre.		VGC, Opposite National Military Cemetary, Airport Road, Abuja	Municipal	FCT
2239	FCT/0340	FCT/0340/S/2	DSS Medical Centre.		VGC, Opposite National Military Cemetary, Airport Road, Abuja	Municipal	FCT
2240	FCT/0340	FCT/0340/S/1	DSS Medical Centre.		VGC, Opposite National Military Cemetary, Airport Road, Abuja	Municipal	FCT
2241	FCT/0340	FCT/0340/S/5	DSS Medical Centre.		VGC, Opposite National Military Cemetary, Airport Road, Abuja	Municipal	FCT
2242	FCT/0340	FCT/0340/S/7	DSS Medical Centre.		VGC, Opposite National Military Cemetary, Airport Road, Abuja	Municipal	FCT
2243	FCT/0344	FCT/0344/S/4	Nigeria Customs Service Med. Centre - Karu		Karu Customs Barracks, Abuja	Municipal	FCT
2244	FCT/0346	FCT/0346/S/3	108 Nigerian Air Force Hospital Abuja		108 Nigerian Air Force Hospital Abuja	Municipal	FCT
2245	FCT/0346	FCT/0346/S/1	108 Nigerian Air Force Hospital Abuja		108 Nigerian Air Force Hospital Abuja	Municipal	FCT
2246	FCT/0346	FCT/0346/S/10	108 Nigerian Air Force Hospital Abuja		108 Nigerian Air Force Hospital Abuja	Municipal	FCT
2247	FCT/0346	FCT/0346/S/5	108 Nigerian Air Force Hospital Abuja		108 Nigerian Air Force Hospital Abuja	Municipal	FCT
2248	FCT/0346	FCT/0346/S/4	108 Nigerian Air Force Hospital Abuja		108 Nigerian Air Force Hospital Abuja	Municipal	FCT
2249	FCT/0346	FCT/0346/S/6	108 Nigerian Air Force Hospital Abuja		108 Nigerian Air Force Hospital Abuja	Municipal	FCT
2250	FCT/0346	FCT/0346/S/2	108 Nigerian Air Force Hospital Abuja		108 Nigerian Air Force Hospital Abuja	Municipal	FCT

2251	FCT/0346	FCT/0346/S/8	108 Nigerian Air Force Hospital Abuja		108 Nigerian Air Force Hospital Abuja	Municipal	FCT
2252	FCT/0346	FCT/0346/S/11	108 Nigerian Air Force Hospital Abuja		108 Nigerian Air Force Hospital Abuja	Municipal	FCT
2253	FCT/0352	FCT/0352/S/4	Cameb Pharmacy	Pharmacy	Opp. ECWA Church, Mpape, Abuja.	Municipal	FCT
2254	FCT/0357	FCT/0357/S/2	Bwari General Hospital		Kawu Road, Bwari, FCT, ABuja	Bwari	FCT
2255	FCT/0357	FCT/0357/S/4	Bwari General Hospital		Kawu Road, Bwari, FCT, ABuja	Bwari	FCT
2256	FCT/0357	FCT/0357/S/1	Bwari General Hospital		Kawu Road, Bwari, FCT, ABuja	Bwari	FCT
2257	FCT/0357	FCT/0357/S/5	Bwari General Hospital		Kawu Road, Bwari, FCT, ABuja	Bwari	FCT
2258	FCT/0358	FCT/0358/S/4	Fredway Pharmaceuticals Ltd	Pharmacy	Plot A Jacob Ngbako Road, Kubwa, Abuja	Bwari	FCT
2259	FCT/0360	FCT/0360/S/4	Abuja Cliniic - Karu		Karu, Abuja	Municipal	FCT
2260	FCT/0362	FCT/0362/S/1	G & S Specialist Clinic		8B Rhine Street (Opp. IBB Way), Maitama	Municipal	FCT
2261	FCT/0362	FCT/0362/S/5	G & S Specialist Clinic		8B Rhine Street (Opp. IBB Way), Maitama	Municipal	FCT
2262	FCT/0366	FCT/0366/S/3	Saffron Hospital Ltd.		1 KM, Left Off Checkpoint, Abuja-Keffi Expressway, Phase IV, Nyanya, FCT	Municipal	FCT
2263	FCT/0366	FCT/0366/S/5	Saffron Hospital Ltd.		1 KM, Left Off Checkpoint, Abuja-Keffi Expressway, Phase IV, Nyanya, FCT	Municipal	FCT
2264	FCT/0366	FCT/0366/S/4	Saffron Hospital Ltd.		1 KM, Left Off Checkpoint, Abuja-Keffi Expressway, Phase IV, Nyanya, FCT	Municipal	FCT
2265	FCT/0378	FCT/0378/S/6	Horrizons Medical Centre	Primary Provider	Plot 777 Bouake Street Wuse Zone 6, Abuja	Municipal	FCT
2266	FCT/0378	FCT/0378/S/5	Horrizons Medical Centre	Primary Provider	Plot 777 Bouake Street Wuse Zone 6, Abuja	Municipal	FCT
2267	FCT/0378	FCT/0378/S/14	Horrizons Medical Centre	Primary Provider	Plot 777 Bouake Street Wuse Zone 6, Abuja	Municipal	FCT
2268	FCT/0378	FCT/0378/S/1	Horrizons Medical Centre	Primary Provider	Plot 777 Bouake Street Wuse Zone 6, Abuja	Municipal	FCT
2269	FCT/0378	FCT/0378/S/3	Horrizons Medical Centre	Primary Provider	Plot 777 Bouake Street Wuse Zone 6, Abuja	Municipal	FCT
2270	FCT/0378	FCT/0378/S/2	Horrizons Medical Centre	Primary Provider	Plot 777 Bouake Street Wuse Zone 6, Abuja	Municipal	FCT
2271	FCT/0379	FCT/0379/S/4	Bepos Clinic & Maternity	Primary Provider	10, Faskari Street Area 3 Garki Abuja	Municipal	FCT
2272	FCT/0379	FCT/0379/S/5	Bepos Clinic & Maternity	Primary Provider	10, Faskari Street Area 3 Garki Abuja	Municipal	FCT
2273	FCT/0379	FCT/0379/S/2	Bepos Clinic & Maternity	Primary Provider	10, Faskari Street Area 3 Garki Abuja	Municipal	FCT
2274	FCT/0379	FCT/0379/S/3	Bepos Clinic & Maternity	Primary Provider	10, Faskari Street Area 3 Garki Abuja	Municipal	FCT
2275	FCT/0381	FCT/0381/S/5	National Commission for Colleges of Education (NCCE) Staff C	Primary Provider	NCCE Management Staff Quarters Mabushi Wuse II Abuja	Municipal	FCT
2276	FCT/0385	FCT/0385/S/14	Well Point Clinic		House 1, Road 45, Off 1st Ave, Gwarinpa	Municipal	FCT
2277	FCT/0385	FCT/0385/S/5	Well Point Clinic		House 1, Road 45, Off 1st Ave, Gwarinpa	Municipal	FCT
2278	FCT/0385	FCT/0385/S/1	Well Point Clinic		House 1, Road 45, Off 1st Ave, Gwarinpa	Municipal	FCT
2279	FCT/0385	FCT/0385/S/2	Well Point Clinic		House 1, Road 45, Off 1st Ave, Gwarinpa	Municipal	FCT
2280	FCT/0385	FCT/0385/S/6	Well Point Clinic		House 1, Road 45, Off 1st Ave, Gwarinpa	Municipal	FCT
2281	FCT/0385	FCT/0385/S/3	Well Point Clinic		House 1, Road 45, Off 1st Ave, Gwarinpa	Municipal	FCT
2282	FCT/0387	FCT/0387/S/3	Ronella Specialist Hospital		51 Crescent Road, Flat 3, Phase IV, Kubwa, Abuja	Bwari	FCT
2283	FCT/0389	FCT/0389/S/13	Rainbow Clinic & Maternity		Plot 218, Gudu District Apo Qtrs Garki, Abuja	Municipal	FCT
2284	FCT/0389	FCT/0389/S/6	Rainbow Clinic & Maternity		Plot 218, Gudu District Apo Qtrs Garki, Abuja	Municipal	FCT
2285	FCT/0389	FCT/0389/S/5	Rainbow Clinic & Maternity		Plot 218, Gudu District Apo Qtrs Garki, Abuja	Municipal	FCT
2286	FCT/0389	FCT/0389/S/3	Rainbow Clinic & Maternity		Plot 218, Gudu District Apo Qtrs Garki, Abuja	Municipal	FCT
2287	FCT/0389	FCT/0389/S/8	Rainbow Clinic & Maternity		Plot 218, Gudu District Apo Qtrs Garki, Abuja	Municipal	FCT
2288	FCT/0389	FCT/0389/S/1	Rainbow Clinic & Maternity		Plot 218, Gudu District Apo Qtrs Garki, Abuja	Municipal	FCT
2289	FCT/0390	FCT/0390/S/2	Federal Staff Hospital - Jabi		Airport Road, Jabi	Municipal	FCT
2290	FCT/0390	FCT/0390/S/3	Federal Staff Hospital - Jabi		Airport Road, Jabi	Municipal	FCT
2291	FCT/0390	FCT/0390/S/6	Federal Staff Hospital - Jabi		Airport Road, Jabi	Municipal	FCT

2292	FCT/0390	FCT/0390/S/1	Federal Staff Hospital - Jabi		Airport Road, Jabi	Municipal	FCT
2293	FCT/0391	FCT/0391/S/1	Everlasting Care Hospital Limited		Plot 643 Gimbia Street, Area II, Garki, Abuja	Municipal	FCT
2294	FCT/0391	FCT/0391/S/3	Everlasting Care Hospital Limited		Plot 643 Gimbia Street, Area II, Garki, Abuja	Municipal	FCT
2295	FCT/0391	FCT/0391/S/5	Everlasting Care Hospital Limited		Plot 643 Gimbia Street, Area II, Garki, Abuja	Municipal	FCT
2296	FCT/0392	FCT/0392/S/3	Micheal Cross Specialist Hospital		Plot FG Church Road, Karu Site, Karu, Abuja	Municipal	FCT
2297	FCT/0393	FCT/0393/S/2	Convenant Clinic & Maternity		Anygba Street Opp NTA Area 11 Garki	Municipal	FCT
2298	FCT/0393	FCT/0393/S/1	Convenant Clinic & Maternity		Anygba Street Opp NTA Area 11 Garki	Municipal	FCT
2299	FCT/0393	FCT/0393/S/3	Convenant Clinic & Maternity		Anygba Street Opp NTA Area 11 Garki	Municipal	FCT
2300	FCT/0394	FCT/0394/S/2	St Mary Catholic Hospital, Gwagwalada		No 1 FRCN Road, Opposite Police Station Gwagwalada	Gwagwalada	FCT
2301	FCT/0394	FCT/0394/S/7	St Mary Catholic Hospital, Gwagwalada		No 1 FRCN Road, Opposite Police Station Gwagwalada	Gwagwalada	FCT
2302	FCT/0394	FCT/0394/S/6	St Mary Catholic Hospital, Gwagwalada		No 1 FRCN Road, Opposite Police Station Gwagwalada	Gwagwalada	FCT
2303	FCT/0394	FCT/0394/S/4	St Mary Catholic Hospital, Gwagwalada		No 1 FRCN Road, Opposite Police Station Gwagwalada	Gwagwalada	FCT
2304	FCT/0394	FCT/0394/S/15	St Mary Catholic Hospital, Gwagwalada		No 1 FRCN Road, Opposite Police Station Gwagwalada	Gwagwalada	FCT
2305	FCT/0394	FCT/0394/S/1	St Mary Catholic Hospital, Gwagwalada		No 1 FRCN Road, Opposite Police Station Gwagwalada	Gwagwalada	FCT
2306	FCT/0394	FCT/0394/S/5	St Mary Catholic Hospital, Gwagwalada		No 1 FRCN Road, Opposite Police Station Gwagwalada	Gwagwalada	FCT
2307	FCT/0395	FCT/0395/S/5	Precious Clinic & Maternity		House 57, 1st Avenue FHA, Lugbe, Abuja	Municipal	FCT
2308	FCT/0406	FCT/0406/S/5	Usoh Medical Diagnostic Lab.	Laboratory	Area 2 Section 2, Bacita, Close, Garki ,Abuja	Municipal	FCT
2309	FCT/0407	FCT/0407/S/8	The Kings Dental Clinic	Dental	Suite 7A Garki II, Cornershop Garki II, Abuja	Municipal	FCT
2310	FCT/0408	FCT/0408/S/13	Newforesight Vision Eye Clinic	Optometry	Nig. Agric & Rural Dev. Complex, 2B Herbert Marculay Way, Wuse Zone 3, Abuja	Municipal	FCT
2311	FCT/0409	FCT/0409/S/13	Lord's Eye Clinic	Optometry	78 Abigan Street, Wuse Zone 3, Abuja	Municipal	FCT
2312	FCT/0410	FCT/0410/S/8	Kapital Dental Centre	Dental	suite 3 Area 3 Neighbourhood Centre ,Garki, Abuja	Municipal	FCT
2313	FCT/0411	FCT/0411/S/4	Melrose Pharmaceutical Stores	Pharmacy	Suite 1A Angels Plaza, Garki II, Abuja	Municipal	FCT
2314	FCT/0412	FCT/0412/S/4	Zagbayi Pharmacy Nig. Ltd.	Pharmacy	Famag-Jal Plaza, wuse Zone 3 Branch	Municipal	FCT
2315	FCT/0413	FCT/0413/S/4	Federal Ministry Of Defence Clinic	Pharmacy	Ship House Premises, Defence H/quarters, Abuja	Municipal	FCT
2316	FCT/0414	FCT/0414/S/4	Ronybo Pharmacy	Pharmacy	Blessed Plaza, Opposite Customs Clinic, Karu	Municipal	FCT
2317	FCT/0418	FCT/0418/S/4	Medixal Pharmacy Nig Ltd.	Pharmacy	Around Floor, Essence Plaza, Wuse Zone 6, Abuja	Municipal	FCT
2318	FCT/0419	FCT/0419/S/4	Cuteman Pharmacy & Stores	Pharmacy	Asb Shopping Plaza, Ademola Adetokunmbo Crescent Wuse II, Abuja	Municipal	FCT
2319	FCT/0421	FCT/0421/S/4	Excellent Vision Eye Centre	Pharmacy	Metro Plaza, Anex Central Area, Abuja	Municipal	FCT
2320	FCT/0422	FCT/0422/S/4	Sterling Pharmacy Ltd	Pharmacy	Suite 5 Dummec Plaza ,Area 1 Section 1	Municipal	FCT
2321	FCT/0424	FCT/0424/S/4	Azmu Pharmacy	Pharmacy	Suite 8, Mangal Plaza, Plot 1466 Area 11, Garki, Abuja	Municipal	FCT
2322	FCT/0425	FCT/0425/S/4	Kuje General Hospital		Kuje, Abuja.	Kuje	FCT
2323	FCT/0425	FCT/0425/S/5	Kuje General Hospital		Kuje, Abuja.	Kuje	FCT
2324	FCT/0425	FCT/0425/S/3	Kuje General Hospital		Kuje, Abuja.	Kuje	FCT
2325	FCT/0425	FCT/0425/S/1	Kuje General Hospital		Kuje, Abuja.	Kuje	FCT
2326	FCT/0425	FCT/0425/S/2	Kuje General Hospital		Kuje, Abuja.	Kuje	FCT
2327	FCT/0426	FCT/0426/S/4	Nigerian Customs Medical Centre	Pharmacy	Karu Customs Barracks, Karu	Municipal	FCT
2328	FCT/0428	FCT/0428/S/4	Trumed Pharm & Health Shop	Pharmacy	Suite 103,	Municipal	FCT

2329	FCT/0430	FCT/0430/S/5	Carmel Wood Med. Clinic	O&G, Laboratory, Surgery	31, 1st Avenue, Kado Estate, Abuja	Municipal	FCT
2330	FCT/0430	FCT/0430/S/1	Carmel Wood Med. Clinic	O&G, Laboratory, Surgery	31, 1st Avenue, Kado Estate, Abuja	Municipal	FCT
2331	FCT/0430	FCT/0430/S/3	Carmel Wood Med. Clinic	O&G, Laboratory, Surgery	31, 1st Avenue, Kado Estate, Abuja	Municipal	FCT
2332	FCT/0431	FCT/0431/S/4	Luwa Pharmacy Ltd	Pharmacy	1st Avenue Opp Foreign Affairs Qtrs, Kado Baurko, Gwarinpa	Municipal	FCT
2333	FCT/0433	FCT/0433/S/4	Maxi Pharmacy Ltd	Pharmacy	Gwarinpa Plaza, 3rd Avenue, FHA, Abuja	Municipal	FCT
2334	FCT/0436	FCT/0436/S/4	Mak Angels Pharm.	Pharmacy	FHA Phase I, Lugbe, Abuja	Municipal	FCT
2335	FCT/0437	FCT/0437/S/4	Redemption Pharmacy	Pharmacy	FHA Phase I, Lugbe, Abuja	Municipal	FCT
2336	FCT/0439	FCT/0439/S/13	First Optician Eye Clinic	Optometry	Plot 855 Suite 205 DM Yadodo House, Opp. La Meridean Hotel, Area 11, Abuja	Municipal	FCT
2337	FCT/0440	FCT/0440/S/4	Jonik Pharmacy	Pharmacy	Plot 780 Obafemi Awolowo Street, Area 11, Garki, Abuja	Municipal	FCT
2338	FCT/0447	FCT/0447/S/4	Mubaraq Pharmacy	Pharmacy	Suite 3 Maitama Shopping Complex	Municipal	FCT
2339	FCT/0451	FCT/0451/S/5	Federal Staff Hospital	Laboratory	National Assembly Qtrs., Zone E, Apo, Abuja	Municipal	FCT
2340	FCT/0452	FCT/0452/S/4	New Trends Pharmacy Ltd	Pharmacy	Plot 53, 321 Road, Gwarinpa, Abuja	Municipal	FCT
2341	FCT/0457	FCT/0457/S/4	Jonik Pharmacy Ltd	Pharmacy	Shop 2, Wuse II, Lifecamp, Abuja	Municipal	FCT
2342	FCT/0458	FCT/0458/S/4	Nimilen Pharmacy Ltd	Pharmacy	Plot C7 off Deeper Life Junction, Sultan Dasuki Rd, Kubwa	Bwari	FCT
2343	FCT/0459	FCT/0459/S/4	Emma-Ella Pharmacy Ltd	Pharmacy	No.1 Elite Shopping Complex, 26 Adua Close, off Tufashiya Cresc. Lifecamp, Abuja	Municipal	FCT
2344	FCT/0460	FCT/0460/S/4	Rayjones Pharmacy Ltd	Pharmacy	Hamza Abdullahi/Byazhin Junction, Kubwa village, Abuja	Bwari	FCT
2345	FCT/0462	FCT/0462/S/13	Spendour Vixion Eye Clinic	Optometry	Gubabi SHFE Plaza, Utako Modern Market, Abuja	Municipal	FCT
2346	FCT/0471	FCT/0471/S/9	Recovery Psychiatry Services	Psychiatry	C/o Zankli Hospital Premises, Opp. Fed. Min. of Works, Mabushi, Abuja	Municipal	FCT
2347	FCT/0472	FCT/0472/S/5	Dr. Hassans Clinic/Diagnostic Centre		9 Njamena Street, off Aminu Kano Crescent, Wuse II, Abuja	Municipal	FCT
2348	FCT/0478	FCT/0478/S/4	University of Abuja Teaching Hospital, Gwagwalada		Abuja	Gwagwalada	FCT
2349	FCT/0478	FCT/0478/S/6	University of Abuja Teaching Hospital, Gwagwalada		Abuja	Gwagwalada	FCT
2350	FCT/0478	FCT/0478/S/3	University of Abuja Teaching Hospital, Gwagwalada		Abuja	Gwagwalada	FCT
2351	FCT/0478	FCT/0478/S/1	University of Abuja Teaching Hospital, Gwagwalada		Abuja	Gwagwalada	FCT
2352	FCT/0478	FCT/0478/S/8	University of Abuja Teaching Hospital, Gwagwalada		Abuja	Gwagwalada	FCT
2353	FCT/0478	FCT/0478/S/5	University of Abuja Teaching Hospital, Gwagwalada		Abuja	Gwagwalada	FCT
2354	FCT/0478	FCT/0478/S/2	University of Abuja Teaching Hospital, Gwagwalada		Abuja	Gwagwalada	FCT
2355	FCT/0478	FCT/0478/S/15	University of Abuja Teaching Hospital, Gwagwalada		Abuja	Gwagwalada	FCT
2356	FCT/0486	FCT/0486/S/5	Federal Road Safety Commission Medical Centre		Plot 18 Cotonou Crescent, Wuse Zone 6, Abuja	Municipal	FCT
2357	FCT/0486	FCT/0486/S/4	Federal Road Safety Commission Medical Centre		Plot 18 Cotonou Crescent, Wuse Zone 6, Abuja	Municipal	FCT
2358	FCT/0491	FCT/0491/S/4	Freedom Hospital & Health Consultants		405 Phase III Road, Kubwa	Bwari	FCT
2359	FCT/0495	FCT/0495/S/2	Allied Surgery		Off Specalist Hospital Road, Behind Charismatic Revival Church, Gwagwalada	Gwagwalada	FCT

2360	FCT/0495	FCT/0495/S/1	Allied Surgery		Off Specalist Hospital Road, Behind Charismatic Revival Church, Gwagwalada	Gwagwalada	FCT
2361	FCT/0495	FCT/0495/S/10	Allied Surgery		Off Specalist Hospital Road, Behind Charismatic Revival Church, Gwagwalada	Gwagwalada	FCT
2362	FCT/0495	FCT/0495/S/7	Allied Surgery		Off Specalist Hospital Road, Behind Charismatic Revival Church, Gwagwalada	Gwagwalada	FCT
2363	FCT/0495	FCT/0495/S/3	Allied Surgery		Off Specalist Hospital Road, Behind Charismatic Revival Church, Gwagwalada	Gwagwalada	FCT
2364	FCT/0498	FCT/0498/S/4	NIPRID Staff Clinic		NIPRID, Idu, Abuja	Municipal	FCT
2365	FCT/0498	FCT/0498/S/5	NIPRID Staff Clinic		NIPRID, Idu, Abuja	Municipal	FCT
2366	FCT/0510	FCT/0510/S/3	Northland Medical Centre		Okata House Off Old Karu Road, Nyanya, Abuja	Municipal	FCT
2367	FCT/0512	FCT/0512/S/8	Bethel Dental Clinic	Dental, Surgery	Nwora Plaza, Plot 1103 Aminu Kano Crescent, Wuse II, Abuja	Municipal	FCT
2368	FCT/0513	FCT/0513/S/5	Revenue Mobilization, Allocation and Fiscal Commission Staff	Laboratory	Plot 210 Tafawa Balewa Way, Central District, Abuja	Municipal	FCT
2369	FCT/0514	FCT/0514/S/4	ABM Pharmacy	Pharmacy	Shop 44 NYSC Camp Road, Kubwa,Abuja	Bwari	FCT
2370	FCT/0516	FCT/0516/S/5	Unique Medical Diagnosis Centre Laboratory	Laboratory	G.S.S. Road, Phase IV, Kubwa, Abuja	Bwari	FCT
2371	FCT/0518	FCT/0518/S/4	Tarei Pharmacy Ltd	Pharmacy	73, First Gate, Dutse Alhaji, Abuja	Bwari	FCT
2372	FCT/0521	FCT/0521/S/4	Brado Pharmacy	Pharmacy	17B Kwame Mkrumah Crescent, Asokoro	Municipal	FCT
2373	FCT/0525	FCT/0525/S/5	Rhemma Foundation Hospital, Kwali	Laboratory	Kwali	Kwali	FCT
2374	FCT/0526	FCT/0526/S/4	Rapy Galmac Pharmacy	Pharmacy	Shop 7, Secretariat Opposite Bank of the North, Kwali, Abuja	Kwali	FCT
2375	FCT/0527	FCT/0527/S/4	Cell Rich Pharmacy & Chemical Co. Ltd.	Pharmacy	Shop No. 7 Along General Hospital Road, Kuje, Abuja	Kuje	FCT
2376	FCT/0533	FCT/0533/S/13	Alpha Zone Eye Clinic	Optometry	Suite 17, Supreme Plaza Samuel Akintola Garki II	Municipal	FCT
2377	FCT/0534	FCT/0534/S/4	Ja'cane Pharmacy Ltd.	Pharmacy	Plot E49 Nsukka Street Garki Village, Abuja	Municipal	FCT
2378	FCT/0535	FCT/0535/S/4	Steps Pharmacy	Pharmacy	FHA Phase II, Abuja	Municipal	FCT
2379	FCT/0536	FCT/0536/S/4	OAK Chemical & Pharmacy Ltd.	Pharmacy	Plot 53 Adetokunbo Ademola Crescent, Wuse II, Abuja	Municipal	FCT
2380	FCT/0538	FCT/0538/S/4	A Bay Pharmacy	Pharmacy	Suite No 8 Sabond Ale Plaza Jabi, Abuja	Municipal	FCT
2381	FCT/0539	FCT/0539/S/5	Excel Medical Laboratory Services	Laboratory	Shop 110 Hill Side Area A, Nyanya, Abuja	Municipal	FCT
2382	FCT/0540	FCT/0540/S/3	Northerland Medical Laboratory	O&G	Okeph House Old Karu Road, Nyanya Village, Abuja	Municipal	FCT
2383	FCT/0546	FCT/0546/S/5	Garki Hospital		Area 8, Garki, Abuja	Municipal	FCT
2384	FCT/0546	FCT/0546/S/1	Garki Hospital		Area 8, Garki, Abuja	Municipal	FCT
2385	FCT/0546	FCT/0546/S/2	Garki Hospital		Area 8, Garki, Abuja	Municipal	FCT
2386	FCT/0546	FCT/0546/S/6	Garki Hospital		Area 8, Garki, Abuja	Municipal	FCT
2387	FCT/0546	FCT/0546/S/7	Garki Hospital		Area 8, Garki, Abuja	Municipal	FCT
2388	FCT/0546	FCT/0546/S/4	Garki Hospital		Area 8, Garki, Abuja	Municipal	FCT
2389	FCT/0546	FCT/0546/S/3	Garki Hospital		Area 8, Garki, Abuja	Municipal	FCT
2390	FCT/0550	FCT/0550/S/10	Hillcrest Clinics		19 Orau Street Off Nouakchott Street Wuse Zone 1	Municipal	FCT
2391	FCT/0550	FCT/0550/S/2	Hillcrest Clinics		19 Orau Street Off Nouakchott Street Wuse Zone 1	Municipal	FCT
2392	FCT/0550	FCT/0550/S/1	Hillcrest Clinics		19 Orau Street Off Nouakchott Street Wuse Zone 1	Municipal	FCT
2393	FCT/0553	FCT/0553/S/13	Locus Eye Clinic	Optometry	Plot 645 Alex Ekwueme Way, Jabi District, Abuja	Municipal	FCT
2394	FCT/0554	FCT/0554/S/12	Hi-Fi Clinics		40 Suez Crescent, Ibrahim Abacha Estate Wuse Zone 4	Municipal	FCT
2395	FCT/0556	FCT/0556/S/1	Fill Medical Centre		Plot 9 Gado Nasco Road, Phase ll site ll Kubwa	Bwari	FCT
2396	FCT/0556	FCT/0556/S/5	Fill Medical Centre		Plot 9 Gado Nasco Road, Phase ll site ll Kubwa	Bwari	FCT
2397	FCT/0560	FCT/0560/S/15	H J Eye Clinic and Optical Services	Opthamology	Wuse II, Abuja	Municipal	FCT

2398	FCT/0563	FCT/0563/S/15	Fortress Hospital		8 Gender Close, Off Gender Street, Wuse Zone 1	Municipal	FCT
2399	FCT/0564	FCT/0564/S/11	Maj Physiotherapy & Rehabilitation	Physiotherapy	44 Oran Street, near Habib Bank Wuse Zone1	Municipal	FCT
2400	FCT/0565	FCT/0565/S/15	Peniel Eye Clinic	Opthamology	Plot 541 Constitution Avenue, Behind ICPC, Central Business District	Municipal	FCT
2401	FCT/0568	FCT/0568/S/5	University of Abuja Medical Centre, Abuja	CLINIC	GWAGWALADA LGA	Gwagwalada	FCT
2402	FCT/0568	FCT/0568/S/4	University of Abuja Medical Centre, Abuja	CLINIC	GWAGWALADA LGA	Gwagwalada	FCT
2403	FCT/0573	FCT/0573/S/2	Kelina Medical Center		116, 3rd Avenue, Gwarinpa Estate	Municipal	FCT
2404	FCT/0573	FCT/0573/S/5	Kelina Medical Center		116, 3rd Avenue, Gwarinpa Estate	Municipal	FCT
2405	FCT/0573	FCT/0573/S/4	Kelina Medical Center		116, 3rd Avenue, Gwarinpa Estate	Municipal	FCT
2406	FCT/0573	FCT/0573/S/1	Kelina Medical Center		116, 3rd Avenue, Gwarinpa Estate	Municipal	FCT
2407	FCT/0573	FCT/0573/S/12	Kelina Medical Center		116, 3rd Avenue, Gwarinpa Estate	Municipal	FCT
2408	FCT/0573	FCT/0573/S/3	Kelina Medical Center		116, 3rd Avenue, Gwarinpa Estate	Municipal	FCT
2409	FCT/0573	FCT/0573/S/6	Kelina Medical Center		116, 3rd Avenue, Gwarinpa Estate	Municipal	FCT
2410	FCT/0575	FCT/0575/S/7	Clinic Care Medical Services		35 Nouakchott Street, Wuse Zone 1	Municipal	FCT
2411	FCT/0575	FCT/0575/S/2	Clinic Care Medical Services		35 Nouakchott Street, Wuse Zone 1	Municipal	FCT
2412	FCT/0575	FCT/0575/S/5	Clinic Care Medical Services		35 Nouakchott Street, Wuse Zone 1	Municipal	FCT
2413	FCT/0575	FCT/0575/S/1	Clinic Care Medical Services		35 Nouakchott Street, Wuse Zone 1	Municipal	FCT
2414	FCT/0581	FCT/0581/S/4	Estal Pharmacy Limited	Pharmacy	Shop 10 Blessed Plaza, Opp Custom Quarters Karu	Municipal	FCT
2415	FCT/0584	FCT/0584/S/4	Sisters of Nativity Hospital		Jikwoyi Phase 1, Behind Asaka Cement	Municipal	FCT
2416	FCT/0584	FCT/0584/S/3	Sisters of Nativity Hospital		Jikwoyi Phase 1, Behind Asaka Cement	Municipal	FCT
2417	FCT/0584	FCT/0584/S/5	Sisters of Nativity Hospital		Jikwoyi Phase 1, Behind Asaka Cement	Municipal	FCT
2418	FCT/0587	FCT/0587/S/13	De Lens Ophthalmic	Optometry	Suite G14 Ground floor, Febson Mall, Wuse Zone 4, Abuja	Municipal	FCT
2419	FCT/0591	FCT/0591/S/13	Maxrings Eye Clinic	Optometry	Suite BS 108 Old Banex Plaza, Wuse ll, Abuja	Municipal	FCT
2420	FCT/0592	FCT/0592/S/13	More Eye Clinic	Optometry	Plot 3162B IBB Way, Opposite BPE, Maitama	Municipal	FCT
2421	FCT/0593	FCT/0593/S/1	Lifeway Medical Centre		60 Harper Street, Wuse Zone 7	Municipal	FCT
2422	FCT/0593	FCT/0593/S/2	Lifeway Medical Centre		60 Harper Street, Wuse Zone 7	Municipal	FCT
2423	FCT/0593	FCT/0593/S/5	Lifeway Medical Centre		60 Harper Street, Wuse Zone 7	Municipal	FCT
2424	FCT/0593	FCT/0593/S/6	Lifeway Medical Centre		60 Harper Street, Wuse Zone 7	Municipal	FCT
2425	FCT/0593	FCT/0593/S/3	Lifeway Medical Centre		60 Harper Street, Wuse Zone 7	Municipal	FCT
2426	FCT/0595	FCT/0595/S/5	Kubwa Muslim Community Hospital (KMC)		Phase 4, Opposite Jumat Mosque Kubwa	Bwari	FCT
2427	FCT/0595	FCT/0595/S/4	Kubwa Muslim Community Hospital (KMC)		Phase 4, Opposite Jumat Mosque Kubwa	Bwari	FCT
2428	FCT/0600	FCT/0600/S/3	Royal Specialist Hospital		2 Kukawa Street, off Gimbiya Street, Area 11, Garki	Municipal	FCT
2429	FCT/0600	FCT/0600/S/7	Royal Specialist Hospital		2 Kukawa Street, off Gimbiya Street, Area 11, Garki	Municipal	FCT
2430	FCT/0600	FCT/0600/S/6	Royal Specialist Hospital		2 Kukawa Street, off Gimbiya Street, Area 11, Garki	Municipal	FCT
2431	FCT/0600	FCT/0600/S/1	Royal Specialist Hospital		2 Kukawa Street, off Gimbiya Street, Area 11, Garki	Municipal	FCT
2432	FCT/0600	FCT/0600/S/2	Royal Specialist Hospital		2 Kukawa Street, off Gimbiya Street, Area 11, Garki	Municipal	FCT
2433	FCT/0600	FCT/0600/S/5	Royal Specialist Hospital		2 Kukawa Street, off Gimbiya Street, Area 11, Garki	Municipal	FCT
2434	FCT/0601	FCT/0601/S/3	Tolbert Specialists Hospitals Ltd		3 Substation Close off Constitution Avenue, Gaduwa Estate	Municipal	FCT

2435	FCT/0602	FCT/0602/S/7	Prime Scan Medical Services Limited		B13, Amac Plaza, Opp. Heritage House, Zone 3, Wuse	Municipal	FCT
2436	FCT/0602	FCT/0602/S/5	Prime Scan Medical Services Limited		B13, Amac Plaza, Opp. Heritage House, Zone 3, Wuse	Municipal	FCT
2437	FCT/0606	FCT/0606/S/13	Rayfields Eye Care Limited		Suite BA5 First Floor, Apo Sparklight Shopping Mall, Mohammed Umar Lane, Opp Living Faith Church, Durumi	Municipal	FCT
2438	FCT/0610	FCT/0610/S/5	Rubochi General Hospital		Rubochi Town	Municipal	FCT
2439	FCT/0610	FCT/0610/S/1	Rubochi General Hospital		Rubochi Town	Municipal	FCT
2440	FCT/0610	FCT/0610/S/4	Rubochi General Hospital		Rubochi Town	Municipal	FCT
2441	FCT/0610	FCT/0610/S/3	Rubochi General Hospital		Rubochi Town	Municipal	FCT
2442	FCT/0611	FCT/0611/S/5	Samto Medical Services		4th/2nd Avenue, Supreme Court Quarters, Karu	Municipal	FCT
2443	FCT/0612	FCT/0612/S/5	Family Care Multiclinics		9, Lord Lugard Street, Asokoro	Municipal	FCT
2444	FCT/0612	FCT/0612/S/1	Family Care Multiclinics		9, Lord Lugard Street, Asokoro	Municipal	FCT
2445	FCT/0612	FCT/0612/S/3	Family Care Multiclinics		9, Lord Lugard Street, Asokoro	Municipal	FCT
2446	FCT/0612	FCT/0612/S/7	Family Care Multiclinics		9, Lord Lugard Street, Asokoro	Municipal	FCT
2447	FCT/0614	FCT/0614/S/1	Wellington Clinics Limited		10 Vanem Street, off Euphrates Street, Maitama	Municipal	FCT
2448	FCT/0614	FCT/0614/S/5	Wellington Clinics Limited		10 Vanem Street, off Euphrates Street, Maitama	Municipal	FCT
2449	FCT/0614	FCT/0614/S/7	Wellington Clinics Limited		10 Vanem Street, off Euphrates Street, Maitama	Municipal	FCT
2450	FCT/0615	FCT/0615/S/3	Vintage Medical Centre		F Road, CITEC Estate, Mbora District	Municipal	FCT
2451	FCT/0615	FCT/0615/S/2	Vintage Medical Centre		F Road, CITEC Estate, Mbora District	Municipal	FCT
2452	FCT/0615	FCT/0615/S/6	Vintage Medical Centre		F Road, CITEC Estate, Mbora District	Municipal	FCT
2453	FCT/0615	FCT/0615/S/1	Vintage Medical Centre		F Road, CITEC Estate, Mbora District	Municipal	FCT
2454	FCT/0616	FCT/0616/S/5	Crystal Diagnostic & Care Limited		25 Niamey Street, off Ouagadougou, Wuse Zone 2	Municipal	FCT
2455	FCT/0616	FCT/0616/S/7	Crystal Diagnostic & Care Limited		25 Niamey Street, off Ouagadougou, Wuse Zone 2	Municipal	FCT
2456	FCT/0619	FCT/0619/S/5	Medimax Hospital		11, 612 Road (7th Avenue) Gwarinpa	Municipal	FCT
2457	FCT/0619	FCT/0619/S/3	Medimax Hospital		11, 612 Road (7th Avenue) Gwarinpa	Municipal	FCT
2458	FCT/0620	FCT/0620/S/10	Supreme Clinic, Orthopaedic/Trauma		15 Lingu Crescent, off Aminu Kano Crescent, Wuse 2	Municipal	FCT
2459	FCT/0621	FCT/0621/S/6	Seraph Medical Practice		46, 3rd Avenue, EFAB Estate, Mbora, Life Camp	Municipal	FCT
2460	FCT/0626	FCT/0626/S/8	Excel Specialist Dental Clinic		Suite 17, Phase 1 Corner Shop, Gwagwalada	Gwagwalada	FCT
2461	FCT/0629	FCT/0629/S/13	Aiso Vision Centre		Suite 310B, DBM Plaza, Aminu Kano Crescent, Wuse 2	Municipal	FCT
2462	FCT/0630	FCT/0630/S/13	True Sight Enterprises		Suite 17, First Floor, Tolse Plaza, Lawan Gwadabe Crescent, Zone E Apo Legislative Qtrs	Municipal	FCT
2463	FCT/0633	FCT/0633/S/8	Alpha Hills Dental Clinic		Plot 22 Algiers Street, Zone 5, Wuse	Municipal	FCT
2464	FCT/0634	FCT/0634/S/7	Atlas Diagnostic Center		48 Akintola Boulevard, Opp Monday Markrt, Garki 2	Municipal	FCT
2465	FCT/0634	FCT/0634/S/5	Atlas Diagnostic Center		48 Akintola Boulevard, Opp Monday Markrt, Garki 2	Municipal	FCT
2466	FCT/0637	FCT/0637/S/14	Body Check International		Abraham Plaza, Plot 6 Ehukinam Street, beside ABC Transport Company, Utako	Municipal	FCT
2467	FCT/0638	FCT/0638/S/1	EFAB Medical Centre		28, 2nd Avenue (Adjacent to Mosque) RFAB City Estate, Mbora 1, Life Camp	Municipal	FCT
2468	FCT/0639	FCT/0639/S/3	Zenith Medical and Kidney Centre		28 Ubiaja Crescent, Garki 2	Municipal	FCT

2469	FCT/0639	FCT/0639/S/5	Zenith Medical and Kidney Centre		28 Ubiaja Crescent, Garki 2	Municipal	FCT
2470	FCT/0639	FCT/0639/S/2	Zenith Medical and Kidney Centre		28 Ubiaja Crescent, Garki 2	Municipal	FCT
2471	FCT/0640	FCT/0640/S/5	New Edge Medical Lab		Room 209, Ansar Plaza by Berger Junction Mpape, Abuja	Municipal	FCT
2472	FCT/0641	FCT/0641/S/5	New Edge Health Services Limited		1st Floor, Suite 18, Wing B, Deo Gratias Plaza, Opp Utako Market	Municipal	FCT
2473	FCT/0643	FCT/0643/S/13	Bridge of Sight Consult Limited		Plor 83, Road 21, Phase 2, FHA Lugbe	Municipal	FCT
2474	FCT/0645	FCT/0645/S/3	Open Healthcare Ltd	PP, O & G	11, Ademola Awosika Road, Kubwa Ext. 3, off Bwari-Dutse Expressway, Abuja	Municipal	FCT
2475	FCT/0646	FCT/0646/S/8	Evening Light Clinics		Suite CDS 5, 1st Floor, Harmonic Plaza, Mike Akhigbe way (near) Apostolic Faith Church) Jabi, Abuja.	Municipal	FCT
2476	FCT/0647	FCT/0647/S/13	Maldor Eyecare Center & Optical Services Ltd		Suite CSF 04, K-City Plaza, Plot 528 Aminu Kano crescent, Wuse 2 Abuja	Municipal	FCT
2477	FCT/0648	FCT/0648/S/4	Jethro Pharmacy and Stores		90, Ademola Adetokunbo crescent, Wuse 2, Abuja	Municipal	FCT
2478	FCT/0649	FCT/0649/S/9	Synapse Centre for Psycological Medicine		24, Bujumburu Street, Off Aminu Kano Crescent, Wuse 2, Abuja	Municipal	FCT
2479	FCT/0650	FCT/0650/S/1	Salama Infirmary Hospital and Maternity		No 24, 64 crescent, After Chaley Boy Boulevard, Gwarinpa	Municipal	FCT
2480	FCT/0650	FCT/0650/S/3	Salama Infirmary Hospital and Maternity		No 24, 64 crescent, After Chaley Boy Boulevard, Gwarinpa	Municipal	FCT
2481	FCT/0650	FCT/0650/S/2	Salama Infirmary Hospital and Maternity		No 24, 64 crescent, After Chaley Boy Boulevard, Gwarinpa	Municipal	FCT
2482	FCT/0651	FCT/0651/S/1	Chivar Clinics and Urology Center		30, Agadez street, off Aminu Kano crescent, Wuse 2, Abuja	Municipal	FCT
2483	FCT/0651	FCT/0651/S/16	Chivar Clinics and Urology Center		30, Agadez street, off Aminu Kano crescent, Wuse 2, Abuja	Municipal	FCT
2484	FCT/0651	FCT/0651/S/14	Chivar Clinics and Urology Center		30, Agadez street, off Aminu Kano crescent, Wuse 2, Abuja	Municipal	FCT
2485	FCT/0651	FCT/0651/S/17	Chivar Clinics and Urology Center		30, Agadez street, off Aminu Kano crescent, Wuse 2, Abuja	Municipal	FCT
2486	FCT/0651	FCT/0651/S/2	Chivar Clinics and Urology Center		30, Agadez street, off Aminu Kano crescent, Wuse 2, Abuja	Municipal	FCT
2487	FCT/0651	FCT/0651/S/5	Chivar Clinics and Urology Center		30, Agadez street, off Aminu Kano crescent, Wuse 2, Abuja	Municipal	FCT
2488	FCT/0652	FCT/0652/S/13	Zikora Hospital		Polt 16A, End of 69 road (Off 6th Avenue - Galadima Estate) Gwarinpa, Abuja	Municipal	FCT
2489	FCT/0653	FCT/0653/S/1	Primus International Super Specialist Hospital		Karu New Extension, Behind Customs Quarters, Karu, Abuja	Municipal	FCT
2490	FCT/0653	FCT/0653/S/7	Primus International Super Specialist Hospital		Karu New Extension, Behind Customs Quarters, Karu, Abuja	Municipal	FCT
2491	FCT/0653	FCT/0653/S/4	Primus International Super Specialist Hospital		Karu New Extension, Behind Customs Quarters, Karu, Abuja	Municipal	FCT
2492	FCT/0653	FCT/0653/S/5	Primus International Super Specialist Hospital		Karu New Extension, Behind Customs Quarters, Karu, Abuja	Municipal	FCT
2493	FCT/0653	FCT/0653/S/10	Primus International Super Specialist Hospital		Karu New Extension, Behind Customs Quarters, Karu, Abuja	Municipal	FCT
2494	FCT/0655	FCT/0655/S/5	Penthouse Clinics Ltd		Plot 170, Kado Karimu Rd, after Kado Fish Market, Abuja – FCT.	Municipal	FCT
2495	FCT/0657	FCT/0657/S/12	Fountain sides Hospital		Plot 73A 1st Avenue opp. Bonny B Corner Shop Gwarimpa Abuja	Municipal	FCT
2496	FCT/0657	FCT/0657/S/1	Fountain sides Hospital		Plot 73A 1st Avenue opp. Bonny B Corner Shop Gwarimpa Abuja	Municipal	FCT
2497	FCT/0657	FCT/0657/S/3	Fountain sides Hospital		Plot 73A 1st Avenue opp. Bonny B Corner Shop Gwarimpa Abuja	Municipal	FCT

2498	FCT/0657	FCT/0657/S/10	Fountain sides Hospital		Plot 73A 1st Avenue opp. Bonny B Corner Shop Gwarimpa Abuja	Municipal	FCT
2499	FCT/0658	FCT/0658/S/10	Cedacrest Hospital		Plot 195 Apo-Dutse way off. Oladipo Diya way, by Amina Court Estate, Gudu Abuja	Municipal	FCT
2500	FCT/0658	FCT/0658/S/1	Cedacrest Hospital		Plot 195 Apo-Dutse way off. Oladipo Diya way, by Amina Court Estate, Gudu Abuja	Municipal	FCT
2501	FCT/0659	FCT/0659/S/5	Cornelian Maternity & Rural Healthcare Centre		Holy Child Lane, Opposite Loyola Jesuit Maingate, Gidan Mangoro, Abuja-FCT.	Municipal	FCT
2502	FCT/0659	FCT/0659/S/2	Cornelian Maternity & Rural Healthcare Centre		Holy Child Lane, Opposite Loyola Jesuit Maingate, Gidan Mangoro, Abuja-FCT.	Municipal	FCT
2503	FCT/0659	FCT/0659/S/3	Cornelian Maternity & Rural Healthcare Centre		Holy Child Lane, Opposite Loyola Jesuit Maingate, Gidan Mangoro, Abuja-FCT.	Municipal	FCT
2504	FCT/0659	FCT/0659/S/1	Cornelian Maternity & Rural Healthcare Centre		Holy Child Lane, Opposite Loyola Jesuit Maingate, Gidan Mangoro, Abuja-FCT.	Municipal	FCT
2505	FCT/0660	FCT/0660/S/13	Icon Eye Clinic		Suite B16, Shakir Plaza, by Assemblies of God, Area 11, Garki, Abuja, FCT	Municipal	FCT
2506	FCT/0663	FCT/0663/S/1	Zaman Clinic & Maternity		25 Road 21B, Babangida Rd. FHA, Lugbe, Abuja	Municipal	FCT
2507	FCT/0663	FCT/0663/S/3	Zaman Clinic & Maternity		25 Road 21B, Babangida Rd. FHA, Lugbe, Abuja	Municipal	FCT
2508	FCT/0665	FCT/0665/S/1	Vic-James Hospital		Plot 211, Cadastral Zone 07-05, Gbazango Extension, Kubwa, Abuja	Bwari	FCT
2509	FCT/0666	FCT/0666/S/8	QH Specialist Dental Clinic and Resource Centre		Suite C3, Sysak Plaza, Opp Redeemed Church, Road 5213, Off 1st Avenue, Gwarinpa, Abuja	Municipal	FCT
2510	FCT/0667	FCT/0667/S/5	Nigerian Security and Civil Defence Corps Medical Center		Sauka Academy (Beside Immigration Passport Office), off Airport Road, Abuja.	Municipal	FCT
2511	FCT/0667	FCT/0667/S/4	Nigerian Security and Civil Defence Corps Medical Center		Sauka Academy (Beside Immigration Passport Office), off Airport Road, Abuja.	Municipal	FCT
2512	FCT/0671	FCT/0671/S/11	Optimal Health Centre International Limited		4 Bawku Street, Off Morovia Street, Wuse 2, Abuja	Municipal	FCT
2513	FCT/0674	FCT/0674/S/3	Louis-Pastuer Hospital LTD		813C, 64 Crescent, Gwarinpa 2 Estate, Map Global Junction, Abuja	Municipal	FCT
2514	FCT/0674	FCT/0674/S/4	Louis-Pastuer Hospital LTD		813C, 64 Crescent, Gwarinpa 2 Estate, Map Global Junction, Abuja	Municipal	FCT
2515	FCT/0674	FCT/0674/S/6	Louis-Pastuer Hospital LTD		813C, 64 Crescent, Gwarinpa 2 Estate, Map Global Junction, Abuja	Municipal	FCT
2516	FCT/0674	FCT/0674/S/1	Louis-Pastuer Hospital LTD		813C, 64 Crescent, Gwarinpa 2 Estate, Map Global Junction, Abuja	Municipal	FCT
2517	FCT/0676	FCT/0676/S/14	LeahJada-Joseph Medical Centre		Plot 7, Chris Chukwukelu Street, Behind Central Mosque, Gwagwalada, F.C.T	Gwagwalada	FCT
2518	FCT/0676	FCT/0676/S/5	LeahJada-Joseph Medical Centre		Plot 7, Chris Chukwukelu Street, Behind Central Mosque, Gwagwalada, F.C.T	Gwagwalada	FCT
2519	FCT/0677	FCT/0677/S/4	Ages Pharmacy &Stores		Plot 15, Phase 2, Kurudu Army Estate, Kurudu, F.C.T	Municipal	FCT
2520	FCT/0679	FCT/0679/S/2	Jehova Adonai Eagle Hospital		314, Street B, Phase 1, Gwagwalada, F.C.T	Gwagwalada	FCT
2521	FCT/0679	FCT/0679/S/3	Jehova Adonai Eagle Hospital		314, Street B, Phase 1, Gwagwalada, F.C.T	Gwagwalada	FCT
2522	FCT/0680	FCT/0680/S/6	Joyland Medical Centre & Children Hospital		Main Close, Valencia Garden Estate, Dakwo District, F.C.T	Municipal	FCT
2523	FCT/0682	FCT/0682/S/13	Atlanta Eye Clinic		Suite 1, Cherry Hill Plaza, Eke A. Yusuf Close, Utako, Abuja	Municipal	FCT
2524	FCT/0684	FCT/0684/S/13	Opticare Limited		Suite 1A, Macluma Plaza, 2 Mambolo Close, Wuse 2, F.C.T	Municipal	FCT

2525	FCT/0686	FCT/0686/S/13	Davik Eye Clinic		Plot 114/115 Phase IV, Mami Road Nyanya, F.C.T	Municipal	FCT
2526	FCT/0687	FCT/0687/S/8	Dazzle Dental Clinics LTD		Suite FC 14 Banex Plaza, Plot 750, Aminu Kano Crescent, Zone A7, Wuse 2, Abuja	Municipal	FCT
2527	FCT/0689	FCT/0689/S/13	Princess Vision Eye Clinic Limited		Suite 12A Anon Plaza, Besides NNPC Filling Station, Gudu, F.C.T	Municipal	FCT
2528	FCT/0690	FCT/0690/S/4	Nigeria Atomic Energy Commission Staff Clinic		SHETCO Complex, Sheda, F.C.T	Kwali	FCT
2529	FCT/0691	FCT/0691/S/13	NuVision Eye Clinic		Suite A36/A52 Area 11 Shopping Mall (Efab Mall), Gasrki, F.C.T	Municipal	FCT
2530	FCT/0692	FCT/0692/S/5	Decency Amana Medical Lab.		White House, Opposite Assemblies of God Church, Karshi, F.C.T	Municipal	FCT
2531	FCT/0693	FCT/0693/S/3	The Comforters Place		Plot 12 Democracy Crescent, Gaduwa Estate, F.C.T	Municipal	FCT
2532	FCT/0695	FCT/0695/S/2	Guinea Savannah Medical Center		Communal Center, NNPC Housing Estate Area 2,Garki	Municipal	FCT
2533	FCT/0695	FCT/0695/S/1	Guinea Savannah Medical Center		Communal Center, NNPC Housing Estate Area 2,Garki	Municipal	FCT
2534	FCT/0695	FCT/0695/S/3	Guinea Savannah Medical Center		Communal Center, NNPC Housing Estate Area 2,Garki	Municipal	FCT
2535	FCT/0695	FCT/0695/S/6	Guinea Savannah Medical Center		Communal Center, NNPC Housing Estate Area 2,Garki	Municipal	FCT
2536	FCT/0696	FCT/0696/S/13	Atlanta Eye Clinic		Suite 1, Cherry Hill Plaza, Eke A. Yusuf Close, Utako, Abuja F.C.T	Municipal	FCT
2537	FCT/0700	FCT/0700/S/15	Florida Eye Clinic		Amma House Plot 432, Yakubu Bam Street (Opp. National Hospital) Abuja-FCT	Municipal	FCT
2538	FCT/0701	FCT/0701/S/8	Glow Dental Clinic		Suite 4 Highbury Plaza, GadaNasco Road, Kubuwa, Abuja	Bwari	FCT
2539	FCT/0702	FCT/0702/S/13	Happy Eye Vision Clinic.		40 Suarez Crescent, Ibrahim Abacha Estate Wuse Zone 4 Abuja FCT.	Municipal	FCT
2540	FCT/0703	FCT/0703/S/11	Fitness Global Consult And Physiotherapy Clinic		No. 20 Limpopo Street Off Yedseram Street, FHA-Maitama	Municipal	FCT
2541	FCT/0707	FCT/0707/S/2	Martin Luther- Obama Specialist Hospital.		51, Yellow House, Loyola Street, Opp. Loyola Jesuit College Staff Gate, Gidan Mangoro, Abuja F.C.T	Municipal	FCT
2542	FCT/0707	FCT/0707/S/1	Martin Luther- Obama Specialist Hospital.		51, Yellow House, Loyola Street, Opp. Loyola Jesuit College Staff Gate, Gidan Mangoro, Abuja F.C.T	Municipal	FCT
2543	FCT/0708	FCT/0708/S/8	Mouth-Mirror Dental Services Limi Hospital		Behind Independent Corrupt Practices Commission Central Business Area Abuja F.C.T	Municipal	FCT
2544	FCT/0710	FCT/0710/S/7	Lifebridge Medical Diagnostics Center		15A Yawuri Street, Garki 11 Abuja F.C.T	Municipal	FCT
2545	FCT/0710	FCT/0710/S/5	Lifebridge Medical Diagnostics Center		15A Yawuri Street, Garki 11 Abuja F.C.T	Municipal	FCT
2546	FCT/0710	FCT/0710/S/14	Lifebridge Medical Diagnostics Center		15A Yawuri Street, Garki 11 Abuja F.C.T	Municipal	FCT
2547	FCT/0711	FCT/0711/S/1	Modelcare Hospital		5, Jaba Close Off Dunukofia Str. By FCDA Minister`s Gate, Garki Area 11, Abuja F.C.T	Municipal	FCT
2548	FCT/0711	FCT/0711/S/6	Modelcare Hospital		5, Jaba Close Off Dunukofia Str. By FCDA Minister`s Gate, Garki Area 11, Abuja F.C.T	Municipal	FCT
2549	FCT/0711	FCT/0711/S/15	Modelcare Hospital		5, Jaba Close Off Dunukofia Str. By FCDA Minister`s Gate, Garki Area 11, Abuja F.C.T	Municipal	FCT
2550	FCT/0711	FCT/0711/S/2	Modelcare Hospital		5, Jaba Close Off Dunukofia Str. By FCDA Minister`s Gate, Garki Area 11, Abuja F.C.T	Municipal	FCT
2551	FCT/0711	FCT/0711/S/5	Modelcare Hospital		5, Jaba Close Off Dunukofia Str. By FCDA Minister`s Gate, Garki Area 11, Abuja F.C.T	Municipal	FCT

2552	FCT/0711	FCT/0711/S/3	Modelcare Hospital		5, Jaba Close Off Dunukofia Str. By FCDA Minister`s Gate, Garki Area 11, Abuja F.C.T	Municipal	FCT
2553	FCT/0711	FCT/0711/S/12	Modelcare Hospital		5, Jaba Close Off Dunukofia Str. By FCDA Minister`s Gate, Garki Area 11, Abuja F.C.T	Municipal	FCT
2554	FCT/0713	FCT/0713/S/13	Irolyn Optical Care Clinic		Suite 11, Opposite Kuje Area Council, Kuje Secretariat Road, Kuje F.C.T	Kuje	FCT
2555	FCT/0715	FCT/0715/S/19	Althea Specialty Clinics Ltd		4 Bima Close Off Otukpo Str. Off Gimbiya Str. Garki Area 11 Abuja F.C.T	Municipal	FCT
2556	FCT/0716	FCT/0716/S/7	Freedomscan Medical Centre		Area B, Last Road, Nyanya, Abuja F.C.T	Municipal	FCT
2557	FCT/0717	FCT/0717/S/8	Global Smile Dental Care Center		50, Ebitu Ukiwe Street, Jabi District, Abuja F.C.T	Municipal	FCT
2558	FCT/0719	FCT/0719/S/8	Kings And Queens Dental & Maxillofacial Clinic		Suite 1 Emmamac Plaza Near Radio House, Gwagwalada F.C.T	Gwagwalada	FCT
2559	FCT/0720	FCT/0720/S/13	Sight Haven Eye Clinic		17 Gwandu Str. Off Uke Crescent (Fereprod Med. Centre) Garki Area 11	Municipal	FCT
2560	FCT/0721	FCT/0721/S/5	Sisto Medical Services		Caxton Idowu Abayomi Shopping Plaza, Shop GA 22, Post Service Housing Estate, Phase 2, Kurudu Army Barracks, Abuja F.C.T	Municipal	FCT
2561	FCT/0722	FCT/0722/S/3	Fountain Trust Medical & Diagnostic Centre Ltd		No. 6 Pastor Enoch Adeboye Street Off 24 Crescent, 2nd Avenue Gwarimpa Estate, Abuja F.C.T	Municipal	FCT
2562	FCT/0723	FCT/0723/S/11	Rosemech Physiotherapy Ltd		(Garki Gen. Hospital) Tafawa Balawa Way Garki Area 8 Abuja F.C.T	Municipal	FCT
2563	FCT/0725	FCT/0725/S/7	Community Hospital & Diagnostic Centre		Plot 342 Kubwa Extension 111 Dutse Alhaji Abuja F.C.T	Bwari	FCT
2564	FCT/0725	FCT/0725/S/2	Community Hospital & Diagnostic Centre		Plot 342 Kubwa Extension 111 Dutse Alhaji Abuja F.C.T	Bwari	FCT
2565	FCT/0725	FCT/0725/S/6	Community Hospital & Diagnostic Centre		Plot 342 Kubwa Extension 111 Dutse Alhaji Abuja F.C.T	Bwari	FCT
2566	FCT/0725	FCT/0725/S/5	Community Hospital & Diagnostic Centre		Plot 342 Kubwa Extension 111 Dutse Alhaji Abuja F.C.T	Bwari	FCT
2567	FCT/0725	FCT/0725/S/1	Community Hospital & Diagnostic Centre		Plot 342 Kubwa Extension 111 Dutse Alhaji Abuja F.C.T	Bwari	FCT
2568	FCT/0725	FCT/0725/S/3	Community Hospital & Diagnostic Centre		Plot 342 Kubwa Extension 111 Dutse Alhaji Abuja F.C.T	Bwari	FCT
2569	FCT/0727	FCT/0727/S/13	The Eye Place		23, Ekukinam Street, Utako District Abuja F.C.T	Municipal	FCT
2570	FCT/0729	FCT/0729/S/1	Katameya First call Hospital Limited		Plot 167 Cadastral Zone B09 Beside Next Shopping Mall, Kado Kuchi, Abuja. F.C.T	Municipal	FCT
2571	FCT/0729	FCT/0729/S/5	Katameya First call Hospital Limited		Plot 167 Cadastral Zone B09 Beside Next Shopping Mall, Kado Kuchi, Abuja. F.C.T	Municipal	FCT
2572	FCT/0729	FCT/0729/S/3	Katameya First call Hospital Limited		Plot 167 Cadastral Zone B09 Beside Next Shopping Mall, Kado Kuchi, Abuja. F.C.T	Municipal	FCT
2573	FCT/0730	FCT/0730/S/5	Menovas Consultants Clinic		Plot 53 Road B, Bentell Villa Estate Gaduwa District, Abuja F.C.T	Municipal	FCT
2574	FCT/0732	FCT/0732/S/5	Jodi Medical Center		2 Almurie Omanze Street, Off Ladoke Akintola Boulevard, Garki 2, Abuja F.C.T	Municipal	FCT
2575	FCT/0732	FCT/0732/S/1	Jodi Medical Center		2 Almurie Omanze Street, Off Ladoke Akintola Boulevard, Garki 2, Abuja F.C.T	Municipal	FCT
2576	FCT/0732	FCT/0732/S/3	Jodi Medical Center		2 Almurie Omanze Street, Off Ladoke Akintola Boulevard, Garki 2, Abuja F.C.T	Municipal	FCT
2577	FCT/0733	FCT/0733/S/5	Queen Of All Saints Diagnostic & Medical Laboratory Ltd.		No. 17 Monbasa Street Wuse Zone 5 Abuja F.C.T	Municipal	FCT

2578	FCT/0735	FCT/0735/S/13	Through Eye Care Clinic		Plot 359 Obafemi Awolowo Way Or No.24 Ebitu Ukaiwe Street Jabi District Abuja F.C.T	Municipal	FCT
2579	FCT/0738	FCT/0738/S/7	Allied Surgery Limited		Phase 1 expansion along Teaching Hospital Road Behind Eco Bank, Gwagwalada	Gwagwalada	FCT
2580	FCT/0738	FCT/0738/S/2	Allied Surgery Limited		Phase 1 expansion along Teaching Hospital Road Behind Eco Bank, Gwagwalada	Gwagwalada	FCT
2581	FCT/0738	FCT/0738/S/3	Allied Surgery Limited		Phase 1 expansion along Teaching Hospital Road Behind Eco Bank, Gwagwalada	Gwagwalada	FCT
2582	FCT/0739	FCT/0739/S/2	Premier Heart Hospital and Clinics		45 Fourth Avenue F.H.A Gwarimpa Abuja	Municipal	FCT
2583	FCT/0739	FCT/0739/S/5	Premier Heart Hospital and Clinics		45 Fourth Avenue F.H.A Gwarimpa Abuja	Municipal	FCT
2584	FCT/0740	FCT/0740/S/11	Bamfak Rehab And Physiotherapy Limited		19 Dahiru Bauchi Crescent (12th Crescent), Off 1st Avenue, Federal Government Housing Estate, Gwarinpa, Abuja, FCT	Municipal	FCT
2585	FCT/0741	FCT/0741/S/1	Royal Orthopaedic Hospital and Medicare		28,Ubiaja Crescent, Garki 11,Abuja FCT	Municipal	FCT
2586	FCT/0741	FCT/0741/S/10	Royal Orthopaedic Hospital and Medicare		28,Ubiaja Crescent, Garki 11,Abuja FCT	Municipal	FCT
2587	FCT/0741	FCT/0741/S/7	Royal Orthopaedic Hospital and Medicare		28,Ubiaja Crescent, Garki 11,Abuja FCT	Municipal	FCT
2588	GM/0001	GM/0001/S/4	General Hospital Billiri		Along Yola Road,Gombe	Billiri	Gombe
2589	GM/0001	GM/0001/S/6	General Hospital Billiri		Along Yola Road,Gombe	Billiri	Gombe
2590	GM/0001	GM/0001/S/5	General Hospital Billiri		Along Yola Road,Gombe	Billiri	Gombe
2591	GM/0001	GM/0001/S/1	General Hospital Billiri		Along Yola Road,Gombe	Billiri	Gombe
2592	GM/0001	GM/0001/S/3	General Hospital Billiri		Along Yola Road,Gombe	Billiri	Gombe
2593	GM/0001	GM/0001/S/2	General Hospital Billiri		Along Yola Road,Gombe	Billiri	Gombe
2594	GM/0002	GM/0002/S/6	Federal Teaching Hospital, Gombe	Primary	Gombe	Gombe	Gombe
2595	GM/0002	GM/0002/S/1	Federal Teaching Hospital, Gombe	Primary	Gombe	Gombe	Gombe
2596	GM/0002	GM/0002/S/2	Federal Teaching Hospital, Gombe	Primary	Gombe	Gombe	Gombe
2597	GM/0002	GM/0002/S/5	Federal Teaching Hospital, Gombe	Primary	Gombe	Gombe	Gombe
2598	GM/0002	GM/0002/S/4	Federal Teaching Hospital, Gombe	Primary	Gombe	Gombe	Gombe
2599	GM/0002	GM/0002/S/8	Federal Teaching Hospital, Gombe	Primary	Gombe	Gombe	Gombe
2600	GM/0002	GM/0002/S/12	Federal Teaching Hospital, Gombe	Primary	Gombe	Gombe	Gombe
2601	GM/0002	GM/0002/S/13	Federal Teaching Hospital, Gombe	Primary	Gombe	Gombe	Gombe
2602	GM/0002	GM/0002/S/11	Federal Teaching Hospital, Gombe	Primary	Gombe	Gombe	Gombe
2603	GM/0002	GM/0002/S/3	Federal Teaching Hospital, Gombe	Primary	Gombe	Gombe	Gombe
2604	GM/0002	GM/0002/S/7	Federal Teaching Hospital, Gombe	Primary	Gombe	Gombe	Gombe
2605	GM/0003	GM/0003/S/5	Doma Hospital		5. Commercial Area,Gombe	Gombe	Gombe
2606	GM/0003	GM/0003/S/14	Doma Hospital		5. Commercial Area,Gombe	Gombe	Gombe
2607	GM/0003	GM/0003/S/7	Doma Hospital		5. Commercial Area,Gombe	Gombe	Gombe
2608	GM/0004	GM/0004/S/5	Miyelti Hospital		A.Y. Guest House, Dukku Road	Gombe	Gombe
2609	GM/0004	GM/0004/S/7	Miyelti Hospital		A.Y. Guest House, Dukku Road	Gombe	Gombe
2610	GM/0005	GM/0005/S/1	Yarma Memorial Hospital		Buba Shongo Quarters,Gombe	Gombe	Gombe
2611	GM/0005	GM/0005/S/2	Yarma Memorial Hospital		Buba Shongo Quarters,Gombe	Gombe	Gombe
2612	GM/0009	GM/0009/S/5	Musaba Medical Centre		Dukku Road	Gombe	Gombe
2613	GM/0010	GM/0010/S/1	Hamdala Specialist Clinic		Near Pantami Stadium,Gombe	Gombe	Gombe
2614	GM/0013	GM/0013/S/1	Sabana Specialist Hospital Ltd.		Federal Lowcost, Gombe	Gombe	Gombe

2615	GM/0013	GM/0013/S/4	Sabana Specialist Hospital Ltd.		Federal Lowcost, Gombe	Gombe	Gombe
2616	GM/0013	GM/0013/S/5	Sabana Specialist Hospital Ltd.		Federal Lowcost, Gombe	Gombe	Gombe
2617	GM/0014	GM/0014/S/1	Specialist Hospital, Jekadafari	Primary	Jekadafari	Gombe	Gombe
2618	GM/0014	GM/0014/S/6	Specialist Hospital, Jekadafari	Primary	Jekadafari	Gombe	Gombe
2619	GM/0014	GM/0014/S/3	Specialist Hospital, Jekadafari	Primary	Jekadafari	Gombe	Gombe
2620	GM/0014	GM/0014/S/8	Specialist Hospital, Jekadafari	Primary	Jekadafari	Gombe	Gombe
2621	GM/0014	GM/0014/S/9	Specialist Hospital, Jekadafari	Primary	Jekadafari	Gombe	Gombe
2622	GM/0025	GM/0025/S/5	General Hospital, Kaltungo		Kaltungo,Along Yola Road, Gombe	Kaltungo	Gombe
2623	GM/0025	GM/0025/S/6	General Hospital, Kaltungo		Kaltungo,Along Yola Road, Gombe	Kaltungo	Gombe
2624	GM/0025	GM/0025/S/14	General Hospital, Kaltungo		Kaltungo,Along Yola Road, Gombe	Kaltungo	Gombe
2625	GM/0025	GM/0025/S/4	General Hospital, Kaltungo		Kaltungo,Along Yola Road, Gombe	Kaltungo	Gombe
2626	GM/0025	GM/0025/S/7	General Hospital, Kaltungo		Kaltungo,Along Yola Road, Gombe	Kaltungo	Gombe
2627	GM/0025	GM/0025/S/2	General Hospital, Kaltungo		Kaltungo,Along Yola Road, Gombe	Kaltungo	Gombe
2628	GM/0025	GM/0025/S/1	General Hospital, Kaltungo		Kaltungo,Along Yola Road, Gombe	Kaltungo	Gombe
2629	GM/0025	GM/0025/S/3	General Hospital, Kaltungo		Kaltungo,Along Yola Road, Gombe	Kaltungo	Gombe
2630	GM/0026	GM/0026/S/4	Kumo General Hospital		Kumo L.G.A	Akko	Gombe
2631	GM/0026	GM/0026/S/3	Kumo General Hospital		Kumo L.G.A	Akko	Gombe
2632	GM/0026	GM/0026/S/1	Kumo General Hospital		Kumo L.G.A	Akko	Gombe
2633	GM/0028	GM/0028/S/5	Bimma Med. Centre		Fed. Lowcost, Gombe	Gombe	Gombe
2634	GM/0036	GM/0036/S/5	General Hospital Dukku		Dukku Town	Dukku	Gombe
2635	GM/0037	GM/0037/S/5	General Hospital Zambuk		Zambuk Town	Yalmaltu/ Deba	Gombe
2636	GM/0051	GM/0051/S	General Hospital, Kumo	O&G, Surgery, Paediatrics	Kumo, Gombe State	Akko	Gombe
2637	GM/0051	GM/0051/S/6	General Hospital, Kumo	O&G, Surgery, Paediatrics	Kumo, Gombe State	Akko	Gombe
2638	GM/0051	GM/0051/S/1	General Hospital, Kumo	O&G, Surgery, Paediatrics	Kumo, Gombe State	Akko	Gombe
2639	GM/0137	GM/0137/S/5	Madi Memorial Clinic and Maternity		Tunfure Investment Estate, Gombe	Akko	Gombe
2640	GM/0137	GM/0137/S/1	Madi Memorial Clinic and Maternity		Tunfure Investment Estate, Gombe	Akko	Gombe
2641	GM/0137	GM/0137/S/14	Madi Memorial Clinic and Maternity		Tunfure Investment Estate, Gombe	Akko	Gombe
2642	GM/0139	GM/0139/S/5	Madina Specialist Eye & medical Center Ltd.		Madina Specialist & Medical Centre Limited	Gombe	Gombe
2643	GM/0143	GM/0143/S/5	General Hospital Kashere		Kashere, Gombe State	Akko	Gombe
2644	GM/0143	GM/0143/S/4	General Hospital Kashere		Kashere, Gombe State	Akko	Gombe
2645	IM/0002	IM/0002/S/5	Corpus Christi(Monice Memorial) Hospital		Amuzi Mbaise	Ahiazu Mbaise	Imo
2646	IM/0004	IM/0004/S/1	Jasman Hospital Ltd.		Udo Ezinihitte Mbaise, Imo	Ezinihitte Mbaise	Imo
2647	IM/0009	IM/0009/S/8	General Hospital, Okigwe		Okigwe, Imo State	Okigwe (Okigwe)	Imo
2648	IM/0009	IM/0009/S/2	General Hospital, Okigwe		Okigwe, Imo State	Okigwe (Okigwe)	Imo
2649	IM/0009	IM/0009/S/4	General Hospital, Okigwe		Okigwe, Imo State	Okigwe (Okigwe)	Imo
2650	IM/0009	IM/0009/S/3	General Hospital, Okigwe		Okigwe, Imo State	Okigwe (Okigwe)	Imo
2651	IM/0009	IM/0009/S/5	General Hospital, Okigwe		Okigwe, Imo State	Okigwe (Okigwe)	Imo
2652	IM/0010	IM/0010/S/4	Dijon Pharmacy Ltd.	Pharmacy	No. Ubahu Road, Okigwe, Owerri	Okigwe (Okigwe)	Imo
2653	IM/0011	IM/0011/S/4	Chisom Pharmacy	Pharmacy	3, Umuchima Road, Okigwe, Owerri	Okigwe (Okigwe)	Imo
2654	IM/0012	IM/0012/S/6	State Teaching Hospital	General Surgery, O & G, Paediatrics, ENT, Ophthalmology, Dental, Laboratory, Pharmacy	Orlu, Imo State	Okigwe (Okigwe)	Imo

2655	IM/0012	IM/0012/S/15	State Teaching Hospital	General Surgery, O & G, Paediatrics, ENT, Ophthalmology, Dental, Laboratory, Pharmacy	Orlu, Imo State	Okigwe (Okigwe)	Imo
2656	IM/0012	IM/0012/S/5	State Teaching Hospital	General Surgery, O & G, Paediatrics, ENT, Ophthalmology, Dental, Laboratory, Pharmacy	Orlu, Imo State	Okigwe (Okigwe)	Imo
2657	IM/0012	IM/0012/S/1	State Teaching Hospital	General Surgery, O & G, Paediatrics, ENT, Ophthalmology, Dental, Laboratory, Pharmacy	Orlu, Imo State	Okigwe (Okigwe)	Imo
2658	IM/0012	IM/0012/S/3	State Teaching Hospital	General Surgery, O & G, Paediatrics, ENT, Ophthalmology, Dental, Laboratory, Pharmacy	Orlu, Imo State	Okigwe (Okigwe)	Imo
2659	IM/0012	IM/0012/S/12	State Teaching Hospital	General Surgery, O & G, Paediatrics, ENT, Ophthalmology, Dental, Laboratory, Pharmacy	Orlu, Imo State	Okigwe (Okigwe)	Imo
2660	IM/0012	IM/0012/S/8	State Teaching Hospital	General Surgery, O & G, Paediatrics, ENT, Ophthalmology, Dental, Laboratory, Pharmacy	Orlu, Imo State	Okigwe (Okigwe)	Imo
2661	IM/0012	IM/0012/S/4	State Teaching Hospital	General Surgery, O & G, Paediatrics, ENT, Ophthalmology, Dental, Laboratory, Pharmacy	Orlu, Imo State	Okigwe (Okigwe)	Imo
2662	IM/0013	IM/0013/S/3	Our Lady Of Mercy Hospital & Mat.		102/104 Royce Road, Owerri	Owerri West (Umuguma)	Imo
2663	IM/0015	IM/0015/S/3	First Concepts Hospital		105, Ikenegbu Layout, Owerri	Owerri Urban (Owerri)	Imo
2664	IM/0015	IM/0015/S/5	First Concepts Hospital		105, Ikenegbu Layout, Owerri	Owerri Urban (Owerri)	Imo
2665	IM/0017	IM/0017/S/1	Angboso Specialist Hospital		Plot P6 Work Layout, Owerri	Owerri Urban (Owerri)	Imo
2666	IM/0018	IM/0018/S/3	Umezuruike Hospital		21 Umezuruike Street, Owerri	Owerri Urban (Owerri)	Imo
2667	IM/0018	IM/0018/S/7	Umezuruike Hospital		21 Umezuruike Street, Owerri	Owerri Urban (Owerri)	Imo
2668	IM/0018	IM/0018/S/1	Umezuruike Hospital		21 Umezuruike Street, Owerri	Owerri Urban (Owerri)	Imo
2669	IM/0018	IM/0018/S/2	Umezuruike Hospital		21 Umezuruike Street, Owerri	Owerri Urban (Owerri)	Imo
2670	IM/0018	IM/0018/S/13	Umezuruike Hospital		21 Umezuruike Street, Owerri	Owerri Urban (Owerri)	Imo
2671	IM/0022	IM/0022/S/3	Ngozi Hospital & Maternity		Plot 72, Okigwe Road Layout, Owerri.	Owerri Urban (Owerri)	Imo
2672	IM/0024	IM/0024/S/1	Ezem Medical Centre		4 Osuji Street, Owerri	Owerri Urban (Owerri)	Imo
2673	IM/0024	IM/0024/S/5	Ezem Medical Centre		4 Osuji Street, Owerri	Owerri Urban (Owerri)	Imo
2674	IM/0024	IM/0024/S/3	Ezem Medical Centre		4 Osuji Street, Owerri	Owerri Urban (Owerri)	Imo
2675	IM/0029	IM/0029/S/3	St. Elizabeth Hospital		10 Govt. House/Shell Camp Road, Owerri, Imo State	Owerri Urban (Owerri)	Imo
2676	IM/0030	IM/0030/S/5	Akaraugo Hospital & Maternity		3, Egbukole Street, Ikenegbu, Imo State	Owerri Urban (Owerri)	Imo
2677	IM/0030	IM/0030/S/14	Akaraugo Hospital & Maternity		3, Egbukole Street, Ikenegbu, Imo State	Owerri Urban (Owerri)	Imo

2678	IM/0031	IM/0031/S/2	Life Spring Specialist Hospital		Plot 49, Okigwe Road Layout, Owerri, Imo State	Owerri Urban (Owerri)	Imo
2679	IM/0032	IM/0032/S/5	Jone Medical Centre		Plot 189 Ikenegbu Layout Extension, through Chukwuma Nwoha Street, Owerri, Imo Sate	Owerri Urban (Owerri)	Imo
2680	IM/0032	IM/0032/S/3	Jone Medical Centre		Plot 189 Ikenegbu Layout Extension, through Chukwuma Nwoha Street, Owerri, Imo Sate	Owerri Urban (Owerri)	Imo
2681	IM/0032	IM/0032/S/2	Jone Medical Centre		Plot 189 Ikenegbu Layout Extension, through Chukwuma Nwoha Street, Owerri, Imo Sate	Owerri Urban (Owerri)	Imo
2682	IM/0034	IM/0034/S/5	Ikenegbu Hospital & Maternity		Plot 11 & 13 Amadi Crescent, Okigwe Road Layout, Owerri, Imo	Owerri Urban (Owerri)	Imo
2683	IM/0034	IM/0034/S/4	Ikenegbu Hospital & Maternity		Plot 11 & 13 Amadi Crescent, Okigwe Road Layout, Owerri, Imo	Owerri Urban (Owerri)	Imo
2684	IM/0034	IM/0034/S/1	Ikenegbu Hospital & Maternity		Plot 11 & 13 Amadi Crescent, Okigwe Road Layout, Owerri, Imo	Owerri Urban (Owerri)	Imo
2685	IM/0034	IM/0034/S/6	Ikenegbu Hospital & Maternity		Plot 11 & 13 Amadi Crescent, Okigwe Road Layout, Owerri, Imo	Owerri Urban (Owerri)	Imo
2686	IM/0034	IM/0034/S/3	Ikenegbu Hospital & Maternity		Plot 11 & 13 Amadi Crescent, Okigwe Road Layout, Owerri, Imo	Owerri Urban (Owerri)	Imo
2687	IM/0034	IM/0034/S/2	Ikenegbu Hospital & Maternity		Plot 11 & 13 Amadi Crescent, Okigwe Road Layout, Owerri, Imo	Owerri Urban (Owerri)	Imo
2688	IM/0034	IM/0034/S/7	Ikenegbu Hospital & Maternity		Plot 11 & 13 Amadi Crescent, Okigwe Road Layout, Owerri, Imo	Owerri Urban (Owerri)	Imo
2689	IM/0035	IM/0035/S/3	St. David's Hospital		14, Mbari Street, Ikenegbu, Owerri, Imo State	Owerri Urban (Owerri)	Imo
2690	IM/0042	IM/0042/S/3	St. David's Hospital	O & G, Surgery, Radiology, Laboratory, ECG, Pharmacy, Int. medicine, Paediatrics,Ortho paedic surgery, Physiotherapy	14, Mbari Street, Ikenegbu, Owerri, Imo State	Owerri Urban (Owerri)	Imo
2691	IM/0047	IM/0047/S/4	Pharm. Sense Pharmacy	Pharmacy	MCC Road, Owerri, Imo State	Owerri Urban (Owerri)	Imo
2692	IM/0049	IM/0049/S/4	Choice Pharmacy	Pharmacy	4 Ihechiowa Street, Ikenegbu Ext. Owerri	Owerri Urban (Owerri)	Imo
2693	IM/0050	IM/0050/S/4	Pathway Pharmacy Ltd.	Pharmacy	Plot 170 Ikenegbu Layout, Owerri	Owerri Urban (Owerri)	Imo
2694	IM/0052	IM/0052/S/4	Little Wood Pharmacy Ltd.	Pharmacy	129, Royce Road, Owerri	Owerri Urban (Owerri)	Imo
2695	IM/0054	IM/0054/S/4	Drug Wyse Pharmacy Ltd.	Pharmacy	62, Wethdral Road, Owerri	Owerri Urban (Owerri)	Imo
2696	IM/0055	IM/0055/S/4	Adonai Pharmacy Ltd.	Pharmacy	125, Tetlow Road, Owerri	Owerri Urban (Owerri)	Imo
2697	IM/0056	IM/0056/S/4	Akali Pharmacy Ltd.	Pharmacy	34 Tetlow Road, Owerri	Owerri Urban (Owerri)	Imo
2698	IM/0060	IM/0060/S/5	New Cross Hospital Ltd.		5, New Cross Hospital Street, Ugwuorji, Owerri	Owerri North (Orie Uratta)	Imo
2699	IM/0061	IM/0061/S/3	Aladinma Hospital		Transfiguration Road, Owerri, Imo State	Owerri North (Orie Uratta)	Imo
2700	IM/0061	IM/0061/S/1	Aladinma Hospital		Transfiguration Road, Owerri, Imo State	Owerri North (Orie Uratta)	Imo
2701	IM/0064	IM/0064/S/4	Villa Pharm. Chem Ltd.	Pharmacy	18A Orlu Road, Owerri	Owerri Urban (Owerri)	Imo
2702	IM/0066	IM/0066/S/6	Uchenna Hospital & Maternity		10/12 Eniweruzoh Crescent Owerri	Owerri West (Umuguma)	Imo
2703	IM/0066	IM/0066/S/1	Uchenna Hospital & Maternity		10/12 Eniweruzoh Crescent Owerri	Owerri West (Umuguma)	Imo
2704	IM/0066	IM/0066/S/3	Uchenna Hospital & Maternity		10/12 Eniweruzoh Crescent Owerri	Owerri West (Umuguma)	Imo
2705	IM/0068	IM/0068/S/10	Christiana Specialist Hospital		Egbu/Emuoba Rd., Owerri	Owerri Urban (Owerri)	Imo
2706	IM/0070	IM/0070/S/1	Six C. Specialist Clinic		28/30 Orlu Road, Imo State	Owerri Urban (Owerri)	Imo
2707	IM/0073	IM/0073/S/2	Capital Health Hospital		44 Oyima Street,Owerri, Imo State	Owerri Urban (Owerri)	Imo
2708	IM/0075	IM/0075/S/8	General Hospital	General Surgery, O & G, Paediatrics, Dental, Laboratory, Pharmacy	New Owerri, Owerri, Imo State	Owerri Urban (Owerri)	Imo

2709	IM/0075	IM/0075/S/5	General Hospital	General Surgery, O & G, Paediatrics, Dental, Laboratory, Pharmacy	New Owerri, Owerri, Imo State	Owerri Urban (Owerri)	Imo
2710	IM/0075	IM/0075/S/4	General Hospital	General Surgery, O & G, Paediatrics, Dental, Laboratory, Pharmacy	New Owerri, Owerri, Imo State	Owerri Urban (Owerri)	Imo
2711	IM/0075	IM/0075/S/3	General Hospital	General Surgery, O & G, Paediatrics, Dental, Laboratory, Pharmacy	New Owerri, Owerri, Imo State	Owerri Urban (Owerri)	Imo
2712	IM/0075	IM/0075/S	General Hospital	General Surgery, O & G, Paediatrics, Dental, Laboratory, Pharmacy	New Owerri, Owerri, Imo State	Owerri Urban (Owerri)	Imo
2713	IM/0075	IM/0075/S/6	General Hospital	General Surgery, O & G, Paediatrics, Dental, Laboratory, Pharmacy	New Owerri, Owerri, Imo State	Owerri Urban (Owerri)	Imo
2714	IM/0081	IM/0081/S/6	Oliva Specialist Hospital	Paediatrics	Concord Village, Owerri, Imo State	Owerri Urban (Owerri)	Imo
2715	IM/0083	IM/0083/S/15	Floxy Pan-Opticum Eye Specialist Hosp.	Opthalmology	464, Tony Okeke Str., Prefab Owerri	Owerri Urban (Owerri)	Imo
2716	IM/0086	IM/0086/S/4	Oben Pharmacy Ltd.	Pharmacy	Emekuku, Km8, Emekuku Road	Ahiazu Mbaise	Imo
2717	IM/0093	IM/0093/S/1	Imo State Specialist Hospital, Owerri		New Owerri, Imo State	Owerri North (Orie Uratta)	Imo
2718	IM/0093	IM/0093/S/4	Imo State Specialist Hospital, Owerri		New Owerri, Imo State	Owerri North (Orie Uratta)	Imo
2719	IM/0093	IM/0093/S/5	Imo State Specialist Hospital, Owerri		New Owerri, Imo State	Owerri North (Orie Uratta)	Imo
2720	IM/0093	IM/0093/S/8	Imo State Specialist Hospital, Owerri		New Owerri, Imo State	Owerri North (Orie Uratta)	Imo
2721	IM/0093	IM/0093/S/6	Imo State Specialist Hospital, Owerri		New Owerri, Imo State	Owerri North (Orie Uratta)	Imo
2722	IM/0093	IM/0093/S/3	Imo State Specialist Hospital, Owerri		New Owerri, Imo State	Owerri North (Orie Uratta)	Imo
2723	IM/0094	IM/0094/S/2	Federal Medical Centre, Owerri		Owerri, Imo State	Owerri Urban (Owerri)	Imo
2724	IM/0094	IM/0094/S/5	Federal Medical Centre, Owerri		Owerri, Imo State	Owerri Urban (Owerri)	Imo
2725	IM/0094	IM/0094/S/7	Federal Medical Centre, Owerri		Owerri, Imo State	Owerri Urban (Owerri)	Imo
2726	IM/0094	IM/0094/S/4	Federal Medical Centre, Owerri		Owerri, Imo State	Owerri Urban (Owerri)	Imo
2727	IM/0094	IM/0094/S/11	Federal Medical Centre, Owerri		Owerri, Imo State	Owerri Urban (Owerri)	Imo
2728	IM/0094	IM/0094/S/10	Federal Medical Centre, Owerri		Owerri, Imo State	Owerri Urban (Owerri)	Imo
2729	IM/0094	IM/0094/S/8	Federal Medical Centre, Owerri		Owerri, Imo State	Owerri Urban (Owerri)	Imo
2730	IM/0094	IM/0094/S/12	Federal Medical Centre, Owerri		Owerri, Imo State	Owerri Urban (Owerri)	Imo
2731	IM/0094	IM/0094/S/6	Federal Medical Centre, Owerri		Owerri, Imo State	Owerri Urban (Owerri)	Imo
2732	IM/0094	IM/0094/S/1	Federal Medical Centre, Owerri		Owerri, Imo State	Owerri Urban (Owerri)	Imo
2733	IM/0094	IM/0094/S/3	Federal Medical Centre, Owerri		Owerri, Imo State	Owerri Urban (Owerri)	Imo
2734	IM/0094	IM/0094/S/15	Federal Medical Centre, Owerri		Owerri, Imo State	Owerri Urban (Owerri)	Imo
2735	IM/0100	IM/0100/S/2	Uchendu Hospital		98 Douglas Rd. Owerri	Owerri Urban (Owerri)	Imo
2736	IM/0105	IM/0105/S/5	Nkire Med. Diagnostic Lab.	Laboratory	Eke-Nguru-Aboh Road (Near Customary Court), Imo State	Aboh Mbaise	Imo
2737	IM/0108	IM/0108/S/4	Destiny Pharmacy	Pharmacy	Works Layout, Near IMSU Junction, Owerri	Owerri Urban (Owerri)	Imo
2738	IM/0112	IM/0112/S/5	Federal Polytechnic Medical Centre, Nekede		Nekede, Owerri, Imo State	Owerri West (Umuguma)	Imo
2739	IM/0112	IM/0112/S/4	Federal Polytechnic Medical Centre, Nekede		Nekede, Owerri, Imo State	Owerri West (Umuguma)	Imo
2740	IM/0115	IM/0115/S/5	Federal University of Technology Medical Centre		Ihiagwa, Owerri, Imo State	Owerri West (Umuguma)	Imo

2741	IM/0115	IM/0115/S/4	Federal University of Technology Medical Centre		Ihiagwa, Owerri, Imo State	Owerri West (Umuguma)	Imo
2742	IM/0115	IM/0115/S/1	Federal University of Technology Medical Centre		Ihiagwa, Owerri, Imo State	Owerri West (Umuguma)	Imo
2743	IM/0118	IM/0118/S/3	Amanda Hospital		Plot 412/415 Works Layout, Owerri, Imo State	Owerri North (Orie Uratta)	Imo
2744	IM/0118	IM/0118/S/5	Amanda Hospital		Plot 412/415 Works Layout, Owerri, Imo State	Owerri North (Orie Uratta)	Imo
2745	IM/0118	IM/0118/S/7	Amanda Hospital		Plot 412/415 Works Layout, Owerri, Imo State	Owerri North (Orie Uratta)	Imo
2746	IM/0124	IM/0124/S/1	International Christian Hospitals		11 WAAST AVENUE, IKENEGBU	Owerri Urban (Owerri)	Imo
2747	IM/0124	IM/0124/S/5	International Christian Hospitals		11 WAAST AVENUE, IKENEGBU	Owerri Urban (Owerri)	Imo
2748	IM/0125	IM/0125/S/3	EBUBEDIKE MEMORIAL HOSPITAL		71, OPARANOZIE STR. OWERRI	Owerri Urban (Owerri)	Imo
2749	IM/0129	IM/0129/S/2	ETITI MEDICAL CENTER		Etiti Isinweke Ihitte Owerri	Ihitte/Uboma (Isinweke)	Imo
2750	IM/0136	IM/0136/S/5	CHARLES MED. LAB.	Laboratory	8, NEW NO 4 MBAISE ROAD,OWERRI	Owerri Urban (Owerri)	Imo
2751	IM/0138	IM/0138/S/4	CORPUS CHRISTI(MONICA MEMORIAL) HOSPITAL	Laboratory	AMUZI AHIARA	Ahiazu Mbaise	Imo
2752	IM/0138	IM/0138/S/5	CORPUS CHRISTI(MONICA MEMORIAL) HOSPITAL	Laboratory	AMUZI AHIARA	Ahiazu Mbaise	Imo
2753	IM/0139	IM/0139/S/5	KENIKON MEDICAL DIAG	LAB.	PLOT 267, IKENEGBU L/OUT, OWERRI	Owerri Urban (Owerri)	Imo
2754	IM/0140	IM/0140/S/4	L'DUTY CHEMIST	Pharmacy	103, WETHERAL ROAD, OWERRI	Owerri Urban (Owerri)	Imo
2755	IM/0144	IM/0144/S/15	Niger Optical & Services	Opthalmology	3, IHIOMA ROAD, ORLU	Orlu	Imo
2756	IM/0146	IM/0146/S/15	RISLORI OPTICAL SERVICES & EYE CLINIC	Opthalmology	PLOT 271, IKENEGBU L/OUT, OWERRI	Owerri Urban (Owerri)	Imo
2757	IM/0151	IM/0151/S/15	Sheerah Vision Eye Clinic	Opthalmology	1 Umez Eronine Street, Owerri	Owerri Urban (Owerri)	Imo
2758	IM/0173	IM/0173/S/10	Imo State University Teaching Hospital Primary Health Centre		Orlu	Orlu	Imo
2759	IM/0185	IM/0185/S/15	Camella Eye Clinic	Opthalmology	126 Okigwe Road, Opp Summit Hotel, Owerri	Owerri Urban (Owerri)	Imo
2760	IM/0186	IM/0186/S/4	Milam Pharmacy	Pharmacy	4 Douglas Road, Owerri	Owerri Urban (Owerri)	Imo
2761	IM/0189	IM/0189/S/3	New Annex Specialist Hospital		Plot 989, Amakohia, Owerri	Owerri North (Orie Uratta)	Imo
2762	IM/0192	IM/0192/S/5	Step 1 Medical Laboratories		146, Royce Road, Owerri, Imo State	Owerri Urban (Owerri)	Imo
2763	IM/0194	IM/0194/S/5	Bettina Medical Centre	Laboratory, O & G	11 Old Okigwe Road, Owerri	Owerri Urban (Owerri)	Imo
2764	IM/0194	IM/0194/S/3	Bettina Medical Centre	Laboratory, O & G	11 Old Okigwe Road, Owerri	Owerri Urban (Owerri)	Imo
2765	IM/0196	IM/0196/S/7	Alvan Ikoku Medical Center		Owerri	Owerri Urban (Owerri)	Imo
2766	IM/0196	IM/0196/S/4	Alvan Ikoku Medical Center		Owerri	Owerri Urban (Owerri)	Imo
2767	IM/0196	IM/0196/S/5	Alvan Ikoku Medical Center		Owerri	Owerri Urban (Owerri)	Imo
2768	IM/0197	IM/0197/S/3	Vaden Specialist Clinic and Maternity		4B, Solid Rock road, off World Bank Road, Owerri	Owerri Urban (Owerri)	Imo
2769	IM/0198	IM/0198/S/15	St. Joseph's Eye Hospital		Mgbirichi, Ohaji Egbema, Imo State	Ohaji/Egbema (Mmahu-Egbema)	Imo
2770	IM/0200	IM/0200/S/5	Six Star Laboratory Ltd		No. 5, Anyanwuocha Street, Akwakuma, Owerri, Imo State	Owerri North (Orie Uratta)	Imo
2771	IM/0202	IM/0202/S/4	Triple A Pharmacy Plus		No. 9, Mere Street, Owerri, Imo State	Owerri Urban (Owerri)	Imo
2772	IM/0203	IM/0203/S/5	A C E Medical Laboratory & Diagnostic Centre		No. 9, Mere Street, Owerri, Imo State	Owerri Urban (Owerri)	Imo
2773	IM/0204	IM/0204/S/1	Steps of Faith Hospital		No. 9, Mere Street, Owerri, Imo State	Owerri Urban (Owerri)	Imo
2774	IM/0204	IM/0204/S/2	Steps of Faith Hospital		No. 9, Mere Street, Owerri, Imo State	Owerri Urban (Owerri)	Imo
2775	IM/0205	IM/0205/S/5	Human Race Specialist Medical Diagnostic Centre		Plot 111/112, Naze Industrial Estate, Naze, Owerri, Imo State	Owerri North (Orie Uratta)	Imo
2776	IM/0205	IM/0205/S/7	Human Race Specialist Medical Diagnostic Centre		Plot 111/112, Naze Industrial Estate, Naze, Owerri, Imo State	Owerri North (Orie Uratta)	Imo

2777	IM/0208	IM/0208/S/6	FamilyCare Specialist Clinics		New Owerri, Imo State	Owerri West (Umuguma)	Imo
2778	IM/0208	IM/0208/S/3	FamilyCare Specialist Clinics		New Owerri, Imo State	Owerri West (Umuguma)	Imo
2779	IM/0208	IM/0208/S/1	FamilyCare Specialist Clinics		New Owerri, Imo State	Owerri West (Umuguma)	Imo
2780	IM/0209	IM/0209/S/3	St. Damian's Hospital		Okporo Orlu, Imo State	Orlu	Imo
2781	IM/0209	IM/0209/S/5	St. Damian's Hospital		Okporo Orlu, Imo State	Orlu	Imo
2782	IM/0209	IM/0209/S/2	St. Damian's Hospital		Okporo Orlu, Imo State	Orlu	Imo
2783	IM/0212	IM/0212/S/3	Salvation Hospital and Maternity		Nwaorienkpu, Ekwe, Imo State	Isu (Umundugba)	Imo
2784	IM/0212	IM/0212/S/7	Salvation Hospital and Maternity		Nwaorienkpu, Ekwe, Imo State	Isu (Umundugba)	Imo
2785	IM/0213	IM/0213/S/8	Anneth Health Services		Plot 14 Civic Centre Ext.No 8 Athan Ogoh Avenue,New Owerri Imo State	Owerri Urban (Owerri)	Imo
2786	IM/0215	IM/0215/S/5	Digoe Hospital		5,Orlu Road Owerri Imo State	Owerri North (Orie Uratta)	Imo
2787	IM/0217	IM/0217/S/5	Digoe Hospital		5,Orlu Road Owerri Imo State	Owerri North (Orie Uratta)	Imo
2788	IM/0219	IM/0219/S/1	Zennia Hospital & Maternity		No 1 Road 17, Federal Housing Umuguma, Owerri Imo State	Owerri West (Umuguma)	Imo
2789	IM/0219	IM/0219/S/2	Zennia Hospital & Maternity		No 1 Road 17, Federal Housing Umuguma, Owerri Imo State	Owerri West (Umuguma)	Imo
2790	IM/0219	IM/0219/S/5	Zennia Hospital & Maternity		No 1 Road 17, Federal Housing Umuguma, Owerri Imo State	Owerri West (Umuguma)	Imo
2791	IM/0220	IM/0220/S/13	Silver Spring Eye Care Ltd		Plot 12 Ikenegbu Ext, Off MCC Road, By London Bus Stop, Owerri Imo State	Owerri Urban (Owerri)	Imo
2792	IM/0227	IM/0227/S/8	Patriot Dental Clinic		98A Okigwe Road, Owerri, Imo State	Owerri Urban (Owerri)	Imo
2793	JG/0001	JG/0001/S/3	General Hospital, Dutse		Dutse, Jigawa State	Dutse	Jigawa
2794	JG/0001	JG/0001/S/4	General Hospital, Dutse		Dutse, Jigawa State	Dutse	Jigawa
2795	JG/0001	JG/0001/S/2	General Hospital, Dutse		Dutse, Jigawa State	Dutse	Jigawa
2796	JG/0001	JG/0001/S/6	General Hospital, Dutse		Dutse, Jigawa State	Dutse	Jigawa
2797	JG/0001	JG/0001/S/5	General Hospital, Dutse		Dutse, Jigawa State	Dutse	Jigawa
2798	JG/0002	JG/0002/S/6	Jahun General Hospital		Jahun, Jigawa State	Jahun	Jigawa
2799	JG/0002	JG/0002/S/3	Jahun General Hospital		Jahun, Jigawa State	Jahun	Jigawa
2800	JG/0002	JG/0002/S/5	Jahun General Hospital		Jahun, Jigawa State	Jahun	Jigawa
2801	JG/0003	JG/0003/S/15	General Hospital, Hadejia		Hadejia, Jigawa State	Hadejia	Jigawa
2802	JG/0003	JG/0003/S/2	General Hospital, Hadejia		Hadejia, Jigawa State	Hadejia	Jigawa
2803	JG/0003	JG/0003/S/3	General Hospital, Hadejia		Hadejia, Jigawa State	Hadejia	Jigawa
2804	JG/0003	JG/0003/S/1	General Hospital, Hadejia		Hadejia, Jigawa State	Hadejia	Jigawa
2805	JG/0003	JG/0003/S/5	General Hospital, Hadejia		Hadejia, Jigawa State	Hadejia	Jigawa
2806	JG/0005	JG/0005/S/4	General Hospital, Gumel		Gumel, Jigawa State	Gumel	Jigawa
2807	JG/0005	JG/0005/S/15	General Hospital, Gumel		Gumel, Jigawa State	Gumel	Jigawa
2808	JG/0005	JG/0005/S/5	General Hospital, Gumel		Gumel, Jigawa State	Gumel	Jigawa
2809	JG/0005	JG/0005/S/3	General Hospital, Gumel		Gumel, Jigawa State	Gumel	Jigawa
2810	JG/0005	JG/0005/S/2	General Hospital, Gumel		Gumel, Jigawa State	Gumel	Jigawa
2811	JG/0005	JG/0005/S/1	General Hospital, Gumel		Gumel, Jigawa State	Gumel	Jigawa
2812	JG/0006	JG/0006/S/6	General Hospital, Kazaure		Kazaure, Jigawa State	Kazaure	Jigawa
2813	JG/0006	JG/0006/S/1	General Hospital, Kazaure		Kazaure, Jigawa State	Kazaure	Jigawa
2814	JG/0006	JG/0006/S/15	General Hospital, Kazaure		Kazaure, Jigawa State	Kazaure	Jigawa
2815	JG/0006	JG/0006/S/4	General Hospital, Kazaure		Kazaure, Jigawa State	Kazaure	Jigawa
2816	JG/0008	JG/0008/S/2	General Hospital, Ringim		Ringim, Jigawa State	Ringim	Jigawa
2817	JG/0008	JG/0008/S/6	General Hospital, Ringim		Ringim, Jigawa State	Ringim	Jigawa

2818	JG/0008	JG/0008/S/3	General Hospital, Ringim		Ringim, Jigawa State	Ringim	Jigawa
2819	JG/0008	JG/0008/S/5	General Hospital, Ringim		Ringim, Jigawa State	Ringim	Jigawa
2820	JG/0008	JG/0008/S/4	General Hospital, Ringim		Ringim, Jigawa State	Ringim	Jigawa
2821	JG/0009	JG/0009/S/12	Federal Medical Centre, Birnin Kudu		Brnin Kudu, Jigawa State	Birnin Kudu	Jigawa
2822	JG/0009	JG/0009/S/7	Federal Medical Centre, Birnin Kudu		Brnin Kudu, Jigawa State	Birnin Kudu	Jigawa
2823	JG/0009	JG/0009/S/11	Federal Medical Centre, Birnin Kudu		Brnin Kudu, Jigawa State	Birnin Kudu	Jigawa
2824	JG/0009	JG/0009/S/1	Federal Medical Centre, Birnin Kudu		Brnin Kudu, Jigawa State	Birnin Kudu	Jigawa
2825	JG/0009	JG/0009/S/8	Federal Medical Centre, Birnin Kudu		Brnin Kudu, Jigawa State	Birnin Kudu	Jigawa
2826	JG/0009	JG/0009/S/5	Federal Medical Centre, Birnin Kudu		Brnin Kudu, Jigawa State	Birnin Kudu	Jigawa
2827	JG/0009	JG/0009/S/2	Federal Medical Centre, Birnin Kudu		Brnin Kudu, Jigawa State	Birnin Kudu	Jigawa
2828	JG/0009	JG/0009/S/3	Federal Medical Centre, Birnin Kudu		Brnin Kudu, Jigawa State	Birnin Kudu	Jigawa
2829	JG/0009	JG/0009/S/6	Federal Medical Centre, Birnin Kudu		Brnin Kudu, Jigawa State	Birnin Kudu	Jigawa
2830	JG/0009	JG/0009/S/4	Federal Medical Centre, Birnin Kudu		Brnin Kudu, Jigawa State	Birnin Kudu	Jigawa
2831	JG/0009	JG/0009/S/15	Federal Medical Centre, Birnin Kudu		Brnin Kudu, Jigawa State	Birnin Kudu	Jigawa
2832	JG/0019	JG/0019/S/13	Police Clinics, Jigawa		Jigawa State	Dutse	Jigawa
2833	JG/0019	JG/0019/S/8	Police Clinics, Jigawa		Jigawa State	Dutse	Jigawa
2834	JG/0019	JG/0019/S/5	Police Clinics, Jigawa		Jigawa State	Dutse	Jigawa
2835	JG/0019	JG/0019/S/14	Police Clinics, Jigawa		Jigawa State	Dutse	Jigawa
2836	JG/0019	JG/0019/S/11	Police Clinics, Jigawa		Jigawa State	Dutse	Jigawa
2837	JG/0019	JG/0019/S/1	Police Clinics, Jigawa		Jigawa State	Dutse	Jigawa
2838	JG/0019	JG/0019/S/7	Police Clinics, Jigawa		Jigawa State	Dutse	Jigawa
2839	JG/0027	JG/0027/S/1	Rasheed Shekoni Specialist Hospital		Dutse, Jigawa	Dutse	Jigawa
2840	JG/0027	JG/0027/S/7	Rasheed Shekoni Specialist Hospital		Dutse, Jigawa	Dutse	Jigawa
2841	JG/0027	JG/0027/S/2	Rasheed Shekoni Specialist Hospital		Dutse, Jigawa	Dutse	Jigawa
2842	JG/0027	JG/0027/S/3	Rasheed Shekoni Specialist Hospital		Dutse, Jigawa	Dutse	Jigawa
2843	JG/0027	JG/0027/S/4	Rasheed Shekoni Specialist Hospital		Dutse, Jigawa	Dutse	Jigawa
2844	JG/0027	JG/0027/S/6	Rasheed Shekoni Specialist Hospital		Dutse, Jigawa	Dutse	Jigawa
2845	JG/0027	JG/0027/S/5	Rasheed Shekoni Specialist Hospital		Dutse, Jigawa	Dutse	Jigawa
2846	JG/0027	JG/0027/S/13	Rasheed Shekoni Specialist Hospital		Dutse, Jigawa	Dutse	Jigawa
2847	JG/0027	JG/0027/S/12	Rasheed Shekoni Specialist Hospital		Dutse, Jigawa	Dutse	Jigawa
2848	JG/0027	JG/0027/S/8	Rasheed Shekoni Specialist Hospital		Dutse, Jigawa	Dutse	Jigawa
2849	KB/0001	KB/0001/S/15	General Hospital Argungu		Argungu	Argungu	Kebbi
2850	KB/0001	KB/0001/S/5	General Hospital Argungu		Argungu	Argungu	Kebbi
2851	KB/0001	KB/0001/S/1	General Hospital Argungu		Argungu	Argungu	Kebbi
2852	KB/0001	KB/0001/S/4	General Hospital Argungu		Argungu	Argungu	Kebbi
2853	KB/0001	KB/0001/S/7	General Hospital Argungu		Argungu	Argungu	Kebbi
2854	KB/0001	KB/0001/S/8	General Hospital Argungu		Argungu	Argungu	Kebbi
2855	KB/0003	KB/0003/S/6	Federal Medical Center - Birnin Kebbi		Birnin Kebbi	Birnin Kebbi	Kebbi
2856	KB/0003	KB/0003/S/1	Federal Medical Center - Birnin Kebbi		Birnin Kebbi	Birnin Kebbi	Kebbi
2857	KB/0003	KB/0003/S/11	Federal Medical Center - Birnin Kebbi		Birnin Kebbi	Birnin Kebbi	Kebbi
2858	KB/0003	KB/0003/S/3	Federal Medical Center - Birnin Kebbi		Birnin Kebbi	Birnin Kebbi	Kebbi
2859	KB/0003	KB/0003/S/7	Federal Medical Center - Birnin Kebbi		Birnin Kebbi	Birnin Kebbi	Kebbi

2860	KB/0003	KB/0003/S/10	Federal Medical Center - Birnin Kebbi		Birnin Kebbi	Birnin Kebbi	Kebbi
2861	KB/0003	KB/0003/S/15	Federal Medical Center - Birnin Kebbi		Birnin Kebbi	Birnin Kebbi	Kebbi
2862	KB/0003	KB/0003/S/8	Federal Medical Center - Birnin Kebbi		Birnin Kebbi	Birnin Kebbi	Kebbi
2863	KB/0003	KB/0003/S/5	Federal Medical Center - Birnin Kebbi		Birnin Kebbi	Birnin Kebbi	Kebbi
2864	KB/0003	KB/0003/S/2	Federal Medical Center - Birnin Kebbi		Birnin Kebbi	Birnin Kebbi	Kebbi
2865	KB/0003	KB/0003/S/4	Federal Medical Center - Birnin Kebbi		Birnin Kebbi	Birnin Kebbi	Kebbi
2866	KB/0008	KB/0008/S/11	Sir Yahaya Memorial Hospital		Ahmadu Bello Way, Birnin Kebbi	Birnin Kebbi	Kebbi
2867	KB/0008	KB/0008/S/1	Sir Yahaya Memorial Hospital		Ahmadu Bello Way, Birnin Kebbi	Birnin Kebbi	Kebbi
2868	KB/0008	KB/0008/S/7	Sir Yahaya Memorial Hospital		Ahmadu Bello Way, Birnin Kebbi	Birnin Kebbi	Kebbi
2869	KB/0008	KB/0008/S/5	Sir Yahaya Memorial Hospital		Ahmadu Bello Way, Birnin Kebbi	Birnin Kebbi	Kebbi
2870	KB/0008	KB/0008/S/4	Sir Yahaya Memorial Hospital		Ahmadu Bello Way, Birnin Kebbi	Birnin Kebbi	Kebbi
2871	KB/0008	KB/0008/S/6	Sir Yahaya Memorial Hospital		Ahmadu Bello Way, Birnin Kebbi	Birnin Kebbi	Kebbi
2872	KB/0008	KB/0008/S/2	Sir Yahaya Memorial Hospital		Ahmadu Bello Way, Birnin Kebbi	Birnin Kebbi	Kebbi
2873	KB/0012	KB/0012/S/4	Zetta Pharmacy	Pharmacy	Opp. Haliru Abdul Secretariat, Rafiu Atiku Rd, B/Kebbi	Birnin Kebbi	Kebbi
2874	KB/0019	KB/0019/S/5	Apex Medical Diagnostics	Laboratory	Along Nagari Science College Rd, Birnin Kebbi	Birnin Kebbi	Kebbi
2875	KB/0020	KB/0020/S/5	General Hospital, Yauri	-	Yauri	Yauri	Kebbi
2876	KB/0020	KB/0020/S/6	General Hospital, Yauri	-	Yauri	Yauri	Kebbi
2877	KB/0020	KB/0020/S/1	General Hospital, Yauri	-	Yauri	Yauri	Kebbi
2878	KB/0020	KB/0020/S/8	General Hospital, Yauri	-	Yauri	Yauri	Kebbi
2879	KB/0020	KB/0020/S/15	General Hospital, Yauri	-	Yauri	Yauri	Kebbi
2880	KB/0020	KB/0020/S/3	General Hospital, Yauri	-	Yauri	Yauri	Kebbi
2881	KB/0020	KB/0020/S/4	General Hospital, Yauri	-	Yauri	Yauri	Kebbi
2882	KB/0020	KB/0020/S/7	General Hospital, Yauri	-	Yauri	Yauri	Kebbi
2883	KB/0027	KB/0027/S/8	Martha Bamaiyi General Hospital Zuru		Zuru Bye-Pass Behind First Bank, Zuru, Kebbi State	Zuru	Kebbi
2884	KB/0027	KB/0027/S/4	Martha Bamaiyi General Hospital Zuru		Zuru Bye-Pass Behind First Bank, Zuru, Kebbi State	Zuru	Kebbi
2885	KB/0027	KB/0027/S/1	Martha Bamaiyi General Hospital Zuru		Zuru Bye-Pass Behind First Bank, Zuru, Kebbi State	Zuru	Kebbi
2886	KB/0027	KB/0027/S/5	Martha Bamaiyi General Hospital Zuru		Zuru Bye-Pass Behind First Bank, Zuru, Kebbi State	Zuru	Kebbi
2887	KB/0027	KB/0027/S/3	Martha Bamaiyi General Hospital Zuru		Zuru Bye-Pass Behind First Bank, Zuru, Kebbi State	Zuru	Kebbi
2888	KB/0029	KB/0029/S/4	Junju Pharmacy	Pharmacy	Emir Haruna Road Opp Texaco Filling Station, Kebbi State	Birnin Kebbi	Kebbi
2889	KB/0030	KB/0030/S/4	Kaduna Pharmaceutical Ltd.	Pharmacy	123 Ahmadu Bello Way, P.O.Box 255, Zuru	Zuru	Kebbi
2890	KB/0033	KB/0033/S/4	junju pharmacy	Pharmacy	emir. haruna road, opp Texaco filling station	Birnin Kebbi	Kebbi
2891	KB/0034	KB/0034/S/4	Kauna pharmaceutical ltd.	Pharmacy	123, ahmadu bello way, zuru	Zuru	Kebbi
2892	KB/0039	KB/0039/S/2	University Clinic (F.U.B.K)		Federal University Birnin Kebbi, Kebbi State	Birnin Kebbi	Kebbi
2893	KD/0002	KD/0002/S/4	Complete Pharmacy Ltd.	Pharmacy	L. 9, Chukun Road, Sabon Tasha, kaduna	Kaduna South	Kaduna
2894	KD/0005	KD/0005/S/2	Air Force Medical Centre Mando		Kaduna	Igabi	Kaduna
2895	KD/0005	KD/0005/S/5	Air Force Medical Centre Mando		Kaduna	Igabi	Kaduna
2896	KD/0005	KD/0005/S/4	Air Force Medical Centre Mando		Kaduna	Igabi	Kaduna
2897	KD/0005	KD/0005/S/7	Air Force Medical Centre Mando		Kaduna	Igabi	Kaduna

2898	KD/0005	KD/0005/S/8	Air Force Medical Centre Mando		Kaduna	Igabi	Kaduna
2899	KD/0005	KD/0005/S/11	Air Force Medical Centre Mando		Kaduna	Igabi	Kaduna
2900	KD/0005	KD/0005/S/14	Air Force Medical Centre Mando		Kaduna	Igabi	Kaduna
2901	KD/0005	KD/0005/S/1	Air Force Medical Centre Mando		Kaduna	Igabi	Kaduna
2902	KD/0005	KD/0005/S/3	Air Force Medical Centre Mando		Kaduna	Igabi	Kaduna
2903	KD/0005	KD/0005/S/6	Air Force Medical Centre Mando		Kaduna	Igabi	Kaduna
2904	KD/0008	KD/0008/S/5	Abdul-Azeez Memorial Hospital Clinic & Maternity		D.B. 10, Kabala West Road, Near Amaboni Hotal Tudun Wada,Kad	Kaduna South	Kaduna
2905	KD/0009	KD/0009/S/9	ABUTH Neuro Psychiatrics	Psychiatrics, Medicine	Independence Way, Kaduna State	Kaduna North	Kaduna
2906	KD/0009	KD/0009/S/2	ABUTH Neuro Psychiatrics	Psychiatrics, Medicine	Independence Way, Kaduna State	Kaduna North	Kaduna
2907	KD/0010	KD/0010/S/14	Giwa Hospital And Specialist Clinic		2. Giwa Road Abakpa Kaduna	Kaduna North	Kaduna
2908	KD/0010	KD/0010/S/3	Giwa Hospital And Specialist Clinic		2. Giwa Road Abakpa Kaduna	Kaduna North	Kaduna
2909	KD/0010	KD/0010/S/4	Giwa Hospital And Specialist Clinic		2. Giwa Road Abakpa Kaduna	Kaduna North	Kaduna
2910	KD/0010	KD/0010/S/2	Giwa Hospital And Specialist Clinic		2. Giwa Road Abakpa Kaduna	Kaduna North	Kaduna
2911	KD/0010	KD/0010/S/5	Giwa Hospital And Specialist Clinic		2. Giwa Road Abakpa Kaduna	Kaduna North	Kaduna
2912	KD/0010	KD/0010/S/1	Giwa Hospital And Specialist Clinic		2. Giwa Road Abakpa Kaduna	Kaduna North	Kaduna
2913	KD/0010	KD/0010/S/6	Giwa Hospital And Specialist Clinic		2. Giwa Road Abakpa Kaduna	Kaduna North	Kaduna
2914	KD/0010	KD/0010/S/7	Giwa Hospital And Specialist Clinic		2. Giwa Road Abakpa Kaduna	Kaduna North	Kaduna
2915	KD/0010	KD/0010/S/8	Giwa Hospital And Specialist Clinic		2. Giwa Road Abakpa Kaduna	Kaduna North	Kaduna
2916	KD/0012	KD/0012/S/15	Alba Clinic & Medical Centre		25, Constitution Road, Kaduna	Kaduna North	Kaduna
2917	KD/0012	KD/0012/S/12	Alba Clinic & Medical Centre		25, Constitution Road, Kaduna	Kaduna North	Kaduna
2918	KD/0012	KD/0012/S/7	Alba Clinic & Medical Centre		25, Constitution Road, Kaduna	Kaduna North	Kaduna
2919	KD/0012	KD/0012/S/1	Alba Clinic & Medical Centre		25, Constitution Road, Kaduna	Kaduna North	Kaduna
2920	KD/0012	KD/0012/S/6	Alba Clinic & Medical Centre		25, Constitution Road, Kaduna	Kaduna North	Kaduna
2921	KD/0012	KD/0012/S/5	Alba Clinic & Medical Centre		25, Constitution Road, Kaduna	Kaduna North	Kaduna
2922	KD/0012	KD/0012/S/14	Alba Clinic & Medical Centre		25, Constitution Road, Kaduna	Kaduna North	Kaduna
2923	KD/0012	KD/0012/S/3	Alba Clinic & Medical Centre		25, Constitution Road, Kaduna	Kaduna North	Kaduna
2924	KD/0013	KD/0013/S/5	Sefa Specialist Hospital		3, College Road, Kaduna	Kaduna North	Kaduna
2925	KD/0013	KD/0013/S/6	Sefa Specialist Hospital		3, College Road, Kaduna	Kaduna North	Kaduna
2926	KD/0013	KD/0013/S/1	Sefa Specialist Hospital		3, College Road, Kaduna	Kaduna North	Kaduna
2927	KD/0013	KD/0013/S/2	Sefa Specialist Hospital		3, College Road, Kaduna	Kaduna North	Kaduna
2928	KD/0013	KD/0013/S/3	Sefa Specialist Hospital		3, College Road, Kaduna	Kaduna North	Kaduna
2929	KD/0014	KD/0014/S/6	Covenant Hospital & Maternity		Au 6, Benin Street Kaduna.	Kaduna North	Kaduna
2930	KD/0014	KD/0014/S/3	Covenant Hospital & Maternity		Au 6, Benin Street Kaduna.	Kaduna North	Kaduna
2931	KD/0014	KD/0014/S/5	Covenant Hospital & Maternity		Au 6, Benin Street Kaduna.	Kaduna North	Kaduna
2932	KD/0015	KD/0015/S/3	Belmont Specialist Hospital		S.S. 5 Nasarawa Road, Kaduna	Kaduna North	Kaduna
2933	KD/0015	KD/0015/S/5	Belmont Specialist Hospital		S.S. 5 Nasarawa Road, Kaduna	Kaduna North	Kaduna
2934	KD/0015	KD/0015/S/6	Belmont Specialist Hospital		S.S. 5 Nasarawa Road, Kaduna	Kaduna North	Kaduna
2935	KD/0016	KD/0016/S/1	Jowako Hospital		F 15 Jos Road, Kaduna	Kaduna North	Kaduna
2936	KD/0016	KD/0016/S/5	Jowako Hospital		F 15 Jos Road, Kaduna	Kaduna North	Kaduna
2937	KD/0016	KD/0016/S/3	Jowako Hospital		F 15 Jos Road, Kaduna	Kaduna North	Kaduna
2938	KD/0016	KD/0016/S/2	Jowako Hospital		F 15 Jos Road, Kaduna	Kaduna North	Kaduna
2939	KD/0017	KD/0017/S/3	Kagoro Sheshan Clinic	-	Bb11 & 12 Nupe Road, Kaduna	Kaduna North	Kaduna
2940	KD/0017	KD/0017/S/1	Kagoro Sheshan Clinic	-	Bb11 & 12 Nupe Road, Kaduna	Kaduna North	Kaduna
2941	KD/0018	KD/0018/S/3	Chasel Hospital		15, Isa Kaita Road, Kaduna	Kaduna North	Kaduna
2942	KD/0018	KD/0018/S/8	Chasel Hospital		15, Isa Kaita Road, Kaduna	Kaduna North	Kaduna
2943	KD/0018	KD/0018/S/2	Chasel Hospital		15, Isa Kaita Road, Kaduna	Kaduna North	Kaduna

2944	KD/0018	KD/0018/S/1	Chasel Hospital		15, Isa Kaita Road, Kaduna	Kaduna North	Kaduna
2945	KD/0019	KD/0019/S/2	Garkuwa Specialist Hospital		1a Sultan Road, Off Isa Kaita Road, Kaduna	Kaduna North	Kaduna
2946	KD/0019	KD/0019/S/1	Garkuwa Specialist Hospital		1a Sultan Road, Off Isa Kaita Road, Kaduna	Kaduna North	Kaduna
2947	KD/0019	KD/0019/S/3	Garkuwa Specialist Hospital		1a Sultan Road, Off Isa Kaita Road, Kaduna	Kaduna North	Kaduna
2948	KD/0019	KD/0019/S/15	Garkuwa Specialist Hospital		1a Sultan Road, Off Isa Kaita Road, Kaduna	Kaduna North	Kaduna
2949	KD/0019	KD/0019/S/6	Garkuwa Specialist Hospital		1a Sultan Road, Off Isa Kaita Road, Kaduna	Kaduna North	Kaduna
2950	KD/0019	KD/0019/S/5	Garkuwa Specialist Hospital		1a Sultan Road, Off Isa Kaita Road, Kaduna	Kaduna North	Kaduna
2951	KD/0019	KD/0019/S/4	Garkuwa Specialist Hospital		1a Sultan Road, Off Isa Kaita Road, Kaduna	Kaduna North	Kaduna
2952	KD/0019	KD/0019/S/12	Garkuwa Specialist Hospital		1a Sultan Road, Off Isa Kaita Road, Kaduna	Kaduna North	Kaduna
2953	KD/0019	KD/0019/S/8	Garkuwa Specialist Hospital		1a Sultan Road, Off Isa Kaita Road, Kaduna	Kaduna North	Kaduna
2954	KD/0020	KD/0020/S/1	Iyali Hospital & Maternity		7, Kagoro Close, Off Gwari Crescent, Ungwar Rimi Gra, Kaduna	Kaduna North	Kaduna
2955	KD/0020	KD/0020/S/2	Iyali Hospital & Maternity		7, Kagoro Close, Off Gwari Crescent, Ungwar Rimi Gra, Kaduna	Kaduna North	Kaduna
2956	KD/0020	KD/0020/S/3	Iyali Hospital & Maternity		7, Kagoro Close, Off Gwari Crescent, Ungwar Rimi Gra, Kaduna	Kaduna North	Kaduna
2957	KD/0020	KD/0020/S/5	Iyali Hospital & Maternity		7, Kagoro Close, Off Gwari Crescent, Ungwar Rimi Gra, Kaduna	Kaduna North	Kaduna
2958	KD/0021	KD/0021/S/15	Rimi Clinics & Maternity Nig. Ltd.		24, Kubaka Road, Off Ja Abdulkadir Road, Ungwan Rimi, Kaduna	Kaduna North	Kaduna
2959	KD/0021	KD/0021/S/6	Rimi Clinics & Maternity Nig. Ltd.		24, Kubaka Road, Off Ja Abdulkadir Road, Ungwan Rimi, Kaduna	Kaduna North	Kaduna
2960	KD/0021	KD/0021/S/1	Rimi Clinics & Maternity Nig. Ltd.		24, Kubaka Road, Off Ja Abdulkadir Road, Ungwan Rimi, Kaduna	Kaduna North	Kaduna
2961	KD/0021	KD/0021/S/3	Rimi Clinics & Maternity Nig. Ltd.		24, Kubaka Road, Off Ja Abdulkadir Road, Ungwan Rimi, Kaduna	Kaduna North	Kaduna
2962	KD/0026	KD/0026/S/3	Eagle Hospital, Kaduna		No. 3, Kujama Street/Gwari Road, Sao tasha, kaduna	Kaduna North	Kaduna
2963	KD/0028	KD/0028/S/5	Barau Dikko Teaching Hospital, Kaduna		Barau Dikko Teaching Hospital, Kaduna	Kaduna North	Kaduna
2964	KD/0028	KD/0028/S/4	Barau Dikko Teaching Hospital, Kaduna		Barau Dikko Teaching Hospital, Kaduna	Kaduna North	Kaduna
2965	KD/0028	KD/0028/S/3	Barau Dikko Teaching Hospital, Kaduna		Barau Dikko Teaching Hospital, Kaduna	Kaduna North	Kaduna
2966	KD/0028	KD/0028/S/11	Barau Dikko Teaching Hospital, Kaduna		Barau Dikko Teaching Hospital, Kaduna	Kaduna North	Kaduna
2967	KD/0028	KD/0028/S/10	Barau Dikko Teaching Hospital, Kaduna		Barau Dikko Teaching Hospital, Kaduna	Kaduna North	Kaduna
2968	KD/0028	KD/0028/S/13	Barau Dikko Teaching Hospital, Kaduna		Barau Dikko Teaching Hospital, Kaduna	Kaduna North	Kaduna
2969	KD/0028	KD/0028/S/6	Barau Dikko Teaching Hospital, Kaduna		Barau Dikko Teaching Hospital, Kaduna	Kaduna North	Kaduna
2970	KD/0028	KD/0028/S/2	Barau Dikko Teaching Hospital, Kaduna		Barau Dikko Teaching Hospital, Kaduna	Kaduna North	Kaduna
2971	KD/0028	KD/0028/S/7	Barau Dikko Teaching Hospital, Kaduna		Barau Dikko Teaching Hospital, Kaduna	Kaduna North	Kaduna
2972	KD/0029	KD/0029/S/3	Jinya Specialist Hospital		7, Bida Road Kaduna	Kaduna North	Kaduna
2973	KD/0029	KD/0029/S/2	Jinya Specialist Hospital		7, Bida Road Kaduna	Kaduna North	Kaduna
2974	KD/0029	KD/0029/S/1	Jinya Specialist Hospital		7, Bida Road Kaduna	Kaduna North	Kaduna
2975	KD/0029	KD/0029/S/7	Jinya Specialist Hospital		7, Bida Road Kaduna	Kaduna North	Kaduna
2976	KD/0029	KD/0029/S/6	Jinya Specialist Hospital		7, Bida Road Kaduna	Kaduna North	Kaduna
2977	KD/0030	KD/0030/S/2	Kabala Hospital		F 1a Market Road, Kaduna	Kaduna North	Kaduna
2978	KD/0031	KD/0031/S/15	Guiness Eye Hospital(ABUTH)	Opthalmology,	Tafawa Balewa Way Kaduna	Kaduna North	Kaduna
2979	KD/0033	KD/0033/S/4	Lemu Pharmacy Ltd.,	Pharmacy	CA, 17, School Road, U/Rimi, Kaduna	Kaduna North	Kaduna

2980	KD/0035	KD/0035/S/4	Pacenel Int. Pharm. & Stores Ltd.,	Pharmacy	Suite 3, Shopping Complex, Kashim Ibrahim,Yakubu Avenue Kad.	Kaduna North	Kaduna
2981	KD/0036	KD/0036/S/4	Ishara Pharmaceuticals Ltd.,	Pharmacy	Shop 53, Safaha Shopping Complex, Kaduna	Kaduna North	Kaduna
2982	KD/0037	KD/0037/S/4	Osbud-K Pharmacy	Pharmacy	Kaduna Club Cornershop, Muh''d Buhari Way, Off Hamdala Hotel, Kaduna.	Kaduna North	Kaduna
2983	KD/0038	KD/0038/S/4	Rhemedic Pharmacy	Pharmacy	40 Isa Kaita Road, Kaduna	Kaduna North	Kaduna
2984	KD/0039	KD/0039/S/4	Gold Standard Pharmacy & Stores	Pharmacy	C1 A1 Shehu Laminu Road, U/Rimi Kaduna	Kaduna North	Kaduna
2985	KD/0040	KD/0040/S/4	Aban Pharmacy	Pharmacy	Opp. ABU Hospital, Main Gate, Kaduna	Kaduna North	Kaduna
2986	KD/0041	KD/0041/S/4	Jagsil Pharmacy & Stores	Pharmacy	128 College Road, U/Dosa, Kaduna	Kaduna North	Kaduna
2987	KD/0042	KD/0042/S/5	Echo Scan Services Ltd.,	Laboratory	4, Katsina Road, Kaduna	Kaduna North	Kaduna
2988	KD/0043	KD/0043/S/5	Diamond Diagnostic Care Lab	Laboratory	2nd Floor, 14 Abubakar Kigo/Constitution Road, Kaduna	Kaduna North	Kaduna
2989	KD/0047	KD/0047/S/1	Etomie Oral and Maxillofacial Surgery Spe. Hospital	Maxillo-Facial Surgery	6, Shehu Ibrahim Close, Malalid North East Extension.	Kaduna North	Kaduna
2990	KD/0048	KD/0048/S/5	Jenicare Pharmacy	Pharmacy	Shop No. 9 Image Plaza, No.2, Blk B Zaria/Jolly Tanko Road, Barnawa, Kaduna.	Ibadan South West	Oyo
2991	KD/0049	KD/0049/S/4	Samen Pharmacy Ltd.	Pharmacy	No H3, Waff Road, Adjacent, Command Guest House, Kaduna.	Kaduna North	Kaduna
2992	KD/0053	KD/0053/S/15	Arewa Eye Centre	Ophthalmology	XX 3 Daura Road, Kaduna	Kaduna North	Kaduna
2993	KD/0060	KD/0060/S/19	Habbat Skin Clinic & Med. Centre	Dermatology	No. 10 Shehu Laminu Road, U/Rimi, Kaduna	Kaduna North	Kaduna
2994	KD/0061	KD/0061/S/12	National Ear Centre	ENT	No. 20 Katuru Road, (Yusuf Dantoho Hospital) Kaduna	Kaduna South	Kaduna
2995	KD/0062	KD/0062/S/11	J. Adams Physiotherapy & Medical Fitness Centre	Pysiotherapy	5B, Zaki Road, Abakpa GRA, Kaduna	Kaduna North	Kaduna
2996	KD/0064	KD/0064/S/8	The Dental Surgery	Dental	7, Wurno Road, GRA, Kaduna	Kaduna North	Kaduna
2997	KD/0065	KD/0065/S/15	Thelish Eye Centre	Ophthalmology	5, Ali Akilu Road, Kaduna	Kaduna North	Kaduna
2998	KD/0066	KD/0066/S/4	Aslad Pharmacy Ltd.,	Pharmacy	AY 158 Ibrahim Taiwo Road, Kaduna	Kaduna North	Kaduna
2999	KD/0067	KD/0067/S/4	Lafian Jiki Pharmacy & Stores Ltd.	Pharmacy	Warri Street/Kano Road, Kaduna	Kaduna North	Kaduna
3000	KD/0068	KD/0068/S/4	Oris Pharmacy	Pharmacy	W10 Zaira Road, Kaduna	Kaduna North	Kaduna
3001	KD/0069	KD/0069/S/4	Enbokay Pharmacy & General Layout	Pharmacy	Suites 1 & 2, Block B, Constitution Road, Kaduna	Kaduna North	Kaduna
3002	KD/0070	KD/0070/S/4	Karama Pharmacy & Chemical Co. Ltd.	Pharmacy	10, Independence Way, (Recreation Club) Kaduna	Kaduna North	Kaduna
3003	KD/0071	KD/0071/S/4	Ishara Pharmaceuticals Ltd.,	Pharmacy	1, Mohammed Buhari Way, Shopping Complex, Kaduna	Kaduna North	Kaduna
3004	KD/0072	KD/0072/S/4	Faliko Pharmacy Ltd.,	Pharmacy	Shop 12 Mohammed Buhari Way, Shopping Complex, Kaduna	Kaduna North	Kaduna
3005	KD/0074	KD/0074/S/2	C-Jay Medical Centre	Internal Medicine	Kaduna, Kaduna State	Kaduna South	Kaduna
3006	KD/0076	KD/0076/S/6	Salamatu Hospital & Maternity		2, Bimin Yero Road, Off Kabala West, Road, Kaduna	Kaduna South	Kaduna
3007	KD/0076	KD/0076/S/5	Salamatu Hospital & Maternity		2, Bimin Yero Road, Off Kabala West, Road, Kaduna	Kaduna South	Kaduna
3008	KD/0076	KD/0076/S/3	Salamatu Hospital & Maternity		2, Bimin Yero Road, Off Kabala West, Road, Kaduna	Kaduna South	Kaduna
3009	KD/0078	KD/0078/S/5	Fountain Hospital & Maternity	-	Ap 13 Community Bank Road, Prp Bus Stop/Gamagira, Tudun Wada	Kaduna South	Kaduna
3010	KD/0078	KD/0078/S/3	Fountain Hospital & Maternity	-	Ap 13 Community Bank Road, Prp Bus Stop/Gamagira, Tudun Wada	Kaduna South	Kaduna
3011	KD/0080	KD/0080/S/8	Shehu Kangiwa Medical Centre	-	(Sick Bay), Kaduna Polytechnic, T/Wada Kaduna	Kaduna South	Kaduna
3012	KD/0080	KD/0080/S/4	Shehu Kangiwa Medical Centre	-	(Sick Bay), Kaduna Polytechnic, T/Wada Kaduna	Kaduna South	Kaduna

3013	KD/0080	KD/0080/S/5	Shehu Kangiwa Medical Centre	-	(Sick Bay), Kaduna Polytechnic, T/Wada Kaduna	Kaduna South	Kaduna
3014	KD/0080	KD/0080/S/3	Shehu Kangiwa Medical Centre	-	(Sick Bay), Kaduna Polytechnic, T/Wada Kaduna	Kaduna South	Kaduna
3015	KD/0080	KD/0080/S/11	Shehu Kangiwa Medical Centre	-	(Sick Bay), Kaduna Polytechnic, T/Wada Kaduna	Kaduna South	Kaduna
3016	KD/0081	KD/0081/S/2	Yusuf Dantsoho Hospital		T/Wada, Kaduna	Kaduna South	Kaduna
3017	KD/0081	KD/0081/S/3	Yusuf Dantsoho Hospital		T/Wada, Kaduna	Kaduna South	Kaduna
3018	KD/0081	KD/0081/S/7	Yusuf Dantsoho Hospital		T/Wada, Kaduna	Kaduna South	Kaduna
3019	KD/0081	KD/0081/S/4	Yusuf Dantsoho Hospital		T/Wada, Kaduna	Kaduna South	Kaduna
3020	KD/0081	KD/0081/S/8	Yusuf Dantsoho Hospital		T/Wada, Kaduna	Kaduna South	Kaduna
3021	KD/0081	KD/0081/S/5	Yusuf Dantsoho Hospital		T/Wada, Kaduna	Kaduna South	Kaduna
3022	KD/0081	KD/0081/S/6	Yusuf Dantsoho Hospital		T/Wada, Kaduna	Kaduna South	Kaduna
3023	KD/0081	KD/0081/S/1	Yusuf Dantsoho Hospital		T/Wada, Kaduna	Kaduna South	Kaduna
3024	KD/0082	KD/0082/S/3	Maneks Hospital Ltd.		A1 Block 5, Makera Road, Kakuri Kaduna	Kaduna South	Kaduna
3025	KD/0082	KD/0082/S/10	Maneks Hospital Ltd.		A1 Block 5, Makera Road, Kakuri Kaduna	Kaduna South	Kaduna
3026	KD/0082	KD/0082/S/1	Maneks Hospital Ltd.		A1 Block 5, Makera Road, Kakuri Kaduna	Kaduna South	Kaduna
3027	KD/0082	KD/0082/S/8	Maneks Hospital Ltd.		A1 Block 5, Makera Road, Kakuri Kaduna	Kaduna South	Kaduna
3028	KD/0082	KD/0082/S/6	Maneks Hospital Ltd.		A1 Block 5, Makera Road, Kakuri Kaduna	Kaduna South	Kaduna
3029	KD/0082	KD/0082/S/15	Maneks Hospital Ltd.		A1 Block 5, Makera Road, Kakuri Kaduna	Kaduna South	Kaduna
3030	KD/0082	KD/0082/S/2	Maneks Hospital Ltd.		A1 Block 5, Makera Road, Kakuri Kaduna	Kaduna South	Kaduna
3031	KD/0083	KD/0083/S/1	Amina Hospital Ltd.		Gwari Avenue, Kachia Road, Kaduna	Kaduna South	Kaduna
3032	KD/0083	KD/0083/S/6	Amina Hospital Ltd.		Gwari Avenue, Kachia Road, Kaduna	Kaduna South	Kaduna
3033	KD/0083	KD/0083/S/2	Amina Hospital Ltd.		Gwari Avenue, Kachia Road, Kaduna	Kaduna South	Kaduna
3034	KD/0083	KD/0083/S/3	Amina Hospital Ltd.		Gwari Avenue, Kachia Road, Kaduna	Kaduna South	Kaduna
3035	KD/0084	KD/0084/S/5	Ishaku Hospital		Gwari Avenue, Kachia Road, Kaduna	Kaduna South	Kaduna
3036	KD/0084	KD/0084/S/3	Ishaku Hospital		Gwari Avenue, Kachia Road, Kaduna	Kaduna South	Kaduna
3037	KD/0084	KD/0084/S/1	Ishaku Hospital		Gwari Avenue, Kachia Road, Kaduna	Kaduna South	Kaduna
3038	KD/0085	KD/0085/S/5	Giltoe Aris Specialist Hospital		17/19 Maiduwa Road, Near Barnawa Market, Kaduna	Kaduna South	Kaduna
3039	KD/0085	KD/0085/S/3	Giltoe Aris Specialist Hospital		17/19 Maiduwa Road, Near Barnawa Market, Kaduna	Kaduna South	Kaduna
3040	KD/0086	KD/0086/S/2	C Jay Medical Centre		Near Dambo Int''''L College, No 13, Kubani Crescent, Barnawa Kd	Kaduna South	Kaduna
3041	KD/0087	KD/0087/S/8	Gwamna Awan Hospital		Nasarawa, Kaduna	Kaduna South	Kaduna
3042	KD/0087	KD/0087/S/7	Gwamna Awan Hospital		Nasarawa, Kaduna	Kaduna South	Kaduna
3043	KD/0087	KD/0087/S/4	Gwamna Awan Hospital		Nasarawa, Kaduna	Kaduna South	Kaduna
3044	KD/0087	KD/0087/S/3	Gwamna Awan Hospital		Nasarawa, Kaduna	Kaduna South	Kaduna
3045	KD/0087	KD/0087/S/1	Gwamna Awan Hospital		Nasarawa, Kaduna	Kaduna South	Kaduna
3046	KD/0087	KD/0087/S/5	Gwamna Awan Hospital		Nasarawa, Kaduna	Kaduna South	Kaduna
3047	KD/0089	KD/0089/S/7	44 Armed Forces Reference Hospital		Kaduna	Kaduna South	Kaduna
3048	KD/0089	KD/0089/S/11	44 Armed Forces Reference Hospital		Kaduna	Kaduna South	Kaduna
3049	KD/0089	KD/0089/S/6	44 Armed Forces Reference Hospital		Kaduna	Kaduna South	Kaduna
3050	KD/0089	KD/0089/S/12	44 Armed Forces Reference Hospital		Kaduna	Kaduna South	Kaduna
3051	KD/0089	KD/0089/S/2	44 Armed Forces Reference Hospital		Kaduna	Kaduna South	Kaduna
3052	KD/0089	KD/0089/S/3	44 Armed Forces Reference Hospital		Kaduna	Kaduna South	Kaduna
3053	KD/0089	KD/0089/S/1	44 Armed Forces Reference Hospital		Kaduna	Kaduna South	Kaduna

3054	KD/0090	KD/0090/S/5	Federal Neuropsychiatric Hospital	Psychiatry, Laboratory, Pharmacy	Barnawa, Kaduna	Kaduna South	Kaduna
3055	KD/0090	KD/0090/S/9	Federal Neuropsychiatric Hospital	Psychiatry, Laboratory, Pharmacy	Barnawa, Kaduna	Kaduna South	Kaduna
3056	KD/0090	KD/0090/S/4	Federal Neuropsychiatric Hospital	Psychiatry, Laboratory, Pharmacy	Barnawa, Kaduna	Kaduna South	Kaduna
3057	KD/0091	KD/0091/S/4	Ghali Pharmacy	Pharmacy	Kinsasha Road, U/Rimi, Kaduna	Kaduna North	Kaduna
3058	KD/0092	KD/0092/S/4	Godiya Pharmacy Co., Ltd.	Pharmacy	58/59, Lemu Road, T/Nupawa	Kaduna South	Kaduna
3059	KD/0093	KD/0093/S/4	Osbud-K Pharmacy	Pharmacy	50, Lemu Road, Beside T/wada, Gen. Hospital, Kaduna	Kaduna South	Kaduna
3060	KD/0094	KD/0094/S/4	Redox Pharmaceuticals	Pharmacy	98, Aliyu Makama Road, Barnawa, Kaduna	Kaduna South	Kaduna
3061	KD/0102	KD/0102/S/15	Premier Eye Hospital	Ophthalmology	3, Salama Close (Off. College Rd) Kaura Avenue, Kaduna	Kaduna South	Kaduna
3062	KD/0105	KD/0105/S/4	Nukeka Pharmacy Ltd.	Pharmacy	6, Halima Road Ungwan Sunday, Sabo Kaduna	Chikun	Kaduna
3063	KD/0108	KD/0108/S/4	B & K Pharmacy Ltd.,	Pharmacy	Algeria Rd., Opp. Barnawa Baptist Church, Barnawa Kaduna	Kaduna South	Kaduna
3064	KD/0109	KD/0109/S/4	Maneks Pharmacy & Stores Ltd.,	Pharmacy	A1 Blo0ck 5 Makera, Kakuri Kaduna	Kaduna South	Kaduna
3065	KD/0110	KD/0110/S/4	Tada Pharmacy & Stores Ltd.,	Pharmacy	BZ 172, Junction Road, Kaduna	Kaduna North	Kaduna
3066	KD/0112	KD/0112/S/4	Almadina Clinic		No. 28 Queen Elizabeth Road, Gra Zairia	Sabon Gari	Kaduna
3067	KD/0112	KD/0112/S/3	Almadina Clinic		No. 28 Queen Elizabeth Road, Gra Zairia	Sabon Gari	Kaduna
3068	KD/0112	KD/0112/S/13	Almadina Clinic		No. 28 Queen Elizabeth Road, Gra Zairia	Sabon Gari	Kaduna
3069	KD/0112	KD/0112/S/1	Almadina Clinic		No. 28 Queen Elizabeth Road, Gra Zairia	Sabon Gari	Kaduna
3070	KD/0112	KD/0112/S/2	Almadina Clinic		No. 28 Queen Elizabeth Road, Gra Zairia	Sabon Gari	Kaduna
3071	KD/0112	KD/0112/S/6	Almadina Clinic		No. 28 Queen Elizabeth Road, Gra Zairia	Sabon Gari	Kaduna
3072	KD/0113	KD/0113/S/2	Ladiya Hospital		64, Benin Street, Zaira	Sabon Gari	Kaduna
3073	KD/0113	KD/0113/S/3	Ladiya Hospital		64, Benin Street, Zaira	Sabon Gari	Kaduna
3074	KD/0115	KD/0115/S/4	Bayin Pharmacy & Stores Nig. Ltd.	Pharmacy	No. 28 Queen Elizabeth Road, GRA Zaira, Kaduna	Sabon Gari	Kaduna
3075	KD/0119	KD/0119/S/15	University Health Service	Ophthalmology, Internal Medicine, Dental Pharmacy, Laboratory	ABU Zaira, Kaduna	Sabon Gari	Kaduna
3076	KD/0119	KD/0119/S/8	University Health Service	Ophthalmology, Internal Medicine, Dental Pharmacy, Laboratory	ABU Zaira, Kaduna	Sabon Gari	Kaduna
3077	KD/0119	KD/0119/S/4	University Health Service	Ophthalmology, Internal Medicine, Dental Pharmacy, Laboratory	ABU Zaira, Kaduna	Sabon Gari	Kaduna
3078	KD/0119	KD/0119/S/2	University Health Service	Ophthalmology, Internal Medicine, Dental Pharmacy, Laboratory	ABU Zaira, Kaduna	Sabon Gari	Kaduna
3079	KD/0119	KD/0119/S/5	University Health Service	Ophthalmology, Internal Medicine, Dental Pharmacy, Laboratory	ABU Zaira, Kaduna	Sabon Gari	Kaduna
3080	KD/0122	KD/0122/S/4	Ijabah Pharmacy Nig., Ltd.	Pharmacy	No. K. 14, Old Jos Road, Tudun Wada, Zaira Kaduna	Kaduna South	Kaduna
3081	KD/0123	KD/0123/S/2	Salama InfIrmary Hosp. & Maternity		Sokoto Road, Kwangla, Zaira	Zaria	Kaduna
3082	KD/0123	KD/0123/S/5	Salama InfIrmary Hosp. & Maternity		Sokoto Road, Kwangla, Zaira	Zaria	Kaduna
3083	KD/0124	KD/0124/S/5	St. Luke's Anglican Hospital		Wusasa Zaira	Zaria	Kaduna
3084	KD/0124	KD/0124/S/4	St. Luke's Anglican Hospital		Wusasa Zaira	Zaria	Kaduna
3085	KD/0124	KD/0124/S/1	St. Luke's Anglican Hospital		Wusasa Zaira	Zaria	Kaduna

3086	KD/0124	KD/0124/S/3	St. Luke's Anglican Hospital		Wusasa Zaira	Zaria	Kaduna
3087	KD/0126	KD/0126/S/2	Hajia Gambo Sawaba Gen. Hospital		Kofar-Gayan, Zaria	Zaria	Kaduna
3088	KD/0126	KD/0126/S/3	Hajia Gambo Sawaba Gen. Hospital		Kofar-Gayan, Zaria	Zaria	Kaduna
3089	KD/0126	KD/0126/S/1	Hajia Gambo Sawaba Gen. Hospital		Kofar-Gayan, Zaria	Zaria	Kaduna
3090	KD/0126	KD/0126/S/5	Hajia Gambo Sawaba Gen. Hospital		Kofar-Gayan, Zaria	Zaria	Kaduna
3091	KD/0133	KD/0133/S/4	Giljane Pharmacy	Pharmacy	21 D, Tafawa Balewa Way, ABUTH Shopping Complex Kaduna	Kaduna North	Kaduna
3092	KD/0134	KD/0134/S/4	Woskab Pharmacy	Pharmacy	2A, Alkali Road, By Scoa Motors, Kaduna	Kaduna North	Kaduna
3093	KD/0135	KD/0135/S/5	Euro Hospital	O&G, Surgery, Pharmacy, Laboratory	Kaduna	Kaduna North	Kaduna
3094	KD/0135	KD/0135/S/1	Euro Hospital	O&G, Surgery, Pharmacy, Laboratory	Kaduna	Kaduna North	Kaduna
3095	KD/0135	KD/0135/S/4	Euro Hospital	O&G, Surgery, Pharmacy, Laboratory	Kaduna	Kaduna North	Kaduna
3096	KD/0135	KD/0135/S/3	Euro Hospital	O&G, Surgery, Pharmacy, Laboratory	Kaduna	Kaduna North	Kaduna
3097	KD/0136	KD/0136/S/4	El-Mouktar Pharm.	Pharmacy	No 1, Sultan Bello Road, Ungwan Sarki, Kaduna	Kaduna North	Kaduna
3098	KD/0143	KD/0143/S/8	Topaz Dental Clinic	Dental	Safaha Complex, No. 6, Ibrahim Zaki Rd. U/Rimi Kaduna	Kaduna North	Kaduna
3099	KD/0153	KD/0153/S/12	Nigerian Defence Academy		Kawo, Kaduna	Kaduna North	Kaduna
3100	KD/0153	KD/0153/S/4	Nigerian Defence Academy		Kawo, Kaduna	Kaduna North	Kaduna
3101	KD/0153	KD/0153/S/5	Nigerian Defence Academy		Kawo, Kaduna	Kaduna North	Kaduna
3102	KD/0157	KD/0157/S/2	National Eye Centre		Mando Road, Off Nnamdi Azikiwe Way, Kaduna	Igabi	Kaduna
3103	KD/0157	KD/0157/S/5	National Eye Centre		Mando Road, Off Nnamdi Azikiwe Way, Kaduna	Igabi	Kaduna
3104	KD/0157	KD/0157/S/15	National Eye Centre		Mando Road, Off Nnamdi Azikiwe Way, Kaduna	Igabi	Kaduna
3105	KD/0157	KD/0157/S/12	National Eye Centre		Mando Road, Off Nnamdi Azikiwe Way, Kaduna	Igabi	Kaduna
3106	KD/0157	KD/0157/S/7	National Eye Centre		Mando Road, Off Nnamdi Azikiwe Way, Kaduna	Igabi	Kaduna
3107	KD/0157	KD/0157/S/4	National Eye Centre		Mando Road, Off Nnamdi Azikiwe Way, Kaduna	Igabi	Kaduna
3108	KD/0160	KD/0160/S/5	General Hospital, Giwa		Giwa, Kaduna State	Giwa	Kaduna
3109	KD/0160	KD/0160/S/2	General Hospital, Giwa		Giwa, Kaduna State	Giwa	Kaduna
3110	KD/0160	KD/0160/S/1	General Hospital, Giwa		Giwa, Kaduna State	Giwa	Kaduna
3111	KD/0160	KD/0160/S/3	General Hospital, Giwa		Giwa, Kaduna State	Giwa	Kaduna
3112	KD/0162	KD/0162/S/1	St Patrick Ibrahim Yakowa Memorial Hospital, Kafanchan		Kafanchan, Kaduna State.	Jema'a	Kaduna
3113	KD/0162	KD/0162/S/2	St Patrick Ibrahim Yakowa Memorial Hospital, Kafanchan		Kafanchan, Kaduna State.	Jema'a	Kaduna
3114	KD/0162	KD/0162/S/6	St Patrick Ibrahim Yakowa Memorial Hospital, Kafanchan		Kafanchan, Kaduna State.	Jema'a	Kaduna
3115	KD/0162	KD/0162/S/7	St Patrick Ibrahim Yakowa Memorial Hospital, Kafanchan		Kafanchan, Kaduna State.	Jema'a	Kaduna
3116	KD/0162	KD/0162/S/4	St Patrick Ibrahim Yakowa Memorial Hospital, Kafanchan		Kafanchan, Kaduna State.	Jema'a	Kaduna
3117	KD/0162	KD/0162/S/5	St Patrick Ibrahim Yakowa Memorial Hospital, Kafanchan		Kafanchan, Kaduna State.	Jema'a	Kaduna
3118	KD/0162	KD/0162/S/3	St Patrick Ibrahim Yakowa Memorial Hospital, Kafanchan		Kafanchan, Kaduna State.	Jema'a	Kaduna
3119	KD/0163	KD/0163/S/6	Savannah Poly Clinic		Samaru, Zaria.	Zaria	Kaduna

3120	KD/0163	KD/0163/S/2	Savannah Poly Clinic		Samaru, Zaria.	Zaria	Kaduna
3121	KD/0163	KD/0163/S/1	Savannah Poly Clinic		Samaru, Zaria.	Zaria	Kaduna
3122	KD/0168	KD/0168/S/5	Maneks Medical Laboratory Ltd.	Laboratory	A1 Block 5, Makera Road, Kakuri, Kaduna	Kaduna South	Kaduna
3123	KD/0169	KD/0169/S/7	Maneks Medical Diagnostic Centre Ltd.	Radiology	A1 Block 5, Makera Road, Kakuri, Kaduna	Kaduna South	Kaduna
3124	KD/0170	KD/0170/S/2	Mends Specialist Hospital & Aviation Medical Centre		5, Abba Kyari Close, Ungwan Rimi GRA, Kaduna	Kaduna North	Kaduna
3125	KD/0170	KD/0170/S/11	Mends Specialist Hospital & Aviation Medical Centre		5, Abba Kyari Close, Ungwan Rimi GRA, Kaduna	Kaduna North	Kaduna
3126	KD/0170	KD/0170/S/5	Mends Specialist Hospital & Aviation Medical Centre		5, Abba Kyari Close, Ungwan Rimi GRA, Kaduna	Kaduna North	Kaduna
3127	KD/0170	KD/0170/S/10	Mends Specialist Hospital & Aviation Medical Centre		5, Abba Kyari Close, Ungwan Rimi GRA, Kaduna	Kaduna North	Kaduna
3128	KD/0170	KD/0170/S/3	Mends Specialist Hospital & Aviation Medical Centre		5, Abba Kyari Close, Ungwan Rimi GRA, Kaduna	Kaduna North	Kaduna
3129	KD/0170	KD/0170/S/7	Mends Specialist Hospital & Aviation Medical Centre		5, Abba Kyari Close, Ungwan Rimi GRA, Kaduna	Kaduna North	Kaduna
3130	KD/0170	KD/0170/S/15	Mends Specialist Hospital & Aviation Medical Centre		5, Abba Kyari Close, Ungwan Rimi GRA, Kaduna	Kaduna North	Kaduna
3131	KD/0170	KD/0170/S/1	Mends Specialist Hospital & Aviation Medical Centre		5, Abba Kyari Close, Ungwan Rimi GRA, Kaduna	Kaduna North	Kaduna
3132	KD/0179	KD/0179/S/4	Farimed Pharmacy & Stores Ltd	Pharmacy	Mobil Filling Station Shopping Complex, 5 Independence Way, Kaduna	Kaduna North	Kaduna
3133	KD/0181	KD/0181/S/4	Y-Fab Pharmacy	Pharmacy	Art & Culture Shopping Complex, 30 Waff Rd., Kaduna, Kaduna State	Kaduna North	Kaduna
3134	KD/0182	KD/0182/S/6	Abi Clinics & Hospital		BZ 104 Ogori Road, Sardauna Crescent, Kaduna	Kaduna North	Kaduna
3135	KD/0187	KD/0187/S/3	Monarch Specialist Hospital		N/k 12, Arochukwu Road, Kaduna	Kaduna North	Kaduna
3136	KD/0188	KD/0188/S/4	Dana Clinic	Primary Provider	Old Airport Road, Mando, Kaduna	Igabi	Kaduna
3137	KD/0189	KD/0189/S/5	Prime Specialist Hospital	Primary Provider	Az 16 Bakori Road, Kaduna	Kaduna North	Kaduna
3138	KD/0189	KD/0189/S/6	Prime Specialist Hospital	Primary Provider	Az 16 Bakori Road, Kaduna	Kaduna North	Kaduna
3139	KD/0189	KD/0189/S/3	Prime Specialist Hospital	Primary Provider	Az 16 Bakori Road, Kaduna	Kaduna North	Kaduna
3140	KD/0189	KD/0189/S/1	Prime Specialist Hospital	Primary Provider	Az 16 Bakori Road, Kaduna	Kaduna North	Kaduna
3141	KD/0190	KD/0190/S/3	Anna-Kitch Medical Centre	Primary Provider	15, Wusasa Road, Zaria	Zaria	Kaduna
3142	KD/0190	KD/0190/S/1	Anna-Kitch Medical Centre	Primary Provider	15, Wusasa Road, Zaria	Zaria	Kaduna
3143	KD/0190	KD/0190/S/5	Anna-Kitch Medical Centre	Primary Provider	15, Wusasa Road, Zaria	Zaria	Kaduna
3144	KD/0193	KD/0193/S/2	Multi Clinic Nig. Ltd.	Primary Provider	26A Yahaya Road, Unguwa Rimi G.R.A., Kaduna	Kaduna North	Kaduna
3145	KD/0193	KD/0193/S/1	Multi Clinic Nig. Ltd.	Primary Provider	26A Yahaya Road, Unguwa Rimi G.R.A., Kaduna	Kaduna North	Kaduna
3146	KD/0193	KD/0193/S/3	Multi Clinic Nig. Ltd.	Primary Provider	26A Yahaya Road, Unguwa Rimi G.R.A., Kaduna	Kaduna North	Kaduna
3147	KD/0193	KD/0193/S/15	Multi Clinic Nig. Ltd.	Primary Provider	26A Yahaya Road, Unguwa Rimi G.R.A., Kaduna	Kaduna North	Kaduna
3148	KD/0193	KD/0193/S/6	Multi Clinic Nig. Ltd.	Primary Provider	26A Yahaya Road, Unguwa Rimi G.R.A., Kaduna	Kaduna North	Kaduna
3149	KD/0193	KD/0193/S/5	Multi Clinic Nig. Ltd.	Primary Provider	26A Yahaya Road, Unguwa Rimi G.R.A., Kaduna	Kaduna North	Kaduna
3150	KD/0195	KD/0195/S/5	Oxford Hospital		38, Airforce Road, Samaru RD, Makera, Kaduna	Kaduna South	Kaduna
3151	KD/0195	KD/0195/S/6	Oxford Hospital		38, Airforce Road, Samaru RD, Makera, Kaduna	Kaduna South	Kaduna
3152	KD/0195	KD/0195/S/2	Oxford Hospital		38, Airforce Road, Samaru RD, Makera, Kaduna	Kaduna South	Kaduna
3153	KD/0195	KD/0195/S/1	Oxford Hospital		38, Airforce Road, Samaru RD, Makera, Kaduna	Kaduna South	Kaduna

3154	KD/0197	KD/0197/S/2	Hitaf Specialist		D% Garbajar Abdulkadir Rd, Off Rimi drive, U/Rimi, Kaduna	Kaduna North	Kaduna
3155	KD/0200	KD/0200/S/2	St Gerard Catholic Hospital		Kakuri Kaduna	Kaduna South	Kaduna
3156	KD/0200	KD/0200/S/11	St Gerard Catholic Hospital		Kakuri Kaduna	Kaduna South	Kaduna
3157	KD/0200	KD/0200/S/5	St Gerard Catholic Hospital		Kakuri Kaduna	Kaduna South	Kaduna
3158	KD/0200	KD/0200/S/7	St Gerard Catholic Hospital		Kakuri Kaduna	Kaduna South	Kaduna
3159	KD/0200	KD/0200/S/4	St Gerard Catholic Hospital		Kakuri Kaduna	Kaduna South	Kaduna
3160	KD/0200	KD/0200/S/6	St Gerard Catholic Hospital		Kakuri Kaduna	Kaduna South	Kaduna
3161	KD/0200	KD/0200/S/10	St Gerard Catholic Hospital		Kakuri Kaduna	Kaduna South	Kaduna
3162	KD/0200	KD/0200/S/1	St Gerard Catholic Hospital		Kakuri Kaduna	Kaduna South	Kaduna
3163	KD/0200	KD/0200/S/3	St Gerard Catholic Hospital		Kakuri Kaduna	Kaduna South	Kaduna
3164	KD/0201	KD/0201/S/5	National Teachers Institute Staff Clinic		Km 5 Kaduna-Zaria Expressway, Kaduna	Igabi	Kaduna
3165	KD/0203	KD/0203/S/8	Patriot Dental Clinic	Dental	4B, Katsina Road Kaduna	Kaduna North	Kaduna
3166	KD/0208	KD/0208/S/4	Katari Pharmacy	Pharmacy	PP6 Warri Street By Ibrahim Taiwo Kaduna	Kaduna South	Kaduna
3167	KD/0210	KD/0210/S/4	Nefe Pharmacy	Pharmacy	3 Market Road Barnawa Kaduna	Kaduna South	Kaduna
3168	KD/0211	KD/0211/S/14	Medi-Scan Ltd	Radiology, X-Ray,U-Scan	LL6 Ahmadu Bello Way Kaduna	Kaduna North	Kaduna
3169	KD/0211	KD/0211/S/7	Medi-Scan Ltd	Radiology, X-Ray,U-Scan	LL6 Ahmadu Bello Way Kaduna	Kaduna North	Kaduna
3170	KD/0212	KD/0212/S/4	Gaba Pharmacy	Pharmacy	No. 20 Kataf Road Sabon Tasha Kaduna	Chikun	Kaduna
3171	KD/0215	KD/0215/S/4	Godek Pharmacy	Pharmacy	Z 5, Zaire Rd Barnawa Shopping Complex	Kaduna South	Kaduna
3172	KD/0217	KD/0217/S/13	Niger Optical Services (Nig) Ltd	Optometry	20 Hospital Road Tuoun Wada, Zaria	Zaria	Kaduna
3173	KD/0218	KD/0218/S/13	Niger Optical Services (Nig) Ltd	Optometry	NC 367 ,IBB Way Kaduna	Kaduna North	Kaduna
3174	KD/0223	KD/0223/S/7	A B U Teaching Hospital, Shika		Shika, Zaria, Kaduna State	Sabon Gari	Kaduna
3175	KD/0223	KD/0223/S/5	A B U Teaching Hospital, Shika		Shika, Zaria, Kaduna State	Sabon Gari	Kaduna
3176	KD/0223	KD/0223/S/4	A B U Teaching Hospital, Shika		Shika, Zaria, Kaduna State	Sabon Gari	Kaduna
3177	KD/0223	KD/0223/S/10	A B U Teaching Hospital, Shika		Shika, Zaria, Kaduna State	Sabon Gari	Kaduna
3178	KD/0223	KD/0223/S/12	A B U Teaching Hospital, Shika		Shika, Zaria, Kaduna State	Sabon Gari	Kaduna
3179	KD/0223	KD/0223/S/6	A B U Teaching Hospital, Shika		Shika, Zaria, Kaduna State	Sabon Gari	Kaduna
3180	KD/0223	KD/0223/S/2	A B U Teaching Hospital, Shika		Shika, Zaria, Kaduna State	Sabon Gari	Kaduna
3181	KD/0223	KD/0223/S/3	A B U Teaching Hospital, Shika		Shika, Zaria, Kaduna State	Sabon Gari	Kaduna
3182	KD/0223	KD/0223/S/1	A B U Teaching Hospital, Shika		Shika, Zaria, Kaduna State	Sabon Gari	Kaduna
3183	KD/0223	KD/0223/S/14	A B U Teaching Hospital, Shika		Shika, Zaria, Kaduna State	Sabon Gari	Kaduna
3184	KD/0226	KD/0226/S/5	Biba Hospital		AE 7, Matazu Close, off Rigachukwu Road, T/Wada, Kaduna	Kaduna South	Kaduna
3185	KD/0230	KD/0230/S/1	Jibrin Mai-Gwari Hospital		Birnin Gwari, Kaduna	Birnin Gwari	Kaduna
3186	KD/0230	KD/0230/S/2	Jibrin Mai-Gwari Hospital		Birnin Gwari, Kaduna	Birnin Gwari	Kaduna
3187	KD/0230	KD/0230/S/5	Jibrin Mai-Gwari Hospital		Birnin Gwari, Kaduna	Birnin Gwari	Kaduna
3188	KD/0230	KD/0230/S/3	Jibrin Mai-Gwari Hospital		Birnin Gwari, Kaduna	Birnin Gwari	Kaduna
3189	KD/0231	KD/0231/S/4	National Board for Tech. Education (NBTE), Medical Centre		Plot B Bida Road, Kaduna	Kaduna North	Kaduna
3190	KD/0231	KD/0231/S/5	National Board for Tech. Education (NBTE), Medical Centre		Plot B Bida Road, Kaduna	Kaduna North	Kaduna
3191	KD/0239	KD/0239/S/6	Horeb Specialist Hospital		4 Force Road Maraba Estate, Kaduna	Kaduna North	Kaduna
3192	KD/0244	KD/0244/S/3	Mayfair Clinic Nigeria Ltd.		8 Crescent Rd Sabon Gari, Zaria	Zaria	Kaduna
3193	KD/0252	KD/0252/S/10	Skills Specialist Clinic		No. 16 Kurmi Avenue, Kabala Costain, Kaduna	Kaduna North	Kaduna
3194	KD/0253	KD/0253/S/1	Sovanel Orthopaedic Clinic & Med. Clinic		Abubakar Kigo Rd, New Extension, Kaduna	Kaduna North	Kaduna

3195	KD/0253	KD/0253/S/10	Sovanel Orthopaedic Clinic & Med. Clinic		Abubakar Kigo Rd, New Extension, Kaduna	Kaduna North	Kaduna
3196	KD/0255	KD/0255/S/5	Biomedical Laboratory Services	Laboratory	33F Obekpa Road, Off Independence Way, Kaduna	Kaduna North	Kaduna
3197	KD/0256	KD/0256/S/5	Dan-Musa X-ray Centre	Radiology	No. 6 Dan-Musa/Yoruba Road, Ori-Apata, Kaduna	Kaduna North	Kaduna
3198	KD/0257	KD/0257/S/4	Dr. Gwamna Awan General Hospital	Pharmacy	Kakuri, Kaduna	Kaduna South	Kaduna
3199	KD/0258	KD/0258/S/4	Dunuya Pharmacy Ltd	Pharmacy	KK2 Abijan Street, Kaduna	Kaduna North	Kaduna
3200	KD/0259	KD/0259/S/5	E.E. Laboratories	Laboratory	W. 7 Ahmadu Bello Way, Katsina Road Round-About, Kaduna	Kaduna North	Kaduna
3201	KD/0260	KD/0260/S/4	GENS Pharmacy Ltd	Pharmacy	A5 College Road, Kaduna	Kaduna South	Kaduna
3202	KD/0261	KD/0261/S/12	Hi-Fi Consultant Clinic	ENT	2A Dalali Rd Malali Kaduna	Kaduna North	Kaduna
3203	KD/0264	KD/0264/S/4	Ismus Pharmacy Ltd	Pharmacy	B1/B2 Lafia Road, Kaduna	Kaduna North	Kaduna
3204	KD/0266	KD/0266/S/8	Lloyd Dental Clinic	Dental	Duniya House (Nasco Carpets), AM8 Abuja Road/Lagos Street, Kaduna	Kaduna North	Kaduna
3205	KD/0270	KD/0270/S/4	Nay-Day Pharmacy Ltd	Pharmacy	1 Waziri Ibrahim Crescent, Kaduna	Kaduna North	Kaduna
3206	KD/0271	KD/0271/S/8	NDA Dental Clinic	Dental	NDA, Kaduna	Kaduna North	Kaduna
3207	KD/0272	KD/0272/S/4	NDA Drug Revolving Scheme	Pharmacy	NDA Medical Centre, Kaduna	Kaduna North	Kaduna
3208	KD/0274	KD/0274/S/4	Osbud-K Pharmacy	Pharmacy	KK 12 Yoruba Road, Kaduna	Kaduna North	Kaduna
3209	KD/0275	KD/0275/S/4	Phoenix Pharmacy & Stores	Pharmacy	No. 2 Zaria/Shagari Road, Narayi, Kaduna	Chikun	Kaduna
3210	KD/0277	KD/0277/S/4	Somid Pharmacy Ltd	Pharmacy	AV8 Benin/Kano Road, Kaduna	Kaduna North	Kaduna
3211	KD/0278	KD/0278/S/4	Sufficient Pharmacy & Stores	Pharmacy	No 23 Ribadu Road, Off Kofar Gamji, Kaduna	Kaduna North	Kaduna
3212	KD/0279	KD/0279/S/7	X-ray Ultrasound Scan Centre	Radiology	XX3, Dauda Road, Kaduna	Kaduna North	Kaduna
3213	KD/0280	KD/0280/S/1	Diamond Specialist Hospital		4 MM Lawan Jaafaru Isa Road, GRA, Off Isa Kaita Road	Kaduna North	Kaduna
3214	KD/0280	KD/0280/S/5	Diamond Specialist Hospital		4 MM Lawan Jaafaru Isa Road, GRA, Off Isa Kaita Road	Kaduna North	Kaduna
3215	KD/0280	KD/0280/S/6	Diamond Specialist Hospital		4 MM Lawan Jaafaru Isa Road, GRA, Off Isa Kaita Road	Kaduna North	Kaduna
3216	KD/0280	KD/0280/S/3	Diamond Specialist Hospital		4 MM Lawan Jaafaru Isa Road, GRA, Off Isa Kaita Road	Kaduna North	Kaduna
3217	KD/0280	KD/0280/S/2	Diamond Specialist Hospital		4 MM Lawan Jaafaru Isa Road, GRA, Off Isa Kaita Road	Kaduna North	Kaduna
3218	KD/0282	KD/0282/S/15	Chaha Eye Hospital & General Clinics		42B Jabi Road off Alkali Road GRA Kaduna	Kaduna North	Kaduna
3219	KD/0284	KD/0284/S/5	Zaria Clinic & Medical Centre		16 Hospital Road T/Wada, Zaria	Zaria	Kaduna
3220	KD/0284	KD/0284/S/7	Zaria Clinic & Medical Centre		16 Hospital Road T/Wada, Zaria	Zaria	Kaduna
3221	KD/0286	KD/0286/S/14	Jalva Radiological Diagnostic Centre	USS, Radiology	Babadoko Shopping Complex, Zaria	Zaria	Kaduna
3222	KD/0286	KD/0286/S/7	Jalva Radiological Diagnostic Centre	USS, Radiology	Babadoko Shopping Complex, Zaria	Zaria	Kaduna
3223	KD/0287	KD/0287/S/13	Loryb Eye Clinic	Optometry	9A Sokoto Road, Sabon Gari, Zaria	Zaria	Kaduna
3224	KD/0292	KD/0292/S/5	E.C. Frank Medical Centre Diagnostic Labs. AJ	Laboratory	29/31 Benin Street, Yoruba Road, Kaduna	Kaduna North	Kaduna
3225	KD/0294	KD/0294/S/3	Salvation Hospital & Maternity		2B Algbria Crescent, Barnawa, Kaduna	Kaduna South	Kaduna
3226	KD/0294	KD/0294/S/2	Salvation Hospital & Maternity		2B Algbria Crescent, Barnawa, Kaduna	Kaduna South	Kaduna
3227	KD/0295	KD/0295/S/14	Sa'a Scan Service	USS	10B Na-Magwatmatse Road, U/Rimi, Kaduna	Kaduna North	Kaduna
3228	KD/0296	KD/0296/S/4	Fomwan Hospital		28 Musana Road, Malali, Kaduna	Kaduna North	Kaduna
3229	KD/0296	KD/0296/S/5	Fomwan Hospital		28 Musana Road, Malali, Kaduna	Kaduna North	Kaduna
3230	KD/0298	KD/0298/S/1	Musaka Specialist Hospital		10B Nagwamatse Road, U/Rimi, Kaduna	Kaduna North	Kaduna
3231	KD/0298	KD/0298/S/5	Musaka Specialist Hospital		10B Nagwamatse Road, U/Rimi, Kaduna	Kaduna North	Kaduna
3232	KD/0301	KD/0301/S/4	Farmek Pharmacy Ltd	Pharmacy	80 Iya Road, Sabon Gari, Zaria	Sabon Gari	Kaduna
3233	KD/0302	KD/0302/S/4	Livseal Pharmacy	Pharmacy	63 Sokoto Road, Samaru, Zaria	Sabon Gari	Kaduna
3234	KD/0308	KD/0308/S/1	Albarka Hospital		BZ170, Offa Road by Sardauna Crescent, Kaduna	Kaduna North	Kaduna

3235	KD/0308	KD/0308/S/3	Albarka Hospital		BZ170, Offa Road by Sardauna Crescent, Kaduna	Kaduna North	Kaduna
3236	KD/0309	KD/0309/S/1	St Louis Hospital	Internal Medicine, Laboratory, Radiology, Pharmacy, Opthamology, General Surgey	Zonkwa Kaduna Road, Zangon Kataf	Zangon Kataf	Kaduna
3237	KD/0309	KD/0309/S/15	St Louis Hospital	Internal Medicine, Laboratory, Radiology, Pharmacy, Opthamology, General Surgey	Zonkwa Kaduna Road, Zangon Kataf	Zangon Kataf	Kaduna
3238	KD/0309	KD/0309/S/4	St Louis Hospital	Internal Medicine, Laboratory, Radiology, Pharmacy, Opthamology, General Surgey	Zonkwa Kaduna Road, Zangon Kataf	Zangon Kataf	Kaduna
3239	KD/0312	KD/0312/S/5	Monarch Medical Laboratory		NK 12 Arochukwu by Lokoja Road, Kaduna	Kaduna North	Kaduna
3240	KD/0313	KD/0313/S/4	Joniks Pharmacy & Store Ltd	Pharmacy	AL 17 Abuja Road, Kaduna	Kaduna North	Kaduna
3241	KD/0319	KD/0319/S/4	Dabo Mohammed Lere Memorial Hospital		Along Jos Road, Saminaka	Lere	Kaduna
3242	KD/0319	KD/0319/S/5	Dabo Mohammed Lere Memorial Hospital		Along Jos Road, Saminaka	Lere	Kaduna
3243	KD/0319	KD/0319/S/7	Dabo Mohammed Lere Memorial Hospital		Along Jos Road, Saminaka	Lere	Kaduna
3244	KD/0327	KD/0327/S/2	Muslim Specialist Hospital		Wusasa, Zaria	Sabon Gari	Kaduna
3245	KD/0327	KD/0327/S/3	Muslim Specialist Hospital		Wusasa, Zaria	Sabon Gari	Kaduna
3246	KD/0327	KD/0327/S/1	Muslim Specialist Hospital		Wusasa, Zaria	Sabon Gari	Kaduna
3247	KD/0330	KD/0330/S/2	Aloaye Clinic and Maternity		4 Gaskiya Road, Zaria	Zaria	Kaduna
3248	KD/0333	KD/0333/S/6	Harmony Hospital		38 Nuhu Aliyu Crescent, Barnawa Layout, Kaduna	Kaduna North	Kaduna
3249	KD/0333	KD/0333/S/3	Harmony Hospital		38 Nuhu Aliyu Crescent, Barnawa Layout, Kaduna	Kaduna North	Kaduna
3250	KD/0333	KD/0333/S/2	Harmony Hospital		38 Nuhu Aliyu Crescent, Barnawa Layout, Kaduna	Kaduna North	Kaduna
3251	KD/0333	KD/0333/S/1	Harmony Hospital		38 Nuhu Aliyu Crescent, Barnawa Layout, Kaduna	Kaduna North	Kaduna
3252	KD/0333	KD/0333/S/5	Harmony Hospital		38 Nuhu Aliyu Crescent, Barnawa Layout, Kaduna	Kaduna North	Kaduna
3253	KD/0333	KD/0333/S/14	Harmony Hospital		38 Nuhu Aliyu Crescent, Barnawa Layout, Kaduna	Kaduna North	Kaduna
3254	KD/0339	KD/0339/S/4	Shubal Pharmacy		D1/D2 Akali Road, near Muritala Square, GRA, Kaduna	Kaduna North	Kaduna
3255	KD/0340	KD/0340/S/14	Crystal Clinic and Maternity		6 Makera Road, Opp, Kaduna South LGA Secretariat, Kakuri	Kaduna South	Kaduna
3256	KD/0342	KD/0342/S/3	Dallof Special;ist Hospital Limitred		136 Bayajida Roadf, Angwa Rimi, GRA Kaduna	Kaduna North	Kaduna
3257	KD/0342	KD/0342/S/4	Dallof Special;ist Hospital Limitred		136 Bayajida Roadf, Angwa Rimi, GRA Kaduna	Kaduna North	Kaduna
3258	KD/0342	KD/0342/S/5	Dallof Special;ist Hospital Limitred		136 Bayajida Roadf, Angwa Rimi, GRA Kaduna	Kaduna North	Kaduna
3259	KD/0346	KD/0346/S/8	Dialogue Sepcialist Clinics Ltd.		#1, Kukan Road, U/Dosa, Kaduna	Kaduna North	Kaduna
3260	KD/0346	KD/0346/S/1	Dialogue Sepcialist Clinics Ltd.		#1, Kukan Road, U/Dosa, Kaduna	Kaduna North	Kaduna
3261	KD/0346	KD/0346/S/4	Dialogue Sepcialist Clinics Ltd.		#1, Kukan Road, U/Dosa, Kaduna	Kaduna North	Kaduna
3262	KD/0346	KD/0346/S/6	Dialogue Sepcialist Clinics Ltd.		#1, Kukan Road, U/Dosa, Kaduna	Kaduna North	Kaduna
3263	KD/0346	KD/0346/S/15	Dialogue Sepcialist Clinics Ltd.		#1, Kukan Road, U/Dosa, Kaduna	Kaduna North	Kaduna
3264	KD/0346	KD/0346/S/3	Dialogue Sepcialist Clinics Ltd.		#1, Kukan Road, U/Dosa, Kaduna	Kaduna North	Kaduna
3265	KD/0346	KD/0346/S/12	Dialogue Sepcialist Clinics Ltd.		#1, Kukan Road, U/Dosa, Kaduna	Kaduna North	Kaduna
3266	KD/0346	KD/0346/S/2	Dialogue Sepcialist Clinics Ltd.		#1, Kukan Road, U/Dosa, Kaduna	Kaduna North	Kaduna
3267	KD/0346	KD/0346/S/5	Dialogue Sepcialist Clinics Ltd.		#1, Kukan Road, U/Dosa, Kaduna	Kaduna North	Kaduna
3268	KD/0351	KD/0351/S/2	Terra Medical Center		# 5, Alh nasiru Idi close, Hanwa new extension, Zaria	Zaria	Kaduna

3269	KD/0361	KD/0361/S/4	Alba Clinics & Medical Centre Ltd		25, Constitution Rd, Kaduna	Kaduna North	Kaduna
3270	KD/0361	KD/0361/S/7	Alba Clinics & Medical Centre Ltd		25, Constitution Rd, Kaduna	Kaduna North	Kaduna
3271	KD/0362	KD/0362/S/3	Dan Giwa Memorial Hospital		By Noruin Guest House, Maraban Rido kaduna, Kaduna State	Kaduna South	Kaduna
3272	KD/0368	KD/0368/S/4	Jubril Maigwari Memorial Hospital		Birin Gwari, Kaduna, Kaduna State.	Birnin Gwari	Kaduna
3273	KD/0372	KD/0372/S/5	Fisabil Hospital		22, NIMASA Road, off Katuru Road, From Rabah Road, Badarawa, Kaduna, Kaduna State	Kaduna North	Kaduna
3274	KD/0372	KD/0372/S/7	Fisabil Hospital		22, NIMASA Road, off Katuru Road, From Rabah Road, Badarawa, Kaduna, Kaduna State	Kaduna North	Kaduna
3275	KD/0374	KD/0374/S/14	Ahmadu Bello University Health Services (Main Campus)		Samaru Zaria, Kaduna State	Zaria	Kaduna
3276	KD/0375	KD/0375/S/5	First Scan Diagnostic Services		4 Alkakli Road Kaduna, Kaduna State	Kaduna North	Kaduna
3277	KD/0375	KD/0375/S/7	First Scan Diagnostic Services		4 Alkakli Road Kaduna, Kaduna State	Kaduna North	Kaduna
3278	KD/0376	KD/0376/S/3	Pal Hospital, Zaria		No. 15, River Road, GRA, Zaria, Kaduna State	Sabon Gari	Kaduna
3279	KD/0376	KD/0376/S/4	Pal Hospital, Zaria		No. 15, River Road, GRA, Zaria, Kaduna State	Sabon Gari	Kaduna
3280	KD/0376	KD/0376/S/5	Pal Hospital, Zaria		No. 15, River Road, GRA, Zaria, Kaduna State	Sabon Gari	Kaduna
3281	KD/0376	KD/0376/S/7	Pal Hospital, Zaria		No. 15, River Road, GRA, Zaria, Kaduna State	Sabon Gari	Kaduna
3282	KD/0379	KD/0379/S/3	Belside Hospital		No. 5 Kanta Road, Kaduna, Kaduna State	Kaduna North	Kaduna
3283	KD/0380	KD/0380/S/4	Garden City Specialist Hospital		No.2 Sultan Road Ungwa Rimi, Kaduna State	Kaduna South	Kaduna
3284	KD/0380	KD/0380/S/3	Garden City Specialist Hospital		No.2 Sultan Road Ungwa Rimi, Kaduna State	Kaduna South	Kaduna
3285	KD/0380	KD/0380/S/14	Garden City Specialist Hospital		No.2 Sultan Road Ungwa Rimi, Kaduna State	Kaduna South	Kaduna
3286	KD/0380	KD/0380/S/5	Garden City Specialist Hospital		No.2 Sultan Road Ungwa Rimi, Kaduna State	Kaduna South	Kaduna
3287	KD/0381	KD/0381/S/7	Ahaz Medical & Diagnostic Services Ltd.		2A. Dala By Korau Road, Ungwa Rimi GRA Kaduna State	Kaduna North	Kaduna
3288	KD/0381	KD/0381/S/5	Ahaz Medical & Diagnostic Services Ltd.		2A. Dala By Korau Road, Ungwa Rimi GRA Kaduna State	Kaduna North	Kaduna
3289	KD/0385	KD/0385/S/5	DNA LABS		Q5, Danja Road Off Katuru Road, Ungwa Sarki Kaduna State.	Kaduna North	Kaduna
3290	KD/0390	KD/0390/S/8	Pearl Dental Clinic		4, Isa Kaita Road, Kaduna State	Kaduna North	Kaduna
3291	KD/0391	KD/0391/S/1	NNPC Industrial Hospital Kaduna		Km 16, Kachia Road, NNPC Housing Estate Kaduna State	Kaduna South	Kaduna
3292	KD/0391	KD/0391/S/7	NNPC Industrial Hospital Kaduna		Km 16, Kachia Road, NNPC Housing Estate Kaduna State	Kaduna South	Kaduna
3293	KD/0391	KD/0391/S/5	NNPC Industrial Hospital Kaduna		Km 16, Kachia Road, NNPC Housing Estate Kaduna State	Kaduna South	Kaduna
3294	KD/0391	KD/0391/S/3	NNPC Industrial Hospital Kaduna		Km 16, Kachia Road, NNPC Housing Estate Kaduna State	Kaduna South	Kaduna
3295	KD/0391	KD/0391/S/2	NNPC Industrial Hospital Kaduna		Km 16, Kachia Road, NNPC Housing Estate Kaduna State	Kaduna South	Kaduna
3296	KD/0391	KD/0391/S/6	NNPC Industrial Hospital Kaduna		Km 16, Kachia Road, NNPC Housing Estate Kaduna State	Kaduna South	Kaduna
3297	KD/0393	KD/0393/S/14	University Health Services		Ahmadu Bello University Zaria, Kaduna State	Zaria	Kaduna
3298	KD/0393	KD/0393/S/12	University Health Services		Ahmadu Bello University Zaria, Kaduna State	Zaria	Kaduna
3299	KD/0393	KD/0393/S/6	University Health Services		Ahmadu Bello University Zaria, Kaduna State	Zaria	Kaduna
3300	KD/0393	KD/0393/S/3	University Health Services		Ahmadu Bello University Zaria, Kaduna State	Zaria	Kaduna
3301	KD/0395	KD/0395/S/5	Women’s Health International		52, Hanwa GRA Zaria, Kaduna State.	Zaria	Kaduna
3302	KD/0395	KD/0395/S/3	Women’s Health International		52, Hanwa GRA Zaria, Kaduna State.	Zaria	Kaduna
3303	KD/0395	KD/0395/S/14	Women’s Health International		52, Hanwa GRA Zaria, Kaduna State.	Zaria	Kaduna
3304	KD/0396	KD/0396/S/10	AP Smart Hospital		Gwagwada Street, Trikania, Kaduna State.	Kaduna South	Kaduna

3305	KD/0398	KD/0398/S/15	Rakiya Memorial Eye & Specialist Hospitals		1 Karau Road, Angwan Rimi GRA Kaduna, Kaduna State	Kaduna North	Kaduna
3306	KG/0001	KG/0001/S/4	ASCL Medical Centre, Ajaokuta		Ajaokuta Steel Co. Ltd., Kogi State	Ajaokuta	Kogi
3307	KG/0001	KG/0001/S/5	ASCL Medical Centre, Ajaokuta		Ajaokuta Steel Co. Ltd., Kogi State	Ajaokuta	Kogi
3308	KG/0001	KG/0001/S/1	ASCL Medical Centre, Ajaokuta		Ajaokuta Steel Co. Ltd., Kogi State	Ajaokuta	Kogi
3309	KG/0001	KG/0001/S/6	ASCL Medical Centre, Ajaokuta		Ajaokuta Steel Co. Ltd., Kogi State	Ajaokuta	Kogi
3310	KG/0001	KG/0001/S/3	ASCL Medical Centre, Ajaokuta		Ajaokuta Steel Co. Ltd., Kogi State	Ajaokuta	Kogi
3311	KG/0001	KG/0001/S/2	ASCL Medical Centre, Ajaokuta		Ajaokuta Steel Co. Ltd., Kogi State	Ajaokuta	Kogi
3312	KG/0004	KG/0004/S/6	Kogi State University Teaching Hospital, Anyigba	Obst, & Gynae. Peadiatrics, General Surgery, Internal Medicine, Pharmacy, Laboratory	Ayingba	Dekina	Kogi
3313	KG/0004	KG/0004/S/5	Kogi State University Teaching Hospital, Anyigba	Obst, & Gynae. Peadiatrics, General Surgery, Internal Medicine, Pharmacy, Laboratory	Ayingba	Dekina	Kogi
3314	KG/0004	KG/0004/S/3	Kogi State University Teaching Hospital, Anyigba	Obst, & Gynae. Peadiatrics, General Surgery, Internal Medicine, Pharmacy, Laboratory	Ayingba	Dekina	Kogi
3315	KG/0004	KG/0004/S/1	Kogi State University Teaching Hospital, Anyigba	Obst, & Gynae. Peadiatrics, General Surgery, Internal Medicine, Pharmacy, Laboratory	Ayingba	Dekina	Kogi
3316	KG/0004	KG/0004/S/2	Kogi State University Teaching Hospital, Anyigba	Obst, & Gynae. Peadiatrics, General Surgery, Internal Medicine, Pharmacy, Laboratory	Ayingba	Dekina	Kogi
3317	KG/0004	KG/0004/S/4	Kogi State University Teaching Hospital, Anyigba	Obst, & Gynae. Peadiatrics, General Surgery, Internal Medicine, Pharmacy, Laboratory	Ayingba	Dekina	Kogi
3318	KG/0005	KG/0005/S/4	General Hospital Dekina		Dekina	Dekina	Kogi
3319	KG/0005	KG/0005/S/3	General Hospital Dekina		Dekina	Dekina	Kogi
3320	KG/0005	KG/0005/S/5	General Hospital Dekina		Dekina	Dekina	Kogi
3321	KG/0005	KG/0005/S/1	General Hospital Dekina		Dekina	Dekina	Kogi
3322	KG/0005	KG/0005/S/2	General Hospital Dekina		Dekina	Dekina	Kogi
3323	KG/0009	KG/0009/S/5	General Hospital, Idah		Idah	Idah	Kogi
3324	KG/0009	KG/0009/S/7	General Hospital, Idah		Idah	Idah	Kogi
3325	KG/0009	KG/0009/S/1	General Hospital, Idah		Idah	Idah	Kogi
3326	KG/0009	KG/0009/S/4	General Hospital, Idah		Idah	Idah	Kogi
3327	KG/0009	KG/0009/S/3	General Hospital, Idah		Idah	Idah	Kogi
3328	KG/0009	KG/0009/S/2	General Hospital, Idah		Idah	Idah	Kogi
3329	KG/0009	KG/0009/S/6	General Hospital, Idah		Idah	Idah	Kogi
3330	KG/0012	KG/0012/S/1	Niger Hospital			Lokoja	Kogi
3331	KG/0012	KG/0012/S/6	Niger Hospital			Lokoja	Kogi
3332	KG/0012	KG/0012/S/3	Niger Hospital			Lokoja	Kogi

3333	KG/0012	KG/0012/S/2	Niger Hospital			Lokoja	Kogi
3334	KG/0017	KG/0017/S/2	General Hospital, Okene		Okene	Okene	Kogi
3335	KG/0017	KG/0017/S/6	General Hospital, Okene		Okene	Okene	Kogi
3336	KG/0017	KG/0017/S/3	General Hospital, Okene		Okene	Okene	Kogi
3337	KG/0017	KG/0017/S/4	General Hospital, Okene		Okene	Okene	Kogi
3338	KG/0017	KG/0017/S/5	General Hospital, Okene		Okene	Okene	Kogi
3339	KG/0017	KG/0017/S/1	General Hospital, Okene		Okene	Okene	Kogi
3340	KG/0019	KG/0019/S/3	Ecwa Hospital		Mission Road Egbe	Yagba West	Kogi
3341	KG/0019	KG/0019/S/6	Ecwa Hospital		Mission Road Egbe	Yagba West	Kogi
3342	KG/0019	KG/0019/S/1	Ecwa Hospital		Mission Road Egbe	Yagba West	Kogi
3343	KG/0019	KG/0019/S/2	Ecwa Hospital		Mission Road Egbe	Yagba West	Kogi
3344	KG/0019	KG/0019/S/4	Ecwa Hospital		Mission Road Egbe	Yagba West	Kogi
3345	KG/0019	KG/0019/S/5	Ecwa Hospital		Mission Road Egbe	Yagba West	Kogi
3346	KG/0020	KG/0020/S/5	Federal Medical Center Lokoja		Lokoja	Lokoja	Kogi
3347	KG/0020	KG/0020/S/1	Federal Medical Center Lokoja		Lokoja	Lokoja	Kogi
3348	KG/0020	KG/0020/S/3	Federal Medical Center Lokoja		Lokoja	Lokoja	Kogi
3349	KG/0020	KG/0020/S/2	Federal Medical Center Lokoja		Lokoja	Lokoja	Kogi
3350	KG/0020	KG/0020/S/4	Federal Medical Center Lokoja		Lokoja	Lokoja	Kogi
3351	KG/0020	KG/0020/S/6	Federal Medical Center Lokoja		Lokoja	Lokoja	Kogi
3352	KG/0021	KG/0021/S/6	General Hospital, Kabba		Kabba	Kabba/Bunu	Kogi
3353	KG/0021	KG/0021/S/5	General Hospital, Kabba		Kabba	Kabba/Bunu	Kogi
3354	KG/0021	KG/0021/S/3	General Hospital, Kabba		Kabba	Kabba/Bunu	Kogi
3355	KG/0021	KG/0021/S/2	General Hospital, Kabba		Kabba	Kabba/Bunu	Kogi
3356	KG/0021	KG/0021/S/1	General Hospital, Kabba		Kabba	Kabba/Bunu	Kogi
3357	KG/0023	KG/0023/S/2	General Hospital, Ankpa		Ankpa	Ankpa	Kogi
3358	KG/0023	KG/0023/S/4	General Hospital, Ankpa		Ankpa	Ankpa	Kogi
3359	KG/0023	KG/0023/S/5	General Hospital, Ankpa		Ankpa	Ankpa	Kogi
3360	KG/0023	KG/0023/S/1	General Hospital, Ankpa		Ankpa	Ankpa	Kogi
3361	KG/0033	KG/0033/S/5	General Hospital, Ayetoro Gbede		Ayetoro Gbede	Ijumu	Kogi
3362	KG/0039	KG/0039/S/2	Omeza Ali	O & G, Peadiatrics, General Surgery, Internal Medicine	Behind Mobil Petrol Station, Off Old Kabba/Okene Rd., Lokoja	Lokoja	Kogi
3363	KG/0039	KG/0039/S/1	Omeza Ali	O & G, Peadiatrics, General Surgery, Internal Medicine	Behind Mobil Petrol Station, Off Old Kabba/Okene Rd., Lokoja	Lokoja	Kogi
3364	KG/0039	KG/0039/S/6	Omeza Ali	O & G, Peadiatrics, General Surgery, Internal Medicine	Behind Mobil Petrol Station, Off Old Kabba/Okene Rd., Lokoja	Lokoja	Kogi
3365	KG/0039	KG/0039/S/3	Omeza Ali	O & G, Peadiatrics, General Surgery, Internal Medicine	Behind Mobil Petrol Station, Off Old Kabba/Okene Rd., Lokoja	Lokoja	Kogi
3366	KG/0041	KG/0041/S/4	Suraj Pharmacy & Gen. Ent. Ltd	Pharmacy	2 Ibrahim Aliyu Street G.R.A. Lokoja	Lokoja	Kogi
3367	KG/0042	KG/0042/S/4	Dave-Mercy Pharmacy	Pharmacy	Salihu Ibrahim Rd., Lokoja	Lokoja	Kogi
3368	KG/0043	KG/0043/S/4	Saleez Drugs/Pharmacy	Pharmacy	8. Hospital Rd., Okene	Okene	Kogi
3369	KG/0044	KG/0044/S/4	Broadway Pharmacy	Pharmacy	Osaro Way, Kabba	Kabba/Bunu	Kogi
3370	KG/0046	KG/0046/S/3	Federal College Of Education		Federal College Of Education Okene	Okene	Kogi
3371	KG/0047	KG/0047/S/1	Grimard Catholic Hospital		Grimard Catholic Hospital Anyiagba	Dekina	Kogi
3372	KG/0047	KG/0047/S/6	Grimard Catholic Hospital		Grimard Catholic Hospital Anyiagba	Dekina	Kogi
3373	KG/0047	KG/0047/S/7	Grimard Catholic Hospital		Grimard Catholic Hospital Anyiagba	Dekina	Kogi

3374	KG/0047	KG/0047/S/3	Grimard Catholic Hospital		Grimard Catholic Hospital Anyiagba	Dekina	Kogi
3375	KG/0047	KG/0047/S/2	Grimard Catholic Hospital		Grimard Catholic Hospital Anyiagba	Dekina	Kogi
3376	KG/0047	KG/0047/S/4	Grimard Catholic Hospital		Grimard Catholic Hospital Anyiagba	Dekina	Kogi
3377	KG/0047	KG/0047/S/5	Grimard Catholic Hospital		Grimard Catholic Hospital Anyiagba	Dekina	Kogi
3378	KG/0052	KG/0052/S/4	Opeyemi Pharmacy	Pharmacy	No 52 Market Road Egbe	Yagba West	Kogi
3379	KG/0053	KG/0053/S/4	Yotan Pharmacy	Pharmacy	No. 19 Lafia Street, Okene	Kabba/Bunu	Kogi
3380	KG/0058	KG/0058/S/4	Bonafide Pharmacy Ltd.	Pharmacy	No 19, IBB Way Almost Opp. Bank Of The North Lokoja	Lokoja	Kogi
3381	KG/0059	KG/0059/S/4	Egbe Hospital	Radiology, Laboratory	Hospital Road Egbe Road	Yagba West	Kogi
3382	KG/0061	KG/0061/S/5	Federal Polytechnic Medical Centre		Idah - Ajaka Road, Kogi State	Idah	Kogi
3383	KG/0061	KG/0061/S/4	Federal Polytechnic Medical Centre		Idah - Ajaka Road, Kogi State	Idah	Kogi
3384	KG/0062	KG/0062/S/4	Fed. College of Education Medical Centre, Okene		Okene, Kogi State	Okene	Kogi
3385	KG/0062	KG/0062/S/5	Fed. College of Education Medical Centre, Okene		Okene, Kogi State	Okene	Kogi
3386	KG/0069	KG/0069/S/3	PACIFIC HOSPITAL	O&G	OYIFU LAYOUT	Dekina	Kogi
3387	KG/0070	KG/0070/S/5	SAUKI MEDICAL DIAGNOSTIC LAB.	Laboratory	5,IDAKOYIBO STREET, OKENE	Okene	Kogi
3388	KG/0074	KG/0074/S/5	Police Clinic		Police State Command, Lokoja	Lokoja	Kogi
3389	KG/0082	KG/0082/S/5	Good Shepherd Medical Centre		No 10, Egume /Ankpa Road, Anyigba, Kogi State.	Dekina	Kogi
3390	KG/0082	KG/0082/S/4	Good Shepherd Medical Centre		No 10, Egume /Ankpa Road, Anyigba, Kogi State.	Dekina	Kogi
3391	KG/0083	KG/0083/S/10	Cross Nation Consultant Hospital		Phase 2, Behind Rewal House, Lokoja, Kogi State	Lokoja	Kogi
3392	KG/0084	KG/0084/S/1	Kogi State Specialist Hospital	Primary, Dental, ENT, Internal Medicine, O&G, Ophthamology, Physiotherapy, General Surgery	Along Okene-Kabba Road	Lokoja	Kogi
3393	KG/0084	KG/0084/S/15	Kogi State Specialist Hospital	Primary, Dental, ENT, Internal Medicine, O&G, Ophthamology, Physiotherapy, General Surgery	Along Okene-Kabba Road	Lokoja	Kogi
3394	KG/0084	KG/0084/S/2	Kogi State Specialist Hospital	Primary, Dental, ENT, Internal Medicine, O&G, Ophthamology, Physiotherapy, General Surgery	Along Okene-Kabba Road	Lokoja	Kogi
3395	KG/0084	KG/0084/S/12	Kogi State Specialist Hospital	Primary, Dental, ENT, Internal Medicine, O&G, Ophthamology, Physiotherapy, General Surgery	Along Okene-Kabba Road	Lokoja	Kogi
3396	KG/0084	KG/0084/S/8	Kogi State Specialist Hospital	Primary, Dental, ENT, Internal Medicine, O&G, Ophthamology, Physiotherapy, General Surgery	Along Okene-Kabba Road	Lokoja	Kogi
3397	KG/0084	KG/0084/S/11	Kogi State Specialist Hospital	Primary, Dental, ENT, Internal Medicine, O&G, Ophthamology, Physiotherapy, General Surgery	Along Okene-Kabba Road	Lokoja	Kogi
3398	KG/0084	KG/0084/S/3	Kogi State Specialist Hospital	Primary, Dental, ENT, Internal Medicine, O&G, Ophthamology, Physiotherapy, General Surgery	Along Okene-Kabba Road	Lokoja	Kogi
3399	KG/0087	KG/0087/S/8	Divine Dental Clinic		Behind Equity Plaza (CAC Building)	Lokoja	Kogi
3400	KG/0088	KG/0088/S/4	Bonafide Pharmacy Limited		OPP. Bank of The North, Lokoja	Lokoja	Kogi
3401	KG/0094	KG/0094/S/4	Shushan Pharmacy LTD		5, IBB Way, By FMC junction, Lokoja, Kogi Stat	Lokoja	Kogi

3402	KG/0096	KG/0096/S/1	Zenith Specialist Hospital		Olunle Street, Near Army Signal, Lokoja, Kogi Stat	Lokoja	Kogi
3403	KG/0101	KG/0101/S/13	Eyez Alive		Goshen Plaza, Opp. old living faith junction, lokongoma, phase 1, Lokoja, Kogi state	Lokoja	Kogi
3404	KG/0104	KG/0104/S/3	Hillcrest Clinic And Maternity		Opposite Dunamis church, along Ganaja road, lokoja, Kogi state	Lokoja	Kogi
3405	KG/0106	KG/0106/S/3	Rehoboth Specialist Hospital		2nd gate, phase II, lokongoma, lokoja, Kogi state	Lokoja	Kogi
3406	KG/0106	KG/0106/S/6	Rehoboth Specialist Hospital		2nd gate, phase II, lokongoma, lokoja, Kogi state	Lokoja	Kogi
3407	KG/0107	KG/0107/S/3	Neighbour Multicare Hospital And Women Welfare		Omata street, Anyigba, Kogi state	Dekina	Kogi
3408	KG/0108	KG/0108/S/3	Neighbour Multicare Hospital And Women Welfare		Omata street, Anyigba, Kogi state	Dekina	Kogi
3409	KG/0110	KG/0110/S/13	Carewell Eye Clinic		No 6, Saliu Ibrahim street, lokoja, Kogi state	Lokoja	Kogi
3410	KG/0111	KG/0111/S/3	Concord Hospital		1st 200 unit estate, opposite total filling station, Gadumo, Lokoja, Kogi state	Lokoja	Kogi
3411	KG/0112	KG/0112/S/15	MDR-Lighthouse Medical Eye and Specialist Laser Centre		Government House Road, By Kogi Hotel Round About GRA Lokoja	Lokoja	Kogi
3412	KG/0113	KG/0113/S/3	Helping Hands Women Hospital		Behind T-Square Hotel, Zone 8 Roundabout, Lokoja	Lokoja	Kogi
3413	KN/0003	KN/0003/S/3	Sheikh Mohammed Jidda General Hospital	Medicine, O&G, Paediatrics	Sabon Gari Kano	Sabon Gari	Kaduna
3414	KN/0007	KN/0007/S/6	Hasiya Bayero Peadiatrics Hospital	Paediatrics, Pharmacy	Kano City	Kano Municipal	Kano
3415	KN/0007	KN/0007/S/4	Hasiya Bayero Peadiatrics Hospital	Paediatrics, Pharmacy	Kano City	Kano Municipal	Kano
3416	KN/0015	KN/0015/S/15	Sir Mohammed Sanusi Specialist Hosp.		Yan Kaba, Kano	Nasarawa	Kano
3417	KN/0015	KN/0015/S/6	Sir Mohammed Sanusi Specialist Hosp.		Yan Kaba, Kano	Nasarawa	Kano
3418	KN/0015	KN/0015/S/10	Sir Mohammed Sanusi Specialist Hosp.		Yan Kaba, Kano	Nasarawa	Kano
3419	KN/0015	KN/0015/S/2	Sir Mohammed Sanusi Specialist Hosp.		Yan Kaba, Kano	Nasarawa	Kano
3420	KN/0026	KN/0026/S/5	Khadijat Memorial Hospital		No. 20 Katsina Road, Off Hajj Camp Road, Opp Customs Barrack	Fagge	Kano
3421	KN/0026	KN/0026/S/1	Khadijat Memorial Hospital		No. 20 Katsina Road, Off Hajj Camp Road, Opp Customs Barrack	Fagge	Kano
3422	KN/0026	KN/0026/S/3	Khadijat Memorial Hospital		No. 20 Katsina Road, Off Hajj Camp Road, Opp Customs Barrack	Fagge	Kano
3423	KN/0028	KN/0028/S/2	Mos-Metro Clinic		Sabon Gari, Kano	Fagge	Kano
3424	KN/0028	KN/0028/S/3	Mos-Metro Clinic		Sabon Gari, Kano	Fagge	Kano
3425	KN/0029	KN/0029/S/2	Unicare Clinic & Maternity		Katsina Road , Kano	Fagge	Kano
3426	KN/0029	KN/0029/S/1	Unicare Clinic & Maternity		Katsina Road , Kano	Fagge	Kano
3427	KN/0030	KN/0030/S/1	Topcare Specialist Medical Centre		No; 118 Hadejia Rd , Kano	Nasarawa	Kano
3428	KN/0030	KN/0030/S/2	Topcare Specialist Medical Centre		No; 118 Hadejia Rd , Kano	Nasarawa	Kano
3429	KN/0030	KN/0030/S/3	Topcare Specialist Medical Centre		No; 118 Hadejia Rd , Kano	Nasarawa	Kano
3430	KN/0030	KN/0030/S/9	Topcare Specialist Medical Centre		No; 118 Hadejia Rd , Kano	Nasarawa	Kano
3431	KN/0030	KN/0030/S/6	Topcare Specialist Medical Centre		No; 118 Hadejia Rd , Kano	Nasarawa	Kano
3432	KN/0031	KN/0031/S/3	Ivory Clinic & Maternity		Aminu Kano, Kano	Kano Municipal	Kano
3433	KN/0031	KN/0031/S/2	Ivory Clinic & Maternity		Aminu Kano, Kano	Kano Municipal	Kano
3434	KN/0031	KN/0031/S/1	Ivory Clinic & Maternity		Aminu Kano, Kano	Kano Municipal	Kano
3435	KN/0032	KN/0032/S/8	Premier Clinics		Gyadi Gyadi, Kano	Tarauni	Kano
3436	KN/0032	KN/0032/S/1	Premier Clinics		Gyadi Gyadi, Kano	Tarauni	Kano
3437	KN/0032	KN/0032/S/3	Premier Clinics		Gyadi Gyadi, Kano	Tarauni	Kano

3438	KN/0032	KN/0032/S/2	Premier Clinics		Gyadi Gyadi, Kano	Tarauni	Kano
3439	KN/0034	KN/0034/S/2	Saymays Specialist Hospital Ltd		Zoo Road, Kano	Kano Municipal	Kano
3440	KN/0034	KN/0034/S/6	Saymays Specialist Hospital Ltd		Zoo Road, Kano	Kano Municipal	Kano
3441	KN/0034	KN/0034/S/3	Saymays Specialist Hospital Ltd		Zoo Road, Kano	Kano Municipal	Kano
3442	KN/0035	KN/0035/S/7	International Clinic & Hospital Ltd.		40, Niger Street, Airport Road, Kano	Nasarawa	Kano
3443	KN/0035	KN/0035/S/6	International Clinic & Hospital Ltd.		40, Niger Street, Airport Road, Kano	Nasarawa	Kano
3444	KN/0035	KN/0035/S/8	International Clinic & Hospital Ltd.		40, Niger Street, Airport Road, Kano	Nasarawa	Kano
3445	KN/0035	KN/0035/S/3	International Clinic & Hospital Ltd.		40, Niger Street, Airport Road, Kano	Nasarawa	Kano
3446	KN/0035	KN/0035/S/5	International Clinic & Hospital Ltd.		40, Niger Street, Airport Road, Kano	Nasarawa	Kano
3447	KN/0035	KN/0035/S/2	International Clinic & Hospital Ltd.		40, Niger Street, Airport Road, Kano	Nasarawa	Kano
3448	KN/0035	KN/0035/S/14	International Clinic & Hospital Ltd.		40, Niger Street, Airport Road, Kano	Nasarawa	Kano
3449	KN/0036	KN/0036/S/2	Medicus Clinics		No. 2 Beirut Road, Kano	Kano Municipal	Kano
3450	KN/0036	KN/0036/S/1	Medicus Clinics		No. 2 Beirut Road, Kano	Kano Municipal	Kano
3451	KN/0036	KN/0036/S/5	Medicus Clinics		No. 2 Beirut Road, Kano	Kano Municipal	Kano
3452	KN/0036	KN/0036/S/3	Medicus Clinics		No. 2 Beirut Road, Kano	Kano Municipal	Kano
3453	KN/0037	KN/0037/S/2	Al-Ammeen Medical Centre		Maiduguri Road, Kano	Tarauni	Kano
3454	KN/0037	KN/0037/S/1	Al-Ammeen Medical Centre		Maiduguri Road, Kano	Tarauni	Kano
3455	KN/0037	KN/0037/S/3	Al-Ammeen Medical Centre		Maiduguri Road, Kano	Tarauni	Kano
3456	KN/0037	KN/0037/S/6	Al-Ammeen Medical Centre		Maiduguri Road, Kano	Tarauni	Kano
3457	KN/0041	KN/0041/S/5	Warshi Hospital		Dakata, Kano	Tarauni	Kano
3458	KN/0041	KN/0041/S/2	Warshi Hospital		Dakata, Kano	Tarauni	Kano
3459	KN/0041	KN/0041/S/3	Warshi Hospital		Dakata, Kano	Tarauni	Kano
3460	KN/0041	KN/0041/S/1	Warshi Hospital		Dakata, Kano	Tarauni	Kano
3461	KN/0041	KN/0041/S/6	Warshi Hospital		Dakata, Kano	Tarauni	Kano
3462	KN/0042	KN/0042/S/14	Zaks Clinic and Maternity Ltd.		Opp. Corner Dankura, Behind PDP HQ. Zoo Road, Kano	Kano Municipal	Kano
3463	KN/0042	KN/0042/S/3	Zaks Clinic and Maternity Ltd.		Opp. Corner Dankura, Behind PDP HQ. Zoo Road, Kano	Kano Municipal	Kano
3464	KN/0042	KN/0042/S/6	Zaks Clinic and Maternity Ltd.		Opp. Corner Dankura, Behind PDP HQ. Zoo Road, Kano	Kano Municipal	Kano
3465	KN/0042	KN/0042/S/1	Zaks Clinic and Maternity Ltd.		Opp. Corner Dankura, Behind PDP HQ. Zoo Road, Kano	Kano Municipal	Kano
3466	KN/0042	KN/0042/S/2	Zaks Clinic and Maternity Ltd.		Opp. Corner Dankura, Behind PDP HQ. Zoo Road, Kano	Kano Municipal	Kano
3467	KN/0043	KN/0043/S/3	Global Clinic		No. 1, 33rd Link, Off New Court road, Gyadi-Gyadi, Kano	Kano Municipal	Kano
3468	KN/0043	KN/0043/S/6	Global Clinic		No. 1, 33rd Link, Off New Court road, Gyadi-Gyadi, Kano	Kano Municipal	Kano
3469	KN/0044	KN/0044/S/2	Classic Clinic Ltd.		1a Abbass Road, Kano	Nasarawa	Kano
3470	KN/0044	KN/0044/S/6	Classic Clinic Ltd.		1a Abbass Road, Kano	Nasarawa	Kano
3471	KN/0046	KN/0046/S/2	Ideal Hospital	-	Sabon Gari, Kano	Fagge	Kano
3472	KN/0046	KN/0046/S/1	Ideal Hospital	-	Sabon Gari, Kano	Fagge	Kano
3473	KN/0046	KN/0046/S/3	Ideal Hospital	-	Sabon Gari, Kano	Fagge	Kano
3474	KN/0046	KN/0046/S/5	Ideal Hospital	-	Sabon Gari, Kano	Fagge	Kano
3475	KN/0054	KN/0054/S/1	Arewa Surgery	Surgery, Oncology, ENT, O & G, Pediatrics		Fagge	Kano
3476	KN/0054	KN/0054/S/6	Arewa Surgery	Surgery, Oncology, ENT, O & G, Pediatrics		Fagge	Kano
3477	KN/0054	KN/0054/S/3	Arewa Surgery	Surgery, Oncology, ENT, O & G, Pediatrics		Fagge	Kano
3478	KN/0054	KN/0054/S/12	Arewa Surgery	Surgery, Oncology, ENT, O & G, Pediatrics		Fagge	Kano
3479	KN/0065	KN/0065/S/4	Madaci Pharmacy Ltd.	Pharmacy	No. 60 Tudun Bojuwa, Kurna Asabe, Kano	Fagge	Kano
3480	KN/0067	KN/0067/S/5	Express Diagnostic Laboratories Ltd.	Laboratory	Gandun Albasa, Off Zoo Road, Kano	Kano Municipal	Kano
3481	KN/0070	KN/0070/S/1	Barewa Clinic & Maternity		11, Zaria Road, Kano.	Kumbotso	Kano
3482	KN/0070	KN/0070/S/14	Barewa Clinic & Maternity		11, Zaria Road, Kano.	Kumbotso	Kano

3483	KN/0070	KN/0070/S/6	Barewa Clinic & Maternity		11, Zaria Road, Kano.	Kumbotso	Kano
3484	KN/0070	KN/0070/S/2	Barewa Clinic & Maternity		11, Zaria Road, Kano.	Kumbotso	Kano
3485	KN/0070	KN/0070/S/3	Barewa Clinic & Maternity		11, Zaria Road, Kano.	Kumbotso	Kano
3486	KN/0070	KN/0070/S/5	Barewa Clinic & Maternity		11, Zaria Road, Kano.	Kumbotso	Kano
3487	KN/0075	KN/0075/S/15	General Hospital, Gwarzo		P.O. BOX 10911 Kano,	Gwarzo	Kano
3488	KN/0075	KN/0075/S/2	General Hospital, Gwarzo		P.O. BOX 10911 Kano,	Gwarzo	Kano
3489	KN/0075	KN/0075/S/1	General Hospital, Gwarzo		P.O. BOX 10911 Kano,	Gwarzo	Kano
3490	KN/0076	KN/0076/S/15	Murtala Mohammed Spec. Hospital		Kano City, Kano State	Kano Municipal	Kano
3491	KN/0076	KN/0076/S/8	Murtala Mohammed Spec. Hospital		Kano City, Kano State	Kano Municipal	Kano
3492	KN/0076	KN/0076/S/4	Murtala Mohammed Spec. Hospital		Kano City, Kano State	Kano Municipal	Kano
3493	KN/0076	KN/0076/S/5	Murtala Mohammed Spec. Hospital		Kano City, Kano State	Kano Municipal	Kano
3494	KN/0076	KN/0076/S/7	Murtala Mohammed Spec. Hospital		Kano City, Kano State	Kano Municipal	Kano
3495	KN/0077	KN/0077/S/2	General Hospital, Wudil		Wudil, Kano, Kano State	Wudil	Kano
3496	KN/0077	KN/0077/S/3	General Hospital, Wudil		Wudil, Kano, Kano State	Wudil	Kano
3497	KN/0077	KN/0077/S/1	General Hospital, Wudil		Wudil, Kano, Kano State	Wudil	Kano
3498	KN/0077	KN/0077/S/4	General Hospital, Wudil		Wudil, Kano, Kano State	Wudil	Kano
3499	KN/0077	KN/0077/S/5	General Hospital, Wudil		Wudil, Kano, Kano State	Wudil	Kano
3500	KN/0078	KN/0078/S/7	Mohammed Abdullahi Wase Spec. Hospital		Nasarawa, Kano, Kano State	Nasarawa	Kano
3501	KN/0078	KN/0078/S/5	Mohammed Abdullahi Wase Spec. Hospital		Nasarawa, Kano, Kano State	Nasarawa	Kano
3502	KN/0078	KN/0078/S/12	Mohammed Abdullahi Wase Spec. Hospital		Nasarawa, Kano, Kano State	Nasarawa	Kano
3503	KN/0078	KN/0078/S/6	Mohammed Abdullahi Wase Spec. Hospital		Nasarawa, Kano, Kano State	Nasarawa	Kano
3504	KN/0078	KN/0078/S/10	Mohammed Abdullahi Wase Spec. Hospital		Nasarawa, Kano, Kano State	Nasarawa	Kano
3505	KN/0078	KN/0078/S/2	Mohammed Abdullahi Wase Spec. Hospital		Nasarawa, Kano, Kano State	Nasarawa	Kano
3506	KN/0078	KN/0078/S/1	Mohammed Abdullahi Wase Spec. Hospital		Nasarawa, Kano, Kano State	Nasarawa	Kano
3507	KN/0078	KN/0078/S/15	Mohammed Abdullahi Wase Spec. Hospital		Nasarawa, Kano, Kano State	Nasarawa	Kano
3508	KN/0078	KN/0078/S/4	Mohammed Abdullahi Wase Spec. Hospital		Nasarawa, Kano, Kano State	Nasarawa	Kano
3509	KN/0079	KN/0079/S/1	General Hospital, Danbatta		Danbata, Kano, Kano State	Danbata	Kano
3510	KN/0079	KN/0079/S/4	General Hospital, Danbatta		Danbata, Kano, Kano State	Danbata	Kano
3511	KN/0079	KN/0079/S/2	General Hospital, Danbatta		Danbata, Kano, Kano State	Danbata	Kano
3512	KN/0079	KN/0079/S/5	General Hospital, Danbatta		Danbata, Kano, Kano State	Danbata	Kano
3513	KN/0081	KN/0081/S/2	Sheikh Mohammed Jida Gen. Hospital		Sabon Gari, Kano, Kano State	Fagge	Kano
3514	KN/0081	KN/0081/S/15	Sheikh Mohammed Jida Gen. Hospital		Sabon Gari, Kano, Kano State	Fagge	Kano
3515	KN/0081	KN/0081/S/6	Sheikh Mohammed Jida Gen. Hospital		Sabon Gari, Kano, Kano State	Fagge	Kano
3516	KN/0082	KN/0082/S/6	Aminu Kano Teaching Hospital		Gyadi Gyadi, Kano, Kano State	Tarauni	Kano
3517	KN/0082	KN/0082/S/9	Aminu Kano Teaching Hospital		Gyadi Gyadi, Kano, Kano State	Tarauni	Kano
3518	KN/0082	KN/0082/S/1	Aminu Kano Teaching Hospital		Gyadi Gyadi, Kano, Kano State	Tarauni	Kano
3519	KN/0082	KN/0082/S/12	Aminu Kano Teaching Hospital		Gyadi Gyadi, Kano, Kano State	Tarauni	Kano
3520	KN/0082	KN/0082/S/2	Aminu Kano Teaching Hospital		Gyadi Gyadi, Kano, Kano State	Tarauni	Kano
3521	KN/0082	KN/0082/S/10	Aminu Kano Teaching Hospital		Gyadi Gyadi, Kano, Kano State	Tarauni	Kano
3522	KN/0082	KN/0082/S/5	Aminu Kano Teaching Hospital		Gyadi Gyadi, Kano, Kano State	Tarauni	Kano

3523	KN/0082	KN/0082/S/8	Aminu Kano Teaching Hospital		Gyadi Gyadi, Kano, Kano State	Tarauni	Kano
3524	KN/0082	KN/0082/S/4	Aminu Kano Teaching Hospital		Gyadi Gyadi, Kano, Kano State	Tarauni	Kano
3525	KN/0082	KN/0082/S/7	Aminu Kano Teaching Hospital		Gyadi Gyadi, Kano, Kano State	Tarauni	Kano
3526	KN/0086	KN/0086/S/5	Bayero University Health Centre		Kano, Kano State	Ungogo	Kano
3527	KN/0086	KN/0086/S/4	Bayero University Health Centre		Kano, Kano State	Ungogo	Kano
3528	KN/0091	KN/0091/S/7	National Orthopaedic Hospital, Kano		Kano, Kano	Dala	Kano
3529	KN/0091	KN/0091/S/10	National Orthopaedic Hospital, Kano		Kano, Kano	Dala	Kano
3530	KN/0091	KN/0091/S/11	National Orthopaedic Hospital, Kano		Kano, Kano	Dala	Kano
3531	KN/0091	KN/0091/S/4	National Orthopaedic Hospital, Kano		Kano, Kano	Dala	Kano
3532	KN/0091	KN/0091/S/3	National Orthopaedic Hospital, Kano		Kano, Kano	Dala	Kano
3533	KN/0095	KN/0095/S/3	Standard Special Hospital	Primary Provider	8A Lamido Crescent Nassarawa Kano	Nasarawa	Kano
3534	KN/0095	KN/0095/S/6	Standard Special Hospital	Primary Provider	8A Lamido Crescent Nassarawa Kano	Nasarawa	Kano
3535	KN/0096	KN/0096/S/14	Cordial Hospital & Maternity	Primary Provider	68 Sarkin Yaki Nomansland Kano	Fagge	Kano
3536	KN/0096	KN/0096/S/5	Cordial Hospital & Maternity	Primary Provider	68 Sarkin Yaki Nomansland Kano	Fagge	Kano
3537	KN/0104	KN/0104/S/4	Zeenat Pharmacy & Stores	Pharmacy	C4 Farm Centre, Opp Marhaba Cinema Kano	Tarauni	Kano
3538	KN/0108	KN/0108/S/5	Good Pasture Clinic		6, Citta Avenue, Noman''''''s Land, Kano	Fagge	Kano
3539	KN/0108	KN/0108/S/7	Good Pasture Clinic		6, Citta Avenue, Noman''''''s Land, Kano	Fagge	Kano
3540	KN/0109	KN/0109/S/5	Lafiya Surgery		52, Ogoja Street, Sabon Gari, Kano	Fagge	Kano
3541	KN/0109	KN/0109/S/3	Lafiya Surgery		52, Ogoja Street, Sabon Gari, Kano	Fagge	Kano
3542	KN/0109	KN/0109/S/1	Lafiya Surgery		52, Ogoja Street, Sabon Gari, Kano	Fagge	Kano
3543	KN/0109	KN/0109/S/14	Lafiya Surgery		52, Ogoja Street, Sabon Gari, Kano	Fagge	Kano
3544	KN/0109	KN/0109/S/7	Lafiya Surgery		52, Ogoja Street, Sabon Gari, Kano	Fagge	Kano
3545	KN/0115	KN/0115/S/3	Al Noury Specialist Hospital		No 1 Iyaka Road off Zaria Road, Kano	Tarauni	Kano
3546	KN/0120	KN/0120/S/4	Nigerian Law School Medical Centre		Tiga Road, Off Zaria - Kano Express Road, Kano	Bebeji	Kano
3547	KN/0123	KN/0123/S/4	ALCON Pharmacy	Pharmacy	No. 11A Bompai Road, Kano	Nasarawa	Kano
3548	KN/0124	KN/0124/S/4	Al-Huda Pharm. Stores	Pharmacy	125 Bello Road, Kano	Fagge	Kano
3549	KN/0125	KN/0125/S/3	Alnoury Specialist Hospital	O&G	No. 1 Iyaka Road, off Zaria Rd, Gyadi Gyadi, Kano State	Tarauni	Kano
3550	KN/0130	KN/0130/S/4	Lumba Pharmacy Ltd	Pharmacy	113 Court Rd, Gyadi-Gyadi, Kano	Tarauni	Kano
3551	KN/0131	KN/0131/S/4	Model Pharmaceutical & Supplies Ltd	Pharmacy	No. 2A Bompai Road, Kano.	Nasarawa	Kano
3552	KN/0133	KN/0133/S/4	Sylmat Pharmacy	Pharmacy	No. 264 Hadejia Road, Kano	Nasarawa	Kano
3553	KN/0134	KN/0134/S/4	Vitamex Pharmaceuticals	Pharmacy	Plot 302, New Hospital Road, Gyadi Gyadi, Kano	Tarauni	Kano
3554	KN/0135	KN/0135/S/3	Pinnacle Specialist Hospital, Kano		Plot 11-12 Hausawa Quarters, Kano	Kumbotso	Kano
3555	KN/0135	KN/0135/S/1	Pinnacle Specialist Hospital, Kano		Plot 11-12 Hausawa Quarters, Kano	Kumbotso	Kano
3556	KN/0135	KN/0135/S/6	Pinnacle Specialist Hospital, Kano		Plot 11-12 Hausawa Quarters, Kano	Kumbotso	Kano
3557	KN/0135	KN/0135/S/2	Pinnacle Specialist Hospital, Kano		Plot 11-12 Hausawa Quarters, Kano	Kumbotso	Kano
3558	KN/0139	KN/0139/S/4	Carey-gold Pharmacy	Pharmacy	Tarauni Market Road, Off Sabo Bakin Zuwo Road, Kano	Tarauni	Kano
3559	KN/0141	KN/0141/S/4	ECWA Hospital	Pharmacy, Opthamology, Laboratory	Off Airport Road, Kano	Aba North	Abia
3560	KN/0141	KN/0141/S/15	ECWA Hospital	Pharmacy, Opthamology, Laboratory	Off Airport Road, Kano	Aba North	Abia
3561	KN/0146	KN/0146/S/5	Goldstone Diagnostic Services Nig. Ltd	Laboratory	Sabo Gari	Abakaliki	Ebonyi

3562	KN/0149	KN/0149/S/2	Amity Hospital & Medical Services Ltd		10, 13 Link off court Rd, Hausawa, Kano State	Tarauni	Kano
3563	KN/0149	KN/0149/S/1	Amity Hospital & Medical Services Ltd		10, 13 Link off court Rd, Hausawa, Kano State	Tarauni	Kano
3564	KN/0149	KN/0149/S/3	Amity Hospital & Medical Services Ltd		10, 13 Link off court Rd, Hausawa, Kano State	Tarauni	Kano
3565	KN/0149	KN/0149/S/6	Amity Hospital & Medical Services Ltd		10, 13 Link off court Rd, Hausawa, Kano State	Tarauni	Kano
3566	KN/0151	KN/0151/S/10	Fazma Orthopedic Hospital		181, Airport Road, Kano	Nasarawa	Kano
3567	KN/0154	KN/0154/S/6	Wisdom Hospital		2, Abdullahi Bayero Street, Beside Nasarawa Hosp, Kano State	Nasarawa	Kano
3568	KN/0154	KN/0154/S/1	Wisdom Hospital		2, Abdullahi Bayero Street, Beside Nasarawa Hosp, Kano State	Nasarawa	Kano
3569	KN/0155	KN/0155/S/1	NOL International Hospital and Diagnosis Centre Limited		1A Sultan Road Nassarawa GRA, Kano	Tarauni	Kano
3570	KN/0155	KN/0155/S/2	NOL International Hospital and Diagnosis Centre Limited		1A Sultan Road Nassarawa GRA, Kano	Tarauni	Kano
3571	KN/0155	KN/0155/S/3	NOL International Hospital and Diagnosis Centre Limited		1A Sultan Road Nassarawa GRA, Kano	Tarauni	Kano
3572	KN/0155	KN/0155/S/5	NOL International Hospital and Diagnosis Centre Limited		1A Sultan Road Nassarawa GRA, Kano	Tarauni	Kano
3573	KN/0155	KN/0155/S/6	NOL International Hospital and Diagnosis Centre Limited		1A Sultan Road Nassarawa GRA, Kano	Tarauni	Kano
3574	KN/0156	KN/0156/S/2	Al Farma Ultra Modern Hospital and Diagnostic Centre		7A Katsina Road Opp. Federal Secretariat, Kano	Fagge	Kano
3575	KN/0156	KN/0156/S/3	Al Farma Ultra Modern Hospital and Diagnostic Centre		7A Katsina Road Opp. Federal Secretariat, Kano	Fagge	Kano
3576	KN/0156	KN/0156/S/7	Al Farma Ultra Modern Hospital and Diagnostic Centre		7A Katsina Road Opp. Federal Secretariat, Kano	Fagge	Kano
3577	KN/0156	KN/0156/S/8	Al Farma Ultra Modern Hospital and Diagnostic Centre		7A Katsina Road Opp. Federal Secretariat, Kano	Fagge	Kano
3578	KN/0156	KN/0156/S/1	Al Farma Ultra Modern Hospital and Diagnostic Centre		7A Katsina Road Opp. Federal Secretariat, Kano	Fagge	Kano
3579	KN/0161	KN/0161/S/4	Abubakar Imam Urology Centre		144, Murtala Mohammed Way, Opp Triumph Newspaper, Akija, Kano, Kano State.	Fagge	Kano
3580	KN/0161	KN/0161/S/18	Abubakar Imam Urology Centre		144, Murtala Mohammed Way, Opp Triumph Newspaper, Akija, Kano, Kano State.	Fagge	Kano
3581	KN/0166	KN/0166/S/5	Waziri Shehu Gidado General Hospital		Bachirawa Katsina Road, Kano State	Fagge	Kano
3582	KN/0166	KN/0166/S/4	Waziri Shehu Gidado General Hospital		Bachirawa Katsina Road, Kano State	Fagge	Kano
3583	KN/0167	KN/0167/S/10	Albarka Clinic		Kofar/Dawanau Dandiwshe Kano, Kano State.	Dala	Kano
3584	KN/0168	KN/0168/S/5	Klinchex Laboratory & Diagnostics		No. 3 Opp. B.U.K Old Site Kano, Kano State	Gwale	Kano
3585	KN/0169	KN/0169/S/15	Maryam Eye Centre		Sabuwa Abuja Off. Aforestation Road Off. Gwarzo Road Kabuga, Kano, Kano State.	Gwale	Kano
3586	KN/0170	KN/0170/S/3	Sumar Specialist Clinics		Airport Road, Behind Kabo Holdings By Kriss Nursery & Primary School, Kano, Kano State.	Nasarawa	Kano
3587	KN/0171	KN/0171/S/3	Greenland Clinics And Maternity Ltd		No. 49a Sarki Yaki Street Nomansland Kano State.	Nasarawa	Kano
3588	KN/0172	KN/0172/S/1	Prime Specialist Hospital		19 Lamodo Crescent Kano, Kano State	Nasarawa	Kano
3589	KN/0174	KN/0174/S/11	Sauki Gym & Physiotherapy Clinic		No. 16 Zaria Road, Kano, Kano State	Tarauni	Kano

3590	KN/0175	KN/0175/S/11	Sauki Gym & Physiotherapy Clinic		No. 16 Zaria Road, Kano, Kano State.	Tarauni	Kano
3591	KN/0176	KN/0176/S/12	Mai Akoko Clinic		No. 96 Naibawa Yanlemo, Kano, Kano State	Kumbotso	Kano
3592	KN/0177	KN/0177/S/1	Oasis Clinic Maternity		Ungwu Uku U- Turn Behind Matrix Filling Station, Opposite Zenith Bank, Kano, Kano State.	Kumbotso	Kano
3593	KN/0177	KN/0177/S/5	Oasis Clinic Maternity		Ungwu Uku U- Turn Behind Matrix Filling Station, Opposite Zenith Bank, Kano, Kano State.	Kumbotso	Kano
3594	KN/0177	KN/0177/S/3	Oasis Clinic Maternity		Ungwu Uku U- Turn Behind Matrix Filling Station, Opposite Zenith Bank, Kano, Kano State.	Kumbotso	Kano
3595	KN/0178	KN/0178/S/3	City Clinic & Maternity		No. 149 Daurawa Quarters, Maiduguri Road, Kano, Kano State.	Tarauni	Kano
3596	KN/0178	KN/0178/S/6	City Clinic & Maternity		No. 149 Daurawa Quarters, Maiduguri Road, Kano, Kano State.	Tarauni	Kano
3597	KN/0178	KN/0178/S/5	City Clinic & Maternity		No. 149 Daurawa Quarters, Maiduguri Road, Kano, Kano State.	Tarauni	Kano
3598	KN/0179	KN/0179/S/12	Multicare ENT		No. 66 Lamido Crescent, Nasarawa G.R.A Kano, Kano State.	Nasarawa	Kano
3599	KN/0181	KN/0181/S/5	Northwest University Teaching Hospital		Off Zaria Road Kano	Gwale	Kano
3600	KN/0181	KN/0181/S/2	Northwest University Teaching Hospital		Off Zaria Road Kano	Gwale	Kano
3601	KN/0181	KN/0181/S/4	Northwest University Teaching Hospital		Off Zaria Road Kano	Gwale	Kano
3602	KN/0182	KN/0182/S/15	Al-Ihsan Expert Medical and Diagnostic Centre		312,Tudu Fulani, Darmanawa Qtrs. Kano	Kumbotso	Kano
3603	KN/0182	KN/0182/S/1	Al-Ihsan Expert Medical and Diagnostic Centre		312,Tudu Fulani, Darmanawa Qtrs. Kano	Kumbotso	Kano
3604	KT/0001	KT/0001/S/14	Bakori Clinic & Maternity		1, Raba Road, Bakori, behind Sharia Court, Bakori, Katsina State	Bakori	Katsina
3605	KT/0005	KT/0005/S/8	General Hospital, Daura		Daura	Daura	Katsina
3606	KT/0005	KT/0005/S/7	General Hospital, Daura		Daura	Daura	Katsina
3607	KT/0005	KT/0005/S/5	General Hospital, Daura		Daura	Daura	Katsina
3608	KT/0005	KT/0005/S/4	General Hospital, Daura		Daura	Daura	Katsina
3609	KT/0009	KT/0009/S/15	General Hospital, Dutsinma		Dutsinma	Dutsin-Ma	Katsina
3610	KT/0009	KT/0009/S/5	General Hospital, Dutsinma		Dutsinma	Dutsin-Ma	Katsina
3611	KT/0009	KT/0009/S/8	General Hospital, Dutsinma		Dutsinma	Dutsin-Ma	Katsina
3612	KT/0009	KT/0009/S/4	General Hospital, Dutsinma		Dutsinma	Dutsin-Ma	Katsina
3613	KT/0012	KT/0012/S/15	General Hospital Funtua		Funtua	Funtua	Katsina
3614	KT/0012	KT/0012/S/14	General Hospital Funtua		Funtua	Funtua	Katsina
3615	KT/0012	KT/0012/S/1	General Hospital Funtua		Funtua	Funtua	Katsina
3616	KT/0012	KT/0012/S/3	General Hospital Funtua		Funtua	Funtua	Katsina
3617	KT/0012	KT/0012/S/4	General Hospital Funtua		Funtua	Funtua	Katsina
3618	KT/0012	KT/0012/S/7	General Hospital Funtua		Funtua	Funtua	Katsina
3619	KT/0012	KT/0012/S/5	General Hospital Funtua		Funtua	Funtua	Katsina
3620	KT/0012	KT/0012/S/8	General Hospital Funtua		Funtua	Funtua	Katsina
3621	KT/0013	KT/0013/S/1	Khadijat Medical Center		No. 53 Katsina Road Funtua	Funtua	Katsina
3622	KT/0013	KT/0013/S/3	Khadijat Medical Center		No. 53 Katsina Road Funtua	Funtua	Katsina
3623	KT/0013	KT/0013/S/4	Khadijat Medical Center		No. 53 Katsina Road Funtua	Funtua	Katsina
3624	KT/0014	KT/0014/S/3	Bistit Hospital		No. 36 Katsina Road	Funtua	Katsina
3625	KT/0014	KT/0014/S/5	Bistit Hospital		No. 36 Katsina Road	Funtua	Katsina
3626	KT/0016	KT/0016/S/5	Nakowa Hospital & Ultra Sound Center		Sokoto Road, Funtua	Funtua	Katsina

3627	KT/0018	KT/0018/S/4	Khadija Medi Pharm. Co. Ltd	Pharmacy	53 Katsina Road, Funtua	Funtua	Katsina
3628	KT/0020	KT/0020/S/4	General Hospital, Kankiya		Kankiya	Kankia	Katsina
3629	KT/0020	KT/0020/S/5	General Hospital, Kankiya		Kankiya	Kankia	Katsina
3630	KT/0020	KT/0020/S/7	General Hospital, Kankiya		Kankiya	Kankia	Katsina
3631	KT/0020	KT/0020/S/8	General Hospital, Kankiya		Kankiya	Kankia	Katsina
3632	KT/0021	KT/0021/S/1	Federal Medical Center, Katsina		Murtala Mohammed Way	Katsina	Katsina
3633	KT/0021	KT/0021/S/4	Federal Medical Center, Katsina		Murtala Mohammed Way	Katsina	Katsina
3634	KT/0021	KT/0021/S/2	Federal Medical Center, Katsina		Murtala Mohammed Way	Katsina	Katsina
3635	KT/0021	KT/0021/S/11	Federal Medical Center, Katsina		Murtala Mohammed Way	Katsina	Katsina
3636	KT/0021	KT/0021/S/7	Federal Medical Center, Katsina		Murtala Mohammed Way	Katsina	Katsina
3637	KT/0021	KT/0021/S/5	Federal Medical Center, Katsina		Murtala Mohammed Way	Katsina	Katsina
3638	KT/0021	KT/0021/S/6	Federal Medical Center, Katsina		Murtala Mohammed Way	Katsina	Katsina
3639	KT/0021	KT/0021/S/15	Federal Medical Center, Katsina		Murtala Mohammed Way	Katsina	Katsina
3640	KT/0021	KT/0021/S/3	Federal Medical Center, Katsina		Murtala Mohammed Way	Katsina	Katsina
3641	KT/0024	KT/0024/S/4	Federal College Of Education Staff Clinic		Katsina	Katsina	Katsina
3642	KT/0030	KT/0030/S/4	Rahusa Pharmacy & Stores	Pharmacy	Behind General Hospital	Kankia	Katsina
3643	KT/0031	KT/0031/S/4	Sulhu Pharmaceutical Chemist	Pharmacy	No.4 Kaduna Street, Kofar Keke	Katsina	Katsina
3644	KT/0036	KT/0036/S/15	General Hospital Mani		Mani-Mashi Road Katsina	Mani	Katsina
3645	KT/0036	KT/0036/S/4	General Hospital Mani		Mani-Mashi Road Katsina	Mani	Katsina
3646	KT/0036	KT/0036/S/8	General Hospital Mani		Mani-Mashi Road Katsina	Mani	Katsina
3647	KT/0036	KT/0036/S/5	General Hospital Mani		Mani-Mashi Road Katsina	Mani	Katsina
3648	KT/0039	KT/0039/S/5	Katsina General Hospital		Katsina, Katsina State	Katsina	Katsina
3649	KT/0039	KT/0039/S/8	Katsina General Hospital		Katsina, Katsina State	Katsina	Katsina
3650	KT/0039	KT/0039/S/4	Katsina General Hospital		Katsina, Katsina State	Katsina	Katsina
3651	KT/0039	KT/0039/S/2	Katsina General Hospital		Katsina, Katsina State	Katsina	Katsina
3652	KT/0039	KT/0039/S/6	Katsina General Hospital		Katsina, Katsina State	Katsina	Katsina
3653	KT/0039	KT/0039/S/1	Katsina General Hospital		Katsina, Katsina State	Katsina	Katsina
3654	KT/0039	KT/0039/S/3	Katsina General Hospital		Katsina, Katsina State	Katsina	Katsina
3655	KT/0039	KT/0039/S/15	Katsina General Hospital		Katsina, Katsina State	Katsina	Katsina
3656	KT/0039	KT/0039/S/7	Katsina General Hospital		Katsina, Katsina State	Katsina	Katsina
3657	KT/0040	KT/0040/S/5	Muslim Community Laboratory	Laboratory	School of Health Technology, Katsina	Katsina	Katsina
3658	KT/0042	KT/0042/S/7	General Hospital, Malufashi		Malufashi, Katsina State	Malufashi	Katsina
3659	KT/0042	KT/0042/S/4	General Hospital, Malufashi		Malufashi, Katsina State	Malufashi	Katsina
3660	KT/0042	KT/0042/S/8	General Hospital, Malufashi		Malufashi, Katsina State	Malufashi	Katsina
3661	KT/0042	KT/0042/S/5	General Hospital, Malufashi		Malufashi, Katsina State	Malufashi	Katsina
3662	KT/0055	KT/0055/S/5	New Funtua Clinic		3 Jabir Roundabout, Zaria Road, Funtua	Funtua	Katsina
3663	KT/0063	KT/0063/S/4	Mubek Pharmacy		federal Medical Centre, Katsina	Katsina	Katsina
3664	KT/0067	KT/0067/S/5	Maternal and Children Hospital		Kafur Road. Malumfashi	Malufashi	Katsina
3665	KT/0067	KT/0067/S/6	Maternal and Children Hospital		Kafur Road. Malumfashi	Malufashi	Katsina
3666	KT/0067	KT/0067/S/7	Maternal and Children Hospital		Kafur Road. Malumfashi	Malufashi	Katsina
3667	KT/0067	KT/0067/S/4	Maternal and Children Hospital		Kafur Road. Malumfashi	Malufashi	Katsina
3668	KT/0067	KT/0067/S/3	Maternal and Children Hospital		Kafur Road. Malumfashi	Malufashi	Katsina
3669	KT/0067	KT/0067/S/1	Maternal and Children Hospital		Kafur Road. Malumfashi	Malufashi	Katsina
3670	KT/0068	KT/0068/S/3	K Dara Specialist Clinic Limited		Gidan Dawa	Katsina	Katsina

3671	KT/0070	KT/0070/S/1	Turai Umar Yar'Adua Maternity Children Hospital		Gidan Dawa off Hassan Usman Katsina Road, Katsina	Katsina	Katsina
3672	KT/0070	KT/0070/S/4	Turai Umar Yar'Adua Maternity Children Hospital		Gidan Dawa off Hassan Usman Katsina Road, Katsina	Katsina	Katsina
3673	KT/0070	KT/0070/S/5	Turai Umar Yar'Adua Maternity Children Hospital		Gidan Dawa off Hassan Usman Katsina Road, Katsina	Katsina	Katsina
3674	KT/0070	KT/0070/S/6	Turai Umar Yar'Adua Maternity Children Hospital		Gidan Dawa off Hassan Usman Katsina Road, Katsina	Katsina	Katsina
3675	KT/0070	KT/0070/S/7	Turai Umar Yar'Adua Maternity Children Hospital		Gidan Dawa off Hassan Usman Katsina Road, Katsina	Katsina	Katsina
3676	KT/0070	KT/0070/S/3	Turai Umar Yar'Adua Maternity Children Hospital		Gidan Dawa off Hassan Usman Katsina Road, Katsina	Katsina	Katsina
3677	KT/0073	KT/0073/S/8	General Hospital Musawa		Hospital Rd, Shaishawu Musawa, Katsina	Musawa	Katsina
3678	KT/0073	KT/0073/S/5	General Hospital Musawa		Hospital Rd, Shaishawu Musawa, Katsina	Musawa	Katsina
3679	KT/0073	KT/0073/S/1	General Hospital Musawa		Hospital Rd, Shaishawu Musawa, Katsina	Musawa	Katsina
3680	KT/0073	KT/0073/S/3	General Hospital Musawa		Hospital Rd, Shaishawu Musawa, Katsina	Musawa	Katsina
3681	KT/0076	KT/0076/S/4	Alheri Clinic		Lawrence Onoja Road, Kofar Kaura, New Layout katsina, Katsina State	Katsina	Katsina
3682	KT/0076	KT/0076/S/2	Alheri Clinic		Lawrence Onoja Road, Kofar Kaura, New Layout katsina, Katsina State	Katsina	Katsina
3683	KT/0076	KT/0076/S/5	Alheri Clinic		Lawrence Onoja Road, Kofar Kaura, New Layout katsina, Katsina State	Katsina	Katsina
3684	KT/0076	KT/0076/S/3	Alheri Clinic		Lawrence Onoja Road, Kofar Kaura, New Layout katsina, Katsina State	Katsina	Katsina
3685	KT/0076	KT/0076/S/1	Alheri Clinic		Lawrence Onoja Road, Kofar Kaura, New Layout katsina, Katsina State	Katsina	Katsina
3686	KT/0082	KT/0082/S/7	Katsina State Orthopaedic & Speciality Hospital		Dutsinma Road, Katsina	Katsina	Katsina
3687	KT/0082	KT/0082/S/10	Katsina State Orthopaedic & Speciality Hospital		Dutsinma Road, Katsina	Katsina	Katsina
3688	KT/0082	KT/0082/S/11	Katsina State Orthopaedic & Speciality Hospital		Dutsinma Road, Katsina	Katsina	Katsina
3689	KT/0082	KT/0082/S/1	Katsina State Orthopaedic & Speciality Hospital		Dutsinma Road, Katsina	Katsina	Katsina
3690	KT/0082	KT/0082/S/5	Katsina State Orthopaedic & Speciality Hospital		Dutsinma Road, Katsina	Katsina	Katsina
3691	KW/0001	KW/0001/S/5	Temitope Hospital		Sabo Line Isale, Off Amilegbe Road, Ilorin	Ilorin East	Kwara
3692	KW/0002	KW/0002/S/4	Ola Olu Hospital		Opp. International Tobacco Coy. Garrage Offa Road, Iiorin	Ilorin East	Kwara
3693	KW/0002	KW/0002/S/1	Ola Olu Hospital		Opp. International Tobacco Coy. Garrage Offa Road, Iiorin	Ilorin East	Kwara
3694	KW/0002	KW/0002/S/5	Ola Olu Hospital		Opp. International Tobacco Coy. Garrage Offa Road, Iiorin	Ilorin East	Kwara
3695	KW/0002	KW/0002/S/7	Ola Olu Hospital		Opp. International Tobacco Coy. Garrage Offa Road, Iiorin	Ilorin East	Kwara
3696	KW/0005	KW/0005/S/3	Eyitayo Hospital		No. 40, Ojo-Iya Road, Ilorin	Ilorin East	Kwara
3697	KW/0006	KW/0006/S/2	Oorelope Hospital		No. 30, Opo-Malu Road, Ilorin, Kwara State	Ilorin East	Kwara
3698	KW/0006	KW/0006/S/4	Oorelope Hospital		No. 30, Opo-Malu Road, Ilorin, Kwara State	Ilorin East	Kwara
3699	KW/0006	KW/0006/S/3	Oorelope Hospital		No. 30, Opo-Malu Road, Ilorin, Kwara State	Ilorin East	Kwara
3700	KW/0008	KW/0008/S/6	Jebba Specialist Hospital	Radiology, Paediatrics, Surgery, Pharmacy	Jebba, Ilorin	Patigi	Kwara
3701	KW/0008	KW/0008/S/4	Jebba Specialist Hospital	Radiology, Paediatrics, Surgery, Pharmacy	Jebba, Ilorin	Patigi	Kwara
3702	KW/0012	KW/0012/S/3	Olalomi Hospital - Ilorin		Stadium Road, Ilorin	Ilorin-West	Kwara
3703	KW/0012	KW/0012/S/7	Olalomi Hospital - Ilorin		Stadium Road, Ilorin	Ilorin-West	Kwara

3704	KW/0012	KW/0012/S/1	Olalomi Hospital - Ilorin		Stadium Road, Ilorin	Ilorin-West	Kwara
3705	KW/0015	KW/0015/S/5	Omolola Hospital		Opp. Govt. Sec. School, Along Jebba Road, Ilorin.	Ilorin East	Kwara
3706	KW/0015	KW/0015/S/7	Omolola Hospital		Opp. Govt. Sec. School, Along Jebba Road, Ilorin.	Ilorin East	Kwara
3707	KW/0016	KW/0016/S/5	Omolola Hospital	Laboratory, Radiology	No. 26 Murtala Moh'd Road, Ilorin.	Ilorin East	Kwara
3708	KW/0016	KW/0016/S/7	Omolola Hospital	Laboratory, Radiology	No. 26 Murtala Moh'd Road, Ilorin.	Ilorin East	Kwara
3709	KW/0023	KW/0023/S/4	Sabo Specialist Hospital	Radiology, Laboratory, Pharmacy	Alagbado Okelele, llorin	Patigi	Kwara
3710	KW/0023	KW/0023/S/7	Sabo Specialist Hospital	Radiology, Laboratory, Pharmacy	Alagbado Okelele, llorin	Patigi	Kwara
3711	KW/0023	KW/0023/S/5	Sabo Specialist Hospital	Radiology, Laboratory, Pharmacy	Alagbado Okelele, llorin	Patigi	Kwara
3712	KW/0024	KW/0024/S/1	Olarewaju Hospital		Oro Ago Close Ilorin	Ilorin-South	Kwara
3713	KW/0024	KW/0024/S/3	Olarewaju Hospital		Oro Ago Close Ilorin	Ilorin-South	Kwara
3714	KW/0024	KW/0024/S/5	Olarewaju Hospital		Oro Ago Close Ilorin	Ilorin-South	Kwara
3715	KW/0026	KW/0026/S/4	Offa Specialist Hospital	Radiology, Pharmacy, Laboratory, Surgery	Offa	Patigi	Kwara
3716	KW/0026	KW/0026/S/1	Offa Specialist Hospital	Radiology, Pharmacy, Laboratory, Surgery	Offa	Patigi	Kwara
3717	KW/0026	KW/0026/S/7	Offa Specialist Hospital	Radiology, Pharmacy, Laboratory, Surgery	Offa	Patigi	Kwara
3718	KW/0026	KW/0026/S/5	Offa Specialist Hospital	Radiology, Pharmacy, Laboratory, Surgery	Offa	Patigi	Kwara
3719	KW/0027	KW/0027/S/12	University Teaching Hospital Ilorin		Ilorin, Lagos Road, Ilorin	Ilorin East	Kwara
3720	KW/0027	KW/0027/S/4	University Teaching Hospital Ilorin		Ilorin, Lagos Road, Ilorin	Ilorin East	Kwara
3721	KW/0027	KW/0027/S/6	University Teaching Hospital Ilorin		Ilorin, Lagos Road, Ilorin	Ilorin East	Kwara
3722	KW/0027	KW/0027/S/15	University Teaching Hospital Ilorin		Ilorin, Lagos Road, Ilorin	Ilorin East	Kwara
3723	KW/0027	KW/0027/S/7	University Teaching Hospital Ilorin		Ilorin, Lagos Road, Ilorin	Ilorin East	Kwara
3724	KW/0027	KW/0027/S/11	University Teaching Hospital Ilorin		Ilorin, Lagos Road, Ilorin	Ilorin East	Kwara
3725	KW/0027	KW/0027/S/5	University Teaching Hospital Ilorin		Ilorin, Lagos Road, Ilorin	Ilorin East	Kwara
3726	KW/0027	KW/0027/S/10	University Teaching Hospital Ilorin		Ilorin, Lagos Road, Ilorin	Ilorin East	Kwara
3727	KW/0027	KW/0027/S/3	University Teaching Hospital Ilorin		Ilorin, Lagos Road, Ilorin	Ilorin East	Kwara
3728	KW/0027	KW/0027/S/2	University Teaching Hospital Ilorin		Ilorin, Lagos Road, Ilorin	Ilorin East	Kwara
3729	KW/0027	KW/0027/S/1	University Teaching Hospital Ilorin		Ilorin, Lagos Road, Ilorin	Ilorin East	Kwara
3730	KW/0029	KW/0029/S/3	Oyin Folorunsho		Tanke Bubu Off University Road, Iiorin	Ilorin East	Kwara
3731	KW/0031	KW/0031/S/1	Aisat Memorial Hospital		Off. Jaigbade Residence, Itamerin, Ilorin	Ilorin East	Kwara
3732	KW/0032	KW/0032/S/7	General Hospital, Omuaran		Omuaran	Irepodun	Kwara
3733	KW/0032	KW/0032/S/4	General Hospital, Omuaran		Omuaran	Irepodun	Kwara
3734	KW/0038	KW/0038/S/1	Kiddiz Clinic		Eastern Reservoirs, Olorunshogo, Ilorin	Ilorin East	Kwara
3735	KW/0038	KW/0038/S/6	Kiddiz Clinic		Eastern Reservoirs, Olorunshogo, Ilorin	Ilorin East	Kwara
3736	KW/0039	KW/0039/S/3	Sadiku Hospital		16, Abdulwahab Folawuyo, Unity Road, Ilorin	Ilorin-South	Kwara
3737	KW/0039	KW/0039/S/1	Sadiku Hospital		16, Abdulwahab Folawuyo, Unity Road, Ilorin	Ilorin-South	Kwara
3738	KW/0049	KW/0049/S/11	Ela Memorial Medical Centre		Near Basin Gate, Ilorin	Ilorin-South	Kwara
3739	KW/0051	KW/0051/S/3	Surulere Medical Centre		Off Taiwo Road, Ilorin	Ilorin East	Kwara
3740	KW/0052	KW/0052/S/3	Kosemani Hospital		Emirs Road, Ilorin	Ilorin East	Kwara
3741	KW/0052	KW/0052/S/14	Kosemani Hospital		Emirs Road, Ilorin	Ilorin East	Kwara
3742	KW/0060	KW/0060/S/4	Dave Mercy Pharmacy	Pharmacy	Jebba, Idiagbon Nigeria Paper Road, Adjacent Hotel, Jebba.	Moro	Kwara
3743	KW/0061	KW/0061/S/3	Olarewaju Hospital Lab	Laboratory, Surgery, O & G.	Oro Ago Close, Ilorin	Ilorin East	Kwara

3744	KW/0061	KW/0061/S/1	Olarewaju Hospital Lab	Laboratory, Surgery, O & G.	Oro Ago Close, Ilorin	Ilorin East	Kwara
3745	KW/0061	KW/0061/S/5	Olarewaju Hospital Lab	Laboratory, Surgery, O & G.	Oro Ago Close, Ilorin	Ilorin East	Kwara
3746	KW/0063	KW/0063/S	Ola-Olu Hospital	Laboratory, Radiology, Pharmacy	Opposite Int. Tobacco Coy, Garrage Offa Road, Ilorin	Ilorin-South	Kwara
3747	KW/0064	KW/0064/S/7	Akande X-ray Diagnostic	Radiology	No.4, Sultan Road, Ilorin	Ilorin-South	Kwara
3748	KW/0065	KW/0065/S/8	Heritage Dental Clinic	Dental	16, Ibrahim Taiwo Road, Ilorin	Ilorin East	Kwara
3749	KW/0067	KW/0067/S/1	Olalomi Hospital	Radiology, Surgery, O&G	Old Yidi Road, Behind Ansarudeen Primary Sch., Ilorin	Ilorin-West	Kwara
3750	KW/0067	KW/0067/S/3	Olalomi Hospital	Radiology, Surgery, O&G	Old Yidi Road, Behind Ansarudeen Primary Sch., Ilorin	Ilorin-West	Kwara
3751	KW/0067	KW/0067/S/7	Olalomi Hospital	Radiology, Surgery, O&G	Old Yidi Road, Behind Ansarudeen Primary Sch., Ilorin	Ilorin-West	Kwara
3752	KW/0069	KW/0069/S/8	Alpha Dental Clinic	Dental	116, Abdulazeez Atta Road, llorin	Ilorin-West	Kwara
3753	KW/0073	KW/0073/S/4	Mosaaj Pharmacy	Pharmacy	Behind Queen School, Off CUTH, llorin	Ilorin-West	Kwara
3754	KW/0074	KW/0074/S/4	Coby Pharmacy	Pharmacy	6, NNPC Pipeline Road, Tanke-GRA, llorin	Patigi	Kwara
3755	KW/0075	KW/0075/S/4	Fiolu Pharm. Ltd.	Pharmacy	G. 127, Isale Gambari Road, llorin	Ilorin East	Kwara
3756	KW/0076	KW/0076/S/4	Momrota Pharmacy	Pharmacy	116/117, Abdulazeez Atta Road, Surulere, Adjacent UITH.	Ilorin-West	Kwara
3757	KW/0082	KW/0082/S/4	Folajaiye Diagnostic Medical Lab.	Laboratory	Services, 120 Ibrahim Taiwo Road, llorin	Patigi	Kwara
3758	KW/0083	KW/0083/S/4	Ajeigbe Diagnostic Services Centre	Laboratory	Opp. Specialist Hospital, Offa	Offa	Kwara
3759	KW/0091	KW/0091/S/12	Garin Alimi Hospital		283, Umaru Saro Road, Ilorin	Ilorin-South	Kwara
3760	KW/0091	KW/0091/S/4	Garin Alimi Hospital		283, Umaru Saro Road, Ilorin	Ilorin-South	Kwara
3761	KW/0091	KW/0091/S/5	Garin Alimi Hospital		283, Umaru Saro Road, Ilorin	Ilorin-South	Kwara
3762	KW/0091	KW/0091/S/15	Garin Alimi Hospital		283, Umaru Saro Road, Ilorin	Ilorin-South	Kwara
3763	KW/0091	KW/0091/S/7	Garin Alimi Hospital		283, Umaru Saro Road, Ilorin	Ilorin-South	Kwara
3764	KW/0091	KW/0091/S/3	Garin Alimi Hospital		283, Umaru Saro Road, Ilorin	Ilorin-South	Kwara
3765	KW/0094	KW/0094/S/5	University of Ilorin Health Centre		Ilorin, Kwara State	Ilorin-South	Kwara
3766	KW/0094	KW/0094/S/4	University of Ilorin Health Centre		Ilorin, Kwara State	Ilorin-South	Kwara
3767	KW/0101	KW/0101/S/4	Sarfam Medical Centre		No 34 Fate Rd, Ilorin	Ilorin East	Kwara
3768	KW/0111	KW/0111/S/3	Royal Care Hospital		No 1 Ponyan Rd NNPC Pipeline Gaa-Akanbi Ilorin	Ilorin-South	Kwara
3769	KW/0113	KW/0113/S/7	General Hospital Lafiagi		General Hospital Lafiagi	Edu	Kwara
3770	KW/0114	KW/0114/S/5	General Hospital Kiama		General Hospital Kiama	Kaiama	Kwara
3771	KW/0114	KW/0114/S/1	General Hospital Kiama		General Hospital Kiama	Kaiama	Kwara
3772	KW/0117	KW/0117/S/4	Odeme Pharmacy	Pharmacy	Ahmadu Bello Way Opp Kwara Hotel Ilorin	Ilorin-South	Kwara
3773	KW/0118	KW/0118/S	Gbolafunmi Pharmaceutical Chemist	Pharmacy	Amilegbu Rd Ilorin	Ilorin East	Kwara
3774	KW/0122	KW/0122/S/4	Ilera Pharmaceutical Chemist Nig Ltd	Pharmacy	113 abdullazees Rd Ilorin	Ilorin-West	Kwara
3775	KW/0125	KW/0125/S/11	Ela Memorial Centre	orthopaedic Surgery & Physiotheraphy	Shittu Olufoye Street, Off Lower Niger River Basin Gate Basin Rd Ilorin	Ilorin East	Kwara
3776	KW/0126	KW/0126/S/11	Oleb Physiotherapy	Physiotheraphy	No 19 Unity Rd Ilorin	Ilorin-South	Kwara
3777	KW/0129	KW/0129/S/5	Unity Medical Laboratory Services	Laboratory	120 B Sheikh Adam Abdullahi Street Ipata Oloje Ilorin	Ilorin-West	Kwara
3778	KW/0130	KW/0130/S/16	Femis Laboratory	Laboratory, ECG & Uss	No 4 Unity Rd Ilorin	Ilorin-South	Kwara
3779	KW/0130	KW/0130/S/5	Femis Laboratory	Laboratory, ECG & Uss	No 4 Unity Rd Ilorin	Ilorin-South	Kwara
3780	KW/0130	KW/0130/S/14	Femis Laboratory	Laboratory, ECG & Uss	No 4 Unity Rd Ilorin	Ilorin-South	Kwara
3781	KW/0138	KW/0138/S/5	CIVIL SERVICE HOSPITAL		ILOFA ROAD, ILORIN	Ilorin-South	Kwara
3782	KW/0139	KW/0139/S/5	General Hospital, Share		Share	Irepodun	Kwara
3783	KW/0140	KW/0140/S/4	UITH COMPREHENSIVE HEALTH CENTRE		ESIE	Irepodun	Kwara

3784	KW/0144	KW/0144/S/5	OLUTAYO CLINIC & MATERNITY		SANGO, ILORIN	Ilorin East	Kwara
3785	KW/0146	KW/0146/S/9	OMOSEBI HOSPITAL		GAA AKAMBI ILORIN	Ilorin-South	Kwara
3786	KW/0147	KW/0147/S/6	CHILDREN SPECIALIST HOSP.		CENTRE GBORO	Ilorin-West	Kwara
3787	KW/0147	KW/0147/S/5	CHILDREN SPECIALIST HOSP.		CENTRE GBORO	Ilorin-West	Kwara
3788	KW/0151	KW/0151/S/5	TEMITOPE HOSPITAL	Laboratory	3B, AMILEGBO SABO LINE, ILORIN	Ilorin East	Kwara
3789	KW/0154	KW/0154/S/15	AYO BELLO MEM. EYE CENTRE	Opthalmology	1 AYO BELLO STR, BEHIND WINNER CHAPEL ILORIN	Ilorin-West	Kwara
3790	KW/0155	KW/0155/S/5	COMPREHENSIVE HEALTH CENTRE	Laboratory	IREPODUN	Surulere	Lagos
3791	KW/0156	KW/0156/S/5	Anchormed Hospital	Laboratory	195 AJASSE IPO RD,	Ilorin-West	Kwara
3792	KW/0159	KW/0159/S/8	GOVERNMENT DENTAL CENTRE	DENTAL	UITH PREMISES	Ilorin-West	Kwara
3793	KW/0165	KW/0165/S/5	Dayspring Medical Centre	Laboratory	Beside Agape, Unilorin Remedial Centre, Ilorin	Ilorin-South	Kwara
3794	KW/0167	KW/0167/S/4	Ayobami Pharmacy Co	Pharmacy	Basin Road, Ilorin	Ilorin-South	Kwara
3795	KW/0172	KW/0172/S/7	Albarka Radiodiagnostic Centre		Off Ajase Epo Road, Ilorin	Ilorin East	Kwara
3796	KW/0176	KW/0176/S/1	Crescent Gold Crown Hospital		Behind Mark Petrol Station, Tanke, Ilorin	Ilorin-South	Kwara
3797	KW/0176	KW/0176/S/6	Crescent Gold Crown Hospital		Behind Mark Petrol Station, Tanke, Ilorin	Ilorin-South	Kwara
3798	KW/0176	KW/0176/S/3	Crescent Gold Crown Hospital		Behind Mark Petrol Station, Tanke, Ilorin	Ilorin-South	Kwara
3799	KW/0177	KW/0177/S/8	22 BDE MC, Ilorin		22 Brigade, Sobi Barracks, Ilorin, Kwara state	Ilorin East	Kwara
3800	KW/0177	KW/0177/S/5	22 BDE MC, Ilorin		22 Brigade, Sobi Barracks, Ilorin, Kwara state	Ilorin East	Kwara
3801	KW/0178	KW/0178/S/4	Oko Medical Centre		Idofin Road, Oko	Irepodun	Kwara
3802	KW/0178	KW/0178/S/7	Oko Medical Centre		Idofin Road, Oko	Irepodun	Kwara
3803	KW/0178	KW/0178/S/5	Oko Medical Centre		Idofin Road, Oko	Irepodun	Kwara
3804	KW/0190	KW/0190/S/4	Landmark University Medical Centre		Landmark University, Omuaran, Kwara State	Irepodun	Kwara
3805	KW/0190	KW/0190/S/15	Landmark University Medical Centre		Landmark University, Omuaran, Kwara State	Irepodun	Kwara
3806	KW/0190	KW/0190/S/5	Landmark University Medical Centre		Landmark University, Omuaran, Kwara State	Irepodun	Kwara
3807	KW/0190	KW/0190/S/3	Landmark University Medical Centre		Landmark University, Omuaran, Kwara State	Irepodun	Kwara
3808	KW/0190	KW/0190/S/7	Landmark University Medical Centre		Landmark University, Omuaran, Kwara State	Irepodun	Kwara
3809	KW/0191	KW/0191/S/14	Notre Dame Medical Centre		Along Kabba Kajola Road, Amoyo Town, Kwara State	Ifelodun	Kwara
3810	KW/0198	KW/0198/S/1	Yusjib Industrial Medicare		35, Ajase Ipo Road, (opposite Oando filling station, offa garage), Ilorin, Kwara State	Ilorin-South	Kwara
3811	KW/0198	KW/0198/S/3	Yusjib Industrial Medicare		35, Ajase Ipo Road, (opposite Oando filling station, offa garage), Ilorin, Kwara State	Ilorin-South	Kwara
3812	KW/0199	KW/0199/S/3	Midland Fertility Center		12 Kano Road, Adewole Estate, Ilorin, Kwara State	Ilorin-West	Kwara
3813	KW/0206	KW/0206/S/5	Kwara Advanced Diagnostic center		Along Asa Dam Road, By New Yidi Road Junction, Ilorin, Kwara State	Ilorin-West	Kwara
3814	KW/0206	KW/0206/S/7	Kwara Advanced Diagnostic center		Along Asa Dam Road, By New Yidi Road Junction, Ilorin, Kwara State	Ilorin-West	Kwara
3815	KW/0209	KW/0209/S/3	Group Medical Centre		Pipeline Road, Tanke Ilorin, Kwara State.	Ilorin-South	Kwara
3816	KW/0213	KW/0213/S/1	Akanji Memorial Hospital		136 Sobi Road Ilorin, Kwara State	Ilorin East	Kwara
3817	KW/0213	KW/0213/S/3	Akanji Memorial Hospital		136 Sobi Road Ilorin, Kwara State	Ilorin East	Kwara
3818	KW/0214	KW/0214/S/12	General Hospital Ilorin		Abdulazeez Attah Road Ilorin, Kwara State	Ilorin-West	Kwara
3819	KW/0214	KW/0214/S/15	General Hospital Ilorin		Abdulazeez Attah Road Ilorin, Kwara State	Ilorin-West	Kwara
3820	KW/0214	KW/0214/S/1	General Hospital Ilorin		Abdulazeez Attah Road Ilorin, Kwara State	Ilorin-West	Kwara
3821	KW/0214	KW/0214/S/3	General Hospital Ilorin		Abdulazeez Attah Road Ilorin, Kwara State	Ilorin-West	Kwara
3822	KW/0214	KW/0214/S/4	General Hospital Ilorin		Abdulazeez Attah Road Ilorin, Kwara State	Ilorin-West	Kwara
3823	KW/0214	KW/0214/S/9	General Hospital Ilorin		Abdulazeez Attah Road Ilorin, Kwara State	Ilorin-West	Kwara
3824	KW/0214	KW/0214/S/5	General Hospital Ilorin		Abdulazeez Attah Road Ilorin, Kwara State	Ilorin-West	Kwara
3825	KW/0214	KW/0214/S/8	General Hospital Ilorin		Abdulazeez Attah Road Ilorin, Kwara State	Ilorin-West	Kwara

3826	KW/0214	KW/0214/S/7	General Hospital Ilorin		Abdulazeez Attah Road Ilorin, Kwara State	Ilorin-West	Kwara
3827	KW/0214	KW/0214/S/6	General Hospital Ilorin		Abdulazeez Attah Road Ilorin, Kwara State	Ilorin-West	Kwara
3828	KW/0214	KW/0214/S/2	General Hospital Ilorin		Abdulazeez Attah Road Ilorin, Kwara State	Ilorin-West	Kwara
3829	LA/0002	LA/0002/S/2	Talent Specialist Hospital		Plot 440 4th Avenue, Gowon Estate, Egbeda, Lagos	Agege	Lagos
3830	LA/0010	LA/0010/S/7	Osuntuyi Medical Center, iju_ishaga		255 Iju Rd, Balogun B/Stop, Iju-Ishaga	Agege	Lagos
3831	LA/0010	LA/0010/S/1	Osuntuyi Medical Center, iju_ishaga		255 Iju Rd, Balogun B/Stop, Iju-Ishaga	Agege	Lagos
3832	LA/0010	LA/0010/S/8	Osuntuyi Medical Center, iju_ishaga		255 Iju Rd, Balogun B/Stop, Iju-Ishaga	Agege	Lagos
3833	LA/0010	LA/0010/S/6	Osuntuyi Medical Center, iju_ishaga		255 Iju Rd, Balogun B/Stop, Iju-Ishaga	Agege	Lagos
3834	LA/0010	LA/0010/S/5	Osuntuyi Medical Center, iju_ishaga		255 Iju Rd, Balogun B/Stop, Iju-Ishaga	Agege	Lagos
3835	LA/0010	LA/0010/S/4	Osuntuyi Medical Center, iju_ishaga		255 Iju Rd, Balogun B/Stop, Iju-Ishaga	Agege	Lagos
3836	LA/0010	LA/0010/S/3	Osuntuyi Medical Center, iju_ishaga		255 Iju Rd, Balogun B/Stop, Iju-Ishaga	Agege	Lagos
3837	LA/0010	LA/0010/S/2	Osuntuyi Medical Center, iju_ishaga		255 Iju Rd, Balogun B/Stop, Iju-Ishaga	Agege	Lagos
3838	LA/0012	LA/0012/S/1	General Hospital, Orile-Agege	-	Orile-Agege	Agege	Lagos
3839	LA/0012	LA/0012/S/8	General Hospital, Orile-Agege	-	Orile-Agege	Agege	Lagos
3840	LA/0012	LA/0012/S/7	General Hospital, Orile-Agege	-	Orile-Agege	Agege	Lagos
3841	LA/0012	LA/0012/S/2	General Hospital, Orile-Agege	-	Orile-Agege	Agege	Lagos
3842	LA/0012	LA/0012/S/3	General Hospital, Orile-Agege	-	Orile-Agege	Agege	Lagos
3843	LA/0017	LA/0017/S/1	Mucas Hospital		19 Ogun Str., Adeolu Bus Stop, Dopemu, Agege, Lagos.	Agege	Lagos
3844	LA/0017	LA/0017/S/3	Mucas Hospital		19 Ogun Str., Adeolu Bus Stop, Dopemu, Agege, Lagos.	Agege	Lagos
3845	LA/0019	LA/0019/S/4	Teo Pharmaceutical Nigeria Ltd.	Pharmacy	172, Akowonjo Road, Akowonjo	Alimosho	Lagos
3846	LA/0021	LA/0021/S/1	Fruitful Vine Pharmacy	Pharmacy	115, Idimu Road, Egbeda	Alimosho	Lagos
3847	LA/0022	LA/0022/S/4	Lola Pharmacy & Stores Ltd.	Pharmacy	10, Idimu Road, Egeda Bus Stop Egbeda	Alimosho	Lagos
3848	LA/0023	LA/0023/S/4	Boluke Pharm. Ltd	Pharmacy	69, Old Abeokuta	Alimosho	Lagos
3849	LA/0024	LA/0024/S/4	Boluke Pharm. Ltd	Pharmacy	16 Iju Road, Agege, Lagos	Agege	Lagos
3850	LA/0025	LA/0025/S/4	Bolutife Pharmacy & Stores Ltd.	Pharmacy	104, Ijagemo Road Ijegun Garrage, Ijegun, Lagos	Lagos Mainland	Lagos
3851	LA/0029	LA/0029/S/4	Edimot Pharmacy	Pharmacy	103/107 Old Otta rd, Orile Agege	Agege	Lagos
3852	LA/0033	LA/0033/S/7	Newton Diagnostic Services Ltd.	Radiology	19 Ogun Street,Adeolu Bus Stop, Dopemu	Alimosho	Lagos
3853	LA/0034	LA/0034/S/10	Dr.Essien I. Ekong Hospital	Orthopedric Surgery	1, Kemfat Road, Thomas Village, Ajah.	Agege	Lagos
3854	LA/0036	LA/0036/S/5	Ajeromi General Hospital		Awodi-Ora Ajegunle	Ajeromi/Ifelodun	Lagos
3855	LA/0036	LA/0036/S/1	Ajeromi General Hospital		Awodi-Ora Ajegunle	Ajeromi/Ifelodun	Lagos
3856	LA/0036	LA/0036/S/3	Ajeromi General Hospital		Awodi-Ora Ajegunle	Ajeromi/Ifelodun	Lagos
3857	LA/0036	LA/0036/S/8	Ajeromi General Hospital		Awodi-Ora Ajegunle	Ajeromi/Ifelodun	Lagos
3858	LA/0036	LA/0036/S/4	Ajeromi General Hospital		Awodi-Ora Ajegunle	Ajeromi/Ifelodun	Lagos
3859	LA/0036	LA/0036/S/2	Ajeromi General Hospital		Awodi-Ora Ajegunle	Ajeromi/Ifelodun	Lagos
3860	LA/0036	LA/0036/S/11	Ajeromi General Hospital		Awodi-Ora Ajegunle	Ajeromi/Ifelodun	Lagos
3861	LA/0037	LA/0037/S/5	Prince & Princess Hospital		191 Ojo Road, Ligoli Bus Stop, Ajegunle, Lagos.	Ajeromi/Ifelodun	Lagos
3862	LA/0037	LA/0037/S/2	Prince & Princess Hospital		191 Ojo Road, Ligoli Bus Stop, Ajegunle, Lagos.	Ajeromi/Ifelodun	Lagos
3863	LA/0037	LA/0037/S/4	Prince & Princess Hospital		191 Ojo Road, Ligoli Bus Stop, Ajegunle, Lagos.	Ajeromi/Ifelodun	Lagos
3864	LA/0038	LA/0038/S/1	All Soul's Clinics Ltd		No. 235 Ojo Road, Ajegunle, Lagos	Ajeromi/Ifelodun	Lagos
3865	LA/0038	LA/0038/S/7	All Soul's Clinics Ltd		No. 235 Ojo Road, Ajegunle, Lagos	Ajeromi/Ifelodun	Lagos
3866	LA/0045	LA/0045/S/2	Broad Hospital & Materinty		17 Fehintola Street , Off Isijola Street Ikotun Lagos	Alimosho	Lagos
3867	LA/0045	LA/0045/S/6	Broad Hospital & Materinty		17 Fehintola Street , Off Isijola Street Ikotun Lagos	Alimosho	Lagos
3868	LA/0045	LA/0045/S/5	Broad Hospital & Materinty		17 Fehintola Street , Off Isijola Street Ikotun Lagos	Alimosho	Lagos

3869	LA/0045	LA/0045/S/1	Broad Hospital & Materinty		17 Fehintola Street , Off Isijola Street Ikotun Lagos	Alimosho	Lagos
3870	LA/0045	LA/0045/S/3	Broad Hospital & Materinty		17 Fehintola Street , Off Isijola Street Ikotun Lagos	Alimosho	Lagos
3871	LA/0047	LA/0047/S/5	Veta Hospital Ltd.		1, Veta Close, Araromi Bus Stop Abeoukuta Expressway Iyana-Paja Lagos	Alimosho	Lagos
3872	LA/0047	LA/0047/S/4	Veta Hospital Ltd.		1, Veta Close, Araromi Bus Stop Abeoukuta Expressway Iyana-Paja Lagos	Alimosho	Lagos
3873	LA/0049	LA/0049/S/5	Duramo- Moses, Lab.Diag. Services & Co.	Laboratory	79/81 Almosho Road, Agege	Agege	Lagos
3874	LA/0055	LA/0055/S/4	Malgoz Clincal Lab. Ltd.	Pharmacy	1, Akanbi Soetan St/Old Ojo Road Ajakija B/Stop. Amuwo-Odofi	Amuwo-Odofin	Lagos
3875	LA/0057	LA/0057/S/12	Shepherd Specialist Hospital		2nd Avenue 23 Road, T Close House 38, Festac , Lagos	Amuwo-Odofin	Lagos
3876	LA/0057	LA/0057/S/3	Shepherd Specialist Hospital		2nd Avenue 23 Road, T Close House 38, Festac , Lagos	Amuwo-Odofin	Lagos
3877	LA/0057	LA/0057/S/2	Shepherd Specialist Hospital		2nd Avenue 23 Road, T Close House 38, Festac , Lagos	Amuwo-Odofin	Lagos
3878	LA/0057	LA/0057/S/1	Shepherd Specialist Hospital		2nd Avenue 23 Road, T Close House 38, Festac , Lagos	Amuwo-Odofin	Lagos
3879	LA/0057	LA/0057/S/6	Shepherd Specialist Hospital		2nd Avenue 23 Road, T Close House 38, Festac , Lagos	Amuwo-Odofin	Lagos
3880	LA/0061	LA/0061/S/7	Praise Medical Centre, Festac.		3rd Avenue O'' Close House 2, Festac Town, Lagos.	Amuwo-Odofin	Lagos
3881	LA/0061	LA/0061/S/11	Praise Medical Centre, Festac.		3rd Avenue O'' Close House 2, Festac Town, Lagos.	Amuwo-Odofin	Lagos
3882	LA/0061	LA/0061/S/1	Praise Medical Centre, Festac.		3rd Avenue O'' Close House 2, Festac Town, Lagos.	Amuwo-Odofin	Lagos
3883	LA/0061	LA/0061/S/6	Praise Medical Centre, Festac.		3rd Avenue O'' Close House 2, Festac Town, Lagos.	Amuwo-Odofin	Lagos
3884	LA/0061	LA/0061/S/3	Praise Medical Centre, Festac.		3rd Avenue O'' Close House 2, Festac Town, Lagos.	Amuwo-Odofin	Lagos
3885	LA/0061	LA/0061/S/5	Praise Medical Centre, Festac.		3rd Avenue O'' Close House 2, Festac Town, Lagos.	Amuwo-Odofin	Lagos
3886	LA/0062	LA/0062/S/3	St. Anthony's Medical Centre		19 Badagry Expressway, Festac Town, Lagos	Amuwo-Odofin	Lagos
3887	LA/0062	LA/0062/S/1	St. Anthony's Medical Centre		19 Badagry Expressway, Festac Town, Lagos	Amuwo-Odofin	Lagos
3888	LA/0062	LA/0062/S/5	St. Anthony's Medical Centre		19 Badagry Expressway, Festac Town, Lagos	Amuwo-Odofin	Lagos
3889	LA/0063	LA/0063/S/5	Hova's Place Hospital & Clinic		House 23, T Close, 5th Avenue, Festac Town, Lagos	Amuwo-Odofin	Lagos
3890	LA/0063	LA/0063/S/15	Hova's Place Hospital & Clinic		House 23, T Close, 5th Avenue, Festac Town, Lagos	Amuwo-Odofin	Lagos
3891	LA/0069	LA/0069/S/4	Oxpharm Specialities Ltd.	Pharmacy	32 Road, By 3rd Avenue, Festac Town	Amuwo-Odofin	Lagos
3892	LA/0070	LA/0070/S/4	Davtin Pharmacy & Stores	Pharmacy	21 Road, G Close, House 2, Festac	Amuwo-Odofin	Lagos
3893	LA/0071	LA/0071/S/4	Mak-Mach Ltd.	Pharmacy	No 1, Frank Ojadi Close, Opp Golden Park Estate, Ogidan, Lekki - Epe Express way, Lagos	Amuwo-Odofin	Lagos
3894	LA/0073	LA/0073/S/4	West End Pharmacy Ltd.	Pharmacy	Snow Flakes Plaza, Suite3, 311 Road F Close Festac Town	Amuwo-Odofin	Lagos
3895	LA/0074	LA/0074/S/4	Klemzy Pharmaceutical & Allied Products Ltd.	Pharmacy	5th Ave, F Close, Festac Town	Amuwo-Odofin	Lagos
3896	LA/0075	LA/0075/S/4	Whiteood Pharmaceutical & Allied Products Ltd.	Pharmacy	2nd Ave. 22 Road b/w & F Close(opp FHA water works) Festac	Amuwo-Odofin	Lagos
3897	LA/0076	LA/0076/S/5	Pharm Care Support Services Ltd.	Pharmacy	51 Road, opp NITEL Gate, Festac Town	Amuwo-Odofin	Lagos
3898	LA/0076	LA/0076/S/4	Pharm Care Support Services Ltd.	Pharmacy	51 Road, opp NITEL Gate, Festac Town	Amuwo-Odofin	Lagos
3899	LA/0080	LA/0080/S/3	St. Jude's Hospital		21 Road, B Close, House 3, Festac Town	Amuwo-Odofin	Lagos
3900	LA/0080	LA/0080/S/2	St. Jude's Hospital		21 Road, B Close, House 3, Festac Town	Amuwo-Odofin	Lagos

3901	LA/0080	LA/0080/S/1	St. Jude's Hospital		21 Road, B Close, House 3, Festac Town	Amuwo-Odofin	Lagos
3902	LA/0082	LA/0082/S/4	Ituah Hospital Ltd.		512 Road J. Close Festac Town Lagos	Amuwo-Odofin	Lagos
3903	LA/0082	LA/0082/S/5	Ituah Hospital Ltd.		512 Road J. Close Festac Town Lagos	Amuwo-Odofin	Lagos
3904	LA/0085	LA/0085/S/14	Emel Hospital	-	21 Road Z Close,Festac Town	Amuwo-Odofin	Lagos
3905	LA/0085	LA/0085/S/6	Emel Hospital	-	21 Road Z Close,Festac Town	Amuwo-Odofin	Lagos
3906	LA/0085	LA/0085/S/2	Emel Hospital	-	21 Road Z Close,Festac Town	Amuwo-Odofin	Lagos
3907	LA/0085	LA/0085/S/3	Emel Hospital	-	21 Road Z Close,Festac Town	Amuwo-Odofin	Lagos
3908	LA/0085	LA/0085/S/4	Emel Hospital	-	21 Road Z Close,Festac Town	Amuwo-Odofin	Lagos
3909	LA/0085	LA/0085/S/1	Emel Hospital	-	21 Road Z Close,Festac Town	Amuwo-Odofin	Lagos
3910	LA/0085	LA/0085/S/7	Emel Hospital	-	21 Road Z Close,Festac Town	Amuwo-Odofin	Lagos
3911	LA/0086	LA/0086/S/5	AMC Hospital		House 19, Dubar Road, Mile 2, Lagos	Amuwo-Odofin	Lagos
3912	LA/0086	LA/0086/S/14	AMC Hospital		House 19, Dubar Road, Mile 2, Lagos	Amuwo-Odofin	Lagos
3913	LA/0088	LA/0088/S/2	Lagoon Hospital, Apapa		8 Marine Road , Apapa Lagos	Apapa	Lagos
3914	LA/0088	LA/0088/S/8	Lagoon Hospital, Apapa		8 Marine Road , Apapa Lagos	Apapa	Lagos
3915	LA/0088	LA/0088/S/11	Lagoon Hospital, Apapa		8 Marine Road , Apapa Lagos	Apapa	Lagos
3916	LA/0088	LA/0088/S/5	Lagoon Hospital, Apapa		8 Marine Road , Apapa Lagos	Apapa	Lagos
3917	LA/0088	LA/0088/S/6	Lagoon Hospital, Apapa		8 Marine Road , Apapa Lagos	Apapa	Lagos
3918	LA/0088	LA/0088/S/1	Lagoon Hospital, Apapa		8 Marine Road , Apapa Lagos	Apapa	Lagos
3919	LA/0088	LA/0088/S/10	Lagoon Hospital, Apapa		8 Marine Road , Apapa Lagos	Apapa	Lagos
3920	LA/0088	LA/0088/S/12	Lagoon Hospital, Apapa		8 Marine Road , Apapa Lagos	Apapa	Lagos
3921	LA/0088	LA/0088/S/4	Lagoon Hospital, Apapa		8 Marine Road , Apapa Lagos	Apapa	Lagos
3922	LA/0088	LA/0088/S/7	Lagoon Hospital, Apapa		8 Marine Road , Apapa Lagos	Apapa	Lagos
3923	LA/0088	LA/0088/S/15	Lagoon Hospital, Apapa		8 Marine Road , Apapa Lagos	Apapa	Lagos
3924	LA/0088	LA/0088/S/3	Lagoon Hospital, Apapa		8 Marine Road , Apapa Lagos	Apapa	Lagos
3925	LA/0090	LA/0090/S/5	Dako Medical Centre (Hosp. & Mat. Home)		225b Kirikiri Rd., Trinity Bus Stop, Olodi, Apapa, Lagos.	Apapa	Lagos
3926	LA/0097	LA/0097/S/5	Faleti Medical Centre		204, Kirikiri Road, Trinity B/Stop, Olodi, Apapa, Lagos.	Apapa	Lagos
3927	LA/0099	LA/0099/S/12	Iduna Specialist Hospital		1a, Takorodi Road, Gra, Apapa, Lagos.	Apapa	Lagos
3928	LA/0099	LA/0099/S/3	Iduna Specialist Hospital		1a, Takorodi Road, Gra, Apapa, Lagos.	Apapa	Lagos
3929	LA/0099	LA/0099/S/15	Iduna Specialist Hospital		1a, Takorodi Road, Gra, Apapa, Lagos.	Apapa	Lagos
3930	LA/0099	LA/0099/S/5	Iduna Specialist Hospital		1a, Takorodi Road, Gra, Apapa, Lagos.	Apapa	Lagos
3931	LA/0099	LA/0099/S/1	Iduna Specialist Hospital		1a, Takorodi Road, Gra, Apapa, Lagos.	Apapa	Lagos
3932	LA/0099	LA/0099/S/2	Iduna Specialist Hospital		1a, Takorodi Road, Gra, Apapa, Lagos.	Apapa	Lagos
3933	LA/0099	LA/0099/S/7	Iduna Specialist Hospital		1a, Takorodi Road, Gra, Apapa, Lagos.	Apapa	Lagos
3934	LA/0099	LA/0099/S/6	Iduna Specialist Hospital		1a, Takorodi Road, Gra, Apapa, Lagos.	Apapa	Lagos
3935	LA/0101	LA/0101/S/5	Christ Medical Centre		28 Randle Road, Apapa, Lagos	Apapa	Lagos
3936	LA/0102	LA/0102/S/5	Hilton Hospital		2 Berger Street, Olodi, Apapa, Lagos.	Apapa	Lagos
3937	LA/0104	LA/0104/S/4	Jogen Pharm & Trading company Ltd.	Pharmacy	30 Ojo Road, Apapa	Apapa	Lagos
3938	LA/0105	LA/0105/S/4	Jaykay Pharmacy Ltd.	Pharmacy	9 Randie Crescent, Apapa	Apapa	Lagos
3939	LA/0106	LA/0106/S/4	Jaykay Pharmacy Ltd.	Pharmacy	14 Adeniyi Jones Avenue, Ikeja	Ikeja	Lagos
3940	LA/0111	LA/0111/S/8	General Hospital, Badagry		Badagry	Badagry	Lagos
3941	LA/0112	LA/0112/S/1	Grace-Land Medical Center		147, Badagry Expressway	Ojo	Lagos
3942	LA/0112	LA/0112/S/5	Grace-Land Medical Center		147, Badagry Expressway	Ojo	Lagos
3943	LA/0112	LA/0112/S/7	Grace-Land Medical Center		147, Badagry Expressway	Ojo	Lagos

3944	LA/0112	LA/0112/S/4	Grace-Land Medical Center		147, Badagry Expressway	Ojo	Lagos
3945	LA/0112	LA/0112/S/3	Grace-Land Medical Center		147, Badagry Expressway	Ojo	Lagos
3946	LA/0114	LA/0114/S/5	Kilad Merit Diagnostic Centre	Laboratory	Casidy Bus Stop, Badagry Nexpressway Okokomaiko	Badagry	Lagos
3947	LA/0116	LA/0116/S/15	Udeco Eye Clinic	Ophthamology	Suite E100, Ikota Shopping Complex, Lekki	Eti-Osa	Lagos
3948	LA/0117	LA/0117/S/2	Pennisula Hospital	Internal Medicine,O & G,Radiology,Laboratory,Ophthamology,General Surgery	KM 23, Lekki-Epe Exp. Way,Lekki	Eti-Osa	Lagos
3949	LA/0117	LA/0117/S/1	Pennisula Hospital	Internal Medicine,O & G,Radiology,Laboratory,Ophthamology,General Surgery	KM 23, Lekki-Epe Exp. Way,Lekki	Eti-Osa	Lagos
3950	LA/0117	LA/0117/S/7	Pennisula Hospital	Internal Medicine,O & G,Radiology,Laboratory,Ophthamology,General Surgery	KM 23, Lekki-Epe Exp. Way,Lekki	Eti-Osa	Lagos
3951	LA/0117	LA/0117/S/15	Pennisula Hospital	Internal Medicine,O & G,Radiology,Laboratory,Ophthamology,General Surgery	KM 23, Lekki-Epe Exp. Way,Lekki	Eti-Osa	Lagos
3952	LA/0117	LA/0117/S/5	Pennisula Hospital	Internal Medicine,O & G,Radiology,Laboratory,Ophthamology,General Surgery	KM 23, Lekki-Epe Exp. Way,Lekki	Eti-Osa	Lagos
3953	LA/0117	LA/0117/S/3	Pennisula Hospital	Internal Medicine,O & G,Radiology,Laboratory,Ophthamology,General Surgery	KM 23, Lekki-Epe Exp. Way,Lekki	Eti-Osa	Lagos
3954	LA/0118	LA/0118/S/5	The Royal Infirmary	General Medicine,General Surgery,O&G,Laboratory, Pharmacy	36,Aiyetoro Str. By HFP/NGC off Lekki -Epe Exp. Way, Ajah	Damaturu	Yobe
3955	LA/0118	LA/0118/S/1	The Royal Infirmary	General Medicine,General Surgery,O&G,Laboratory, Pharmacy	36,Aiyetoro Str. By HFP/NGC off Lekki -Epe Exp. Way, Ajah	Damaturu	Yobe
3956	LA/0118	LA/0118/S/3	The Royal Infirmary	General Medicine,General Surgery,O&G,Laboratory, Pharmacy	36,Aiyetoro Str. By HFP/NGC off Lekki -Epe Exp. Way, Ajah	Damaturu	Yobe
3957	LA/0118	LA/0118/S/4	The Royal Infirmary	General Medicine,General Surgery,O&G,Laboratory, Pharmacy	36,Aiyetoro Str. By HFP/NGC off Lekki -Epe Exp. Way, Ajah	Damaturu	Yobe
3958	LA/0118	LA/0118/S/2	The Royal Infirmary	General Medicine,General Surgery,O&G,Laboratory, Pharmacy	36,Aiyetoro Str. By HFP/NGC off Lekki -Epe Exp. Way, Ajah	Damaturu	Yobe
3959	LA/0119	LA/0119/S/8	Oral Dent, Dental Clinics	Dental	E62, Ikota Shopping Complex, Lekki	Eti-Osa	Lagos
3960	LA/0123	LA/0123/S/4	Doreen Pharmacy	Pharmacy	1, Kemfat Road,Thomas Village,Lekki-Epe Express Way	Eti-Osa	Lagos
3961	LA/0125	LA/0125/S/4	General Hospital, Epe		Epe	Epe	Lagos
3962	LA/0125	LA/0125/S/3	General Hospital, Epe		Epe	Epe	Lagos
3963	LA/0125	LA/0125/S/1	General Hospital, Epe		Epe	Epe	Lagos
3964	LA/0127	LA/0127/S/15	Unique Eye Centre Ltd.	Ophthalmology	21,Obalande Rd. Ikoyi	Eti-Osa	Lagos

3965	LA/0129	LA/0129/S/12	Crest Consultant Clinics	Int. Medicine,General Surgery,O &G,Paediatrics,Ophthalmology,ENT, Radiology,Laboratory, Pharmacy	2,Keffi St., S.W. Ikoyi	Eti-Osa	Lagos
3966	LA/0129	LA/0129/S/7	Crest Consultant Clinics	Int. Medicine,General Surgery,O &G,Paediatrics,Ophthalmology,ENT, Radiology,Laboratory, Pharmacy	2,Keffi St., S.W. Ikoyi	Eti-Osa	Lagos
3967	LA/0134	LA/0134/S/5	Doren Specialist Hospital		1 Kemfat Road , Thomas Village, lekki.	Eti-Osa	Lagos
3968	LA/0134	LA/0134/S/7	Doren Specialist Hospital		1 Kemfat Road , Thomas Village, lekki.	Eti-Osa	Lagos
3969	LA/0134	LA/0134/S/2	Doren Specialist Hospital		1 Kemfat Road , Thomas Village, lekki.	Eti-Osa	Lagos
3970	LA/0134	LA/0134/S/1	Doren Specialist Hospital		1 Kemfat Road , Thomas Village, lekki.	Eti-Osa	Lagos
3971	LA/0134	LA/0134/S/10	Doren Specialist Hospital		1 Kemfat Road , Thomas Village, lekki.	Eti-Osa	Lagos
3972	LA/0135	LA/0135/S/2	Kings Hospital		39 Mashalashi Street off Keffi Road, Obalende, Ikoyi	Eti-Osa	Lagos
3973	LA/0135	LA/0135/S/5	Kings Hospital		39 Mashalashi Street off Keffi Road, Obalende, Ikoyi	Eti-Osa	Lagos
3974	LA/0135	LA/0135/S/3	Kings Hospital		39 Mashalashi Street off Keffi Road, Obalende, Ikoyi	Eti-Osa	Lagos
3975	LA/0135	LA/0135/S/1	Kings Hospital		39 Mashalashi Street off Keffi Road, Obalende, Ikoyi	Eti-Osa	Lagos
3976	LA/0136	LA/0136/S/7	Gold Cross Hospital		17B Bourdillon Road, Ikoyi	Eti-Osa	Lagos
3977	LA/0136	LA/0136/S/1	Gold Cross Hospital		17B Bourdillon Road, Ikoyi	Eti-Osa	Lagos
3978	LA/0136	LA/0136/S/10	Gold Cross Hospital		17B Bourdillon Road, Ikoyi	Eti-Osa	Lagos
3979	LA/0136	LA/0136/S/5	Gold Cross Hospital		17B Bourdillon Road, Ikoyi	Eti-Osa	Lagos
3980	LA/0136	LA/0136/S/4	Gold Cross Hospital		17B Bourdillon Road, Ikoyi	Eti-Osa	Lagos
3981	LA/0143	LA/0143/S/5	J-Rapha Hospital		40 Ado Lamgbasa Road, Ajah,	Eti-Osa	Lagos
3982	LA/0143	LA/0143/S/4	J-Rapha Hospital		40 Ado Lamgbasa Road, Ajah,	Eti-Osa	Lagos
3983	LA/0145	LA/0145/S/3	Germaine Health Centre		Km 63 Lagos/Epe Expressway, Lekki, Lagos.	Eti-Osa	Lagos
3984	LA/0145	LA/0145/S/2	Germaine Health Centre		Km 63 Lagos/Epe Expressway, Lekki, Lagos.	Eti-Osa	Lagos
3985	LA/0145	LA/0145/S/7	Germaine Health Centre		Km 63 Lagos/Epe Expressway, Lekki, Lagos.	Eti-Osa	Lagos
3986	LA/0145	LA/0145/S/5	Germaine Health Centre		Km 63 Lagos/Epe Expressway, Lekki, Lagos.	Eti-Osa	Lagos
3987	LA/0145	LA/0145/S/10	Germaine Health Centre		Km 63 Lagos/Epe Expressway, Lekki, Lagos.	Eti-Osa	Lagos
3988	LA/0147	LA/0147/S/7	The Peninsular Hospital		Km 23, Lagos Expressway, Ikota, Lekki, Lagos	Eti-Osa	Lagos
3989	LA/0147	LA/0147/S/5	The Peninsular Hospital		Km 23, Lagos Expressway, Ikota, Lekki, Lagos	Eti-Osa	Lagos
3990	LA/0147	LA/0147/S/1	The Peninsular Hospital		Km 23, Lagos Expressway, Ikota, Lekki, Lagos	Eti-Osa	Lagos
3991	LA/0150	LA/0150/S/1	Udeco Medical Centre		Plot 39, 36 Udeco Medical Road off Chevron Drive, Chevy Estate, Lekki	Eti-Osa	Lagos
3992	LA/0150	LA/0150/S/15	Udeco Medical Centre		Plot 39, 36 Udeco Medical Road off Chevron Drive, Chevy Estate, Lekki	Eti-Osa	Lagos
3993	LA/0150	LA/0150/S/2	Udeco Medical Centre		Plot 39, 36 Udeco Medical Road off Chevron Drive, Chevy Estate, Lekki	Eti-Osa	Lagos
3994	LA/0156	LA/0156/S/4	Pearle Pharmacy	Pharmacy	32, Ajeniya St. Obalende, Ikoyi	Eti-Osa	Lagos

3995	LA/0159	LA/0159/S/4	His-Care Int'l	Pharmacy	Suite 1, New Giwa Shopping Complex, Rumens Road, Off Kingswa	Eti-Osa	Lagos
3996	LA/0160	LA/0160/S/7	Cosmos Diagnostic Centre	Radiology , Mammagram , Laboratory	202 Awolowo Road Folomo Ikoyi	Eti-Osa	Lagos
3997	LA/0160	LA/0160/S/5	Cosmos Diagnostic Centre	Radiology , Mammagram , Laboratory	202 Awolowo Road Folomo Ikoyi	Eti-Osa	Lagos
3998	LA/0167	LA/0167/S/5	Fehinbade Dignostics	Laboratory	2 Lawal Street off Church Bus Stop, Jankara, Ijaiye Ojokoro	Ifako-Ijaye	Lagos
3999	LA/0174	LA/0174/S/7	Eko Hospital, Ikeja		31, Mobolaji Bank, Anthony Way Ikeja	Ikeja	Lagos
4000	LA/0174	LA/0174/S/8	Eko Hospital, Ikeja		31, Mobolaji Bank, Anthony Way Ikeja	Ikeja	Lagos
4001	LA/0174	LA/0174/S/15	Eko Hospital, Ikeja		31, Mobolaji Bank, Anthony Way Ikeja	Ikeja	Lagos
4002	LA/0174	LA/0174/S/1	Eko Hospital, Ikeja		31, Mobolaji Bank, Anthony Way Ikeja	Ikeja	Lagos
4003	LA/0174	LA/0174/S/3	Eko Hospital, Ikeja		31, Mobolaji Bank, Anthony Way Ikeja	Ikeja	Lagos
4004	LA/0174	LA/0174/S/6	Eko Hospital, Ikeja		31, Mobolaji Bank, Anthony Way Ikeja	Ikeja	Lagos
4005	LA/0174	LA/0174/S/2	Eko Hospital, Ikeja		31, Mobolaji Bank, Anthony Way Ikeja	Ikeja	Lagos
4006	LA/0174	LA/0174/S/12	Eko Hospital, Ikeja		31, Mobolaji Bank, Anthony Way Ikeja	Ikeja	Lagos
4007	LA/0174	LA/0174/S/10	Eko Hospital, Ikeja		31, Mobolaji Bank, Anthony Way Ikeja	Ikeja	Lagos
4008	LA/0176	LA/0176/S/3	Bodet Medicare & Maternity		6/8 Odewale St. Alausa, Ikeja	Ikeja	Lagos
4009	LA/0177	LA/0177/S/1	Brafus Specialist Hospital		1b Ajayi Road, Ogba	Ikeja	Lagos
4010	LA/0179	LA/0179/S/5	St. Leo's Catholic Clinic		C/O Catholic Church,10 Toyin Str. Ikeja	Ikeja	Lagos
4011	LA/0181	LA/0181/S/5	Life Support Medical Center	-	1, Oba Docemo Street G.R.A Ikeja, Lagos.	Ikeja	Lagos
4012	LA/0181	LA/0181/S/2	Life Support Medical Center	-	1, Oba Docemo Street G.R.A Ikeja, Lagos.	Ikeja	Lagos
4013	LA/0182	LA/0182/S/6	St. Ives Medical Centre		90 Opebi Rd. Ikeja	Ikeja	Lagos
4014	LA/0182	LA/0182/S/7	St. Ives Medical Centre		90 Opebi Rd. Ikeja	Ikeja	Lagos
4015	LA/0182	LA/0182/S/14	St. Ives Medical Centre		90 Opebi Rd. Ikeja	Ikeja	Lagos
4016	LA/0182	LA/0182/S/4	St. Ives Medical Centre		90 Opebi Rd. Ikeja	Ikeja	Lagos
4017	LA/0182	LA/0182/S/3	St. Ives Medical Centre		90 Opebi Rd. Ikeja	Ikeja	Lagos
4018	LA/0182	LA/0182/S/5	St. Ives Medical Centre		90 Opebi Rd. Ikeja	Ikeja	Lagos
4019	LA/0186	LA/0186/S/3	Ikeja Medical Centre		11 Ogun Modede Street Off, Allen Avenue , Ikeja	Ikeja	Lagos
4020	LA/0186	LA/0186/S/1	Ikeja Medical Centre		11 Ogun Modede Street Off, Allen Avenue , Ikeja	Ikeja	Lagos
4021	LA/0186	LA/0186/S/14	Ikeja Medical Centre		11 Ogun Modede Street Off, Allen Avenue , Ikeja	Ikeja	Lagos
4022	LA/0186	LA/0186/S/8	Ikeja Medical Centre		11 Ogun Modede Street Off, Allen Avenue , Ikeja	Ikeja	Lagos
4023	LA/0186	LA/0186/S/4	Ikeja Medical Centre		11 Ogun Modede Street Off, Allen Avenue , Ikeja	Ikeja	Lagos
4024	LA/0186	LA/0186/S/5	Ikeja Medical Centre		11 Ogun Modede Street Off, Allen Avenue , Ikeja	Ikeja	Lagos
4025	LA/0186	LA/0186/S/15	Ikeja Medical Centre		11 Ogun Modede Street Off, Allen Avenue , Ikeja	Ikeja	Lagos
4026	LA/0186	LA/0186/S/7	Ikeja Medical Centre		11 Ogun Modede Street Off, Allen Avenue , Ikeja	Ikeja	Lagos
4027	LA/0189	LA/0189/S/4	County Hospital Ltd.		41-45 Isheri Road Ogba Ikeja Lagos	Ikeja	Lagos
4028	LA/0189	LA/0189/S/15	County Hospital Ltd.		41-45 Isheri Road Ogba Ikeja Lagos	Ikeja	Lagos
4029	LA/0189	LA/0189/S/7	County Hospital Ltd.		41-45 Isheri Road Ogba Ikeja Lagos	Ikeja	Lagos
4030	LA/0189	LA/0189/S/1	County Hospital Ltd.		41-45 Isheri Road Ogba Ikeja Lagos	Ikeja	Lagos
4031	LA/0189	LA/0189/S/6	County Hospital Ltd.		41-45 Isheri Road Ogba Ikeja Lagos	Ikeja	Lagos
4032	LA/0189	LA/0189/S/14	County Hospital Ltd.		41-45 Isheri Road Ogba Ikeja Lagos	Ikeja	Lagos
4033	LA/0189	LA/0189/S/5	County Hospital Ltd.		41-45 Isheri Road Ogba Ikeja Lagos	Ikeja	Lagos
4034	LA/0189	LA/0189/S/2	County Hospital Ltd.		41-45 Isheri Road Ogba Ikeja Lagos	Ikeja	Lagos
4035	LA/0189	LA/0189/S/3	County Hospital Ltd.		41-45 Isheri Road Ogba Ikeja Lagos	Ikeja	Lagos
4036	LA/0190	LA/0190/S/3	Motayo Hospital Limited		3. Owodumi Street, Off Toyin Street Ikeja Lagos	Ikeja	Lagos

4037	LA/0190	LA/0190/S/7	Motayo Hospital Limited		3. Owodumi Street, Off Toyin Street Ikeja Lagos	Ikeja	Lagos
4038	LA/0190	LA/0190/S/4	Motayo Hospital Limited		3. Owodumi Street, Off Toyin Street Ikeja Lagos	Ikeja	Lagos
4039	LA/0190	LA/0190/S/1	Motayo Hospital Limited		3. Owodumi Street, Off Toyin Street Ikeja Lagos	Ikeja	Lagos
4040	LA/0190	LA/0190/S/8	Motayo Hospital Limited		3. Owodumi Street, Off Toyin Street Ikeja Lagos	Ikeja	Lagos
4041	LA/0190	LA/0190/S/6	Motayo Hospital Limited		3. Owodumi Street, Off Toyin Street Ikeja Lagos	Ikeja	Lagos
4042	LA/0191	LA/0191/S/2	CRI Medi Clinics - Ikeja		Ikeja Opearations Hq Ngb Building 144 Oba Akran Avenue Ikeja	Ikeja	Lagos
4043	LA/0191	LA/0191/S/4	CRI Medi Clinics - Ikeja		Ikeja Opearations Hq Ngb Building 144 Oba Akran Avenue Ikeja	Ikeja	Lagos
4044	LA/0191	LA/0191/S/5	CRI Medi Clinics - Ikeja		Ikeja Opearations Hq Ngb Building 144 Oba Akran Avenue Ikeja	Ikeja	Lagos
4045	LA/0191	LA/0191/S/7	CRI Medi Clinics - Ikeja		Ikeja Opearations Hq Ngb Building 144 Oba Akran Avenue Ikeja	Ikeja	Lagos
4046	LA/0206	LA/0206/S/4	Pharm. Agboola Oguntonade	Pharmacy	1, Oguntonade Close, Okupe Estate, Maryland	Ikeja	Lagos
4047	LA/0209	LA/0209/S/5	Kowa Medical Diagnosis Laboratory	Laboratory	12 Medical Road, Ikeja	Ikeja	Lagos
4048	LA/0211	LA/0211/S/5	Jimak Medical Diagnosis Lab.	Laboratory	17, Medical Road, Simbat Rd. Ikeja	Ikeja	Lagos
4049	LA/0213	LA/0213/S/4	Pharmarision Ltd.	Pharmacy	36, Oluwoleyimu Street, Ikeja	Ikeja	Lagos
4050	LA/0217	LA/0217/S/4	CRI Mediclinic	Pharmacy	Isaac John Street, GRA, Ikeja	Ikeja	Lagos
4051	LA/0220	LA/0220/S/4	Alpha Pharmacy & Stores Ltd.	Pharmacy	2B Alabi Street, Off Toyin Street, Ikeja	Ikeja	Lagos
4052	LA/0222	LA/0222/S/4	Kerson Pharmaceuticals (Nig.) Ltd.	Pharmacy	5, Diya Street, Ifako, Gbadada, Ikeja	Ikeja	Lagos
4053	LA/0226	LA/0226/S/4	Bolar Pharmaceuticals Ltd.	Pharmacy	72A Adeniyi Jones Avenue, Ikeja	Ikeja	Lagos
4054	LA/0228	LA/0228/S/4	Dukeb Pharmaceuticals Ltd.	Pharmacy	9 Olabode Str., Ajao Estate, Ikeja	Ikeja	Lagos
4055	LA/0231	LA/0231/S/4	Boluke Pharm Ltd.	Pharmacy	134, Awolowo Way, Ikeja	Ikeja	Lagos
4056	LA/0232	LA/0232/S/4	Moray Pharm	Pharmacy	21, Kodesoh Street, Ikeja	Ikeja	Lagos
4057	LA/0233	LA/0233/S/4	Pharma Vision Ltd.	Pharmacy	36, Oluwoleyinmi Street, Ikeja	Ikeja	Lagos
4058	LA/0235	LA/0235/S/4	Domat Pharmacy	Pharmacy	80 Obafemi Awolowo Way, Ikeja	Ikeja	Lagos
4059	LA/0237	LA/0237/S/4	New-Gate Hospital		59 Ikorodu	Ikorodu	Lagos
4060	LA/0238	LA/0238/S/5	General Hospital, Ikorodu	-	Ikorodu, Lagos	Ikorodu	Lagos
4061	LA/0238	LA/0238/S/2	General Hospital, Ikorodu	-	Ikorodu, Lagos	Ikorodu	Lagos
4062	LA/0238	LA/0238/S/1	General Hospital, Ikorodu	-	Ikorodu, Lagos	Ikorodu	Lagos
4063	LA/0238	LA/0238/S/6	General Hospital, Ikorodu	-	Ikorodu, Lagos	Ikorodu	Lagos
4064	LA/0238	LA/0238/S/7	General Hospital, Ikorodu	-	Ikorodu, Lagos	Ikorodu	Lagos
4065	LA/0238	LA/0238/S/12	General Hospital, Ikorodu	-	Ikorodu, Lagos	Ikorodu	Lagos
4066	LA/0238	LA/0238/S/8	General Hospital, Ikorodu	-	Ikorodu, Lagos	Ikorodu	Lagos
4067	LA/0238	LA/0238/S/3	General Hospital, Ikorodu	-	Ikorodu, Lagos	Ikorodu	Lagos
4068	LA/0238	LA/0238/S/4	General Hospital, Ikorodu	-	Ikorodu, Lagos	Ikorodu	Lagos
4069	LA/0239	LA/0239/S/7	Ikorodu Hospital & Clinic.	-	9, Muniratu Alojo Street,Off Lagos Road By Afribank,Ikorodu	Ikorodu	Lagos
4070	LA/0239	LA/0239/S/5	Ikorodu Hospital & Clinic.	-	9, Muniratu Alojo Street,Off Lagos Road By Afribank,Ikorodu	Ikorodu	Lagos
4071	LA/0243	LA/0243/S/4	Ramar Pharmacy	Pharmacy	No. 29 Ayangburen Road Opp. NEPA, Ikorodu	Ikorodu	Lagos
4072	LA/0244	LA/0244/S/4	Diagnostics Associates (W.A) Ltd	Laboratory	No.4 Shagamu Rd, Ikorodu	Ikorodu	Lagos
4073	LA/0245	LA/0245/S/4	Jimac Diagnostics Medical Lab.	Laboratory	No.11 Jokodola Close, Ikorodu	Ikorodu	Lagos
4074	LA/0251	LA/0251/S/1	Deji Clinic Ltd.		19 Demurin Street, Catop Chicken George Ketu Lagos	Kosofe	Lagos
4075	LA/0251	LA/0251/S/3	Deji Clinic Ltd.		19 Demurin Street, Catop Chicken George Ketu Lagos	Kosofe	Lagos
4076	LA/0251	LA/0251/S/4	Deji Clinic Ltd.		19 Demurin Street, Catop Chicken George Ketu Lagos	Kosofe	Lagos

4077	LA/0251	LA/0251/S/5	Deji Clinic Ltd.		19 Demurin Street, Catop Chicken George Ketu Lagos	Kosofe	Lagos
4078	LA/0260	LA/0260/S/4	Lydab Pharm. & Stores	Pharmacy	No. 7 Dipo Awolasi Street, Majodo GRA, Ketu	Kosofe	Lagos
4079	LA/0261	LA/0261/S/4	Aflog Pharmacy	Pharmacy	No. 4 Agboyi Road Alopere-Ketu	Kosofe	Lagos
4080	LA/0264	LA/0264/S/4	Demsol Pharmacy Ltd.	Pharmacy	1 Moses Adebisi Str. Beside Total Oshogun Bus-stop, Alapere, Ketu	Kosofe	Lagos
4081	LA/0265	LA/0265/S/4	Med-In Specialist Hospital		1, Osugbo Street Ogudu,Kosofe	Kosofe	Lagos
4082	LA/0265	LA/0265/S/1	Med-In Specialist Hospital		1, Osugbo Street Ogudu,Kosofe	Kosofe	Lagos
4083	LA/0265	LA/0265/S/15	Med-In Specialist Hospital		1, Osugbo Street Ogudu,Kosofe	Kosofe	Lagos
4084	LA/0265	LA/0265/S/12	Med-In Specialist Hospital		1, Osugbo Street Ogudu,Kosofe	Kosofe	Lagos
4085	LA/0265	LA/0265/S/3	Med-In Specialist Hospital		1, Osugbo Street Ogudu,Kosofe	Kosofe	Lagos
4086	LA/0265	LA/0265/S/2	Med-In Specialist Hospital		1, Osugbo Street Ogudu,Kosofe	Kosofe	Lagos
4087	LA/0265	LA/0265/S/8	Med-In Specialist Hospital		1, Osugbo Street Ogudu,Kosofe	Kosofe	Lagos
4088	LA/0265	LA/0265/S/5	Med-In Specialist Hospital		1, Osugbo Street Ogudu,Kosofe	Kosofe	Lagos
4089	LA/0265	LA/0265/S/6	Med-In Specialist Hospital		1, Osugbo Street Ogudu,Kosofe	Kosofe	Lagos
4090	LA/0267	LA/0267/S/5	Avon Medical Services Limited		3rd Floor, West Wing City Hall, Catholic Mission Street, Lagos	Lagos Island	Lagos
4091	LA/0268	LA/0268/S/5	Longing Medical Centre		1 Josepha Close, Off Ogundeji Oguntona St, By Ajala Bus Stop, Ijaiye Ojokoro	Ifako-Ijaye	Lagos
4092	LA/0268	LA/0268/S/3	Longing Medical Centre		1 Josepha Close, Off Ogundeji Oguntona St, By Ajala Bus Stop, Ijaiye Ojokoro	Ifako-Ijaye	Lagos
4093	LA/0268	LA/0268/S/1	Longing Medical Centre		1 Josepha Close, Off Ogundeji Oguntona St, By Ajala Bus Stop, Ijaiye Ojokoro	Ifako-Ijaye	Lagos
4094	LA/0269	LA/0269/S/5	Good Faith Clinic		19, Ojo-Giwa Street, Off Idumagbo Avenue, Lagos.	Lagos Island	Lagos
4095	LA/0275	LA/0275/S/1	St. Paul's Hospital & Maternity Centre		71a Ondo Street (East), Ebute Metta	Lagos Mainland	Lagos
4096	LA/0277	LA/0277/S/1	Mount Sinai Hospital - Ebute Metta		177, Borno Way, Ebute Metta	Lagos Mainland	Lagos
4097	LA/0279	LA/0279/S/4	Federal Medical Centre - Ebute Metta		PMB 1097, Ebute-Metta	Lagos Mainland	Lagos
4098	LA/0279	LA/0279/S/5	Federal Medical Centre - Ebute Metta		PMB 1097, Ebute-Metta	Lagos Mainland	Lagos
4099	LA/0279	LA/0279/S/3	Federal Medical Centre - Ebute Metta		PMB 1097, Ebute-Metta	Lagos Mainland	Lagos
4100	LA/0279	LA/0279/S/1	Federal Medical Centre - Ebute Metta		PMB 1097, Ebute-Metta	Lagos Mainland	Lagos
4101	LA/0279	LA/0279/S/6	Federal Medical Centre - Ebute Metta		PMB 1097, Ebute-Metta	Lagos Mainland	Lagos
4102	LA/0279	LA/0279/S/8	Federal Medical Centre - Ebute Metta		PMB 1097, Ebute-Metta	Lagos Mainland	Lagos
4103	LA/0279	LA/0279/S/7	Federal Medical Centre - Ebute Metta		PMB 1097, Ebute-Metta	Lagos Mainland	Lagos
4104	LA/0279	LA/0279/S/15	Federal Medical Centre - Ebute Metta		PMB 1097, Ebute-Metta	Lagos Mainland	Lagos
4105	LA/0279	LA/0279/S/12	Federal Medical Centre - Ebute Metta		PMB 1097, Ebute-Metta	Lagos Mainland	Lagos
4106	LA/0279	LA/0279/S/2	Federal Medical Centre - Ebute Metta		PMB 1097, Ebute-Metta	Lagos Mainland	Lagos
4107	LA/0288	LA/0288/S/7	St. Luke's Hospital		18 Commercial Avenue, Sabo, Yaba, Lagos.	Lagos Mainland	Lagos
4108	LA/0288	LA/0288/S/4	St. Luke's Hospital		18 Commercial Avenue, Sabo, Yaba, Lagos.	Lagos Mainland	Lagos
4109	LA/0288	LA/0288/S/8	St. Luke's Hospital		18 Commercial Avenue, Sabo, Yaba, Lagos.	Lagos Mainland	Lagos
4110	LA/0288	LA/0288/S/5	St. Luke's Hospital		18 Commercial Avenue, Sabo, Yaba, Lagos.	Lagos Mainland	Lagos

4111	LA/0289	LA/0289/S/14	Psychiatric Hospital(Community/Staff Clinic)		Yaba, Lagos	Lagos Mainland	Lagos
4112	LA/0289	LA/0289/S/5	Psychiatric Hospital(Community/Staff Clinic)		Yaba, Lagos	Lagos Mainland	Lagos
4113	LA/0289	LA/0289/S/4	Psychiatric Hospital(Community/Staff Clinic)		Yaba, Lagos	Lagos Mainland	Lagos
4114	LA/0289	LA/0289/S/7	Psychiatric Hospital(Community/Staff Clinic)		Yaba, Lagos	Lagos Mainland	Lagos
4115	LA/0289	LA/0289/S/11	Psychiatric Hospital(Community/Staff Clinic)		Yaba, Lagos	Lagos Mainland	Lagos
4116	LA/0289	LA/0289/S/9	Psychiatric Hospital(Community/Staff Clinic)		Yaba, Lagos	Lagos Mainland	Lagos
4117	LA/0291	LA/0291/S/1	Marien Clinic	-	7, Montgomery Road, Yaba, Lagos	Lagos Mainland	Lagos
4118	LA/0291	LA/0291/S/2	Marien Clinic	-	7, Montgomery Road, Yaba, Lagos	Lagos Mainland	Lagos
4119	LA/0291	LA/0291/S/3	Marien Clinic	-	7, Montgomery Road, Yaba, Lagos	Lagos Mainland	Lagos
4120	LA/0291	LA/0291/S/5	Marien Clinic	-	7, Montgomery Road, Yaba, Lagos	Lagos Mainland	Lagos
4121	LA/0291	LA/0291/S/6	Marien Clinic	-	7, Montgomery Road, Yaba, Lagos	Lagos Mainland	Lagos
4122	LA/0292	LA/0292/S/4	Carrot-Top Drugs Ltd.	Pharmacy	59, Tapa Str. Opp. LSDPC Housing Estate Gate, Ebute Metta.	Lagos Mainland	Lagos
4123	LA/0294	LA/0294/S/5	Qualimax Diagnostic Laboratories	Laboratory	54, Pedro Road, Famous B/Stop Bariga Sabo, Yaba	Lagos Mainland	Lagos
4124	LA/0295	LA/0295/S/5	Keen Medical Diagnosis & Laboratory Services	Laboratory	No. 248, Borno Way, Off Adekunle str., Yaba Lagos	Lagos Mainland	Lagos
4125	LA/0296	LA/0296/S/5	Blessed-Gaye Madical Diag. centre	Laboratory	340, Borno Way, Alagomeji	Lagos Mainland	Lagos
4126	LA/0302	LA/0302/S/4	Raola Pharmacy & Stores	Pharmacy	27, Commercial Avenue, Yaba	Lagos Mainland	Lagos
4127	LA/0303	LA/0303/S/4	Okolowhite Nig. Ltd, Pharmacy & Stores	Pharmacy	41a Commercial Avenue, Yaba	Lagos Mainland	Lagos
4128	LA/0304	LA/0304/S/4	Josfred International Ltd.	Pharmacy	Suite 149, Mayoung Barracks, Yaba, Lagos.	Lagos Mainland	Lagos
4129	LA/0306	LA/0306/S/12	Ultima Medical Hospital		3. Cappa Avenue Palmgrove Estate Mushin Lagos	Mushin	Lagos
4130	LA/0306	LA/0306/S/5	Ultima Medical Hospital		3. Cappa Avenue Palmgrove Estate Mushin Lagos	Mushin	Lagos
4131	LA/0306	LA/0306/S/6	Ultima Medical Hospital		3. Cappa Avenue Palmgrove Estate Mushin Lagos	Mushin	Lagos
4132	LA/0306	LA/0306/S/3	Ultima Medical Hospital		3. Cappa Avenue Palmgrove Estate Mushin Lagos	Mushin	Lagos
4133	LA/0306	LA/0306/S/10	Ultima Medical Hospital		3. Cappa Avenue Palmgrove Estate Mushin Lagos	Mushin	Lagos
4134	LA/0306	LA/0306/S/2	Ultima Medical Hospital		3. Cappa Avenue Palmgrove Estate Mushin Lagos	Mushin	Lagos
4135	LA/0306	LA/0306/S/1	Ultima Medical Hospital		3. Cappa Avenue Palmgrove Estate Mushin Lagos	Mushin	Lagos
4136	LA/0309	LA/0309/S/2	St. Claire Hospital		44 Alh. Mush Street Papa Ajao Mushin Lagos	Mushin	Lagos
4137	LA/0311	LA/0311/S/5	Hillstar Clinics		98 Palm Avenue Papa Ajao, Mushin Lagos	Mushin	Lagos
4138	LA/0311	LA/0311/S/2	Hillstar Clinics		98 Palm Avenue Papa Ajao, Mushin Lagos	Mushin	Lagos
4139	LA/0311	LA/0311/S/3	Hillstar Clinics		98 Palm Avenue Papa Ajao, Mushin Lagos	Mushin	Lagos
4140	LA/0311	LA/0311/S/1	Hillstar Clinics		98 Palm Avenue Papa Ajao, Mushin Lagos	Mushin	Lagos
4141	LA/0313	LA/0313/S/3	May Clinics Limited		Doxology House, 24/26 Sadiku Street Ilasamaja, Off Isolo Express	Mushin	Lagos
4142	LA/0313	LA/0313/S/2	May Clinics Limited		Doxology House, 24/26 Sadiku Street Ilasamaja, Off Isolo Express	Mushin	Lagos
4143	LA/0313	LA/0313/S/7	May Clinics Limited		Doxology House, 24/26 Sadiku Street Ilasamaja, Off Isolo Express	Mushin	Lagos
4144	LA/0313	LA/0313/S/1	May Clinics Limited		Doxology House, 24/26 Sadiku Street Ilasamaja, Off Isolo Express	Mushin	Lagos
4145	LA/0313	LA/0313/S/6	May Clinics Limited		Doxology House, 24/26 Sadiku Street Ilasamaja, Off Isolo Express	Mushin	Lagos

4146	LA/0313	LA/0313/S/5	May Clinics Limited		Doxology House, 24/26 Sadiku Street Ilasamaja, Off Isolo Express	Mushin	Lagos
4147	LA/0314	LA/0314/S/1	Summit Hospital & Maternity Home		5 Summit Close, Off Mosalashi B/Stop	Mushin	Lagos
4148	LA/0314	LA/0314/S/3	Summit Hospital & Maternity Home		5 Summit Close, Off Mosalashi B/Stop	Mushin	Lagos
4149	LA/0327	LA/0327/S/4	Pharma-Flux Limited	Pharmacy	63, Ajose Str. Off Berlet Bus Stop Oshodi-Apapa Expresssway	Mushin	Lagos
4150	LA/0328	LA/0328/S/4	Firm Pharmacy & Stores	Pharmacy	12 Sadiku Street, Sadiku Bus Stop Ilasamaja	Mushin	Lagos
4151	LA/0329	LA/0329/S/7	Sanor Medical Services Ltd.	Laboratory, Radiology	77A Coker Road, Off Town Planning Way Ilupeju, Lagos	Somolu	Lagos
4152	LA/0329	LA/0329/S/5	Sanor Medical Services Ltd.	Laboratory, Radiology	77A Coker Road, Off Town Planning Way Ilupeju, Lagos	Somolu	Lagos
4153	LA/0332	LA/0332/S/3	Kiladejo Hospital & Specialist Clinics		15ppl Road Off Ppl Bus Stop Badagry Expressway Lagos	Ojo	Lagos
4154	LA/0332	LA/0332/S/2	Kiladejo Hospital & Specialist Clinics		15ppl Road Off Ppl Bus Stop Badagry Expressway Lagos	Ojo	Lagos
4155	LA/0332	LA/0332/S/1	Kiladejo Hospital & Specialist Clinics		15ppl Road Off Ppl Bus Stop Badagry Expressway Lagos	Ojo	Lagos
4156	LA/0340	LA/0340/S/4	Klemzy Pharmaceutical Ltd.	Pharmacy	50, Badagary Expressway	Ojo	Lagos
4157	LA/0341	LA/0341/S/4	Ultima Pharmacy	Pharmacy	57, Ojo igbede Road By Sowemimo B/stop, Off Alabi int''''l	Ojo	Lagos
4158	LA/0346	LA/0346/S/7	Kilad Merit Diagnostic Centre.	Radiology, Laboratory	Casidy B/Stop,Badagry Express Way, Okokomaiko	Ojo	Lagos
4159	LA/0346	LA/0346/S/5	Kilad Merit Diagnostic Centre.	Radiology, Laboratory	Casidy B/Stop,Badagry Express Way, Okokomaiko	Ojo	Lagos
4160	LA/0351	LA/0351/S/1	Dolu Hospital		7.Sunmola Abayomi Str. Motoluku Lagos	Oshodi/Isolo	Lagos
4161	LA/0351	LA/0351/S/3	Dolu Hospital		7.Sunmola Abayomi Str. Motoluku Lagos	Oshodi/Isolo	Lagos
4162	LA/0351	LA/0351/S/5	Dolu Hospital		7.Sunmola Abayomi Str. Motoluku Lagos	Oshodi/Isolo	Lagos
4163	LA/0354	LA/0354/S/2	Divine -Grace Medical Centre		3 Sobogun Rofa Street, Mafoloku-Oshodi	Oshodi/Isolo	Lagos
4164	LA/0357	LA/0357/S/6	Geo Medical Centre		12 Subairu Street, Ladipo Bus Stop, Sogunle	Oshodi/Isolo	Lagos
4165	LA/0357	LA/0357/S/2	Geo Medical Centre		12 Subairu Street, Ladipo Bus Stop, Sogunle	Oshodi/Isolo	Lagos
4166	LA/0357	LA/0357/S/3	Geo Medical Centre		12 Subairu Street, Ladipo Bus Stop, Sogunle	Oshodi/Isolo	Lagos
4167	LA/0358	LA/0358/S/3	One Life Hospital Limited		485 Agege Motor Road, Bolade, Oshodi	Oshodi/Isolo	Lagos
4168	LA/0358	LA/0358/S/7	One Life Hospital Limited		485 Agege Motor Road, Bolade, Oshodi	Oshodi/Isolo	Lagos
4169	LA/0358	LA/0358/S/8	One Life Hospital Limited		485 Agege Motor Road, Bolade, Oshodi	Oshodi/Isolo	Lagos
4170	LA/0358	LA/0358/S/15	One Life Hospital Limited		485 Agege Motor Road, Bolade, Oshodi	Oshodi/Isolo	Lagos
4171	LA/0358	LA/0358/S/2	One Life Hospital Limited		485 Agege Motor Road, Bolade, Oshodi	Oshodi/Isolo	Lagos
4172	LA/0358	LA/0358/S/1	One Life Hospital Limited		485 Agege Motor Road, Bolade, Oshodi	Oshodi/Isolo	Lagos
4173	LA/0358	LA/0358/S/6	One Life Hospital Limited		485 Agege Motor Road, Bolade, Oshodi	Oshodi/Isolo	Lagos
4174	LA/0358	LA/0358/S/5	One Life Hospital Limited		485 Agege Motor Road, Bolade, Oshodi	Oshodi/Isolo	Lagos
4175	LA/0359	LA/0359/S/8	St. Emmanuel Clinics & Hospital		2,Bola Ademuwon Street,Behind Aswani Market,Isolo	Oshodi/Isolo	Lagos
4176	LA/0359	LA/0359/S/3	St. Emmanuel Clinics & Hospital		2,Bola Ademuwon Street,Behind Aswani Market,Isolo	Oshodi/Isolo	Lagos
4177	LA/0359	LA/0359/S/5	St. Emmanuel Clinics & Hospital		2,Bola Ademuwon Street,Behind Aswani Market,Isolo	Oshodi/Isolo	Lagos
4178	LA/0359	LA/0359/S/6	St. Emmanuel Clinics & Hospital		2,Bola Ademuwon Street,Behind Aswani Market,Isolo	Oshodi/Isolo	Lagos
4179	LA/0359	LA/0359/S/7	St. Emmanuel Clinics & Hospital		2,Bola Ademuwon Street,Behind Aswani Market,Isolo	Oshodi/Isolo	Lagos
4180	LA/0359	LA/0359/S/1	St. Emmanuel Clinics & Hospital		2,Bola Ademuwon Street,Behind Aswani Market,Isolo	Oshodi/Isolo	Lagos
4181	LA/0359	LA/0359/S/4	St. Emmanuel Clinics & Hospital		2,Bola Ademuwon Street,Behind Aswani Market,Isolo	Oshodi/Isolo	Lagos

4182	LA/0359	LA/0359/S/11	St. Emmanuel Clinics & Hospital		2,Bola Ademuwon Street,Behind Aswani Market,Isolo	Oshodi/Isolo	Lagos
4183	LA/0363	LA/0363/S/5	Bose Specialist Hospital		7,Ilori Street, Ire-Akari Estate, Isolo	Oshodi/Isolo	Lagos
4184	LA/0365	LA/0365/S/3	Mount Pisgah Hospital Ltd.		140 Isolo Road, Egbe, Lagos State	Agege	Lagos
4185	LA/0365	LA/0365/S/1	Mount Pisgah Hospital Ltd.		140 Isolo Road, Egbe, Lagos State	Agege	Lagos
4186	LA/0368	LA/0368/S/6	Bissalam Hospital Complex		4, Modupe Shita Street, Off Liasu Road, Egbe	Oshodi/Isolo	Lagos
4187	LA/0368	LA/0368/S/8	Bissalam Hospital Complex		4, Modupe Shita Street, Off Liasu Road, Egbe	Oshodi/Isolo	Lagos
4188	LA/0368	LA/0368/S/2	Bissalam Hospital Complex		4, Modupe Shita Street, Off Liasu Road, Egbe	Oshodi/Isolo	Lagos
4189	LA/0368	LA/0368/S/5	Bissalam Hospital Complex		4, Modupe Shita Street, Off Liasu Road, Egbe	Oshodi/Isolo	Lagos
4190	LA/0368	LA/0368/S/1	Bissalam Hospital Complex		4, Modupe Shita Street, Off Liasu Road, Egbe	Oshodi/Isolo	Lagos
4191	LA/0368	LA/0368/S/3	Bissalam Hospital Complex		4, Modupe Shita Street, Off Liasu Road, Egbe	Oshodi/Isolo	Lagos
4192	LA/0370	LA/0370/S/2	General Hospital, Isolo	-	Isolo, Lagos	Oshodi/Isolo	Lagos
4193	LA/0370	LA/0370/S/4	General Hospital, Isolo	-	Isolo, Lagos	Oshodi/Isolo	Lagos
4194	LA/0370	LA/0370/S/8	General Hospital, Isolo	-	Isolo, Lagos	Oshodi/Isolo	Lagos
4195	LA/0370	LA/0370/S/3	General Hospital, Isolo	-	Isolo, Lagos	Oshodi/Isolo	Lagos
4196	LA/0370	LA/0370/S/1	General Hospital, Isolo	-	Isolo, Lagos	Oshodi/Isolo	Lagos
4197	LA/0374	LA/0374/S/4	CRI Mediclinic Lekki Vgc		Plot 232 Road 1 Vgc Lekki Epc Expressway Lagos	Ibeju/Lekki	Lagos
4198	LA/0374	LA/0374/S/5	CRI Mediclinic Lekki Vgc		Plot 232 Road 1 Vgc Lekki Epc Expressway Lagos	Ibeju/Lekki	Lagos
4199	LA/0374	LA/0374/S/14	CRI Mediclinic Lekki Vgc		Plot 232 Road 1 Vgc Lekki Epc Expressway Lagos	Ibeju/Lekki	Lagos
4200	LA/0374	LA/0374/S/7	CRI Mediclinic Lekki Vgc		Plot 232 Road 1 Vgc Lekki Epc Expressway Lagos	Ibeju/Lekki	Lagos
4201	LA/0376	LA/0376/S/5	Adonai Medical Centre		No. 36 Adekunle Kuye Street, Lagos.	Surulere	Lagos
4202	LA/0381	LA/0381/S/4	Hasmut Nig. Ltd Pharmacy	Pharmacy	Ikolun Shopping Centre, 27 Ijegun Road Ikotun	Oshodi/Isolo	Lagos
4203	LA/0382	LA/0382/S/4	Calson Pharmaceutical Stores Ltd.	Pharmacy	3, Oyegoke Str. Orile, Oshodi	Oshodi/Isolo	Lagos
4204	LA/0385	LA/0385/S/4	Prime Pharmaceuticals	Pharmacy	7, Ire Akari Estate Road Isolo Lagos	Oshodi/Isolo	Lagos
4205	LA/0387	LA/0387/S/4	Acorn Pharmacy & Stores	Pharmacy	55, Idimu Road, Chemist Bus Stop Ejibbo	Oshodi/Isolo	Lagos
4206	LA/0389	LA/0389/S/4	Goshen Konsult ltd	Pharmacy	122, Mushin Rd, Isolo	Oshodi/Isolo	Lagos
4207	LA/0390	LA/0390/S/4	Stella-Charlse Pharmacy	Pharmacy	45a Okota Rd	Oshodi/Isolo	Lagos
4208	LA/0391	LA/0391/S/4	Akugbe Pharmacy	Pharmacy	2, Olorunfemi kolawole Str. Okota-isolo	Oshodi/Isolo	Lagos
4209	LA/0394	LA/0394/S/5	Khan Specialist Hospital	Laboratory	33/35 Harisson Sholaya Avenue, Okota, Isolo	Oshodi/Isolo	Lagos
4210	LA/0402	LA/0402/S/3	Medol Clinic		54,Adaranyo Street,Famous B/Stop,(Opp Pedro Pri.Sch.)Shomolu	Somolu	Lagos
4211	LA/0403	LA/0403/S/7	General Hospital Gbagada		Gbagada	Somolu	Lagos
4212	LA/0403	LA/0403/S/3	General Hospital Gbagada		Gbagada	Somolu	Lagos
4213	LA/0403	LA/0403/S/15	General Hospital Gbagada		Gbagada	Somolu	Lagos
4214	LA/0403	LA/0403/S/8	General Hospital Gbagada		Gbagada	Somolu	Lagos
4215	LA/0403	LA/0403/S/4	General Hospital Gbagada		Gbagada	Somolu	Lagos
4216	LA/0403	LA/0403/S/5	General Hospital Gbagada		Gbagada	Somolu	Lagos
4217	LA/0403	LA/0403/S/2	General Hospital Gbagada		Gbagada	Somolu	Lagos
4218	LA/0403	LA/0403/S/1	General Hospital Gbagada		Gbagada	Somolu	Lagos
4219	LA/0403	LA/0403/S/6	General Hospital Gbagada		Gbagada	Somolu	Lagos
4220	LA/0403	LA/0403/S/12	General Hospital Gbagada		Gbagada	Somolu	Lagos
4221	LA/0409	LA/0409/S/4	Casann Pharm. Ltd.	Pharmacy	38, Oworo Road, Oworonsoki	Kosofe	Lagos
4222	LA/0410	LA/0410/S/5	Health Function Laboratory	Laboratory	8, Gbadebo Street, Ojota	Kosofe	Lagos
4223	LA/0415	LA/0415/S/5	Akoka Diagnostics Centre	Laboratory	63 St. Finbirr College Rd. Akoka	Lagos Mainland	Lagos
4224	LA/0416	LA/0416/S/8	Jon-dental Clinic	Dental	20, Shobande St., Akoka Yaba	Lagos Mainland	Lagos

4225	LA/0429	LA/0429/S/1	Olaniba Mem. Spec. Hospital		10a Olatunde Onimole Street, Surulere, Lagos	Surulere	Lagos
4226	LA/0429	LA/0429/S/3	Olaniba Mem. Spec. Hospital		10a Olatunde Onimole Street, Surulere, Lagos	Surulere	Lagos
4227	LA/0431	LA/0431/S/3	Isioma Hospital		28, Molusi Avenue Ikate, Surulere, Lagos	Surulere	Lagos
4228	LA/0435	LA/0435/S/8	Mercy Thomas Oredugba Medical Centre		8, Mogaji Street, Aguda, Surulere, Lagos	Surulere	Lagos
4229	LA/0438	LA/0438/S/5	St. Luke's Medical Centre		61 Teniola Street off Adeshina Street, by Airways Link Road, Ijeshatedo, Surulere	Surulere	Lagos
4230	LA/0438	LA/0438/S/1	St. Luke's Medical Centre		61 Teniola Street off Adeshina Street, by Airways Link Road, Ijeshatedo, Surulere	Surulere	Lagos
4231	LA/0438	LA/0438/S/3	St. Luke's Medical Centre		61 Teniola Street off Adeshina Street, by Airways Link Road, Ijeshatedo, Surulere	Surulere	Lagos
4232	LA/0444	LA/0444/S/3	Smith Medical Centre		3 Eniasoro Beyioku Street, Surulere	Surulere	Lagos
4233	LA/0447	LA/0447/S/1	Model Specialist Hospital		5 Tafawa Balewa Crescent Off Adeniran Ogunsanya	Surulere	Lagos
4234	LA/0447	LA/0447/S/3	Model Specialist Hospital		5 Tafawa Balewa Crescent Off Adeniran Ogunsanya	Surulere	Lagos
4235	LA/0447	LA/0447/S/14	Model Specialist Hospital		5 Tafawa Balewa Crescent Off Adeniran Ogunsanya	Surulere	Lagos
4236	LA/0447	LA/0447/S/10	Model Specialist Hospital		5 Tafawa Balewa Crescent Off Adeniran Ogunsanya	Surulere	Lagos
4237	LA/0448	LA/0448/S/5	First Dominican Hospital Ltd		27, Alhaji Masha Road, Surulere, Lagos.	Surulere	Lagos
4238	LA/0450	LA/0450/S/5	Mezonel Hospital		1 Adetola Street, Aguda-Surulere, Lagos.	Surulere	Lagos
4239	LA/0450	LA/0450/S/14	Mezonel Hospital		1 Adetola Street, Aguda-Surulere, Lagos.	Surulere	Lagos
4240	LA/0455	LA/0455/S/1	Robertson Med. Centre		1-5 James Robertson Street, Surulere, Lagos	Surulere	Lagos
4241	LA/0455	LA/0455/S/2	Robertson Med. Centre		1-5 James Robertson Street, Surulere, Lagos	Surulere	Lagos
4242	LA/0455	LA/0455/S/3	Robertson Med. Centre		1-5 James Robertson Street, Surulere, Lagos	Surulere	Lagos
4243	LA/0456	LA/0456/S/5	Uwemedimo Hospital Ltd		19 Market Street, Ijesha - Surulere, Lagos	Surulere	Lagos
4244	LA/0457	LA/0457/S/3	New day Specialist Clinic Ltd.,		2, Ladele Street, Off Nnobi Street, Ikate, Surulere, Lagos	Surulere	Lagos
4245	LA/0457	LA/0457/S/1	New day Specialist Clinic Ltd.,		2, Ladele Street, Off Nnobi Street, Ikate, Surulere, Lagos	Surulere	Lagos
4246	LA/0457	LA/0457/S/7	New day Specialist Clinic Ltd.,		2, Ladele Street, Off Nnobi Street, Ikate, Surulere, Lagos	Surulere	Lagos
4247	LA/0457	LA/0457/S/5	New day Specialist Clinic Ltd.,		2, Ladele Street, Off Nnobi Street, Ikate, Surulere, Lagos	Surulere	Lagos
4248	LA/0457	LA/0457/S/2	New day Specialist Clinic Ltd.,		2, Ladele Street, Off Nnobi Street, Ikate, Surulere, Lagos	Surulere	Lagos
4249	LA/0457	LA/0457/S/8	New day Specialist Clinic Ltd.,		2, Ladele Street, Off Nnobi Street, Ikate, Surulere, Lagos	Surulere	Lagos
4250	LA/0457	LA/0457/S/6	New day Specialist Clinic Ltd.,		2, Ladele Street, Off Nnobi Street, Ikate, Surulere, Lagos	Surulere	Lagos
4251	LA/0459	LA/0459/S/2	Remilekun Memorial Medical Centre		29A Itire Road, Surulere, Lagos	Surulere	Lagos
4252	LA/0460	LA/0460/S/2	Topaz Hospital		12, Adeniyi Street, By Municipal Library, Lagos	Surulere	Lagos
4253	LA/0460	LA/0460/S/5	Topaz Hospital		12, Adeniyi Street, By Municipal Library, Lagos	Surulere	Lagos
4254	LA/0460	LA/0460/S/1	Topaz Hospital		12, Adeniyi Street, By Municipal Library, Lagos	Surulere	Lagos
4255	LA/0463	LA/0463/S/4	Atoms Medical Laboratories Ltd.	Pharmacy	12 Abiona Close, Off Fololu Road Surulere	Surulere	Lagos
4256	LA/0465	LA/0465/S/3	Dr. J.I.T.O. Unokanjo	O & G	Surulere Lagos	Surulere	Lagos
4257	LA/0467	LA/0467/S/15	Merion Eye Clinic Ltd.	Ophthalmology	30. Banire street, off cole st. off Mabo st. surulere	Surulere	Lagos
4258	LA/0474	LA/0474/S/4	Ben-zyk Pharmacy	Pharmacy	31, Sanusi Str. Off Ogunlana Bus-stop	Surulere	Lagos
4259	LA/0475	LA/0475/S/4	Devine Care Pharmacy	Pharmacy	27, Akerele Str. Surulere	Surulere	Lagos
4260	LA/0478	LA/0478/S/4	Jenkins Pharmacy & Stores	Pharmacy	17, Ojuelegba Road, Surulere	Surulere	Lagos

4261	LA/0479	LA/0479/S/4	Claoric Pharm. & Stores	Pharmacy	76, Bode Thomas Street, Surulere	Surulere	Lagos
4262	LA/0481	LA/0481/S/4	Vita Chemists Ltd.	Pharmacy	75, Tejuosho Street, Surulere	Surulere	Lagos
4263	LA/0484	LA/0484/S/4	Udez Chemists Ltd.	Pharmacy	120 Ojuelegba Road, Surulere	Surulere	Lagos
4264	LA/0487	LA/0487/S/4	Providence Pharmacy.	Pharmacy	77 Itire Road, Surulere	Surulere	Lagos
4265	LA/0489	LA/0489/S/4	Lastdays Pharmacy	Pharmacy	15, Abiodun Wright Ave.,Ikate, Surulere	Surulere	Lagos
4266	LA/0492	LA/0492/S/3	Living Spring Hospital & Mat. Home		49, Alafia Avenue Off Afolabi Obe Street Ori-Oke B/Stop	Oshodi/Isolo	Lagos
4267	LA/0493	LA/0493/S/4	Chi Pharmaceutiacal Ltd.	Pharmacy	Lagos	Lagos Mainland	Lagos
4268	LA/0494	LA/0494/S/12	Phonics Hearing centre		286A, Corporation Drive, Dolphin Estate, Ikoyi.	Eti-Osa	Lagos
4269	LA/0502	LA/0502/S/5	BCL Clinic		10, MacDonald Road, Ikoyi, Lagos	Eti-Osa	Lagos
4270	LA/0502	LA/0502/S/4	BCL Clinic		10, MacDonald Road, Ikoyi, Lagos	Eti-Osa	Lagos
4271	LA/0510	LA/0510/S/5	445 Nigerian Airforce Hospital		Sam Ewang Air Force Base, Ikeja	Ikeja	Lagos
4272	LA/0510	LA/0510/S/8	445 Nigerian Airforce Hospital		Sam Ewang Air Force Base, Ikeja	Ikeja	Lagos
4273	LA/0510	LA/0510/S/15	445 Nigerian Airforce Hospital		Sam Ewang Air Force Base, Ikeja	Ikeja	Lagos
4274	LA/0510	LA/0510/S/2	445 Nigerian Airforce Hospital		Sam Ewang Air Force Base, Ikeja	Ikeja	Lagos
4275	LA/0510	LA/0510/S/3	445 Nigerian Airforce Hospital		Sam Ewang Air Force Base, Ikeja	Ikeja	Lagos
4276	LA/0510	LA/0510/S/10	445 Nigerian Airforce Hospital		Sam Ewang Air Force Base, Ikeja	Ikeja	Lagos
4277	LA/0510	LA/0510/S/1	445 Nigerian Airforce Hospital		Sam Ewang Air Force Base, Ikeja	Ikeja	Lagos
4278	LA/0510	LA/0510/S/11	445 Nigerian Airforce Hospital		Sam Ewang Air Force Base, Ikeja	Ikeja	Lagos
4279	LA/0510	LA/0510/S/12	445 Nigerian Airforce Hospital		Sam Ewang Air Force Base, Ikeja	Ikeja	Lagos
4280	LA/0510	LA/0510/S/7	445 Nigerian Airforce Hospital		Sam Ewang Air Force Base, Ikeja	Ikeja	Lagos
4281	LA/0510	LA/0510/S/4	445 Nigerian Airforce Hospital		Sam Ewang Air Force Base, Ikeja	Ikeja	Lagos
4282	LA/0510	LA/0510/S/6	445 Nigerian Airforce Hospital		Sam Ewang Air Force Base, Ikeja	Ikeja	Lagos
4283	LA/0524	LA/0524/S/4	Lagoon Clinic		11A, Idejo Street, V/Island, Lagos	Eti-Osa	Lagos
4284	LA/0524	LA/0524/S/5	Lagoon Clinic		11A, Idejo Street, V/Island, Lagos	Eti-Osa	Lagos
4285	LA/0526	LA/0526/S/5	Lagoon Hospital, Ikeja		97/101 Obafemi Awolowo Way, Ikeja.	Ikeja	Lagos
4286	LA/0526	LA/0526/S/4	Lagoon Hospital, Ikeja		97/101 Obafemi Awolowo Way, Ikeja.	Ikeja	Lagos
4287	LA/0526	LA/0526/S/7	Lagoon Hospital, Ikeja		97/101 Obafemi Awolowo Way, Ikeja.	Ikeja	Lagos
4288	LA/0529	LA/0529/S/4	Kupa Medical Centre Ltd		4, Lateef Salami Street, Off M/M In''''tl Airport Rd. Ajao Estate, Lagos.	Oshodi/Isolo	Lagos
4289	LA/0535	LA/0535/S/7	Osuntuyi Medical Centre, Obanikoro		6, Alh. Salisu Street, Obanikoro, Lagos.	Mushin	Lagos
4290	LA/0535	LA/0535/S/2	Osuntuyi Medical Centre, Obanikoro		6, Alh. Salisu Street, Obanikoro, Lagos.	Mushin	Lagos
4291	LA/0535	LA/0535/S/4	Osuntuyi Medical Centre, Obanikoro		6, Alh. Salisu Street, Obanikoro, Lagos.	Mushin	Lagos
4292	LA/0535	LA/0535/S/1	Osuntuyi Medical Centre, Obanikoro		6, Alh. Salisu Street, Obanikoro, Lagos.	Mushin	Lagos
4293	LA/0535	LA/0535/S/5	Osuntuyi Medical Centre, Obanikoro		6, Alh. Salisu Street, Obanikoro, Lagos.	Mushin	Lagos
4294	LA/0535	LA/0535/S/3	Osuntuyi Medical Centre, Obanikoro		6, Alh. Salisu Street, Obanikoro, Lagos.	Mushin	Lagos
4295	LA/0539	LA/0539/S/3	Crystal Specialist Hospital		148, Akowonjo Road, Dopemu, Lagos.	Agege	Lagos
4296	LA/0539	LA/0539/S/4	Crystal Specialist Hospital		148, Akowonjo Road, Dopemu, Lagos.	Agege	Lagos
4297	LA/0539	LA/0539/S/7	Crystal Specialist Hospital		148, Akowonjo Road, Dopemu, Lagos.	Agege	Lagos
4298	LA/0539	LA/0539/S/5	Crystal Specialist Hospital		148, Akowonjo Road, Dopemu, Lagos.	Agege	Lagos
4299	LA/0541	LA/0541/S/7	Faith City Hospital, Ajao -estate		16, Asa-Afariogun Street, Off Osolo Way, Ajao Estate, Lagos.	Oshodi/Isolo	Lagos
4300	LA/0541	LA/0541/S/5	Faith City Hospital, Ajao -estate		16, Asa-Afariogun Street, Off Osolo Way, Ajao Estate, Lagos.	Oshodi/Isolo	Lagos
4301	LA/0543	LA/0543/S/5	Faith City Hospital, V/I		2B, Oko-Awo Close, V/Island, Lagos	Eti-Osa	Lagos
4302	LA/0543	LA/0543/S/7	Faith City Hospital, V/I		2B, Oko-Awo Close, V/Island, Lagos	Eti-Osa	Lagos

4303	LA/0545	LA/0545/S/10	R-Jolad Hospital		1, Akinyede Street, By New Garage, Bariga, Lagos.	Kosofe	Lagos
4304	LA/0545	LA/0545/S/2	R-Jolad Hospital		1, Akinyede Street, By New Garage, Bariga, Lagos.	Kosofe	Lagos
4305	LA/0545	LA/0545/S/8	R-Jolad Hospital		1, Akinyede Street, By New Garage, Bariga, Lagos.	Kosofe	Lagos
4306	LA/0545	LA/0545/S/6	R-Jolad Hospital		1, Akinyede Street, By New Garage, Bariga, Lagos.	Kosofe	Lagos
4307	LA/0545	LA/0545/S/12	R-Jolad Hospital		1, Akinyede Street, By New Garage, Bariga, Lagos.	Kosofe	Lagos
4308	LA/0545	LA/0545/S/11	R-Jolad Hospital		1, Akinyede Street, By New Garage, Bariga, Lagos.	Kosofe	Lagos
4309	LA/0545	LA/0545/S/3	R-Jolad Hospital		1, Akinyede Street, By New Garage, Bariga, Lagos.	Kosofe	Lagos
4310	LA/0545	LA/0545/S/7	R-Jolad Hospital		1, Akinyede Street, By New Garage, Bariga, Lagos.	Kosofe	Lagos
4311	LA/0545	LA/0545/S/5	R-Jolad Hospital		1, Akinyede Street, By New Garage, Bariga, Lagos.	Kosofe	Lagos
4312	LA/0545	LA/0545/S/1	R-Jolad Hospital		1, Akinyede Street, By New Garage, Bariga, Lagos.	Kosofe	Lagos
4313	LA/0545	LA/0545/S/4	R-Jolad Hospital		1, Akinyede Street, By New Garage, Bariga, Lagos.	Kosofe	Lagos
4314	LA/0549	LA/0549/S/1	Heals Specialist Hospital		40B, Godwin Omonua Street, Ireakari Estate.	Oshodi/Isolo	Lagos
4315	LA/0552	LA/0552/S/2	Healing Cross Hospital Ltd		34, Apena Street, Okota Isolo, Lagos	Oshodi/Isolo	Lagos
4316	LA/0552	LA/0552/S/3	Healing Cross Hospital Ltd		34, Apena Street, Okota Isolo, Lagos	Oshodi/Isolo	Lagos
4317	LA/0554	LA/0554/S/5	Krown Hospital & Maternity Centre		11, Alhaji Sekoni Street, Off Alimosho Rd, Iyana-Ipaja, Lagos.	Alimosho	Lagos
4318	LA/0554	LA/0554/S/12	Krown Hospital & Maternity Centre		11, Alhaji Sekoni Street, Off Alimosho Rd, Iyana-Ipaja, Lagos.	Alimosho	Lagos
4319	LA/0554	LA/0554/S/6	Krown Hospital & Maternity Centre		11, Alhaji Sekoni Street, Off Alimosho Rd, Iyana-Ipaja, Lagos.	Alimosho	Lagos
4320	LA/0554	LA/0554/S/3	Krown Hospital & Maternity Centre		11, Alhaji Sekoni Street, Off Alimosho Rd, Iyana-Ipaja, Lagos.	Alimosho	Lagos
4321	LA/0554	LA/0554/S/10	Krown Hospital & Maternity Centre		11, Alhaji Sekoni Street, Off Alimosho Rd, Iyana-Ipaja, Lagos.	Alimosho	Lagos
4322	LA/0554	LA/0554/S/7	Krown Hospital & Maternity Centre		11, Alhaji Sekoni Street, Off Alimosho Rd, Iyana-Ipaja, Lagos.	Alimosho	Lagos
4323	LA/0554	LA/0554/S/1	Krown Hospital & Maternity Centre		11, Alhaji Sekoni Street, Off Alimosho Rd, Iyana-Ipaja, Lagos.	Alimosho	Lagos
4324	LA/0557	LA/0557/S/2	Legus Specialist Hospital		34 Akorohunfayo Street, Ikorodu Rd., Igbobi, Lagos	Mushin	Lagos
4325	LA/0557	LA/0557/S/1	Legus Specialist Hospital		34 Akorohunfayo Street, Ikorodu Rd., Igbobi, Lagos	Mushin	Lagos
4326	LA/0557	LA/0557/S/10	Legus Specialist Hospital		34 Akorohunfayo Street, Ikorodu Rd., Igbobi, Lagos	Mushin	Lagos
4327	LA/0557	LA/0557/S/5	Legus Specialist Hospital		34 Akorohunfayo Street, Ikorodu Rd., Igbobi, Lagos	Mushin	Lagos
4328	LA/0557	LA/0557/S/11	Legus Specialist Hospital		34 Akorohunfayo Street, Ikorodu Rd., Igbobi, Lagos	Mushin	Lagos
4329	LA/0557	LA/0557/S/3	Legus Specialist Hospital		34 Akorohunfayo Street, Ikorodu Rd., Igbobi, Lagos	Mushin	Lagos
4330	LA/0557	LA/0557/S/6	Legus Specialist Hospital		34 Akorohunfayo Street, Ikorodu Rd., Igbobi, Lagos	Mushin	Lagos
4331	LA/0561	LA/0561/S/7	Lagos Univ. Teach. Hosp.		Idiaraba, Lagos	Mushin	Lagos
4332	LA/0561	LA/0561/S/3	Lagos Univ. Teach. Hosp.		Idiaraba, Lagos	Mushin	Lagos
4333	LA/0561	LA/0561/S/8	Lagos Univ. Teach. Hosp.		Idiaraba, Lagos	Mushin	Lagos
4334	LA/0561	LA/0561/S/6	Lagos Univ. Teach. Hosp.		Idiaraba, Lagos	Mushin	Lagos

4335	LA/0561	LA/0561/S/15	Lagos Univ. Teach. Hosp.		Idiaraba, Lagos	Mushin	Lagos
4336	LA/0561	LA/0561/S/4	Lagos Univ. Teach. Hosp.		Idiaraba, Lagos	Mushin	Lagos
4337	LA/0561	LA/0561/S/2	Lagos Univ. Teach. Hosp.		Idiaraba, Lagos	Mushin	Lagos
4338	LA/0561	LA/0561/S/12	Lagos Univ. Teach. Hosp.		Idiaraba, Lagos	Mushin	Lagos
4339	LA/0561	LA/0561/S/10	Lagos Univ. Teach. Hosp.		Idiaraba, Lagos	Mushin	Lagos
4340	LA/0561	LA/0561/S/5	Lagos Univ. Teach. Hosp.		Idiaraba, Lagos	Mushin	Lagos
4341	LA/0561	LA/0561/S/1	Lagos Univ. Teach. Hosp.		Idiaraba, Lagos	Mushin	Lagos
4342	LA/0561	LA/0561/S/11	Lagos Univ. Teach. Hosp.		Idiaraba, Lagos	Mushin	Lagos
4343	LA/0562	LA/0562/S/1	Lagos Island Maternity Hosp.		Broad Street, Lagos	Eti-Osa	Lagos
4344	LA/0562	LA/0562/S/4	Lagos Island Maternity Hosp.		Broad Street, Lagos	Eti-Osa	Lagos
4345	LA/0562	LA/0562/S/3	Lagos Island Maternity Hosp.		Broad Street, Lagos	Eti-Osa	Lagos
4346	LA/0562	LA/0562/S/6	Lagos Island Maternity Hosp.		Broad Street, Lagos	Eti-Osa	Lagos
4347	LA/0562	LA/0562/S/5	Lagos Island Maternity Hosp.		Broad Street, Lagos	Eti-Osa	Lagos
4348	LA/0563	LA/0563/S/3	Lagos State University Teach. Hospital		Ikeja, Lagos State	Ikeja	Lagos
4349	LA/0563	LA/0563/S/5	Lagos State University Teach. Hospital		Ikeja, Lagos State	Ikeja	Lagos
4350	LA/0563	LA/0563/S/15	Lagos State University Teach. Hospital		Ikeja, Lagos State	Ikeja	Lagos
4351	LA/0563	LA/0563/S/9	Lagos State University Teach. Hospital		Ikeja, Lagos State	Ikeja	Lagos
4352	LA/0563	LA/0563/S/12	Lagos State University Teach. Hospital		Ikeja, Lagos State	Ikeja	Lagos
4353	LA/0563	LA/0563/S/8	Lagos State University Teach. Hospital		Ikeja, Lagos State	Ikeja	Lagos
4354	LA/0563	LA/0563/S/7	Lagos State University Teach. Hospital		Ikeja, Lagos State	Ikeja	Lagos
4355	LA/0563	LA/0563/S/2	Lagos State University Teach. Hospital		Ikeja, Lagos State	Ikeja	Lagos
4356	LA/0563	LA/0563/S/6	Lagos State University Teach. Hospital		Ikeja, Lagos State	Ikeja	Lagos
4357	LA/0563	LA/0563/S/11	Lagos State University Teach. Hospital		Ikeja, Lagos State	Ikeja	Lagos
4358	LA/0563	LA/0563/S/4	Lagos State University Teach. Hospital		Ikeja, Lagos State	Ikeja	Lagos
4359	LA/0563	LA/0563/S/1	Lagos State University Teach. Hospital		Ikeja, Lagos State	Ikeja	Lagos
4360	LA/0564	LA/0564/S/4	Lagos State General Hospital		Broad Street, Lagos.	Lagos Island	Lagos
4361	LA/0564	LA/0564/S/12	Lagos State General Hospital		Broad Street, Lagos.	Lagos Island	Lagos
4362	LA/0564	LA/0564/S/7	Lagos State General Hospital		Broad Street, Lagos.	Lagos Island	Lagos
4363	LA/0564	LA/0564/S/1	Lagos State General Hospital		Broad Street, Lagos.	Lagos Island	Lagos
4364	LA/0564	LA/0564/S/8	Lagos State General Hospital		Broad Street, Lagos.	Lagos Island	Lagos
4365	LA/0564	LA/0564/S/5	Lagos State General Hospital		Broad Street, Lagos.	Lagos Island	Lagos
4366	LA/0565	LA/0565/S/4	Health Plus	Pharmacy	21, Joel Ogunnaike Street, Off Mobolaji Bank, Ikeja, Lagos.	Ikeja	Lagos
4367	LA/0566	LA/0566/S/1	Elegbeleye Specialist Hospital		1, Elegbeleye Street, Ikosi-Ketu, Lagos	Kosofe	Lagos
4368	LA/0569	LA/0569/S/3	Alheri Hospital		10, Ojodu Abiodun Road, Off Kosoko Road, Berger B/Stop, Ojodu, Lagos	Ikeja	Lagos
4369	LA/0569	LA/0569/S/1	Alheri Hospital		10, Ojodu Abiodun Road, Off Kosoko Road, Berger B/Stop, Ojodu, Lagos	Ikeja	Lagos
4370	LA/0586	LA/0586/S/5	University of Lagos Health Centre		Yaba, Lagos State	Lagos Mainland	Lagos
4371	LA/0586	LA/0586/S/4	University of Lagos Health Centre		Yaba, Lagos State	Lagos Mainland	Lagos

4372	LA/0588	LA/0588/S/3	Police Hospital, Falomo		Ikoyi, Lagos	Eti-Osa	Lagos
4373	LA/0588	LA/0588/S/4	Police Hospital, Falomo		Ikoyi, Lagos	Eti-Osa	Lagos
4374	LA/0588	LA/0588/S/5	Police Hospital, Falomo		Ikoyi, Lagos	Eti-Osa	Lagos
4375	LA/0588	LA/0588/S/8	Police Hospital, Falomo		Ikoyi, Lagos	Eti-Osa	Lagos
4376	LA/0588	LA/0588/S/13	Police Hospital, Falomo		Ikoyi, Lagos	Eti-Osa	Lagos
4377	LA/0588	LA/0588/S/15	Police Hospital, Falomo		Ikoyi, Lagos	Eti-Osa	Lagos
4378	LA/0588	LA/0588/S/2	Police Hospital, Falomo		Ikoyi, Lagos	Eti-Osa	Lagos
4379	LA/0588	LA/0588/S/1	Police Hospital, Falomo		Ikoyi, Lagos	Eti-Osa	Lagos
4380	LA/0588	LA/0588/S/7	Police Hospital, Falomo		Ikoyi, Lagos	Eti-Osa	Lagos
4381	LA/0589	LA/0589/S/3	Cottage Hospital/Comprehensive Health Centre		Police College, Ikeja, Lagos State	Ikeja	Lagos
4382	LA/0589	LA/0589/S/15	Cottage Hospital/Comprehensive Health Centre		Police College, Ikeja, Lagos State	Ikeja	Lagos
4383	LA/0589	LA/0589/S/4	Cottage Hospital/Comprehensive Health Centre		Police College, Ikeja, Lagos State	Ikeja	Lagos
4384	LA/0589	LA/0589/S/7	Cottage Hospital/Comprehensive Health Centre		Police College, Ikeja, Lagos State	Ikeja	Lagos
4385	LA/0589	LA/0589/S/1	Cottage Hospital/Comprehensive Health Centre		Police College, Ikeja, Lagos State	Ikeja	Lagos
4386	LA/0589	LA/0589/S/11	Cottage Hospital/Comprehensive Health Centre		Police College, Ikeja, Lagos State	Ikeja	Lagos
4387	LA/0589	LA/0589/S/8	Cottage Hospital/Comprehensive Health Centre		Police College, Ikeja, Lagos State	Ikeja	Lagos
4388	LA/0589	LA/0589/S/5	Cottage Hospital/Comprehensive Health Centre		Police College, Ikeja, Lagos State	Ikeja	Lagos
4389	LA/0591	LA/0591/S/15	Nigerian Navy Reference Hospital		Navy Town, Ojo, Lagos	Ojo	Lagos
4390	LA/0591	LA/0591/S/7	Nigerian Navy Reference Hospital		Navy Town, Ojo, Lagos	Ojo	Lagos
4391	LA/0591	LA/0591/S/3	Nigerian Navy Reference Hospital		Navy Town, Ojo, Lagos	Ojo	Lagos
4392	LA/0591	LA/0591/S/5	Nigerian Navy Reference Hospital		Navy Town, Ojo, Lagos	Ojo	Lagos
4393	LA/0591	LA/0591/S/8	Nigerian Navy Reference Hospital		Navy Town, Ojo, Lagos	Ojo	Lagos
4394	LA/0591	LA/0591/S/12	Nigerian Navy Reference Hospital		Navy Town, Ojo, Lagos	Ojo	Lagos
4395	LA/0591	LA/0591/S/11	Nigerian Navy Reference Hospital		Navy Town, Ojo, Lagos	Ojo	Lagos
4396	LA/0591	LA/0591/S/1	Nigerian Navy Reference Hospital		Navy Town, Ojo, Lagos	Ojo	Lagos
4397	LA/0591	LA/0591/S/4	Nigerian Navy Reference Hospital		Navy Town, Ojo, Lagos	Ojo	Lagos
4398	LA/0591	LA/0591/S/6	Nigerian Navy Reference Hospital		Navy Town, Ojo, Lagos	Ojo	Lagos
4399	LA/0591	LA/0591/S/2	Nigerian Navy Reference Hospital		Navy Town, Ojo, Lagos	Ojo	Lagos
4400	LA/0593	LA/0593/S/14	Obisessan Navy Medical Centre		Mobil Road, Apapa, Lagos	Apapa	Lagos
4401	LA/0593	LA/0593/S/3	Obisessan Navy Medical Centre		Mobil Road, Apapa, Lagos	Apapa	Lagos
4402	LA/0593	LA/0593/S/2	Obisessan Navy Medical Centre		Mobil Road, Apapa, Lagos	Apapa	Lagos
4403	LA/0593	LA/0593/S/1	Obisessan Navy Medical Centre		Mobil Road, Apapa, Lagos	Apapa	Lagos
4404	LA/0593	LA/0593/S/13	Obisessan Navy Medical Centre		Mobil Road, Apapa, Lagos	Apapa	Lagos
4405	LA/0593	LA/0593/S/7	Obisessan Navy Medical Centre		Mobil Road, Apapa, Lagos	Apapa	Lagos
4406	LA/0593	LA/0593/S/5	Obisessan Navy Medical Centre		Mobil Road, Apapa, Lagos	Apapa	Lagos
4407	LA/0593	LA/0593/S/4	Obisessan Navy Medical Centre		Mobil Road, Apapa, Lagos	Apapa	Lagos
4408	LA/0599	LA/0599/S/9	Ladi-lak Medical Centre	Primary	No 53 IGY Ologbin street, Ladi-lak B/Stop, Bariga, Lagos	Somolu	Lagos
4409	LA/0599	LA/0599/S/7	Ladi-lak Medical Centre	Primary	No 53 IGY Ologbin street, Ladi-lak B/Stop, Bariga, Lagos	Somolu	Lagos
4410	LA/0599	LA/0599/S/14	Ladi-lak Medical Centre	Primary	No 53 IGY Ologbin street, Ladi-lak B/Stop, Bariga, Lagos	Somolu	Lagos

4411	LA/0599	LA/0599/S/6	Ladi-lak Medical Centre	Primary	No 53 IGY Ologbin street, Ladi-lak B/Stop, Bariga, Lagos	Somolu	Lagos
4412	LA/0599	LA/0599/S/1	Ladi-lak Medical Centre	Primary	No 53 IGY Ologbin street, Ladi-lak B/Stop, Bariga, Lagos	Somolu	Lagos
4413	LA/0599	LA/0599/S/5	Ladi-lak Medical Centre	Primary	No 53 IGY Ologbin street, Ladi-lak B/Stop, Bariga, Lagos	Somolu	Lagos
4414	LA/0601	LA/0601/S/5	Samaria Hospital		No 17 Dabo Bashorun street off Ali Dada Street, Okota, Lagos	Oshodi/Isolo	Lagos
4415	LA/0601	LA/0601/S/7	Samaria Hospital		No 17 Dabo Bashorun street off Ali Dada Street, Okota, Lagos	Oshodi/Isolo	Lagos
4416	LA/0601	LA/0601/S/4	Samaria Hospital		No 17 Dabo Bashorun street off Ali Dada Street, Okota, Lagos	Oshodi/Isolo	Lagos
4417	LA/0601	LA/0601/S/10	Samaria Hospital		No 17 Dabo Bashorun street off Ali Dada Street, Okota, Lagos	Oshodi/Isolo	Lagos
4418	LA/0602	LA/0602/S/5	Watershed Hospital	Primary	No 3 Dele Orisabiyi street off Ago palace way, Okota, Lagos	Oshodi/Isolo	Lagos
4419	LA/0602	LA/0602/S/3	Watershed Hospital	Primary	No 3 Dele Orisabiyi street off Ago palace way, Okota, Lagos	Oshodi/Isolo	Lagos
4420	LA/0604	LA/0604/S/3	St Annes Infirmary (Queens Clinic)	Primary	5A Dauda Lane, Off Moshood Abiola Way, Ebute Metta	Lagos Mainland	Lagos
4421	LA/0606	LA/0606/S/1	Vones Medical Centre	Primary	9 Barikisu St Onike Yaba	Lagos Mainland	Lagos
4422	LA/0606	LA/0606/S/3	Vones Medical Centre	Primary	9 Barikisu St Onike Yaba	Lagos Mainland	Lagos
4423	LA/0608	LA/0608/S/5	Springtime Medical Centre	Primary Provider	22, Olusesi Str., Opp. Chemron 2nd Gate by the Conservation Centre, Lekki Pennisula	Eti-Osa	Lagos
4424	LA/0608	LA/0608/S/1	Springtime Medical Centre	Primary Provider	22, Olusesi Str., Opp. Chemron 2nd Gate by the Conservation Centre, Lekki Pennisula	Eti-Osa	Lagos
4425	LA/0608	LA/0608/S/3	Springtime Medical Centre	Primary Provider	22, Olusesi Str., Opp. Chemron 2nd Gate by the Conservation Centre, Lekki Pennisula	Eti-Osa	Lagos
4426	LA/0621	LA/0621/S/1	Divine Medical Centre		16, Akanbi Danmole Street, off Ribadu Rd., SW., Ikoyi, Lagos	Eti-Osa	Lagos
4427	LA/0621	LA/0621/S/3	Divine Medical Centre		16, Akanbi Danmole Street, off Ribadu Rd., SW., Ikoyi, Lagos	Eti-Osa	Lagos
4428	LA/0622	LA/0622/S/5	Barnes Hospital		Plot 34B Oniru Road, Dideolu Estate, Victoria Island	Eti-Osa	Lagos
4429	LA/0622	LA/0622/S/7	Barnes Hospital		Plot 34B Oniru Road, Dideolu Estate, Victoria Island	Eti-Osa	Lagos
4430	LA/0622	LA/0622/S/2	Barnes Hospital		Plot 34B Oniru Road, Dideolu Estate, Victoria Island	Eti-Osa	Lagos
4431	LA/0629	LA/0629/S/14	Apex-Care Hospital	Primary	42 Kujore St Ojota	Kosofe	Lagos
4432	LA/0629	LA/0629/S/3	Apex-Care Hospital	Primary	42 Kujore St Ojota	Kosofe	Lagos
4433	LA/0629	LA/0629/S/10	Apex-Care Hospital	Primary	42 Kujore St Ojota	Kosofe	Lagos
4434	LA/0629	LA/0629/S/1	Apex-Care Hospital	Primary	42 Kujore St Ojota	Kosofe	Lagos
4435	LA/0629	LA/0629/S/7	Apex-Care Hospital	Primary	42 Kujore St Ojota	Kosofe	Lagos
4436	LA/0630	LA/0630/S/5	Ameso Specialist Hospital	Primary	7 Jumat Olukoya Street Off Ogudu Rd Ojota	Kosofe	Lagos
4437	LA/0630	LA/0630/S/3	Ameso Specialist Hospital	Primary	7 Jumat Olukoya Street Off Ogudu Rd Ojota	Kosofe	Lagos
4438	LA/0630	LA/0630/S/2	Ameso Specialist Hospital	Primary	7 Jumat Olukoya Street Off Ogudu Rd Ojota	Kosofe	Lagos
4439	LA/0632	LA/0632/S/5	Tmac Specialist Hospital		14, Berkeley Street, off King George Street, Onikan, Lagos Island	Lagos Island	Lagos
4440	LA/0632	LA/0632/S/3	Tmac Specialist Hospital		14, Berkeley Street, off King George Street, Onikan, Lagos Island	Lagos Island	Lagos
4441	LA/0636	LA/0636/S/3	Geo-marie Hospital		35, AdePegbu Street, Abule-Egba, Lagos	Agege	Lagos
4442	LA/0639	LA/0639/S/1	Donas Hospital and Maternity	Primary	67 Owulade Avenue Irawo Bus/Stop Owode	Kosofe	Lagos
4443	LA/0639	LA/0639/S/3	Donas Hospital and Maternity	Primary	67 Owulade Avenue Irawo Bus/Stop Owode	Kosofe	Lagos
4444	LA/0639	LA/0639/S/2	Donas Hospital and Maternity	Primary	67 Owulade Avenue Irawo Bus/Stop Owode	Kosofe	Lagos
4445	LA/0639	LA/0639/S/15	Donas Hospital and Maternity	Primary	67 Owulade Avenue Irawo Bus/Stop Owode	Kosofe	Lagos

4446	LA/0643	LA/0643/S/7	Ayodele Medical Centre		23/25, Jonathan Coker Rd, Off Fagba Junction, Iju Agege	Agege	Lagos
4447	LA/0643	LA/0643/S/1	Ayodele Medical Centre		23/25, Jonathan Coker Rd, Off Fagba Junction, Iju Agege	Agege	Lagos
4448	LA/0643	LA/0643/S/4	Ayodele Medical Centre		23/25, Jonathan Coker Rd, Off Fagba Junction, Iju Agege	Agege	Lagos
4449	LA/0643	LA/0643/S/6	Ayodele Medical Centre		23/25, Jonathan Coker Rd, Off Fagba Junction, Iju Agege	Agege	Lagos
4450	LA/0643	LA/0643/S/10	Ayodele Medical Centre		23/25, Jonathan Coker Rd, Off Fagba Junction, Iju Agege	Agege	Lagos
4451	LA/0643	LA/0643/S/15	Ayodele Medical Centre		23/25, Jonathan Coker Rd, Off Fagba Junction, Iju Agege	Agege	Lagos
4452	LA/0643	LA/0643/S/3	Ayodele Medical Centre		23/25, Jonathan Coker Rd, Off Fagba Junction, Iju Agege	Agege	Lagos
4453	LA/0643	LA/0643/S/5	Ayodele Medical Centre		23/25, Jonathan Coker Rd, Off Fagba Junction, Iju Agege	Agege	Lagos
4454	LA/0643	LA/0643/S/2	Ayodele Medical Centre		23/25, Jonathan Coker Rd, Off Fagba Junction, Iju Agege	Agege	Lagos
4455	LA/0643	LA/0643/S/12	Ayodele Medical Centre		23/25, Jonathan Coker Rd, Off Fagba Junction, Iju Agege	Agege	Lagos
4456	LA/0643	LA/0643/S/8	Ayodele Medical Centre		23/25, Jonathan Coker Rd, Off Fagba Junction, Iju Agege	Agege	Lagos
4457	LA/0644	LA/0644/S/4	First Line Hospital	Primary Health Care	2nd Avenue House 14 By 207 Road Junction, Festac Town	Amuwo-Odofin	Lagos
4458	LA/0644	LA/0644/S/3	First Line Hospital	Primary Health Care	2nd Avenue House 14 By 207 Road Junction, Festac Town	Amuwo-Odofin	Lagos
4459	LA/0644	LA/0644/S/6	First Line Hospital	Primary Health Care	2nd Avenue House 14 By 207 Road Junction, Festac Town	Amuwo-Odofin	Lagos
4460	LA/0644	LA/0644/S/7	First Line Hospital	Primary Health Care	2nd Avenue House 14 By 207 Road Junction, Festac Town	Amuwo-Odofin	Lagos
4461	LA/0644	LA/0644/S/12	First Line Hospital	Primary Health Care	2nd Avenue House 14 By 207 Road Junction, Festac Town	Amuwo-Odofin	Lagos
4462	LA/0644	LA/0644/S/2	First Line Hospital	Primary Health Care	2nd Avenue House 14 By 207 Road Junction, Festac Town	Amuwo-Odofin	Lagos
4463	LA/0644	LA/0644/S/1	First Line Hospital	Primary Health Care	2nd Avenue House 14 By 207 Road Junction, Festac Town	Amuwo-Odofin	Lagos
4464	LA/0646	LA/0646/S/4	Ancilla Catholic Hospital		3, Muyiwa Olojo Street, Iju, Lagos	Agege	Lagos
4465	LA/0646	LA/0646/S/5	Ancilla Catholic Hospital		3, Muyiwa Olojo Street, Iju, Lagos	Agege	Lagos
4466	LA/0646	LA/0646/S/13	Ancilla Catholic Hospital		3, Muyiwa Olojo Street, Iju, Lagos	Agege	Lagos
4467	LA/0646	LA/0646/S/3	Ancilla Catholic Hospital		3, Muyiwa Olojo Street, Iju, Lagos	Agege	Lagos
4468	LA/0646	LA/0646/S/2	Ancilla Catholic Hospital		3, Muyiwa Olojo Street, Iju, Lagos	Agege	Lagos
4469	LA/0646	LA/0646/S/1	Ancilla Catholic Hospital		3, Muyiwa Olojo Street, Iju, Lagos	Agege	Lagos
4470	LA/0647	LA/0647/S/3	Jobi Clinic & Maternity Hospital	Primary	3/5 Adeyeri Owuyo St Ikorodu	Ikorodu	Lagos
4471	LA/0647	LA/0647/S/8	Jobi Clinic & Maternity Hospital	Primary	3/5 Adeyeri Owuyo St Ikorodu	Ikorodu	Lagos
4472	LA/0647	LA/0647/S/7	Jobi Clinic & Maternity Hospital	Primary	3/5 Adeyeri Owuyo St Ikorodu	Ikorodu	Lagos
4473	LA/0647	LA/0647/S/1	Jobi Clinic & Maternity Hospital	Primary	3/5 Adeyeri Owuyo St Ikorodu	Ikorodu	Lagos
4474	LA/0648	LA/0648/S/4	Omni Medical Centre		18, Boyle''s Street, Onikan, Lagos Island, Lagos	Lagos Island	Lagos
4475	LA/0648	LA/0648/S/3	Omni Medical Centre		18, Boyle''s Street, Onikan, Lagos Island, Lagos	Lagos Island	Lagos
4476	LA/0648	LA/0648/S/1	Omni Medical Centre		18, Boyle''s Street, Onikan, Lagos Island, Lagos	Lagos Island	Lagos
4477	LA/0648	LA/0648/S/5	Omni Medical Centre		18, Boyle''s Street, Onikan, Lagos Island, Lagos	Lagos Island	Lagos
4478	LA/0648	LA/0648/S/2	Omni Medical Centre		18, Boyle''s Street, Onikan, Lagos Island, Lagos	Lagos Island	Lagos

4479	LA/0648	LA/0648/S/6	Omni Medical Centre		18, Boyle''s Street, Onikan, Lagos Island, Lagos	Lagos Island	Lagos
4480	LA/0649	LA/0649/S/1	Motolani Medical Centre		13, Alfa-Nla Street, Agege, Lagos	Agege	Lagos
4481	LA/0649	LA/0649/S/5	Motolani Medical Centre		13, Alfa-Nla Street, Agege, Lagos	Agege	Lagos
4482	LA/0649	LA/0649/S/3	Motolani Medical Centre		13, Alfa-Nla Street, Agege, Lagos	Agege	Lagos
4483	LA/0650	LA/0650/S/3	Esiri Specialist Hospital	Primary	5 Alashe Close Ojogbe Ikorodu	Ikorodu	Lagos
4484	LA/0651	LA/0651/S/8	T&S Hospital, Apapa	Primary Provider	26 Bombay Crescent Apapa	Apapa	Lagos
4485	LA/0651	LA/0651/S/15	T&S Hospital, Apapa	Primary Provider	26 Bombay Crescent Apapa	Apapa	Lagos
4486	LA/0655	LA/0655/S/11	Subol Hospital		16/17, Oba Amusa Avenue, Off Ikotun Rd, Idimu-Lagos	Alimosho	Lagos
4487	LA/0655	LA/0655/S/5	Subol Hospital		16/17, Oba Amusa Avenue, Off Ikotun Rd, Idimu-Lagos	Alimosho	Lagos
4488	LA/0655	LA/0655/S/1	Subol Hospital		16/17, Oba Amusa Avenue, Off Ikotun Rd, Idimu-Lagos	Alimosho	Lagos
4489	LA/0655	LA/0655/S/3	Subol Hospital		16/17, Oba Amusa Avenue, Off Ikotun Rd, Idimu-Lagos	Alimosho	Lagos
4490	LA/0655	LA/0655/S/10	Subol Hospital		16/17, Oba Amusa Avenue, Off Ikotun Rd, Idimu-Lagos	Alimosho	Lagos
4491	LA/0655	LA/0655/S/2	Subol Hospital		16/17, Oba Amusa Avenue, Off Ikotun Rd, Idimu-Lagos	Alimosho	Lagos
4492	LA/0655	LA/0655/S/6	Subol Hospital		16/17, Oba Amusa Avenue, Off Ikotun Rd, Idimu-Lagos	Alimosho	Lagos
4493	LA/0655	LA/0655/S/7	Subol Hospital		16/17, Oba Amusa Avenue, Off Ikotun Rd, Idimu-Lagos	Alimosho	Lagos
4494	LA/0655	LA/0655/S/8	Subol Hospital		16/17, Oba Amusa Avenue, Off Ikotun Rd, Idimu-Lagos	Alimosho	Lagos
4495	LA/0655	LA/0655/S/14	Subol Hospital		16/17, Oba Amusa Avenue, Off Ikotun Rd, Idimu-Lagos	Alimosho	Lagos
4496	LA/0655	LA/0655/S/12	Subol Hospital		16/17, Oba Amusa Avenue, Off Ikotun Rd, Idimu-Lagos	Alimosho	Lagos
4497	LA/0655	LA/0655/S/4	Subol Hospital		16/17, Oba Amusa Avenue, Off Ikotun Rd, Idimu-Lagos	Alimosho	Lagos
4498	LA/0656	LA/0656/S/1	Shammah Hospital	Primary Provider	5, Epe Street Off Iludun Street Amukoko Lagos	Ajeromi/Ifelodun	Lagos
4499	LA/0659	LA/0659/S/5	Bethesda Family Clinic and Maternity	Primary Provider	207 Road, A Close House 6. Festac Town Lagos	Amuwo-Odofin	Lagos
4500	LA/0661	LA/0661/S/1	Hanoba Medical Centre		23, Boyle''s Street, Onikan, Lagos	Eti-Osa	Lagos
4501	LA/0661	LA/0661/S/5	Hanoba Medical Centre		23, Boyle''s Street, Onikan, Lagos	Eti-Osa	Lagos
4502	LA/0662	LA/0662/S/3	Ajayi Medical Center	Primary Provider	50 Ekulu Street Ikorodu	Ikorodu	Lagos
4503	LA/0662	LA/0662/S/8	Ajayi Medical Center	Primary Provider	50 Ekulu Street Ikorodu	Ikorodu	Lagos
4504	LA/0662	LA/0662/S/11	Ajayi Medical Center	Primary Provider	50 Ekulu Street Ikorodu	Ikorodu	Lagos
4505	LA/0662	LA/0662/S/7	Ajayi Medical Center	Primary Provider	50 Ekulu Street Ikorodu	Ikorodu	Lagos
4506	LA/0662	LA/0662/S/1	Ajayi Medical Center	Primary Provider	50 Ekulu Street Ikorodu	Ikorodu	Lagos
4507	LA/0662	LA/0662/S/5	Ajayi Medical Center	Primary Provider	50 Ekulu Street Ikorodu	Ikorodu	Lagos
4508	LA/0662	LA/0662/S/12	Ajayi Medical Center	Primary Provider	50 Ekulu Street Ikorodu	Ikorodu	Lagos
4509	LA/0667	LA/0667/S/15	Natafod Consultants Hospital	Primary Provider	104 Cemetry Road, Mosafejo, NEPA B/Stop, Amukoko, Lagos	Ajeromi/Ifelodun	Lagos
4510	LA/0667	LA/0667/S/3	Natafod Consultants Hospital	Primary Provider	104 Cemetry Road, Mosafejo, NEPA B/Stop, Amukoko, Lagos	Ajeromi/Ifelodun	Lagos
4511	LA/0668	LA/0668/S/3	High Rocks Hospital		38 Afariogun street, Oshodi	Oshodi/Isolo	Lagos
4512	LA/0668	LA/0668/S/5	High Rocks Hospital		38 Afariogun street, Oshodi	Oshodi/Isolo	Lagos
4513	LA/0670	LA/0670/S/1	Adesola Clinic		2 Onabola street Pedro Bariga	Somolu	Lagos
4514	LA/0670	LA/0670/S/5	Adesola Clinic		2 Onabola street Pedro Bariga	Somolu	Lagos
4515	LA/0670	LA/0670/S/7	Adesola Clinic		2 Onabola street Pedro Bariga	Somolu	Lagos

4516	LA/0671	LA/0671/S/8	Ropheka Hospital Ltd	Primary	6 Dosu Ogundele Street Miccom Bus/Stop Akowonjo Lagos	Alimosho	Lagos
4517	LA/0672	LA/0672/S/14	Capes Hospital Ltd		16a Alubarika street, Bariga	Somolu	Lagos
4518	LA/0673	LA/0673/S/3	Promise Medical Center Ltd	Primary	112 Dopemu Rd Ayinla B/Stop Dopemu Lagos	Alimosho	Lagos
4519	LA/0673	LA/0673/S/8	Promise Medical Center Ltd	Primary	112 Dopemu Rd Ayinla B/Stop Dopemu Lagos	Alimosho	Lagos
4520	LA/0673	LA/0673/S/1	Promise Medical Center Ltd	Primary	112 Dopemu Rd Ayinla B/Stop Dopemu Lagos	Alimosho	Lagos
4521	LA/0674	LA/0674/S/4	Primex Hospital	Primary	58 Igando Rd Unity Bus/Stop Ikotun-Egbe Lagos	Alimosho	Lagos
4522	LA/0674	LA/0674/S/5	Primex Hospital	Primary	58 Igando Rd Unity Bus/Stop Ikotun-Egbe Lagos	Alimosho	Lagos
4523	LA/0677	LA/0677/S/1	Okiki Clinic & Maternity	Primary	18 Ola Sheu Str Iyana Ipaja by Alimosho Bus/Stop	Alimosho	Lagos
4524	LA/0679	LA/0679/S/5	Mobonike-T Medical Centre	Primary	26, Surulere 1 Bus Stop Dopemu Lagos	Agege	Lagos
4525	LA/0680	LA/0680/S/1	Majoroh Medical Center	Primary Provider	6 Olofin Street Apapa Lagos	Apapa	Lagos
4526	LA/0680	LA/0680/S/8	Majoroh Medical Center	Primary Provider	6 Olofin Street Apapa Lagos	Apapa	Lagos
4527	LA/0683	LA/0683/S/5	Light Hospital	Primary	15 Olumide Onanubi Str Off Folarin Str Alimosho/Off Jimoh B/S Akowonjo	Alimosho	Lagos
4528	LA/0683	LA/0683/S/4	Light Hospital	Primary	15 Olumide Onanubi Str Off Folarin Str Alimosho/Off Jimoh B/S Akowonjo	Alimosho	Lagos
4529	LA/0683	LA/0683/S/7	Light Hospital	Primary	15 Olumide Onanubi Str Off Folarin Str Alimosho/Off Jimoh B/S Akowonjo	Alimosho	Lagos
4530	LA/0683	LA/0683/S/2	Light Hospital	Primary	15 Olumide Onanubi Str Off Folarin Str Alimosho/Off Jimoh B/S Akowonjo	Alimosho	Lagos
4531	LA/0684	LA/0684/S/3	Somito Specialist Hospital	Primary Provider	Plot 937, 13th , Road First Avenue Festac Town Lagos	Amuwo-Odofin	Lagos
4532	LA/0687	LA/0687/S/3	Hamkad Hospital	Primary	39 Olawale Cole Street U-turn B/Stop Abule Egba	Alimosho	Lagos
4533	LA/0687	LA/0687/S/1	Hamkad Hospital	Primary	39 Olawale Cole Street U-turn B/Stop Abule Egba	Alimosho	Lagos
4534	LA/0690	LA/0690/S/7	DaySpring Hospital & Maternity	Primary	1 Popoola Street Off Isijola Street Ikotun Lagos	Alimosho	Lagos
4535	LA/0690	LA/0690/S/3	DaySpring Hospital & Maternity	Primary	1 Popoola Street Off Isijola Street Ikotun Lagos	Alimosho	Lagos
4536	LA/0690	LA/0690/S/5	DaySpring Hospital & Maternity	Primary	1 Popoola Street Off Isijola Street Ikotun Lagos	Alimosho	Lagos
4537	LA/0690	LA/0690/S/1	DaySpring Hospital & Maternity	Primary	1 Popoola Street Off Isijola Street Ikotun Lagos	Alimosho	Lagos
4538	LA/0697	LA/0697/S/1	Adenike Fadeyibi Memorial Hospital	Primary	20 Akowonjo Rd, Egbeda, Akowonjo Lagos	Alimosho	Lagos
4539	LA/0699	LA/0699/S/7	Golden Cross Infirmary Hospital and Maternity Home Ltd.	Primary Provider	22 Road Festac Town Lagos	Amuwo-Odofin	Lagos
4540	LA/0699	LA/0699/S/5	Golden Cross Infirmary Hospital and Maternity Home Ltd.	Primary Provider	22 Road Festac Town Lagos	Amuwo-Odofin	Lagos
4541	LA/0705	LA/0705/S/3	Elphy Hospital		6, Task Force Rd, By PPL B/S, Okokomaiko, Lagos	Ojo	Lagos
4542	LA/0705	LA/0705/S/5	Elphy Hospital		6, Task Force Rd, By PPL B/S, Okokomaiko, Lagos	Ojo	Lagos
4543	LA/0706	LA/0706/S/7	Onyems-B Hospital		16, Oba-Dauda Str., Ojoo Alaba, Lagos	Ojo	Lagos
4544	LA/0706	LA/0706/S/4	Onyems-B Hospital		16, Oba-Dauda Str., Ojoo Alaba, Lagos	Ojo	Lagos
4545	LA/0706	LA/0706/S/1	Onyems-B Hospital		16, Oba-Dauda Str., Ojoo Alaba, Lagos	Ojo	Lagos
4546	LA/0706	LA/0706/S/3	Onyems-B Hospital		16, Oba-Dauda Str., Ojoo Alaba, Lagos	Ojo	Lagos
4547	LA/0714	LA/0714/S/4	The Great Physcian Hospital Ltd		3, Niyi Adebule Str, New Nepa B/S Badagry	Badagry	Lagos
4548	LA/0715	LA/0715/S/7	Ola-Oki Medical Centre		Opp Iberoko Low cost B/S Ibereko, Badagry	Badagry	Lagos
4549	LA/0715	LA/0715/S/4	Ola-Oki Medical Centre		Opp Iberoko Low cost B/S Ibereko, Badagry	Badagry	Lagos

4550	LA/0715	LA/0715/S/1	Ola-Oki Medical Centre		Opp Iberoko Low cost B/S Ibereko, Badagry	Badagry	Lagos
4551	LA/0716	LA/0716/S/11	General Hospital Apapa		16, Ibikunle Akintoye Str, Off Randle Rd, Apapa	Apapa	Lagos
4552	LA/0716	LA/0716/S/8	General Hospital Apapa		16, Ibikunle Akintoye Str, Off Randle Rd, Apapa	Apapa	Lagos
4553	LA/0716	LA/0716/S/3	General Hospital Apapa		16, Ibikunle Akintoye Str, Off Randle Rd, Apapa	Apapa	Lagos
4554	LA/0716	LA/0716/S/5	General Hospital Apapa		16, Ibikunle Akintoye Str, Off Randle Rd, Apapa	Apapa	Lagos
4555	LA/0716	LA/0716/S/4	General Hospital Apapa		16, Ibikunle Akintoye Str, Off Randle Rd, Apapa	Apapa	Lagos
4556	LA/0716	LA/0716/S/1	General Hospital Apapa		16, Ibikunle Akintoye Str, Off Randle Rd, Apapa	Apapa	Lagos
4557	LA/0716	LA/0716/S/6	General Hospital Apapa		16, Ibikunle Akintoye Str, Off Randle Rd, Apapa	Apapa	Lagos
4558	LA/0716	LA/0716/S/15	General Hospital Apapa		16, Ibikunle Akintoye Str, Off Randle Rd, Apapa	Apapa	Lagos
4559	LA/0720	LA/0720/S/3	Unity Hospital ,Ikeja.		138 Obafemi Awolowo Way Ikeja	Ikeja	Lagos
4560	LA/0720	LA/0720/S/1	Unity Hospital ,Ikeja.		138 Obafemi Awolowo Way Ikeja	Ikeja	Lagos
4561	LA/0720	LA/0720/S/4	Unity Hospital ,Ikeja.		138 Obafemi Awolowo Way Ikeja	Ikeja	Lagos
4562	LA/0721	LA/0721/S/6	Adefemi Hospital	Primary Provider	49 Seriki Aro, Avenu Off Obafemi Awolowo Way Lagos	Ikeja	Lagos
4563	LA/0721	LA/0721/S/8	Adefemi Hospital	Primary Provider	49 Seriki Aro, Avenu Off Obafemi Awolowo Way Lagos	Ikeja	Lagos
4564	LA/0721	LA/0721/S/2	Adefemi Hospital	Primary Provider	49 Seriki Aro, Avenu Off Obafemi Awolowo Way Lagos	Ikeja	Lagos
4565	LA/0721	LA/0721/S/5	Adefemi Hospital	Primary Provider	49 Seriki Aro, Avenu Off Obafemi Awolowo Way Lagos	Ikeja	Lagos
4566	LA/0721	LA/0721/S/1	Adefemi Hospital	Primary Provider	49 Seriki Aro, Avenu Off Obafemi Awolowo Way Lagos	Ikeja	Lagos
4567	LA/0721	LA/0721/S/15	Adefemi Hospital	Primary Provider	49 Seriki Aro, Avenu Off Obafemi Awolowo Way Lagos	Ikeja	Lagos
4568	LA/0721	LA/0721/S/3	Adefemi Hospital	Primary Provider	49 Seriki Aro, Avenu Off Obafemi Awolowo Way Lagos	Ikeja	Lagos
4569	LA/0723	LA/0723/S/5	The Duke Medical	Primary Provider	26 Bamishele Street Off Allen Avenue Ikeja	Ikeja	Lagos
4570	LA/0723	LA/0723/S/15	The Duke Medical	Primary Provider	26 Bamishele Street Off Allen Avenue Ikeja	Ikeja	Lagos
4571	LA/0724	LA/0724/S/3	Isalu Hospital	Primary Provider	10 Wempco Road, Off Lateef Jakande Road, Ogba, Ikeja	Ikeja	Lagos
4572	LA/0724	LA/0724/S/5	Isalu Hospital	Primary Provider	10 Wempco Road, Off Lateef Jakande Road, Ogba, Ikeja	Ikeja	Lagos
4573	LA/0726	LA/0726/S/5	RCCG Life Centre Hospital & Maternity		9 Amore Street Off Toyin Street, Ikeja	Ikeja	Lagos
4574	LA/0726	LA/0726/S/3	RCCG Life Centre Hospital & Maternity		9 Amore Street Off Toyin Street, Ikeja	Ikeja	Lagos
4575	LA/0730	LA/0730/S/1	The Valley Surgical and Medical Clinics	Primary Provider	5 Olatayo Alao Street River valley Estate Ojodu, Lagos	Ikeja	Lagos
4576	LA/0731	LA/0731/S/5	Solid Rock Hospital	Primary Provider	Plote 108 Isheri Road Ojodu	Ikeja	Lagos
4577	LA/0731	LA/0731/S/8	Solid Rock Hospital	Primary Provider	Plote 108 Isheri Road Ojodu	Ikeja	Lagos
4578	LA/0731	LA/0731/S/3	Solid Rock Hospital	Primary Provider	Plote 108 Isheri Road Ojodu	Ikeja	Lagos
4579	LA/0731	LA/0731/S/1	Solid Rock Hospital	Primary Provider	Plote 108 Isheri Road Ojodu	Ikeja	Lagos
4580	LA/0731	LA/0731/S/4	Solid Rock Hospital	Primary Provider	Plote 108 Isheri Road Ojodu	Ikeja	Lagos
4581	LA/0732	LA/0732/S/5	Sharon Height Medical Centre		13 Amore Street Off Toyin Street Ikeja	Ikeja	Lagos
4582	LA/0732	LA/0732/S/3	Sharon Height Medical Centre		13 Amore Street Off Toyin Street Ikeja	Ikeja	Lagos
4583	LA/0732	LA/0732/S/1	Sharon Height Medical Centre		13 Amore Street Off Toyin Street Ikeja	Ikeja	Lagos
4584	LA/0732	LA/0732/S/6	Sharon Height Medical Centre		13 Amore Street Off Toyin Street Ikeja	Ikeja	Lagos
4585	LA/0732	LA/0732/S/12	Sharon Height Medical Centre		13 Amore Street Off Toyin Street Ikeja	Ikeja	Lagos
4586	LA/0733	LA/0733/S/6	Mother & Child Hospital	Primary Provider	39A, Adeniyi Jones Avenue Ikeja	Ikeja	Lagos
4587	LA/0733	LA/0733/S/5	Mother & Child Hospital	Primary Provider	39A, Adeniyi Jones Avenue Ikeja	Ikeja	Lagos
4588	LA/0736	LA/0736/S/1	German Friendship		20, Amore Street Off Toyin Street	Ikeja	Lagos

4589	LA/0736	LA/0736/S/3	German Friendship		20, Amore Street Off Toyin Street	Ikeja	Lagos
4590	LA/0736	LA/0736/S/5	German Friendship		20, Amore Street Off Toyin Street	Ikeja	Lagos
4591	LA/0736	LA/0736/S/4	German Friendship		20, Amore Street Off Toyin Street	Ikeja	Lagos
4592	LA/0737	LA/0737/S/5	G & S Medical & Dental Hospital	Primary Provider	15 Muyibi Street, Oveira Ogba, Lagos	Ikeja	Lagos
4593	LA/0737	LA/0737/S/8	G & S Medical & Dental Hospital	Primary Provider	15 Muyibi Street, Oveira Ogba, Lagos	Ikeja	Lagos
4594	LA/0738	LA/0738/S/3	Dominion Medical Consultant	Primary Provider	7 Ade Ojo Street Wemabod Estate, Ikeja, Lagos	Ikeja	Lagos
4595	LA/0738	LA/0738/S/1	Dominion Medical Consultant	Primary Provider	7 Ade Ojo Street Wemabod Estate, Ikeja, Lagos	Ikeja	Lagos
4596	LA/0738	LA/0738/S/14	Dominion Medical Consultant	Primary Provider	7 Ade Ojo Street Wemabod Estate, Ikeja, Lagos	Ikeja	Lagos
4597	LA/0738	LA/0738/S/5	Dominion Medical Consultant	Primary Provider	7 Ade Ojo Street Wemabod Estate, Ikeja, Lagos	Ikeja	Lagos
4598	LA/0740	LA/0740/S/3	Amazing Grace Medical Centre	Primary Provider	PF 910, Omole Phase 1 Omotade Crescent Agidibi Rd. IKeja	Ikeja	Lagos
4599	LA/0745	LA/0745/S/5	Parkande Specialist Hospital		13, Rasaq Balogun Street Off Adetola Street Off Adeniran Ogunsanya	Surulere	Lagos
4600	LA/0745	LA/0745/S/14	Parkande Specialist Hospital		13, Rasaq Balogun Street Off Adetola Street Off Adeniran Ogunsanya	Surulere	Lagos
4601	LA/0745	LA/0745/S/4	Parkande Specialist Hospital		13, Rasaq Balogun Street Off Adetola Street Off Adeniran Ogunsanya	Surulere	Lagos
4602	LA/0745	LA/0745/S/6	Parkande Specialist Hospital		13, Rasaq Balogun Street Off Adetola Street Off Adeniran Ogunsanya	Surulere	Lagos
4603	LA/0745	LA/0745/S/1	Parkande Specialist Hospital		13, Rasaq Balogun Street Off Adetola Street Off Adeniran Ogunsanya	Surulere	Lagos
4604	LA/0745	LA/0745/S/3	Parkande Specialist Hospital		13, Rasaq Balogun Street Off Adetola Street Off Adeniran Ogunsanya	Surulere	Lagos
4605	LA/0748	LA/0748/S/3	Jalupon EState Hospital	Primary Provider	63 Bode Thomas Street Lagos	Surulere	Lagos
4606	LA/0748	LA/0748/S/1	Jalupon EState Hospital	Primary Provider	63 Bode Thomas Street Lagos	Surulere	Lagos
4607	LA/0752	LA/0752/S/8	Primus Hospital		272 Ijesha Rd. Ijesha-tedo	Surulere	Lagos
4608	LA/0768	LA/0768/S/4	Rosewell Pharmacy Ltd	Pharmacy	Signboard Bus stop Adoo Rd Ajah Lagos	Eti-Osa	Lagos
4609	LA/0779	LA/0779/S/13	Krystal Opticals Ltd	Eye services	156 Awolowo Rd SW Ikoyi Lagos	Eti-Osa	Lagos
4610	LA/0780	LA/0780/S/13	Ultimate Eye Clinic	Eye Services	16 Biaduo Str SW Ikoyi Lagos	Eti-Osa	Lagos
4611	LA/0782	LA/0782/S/8	Smiley Dental Clinic	Dental Services	Suite F11, 1st Floor VGC Shopping Mall VGC Lekki Lagos	Eti-Osa	Lagos
4612	LA/0784	LA/0784/S/8	Degason Dental Health Ltd	Dental Services	2A Lalupon Close Off Keffi Str SW Ikoyi Lagos	Eti-Osa	Lagos
4613	LA/0785	LA/0785/S/8	Aduagem Dental Clinic	Dental Services	3B Ligali Ayorinde Str VI Lagos	Eti-Osa	Lagos
4614	LA/0786	LA/0786/S/8	Divine dental Home	Dental Services	8B Fabac Close Off Ligali Ayorinde Str VI Lagos	Eti-Osa	Lagos
4615	LA/0787	LA/0787/S/8	Federal Dental Clinic	Dental Services	No 1 Broad str Lagos	Kosofe	Lagos
4616	LA/0791	LA/0791/S/5	The Health Arena	O&G	13 Strachan Str Off Igbosere Rd Opp Lapal House Lagos	Lagos Island	Lagos
4617	LA/0791	LA/0791/S/3	The Health Arena	O&G	13 Strachan Str Off Igbosere Rd Opp Lapal House Lagos	Lagos Island	Lagos
4618	LA/0804	LA/0804/S/5	Clina Immunoassay Laboratories Ltd	Laboratory	41, Old Road, Amuwo-Odofin Lagos	Amuwo-Odofin	Lagos
4619	LA/0805	LA/0805/S/4	Solutions Pharmaceuticals Nig. Ltd.	Pharmacy	1B Jejumola St. Off Alafia Bust Stop By Oando Filling Station Amukoko Lagos	Amuwo-Odofin	Lagos
4620	LA/0808	LA/0808/S/5	Dunamis Diagnostic Service	Laboratory	367, Ojo Rd. Satellite Town Lagos	Ojo	Lagos
4621	LA/0813	LA/0813/S/5	H-Power Diagnostic Service(Aniyun Hospital)	Laboratory	No 3 Femi Aderibigbe Close Off Diya St Beside Total Filling Station Gbagada	Kosofe	Lagos
4622	LA/0817	LA/0817/S/13	Marole Eye clinic (Optometry)	Optometry	44 Diya Street, Gbagada, Lagos	Kosofe	Lagos
4623	LA/0818	LA/0818/S/8	St Emmanuel Hospital	Physiotheraphy, Dental	No 2 Bola Ademuyinwa St Off Osolo Way Isolo Lagos	Oshodi/Isolo	Lagos

4624	LA/0818	LA/0818/S/11	St Emmanuel Hospital	Physiotheraphy, Dental	No 2 Bola Ademuyinwa St Off Osolo Way Isolo Lagos	Oshodi/Isolo	Lagos
4625	LA/0819	LA/0819/S/5	Heda Hospital	Laboratory	No 2A, Princess Aina jegede close, Ajao Estate Lagos	Oshodi/Isolo	Lagos
4626	LA/0820	LA/0820/S	Mayob Medical Laboratory	Laboratory	No 2 Ifos Rd Iyana Ejogbo B/Stop Oshodi	Oshodi/Isolo	Lagos
4627	LA/0821	LA/0821/S/4	Bayo Superb Pharmacy	Pharmacy	No 11 Olusesi Street Ejigbo	Oshodi/Isolo	Lagos
4628	LA/0822	LA/0822/S/4	Aje Investment Ltd	Pharmacy	No 2 Alaba St Off Pipeline Rd Opp Oboye Junction Orilowo Ejigbo	Oshodi/Isolo	Lagos
4629	LA/0823	LA/0823/S/4	Pinco Pharm Nig Ltd	Pharmacy	No 24 Oke St Pedro Shomolu	Somolu	Lagos
4630	LA/0825	LA/0825/S/4	Remvic Pharmacy	Pharmacy	No 14 Ire-Akari Estate Isolo Lagos	Oshodi/Isolo	Lagos
4631	LA/0826	LA/0826/S/4	Chymasen Pharmacy & Stores	Pharmacy	No Taiwo St Ago Palace Way Okota Lagos	Oshodi/Isolo	Lagos
4632	LA/0830	LA/0830/S/7	Union Diagnostic & Clinical Services	Radiology, Laboratory	37 Tejuosho Street Surulere, Lagos	Surulere	Lagos
4633	LA/0830	LA/0830/S/5	Union Diagnostic & Clinical Services	Radiology, Laboratory	37 Tejuosho Street Surulere, Lagos	Surulere	Lagos
4634	LA/0832	LA/0832/S/4	Santus Pharm & Stores	Pharmacy	272 Ijesha Rd Ijesha	Surulere	Lagos
4635	LA/0833	LA/0833/S/4	Rosec Pharmacy Ltd		Plot 50 Babs Animashaun Extn Off Bode Thomas	Surulere	Lagos
4636	LA/0835	LA/0835/S/7	Prisms Diagnostic Clinic	Radiology	8 Oyediran Str, Off Bode Thomas	Surulere	Lagos
4637	LA/0839	LA/0839/S/4	O Medics Pharmacy	Pharmacy	6 Oduduwa Str, Ikate	Surulere	Lagos
4638	LA/0843	LA/0843/S/4	Jubliee Pharmacy	Pharmacy	20 Adeniyi Adeyioye Str, Ikate	Surulere	Lagos
4639	LA/0844	LA/0844/S/8	Green Fountain Medical Services	Dentistry	16 Akinsemoyin Str, Off Bode Thomas Str	Surulere	Lagos
4640	LA/0845	LA/0845/S/4	Frontliners Pharmacy & Stores	Pharmacy	2 Smith Str, Off Olutire Odo eran, Itire	Surulere	Lagos
4641	LA/0846	LA/0846/S/5	Clinics Laboratory	Labortory	44 Ishaya Rd	Surulere	Lagos
4642	LA/0848	LA/0848/S/5	Bauke Diagnostic Centre	Laboratory	272 ijesha Rd, Ijesha Tedo	Alimosho	Lagos
4643	LA/0849	LA/0849/S/5	Anumels Medical Laboratory	Laboratory	38 Sabiu Ajose Cres, Off Bode Thomas	Surulere	Lagos
4644	LA/0851	LA/0851/S/8	Spectrum Dental Clinic	Dentistry	26, Babs Animashaun, Off Bode Thomas	Surulere	Lagos
4645	LA/0852	LA/0852/S/8	Smiley Dental Care	Dentistry	81 Bode Thomas Str	Surulere	Lagos
4646	LA/0853	LA/0853/S/5	St Lukes Medical Centre	Laboratory, Surgery, O&G	14 Alh Olakunle Str, Off Ommileni Str, Ijesha	Surulere	Lagos
4647	LA/0853	LA/0853/S/3	St Lukes Medical Centre	Laboratory, Surgery, O&G	14 Alh Olakunle Str, Off Ommileni Str, Ijesha	Surulere	Lagos
4648	LA/0853	LA/0853/S/1	St Lukes Medical Centre	Laboratory, Surgery, O&G	14 Alh Olakunle Str, Off Ommileni Str, Ijesha	Surulere	Lagos
4649	LA/0854	LA/0854/S/15	Orbit Vision	Ophthalmology	36, adelabu Str	Surulere	Lagos
4650	LA/0856	LA/0856/S/5	Precise Medical Diagnostic	Radiology, Ultrasanography, Laboratory	167, Iju Road, Fagba B/Stop Agege Lagos	Agege	Lagos
4651	LA/0856	LA/0856/S/7	Precise Medical Diagnostic	Radiology, Ultrasanography, Laboratory	167, Iju Road, Fagba B/Stop Agege Lagos	Agege	Lagos
4652	LA/0856	LA/0856/S/14	Precise Medical Diagnostic	Radiology, Ultrasanography, Laboratory	167, Iju Road, Fagba B/Stop Agege Lagos	Agege	Lagos
4653	LA/0858	LA/0858/S/5	Jeffis Specialist Hospital		9 Adegbola Street, Alakuku, Lagos	Alimosho	Lagos
4654	LA/0858	LA/0858/S/8	Jeffis Specialist Hospital		9 Adegbola Street, Alakuku, Lagos	Alimosho	Lagos
4655	LA/0858	LA/0858/S/3	Jeffis Specialist Hospital		9 Adegbola Street, Alakuku, Lagos	Alimosho	Lagos
4656	LA/0858	LA/0858/S/1	Jeffis Specialist Hospital		9 Adegbola Street, Alakuku, Lagos	Alimosho	Lagos
4657	LA/0859	LA/0859/S/5	O.A.U Medical Diagnostic Centre	Laboratory	577, Awori B/Stop Abule-Egba Lagos-Abeokuta Express Way	Agege	Lagos
4658	LA/0860	LA/0860/S/7	Naz Health Support Services	Laboratory,Radiology	13, Winfunke Olowe St Abule-Taylor B/Stop Off Lagos-Abeokuta Exp Rd	Agege	Lagos
4659	LA/0860	LA/0860/S/5	Naz Health Support Services	Laboratory,Radiology	13, Winfunke Olowe St Abule-Taylor B/Stop Off Lagos-Abeokuta Exp Rd	Agege	Lagos
4660	LA/0861	LA/0861/S/1	Lex Medical Centre		5 Rahat Alabi Street, Iyana Meiran B/Stop, Ijaiye, Lagos	Alimosho	Lagos
4661	LA/0861	LA/0861/S/5	Lex Medical Centre		5 Rahat Alabi Street, Iyana Meiran B/Stop, Ijaiye, Lagos	Alimosho	Lagos
4662	LA/0861	LA/0861/S/2	Lex Medical Centre		5 Rahat Alabi Street, Iyana Meiran B/Stop, Ijaiye, Lagos	Alimosho	Lagos
4663	LA/0861	LA/0861/S/10	Lex Medical Centre		5 Rahat Alabi Street, Iyana Meiran B/Stop, Ijaiye, Lagos	Alimosho	Lagos

4664	LA/0867	LA/0867/S/4	Jaybion Nig Ltd	Pharmacy	Suite 7&8 Block A Bola Ahmed Tinubu Shopping Arcade Pen Cinima Agege	Agege	Lagos
4665	LA/0869	LA/0869/S/7	Ikeja X-Ray Clinic	Radiology	Lagos Airport Hotel	Ikeja	Lagos
4666	LA/0872	LA/0872/S/5	Casay Diagnostic Centre	Laboratory	Plot 20 Lateef Jakande Rd Davidson Plaza Omole	Ikeja	Lagos
4667	LA/0875	LA/0875/S/4	Dhug Pharmacy	Pharmacy	21 Joel Ogunaike Street, GRA, Ikeja, Lagos	Ikeja	Lagos
4668	LA/0877	LA/0877/S/8	D Vine Dental Clinic	Dental	25/27A Isaac John Street GRA, Lagos	Ikeja	Lagos
4669	LA/0879	LA/0879/S/4	Phebiox Pharm.	Pharmacy	11 Osho Street, Opebi, Ikeja, Lagos	Ikeja	Lagos
4670	LA/0881	LA/0881/S/4	Anchor Link Pharm.	Pharmacy	35, Ojodu Abiodun, Ojodu, Lagos	Ikeja	Lagos
4671	LA/0884	LA/0884/S/5	Omunua Laboratory	Laboratory	21/23 Tonade Street, Ikeja, Lagos	Ikeja	Lagos
4672	LA/0890	LA/0890/S/5	Clina Laboratory	Laboratory	Shop 121, Opic Plaza, Mobolaji Bank, Anthony Way, Lagos	Ikeja	Lagos
4673	LA/0893	LA/0893/S/8	Nene Dental Centre	Dentistry	24 Allen Avenue, Lagos	Ikeja	Lagos
4674	LA/0894	LA/0894/S/8	Osagie Dental Clinic	Dentistry	299 Ikorodu Road, Maryland, Olatunde House, Lagos	Ikeja	Lagos
4675	LA/0900	LA/0900/S/4	Pearly Rems Nig Ltd	Pharmacy	Plot 83 52 RD 5th Av Gowon Estate-Egbeda	Alimosho	Lagos
4676	LA/0901	LA/0901/S/4	Peace Way HealthCare Ltd	Pharmacy	34, Igando Road, Ologunfe B/Stop Igando Ikotu Lagos	Alimosho	Lagos
4677	LA/0902	LA/0902/S/4	Myfil Pharmacy	Pharmacy	167, Akowonjo Rd Lagos	Alimosho	Lagos
4678	LA/0903	LA/0903/S/4	Kardinal Pharmaceutical Nig-Ltd	Pharmacy	134, Ijegun Road, Ikotun Lagos	Alimosho	Lagos
4679	LA/0904	LA/0904/S/4	Juta Pharmacy Ltd	Pharmacy	1, Baale Street, Ipaja Rd Ayobo-Ipaja Lagos	Alimosho	Lagos
4680	LA/0906	LA/0906/S/4	Juta Pharmacy Ltd	Pharmacy	381,Isheri-Olofin Roundabout, Isheri-Idimu Lagos	Alimosho	Lagos
4681	LA/0907	LA/0907/S/7	Joas Medical Diagonistix Ventures	Radiology,Ultrasa nography, Laboratory	2, Okesuna Street, Opp Synagogue, Ikotun-Egbe Lagos	Alimosho	Lagos
4682	LA/0907	LA/0907/S/14	Joas Medical Diagonistix Ventures	Radiology,Ultrasa nography, Laboratory	2, Okesuna Street, Opp Synagogue, Ikotun-Egbe Lagos	Alimosho	Lagos
4683	LA/0907	LA/0907/S/5	Joas Medical Diagonistix Ventures	Radiology,Ultrasa nography, Laboratory	2, Okesuna Street, Opp Synagogue, Ikotun-Egbe Lagos	Alimosho	Lagos
4684	LA/0909	LA/0909/S/1	Frola Pharmacy Ltd	Pharmacy	Mobile Police Barrack Rd Car Wash Bus Stop Idimu-Lagos	Alimosho	Lagos
4685	LA/0910	LA/0910/S/4	Egbeda Pharmacy Ltd	Pharmacy	14, Idimu Rd By Egbeda B/Stop Egbeda Lagos	Alimosho	Lagos
4686	LA/0911	LA/0911/S/7	Farnik x-ray and Ultrasound Centre	Radiology,Ultrasa nography	118,Abeokuta Express Way Adeolu Dopemu Lagos	Alimosho	Lagos
4687	LA/0911	LA/0911/S/14	Farnik x-ray and Ultrasound Centre	Radiology,Ultrasa nography	118,Abeokuta Express Way Adeolu Dopemu Lagos	Alimosho	Lagos
4688	LA/0912	LA/0912/S/8	Ropheka Dental Clinic Ltd	Dental	6 Dosu Ogundele Street Miccom Bus/Stop Akowonjo Lagos	Alimosho	Lagos
4689	LA/0920	LA/0920/S/4	Fannimed Hospital		Block 210, Lily Road, LSDPC Med. Estate, Phase IV, Oba-Oguniyi Rd, Ogba, Lagos	Ikeja	Lagos
4690	LA/0920	LA/0920/S/3	Fannimed Hospital		Block 210, Lily Road, LSDPC Med. Estate, Phase IV, Oba-Oguniyi Rd, Ogba, Lagos	Ikeja	Lagos
4691	LA/0920	LA/0920/S/5	Fannimed Hospital		Block 210, Lily Road, LSDPC Med. Estate, Phase IV, Oba-Oguniyi Rd, Ogba, Lagos	Ikeja	Lagos
4692	LA/0922	LA/0922/S/1	Utibe Abasi Hospital - Victoria Island	Primary	897B Balarabe Musa Crescent Victoria Island Lagos	Lagos Island	Lagos
4693	LA/0922	LA/0922/S/7	Utibe Abasi Hospital - Victoria Island	Primary	897B Balarabe Musa Crescent Victoria Island Lagos	Lagos Island	Lagos
4694	LA/0922	LA/0922/S/5	Utibe Abasi Hospital - Victoria Island	Primary	897B Balarabe Musa Crescent Victoria Island Lagos	Lagos Island	Lagos
4695	LA/0922	LA/0922/S/6	Utibe Abasi Hospital - Victoria Island	Primary	897B Balarabe Musa Crescent Victoria Island Lagos	Lagos Island	Lagos
4696	LA/0922	LA/0922/S/3	Utibe Abasi Hospital - Victoria Island	Primary	897B Balarabe Musa Crescent Victoria Island Lagos	Lagos Island	Lagos

4697	LA/0938	LA/0938/S/19	Bidson Clinic & Skin Centre	Dermatology	368, Murtala Mohammed Way, Yaba, Lagos	Lagos Mainland	Lagos
4698	LA/0940	LA/0940/S/4	Caraale Pharmacy Ltd	Pharmacy	77A Bola Street, Ebute-Metta, Lagos	Lagos Mainland	Lagos
4699	LA/0941	LA/0941/S/4	Silverline Pharm. Ltd	Pharmacy	39, Akanro Street, Ilasa Maja, Mushin, Lagos	Lagos Mainland	Lagos
4700	LA/0945	LA/0945/S/7	Hospital Support Diagnostic Centre Ltd	Radiology, Laboratory	42, Demurin Str., Elebiju B/Stop, Ketu, Lagos	Kosofe	Lagos
4701	LA/0945	LA/0945/S/5	Hospital Support Diagnostic Centre Ltd	Radiology, Laboratory	42, Demurin Str., Elebiju B/Stop, Ketu, Lagos	Kosofe	Lagos
4702	LA/0947	LA/0947/S/7	A-J Rapha Diagnostic Centre	Radiology	30, Isheri Road, Akiode Omole, Ojodu, Lagos	Ikeja	Lagos
4703	LA/0948	LA/0948/S/5	P.J. Medical Diagnostic Laboratories	Laboratory	2, Makoko Road, (Red Cross Compound) off Herbert Marcaulay Str., Yaba, Lagos	Lagos Mainland	Lagos
4704	LA/0949	LA/0949/S/5	Jat Medical Laboratory Service	Laboratory	59, Lagos Road, Ikorodu, Lagos	Ikorodu	Lagos
4705	LA/0950	LA/0950/S/5	Clina-Labs (Nig) Ltd	Laboratory	31, Glover Str., Ebute-Metta, Lagos	Lagos Mainland	Lagos
4706	LA/0951	LA/0951/S/5	Danifol Biotechnology Laboratories	Laboratory	11, Gbeto Street, off Iyana Church, Iwaya Road, Lagos	Lagos Mainland	Lagos
4707	LA/0953	LA/0953/S/5	Ikate Diagnostic Centre	Radiology, Laboratory	59 Anobi Street, Ikate, Surulere, Lagos	Surulere	Lagos
4708	LA/0953	LA/0953/S/7	Ikate Diagnostic Centre	Radiology, Laboratory	59 Anobi Street, Ikate, Surulere, Lagos	Surulere	Lagos
4709	LA/0962	LA/0962/S/4	Osbud Pharmacy & Stores Ltd.		Giwa Shopping Complex, 22nd Rd, by TEXACO F/Station, Festac Town, Lagos	Amuwo-Odofin	Lagos
4710	LA/0967	LA/0967/S/3	Adeolu hospital		4-6, Oluwole Street, Akoka	Somolu	Lagos
4711	LA/0971	LA/0971/S/5	adeshina hospital		29. daudu street, off church b/stop, oshodi	Oshodi/Isolo	Lagos
4712	LA/0974	LA/0974/S/5	ANNA MARIA HOSPITAL		19A DA SILVA STR. OFF BISOLA DUROSINMI DRIVE, LEKKI PHASE 1	Lagos Island	Lagos
4713	LA/0981	LA/0981/S/6	BIO BATAM HOSPITAL		53 TIJANI STREET IYANA IPAJA OFF LAGOS ABEOKUTA EX/WAY, AGEGE	Agege	Lagos
4714	LA/0982	LA/0982/S/3	BRODIE MENDS MEMORIAL HOSPITAL		8, KUGBUYI STREET MUSHIN	Mushin	Lagos
4715	LA/0982	LA/0982/S/5	BRODIE MENDS MEMORIAL HOSPITAL		8, KUGBUYI STREET MUSHIN	Mushin	Lagos
4716	LA/0985	LA/0985/S/11	CITIZEN MEDICAL CENTER		86 NORMAN WILLIAMS STREET S/W IKOYI	Eti-Osa	Lagos
4717	LA/0985	LA/0985/S/5	CITIZEN MEDICAL CENTER		86 NORMAN WILLIAMS STREET S/W IKOYI	Eti-Osa	Lagos
4718	LA/0985	LA/0985/S/6	CITIZEN MEDICAL CENTER		86 NORMAN WILLIAMS STREET S/W IKOYI	Eti-Osa	Lagos
4719	LA/0985	LA/0985/S/3	CITIZEN MEDICAL CENTER		86 NORMAN WILLIAMS STREET S/W IKOYI	Eti-Osa	Lagos
4720	LA/0985	LA/0985/S/1	CITIZEN MEDICAL CENTER		86 NORMAN WILLIAMS STREET S/W IKOYI	Eti-Osa	Lagos
4721	LA/0992	LA/0992/S/5	F.C.E AKOKA		AKOKA YABA	Somolu	Lagos
4722	LA/0995	LA/0995/S/1	FIRST FAITH MEDICAL CENTRE, SURULERE		5, KARIMU COKER ORILE IGANMU	Surulere	Lagos
4723	LA/0995	LA/0995/S/4	FIRST FAITH MEDICAL CENTRE, SURULERE		5, KARIMU COKER ORILE IGANMU	Surulere	Lagos
4724	LA/0995	LA/0995/S/3	FIRST FAITH MEDICAL CENTRE, SURULERE		5, KARIMU COKER ORILE IGANMU	Surulere	Lagos
4725	LA/0995	LA/0995/S/5	FIRST FAITH MEDICAL CENTRE, SURULERE		5, KARIMU COKER ORILE IGANMU	Surulere	Lagos
4726	LA/0995	LA/0995/S/14	FIRST FAITH MEDICAL CENTRE, SURULERE		5, KARIMU COKER ORILE IGANMU	Surulere	Lagos
4727	LA/0998	LA/0998/S/3	GOODTIDINGS HOSPITAL		37/39 FASORO STREET, SURULERE	Surulere	Lagos
4728	LA/1002	LA/1002/S/3	HOLY FAMILY CATHOLIC MEDICAL CENTER		22 RD FESTAC TOWN	Amuwo-Odofin	Lagos
4729	LA/1004	LA/1004/S/12	IMPERIAL MEDICAL CENTER		65 BRICKFIELD ROAD, EBUTE METTA WEST	Lagos Mainland	Lagos
4730	LA/1004	LA/1004/S/1	IMPERIAL MEDICAL CENTER		65 BRICKFIELD ROAD, EBUTE METTA WEST	Lagos Mainland	Lagos
4731	LA/1004	LA/1004/S/6	IMPERIAL MEDICAL CENTER		65 BRICKFIELD ROAD, EBUTE METTA WEST	Lagos Mainland	Lagos
4732	LA/1004	LA/1004/S/2	IMPERIAL MEDICAL CENTER		65 BRICKFIELD ROAD, EBUTE METTA WEST	Lagos Mainland	Lagos

4733	LA/1004	LA/1004/S/5	IMPERIAL MEDICAL CENTER		65 BRICKFIELD ROAD, EBUTE METTA WEST	Lagos Mainland	Lagos
4734	LA/1004	LA/1004/S/10	IMPERIAL MEDICAL CENTER		65 BRICKFIELD ROAD, EBUTE METTA WEST	Lagos Mainland	Lagos
4735	LA/1004	LA/1004/S/3	IMPERIAL MEDICAL CENTER		65 BRICKFIELD ROAD, EBUTE METTA WEST	Lagos Mainland	Lagos
4736	LA/1004	LA/1004/S/11	IMPERIAL MEDICAL CENTER		65 BRICKFIELD ROAD, EBUTE METTA WEST	Lagos Mainland	Lagos
4737	LA/1004	LA/1004/S/15	IMPERIAL MEDICAL CENTER		65 BRICKFIELD ROAD, EBUTE METTA WEST	Lagos Mainland	Lagos
4738	LA/1007	LA/1007/S/5	JLT SPECIALIST CLINIC & SKIN CENTER		43A KAKAWA STREET, LAGOS	Lagos Island	Lagos
4739	LA/1008	LA/1008/S/6	KRANZ SPECIALIST CLINIC & MAT. HOME		9, UNITY ROAD OFF TOYIN STREET, IKEJA	Ikeja	Lagos
4740	LA/1009	LA/1009/S/1	LIBRA SPECIALIST HOSPITAL		1, ONISEMO STREET,OFF MARSHA SURULERE	Surulere	Lagos
4741	LA/1009	LA/1009/S/5	LIBRA SPECIALIST HOSPITAL		1, ONISEMO STREET,OFF MARSHA SURULERE	Surulere	Lagos
4742	LA/1009	LA/1009/S/3	LIBRA SPECIALIST HOSPITAL		1, ONISEMO STREET,OFF MARSHA SURULERE	Surulere	Lagos
4743	LA/1009	LA/1009/S/6	LIBRA SPECIALIST HOSPITAL		1, ONISEMO STREET,OFF MARSHA SURULERE	Surulere	Lagos
4744	LA/1011	LA/1011/S/6	life line children hospital		133 OGUNLANA DRIVE SURULERE	Surulere	Lagos
4745	LA/1011	LA/1011/S/5	life line children hospital		133 OGUNLANA DRIVE SURULERE	Surulere	Lagos
4746	LA/1013	LA/1013/S/14	living faith spec. hospital		37 modele str off ojuelegba	Surulere	Lagos
4747	LA/1013	LA/1013/S/5	living faith spec. hospital		37 modele str off ojuelegba	Surulere	Lagos
4748	LA/1013	LA/1013/S/2	living faith spec. hospital		37 modele str off ojuelegba	Surulere	Lagos
4749	LA/1014	LA/1014/S/7	longe medical center		126, olusegun osoba road, agbado crossing	Ifako-Ijaye	Lagos
4750	LA/1014	LA/1014/S/5	longe medical center		126, olusegun osoba road, agbado crossing	Ifako-Ijaye	Lagos
4751	LA/1014	LA/1014/S/6	longe medical center		126, olusegun osoba road, agbado crossing	Ifako-Ijaye	Lagos
4752	LA/1019	LA/1019/S/5	Manifel Medical Center		192 ijegun road, ikotun	Alimosho	Lagos
4753	LA/1023	LA/1023/S/7	National Orthopaedic Hospital		120/124 ikorodu rd.	Somolu	Lagos
4754	LA/1023	LA/1023/S/5	National Orthopaedic Hospital		120/124 ikorodu rd.	Somolu	Lagos
4755	LA/1023	LA/1023/S/10	National Orthopaedic Hospital		120/124 ikorodu rd.	Somolu	Lagos
4756	LA/1023	LA/1023/S/11	National Orthopaedic Hospital		120/124 ikorodu rd.	Somolu	Lagos
4757	LA/1023	LA/1023/S/4	National Orthopaedic Hospital		120/124 ikorodu rd.	Somolu	Lagos
4758	LA/1029	LA/1029/S/6	Optimal Specialist Hospital		9 Gbaja Street Surulere	Surulere	Lagos
4759	LA/1029	LA/1029/S/3	Optimal Specialist Hospital		9 Gbaja Street Surulere	Surulere	Lagos
4760	LA/1029	LA/1029/S/5	Optimal Specialist Hospital		9 Gbaja Street Surulere	Surulere	Lagos
4761	LA/1029	LA/1029/S/4	Optimal Specialist Hospital		9 Gbaja Street Surulere	Surulere	Lagos
4762	LA/1034	LA/1034/S/5	QUINTA HOSPITAL		182, KIRIKIRI ROAD, OLODI APAPA	Ajeromi/Ifelodun	Lagos
4763	LA/1046	LA/1046/S/5	ST. CLEMENT HOSPITAL		321 RD. G-CLOSE 3RD AVENUE FESTAC TOWN	Amuwo-Odofin	Lagos
4764	LA/1046	LA/1046/S/3	ST. CLEMENT HOSPITAL		321 RD. G-CLOSE 3RD AVENUE FESTAC TOWN	Amuwo-Odofin	Lagos
4765	LA/1046	LA/1046/S/14	ST. CLEMENT HOSPITAL		321 RD. G-CLOSE 3RD AVENUE FESTAC TOWN	Amuwo-Odofin	Lagos
4766	LA/1047	LA/1047/S/7	ST. LOUIS MEDICAL CENTER		7A/9A ABIBU OKI MARINA	Lagos Island	Lagos
4767	LA/1047	LA/1047/S/5	ST. LOUIS MEDICAL CENTER		7A/9A ABIBU OKI MARINA	Lagos Island	Lagos
4768	LA/1050	LA/1050/S/4	ST.KIZITO (PHC)		ILASAN HOUSING ESTATE JAKANDE, LEKKI	Eti-Osa	Lagos
4769	LA/1050	LA/1050/S/5	ST.KIZITO (PHC)		ILASAN HOUSING ESTATE JAKANDE, LEKKI	Eti-Osa	Lagos
4770	LA/1053	LA/1053/S/5	STEPHENS HOSPITAL		4 OYEWUMI CLOSE OFF FALOLU STR. SURULERE	Surulere	Lagos
4771	LA/1054	LA/1054/S/3	General Hospital, Surulere		Randle Avenue	Surulere	Lagos
4772	LA/1054	LA/1054/S/6	General Hospital, Surulere		Randle Avenue	Surulere	Lagos
4773	LA/1054	LA/1054/S/5	General Hospital, Surulere		Randle Avenue	Surulere	Lagos

4774	LA/1054	LA/1054/S/1	General Hospital, Surulere		Randle Avenue	Surulere	Lagos
4775	LA/1054	LA/1054/S/4	General Hospital, Surulere		Randle Avenue	Surulere	Lagos
4776	LA/1054	LA/1054/S/8	General Hospital, Surulere		Randle Avenue	Surulere	Lagos
4777	LA/1054	LA/1054/S/2	General Hospital, Surulere		Randle Avenue	Surulere	Lagos
4778	LA/1054	LA/1054/S/7	General Hospital, Surulere		Randle Avenue	Surulere	Lagos
4779	LA/1059	LA/1059/S/2	UPLIFT MEDICALCENTER		15, ADMIRALTY WAY, LEKKI PHASE 1	Eti-Osa	Lagos
4780	LA/1059	LA/1059/S/5	UPLIFT MEDICALCENTER		15, ADMIRALTY WAY, LEKKI PHASE 1	Eti-Osa	Lagos
4781	LA/1059	LA/1059/S/6	UPLIFT MEDICALCENTER		15, ADMIRALTY WAY, LEKKI PHASE 1	Eti-Osa	Lagos
4782	LA/1059	LA/1059/S/7	UPLIFT MEDICALCENTER		15, ADMIRALTY WAY, LEKKI PHASE 1	Eti-Osa	Lagos
4783	LA/1059	LA/1059/S/1	UPLIFT MEDICALCENTER		15, ADMIRALTY WAY, LEKKI PHASE 1	Eti-Osa	Lagos
4784	LA/1059	LA/1059/S/4	UPLIFT MEDICALCENTER		15, ADMIRALTY WAY, LEKKI PHASE 1	Eti-Osa	Lagos
4785	LA/1059	LA/1059/S/3	UPLIFT MEDICALCENTER		15, ADMIRALTY WAY, LEKKI PHASE 1	Eti-Osa	Lagos
4786	LA/1062	LA/1062/S/15	UNITY HOSPITAL, Surulere		81, OJUELEGBA ROAD, SURULERE	Surulere	Lagos
4787	LA/1062	LA/1062/S/3	UNITY HOSPITAL, Surulere		81, OJUELEGBA ROAD, SURULERE	Surulere	Lagos
4788	LA/1062	LA/1062/S/1	UNITY HOSPITAL, Surulere		81, OJUELEGBA ROAD, SURULERE	Surulere	Lagos
4789	LA/1062	LA/1062/S/6	UNITY HOSPITAL, Surulere		81, OJUELEGBA ROAD, SURULERE	Surulere	Lagos
4790	LA/1062	LA/1062/S/5	UNITY HOSPITAL, Surulere		81, OJUELEGBA ROAD, SURULERE	Surulere	Lagos
4791	LA/1069	LA/1069/S/2	JON-KEN	Laboratory,orthopaedics,O&G,int.med,surgery,RADIOLGY,opthalmolgy,paediatrics,ENT,Urology, Dental	20, SHONIBARE STREET, AKOKA	Somolu	Lagos
4792	LA/1069	LA/1069/S/6	JON-KEN	Laboratory,orthopaedics,O&G,int.med,surgery,RADIOLGY,opthalmolgy,paediatrics,ENT,Urology, Dental	20, SHONIBARE STREET, AKOKA	Somolu	Lagos
4793	LA/1069	LA/1069/S/3	JON-KEN	Laboratory,orthopaedics,O&G,int.med,surgery,RADIOLGY,opthalmolgy,paediatrics,ENT,Urology, Dental	20, SHONIBARE STREET, AKOKA	Somolu	Lagos
4794	LA/1069	LA/1069/S/5	JON-KEN	Laboratory,orthopaedics,O&G,int.med,surgery,RADIOLGY,opthalmolgy,paediatrics,ENT,Urology, Dental	20, SHONIBARE STREET, AKOKA	Somolu	Lagos
4795	LA/1069	LA/1069/S/10	JON-KEN	Laboratory,orthopaedics,O&G,int.med,surgery,RADIOLGY,opthalmolgy,paediatrics,ENT,Urology, Dental	20, SHONIBARE STREET, AKOKA	Somolu	Lagos
4796	LA/1069	LA/1069/S/1	JON-KEN	Laboratory,orthopaedics,O&G,int.med,surgery,RADIOLGY,opthalmolgy,paediatrics,ENT,Urology, Dental	20, SHONIBARE STREET, AKOKA	Somolu	Lagos
4797	LA/1069	LA/1069/S/7	JON-KEN	Laboratory,orthopaedics,O&G,int.med,surgery,RADIOLGY,opthalmolgy,paediatrics,ENT,Urology, Dental	20, SHONIBARE STREET, AKOKA	Somolu	Lagos
4798	LA/1069	LA/1069/S/15	JON-KEN	Laboratory,orthopaedics,O&G,int.med,surgery,RADIOLGY,opthalmolgy,paediatrics,ENT,Urology, Dental	20, SHONIBARE STREET, AKOKA	Somolu	Lagos

4799	LA/1069	LA/1069/S/12	JON-KEN	Laboratory,orthopaedics,O&G,int.med,surgery,RADIOLGY,opthalmolgy,paediatrics,ENT,Urology, Dental	20, SHONIBARE STREET, AKOKA	Somolu	Lagos
4800	LA/1069	LA/1069/S/8	JON-KEN	Laboratory,orthopaedics,O&G,int.med,surgery,RADIOLGY,opthalmolgy,paediatrics,ENT,Urology, Dental	20, SHONIBARE STREET, AKOKA	Somolu	Lagos
4801	LA/1069	LA/1069/S/18	JON-KEN	Laboratory,orthopaedics,O&G,int.med,surgery,RADIOLGY,opthalmolgy,paediatrics,ENT,Urology, Dental	20, SHONIBARE STREET, AKOKA	Somolu	Lagos
4802	LA/1090	LA/1090/S/4	MARIA-JID NIG. LTD	Pharmacy	46,FASHORO ROAD,MUSHIN	Mushin	Lagos
4803	LA/1092	LA/1092/S/4	MEDIPLUS PHARMACY	Pharmacy	45A SAKA TINUBU STR. V/ISLAND	Eti-Osa	Lagos
4804	LA/1093	LA/1093/S/14	Noble Medicals (African X-ray House)	Radiology/uss	140, Okota Road,College b/stop,okota	Oshodi/Isolo	Lagos
4805	LA/1093	LA/1093/S/7	Noble Medicals (African X-ray House)	Radiology/uss	140, Okota Road,College b/stop,okota	Oshodi/Isolo	Lagos
4806	LA/1094	LA/1094/S/4	NURUSAT PHARM	Pharmacy	13,JADEOSHOLA, OSHODI STR.OFF BROWN ROAD,SURULERE	Surulere	Lagos
4807	LA/1095	LA/1095/S/5	LIFE CARE DIAGNOSTICS CENTRE	LABORATORY,USS	51/512 ROAD JUNCTION FESTAC TOWN	Amuwo-Odofin	Lagos
4808	LA/1095	LA/1095/S/14	LIFE CARE DIAGNOSTICS CENTRE	LABORATORY,USS	51/512 ROAD JUNCTION FESTAC TOWN	Amuwo-Odofin	Lagos
4809	LA/1097	LA/1097/S/7	On-George Medical Services	Radiology,USS	1A, Adeoye Fafore Street On-George B/Stop, Ikotun Road	Alimosho	Lagos
4810	LA/1097	LA/1097/S/14	On-George Medical Services	Radiology,USS	1A, Adeoye Fafore Street On-George B/Stop, Ikotun Road	Alimosho	Lagos
4811	LA/1100	LA/1100/S/8	osagie dental clinic	DENTAL	14,boyle''s street,onikan	Lagos Island	Lagos
4812	LA/1101	LA/1101/S/4	ABJ PHARMACY	Pharmacy	31 BROWN ROAD, AGUDA, SURULERE	Surulere	Lagos
4813	LA/1104	LA/1104/S/4	AJEKINS PHARMACY IFAKO	Pharmacy	11A IBRAHIM ONOSHOKUN STR BY NEPA B/STOP	Kosofe	Lagos
4814	LA/1108	LA/1108/S/5	ANOINTED MED. DIAGNOSTIC	LABORATORY	12,AROWOJOBE STR. MAFOLUKU OSHODI	Oshodi/Isolo	Lagos
4815	LA/1109	LA/1109/S/15	ANUR EYE CENTER	Opthalmology	16, AKINSEMOYIN STR OFF BODE THOMAS	Surulere	Lagos
4816	LA/1110	LA/1110/S/15	BAF EYE CENTER	Opthalmology	10, Folayemi Street, off Coker Road, Ilupeju	Mushin	Lagos
4817	LA/1114	LA/1114/S/5	BIOMEDIC DIAGNOSTIC	LABORATORY	65, ADENIRAN OGUNSANYA ,SURULERE	Surulere	Lagos
4818	LA/1117	LA/1117/S/5	COMPREHENSIVE DIAG.CENTER	Laboratory	15 BODE THOMAS STR, SURULERE	Surulere	Lagos
4819	LA/1118	LA/1118/S/8	CONTEMPORARY DENTAL CLINIC	DENTAL	63 BODE THOMAS STR, SURULERE	Surulere	Lagos
4820	LA/1122	LA/1122/S/5	DE-KING MED. LAB	Laboratory	61, ISOLO ROAD, EGBE	Alimosho	Lagos
4821	LA/1123	LA/1123/S/8	DIAMOND DENTAL	DENTAL	142, BODE THOMAS ROAD, SURULERE	Surulere	Lagos
4822	LA/1124	LA/1124/S/5	DIAMOND MED. DIAGNOSTIC	Laboratory	76, BANK ANTHONY WAY, IKEJA	Ikeja	Lagos
4823	LA/1125	LA/1125/S/5	DIAMOND MED. DIAGNOSTIC	Laboratory	88 OLD OTA ROAD, ORILE AGEGE	Agege	Lagos
4824	LA/1126	LA/1126/S/5	EBENEZER MED. DIAGNOSTIC	Laboratory	10A OLATUNDE ONIMOLE SURULERE	Surulere	Lagos
4825	LA/1128	LA/1128/S/7	EL-LAB L.T.D	Radiology/USS, Laboratory	PLOT A32 RD. SHOPPING COMPLEX FESTAC TOWN	Amuwo-Odofin	Lagos
4826	LA/1128	LA/1128/S/14	EL-LAB L.T.D	Radiology/USS, Laboratory	PLOT A32 RD. SHOPPING COMPLEX FESTAC TOWN	Amuwo-Odofin	Lagos
4827	LA/1128	LA/1128/S/5	EL-LAB L.T.D	Radiology/USS, Laboratory	PLOT A32 RD. SHOPPING COMPLEX FESTAC TOWN	Amuwo-Odofin	Lagos
4828	LA/1131	LA/1131/S/4	FIRST- EL SHADDAI HOSPITAL	Pharmacy	35 SAMUEL STREET, AKOWONJO	Alimosho	Lagos
4829	LA/1140	LA/1140/S/4	HESS NIG. LTD.	Pharmacy	130 AWOLOWO WAY, IKOYI	Eti-Osa	Lagos
4830	LA/1143	LA/1143/S/4	HOPE CEE PHARMACY	Pharmacy	6, THOMAS STREET, EBUTE METTA WEST	Lagos Mainland	Lagos
4831	LA/1144	LA/1144/S/5	INTEGRATED DIAG. LAB	LAB	22 MONTGOMERY STR. YABA	Somolu	Lagos
4832	LA/1148	LA/1148/S/7	JOY DIAGNOSTIC	Radiology	93 MUYIBI STR. OFF WILMER OLODI APAPA	Ajeromi/Ifelodun	Lagos

4833	LA/1150	LA/1150/S/6	KIDZ CARE PAEDIATRIC CLINIC	Paediatrics	SHOP G13, VGC SHOPPING COMPLEX, VGC	Eti-Osa	Lagos
4834	LA/1151	LA/1151/S/13	LADKEM EYE HOSPITAL	Opthalmology, OPTOMETRY	27, NURU PNIWO STR. AGUDA SURULERE	Surulere	Lagos
4835	LA/1151	LA/1151/S/15	LADKEM EYE HOSPITAL	Opthalmology, OPTOMETRY	27, NURU PNIWO STR. AGUDA SURULERE	Surulere	Lagos
4836	LA/1152	LA/1152/S/5	PATHFINDER DIAG. LAB	LAB	22 ROAD. K CLOSE BLK4 FLAT4 FESTAC TOWN	Amuwo-Odofin	Lagos
4837	LA/1153	LA/1153/S/4	PHARMFLASH NIG. LTD	Pharmacy	38,CEMETARY STR. EBUTE METTA EAST	Lagos Mainland	Lagos
4838	LA/1157	LA/1157/S/4	REHOBOTH PHARMACY	Pharmacy	32, ISHOLA ANIGBAJUMO OSHODI	Oshodi/Isolo	Lagos
4839	LA/1158	LA/1158/S/4	ROCK AID PHARMACY	Pharmacy	15, ADEBOLA OJOMU STR. AGUDA	Surulere	Lagos
4840	LA/1159	LA/1159/S/4	SALEM GATE PHARMACY	Pharmacy	13, ALH. MASHA ROAD, SURULERE	Surulere	Lagos
4841	LA/1161	LA/1161/S/13	SMILEY OPTICAL SERV.	OPTOMETRY	92,ADENIRAN OGUNSANYA SURULERE	Surulere	Lagos
4842	LA/1162	LA/1162/S/7	SOLAFORT DIAG. CENTER	Radiology,USS	24, IJAOYE STR. JIBOWU STR. YABA	Lagos Island	Lagos
4843	LA/1162	LA/1162/S/14	SOLAFORT DIAG. CENTER	Radiology,USS	24, IJAOYE STR. JIBOWU STR. YABA	Lagos Island	Lagos
4844	LA/1167	LA/1167/S/1	ST.PAUL'S HOSPITAL	Surgery	71A ONDO STR.EBUTE METTA	Lagos Mainland	Lagos
4845	LA/1169	LA/1169/S/7	SURULERE CLINI-X-RAY	Radiology	31, AYINDE GIWA STR. SURULERE	Surulere	Lagos
4846	LA/1170	LA/1170/S/5	THARMA PRECISION DIAG. SERV.	Laboratory	SUIT 11, ZUMA OFFICE COMPLEX,H CLOSE.FESTAC TOWN	Amuwo-Odofin	Lagos
4847	LA/1173	LA/1173/S/11	TONS PHYSIOTHERAPY CLINIC	PHYSIOTHERAPY	37, AKOBI CRESCENT, S/LERE	Surulere	Lagos
4848	LA/1174	LA/1174/S/4	TONY CLAIRE PHARMACY	Pharmacy	126,IDEWU STR. OLODI APAPA	Ajeromi/Ifelodun	Lagos
4849	LA/1175	LA/1175/S/5	TRUST MED. DIAG	LAB	21,SURULERE STR. DOPEMU AGEGE	Agege	Lagos
4850	LA/1176	LA/1176/S/4	TRIPOD PHARMACY	Pharmacy	24, AGO PALACE WAY OKOTA	Oshodi/Isolo	Lagos
4851	LA/1177	LA/1177/S/15	TRUVISION EYE CARE CENTER	Opthalmology	37, IGBOSERE ROAD, LAGOS	Lagos Island	Lagos
4852	LA/1180	LA/1180/S/4	VICTORY DRUGS	Pharmacy	PLOT 57A.11ROAD. FESTAC TOWN	Amuwo-Odofin	Lagos
4853	LA/1181	LA/1181/S/13	VISTA OPTICS L.T.D	OPTOMETRY	7, MANUWA STREET, S,W IKOYI	Eti-Osa	Lagos
4854	LA/1185	LA/1185/S/7	X-RITE DIAGNOSTIC CENTER	Radiology	10, OSAPA LONDON STR. LEKKI	Eti-Osa	Lagos
4855	LA/1189	LA/1189/S/5	Redeemed Medical Centre		Elias Close, Okokomaiko, Lagos	Ojo	Lagos
4856	LA/1190	LA/1190/S/15	T & S Hospital	Dental, Opthamology	26 Bombay Crescent, Apapa, Lagos	Apapa	Lagos
4857	LA/1190	LA/1190/S/8	T & S Hospital	Dental, Opthamology	26 Bombay Crescent, Apapa, Lagos	Apapa	Lagos
4858	LA/1192	LA/1192/S/4	Golbyhealth Care Ltd.	Pharmacy	3.Oladoyinbo Str. By Avis Bus Stop Aguda-Ogba. Lagos	Agege	Lagos
4859	LA/1193	LA/1193/S/1	Healthwise Initiatives Ltd.	Pharmacy	37b Coker Road, Ilupeju, Lagos.	Mushin	Lagos
4860	LA/1197	LA/1197/S/1	AFRIMED SPECIALIST HOSPITAL		1 Williams Street, off Diya Street, Sawmill, Gbagada Lagos.	Somolu	Lagos
4861	LA/1197	LA/1197/S/6	AFRIMED SPECIALIST HOSPITAL		1 Williams Street, off Diya Street, Sawmill, Gbagada Lagos.	Somolu	Lagos
4862	LA/1197	LA/1197/S/2	AFRIMED SPECIALIST HOSPITAL		1 Williams Street, off Diya Street, Sawmill, Gbagada Lagos.	Somolu	Lagos
4863	LA/1197	LA/1197/S/3	AFRIMED SPECIALIST HOSPITAL		1 Williams Street, off Diya Street, Sawmill, Gbagada Lagos.	Somolu	Lagos
4864	LA/1199	LA/1199/S/6	Folabi Medical Center		78 Oworonsoki Road, Labule Bus Stop, Lagos	Kosofe	Lagos
4865	LA/1199	LA/1199/S/1	Folabi Medical Center		78 Oworonsoki Road, Labule Bus Stop, Lagos	Kosofe	Lagos
4866	LA/1199	LA/1199/S/3	Folabi Medical Center		78 Oworonsoki Road, Labule Bus Stop, Lagos	Kosofe	Lagos
4867	LA/1200	LA/1200/S/8	Concise Diagnostic Centre	Laboratory	14 Akinbayode Street, Papa Ajao, Mushin, Lagos	Mushin	Lagos
4868	LA/1201	LA/1201/S/6	Jim-Sam Hospital & Maternity		22 Gaskiya College Road, Ijora Badia, Apapa, Lagos	Apapa	Lagos
4869	LA/1201	LA/1201/S/1	Jim-Sam Hospital & Maternity		22 Gaskiya College Road, Ijora Badia, Apapa, Lagos	Apapa	Lagos
4870	LA/1201	LA/1201/S/3	Jim-Sam Hospital & Maternity		22 Gaskiya College Road, Ijora Badia, Apapa, Lagos	Apapa	Lagos
4871	LA/1201	LA/1201/S/5	Jim-Sam Hospital & Maternity		22 Gaskiya College Road, Ijora Badia, Apapa, Lagos	Apapa	Lagos
4872	LA/1214	LA/1214/S/13	Dena & Kich Eye Clinic	Optometry	41 Osolo Way Off M/M Airport, Ajao Estate	Oshodi/Isolo	Lagos
4873	LA/1217	LA/1217/S/13	Common Sight Ltd	Optometry	82 Old Ojo Road, Kuje, Amuwo	Amuwo-Odofin	Lagos

4874	LA/1224	LA/1224/S/5	Jim Sim Hospital & Maternity		22 College Road, Ijora Badia, Ajao Estate	Amuwo-Odofin	Lagos
4875	LA/1224	LA/1224/S/1	Jim Sim Hospital & Maternity		22 College Road, Ijora Badia, Ajao Estate	Amuwo-Odofin	Lagos
4876	LA/1224	LA/1224/S/3	Jim Sim Hospital & Maternity		22 College Road, Ijora Badia, Ajao Estate	Amuwo-Odofin	Lagos
4877	LA/1224	LA/1224/S/6	Jim Sim Hospital & Maternity		22 College Road, Ijora Badia, Ajao Estate	Amuwo-Odofin	Lagos
4878	LA/1229	LA/1229/S/1	Rockaid Pharmacy Store Limited	Pharmacy	15 Adebola Ojomu Street, Aguda	Abakaliki	Ebonyi
4879	LA/1230	LA/1230/S/1	Specmakers Opthalmic Services	Optometry	129 Okota Road Near Cele B/Stop	Oshodi/Isolo	Lagos
4880	LA/1232	LA/1232/S/15	Adetula Eye Clinic	Opthamology	46 Shasha Akowonjo	Alimosho	Lagos
4881	LA/1233	LA/1233/S/4	DE-Health Link	Pharmacy, Laboratory	125 Dopemu Road, Agege	Agege	Lagos
4882	LA/1233	LA/1233/S/5	DE-Health Link	Pharmacy, Laboratory	125 Dopemu Road, Agege	Agege	Lagos
4883	LA/1234	LA/1234/S/15	Phaton Best Eye & Optical Centre	Opthamology	185 Ikorodu Road, Palmgrove	Lagos Mainland	Lagos
4884	LA/1235	LA/1235/S/5	Island Diagnostic	Laboratory	44 Obalende Road, Obalende	Eti-Osa	Lagos
4885	LA/1237	LA/1237/S/15	Truvision Limited	Opthamology	84 Ozumba Mbadiwe, Victoria Island	Eti-Osa	Lagos
4886	LA/1239	LA/1239/S/8	Billings Dental Clinic	Dental	Plot 26, Block 66, Kayode Taiwo Street, Magodo, GRA,	Ikeja	Lagos
4887	LA/1241	LA/1241/S/3	Good Seed Specialist Clinics	O&G	16 Okeho Street Ire-Akari Estate	Oshodi/Isolo	Lagos
4888	LA/1242	LA/1242/S/5	Ajayi Memorial Hospital	Laboratory	159 Moshood Abiola Way, Ebute Metta	Lagos Mainland	Lagos
4889	LA/1245	LA/1245/S/6	Inland Specialist Hospital	General Surgery, Laboratory, O & G, Paediatrics, Pharmacy	11 Bola Owodunni Street, Behind Eskay Filling Station, Alapere, Ketu	Kosofe	Lagos
4890	LA/1245	LA/1245/S/5	Inland Specialist Hospital	General Surgery, Laboratory, O & G, Paediatrics, Pharmacy	11 Bola Owodunni Street, Behind Eskay Filling Station, Alapere, Ketu	Kosofe	Lagos
4891	LA/1245	LA/1245/S/4	Inland Specialist Hospital	General Surgery, Laboratory, O & G, Paediatrics, Pharmacy	11 Bola Owodunni Street, Behind Eskay Filling Station, Alapere, Ketu	Kosofe	Lagos
4892	LA/1245	LA/1245/S/3	Inland Specialist Hospital	General Surgery, Laboratory, O & G, Paediatrics, Pharmacy	11 Bola Owodunni Street, Behind Eskay Filling Station, Alapere, Ketu	Kosofe	Lagos
4893	LA/1245	LA/1245/S/1	Inland Specialist Hospital	General Surgery, Laboratory, O & G, Paediatrics, Pharmacy	11 Bola Owodunni Street, Behind Eskay Filling Station, Alapere, Ketu	Kosofe	Lagos
4894	LA/1246	LA/1246/S/3	Golden Victory Hospital	O&G, Paediatrics	49B Shasha Road, Shasha	Alimosho	Lagos
4895	LA/1246	LA/1246/S/6	Golden Victory Hospital	O&G, Paediatrics	49B Shasha Road, Shasha	Alimosho	Lagos
4896	LA/1249	LA/1249/S/8	Benjo Dental Clinic & Othopedic Practice	Dental	30 Adekunle Fajuyi Way	Ikeja	Lagos
4897	LA/1251	LA/1251/S/13	First Eye Clinic	Optometry	66 Ijaiye Road, Ogba B/stop, Ikeja	Ikeja	Lagos
4898	LA/1253	LA/1253/S/5	Nigerian Institute of Medical Research	Laboratory	6 Edmund Crescent, Off M/Mohammed Way, Yaba	Lagos Mainland	Lagos
4899	LA/1254	LA/1254/S/8	Asman Dental	Dental	10 Bilikisu Iyede Street, Onike Roundabout	Lagos Mainland	Lagos
4900	LA/1255	LA/1255/S/12	Royal Saints Specialist Hospital	ENT	Raka Plaza, Plot 1439, Sanusi Fafunwa, Victoria Island	Eti-Osa	Lagos
4901	LA/1257	LA/1257/S/5	Holy Rosary Medical Centre		321 Road A Close House 18, Festac Town	Amuwo-Odofin	Lagos
4902	LA/1259	LA/1259/S/1	Duro Soleye Hospital		34 Allen Avenue, Ikeja	Ikeja	Lagos
4903	LA/1259	LA/1259/S/8	Duro Soleye Hospital		34 Allen Avenue, Ikeja	Ikeja	Lagos
4904	LA/1259	LA/1259/S/2	Duro Soleye Hospital		34 Allen Avenue, Ikeja	Ikeja	Lagos
4905	LA/1260	LA/1260/S/5	Yombo Hospital and Maternity Home		23 Oluyombo Street, Ikosi, Ketu	Kosofe	Lagos
4906	LA/1260	LA/1260/S/1	Yombo Hospital and Maternity Home		23 Oluyombo Street, Ikosi, Ketu	Kosofe	Lagos
4907	LA/1260	LA/1260/S/3	Yombo Hospital and Maternity Home		23 Oluyombo Street, Ikosi, Ketu	Kosofe	Lagos
4908	LA/1261	LA/1261/S/4	Fellowship Hospital Ltd		9 Oliyide Street, Off Unity Road Ikeja	Ikeja	Lagos
4909	LA/1261	LA/1261/S/3	Fellowship Hospital Ltd		9 Oliyide Street, Off Unity Road Ikeja	Ikeja	Lagos

4910	LA/1261	LA/1261/S/1	Fellowship Hospital Ltd		9 Oliyide Street, Off Unity Road Ikeja	Ikeja	Lagos
4911	LA/1261	LA/1261/S/5	Fellowship Hospital Ltd		9 Oliyide Street, Off Unity Road Ikeja	Ikeja	Lagos
4912	LA/1270	LA/1270/S/1	Santa Maria Hospital		10, Santa Maria Street, Egan Igando	Alimosho	Lagos
4913	LA/1270	LA/1270/S/4	Santa Maria Hospital		10, Santa Maria Street, Egan Igando	Alimosho	Lagos
4914	LA/1270	LA/1270/S/2	Santa Maria Hospital		10, Santa Maria Street, Egan Igando	Alimosho	Lagos
4915	LA/1270	LA/1270/S/7	Santa Maria Hospital		10, Santa Maria Street, Egan Igando	Alimosho	Lagos
4916	LA/1270	LA/1270/S/3	Santa Maria Hospital		10, Santa Maria Street, Egan Igando	Alimosho	Lagos
4917	LA/1273	LA/1273/S/5	Junction Bus Stop Diagnostic Centre,	Laboratory	66 Oworo Road, Bus Stop, Oworonsoki, Lagos	Kosofe	Lagos
4918	LA/1275	LA/1275/S/3	Jaiyeola clinic		4 Bankole Street by Famous Bust Stop, Pedro, Shomolu, Bariga	Somolu	Lagos
4919	LA/1275	LA/1275/S/2	Jaiyeola clinic		4 Bankole Street by Famous Bust Stop, Pedro, Shomolu, Bariga	Somolu	Lagos
4920	LA/1275	LA/1275/S/1	Jaiyeola clinic		4 Bankole Street by Famous Bust Stop, Pedro, Shomolu, Bariga	Somolu	Lagos
4921	LA/1278	LA/1278/S/1	Tinu Hospital	Surgery, O&G, Laboratory	86 Mushin Road, Isolo	Oshodi/Isolo	Lagos
4922	LA/1278	LA/1278/S/3	Tinu Hospital	Surgery, O&G, Laboratory	86 Mushin Road, Isolo	Oshodi/Isolo	Lagos
4923	LA/1278	LA/1278/S/5	Tinu Hospital	Surgery, O&G, Laboratory	86 Mushin Road, Isolo	Oshodi/Isolo	Lagos
4924	LA/1279	LA/1279/S/13	Dunamis Eye Centre	Optometry	203 Igbosere Road, Lagos Island	Lagos Island	Lagos
4925	LA/1280	LA/1280/S/8	Don Dental Clinic	Dentistry	3 Randle Road, Apapa, Lagos	Apapa	Lagos
4926	LA/1281	LA/1281/S/8	Rabboni Dental Clinic	Dentistry	Suite 22 & 23 Ultimate Plaza, 21 Road, Festac Town, Lagos	Amuwo-Odofin	Lagos
4927	LA/1282	LA/1282/S/5	Health Point Diagnostic Centre	Laboratory	73 College Road, Estate Bus Stop, Ogba, Lagos	Ifako-Ijaye	Lagos
4928	LA/1283	LA/1283/S/3	A. B. 10 Hospital		2 Adeleke Odunuga Close, Harmony Estate, Ifako-Ijaye	Ifako-Ijaye	Lagos
4929	LA/1283	LA/1283/S/4	A. B. 10 Hospital		2 Adeleke Odunuga Close, Harmony Estate, Ifako-Ijaye	Ifako-Ijaye	Lagos
4930	LA/1286	LA/1286/S/7	Yaba College of Technology Medical Centre		Herbert Macaulay Way, Yaba	Lagos Mainland	Lagos
4931	LA/1286	LA/1286/S/5	Yaba College of Technology Medical Centre		Herbert Macaulay Way, Yaba	Lagos Mainland	Lagos
4932	LA/1286	LA/1286/S/4	Yaba College of Technology Medical Centre		Herbert Macaulay Way, Yaba	Lagos Mainland	Lagos
4933	LA/1290	LA/1290/S/5	Angel Hospital		20 Ecwa Church Road, Coker Village, Orile Iganmu	Surulere	Lagos
4934	LA/1290	LA/1290/S/14	Angel Hospital		20 Ecwa Church Road, Coker Village, Orile Iganmu	Surulere	Lagos
4935	LA/1299	LA/1299/S/1	Immanuel (M) Hospital		19 Adebayo Mokuolu Street, Anthony Village, Lagos	Kosofe	Lagos
4936	LA/1299	LA/1299/S/3	Immanuel (M) Hospital		19 Adebayo Mokuolu Street, Anthony Village, Lagos	Kosofe	Lagos
4937	LA/1304	LA/1304/S/3	St Mary's Specialist Hospital		Plot 458 Titilayo Adedoyin Street, Omole Phase 1, Ikeja	Ikeja	Lagos
4938	LA/1304	LA/1304/S/6	St Mary's Specialist Hospital		Plot 458 Titilayo Adedoyin Street, Omole Phase 1, Ikeja	Ikeja	Lagos
4939	LA/1311	LA/1311/S/5	Blue Cross Hospital		48 Ijaiye Road, Ogba	Ifako-Ijaye	Lagos
4940	LA/1311	LA/1311/S/7	Blue Cross Hospital		48 Ijaiye Road, Ogba	Ifako-Ijaye	Lagos
4941	LA/1313	LA/1313/S/5	P & G Medical Centre		29 Sanni Labode Street, New Oko-Oba, Lagos	Ifako-Ijaye	Lagos
4942	LA/1315	LA/1315/S/5	Tolu Medical Centre		25 Amodu Tijani Street, Tolu Bus Stop, Olodi Apapa	Apapa	Lagos
4943	LA/1315	LA/1315/S/14	Tolu Medical Centre		25 Amodu Tijani Street, Tolu Bus Stop, Olodi Apapa	Apapa	Lagos
4944	LA/1317	LA/1317/S/5	Option Medical Laboratory	Laboratory	197/201 Ojo Road, Ajegunle	Ajeromi/Ifelodun	Lagos
4945	LA/1318	LA/1318/S/5	Foresight Diagnostic Centre	Laboratory	64 Ilogbo Road, Ajangbadi, Lagos	Ojo	Lagos
4946	LA/1323	LA/1323/S/5	Rikky Hospital		193/197 Ojo Road Ajegunle	Ajeromi/Ifelodun	Lagos

4947	LA/1327	LA/1327/S/5	Biomass Nigeria Limited		3 Old Isheri Ijaye Road Aguda	Ikeja	Lagos
4948	LA/1333	LA/1333/S/2	Dialyzer Medical Centre		60, Arowojobe Street, Oseni Bus Stop, Oshodi	Oshodi/Isolo	Lagos
4949	LA/1333	LA/1333/S/5	Dialyzer Medical Centre		60, Arowojobe Street, Oseni Bus Stop, Oshodi	Oshodi/Isolo	Lagos
4950	LA/1333	LA/1333/S/1	Dialyzer Medical Centre		60, Arowojobe Street, Oseni Bus Stop, Oshodi	Oshodi/Isolo	Lagos
4951	LA/1333	LA/1333/S/3	Dialyzer Medical Centre		60, Arowojobe Street, Oseni Bus Stop, Oshodi	Oshodi/Isolo	Lagos
4952	LA/1334	LA/1334/S/3	Molayo Medical Centre		20 Akinyele Street, Oko Oba	Agege	Lagos
4953	LA/1335	LA/1335/S/5	Nigeria French Language Clinic		Nigeria French Village, Badagry	Badagry	Lagos
4954	LA/1335	LA/1335/S/4	Nigeria French Language Clinic		Nigeria French Village, Badagry	Badagry	Lagos
4955	LA/1340	LA/1340/S/8	Ave Dental Clinic	Dental	68 Ago Palace Way Okota, Lagos	Oshodi/Isolo	Lagos
4956	LA/1346	LA/1346/S/4	Merit Pharmacy		1 Toyin Street, Ikeja	Ikeja	Lagos
4957	LA/1348	LA/1348/S/4	You and Lord Nig Ltd Pharmaceutical	Pharmacy	73 College Road, Ifako, Agege	Ifako-Ijaye	Lagos
4958	LA/1356	LA/1356/S/3	Eromon Specialist Hospital		47 Ganiyat Street, Monkey Village, Mazamaza, near Navy gate	Amuwo-Odofin	Lagos
4959	LA/1358	LA/1358/S/8	Lancet Dental & Eye clinic	Dental	41 Old Ojo Road, Maza-Maza	Amuwo-Odofin	Lagos
4960	LA/1363	LA/1363/S/5	Sentinel Hospital	Laboratory	1 Benson Akinyele Street, Okota	Oshodi/Isolo	Lagos
4961	LA/1363	LA/1363/S/1	Sentinel Hospital	Laboratory	1 Benson Akinyele Street, Okota	Oshodi/Isolo	Lagos
4962	LA/1363	LA/1363/S/3	Sentinel Hospital	Laboratory	1 Benson Akinyele Street, Okota	Oshodi/Isolo	Lagos
4963	LA/1364	LA/1364/S/13	Bose Eye Clinic	Optometry	102A Adeniji Jones, Ikeja	Ikeja	Lagos
4964	LA/1368	LA/1368/S/1	House of Care Hospital		1 Ajayi Street off Ogundana Street, Allen Avenue, Ikeja	Ikeja	Lagos
4965	LA/1370	LA/1370/S/5	Royan Hospital		7 Aina Street Ojodu	Ikeja	Lagos
4966	LA/1370	LA/1370/S/3	Royan Hospital		7 Aina Street Ojodu	Ikeja	Lagos
4967	LA/1370	LA/1370/S/6	Royan Hospital		7 Aina Street Ojodu	Ikeja	Lagos
4968	LA/1370	LA/1370/S/7	Royan Hospital		7 Aina Street Ojodu	Ikeja	Lagos
4969	LA/1370	LA/1370/S/1	Royan Hospital		7 Aina Street Ojodu	Ikeja	Lagos
4970	LA/1373	LA/1373/S/3	Bernice Clinic & Maternity		No. 5 Fadare Street, Off Adedoyin Street Kosofe, Mile 12, Ketu, Lagos	Kosofe	Lagos
4971	LA/1386	LA/1386/S/5	El-Bethel Hospital		323, Old Ojo Road, Oluti Bus Stop	Amuwo-Odofin	Lagos
4972	LA/1386	LA/1386/S/1	El-Bethel Hospital		323, Old Ojo Road, Oluti Bus Stop	Amuwo-Odofin	Lagos
4973	LA/1387	LA/1387/S/7	First City Diagnosis		Plot 3, Jerry Iriabe Street, Lekki Phase 1	Eti-Osa	Lagos
4974	LA/1399	LA/1399/S/3	First Graceland Hospital		KM 42, Lekki-Epe Express Way, Abijoh Aja	Ibeju/Lekki	Lagos
4975	LA/1417	LA/1417/S/5	Sam Medical Laboratory		30/32 Adenle Street, Alfa-Nla, Oke Koto, Agege	Agege	Lagos
4976	LA/1418	LA/1418/S/8	The Olives Dental Clinic		30/32 Adenle Street, Alfa-Nla, Oke Koto, Agege	Agege	Lagos
4977	LA/1419	LA/1419/S/7	Esteem Diagnostic Centre		142 Herbert Macaulay Way, Yaba	Lagos Mainland	Lagos
4978	LA/1420	LA/1420/S/15	Cathem Eye Hospital		78, Adeniyi Jones, Ikeja	Ikeja	Lagos
4979	LA/1421	LA/1421/S/5	Bepo Exact Medical Diagnostics		610 Abeokuta Express Way, Ijaiye	Ifako-Ijaye	Lagos
4980	LA/1421	LA/1421/S/7	Bepo Exact Medical Diagnostics		610 Abeokuta Express Way, Ijaiye	Ifako-Ijaye	Lagos
4981	LA/1422	LA/1422/S/6	The Shepherd Medical Centre		2 Salvation Road Opebi	Ikeja	Lagos
4982	LA/1423	LA/1423/S/12	Baaji Specialist Clinic		11 Emina Crescent, Off Toyin Street	Ikeja	Lagos
4983	LA/1424	LA/1424/S/5	Nkrees Lab & Diagnostic Centre		54, Old Ojo Road, Mosholashi Bus Stop, Kuje	Amuwo-Odofin	Lagos
4984	LA/1425	LA/1425/S/5	CJ Diamond Medical Diagnostic Centre		76, Mobolaji Bank Anthony Way	Ikeja	Lagos
4985	LA/1426	LA/1426/S/3	Jaycee Ojini Specialist Hospital		26 Adedapo Street, Tejuosho Surulere	Abakaliki	Ebonyi
4986	LA/1426	LA/1426/S/2	Jaycee Ojini Specialist Hospital		26 Adedapo Street, Tejuosho Surulere	Abakaliki	Ebonyi
4987	LA/1427	LA/1427/S/4	Sanlix Pharmacy		512 Road, F Close, House 8, Festac	Amuwo-Odofin	Lagos
4988	LA/1428	LA/1428/S/15	Radiant Optics		101 Allen Avenue,	Ikeja	Lagos
4989	LA/1434	LA/1434/S/4	Aviation Medical Clinics Headquarters (FAAN)		Murtala Mohammed Way, Domestic Airport, Ikeja	Ikeja	Lagos
4990	LA/1434	LA/1434/S/5	Aviation Medical Clinics Headquarters (FAAN)		Murtala Mohammed Way, Domestic Airport, Ikeja	Ikeja	Lagos
4991	LA/1436	LA/1436/S/5	Bob Specialist Hospitals		22 Olugbede Street, off Aeor Road, Egbeda Estate	Alimosho	Lagos

4992	LA/1436	LA/1436/S/3	Bob Specialist Hospitals		22 Olugbede Street, off Aeor Road, Egbeda Estate	Alimosho	Lagos
4993	LA/1437	LA/1437/S/4	Chemart Pharmacy Limited		Centage Plaza, 14 Allen Avenue. Ikeja	Ikeja	Lagos
4994	LA/1440	LA/1440/S/1	Britannia Hospital		Block 91, Plot 13 Aliu Animashaun Avenue, Lekki	Eti-Osa	Lagos
4995	LA/1440	LA/1440/S/3	Britannia Hospital		Block 91, Plot 13 Aliu Animashaun Avenue, Lekki	Eti-Osa	Lagos
4996	LA/1440	LA/1440/S/6	Britannia Hospital		Block 91, Plot 13 Aliu Animashaun Avenue, Lekki	Eti-Osa	Lagos
4997	LA/1440	LA/1440/S/2	Britannia Hospital		Block 91, Plot 13 Aliu Animashaun Avenue, Lekki	Eti-Osa	Lagos
4998	LA/1442	LA/1442/S/5	Bolakunmi-Idowu Memorial Medical Center		5 Segun Ishola Street, Owuwu, Ikorodu	Ikorodu	Lagos
4999	LA/1448	LA/1448/S/1	Ohbee Hospital		1, Kudaki Road, Hostel Bus stop, Egbe, Lagos, Lagos state	Alimosho	Lagos
5000	LA/1448	LA/1448/S/3	Ohbee Hospital		1, Kudaki Road, Hostel Bus stop, Egbe, Lagos, Lagos state	Alimosho	Lagos
5001	LA/1448	LA/1448/S/10	Ohbee Hospital		1, Kudaki Road, Hostel Bus stop, Egbe, Lagos, Lagos state	Alimosho	Lagos
5002	LA/1449	LA/1449/S/3	Rivet Specialist Hospital		2/3 Cobham Street, Off Olutosin Ajayi Street, Ajao Estate, Lagos State	Oshodi/Isolo	Lagos
5003	LA/1449	LA/1449/S/2	Rivet Specialist Hospital		2/3 Cobham Street, Off Olutosin Ajayi Street, Ajao Estate, Lagos State	Oshodi/Isolo	Lagos
5004	LA/1449	LA/1449/S/6	Rivet Specialist Hospital		2/3 Cobham Street, Off Olutosin Ajayi Street, Ajao Estate, Lagos State	Oshodi/Isolo	Lagos
5005	LA/1449	LA/1449/S/1	Rivet Specialist Hospital		2/3 Cobham Street, Off Olutosin Ajayi Street, Ajao Estate, Lagos State	Oshodi/Isolo	Lagos
5006	LA/1451	LA/1451/S/3	Ambassador Medical Clinics		38/44 Mashalashi street off St. Gregory Road, Obalende, Ikoyi, Lagos State	Eti-Osa	Lagos
5007	LA/1451	LA/1451/S/1	Ambassador Medical Clinics		38/44 Mashalashi street off St. Gregory Road, Obalende, Ikoyi, Lagos State	Eti-Osa	Lagos
5008	LA/1454	LA/1454/S/13	Sparkling Eyes and Optical Services		16, Ibokun Street, Off Coker Road, By Town Planning Way, Ilupeju, Lagos State	Mushin	Lagos
5009	LA/1455	LA/1455/S/6	Chinaza Specialist Hospital		2, Hakeem Onitiri Street, Off Ali Dada Str,Off Ago Palace Way, Okota, Lagos State	Oshodi/Isolo	Lagos
5010	LA/1455	LA/1455/S/1	Chinaza Specialist Hospital		2, Hakeem Onitiri Street, Off Ali Dada Str,Off Ago Palace Way, Okota, Lagos State	Oshodi/Isolo	Lagos
5011	LA/1455	LA/1455/S/3	Chinaza Specialist Hospital		2, Hakeem Onitiri Street, Off Ali Dada Str,Off Ago Palace Way, Okota, Lagos State	Oshodi/Isolo	Lagos
5012	LA/1458	LA/1458/S/5	King Solomon Hospital		4b Bola Crescent, Anthony Village, Somolu ,Lagos State	Kosofe	Lagos
5013	LA/1458	LA/1458/S/2	King Solomon Hospital		4b Bola Crescent, Anthony Village, Somolu ,Lagos State	Kosofe	Lagos
5014	LA/1458	LA/1458/S/3	King Solomon Hospital		4b Bola Crescent, Anthony Village, Somolu ,Lagos State	Kosofe	Lagos
5015	LA/1458	LA/1458/S/7	King Solomon Hospital		4b Bola Crescent, Anthony Village, Somolu ,Lagos State	Kosofe	Lagos
5016	LA/1459	LA/1459/S/13	Eye spec opticals		25A, Toyin street, Ikeja, Lagos State	Ikeja	Lagos
5017	LA/1462	LA/1462/S/3	Able God Hospital and Maternity Home		52, Ijesha Road, Opp Mobil Petrol Station, Adedeji, B/stop, ijeshatedo Lagos State	Surulere	Lagos
5018	LA/1462	LA/1462/S/1	Able God Hospital and Maternity Home		52, Ijesha Road, Opp Mobil Petrol Station, Adedeji, B/stop, ijeshatedo Lagos State	Surulere	Lagos
5019	LA/1463	LA/1463/S/3	Crest Hospital		156/160 Isuti Road, Oremeji B/Stop, Egan, Lagos State	Alimosho	Lagos

5020	LA/1465	LA/1465/S/13	Providence Optometric Clinic		6, Pearse Street,Off Ecobank Near Tejuosho Market, Yaba, Lagos State	Lagos Mainland	Lagos
5021	LA/1467	LA/1467/S/4	Pharcept Hospital		Plot 74a, Block K, Olajomehin Road, Home of Grace B/Stop, Agbede Meeting, Ikorodu, Lagos State	Ikorodu	Lagos
5022	LA/1467	LA/1467/S/5	Pharcept Hospital		Plot 74a, Block K, Olajomehin Road, Home of Grace B/Stop, Agbede Meeting, Ikorodu, Lagos State	Ikorodu	Lagos
5023	LA/1467	LA/1467/S/1	Pharcept Hospital		Plot 74a, Block K, Olajomehin Road, Home of Grace B/Stop, Agbede Meeting, Ikorodu, Lagos State	Ikorodu	Lagos
5024	LA/1470	LA/1470/S/5	Ore Laboratory Services		4, Olatilewa Street, Lawanson, 1st Floor, Surulere, (Beside SS Mulumba & David Catholic Church) , Lagos State	Surulere	Lagos
5025	LA/1472	LA/1472/S/5	St. Catherine of Siena Medical Centre		45/49 Old Ewu Road, Mafoluku, Oshodi, Lagos State	Oshodi/Isolo	Lagos
5026	LA/1474	LA/1474/S/8	First Consultant Dental Clinic		20/22 Ilupeju bye pass (Teju Hospital Premises) Ilupeju estate, Lagos State	Mushin	Lagos
5027	LA/1475	LA/1475/S/3	Standard Lifecare Clinic		5, Paul Street, Off Sanni Balogun,Off Egba Road, New Oko Oba, Abule Egba Lagos State	Ifako-Ijaye	Lagos
5028	LA/1476	LA/1476/S/8	Evans Dental Services LTD		13A, Ajayi road, Off Yaya Abatan road, Ogba, Ikeja, Lagos State	Ifako-Ijaye	Lagos
5029	LA/1479	LA/1479/S/8	Iwosan Dental Clinic		73, itire road, surulere, Lagos State	Surulere	Lagos
5030	LA/1480	LA/1480/S/5	Beulah medical Laboratory services		Beulah Plaza: bethel suite; 14, Saint Finbarr's college road, Pako b/stop, Akoka, Yaba, Lagos State	Somolu	Lagos
5031	LA/1480	LA/1480/S/4	Beulah medical Laboratory services		Beulah Plaza: bethel suite; 14, Saint Finbarr's college road, Pako b/stop, Akoka, Yaba, Lagos State	Somolu	Lagos
5032	LA/1483	LA/1483/S/3	Caring Trust Medical Clinic		59, Jubrila Street, Johnson Bus Stop, Ijeshatedo, Lagos State	Surulere	Lagos
5033	LA/1484	LA/1484/S/13	ROYALTY EYE CLINIC		48, olorunlogbon street, ANTHONY VILLAGE, Lagos State	Kosofe	Lagos
5034	LA/1485	LA/1485/S/11	Ageless Physiotherapy Clinic		19b, Ogundana Street, Hilton Bus stop, Allen Avenue, Ikeja Lagos State	Ikeja	Lagos
5035	LA/1490	LA/1490/S/11	Omega Physiotherapy and Backpain Clinic		God is Able Plaza, 24 Road, Festac, Lagos State	Amuwo-Odofin	Lagos
5036	LA/1491	LA/1491/S/14	Tropical Diagnostic development center limited		223, Old Abeokuta motor road, opp railway police station, Pen Cinema, Lagos State	Agege	Lagos
5037	LA/1491	LA/1491/S/13	Tropical Diagnostic development center limited		223, Old Abeokuta motor road, opp railway police station, Pen Cinema, Lagos State	Agege	Lagos
5038	LA/1491	LA/1491/S/5	Tropical Diagnostic development center limited		223, Old Abeokuta motor road, opp railway police station, Pen Cinema, Lagos State	Agege	Lagos
5039	LA/1492	LA/1492/S/7	Agape Care Diagnostic Services		52, Ago Palace Way, Popoola B/Stop, Okota, Isolo - Lagos, Lagos State	Oshodi/Isolo	Lagos
5040	LA/1492	LA/1492/S/5	Agape Care Diagnostic Services		52, Ago Palace Way, Popoola B/Stop, Okota, Isolo - Lagos, Lagos State	Oshodi/Isolo	Lagos
5041	LA/1494	LA/1494/S/1	Renaissance Medical		12, Adeola Odeku Victoria Island, Lagos State	Eti-Osa	Lagos
5042	LA/1494	LA/1494/S/2	Renaissance Medical		12, Adeola Odeku Victoria Island, Lagos State	Eti-Osa	Lagos

5043	LA/1495	LA/1495/S/5	A. J Rapha Diagnostic Center		10, Bashiru Street behind G.T. Bank, OJodu, Lagos State	Ikeja	Lagos
5044	LA/1495	LA/1495/S/7	A. J Rapha Diagnostic Center		10, Bashiru Street behind G.T. Bank, OJodu, Lagos State	Ikeja	Lagos
5045	LA/1504	LA/1504/S/8	Divine Care Dental clinic		Unit 2, LSPDC Complex, Gbaja Road, Off Akere Rd, Surulere, Lagos State	Surulere	Lagos
5046	LA/1505	LA/1505/S/4	Mascot pharmacy and stores		1, Munilari close, Cele Bus stop, Church Street, Shagari Estate, Lagos State	Alimosho	Lagos
5047	LA/1506	LA/1506/S/4	In God we trust pharmacy		2, Church close, Off Babalola Egbeda, Lagos State	Alimosho	Lagos
5048	LA/1507	LA/1507/S/8	Celon dental clinic		2, Olayemi street, off Nnobi street, Kilo bus stop, ikate surulere, Lagos State	Surulere	Lagos
5049	LA/1510	LA/1510/S/9	Mayfield Medical Clinic		308 Borno Way, Alagomeji, Yaba Lagos State	Lagos Mainland	Lagos
5050	LA/1513	LA/1513/S/1	Dr Abimbola Awoniyi Memorial Hospital		183 Bamgbose street Lagos Island, Lagos State	Lagos Island	Lagos
5051	LA/1513	LA/1513/S/5	Dr Abimbola Awoniyi Memorial Hospital		183 Bamgbose street Lagos Island, Lagos State	Lagos Island	Lagos
5052	LA/1513	LA/1513/S/2	Dr Abimbola Awoniyi Memorial Hospital		183 Bamgbose street Lagos Island, Lagos State	Lagos Island	Lagos
5053	LA/1513	LA/1513/S/6	Dr Abimbola Awoniyi Memorial Hospital		183 Bamgbose street Lagos Island, Lagos State	Lagos Island	Lagos
5054	LA/1513	LA/1513/S/3	Dr Abimbola Awoniyi Memorial Hospital		183 Bamgbose street Lagos Island, Lagos State	Lagos Island	Lagos
5055	LA/1513	LA/1513/S/7	Dr Abimbola Awoniyi Memorial Hospital		183 Bamgbose street Lagos Island, Lagos State	Lagos Island	Lagos
5056	LA/1520	LA/1520/S/8	Alalade Memorial Hospital		No 67, Oshodi- Abeokuta Express Road, Dopemu, Ikeja, Lagos State	Ikeja	Lagos
5057	LA/1520	LA/1520/S/15	Alalade Memorial Hospital		No 67, Oshodi- Abeokuta Express Road, Dopemu, Ikeja, Lagos State	Ikeja	Lagos
5058	LA/1520	LA/1520/S/5	Alalade Memorial Hospital		No 67, Oshodi- Abeokuta Express Road, Dopemu, Ikeja, Lagos State	Ikeja	Lagos
5059	LA/1529	LA/1529/S/13	Modern eye clinic		plot 16/18, Isheri road Opp. Omole estate, Akiode bus stop, Ojodu, Lagos State	Ojo	Lagos
5060	LA/1530	LA/1530/S/15	Sight Centre Ltd		11 Olubi Str, Opp Town Hall Gate, Ita Elewa, Ikorodu, Lagos State	Ikorodu	Lagos
5061	LA/1531	LA/1531/S/8	Family Dental Care		92, Awolowo road, Ikoyi, Lagos State	Eti-Osa	Lagos
5062	LA/1533	LA/1533/S/6	St. Anthony's Medical Centre & Maternity Home		19, Lagos -Badagry Express Way Ojo, Lagos State	Ojo	Lagos
5063	LA/1533	LA/1533/S/10	St. Anthony's Medical Centre & Maternity Home		19, Lagos -Badagry Express Way Ojo, Lagos State	Ojo	Lagos
5064	LA/1533	LA/1533/S/3	St. Anthony's Medical Centre & Maternity Home		19, Lagos -Badagry Express Way Ojo, Lagos State	Ojo	Lagos
5065	LA/1534	LA/1534/S/11	Physio - Fit Physiotherapy Clinic		Block 1, Plot 4, Agric road by Abattoir new Oko- oba (Behind Oceanic Bank) Fagba, Iju, Agege, Lagos State	Agege	Lagos
5066	LA/1537	LA/1537/S/8	Dental Plus Ltd		No. 2 Deji Odunuga Street Off Adebayo Mokuolu Street, Anthony village, Lagos State	Kosofe	Lagos
5067	LA/1539	LA/1539/S/3	Lifegate Specialist Hospital		2B, Abiodun Jagun Str., Ogba , Ikeja,Lagos State	Ikeja	Lagos
5068	LA/1540	LA/1540/S/5	Anthony cardinal okogie's medical center		1, akinsegun street, Oko Oba, Agege,Lagos State	Agege	Lagos
5069	LA/1541	LA/1541/S/1	Bolutife Nursing Homel		7, Vero Acha street, Jankara, Ijaiye- Ojokoro B/Stop, Lagos State	Oshodi/Isolo	Lagos
5070	LA/1541	LA/1541/S/3	Bolutife Nursing Homel		7, Vero Acha street, Jankara, Ijaiye- Ojokoro B/Stop, Lagos State	Oshodi/Isolo	Lagos

5071	LA/1542	LA/1542/S/11	wellpath physiotherapy		10, Baidua street off Keffi street, Ikoyi, Lagos State	Eti-Osa	Lagos
5072	LA/1543	LA/1543/S/10	Liberty Specialist Hospital		2, Folayemi Street off Coker Road, Ilupeju, Lagos State	Mushin	Lagos
5073	LA/1543	LA/1543/S/5	Liberty Specialist Hospital		2, Folayemi Street off Coker Road, Ilupeju, Lagos State	Mushin	Lagos
5074	LA/1544	LA/1544/S/8	Royash dental services		plot 9, blk 10, Ogba-ijaiye road, Lagos State	Ikeja	Lagos
5075	LA/1545	LA/1545/S/4	Juta pharmacy		182, ikotun road, council b-stop, idimu, Lagos State	Alimosho	Lagos
5076	LA/1548	LA/1548/S/8	SNOW WHITE DENTAL CLINIC		10, ATUNWA STREET, OFF UNITY RD/OLA AYINDE STREET, IKEJA, Lagos State	Ikeja	Lagos
5077	LA/1551	LA/1551/S/5	Help Immunoassay Laboratories		1 Ikorodu Road Maryland, Lagos State	Ikorodu	Lagos
5078	LA/1552	LA/1552/S/13	Metro Eye Clinic		22, Keffi St, Ikoyi ,Lagos State	Eti-Osa	Lagos
5079	LA/1553	LA/1553/S/5	Urbane Medical Clinic		16, BAIYEWU CLOSE, OFF AJAYI RD, OGBA, Lagos State	Ifako-Ijaye	Lagos
5080	LA/1554	LA/1554/S/6	Onwusikawa children's medical center		20, Ogunbela Avenue, AGO PALACE, Okota, Lagos State	Oshodi/Isolo	Lagos
5081	LA/1557	LA/1557/S/1	Mary Ade specialist hospital		2, Tijani Ashogbon street, ilaje b/stop, Bariga, Lagos State	Somolu	Lagos
5082	LA/1557	LA/1557/S/5	Mary Ade specialist hospital		2, Tijani Ashogbon street, ilaje b/stop, Bariga, Lagos State	Somolu	Lagos
5083	LA/1557	LA/1557/S/3	Mary Ade specialist hospital		2, Tijani Ashogbon street, ilaje b/stop, Bariga, Lagos State	Somolu	Lagos
5084	LA/1558	LA/1558/S/13	Boost opticals limited		Suite H 108, Ikota shopping complex VGC, Lekki, Lagos State	Eti-Osa	Lagos
5085	LA/1563	LA/1563/S/1	West Care Specialist Hospital		32, Samuel Street Vulcanizer bus stop Egbeda, Akowonjo, Lagos State	Alimosho	Lagos
5086	LA/1563	LA/1563/S/8	West Care Specialist Hospital		32, Samuel Street Vulcanizer bus stop Egbeda, Akowonjo, Lagos State	Alimosho	Lagos
5087	LA/1563	LA/1563/S/3	West Care Specialist Hospital		32, Samuel Street Vulcanizer bus stop Egbeda, Akowonjo, Lagos State	Alimosho	Lagos
5088	LA/1563	LA/1563/S/6	West Care Specialist Hospital		32, Samuel Street Vulcanizer bus stop Egbeda, Akowonjo, Lagos State	Alimosho	Lagos
5089	LA/1563	LA/1563/S/2	West Care Specialist Hospital		32, Samuel Street Vulcanizer bus stop Egbeda, Akowonjo, Lagos State	Alimosho	Lagos
5090	LA/1568	LA/1568/S/1	St. Nicholas Hospital		58 Campbell Street Lagos Island, Lagos State	Lagos Island	Lagos
5091	LA/1568	LA/1568/S/7	St. Nicholas Hospital		58 Campbell Street Lagos Island, Lagos State	Lagos Island	Lagos
5092	LA/1568	LA/1568/S/4	St. Nicholas Hospital		58 Campbell Street Lagos Island, Lagos State	Lagos Island	Lagos
5093	LA/1568	LA/1568/S/3	St. Nicholas Hospital		58 Campbell Street Lagos Island, Lagos State	Lagos Island	Lagos
5094	LA/1568	LA/1568/S/14	St. Nicholas Hospital		58 Campbell Street Lagos Island, Lagos State	Lagos Island	Lagos
5095	LA/1568	LA/1568/S/2	St. Nicholas Hospital		58 Campbell Street Lagos Island, Lagos State	Lagos Island	Lagos
5096	LA/1568	LA/1568/S/6	St. Nicholas Hospital		58 Campbell Street Lagos Island, Lagos State	Lagos Island	Lagos
5097	LA/1568	LA/1568/S/5	St. Nicholas Hospital		58 Campbell Street Lagos Island, Lagos State	Lagos Island	Lagos
5098	LA/1572	LA/1572/S/1	Bolutife Hospital		26, Eweje street, Mafoluku, Oshodi, Lagos State	Oshodi/Isolo	Lagos
5099	LA/1572	LA/1572/S/3	Bolutife Hospital		26, Eweje street, Mafoluku, Oshodi, Lagos State	Oshodi/Isolo	Lagos
5100	LA/1573	LA/1573/S/4	Abek Pharmacy		143, Ipaja road, Agege, Lagos State	Agege	Lagos
5101	LA/1574	LA/1574/S/13	Rekis Eye Centre Limited		Olatunji House, Ground Floor, 299, Ikorodu Road, Idi-Iroko, Maryland, Lagos State	Kosofe	Lagos
5102	LA/1575	LA/1575/S/15	Faratori eye center		187, igbosere road, lagos island, Lagos State	Lagos Island	Lagos

5103	LA/1578	LA/1578/S/14	Ojuelegba Scan & x-ray Centre		77 Ojuelegba road, Lagos State	Surulere	Lagos
5104	LA/1578	LA/1578/S/7	Ojuelegba Scan & x-ray Centre		77 Ojuelegba road, Lagos State	Surulere	Lagos
5105	LA/1578	LA/1578/S/5	Ojuelegba Scan & x-ray Centre		77 Ojuelegba road, Lagos State	Surulere	Lagos
5106	LA/1581	LA/1581/S/3	Ahmadiya Muslim Hospital		39, Payne crescent, Apapa, Lagos State	Ajeromi/Ifelodun	Lagos
5107	LA/1581	LA/1581/S/2	Ahmadiya Muslim Hospital		39, Payne crescent, Apapa, Lagos State	Ajeromi/Ifelodun	Lagos
5108	LA/1581	LA/1581/S/7	Ahmadiya Muslim Hospital		39, Payne crescent, Apapa, Lagos State	Ajeromi/Ifelodun	Lagos
5109	LA/1585	LA/1585/S/8	Hygei dental and eye clinic		14, sowemimo street, GRA, Ikeja, Lagos State	Ikeja	Lagos
5110	LA/1585	LA/1585/S/15	Hygei dental and eye clinic		14, sowemimo street, GRA, Ikeja, Lagos State	Ikeja	Lagos
5111	LA/1587	LA/1587/S/13	Crystal eye consult		140, Bode Thomas, surulere, Lagos State	Surulere	Lagos
5112	LA/1588	LA/1588/S/13	Just Vision Optical & Eye Centre		62 Adeniran Ogunsanya St Surulere, Lagos State	Surulere	Lagos
5113	LA/1589	LA/1589/S/8	Nabille Dental surgey		47a corporation drive, dolphin estate, Ikoyi, Lagos State	Eti-Osa	Lagos
5114	LA/1591	LA/1591/S/1	St. Raphael Divine Mercy Specialist Hospital		Boge Town, Ijede Road, Ikorodu, Lagos State	Ikorodu	Lagos
5115	LA/1591	LA/1591/S/4	St. Raphael Divine Mercy Specialist Hospital		Boge Town, Ijede Road, Ikorodu, Lagos State	Ikorodu	Lagos
5116	LA/1591	LA/1591/S/13	St. Raphael Divine Mercy Specialist Hospital		Boge Town, Ijede Road, Ikorodu, Lagos State	Ikorodu	Lagos
5117	LA/1591	LA/1591/S/3	St. Raphael Divine Mercy Specialist Hospital		Boge Town, Ijede Road, Ikorodu, Lagos State	Ikorodu	Lagos
5118	LA/1591	LA/1591/S/5	St. Raphael Divine Mercy Specialist Hospital		Boge Town, Ijede Road, Ikorodu, Lagos State	Ikorodu	Lagos
5119	LA/1598	LA/1598/S/5	Hallmark clinical and children diagnostic lab		4 Aradula Close Sabo-Oniba, btw peoples and church bus stops, off Alaba int'l mkt, Lagos State	Ojo	Lagos
5120	LA/1605	LA/1605/S/4	Teslon Pharmacy Nig Ltd		204, lagos Rd, Agric B/S Ikorodu, Lagos State	Ikorodu	Lagos
5121	LA/1606	LA/1606/S/4	Edmic Pharmacy Ltd		248 Iju water works rd, Iju, Lagos State	Ifako-Ijaye	Lagos
5122	LA/1607	LA/1607/S/6	Theo Hospital		25, Davies street off obanle aro bus stop, ketu, Lagos State	Kosofe	Lagos
5123	LA/1607	LA/1607/S/5	Theo Hospital		25, Davies street off obanle aro bus stop, ketu, Lagos State	Kosofe	Lagos
5124	LA/1607	LA/1607/S/3	Theo Hospital		25, Davies street off obanle aro bus stop, ketu, Lagos State	Kosofe	Lagos
5125	LA/1607	LA/1607/S/1	Theo Hospital		25, Davies street off obanle aro bus stop, ketu, Lagos State	Kosofe	Lagos
5126	LA/1609	LA/1609/S/5	Tejuosho Diag & ClinIcal Serv. Ltd		Shop 7 & 8, Con-oil Plaza, Opp Ikeja Gen, Hosp. Ikeja, Lagos State	Ikeja	Lagos
5127	LA/1609	LA/1609/S/14	Tejuosho Diag & ClinIcal Serv. Ltd		Shop 7 & 8, Con-oil Plaza, Opp Ikeja Gen, Hosp. Ikeja, Lagos State	Ikeja	Lagos
5128	LA/1613	LA/1613/S/3	Magodo Specialist Hospital LIMITED		10, Jaiye Oyedotun street, Magodo GRA, Lagos State	Kosofe	Lagos
5129	LA/1615	LA/1615/S/3	The Heritage Hospital LTD		29, Anyantuga St. Mushin Lagos State	Mushin	Lagos
5130	LA/1616	LA/1616/S/13	Phoenix eye clinic		Scapular Plaza, Km 17, Lekki-Epe expressway(beside blue island), Lagos State	Eti-Osa	Lagos
5131	LA/1625	LA/1625/S/8	Roberts dental specialist clinic		19, ogunlana drive (1st floor) surulere, Lagos State	Surulere	Lagos
5132	LA/1628	LA/1628/S/4	Interpharm Ltd.		113 Oriwu Rd, Ikorodu, Lagos State	Ikorodu	Lagos
5133	LA/1631	LA/1631/S/4	Pharm-Affairs (Nig) Ltd		122 Ogudu Rd, Ogudu GRA, Lagos State	Kosofe	Lagos
5134	LA/1634	LA/1634/S/15	FOLAREM eye SPECIALIST HOSPITAL		25, Iperu Akesan street by alagutan traffic light junction, iyana ipaja, Lagos State	Alimosho	Lagos
5135	LA/1635	LA/1635/S/8	Kapul Dental Clinic		Excelssior Hotel 3-15 Ede Str Apapa, Lagos State	Apapa	Lagos
5136	LA/1636	LA/1636/S/5	G.G Able Medical Diagnostic center		194, ijegun road, Ikotun, Lagos State	Alimosho	Lagos

5137	LA/1639	LA/1639/S/7	AA & A Medical Diagnostics		49, Ishaga RD Surulere, Lagos State	Surulere	Lagos
5138	LA/1640	LA/1640/S/8	Raphina Dental Clinic		18, Itire road by randle avenue, itire junction, surulere, Lagos State	Surulere	Lagos
5139	LA/1641	LA/1641/S/5	Maranatha medical diagnostics		Alice shopping center, 101 aina street; off isheri road, grammar school b/st, ojodu-Ikeja, Lagos State	Ikeja	Lagos
5140	LA/1641	LA/1641/S/7	Maranatha medical diagnostics		Alice shopping center, 101 aina street; off isheri road, grammar school b/st, ojodu-Ikeja, Lagos State	Ikeja	Lagos
5141	LA/1644	LA/1644/S/4	Fenomas Pharm Ltd		41 Daodu Str. Oshodi, Lagos State	Oshodi/Isolo	Lagos
5142	LA/1646	LA/1646/S/4	Vatican Bells pharmaceuticals LTD		60,Ogunlana Drive surulere, Lagos State	Surulere	Lagos
5143	LA/1648	LA/1648/S/1	Fuja Medical Centre		23 Elegbata St. Apongbon, Lagos Island, Lagos State	Lagos Island	Lagos
5144	LA/1648	LA/1648/S/3	Fuja Medical Centre		23 Elegbata St. Apongbon, Lagos Island, Lagos State	Lagos Island	Lagos
5145	LA/1648	LA/1648/S/5	Fuja Medical Centre		23 Elegbata St. Apongbon, Lagos Island, Lagos State	Lagos Island	Lagos
5146	LA/1650	LA/1650/S/4	Civic Pharmaceutical Ltd		176 Abeokuta Exp-Way, Iyana-Ipaja, Lagos State	Alimosho	Lagos
5147	LA/1651	LA/1651/S/8	Stellar dental clinic		45, olufemi road, off ogulana drive, surulere, Lagos State	Surulere	Lagos
5148	LA/1652	LA/1652/S/7	Funtom Spec.Med.Diag. Centre		19b Iganmu Rd New rd B/stop Lagos State	Surulere	Lagos
5149	LA/1658	LA/1658/S/3	True Care Model Hospital Ltd		19, AdeyemiApena Street,Papa, Epe. Behind The General Hospital, Epe Lagos State	Epe	Lagos
5150	LA/1661	LA/1661/S/5	Nigeria Customs service Medical Centre,		Nigeria Customs Service Medical Centre, Seme Badagri Lagos State	Badagry	Lagos
5151	LA/1661	LA/1661/S/4	Nigeria Customs service Medical Centre,		Nigeria Customs Service Medical Centre, Seme Badagri Lagos State	Badagry	Lagos
5152	LA/1662	LA/1662/S/5	Mojol Hospital Laboratory		20, Church Street, Sasa, Bammake, Lagos State	Alimosho	Lagos
5153	LA/1664	LA/1664/S/4	Broad Associates Limited		No 1B Haruna Street, Off College Road, Ifako Ijaye, Lagos State	Ifako-Ijaye	Lagos
5154	LA/1669	LA/1669/S/4	Lanbib Pharmacy & stores LTD		Shop 496, School bus stop, Abesan estate, Ipaja, Lagos State	Alimosho	Lagos
5155	LA/1671	LA/1671/S/4	Pepat Ventures (Nig) Ltd		11 adeola Str, Medina Estate, Gbagada, Lagos State	Kosofe	Lagos
5156	LA/1673	LA/1673/S/4	Robson Pharm. Ltd		16 Oresegun Crescent,Off Odunsi Str, Bariga, Lagos State	Somolu	Lagos
5157	LA/1676	LA/1676/S/4	Fontana Chemists LTD		23,Ogunlana Drive, Surulere, Lagos State	Surulere	Lagos
5158	LA/1680	LA/1680/S/4	Govatek Pharm & Stores		260, Isawo Rd, RSA B/stop, Abule Cole, Ajaguro Agric, Owutu, Ikorodu, Lagos State	Ikorodu	Lagos
5159	LA/1681	LA/1681/S/1	Rabboni specialist Hospital		7, Eyiowuawi street, Pedro Lagos Lagos State	Somolu	Lagos
5160	LA/1683	LA/1683/S/14	Mt. MORIAH hospital		1/3, Jolade ayoola street, off mosalasi road, Egan, Lagos State	Alimosho	Lagos
5161	LA/1684	LA/1684/S/5	Global viva medical		113, ojuelegba road, surulere, Lagos State	Surulere	Lagos
5162	LA/1686	LA/1686/S/5	Viscol Medical Laboratory		12B Strachan Str. Off Lapal House Lagos Island, Lagos State	Lagos Island	Lagos
5163	LA/1688	LA/1688/S/13	Swift eye care		9, Amodu Ojikutu str. Off Bishop Oluwole str V/I, Lagos State	Eti-Osa	Lagos
5164	LA/1689	LA/1689/S/1	His Stripes Specialist Hospital		19/22 Savage St. Orile-Iganmu, Surulere, Lagos State	Surulere	Lagos
5165	LA/1693	LA/1693/S/4	Lanyard Pharmacy		86, Ago Palace way, By Century B/Stop, Okota Isolo, Lagos State	Apapa	Lagos
5166	LA/1694	LA/1694/S/15	Maxi Specialist Eye Clinic& Optical Services		113, Allen Avenue, Ikeja, Lagos State	Oshodi/Isolo	Lagos
5167	LA/1697	LA/1697/S/5	Mayflower Clinics		1, Ibari Str, Ifako, Agege, Lagos, Lagos State	Alimosho	Lagos

5168	LA/1697	LA/1697/S/1	Mayflower Clinics		1, Ibari Str, Ifako, Agege, Lagos, Lagos State	Alimosho	Lagos
5169	LA/1698	LA/1698/S/4	Foundation Pharmacy		23, Emmanuel Street, Off Jagunmolu Street, Bariga, Lagos.	Somolu	Lagos
5170	LA/1700	LA/1700/S/4	Lumaco Pharmaceuticals Limited		1, Toyan Street, Obalende, Lagos	Lagos Island	Lagos
5171	LA/1701	LA/1701/S/13	Raye Eye Clinic		59, Olatilewa Street, Off Ogunlana drive, Surulere, Lagos	Surulere	Lagos
5172	LA/1702	LA/1702/S/4	Pharmacare Support Services		51 Road, Opposite NITEL Gate, Festac Town, Lagos.	Amuwo-Odofin	Lagos
5173	LA/1703	LA/1703/S/4	Pharmacare Support Services		29, Community Road, Satellite Town, Lagos	Amuwo-Odofin	Lagos
5174	LA/1708	LA/1708/S/4	Anynta Pharm(Nig) Ltd		10, Kasumu Street, Shangisha- Ketu, Lagos	Kosofe	Lagos
5175	LA/1709	LA/1709/S/5	Waxlab Medical Diagnostic Centre		2, Isiaka Olufemi Street, Off LASU- Isheri Road, Olorufemi Bus Stop, Lagos	Alimosho	Lagos
5176	LA/1710	LA/1710/S/4	Unilag Community Pharmacy		UNILAG main Campus, Lagos.	Lagos Mainland	Lagos
5177	LA/1712	LA/1712/S/5	Prestige Diagnostic Centre		194,Ojo-Igbede Road, Ajangbadi, Ilemba-Hausa, Ojo, Lagos State	Ojo	Lagos
5178	LA/1713	LA/1713/S/15	Radiant Optics		Plot 307, WoolWorths Plaza, Adeola Odeku, Victoria Island, Lagos	Eti-Osa	Lagos
5179	LA/1716	LA/1716/S/1	The Lister Medical centre		106, Obunta Avenue, Off Ajao Road, off Adeniyi Jones, Ikeja, Lagos State	Ikeja	Lagos
5180	LA/1717	LA/1717/S/4	Caratiyah Pharmacy And Stores.		90, Dopemu Road, Alhaji Shifau Bus Stop, Agege Lagos State	Agege	Lagos
5181	LA/1719	LA/1719/S/11	Blooming care Hospital		30. Baale Animashahun Road Alakuko.	Ifako-Ijaye	Lagos
5182	LA/1719	LA/1719/S/3	Blooming care Hospital		30. Baale Animashahun Road Alakuko.	Ifako-Ijaye	Lagos
5183	LA/1719	LA/1719/S/8	Blooming care Hospital		30. Baale Animashahun Road Alakuko.	Ifako-Ijaye	Lagos
5184	LA/1719	LA/1719/S/5	Blooming care Hospital		30. Baale Animashahun Road Alakuko.	Ifako-Ijaye	Lagos
5185	LA/1720	LA/1720/S/3	Holy Family Hospital And Maternity Home, Satellite Town		1, Ijegun Road, Satellite Town	Ojo	Lagos
5186	LA/1722	LA/1722/S/15	Opti lens Eyecare		9, Olatunde Close, Off Falolu Road, Surulere, Lagos State	Surulere	Lagos
5187	LA/1724	LA/1724/S/10	Albina Mejeel Specialist Clinic		13,Asani Akibon Street, Victory Estate,Iba Ojo Lagos State.	Ojo	Lagos
5188	LA/1727	LA/1727/S/8	Havilla Specialist Dental Clinic		2, Oyedale Ogunniyi Street, Anthony Village, Lagos State	Kosofe	Lagos
5189	LA/1729	LA/1729/S/15	Hephzibah Eye Care		32 Admiralty Way, Opp. First Bank, Lekki Phase 1 Lagos State	Eti-Osa	Lagos
5190	LA/1730	LA/1730/S/15	Viewpoint Specialist Eye Centre		118, Murtala Moh`d Way, Ebute Metta, Lagos State	Lagos Mainland	Lagos
5191	LA/1731	LA/1731/S/6	Outreach Women And Children Hospital		4th Avenue By 3rd Avenue Junction, Festac Town Lagos State	Amuwo-Odofin	Lagos
5192	LA/1731	LA/1731/S/3	Outreach Women And Children Hospital		4th Avenue By 3rd Avenue Junction, Festac Town Lagos State	Amuwo-Odofin	Lagos
5193	LA/1733	LA/1733/S/4	Sata Pharmacy		270, Agege Motor Road, Mushin, Lagos State	Mushin	Lagos
5194	LA/1736	LA/1736/S/2	Cardiac And Renal Center		No. 1, Hospital Rd. Within Gbagada General Hospital, Lagos State.	Kosofe	Lagos
5195	LA/1737	LA/1737/S/4	Chestra Pharmacy And Stores LTD		No. 1, Solomon Oroale Street, Itedo Village, Lekki Phase 1, Lagos State.	Eti-Osa	Lagos
5196	LA/1738	LA/1738/S/8	Toothcom Dental Clinic		Plot 3012/No 14, Rafiu Babatunde Tinubu Rd. Amuwo Odofin, Lagos State	Amuwo-Odofin	Lagos
5197	LA/1739	LA/1739/S/2	Ostard Specialist Hospital		161, Idewu Street, Olodi Apapa, Lagos State	Ajeromi/Ifelodun	Lagos
5198	LA/1741	LA/1741/S/8	Peve Dental Clinic		Block B, Suite 36, Primal Tek Shopping Plaza, Opp. Iju Garage, Pen Cinema, Agege, Lagos State	Ifako-Ijaye	Lagos
5199	LA/1742	LA/1742/S/13	Vision Plus Specialist Eye Clinic		24,Allen Avenue, Ikeja, Lagos State	Ikeja	Lagos

5200	LA/1755	LA/1755/S/3	Peterhoff Specialist Hospital		4, Ganiyu Osebaby Street, Off Dr. Fasheun Avenue, Ago, Lagos State.	Oshodi/Isolo	Lagos
5201	LA/1755	LA/1755/S/1	Peterhoff Specialist Hospital		4, Ganiyu Osebaby Street, Off Dr. Fasheun Avenue, Ago, Lagos State.	Oshodi/Isolo	Lagos
5202	LA/1759	LA/1759/S/3	Louise Med Hospital Limited		Plot 14, Blk. 95, Omorinre Johnson Street, Lekki Phase 1, Lagos State	Eti-Osa	Lagos
5203	LA/1759	LA/1759/S/6	Louise Med Hospital Limited		Plot 14, Blk. 95, Omorinre Johnson Street, Lekki Phase 1, Lagos State	Eti-Osa	Lagos
5204	LA/1759	LA/1759/S/1	Louise Med Hospital Limited		Plot 14, Blk. 95, Omorinre Johnson Street, Lekki Phase 1, Lagos State	Eti-Osa	Lagos
5205	LA/1760	LA/1760/S/5	Riverside Diagnostic Hospital Limited		Plot 1397e Block 63a, Shalom Road, Off Raji Rasaki Road, Near Apple Junction, Amuwo Odofin, Lagos State	Amuwo-Odofin	Lagos
5206	LA/1765	LA/1765/S/7	Foundation Medical Laboratory		No. 38, Enitan Street, Aguda, Surulere, lagos State	Surulere	Lagos
5207	LA/1769	LA/1769/S/10	St. Julius Hospital		No. 17, Adekunbi Street, off Toyin Street, Ikeja, Lagos	Ikeja	Lagos
5208	LA/1769	LA/1769/S/3	St. Julius Hospital		No. 17, Adekunbi Street, off Toyin Street, Ikeja, Lagos	Ikeja	Lagos
5209	LA/1770	LA/1770/S/8	Dental Plus		Plot 286, Muri Okuola Street (Near Price Water House coopers) Victoria Island Lagos State.	Eti-Osa	Lagos
5210	LA/1775	LA/1775/S/5	NNPC Clinic		No. 1B, Muri Okunola Street, Lagos State	Eti-Osa	Lagos
5211	LA/1775	LA/1775/S/2	NNPC Clinic		No. 1B, Muri Okunola Street, Lagos State	Eti-Osa	Lagos
5212	LA/1775	LA/1775/S/1	NNPC Clinic		No. 1B, Muri Okunola Street, Lagos State	Eti-Osa	Lagos
5213	LA/1775	LA/1775/S/4	NNPC Clinic		No. 1B, Muri Okunola Street, Lagos State	Eti-Osa	Lagos
5214	LA/1775	LA/1775/S/7	NNPC Clinic		No. 1B, Muri Okunola Street, Lagos State	Eti-Osa	Lagos
5215	LA/1775	LA/1775/S/12	NNPC Clinic		No. 1B, Muri Okunola Street, Lagos State	Eti-Osa	Lagos
5216	LA/1775	LA/1775/S/15	NNPC Clinic		No. 1B, Muri Okunola Street, Lagos State	Eti-Osa	Lagos
5217	LA/1775	LA/1775/S/11	NNPC Clinic		No. 1B, Muri Okunola Street, Lagos State	Eti-Osa	Lagos
5218	LA/1775	LA/1775/S/10	NNPC Clinic		No. 1B, Muri Okunola Street, Lagos State	Eti-Osa	Lagos
5219	LA/1778	LA/1778/S/15	Ocuville Eye Centre		95, Ogudu Road, Ojota, Lagos State	Kosofe	Lagos
5220	LA/1779	LA/1779/S/13	Sightcity Eye Clinic		FMDA Building Plot 1398D, Tiamiyu Savage Street, Victoria Island Lagos State	Eti-Osa	Lagos
5221	LA/1788	LA/1788/S/3	Kingswill Specialist Hospital		3, Ayinuola Close/18b Rafiu Babatunde Road, By Apple Junction Amuwo- Odofin Lagos State	Amuwo-Odofin	Lagos
5222	LA/1788	LA/1788/S/1	Kingswill Specialist Hospital		3, Ayinuola Close/18b Rafiu Babatunde Road, By Apple Junction Amuwo- Odofin Lagos State	Amuwo-Odofin	Lagos
5223	LA/1794	LA/1794/S/3	Bee Hess Hospital		155, Akowonjo Road, Akowonjo , Lagos State	Alimosho	Lagos
5224	LA/1794	LA/1794/S/2	Bee Hess Hospital		155, Akowonjo Road, Akowonjo , Lagos State	Alimosho	Lagos
5225	LA/1794	LA/1794/S/6	Bee Hess Hospital		155, Akowonjo Road, Akowonjo , Lagos State	Alimosho	Lagos
5226	LA/1795	LA/1795/S/8	Building Smiles Dental Clinic		70, Opebi Rd. Ikeja, Lagos State	Ikeja	Lagos
5227	LA/1798	LA/1798/S/5	Mifam Medical Laboratory Services		Mifam Medical Laboratory Services	Ajeromi/Ifelodun	Lagos
5228	LA/1803	LA/1803/S/1	Mercy Stripes Specialist Hospital		30, Philip Taiwo Street, Coker Estate, Orisunbare, Shasha Lagos Lagos State	Alimosho	Lagos
5229	LA/1803	LA/1803/S/6	Mercy Stripes Specialist Hospital		30, Philip Taiwo Street, Coker Estate, Orisunbare, Shasha Lagos Lagos State	Alimosho	Lagos

5230	LA/1803	LA/1803/S/3	Mercy Stripes Specialist Hospital		30, Philip Taiwo Street, Coker Estate, Orisunbare, Shasha Lagos Lagos State	Alimosho	Lagos
5231	LA/1803	LA/1803/S/2	Mercy Stripes Specialist Hospital		30, Philip Taiwo Street, Coker Estate, Orisunbare, Shasha Lagos Lagos State	Alimosho	Lagos
5232	LA/1804	LA/1804/S/13	Meb Eye Clinic (Eye Clinic & Optical Services)		1, Charity Road, Abule-Egba, Lagos. Lagos State	Ifako-Ijaye	Lagos
5233	LA/1813	LA/1813/S/1	Macedonia Specialist Hospital		28,Ijaiye Road, Ogba	Ifako-Ijaye	Lagos
5234	LA/1813	LA/1813/S/3	Macedonia Specialist Hospital		28,Ijaiye Road, Ogba	Ifako-Ijaye	Lagos
5235	LA/1814	LA/1814/S/4	Epsilon Pharmacy Ltd		Shop 4/5,Kanyinsola Plaza, Shagari Estate, Ipaja Lagos State	Alimosho	Lagos
5236	LA/1815	LA/1815/S/4	Hess Tee Pharmacy		8,Ifoshi Road, Iyana Ejigbo Lagos State	Oshodi/Isolo	Lagos
5237	LA/1817	LA/1817/S/7	Promise Diagnostics		132, Dopemu Road, Dopemu-Agege, Lagos, Lagos State	Agege	Lagos
5238	LA/1817	LA/1817/S/14	Promise Diagnostics		132, Dopemu Road, Dopemu-Agege, Lagos, Lagos State	Agege	Lagos
5239	LA/1819	LA/1819/S/13	Mega Vision Limited		15, Allen Avenue, Ikeja, Lagos State	Ikeja	Lagos
5240	LA/1820	LA/1820/S/8	Platinum Dental Surgery		106, Aladelola Street, Ikosi Ketu, Lagos, Lagos State	Kosofe	Lagos
5241	LA/1822	LA/1822/S/5	Our Lady and St. Francis Catholic Hospital		Along Super Flamingo Street, Toga-Zanmu Village, Lagos/Badagry Expressway, Lagos State	Badagry	Lagos
5242	LA/1823	LA/1823/S/15	Mami Eye Clinic		38, Olowu Street, Ikeja, Lagos Street	Ikeja	Lagos
5243	LA/1824	LA/1824/S/13	New Look Eye Clinic & Optical Services Ltd		1, Idimu Road, Ikotun, Lagos, Lagos State	Alimosho	Lagos
5244	LA/1825	LA/1825/S/15	Precious Eye Hospital Ltd		1 Saluwala Kadiku Street, Happyland Estate, Lekki-Epe Expressway, Lagos State	Eti-Osa	Lagos
5245	LA/1827	LA/1827/S/13	Procare Vision		68, Queen Street, Yaba, Lagos, Lagos State	Lagos Mainland	Lagos
5246	LA/1833	LA/1833/S/13	Vision home Eyecare		29, Opebi Road, Ikeja, Lagos State	Ikeja	Lagos
5247	LA/1837	LA/1837/S/3	IFPF Hospital		374, Ikorodu Road, Maryland, Lagos State	Kosofe	Lagos
5248	LA/1838	LA/1838/S/5	Diagnostic Palace		Elesho B/Stop, Iyanaera, Off Lagos-Badagry Expressway, Ijanikin, Lagos State	Ojo	Lagos
5249	LA/1839	LA/1839/S/5	St. Edward Specialist Hospital & Cardiac Centre		4, Dolapo Oshonaike Street Off Ado Road, Ajah, Lekki, Lagos State	Eti-Osa	Lagos
5250	LA/1841	LA/1841/S/13	Eaglesyte Opticals		33, Karimu Street, Itire Road, Surulere, Lagos State	Surulere	Lagos
5251	LA/1843	LA/1843/S/13	Standard Optical Limited		1b, Point Road, Polysonic Mall, Apapa, Lagos State	Apapa	Lagos
5252	LA/1844	LA/1844/S/13	Standard Optical Limited		1b, Point Road, Polysonic Mall, Apapa, Lagos State	Apapa	Lagos
5253	NG/0002	NG/0002/S/5	General Hospital, Agaie	-	Agaie, Minna	Agaie	Niger
5254	NG/0002	NG/0002/S/1	General Hospital, Agaie	-	Agaie, Minna	Agaie	Niger
5255	NG/0002	NG/0002/S/6	General Hospital, Agaie	-	Agaie, Minna	Agaie	Niger
5256	NG/0002	NG/0002/S/4	General Hospital, Agaie	-	Agaie, Minna	Agaie	Niger
5257	NG/0002	NG/0002/S/2	General Hospital, Agaie	-	Agaie, Minna	Agaie	Niger
5258	NG/0003	NG/0003/S/7	Federal Medical Centre, Bida		Bida.	Bida	Niger
5259	NG/0003	NG/0003/S/6	Federal Medical Centre, Bida		Bida.	Bida	Niger
5260	NG/0003	NG/0003/S/11	Federal Medical Centre, Bida		Bida.	Bida	Niger
5261	NG/0003	NG/0003/S/4	Federal Medical Centre, Bida		Bida.	Bida	Niger
5262	NG/0003	NG/0003/S/8	Federal Medical Centre, Bida		Bida.	Bida	Niger
5263	NG/0003	NG/0003/S/1	Federal Medical Centre, Bida		Bida.	Bida	Niger
5264	NG/0003	NG/0003/S/3	Federal Medical Centre, Bida		Bida.	Bida	Niger
5265	NG/0003	NG/0003/S/5	Federal Medical Centre, Bida		Bida.	Bida	Niger

5266	NG/0009	NG/0009/S/4	Fem-Universal Pharmacy	Pharmacy	ABK House, BCC Road, Bida	Bida	Niger
5267	NG/0013	NG/0013/S/1	General Hospital, Minna.		Hospital Road, Minna.	Chanchaga	Niger
5268	NG/0013	NG/0013/S/3	General Hospital, Minna.		Hospital Road, Minna.	Chanchaga	Niger
5269	NG/0013	NG/0013/S/7	General Hospital, Minna.		Hospital Road, Minna.	Chanchaga	Niger
5270	NG/0013	NG/0013/S/8	General Hospital, Minna.		Hospital Road, Minna.	Chanchaga	Niger
5271	NG/0013	NG/0013/S/12	General Hospital, Minna.		Hospital Road, Minna.	Chanchaga	Niger
5272	NG/0013	NG/0013/S/5	General Hospital, Minna.		Hospital Road, Minna.	Chanchaga	Niger
5273	NG/0013	NG/0013/S/6	General Hospital, Minna.		Hospital Road, Minna.	Chanchaga	Niger
5274	NG/0013	NG/0013/S/15	General Hospital, Minna.		Hospital Road, Minna.	Chanchaga	Niger
5275	NG/0013	NG/0013/S/4	General Hospital, Minna.		Hospital Road, Minna.	Chanchaga	Niger
5276	NG/0014	NG/0014/S/11	IBB Specialist Hospital		Biko Road, Chanchangi, Minna	Chanchaga	Niger
5277	NG/0014	NG/0014/S/7	IBB Specialist Hospital		Biko Road, Chanchangi, Minna	Chanchaga	Niger
5278	NG/0014	NG/0014/S/4	IBB Specialist Hospital		Biko Road, Chanchangi, Minna	Chanchaga	Niger
5279	NG/0014	NG/0014/S/5	IBB Specialist Hospital		Biko Road, Chanchangi, Minna	Chanchaga	Niger
5280	NG/0014	NG/0014/S/10	IBB Specialist Hospital		Biko Road, Chanchangi, Minna	Chanchaga	Niger
5281	NG/0014	NG/0014/S/12	IBB Specialist Hospital		Biko Road, Chanchangi, Minna	Chanchaga	Niger
5282	NG/0014	NG/0014/S/1	IBB Specialist Hospital		Biko Road, Chanchangi, Minna	Chanchaga	Niger
5283	NG/0014	NG/0014/S/3	IBB Specialist Hospital		Biko Road, Chanchangi, Minna	Chanchaga	Niger
5284	NG/0016	NG/0016/S/1	Bay Specialist Hospital		Plot 33, Bay Clinic Road, Tunga, Minna	Chanchaga	Niger
5285	NG/0017	NG/0017/S/3	Imani Specialist Hospital		Minna.	Chanchaga	Niger
5286	NG/0017	NG/0017/S/14	Imani Specialist Hospital		Minna.	Chanchaga	Niger
5287	NG/0017	NG/0017/S/1	Imani Specialist Hospital		Minna.	Chanchaga	Niger
5288	NG/0020	NG/0020/S/1	Standard Hospital		Old Airport Quarters, Minna	Chanchaga	Niger
5289	NG/0020	NG/0020/S/7	Standard Hospital		Old Airport Quarters, Minna	Chanchaga	Niger
5290	NG/0020	NG/0020/S/3	Standard Hospital		Old Airport Quarters, Minna	Chanchaga	Niger
5291	NG/0020	NG/0020/S/5	Standard Hospital		Old Airport Quarters, Minna	Chanchaga	Niger
5292	NG/0022	NG/0022/S/7	Alheri Specialist Hospital		Minna	Chanchaga	Niger
5293	NG/0024	NG/0024/S/2	General Hospital, Mokwa		Mokwa	Mokwa	Niger
5294	NG/0024	NG/0024/S/1	General Hospital, Mokwa		Mokwa	Mokwa	Niger
5295	NG/0024	NG/0024/S/3	General Hospital, Mokwa		Mokwa	Mokwa	Niger
5296	NG/0024	NG/0024/S/6	General Hospital, Mokwa		Mokwa	Mokwa	Niger
5297	NG/0024	NG/0024/S/4	General Hospital, Mokwa		Mokwa	Mokwa	Niger
5298	NG/0024	NG/0024/S/5	General Hospital, Mokwa		Mokwa	Mokwa	Niger
5299	NG/0027	NG/0027/S/12	General Hospital, Minna	Gen. Medicine, Laboratory, Pharmacy, Dentistry, Radiology, O&G, Surgery, Paediatrics, ENT, Opthamology	Hospital Road, Minna	Chanchaga	Niger
5300	NG/0027	NG/0027/S/6	General Hospital, Minna	Gen. Medicine, Laboratory, Pharmacy, Dentistry, Radiology, O&G, Surgery, Paediatrics, ENT, Opthamology	Hospital Road, Minna	Chanchaga	Niger

5301	NG/0027	NG/0027/S/1	General Hospital, Minna	Gen. Medicine, Laboratory, Pharmacy, Dentistry, Radiology, O&G, Surgery, Paediatrics, ENT, Opthamology	Hospital Road, Minna	Chanchaga	Niger
5302	NG/0027	NG/0027/S/3	General Hospital, Minna	Gen. Medicine, Laboratory, Pharmacy, Dentistry, Radiology, O&G, Surgery, Paediatrics, ENT, Opthamology	Hospital Road, Minna	Chanchaga	Niger
5303	NG/0027	NG/0027/S/7	General Hospital, Minna	Gen. Medicine, Laboratory, Pharmacy, Dentistry, Radiology, O&G, Surgery, Paediatrics, ENT, Opthamology	Hospital Road, Minna	Chanchaga	Niger
5304	NG/0027	NG/0027/S/8	General Hospital, Minna	Gen. Medicine, Laboratory, Pharmacy, Dentistry, Radiology, O&G, Surgery, Paediatrics, ENT, Opthamology	Hospital Road, Minna	Chanchaga	Niger
5305	NG/0027	NG/0027/S/4	General Hospital, Minna	Gen. Medicine, Laboratory, Pharmacy, Dentistry, Radiology, O&G, Surgery, Paediatrics, ENT, Opthamology	Hospital Road, Minna	Chanchaga	Niger
5306	NG/0027	NG/0027/S/5	General Hospital, Minna	Gen. Medicine, Laboratory, Pharmacy, Dentistry, Radiology, O&G, Surgery, Paediatrics, ENT, Opthamology	Hospital Road, Minna	Chanchaga	Niger
5307	NG/0027	NG/0027/S/2	General Hospital, Minna	Gen. Medicine, Laboratory, Pharmacy, Dentistry, Radiology, O&G, Surgery, Paediatrics, ENT, Opthamology	Hospital Road, Minna	Chanchaga	Niger
5308	NG/0027	NG/0027/S/15	General Hospital, Minna	Gen. Medicine, Laboratory, Pharmacy, Dentistry, Radiology, O&G, Surgery, Paediatrics, ENT, Opthamology	Hospital Road, Minna	Chanchaga	Niger
5309	NG/0028	NG/0028/S/1	Imani Specialist Hospital & Diagnostic Centre	O & G, Surgery, Ultrasound.	Plot NGS 4989, Nnamdi Azikiwe Way, Opp., Shiroro Hotel Junction by NEPA Step down Station, Minna	Chanchaga	Niger
5310	NG/0028	NG/0028/S/3	Imani Specialist Hospital & Diagnostic Centre	O & G, Surgery, Ultrasound.	Plot NGS 4989, Nnamdi Azikiwe Way, Opp., Shiroro Hotel Junction by NEPA Step down Station, Minna	Chanchaga	Niger
5311	NG/0028	NG/0028/S/14	Imani Specialist Hospital & Diagnostic Centre	O & G, Surgery, Ultrasound.	Plot NGS 4989, Nnamdi Azikiwe Way, Opp., Shiroro Hotel Junction by NEPA Step down Station, Minna	Chanchaga	Niger
5312	NG/0031	NG/0031/S/4	Zagbayi Pharmacy	Pharmacy	150Y Bosso Road, Behind Habib Bank, Minna	Chanchaga	Niger

5313	NG/0032	NG/0032/S/4	Nauzo Pharmacy Ltd	Pharmacy	NTD 36, Paiko Road, Tunga, Minna	Chanchaga	Niger
5314	NG/0036	NG/0036/S/4	Na'uzo Pharmacy	Pharmacy	Kuta Road, Minna	Lavun	Niger
5315	NG/0038	NG/0038/S/4	Femad Pharmacy & Stores	Pharmacy	Plot 35, Top Medical Road, Minna	Chanchaga	Niger
5316	NG/0039	NG/0039/S/4	Farayola Pharm. Ltd.	Pharmacy	Opp. F.U.T. Bosso Road, Minna	Chanchaga	Niger
5317	NG/0041	NG/0041/S/4	Sekkami Pharm. & Stores	Pharmacy	72, Niteco Road, Minna	Chanchaga	Niger
5318	NG/0042	NG/0042/S/4	Oxgate Pharm Ltd.	Pharmacy	56, Yusuf Kolawole Road, Tunga, Minna	Chanchaga	Niger
5319	NG/0046	NG/0046/S/4	Shebab Pharmacy	Pharmacy	Paiko Road, Chanchangi, Minna	Bosso	Niger
5320	NG/0048	NG/0048/S/6	Goje Medical Centre		Kontangora	Kontagora	Niger
5321	NG/0049	NG/0049/S/7	General Hospital, Kontangora		Kontangora	Kontagora	Niger
5322	NG/0049	NG/0049/S/8	General Hospital, Kontangora		Kontangora	Kontagora	Niger
5323	NG/0049	NG/0049/S/2	General Hospital, Kontangora		Kontangora	Kontagora	Niger
5324	NG/0049	NG/0049/S/6	General Hospital, Kontangora		Kontangora	Kontagora	Niger
5325	NG/0049	NG/0049/S/4	General Hospital, Kontangora		Kontangora	Kontagora	Niger
5326	NG/0049	NG/0049/S/5	General Hospital, Kontangora		Kontangora	Kontagora	Niger
5327	NG/0053	NG/0053/S/1	Rural Hospital, Lapai		Lapai	Lapai	Niger
5328	NG/0053	NG/0053/S/2	Rural Hospital, Lapai		Lapai	Lapai	Niger
5329	NG/0053	NG/0053/S/3	Rural Hospital, Lapai		Lapai	Lapai	Niger
5330	NG/0053	NG/0053/S/6	Rural Hospital, Lapai		Lapai	Lapai	Niger
5331	NG/0053	NG/0053/S/4	Rural Hospital, Lapai		Lapai	Lapai	Niger
5332	NG/0055	NG/0055/S/4	Hephizbah Pharmaceuticals	Pharmacy	2, Halal Shopping Complex, Bida Road, Lapai	Lapai	Niger
5333	NG/0056	NG/0056/S/5	Rural Hospital, Kutigi	Pharmacy, Laboratory	Kutigi, Minna	Lapai	Niger
5334	NG/0056	NG/0056/S/4	Rural Hospital, Kutigi	Pharmacy, Laboratory	Kutigi, Minna	Lapai	Niger
5335	NG/0059	NG/0059/S/5	Base Medical Centre, Suleja		Suleiman Barau Road, Opp. Rimeview Hotel, Suleja	Suleja	Niger
5336	NG/0060	NG/0060/S/5	Citizen Hospital		Suleja	Suleja	Niger
5337	NG/0061	NG/0061/S/5	Fary Group Clinic		105, Suleiman Barau Road, Suleja	Suleja	Niger
5338	NG/0062	NG/0062/S/8	General Hospital, Suleja		Suleja	Suleja	Niger
5339	NG/0062	NG/0062/S/1	General Hospital, Suleja		Suleja	Suleja	Niger
5340	NG/0062	NG/0062/S/7	General Hospital, Suleja		Suleja	Suleja	Niger
5341	NG/0062	NG/0062/S/4	General Hospital, Suleja		Suleja	Suleja	Niger
5342	NG/0062	NG/0062/S/5	General Hospital, Suleja		Suleja	Suleja	Niger
5343	NG/0062	NG/0062/S/3	General Hospital, Suleja		Suleja	Suleja	Niger
5344	NG/0062	NG/0062/S/2	General Hospital, Suleja		Suleja	Suleja	Niger
5345	NG/0062	NG/0062/S/6	General Hospital, Suleja		Suleja	Suleja	Niger
5346	NG/0062	NG/0062/S/14	General Hospital, Suleja		Suleja	Suleja	Niger
5347	NG/0064	NG/0064/S/5	Maraba Hospital - Suleija		Maganda, Off Suleiman, Barau Road	Suleja	Niger
5348	NG/0074	NG/0074/S/4	Dema Q Pharmacy	Pharmacy	Shop 3 Besides Timbe Shed, Kwanba, Suleja	Suleja	Niger
5349	NG/0075	NG/0075/S/4	Bee Pee Pharmacy	Pharmacy	No.9, Suleja Road, Mandala	Suleja	Niger
5350	NG/0076	NG/0076/S/4	Tsowa Pharmaceuticals	Pharmacy	58A, Suleiman Barau Road, Suleja	Suleja	Niger
5351	NG/0079	NG/0079/S/4	Dixions Med. Pharm. Ltd.	Pharmacy	Farins Ruwa, New 1st Bank, Minna Road, Suleja	Suleja	Niger
5352	NG/0080	NG/0080/S/4	Goben Pharm. & Gen. Enterprises	Pharmacy	62, Morocco Road	Suleja	Niger
5353	NG/0081	NG/0081/S/3	Rural Health Hospital, Wushishi		Wushishi	Wushishi	Niger
5354	NG/0081	NG/0081/S/4	Rural Health Hospital, Wushishi		Wushishi	Wushishi	Niger
5355	NG/0081	NG/0081/S/6	Rural Health Hospital, Wushishi		Wushishi	Wushishi	Niger
5356	NG/0081	NG/0081/S/1	Rural Health Hospital, Wushishi		Wushishi	Wushishi	Niger
5357	NG/0081	NG/0081/S/5	Rural Health Hospital, Wushishi		Wushishi	Wushishi	Niger
5358	NG/0081	NG/0081/S/2	Rural Health Hospital, Wushishi		Wushishi	Wushishi	Niger
5359	NG/0085	NG/0085/S/3	General Hospital, Kutigi		Kutigi	Gbako	Niger
5360	NG/0085	NG/0085/S/2	General Hospital, Kutigi		Kutigi	Gbako	Niger

5361	NG/0085	NG/0085/S/1	General Hospital, Kutigi		Kutigi	Gbako	Niger
5362	NG/0086	NG/0086/S/4	General Hosipital, Tungan Magajia		Tungan Magajia	Mariga	Niger
5363	NG/0086	NG/0086/S/1	General Hosipital, Tungan Magajia		Tungan Magajia	Mariga	Niger
5364	NG/0086	NG/0086/S/3	General Hosipital, Tungan Magajia		Tungan Magajia	Mariga	Niger
5365	NG/0086	NG/0086/S/2	General Hosipital, Tungan Magajia		Tungan Magajia	Mariga	Niger
5366	NG/0087	NG/0087/S/5	Rural Hospital, Nasco Road		Nasco Road	Magama	Niger
5367	NG/0087	NG/0087/S/4	Rural Hospital, Nasco Road		Nasco Road	Magama	Niger
5368	NG/0088	NG/0088/S/2	General Hospital, New Bussa		New Bussa	Bosso	Niger
5369	NG/0088	NG/0088/S/1	General Hospital, New Bussa		New Bussa	Bosso	Niger
5370	NG/0088	NG/0088/S/8	General Hospital, New Bussa		New Bussa	Bosso	Niger
5371	NG/0088	NG/0088/S/4	General Hospital, New Bussa		New Bussa	Bosso	Niger
5372	NG/0088	NG/0088/S/6	General Hospital, New Bussa		New Bussa	Bosso	Niger
5373	NG/0088	NG/0088/S/5	General Hospital, New Bussa		New Bussa	Bosso	Niger
5374	NG/0088	NG/0088/S/3	General Hospital, New Bussa		New Bussa	Bosso	Niger
5375	NG/0089	NG/0089/S/5	Rural Hospital	Pharmacy, Laboratory	Nasco	Kontagora	Niger
5376	NG/0089	NG/0089/S/4	Rural Hospital	Pharmacy, Laboratory	Nasco	Kontagora	Niger
5377	NG/0093	NG/0093/S/3	Top Medical Centre		Plot 4 Paiko Road, Tunga, Minna, Niger State	Chanchaga	Niger
5378	NG/0093	NG/0093/S/2	Top Medical Centre		Plot 4 Paiko Road, Tunga, Minna, Niger State	Chanchaga	Niger
5379	NG/0093	NG/0093/S/5	Top Medical Centre		Plot 4 Paiko Road, Tunga, Minna, Niger State	Chanchaga	Niger
5380	NG/0094	NG/0094/S/4	General Hospital, Kagara		Kagara, Niger State	Gurara	Niger
5381	NG/0094	NG/0094/S/5	General Hospital, Kagara		Kagara, Niger State	Gurara	Niger
5382	NG/0094	NG/0094/S/2	General Hospital, Kagara		Kagara, Niger State	Gurara	Niger
5383	NG/0094	NG/0094/S/3	General Hospital, Kagara		Kagara, Niger State	Gurara	Niger
5384	NG/0094	NG/0094/S/6	General Hospital, Kagara		Kagara, Niger State	Gurara	Niger
5385	NG/0094	NG/0094/S/1	General Hospital, Kagara		Kagara, Niger State	Gurara	Niger
5386	NG/0102	NG/0102/S/6	Wasiha Medical Centre		Plot 10B Off Niiteco Road, Tunga, Minna	Chanchaga	Niger
5387	NG/0102	NG/0102/S/5	Wasiha Medical Centre		Plot 10B Off Niiteco Road, Tunga, Minna	Chanchaga	Niger
5388	NG/0106	NG/0106/S/5	Masol Hospital & Maternity		Off Our Lady Of Fatima Church, Niteco Road, Tunga, Minna	Chanchaga	Niger
5389	NG/0107	NG/0107/S/5	Liberty Hospital		Opposite 1st Bank Plc, Minna Road, Suleja	Suleja	Niger
5390	NG/0107	NG/0107/S/4	Liberty Hospital		Opposite 1st Bank Plc, Minna Road, Suleja	Suleja	Niger
5391	NG/0112	NG/0112/S/5	Unis Medical Laboratory	Laboratory	6 victory Rd Suleja	Suleja	Niger
5392	NG/0113	NG/0113/S/5	The Delight Hospital	Laboratory	Plot 66, Suleiman Barrau Rd Suleja	Suleja	Niger
5393	NG/0115	NG/0115/S/4	Beriky Pharmacy	Pharmacy	No 167 Minna Rd Shop 6 LG Stores Suleja	Suleja	Niger
5394	NG/0118	NG/0118/S/4	Newcity Gate Pharmacy Ltd	Pharmacy	2 Hassan Dallatu Rd Suleja	Suleja	Niger
5395	NG/0121	NG/0121/S/1	Yebosoko Hospital	Laboratory, Paediatrics, Surgery, O & G	Banitu Badagry Rd, Bida	Bida	Niger
5396	NG/0121	NG/0121/S/3	Yebosoko Hospital	Laboratory, Paediatrics, Surgery, O & G	Banitu Badagry Rd, Bida	Bida	Niger
5397	NG/0121	NG/0121/S/6	Yebosoko Hospital	Laboratory, Paediatrics, Surgery, O & G	Banitu Badagry Rd, Bida	Bida	Niger
5398	NG/0121	NG/0121/S/5	Yebosoko Hospital	Laboratory, Paediatrics, Surgery, O & G	Banitu Badagry Rd, Bida	Bida	Niger
5399	NG/0123	NG/0123/S/5	Maraba Hospital	Opthalmology, Laboratory	Suleiman Barau Rd NEPA Office Suleja	Suleja	Niger
5400	NG/0130	NG/0130/S/7	Federal Polytechnic Bida Medical Centre		Bida, Niger State	Bida	Niger
5401	NG/0130	NG/0130/S/4	Federal Polytechnic Bida Medical Centre		Bida, Niger State	Bida	Niger
5402	NG/0130	NG/0130/S/5	Federal Polytechnic Bida Medical Centre		Bida, Niger State	Bida	Niger

5403	NG/0148	NG/0148/S/4	VEEIT PHARMACY	Pharmacy	PLOT 55, PETER SAUKI ROAD, TUNGA, MINNA	Chanchaga	Niger
5404	NG/0151	NG/0151/S/4	AJIB PHARMACY	PHARM	SW 78 IBO ROAD, MINNA	Chanchaga	Niger
5405	NG/0152	NG/0152/S/4	HEPHZIBAH PHARMACY	Pharmacy	KETERENAWARI ROAD, SW 440, MINNA	Chanchaga	Niger
5406	NG/0156	NG/0156/S/4	Jem Dental Clinic	Dental Surgery	105 Suleiman Barau Road, Suleja	Suleja	Niger
5407	NG/0160	NG/0160/S/2	General Hospital, Kutigi	O&G, General Surgey, Internal Medicine	Lagos Road, Kutigi	Lavun	Niger
5408	NG/0160	NG/0160/S/1	General Hospital, Kutigi	O&G, General Surgey, Internal Medicine	Lagos Road, Kutigi	Lavun	Niger
5409	NG/0160	NG/0160/S/3	General Hospital, Kutigi	O&G, General Surgey, Internal Medicine	Lagos Road, Kutigi	Lavun	Niger
5410	NG/0161	NG/0161/S/4	General Hospital, Bida	Dentsl Surgey, O&G, General Surgery, Pharmacy	Minna Road, Bida	Bida	Niger
5411	NG/0161	NG/0161/S/1	General Hospital, Bida	Dentsl Surgey, O&G, General Surgery, Pharmacy	Minna Road, Bida	Bida	Niger
5412	NG/0161	NG/0161/S/8	General Hospital, Bida	Dentsl Surgey, O&G, General Surgery, Pharmacy	Minna Road, Bida	Bida	Niger
5413	NG/0161	NG/0161/S/3	General Hospital, Bida	Dentsl Surgey, O&G, General Surgery, Pharmacy	Minna Road, Bida	Bida	Niger
5414	NG/0162	NG/0162/S/4	General Hospital, Tungan Magajiya	Pharmacy, O&G, Internal Medicine, General Surgery	2 Kontagora Road Tungan Magajiya	Rijau	Niger
5415	NG/0162	NG/0162/S/1	General Hospital, Tungan Magajiya	Pharmacy, O&G, Internal Medicine, General Surgery	2 Kontagora Road Tungan Magajiya	Rijau	Niger
5416	NG/0162	NG/0162/S/2	General Hospital, Tungan Magajiya	Pharmacy, O&G, Internal Medicine, General Surgery	2 Kontagora Road Tungan Magajiya	Rijau	Niger
5417	NG/0162	NG/0162/S/3	General Hospital, Tungan Magajiya	Pharmacy, O&G, Internal Medicine, General Surgery	2 Kontagora Road Tungan Magajiya	Rijau	Niger
5418	NG/0163	NG/0163/S/1	Rural Hospital, Auna	General Surgery, Paediatrics, O&G, Internal Medicine	Salka Road, Auna	Nasko	Niger
5419	NG/0163	NG/0163/S/3	Rural Hospital, Auna	General Surgery, Paediatrics, O&G, Internal Medicine	Salka Road, Auna	Nasko	Niger
5420	NG/0163	NG/0163/S/2	Rural Hospital, Auna	General Surgery, Paediatrics, O&G, Internal Medicine	Salka Road, Auna	Nasko	Niger
5421	NG/0163	NG/0163/S/6	Rural Hospital, Auna	General Surgery, Paediatrics, O&G, Internal Medicine	Salka Road, Auna	Nasko	Niger
5422	NG/0164	NG/0164/S/2	Rural Hospital, Kuta	Internal Medicine, Pharmacy, O&G	Gwada Road, Kuta	Shiroro	Niger
5423	NG/0164	NG/0164/S/4	Rural Hospital, Kuta	Internal Medicine, Pharmacy, O&G	Gwada Road, Kuta	Shiroro	Niger
5424	NG/0164	NG/0164/S/7	Rural Hospital, Kuta	Internal Medicine, Pharmacy, O&G	Gwada Road, Kuta	Shiroro	Niger
5425	NG/0164	NG/0164/S/3	Rural Hospital, Kuta	Internal Medicine, Pharmacy, O&G	Gwada Road, Kuta	Shiroro	Niger
5426	NG/0166	NG/0166/S/4	General Hospital. Kafin-Koro	Pharmacy, O&G	Hospital Road, Kafin-Koro	Paikoro	Niger
5427	NG/0166	NG/0166/S/3	General Hospital. Kafin-Koro	Pharmacy, O&G	Hospital Road, Kafin-Koro	Paikoro	Niger
5428	NG/0168	NG/0168/S/3	General Hospital		Hospital Road, Kafin-Koro	Paikoro	Niger
5429	NG/0168	NG/0168/S/4	General Hospital		Hospital Road, Kafin-Koro	Paikoro	Niger
5430	NG/0255	NG/0255/S/14	Basic Health Clinic, Agwara		Agwara, Niger State	Agwara	Niger

5431	NG/0255	NG/0255/S/5	Basic Health Clinic, Agwara		Agwara, Niger State	Agwara	Niger
5432	NG/0255	NG/0255/S/6	Basic Health Clinic, Agwara		Agwara, Niger State	Agwara	Niger
5433	NG/0255	NG/0255/S/4	Basic Health Clinic, Agwara		Agwara, Niger State	Agwara	Niger
5434	NG/0255	NG/0255/S/1	Basic Health Clinic, Agwara		Agwara, Niger State	Agwara	Niger
5435	NG/0255	NG/0255/S/2	Basic Health Clinic, Agwara		Agwara, Niger State	Agwara	Niger
5436	NG/0255	NG/0255/S/3	Basic Health Clinic, Agwara		Agwara, Niger State	Agwara	Niger
5437	NG/0257	NG/0257/S/2	New Bethel Hospital & Maternity		Bosso Low Cost Senior Staff Qtrs	Bosso	Niger
5438	NG/0265	NG/0265/S/5	Abdulsalam Abubakar General Hospital		Gulu, Niger State	Lapai	Niger
5439	NG/0265	NG/0265/S/4	Abdulsalam Abubakar General Hospital		Gulu, Niger State	Lapai	Niger
5440	NG/0271	NG/0271/S/5	Umaru Musa Yar Adua Memorial Hospital		Abuja-Kaduna Road, Sabon Wuse, Niger State	Tafa	Niger
5441	NG/0271	NG/0271/S/4	Umaru Musa Yar Adua Memorial Hospital		Abuja-Kaduna Road, Sabon Wuse, Niger State	Tafa	Niger
5442	NG/0271	NG/0271/S/1	Umaru Musa Yar Adua Memorial Hospital		Abuja-Kaduna Road, Sabon Wuse, Niger State	Tafa	Niger
5443	NG/0271	NG/0271/S/7	Umaru Musa Yar Adua Memorial Hospital		Abuja-Kaduna Road, Sabon Wuse, Niger State	Tafa	Niger
5444	NG/0271	NG/0271/S/8	Umaru Musa Yar Adua Memorial Hospital		Abuja-Kaduna Road, Sabon Wuse, Niger State	Tafa	Niger
5445	NG/0272	NG/0272/S/5	Bazza Jasim Clinic			Chanchaga	Niger
5446	NG/0272	NG/0272/S/4	Bazza Jasim Clinic			Chanchaga	Niger
5447	NG/0272	NG/0272/S/3	Bazza Jasim Clinic			Chanchaga	Niger
5448	NG/0276	NG/0276/S/8	Kizito Dental Clinic		74 Morocco Road, Suleja, Niger State	Suleja	Niger
5449	NG/0279	NG/0279/S/5	Zuma Total Care Hospital		Suleja, Niger State	Suleja	Niger
5450	NG/0279	NG/0279/S/13	Zuma Total Care Hospital		Suleja, Niger State	Suleja	Niger
5451	NG/0279	NG/0279/S/4	Zuma Total Care Hospital		Suleja, Niger State	Suleja	Niger
5452	NG/0279	NG/0279/S/7	Zuma Total Care Hospital		Suleja, Niger State	Suleja	Niger
5453	NG/0280	NG/0280/S/14	Diadem Medical & Ultra Diagnostic Limited		Behind Eco bank Plc, Minna Road, Suleja, Niger State.	Suleja	Niger
5454	NG/0283	NG/0283/S/1	Berith Specialist Hospital		Plot 16087, Minna Road, Kwamba Layout, Suleja, Niger State.	Suleja	Niger
5455	NG/0283	NG/0283/S/5	Berith Specialist Hospital		Plot 16087, Minna Road, Kwamba Layout, Suleja, Niger State.	Suleja	Niger
5456	NG/0283	NG/0283/S/8	Berith Specialist Hospital		Plot 16087, Minna Road, Kwamba Layout, Suleja, Niger State.	Suleja	Niger
5457	NG/0283	NG/0283/S/3	Berith Specialist Hospital		Plot 16087, Minna Road, Kwamba Layout, Suleja, Niger State.	Suleja	Niger
5458	NG/0283	NG/0283/S/2	Berith Specialist Hospital		Plot 16087, Minna Road, Kwamba Layout, Suleja, Niger State.	Suleja	Niger
5459	NG/0283	NG/0283/S/7	Berith Specialist Hospital		Plot 16087, Minna Road, Kwamba Layout, Suleja, Niger State.	Suleja	Niger
5460	NG/0283	NG/0283/S/6	Berith Specialist Hospital		Plot 16087, Minna Road, Kwamba Layout, Suleja, Niger State.	Suleja	Niger
5461	NG/0283	NG/0283/S/4	Berith Specialist Hospital		Plot 16087, Minna Road, Kwamba Layout, Suleja, Niger State.	Suleja	Niger
5462	NG/0285	NG/0285/S/8	Chelle Dental Clinic		No. 10811, Nulge Shopping Complex, Tunga, Minna, Niger State	Chanchaga	Niger
5463	NG/0286	NG/0286/S/7	Optima Scan Services Limited.		By 7up Depot Shiroro Road, Minna Niger State.	Chanchaga	Niger
5464	NG/0286	NG/0286/S/5	Optima Scan Services Limited.		By 7up Depot Shiroro Road, Minna Niger State.	Chanchaga	Niger
5465	NW/0001	NW/0001/S/7	May Day Specialist Hospital & Maternity	-	Esu Karu Road, opp Skye Bank Mararaba	Karu	Nasarawa
5466	NW/0001	NW/0001/S/4	May Day Specialist Hospital & Maternity	-	Esu Karu Road, opp Skye Bank Mararaba	Karu	Nasarawa

5467	NW/0001	NW/0001/S/3	May Day Specialist Hospital & Maternity	-	Esu Karu Road, opp Skye Bank Mararaba	Karu	Nasarawa
5468	NW/0001	NW/0001/S/2	May Day Specialist Hospital & Maternity	-	Esu Karu Road, opp Skye Bank Mararaba	Karu	Nasarawa
5469	NW/0001	NW/0001/S/1	May Day Specialist Hospital & Maternity	-	Esu Karu Road, opp Skye Bank Mararaba	Karu	Nasarawa
5470	NW/0001	NW/0001/S/5	May Day Specialist Hospital & Maternity	-	Esu Karu Road, opp Skye Bank Mararaba	Karu	Nasarawa
5471	NW/0001	NW/0001/S/6	May Day Specialist Hospital & Maternity	-	Esu Karu Road, opp Skye Bank Mararaba	Karu	Nasarawa
5472	NW/0003	NW/0003/S/10	Federal Medical Centre, keffi	Radiology, Internal Med., Gen. Surgery, Paediatrics, O & G, Pharmacy, Laboratory, Dental, Opthalmology, Orthalmology, Physiotherapy	Keffi	Keffi	Nasarawa
5473	NW/0003	NW/0003/S/3	Federal Medical Centre, keffi	Radiology, Internal Med., Gen. Surgery, Paediatrics, O & G, Pharmacy, Laboratory, Dental, Opthalmology, Orthalmology, Physiotherapy	Keffi	Keffi	Nasarawa
5474	NW/0003	NW/0003/S/15	Federal Medical Centre, keffi	Radiology, Internal Med., Gen. Surgery, Paediatrics, O & G, Pharmacy, Laboratory, Dental, Opthalmology, Orthalmology, Physiotherapy	Keffi	Keffi	Nasarawa
5475	NW/0003	NW/0003/S/8	Federal Medical Centre, keffi	Radiology, Internal Med., Gen. Surgery, Paediatrics, O & G, Pharmacy, Laboratory, Dental, Opthalmology, Orthalmology, Physiotherapy	Keffi	Keffi	Nasarawa
5476	NW/0003	NW/0003/S/5	Federal Medical Centre, keffi	Radiology, Internal Med., Gen. Surgery, Paediatrics, O & G, Pharmacy, Laboratory, Dental, Opthalmology, Orthalmology, Physiotherapy	Keffi	Keffi	Nasarawa
5477	NW/0003	NW/0003/S/2	Federal Medical Centre, keffi	Radiology, Internal Med., Gen. Surgery, Paediatrics, O & G, Pharmacy, Laboratory, Dental, Opthalmology, Orthalmology, Physiotherapy	Keffi	Keffi	Nasarawa
5478	NW/0003	NW/0003/S/1	Federal Medical Centre, keffi	Radiology, Internal Med., Gen. Surgery, Paediatrics, O & G, Pharmacy, Laboratory, Dental, Opthalmology, Orthalmology, Physiotherapy	Keffi	Keffi	Nasarawa

5479	NW/0003	NW/0003/S/4	Federal Medical Centre, keffi	Radiology, Internal Med., Gen. Surgery, Paediatrics, O & G, Pharmacy, Laboratory, Dental, Opthalmology, Orthalmology, Physiotherapy	Keffi	Keffi	Nasarawa
5480	NW/0003	NW/0003/S/11	Federal Medical Centre, keffi	Radiology, Internal Med., Gen. Surgery, Paediatrics, O & G, Pharmacy, Laboratory, Dental, Opthalmology, Orthalmology, Physiotherapy	Keffi	Keffi	Nasarawa
5481	NW/0003	NW/0003/S/6	Federal Medical Centre, keffi	Radiology, Internal Med., Gen. Surgery, Paediatrics, O & G, Pharmacy, Laboratory, Dental, Opthalmology, Orthalmology, Physiotherapy	Keffi	Keffi	Nasarawa
5482	NW/0003	NW/0003/S/7	Federal Medical Centre, keffi	Radiology, Internal Med., Gen. Surgery, Paediatrics, O & G, Pharmacy, Laboratory, Dental, Opthalmology, Orthalmology, Physiotherapy	Keffi	Keffi	Nasarawa
5483	NW/0004	NW/0004/S/4	Adonai Hospital		2, Adonai Close Off Old Karu Road, Mararaba	Karu	Nasarawa
5484	NW/0004	NW/0004/S/3	Adonai Hospital		2, Adonai Close Off Old Karu Road, Mararaba	Karu	Nasarawa
5485	NW/0004	NW/0004/S/1	Adonai Hospital		2, Adonai Close Off Old Karu Road, Mararaba	Karu	Nasarawa
5486	NW/0004	NW/0004/S/2	Adonai Hospital		2, Adonai Close Off Old Karu Road, Mararaba	Karu	Nasarawa
5487	NW/0006	NW/0006/S/4	General Hospital, Keffi		Keffi	Keffi	Nasarawa
5488	NW/0006	NW/0006/S/5	General Hospital, Keffi		Keffi	Keffi	Nasarawa
5489	NW/0008	NW/0008/S/5	General Hospital, Uke		Uke	Karu	Nasarawa
5490	NW/0008	NW/0008/S/4	General Hospital, Uke		Uke	Karu	Nasarawa
5491	NW/0009	NW/0009/S/1	General Hospital, Akwanga		Akwanga	Akwanga	Nasarawa
5492	NW/0009	NW/0009/S/4	General Hospital, Akwanga		Akwanga	Akwanga	Nasarawa
5493	NW/0011	NW/0011/S/5	General Hospital, Nasarawa Eggon		Nasarawa Eggon	Nasarawa Eggon	Nasarawa
5494	NW/0011	NW/0011/S/4	General Hospital, Nasarawa Eggon		Nasarawa Eggon	Nasarawa Eggon	Nasarawa
5495	NW/0012	NW/0012/S/5	General Hospital, Keana		Keana	Keana	Nasarawa
5496	NW/0013	NW/0013/S/5	General Hospital, Awe	Pharmacy, Laboratory	Awe, Nasarawa State	Awe	Nasarawa
5497	NW/0013	NW/0013/S/4	General Hospital, Awe	Pharmacy, Laboratory	Awe, Nasarawa State	Awe	Nasarawa
5498	NW/0014	NW/0014/S/5	General Hospital, Obi		Obi, Nasarawa State	Obi	Nasarawa
5499	NW/0014	NW/0014/S/4	General Hospital, Obi		Obi, Nasarawa State	Obi	Nasarawa
5500	NW/0015	NW/0015/S/4	General Hospital,Wamba		Wamba, Nasarawa State	Wamba	Nasarawa
5501	NW/0015	NW/0015/S/5	General Hospital,Wamba		Wamba, Nasarawa State	Wamba	Nasarawa
5502	NW/0016	NW/0016/S/2	Dalhatu Araf (Specialist Hospital)		Nasarawa	Lafia	Nasarawa
5503	NW/0016	NW/0016/S/6	Dalhatu Araf (Specialist Hospital)		Nasarawa	Lafia	Nasarawa
5504	NW/0016	NW/0016/S/3	Dalhatu Araf (Specialist Hospital)		Nasarawa	Lafia	Nasarawa
5505	NW/0016	NW/0016/S/7	Dalhatu Araf (Specialist Hospital)		Nasarawa	Lafia	Nasarawa
5506	NW/0016	NW/0016/S/5	Dalhatu Araf (Specialist Hospital)		Nasarawa	Lafia	Nasarawa
5507	NW/0016	NW/0016/S/11	Dalhatu Araf (Specialist Hospital)		Nasarawa	Lafia	Nasarawa

5508	NW/0016	NW/0016/S/1	Dalhatu Araf (Specialist Hospital)		Nasarawa	Lafia	Nasarawa
5509	NW/0016	NW/0016/S/8	Dalhatu Araf (Specialist Hospital)		Nasarawa	Lafia	Nasarawa
5510	NW/0016	NW/0016/S/15	Dalhatu Araf (Specialist Hospital)		Nasarawa	Lafia	Nasarawa
5511	NW/0016	NW/0016/S/4	Dalhatu Araf (Specialist Hospital)		Nasarawa	Lafia	Nasarawa
5512	NW/0020	NW/0020/S/5	Sauki Hospital	Laboratory	Shendam Road, Lafia	Lafia	Nasarawa
5513	NW/0030	NW/0030/S/4	Meblink Pharmacy	Pharmacy	Sharp Corner, Shop 2, Mararaba	Karu	Nasarawa
5514	NW/0032	NW/0032/S/4	A. Chuks Pharmacy	Pharmacy	Keffi Road Beside Pico Aluminium Mararaba	Karu	Nasarawa
5515	NW/0033	NW/0033/S/4	Medical Ink Pharmacy	Pharmacy	Sharp Corner, Shop 2, Mararaba	Karu	Nasarawa
5516	NW/0034	NW/0034/S/4	Isaj Pharmacy, Chemist (Nig.) Ltd.	Pharmacy	No. 61 Umaru Makama Dogo Road, Nasarawa	Nasarawa	Kano
5517	NW/0036	NW/0036/S/4	Honey Darn Pharmacy	Pharmacy	Jos Road, Lafia	Lafia	Nasarawa
5518	NW/0039	NW/0039/S/1	Multi Tex laboratory	Laboratory	Mararaba Opp. Midland Plaza, Mararaba	Karu	Nasarawa
5519	NW/0040	NW/0040/S/5	El-Shaddai Medical Lab. Ltd.	Laboratory	Behind Meblink Pharmacy, Sharp Corner, B/Stop Mararaba	Karu	Nasarawa
5520	NW/0041	NW/0041/S/4	Salem Pharmacy	Pharmacy	Opp. Aso Rd., Mararaba, Gurku	Karu	Nasarawa
5521	NW/0050	NW/0050/S/3	Nagari Allah Magani Clinic & Maternity		Behind Chief Magistrate Court, Keffi.	Keffi	Nasarawa
5522	NW/0050	NW/0050/S/4	Nagari Allah Magani Clinic & Maternity		Behind Chief Magistrate Court, Keffi.	Keffi	Nasarawa
5523	NW/0050	NW/0050/S/7	Nagari Allah Magani Clinic & Maternity		Behind Chief Magistrate Court, Keffi.	Keffi	Nasarawa
5524	NW/0050	NW/0050/S/1	Nagari Allah Magani Clinic & Maternity		Behind Chief Magistrate Court, Keffi.	Keffi	Nasarawa
5525	NW/0052	NW/0052/S/6	Maraba Guruku Medical Center	Primary	Mararaba Int. Mkt Mararaba	Karu	Nasarawa
5526	NW/0052	NW/0052/S/5	Maraba Guruku Medical Center	Primary	Mararaba Int. Mkt Mararaba	Karu	Nasarawa
5527	NW/0052	NW/0052/S/2	Maraba Guruku Medical Center	Primary	Mararaba Int. Mkt Mararaba	Karu	Nasarawa
5528	NW/0052	NW/0052/S/7	Maraba Guruku Medical Center	Primary	Mararaba Int. Mkt Mararaba	Karu	Nasarawa
5529	NW/0052	NW/0052/S/3	Maraba Guruku Medical Center	Primary	Mararaba Int. Mkt Mararaba	Karu	Nasarawa
5530	NW/0052	NW/0052/S/4	Maraba Guruku Medical Center	Primary	Mararaba Int. Mkt Mararaba	Karu	Nasarawa
5531	NW/0052	NW/0052/S/1	Maraba Guruku Medical Center	Primary	Mararaba Int. Mkt Mararaba	Karu	Nasarawa
5532	NW/0060	NW/0060/S/5	Biomart Medical Laboratory	Laboratory	Along Expressy Bread/Deeper Life Church str Sharp Corner Maraba	Karu	Nasarawa
5533	NW/0062	NW/0062/S/4	Celex-Collins Pharmacy	Pharmacy	5 Abubakar Burga Rd	Keffi	Nasarawa
5534	NW/0074	NW/0074/S/4	OUR LADY OF APOSTLES HOSPITAL		OLA HOSPITAL, AKWANGA	Akwanga	Nasarawa
5535	NW/0074	NW/0074/S/1	OUR LADY OF APOSTLES HOSPITAL		OLA HOSPITAL, AKWANGA	Akwanga	Nasarawa
5536	NW/0074	NW/0074/S/5	OUR LADY OF APOSTLES HOSPITAL		OLA HOSPITAL, AKWANGA	Akwanga	Nasarawa
5537	NW/0086	NW/0086/S/5	Innovative Biotech Ltd	Laboratory	1 Abdul Abubakar Street, GRA, Keffi	Keffi	Nasarawa
5538	NW/0093	NW/0093/S/4	Nasarawa State University Health Centre		Keffi	Keffi	Nasarawa
5539	NW/0093	NW/0093/S/5	Nasarawa State University Health Centre		Keffi	Keffi	Nasarawa
5540	NW/0098	NW/0098/S/5	Livia Shammah Hospitals Limited		Opp. MRS Filling Station, beside Zimara Int. School, Abuja-Keffi Exp, Road Mararaba.	Karu	Nasarawa
5541	NW/0098	NW/0098/S/13	Livia Shammah Hospitals Limited		Opp. MRS Filling Station, beside Zimara Int. School, Abuja-Keffi Exp, Road Mararaba.	Karu	Nasarawa
5542	NW/0098	NW/0098/S/8	Livia Shammah Hospitals Limited		Opp. MRS Filling Station, beside Zimara Int. School, Abuja-Keffi Exp, Road Mararaba.	Karu	Nasarawa
5543	NW/0098	NW/0098/S/3	Livia Shammah Hospitals Limited		Opp. MRS Filling Station, beside Zimara Int. School, Abuja-Keffi Exp, Road Mararaba.	Karu	Nasarawa
5544	NW/0099	NW/0099/S/3	Shammah Clinics	Obs & Gynae	Off Criss Park Street, Mararaba, Nassarawa State	Karu	Nasarawa
5545	NW/0101	NW/0101/S/4	Lamb of God Phamacy	Pharmacy	1 Shendam Road, Lafia	Lafia	Nasarawa

5546	NW/0107	NW/0107/S/4	General Hospital Doma		Agbashi Road, Doma, Nasarawa State.	Doma	Nasarawa
5547	NW/0107	NW/0107/S/5	General Hospital Doma		Agbashi Road, Doma, Nasarawa State.	Doma	Nasarawa
5548	NW/0108	NW/0108/S/4	God is able Pharmacy		Km 20, Abuja-Keffi Road, Masaka U-turn, Karu, Nasarawa State	Karu	Nasarawa
5549	NW/0119	NW/0119/S/3	Edither Choice Hospital		1 Mohammed Mana Street Off Abacha Road,	Karu	Nasarawa
5550	NW/0119	NW/0119/S/1	Edither Choice Hospital		1 Mohammed Mana Street Off Abacha Road,	Karu	Nasarawa
5551	NW/0121	NW/0121/S/4	Oshyeg'Ba Josh Medical Centre Lafia		Behind Ministry Of Justice, Off Shendam Road, Lafia Nasarawa State	Lafia	Nasarawa
5552	NW/0128	NW/0128/S/1	Oshyeg'Ba Josh Medical Centre Lafia		Behind Ministry Of Justice, Off Shendam Road, Lafia Nasarawa State	Lafia	Nasarawa
5553	NW/0136	NW/0136/S/5	San Health Project Lafia East		Newtaal Road Along Lafia East Primary School Lafia	Lafia	Nasarawa
5554	OD/0001	OD/0001/S/8	State Specialist Hospital,Ikare		Ikare Akoko	Akure South	Ondo
5555	OD/0001	OD/0001/S/15	State Specialist Hospital,Ikare		Ikare Akoko	Akure South	Ondo
5556	OD/0001	OD/0001/S/5	State Specialist Hospital,Ikare		Ikare Akoko	Akure South	Ondo
5557	OD/0001	OD/0001/S/7	State Specialist Hospital,Ikare		Ikare Akoko	Akure South	Ondo
5558	OD/0001	OD/0001/S/4	State Specialist Hospital,Ikare		Ikare Akoko	Akure South	Ondo
5559	OD/0005	OD/0005/S/2	Sijuwade Specialist Hospital		10,SIJUWADE BAA-SEGUN RD, AKURE	Akure North	Ondo
5560	OD/0005	OD/0005/S/6	Sijuwade Specialist Hospital		10,SIJUWADE BAA-SEGUN RD, AKURE	Akure North	Ondo
5561	OD/0005	OD/0005/S/7	Sijuwade Specialist Hospital		10,SIJUWADE BAA-SEGUN RD, AKURE	Akure North	Ondo
5562	OD/0005	OD/0005/S/14	Sijuwade Specialist Hospital		10,SIJUWADE BAA-SEGUN RD, AKURE	Akure North	Ondo
5563	OD/0005	OD/0005/S/4	Sijuwade Specialist Hospital		10,SIJUWADE BAA-SEGUN RD, AKURE	Akure North	Ondo
5564	OD/0006	OD/0006/S/5	St. David's Hospital		OBA ADESIDA RD.AKURE	Akure North	Ondo
5565	OD/0007	OD/0007/S/7	St. John & Mary Hospital		2 Adebayo Onilane Isikan Allase Akure	Akure North	Ondo
5566	OD/0007	OD/0007/S/5	St. John & Mary Hospital		2 Adebayo Onilane Isikan Allase Akure	Akure North	Ondo
5567	OD/0009	OD/0009/S/1	Joe-Jane Medicine Centre		Okejebu Rd. Akure	Akure North	Ondo
5568	OD/0009	OD/0009/S/5	Joe-Jane Medicine Centre		Okejebu Rd. Akure	Akure North	Ondo
5569	OD/0009	OD/0009/S/7	Joe-Jane Medicine Centre		Okejebu Rd. Akure	Akure North	Ondo
5570	OD/0010	OD/0010/S/7	Abitoye Hospital		101, Ijoka Road, Akure	Akure North	Ondo
5571	OD/0011	OD/0011/S/3	State Specialist Hospital, Akure		Akure	Akure North	Ondo
5572	OD/0011	OD/0011/S/7	State Specialist Hospital, Akure		Akure	Akure North	Ondo
5573	OD/0011	OD/0011/S/4	State Specialist Hospital, Akure		Akure	Akure North	Ondo
5574	OD/0011	OD/0011/S/1	State Specialist Hospital, Akure		Akure	Akure North	Ondo
5575	OD/0011	OD/0011/S/2	State Specialist Hospital, Akure		Akure	Akure North	Ondo
5576	OD/0011	OD/0011/S/12	State Specialist Hospital, Akure		Akure	Akure North	Ondo
5577	OD/0011	OD/0011/S/5	State Specialist Hospital, Akure		Akure	Akure North	Ondo
5578	OD/0011	OD/0011/S/8	State Specialist Hospital, Akure		Akure	Akure North	Ondo
5579	OD/0011	OD/0011/S/11	State Specialist Hospital, Akure		Akure	Akure North	Ondo
5580	OD/0011	OD/0011/S/15	State Specialist Hospital, Akure		Akure	Akure North	Ondo
5581	OD/0015	OD/0015/S/7	State Specialist Hospital,Okitipupa		Okitipupa	Okitipupa	Ondo
5582	OD/0015	OD/0015/S/8	State Specialist Hospital,Okitipupa		Okitipupa	Okitipupa	Ondo
5583	OD/0015	OD/0015/S/4	State Specialist Hospital,Okitipupa		Okitipupa	Okitipupa	Ondo
5584	OD/0015	OD/0015/S/15	State Specialist Hospital,Okitipupa		Okitipupa	Okitipupa	Ondo
5585	OD/0015	OD/0015/S/5	State Specialist Hospital,Okitipupa		Okitipupa	Okitipupa	Ondo
5586	OD/0018	OD/0018/S/2	Federal Medical Centre - Owo		Owo	Owo	Ondo
5587	OD/0018	OD/0018/S/6	Federal Medical Centre - Owo		Owo	Owo	Ondo
5588	OD/0018	OD/0018/S/10	Federal Medical Centre - Owo		Owo	Owo	Ondo

5589	OD/0018	OD/0018/S/4	Federal Medical Centre - Owo		Owo	Owo	Ondo
5590	OD/0018	OD/0018/S/5	Federal Medical Centre - Owo		Owo	Owo	Ondo
5591	OD/0018	OD/0018/S/3	Federal Medical Centre - Owo		Owo	Owo	Ondo
5592	OD/0018	OD/0018/S/1	Federal Medical Centre - Owo		Owo	Owo	Ondo
5593	OD/0018	OD/0018/S/7	Federal Medical Centre - Owo		Owo	Owo	Ondo
5594	OD/0021	OD/0021/S/5	State Specialist Hospital,Ondo		Ondo	Akure North	Ondo
5595	OD/0021	OD/0021/S/7	State Specialist Hospital,Ondo		Ondo	Akure North	Ondo
5596	OD/0021	OD/0021/S/4	State Specialist Hospital,Ondo		Ondo	Akure North	Ondo
5597	OD/0021	OD/0021/S/8	State Specialist Hospital,Ondo		Ondo	Akure North	Ondo
5598	OD/0021	OD/0021/S/15	State Specialist Hospital,Ondo		Ondo	Akure North	Ondo
5599	OD/0021	OD/0021/S/11	State Specialist Hospital,Ondo		Ondo	Akure North	Ondo
5600	OD/0022	OD/0022/S/7	Oludare Hospital		Fanibi Layout, Akure	Akure North	Ondo
5601	OD/0022	OD/0022/S/5	Oludare Hospital		Fanibi Layout, Akure	Akure North	Ondo
5602	OD/0022	OD/0022/S/4	Oludare Hospital		Fanibi Layout, Akure	Akure North	Ondo
5603	OD/0027	OD/0027/S/4	Tolfad Pharmacy	Pharmacy	Akure	Akure South	Ondo
5604	OD/0030	OD/0030/S/4	Betak Pharmacy	Pharmacy	Ikare Akoko	Akoko North East	Ondo
5605	OD/0031	OD/0031/S/4	Akinbinu Pharmacy	Pharmacy	Ondo	Ondo West	Ondo
5606	OD/0032	OD/0032/S/4	Finolex Pharmacy	Pharamacy	Ondo	Ondo West	Ondo
5607	OD/0035	OD/0035/S/11	Joymas Phsiotherapy	Physiotherapy	Owo Road, Akure	Akure South	Ondo
5608	OD/0036	OD/0036/S/4	Welfast Pharmacy	Pharmacy	135, Hospital Road, Akure	Akure South	Ondo
5609	OD/0037	OD/0037/S/4	Dele Ayo Pharmacy Limited	Pharmacy	No. 40 (Old 27) Ijebu Street Akure	Akure South	Ondo
5610	OD/0039	OD/0039/S/4	De-Zubik Pharmaceutical Ltd	Pharmacy	15 Ondo Road Akure	Akure South	Ondo
5611	OD/0041	OD/0041/S/4	Tunde & Tunde Pharmacy	Pharmacy	5 Aguda Street Ijokodo, Okitipupa	Okitipupa	Ondo
5612	OD/0045	OD/0045/S/4	Adeyemi College Of Education Health Centre		Ondo State	Akure North	Ondo
5613	OD/0047	OD/0047/S/3	Ayo Specialist Hospital	O & G	Off Illesha Rd., Akure	Akure South	Ondo
5614	OD/0052	OD/0052/S/1	Liberty Hospital & Maternity	General Surgery	Oluwatunyi Quarters off Ijoko Road ,Akure	Akure South	Ondo
5615	OD/0053	OD/0053/S/7	First Mercy Specialist Hospital		19c Gbogi street, off Osa Adesida Road, Akure	Akure South	Ondo
5616	OD/0053	OD/0053/S/4	First Mercy Specialist Hospital		19c Gbogi street, off Osa Adesida Road, Akure	Akure South	Ondo
5617	OD/0053	OD/0053/S/3	First Mercy Specialist Hospital		19c Gbogi street, off Osa Adesida Road, Akure	Akure South	Ondo
5618	OD/0053	OD/0053/S/6	First Mercy Specialist Hospital		19c Gbogi street, off Osa Adesida Road, Akure	Akure South	Ondo
5619	OD/0053	OD/0053/S/5	First Mercy Specialist Hospital		19c Gbogi street, off Osa Adesida Road, Akure	Akure South	Ondo
5620	OD/0053	OD/0053/S/1	First Mercy Specialist Hospital		19c Gbogi street, off Osa Adesida Road, Akure	Akure South	Ondo
5621	OD/0054	OD/0054/S/1	MOMAAK Specialist Hospital		3 Lafe Inn way, Okuta Eleri	Akure South	Ondo
5622	OD/0054	OD/0054/S/12	MOMAAK Specialist Hospital		3 Lafe Inn way, Okuta Eleri	Akure South	Ondo
5623	OD/0057	OD/0057/S/3	City Specialist Hospital		56 Oke Arata street, Igbo-Oliki, Akure, Ondo State	Akure South	Ondo
5624	OD/0058	OD/0058/S/7	Adedewe Okunriboye Hospital		Opposite Celestial Church, Ondo Road Bye pass, Akure	Akure South	Ondo
5625	OD/0058	OD/0058/S/5	Adedewe Okunriboye Hospital		Opposite Celestial Church, Ondo Road Bye pass, Akure	Akure South	Ondo
5626	OD/0062	OD/0062/S/6	Ogunsusi Memorial Hospital		1 Zion Estate, Ile-oluji	Ileoluji/Okeigbo	Ondo
5627	OD/0064	OD/0064/S/1	Fujah Specialist Hospital		22, Adetutu Street, Opp. Isikan Market, Akure, Ondo State	Akure South	Ondo
5628	OD/0064	OD/0064/S/10	Fujah Specialist Hospital		22, Adetutu Street, Opp. Isikan Market, Akure, Ondo State	Akure South	Ondo
5629	OD/0064	OD/0064/S/3	Fujah Specialist Hospital		22, Adetutu Street, Opp. Isikan Market, Akure, Ondo State	Akure South	Ondo
5630	OD/0068	OD/0068/S/4	Kibs Pharmacy	Pharmacy	91 Oba adesida Rd Akure	Akure South	Ondo
5631	OD/0069	OD/0069/S/15	Stephens Eye & Medical Centre	Opthalmology	7A Mode Street Yaba Ondo	Ondo West	Ondo
5632	OD/0072	OD/0072/S/11	E. M. Physiotheraphy Services	Physiotheraphy	61 Brig Ademulegun Rd Ondo	Ondo West	Ondo
5633	OD/0074	OD/0074/S/5	Police Clinics, Ondo		Ondo State	Ondo West	Ondo
5634	OD/0074	OD/0074/S/3	Police Clinics, Ondo		Ondo State	Ondo West	Ondo

5635	OD/0074	OD/0074/S/13	Police Clinics, Ondo		Ondo State	Ondo West	Ondo
5636	OD/0074	OD/0074/S/6	Police Clinics, Ondo		Ondo State	Ondo West	Ondo
5637	OD/0074	OD/0074/S/4	Police Clinics, Ondo		Ondo State	Ondo West	Ondo
5638	OD/0074	OD/0074/S/2	Police Clinics, Ondo		Ondo State	Ondo West	Ondo
5639	OD/0074	OD/0074/S/8	Police Clinics, Ondo		Ondo State	Ondo West	Ondo
5640	OD/0074	OD/0074/S/7	Police Clinics, Ondo		Ondo State	Ondo West	Ondo
5641	OD/0074	OD/0074/S/1	Police Clinics, Ondo		Ondo State	Ondo West	Ondo
5642	OD/0074	OD/0074/S/14	Police Clinics, Ondo		Ondo State	Ondo West	Ondo
5643	OD/0074	OD/0074/S/11	Police Clinics, Ondo		Ondo State	Ondo West	Ondo
5644	OD/0076	OD/0076/S/4	Federal University of Technology Health Centre		Akure, Ondo State	Akure North	Ondo
5645	OD/0076	OD/0076/S/5	Federal University of Technology Health Centre		Akure, Ondo State	Akure North	Ondo
5646	OD/0081	OD/0081/S/5	BABALOLA NINEWELLS SPEC. HOSPITAL		46, ABITOYE STREET,OSHOKOTI LAYOUT,AKURE	Akure South	Ondo
5647	OD/0081	OD/0081/S/1	BABALOLA NINEWELLS SPEC. HOSPITAL		46, ABITOYE STREET,OSHOKOTI LAYOUT,AKURE	Akure South	Ondo
5648	OD/0082	OD/0082/S/14	ARIF MEDICAL CENTER		BEHIND SABO MAIN MARKET ORE	Odigbo	Ondo
5649	OD/0082	OD/0082/S/5	ARIF MEDICAL CENTER		BEHIND SABO MAIN MARKET ORE	Odigbo	Ondo
5650	OD/0082	OD/0082/S/7	ARIF MEDICAL CENTER		BEHIND SABO MAIN MARKET ORE	Odigbo	Ondo
5651	OD/0083	OD/0083/S/1	SHEKINAH HOSPITALS		BEHIND OLD OWO GARAGE, OFF EL-SHADDAI RD. AKURE	Akure South	Ondo
5652	OD/0098	OD/0098/S/5	HALLMARK MEDICAL LAB.SERVICES	LABORATORY	57B, ARAKARE RD.,AKURE	Akure South	Ondo
5653	OD/0105	OD/0105/S/2	Kharis Medical Centre		2 Ondo Bye-Pass JUT, Oyeme Road	Akure South	Ondo
5654	OD/0108	OD/0108/S/5	Sckye Hospital Ltd		83B Oba Adesida Road, Akure	Akure South	Ondo
5655	OD/0108	OD/0108/S/4	Sckye Hospital Ltd		83B Oba Adesida Road, Akure	Akure South	Ondo
5656	OD/0108	OD/0108/S/3	Sckye Hospital Ltd		83B Oba Adesida Road, Akure	Akure South	Ondo
5657	OD/0111	OD/0111/S/15	Tomisan Specialist Eye Clinic		Within Joe Jane Medical Centre, Akure	Akure South	Ondo
5658	OD/0113	OD/0113/S/5	General Hospital, Ile Oluji		Ile Oluji, Ondo State	Ileoluji/Okeigbo	Ondo
5659	OD/0113	OD/0113/S/4	General Hospital, Ile Oluji		Ile Oluji, Ondo State	Ileoluji/Okeigbo	Ondo
5660	OD/0114	OD/0114/S/4	General Hospital		Opp Anglican Grammar School, Oke-Ogbe, Iju-Itaogbolu, Ondo State	Akure North	Ondo
5661	OD/0114	OD/0114/S/7	General Hospital		Opp Anglican Grammar School, Oke-Ogbe, Iju-Itaogbolu, Ondo State	Akure North	Ondo
5662	OD/0117	OD/0117/S/15	Eniola Medical Clinic		Building 2, York City Close, Ago Itunu, Ondo	Ondo West	Ondo
5663	OD/0118	OD/0118/S/7	General Hospital Igbokoda		Omomira Street, Igbokoda	Ilaje	Ondo
5664	OD/0118	OD/0118/S/5	General Hospital Igbokoda		Omomira Street, Igbokoda	Ilaje	Ondo
5665	OD/0118	OD/0118/S/4	General Hospital Igbokoda		Omomira Street, Igbokoda	Ilaje	Ondo
5666	OD/0118	OD/0118/S/3	General Hospital Igbokoda		Omomira Street, Igbokoda	Ilaje	Ondo
5667	OD/0122	OD/0122/S/1	Hopeland Specialist Medical Center		1, Arikawe Street, Akure, Ondo state	Akure South	Ondo
5668	OD/0122	OD/0122/S/3	Hopeland Specialist Medical Center		1, Arikawe Street, Akure, Ondo state	Akure South	Ondo
5669	OD/0127	OD/0127/S/3	Victory Specialist Hospital		4, Victory Street, Ibitayo Estate, Ondo	Ondo West	Ondo
5670	OD/0131	OD/0131/S/1	Owoyemi Specialist Hospital		5, Oshinle road, Akure-ondo, Olu Kayode roundabout, Akure, Ondo State.	Akure South	Ondo
5671	OD/0136	OD/0136/S/4	Pharmacy First O		1, Opp. Owe Akala Junction, Oke Aro Akure Ondo State	Akure North	Ondo
5672	OD/0137	OD/0137/S/4	His Grace KSP Pharm. LTD		1a Aule Road,Akure	Akure South	Ondo
5673	OD/0138	OD/0138/S/4	Julie Bolakit Pharmaceutical LTD		Block 1,Old Customs Buildings, Ondo Road Akure	Akure South	Ondo
5674	OD/0139	OD/0139/S/5	Davosach Med. Lab.		No 133, State Hospital road, Akure	Akure South	Ondo
5675	OD/0140	OD/0140/S/15	Millennium Eye Clinic		State Specialist Hospital Akure Ondo State	Akure South	Ondo
5676	OD/0141	OD/0141/S/4	BOT Pharmacy		144,Hospital Road Akure	Akure South	Ondo
5677	OD/0142	OD/0142/S/13	Britaview Eye Clinic		102a Hospital Road Akure	Akure South	Ondo
5678	OD/0148	OD/0148/S/4	Fegtochi Pharmacy		35 Oluwatuyi Quarters Akure Ondo State	Akure South	Ondo

5679	OD/0149	OD/0149/S/3	Goshenland Specialist Hospital & Fertility Centre		Block A. Plot 35 Aaye Community Oda Road. Akure Ondo State	Akure South	Ondo
5680	OD/0150	OD/0150/S/14	Midas Clinic		65,Oke-Ijebu Street Akure Ondo State	Akure South	Ondo
5681	OD/0150	OD/0150/S/16	Midas Clinic		65,Oke-Ijebu Street Akure Ondo State	Akure South	Ondo
5682	OD/0151	OD/0151/S/7	Paramount Specialist Hospital & Fertility Centre		22 Sydney Emmanuel Crescent, Valentino Street, Ondo State	Ondo West	Ondo
5683	OD/0151	OD/0151/S/3	Paramount Specialist Hospital & Fertility Centre		22 Sydney Emmanuel Crescent, Valentino Street, Ondo State	Ondo West	Ondo
5684	OD/0152	OD/0152/S/13	His Apple Eye Clinic		63A Oyemekun Road, Akure Ondo state	Akure South	Ondo
5685	OD/0153	OD/0153/S/7	Paramount Spec. Hosp. & Fertility Centre		22 Sydney Emmanuel Crescent, Valentino St. Ondo State	Ondo West	Ondo
5686	OD/0155	OD/0155/S/1	Kaymas Medical Centre		Newtown, Oke-Aro, Akure Ondo State	Akure South	Ondo
5687	OD/0156	OD/0156/S/7	Miteda Specialist Hospital		Plot 5,Laoye Adegoke Road, Alagbaka, Akure Ondo State	Akure South	Ondo
5688	OD/0156	OD/0156/S/5	Miteda Specialist Hospital		Plot 5,Laoye Adegoke Road, Alagbaka, Akure Ondo State	Akure South	Ondo
5689	OD/0157	OD/0157/S/3	Blue Print Specialist Clinic		3, Falodun Avenue Oke-Ogun, Owo Ondo State	Owo	Ondo
5690	OD/0158	OD/0158/S/3	Pima Hospital & Maternity		Off Okitipupa Road, Ore Ondo State	Odigbo	Ondo
5691	OG/0001	OG/0001/S/3	Federal Medical Centre, Abeokuta		Bisi Onabanjo Way, Idi-Aba, Abeokuta, Ogun State	Abeokuta South	Ogun
5692	OG/0001	OG/0001/S/4	Federal Medical Centre, Abeokuta		Bisi Onabanjo Way, Idi-Aba, Abeokuta, Ogun State	Abeokuta South	Ogun
5693	OG/0001	OG/0001/S/7	Federal Medical Centre, Abeokuta		Bisi Onabanjo Way, Idi-Aba, Abeokuta, Ogun State	Abeokuta South	Ogun
5694	OG/0001	OG/0001/S/10	Federal Medical Centre, Abeokuta		Bisi Onabanjo Way, Idi-Aba, Abeokuta, Ogun State	Abeokuta South	Ogun
5695	OG/0001	OG/0001/S/15	Federal Medical Centre, Abeokuta		Bisi Onabanjo Way, Idi-Aba, Abeokuta, Ogun State	Abeokuta South	Ogun
5696	OG/0001	OG/0001/S/11	Federal Medical Centre, Abeokuta		Bisi Onabanjo Way, Idi-Aba, Abeokuta, Ogun State	Abeokuta South	Ogun
5697	OG/0001	OG/0001/S/1	Federal Medical Centre, Abeokuta		Bisi Onabanjo Way, Idi-Aba, Abeokuta, Ogun State	Abeokuta South	Ogun
5698	OG/0001	OG/0001/S/2	Federal Medical Centre, Abeokuta		Bisi Onabanjo Way, Idi-Aba, Abeokuta, Ogun State	Abeokuta South	Ogun
5699	OG/0001	OG/0001/S/8	Federal Medical Centre, Abeokuta		Bisi Onabanjo Way, Idi-Aba, Abeokuta, Ogun State	Abeokuta South	Ogun
5700	OG/0001	OG/0001/S/6	Federal Medical Centre, Abeokuta		Bisi Onabanjo Way, Idi-Aba, Abeokuta, Ogun State	Abeokuta South	Ogun
5701	OG/0002	OG/0002/S/10	Mercy Group Clinics		Back Of Uba Building Pansheke, Ibara, Abeokuta	Abeokuta South	Ogun
5702	OG/0002	OG/0002/S/1	Mercy Group Clinics		Back Of Uba Building Pansheke, Ibara, Abeokuta	Abeokuta South	Ogun
5703	OG/0002	OG/0002/S/3	Mercy Group Clinics		Back Of Uba Building Pansheke, Ibara, Abeokuta	Abeokuta South	Ogun
5704	OG/0002	OG/0002/S/4	Mercy Group Clinics		Back Of Uba Building Pansheke, Ibara, Abeokuta	Abeokuta South	Ogun
5705	OG/0004	OG/0004/S/1	Femtob Specialist Hospital		No. 5 Obileye Street, Gra, Ijebu-Ode	Ijebu Ode	Ogun
5706	OG/0006	OG/0006/S/4	Oba Ademola Maternity Hospital		Ijemo, Abeokuta	Abeokuta South	Ogun
5707	OG/0006	OG/0006/S/3	Oba Ademola Maternity Hospital		Ijemo, Abeokuta	Abeokuta South	Ogun
5708	OG/0007	OG/0007/S/11	Neuropsychiatric Hospital		Aro, Abeokuta, Ogun State	Abeokuta North	Ogun
5709	OG/0007	OG/0007/S/5	Neuropsychiatric Hospital		Aro, Abeokuta, Ogun State	Abeokuta North	Ogun
5710	OG/0007	OG/0007/S/9	Neuropsychiatric Hospital		Aro, Abeokuta, Ogun State	Abeokuta North	Ogun
5711	OG/0007	OG/0007/S/4	Neuropsychiatric Hospital		Aro, Abeokuta, Ogun State	Abeokuta North	Ogun
5712	OG/0016	OG/0016/S/8	Ogun State Dental Clinic	Dental	Abeokuta, Ogun State	Ogun Water Side	Ogun

5713	OG/0017	OG/0017/S/4	Fontana Pharmaceutical Chemist & Stores Co. Ltd.	Pharmacy	75, Ejinrin Road, Ijebu-Ode, Ogun State	Ijebu Ode	Ogun
5714	OG/0018	OG/0018/S/4	NATBEL Pharmacy	Pharmacy	A5/32 Hospital Road, Shagamu, Ogun State	Sagamu	Ogun
5715	OG/0019	OG/0019/S/4	Precious Pharmacy & Stores Ltd.	Pharmacy	114, Awolowo Avenue, Omida, Abeokuta, Ogun State	Ogun Water Side	Ogun
5716	OG/0020	OG/0020/S/4	A-Z Pharmacy	Pharmacy	68, Okejigbo Street, Abeokuta, Ogun State	Abeokuta South	Ogun
5717	OG/0021	OG/0021/S/4	Nisam Pharmacy and Stores	Pharmacy	1, Kulo Road, Isabo, Abeokuta, Ogun State	Ogun Water Side	Ogun
5718	OG/0023	OG/0023/S/5	Kenny Ogun Lab. Complex	Laboratory	1, Kenny Ogun Lab. Street., Ilupeju Estate, Ita-Elegba, Abeo	Abeokuta North	Ogun
5719	OG/0026	OG/0026/S/4	State Hospital, Ijebu-Ode		Ijebu-Ode	Ijebu Ode	Ogun
5720	OG/0026	OG/0026/S/5	State Hospital, Ijebu-Ode		Ijebu-Ode	Ijebu Ode	Ogun
5721	OG/0026	OG/0026/S/11	State Hospital, Ijebu-Ode		Ijebu-Ode	Ijebu Ode	Ogun
5722	OG/0026	OG/0026/S/6	State Hospital, Ijebu-Ode		Ijebu-Ode	Ijebu Ode	Ogun
5723	OG/0026	OG/0026/S/3	State Hospital, Ijebu-Ode		Ijebu-Ode	Ijebu Ode	Ogun
5724	OG/0026	OG/0026/S/1	State Hospital, Ijebu-Ode		Ijebu-Ode	Ijebu Ode	Ogun
5725	OG/0027	OG/0027/S/7	State Hospital, Abeokuta		Abeokuta, Ogun State	Abeokuta South	Ogun
5726	OG/0027	OG/0027/S/15	State Hospital, Abeokuta		Abeokuta, Ogun State	Abeokuta South	Ogun
5727	OG/0027	OG/0027/S/11	State Hospital, Abeokuta		Abeokuta, Ogun State	Abeokuta South	Ogun
5728	OG/0027	OG/0027/S/4	State Hospital, Abeokuta		Abeokuta, Ogun State	Abeokuta South	Ogun
5729	OG/0027	OG/0027/S/2	State Hospital, Abeokuta		Abeokuta, Ogun State	Abeokuta South	Ogun
5730	OG/0027	OG/0027/S/3	State Hospital, Abeokuta		Abeokuta, Ogun State	Abeokuta South	Ogun
5731	OG/0027	OG/0027/S/6	State Hospital, Abeokuta		Abeokuta, Ogun State	Abeokuta South	Ogun
5732	OG/0027	OG/0027/S/1	State Hospital, Abeokuta		Abeokuta, Ogun State	Abeokuta South	Ogun
5733	OG/0027	OG/0027/S/5	State Hospital, Abeokuta		Abeokuta, Ogun State	Abeokuta South	Ogun
5734	OG/0028	OG/0028/S/4	Femtob Pharmaceutical Chemist & Stores	Pharmacy	1 Tal Bot Road, Opp. St. Augustine, Catholic Sch., Ijebu-Ode	Ijebu Ode	Ogun
5735	OG/0031	OG/0031/S/5	Hetta Medical Centre		15, Martina Road, Sango, Ota	Ado Odo-Ota	Ogun
5736	OG/0031	OG/0031/S/4	Hetta Medical Centre		15, Martina Road, Sango, Ota	Ado Odo-Ota	Ogun
5737	OG/0031	OG/0031/S/7	Hetta Medical Centre		15, Martina Road, Sango, Ota	Ado Odo-Ota	Ogun
5738	OG/0032	OG/0032/S/1	Larion Medical Centre		8, Laz Ukwuoma Close, Morogbo Agbara, Lagos.	Ado Odo-Ota	Ogun
5739	OG/0034	OG/0034/S/7	Rophe Medical Centre		23, Anambra Crescent, Agbara Estate, Ogun State.	Ado Odo-Ota	Ogun
5740	OG/0034	OG/0034/S/4	Rophe Medical Centre		23, Anambra Crescent, Agbara Estate, Ogun State.	Ado Odo-Ota	Ogun
5741	OG/0034	OG/0034/S/5	Rophe Medical Centre		23, Anambra Crescent, Agbara Estate, Ogun State.	Ado Odo-Ota	Ogun
5742	OG/0036	OG/0036/S/3	Medicare Clinic		105, Idi-Iroko Road, Otta, Ogun State.	Ado Odo-Ota	Ogun
5743	OG/0036	OG/0036/S/6	Medicare Clinic		105, Idi-Iroko Road, Otta, Ogun State.	Ado Odo-Ota	Ogun
5744	OG/0036	OG/0036/S/2	Medicare Clinic		105, Idi-Iroko Road, Otta, Ogun State.	Ado Odo-Ota	Ogun
5745	OG/0036	OG/0036/S/8	Medicare Clinic		105, Idi-Iroko Road, Otta, Ogun State.	Ado Odo-Ota	Ogun
5746	OG/0036	OG/0036/S/7	Medicare Clinic		105, Idi-Iroko Road, Otta, Ogun State.	Ado Odo-Ota	Ogun
5747	OG/0036	OG/0036/S/4	Medicare Clinic		105, Idi-Iroko Road, Otta, Ogun State.	Ado Odo-Ota	Ogun
5748	OG/0036	OG/0036/S/5	Medicare Clinic		105, Idi-Iroko Road, Otta, Ogun State.	Ado Odo-Ota	Ogun
5749	OG/0048	OG/0048/S/3	Tobiloba Clinic & Maternity	Primary Provider	1 Tobiloba Way off Kuforiji-olubi Estate, Adigbe, Abeokuta	Abeokuta North	Ogun
5750	OG/0052	OG/0052/S/2	Mayowa Integrated Medical Services Ltd, Idi-Iroko		1, ALAWODE WAY, IDI-IROKO	Ipokia	Ogun
5751	OG/0053	OG/0053/S/1	Central Specialist Hospital		133 Idiroko Rd Ota	Ado Odo-Ota	Ogun
5752	OG/0055	OG/0055/S/3	Twins Specialist Clinic		2 Gate GRA Ota	Ado Odo-Ota	Ogun

5753	OG/0057	OG/0057/S/7	Ayotola Specialist Hospital		3 Suraju Odunuga Street, Off Baruwa Str, Sagamu	Sagamu	Ogun
5754	OG/0058	OG/0058/S/5	Victoria Memorial Hospital		Morogbo Agbara	Ado Odo-Ota	Ogun
5755	OG/0060	OG/0060/S/2	Soremekun Memorial Hospital		127 Lagos-Abeokuta Rd, Vespa B/Stop, Ifo	Ifo	Ogun
5756	OG/0065	OG/0065/S/1	Owokoniran Memorial Hospital		3 Owokoniran Street, Makun Station, Sagamu	Sagamu	Ogun
5757	OG/0065	OG/0065/S/3	Owokoniran Memorial Hospital		3 Owokoniran Street, Makun Station, Sagamu	Sagamu	Ogun
5758	OG/0073	OG/0073/S/8	Faramed Clinics	Primary Provider	37 Akinwunmi Street Joji Rd. Ota	Ado Odo-Ota	Ogun
5759	OG/0073	OG/0073/S/5	Faramed Clinics	Primary Provider	37 Akinwunmi Street Joji Rd. Ota	Ado Odo-Ota	Ogun
5760	OG/0073	OG/0073/S/3	Faramed Clinics	Primary Provider	37 Akinwunmi Street Joji Rd. Ota	Ado Odo-Ota	Ogun
5761	OG/0076	OG/0076/S/1	Rubee Medical Centre Ltd		Km 38 Abeokuta Motor Road, Sango, Ota	Ado Odo-Ota	Ogun
5762	OG/0076	OG/0076/S/5	Rubee Medical Centre Ltd		Km 38 Abeokuta Motor Road, Sango, Ota	Ado Odo-Ota	Ogun
5763	OG/0076	OG/0076/S/7	Rubee Medical Centre Ltd		Km 38 Abeokuta Motor Road, Sango, Ota	Ado Odo-Ota	Ogun
5764	OG/0080	OG/0080/S/5	Ibukun Clinics		1 Agunloye Street, Otta	Ado Odo-Ota	Ogun
5765	OG/0081	OG/0081/S/5	Kolade Specialist Hospital		68 Idiroko Road, Sango Ota	Ado Odo-Ota	Ogun
5766	OG/0082	OG/0082/S/1	Ota Specialist Hospital Ltd		67 Idiroko Road, Ota	Ado Odo-Ota	Ogun
5767	OG/0083	OG/0083/S/4	Barbardox Pharmacy & Stores	Pharmacy	10 Llubu Street Oke Ilewo Abeokuta	Abeokuta South	Ogun
5768	OG/0089	OG/0089/S/5	VCL Chyme Diagnostic Centre	Laboratory	59 Ijoko Rd. Sango Ota	Ado Odo-Ota	Ogun
5769	OG/0111	OG/0111/S/4	Jesus is Love & Power Hospital	Pharmacy, Laboratory	St. Mary''s Catholic Church Area Alaja, Ifo	Ifo	Ogun
5770	OG/0111	OG/0111/S/5	Jesus is Love & Power Hospital	Pharmacy, Laboratory	St. Mary''s Catholic Church Area Alaja, Ifo	Ifo	Ogun
5771	OG/0112	OG/0112/S/14	Medytop Specialist Clinic		3 Ejimo Alebiosu Str, Isale Ijagba Sagamu	Sagamu	Ogun
5772	OG/0112	OG/0112/S/4	Medytop Specialist Clinic		3 Ejimo Alebiosu Str, Isale Ijagba Sagamu	Sagamu	Ogun
5773	OG/0112	OG/0112/S/1	Medytop Specialist Clinic		3 Ejimo Alebiosu Str, Isale Ijagba Sagamu	Sagamu	Ogun
5774	OG/0112	OG/0112/S/6	Medytop Specialist Clinic		3 Ejimo Alebiosu Str, Isale Ijagba Sagamu	Sagamu	Ogun
5775	OG/0112	OG/0112/S/5	Medytop Specialist Clinic		3 Ejimo Alebiosu Str, Isale Ijagba Sagamu	Sagamu	Ogun
5776	OG/0113	OG/0113/S/3	New Day Specialist Hospital		7 Sode Street, Akarigbo Sabo Sagamu, Ogun State	Sagamu	Ogun
5777	OG/0113	OG/0113/S/1	New Day Specialist Hospital		7 Sode Street, Akarigbo Sabo Sagamu, Ogun State	Sagamu	Ogun
5778	OG/0113	OG/0113/S/2	New Day Specialist Hospital		7 Sode Street, Akarigbo Sabo Sagamu, Ogun State	Sagamu	Ogun
5779	OG/0115	OG/0115/S/1	Ore-Ofe Clinic		88A Ejirin Rd Ijebu-Ode, Ogun State	Ijebu Ode	Ogun
5780	OG/0115	OG/0115/S/3	Ore-Ofe Clinic		88A Ejirin Rd Ijebu-Ode, Ogun State	Ijebu Ode	Ogun
5781	OG/0116	OG/0116/S/2	Sacred Heart Hospital		Lantoro Abeokuta, Ogun State	Abeokuta South	Ogun
5782	OG/0116	OG/0116/S/1	Sacred Heart Hospital		Lantoro Abeokuta, Ogun State	Abeokuta South	Ogun
5783	OG/0116	OG/0116/S/6	Sacred Heart Hospital		Lantoro Abeokuta, Ogun State	Abeokuta South	Ogun
5784	OG/0116	OG/0116/S/7	Sacred Heart Hospital		Lantoro Abeokuta, Ogun State	Abeokuta South	Ogun
5785	OG/0116	OG/0116/S/4	Sacred Heart Hospital		Lantoro Abeokuta, Ogun State	Abeokuta South	Ogun
5786	OG/0116	OG/0116/S/5	Sacred Heart Hospital		Lantoro Abeokuta, Ogun State	Abeokuta South	Ogun
5787	OG/0118	OG/0118/S/5	first medical diag. services	Laboratory	24a, sokenu road, oke-ijeun,abeokuta	Abeokuta South	Ogun
5788	OG/0120	OG/0120/S/5	JAT DIAGNOSTIC CENTER	Laboratory	136 AKARIGBO STREET, SAGAMU	Sagamu	Ogun
5789	OG/0129	OG/0129/S/1	Hossana Specialist Hospital		Oja Odan Road, Ilaro	Egbado South	Ogun
5790	OG/0130	OG/0130/S/8	Access Oral Healthcare Services Dental Clinic		1 Ogo Oluwa Bankole Crescent, Ibara Housing Estate, Abeokuta	Abeokuta South	Ogun
5791	OG/0131	OG/0131/S/1	Korede Hospital Ltd		17 Onikolobo Road, Ibara, Abeokuta	Abeokuta South	Ogun
5792	OG/0131	OG/0131/S/3	Korede Hospital Ltd		17 Onikolobo Road, Ibara, Abeokuta	Abeokuta South	Ogun
5793	OG/0133	OG/0133/S/5	Joseph Specialist Hospital		5, Surulere Street off Lalubu Street, Oke Ilewo, Abeokuta	Abeokuta South	Ogun
5794	OG/0133	OG/0133/S/10	Joseph Specialist Hospital		5, Surulere Street off Lalubu Street, Oke Ilewo, Abeokuta	Abeokuta South	Ogun

5795	OG/0133	OG/0133/S/7	Joseph Specialist Hospital		5, Surulere Street off Lalubu Street, Oke Ilewo, Abeokuta	Abeokuta South	Ogun
5796	OG/0136	OG/0136/S/3	Overcomers Specialist Hospital		1 New Koregun Street, Behind Mouka Foam, Town Planning Area, Ilishan	Ikenne	Ogun
5797	OG/0138	OG/0138/S/4	Blooms Pharmacy & Stores	Pharmacy	56 Bonajo Street, Ijebu Ode	Ijebu Ode	Ogun
5798	OG/0139	OG/0139/S/4	Toun Chemist & Variety Stores	Phamarcy	94 Folagbade Street, Ijebu Ode	Ijebu Ode	Ogun
5799	OG/0142	OG/0142/S/5	Ore Ofe Elshaddai Clinic		6 Isope Road off Ejirin Road, Texaco Junction, Ijebu Ode	Ijebu Ode	Ogun
5800	OG/0144	OG/0144/S/3	Olabisi Onabanjo University Teaching Hospital Shagamu		Shagamu, Ogun State	Sagamu	Ogun
5801	OG/0144	OG/0144/S/4	Olabisi Onabanjo University Teaching Hospital Shagamu		Shagamu, Ogun State	Sagamu	Ogun
5802	OG/0144	OG/0144/S/15	Olabisi Onabanjo University Teaching Hospital Shagamu		Shagamu, Ogun State	Sagamu	Ogun
5803	OG/0144	OG/0144/S/7	Olabisi Onabanjo University Teaching Hospital Shagamu		Shagamu, Ogun State	Sagamu	Ogun
5804	OG/0144	OG/0144/S/5	Olabisi Onabanjo University Teaching Hospital Shagamu		Shagamu, Ogun State	Sagamu	Ogun
5805	OG/0144	OG/0144/S/2	Olabisi Onabanjo University Teaching Hospital Shagamu		Shagamu, Ogun State	Sagamu	Ogun
5806	OG/0144	OG/0144/S/10	Olabisi Onabanjo University Teaching Hospital Shagamu		Shagamu, Ogun State	Sagamu	Ogun
5807	OG/0144	OG/0144/S/1	Olabisi Onabanjo University Teaching Hospital Shagamu		Shagamu, Ogun State	Sagamu	Ogun
5808	OG/0144	OG/0144/S/6	Olabisi Onabanjo University Teaching Hospital Shagamu		Shagamu, Ogun State	Sagamu	Ogun
5809	OG/0149	OG/0149/S/3	Adeyemi Medical Centre		4 Muri Abiola Street, Agbado Station	Ifo	Ogun
5810	OG/0152	OG/0152/S/3	Lafia Kowa Specialist Hospital		12 Ejirin Road, Ijebu Ode	Ijebu Ode	Ogun
5811	OG/0152	OG/0152/S/1	Lafia Kowa Specialist Hospital		12 Ejirin Road, Ijebu Ode	Ijebu Ode	Ogun
5812	OG/0156	OG/0156/S/3	Olu-Ola Specialist Hospital		12B Odo-Egbo Street, Ijebu Ode	Ijebu Ode	Ogun
5813	OG/0156	OG/0156/S/1	Olu-Ola Specialist Hospital		12B Odo-Egbo Street, Ijebu Ode	Ijebu Ode	Ogun
5814	OG/0157	OG/0157/S/5	Apex Medical Diagnostic	Laboratory	8, Ayoola Shopping Complex	Abeokuta South	Ogun
5815	OG/0158	OG/0158/S/1	Akobi Specialist Hospital		Plot 5, Blk vw, Molipa Community layout, Ijebu-Ode	Ijebu Ode	Ogun
5816	OG/0158	OG/0158/S/3	Akobi Specialist Hospital		Plot 5, Blk vw, Molipa Community layout, Ijebu-Ode	Ijebu Ode	Ogun
5817	OG/0170	OG/0170/S/3	Healing Spring Medical Centre		23 Ilishan Road, Iperu	Ijebu Ode	Ogun
5818	OG/0173	OG/0173/S/8	Victory Dental Clinic	Dental	46 Surulere Street, Cele B/Stop, Akute	Ifo	Ogun
5819	OG/0174	OG/0174/S/4	Amazing Grace Hospital	Laboratory, Pharmacy	7 Iloro Street, Off New Road, Ijebu-Ode	Ijebu Ode	Ogun
5820	OG/0174	OG/0174/S/5	Amazing Grace Hospital	Laboratory, Pharmacy	7 Iloro Street, Off New Road, Ijebu-Ode	Ijebu Ode	Ogun
5821	OG/0176	OG/0176/S/3	First Alpha Medical Centre		Kuforiji Olubi Drive, Adigbe, Abeokuta	Abeokuta South	Ogun
5822	OG/0176	OG/0176/S/14	First Alpha Medical Centre		Kuforiji Olubi Drive, Adigbe, Abeokuta	Abeokuta South	Ogun
5823	OG/0176	OG/0176/S/1	First Alpha Medical Centre		Kuforiji Olubi Drive, Adigbe, Abeokuta	Abeokuta South	Ogun
5824	OG/0181	OG/0181/S/3	Beachland Specialist Hospital		Plot 10 Alfirdaus Estate, Arepo, Off Lagos-Abeokuta Express Way	Obafemi/Owode	Ogun
5825	OG/0185	OG/0185/S/3	Surgicare Consult		Psalm 23, Oba Oluwa Road, GRA Housing Estate, Ibara, Abeokuta	Abeokuta South	Ogun
5826	OG/0185	OG/0185/S/1	Surgicare Consult		Psalm 23, Oba Oluwa Road, GRA Housing Estate, Ibara, Abeokuta	Abeokuta South	Ogun
5827	OG/0185	OG/0185/S/5	Surgicare Consult		Psalm 23, Oba Oluwa Road, GRA Housing Estate, Ibara, Abeokuta	Abeokuta South	Ogun
5828	OG/0188	OG/0188/S/3	Babcock University Medical Centre		Ilisan Remo	Ikenne	Ogun
5829	OG/0188	OG/0188/S/5	Babcock University Medical Centre		Ilisan Remo	Ikenne	Ogun

5830	OG/0188	OG/0188/S/4	Babcock University Medical Centre		Ilisan Remo	Ikenne	Ogun
5831	OG/0189	OG/0189/S/3	Otunba Tunwase National Paediatric Centre	Primary, O & G, Paediatrics, Pharmacy, Laboratory	Shagamu-Benin Express Way, Ijebu-Ode	Odogbolu	Ogun
5832	OG/0189	OG/0189/S/6	Otunba Tunwase National Paediatric Centre	Primary, O & G, Paediatrics, Pharmacy, Laboratory	Shagamu-Benin Express Way, Ijebu-Ode	Odogbolu	Ogun
5833	OG/0189	OG/0189/S/5	Otunba Tunwase National Paediatric Centre	Primary, O & G, Paediatrics, Pharmacy, Laboratory	Shagamu-Benin Express Way, Ijebu-Ode	Odogbolu	Ogun
5834	OG/0189	OG/0189/S/4	Otunba Tunwase National Paediatric Centre	Primary, O & G, Paediatrics, Pharmacy, Laboratory	Shagamu-Benin Express Way, Ijebu-Ode	Odogbolu	Ogun
5835	OG/0192	OG/0192/S/5	Shirish Clinic		102 Joju Road Otta	Ado Odo-Ota	Ogun
5836	OG/0199	OG/0199/S/3	Goodluck Hopsital & Maternity Home Ltd		3, The Light Church Close, Mercy Estate, Asokore-Akute	Ifo	Ogun
5837	OG/0201	OG/0201/S/3	First Contact Hospital and Maternity		33B, Kuto Road, Behind Yemi-Ola Executive Stores, Abeokuta, Ogun State	Abeokuta South	Ogun
5838	OG/0206	OG/0206/S/3	Brims Hospital		4, Muraina Soyode Street, behind Olub filling station, Ayetoro road Abeokuta, Ogun State	Abeokuta North	Ogun
5839	OG/0209	OG/0209/S/4	State Hospital Ota, Ogun State		Ota, Ogun State	Ado Odo-Ota	Ogun
5840	OG/0209	OG/0209/S/1	State Hospital Ota, Ogun State		Ota, Ogun State	Ado Odo-Ota	Ogun
5841	OG/0209	OG/0209/S/7	State Hospital Ota, Ogun State		Ota, Ogun State	Ado Odo-Ota	Ogun
5842	OG/0209	OG/0209/S/5	State Hospital Ota, Ogun State		Ota, Ogun State	Ado Odo-Ota	Ogun
5843	OG/0209	OG/0209/S/8	State Hospital Ota, Ogun State		Ota, Ogun State	Ado Odo-Ota	Ogun
5844	OG/0209	OG/0209/S/3	State Hospital Ota, Ogun State		Ota, Ogun State	Ado Odo-Ota	Ogun
5845	OG/0209	OG/0209/S/15	State Hospital Ota, Ogun State		Ota, Ogun State	Ado Odo-Ota	Ogun
5846	OG/0213	OG/0213/S/3	Twins Specialist Clinic		Plot 11, Gunwa Ola Street GRA Extension, Sagamu, Ogun State	Sagamu	Ogun
5847	OG/0214	OG/0214/S/9	Ishwot Consulting Hospital		1 Janet Jibowu Street, Ita-Oshin Behind Old Nepa Office Aro Ogun State.	Abeokuta North	Ogun
5848	OG/0219	OG/0219/S/3	Iyaniwura Specilaist Hospital		I Olujobi Falana close, Behind AP filling station, Mowe town, Sagamu, Ogun state	Sagamu	Ogun
5849	OG/0225	OG/0225/S/5	Ademuyiwa Med. Diag.& Scan Centre		Flat2 Africa Church Cathedral Estate, Querry Rd. ,	Abeokuta North	Ogun
5850	OG/0230	OG/0230/S/4	Micoson Pharmaceuticals		1, Ijoko Road,Sango-Ota Ogun State	Ado Odo-Ota	Ogun
5851	OG/0231	OG/0231/S/13	Catholic Optical Outreaches		28,Awokoya Street Ijebu-Ode Ogun State	Ijebu Ode	Ogun
5852	OG/0232	OG/0232/S/1	Mike Medics Hospital		Aduba Estate, Akinbo Off Ijoko Road ,Akute Ogunn State	Ifo	Ogun
5853	OG/0233	OG/0233/S/3	St. Michael-Israel Specialist Hospital LTD		50,Awujale Street, Itale, Ijebu-Ode Ogun State	Ijebu Ode	Ogun
5854	OG/0233	OG/0233/S/1	St. Michael-Israel Specialist Hospital LTD		50,Awujale Street, Itale, Ijebu-Ode Ogun State	Ijebu Ode	Ogun
5855	OG/0235	OG/0235/S/15	Elohim Eye Specialist		20,Abiola Way, Abeokuta Ogun State	Abeokuta South	Ogun
5856	OG/0236	OG/0236/S/1	Pillars Specialist Clinic		136,Akarigbo Road,Ijoku, Opp.First Bank Plc	Sagamu	Ogun
5857	OG/0236	OG/0236/S/3	Pillars Specialist Clinic		136,Akarigbo Road,Ijoku, Opp.First Bank Plc	Sagamu	Ogun
5858	OG/0237	OG/0237/S/1	Mcpherson University Health Centre		Km 96, Lagos-Ibadan Expressway,Senki-Sotayo, Village Ogun State	Obafemi/Owode	Ogun
5859	OG/0240	OG/0240/S/1	Ojugbele Specialist Hospital		105,Idiroko Road, Oju-Ore Ogun State	Ado Odo-Ota	Ogun
5860	OG/0241	OG/0241/S/4	State Hospital Isara-Remo		Oke-Oja, Isara-Remo,Ogun State	Remo North	Ogun
5861	OG/0241	OG/0241/S/3	State Hospital Isara-Remo		Oke-Oja, Isara-Remo,Ogun State	Remo North	Ogun
5862	OG/0241	OG/0241/S/5	State Hospital Isara-Remo		Oke-Oja, Isara-Remo,Ogun State	Remo North	Ogun

5863	OG/0243	OG/0243/S/10	Faith Specialist Hospital		Plot 5,block 11,Ogun State Housing Corporation, Onipanu Quarters Ogun State	Ifo	Ogun
5864	OG/0244	OG/0244/S/3	Jon-Fem Hospital LTD and Maternity Home		63, Akeja Street, Off Anglican Road, Ilo Ifako Sango-Ota, Ogun State	Ado Odo-Ota	Ogun
5865	OG/0244	OG/0244/S/10	Jon-Fem Hospital LTD and Maternity Home		63, Akeja Street, Off Anglican Road, Ilo Ifako Sango-Ota, Ogun State	Ado Odo-Ota	Ogun
5866	OG/0246	OG/0246/S/1	Angels & Eagle Specialist Hospital		9b Sikiru Jimoh Street, Beside Wemby Suites, GRA, Ibara, Abeokuta Ogun State	Abeokuta South	Ogun
5867	OG/0246	OG/0246/S/6	Angels & Eagle Specialist Hospital		9b Sikiru Jimoh Street, Beside Wemby Suites, GRA, Ibara, Abeokuta Ogun State	Abeokuta South	Ogun
5868	OG/0246	OG/0246/S/4	Angels & Eagle Specialist Hospital		9b Sikiru Jimoh Street, Beside Wemby Suites, GRA, Ibara, Abeokuta Ogun State	Abeokuta South	Ogun
5869	OG/0246	OG/0246/S/3	Angels & Eagle Specialist Hospital		9b Sikiru Jimoh Street, Beside Wemby Suites, GRA, Ibara, Abeokuta Ogun State	Abeokuta South	Ogun
5870	OG/0246	OG/0246/S/11	Angels & Eagle Specialist Hospital		9b Sikiru Jimoh Street, Beside Wemby Suites, GRA, Ibara, Abeokuta Ogun State	Abeokuta South	Ogun
5871	OG/0246	OG/0246/S/5	Angels & Eagle Specialist Hospital		9b Sikiru Jimoh Street, Beside Wemby Suites, GRA, Ibara, Abeokuta Ogun State	Abeokuta South	Ogun
5872	OG/0249	OG/0249/S/7	TBK Rainbow Medical Diagnostic Ltd		OGS Plaza, 30 Akure Road, Ajuwon Bus Stop, Ajuwon Ogun State	Ifo	Ogun
5873	OG/0249	OG/0249/S/5	TBK Rainbow Medical Diagnostic Ltd		OGS Plaza, 30 Akure Road, Ajuwon Bus Stop, Ajuwon Ogun State	Ifo	Ogun
5874	OG/0252	OG/0252/S/3	The Unique Great Shepherd Specialist Hospital		1,Olujide Somolu Rd., Opposite Fire Service HQRS., Onikoko, Abeokuta, Ogun State	Obafemi/Owode	Ogun
5875	OG/0258	OG/0258/S/2	O & A Medical Centre		3, Asero Housing Estate,Asero Abeokuta. Ogun State	Abeokuta South	Ogun
5876	OG/0258	OG/0258/S/3	O & A Medical Centre		3, Asero Housing Estate,Asero Abeokuta. Ogun State	Abeokuta South	Ogun
5877	OG/0263	OG/0263/S/1	Bliss Hospital		127,Akarigbo Street,Ijoku, Sagamu,Ogun State	Sagamu	Ogun
5878	OG/0263	OG/0263/S/3	Bliss Hospital		127,Akarigbo Street,Ijoku, Sagamu,Ogun State	Sagamu	Ogun
5879	OS/0002	OS/0002/S/4	Samtad Pharmacy	Pharmacy	Kutupe-basse, Sabo Quarters, Ikire	Ayedaade	Osun
5880	OS/0011	OS/0011/S/4	Kike Lomo Pharmacy	Pharmacy	Ilesha	Ilesa East	Osun
5881	OS/0012	OS/0012/S/4	Fola Saveway Pharmacy	Pharmacy	Bi 91, Okesa Street, Opp. Ilesa Grammar Sch., Ilesha	Ilesa East	Osun
5882	OS/0013	OS/0013/S/4	Ibukun Oluwa Chemists	Pharmacy	A, 50, Sale Aje Street Ipetu modu	Ife South	Osun
5883	OS/0014	OS/0014/S/3	Klm Muslim Hospital		Feesu Road, Iwo	Iwo	Osun
5884	OS/0014	OS/0014/S/1	Klm Muslim Hospital		Feesu Road, Iwo	Iwo	Osun
5885	OS/0018	OS/0018/S/3	Biket Medical Centre		Off Kuyi Square, Osogbo-Ikirun Road	Olorunda	Osun
5886	OS/0018	OS/0018/S/14	Biket Medical Centre		Off Kuyi Square, Osogbo-Ikirun Road	Olorunda	Osun
5887	OS/0018	OS/0018/S/7	Biket Medical Centre		Off Kuyi Square, Osogbo-Ikirun Road	Olorunda	Osun
5888	OS/0018	OS/0018/S/2	Biket Medical Centre		Off Kuyi Square, Osogbo-Ikirun Road	Olorunda	Osun
5889	OS/0018	OS/0018/S/1	Biket Medical Centre		Off Kuyi Square, Osogbo-Ikirun Road	Olorunda	Osun
5890	OS/0018	OS/0018/S/5	Biket Medical Centre		Off Kuyi Square, Osogbo-Ikirun Road	Olorunda	Osun
5891	OS/0021	OS/0021/S/1	Ladoke Akintola Teaching Hospital		Osogbo	Osogbo	Osun
5892	OS/0021	OS/0021/S/11	Ladoke Akintola Teaching Hospital		Osogbo	Osogbo	Osun
5893	OS/0021	OS/0021/S/6	Ladoke Akintola Teaching Hospital		Osogbo	Osogbo	Osun
5894	OS/0021	OS/0021/S/3	Ladoke Akintola Teaching Hospital		Osogbo	Osogbo	Osun
5895	OS/0021	OS/0021/S/2	Ladoke Akintola Teaching Hospital		Osogbo	Osogbo	Osun
5896	OS/0021	OS/0021/S/10	Ladoke Akintola Teaching Hospital		Osogbo	Osogbo	Osun

5897	OS/0022	OS/0022/S/1	Abake Medical Centre		Odiolowo, Osogbo	Osogbo	Osun
5898	OS/0030	OS/0030/S/4	Ife Olu Chemist & Supermarket	Pharmacy	20 Alekuwodu Rd., Okefia Osogbo	Osogbo	Osun
5899	OS/0040	OS/0040/S/2	Obafemi Awolowo Teaching Hospital		Ile-Ife/Ilesha	Ife Central	Osun
5900	OS/0040	OS/0040/S/6	Obafemi Awolowo Teaching Hospital		Ile-Ife/Ilesha	Ife Central	Osun
5901	OS/0040	OS/0040/S/10	Obafemi Awolowo Teaching Hospital		Ile-Ife/Ilesha	Ife Central	Osun
5902	OS/0040	OS/0040/S/3	Obafemi Awolowo Teaching Hospital		Ile-Ife/Ilesha	Ife Central	Osun
5903	OS/0040	OS/0040/S/1	Obafemi Awolowo Teaching Hospital		Ile-Ife/Ilesha	Ife Central	Osun
5904	OS/0041	OS/0041/S/8	Adenle Memorial Hospital		Ilesha	Ilesa East	Osun
5905	OS/0042	OS/0042/S/4	Adetola Pharmacy Ltd.	Pharmacy	No.7, Alafia Str. Off Aiye Toro Str., Osohbo	Olorunda	Osun
5906	OS/0050	OS/0050/S/19	Obafemi Awolowo University Health Centre		Ile-Ife, Osun State	Ife Central	Osun
5907	OS/0050	OS/0050/S/6	Obafemi Awolowo University Health Centre		Ile-Ife, Osun State	Ife Central	Osun
5908	OS/0050	OS/0050/S/2	Obafemi Awolowo University Health Centre		Ile-Ife, Osun State	Ife Central	Osun
5909	OS/0050	OS/0050/S/10	Obafemi Awolowo University Health Centre		Ile-Ife, Osun State	Ife Central	Osun
5910	OS/0050	OS/0050/S/5	Obafemi Awolowo University Health Centre		Ile-Ife, Osun State	Ife Central	Osun
5911	OS/0050	OS/0050/S/3	Obafemi Awolowo University Health Centre		Ile-Ife, Osun State	Ife Central	Osun
5912	OS/0050	OS/0050/S/4	Obafemi Awolowo University Health Centre		Ile-Ife, Osun State	Ife Central	Osun
5913	OS/0050	OS/0050/S/1	Obafemi Awolowo University Health Centre		Ile-Ife, Osun State	Ife Central	Osun
5914	OS/0053	OS/0053/S/15	Onward Specialist Hospital		Agunbelewo Ilobu Road, Oshogbo	Olorunda	Osun
5915	OS/0053	OS/0053/S/1	Onward Specialist Hospital		Agunbelewo Ilobu Road, Oshogbo	Olorunda	Osun
5916	OS/0053	OS/0053/S/5	Onward Specialist Hospital		Agunbelewo Ilobu Road, Oshogbo	Olorunda	Osun
5917	OS/0053	OS/0053/S/4	Onward Specialist Hospital		Agunbelewo Ilobu Road, Oshogbo	Olorunda	Osun
5918	OS/0053	OS/0053/S/7	Onward Specialist Hospital		Agunbelewo Ilobu Road, Oshogbo	Olorunda	Osun
5919	OS/0053	OS/0053/S/3	Onward Specialist Hospital		Agunbelewo Ilobu Road, Oshogbo	Olorunda	Osun
5920	OS/0058	OS/0058/S/3	Police Clinics, Osun		Osun State	Ede South	Osun
5921	OS/0058	OS/0058/S/1	Police Clinics, Osun		Osun State	Ede South	Osun
5922	OS/0058	OS/0058/S/2	Police Clinics, Osun		Osun State	Ede South	Osun
5923	OS/0061	OS/0061/S/1	Seventh Day Adventist Hospital		LAGERE RD,ILE-IFE	Ife Central	Osun
5924	OS/0061	OS/0061/S/7	Seventh Day Adventist Hospital		LAGERE RD,ILE-IFE	Ife Central	Osun
5925	OS/0061	OS/0061/S/2	Seventh Day Adventist Hospital		LAGERE RD,ILE-IFE	Ife Central	Osun
5926	OS/0061	OS/0061/S/6	Seventh Day Adventist Hospital		LAGERE RD,ILE-IFE	Ife Central	Osun
5927	OS/0061	OS/0061/S/10	Seventh Day Adventist Hospital		LAGERE RD,ILE-IFE	Ife Central	Osun
5928	OS/0061	OS/0061/S/12	Seventh Day Adventist Hospital		LAGERE RD,ILE-IFE	Ife Central	Osun
5929	OS/0061	OS/0061/S/11	Seventh Day Adventist Hospital		LAGERE RD,ILE-IFE	Ife Central	Osun
5930	OS/0061	OS/0061/S/5	Seventh Day Adventist Hospital		LAGERE RD,ILE-IFE	Ife Central	Osun
5931	OS/0061	OS/0061/S/4	Seventh Day Adventist Hospital		LAGERE RD,ILE-IFE	Ife Central	Osun
5932	OS/0061	OS/0061/S/3	Seventh Day Adventist Hospital		LAGERE RD,ILE-IFE	Ife Central	Osun
5933	OS/0062	OS/0062/S/2	Our Lady of Fatima Catholic Hospital (Jaleyemi)		ODI-OLOWO,OSOGBO	Osogbo	Osun
5934	OS/0062	OS/0062/S/14	Our Lady of Fatima Catholic Hospital (Jaleyemi)		ODI-OLOWO,OSOGBO	Osogbo	Osun
5935	OS/0062	OS/0062/S/4	Our Lady of Fatima Catholic Hospital (Jaleyemi)		ODI-OLOWO,OSOGBO	Osogbo	Osun
5936	OS/0062	OS/0062/S/5	Our Lady of Fatima Catholic Hospital (Jaleyemi)		ODI-OLOWO,OSOGBO	Osogbo	Osun

5937	OS/0062	OS/0062/S/7	Our Lady of Fatima Catholic Hospital (Jaleyemi)		ODI-OLOWO,OSOGBO	Osogbo	Osun
5938	OS/0062	OS/0062/S/3	Our Lady of Fatima Catholic Hospital (Jaleyemi)		ODI-OLOWO,OSOGBO	Osogbo	Osun
5939	OS/0066	OS/0066/S/1	Ronbay Hospital & Maternity		TALAFIA IMAM STR, BEHIND FED. POLY EDE	Ede South	Osun
5940	OS/0067	OS/0067/S/1	Life Fountain Specialist Hospital		6 OLAFARE BIOBAKU STR. PARAKIN	Ife Central	Osun
5941	OS/0068	OS/0068/S/1	St. Victoria Specialist Hospital		OPP NTA MOKURO RD, ILE-IFE	Ife Central	Osun
5942	OS/0070	OS/0070/S/1	EBENEZER SPECIALIST HOSP.		17 STATION RD, OSOGBO	Osogbo	Osun
5943	OS/0071	OS/0071/S/1	BALM OF GILEAD SPEC. HOSPITAL		OKE-OPO GRA ILESHA	Ilesa East	Osun
5944	OS/0072	OS/0072/S/5	St. Jude's Hospital		OFF FATIMA COLLEGE RD, LAAKOSIN IKIRE	Irewole	Osun
5945	OS/0072	OS/0072/S/4	St. Jude's Hospital		OFF FATIMA COLLEGE RD, LAAKOSIN IKIRE	Irewole	Osun
5946	OS/0072	OS/0072/S/3	St. Jude's Hospital		OFF FATIMA COLLEGE RD, LAAKOSIN IKIRE	Irewole	Osun
5947	OS/0072	OS/0072/S/7	St. Jude's Hospital		OFF FATIMA COLLEGE RD, LAAKOSIN IKIRE	Irewole	Osun
5948	OS/0072	OS/0072/S/1	St. Jude's Hospital		OFF FATIMA COLLEGE RD, LAAKOSIN IKIRE	Irewole	Osun
5949	OS/0077	OS/0077/S/4	JOSTAD CHEMISTS	Pharmacy	A2, EREJA SQUARE, RISAWE''S COMPOUND, ILESA	Ilesa West	Osun
5950	OS/0082	OS/0082/S/5	OROKI HOSPITAL	Radiology	27 CEMENTRY RD, IGBONNA OSOGBO	Olorunda	Osun
5951	OS/0082	OS/0082/S	OROKI HOSPITAL	Radiology	27 CEMENTRY RD, IGBONNA OSOGBO	Olorunda	Osun
5952	OS/0088	OS/0088/S/1	Our Lady of Lourdes Catholic Hospital & Maternity		Parde Road, Ipetumodu	Ife North	Osun
5953	OS/0088	OS/0088/S/15	Our Lady of Lourdes Catholic Hospital & Maternity		Parde Road, Ipetumodu	Ife North	Osun
5954	OS/0088	OS/0088/S/3	Our Lady of Lourdes Catholic Hospital & Maternity		Parde Road, Ipetumodu	Ife North	Osun
5955	OS/0088	OS/0088/S/5	Our Lady of Lourdes Catholic Hospital & Maternity		Parde Road, Ipetumodu	Ife North	Osun
5956	OS/0088	OS/0088/S/4	Our Lady of Lourdes Catholic Hospital & Maternity		Parde Road, Ipetumodu	Ife North	Osun
5957	OS/0088	OS/0088/S/14	Our Lady of Lourdes Catholic Hospital & Maternity		Parde Road, Ipetumodu	Ife North	Osun
5958	OS/0090	OS/0090/S/3	Victory Hospital	O&G	1 Olumodan Deubi Camp, Iyalode Street off Feasu Road Iwo	Iwo	Osun
5959	OS/0091	OS/0091/S/1	Morning Star Medical Centre		10, Oyetunde Street, Alekuwodo, Osogbo	Osogbo	Osun
5960	OS/0091	OS/0091/S/3	Morning Star Medical Centre		10, Oyetunde Street, Alekuwodo, Osogbo	Osogbo	Osun
5961	OS/0092	OS/0092/S/3	Apex Medical Centre		133 Ibadan Road, Ile Ife	Ife Central	Osun
5962	OS/0092	OS/0092/S/1	Apex Medical Centre		133 Ibadan Road, Ile Ife	Ife Central	Osun
5963	OS/0094	OS/0094/S/2	Oke-Ola Catholic Hospital	Internal Medicine, O & G, General Surgery	Oke-Ola Apomu, Isokan District	Isokan	Osun
5964	OS/0094	OS/0094/S/1	Oke-Ola Catholic Hospital	Internal Medicine, O & G, General Surgery	Oke-Ola Apomu, Isokan District	Isokan	Osun
5965	OS/0094	OS/0094/S/3	Oke-Ola Catholic Hospital	Internal Medicine, O & G, General Surgery	Oke-Ola Apomu, Isokan District	Isokan	Osun
5966	OS/0098	OS/0098/S/1	Demal Clinic		# 30, Kola Adetunji Street, Oke Ijetu Ilesa Garage Area, Oshogbo	Osogbo	Osun
5967	OS/0098	OS/0098/S/3	Demal Clinic		# 30, Kola Adetunji Street, Oke Ijetu Ilesa Garage Area, Oshogbo	Osogbo	Osun
5968	OS/0099	OS/0099/S/3	Living Hope Hospital		Parakin obalufe Layout, Osun state	Ife Central	Osun
5969	OS/0100	OS/0100/S/3	Anu-Olu Hospital and Maternity		1, Alh Wahab Salami Str, Agip Area, Ede	Ede South	Osun
5970	OS/0100	OS/0100/S/1	Anu-Olu Hospital and Maternity		1, Alh Wahab Salami Str, Agip Area, Ede	Ede South	Osun
5971	OS/0103	OS/0103/S/3	Spring Hope Specialist Hospital		Spring House, Old Ikirun Road, Oshogbo, Osun State	Olorunda	Osun

5972	OS/0106	OS/0106/S/3	Emmanuel Medical Centre		8 Deborah Oladogun Street Oshogbo	Osogbo	Osun
5973	OS/0106	OS/0106/S/1	Emmanuel Medical Centre		8 Deborah Oladogun Street Oshogbo	Osogbo	Osun
5974	OS/0106	OS/0106/S/2	Emmanuel Medical Centre		8 Deborah Oladogun Street Oshogbo	Osogbo	Osun
5975	OS/0106	OS/0106/S/6	Emmanuel Medical Centre		8 Deborah Oladogun Street Oshogbo	Osogbo	Osun
5976	OS/0107	OS/0107/S/3	Ayomide Women's Health Specialist Hospital		30 Powerline, Ikirun Road, Osogbo, Osun State.	Osogbo	Osun
5977	OS/0108	OS/0108/S/3	Hamstring Specialist Hospital &Diagnostic Centre		Isiah Okunola Street, off Okinni Grammer School, Okinni, Oshogbo	Egbedore	Osun
5978	OS/0110	OS/0110/S/3	Victory Point Hospital		Ojoda New Town, Ile-idande, Osogbo, Osun State.	Olorunda	Osun
5979	OS/0111	OS/0111/S/3	Ultimate Specialist Hospital		No. 179 Fajuyi Road, Ile- Ife Osun State.	Ife Central	Osun
5980	OS/0113	OS/0113/S/1	Living Hope Medical Centre		No. 26 Fola Aliu Street, Boripe Community, Rinsayo Area, Osogbo Osun State.	Egbedore	Osun
5981	OS/0113	OS/0113/S/2	Living Hope Medical Centre		No. 26 Fola Aliu Street, Boripe Community, Rinsayo Area, Osogbo Osun State.	Egbedore	Osun
5982	OS/0113	OS/0113/S/3	Living Hope Medical Centre		No. 26 Fola Aliu Street, Boripe Community, Rinsayo Area, Osogbo Osun State.	Egbedore	Osun
5983	OS/0118	OS/0118/S/3	Dunafort Hospital & Laboratory Services		Tinumola Area, Osogbo Osun State	Osogbo	Osun
5984	OS/0118	OS/0118/S/1	Dunafort Hospital & Laboratory Services		Tinumola Area, Osogbo Osun State	Osogbo	Osun
5985	OS/0119	OS/0119/S/5	Goodness & Mercy Specialist Hospital		Christ life Centre, Ofatedo, Osogbo Osun State.	Egbedore	Osun
5986	OS/0119	OS/0119/S/1	Goodness & Mercy Specialist Hospital		Christ life Centre, Ofatedo, Osogbo Osun State.	Egbedore	Osun
5987	OS/0119	OS/0119/S/3	Goodness & Mercy Specialist Hospital		Christ life Centre, Ofatedo, Osogbo Osun State.	Egbedore	Osun
5988	OS/0121	OS/0121/S/12	Emmanuel Specialist’s Hospital		E27 Obayemi Street, Agborogboro Layout, Oranfe, Ilesha Road, Ile Ife Osun State.	Ife East	Osun
5989	OS/0121	OS/0121/S/3	Emmanuel Specialist’s Hospital		E27 Obayemi Street, Agborogboro Layout, Oranfe, Ilesha Road, Ile Ife Osun State.	Ife East	Osun
5990	OS/0121	OS/0121/S/1	Emmanuel Specialist’s Hospital		E27 Obayemi Street, Agborogboro Layout, Oranfe, Ilesha Road, Ile Ife Osun State.	Ife East	Osun
5991	OS/0121	OS/0121/S/18	Emmanuel Specialist’s Hospital		E27 Obayemi Street, Agborogboro Layout, Oranfe, Ilesha Road, Ile Ife Osun State.	Ife East	Osun
5992	OS/0125	OS/0125/S/15	Jola-Olu Specialist Hospital		Agunbelewo Ilobu Road, P.O. Box 979, Osogbo Osun State.	Egbedore	Osun
5993	OS/0127	OS/0127/S/8	Flourish Dental Clinic		5, Olanire Crescent, Behind Bovas Petrol Station Osogbo Osun State	Osogbo	Osun
5994	OS/0128	OS/0128/S/8	Flourish Dental Clinic		5, Olanire Crescent, Behind Bovas Petrol Station Osogbo Osun State	Osogbo	Osun
5995	OS/0130	OS/0130/S/3	Alpha Hospital & Maternity Home		9 Adetoro Street, Near Moricaz Mosque, Ede Osun State	Ede South	Osun
5996	OS/0135	OS/0135/S/7	Mannabat Medical Centre		Opp. Old Tajudeen School, Off Sawmill Road, Ido-osun	Egbedore	Osun
5997	OS/0135	OS/0135/S/5	Mannabat Medical Centre		Opp. Old Tajudeen School, Off Sawmill Road, Ido-osun	Egbedore	Osun
5998	OS/0139	OS/0139/S/1	BWB Hospital		No.5 orisumbare Street, Kasmo Area, Oke-baale,Osogbo Osun State	Osogbo	Osun
5999	OS/0140	OS/0140/S/12	Rehoboth Ear, Nose and Throat Hospital		Tajudeen Suleiman Street, Zone3, Behind M-Oyetunji Filling Station, Capital	Osogbo	Osun
6000	OS/0141	OS/0141/S/3	Oke-baale Central Hospital		Opp. Okebaale Police Station, Okebaale, Osogbo Osun State	Osogbo	Osun

6001	OS/0142	OS/0142/S/3	Safe Parturition (SP) Specialist Hospital		6, Abiodun Adeshina Street, Behind GOF Schools, Osogbo Osun State	Osogbo	Osun
6002	OY/0001	OY/0001/S/7	University College Hospital		Ibadan	Ibadan North	Oyo
6003	OY/0001	OY/0001/S/11	University College Hospital		Ibadan	Ibadan North	Oyo
6004	OY/0001	OY/0001/S/15	University College Hospital		Ibadan	Ibadan North	Oyo
6005	OY/0001	OY/0001/S/5	University College Hospital		Ibadan	Ibadan North	Oyo
6006	OY/0001	OY/0001/S/4	University College Hospital		Ibadan	Ibadan North	Oyo
6007	OY/0003	OY/0003/S/2	Tobi Medical Centre Ltd.		57/453 Felele Layout, Ibadan	Ibadan North	Oyo
6008	OY/0003	OY/0003/S/5	Tobi Medical Centre Ltd.		57/453 Felele Layout, Ibadan	Ibadan North	Oyo
6009	OY/0003	OY/0003/S/9	Tobi Medical Centre Ltd.		57/453 Felele Layout, Ibadan	Ibadan North	Oyo
6010	OY/0003	OY/0003/S/6	Tobi Medical Centre Ltd.		57/453 Felele Layout, Ibadan	Ibadan North	Oyo
6011	OY/0003	OY/0003/S/3	Tobi Medical Centre Ltd.		57/453 Felele Layout, Ibadan	Ibadan North	Oyo
6012	OY/0005	OY/0005/S/1	Badejoko Memorial Hospital		Oremeji Mokola Opp. Premier, Hotel Junction, Ibadan	Ibadan North	Oyo
6013	OY/0005	OY/0005/S/5	Badejoko Memorial Hospital		Oremeji Mokola Opp. Premier, Hotel Junction, Ibadan	Ibadan North	Oyo
6014	OY/0005	OY/0005/S/2	Badejoko Memorial Hospital		Oremeji Mokola Opp. Premier, Hotel Junction, Ibadan	Ibadan North	Oyo
6015	OY/0005	OY/0005/S/3	Badejoko Memorial Hospital		Oremeji Mokola Opp. Premier, Hotel Junction, Ibadan	Ibadan North	Oyo
6016	OY/0009	OY/0009/S/1	Adeoye Memorial Specialist Hospital		Nw/ 4/185 George Street, Ibadan	Ibadan North	Oyo
6017	OY/0009	OY/0009/S/5	Adeoye Memorial Specialist Hospital		Nw/ 4/185 George Street, Ibadan	Ibadan North	Oyo
6018	OY/0010	OY/0010/S/1	Mobolaji Hospital		43, Obafemi Awolowo Way, Oke-Bola, Ibadan	Ibadan North	Oyo
6019	OY/0010	OY/0010/S/14	Mobolaji Hospital		43, Obafemi Awolowo Way, Oke-Bola, Ibadan	Ibadan North	Oyo
6020	OY/0010	OY/0010/S/3	Mobolaji Hospital		43, Obafemi Awolowo Way, Oke-Bola, Ibadan	Ibadan North	Oyo
6021	OY/0011	OY/0011/S/2	Idi-Ape Medical Centre		Plot 2 & 6, Afolabi Layout Idi-Ape, Ibadan	Ibadan North	Oyo
6022	OY/0012	OY/0012/S/1	Eyiolawi (Iyolawi) Memorial Hospital		Opp. Islamic High School, Orita Bashanu, Ibadan	Ibadan North	Oyo
6023	OY/0014	OY/0014/S/2	Lanark Specialist Hospital		Sw9/1423, State Hospital Road, Ring Road Area, Ibadan	Ibadan North East	Oyo
6024	OY/0014	OY/0014/S/4	Lanark Specialist Hospital		Sw9/1423, State Hospital Road, Ring Road Area, Ibadan	Ibadan North East	Oyo
6025	OY/0014	OY/0014/S/1	Lanark Specialist Hospital		Sw9/1423, State Hospital Road, Ring Road Area, Ibadan	Ibadan North East	Oyo
6026	OY/0014	OY/0014/S/5	Lanark Specialist Hospital		Sw9/1423, State Hospital Road, Ring Road Area, Ibadan	Ibadan North East	Oyo
6027	OY/0015	OY/0015/S/3	Christus Special Hospital		Zion Building, Kayode Obenbe, Crescent, New Ire Road, Ibadan	Ibadan North	Oyo
6028	OY/0017	OY/0017/S/5	Skyline Specialist Clinic	Laboratory, O & G	Dugbe, Ibadan	Ibadan North East	Oyo
6029	OY/0017	OY/0017/S/3	Skyline Specialist Clinic	Laboratory, O & G	Dugbe, Ibadan	Ibadan North East	Oyo
6030	OY/0020	OY/0020/S/5	Alafia Medical Laboratory Services	Laboratory	Premier Club Close, Owode, Oyo, Oyo State	Ibadan North	Oyo
6031	OY/0028	OY/0028/S/3	Lade Hospital & Maternity		SW 1171b Agege, Ibadan	Ibadan South West	Oyo
6032	OY/0028	OY/0028/S/6	Lade Hospital & Maternity		SW 1171b Agege, Ibadan	Ibadan South West	Oyo
6033	OY/0028	OY/0028/S/1	Lade Hospital & Maternity		SW 1171b Agege, Ibadan	Ibadan South West	Oyo
6034	OY/0028	OY/0028/S/2	Lade Hospital & Maternity		SW 1171b Agege, Ibadan	Ibadan South West	Oyo
6035	OY/0031	OY/0031/S/3	The Vine Hospital & Maternity Centre		Block 10 Plot 24, Alafin Avenue, Oluyole Extension, Ibadan	Ibadan South West	Oyo
6036	OY/0031	OY/0031/S/1	The Vine Hospital & Maternity Centre		Block 10 Plot 24, Alafin Avenue, Oluyole Extension, Ibadan	Ibadan South West	Oyo
6037	OY/0032	OY/0032/S/7	Oke-Ado Hospital		SW7/217, Ibukun Olu Street, Oke Ado, Ibadan	Ibadan South West	Oyo
6038	OY/0032	OY/0032/S/1	Oke-Ado Hospital		SW7/217, Ibukun Olu Street, Oke Ado, Ibadan	Ibadan South West	Oyo

6039	OY/0032	OY/0032/S/3	Oke-Ado Hospital		SW7/217, Ibukun Olu Street, Oke Ado, Ibadan	Ibadan South West	Oyo
6040	OY/0032	OY/0032/S/6	Oke-Ado Hospital		SW7/217, Ibukun Olu Street, Oke Ado, Ibadan	Ibadan South West	Oyo
6041	OY/0032	OY/0032/S/5	Oke-Ado Hospital		SW7/217, Ibukun Olu Street, Oke Ado, Ibadan	Ibadan South West	Oyo
6042	OY/0034	OY/0034/S/14	Trinity Hospital (Nig) Ltd.		84, Fajuyi Road Ekotedo Adamasingga, Ibadan, Oyo State	Ibadan South West	Oyo
6043	OY/0039	OY/0039/S/1	Oke Ado Hospital	O&G, Paediatrics, Gen. Surgery	SW4/217, Ibukun Olu Street, Eko-Tedo, Ibadan	Ibadan South West	Oyo
6044	OY/0039	OY/0039/S/6	Oke Ado Hospital	O&G, Paediatrics, Gen. Surgery	SW4/217, Ibukun Olu Street, Eko-Tedo, Ibadan	Ibadan South West	Oyo
6045	OY/0039	OY/0039/S/3	Oke Ado Hospital	O&G, Paediatrics, Gen. Surgery	SW4/217, Ibukun Olu Street, Eko-Tedo, Ibadan	Ibadan South West	Oyo
6046	OY/0042	OY/0042/S/3	Kejide Specialist Hospital		Sw 9/704a Elewure Str., Challenge, Ibadan	Ibadan North	Oyo
6047	OY/0042	OY/0042/S/8	Kejide Specialist Hospital		Sw 9/704a Elewure Str., Challenge, Ibadan	Ibadan North	Oyo
6048	OY/0042	OY/0042/S/2	Kejide Specialist Hospital		Sw 9/704a Elewure Str., Challenge, Ibadan	Ibadan North	Oyo
6049	OY/0042	OY/0042/S/6	Kejide Specialist Hospital		Sw 9/704a Elewure Str., Challenge, Ibadan	Ibadan North	Oyo
6050	OY/0042	OY/0042/S/7	Kejide Specialist Hospital		Sw 9/704a Elewure Str., Challenge, Ibadan	Ibadan North	Oyo
6051	OY/0042	OY/0042/S/1	Kejide Specialist Hospital		Sw 9/704a Elewure Str., Challenge, Ibadan	Ibadan North	Oyo
6052	OY/0042	OY/0042/S/5	Kejide Specialist Hospital		Sw 9/704a Elewure Str., Challenge, Ibadan	Ibadan North	Oyo
6053	OY/0043	OY/0043/S/2	Mak Clinic		By Alma Guest House Junction, Opp. Nitel New Bodija, Ibadan	Ibadan North	Oyo
6054	OY/0043	OY/0043/S/3	Mak Clinic		By Alma Guest House Junction, Opp. Nitel New Bodija, Ibadan	Ibadan North	Oyo
6055	OY/0043	OY/0043/S/1	Mak Clinic		By Alma Guest House Junction, Opp. Nitel New Bodija, Ibadan	Ibadan North	Oyo
6056	OY/0043	OY/0043/S/5	Mak Clinic		By Alma Guest House Junction, Opp. Nitel New Bodija, Ibadan	Ibadan North	Oyo
6057	OY/0044	OY/0044/S/4	Alafia Hospital	-	Mokola, Ibadan	Ibadan North	Oyo
6058	OY/0044	OY/0044/S/3	Alafia Hospital	-	Mokola, Ibadan	Ibadan North	Oyo
6059	OY/0044	OY/0044/S/1	Alafia Hospital	-	Mokola, Ibadan	Ibadan North	Oyo
6060	OY/0044	OY/0044/S/7	Alafia Hospital	-	Mokola, Ibadan	Ibadan North	Oyo
6061	OY/0044	OY/0044/S/5	Alafia Hospital	-	Mokola, Ibadan	Ibadan North	Oyo
6062	OY/0050	OY/0050/S/5	State Hospital	Gen Surgery, O & G, Pharmacy, Physiotherapy, Laboratory	Ibadan	Ibadan North	Oyo
6063	OY/0050	OY/0050/S/1	State Hospital	Gen Surgery, O & G, Pharmacy, Physiotherapy, Laboratory	Ibadan	Ibadan North	Oyo
6064	OY/0050	OY/0050/S/11	State Hospital	Gen Surgery, O & G, Pharmacy, Physiotherapy, Laboratory	Ibadan	Ibadan North	Oyo
6065	OY/0050	OY/0050/S/4	State Hospital	Gen Surgery, O & G, Pharmacy, Physiotherapy, Laboratory	Ibadan	Ibadan North	Oyo
6066	OY/0050	OY/0050/S/3	State Hospital	Gen Surgery, O & G, Pharmacy, Physiotherapy, Laboratory	Ibadan	Ibadan North	Oyo
6067	OY/0051	OY/0051/S/1	Teju Specialist Hospital		6 Alh. Animasaun Street, Ring Road, Ibadan	Ibadan South-East	Oyo
6068	OY/0051	OY/0051/S/3	Teju Specialist Hospital		6 Alh. Animasaun Street, Ring Road, Ibadan	Ibadan South-East	Oyo
6069	OY/0051	OY/0051/S/6	Teju Specialist Hospital		6 Alh. Animasaun Street, Ring Road, Ibadan	Ibadan South-East	Oyo
6070	OY/0051	OY/0051/S/2	Teju Specialist Hospital		6 Alh. Animasaun Street, Ring Road, Ibadan	Ibadan South-East	Oyo
6071	OY/0051	OY/0051/S/4	Teju Specialist Hospital		6 Alh. Animasaun Street, Ring Road, Ibadan	Ibadan South-East	Oyo
6072	OY/0051	OY/0051/S/5	Teju Specialist Hospital		6 Alh. Animasaun Street, Ring Road, Ibadan	Ibadan South-East	Oyo
6073	OY/0051	OY/0051/S/7	Teju Specialist Hospital		6 Alh. Animasaun Street, Ring Road, Ibadan	Ibadan South-East	Oyo
6074	OY/0052	OY/0052/S/1	Toun Memorial Specialist Hospital		Km 1/2 New Life Road, Ibadan	Ibadan South-East	Oyo
6075	OY/0052	OY/0052/S/7	Toun Memorial Specialist Hospital		Km 1/2 New Life Road, Ibadan	Ibadan South-East	Oyo
6076	OY/0052	OY/0052/S/5	Toun Memorial Specialist Hospital		Km 1/2 New Life Road, Ibadan	Ibadan South-East	Oyo

6077	OY/0052	OY/0052/S/6	Toun Memorial Specialist Hospital		Km 1/2 New Life Road, Ibadan	Ibadan South-East	Oyo
6078	OY/0052	OY/0052/S/3	Toun Memorial Specialist Hospital		Km 1/2 New Life Road, Ibadan	Ibadan South-East	Oyo
6079	OY/0063	OY/0063/S/5	Dorji Hospital		14, Avenue New Bodija, Ibadan	Ibadan North	Oyo
6080	OY/0063	OY/0063/S/7	Dorji Hospital		14, Avenue New Bodija, Ibadan	Ibadan North	Oyo
6081	OY/0065	OY/0065/S/6	Molly Specialist Hospital		No.6, American Quarters Yidi, Ibadan	Ibadan South West	Oyo
6082	OY/0065	OY/0065/S/2	Molly Specialist Hospital		No.6, American Quarters Yidi, Ibadan	Ibadan South West	Oyo
6083	OY/0065	OY/0065/S/5	Molly Specialist Hospital		No.6, American Quarters Yidi, Ibadan	Ibadan South West	Oyo
6084	OY/0065	OY/0065/S/12	Molly Specialist Hospital		No.6, American Quarters Yidi, Ibadan	Ibadan South West	Oyo
6085	OY/0065	OY/0065/S/11	Molly Specialist Hospital		No.6, American Quarters Yidi, Ibadan	Ibadan South West	Oyo
6086	OY/0065	OY/0065/S/4	Molly Specialist Hospital		No.6, American Quarters Yidi, Ibadan	Ibadan South West	Oyo
6087	OY/0065	OY/0065/S/3	Molly Specialist Hospital		No.6, American Quarters Yidi, Ibadan	Ibadan South West	Oyo
6088	OY/0065	OY/0065/S/1	Molly Specialist Hospital		No.6, American Quarters Yidi, Ibadan	Ibadan South West	Oyo
6089	OY/0065	OY/0065/S/7	Molly Specialist Hospital		No.6, American Quarters Yidi, Ibadan	Ibadan South West	Oyo
6090	OY/0065	OY/0065/S/10	Molly Specialist Hospital		No.6, American Quarters Yidi, Ibadan	Ibadan South West	Oyo
6091	OY/0067	OY/0067/S/3	Lad Medical Centre		1 Lad Hospital Road, Beside Police Barracks, Orita Challenge, Ibadan	Ibadan South West	Oyo
6092	OY/0067	OY/0067/S/5	Lad Medical Centre		1 Lad Hospital Road, Beside Police Barracks, Orita Challenge, Ibadan	Ibadan South West	Oyo
6093	OY/0067	OY/0067/S/1	Lad Medical Centre		1 Lad Hospital Road, Beside Police Barracks, Orita Challenge, Ibadan	Ibadan South West	Oyo
6094	OY/0068	OY/0068/S/1	Al-Hayyu Hospital	Primary, General Surgery, Laboratory, Internal Medicine, Radiology	Saw-Mill Ife Road, Ibadan	Ibadan North East	Oyo
6095	OY/0068	OY/0068/S/2	Al-Hayyu Hospital	Primary, General Surgery, Laboratory, Internal Medicine, Radiology	Saw-Mill Ife Road, Ibadan	Ibadan North East	Oyo
6096	OY/0068	OY/0068/S/5	Al-Hayyu Hospital	Primary, General Surgery, Laboratory, Internal Medicine, Radiology	Saw-Mill Ife Road, Ibadan	Ibadan North East	Oyo
6097	OY/0068	OY/0068/S/7	Al-Hayyu Hospital	Primary, General Surgery, Laboratory, Internal Medicine, Radiology	Saw-Mill Ife Road, Ibadan	Ibadan North East	Oyo
6098	OY/0068	OY/0068/S/3	Al-Hayyu Hospital	Primary, General Surgery, Laboratory, Internal Medicine, Radiology	Saw-Mill Ife Road, Ibadan	Ibadan North East	Oyo
6099	OY/0070	OY/0070/S/14	Asvon Hospital		Km 11/4 Opp. New Gbagi Market, New Life Road, Ibadan	Ibadan North East	Oyo
6100	OY/0070	OY/0070/S/5	Asvon Hospital		Km 11/4 Opp. New Gbagi Market, New Life Road, Ibadan	Ibadan North East	Oyo
6101	OY/0070	OY/0070/S/2	Asvon Hospital		Km 11/4 Opp. New Gbagi Market, New Life Road, Ibadan	Ibadan North East	Oyo
6102	OY/0071	OY/0071/S/3	New Bodija Hospital Group		3, Ayoola Drive, New Bodija, Ibadan	Ibadan North	Oyo
6103	OY/0072	OY/0072/S/5	Lafia Hospital		Sw 9/960 Abeokuta Road, Apata, Ibadan	Ibadan South West	Oyo
6104	OY/0072	OY/0072/S/7	Lafia Hospital		Sw 9/960 Abeokuta Road, Apata, Ibadan	Ibadan South West	Oyo
6105	OY/0072	OY/0072/S/3	Lafia Hospital		Sw 9/960 Abeokuta Road, Apata, Ibadan	Ibadan South West	Oyo

6106	OY/0073	OY/0073/S/1	Fadebi Memorial Hospital		Km 7, Iwo Road (Oki), Ibadan	Ibadan North East	Oyo
6107	OY/0073	OY/0073/S/3	Fadebi Memorial Hospital		Km 7, Iwo Road (Oki), Ibadan	Ibadan North East	Oyo
6108	OY/0075	OY/0075/S/5	Medical Practitioners Services		Mokola Roundabout, Ibadan	Ibadan North	Oyo
6109	OY/0075	OY/0075/S/3	Medical Practitioners Services		Mokola Roundabout, Ibadan	Ibadan North	Oyo
6110	OY/0075	OY/0075/S/1	Medical Practitioners Services		Mokola Roundabout, Ibadan	Ibadan North	Oyo
6111	OY/0076	OY/0076/S/5	Imperial Hospital		Yemetu, Ibadan	Ibadan North	Oyo
6112	OY/0077	OY/0077/S/2	Beta-Life Hospital		14, Popoola Yemoja Street, Off Awolowo Avenue, Oke Ado, Ibadan	Ibadan South West	Oyo
6113	OY/0077	OY/0077/S/3	Beta-Life Hospital		14, Popoola Yemoja Street, Off Awolowo Avenue, Oke Ado, Ibadan	Ibadan South West	Oyo
6114	OY/0077	OY/0077/S/6	Beta-Life Hospital		14, Popoola Yemoja Street, Off Awolowo Avenue, Oke Ado, Ibadan	Ibadan South West	Oyo
6115	OY/0077	OY/0077/S/1	Beta-Life Hospital		14, Popoola Yemoja Street, Off Awolowo Avenue, Oke Ado, Ibadan	Ibadan South West	Oyo
6116	OY/0078	OY/0078/S/12	Highland Specialist Hospital		The House Of Hearing, Adeoyo Hospital Road, Yemetu, Ibadan	Ibadan North	Oyo
6117	OY/0079	OY/0079/S/2	St. Mary's Catholic General Hospital, Eleta, Ibadan		Eleta, Ibadan, Oyo State	Ibadan South West	Oyo
6118	OY/0079	OY/0079/S/5	St. Mary's Catholic General Hospital, Eleta, Ibadan		Eleta, Ibadan, Oyo State	Ibadan South West	Oyo
6119	OY/0079	OY/0079/S/4	St. Mary's Catholic General Hospital, Eleta, Ibadan		Eleta, Ibadan, Oyo State	Ibadan South West	Oyo
6120	OY/0079	OY/0079/S/3	St. Mary's Catholic General Hospital, Eleta, Ibadan		Eleta, Ibadan, Oyo State	Ibadan South West	Oyo
6121	OY/0079	OY/0079/S/1	St. Mary's Catholic General Hospital, Eleta, Ibadan		Eleta, Ibadan, Oyo State	Ibadan South West	Oyo
6122	OY/0079	OY/0079/S/7	St. Mary's Catholic General Hospital, Eleta, Ibadan		Eleta, Ibadan, Oyo State	Ibadan South West	Oyo
6123	OY/0079	OY/0079/S/6	St. Mary's Catholic General Hospital, Eleta, Ibadan		Eleta, Ibadan, Oyo State	Ibadan South West	Oyo
6124	OY/0081	OY/0081/S/5	Skyline Specialist Clinic		14, Olalekan Alabi Close, Off Abayomi Bus Stop, Iwo Road, Ibadan, Oyo State	Ibadan North	Oyo
6125	OY/0081	OY/0081/S/3	Skyline Specialist Clinic		14, Olalekan Alabi Close, Off Abayomi Bus Stop, Iwo Road, Ibadan, Oyo State	Ibadan North	Oyo
6126	OY/0083	OY/0083/S/4	Baptist Hospital		Oyo, Ibadan	Oyo West	Oyo
6127	OY/0083	OY/0083/S/1	Baptist Hospital		Oyo, Ibadan	Oyo West	Oyo
6128	OY/0083	OY/0083/S/5	Baptist Hospital		Oyo, Ibadan	Oyo West	Oyo
6129	OY/0083	OY/0083/S/7	Baptist Hospital		Oyo, Ibadan	Oyo West	Oyo
6130	OY/0083	OY/0083/S/6	Baptist Hospital		Oyo, Ibadan	Oyo West	Oyo
6131	OY/0083	OY/0083/S/2	Baptist Hospital		Oyo, Ibadan	Oyo West	Oyo
6132	OY/0083	OY/0083/S/3	Baptist Hospital		Oyo, Ibadan	Oyo West	Oyo
6133	OY/0086	OY/0086/S/3	Oyin Specialist Hospital		Airport Road, Ibadan	Ibadan North	Oyo
6134	OY/0088	OY/0088/S/5	Giftrolab	Laboratory	Yemetu N/4263B, Adeoye Road, beside Mobil Petrol Station	Ibadan North	Oyo
6135	OY/0090	OY/0090/S/5	Emma Laboratory	Laboratory	Lateef Afolabi House, Behind BZN Petrol Station, Oyo.	Ibadan North West	Oyo
6136	OY/0092	OY/0092/S/4	Kamy Pharmacy	Pharmacy	NW4/183, George street, Odi-Ora Eko Tedo	Ibadan North	Oyo
6137	OY/0093	OY/0093/S/4	Bimot Pharmacy (Nig)	Pharmacy	No.4, Ojo Ikolaba Street, Ikolaba	Ibadan North	Oyo
6138	OY/0094	OY/0094/S/4	Shekinah Glory Pharmacy	Pharmacy	Ibadan, Oyo State.	Ibadan North	Oyo
6139	OY/0096	OY/0096/S/4	Layis Pharmacy	Pharmacy	Oke-Ado, Sabo, Ogbomoso, baptist Medical Centre, Ogbomoso	Ogbomoso South	Oyo
6140	OY/0097	OY/0097/S/4	Kavillne Pharmacy	Pharmacy	Apake, Ilorin, Ogbomoso	Ogbomoso South	Oyo

6141	OY/0101	OY/0101/S/5	Ultimate Medical Diagnostic Lab.	Laboratory	Oare Ofe Commercial Complex, Opp. Jidex Industries, Apata Du	Ibadan South West	Oyo
6142	OY/0102	OY/0102/S/5	Topmed laboratory Services	Laboratory	Plot 10, balogun Kobomoje Street, Near 'D' Ravans, Ring Road	Ibadan South West	Oyo
6143	OY/0103	OY/0103/S/5	Confidence med. Diag. Centre	Laboratory	13, Poly Road, Sango	Ibadan North	Oyo
6144	OY/0104	OY/0104/S/5	ACE Lab. Med. Diag. Centre	Laboratory	114/742A Yemetu Market, Adeoye Street	Ibadan North	Oyo
6145	OY/0105	OY/0105/S/5	Breeze Diag. Medical Lab. Centre	Laboratory	2, W.O. Lawal Str., Opp. NEPA Zonal Hqrs, Ring Road	Ibadan South West	Oyo
6146	OY/0106	OY/0106/S/5	Search Med. Diag. Centre Ltd	Laboratory	238, Obafemi Awolowo Way, Opp. UMC	Ibadan North	Oyo
6147	OY/0122	OY/0122/S/4	Ireti Oluwa Hospital		Apake Area Ogbomosho, Ibadan	Ogbomoso South	Oyo
6148	OY/0122	OY/0122/S/5	Ireti Oluwa Hospital		Apake Area Ogbomosho, Ibadan	Ogbomoso South	Oyo
6149	OY/0130	OY/0130/S/7	Two Tees X-Ray Centre	Radiology	No. 4/460, Aladorin Street Yemetu, Ibadan	Ibadan North	Oyo
6150	OY/0131	OY/0131/S/11	Oluwaseun Physiotheraphy Clinic	Physiotherapy	9, Sanda Street Molete, Ibadan	Ibadan North	Oyo
6151	OY/0132	OY/0132/S/8	Modot Dental Clinic	Dental	Uncle Joe Building Oyo Road, Mokola, Ibadan	Ibadan North	Oyo
6152	OY/0134	OY/0134/S/8	Tos Dental Clinic	Dental	53, Oyo Road, Ibadan	Ibadan South West	Oyo
6153	OY/0139	OY/0139/S/4	Oluyole Chemist	Pharmacy	NW7/555, Eleyele, Ibadan	Ibadan North	Oyo
6154	OY/0140	OY/0140/S/4	Babmor Pharmacy	Pharmacy	30, Oyo Road, Agbowo-Ibadan	Ibadan North	Oyo
6155	OY/0142	OY/0142/S/4	Bamulot Pharmacy & Stores	Pharmacy	Opp. New Gbagi Market, Old Ife Road, Ibadan	Ibadan South West	Oyo
6156	OY/0143	OY/0143/S/4	Skybond Pharmacy L td.	Pharmacy	Liberty Junction, Oke-Ado, Ibadan	Ibadan South West	Oyo
6157	OY/0144	OY/0144/S/4	Okay Pharmacy & Stores	Pharmacy	Agbowo Shopping Complex, Opp. U.I. Ibadan	Ibadan North	Oyo
6158	OY/0145	OY/0145/S/4	Pharmalot Ltd.	Pharmacy	Unit 3, Exescee Shopping Complex, Bode Wasinmi, Ibadan	Ibadan North	Oyo
6159	OY/0146	OY/0146/S/4	Doksy Pharmacy	Pharmacy	Gbaremun Market Poly Road, Ibadan	Ibadan North	Oyo
6160	OY/0148	OY/0148/S/4	Glolade Pharmacy Ltd.	Pharmacy	Ojoo T. Junction, Ibadan	Ibadan North	Oyo
6161	OY/0149	OY/0149/S/4	Tanike Pharmacy Ltd.	Pharmacy	Opp. Texaco Petrol Station, Monatan, Ibadan	Ibadan North	Oyo
6162	OY/0150	OY/0150/S/4	Funmac Pharmacy	Pharmacy	Oluyole Estate Junction, Ring Road, Ibadan	Ibadan South West	Oyo
6163	OY/0151	OY/0151/S/4	Fadey Allamnda Pharmacy	Pharmacy	E9/912 Abayomi Iwo Road, Ibadan	Ibadan North	Oyo
6164	OY/0152	OY/0152/S/4	Nubifal Pharmacy & Stores Ltd	Pharmacy	1, Bamigbola Avenue Ashi, Ibadan	Ibadan North	Oyo
6165	OY/0153	OY/0153/S/4	Trumed Ltd.	Pharmacy	SW9/1427, New Adeoyo Rd., Ring Road, Ibadan	Ibadan South West	Oyo
6166	OY/0157	OY/0157/S/4	Boset (Nig) Ltd.	Pharmacy	SW9/159B Abeokuta Road, Apata, Ibadan	Ibadan South West	Oyo
6167	OY/0159	OY/0159/S/4	Gaal Pharmaceutical Shop	Pharmacy	3, Hamdu Ajibade Complex, Agodi Gate, Ibadan	Ibadan North	Oyo
6168	OY/0160	OY/0160/S/4	Tellade Pharmacy & Stores	Pharmacy	Alhaji Alabi Shopping Complex, Molete-Junction, Ibadan, Oyo State	Ibadan North	Oyo
6169	OY/0165	OY/0165/S/3	2 Div Hospital		Adekunle Fajuyi Cantonment, Ibadan	Ibadan North	Oyo
6170	OY/0165	OY/0165/S/4	2 Div Hospital		Adekunle Fajuyi Cantonment, Ibadan	Ibadan North	Oyo
6171	OY/0165	OY/0165/S/8	2 Div Hospital		Adekunle Fajuyi Cantonment, Ibadan	Ibadan North	Oyo
6172	OY/0165	OY/0165/S/5	2 Div Hospital		Adekunle Fajuyi Cantonment, Ibadan	Ibadan North	Oyo
6173	OY/0165	OY/0165/S/1	2 Div Hospital		Adekunle Fajuyi Cantonment, Ibadan	Ibadan North	Oyo
6174	OY/0165	OY/0165/S/7	2 Div Hospital		Adekunle Fajuyi Cantonment, Ibadan	Ibadan North	Oyo
6175	OY/0167	OY/0167/S/3	Muslim Hospital		Afote Road, Shaki	Saki West	Oyo
6176	OY/0167	OY/0167/S/5	Muslim Hospital		Afote Road, Shaki	Saki West	Oyo
6177	OY/0167	OY/0167/S/7	Muslim Hospital		Afote Road, Shaki	Saki West	Oyo
6178	OY/0167	OY/0167/S/1	Muslim Hospital		Afote Road, Shaki	Saki West	Oyo
6179	OY/0172	OY/0172/S/3	Ireti Oluwa Hospital		Kajola Saw-Mill, Apake, Ogbomosho	Ogbomoso North	Oyo
6180	OY/0172	OY/0172/S/5	Ireti Oluwa Hospital		Kajola Saw-Mill, Apake, Ogbomosho	Ogbomoso North	Oyo
6181	OY/0172	OY/0172/S/4	Ireti Oluwa Hospital		Kajola Saw-Mill, Apake, Ogbomosho	Ogbomoso North	Oyo
6182	OY/0173	OY/0173/S/7	General Hospital, Iseyin		Iseyin, Oyo State.	Iseyin	Oyo

6183	OY/0173	OY/0173/S/3	General Hospital, Iseyin		Iseyin, Oyo State.	Iseyin	Oyo
6184	OY/0173	OY/0173/S/1	General Hospital, Iseyin		Iseyin, Oyo State.	Iseyin	Oyo
6185	OY/0173	OY/0173/S/4	General Hospital, Iseyin		Iseyin, Oyo State.	Iseyin	Oyo
6186	OY/0173	OY/0173/S/5	General Hospital, Iseyin		Iseyin, Oyo State.	Iseyin	Oyo
6187	OY/0174	OY/0174/S/1	Our Lady Catholic Hospital		Iseyin, Oyo State.	Iseyin	Oyo
6188	OY/0174	OY/0174/S/5	Our Lady Catholic Hospital		Iseyin, Oyo State.	Iseyin	Oyo
6189	OY/0174	OY/0174/S/3	Our Lady Catholic Hospital		Iseyin, Oyo State.	Iseyin	Oyo
6190	OY/0174	OY/0174/S/4	Our Lady Catholic Hospital		Iseyin, Oyo State.	Iseyin	Oyo
6191	OY/0174	OY/0174/S/15	Our Lady Catholic Hospital		Iseyin, Oyo State.	Iseyin	Oyo
6192	OY/0174	OY/0174/S/7	Our Lady Catholic Hospital		Iseyin, Oyo State.	Iseyin	Oyo
6193	OY/0175	OY/0175/S/11	Adeoyo Maternity Hospital		Yemetu Road, Ibadan.	Ibadan North	Oyo
6194	OY/0175	OY/0175/S/5	Adeoyo Maternity Hospital		Yemetu Road, Ibadan.	Ibadan North	Oyo
6195	OY/0175	OY/0175/S/3	Adeoyo Maternity Hospital		Yemetu Road, Ibadan.	Ibadan North	Oyo
6196	OY/0175	OY/0175/S/6	Adeoyo Maternity Hospital		Yemetu Road, Ibadan.	Ibadan North	Oyo
6197	OY/0175	OY/0175/S/4	Adeoyo Maternity Hospital		Yemetu Road, Ibadan.	Ibadan North	Oyo
6198	OY/0175	OY/0175/S/15	Adeoyo Maternity Hospital		Yemetu Road, Ibadan.	Ibadan North	Oyo
6199	OY/0175	OY/0175/S/7	Adeoyo Maternity Hospital		Yemetu Road, Ibadan.	Ibadan North	Oyo
6200	OY/0176	OY/0176/S/4	Ring Road Hospital		Ring Road, Ibadan	Ibadan South West	Oyo
6201	OY/0176	OY/0176/S/15	Ring Road Hospital		Ring Road, Ibadan	Ibadan South West	Oyo
6202	OY/0176	OY/0176/S/11	Ring Road Hospital		Ring Road, Ibadan	Ibadan South West	Oyo
6203	OY/0176	OY/0176/S/7	Ring Road Hospital		Ring Road, Ibadan	Ibadan South West	Oyo
6204	OY/0176	OY/0176/S/5	Ring Road Hospital		Ring Road, Ibadan	Ibadan South West	Oyo
6205	OY/0176	OY/0176/S/3	Ring Road Hospital		Ring Road, Ibadan	Ibadan South West	Oyo
6206	OY/0176	OY/0176/S/10	Ring Road Hospital		Ring Road, Ibadan	Ibadan South West	Oyo
6207	OY/0177	OY/0177/S/7	Jerico General Hospital		Magazine Road, Jerico, Ibadan.	Ibadan North	Oyo
6208	OY/0177	OY/0177/S/4	Jerico General Hospital		Magazine Road, Jerico, Ibadan.	Ibadan North	Oyo
6209	OY/0177	OY/0177/S/3	Jerico General Hospital		Magazine Road, Jerico, Ibadan.	Ibadan North	Oyo
6210	OY/0177	OY/0177/S/1	Jerico General Hospital		Magazine Road, Jerico, Ibadan.	Ibadan North	Oyo
6211	OY/0177	OY/0177/S/5	Jerico General Hospital		Magazine Road, Jerico, Ibadan.	Ibadan North	Oyo
6212	OY/0177	OY/0177/S/11	Jerico General Hospital		Magazine Road, Jerico, Ibadan.	Ibadan North	Oyo
6213	OY/0178	OY/0178/S/1	State Hospital, Oyo		Oyo, Oyo State	Oyo West	Oyo
6214	OY/0178	OY/0178/S/5	State Hospital, Oyo		Oyo, Oyo State	Oyo West	Oyo
6215	OY/0178	OY/0178/S/4	State Hospital, Oyo		Oyo, Oyo State	Oyo West	Oyo
6216	OY/0178	OY/0178/S/11	State Hospital, Oyo		Oyo, Oyo State	Oyo West	Oyo
6217	OY/0178	OY/0178/S/7	State Hospital, Oyo		Oyo, Oyo State	Oyo West	Oyo
6218	OY/0178	OY/0178/S/3	State Hospital, Oyo		Oyo, Oyo State	Oyo West	Oyo
6219	OY/0179	OY/0179/S/5	State Hospital, Ogbomoso		Ogbomosho, Oyo State.	Ogbomoso South	Oyo
6220	OY/0179	OY/0179/S/4	State Hospital, Ogbomoso		Ogbomosho, Oyo State.	Ogbomoso South	Oyo
6221	OY/0179	OY/0179/S/7	State Hospital, Ogbomoso		Ogbomosho, Oyo State.	Ogbomoso South	Oyo
6222	OY/0179	OY/0179/S/1	State Hospital, Ogbomoso		Ogbomosho, Oyo State.	Ogbomoso South	Oyo
6223	OY/0179	OY/0179/S/3	State Hospital, Ogbomoso		Ogbomosho, Oyo State.	Ogbomoso South	Oyo
6224	OY/0180	OY/0180/S/4	Oni Memorial Children Hospital		Ring Road, Ibadan	Ibadan South West	Oyo
6225	OY/0180	OY/0180/S/6	Oni Memorial Children Hospital		Ring Road, Ibadan	Ibadan South West	Oyo
6226	OY/0180	OY/0180/S/5	Oni Memorial Children Hospital		Ring Road, Ibadan	Ibadan South West	Oyo
6227	OY/0180	OY/0180/S/7	Oni Memorial Children Hospital		Ring Road, Ibadan	Ibadan South West	Oyo
6228	OY/0182	OY/0182/S/8	State Hospital, Saki		Saki, Sango Road, Ibadan, Oyo State	Saki West	Oyo
6229	OY/0182	OY/0182/S/7	State Hospital, Saki		Saki, Sango Road, Ibadan, Oyo State	Saki West	Oyo
6230	OY/0182	OY/0182/S/5	State Hospital, Saki		Saki, Sango Road, Ibadan, Oyo State	Saki West	Oyo

6231	OY/0182	OY/0182/S/3	State Hospital, Saki		Saki, Sango Road, Ibadan, Oyo State	Saki West	Oyo
6232	OY/0182	OY/0182/S/1	State Hospital, Saki		Saki, Sango Road, Ibadan, Oyo State	Saki West	Oyo
6233	OY/0182	OY/0182/S/4	State Hospital, Saki		Saki, Sango Road, Ibadan, Oyo State	Saki West	Oyo
6234	OY/0183	OY/0183/S/1	Baptist Medical Centre, Saki		Ajegunle Area, Saki, Oyo State	Saki East	Oyo
6235	OY/0183	OY/0183/S/3	Baptist Medical Centre, Saki		Ajegunle Area, Saki, Oyo State	Saki East	Oyo
6236	OY/0183	OY/0183/S/6	Baptist Medical Centre, Saki		Ajegunle Area, Saki, Oyo State	Saki East	Oyo
6237	OY/0183	OY/0183/S/4	Baptist Medical Centre, Saki		Ajegunle Area, Saki, Oyo State	Saki East	Oyo
6238	OY/0183	OY/0183/S/5	Baptist Medical Centre, Saki		Ajegunle Area, Saki, Oyo State	Saki East	Oyo
6239	OY/0183	OY/0183/S/7	Baptist Medical Centre, Saki		Ajegunle Area, Saki, Oyo State	Saki East	Oyo
6240	OY/0183	OY/0183/S/15	Baptist Medical Centre, Saki		Ajegunle Area, Saki, Oyo State	Saki East	Oyo
6241	OY/0184	OY/0184/S/7	Jerico Nursing Home		Magazine Road, Ibadan.	Ibadan South-East	Oyo
6242	OY/0184	OY/0184/S/11	Jerico Nursing Home		Magazine Road, Ibadan.	Ibadan South-East	Oyo
6243	OY/0184	OY/0184/S/1	Jerico Nursing Home		Magazine Road, Ibadan.	Ibadan South-East	Oyo
6244	OY/0184	OY/0184/S/4	Jerico Nursing Home		Magazine Road, Ibadan.	Ibadan South-East	Oyo
6245	OY/0184	OY/0184/S/3	Jerico Nursing Home		Magazine Road, Ibadan.	Ibadan South-East	Oyo
6246	OY/0184	OY/0184/S/5	Jerico Nursing Home		Magazine Road, Ibadan.	Ibadan South-East	Oyo
6247	OY/0188	OY/0188/S/5	Cottage Hospital/Comprehensive Health Centre		Oyo Police Command, Ibadan, Oyo State	Ibadan North	Oyo
6248	OY/0188	OY/0188/S/3	Cottage Hospital/Comprehensive Health Centre		Oyo Police Command, Ibadan, Oyo State	Ibadan North	Oyo
6249	OY/0188	OY/0188/S/1	Cottage Hospital/Comprehensive Health Centre		Oyo Police Command, Ibadan, Oyo State	Ibadan North	Oyo
6250	OY/0188	OY/0188/S/8	Cottage Hospital/Comprehensive Health Centre		Oyo Police Command, Ibadan, Oyo State	Ibadan North	Oyo
6251	OY/0188	OY/0188/S/6	Cottage Hospital/Comprehensive Health Centre		Oyo Police Command, Ibadan, Oyo State	Ibadan North	Oyo
6252	OY/0188	OY/0188/S/15	Cottage Hospital/Comprehensive Health Centre		Oyo Police Command, Ibadan, Oyo State	Ibadan North	Oyo
6253	OY/0188	OY/0188/S/2	Cottage Hospital/Comprehensive Health Centre		Oyo Police Command, Ibadan, Oyo State	Ibadan North	Oyo
6254	OY/0188	OY/0188/S/7	Cottage Hospital/Comprehensive Health Centre		Oyo Police Command, Ibadan, Oyo State	Ibadan North	Oyo
6255	OY/0188	OY/0188/S/4	Cottage Hospital/Comprehensive Health Centre		Oyo Police Command, Ibadan, Oyo State	Ibadan North	Oyo
6256	OY/0189	OY/0189/S/14	Queen's Hospital	Primary	38, Iya Olobe St. Dugbe Eketedo Ibadan	Ibadan North West	Oyo
6257	OY/0189	OY/0189/S/2	Queen's Hospital	Primary	38, Iya Olobe St. Dugbe Eketedo Ibadan	Ibadan North West	Oyo
6258	OY/0191	OY/0191/S/5	Unity Medical Centre	Primary	31 Eleyele Rd Ibadan	Ibadan North	Oyo
6259	OY/0191	OY/0191/S/1	Unity Medical Centre	Primary	31 Eleyele Rd Ibadan	Ibadan North	Oyo
6260	OY/0192	OY/0192/S/1	St. Joseph Medical Centre	Primary	No 27 Akintola Rd Ekotedo Ibadan	Ibadan North East	Oyo
6261	OY/0193	OY/0193/S/2	Full House Spec. Dermatological Hospital	Primary, Dermatology	37, Adeniran Onyulola Ave, Ring Rd Ibadan	Ibadan South West	Oyo
6262	OY/0195	OY/0195/S/1	Anu Oluwa Specialist Hospital	Primary	19 Bluegate Avenue, Oluyole Estate Extension, Ibadan	Ibadan South West	Oyo
6263	OY/0195	OY/0195/S/3	Anu Oluwa Specialist Hospital	Primary	19 Bluegate Avenue, Oluyole Estate Extension, Ibadan	Ibadan South West	Oyo
6264	OY/0196	OY/0196/S/3	Joy Hospital & Maternity Centre		32 Morgan St. Oke-Ado Ibadan	Ibadan South West	Oyo
6265	OY/0198	OY/0198/S/5	Mommoh Memorial Hospital		Idi-Ope Oyo	Oyo West	Oyo
6266	OY/0206	OY/0206/S/7	Shalom Medical Centre	Primary	Sabo Area Ogbomosho	Ogbomoso North	Oyo
6267	OY/0206	OY/0206/S/1	Shalom Medical Centre	Primary	Sabo Area Ogbomosho	Ogbomoso North	Oyo
6268	OY/0206	OY/0206/S/5	Shalom Medical Centre	Primary	Sabo Area Ogbomosho	Ogbomoso North	Oyo
6269	OY/0211	OY/0211/S/3	Zoe Specialist Mission Hospital		No.4 Adeogun Str., Oluyele Estate, Ibadan	Ibadan North West	Oyo

6270	OY/0211	OY/0211/S/1	Zoe Specialist Mission Hospital		No.4 Adeogun Str., Oluyele Estate, Ibadan	Ibadan North West	Oyo
6271	OY/0216	OY/0216/S/8	Alfa Dental Clinic	Dental	Shop No 4 Cocco House Anex, Dugbe, Ibadan	Ibadan North West	Oyo
6272	OY/0218	OY/0218/S/13	Wesco Clinic	Optometry	55 Oyo Road, Opp AG Leventis Sango, Ibadan	Ibadan North	Oyo
6273	OY/0219	OY/0219/S/13	Canan Optical Services	Optometry	82 Fajuyi Road Adamasingba Opp gate 3, Ibadan	Ibadan North West	Oyo
6274	OY/0221	OY/0221/S/5	Narkod Biomedical Lab.	Laboratory	19 Lagos Bypass, Idiodo Challenge	Ibadan South West	Oyo
6275	OY/0225	OY/0225/S/13	Sharp View Opthometric Clinic	Opthometry	64 Lagos Road Challenge, Ibadan	Ibadan South West	Oyo
6276	OY/0226	OY/0226/S/11	Jay-Kay Physiotherapy Clinic	Physiotherapy	8 Road Mokola Roundabout, Ibadan	Ibadan North	Oyo
6277	OY/0227	OY/0227/S/5	Amedic Medical Laboratory	Laboratory	96 Ile-Ekp Bus Stop, Iwo Road	Ibadan North East	Oyo
6278	OY/0228	OY/0228/S/4	St Mary's General Hospital	O&G,Surgery,Medicine,Paediatrics,Laboratory,X-ray,Pharmacy	Eleta, Near Academy	Ibadan South-East	Oyo
6279	OY/0228	OY/0228/S/5	St Mary's General Hospital	O&G,Surgery,Medicine,Paediatrics,Laboratory,X-ray,Pharmacy	Eleta, Near Academy	Ibadan South-East	Oyo
6280	OY/0228	OY/0228/S/7	St Mary's General Hospital	O&G,Surgery,Medicine,Paediatrics,Laboratory,X-ray,Pharmacy	Eleta, Near Academy	Ibadan South-East	Oyo
6281	OY/0228	OY/0228/S/3	St Mary's General Hospital	O&G,Surgery,Medicine,Paediatrics,Laboratory,X-ray,Pharmacy	Eleta, Near Academy	Ibadan South-East	Oyo
6282	OY/0228	OY/0228/S/1	St Mary's General Hospital	O&G,Surgery,Medicine,Paediatrics,Laboratory,X-ray,Pharmacy	Eleta, Near Academy	Ibadan South-East	Oyo
6283	OY/0228	OY/0228/S/2	St Mary's General Hospital	O&G,Surgery,Medicine,Paediatrics,Laboratory,X-ray,Pharmacy	Eleta, Near Academy	Ibadan South-East	Oyo
6284	OY/0228	OY/0228/S/6	St Mary's General Hospital	O&G,Surgery,Medicine,Paediatrics,Laboratory,X-ray,Pharmacy	Eleta, Near Academy	Ibadan South-East	Oyo
6285	OY/0229	OY/0229/S/13	Joanness Ophtalmic Centre	Opthometry	2nd Floor, Oyeks Plaza, Beside Mobil Filling Station, Awolowo Junction, Bodija, Ibadan	Ibadan South West	Oyo
6286	OY/0230	OY/0230/S/4	Maranatha Pharmacy	Pharmacy	Shop 8, Students'' Hostel I. A. R &T, Apata, Ibadan	Ido	Oyo
6287	OY/0231	OY/0231/S/4	Maranatha Pharmacy	Pharmacy	NCRI Quarters, Moore Plantation, Ibadan	Ido	Oyo
6288	OY/0232	OY/0232/S/5	Divine Diagnostic Centre	Laboratory	12 Akintola Street, Behind Agboro Shopping Complex, UI, Ibadan	Ibadan South West	Oyo
6289	OY/0237	OY/0237/S/4	Living Faith Pharmacy Ltd	Pharmacy	35A Ilaka Street, Near Akesan Market,Oyo	Oyo West	Oyo
6290	OY/0238	OY/0238/S/4	Omodunbi Pharmacy	Pharmacy	1 Abiodun Atiba Road, Behind Akesan garage, Oyo	Oyo East	Oyo
6291	OY/0239	OY/0239/S/4	Damorex Pharmacy	Pharmacy	Sw9/958 Abeokuta Road, Apata, Ibadan	Ido	Oyo
6292	OY/0240	OY/0240/S/14	Debo Ultra Sound Centre X-ray	USS,X-ray	Oke Balogun Road, Oyo	Oyo East	Oyo
6293	OY/0240	OY/0240/S/7	Debo Ultra Sound Centre X-ray	USS,X-ray	Oke Balogun Road, Oyo	Oyo East	Oyo
6294	OY/0241	OY/0241/S/4	Pharmaklinic Health & Pharmacy Shop	Pharmacy	11 Aare Avenue, Bodija	Ibadan North	Oyo
6295	OY/0242	OY/0242/S/3	Bowen University Teaching Hospital, Ogbomoso		Ogbomosho, Oyo State	Ogbomoso North	Oyo
6296	OY/0242	OY/0242/S/5	Bowen University Teaching Hospital, Ogbomoso		Ogbomosho, Oyo State	Ogbomoso North	Oyo
6297	OY/0242	OY/0242/S/4	Bowen University Teaching Hospital, Ogbomoso		Ogbomosho, Oyo State	Ogbomoso North	Oyo
6298	OY/0242	OY/0242/S/7	Bowen University Teaching Hospital, Ogbomoso		Ogbomosho, Oyo State	Ogbomoso North	Oyo
6299	OY/0242	OY/0242/S/8	Bowen University Teaching Hospital, Ogbomoso		Ogbomosho, Oyo State	Ogbomoso North	Oyo
6300	OY/0242	OY/0242/S/1	Bowen University Teaching Hospital, Ogbomoso		Ogbomosho, Oyo State	Ogbomoso North	Oyo
6301	OY/0242	OY/0242/S/2	Bowen University Teaching Hospital, Ogbomoso		Ogbomosho, Oyo State	Ogbomoso North	Oyo

6302	OY/0242	OY/0242/S/6	Bowen University Teaching Hospital, Ogbomoso		Ogbomosho, Oyo State	Ogbomoso North	Oyo
6303	OY/0243	OY/0243/S/9	New World Specialist Hospital		No. 7, Omona Yajo Close, Behind Children Home Sch. College Cresc., Molete Area, Ibadan	Ibadan South West	Oyo
6304	OY/0243	OY/0243/S/1	New World Specialist Hospital		No. 7, Omona Yajo Close, Behind Children Home Sch. College Cresc., Molete Area, Ibadan	Ibadan South West	Oyo
6305	OY/0243	OY/0243/S/5	New World Specialist Hospital		No. 7, Omona Yajo Close, Behind Children Home Sch. College Cresc., Molete Area, Ibadan	Ibadan South West	Oyo
6306	OY/0243	OY/0243/S/4	New World Specialist Hospital		No. 7, Omona Yajo Close, Behind Children Home Sch. College Cresc., Molete Area, Ibadan	Ibadan South West	Oyo
6307	OY/0245	OY/0245/S/14	Bisol Medical Centre	Primary	Ilora, Beside Immanuel Baptist Church Mafojubaye Street, Ilora	Afijio	Oyo
6308	OY/0250	OY/0250/S/7	Catholic Hospital, Oluyoro	General Surgery, Laboratory, O & G, Radiology, Paediatrics, Pharmacy, Orthopaedics	Oluyoro, Ibadan, Oyo State	Ibadan North East	Oyo
6309	OY/0250	OY/0250/S/5	Catholic Hospital, Oluyoro	General Surgery, Laboratory, O & G, Radiology, Paediatrics, Pharmacy, Orthopaedics	Oluyoro, Ibadan, Oyo State	Ibadan North East	Oyo
6310	OY/0250	OY/0250/S/1	Catholic Hospital, Oluyoro	General Surgery, Laboratory, O & G, Radiology, Paediatrics, Pharmacy, Orthopaedics	Oluyoro, Ibadan, Oyo State	Ibadan North East	Oyo
6311	OY/0250	OY/0250/S/10	Catholic Hospital, Oluyoro	General Surgery, Laboratory, O & G, Radiology, Paediatrics, Pharmacy, Orthopaedics	Oluyoro, Ibadan, Oyo State	Ibadan North East	Oyo
6312	OY/0250	OY/0250/S/6	Catholic Hospital, Oluyoro	General Surgery, Laboratory, O & G, Radiology, Paediatrics, Pharmacy, Orthopaedics	Oluyoro, Ibadan, Oyo State	Ibadan North East	Oyo
6313	OY/0250	OY/0250/S/4	Catholic Hospital, Oluyoro	General Surgery, Laboratory, O & G, Radiology, Paediatrics, Pharmacy, Orthopaedics	Oluyoro, Ibadan, Oyo State	Ibadan North East	Oyo
6314	OY/0250	OY/0250/S/3	Catholic Hospital, Oluyoro	General Surgery, Laboratory, O & G, Radiology, Paediatrics, Pharmacy, Orthopaedics	Oluyoro, Ibadan, Oyo State	Ibadan North East	Oyo
6315	OY/0251	OY/0251/S/2	Bethel Specialist Hospital		30, Oyo Road, Coca/Oremeji Area Ajibade Bus Stop, Ibadan	Ibadan North	Oyo
6316	OY/0251	OY/0251/S/3	Bethel Specialist Hospital		30, Oyo Road, Coca/Oremeji Area Ajibade Bus Stop, Ibadan	Ibadan North	Oyo
6317	OY/0251	OY/0251/S/1	Bethel Specialist Hospital		30, Oyo Road, Coca/Oremeji Area Ajibade Bus Stop, Ibadan	Ibadan North	Oyo
6318	OY/0252	OY/0252/S/3	University of Ibadan Health Services (Jaja Health Clinic)		Ibadan, Oyo State	Ibadan North	Oyo
6319	OY/0252	OY/0252/S/1	University of Ibadan Health Services (Jaja Health Clinic)		Ibadan, Oyo State	Ibadan North	Oyo
6320	OY/0252	OY/0252/S/9	University of Ibadan Health Services (Jaja Health Clinic)		Ibadan, Oyo State	Ibadan North	Oyo

6321	OY/0252	OY/0252/S/5	University of Ibadan Health Services (Jaja Health Clinic)		Ibadan, Oyo State	Ibadan North	Oyo
6322	OY/0252	OY/0252/S/2	University of Ibadan Health Services (Jaja Health Clinic)		Ibadan, Oyo State	Ibadan North	Oyo
6323	OY/0252	OY/0252/S/11	University of Ibadan Health Services (Jaja Health Clinic)		Ibadan, Oyo State	Ibadan North	Oyo
6324	OY/0252	OY/0252/S/4	University of Ibadan Health Services (Jaja Health Clinic)		Ibadan, Oyo State	Ibadan North	Oyo
6325	OY/0256	OY/0256/S/3	Abiola Memorial Hospital		N4/790 Yemetu, Ibadan	Ibadan North	Oyo
6326	OY/0258	OY/0258/S/1	Alawaye Hospital & Nursing Home Ltd		7, Alawaye Hospital Road, Iwo Road, Ibadan	Ibadan North West	Oyo
6327	OY/0258	OY/0258/S/3	Alawaye Hospital & Nursing Home Ltd		7, Alawaye Hospital Road, Iwo Road, Ibadan	Ibadan North West	Oyo
6328	OY/0263	OY/0263/S/1	Banby Specialist Hospital		Plot 11, Blk XLII, Akobo Estate, Olorunda-Abaa Rd, Ibadan	Lagelu	Oyo
6329	OY/0263	OY/0263/S/3	Banby Specialist Hospital		Plot 11, Blk XLII, Akobo Estate, Olorunda-Abaa Rd, Ibadan	Lagelu	Oyo
6330	OY/0263	OY/0263/S/6	Banby Specialist Hospital		Plot 11, Blk XLII, Akobo Estate, Olorunda-Abaa Rd, Ibadan	Lagelu	Oyo
6331	OY/0263	OY/0263/S/4	Banby Specialist Hospital		Plot 11, Blk XLII, Akobo Estate, Olorunda-Abaa Rd, Ibadan	Lagelu	Oyo
6332	OY/0263	OY/0263/S/5	Banby Specialist Hospital		Plot 11, Blk XLII, Akobo Estate, Olorunda-Abaa Rd, Ibadan	Lagelu	Oyo
6333	OY/0263	OY/0263/S/11	Banby Specialist Hospital		Plot 11, Blk XLII, Akobo Estate, Olorunda-Abaa Rd, Ibadan	Lagelu	Oyo
6334	OY/0263	OY/0263/S/7	Banby Specialist Hospital		Plot 11, Blk XLII, Akobo Estate, Olorunda-Abaa Rd, Ibadan	Lagelu	Oyo
6335	OY/0270	OY/0270/S/14	IBADAN CENTRAL HOSPITAL		OSOSAMI RD, OFF RING RD,IBADAN	Ibadan South West	Oyo
6336	OY/0271	OY/0271/S/8	FORTUNE HOSPITAL & DENTAL CENTRE		32 MKO ABIOLA WAY , RING RD, IBADAN, ANFANI ROUNDABOUT	Ibadan South West	Oyo
6337	OY/0271	OY/0271/S/6	FORTUNE HOSPITAL & DENTAL CENTRE		32 MKO ABIOLA WAY , RING RD, IBADAN, ANFANI ROUNDABOUT	Ibadan South West	Oyo
6338	OY/0271	OY/0271/S/2	FORTUNE HOSPITAL & DENTAL CENTRE		32 MKO ABIOLA WAY , RING RD, IBADAN, ANFANI ROUNDABOUT	Ibadan South West	Oyo
6339	OY/0272	OY/0272/S/3	HERITAGE COMPREHENSIVE HOSPITAL		57 ANFANI RD, RING RD, IBADAN	Ibadan South West	Oyo
6340	OY/0272	OY/0272/S/1	HERITAGE COMPREHENSIVE HOSPITAL		57 ANFANI RD, RING RD, IBADAN	Ibadan South West	Oyo
6341	OY/0275	OY/0275/S/5	MERCYLAND HOSPITAL & DENTAL		OPP. NEW GBAGI MARKET NEW IFE RD,IBADAN	Egbeda	Oyo
6342	OY/0275	OY/0275/S/8	MERCYLAND HOSPITAL & DENTAL		OPP. NEW GBAGI MARKET NEW IFE RD,IBADAN	Egbeda	Oyo
6343	OY/0276	OY/0276/S/3	EBENEZER CLINIC (HOSP. & MAT. HOME)		3/5 ONI LANE, OFF YEMETU ADEOYO RD,IBADAN	Ibadan North	Oyo
6344	OY/0276	OY/0276/S/1	EBENEZER CLINIC (HOSP. & MAT. HOME)		3/5 ONI LANE, OFF YEMETU ADEOYO RD,IBADAN	Ibadan North	Oyo
6345	OY/0278	OY/0278/S/3	Shalom Group Medical Centre		Aboderin Close, Agbaje Orita Challenge, Ibadan	Oluyole	Oyo
6346	OY/0285	OY/0285/S/14	Peamak Hospital & Maternity Centre		Alalubosa Road Oyo	Oyo West	Oyo
6347	OY/0285	OY/0285/S/4	Peamak Hospital & Maternity Centre		Alalubosa Road Oyo	Oyo West	Oyo
6348	OY/0285	OY/0285/S/7	Peamak Hospital & Maternity Centre		Alalubosa Road Oyo	Oyo West	Oyo
6349	OY/0290	OY/0290/S/5	ST.Jacob's Msission Hospital		Road 26, Owode Housing Estate, Beside Tafo Event Center, Along Abeokuta Road, Apata, Ibadan	Ido	Oyo
6350	OY/0290	OY/0290/S/1	ST.Jacob's Msission Hospital		Road 26, Owode Housing Estate, Beside Tafo Event Center, Along Abeokuta Road, Apata, Ibadan	Ido	Oyo
6351	OY/0291	OY/0291/S/3	ST.VINCENT MEDICAL CENTRE		34 AARE AVENUE , NEW BODIJA ESTATE,IBADAN	Ibadan North	Oyo
6352	OY/0297	OY/0297/S/4	AREYETALE PHARMACY	Pharmacy	107 ASHI RD, IBADAN	Ibadan North	Oyo
6353	OY/0303	OY/0303/S/4	BOSTAY NIG LTD	Pharmacy	IYANA CELE B/STOP OPP NNPC DEPOT, APATA	Ido	Oyo

6354	OY/0308	OY/0308/S/4	GILEAD PHARMACEUTICALS	Pharmacy	91-93 LAGOS RD, CHALLANGE (BESIDE TEXACO), IBADAN	Ibadan South West	Oyo
6355	OY/0326	OY/0326/S/4	NINIOLA PHARMACY	PHARMACY	6 OJOO STR, ACADEMY	Lagelu	Oyo
6356	OY/0327	OY/0327/S/7	OKE- ADO HOSPITAL	RADIOLOGY	SW 7/7 IBUKUN OLU ST, OKE-ADO , IBADAN	Ibadan South West	Oyo
6357	OY/0328	OY/0328/S/7	ORE- OFE X-RAY CENTRE	RADIOLOGY	BLK 1, PLOT 7 BEHIND IBADAN S-W LG SECT. OLUYOLE EST.RING RD,	Ibadan South West	Oyo
6358	OY/0334	OY/0334/S/5	STRONG TOWER MEDICAL DIAG. LAB	LABORATORY	SW 9/1150 AGAJE B/STOP, ORITA CHALLENGE ,IBADAN	Oluyole	Oyo
6359	OY/0340	OY/0340/S/4	WEST PHARM. LTD	PHARMACY	105 OBAFEMI AWOLOWO RD, OKE- ADO. IBADAN	Ibadan North West	Oyo
6360	OY/0342	OY/0342/S/4	TALADON NIG. LTD	PHARMACY	6 QUEEN ELIZABETH RD, MOKOLA, IBADAN	Ibadan North West	Oyo
6361	OY/0345	OY/0345/S/3	Chrisbo Medical Centre		House 11, Plot 1, Phase III, Bashorun Estate, Akobo, Ibadan	Lagelu	Oyo
6362	OY/0347	OY/0347/S/8	Grace Dental Clinic	Dental	Behind Agbowo Shopping Complex Inside Doctor''s Clinic UI Ibadan	Ibadan North	Oyo
6363	OY/0348	OY/0348/S/1	Foremost Base Hospital Limited		1 Rufai Street N6A/958 Coca Cola Area, Ibadan	Ibadan North	Oyo
6364	OY/0348	OY/0348/S/3	Foremost Base Hospital Limited		1 Rufai Street N6A/958 Coca Cola Area, Ibadan	Ibadan North	Oyo
6365	OY/0348	OY/0348/S/4	Foremost Base Hospital Limited		1 Rufai Street N6A/958 Coca Cola Area, Ibadan	Ibadan North	Oyo
6366	OY/0350	OY/0350/S/1	St Rita's Clinic & Maternity Home		9 Maboreje Street, Iwo Road, Ibadan	Ibadan North East	Oyo
6367	OY/0351	OY/0351/S/13	Larap Eye Clinic	Optometry	95 Adeoyo Road, Alawada Junction, Ibadan	Ibadan North	Oyo
6368	OY/0353	OY/0353/S/13	Corporate Eye Clinic	Optometry	20 Aare Avenue, New Bodija, Ibadan	Ibadan North	Oyo
6369	OY/0354	OY/0354/S/3	St Patric's Hospital		107/780 Idi Ope	Ibadan North	Oyo
6370	OY/0356	OY/0356/S/1	T.Z. Medical Centre	Surgery	Agbakin Layout, Iwo Road, Ibadean	Ibadan North East	Oyo
6371	OY/0360	OY/0360/S/2	KAY KAY SPECIALIST		PLOT 15 BLK 10 ORITA BASHERUN ESTATE,AKOBO IBADAN LAGELU L.G.A	Lagelu	Oyo
6372	OY/0360	OY/0360/S/1	KAY KAY SPECIALIST		PLOT 15 BLK 10 ORITA BASHERUN ESTATE,AKOBO IBADAN LAGELU L.G.A	Lagelu	Oyo
6373	OY/0360	OY/0360/S/15	KAY KAY SPECIALIST		PLOT 15 BLK 10 ORITA BASHERUN ESTATE,AKOBO IBADAN LAGELU L.G.A	Lagelu	Oyo
6374	OY/0361	OY/0361/S/1	Iye Hospital Ltd		22 Osibodu Layout Lagos/Ibadan Express Road, Ibadan	Ibadan South West	Oyo
6375	OY/0361	OY/0361/S/3	Iye Hospital Ltd		22 Osibodu Layout Lagos/Ibadan Express Road, Ibadan	Ibadan South West	Oyo
6376	OY/0361	OY/0361/S/6	Iye Hospital Ltd		22 Osibodu Layout Lagos/Ibadan Express Road, Ibadan	Ibadan South West	Oyo
6377	OY/0363	OY/0363/S/3	Samron Medical Centre		62 Adebiyi Street, Joyce - B Road off Ring Road Ibadan	Ibadan South West	Oyo
6378	OY/0363	OY/0363/S/5	Samron Medical Centre		62 Adebiyi Street, Joyce - B Road off Ring Road Ibadan	Ibadan South West	Oyo
6379	OY/0363	OY/0363/S/2	Samron Medical Centre		62 Adebiyi Street, Joyce - B Road off Ring Road Ibadan	Ibadan South West	Oyo
6380	OY/0363	OY/0363/S/1	Samron Medical Centre		62 Adebiyi Street, Joyce - B Road off Ring Road Ibadan	Ibadan South West	Oyo
6381	OY/0366	OY/0366/S/2	St Dominic Catholic Hospital		Ogungbade Village, New Ife Road, Ibadan	Egbeda	Oyo
6382	OY/0366	OY/0366/S/5	St Dominic Catholic Hospital		Ogungbade Village, New Ife Road, Ibadan	Egbeda	Oyo
6383	OY/0366	OY/0366/S/3	St Dominic Catholic Hospital		Ogungbade Village, New Ife Road, Ibadan	Egbeda	Oyo
6384	OY/0371	OY/0371/S/13	Trinity Optometry Clinic	Optometry	23 Ring Road Chemist Bus Stop, Ibadan	Ibadan South West	Oyo
6385	OY/0372	OY/0372/S/3	Immaculate Hospital		23 Odutola Road Off Ososami Road, Oke Ado, Ibadan	Ibadan South West	Oyo
6386	OY/0393	OY/0393/S/7	Muslim Ummah Hospital		KM 5 Kiamo Road Kisi	Irepo	Oyo
6387	OY/0393	OY/0393/S/4	Muslim Ummah Hospital		KM 5 Kiamo Road Kisi	Irepo	Oyo
6388	OY/0393	OY/0393/S/1	Muslim Ummah Hospital		KM 5 Kiamo Road Kisi	Irepo	Oyo

6389	OY/0401	OY/0401/S/5	Blossom Biomedical Laboratory	Laboratory	17 Queen Elizabeth Road, Opp Group Medical, Mokola Roundabout	Ibadan North	Oyo
6390	OY/0406	OY/0406/S/1	Camaria Specialist Hospital		13 Camaria Road, Oluwa Bashorun, Ibadan	Ibadan North	Oyo
6391	OY/0408	OY/0408/S/2	Elyon Sepcialist Clinic		72 Iwo Road, Ibadan	Ibadan North East	Oyo
6392	OY/0417	OY/0417/S/10	Sunnydale Hospital		1 Main Avenue, Alalubosa, GRA, Ibadan	Ibadan South West	Oyo
6393	OY/0421	OY/0421/S/8	Ire Ayo Dental Clinic	Dental	100 Adekunle Fajuyi Street, Suite 28, Danak, Adamasingba, Dugbe, Ibadan	Ibadan North East	Oyo
6394	OY/0426	OY/0426/S/1	Ayofunmi Hospital and Maternity		43 Polytechnic Road, Sango, Ibadan	Ibadan North	Oyo
6395	OY/0426	OY/0426/S/3	Ayofunmi Hospital and Maternity		43 Polytechnic Road, Sango, Ibadan	Ibadan North	Oyo
6396	OY/0428	OY/0428/S/15	Eleta Eye Institute	Opthalmology	Eleta, Ibadan	Ibadan South-East	Oyo
6397	OY/0429	OY/0429/S/1	Vine Branch Maternity and Infant Welfare Centre		Plot 4, Block 5, Road 302 Agodi GRA, Ibadan	Ibadan North East	Oyo
6398	OY/0429	OY/0429/S/3	Vine Branch Maternity and Infant Welfare Centre		Plot 4, Block 5, Road 302 Agodi GRA, Ibadan	Ibadan North East	Oyo
6399	OY/0429	OY/0429/S/6	Vine Branch Maternity and Infant Welfare Centre		Plot 4, Block 5, Road 302 Agodi GRA, Ibadan	Ibadan North East	Oyo
6400	OY/0434	OY/0434/S/7	Aramed Medical Centre		KM 15, Lagos-Ibadan Expressway, Ibadan	Oluyole	Oyo
6401	OY/0434	OY/0434/S/4	Aramed Medical Centre		KM 15, Lagos-Ibadan Expressway, Ibadan	Oluyole	Oyo
6402	OY/0434	OY/0434/S/5	Aramed Medical Centre		KM 15, Lagos-Ibadan Expressway, Ibadan	Oluyole	Oyo
6403	OY/0434	OY/0434/S/1	Aramed Medical Centre		KM 15, Lagos-Ibadan Expressway, Ibadan	Oluyole	Oyo
6404	OY/0434	OY/0434/S/3	Aramed Medical Centre		KM 15, Lagos-Ibadan Expressway, Ibadan	Oluyole	Oyo
6405	OY/0435	OY/0435/S/15	Foresight Specialist Eye Clinic		14, Awosika Avenue, off Awolowo Avenue, Old Bodija, Ibadan	Ibadan North	Oyo
6406	OY/0438	OY/0438/S/15	GFF Eye Clinic and Optical Services		6/484 Mokola Oremeji, Opposite Premier Hotel Junction, Oyo Road, Ibadan	Ibadan North	Oyo
6407	OY/0439	OY/0439/S/1	Hallel Specialist Hospital		# 11, Ilupeju Estate, Akobo-ojurin, Ibadan.	Lagelu	Oyo
6408	OY/0439	OY/0439/S/3	Hallel Specialist Hospital		# 11, Ilupeju Estate, Akobo-ojurin, Ibadan.	Lagelu	Oyo
6409	OY/0443	OY/0443/S/4	Yewatol Pharmacy		# 69, Anfani Layout Ibadan	Ibadan South West	Oyo
6410	OY/0444	OY/0444/S/4	Jare Pharmacy		# 1, Hospital Road, Sango Eruwa, Ibadan	Ibarapa Central	Oyo
6411	OY/0445	OY/0445/S/5	Lifelong Biomedical Diagnostic Centre		Opp Police Barracks playing ground, Police Barracks B/S, Yemetu, Ibadan	Ibadan North	Oyo
6412	OY/0446	OY/0446/S/4	St Anne's Specialist Hospital		#9, Moremi Rd, off Aare Avenue, Bodija, Ibadan	Ibadan North	Oyo
6413	OY/0446	OY/0446/S/3	St Anne's Specialist Hospital		#9, Moremi Rd, off Aare Avenue, Bodija, Ibadan	Ibadan North	Oyo
6414	OY/0446	OY/0446/S/5	St Anne's Specialist Hospital		#9, Moremi Rd, off Aare Avenue, Bodija, Ibadan	Ibadan North	Oyo
6415	OY/0449	OY/0449/S/2	Anu Oluwa Specialist Hospital, Ibadan		19, Bluegate Avenue, Oluyole Estate Extension, Ibadan	Ibadan South West	Oyo
6416	OY/0453	OY/0453/S/2	Oluwafemi Specialist Hospital		Gbogun Owode, Apata, Ibadan, Oyo State	Ido	Oyo
6417	OY/0462	OY/0462/S/5	The Horizon Hospital		23, Odutola Street, Behind Conac Optical Imalefalafia,Oke-Ado, Ibadan,Oyo State	Ibadan South West	Oyo
6418	OY/0462	OY/0462/S/3	The Horizon Hospital		23, Odutola Street, Behind Conac Optical Imalefalafia,Oke-Ado, Ibadan,Oyo State	Ibadan South West	Oyo
6419	OY/0464	OY/0464/S/5	Molete Diagnostic & Clinical Services Limited		270,Obafemi Awolowo Way, Molete, Oyo State	Ibadan South West	Oyo
6420	OY/0466	OY/0466/S/3	Royal Crown Specialist Hospital		Plot 7, Phase II Akobo, Ibadan, Oyo State	Lagelu	Oyo
6421	OY/0472	OY/0472/S/4	Omak Pharmacy		Suit 1- 2 Victoria's Place JK Bus Stop Agbowo, Ibadan, Oyo State	Ibadan North	Oyo
6422	OY/0473	OY/0473/S/4	Mettlesome Pharmacy		E7/9496, Babanla Road, Oremeji Agugu, Ibadan, Oyo State	Ona-Ara	Oyo

6423	OY/0475	OY/0475/S/5	College Medical Centre		Federal College Of Animal Health And Production Technology Ibadan, Oyo State	Ibadan South West	Oyo
6424	OY/0478	OY/0478/S/13	Joffany Eye Clinic		30, MKO Abiola Way, Anfani Junction, Ring road, Ibadan Oyo State.	Ibadan South West	Oyo
6425	OY/0481	OY/0481/S/15	Sight For Life Eye Centre		1 PDC Housing Estate, General Gas, Akobo, Ibadan Oyo State	Lagelu	Oyo
6426	OY/0482	OY/0482/S/5	Total Care Laboratory Ltd		Number 7, Arulogun Close, Awosika Avenue Bodija, Ibadan Oyo State	Ibadan North	Oyo
6427	OY/0484	OY/0484/S/8	Bozid Dental Clinic		Suite 58, Jasbeed Mall, Ashi-Bodija, Ibadan Oyo State	Ibadan North	Oyo
6428	OY/0485	OY/0485/S/1	Redeemer's Health Centre		37 Awolowo Avenue Old Bodija, Ibadan Oyo State	Ibadan North	Oyo
6429	OY/0485	OY/0485/S/14	Redeemer's Health Centre		37 Awolowo Avenue Old Bodija, Ibadan Oyo State	Ibadan North	Oyo
6430	OY/0486	OY/0486/S/7	Pen Atalanta Diagnostic Centre		Ajadi Street, Behind Total Petrol Station, Monatan, Ibadan Oyo State	Egbeda	Oyo
6431	OY/0486	OY/0486/S/5	Pen Atalanta Diagnostic Centre		Ajadi Street, Behind Total Petrol Station, Monatan, Ibadan Oyo State	Egbeda	Oyo
6432	OY/0492	OY/0492/S/13	Designer Eye Clinic		130 Oyo Road Opposite Niser Ojoo Ibadan Oyo State	Akinyele	Oyo
6433	OY/0495	OY/0495/S/13	Epic Eye Care		Railway Shopping Complex Dugbe, Ibadan Oyo State	Ibadan North West	Oyo
6434	OY/0499	OY/0499/S/4	Hermon Pharmcare Nig. Ltd.		1 Irepodun Close, Off Ososami New Adeoyo Round About Ring Road Ibadan Oyo State	Ibadan South West	Oyo
6435	OY/0502	OY/0502/S/3	Delight Hospital & Fertility Centre		No. 1, Emmanuel Close, Elewura, Ring road, Ibadan Oyo State	Ibadan South West	Oyo
6436	OY/0502	OY/0502/S/7	Delight Hospital & Fertility Centre		No. 1, Emmanuel Close, Elewura, Ring road, Ibadan Oyo State	Ibadan South West	Oyo
6437	OY/0504	OY/0504/S/6	God's Knot Hospital		7, Ologuneru Rd. Iyana Ekerin Beside Police Station Ibadan Oyo State	Ido	Oyo
6438	OY/0504	OY/0504/S/1	God's Knot Hospital		7, Ologuneru Rd. Iyana Ekerin Beside Police Station Ibadan Oyo State	Ido	Oyo
6439	OY/0504	OY/0504/S/2	God's Knot Hospital		7, Ologuneru Rd. Iyana Ekerin Beside Police Station Ibadan Oyo State	Ido	Oyo
6440	OY/0504	OY/0504/S/3	God's Knot Hospital		7, Ologuneru Rd. Iyana Ekerin Beside Police Station Ibadan Oyo State	Ido	Oyo
6441	OY/0505	OY/0505/S/13	Covenant Vision Clinic		6 Olalekan Alabi Road, Iwo Road, Ibadan Oyo State	Ibadan North East	Oyo
6442	OY/0506	OY/0506/S/4	Global T- Pharmacy & Store		Kumapayi Road ,Olodo Ibadan	Egbeda	Oyo
6443	OY/0508	OY/0508/S/4	Central Copharm LTD		Coopharmacy Building, Opp Police Post Molete Ibadan	Ibadan South West	Oyo
6444	OY/0509	OY/0509/S/4	Olabola Pharmacy		50,Abeokuta Road, Odo Ona Ibadan Oyo State	Ibadan South West	Oyo
6445	OY/0510	OY/0510/S/4	Beatmol Pharmacy		Plot 18,Block 34,Oluyole Estate ,Ring road Ibadan	Ibadan South West	Oyo
6446	OY/0511	OY/0511/S/8	Jubilee Hospital		Samonda Market Complex, Sango Behind Oyo State Trade Fair Complex, Ibadan Oyo State.	Ido	Oyo
6447	OY/0511	OY/0511/S/3	Jubilee Hospital		Samonda Market Complex, Sango Behind Oyo State Trade Fair Complex, Ibadan Oyo State.	Ido	Oyo
6448	OY/0513	OY/0513/S/3	Moviv Specialist Hopital Limited		Adetoun Area, Off Eruwa Road Ologuneru Eleyele, Ibadan Oyo State.	Ido	Oyo
6449	OY/0513	OY/0513/S/2	Moviv Specialist Hopital Limited		Adetoun Area, Off Eruwa Road Ologuneru Eleyele, Ibadan Oyo State.	Ido	Oyo
6450	OY/0513	OY/0513/S/1	Moviv Specialist Hopital Limited		Adetoun Area, Off Eruwa Road Ologuneru Eleyele, Ibadan Oyo State.	Ido	Oyo
6451	OY/0513	OY/0513/S/5	Moviv Specialist Hopital Limited		Adetoun Area, Off Eruwa Road Ologuneru Eleyele, Ibadan Oyo State.	Ido	Oyo

6452	OY/0515	OY/0515/S/4	Favour-Bel Pharmacy		No 5 Akintunde Ighalo Estate Agric. Olomi, Ibadan Oyo State	Oluyole	Oyo
6453	OY/0516	OY/0516/S/5	Ayokemi Hospital & ultrasound centre		Adex Bus/Stop Behind filling Station, Monatan Ibadan, Oyo State.	Lagelu	Oyo
6454	OY/0516	OY/0516/S/6	Ayokemi Hospital & ultrasound centre		Adex Bus/Stop Behind filling Station, Monatan Ibadan, Oyo State.	Lagelu	Oyo
6455	OY/0516	OY/0516/S/3	Ayokemi Hospital & ultrasound centre		Adex Bus/Stop Behind filling Station, Monatan Ibadan, Oyo State.	Lagelu	Oyo
6456	OY/0516	OY/0516/S/11	Ayokemi Hospital & ultrasound centre		Adex Bus/Stop Behind filling Station, Monatan Ibadan, Oyo State.	Lagelu	Oyo
6457	OY/0516	OY/0516/S/14	Ayokemi Hospital & ultrasound centre		Adex Bus/Stop Behind filling Station, Monatan Ibadan, Oyo State.	Lagelu	Oyo
6458	OY/0516	OY/0516/S/2	Ayokemi Hospital & ultrasound centre		Adex Bus/Stop Behind filling Station, Monatan Ibadan, Oyo State.	Lagelu	Oyo
6459	OY/0516	OY/0516/S/1	Ayokemi Hospital & ultrasound centre		Adex Bus/Stop Behind filling Station, Monatan Ibadan, Oyo State.	Lagelu	Oyo
6460	OY/0516	OY/0516/S/8	Ayokemi Hospital & ultrasound centre		Adex Bus/Stop Behind filling Station, Monatan Ibadan, Oyo State.	Lagelu	Oyo
6461	OY/0518	OY/0518/S/1	Ladoke Akintola University of Technology Teaching Hospital		Ogbomoso, Oyo State	Ogbomoso North	Oyo
6462	OY/0518	OY/0518/S/12	Ladoke Akintola University of Technology Teaching Hospital		Ogbomoso, Oyo State	Ogbomoso North	Oyo
6463	OY/0518	OY/0518/S/11	Ladoke Akintola University of Technology Teaching Hospital		Ogbomoso, Oyo State	Ogbomoso North	Oyo
6464	OY/0518	OY/0518/S/3	Ladoke Akintola University of Technology Teaching Hospital		Ogbomoso, Oyo State	Ogbomoso North	Oyo
6465	OY/0518	OY/0518/S/4	Ladoke Akintola University of Technology Teaching Hospital		Ogbomoso, Oyo State	Ogbomoso North	Oyo
6466	OY/0518	OY/0518/S/2	Ladoke Akintola University of Technology Teaching Hospital		Ogbomoso, Oyo State	Ogbomoso North	Oyo
6467	OY/0518	OY/0518/S/7	Ladoke Akintola University of Technology Teaching Hospital		Ogbomoso, Oyo State	Ogbomoso North	Oyo
6468	OY/0518	OY/0518/S/5	Ladoke Akintola University of Technology Teaching Hospital		Ogbomoso, Oyo State	Ogbomoso North	Oyo
6469	OY/0518	OY/0518/S/6	Ladoke Akintola University of Technology Teaching Hospital		Ogbomoso, Oyo State	Ogbomoso North	Oyo
6470	OY/0518	OY/0518/S/15	Ladoke Akintola University of Technology Teaching Hospital		Ogbomoso, Oyo State	Ogbomoso North	Oyo
6471	OY/0520	OY/0520/S/2	Ladoke Akintola University Of Technology		Ilorin Road Ogbomoso Oyo State	Ogbomoso North	Oyo
6472	OY/0520	OY/0520/S/3	Ladoke Akintola University Of Technology		Ilorin Road Ogbomoso Oyo State	Ogbomoso North	Oyo
6473	OY/0520	OY/0520/S/1	Ladoke Akintola University Of Technology		Ilorin Road Ogbomoso Oyo State	Ogbomoso North	Oyo
6474	OY/0520	OY/0520/S/15	Ladoke Akintola University Of Technology		Ilorin Road Ogbomoso Oyo State	Ogbomoso North	Oyo
6475	OY/0520	OY/0520/S/12	Ladoke Akintola University Of Technology		Ilorin Road Ogbomoso Oyo State	Ogbomoso North	Oyo
6476	OY/0520	OY/0520/S/11	Ladoke Akintola University Of Technology		Ilorin Road Ogbomoso Oyo State	Ogbomoso North	Oyo
6477	OY/0520	OY/0520/S/7	Ladoke Akintola University Of Technology		Ilorin Road Ogbomoso Oyo State	Ogbomoso North	Oyo

6478	OY/0520	OY/0520/S/6	Ladoke Akintola University Of Technology		Ilorin Road Ogbomoso Oyo State	Ogbomoso North	Oyo
6479	OY/0520	OY/0520/S/5	Ladoke Akintola University Of Technology		Ilorin Road Ogbomoso Oyo State	Ogbomoso North	Oyo
6480	OY/0520	OY/0520/S/4	Ladoke Akintola University Of Technology		Ilorin Road Ogbomoso Oyo State	Ogbomoso North	Oyo
6481	OY/0523	OY/0523/S/10	Popular Hospital		34,Arigidi Street, Old Bodija, Ibadan Oyo State	Ibadan North	Oyo
6482	OY/0524	OY/0524/S/3	Sure Hope Clinic		Carpenter Bus Stop, Ologuneru Eruwa Road Ibadan, Oyo State	Ido	Oyo
6483	OY/0525	OY/0525/S/3	Iremm Medical Centre		3,Ireakari Estate,Oluyole	Oluyole	Oyo
6484	OY/0526	OY/0526/S/13	Conac Optical		130,Obafemi Awolowo Oke Ado,Ibadan Oyo State	Ibadan North West	Oyo
6485	OY/0527	OY/0527/S/5	Lab Master Diagnostic Center		516,tolulope plaza JK bustop, Bodija, Ibadan	Ibadan North	Oyo
6486	OY/0529	OY/0529/S/4	Gracepath K Venture & Pharmacy		Suite 4, To God be the Glory Forever shopping complex, Ibadan	Ido	Oyo
6487	OY/0531	OY/0531/S/7	Total Care Diagnostic Centre		7, Arulogun Close Old Bodija, Ibadan	Ibadan North	Oyo
6488	OY/0531	OY/0531/S/5	Total Care Diagnostic Centre		7, Arulogun Close Old Bodija, Ibadan	Ibadan North	Oyo
6489	PL/0007	PL/0007/S/3	Eldin Specialist Hospital		12 Dogon Dutse Road, Jos	Jos North	Plateau
6490	PL/0007	PL/0007/S/4	Eldin Specialist Hospital		12 Dogon Dutse Road, Jos	Jos North	Plateau
6491	PL/0007	PL/0007/S/7	Eldin Specialist Hospital		12 Dogon Dutse Road, Jos	Jos North	Plateau
6492	PL/0007	PL/0007/S/5	Eldin Specialist Hospital		12 Dogon Dutse Road, Jos	Jos North	Plateau
6493	PL/0009	PL/0009/S/5	Sauki Clinic & Hospital		Joseph Gomwalk Road	Jos North	Plateau
6494	PL/0009	PL/0009/S/2	Sauki Clinic & Hospital		Joseph Gomwalk Road	Jos North	Plateau
6495	PL/0010	PL/0010/S/3	Kauna Hospital		35 Ahmadu Bello Way, On Rwang-Pam	Jos North	Plateau
6496	PL/0011	PL/0011/S/4	General Hospital, Langtang		Langtang, Jos	Jos North	Plateau
6497	PL/0011	PL/0011/S/8	General Hospital, Langtang		Langtang, Jos	Jos North	Plateau
6498	PL/0011	PL/0011/S/1	General Hospital, Langtang		Langtang, Jos	Jos North	Plateau
6499	PL/0011	PL/0011/S/5	General Hospital, Langtang		Langtang, Jos	Jos North	Plateau
6500	PL/0014	PL/0014/S/7	Chillas Specialist Hospital		20 Zaria Bye Pass, Jos	Pankshin	Plateau
6501	PL/0014	PL/0014/S/4	Chillas Specialist Hospital		20 Zaria Bye Pass, Jos	Pankshin	Plateau
6502	PL/0014	PL/0014/S/1	Chillas Specialist Hospital		20 Zaria Bye Pass, Jos	Pankshin	Plateau
6503	PL/0014	PL/0014/S/3	Chillas Specialist Hospital		20 Zaria Bye Pass, Jos	Pankshin	Plateau
6504	PL/0015	PL/0015/S/1	General Hospital, Pankshin		Pankshin	Pankshin	Plateau
6505	PL/0015	PL/0015/S/5	General Hospital, Pankshin		Pankshin	Pankshin	Plateau
6506	PL/0015	PL/0015/S/8	General Hospital, Pankshin		Pankshin	Pankshin	Plateau
6507	PL/0015	PL/0015/S/7	General Hospital, Pankshin		Pankshin	Pankshin	Plateau
6508	PL/0015	PL/0015/S/2	General Hospital, Pankshin		Pankshin	Pankshin	Plateau
6509	PL/0018	PL/0018/S/1	Alpha Medical Centre		7, Delimi Street, Jos	Jos North	Plateau
6510	PL/0018	PL/0018/S/3	Alpha Medical Centre		7, Delimi Street, Jos	Jos North	Plateau
6511	PL/0019	PL/0019/S/15	Adoose Specialist Hospital		25, Ibrahim Dasuki Street	Jos North	Plateau
6512	PL/0022	PL/0022/S/4	General Hospital, Shendam		Shendam	Shendam	Plateau
6513	PL/0022	PL/0022/S/8	General Hospital, Shendam		Shendam	Shendam	Plateau
6514	PL/0022	PL/0022/S/7	General Hospital, Shendam		Shendam	Shendam	Plateau
6515	PL/0026	PL/0026/S/4	Lamed Pharmacy	Pharmacy	Bauchi Road	Jos North	Plateau
6516	PL/0029	PL/0029/S/4	De-Havilah Pharmacy Ltd.	Pharmacy	KB 79T Gada Biu, Jos	Jos North	Plateau
6517	PL/0032	PL/0032/S/4	Lizcon Pharmacy	Pharmacy	Infront of Plateau Poly, Jos	Jos North	Plateau
6518	PL/0033	PL/0033/S/4	Dajobek Pharm. Ltd.	Pharmacy	71, Yakubu Gowon Way, Dadin Kowa, Jos	Jos North	Plateau
6519	PL/0035	PL/0035/S/4	Barns Pharmacy & Chem. Ltd.	Pharmacy	Barkin Ladi Road. Bukuru, Jos	Jos South	Plateau

6520	PL/0036	PL/0036/S/4	Tinna Pharmaceuticals Chemist	Pharmacy	N0. 18 Ahmadu Bello Way, Jos	Jos North	Plateau
6521	PL/0039	PL/0039/S/8	Dental Clinic	Dental	Ibrahim Taiwo Road, Beside Specialist Hospital, Jos	Jos North	Plateau
6522	PL/0041	PL/0041/S/4	Water Shade Stores	Pharmacy	4, Maingo Road, Jos	Jos North	Plateau
6523	PL/0043	PL/0043/S/4	Micony Nig. Ltd.	Pharmacy	24, Beach Road, Jos	Jos North	Plateau
6524	PL/0045	PL/0045/S/4	Dumjos Nig. Ltd.	Pharmacy	27, Ahmadu Bello Way, Jos	Jos North	Plateau
6525	PL/0046	PL/0046/S/4	Ice Me Central Pharmacy	Pharmacy	12, Murtala Mohammed Way, Opp. Juth, Jos	Jos North	Plateau
6526	PL/0048	PL/0048/S/4	Barkin Ladi Pharmacy	Pharmacy	19, Lawerence Onaja Road, Barkin Ladi	Barikin Ladi	Plateau
6527	PL/0054	PL/0054/S/4	Orit Pharmacy	Pharmacy	7, Old Airport Road, Junction, Jos	Jos North	Plateau
6528	PL/0055	PL/0055/S/4	Pfamex Nig. Ltd.	Pharmacy	4, Mission Street, Jos	Jos North	Plateau
6529	PL/0058	PL/0058/S/3	463 Nigerian Airforce Hospital		NAF Station, Jos	Jos North	Plateau
6530	PL/0058	PL/0058/S/6	463 Nigerian Airforce Hospital		NAF Station, Jos	Jos North	Plateau
6531	PL/0058	PL/0058/S/1	463 Nigerian Airforce Hospital		NAF Station, Jos	Jos North	Plateau
6532	PL/0058	PL/0058/S/5	463 Nigerian Airforce Hospital		NAF Station, Jos	Jos North	Plateau
6533	PL/0058	PL/0058/S/2	463 Nigerian Airforce Hospital		NAF Station, Jos	Jos North	Plateau
6534	PL/0058	PL/0058/S/7	463 Nigerian Airforce Hospital		NAF Station, Jos	Jos North	Plateau
6535	PL/0058	PL/0058/S/4	463 Nigerian Airforce Hospital		NAF Station, Jos	Jos North	Plateau
6536	PL/0058	PL/0058/S/11	463 Nigerian Airforce Hospital		NAF Station, Jos	Jos North	Plateau
6537	PL/0058	PL/0058/S/15	463 Nigerian Airforce Hospital		NAF Station, Jos	Jos North	Plateau
6538	PL/0058	PL/0058/S/8	463 Nigerian Airforce Hospital		NAF Station, Jos	Jos North	Plateau
6539	PL/0064	PL/0064/S/4	University of Jos Health Centre		Jos, Plateau State	Jos North	Plateau
6540	PL/0064	PL/0064/S/5	University of Jos Health Centre		Jos, Plateau State	Jos North	Plateau
6541	PL/0067	PL/0067/S/7	Matanmi Hospital	Primary Provider	89 Masallachin Juma''a Jos	Jos North	Plateau
6542	PL/0070	PL/0070/S/3	Nissi Dominus Medical Centre	Primary Provider	Jaktu Estate Mangu	Mangu	Plateau
6543	PL/0070	PL/0070/S/2	Nissi Dominus Medical Centre	Primary Provider	Jaktu Estate Mangu	Mangu	Plateau
6544	PL/0070	PL/0070/S/1	Nissi Dominus Medical Centre	Primary Provider	Jaktu Estate Mangu	Mangu	Plateau
6545	PL/0070	PL/0070/S/5	Nissi Dominus Medical Centre	Primary Provider	Jaktu Estate Mangu	Mangu	Plateau
6546	PL/0072	PL/0072/S/5	Samantha Lucil Hospital	Primary Provider	16, Enugu-Agidi street Jos	Jos North	Plateau
6547	PL/0072	PL/0072/S/3	Samantha Lucil Hospital	Primary Provider	16, Enugu-Agidi street Jos	Jos North	Plateau
6548	PL/0072	PL/0072/S/1	Samantha Lucil Hospital	Primary Provider	16, Enugu-Agidi street Jos	Jos North	Plateau
6549	PL/0075	PL/0075/S/4	Our Lady Of Apostle Hospital (O. L. A.)	Primary Provider	1 Zaria By pass Jos	Jos North	Plateau
6550	PL/0075	PL/0075/S/10	Our Lady Of Apostle Hospital (O. L. A.)	Primary Provider	1 Zaria By pass Jos	Jos North	Plateau
6551	PL/0075	PL/0075/S/15	Our Lady Of Apostle Hospital (O. L. A.)	Primary Provider	1 Zaria By pass Jos	Jos North	Plateau
6552	PL/0075	PL/0075/S/11	Our Lady Of Apostle Hospital (O. L. A.)	Primary Provider	1 Zaria By pass Jos	Jos North	Plateau
6553	PL/0075	PL/0075/S/2	Our Lady Of Apostle Hospital (O. L. A.)	Primary Provider	1 Zaria By pass Jos	Jos North	Plateau
6554	PL/0075	PL/0075/S/6	Our Lady Of Apostle Hospital (O. L. A.)	Primary Provider	1 Zaria By pass Jos	Jos North	Plateau
6555	PL/0075	PL/0075/S/1	Our Lady Of Apostle Hospital (O. L. A.)	Primary Provider	1 Zaria By pass Jos	Jos North	Plateau
6556	PL/0075	PL/0075/S/3	Our Lady Of Apostle Hospital (O. L. A.)	Primary Provider	1 Zaria By pass Jos	Jos North	Plateau
6557	PL/0075	PL/0075/S/5	Our Lady Of Apostle Hospital (O. L. A.)	Primary Provider	1 Zaria By pass Jos	Jos North	Plateau
6558	PL/0076	PL/0076/S/6	Solat Women Hospital	Primary Provider	44 Kashim Ibrahim Street Jos	Jos North	Plateau
6559	PL/0076	PL/0076/S/3	Solat Women Hospital	Primary Provider	44 Kashim Ibrahim Street Jos	Jos North	Plateau
6560	PL/0078	PL/0078/S/4	Federal Lowcost Clinic And Maternity	Primary Provider	Federal Lowcost Miango Road Jos	Jos South	Plateau
6561	PL/0078	PL/0078/S/5	Federal Lowcost Clinic And Maternity	Primary Provider	Federal Lowcost Miango Road Jos	Jos South	Plateau
6562	PL/0080	PL/0080/S/2	Lawrak Hospital	Primary Provider	21, Temple Road Off Miango Road Jos	Jos South	Plateau
6563	PL/0080	PL/0080/S/1	Lawrak Hospital	Primary Provider	21, Temple Road Off Miango Road Jos	Jos South	Plateau
6564	PL/0084	PL/0084/S/8	Hopmat Dental Clinic	Dental	12 Beach Rd Jos	Jos North	Plateau

6565	PL/0085	PL/0085/S/4	Monterri Pharmacy Ltd	Pharmacy	26 Miango Rd Kufang	Jos South	Plateau
6566	PL/0088	PL/0088/S/4	Choosey Pharmacy	Pharmacy	69/21 Masalachi Jumaa Jos	Jos North	Plateau
6567	PL/0090	PL/0090/S/5	Ebikon Medical Dagnostic Laboratory	Laboratory	10T Rukuba Rd Kayus House Rukuba Rd Jos	Jos North	Plateau
6568	PL/0094	PL/0094/S/4	Yachi Pharmacy	Pharmacy	2 Old Bukuru Rd Sabon Barki Jos	Jos South	Plateau
6569	PL/0095	PL/0095/S/13	Niger Optical Services Company	Optometry	61/14B Bauchi Rd Zololo Junction Jos	Jos North	Plateau
6570	PL/0100	PL/0100/S/7	Jos University Teaching Hospital		Murtala Mohammed Way, Jos	Jos North	Plateau
6571	PL/0100	PL/0100/S/11	Jos University Teaching Hospital		Murtala Mohammed Way, Jos	Jos North	Plateau
6572	PL/0100	PL/0100/S/2	Jos University Teaching Hospital		Murtala Mohammed Way, Jos	Jos North	Plateau
6573	PL/0100	PL/0100/S/1	Jos University Teaching Hospital		Murtala Mohammed Way, Jos	Jos North	Plateau
6574	PL/0100	PL/0100/S/5	Jos University Teaching Hospital		Murtala Mohammed Way, Jos	Jos North	Plateau
6575	PL/0100	PL/0100/S/3	Jos University Teaching Hospital		Murtala Mohammed Way, Jos	Jos North	Plateau
6576	PL/0100	PL/0100/S/6	Jos University Teaching Hospital		Murtala Mohammed Way, Jos	Jos North	Plateau
6577	PL/0100	PL/0100/S/13	Jos University Teaching Hospital		Murtala Mohammed Way, Jos	Jos North	Plateau
6578	PL/0102	PL/0102/S/15	Plateau Specialist Hospital		Jos, Plateau State	Jos North	Plateau
6579	PL/0102	PL/0102/S/1	Plateau Specialist Hospital		Jos, Plateau State	Jos North	Plateau
6580	PL/0102	PL/0102/S/7	Plateau Specialist Hospital		Jos, Plateau State	Jos North	Plateau
6581	PL/0102	PL/0102/S/6	Plateau Specialist Hospital		Jos, Plateau State	Jos North	Plateau
6582	PL/0102	PL/0102/S/4	Plateau Specialist Hospital		Jos, Plateau State	Jos North	Plateau
6583	PL/0102	PL/0102/S/3	Plateau Specialist Hospital		Jos, Plateau State	Jos North	Plateau
6584	PL/0102	PL/0102/S/5	Plateau Specialist Hospital		Jos, Plateau State	Jos North	Plateau
6585	PL/0108	PL/0108/S/1	Hwolshe Medical Centre		Tudun Wada Ring Road, P O Box 8103, Anglo, Jos.	Jos South	Plateau
6586	PL/0108	PL/0108/S/3	Hwolshe Medical Centre		Tudun Wada Ring Road, P O Box 8103, Anglo, Jos.	Jos South	Plateau
6587	PL/0108	PL/0108/S/2	Hwolshe Medical Centre		Tudun Wada Ring Road, P O Box 8103, Anglo, Jos.	Jos South	Plateau
6588	PL/0116	PL/0116/S/5	Focus Hospital		No. 7 NEPA Close, Tudun Wada Layout, Jos	Jos North	Plateau
6589	PL/0121	PL/0121/S/5	Vom Christian Hospital		Vom, Plateau State	Jos South	Plateau
6590	PL/0121	PL/0121/S/4	Vom Christian Hospital		Vom, Plateau State	Jos South	Plateau
6591	PL/0121	PL/0121/S/3	Vom Christian Hospital		Vom, Plateau State	Jos South	Plateau
6592	PL/0121	PL/0121/S/1	Vom Christian Hospital		Vom, Plateau State	Jos South	Plateau
6593	PL/0121	PL/0121/S/7	Vom Christian Hospital		Vom, Plateau State	Jos South	Plateau
6594	PL/0124	PL/0124/S/7	Skane Radio-Diagnostic Centre Nig. Ltd	Radiology/Sonolo gy	32B Ibrahim Taiwo Road, Jos, Opp. Plateau Hospital, Jos	Jos North	Plateau
6595	PL/0125	PL/0125/S/1	Mozad-Jones Pharmacy Ltd	Pharmacy	32, Ahmadu Bello Way, Jos	Jos North	Plateau
6596	PL/0128	PL/0128/S/8	Stan Fields Dental Clinic	Dental Surgery	22, Zaria Crescent Off Zaria Bye-Pass, Jos	Jos North	Plateau
6597	PL/0129	PL/0129/S/4	Jora Pharmacy Ltd	Pharmacy	13, Tudun Wada Ring Road, Jos	Jos North	Plateau
6598	PL/0133	PL/0133/S/4	GYFT'S Pharmacy Ltd	Pharmacy	Plot 20 Block Y Opp. ITFH Quarters, Jos	Mangu	Plateau
6599	PL/0134	PL/0134/S/4	Tahaf Pharmacy	Pharmacy	10, Murtala Mohammed Way, Opp. JUTH, Jos	Jos North	Plateau
6600	PL/0135	PL/0135/S/4	Trasure Base Pharmacy Ltd	Pharmacy	24, Murtala Mohammed Way, Jos	Jos North	Plateau
6601	PL/0136	PL/0136/S/4	Elyon Pharmacy	Pharmacy	12, CBN Road Opp House of Assembly, Jos	Jos North	Plateau
6602	PL/0137	PL/0137/S/4	Sali Pharmacy	Pharmacy	8, Jos Road, Opp. First Bank, Bukuru, Jos	Shendam	Plateau
6603	PL/0138	PL/0138/S/4	Sali Pharmacy	Pharmacy	6, Solomon Lar Way, Shendam, Jos	Shendam	Plateau
6604	PL/0148	PL/0148/S/5	Mandela Klinik		Beside Police Station K/Vom	Jos South	Plateau
6605	PL/0150	PL/0150/S/1	Bingham University Teaching Hospital, Jos		23 Zaria By Pass, Jos	Jos North	Plateau
6606	PL/0150	PL/0150/S/2	Bingham University Teaching Hospital, Jos		23 Zaria By Pass, Jos	Jos North	Plateau

6607	PL/0150	PL/0150/S/4	Bingham University Teaching Hospital, Jos		23 Zaria By Pass, Jos	Jos North	Plateau
6608	PL/0150	PL/0150/S/7	Bingham University Teaching Hospital, Jos		23 Zaria By Pass, Jos	Jos North	Plateau
6609	PL/0150	PL/0150/S/15	Bingham University Teaching Hospital, Jos		23 Zaria By Pass, Jos	Jos North	Plateau
6610	PL/0150	PL/0150/S/12	Bingham University Teaching Hospital, Jos		23 Zaria By Pass, Jos	Jos North	Plateau
6611	PL/0150	PL/0150/S/8	Bingham University Teaching Hospital, Jos		23 Zaria By Pass, Jos	Jos North	Plateau
6612	PL/0150	PL/0150/S/6	Bingham University Teaching Hospital, Jos		23 Zaria By Pass, Jos	Jos North	Plateau
6613	PL/0150	PL/0150/S/3	Bingham University Teaching Hospital, Jos		23 Zaria By Pass, Jos	Jos North	Plateau
6614	PL/0153	PL/0153/S/3	Graceville Royal Hospitals		23T Unity Lane, Dadin Kowa, Jos	Jos South	Plateau
6615	PL/0153	PL/0153/S/1	Graceville Royal Hospitals		23T Unity Lane, Dadin Kowa, Jos	Jos South	Plateau
6616	PL/0154	PL/0154/S/4	Ehigocho Pharmacy	Pharmacy	Yingi Village, Rayafield, Jos	Jos North	Plateau
6617	PL/0157	PL/0157/S/5	Gaccon Hospital &Maternity		2 Alheri Police Station, Jos, Plateau State.	Jos North	Plateau
6618	PL/0157	PL/0157/S/2	Gaccon Hospital &Maternity		2 Alheri Police Station, Jos, Plateau State.	Jos North	Plateau
6619	PL/0157	PL/0157/S/1	Gaccon Hospital &Maternity		2 Alheri Police Station, Jos, Plateau State.	Jos North	Plateau
6620	PL/0157	PL/0157/S/3	Gaccon Hospital &Maternity		2 Alheri Police Station, Jos, Plateau State.	Jos North	Plateau
6621	PL/0157	PL/0157/S/6	Gaccon Hospital &Maternity		2 Alheri Police Station, Jos, Plateau State.	Jos North	Plateau
6622	PL/0158	PL/0158/S/1	Dee Medical Centre		Plot BP 1669/21 Bukuru Express way, Off Kugiya Road, Jos, Plateau State.	Jos South	Plateau
6623	PL/0158	PL/0158/S/4	Dee Medical Centre		Plot BP 1669/21 Bukuru Express way, Off Kugiya Road, Jos, Plateau State.	Jos South	Plateau
6624	PL/0158	PL/0158/S/3	Dee Medical Centre		Plot BP 1669/21 Bukuru Express way, Off Kugiya Road, Jos, Plateau State.	Jos South	Plateau
6625	PL/0158	PL/0158/S/5	Dee Medical Centre		Plot BP 1669/21 Bukuru Express way, Off Kugiya Road, Jos, Plateau State.	Jos South	Plateau
6626	PL/0158	PL/0158/S/2	Dee Medical Centre		Plot BP 1669/21 Bukuru Express way, Off Kugiya Road, Jos, Plateau State.	Jos South	Plateau
6627	PL/0158	PL/0158/S/15	Dee Medical Centre		Plot BP 1669/21 Bukuru Express way, Off Kugiya Road, Jos, Plateau State.	Jos South	Plateau
6628	PL/0159	PL/0159/S/2	Fertile Ground Hospital		Plot 1234, Zaramaganda, Jos, Adjacent Golden Bread, Plateau State.	Jos South	Plateau
6629	PL/0159	PL/0159/S/6	Fertile Ground Hospital		Plot 1234, Zaramaganda, Jos, Adjacent Golden Bread, Plateau State.	Jos South	Plateau
6630	PL/0159	PL/0159/S/3	Fertile Ground Hospital		Plot 1234, Zaramaganda, Jos, Adjacent Golden Bread, Plateau State.	Jos South	Plateau
6631	PL/0159	PL/0159/S/1	Fertile Ground Hospital		Plot 1234, Zaramaganda, Jos, Adjacent Golden Bread, Plateau State.	Jos South	Plateau
6632	PL/0161	PL/0161/S/6	Marysol Specialist Hospital		Kwang Delimi, Off Rayfield Resort Road, Jos, Plateau State.	Jos South	Plateau
6633	PL/0161	PL/0161/S/1	Marysol Specialist Hospital		Kwang Delimi, Off Rayfield Resort Road, Jos, Plateau State.	Jos South	Plateau
6634	PL/0161	PL/0161/S/3	Marysol Specialist Hospital		Kwang Delimi, Off Rayfield Resort Road, Jos, Plateau State.	Jos South	Plateau
6635	PL/0161	PL/0161/S/2	Marysol Specialist Hospital		Kwang Delimi, Off Rayfield Resort Road, Jos, Plateau State.	Jos South	Plateau
6636	PL/0161	PL/0161/S/5	Marysol Specialist Hospital		Kwang Delimi, Off Rayfield Resort Road, Jos, Plateau State.	Jos South	Plateau
6637	PL/0161	PL/0161/S/4	Marysol Specialist Hospital		Kwang Delimi, Off Rayfield Resort Road, Jos, Plateau State.	Jos South	Plateau
6638	PL/0162	PL/0162/S/10	Rayfield Medical Service LTD		30, Raphael Davon Street, Rayfield Jos, Plateau State.	Jos South	Plateau

6639	PL/0162	PL/0162/S/3	Rayfield Medical Service LTD		30, Raphael Davon Street, Rayfield Jos, Plateau State.	Jos South	Plateau
6640	PL/0162	PL/0162/S/5	Rayfield Medical Service LTD		30, Raphael Davon Street, Rayfield Jos, Plateau State.	Jos South	Plateau
6641	PL/0162	PL/0162/S/6	Rayfield Medical Service LTD		30, Raphael Davon Street, Rayfield Jos, Plateau State.	Jos South	Plateau
6642	PL/0162	PL/0162/S/15	Rayfield Medical Service LTD		30, Raphael Davon Street, Rayfield Jos, Plateau State.	Jos South	Plateau
6643	PL/0162	PL/0162/S/1	Rayfield Medical Service LTD		30, Raphael Davon Street, Rayfield Jos, Plateau State.	Jos South	Plateau
6644	PL/0162	PL/0162/S/4	Rayfield Medical Service LTD		30, Raphael Davon Street, Rayfield Jos, Plateau State.	Jos South	Plateau
6645	PL/0163	PL/0163/S/2	Adventist Health International		Seventh Day Adventist Hospital, Jengre, Plateau State.	Bassa	Kogi
6646	PL/0163	PL/0163/S/3	Adventist Health International		Seventh Day Adventist Hospital, Jengre, Plateau State.	Bassa	Kogi
6647	PL/0163	PL/0163/S/4	Adventist Health International		Seventh Day Adventist Hospital, Jengre, Plateau State.	Bassa	Kogi
6648	PL/0163	PL/0163/S/1	Adventist Health International		Seventh Day Adventist Hospital, Jengre, Plateau State.	Bassa	Kogi
6649	PL/0163	PL/0163/S/5	Adventist Health International		Seventh Day Adventist Hospital, Jengre, Plateau State.	Bassa	Kogi
6650	PL/0167	PL/0167/S/5	Parkview Family Medicine Royal Hospital		By Solomon Lar Amusement Park, Domkat Bali Way, Jos, Plateau State	Jos South	Plateau
6651	PL/0167	PL/0167/S/3	Parkview Family Medicine Royal Hospital		By Solomon Lar Amusement Park, Domkat Bali Way, Jos, Plateau State	Jos South	Plateau
6652	PL/0167	PL/0167/S/4	Parkview Family Medicine Royal Hospital		By Solomon Lar Amusement Park, Domkat Bali Way, Jos, Plateau State	Jos South	Plateau
6653	PL/0169	PL/0169/S/3	Hope Hill Specialist Medical Centre		No. 7C, Hospital Place, Opp. Four Square Gospel Church, Along Green Valley Hotel, Jos	Jos North	Plateau
6654	PL/0169	PL/0169/S/1	Hope Hill Specialist Medical Centre		No. 7C, Hospital Place, Opp. Four Square Gospel Church, Along Green Valley Hotel, Jos	Jos North	Plateau
6655	PL/0170	PL/0170/S/8	Regional Centre for Oral Health for Africa (RCORTI)		3, CBN rd., Jos Plateau State	Jos North	Plateau
6656	PL/0171	PL/0171/S/3	Al Noor Clinic & Maternity		5, Madara Junction Angwan Rogo Jos Plateau State	Langtang North	Plateau
6657	RV/0001	RV/0001/S/3	Olivet Clinic		18 Ede Street, Ogbunabali Street	Port Harcourt	Rivers
6658	RV/0001	RV/0001/S/8	Olivet Clinic		18 Ede Street, Ogbunabali Street	Port Harcourt	Rivers
6659	RV/0001	RV/0001/S/2	Olivet Clinic		18 Ede Street, Ogbunabali Street	Port Harcourt	Rivers
6660	RV/0002	RV/0002/S/1	Olunwa Medical Centre		4 Amadi Ama Road, Trans-Amadi Layout (Rain-Bow Town)	Port Harcourt	Rivers
6661	RV/0002	RV/0002/S/5	Olunwa Medical Centre		4 Amadi Ama Road, Trans-Amadi Layout (Rain-Bow Town)	Port Harcourt	Rivers
6662	RV/0002	RV/0002/S/3	Olunwa Medical Centre		4 Amadi Ama Road, Trans-Amadi Layout (Rain-Bow Town)	Port Harcourt	Rivers
6663	RV/0003	RV/0003/S/5	Maryland Health Care		No.1 Igwuruta Road, Rumuokurushi	Port Harcourt	Rivers
6664	RV/0004	RV/0004/S/5	Springs Hospital		458, Ikwere Road by Kala B/Stop, Mile 5, Diobu	Obio/Akpor	Rivers
6665	RV/0004	RV/0004/S/2	Springs Hospital		458, Ikwere Road by Kala B/Stop, Mile 5, Diobu	Obio/Akpor	Rivers
6666	RV/0004	RV/0004/S/4	Springs Hospital		458, Ikwere Road by Kala B/Stop, Mile 5, Diobu	Obio/Akpor	Rivers
6667	RV/0004	RV/0004/S/1	Springs Hospital		458, Ikwere Road by Kala B/Stop, Mile 5, Diobu	Obio/Akpor	Rivers
6668	RV/0004	RV/0004/S/3	Springs Hospital		458, Ikwere Road by Kala B/Stop, Mile 5, Diobu	Obio/Akpor	Rivers
6669	RV/0004	RV/0004/S/7	Springs Hospital		458, Ikwere Road by Kala B/Stop, Mile 5, Diobu	Obio/Akpor	Rivers
6670	RV/0004	RV/0004/S/6	Springs Hospital		458, Ikwere Road by Kala B/Stop, Mile 5, Diobu	Obio/Akpor	Rivers

6671	RV/0005	RV/0005/S/7	Sophia Clinic		27 Old Aba Road, Rumuogba, Port Harcourt	Obio/Akpor	Rivers
6672	RV/0005	RV/0005/S/3	Sophia Clinic		27 Old Aba Road, Rumuogba, Port Harcourt	Obio/Akpor	Rivers
6673	RV/0005	RV/0005/S/1	Sophia Clinic		27 Old Aba Road, Rumuogba, Port Harcourt	Obio/Akpor	Rivers
6674	RV/0005	RV/0005/S/6	Sophia Clinic		27 Old Aba Road, Rumuogba, Port Harcourt	Obio/Akpor	Rivers
6675	RV/0005	RV/0005/S/5	Sophia Clinic		27 Old Aba Road, Rumuogba, Port Harcourt	Obio/Akpor	Rivers
6676	RV/0005	RV/0005/S/2	Sophia Clinic		27 Old Aba Road, Rumuogba, Port Harcourt	Obio/Akpor	Rivers
6677	RV/0005	RV/0005/S/10	Sophia Clinic		27 Old Aba Road, Rumuogba, Port Harcourt	Obio/Akpor	Rivers
6678	RV/0006	RV/0006/S/5	Ropheka Specialist Clinic		39, Elelenwo Street, Rumueme, P/Harcourt	Port Harcourt	Rivers
6679	RV/0006	RV/0006/S/4	Ropheka Specialist Clinic		39, Elelenwo Street, Rumueme, P/Harcourt	Port Harcourt	Rivers
6680	RV/0006	RV/0006/S/1	Ropheka Specialist Clinic		39, Elelenwo Street, Rumueme, P/Harcourt	Port Harcourt	Rivers
6681	RV/0006	RV/0006/S/3	Ropheka Specialist Clinic		39, Elelenwo Street, Rumueme, P/Harcourt	Port Harcourt	Rivers
6682	RV/0007	RV/0007/S/3	Anchor Hospital		km 15 P/H - Aba Expressway	Obio/Akpor	Rivers
6683	RV/0007	RV/0007/S/7	Anchor Hospital		km 15 P/H - Aba Expressway	Obio/Akpor	Rivers
6684	RV/0007	RV/0007/S/2	Anchor Hospital		km 15 P/H - Aba Expressway	Obio/Akpor	Rivers
6685	RV/0007	RV/0007/S/4	Anchor Hospital		km 15 P/H - Aba Expressway	Obio/Akpor	Rivers
6686	RV/0007	RV/0007/S/5	Anchor Hospital		km 15 P/H - Aba Expressway	Obio/Akpor	Rivers
6687	RV/0007	RV/0007/S/1	Anchor Hospital		km 15 P/H - Aba Expressway	Obio/Akpor	Rivers
6688	RV/0008	RV/0008/S/6	Princess Medical Centre		No.7 National Supply Road, Canco/Bewac Junction, Trans-Amadi Industrial Layout, P/Harcourt	Obio/Akpor	Rivers
6689	RV/0008	RV/0008/S/4	Princess Medical Centre		No.7 National Supply Road, Canco/Bewac Junction, Trans-Amadi Industrial Layout, P/Harcourt	Obio/Akpor	Rivers
6690	RV/0008	RV/0008/S/5	Princess Medical Centre		No.7 National Supply Road, Canco/Bewac Junction, Trans-Amadi Industrial Layout, P/Harcourt	Obio/Akpor	Rivers
6691	RV/0008	RV/0008/S/1	Princess Medical Centre		No.7 National Supply Road, Canco/Bewac Junction, Trans-Amadi Industrial Layout, P/Harcourt	Obio/Akpor	Rivers
6692	RV/0008	RV/0008/S/3	Princess Medical Centre		No.7 National Supply Road, Canco/Bewac Junction, Trans-Amadi Industrial Layout, P/Harcourt	Obio/Akpor	Rivers
6693	RV/0008	RV/0008/S/2	Princess Medical Centre		No.7 National Supply Road, Canco/Bewac Junction, Trans-Amadi Industrial Layout, P/Harcourt	Obio/Akpor	Rivers
6694	RV/0009	RV/0009/S/5	St. Jude		40 Elelenwo Road (Old Refinery Road) Oil Mill Junction, Rumukwurushi, PH	Port Harcourt	Rivers
6695	RV/0009	RV/0009/S/1	St. Jude		40 Elelenwo Road (Old Refinery Road) Oil Mill Junction, Rumukwurushi, PH	Port Harcourt	Rivers
6696	RV/0009	RV/0009/S/3	St. Jude		40 Elelenwo Road (Old Refinery Road) Oil Mill Junction, Rumukwurushi, PH	Port Harcourt	Rivers
6697	RV/0009	RV/0009/S/7	St. Jude		40 Elelenwo Road (Old Refinery Road) Oil Mill Junction, Rumukwurushi, PH	Port Harcourt	Rivers
6698	RV/0010	RV/0010/S/11	New Orlins Clinic		9 Orazi Road, Rumola	Obio/Akpor	Rivers
6699	RV/0010	RV/0010/S/6	New Orlins Clinic		9 Orazi Road, Rumola	Obio/Akpor	Rivers
6700	RV/0010	RV/0010/S/2	New Orlins Clinic		9 Orazi Road, Rumola	Obio/Akpor	Rivers
6701	RV/0010	RV/0010/S/1	New Orlins Clinic		9 Orazi Road, Rumola	Obio/Akpor	Rivers
6702	RV/0010	RV/0010/S/3	New Orlins Clinic		9 Orazi Road, Rumola	Obio/Akpor	Rivers
6703	RV/0010	RV/0010/S/5	New Orlins Clinic		9 Orazi Road, Rumola	Obio/Akpor	Rivers
6704	RV/0011	RV/0011/S/1	Harley Clinic		21B Akaninwor Road, Oroazi, Port Harcourt	Obio/Akpor	Rivers
6705	RV/0011	RV/0011/S/15	Harley Clinic		21B Akaninwor Road, Oroazi, Port Harcourt	Obio/Akpor	Rivers

6706	RV/0013	RV/0013/S/5	Family Medical Centre		39 Elelewon Road, Rumuokwurushi	Obio/Akpor	Rivers
6707	RV/0013	RV/0013/S/1	Family Medical Centre		39 Elelewon Road, Rumuokwurushi	Obio/Akpor	Rivers
6708	RV/0013	RV/0013/S/3	Family Medical Centre		39 Elelewon Road, Rumuokwurushi	Obio/Akpor	Rivers
6709	RV/0015	RV/0015/S/5	Eddy Medical Centre		4, Ndoki Lane, Behind 53 Market Road, Rumuomasi, P/H	Obio/Akpor	Rivers
6710	RV/0015	RV/0015/S/3	Eddy Medical Centre		4, Ndoki Lane, Behind 53 Market Road, Rumuomasi, P/H	Obio/Akpor	Rivers
6711	RV/0015	RV/0015/S/1	Eddy Medical Centre		4, Ndoki Lane, Behind 53 Market Road, Rumuomasi, P/H	Obio/Akpor	Rivers
6712	RV/0015	RV/0015/S/2	Eddy Medical Centre		4, Ndoki Lane, Behind 53 Market Road, Rumuomasi, P/H	Obio/Akpor	Rivers
6713	RV/0016	RV/0016/S/5	Queens Clinic		38 Rumuolumini Rd Wimpy Junction	Obio/Akpor	Rivers
6714	RV/0016	RV/0016/S/4	Queens Clinic		38 Rumuolumini Rd Wimpy Junction	Obio/Akpor	Rivers
6715	RV/0016	RV/0016/S/2	Queens Clinic		38 Rumuolumini Rd Wimpy Junction	Obio/Akpor	Rivers
6716	RV/0016	RV/0016/S/1	Queens Clinic		38 Rumuolumini Rd Wimpy Junction	Obio/Akpor	Rivers
6717	RV/0016	RV/0016/S/3	Queens Clinic		38 Rumuolumini Rd Wimpy Junction	Obio/Akpor	Rivers
6718	RV/0016	RV/0016/S/6	Queens Clinic		38 Rumuolumini Rd Wimpy Junction	Obio/Akpor	Rivers
6719	RV/0019	RV/0019/S/3	Siya Medical Centre		112, Old Aba Road, Rumuobiakani, Port Harcourt	Obio/Akpor	Rivers
6720	RV/0019	RV/0019/S/1	Siya Medical Centre		112, Old Aba Road, Rumuobiakani, Port Harcourt	Obio/Akpor	Rivers
6721	RV/0019	RV/0019/S/10	Siya Medical Centre		112, Old Aba Road, Rumuobiakani, Port Harcourt	Obio/Akpor	Rivers
6722	RV/0019	RV/0019/S/5	Siya Medical Centre		112, Old Aba Road, Rumuobiakani, Port Harcourt	Obio/Akpor	Rivers
6723	RV/0024	RV/0024/S/1	Ebony Hospital Ltd		9, Orazi Road, Rumulia	Obio/Akpor	Rivers
6724	RV/0024	RV/0024/S/3	Ebony Hospital Ltd		9, Orazi Road, Rumulia	Obio/Akpor	Rivers
6725	RV/0024	RV/0024/S/4	Ebony Hospital Ltd		9, Orazi Road, Rumulia	Obio/Akpor	Rivers
6726	RV/0024	RV/0024/S/5	Ebony Hospital Ltd		9, Orazi Road, Rumulia	Obio/Akpor	Rivers
6727	RV/0024	RV/0024/S/7	Ebony Hospital Ltd		9, Orazi Road, Rumulia	Obio/Akpor	Rivers
6728	RV/0024	RV/0024/S/10	Ebony Hospital Ltd		9, Orazi Road, Rumulia	Obio/Akpor	Rivers
6729	RV/0027	RV/0027/S/8	Lancet Dental Clinic	Dental	208B, Aba Express	Obio/Akpor	Rivers
6730	RV/0037	RV/0037/S/3	Fanny Royal Hospital		4, Bimkol Crescent G.R.A. Phase III	Obio/Akpor	Rivers
6731	RV/0037	RV/0037/S/5	Fanny Royal Hospital		4, Bimkol Crescent G.R.A. Phase III	Obio/Akpor	Rivers
6732	RV/0037	RV/0037/S/7	Fanny Royal Hospital		4, Bimkol Crescent G.R.A. Phase III	Obio/Akpor	Rivers
6733	RV/0038	RV/0038/S/7	Aggrey Clinic Ltd.		107 Aggrey Road Rivers	Port Harcourt	Rivers
6734	RV/0038	RV/0038/S/5	Aggrey Clinic Ltd.		107 Aggrey Road Rivers	Port Harcourt	Rivers
6735	RV/0038	RV/0038/S/6	Aggrey Clinic Ltd.		107 Aggrey Road Rivers	Port Harcourt	Rivers
6736	RV/0038	RV/0038/S/8	Aggrey Clinic Ltd.		107 Aggrey Road Rivers	Port Harcourt	Rivers
6737	RV/0038	RV/0038/S/1	Aggrey Clinic Ltd.		107 Aggrey Road Rivers	Port Harcourt	Rivers
6738	RV/0038	RV/0038/S/3	Aggrey Clinic Ltd.		107 Aggrey Road Rivers	Port Harcourt	Rivers
6739	RV/0038	RV/0038/S/2	Aggrey Clinic Ltd.		107 Aggrey Road Rivers	Port Harcourt	Rivers
6740	RV/0039	RV/0039/S/1	Odessa Med. Services Ltd.		5 Royal Paragon Hotel Avenue, Iwofe Road, Rivers	Port Harcourt	Rivers
6741	RV/0039	RV/0039/S/3	Odessa Med. Services Ltd.		5 Royal Paragon Hotel Avenue, Iwofe Road, Rivers	Port Harcourt	Rivers
6742	RV/0039	RV/0039/S/5	Odessa Med. Services Ltd.		5 Royal Paragon Hotel Avenue, Iwofe Road, Rivers	Port Harcourt	Rivers
6743	RV/0040	RV/0040/S/12	Meridian Hospitals		21, Ibokwe Street, D.Line	Port Harcourt	Rivers
6744	RV/0040	RV/0040/S/3	Meridian Hospitals		21, Ibokwe Street, D.Line	Port Harcourt	Rivers
6745	RV/0040	RV/0040/S/10	Meridian Hospitals		21, Ibokwe Street, D.Line	Port Harcourt	Rivers
6746	RV/0040	RV/0040/S/6	Meridian Hospitals		21, Ibokwe Street, D.Line	Port Harcourt	Rivers
6747	RV/0040	RV/0040/S/2	Meridian Hospitals		21, Ibokwe Street, D.Line	Port Harcourt	Rivers
6748	RV/0040	RV/0040/S/7	Meridian Hospitals		21, Ibokwe Street, D.Line	Port Harcourt	Rivers
6749	RV/0040	RV/0040/S/1	Meridian Hospitals		21, Ibokwe Street, D.Line	Port Harcourt	Rivers
6750	RV/0040	RV/0040/S/5	Meridian Hospitals		21, Ibokwe Street, D.Line	Port Harcourt	Rivers
6751	RV/0042	RV/0042/S/5	Ponyx Clinic		2, Chuku Olunda Street, Off Anon Lodge, by Waterline Junction, Rumukalagbor	Obio/Akpor	Rivers
6752	RV/0042	RV/0042/S/7	Ponyx Clinic		2, Chuku Olunda Street, Off Anon Lodge, by Waterline Junction, Rumukalagbor	Obio/Akpor	Rivers

6753	RV/0042	RV/0042/S/8	Ponyx Clinic		2, Chuku Olunda Street, Off Anon Lodge, by Waterline Junction, Rumukalagbor	Obio/Akpor	Rivers
6754	RV/0043	RV/0043/S/3	Hossana Clinic/ Maternity		33, Abel Jumbo St. Mile 2, P/Harcourt	Port Harcourt	Rivers
6755	RV/0043	RV/0043/S/2	Hossana Clinic/ Maternity		33, Abel Jumbo St. Mile 2, P/Harcourt	Port Harcourt	Rivers
6756	RV/0043	RV/0043/S/1	Hossana Clinic/ Maternity		33, Abel Jumbo St. Mile 2, P/Harcourt	Port Harcourt	Rivers
6757	RV/0043	RV/0043/S/6	Hossana Clinic/ Maternity		33, Abel Jumbo St. Mile 2, P/Harcourt	Port Harcourt	Rivers
6758	RV/0043	RV/0043/S/5	Hossana Clinic/ Maternity		33, Abel Jumbo St. Mile 2, P/Harcourt	Port Harcourt	Rivers
6759	RV/0044	RV/0044/S/7	Destiny Hospital & Maternity		38, Ihediohama Diobu Mile 2, P/Harcourt	Port Harcourt	Rivers
6760	RV/0044	RV/0044/S/5	Destiny Hospital & Maternity		38, Ihediohama Diobu Mile 2, P/Harcourt	Port Harcourt	Rivers
6761	RV/0044	RV/0044/S/1	Destiny Hospital & Maternity		38, Ihediohama Diobu Mile 2, P/Harcourt	Port Harcourt	Rivers
6762	RV/0044	RV/0044/S/3	Destiny Hospital & Maternity		38, Ihediohama Diobu Mile 2, P/Harcourt	Port Harcourt	Rivers
6763	RV/0044	RV/0044/S/6	Destiny Hospital & Maternity		38, Ihediohama Diobu Mile 2, P/Harcourt	Port Harcourt	Rivers
6764	RV/0044	RV/0044/S/2	Destiny Hospital & Maternity		38, Ihediohama Diobu Mile 2, P/Harcourt	Port Harcourt	Rivers
6765	RV/0045	RV/0045/S/3	Calvary Clinic		7a, Haardy U. Street, Orazi, P/Harcourt	Port Harcourt	Rivers
6766	RV/0045	RV/0045/S/6	Calvary Clinic		7a, Haardy U. Street, Orazi, P/Harcourt	Port Harcourt	Rivers
6767	RV/0045	RV/0045/S/5	Calvary Clinic		7a, Haardy U. Street, Orazi, P/Harcourt	Port Harcourt	Rivers
6768	RV/0045	RV/0045/S/4	Calvary Clinic		7a, Haardy U. Street, Orazi, P/Harcourt	Port Harcourt	Rivers
6769	RV/0045	RV/0045/S/1	Calvary Clinic		7a, Haardy U. Street, Orazi, P/Harcourt	Port Harcourt	Rivers
6770	RV/0047	RV/0047/S/7	University Of Port Harcourt Teaching Hospital		Port Harcourt	Port Harcourt	Rivers
6771	RV/0047	RV/0047/S/5	University Of Port Harcourt Teaching Hospital		Port Harcourt	Port Harcourt	Rivers
6772	RV/0047	RV/0047/S/3	University Of Port Harcourt Teaching Hospital		Port Harcourt	Port Harcourt	Rivers
6773	RV/0047	RV/0047/S/4	University Of Port Harcourt Teaching Hospital		Port Harcourt	Port Harcourt	Rivers
6774	RV/0047	RV/0047/S/1	University Of Port Harcourt Teaching Hospital		Port Harcourt	Port Harcourt	Rivers
6775	RV/0048	RV/0048/S/3	Braithwaite Memorial Hospital		Port Harcourt	Port Harcourt	Rivers
6776	RV/0048	RV/0048/S/6	Braithwaite Memorial Hospital		Port Harcourt	Port Harcourt	Rivers
6777	RV/0048	RV/0048/S/5	Braithwaite Memorial Hospital		Port Harcourt	Port Harcourt	Rivers
6778	RV/0048	RV/0048/S/11	Braithwaite Memorial Hospital		Port Harcourt	Port Harcourt	Rivers
6779	RV/0048	RV/0048/S/4	Braithwaite Memorial Hospital		Port Harcourt	Port Harcourt	Rivers
6780	RV/0048	RV/0048/S/7	Braithwaite Memorial Hospital		Port Harcourt	Port Harcourt	Rivers
6781	RV/0049	RV/0049/S/5	Ashford And Patrice Clinic Ltd.		38 Manila Pepple D. Line, Ph, Rivers State.	Port Harcourt	Rivers
6782	RV/0049	RV/0049/S/4	Ashford And Patrice Clinic Ltd.		38 Manila Pepple D. Line, Ph, Rivers State.	Port Harcourt	Rivers
6783	RV/0049	RV/0049/S/2	Ashford And Patrice Clinic Ltd.		38 Manila Pepple D. Line, Ph, Rivers State.	Port Harcourt	Rivers
6784	RV/0049	RV/0049/S/6	Ashford And Patrice Clinic Ltd.		38 Manila Pepple D. Line, Ph, Rivers State.	Port Harcourt	Rivers
6785	RV/0049	RV/0049/S/10	Ashford And Patrice Clinic Ltd.		38 Manila Pepple D. Line, Ph, Rivers State.	Port Harcourt	Rivers
6786	RV/0049	RV/0049/S/3	Ashford And Patrice Clinic Ltd.		38 Manila Pepple D. Line, Ph, Rivers State.	Port Harcourt	Rivers
6787	RV/0049	RV/0049/S/1	Ashford And Patrice Clinic Ltd.		38 Manila Pepple D. Line, Ph, Rivers State.	Port Harcourt	Rivers
6788	RV/0050	RV/0050/S/3	Providence Clinic & Maternity		11 Railway Close, D-Line Ph.	Port Harcourt	Rivers
6789	RV/0050	RV/0050/S/6	Providence Clinic & Maternity		11 Railway Close, D-Line Ph.	Port Harcourt	Rivers
6790	RV/0050	RV/0050/S/7	Providence Clinic & Maternity		11 Railway Close, D-Line Ph.	Port Harcourt	Rivers
6791	RV/0050	RV/0050/S/5	Providence Clinic & Maternity		11 Railway Close, D-Line Ph.	Port Harcourt	Rivers
6792	RV/0050	RV/0050/S/1	Providence Clinic & Maternity		11 Railway Close, D-Line Ph.	Port Harcourt	Rivers
6793	RV/0050	RV/0050/S/2	Providence Clinic & Maternity		11 Railway Close, D-Line Ph.	Port Harcourt	Rivers

6794	RV/0051	RV/0051/S/1	Vita Medical Centre		27, Nsukka Street, Mile1 Diobu,	Port Harcourt	Rivers
6795	RV/0051	RV/0051/S/5	Vita Medical Centre		27, Nsukka Street, Mile1 Diobu,	Port Harcourt	Rivers
6796	RV/0051	RV/0051/S/3	Vita Medical Centre		27, Nsukka Street, Mile1 Diobu,	Port Harcourt	Rivers
6797	RV/0051	RV/0051/S/2	Vita Medical Centre		27, Nsukka Street, Mile1 Diobu,	Port Harcourt	Rivers
6798	RV/0052	RV/0052/S/3	Seaside Specialist Surgery		89, VictorialStreet	Port Harcourt	Rivers
6799	RV/0052	RV/0052/S/1	Seaside Specialist Surgery		89, VictorialStreet	Port Harcourt	Rivers
6800	RV/0052	RV/0052/S/7	Seaside Specialist Surgery		89, VictorialStreet	Port Harcourt	Rivers
6801	RV/0054	RV/0054/S/6	Nobsams Clinics		44 Trans Amadi Industrial Layout, Oginigba	Port Harcourt	Rivers
6802	RV/0054	RV/0054/S/2	Nobsams Clinics		44 Trans Amadi Industrial Layout, Oginigba	Port Harcourt	Rivers
6803	RV/0054	RV/0054/S/4	Nobsams Clinics		44 Trans Amadi Industrial Layout, Oginigba	Port Harcourt	Rivers
6804	RV/0054	RV/0054/S/5	Nobsams Clinics		44 Trans Amadi Industrial Layout, Oginigba	Port Harcourt	Rivers
6805	RV/0054	RV/0054/S/3	Nobsams Clinics		44 Trans Amadi Industrial Layout, Oginigba	Port Harcourt	Rivers
6806	RV/0054	RV/0054/S/1	Nobsams Clinics		44 Trans Amadi Industrial Layout, Oginigba	Port Harcourt	Rivers
6807	RV/0062	RV/0062/S/1	Meridian Hospitals	Surgery, O&G, Medicine, Paediatrics, Orthopaedics, Radiology, ENT,Laboratory	128, Ikwere Road, P/Harcourt.	Port Harcourt	Rivers
6808	RV/0062	RV/0062/S/3	Meridian Hospitals	Surgery, O&G, Medicine, Paediatrics, Orthopaedics, Radiology, ENT,Laboratory	128, Ikwere Road, P/Harcourt.	Port Harcourt	Rivers
6809	RV/0062	RV/0062/S/2	Meridian Hospitals	Surgery, O&G, Medicine, Paediatrics, Orthopaedics, Radiology, ENT,Laboratory	128, Ikwere Road, P/Harcourt.	Port Harcourt	Rivers
6810	RV/0062	RV/0062/S/10	Meridian Hospitals	Surgery, O&G, Medicine, Paediatrics, Orthopaedics, Radiology, ENT,Laboratory	128, Ikwere Road, P/Harcourt.	Port Harcourt	Rivers
6811	RV/0062	RV/0062/S/12	Meridian Hospitals	Surgery, O&G, Medicine, Paediatrics, Orthopaedics, Radiology, ENT,Laboratory	128, Ikwere Road, P/Harcourt.	Port Harcourt	Rivers
6812	RV/0062	RV/0062/S/6	Meridian Hospitals	Surgery, O&G, Medicine, Paediatrics, Orthopaedics, Radiology, ENT,Laboratory	128, Ikwere Road, P/Harcourt.	Port Harcourt	Rivers
6813	RV/0062	RV/0062/S/7	Meridian Hospitals	Surgery, O&G, Medicine, Paediatrics, Orthopaedics, Radiology, ENT,Laboratory	128, Ikwere Road, P/Harcourt.	Port Harcourt	Rivers
6814	RV/0062	RV/0062/S/5	Meridian Hospitals	Surgery, O&G, Medicine, Paediatrics, Orthopaedics, Radiology, ENT,Laboratory	128, Ikwere Road, P/Harcourt.	Port Harcourt	Rivers
6815	RV/0065	RV/0065/S/1	Calvary Eye Care	Optometric	421, Trans-Amadi Road, Opp. Pasbod Breweries Oginigba	Port Harcourt	Rivers
6816	RV/0066	RV/0066/S/15	Korene Eye Clinic Nig. Ltd	Ophthalmology	28, Old Aba Road	Obio/Akpor	Rivers
6817	RV/0069	RV/0069/S/4	Valorz Pharmacy	Pharmacy	76, Old Aba Rd. Rumuomasi.	Obio/Akpor	Rivers
6818	RV/0079	RV/0079/S/5	Christ The King Hospital		145 Old Aba/P.H Road, Mbano Camp, Oyigbo, Rivers State	Port Harcourt	Rivers
6819	RV/0080	RV/0080/S/5	St. Mary's Hospital & Maternity		5, Estate Road, Woji	Obio/Akpor	Rivers
6820	RV/0080	RV/0080/S/6	St. Mary's Hospital & Maternity		5, Estate Road, Woji	Obio/Akpor	Rivers
6821	RV/0080	RV/0080/S/2	St. Mary's Hospital & Maternity		5, Estate Road, Woji	Obio/Akpor	Rivers

6822	RV/0080	RV/0080/S/10	St. Mary's Hospital & Maternity		5, Estate Road, Woji	Obio/Akpor	Rivers
6823	RV/0080	RV/0080/S/1	St. Mary's Hospital & Maternity		5, Estate Road, Woji	Obio/Akpor	Rivers
6824	RV/0080	RV/0080/S/3	St. Mary's Hospital & Maternity		5, Estate Road, Woji	Obio/Akpor	Rivers
6825	RV/0081	RV/0081/S/5	Teme Hospital Ltd		10, New Hospital Lane	Port Harcourt	Rivers
6826	RV/0081	RV/0081/S/7	Teme Hospital Ltd		10, New Hospital Lane	Port Harcourt	Rivers
6827	RV/0081	RV/0081/S/10	Teme Hospital Ltd		10, New Hospital Lane	Port Harcourt	Rivers
6828	RV/0081	RV/0081/S/6	Teme Hospital Ltd		10, New Hospital Lane	Port Harcourt	Rivers
6829	RV/0081	RV/0081/S/2	Teme Hospital Ltd		10, New Hospital Lane	Port Harcourt	Rivers
6830	RV/0081	RV/0081/S/1	Teme Hospital Ltd		10, New Hospital Lane	Port Harcourt	Rivers
6831	RV/0081	RV/0081/S/3	Teme Hospital Ltd		10, New Hospital Lane	Port Harcourt	Rivers
6832	RV/0082	RV/0082/S/1	Triumph Hospital		2, Estate Road Off Aba Road, Rumuogba	Obio/Akpor	Rivers
6833	RV/0082	RV/0082/S/3	Triumph Hospital		2, Estate Road Off Aba Road, Rumuogba	Obio/Akpor	Rivers
6834	RV/0082	RV/0082/S/5	Triumph Hospital		2, Estate Road Off Aba Road, Rumuogba	Obio/Akpor	Rivers
6835	RV/0084	RV/0084/S/3	Kez Clinic		16, Chief Wopara Street, Rumuomasi	Obio/Akpor	Rivers
6836	RV/0084	RV/0084/S/2	Kez Clinic		16, Chief Wopara Street, Rumuomasi	Obio/Akpor	Rivers
6837	RV/0084	RV/0084/S/10	Kez Clinic		16, Chief Wopara Street, Rumuomasi	Obio/Akpor	Rivers
6838	RV/0084	RV/0084/S/1	Kez Clinic		16, Chief Wopara Street, Rumuomasi	Obio/Akpor	Rivers
6839	RV/0084	RV/0084/S/5	Kez Clinic		16, Chief Wopara Street, Rumuomasi	Obio/Akpor	Rivers
6840	RV/0085	RV/0085/S/5	Empire Specialist Clinic & Trauma		19, Echinwo, Elekahia	Obio/Akpor	Rivers
6841	RV/0085	RV/0085/S/10	Empire Specialist Clinic & Trauma		19, Echinwo, Elekahia	Obio/Akpor	Rivers
6842	RV/0085	RV/0085/S/2	Empire Specialist Clinic & Trauma		19, Echinwo, Elekahia	Obio/Akpor	Rivers
6843	RV/0085	RV/0085/S/3	Empire Specialist Clinic & Trauma		19, Echinwo, Elekahia	Obio/Akpor	Rivers
6844	RV/0085	RV/0085/S/1	Empire Specialist Clinic & Trauma		19, Echinwo, Elekahia	Obio/Akpor	Rivers
6845	RV/0086	RV/0086/S/5	Alma Hospital		15, Wani Street, Oroworukwo	Port Harcourt	Rivers
6846	RV/0088	RV/0088/S/1	First Rivers Hospital		250a Ph/Aba Expressway	Obio/Akpor	Rivers
6847	RV/0088	RV/0088/S/3	First Rivers Hospital		250a Ph/Aba Expressway	Obio/Akpor	Rivers
6848	RV/0088	RV/0088/S/2	First Rivers Hospital		250a Ph/Aba Expressway	Obio/Akpor	Rivers
6849	RV/0088	RV/0088/S/6	First Rivers Hospital		250a Ph/Aba Expressway	Obio/Akpor	Rivers
6850	RV/0088	RV/0088/S/10	First Rivers Hospital		250a Ph/Aba Expressway	Obio/Akpor	Rivers
6851	RV/0089	RV/0089/S/10	Sonabel Medical Centre		14 Owabie Road, Mile 4 Rumueme	Port Harcourt	Rivers
6852	RV/0089	RV/0089/S/6	Sonabel Medical Centre		14 Owabie Road, Mile 4 Rumueme	Port Harcourt	Rivers
6853	RV/0089	RV/0089/S/3	Sonabel Medical Centre		14 Owabie Road, Mile 4 Rumueme	Port Harcourt	Rivers
6854	RV/0089	RV/0089/S/1	Sonabel Medical Centre		14 Owabie Road, Mile 4 Rumueme	Port Harcourt	Rivers
6855	RV/0089	RV/0089/S/5	Sonabel Medical Centre		14 Owabie Road, Mile 4 Rumueme	Port Harcourt	Rivers
6856	RV/0089	RV/0089/S/4	Sonabel Medical Centre		14 Owabie Road, Mile 4 Rumueme	Port Harcourt	Rivers
6857	RV/0089	RV/0089/S/2	Sonabel Medical Centre		14 Owabie Road, Mile 4 Rumueme	Port Harcourt	Rivers
6858	RV/0089	RV/0089/S/7	Sonabel Medical Centre		14 Owabie Road, Mile 4 Rumueme	Port Harcourt	Rivers
6859	RV/0089	RV/0089/S/15	Sonabel Medical Centre		14 Owabie Road, Mile 4 Rumueme	Port Harcourt	Rivers
6860	RV/0090	RV/0090/S/11	New Mile One Hospital		15 Emenike Street, Mile 1 Diobu	Port Harcourt	Rivers
6861	RV/0090	RV/0090/S/2	New Mile One Hospital		15 Emenike Street, Mile 1 Diobu	Port Harcourt	Rivers
6862	RV/0090	RV/0090/S/5	New Mile One Hospital		15 Emenike Street, Mile 1 Diobu	Port Harcourt	Rivers
6863	RV/0090	RV/0090/S/1	New Mile One Hospital		15 Emenike Street, Mile 1 Diobu	Port Harcourt	Rivers
6864	RV/0090	RV/0090/S/3	New Mile One Hospital		15 Emenike Street, Mile 1 Diobu	Port Harcourt	Rivers
6865	RV/0090	RV/0090/S/15	New Mile One Hospital		15 Emenike Street, Mile 1 Diobu	Port Harcourt	Rivers
6866	RV/0090	RV/0090/S/6	New Mile One Hospital		15 Emenike Street, Mile 1 Diobu	Port Harcourt	Rivers
6867	RV/0090	RV/0090/S/7	New Mile One Hospital		15 Emenike Street, Mile 1 Diobu	Port Harcourt	Rivers
6868	RV/0090	RV/0090/S/4	New Mile One Hospital		15 Emenike Street, Mile 1 Diobu	Port Harcourt	Rivers
6869	RV/0091	RV/0091/S/5	Valentine Hospitals		Plot B2, 0pp. 20 Forces Av. Old Gra	Port Harcourt	Rivers
6870	RV/0091	RV/0091/S/4	Valentine Hospitals		Plot B2, 0pp. 20 Forces Av. Old Gra	Port Harcourt	Rivers
6871	RV/0092	RV/0092/S/4	Rivon Clinic		Aba/Ph Expressway, Genesis Junction	Obio/Akpor	Rivers
6872	RV/0092	RV/0092/S/10	Rivon Clinic		Aba/Ph Expressway, Genesis Junction	Obio/Akpor	Rivers

6873	RV/0092	RV/0092/S/1	Rivon Clinic		Aba/Ph Expressway, Genesis Junction	Obio/Akpor	Rivers
6874	RV/0092	RV/0092/S/3	Rivon Clinic		Aba/Ph Expressway, Genesis Junction	Obio/Akpor	Rivers
6875	RV/0092	RV/0092/S/5	Rivon Clinic		Aba/Ph Expressway, Genesis Junction	Obio/Akpor	Rivers
6876	RV/0092	RV/0092/S/6	Rivon Clinic		Aba/Ph Expressway, Genesis Junction	Obio/Akpor	Rivers
6877	RV/0092	RV/0092/S/2	Rivon Clinic		Aba/Ph Expressway, Genesis Junction	Obio/Akpor	Rivers
6878	RV/0092	RV/0092/S/7	Rivon Clinic		Aba/Ph Expressway, Genesis Junction	Obio/Akpor	Rivers
6879	RV/0092	RV/0092/S/11	Rivon Clinic		Aba/Ph Expressway, Genesis Junction	Obio/Akpor	Rivers
6880	RV/0093	RV/0093/S/4	St. Catherines Specialist Clinic		17b Ahiamakara Road, By Nwaya Junction, Trans-Amadi	Port Harcourt	Rivers
6881	RV/0093	RV/0093/S/5	St. Catherines Specialist Clinic		17b Ahiamakara Road, By Nwaya Junction, Trans-Amadi	Port Harcourt	Rivers
6882	RV/0093	RV/0093/S/6	St. Catherines Specialist Clinic		17b Ahiamakara Road, By Nwaya Junction, Trans-Amadi	Port Harcourt	Rivers
6883	RV/0093	RV/0093/S/2	St. Catherines Specialist Clinic		17b Ahiamakara Road, By Nwaya Junction, Trans-Amadi	Port Harcourt	Rivers
6884	RV/0093	RV/0093/S/1	St. Catherines Specialist Clinic		17b Ahiamakara Road, By Nwaya Junction, Trans-Amadi	Port Harcourt	Rivers
6885	RV/0093	RV/0093/S/3	St. Catherines Specialist Clinic		17b Ahiamakara Road, By Nwaya Junction, Trans-Amadi	Port Harcourt	Rivers
6886	RV/0094	RV/0094/S/1	Pristine Med. Consultant		7 Nwviuke Rd., Off 125 Okporo Rd., Rumuogba	Obio/Akpor	Rivers
6887	RV/0094	RV/0094/S/2	Pristine Med. Consultant		7 Nwviuke Rd., Off 125 Okporo Rd., Rumuogba	Obio/Akpor	Rivers
6888	RV/0094	RV/0094/S/5	Pristine Med. Consultant		7 Nwviuke Rd., Off 125 Okporo Rd., Rumuogba	Obio/Akpor	Rivers
6889	RV/0094	RV/0094/S/3	Pristine Med. Consultant		7 Nwviuke Rd., Off 125 Okporo Rd., Rumuogba	Obio/Akpor	Rivers
6890	RV/0095	RV/0095/S/5	Sapiens Clinic		2a Mini Ezekwu Street, Off Okporo Rd., Rumuogba	Obio/Akpor	Rivers
6891	RV/0095	RV/0095/S/6	Sapiens Clinic		2a Mini Ezekwu Street, Off Okporo Rd., Rumuogba	Obio/Akpor	Rivers
6892	RV/0095	RV/0095/S/1	Sapiens Clinic		2a Mini Ezekwu Street, Off Okporo Rd., Rumuogba	Obio/Akpor	Rivers
6893	RV/0095	RV/0095/S/2	Sapiens Clinic		2a Mini Ezekwu Street, Off Okporo Rd., Rumuogba	Obio/Akpor	Rivers
6894	RV/0095	RV/0095/S/3	Sapiens Clinic		2a Mini Ezekwu Street, Off Okporo Rd., Rumuogba	Obio/Akpor	Rivers
6895	RV/0096	RV/0096/S/2	St. Patrick Hospital		3 Ogbunabali Street	Port Harcourt	Rivers
6896	RV/0096	RV/0096/S/5	St. Patrick Hospital		3 Ogbunabali Street	Port Harcourt	Rivers
6897	RV/0096	RV/0096/S/1	St. Patrick Hospital		3 Ogbunabali Street	Port Harcourt	Rivers
6898	RV/0096	RV/0096/S/6	St. Patrick Hospital		3 Ogbunabali Street	Port Harcourt	Rivers
6899	RV/0096	RV/0096/S/15	St. Patrick Hospital		3 Ogbunabali Street	Port Harcourt	Rivers
6900	RV/0096	RV/0096/S/3	St. Patrick Hospital		3 Ogbunabali Street	Port Harcourt	Rivers
6901	RV/0096	RV/0096/S/11	St. Patrick Hospital		3 Ogbunabali Street	Port Harcourt	Rivers
6902	RV/0096	RV/0096/S/7	St. Patrick Hospital		3 Ogbunabali Street	Port Harcourt	Rivers
6903	RV/0097	RV/0097/S/1	Alphonso Hospital		Amadi Bus Stop, Along Refinery Rd., Elelenwon	Obio/Akpor	Rivers
6904	RV/0097	RV/0097/S/3	Alphonso Hospital		Amadi Bus Stop, Along Refinery Rd., Elelenwon	Obio/Akpor	Rivers
6905	RV/0097	RV/0097/S/5	Alphonso Hospital		Amadi Bus Stop, Along Refinery Rd., Elelenwon	Obio/Akpor	Rivers
6906	RV/0097	RV/0097/S/2	Alphonso Hospital		Amadi Bus Stop, Along Refinery Rd., Elelenwon	Obio/Akpor	Rivers
6907	RV/0097	RV/0097/S/7	Alphonso Hospital		Amadi Bus Stop, Along Refinery Rd., Elelenwon	Obio/Akpor	Rivers
6908	RV/0097	RV/0097/S/6	Alphonso Hospital		Amadi Bus Stop, Along Refinery Rd., Elelenwon	Obio/Akpor	Rivers
6909	RV/0099	RV/0099/S/2	Morning Star Hospital		19 Isiokpo Street, D/Line	Port Harcourt	Rivers
6910	RV/0099	RV/0099/S/5	Morning Star Hospital		19 Isiokpo Street, D/Line	Port Harcourt	Rivers
6911	RV/0099	RV/0099/S/6	Morning Star Hospital		19 Isiokpo Street, D/Line	Port Harcourt	Rivers
6912	RV/0099	RV/0099/S/3	Morning Star Hospital		19 Isiokpo Street, D/Line	Port Harcourt	Rivers
6913	RV/0099	RV/0099/S/1	Morning Star Hospital		19 Isiokpo Street, D/Line	Port Harcourt	Rivers
6914	RV/0099	RV/0099/S/4	Morning Star Hospital		19 Isiokpo Street, D/Line	Port Harcourt	Rivers
6915	RV/0101	RV/0101/S/4	Woji Cottage Hospital		Accon Road, Woji Town, Rivers State	Port Harcourt	Rivers

6916	RV/0101	RV/0101/S/1	Woji Cottage Hospital		Accon Road, Woji Town, Rivers State	Port Harcourt	Rivers
6917	RV/0101	RV/0101/S/3	Woji Cottage Hospital		Accon Road, Woji Town, Rivers State	Port Harcourt	Rivers
6918	RV/0101	RV/0101/S/6	Woji Cottage Hospital		Accon Road, Woji Town, Rivers State	Port Harcourt	Rivers
6919	RV/0101	RV/0101/S/2	Woji Cottage Hospital		Accon Road, Woji Town, Rivers State	Port Harcourt	Rivers
6920	RV/0102	RV/0102/S/4	Palmers Hospital & Mat. Home		5/7 Agip Road Rumueme	Obio/Akpor	Rivers
6921	RV/0102	RV/0102/S/2	Palmers Hospital & Mat. Home		5/7 Agip Road Rumueme	Obio/Akpor	Rivers
6922	RV/0102	RV/0102/S/7	Palmers Hospital & Mat. Home		5/7 Agip Road Rumueme	Obio/Akpor	Rivers
6923	RV/0102	RV/0102/S/6	Palmers Hospital & Mat. Home		5/7 Agip Road Rumueme	Obio/Akpor	Rivers
6924	RV/0102	RV/0102/S/5	Palmers Hospital & Mat. Home		5/7 Agip Road Rumueme	Obio/Akpor	Rivers
6925	RV/0102	RV/0102/S/3	Palmers Hospital & Mat. Home		5/7 Agip Road Rumueme	Obio/Akpor	Rivers
6926	RV/0102	RV/0102/S/10	Palmers Hospital & Mat. Home		5/7 Agip Road Rumueme	Obio/Akpor	Rivers
6927	RV/0102	RV/0102/S/1	Palmers Hospital & Mat. Home		5/7 Agip Road Rumueme	Obio/Akpor	Rivers
6928	RV/0103	RV/0103/S/3	Cresthill Medical Centre		2a Uyo Street Rumueomasi	Obio/Akpor	Rivers
6929	RV/0103	RV/0103/S/5	Cresthill Medical Centre		2a Uyo Street Rumueomasi	Obio/Akpor	Rivers
6930	RV/0103	RV/0103/S/1	Cresthill Medical Centre		2a Uyo Street Rumueomasi	Obio/Akpor	Rivers
6931	RV/0104	RV/0104/S/6	St. Martins Hospital		21 Stadium Road Port Harcourt	Obio/Akpor	Rivers
6932	RV/0104	RV/0104/S/10	St. Martins Hospital		21 Stadium Road Port Harcourt	Obio/Akpor	Rivers
6933	RV/0104	RV/0104/S/2	St. Martins Hospital		21 Stadium Road Port Harcourt	Obio/Akpor	Rivers
6934	RV/0104	RV/0104/S/7	St. Martins Hospital		21 Stadium Road Port Harcourt	Obio/Akpor	Rivers
6935	RV/0104	RV/0104/S/4	St. Martins Hospital		21 Stadium Road Port Harcourt	Obio/Akpor	Rivers
6936	RV/0104	RV/0104/S/3	St. Martins Hospital		21 Stadium Road Port Harcourt	Obio/Akpor	Rivers
6937	RV/0104	RV/0104/S/5	St. Martins Hospital		21 Stadium Road Port Harcourt	Obio/Akpor	Rivers
6938	RV/0104	RV/0104/S/1	St. Martins Hospital		21 Stadium Road Port Harcourt	Obio/Akpor	Rivers
6939	RV/0106	RV/0106/S/7	Halten Clinic		11, Agudama Street, D/Line	Port Harcourt	Rivers
6940	RV/0106	RV/0106/S/4	Halten Clinic		11, Agudama Street, D/Line	Port Harcourt	Rivers
6941	RV/0106	RV/0106/S/5	Halten Clinic		11, Agudama Street, D/Line	Port Harcourt	Rivers
6942	RV/0106	RV/0106/S/6	Halten Clinic		11, Agudama Street, D/Line	Port Harcourt	Rivers
6943	RV/0106	RV/0106/S/2	Halten Clinic		11, Agudama Street, D/Line	Port Harcourt	Rivers
6944	RV/0106	RV/0106/S/1	Halten Clinic		11, Agudama Street, D/Line	Port Harcourt	Rivers
6945	RV/0106	RV/0106/S/3	Halten Clinic		11, Agudama Street, D/Line	Port Harcourt	Rivers
6946	RV/0108	RV/0108/S/2	Carmel Clinic		3, Eleworks Drive, Rumuibekwe	Obio/Akpor	Rivers
6947	RV/0110	RV/0110/S/5	Ansett Diagnostic Hospital Limited		9, New Layout Drive, Oginigba Town, Trans-Amadi Industrial Layout	Port Harcourt	Rivers
6948	RV/0113	RV/0113/S/5	Harley Clinic	O&G, Laboratory	21B, Elizabamorazi Road	Port Harcourt	Rivers
6949	RV/0113	RV/0113/S/3	Harley Clinic	O&G, Laboratory	21B, Elizabamorazi Road	Port Harcourt	Rivers
6950	RV/0116	RV/0116/S/15	Opt-vue Eye Care	Ophthalmology	20 Forces Avenue, Old G.R.A.	Port Harcourt	Rivers
6951	RV/0125	RV/0125/S/8	Cimbak Dental Clinic	Dental	15, Ekere street, Rumubiakani	Obio/Akpor	Rivers
6952	RV/0127	RV/0127/S/13	Vonnie Vision	Optometric	Plot 23, Close 8, Elekahia	Port Harcourt	Rivers
6953	RV/0129	RV/0129/S/13	Garet Eye Clinic	Optometric, Ophthalmology	61B, Aba Road Beside Matinique restaurant	Obio/Akpor	Rivers
6954	RV/0129	RV/0129/S/15	Garet Eye Clinic	Optometric, Ophthalmology	61B, Aba Road Beside Matinique restaurant	Obio/Akpor	Rivers
6955	RV/0130	RV/0130/S/5	Image Diagnostics	Radiology, Laboratory	23, Okoroji Street, D/Line	Port Harcourt	Rivers
6956	RV/0130	RV/0130/S/7	Image Diagnostics	Radiology, Laboratory	23, Okoroji Street, D/Line	Port Harcourt	Rivers
6957	RV/0133	RV/0133/S/13	Rambod Optometric	Optometry	29/30, Kaduna Street, D/Line	Port Harcourt	Rivers
6958	RV/0134	RV/0134/S/13	Nice View Eye Clinic	Optometric	222, Ikwere Road, Mile 3, Beside Diamond Bank	Port Harcourt	Rivers
6959	RV/0138	RV/0138/S/4	Mc Pee Pharm. & Mech. Coy. Ltd	Pharmacy	Rumuokita Junction	Obio/Akpor	Rivers
6960	RV/0141	RV/0141/S/4	Kebs Pharm. Ltd	Pharmacy	12, Rumuogba Off Woji Junction.	Obio/Akpor	Rivers

6961	RV/0142	RV/0142/S/4	Chindux Nig. Ltd.	Pharmacy	126, Woji Road, Rumuolu, P/Harcourt	Port Harcourt	Rivers
6962	RV/0144	RV/0144/S/4	Llyods Pharm. Ltd	Pharmacy	27, Agi Road, Mile 4, Rumuemene	Port Harcourt	Rivers
6963	RV/0149	RV/0149/S/4	Juchez Nig. Ltd.	Pharmacy	2, Ilorin Street Opp. Whyte, Shopping Complex Off Hospital R	Port Harcourt	Rivers
6964	RV/0153	RV/0153/S/4	Capino Pharmacy	Pharmacy	3, Rumuokoro Street, Rumuomasi	Obio/Akpor	Rivers
6965	RV/0156	RV/0156/S/4	Olijibomen Nig. Ltd	Pharmacy	204, Aba Road, Rumuola	Obio/Akpor	Rivers
6966	RV/0157	RV/0157/S/4	Bydow Pharmacy	Pharmacy	19, Agudama Street, D/Line	Port Harcourt	Rivers
6967	RV/0166	RV/0166/S/5	Port Harcourt Medical Investigation Centre	Laboratory	15 Ikwere Street, By Emenike Junction Bus Stop	Port Harcourt	Rivers
6968	RV/0167	RV/0167/S/5	Gekon Medical & Dia.Services	Laboratory	2, Nvuike Road, Off Okporo Road, Rumuadara	Port Harcourt	Rivers
6969	RV/0172	RV/0172/S/4	Barata Pharmacy	Pharmacy	416 Ikerre Road	Obio/Akpor	Rivers
6970	RV/0173	RV/0173/S/4	Condios Pharmacy	Pharmacy	141 Onne Road, G.R.A.	Port Harcourt	Rivers
6971	RV/0176	RV/0176/S/4	E.J. Pharmacy Ltd	Pharmacy	26 Hospital Road	Port Harcourt	Rivers
6972	RV/0177	RV/0177/S/4	Gravas Venture Ltd	Pharmacy	46 Woji Road	Obio/Akpor	Rivers
6973	RV/0178	RV/0178/S/4	Sodel Pharmacy & Store Ltd	Pharmacy	23 Market road, rumuomasi	Obio/Akpor	Rivers
6974	RV/0180	RV/0180/S/4	Akoltem Nig. Ltd.,	Pharmacy	193 Aba Road, Rumuola Junction	Obio/Akpor	Rivers
6975	RV/0181	RV/0181/S/4	Ausjone Pharmacy Ltd	Pharmacy	118 Rumuola Road	Obio/Akpor	Rivers
6976	RV/0185	RV/0185/S/4	Wilson Pharmaceutical Co.Nig.	Pharmacy	13 East-West Road, Rumuodumaya	Port Harcourt	Rivers
6977	RV/0196	RV/0196/S/4	Midline pharmacy Nig.	Pharmacy	429, Ikwere Road, mile 5, Rumuepirikom	Obio/Akpor	Rivers
6978	RV/0197	RV/0197/S/4	Sita Pharm. Ltd	Pharmacy	350A, Oluobasanjo Road	Port Harcourt	Rivers
6979	RV/0200	RV/0200/S/4	Kayol Nig. Ltd.	Pharmacy	Amadi-Ama Round Abount	Port Harcourt	Rivers
6980	RV/0202	RV/0202/S/4	Gopez Nig. Ltd.	Pharmacy	3, Lagos Street	Port Harcourt	Rivers
6981	RV/0205	RV/0205/S/4	Tegal Pharmacy	Pharmacy	Rumuokoro Junction	Obio/Akpor	Rivers
6982	RV/0209	RV/0209/S/5	Hopeville Specialist Hospital		Elijbolo-Eneka Road, Off New Airport Road, Eliozu	Obio/Akpor	Rivers
6983	RV/0209	RV/0209/S/11	Hopeville Specialist Hospital		Elijbolo-Eneka Road, Off New Airport Road, Eliozu	Obio/Akpor	Rivers
6984	RV/0209	RV/0209/S/1	Hopeville Specialist Hospital		Elijbolo-Eneka Road, Off New Airport Road, Eliozu	Obio/Akpor	Rivers
6985	RV/0209	RV/0209/S/10	Hopeville Specialist Hospital		Elijbolo-Eneka Road, Off New Airport Road, Eliozu	Obio/Akpor	Rivers
6986	RV/0209	RV/0209/S/7	Hopeville Specialist Hospital		Elijbolo-Eneka Road, Off New Airport Road, Eliozu	Obio/Akpor	Rivers
6987	RV/0210	RV/0210/S/6	Pamo Clinics & Hospital Ltd.		300 Aba Rd., Rumuomasi	Obio/Akpor	Rivers
6988	RV/0210	RV/0210/S/5	Pamo Clinics & Hospital Ltd.		300 Aba Rd., Rumuomasi	Obio/Akpor	Rivers
6989	RV/0210	RV/0210/S/2	Pamo Clinics & Hospital Ltd.		300 Aba Rd., Rumuomasi	Obio/Akpor	Rivers
6990	RV/0210	RV/0210/S/7	Pamo Clinics & Hospital Ltd.		300 Aba Rd., Rumuomasi	Obio/Akpor	Rivers
6991	RV/0210	RV/0210/S/4	Pamo Clinics & Hospital Ltd.		300 Aba Rd., Rumuomasi	Obio/Akpor	Rivers
6992	RV/0210	RV/0210/S/3	Pamo Clinics & Hospital Ltd.		300 Aba Rd., Rumuomasi	Obio/Akpor	Rivers
6993	RV/0210	RV/0210/S/1	Pamo Clinics & Hospital Ltd.		300 Aba Rd., Rumuomasi	Obio/Akpor	Rivers
6994	RV/0211	RV/0211/S/5	Bosom Hospital		6, Akaniwo St, Off Eligbam Rd.	Obio/Akpor	Rivers
6995	RV/0211	RV/0211/S/2	Bosom Hospital		6, Akaniwo St, Off Eligbam Rd.	Obio/Akpor	Rivers
6996	RV/0211	RV/0211/S/4	Bosom Hospital		6, Akaniwo St, Off Eligbam Rd.	Obio/Akpor	Rivers
6997	RV/0211	RV/0211/S/15	Bosom Hospital		6, Akaniwo St, Off Eligbam Rd.	Obio/Akpor	Rivers
6998	RV/0211	RV/0211/S/10	Bosom Hospital		6, Akaniwo St, Off Eligbam Rd.	Obio/Akpor	Rivers
6999	RV/0211	RV/0211/S/6	Bosom Hospital		6, Akaniwo St, Off Eligbam Rd.	Obio/Akpor	Rivers
7000	RV/0211	RV/0211/S/3	Bosom Hospital		6, Akaniwo St, Off Eligbam Rd.	Obio/Akpor	Rivers
7001	RV/0211	RV/0211/S/1	Bosom Hospital		6, Akaniwo St, Off Eligbam Rd.	Obio/Akpor	Rivers
7002	RV/0211	RV/0211/S/11	Bosom Hospital		6, Akaniwo St, Off Eligbam Rd.	Obio/Akpor	Rivers
7003	RV/0212	RV/0212/S/3	Cumi Medical Centre		3, Endless Road, Off Elitor Rd, Woji	Obio/Akpor	Rivers
7004	RV/0212	RV/0212/S/6	Cumi Medical Centre		3, Endless Road, Off Elitor Rd, Woji	Obio/Akpor	Rivers
7005	RV/0212	RV/0212/S/5	Cumi Medical Centre		3, Endless Road, Off Elitor Rd, Woji	Obio/Akpor	Rivers
7006	RV/0212	RV/0212/S/1	Cumi Medical Centre		3, Endless Road, Off Elitor Rd, Woji	Obio/Akpor	Rivers
7007	RV/0212	RV/0212/S/4	Cumi Medical Centre		3, Endless Road, Off Elitor Rd, Woji	Obio/Akpor	Rivers

7008	RV/0213	RV/0213/S/2	Marina Hospital		16 Akwa St, Off 35 Aba Rd, By Savannah Bank	Obio/Akpor	Rivers	
7009	RV/0213	RV/0213/S/1	Marina Hospital		16 Akwa St, Off 35 Aba Rd, By Savannah Bank	Obio/Akpor	Rivers	
7010	RV/0213	RV/0213/S/6	Marina Hospital		16 Akwa St, Off 35 Aba Rd, By Savannah Bank	Obio/Akpor	Rivers	
7011	RV/0213	RV/0213/S/3	Marina Hospital		16 Akwa St, Off 35 Aba Rd, By Savannah Bank	Obio/Akpor	Rivers	
7012	RV/0213	RV/0213/S/5	Marina Hospital		16 Akwa St, Off 35 Aba Rd, By Savannah Bank	Obio/Akpor	Rivers	
7013	RV/0214	RV/0214/S/6	Lifetime Medical Centre		1A Worukwo St, Waterline, off Olusegun Obasanjo Rd.	Port Harcourt	Rivers	
7014	RV/0214	RV/0214/S/3	Lifetime Medical Centre		1A Worukwo St, Waterline, off Olusegun Obasanjo Rd.	Port Harcourt	Rivers	
7015	RV/0214	RV/0214/S/1	Lifetime Medical Centre		1A Worukwo St, Waterline, off Olusegun Obasanjo Rd.	Port Harcourt	Rivers	
7016	RV/0214	RV/0214/S/5	Lifetime Medical Centre		1A Worukwo St, Waterline, off Olusegun Obasanjo Rd.	Port Harcourt	Rivers	
7017	RV/0215	RV/0215/S/6	Immanuel Clinic		4, Nkanwa Lane, off NTA Rd Rumnokuta	Port Harcourt	Rivers	
7018	RV/0215	RV/0215/S/3	Immanuel Clinic		4, Nkanwa Lane, off NTA Rd Rumnokuta	Port Harcourt	Rivers	
7019	RV/0215	RV/0215/S/1	Immanuel Clinic		4, Nkanwa Lane, off NTA Rd Rumnokuta	Port Harcourt	Rivers	
7020	RV/0215	RV/0215/S/2	Immanuel Clinic		4, Nkanwa Lane, off NTA Rd Rumnokuta	Port Harcourt	Rivers	
7021	RV/0215	RV/0215/S/5	Immanuel Clinic		4, Nkanwa Lane, off NTA Rd Rumnokuta	Port Harcourt	Rivers	
7022	RV/0216	RV/0216/S/5	Jeconiah Children's Clinic		4, Okoroji Lane, D/Line	Port Harcourt	Rivers	
7023	RV/0216	RV/0216/S/6	Jeconiah Children's Clinic		4, Okoroji Lane, D/Line	Port Harcourt	Rivers	
7024	RV/0216	RV/0216/S/4	Jeconiah Children's Clinic		4, Okoroji Lane, D/Line	Port Harcourt	Rivers	
7025	RV/0216	RV/0216/S/1	Jeconiah Children's Clinic		4, Okoroji Lane, D/Line	Port Harcourt	Rivers	
7026	RV/0216	RV/0216/S/3	Jeconiah Children's Clinic		4, Okoroji Lane, D/Line	Port Harcourt	Rivers	
7027	RV/0218	RV/0218/S/4	Nigeria Customs Service Med. Centre		Nigeria Customs Service Medical Centre Port Harcourt-1,Rivers State	Port Harcourt	Rivers	
7028	RV/0226	RV/0226/S/3	Castle Clinics		7B Shell Location Road, Rumuadaolu, Off 42 Rumuola Rd., P/H	Obio/Akpor	Rivers	
7029	RV/0226	RV/0226/S/1	Castle Clinics		7B Shell Location Road, Rumuadaolu, Off 42 Rumuola Rd., P/H	Obio/Akpor	Rivers	
7030	RV/0226	RV/0226/S/11	Castle Clinics		7B Shell Location Road, Rumuadaolu, Off 42 Rumuola Rd., P/H	Obio/Akpor	Rivers	
7031	RV/0226	RV/0226/S/5	Castle Clinics		7B Shell Location Road, Rumuadaolu, Off 42 Rumuola Rd., P/H	Obio/Akpor	Rivers	
7032	RV/0226	RV/0226/S/2	Castle Clinics		7B Shell Location Road, Rumuadaolu, Off 42 Rumuola Rd., P/H	Obio/Akpor	Rivers	
7033	RV/0226	RV/0226/S/6	Castle Clinics		7B Shell Location Road, Rumuadaolu, Off 42 Rumuola Rd., P/H	Obio/Akpor	Rivers	
7034	RV/0230	RV/0230/S/5	Health Wise Diagnostic Laboratory	Laboratory	No.5 Chief Wopara Street, Rumuomasi, P/Harcourt	Obio/Akpor	Rivers	
7035	RV/0237	RV/0237/S/7	Cottage Hospital/Comprehensi ve Health Centre		Rivers State Police Command, Rivers State	Port Harcourt	Rivers	
7036	RV/0237	RV/0237/S/1	Cottage Hospital/Comprehensi ve Health Centre		Rivers State Police Command, Rivers State	Port Harcourt	Rivers	
7037	RV/0237	RV/0237/S/5	Cottage Hospital/Comprehensi ve Health Centre		Rivers State Police Command, Rivers State	Port Harcourt	Rivers	
7038	RV/0237	RV/0237/S/4	Cottage Hospital/Comprehensi ve Health Centre		Rivers State Police Command, Rivers State	Port Harcourt	Rivers	
7039	RV/0237	RV/0237/S/8	Cottage Hospital/Comprehensi ve Health Centre		Rivers State Police Command, Rivers State	Port Harcourt	Rivers	
7040	RV/0237	RV/0237/S/3	Cottage Hospital/Comprehensi ve Health Centre		Rivers State Police Command, Rivers State	Port Harcourt	Rivers	
7041	RV/0241	RV/0241/S/1	Precious Life Medical Centre		4 Okorodo street D/line	Port Harcourt	Rivers	
7042	RV/0241	RV/0241/S/3	Precious Life Medical Centre		4 Okorodo street D/line	Port Harcourt	Rivers	
7043	RV/0241	RV/0241/S/5	Precious Life Medical Centre		4 Okorodo street D/line	Port Harcourt	Rivers	

7044	RV/0242	RV/0242/S/2	Sondel Clinic And Maternity		Plot 12 Phase 1 Borikiri Residential layout	Port Harcourt	Rivers
7045	RV/0242	RV/0242/S/5	Sondel Clinic And Maternity		Plot 12 Phase 1 Borikiri Residential layout	Port Harcourt	Rivers
7046	RV/0243	RV/0243/S/6	St Michael's Clinic		4th Street, Block 4 Elekaha Housing Estate	Port Harcourt	Rivers
7047	RV/0243	RV/0243/S/2	St Michael's Clinic		4th Street, Block 4 Elekaha Housing Estate	Port Harcourt	Rivers
7048	RV/0243	RV/0243/S/3	St Michael's Clinic		4th Street, Block 4 Elekaha Housing Estate	Port Harcourt	Rivers
7049	RV/0243	RV/0243/S/1	St Michael's Clinic		4th Street, Block 4 Elekaha Housing Estate	Port Harcourt	Rivers
7050	RV/0243	RV/0243/S/5	St Michael's Clinic		4th Street, Block 4 Elekaha Housing Estate	Port Harcourt	Rivers
7051	RV/0244	RV/0244/S/5	Divine Grace Clinic/Maternity		21 Elijiji Road, Off Woji Road , P/Harcourt	Obio/Akpor	Rivers
7052	RV/0244	RV/0244/S/3	Divine Grace Clinic/Maternity		21 Elijiji Road, Off Woji Road , P/Harcourt	Obio/Akpor	Rivers
7053	RV/0244	RV/0244/S/1	Divine Grace Clinic/Maternity		21 Elijiji Road, Off Woji Road , P/Harcourt	Obio/Akpor	Rivers
7054	RV/0244	RV/0244/S/2	Divine Grace Clinic/Maternity		21 Elijiji Road, Off Woji Road , P/Harcourt	Obio/Akpor	Rivers
7055	RV/0245	RV/0245/S/3	Udo PolyClinics		9/10 Chief Ejim""s Street Rumubiakani P/H	Obio/Akpor	Rivers
7056	RV/0245	RV/0245/S/5	Udo PolyClinics		9/10 Chief Ejim""s Street Rumubiakani P/H	Obio/Akpor	Rivers
7057	RV/0245	RV/0245/S/1	Udo PolyClinics		9/10 Chief Ejim""s Street Rumubiakani P/H	Obio/Akpor	Rivers
7058	RV/0245	RV/0245/S/6	Udo PolyClinics		9/10 Chief Ejim""s Street Rumubiakani P/H	Obio/Akpor	Rivers
7059	RV/0245	RV/0245/S/2	Udo PolyClinics		9/10 Chief Ejim""s Street Rumubiakani P/H	Obio/Akpor	Rivers
7060	RV/0245	RV/0245/S/4	Udo PolyClinics		9/10 Chief Ejim""s Street Rumubiakani P/H	Obio/Akpor	Rivers
7061	RV/0245	RV/0245/S/7	Udo PolyClinics		9/10 Chief Ejim""s Street Rumubiakani P/H	Obio/Akpor	Rivers
7062	RV/0246	RV/0246/S/3	Datta Medical Centre		21A Woji Road, Off Old Aba Road, P/H	Obio/Akpor	Rivers
7063	RV/0246	RV/0246/S/1	Datta Medical Centre		21A Woji Road, Off Old Aba Road, P/H	Obio/Akpor	Rivers
7064	RV/0246	RV/0246/S/5	Datta Medical Centre		21A Woji Road, Off Old Aba Road, P/H	Obio/Akpor	Rivers
7065	RV/0247	RV/0247/S/1	Higgwe Memorial Hospital		644 Ikwerre Road Rumuokoro P/H	Obio/Akpor	Rivers
7066	RV/0247	RV/0247/S/7	Higgwe Memorial Hospital		644 Ikwerre Road Rumuokoro P/H	Obio/Akpor	Rivers
7067	RV/0250	RV/0250/S/5	Eli Johnson Specialist Hospital		13 Bori Road, Rumuibekwe Housing Estate, P/H	Obio/Akpor	Rivers
7068	RV/0251	RV/0251/S/5	Health Fountains Hospital		NTA/Uniport Road , Ozuoba, P/H	Obio/Akpor	Rivers
7069	RV/0251	RV/0251/S/3	Health Fountains Hospital		NTA/Uniport Road , Ozuoba, P/H	Obio/Akpor	Rivers
7070	RV/0251	RV/0251/S/1	Health Fountains Hospital		NTA/Uniport Road , Ozuoba, P/H	Obio/Akpor	Rivers
7071	RV/0253	RV/0253/S/3	Summit Clinic		17 Omoku Street, D/line, P/H	Port Harcourt	Rivers
7072	RV/0253	RV/0253/S/1	Summit Clinic		17 Omoku Street, D/line, P/H	Port Harcourt	Rivers
7073	RV/0253	RV/0253/S/5	Summit Clinic		17 Omoku Street, D/line, P/H	Port Harcourt	Rivers
7074	RV/0254	RV/0254/S/1	Daily Spring Hospital Ltd		11 Rumuigbo Street, Old GRA, P/H	Port Harcourt	Rivers
7075	RV/0254	RV/0254/S/7	Daily Spring Hospital Ltd		11 Rumuigbo Street, Old GRA, P/H	Port Harcourt	Rivers
7076	RV/0254	RV/0254/S/5	Daily Spring Hospital Ltd		11 Rumuigbo Street, Old GRA, P/H	Port Harcourt	Rivers
7077	RV/0254	RV/0254/S/6	Daily Spring Hospital Ltd		11 Rumuigbo Street, Old GRA, P/H	Port Harcourt	Rivers
7078	RV/0254	RV/0254/S/3	Daily Spring Hospital Ltd		11 Rumuigbo Street, Old GRA, P/H	Port Harcourt	Rivers
7079	RV/0254	RV/0254/S/2	Daily Spring Hospital Ltd		11 Rumuigbo Street, Old GRA, P/H	Port Harcourt	Rivers
7080	RV/0255	RV/0255/S/1	Silvia Specialist Clinic/Maternity		6 Circular Road, Phase 2 Presidential Estate	Port Harcourt	Rivers
7081	RV/0255	RV/0255/S/6	Silvia Specialist Clinic/Maternity		6 Circular Road, Phase 2 Presidential Estate	Port Harcourt	Rivers
7082	RV/0255	RV/0255/S/5	Silvia Specialist Clinic/Maternity		6 Circular Road, Phase 2 Presidential Estate	Port Harcourt	Rivers
7083	RV/0255	RV/0255/S/14	Silvia Specialist Clinic/Maternity		6 Circular Road, Phase 2 Presidential Estate	Port Harcourt	Rivers
7084	RV/0255	RV/0255/S/15	Silvia Specialist Clinic/Maternity		6 Circular Road, Phase 2 Presidential Estate	Port Harcourt	Rivers
7085	RV/0255	RV/0255/S/2	Silvia Specialist Clinic/Maternity		6 Circular Road, Phase 2 Presidential Estate	Port Harcourt	Rivers
7086	RV/0255	RV/0255/S/10	Silvia Specialist Clinic/Maternity		6 Circular Road, Phase 2 Presidential Estate	Port Harcourt	Rivers

7087	RV/0256	RV/0256/S/5	El- Bene Hospital		5 El Bene Close, Off Ada George Road Rumuchiorlu Street, by Open Doors Church Junction, Rumueme, Mile 4, P/H	Port Harcourt	Rivers
7088	RV/0256	RV/0256/S/3	El- Bene Hospital		5 El Bene Close, Off Ada George Road Rumuchiorlu Street, by Open Doors Church Junction, Rumueme, Mile 4, P/H	Port Harcourt	Rivers
7089	RV/0256	RV/0256/S/1	El- Bene Hospital		5 El Bene Close, Off Ada George Road Rumuchiorlu Street, by Open Doors Church Junction, Rumueme, Mile 4, P/H	Port Harcourt	Rivers
7090	RV/0257	RV/0257/S/6	Seiyefa Clinic		11 Harold Wilson Drive , Borikiri, P/H	Port Harcourt	Rivers
7091	RV/0257	RV/0257/S/2	Seiyefa Clinic		11 Harold Wilson Drive , Borikiri, P/H	Port Harcourt	Rivers
7092	RV/0257	RV/0257/S/1	Seiyefa Clinic		11 Harold Wilson Drive , Borikiri, P/H	Port Harcourt	Rivers
7093	RV/0257	RV/0257/S/3	Seiyefa Clinic		11 Harold Wilson Drive , Borikiri, P/H	Port Harcourt	Rivers
7094	RV/0257	RV/0257/S/7	Seiyefa Clinic		11 Harold Wilson Drive , Borikiri, P/H	Port Harcourt	Rivers
7095	RV/0257	RV/0257/S/5	Seiyefa Clinic		11 Harold Wilson Drive , Borikiri, P/H	Port Harcourt	Rivers
7096	RV/0258	RV/0258/S/2	Heart Health Medical Services		14 Omoku Street, D/line, P/H	Port Harcourt	Rivers
7097	RV/0260	RV/0260/S/3	Hope Clinics		3/4 Wokogoloma Street, Waterline, P/H	Port Harcourt	Rivers
7098	RV/0260	RV/0260/S/5	Hope Clinics		3/4 Wokogoloma Street, Waterline, P/H	Port Harcourt	Rivers
7099	RV/0260	RV/0260/S/1	Hope Clinics		3/4 Wokogoloma Street, Waterline, P/H	Port Harcourt	Rivers
7100	RV/0261	RV/0261/S/5	Krisany Medical Services		5B Asinobi/Agudama Street, D/line, P/H	Port Harcourt	Rivers
7101	RV/0261	RV/0261/S/3	Krisany Medical Services		5B Asinobi/Agudama Street, D/line, P/H	Port Harcourt	Rivers
7102	RV/0261	RV/0261/S/6	Krisany Medical Services		5B Asinobi/Agudama Street, D/line, P/H	Port Harcourt	Rivers
7103	RV/0261	RV/0261/S/1	Krisany Medical Services		5B Asinobi/Agudama Street, D/line, P/H	Port Harcourt	Rivers
7104	RV/0263	RV/0263/S/5	Atinu Critical Care Hospital		Old Refinery Road , P/H	Obio/Akpor	Rivers
7105	RV/0263	RV/0263/S/1	Atinu Critical Care Hospital		Old Refinery Road , P/H	Obio/Akpor	Rivers
7106	RV/0263	RV/0263/S/3	Atinu Critical Care Hospital		Old Refinery Road , P/H	Obio/Akpor	Rivers
7107	RV/0265	RV/0265/S/1	Promise Land Clinic		4 Bende Street, Rumuomas, i P/H	Obio/Akpor	Rivers
7108	RV/0265	RV/0265/S/3	Promise Land Clinic		4 Bende Street, Rumuomas, i P/H	Obio/Akpor	Rivers
7109	RV/0265	RV/0265/S/5	Promise Land Clinic		4 Bende Street, Rumuomas, i P/H	Obio/Akpor	Rivers
7110	RV/0265	RV/0265/S/2	Promise Land Clinic		4 Bende Street, Rumuomas, i P/H	Obio/Akpor	Rivers
7111	RV/0265	RV/0265/S/6	Promise Land Clinic		4 Bende Street, Rumuomas, i P/H	Obio/Akpor	Rivers
7112	RV/0267	RV/0267/S/3	Salem Hospital		16 Location Road, Oyigbo, P/H	Oyigbo	Rivers
7113	RV/0267	RV/0267/S/1	Salem Hospital		16 Location Road, Oyigbo, P/H	Oyigbo	Rivers
7114	RV/0297	RV/0297/S/11	Body Care Nig. Ltd	Physiotherapy	46 Chief Wopara Street, Rumubiakani, P/H	Obio/Akpor	Rivers
7115	RV/0299	RV/0299/S/10	Riverside Clinics	Internal Medicine,Radiology/USS,Paediatrics, General Surgery,O&G,Laboratory,Orthopaedics	11 Harbour Road, P/H	Port Harcourt	Rivers
7116	RV/0299	RV/0299/S/6	Riverside Clinics	Internal Medicine,Radiology/USS,Paediatrics, General Surgery,O&G,Laboratory,Orthopaedics	11 Harbour Road, P/H	Port Harcourt	Rivers
7117	RV/0299	RV/0299/S/5	Riverside Clinics	Internal Medicine,Radiology/USS,Paediatrics, General Surgery,O&G,Laboratory,Orthopaedics	11 Harbour Road, P/H	Port Harcourt	Rivers

7118	RV/0299	RV/0299/S/3	Riverside Clinics	Internal Medicine,Radiology/USS,Paediatrics, General Surgery,O&G,Laboratory,Orthopaedics	11 Harbour Road, P/H	Port Harcourt	Rivers
7119	RV/0299	RV/0299/S/1	Riverside Clinics	Internal Medicine,Radiology/USS,Paediatrics, General Surgery,O&G,Laboratory,Orthopaedics	11 Harbour Road, P/H	Port Harcourt	Rivers
7120	RV/0299	RV/0299/S/2	Riverside Clinics	Internal Medicine,Radiology/USS,Paediatrics, General Surgery,O&G,Laboratory,Orthopaedics	11 Harbour Road, P/H	Port Harcourt	Rivers
7121	RV/0311	RV/0311/S/8	St Michael Dental Clinic	Dental	52 PH-Aba Expressway, P/H	Port Harcourt	Rivers
7122	RV/0312	RV/0312/S/15	Flomat Eye Care	Opthalmic	169 Aba Road, P/H	Port Harcourt	Rivers
7123	RV/0313	RV/0313/S/15	C&C Optician Ltd	Opthalmology	63B King Perekule Street, GRA Phase II, P/H	Surulere	Lagos
7124	RV/0315	RV/0315/S/13	Niger Optical Services Ltd	Optometry	18 Emenike Street, Mile One, Diobu, Rivers State	Port Harcourt	Rivers
7125	RV/0316	RV/0316/S/13	Niger Optical Services Ltd	Optometry	Plot 8, Aba- P/Harcourt Expr. Road, Rumuibekwe	Obio/Akpor	Rivers
7126	RV/0318	RV/0318/S/5	Savanna Atlantic Diagnostic	Laboratory	5 PH-Aba Expressway, Opp. Occeanic Bank, P/Harcourt	Obio/Akpor	Rivers
7127	RV/0320	RV/0320/S/5	Nina Laboratory Ltd	Laboratory	50, 2nd Floor Woji Road, Rumuolu, P/Harcourt	Obio/Akpor	Rivers
7128	RV/0323	RV/0323/S/5	Raha Medical Laboratory	Laboratory	24 Bende Street, Rumuomasi, P/Harcourt	Port Harcourt	Rivers
7129	RV/0325	RV/0325/S/4	Oliskan Pharm. Chemist	Pharmacy	1 Hospital Road by Station B/Stop, P/Harcourt	Port Harcourt	Rivers
7130	RV/0326	RV/0326/S/4	Medicine Mart	Pharmacy	90, Evo Road, GRA Phase II, P/Harcourt	Port Harcourt	Rivers
7131	RV/0328	RV/0328/S/4	Edyee Pharm. Chemist Ltd	Pharmacy	108 Ahoada Road, Omoku, P/Harcourt, River State	Port Harcourt	Rivers
7132	RV/0329	RV/0329/S/4	Olive Hands Pharmacy & Stores	Pharmacy	10B Omoku Street, off Olu Obasanjo Str., P/Harcourt	Port Harcourt	Rivers
7133	RV/0331	RV/0331/S/4	Luck Pharmacy International	Pharmacy	4A, Ohiamini Street Off Olu-Obasanjo Road, P/Harcourt	Port Harcourt	Rivers
7134	RV/0333	RV/0333/S/4	I & C Pharmacy	Pharmacy	8 Agip Road, Rumueme	Obio/Akpor	Rivers
7135	RV/0334	RV/0334/S/4	Mentor Health Service (Pharm)	Pharmacy	3 Elelenwo Road, off Oil Mill Junction, R/kurushi, P/Harcourt	Obio/Akpor	Rivers
7136	RV/0335	RV/0335/S/4	Ostar Pharmacy Ltd.	Pharmacy	29, Agudama Street, D/Line, P/Harcourt	Port Harcourt	Rivers
7137	RV/0337	RV/0337/S/4	Choola Pharmacy	Pharmacy	18 Rumuodaola, Rumuola, P/Harcourt	Port Harcourt	Rivers
7138	RV/0339	RV/0339/S/5	Saphonyx Pharm. Ltd	Pharmacy	106, Rumuola Road, P/Harcourt	Port Harcourt	Rivers
7139	RV/0340	RV/0340/S/4	Sazel Pharmacy Ltd	Pharmacy	103, NTA-Choba Road, P/Harcourt	Obio/Akpor	Rivers
7140	RV/0341	RV/0341/S/4	Gottgabe Pharm.	Pharmacy	Suite 9 Grumo Plaza, Rumukwurushi, P/Harcourt	Obio/Akpor	Rivers
7141	RV/0342	RV/0342/S/4	Biomade Pharm. Ltd	Pharmacy	38, Igwuruta Road, Rumukwurushi, P/Harcourt	Obio/Akpor	Rivers
7142	RV/0345	RV/0345/S/4	Klavid Pharm. Ltd.	Pharmacy	Suite 022 Parklane Boulevard, P/Harcourt	Port Harcourt	Rivers
7143	RV/0346	RV/0346/S/4	Nalyn Global Pharmacy	Pharmacy	36 Elekahia Road, P/Harcourt	Port Harcourt	Rivers
7144	RV/0347	RV/0347/S/4	Lizendic Pharm & Stores	Pharmacy	259, Okporo Road, East-West Road, off Rumuodara Junction, P/Harcourt	Obio/Akpor	Rivers
7145	RV/0351	RV/0351/S/3	CRI Medi Clinic		Plot 80A Second Avenue, Along Dr. Peter Odili Street, P/Harcourt	Port Harcourt	Rivers
7146	RV/0351	RV/0351/S/6	CRI Medi Clinic		Plot 80A Second Avenue, Along Dr. Peter Odili Street, P/Harcourt	Port Harcourt	Rivers
7147	RV/0351	RV/0351/S/2	CRI Medi Clinic		Plot 80A Second Avenue, Along Dr. Peter Odili Street, P/Harcourt	Port Harcourt	Rivers
7148	RV/0351	RV/0351/S/5	CRI Medi Clinic		Plot 80A Second Avenue, Along Dr. Peter Odili Street, P/Harcourt	Port Harcourt	Rivers

7149	RV/0351	RV/0351/S/4	CRI Medi Clinic		Plot 80A Second Avenue, Along Dr. Peter Odili Street, P/Harcourt	Port Harcourt	Rivers
7150	RV/0351	RV/0351/S/1	CRI Medi Clinic		Plot 80A Second Avenue, Along Dr. Peter Odili Street, P/Harcourt	Port Harcourt	Rivers
7151	RV/0353	RV/0353/S/1	Bethsaida Clinic & Maternity		21, Pipeline Satelite Town, Oyibo, Rivers State	Oyigbo	Rivers
7152	RV/0356	RV/0356/S/5	Life Care Medical Centre		3 Azikiwe street Mile 11 Diobu P/ Harcourt	Port Harcourt	Rivers
7153	RV/0356	RV/0356/S/4	Life Care Medical Centre		3 Azikiwe street Mile 11 Diobu P/ Harcourt	Port Harcourt	Rivers
7154	RV/0356	RV/0356/S/7	Life Care Medical Centre		3 Azikiwe street Mile 11 Diobu P/ Harcourt	Port Harcourt	Rivers
7155	RV/0356	RV/0356/S/13	Life Care Medical Centre		3 Azikiwe street Mile 11 Diobu P/ Harcourt	Port Harcourt	Rivers
7156	RV/0356	RV/0356/S/3	Life Care Medical Centre		3 Azikiwe street Mile 11 Diobu P/ Harcourt	Port Harcourt	Rivers
7157	RV/0356	RV/0356/S/1	Life Care Medical Centre		3 Azikiwe street Mile 11 Diobu P/ Harcourt	Port Harcourt	Rivers
7158	RV/0357	RV/0357/S/5	Life Clinics Medical Centre		15 Uyo street, Mile 1 Diobu P/ Harcourt	Port Harcourt	Rivers
7159	RV/0357	RV/0357/S/1	Life Clinics Medical Centre		15 Uyo street, Mile 1 Diobu P/ Harcourt	Port Harcourt	Rivers
7160	RV/0357	RV/0357/S/14	Life Clinics Medical Centre		15 Uyo street, Mile 1 Diobu P/ Harcourt	Port Harcourt	Rivers
7161	RV/0357	RV/0357/S/13	Life Clinics Medical Centre		15 Uyo street, Mile 1 Diobu P/ Harcourt	Port Harcourt	Rivers
7162	RV/0359	RV/0359/S/1	Rehoboth Specialist Hospital		2 Winners'''' way off Afam str,D-line P/Harcourt	Port Harcourt	Rivers
7163	RV/0359	RV/0359/S/2	Rehoboth Specialist Hospital		2 Winners'''' way off Afam str,D-line P/Harcourt	Port Harcourt	Rivers
7164	RV/0359	RV/0359/S/10	Rehoboth Specialist Hospital		2 Winners'''' way off Afam str,D-line P/Harcourt	Port Harcourt	Rivers
7165	RV/0359	RV/0359/S/4	Rehoboth Specialist Hospital		2 Winners'''' way off Afam str,D-line P/Harcourt	Port Harcourt	Rivers
7166	RV/0359	RV/0359/S/5	Rehoboth Specialist Hospital		2 Winners'''' way off Afam str,D-line P/Harcourt	Port Harcourt	Rivers
7167	RV/0359	RV/0359/S/3	Rehoboth Specialist Hospital		2 Winners'''' way off Afam str,D-line P/Harcourt	Port Harcourt	Rivers
7168	RV/0361	RV/0361/S/3	TRUVINE SPECIALIST CLINIC		6 OHIAMINI RD, BEHIND COLL OF ARTS & SCI OFF R/OLA P/HARCOURT	Obio/Akpor	Rivers
7169	RV/0361	RV/0361/S/10	TRUVINE SPECIALIST CLINIC		6 OHIAMINI RD, BEHIND COLL OF ARTS & SCI OFF R/OLA P/HARCOURT	Obio/Akpor	Rivers
7170	RV/0361	RV/0361/S/1	TRUVINE SPECIALIST CLINIC		6 OHIAMINI RD, BEHIND COLL OF ARTS & SCI OFF R/OLA P/HARCOURT	Obio/Akpor	Rivers
7171	RV/0363	RV/0363/S/8	FEDERAL STAFF DENTAL CLINIC		FEDERAL SECRETARIAT, ABA RD,P/HARCURT	Obio/Akpor	Rivers
7172	RV/0366	RV/0366/S/5	OGO DIAGNOSTIC CENTRE	Laboratory	16 AHOADA RD, OMOKU	Ogba/Egbema/Ndoni	Rivers
7173	RV/0371	RV/0371/S/4	Phamarama Pharmacy & Store	Pharmacy	48 Force Avenue, Old GRA, Port Harcourt	Port Harcourt	Rivers
7174	RV/0373	RV/0373/S/4	Carelink Pharmacy		54 Abuloma Road, Trans-Amadi	Port Harcourt	Rivers
7175	RV/0374	RV/0374/S/8	Esteem Dental Clinic	Dental	51 Isiokpo Street Off Aguadama Street D/Line Port Harcourt	Port Harcourt	Rivers
7176	RV/0375	RV/0375/S/7	AB Health Consortium	Laboratory, Radiology	Nzimiro Street, Amadi Flats, Port Harcourt	Port Harcourt	Rivers
7177	RV/0375	RV/0375/S/5	AB Health Consortium	Laboratory, Radiology	Nzimiro Street, Amadi Flats, Port Harcourt	Port Harcourt	Rivers
7178	RV/0378	RV/0378/S/3	The Shield Clinics		Plot 4 Close D, Peace Estate, Trans Woji Road, Port Harcourt	Obio/Akpor	Rivers
7179	RV/0380	RV/0380/S/8	First Gate Dental Clinic		4a Barley Street, Old G.R.A, Port Harcourt Rivers State	Port Harcourt	Rivers
7180	RV/0381	RV/0381/S/6	Adanta Children`s Hospital		44 Evo Road GRA Phase2, Port Harcourt Rivers State	Port Harcourt	Rivers
7181	RV/0388	RV/0388/S/5	Life Line Clinic		30 Rumunduru RD, Eliowhani Kingdom ,Port Harcourt Rivers State	Obio/Akpor	Rivers
7182	RV/0390	RV/0390/S/3	Noble Medical Consultants &Fertility Hospital		No.1 Bloom breed School Road Mgbuoba Opp. NTA Gate Rivers State	Obio/Akpor	Rivers

7183	RV/0392	RV/0392/S/2	Kendox Medical Services		No 7,Moses Chinda Avenue, Off 194 Elelenwo Port Harcourt Rivers State	Obio/Akpor	Rivers
7184	RV/0392	RV/0392/S/5	Kendox Medical Services		No 7,Moses Chinda Avenue, Off 194 Elelenwo Port Harcourt Rivers State	Obio/Akpor	Rivers
7185	RV/0392	RV/0392/S/6	Kendox Medical Services		No 7,Moses Chinda Avenue, Off 194 Elelenwo Port Harcourt Rivers State	Obio/Akpor	Rivers
7186	RV/0392	RV/0392/S/1	Kendox Medical Services		No 7,Moses Chinda Avenue, Off 194 Elelenwo Port Harcourt Rivers State	Obio/Akpor	Rivers
7187	RV/0392	RV/0392/S/3	Kendox Medical Services		No 7,Moses Chinda Avenue, Off 194 Elelenwo Port Harcourt Rivers State	Obio/Akpor	Rivers
7188	RV/0395	RV/0395/S/3	Jose Vital Hospital Limited		Plot 19,East West RD, By Rumuokoro Near St. Jude Catholic Church, Port Harcourt	Obio/Akpor	Rivers
7189	RV/0395	RV/0395/S/1	Jose Vital Hospital Limited		Plot 19,East West RD, By Rumuokoro Near St. Jude Catholic Church, Port Harcourt	Obio/Akpor	Rivers
7190	RV/0398	RV/0398/S/3	Danferd Specialist Clinic		246,Ada George Road, Opposite Romans Filling Station By Okuton Junction River State	Obio/Akpor	Rivers
7191	RV/0402	RV/0402/S/2	Atlantic Medical Center		76c Emekuku Street/Line, Port Harcourt, Rivers State	Port Harcourt	Rivers
7192	RV/0402	RV/0402/S/3	Atlantic Medical Center		76c Emekuku Street/Line, Port Harcourt, Rivers State	Port Harcourt	Rivers
7193	RV/0402	RV/0402/S/7	Atlantic Medical Center		76c Emekuku Street/Line, Port Harcourt, Rivers State	Port Harcourt	Rivers
7194	RV/0402	RV/0402/S/1	Atlantic Medical Center		76c Emekuku Street/Line, Port Harcourt, Rivers State	Port Harcourt	Rivers
7195	RV/0403	RV/0403/S/1	Spring Rose Hospital		No.22 Rumuagbola Road Port Harcourt River State	Obio/Akpor	Rivers
7196	RV/0403	RV/0403/S/5	Spring Rose Hospital		No.22 Rumuagbola Road Port Harcourt River State	Obio/Akpor	Rivers
7197	RV/0403	RV/0403/S/2	Spring Rose Hospital		No.22 Rumuagbola Road Port Harcourt River State	Obio/Akpor	Rivers
7198	RV/0403	RV/0403/S/4	Spring Rose Hospital		No.22 Rumuagbola Road Port Harcourt River State	Obio/Akpor	Rivers
7199	RV/0403	RV/0403/S/6	Spring Rose Hospital		No.22 Rumuagbola Road Port Harcourt River State	Obio/Akpor	Rivers
7200	RV/0403	RV/0403/S/3	Spring Rose Hospital		No.22 Rumuagbola Road Port Harcourt River State	Obio/Akpor	Rivers
7201	RV/0404	RV/0404/S/3	El-Joe Dam Hospital		Off Shell Location Road, Mgbuoba, By Four Square Gospel Church Port Harcourt Rivers State	Obio/Akpor	Rivers
7202	RV/0407	RV/0407/S/1	Life Forte Specialist Hospital		3,Oluwosu Street, Off Ude Road Rumuollumeni, Port Harcourt Rivers State	Obio/Akpor	Rivers
7203	RV/0408	RV/0408/S/2	Hilton Clinics		No 2 Ejekwu Willie Close, Opp. Silver Spoon Hotel Ada George By Wimpey Road, Mile 4,Port Harcourt Rivers State	Obio/Akpor	Rivers
7204	RV/0409	RV/0409/S/5	Pearl Clinics & Maternity		4,Manilla Pepple Street, D/Line, Port Harcourt River State	Obio/Akpor	Rivers
7205	RV/0409	RV/0409/S/3	Pearl Clinics & Maternity		4,Manilla Pepple Street, D/Line, Port Harcourt River State	Obio/Akpor	Rivers
7206	RV/0409	RV/0409/S/1	Pearl Clinics & Maternity		4,Manilla Pepple Street, D/Line, Port Harcourt River State	Obio/Akpor	Rivers
7207	RV/0409	RV/0409/S/2	Pearl Clinics & Maternity		4,Manilla Pepple Street, D/Line, Port Harcourt River State	Obio/Akpor	Rivers
7208	RV/0414	RV/0414/S/3	Health Wise Hospital		No 3,Benin Street-Line Off 27 Aba Road By Oando Filling Station, Port Harcourt Rivers State	Port Harcourt	Rivers

7209	RV/0414	RV/0414/S/1	Health Wise Hospital		No 3,Benin Street-Line Off 27 Aba Road By Oando Filling Station, Port Harcourt Rivers State	Port Harcourt	Rivers
7210	RV/0414	RV/0414/S/14	Health Wise Hospital		No 3,Benin Street-Line Off 27 Aba Road By Oando Filling Station, Port Harcourt Rivers State	Port Harcourt	Rivers
7211	RV/0414	RV/0414/S/5	Health Wise Hospital		No 3,Benin Street-Line Off 27 Aba Road By Oando Filling Station, Port Harcourt Rivers State	Port Harcourt	Rivers
7212	RV/0416	RV/0416/S/4	AB Pharmaceuticals Nig Limited		48,Ada George Road, Port Harcourt Rivers State	Obio/Akpor	Rivers
7213	RV/0417	RV/0417/S/13	Lavinda Eye Specialist Hospital		No 1, Omubo Close Off Cambell Avenue(By Sumitel Hotel) Off Peter Odili Road Rivers State	Port Harcourt	Rivers
7214	RV/0419	RV/0419/S/13	Cynard Eye Clinic		No 14,Trans-Woji/Slaughter Rd, Woji, Port Harcourt River State	Obio/Akpor	Rivers
7215	RV/0422	RV/0422/S/19	Aggrey Clinic LTD (ANNEX)		3C Gorges Close, Nkpogu, Off Transamadi, Port Harcourt River State	Port Harcourt	Rivers
7216	RV/0423	RV/0423/S/3	Olivet Clinic		18 Ede Street, Off Ogbunabali Street Port Harcourt Rivers State	Obio/Akpor	Rivers
7217	RV/0423	RV/0423/S/2	Olivet Clinic		18 Ede Street, Off Ogbunabali Street Port Harcourt Rivers State	Obio/Akpor	Rivers
7218	RV/0425	RV/0425/S/13	Perfect Sight Eye Care Centre		80, Stadium Road Rumomasi Port Harcourt.	Obio/Akpor	Rivers
7219	RV/0426	RV/0426/S/13	Perfect Sight Eye Care Centre		80, Stadium Road Rumomasi Port Harcourt.	Obio/Akpor	Rivers
7220	RV/0428	RV/0428/S/2	Military Hospital Port-Harcourt		Aba Road Port-Harcourt, Rivers state	Obio/Akpor	Rivers
7221	RV/0428	RV/0428/S/11	Military Hospital Port-Harcourt		Aba Road Port-Harcourt, Rivers state	Obio/Akpor	Rivers
7222	RV/0428	RV/0428/S/5	Military Hospital Port-Harcourt		Aba Road Port-Harcourt, Rivers state	Obio/Akpor	Rivers
7223	RV/0428	RV/0428/S/8	Military Hospital Port-Harcourt		Aba Road Port-Harcourt, Rivers state	Obio/Akpor	Rivers
7224	RV/0428	RV/0428/S/3	Military Hospital Port-Harcourt		Aba Road Port-Harcourt, Rivers state	Obio/Akpor	Rivers
7225	RV/0428	RV/0428/S/7	Military Hospital Port-Harcourt		Aba Road Port-Harcourt, Rivers state	Obio/Akpor	Rivers
7226	RV/0428	RV/0428/S/4	Military Hospital Port-Harcourt		Aba Road Port-Harcourt, Rivers state	Obio/Akpor	Rivers
7227	RV/0428	RV/0428/S/6	Military Hospital Port-Harcourt		Aba Road Port-Harcourt, Rivers state	Obio/Akpor	Rivers
7228	RV/0428	RV/0428/S/1	Military Hospital Port-Harcourt		Aba Road Port-Harcourt, Rivers state	Obio/Akpor	Rivers
7229	RV/0430	RV/0430/S/2	Cova Care Ltd		8, Main Street, Off Farm Road 2, Off G.U Ake Road, Eliozu. Rivers State	Obio/Akpor	Rivers
7230	RV/0431	RV/0431/S/1	Sophike Medical Centre		Plot 9, Road 7, Federal Housing Estate, Off Peter Odili Road, Ports-Harcourt. Rivers State	Obio/Akpor	Rivers
7231	RV/0431	RV/0431/S/6	Sophike Medical Centre		Plot 9, Road 7, Federal Housing Estate, Off Peter Odili Road, Ports-Harcourt. Rivers State	Obio/Akpor	Rivers
7232	RV/0431	RV/0431/S/2	Sophike Medical Centre		Plot 9, Road 7, Federal Housing Estate, Off Peter Odili Road, Ports-Harcourt. Rivers State	Obio/Akpor	Rivers
7233	RV/0431	RV/0431/S/3	Sophike Medical Centre		Plot 9, Road 7, Federal Housing Estate, Off Peter Odili Road, Ports-Harcourt. Rivers State	Obio/Akpor	Rivers
7234	RV/0431	RV/0431/S/14	Sophike Medical Centre		Plot 9, Road 7, Federal Housing Estate, Off Peter Odili Road, Ports-Harcourt. Rivers State	Obio/Akpor	Rivers
7235	RV/0431	RV/0431/S/5	Sophike Medical Centre		Plot 9, Road 7, Federal Housing Estate, Off Peter Odili Road, Ports-Harcourt. Rivers State	Obio/Akpor	Rivers
7236	RV/0432	RV/0432/S/13	Vision Care Eye Clinic		Plot 131/132 Stadium Road, Rumuomasi, Port- Harcourt. Rivers State	Obio/Akpor	Rivers
7237	RV/0434	RV/0434/S/1	Ell-Specialist Hospitals Limited		7, Umuechem Street, D/Line, Port-Harcourt. Rivers State	Obio/Akpor	Rivers

7238	RV/0439	RV/0439/S/2	Nimley Clinic		17, Churchill Road, Creek Road Extension, Port-Harcourt. Rivers State	Obio/Akpor	Rivers
7239	RV/0439	RV/0439/S/3	Nimley Clinic		17, Churchill Road, Creek Road Extension, Port-Harcourt. Rivers State	Obio/Akpor	Rivers
7240	RV/0439	RV/0439/S/1	Nimley Clinic		17, Churchill Road, Creek Road Extension, Port-Harcourt. Rivers State	Obio/Akpor	Rivers
7241	RV/0440	RV/0440/S/6	Bennod International Hospital Limited		28 Ada George Road, Port-Harcourt -. Rivers State	Obio/Akpor	Rivers
7242	RV/0440	RV/0440/S/3	Bennod International Hospital Limited		28 Ada George Road, Port-Harcourt -. Rivers State	Obio/Akpor	Rivers
7243	RV/0440	RV/0440/S/1	Bennod International Hospital Limited		28 Ada George Road, Port-Harcourt -. Rivers State	Obio/Akpor	Rivers
7244	RV/0440	RV/0440/S/2	Bennod International Hospital Limited		28 Ada George Road, Port-Harcourt -. Rivers State	Obio/Akpor	Rivers
7245	RV/0443	RV/0443/S/19	Magnum Nuvon Medical Centre Ltd		18, Canon Ndu Street, Elelenwo, Rivers State	Obio/Akpor	Rivers
7246	RV/0444	RV/0444/S/8	Smile Evolution Dental Care		4, Amadi Ama Road, Trans-Amadi, Port-Harcourt. Rivers State.	Obio/Akpor	Rivers
7247	RV/0445	RV/0445/S/13	Morning Star Eyecare & Optical Clinic		67, Rumuola Road, Opp Collage Of Arts & Science, Rumuola, Port- Harcourt. Rivers State	Obio/Akpor	Rivers
7248	RV/0449	RV/0449/S/19	Obani Clinic		77b Woji Road, GRA Phase 2, Port Harcourt, Rivers State	Obio/Akpor	Rivers
7249	RV/0450	RV/0450/S/15	The Ophthalmic Specialist Company Ltd		9, Evo Road, GRA Phase 2, Port-Harcourt, Rivers State	Obio/Akpor	Rivers
7250	RV/0451	RV/0451/S/2	Abundant Health Specialist Ltd		9, Aleruchi Close, Off No.5 Ada George Road, Port-Harcourt Rivers State	Obio/Akpor	Rivers
7251	RV/0452	RV/0452/S/3	Greencare Medical Consultants Ltd		15, East West Worlu Road (Baba), Off Obiwali Road, Rumigbo, Port-Harcourt , Rivers State	Obio/Akpor	Rivers
7252	RV/0452	RV/0452/S/1	Greencare Medical Consultants Ltd		15, East West Worlu Road (Baba), Off Obiwali Road, Rumigbo, Port-Harcourt , Rivers State	Obio/Akpor	Rivers
7253	RV/0453	RV/0453/S/3	Save A Life Mission Hospital		38,Uyo St. Off Stadium Road, Rumuomasi, Port-Harcourt, Rivers State	Obio/Akpor	Rivers
7254	RV/0453	RV/0453/S/5	Save A Life Mission Hospital		38,Uyo St. Off Stadium Road, Rumuomasi, Port-Harcourt, Rivers State	Obio/Akpor	Rivers
7255	RV/0453	RV/0453/S/4	Save A Life Mission Hospital		38,Uyo St. Off Stadium Road, Rumuomasi, Port-Harcourt, Rivers State	Obio/Akpor	Rivers
7256	RV/0453	RV/0453/S/1	Save A Life Mission Hospital		38,Uyo St. Off Stadium Road, Rumuomasi, Port-Harcourt, Rivers State	Obio/Akpor	Rivers
7257	RV/0453	RV/0453/S/2	Save A Life Mission Hospital		38,Uyo St. Off Stadium Road, Rumuomasi, Port-Harcourt, Rivers State	Obio/Akpor	Rivers
7258	RV/0453	RV/0453/S/6	Save A Life Mission Hospital		38,Uyo St. Off Stadium Road, Rumuomasi, Port-Harcourt, Rivers State	Obio/Akpor	Rivers
7259	RV/0453	RV/0453/S/7	Save A Life Mission Hospital		38,Uyo St. Off Stadium Road, Rumuomasi, Port-Harcourt, Rivers State	Obio/Akpor	Rivers
7260	RV/0453	RV/0453/S/13	Save A Life Mission Hospital		38,Uyo St. Off Stadium Road, Rumuomasi, Port-Harcourt, Rivers State	Obio/Akpor	Rivers
7261	RV/0453	RV/0453/S/14	Save A Life Mission Hospital		38,Uyo St. Off Stadium Road, Rumuomasi, Port-Harcourt, Rivers State	Obio/Akpor	Rivers
7262	RV/0453	RV/0453/S/15	Save A Life Mission Hospital		38,Uyo St. Off Stadium Road, Rumuomasi, Port-Harcourt, Rivers State	Obio/Akpor	Rivers
7263	RV/0455	RV/0455/S/13	Josphinns Eye Clinic		14,Kanna Street, D/Line, Port-Harcourt, Rivers State	Obio/Akpor	Rivers
7264	RV/0456	RV/0456/S/3	Ofure Specialist Clinic		Plot 65,Okporo Rd, Behind Eco bank, Off Artillery, Port-Harcourt, Rivers State	Obio/Akpor	Rivers
7265	RV/0456	RV/0456/S/2	Ofure Specialist Clinic		Plot 65,Okporo Rd, Behind Eco bank, Off Artillery, Port-Harcourt, Rivers State	Obio/Akpor	Rivers

7266	RV/0456	RV/0456/S/1	Ofure Specialist Clinic		Plot 65,Okporo Rd, Behind Eco bank, Off Artillery, Port-Harcourt, Rivers State	Obio/Akpor	Rivers
7267	RV/0456	RV/0456/S/6	Ofure Specialist Clinic		Plot 65,Okporo Rd, Behind Eco bank, Off Artillery, Port-Harcourt, Rivers State	Obio/Akpor	Rivers
7268	RV/0457	RV/0457/S/2	Westhill Medical Services Ltd		4,Wogu Street ,Off Omoku Rd, D/Line Port-Harcourt Rivers State	Obio/Akpor	Rivers
7269	RV/0461	RV/0461/S/13	Beracca Eye Clinic Ltd		9,Rumuogba Estate, Rumuogba, Port-Harcourt ,Rivers State	Obio/Akpor	Rivers
7270	RV/0462	RV/0462/S/1	Ultimate Specialist Clinic & Hospital		67,Royal Avenue, Off Odili/Okuru/Abuloma Link Road, Port-Harcourt, Rivers State	Port Harcourt	Rivers
7271	RV/0462	RV/0462/S/6	Ultimate Specialist Clinic & Hospital		67,Royal Avenue, Off Odili/Okuru/Abuloma Link Road, Port-Harcourt, Rivers State	Port Harcourt	Rivers
7272	RV/0462	RV/0462/S/19	Ultimate Specialist Clinic & Hospital		67,Royal Avenue, Off Odili/Okuru/Abuloma Link Road, Port-Harcourt, Rivers State	Port Harcourt	Rivers
7273	RV/0462	RV/0462/S/3	Ultimate Specialist Clinic & Hospital		67,Royal Avenue, Off Odili/Okuru/Abuloma Link Road, Port-Harcourt, Rivers State	Port Harcourt	Rivers
7274	RV/0463	RV/0463/S/1	Oak Endoscopy Centre		Plot 150, Rumuogba Estate, Port-Harcourt, Rivers State	Obio/Akpor	Rivers
7275	RV/0463	RV/0463/S/17	Oak Endoscopy Centre		Plot 150, Rumuogba Estate, Port-Harcourt, Rivers State	Obio/Akpor	Rivers
7276	RV/0464	RV/0464/S/5	International Trauma & Critical Care Centre Ltd		11, Emenike Street, Mile One Port-Harcourt, Rivers State	Port Harcourt	Rivers
7277	RV/0464	RV/0464/S/4	International Trauma & Critical Care Centre Ltd		11, Emenike Street, Mile One Port-Harcourt, Rivers State	Port Harcourt	Rivers
7278	RV/0464	RV/0464/S/6	International Trauma & Critical Care Centre Ltd		11, Emenike Street, Mile One Port-Harcourt, Rivers State	Port Harcourt	Rivers
7279	RV/0464	RV/0464/S/3	International Trauma & Critical Care Centre Ltd		11, Emenike Street, Mile One Port-Harcourt, Rivers State	Port Harcourt	Rivers
7280	RV/0464	RV/0464/S/10	International Trauma & Critical Care Centre Ltd		11, Emenike Street, Mile One Port-Harcourt, Rivers State	Port Harcourt	Rivers
7281	RV/0464	RV/0464/S/2	International Trauma & Critical Care Centre Ltd		11, Emenike Street, Mile One Port-Harcourt, Rivers State	Port Harcourt	Rivers
7282	RV/0464	RV/0464/S/1	International Trauma & Critical Care Centre Ltd		11, Emenike Street, Mile One Port-Harcourt, Rivers State	Port Harcourt	Rivers
7283	RV/0465	RV/0465/S/13	Vonnie Vision Eye Care Consultants Ltd		Close 8,Plot 2b Elekahia Housing Estate, Port-Harcourt, Rivers State	Port Harcourt	Rivers
7284	RV/0466	RV/0466/S/5	NNPC Medical Services		NNPC-Eleme/Moscow Road, Port-Harcourt Rivers State	Tai	Rivers
7285	RV/0466	RV/0466/S/4	NNPC Medical Services		NNPC-Eleme/Moscow Road, Port-Harcourt Rivers State	Tai	Rivers
7286	RV/0466	RV/0466/S/3	NNPC Medical Services		NNPC-Eleme/Moscow Road, Port-Harcourt Rivers State	Tai	Rivers
7287	RV/0466	RV/0466/S/2	NNPC Medical Services		NNPC-Eleme/Moscow Road, Port-Harcourt Rivers State	Tai	Rivers
7288	RV/0466	RV/0466/S/18	NNPC Medical Services		NNPC-Eleme/Moscow Road, Port-Harcourt Rivers State	Tai	Rivers
7289	RV/0466	RV/0466/S/8	NNPC Medical Services		NNPC-Eleme/Moscow Road, Port-Harcourt Rivers State	Tai	Rivers
7290	RV/0466	RV/0466/S/10	NNPC Medical Services		NNPC-Eleme/Moscow Road, Port-Harcourt Rivers State	Tai	Rivers
7291	RV/0466	RV/0466/S/1	NNPC Medical Services		NNPC-Eleme/Moscow Road, Port-Harcourt Rivers State	Tai	Rivers
7292	RV/0466	RV/0466/S/11	NNPC Medical Services		NNPC-Eleme/Moscow Road, Port-Harcourt Rivers State	Tai	Rivers
7293	RV/0466	RV/0466/S/13	NNPC Medical Services		NNPC-Eleme/Moscow Road, Port-Harcourt Rivers State	Tai	Rivers

7294	RV/0466	RV/0466/S/14	NNPC Medical Services		NNPC-Eleme/Moscow Road, Port-Harcourt Rivers State	Tai	Rivers
7295	RV/0466	RV/0466/S/9	NNPC Medical Services		NNPC-Eleme/Moscow Road, Port-Harcourt Rivers State	Tai	Rivers
7296	RV/0466	RV/0466/S/6	NNPC Medical Services		NNPC-Eleme/Moscow Road, Port-Harcourt Rivers State	Tai	Rivers
7297	SO/0001	SO/0001/S/9	Federal Neurophychiatric Hospital	Neuropsychiatry	Kware, Sokoto	Kware	Sokoto
7298	SO/0002	SO/0002/S/3	Sokoto Clinic		Sokoto	Sokoto South	Sokoto
7299	SO/0004	SO/0004/S/12	Shepherd Specialist Clinic		No. 32 Zaria Rd. Ung. Rogo Area	Sokoto South	Sokoto
7300	SO/0005	SO/0005/S/3	Sahel Specialist Hospital		Sultan Abubakar Rd. Sokoto	Sokoto South	Sokoto
7301	SO/0006	SO/0006/S/3	Specialist Hospital		Abdullahi Fodiyo Road, Sokoto	Sokoto South	Sokoto
7302	SO/0006	SO/0006/S/15	Specialist Hospital		Abdullahi Fodiyo Road, Sokoto	Sokoto South	Sokoto
7303	SO/0006	SO/0006/S/8	Specialist Hospital		Abdullahi Fodiyo Road, Sokoto	Sokoto South	Sokoto
7304	SO/0006	SO/0006/S/1	Specialist Hospital		Abdullahi Fodiyo Road, Sokoto	Sokoto South	Sokoto
7305	SO/0006	SO/0006/S/12	Specialist Hospital		Abdullahi Fodiyo Road, Sokoto	Sokoto South	Sokoto
7306	SO/0006	SO/0006/S/5	Specialist Hospital		Abdullahi Fodiyo Road, Sokoto	Sokoto South	Sokoto
7307	SO/0006	SO/0006/S/2	Specialist Hospital		Abdullahi Fodiyo Road, Sokoto	Sokoto South	Sokoto
7308	SO/0006	SO/0006/S/4	Specialist Hospital		Abdullahi Fodiyo Road, Sokoto	Sokoto South	Sokoto
7309	SO/0009	SO/0009/S/5	Karaye Hospital		Ali Akilu Rd. Sokoto	Sokoto South	Sokoto
7310	SO/0010	SO/0010/S/3	Usman Danfodio University Teaching Hospital		Behind Standard Trust Bank, Kano Rd. Sokoto	Sokoto South	Sokoto
7311	SO/0010	SO/0010/S/11	Usman Danfodio University Teaching Hospital		Behind Standard Trust Bank, Kano Rd. Sokoto	Sokoto South	Sokoto
7312	SO/0010	SO/0010/S/8	Usman Danfodio University Teaching Hospital		Behind Standard Trust Bank, Kano Rd. Sokoto	Sokoto South	Sokoto
7313	SO/0010	SO/0010/S/5	Usman Danfodio University Teaching Hospital		Behind Standard Trust Bank, Kano Rd. Sokoto	Sokoto South	Sokoto
7314	SO/0010	SO/0010/S/4	Usman Danfodio University Teaching Hospital		Behind Standard Trust Bank, Kano Rd. Sokoto	Sokoto South	Sokoto
7315	SO/0010	SO/0010/S/7	Usman Danfodio University Teaching Hospital		Behind Standard Trust Bank, Kano Rd. Sokoto	Sokoto South	Sokoto
7316	SO/0010	SO/0010/S/12	Usman Danfodio University Teaching Hospital		Behind Standard Trust Bank, Kano Rd. Sokoto	Sokoto South	Sokoto
7317	SO/0010	SO/0010/S/15	Usman Danfodio University Teaching Hospital		Behind Standard Trust Bank, Kano Rd. Sokoto	Sokoto South	Sokoto
7318	SO/0010	SO/0010/S/6	Usman Danfodio University Teaching Hospital		Behind Standard Trust Bank, Kano Rd. Sokoto	Sokoto South	Sokoto
7319	SO/0010	SO/0010/S/2	Usman Danfodio University Teaching Hospital		Behind Standard Trust Bank, Kano Rd. Sokoto	Sokoto South	Sokoto
7320	SO/0010	SO/0010/S/1	Usman Danfodio University Teaching Hospital		Behind Standard Trust Bank, Kano Rd. Sokoto	Sokoto South	Sokoto
7321	SO/0011	SO/0011/S/3	Al-Fijir Specialist Medical Center	-	No. 32 Zaria Road, Unguwan Rogo Area	Sokoto South	Sokoto
7322	SO/0013	SO/0013/S/4	Primecare Pharmaceuticals	Pharmacy	15A, Kano Road, Sokoto	Sokoto South	Sokoto
7323	SO/0018	SO/0018/S/4	French-Afrique Pharmacy	Pharmacy	Plot 3 Western By Pass RD Sokoto	Wamakko	Sokoto
7324	SO/0019	SO/0019/S/4	Inland Pharmacy	Pharmacy	10, Maiduguri Road	Sokoto South	Sokoto
7325	SO/0020	SO/0020/S/4	Binji Pharmacy	Pharmacy	Gwiwa Low Cost Housing, Kaduna Rd.	Wamakko	Sokoto
7326	SO/0021	SO/0021/S/4	Freedom Pharmacy	Pharmacy	29, Sultan Abubakar Rd.	Sokoto South	Sokoto
7327	SO/0022	SO/0022/S/4	Wisdom Kings Pharm.	Pharmacy	No. 114 Ahmadu Bello Way	Sokoto South	Sokoto
7328	SO/0023	SO/0023/S/4	Freedom Pharmacy	Pharamcy	No. 9 Kano Rd	Sokoto South	Sokoto
7329	SO/0024	SO/0024/S/4	Passmark Gold Pharmacy	Pharmacy	No. 36 Aliyu Jodi Rd.	Sokoto South	Sokoto
7330	SO/0025	SO/0025/S/4	Kaycee Pharmacy	Pharmacy	16, Aliyu Jodi Rd.	Sokoto South	Sokoto
7331	SO/0026	SO/0026/S/4	Maigobir Pharamcy	Pharmacy	No. 29 Alh. Halima Estate, Kilgoria Rd	Sokoto South	Sokoto
7332	SO/0028	SO/0028/S/3	Sokoto Clinic	O & G, Gen. Medicine	Sokoto	Sokoto North	Sokoto
7333	SO/0033	SO/0033/S/1	Free Hand Specialist Hospital		31, Shuni Rd, Sokoto	Sokoto North	Sokoto

7334	SO/0072	SO/0072/S/3	An-Nisha Clinic		2, Garba Mohammed Rd, opp School of Works, Runjin Sabo, Sokoto	Sokoto North	Sokoto
7335	SO/0073	SO/0073/S/3	Ubandoma Soecialist Clinic		# 24, Besse Road, Rujin Sambo Area, Sokoto	Sokoto North	Sokoto
7336	SO/0075	SO/0075/S/15	State Specialist Hospital		Sultan Abubakar Road, Sokoto, Sokoto State	Sokoto South	Sokoto
7337	SO/0075	SO/0075/S/6	State Specialist Hospital		Sultan Abubakar Road, Sokoto, Sokoto State	Sokoto South	Sokoto
7338	SO/0075	SO/0075/S/5	State Specialist Hospital		Sultan Abubakar Road, Sokoto, Sokoto State	Sokoto South	Sokoto
7339	SO/0075	SO/0075/S/2	State Specialist Hospital		Sultan Abubakar Road, Sokoto, Sokoto State	Sokoto South	Sokoto
7340	SO/0075	SO/0075/S/12	State Specialist Hospital		Sultan Abubakar Road, Sokoto, Sokoto State	Sokoto South	Sokoto
7341	SO/0075	SO/0075/S/8	State Specialist Hospital		Sultan Abubakar Road, Sokoto, Sokoto State	Sokoto South	Sokoto
7342	SO/0075	SO/0075/S/7	State Specialist Hospital		Sultan Abubakar Road, Sokoto, Sokoto State	Sokoto South	Sokoto
7343	SO/0075	SO/0075/S/3	State Specialist Hospital		Sultan Abubakar Road, Sokoto, Sokoto State	Sokoto South	Sokoto
7344	SO/0075	SO/0075/S/1	State Specialist Hospital		Sultan Abubakar Road, Sokoto, Sokoto State	Sokoto South	Sokoto
7345	SO/0075	SO/0075/S/4	State Specialist Hospital		Sultan Abubakar Road, Sokoto, Sokoto State	Sokoto South	Sokoto
7346	SO/0076	SO/0076/S/10	Bone And Joint Orthopedic Hospital		2 Besse Road Rijin Sambo, Sokoto State.	Sokoto North	Sokoto
7347	SO/0077	SO/0077/S/3	Zefafi Women Specialist Hospital		No. 1 Maikahon Karo Road Beside Giginya Stadium Sokoto, Sokoto State	Sokoto South	Sokoto
7348	SO/0080	SO/0080/S/6	Caliphate Multispecialty Hospital		No. 2 Nagwamatse Road, Sokoto, Sokoto State.	Sokoto South	Sokoto
7349	SO/0080	SO/0080/S/3	Caliphate Multispecialty Hospital		No. 2 Nagwamatse Road, Sokoto, Sokoto State.	Sokoto South	Sokoto
7350	SO/0080	SO/0080/S/1	Caliphate Multispecialty Hospital		No. 2 Nagwamatse Road, Sokoto, Sokoto State.	Sokoto South	Sokoto
7351	SO/0080	SO/0080/S/14	Caliphate Multispecialty Hospital		No. 2 Nagwamatse Road, Sokoto, Sokoto State.	Sokoto South	Sokoto
7352	SO/0080	SO/0080/S/2	Caliphate Multispecialty Hospital		No. 2 Nagwamatse Road, Sokoto, Sokoto State.	Sokoto South	Sokoto
7353	SO/0080	SO/0080/S/7	Caliphate Multispecialty Hospital		No. 2 Nagwamatse Road, Sokoto, Sokoto State.	Sokoto South	Sokoto
7354	SO/0081	SO/0081/S/7	Medistop Clinical Diagnostic		No. 7 Dange Road Mabera, Sokoto, Sokoto State	Sokoto South	Sokoto
7355	SO/0081	SO/0081/S/5	Medistop Clinical Diagnostic		No. 7 Dange Road Mabera, Sokoto, Sokoto State	Sokoto South	Sokoto
7356	TR/0001	TR/0001/S/3	Jinya Medical Centre		3c Gombe Road, Jalingo	Jalingo	Taraba
7357	TR/0001	TR/0001/S/1	Jinya Medical Centre		3c Gombe Road, Jalingo	Jalingo	Taraba
7358	TR/0001	TR/0001/S/2	Jinya Medical Centre		3c Gombe Road, Jalingo	Jalingo	Taraba
7359	TR/0001	TR/0001/S/5	Jinya Medical Centre		3c Gombe Road, Jalingo	Jalingo	Taraba
7360	TR/0005	TR/0005/S/3	Gateway Hospital		No. 3c Gombi Road, Near State Low Cost, Jalingo	Jalingo	Taraba
7361	TR/0005	TR/0005/S/6	Gateway Hospital		No. 3c Gombi Road, Near State Low Cost, Jalingo	Jalingo	Taraba
7362	TR/0005	TR/0005/S/5	Gateway Hospital		No. 3c Gombi Road, Near State Low Cost, Jalingo	Jalingo	Taraba
7363	TR/0005	TR/0005/S/2	Gateway Hospital		No. 3c Gombi Road, Near State Low Cost, Jalingo	Jalingo	Taraba
7364	TR/0005	TR/0005/S/1	Gateway Hospital		No. 3c Gombi Road, Near State Low Cost, Jalingo	Jalingo	Taraba
7365	TR/0009	TR/0009/S/11	Federal Medical Centre - Jalingo		No. 29 Idris Makeri Street, Jalingo	Jalingo	Taraba
7366	TR/0009	TR/0009/S/2	Federal Medical Centre - Jalingo		No. 29 Idris Makeri Street, Jalingo	Jalingo	Taraba
7367	TR/0009	TR/0009/S/6	Federal Medical Centre - Jalingo		No. 29 Idris Makeri Street, Jalingo	Jalingo	Taraba
7368	TR/0009	TR/0009/S/9	Federal Medical Centre - Jalingo		No. 29 Idris Makeri Street, Jalingo	Jalingo	Taraba
7369	TR/0009	TR/0009/S/7	Federal Medical Centre - Jalingo		No. 29 Idris Makeri Street, Jalingo	Jalingo	Taraba
7370	TR/0009	TR/0009/S/3	Federal Medical Centre - Jalingo		No. 29 Idris Makeri Street, Jalingo	Jalingo	Taraba
7371	TR/0009	TR/0009/S/1	Federal Medical Centre - Jalingo		No. 29 Idris Makeri Street, Jalingo	Jalingo	Taraba
7372	TR/0009	TR/0009/S/15	Federal Medical Centre - Jalingo		No. 29 Idris Makeri Street, Jalingo	Jalingo	Taraba

7373	TR/0009	TR/0009/S/10	Federal Medical Centre - Jalingo		No. 29 Idris Makeri Street, Jalingo	Jalingo	Taraba
7374	TR/0009	TR/0009/S/4	Federal Medical Centre - Jalingo		No. 29 Idris Makeri Street, Jalingo	Jalingo	Taraba
7375	TR/0009	TR/0009/S/5	Federal Medical Centre - Jalingo		No. 29 Idris Makeri Street, Jalingo	Jalingo	Taraba
7376	TR/0013	TR/0013/S/5	General Hospital, Zing		Bitaka Road, Zing	Zing	Taraba
7377	TR/0013	TR/0013/S/1	General Hospital, Zing		Bitaka Road, Zing	Zing	Taraba
7378	TR/0013	TR/0013/S/4	General Hospital, Zing		Bitaka Road, Zing	Zing	Taraba
7379	TR/0028	TR/0028/S/5	Jonas clinics & Maternity		No 1, Old Garage Round-about, Takum	Takum	Taraba
7380	TR/0029	TR/0029/S/5	Niger Clinic		Opp water Board, Yakum	Takum	Taraba
7381	TR/0038	TR/0038/S/4	General Hospital, Gembu		Gembu, Taraba State	Sardauna	Taraba
7382	TR/0038	TR/0038/S/5	General Hospital, Gembu		Gembu, Taraba State	Sardauna	Taraba
7383	TR/0043	TR/0043/S/5	Kwararafa Hospital & Maternity		8, After Zion Church, Along Air Strip, Wukari	Wukari	Taraba
7384	TR/0044	TR/0044/S/14	Danny's Wellness Clinics Ltd		Presidential Lodge Area, Specialist Hospital Road, Jalingo	Jalingo	Taraba
7385	TR/0044	TR/0044/S/5	Danny's Wellness Clinics Ltd		Presidential Lodge Area, Specialist Hospital Road, Jalingo	Jalingo	Taraba
7386	TR/0045	TR/0045/S/2	Taraba Specialist Hospital		1, Jolly Nyame Way, Off By-Pass, Jalingo Taraba state	Jalingo	Taraba
7387	TR/0045	TR/0045/S/7	Taraba Specialist Hospital		1, Jolly Nyame Way, Off By-Pass, Jalingo Taraba state	Jalingo	Taraba
7388	TR/0045	TR/0045/S/10	Taraba Specialist Hospital		1, Jolly Nyame Way, Off By-Pass, Jalingo Taraba state	Jalingo	Taraba
7389	TR/0045	TR/0045/S/6	Taraba Specialist Hospital		1, Jolly Nyame Way, Off By-Pass, Jalingo Taraba state	Jalingo	Taraba
7390	TR/0045	TR/0045/S/4	Taraba Specialist Hospital		1, Jolly Nyame Way, Off By-Pass, Jalingo Taraba state	Jalingo	Taraba
7391	TR/0045	TR/0045/S/5	Taraba Specialist Hospital		1, Jolly Nyame Way, Off By-Pass, Jalingo Taraba state	Jalingo	Taraba
7392	TR/0045	TR/0045/S/14	Taraba Specialist Hospital		1, Jolly Nyame Way, Off By-Pass, Jalingo Taraba state	Jalingo	Taraba
7393	TR/0045	TR/0045/S/3	Taraba Specialist Hospital		1, Jolly Nyame Way, Off By-Pass, Jalingo Taraba state	Jalingo	Taraba
7394	TR/0045	TR/0045/S/1	Taraba Specialist Hospital		1, Jolly Nyame Way, Off By-Pass, Jalingo Taraba state	Jalingo	Taraba
7395	TR/0046	TR/0046/S/14	Sauki Hospital & Maternity		NO. 8, Near Zion Baptist Church Sabon Gari, Jalingo, Taraba State.	Jalingo	Taraba
7396	TR/0047	TR/0047/S/1	First Referral Hospital.		Serti Taraba State	Gashaka	Taraba
7397	TR/0048	TR/0048/S/4	University Health Services Centre		Federal University Wukari, Taraba State	Wukari	Taraba
7398	TR/0048	TR/0048/S/5	University Health Services Centre		Federal University Wukari, Taraba State	Wukari	Taraba
7399	TR/0049	TR/0049/S/3	First Nasarawo Hospital		Sintali B Jalingo	Jalingo	Taraba
7400	TR/0049	TR/0049/S/1	First Nasarawo Hospital		Sintali B Jalingo	Jalingo	Taraba
7401	TR/0050	TR/0050/S/14	Kwenas Clinic & Maternity		Near Old Garage, Wukari	Wukari	Taraba
7402	TR/0055	TR/0055/S/5	Waritoma - Rahila Hospital		Opp. Shagari Low Cost, Along Takum Road, Wukari Taraba State	Wukari	Taraba
7403	TR/0055	TR/0055/S/1	Waritoma - Rahila Hospital		Opp. Shagari Low Cost, Along Takum Road, Wukari Taraba State	Wukari	Taraba
7404	TR/0055	TR/0055/S/3	Waritoma - Rahila Hospital		Opp. Shagari Low Cost, Along Takum Road, Wukari Taraba State	Wukari	Taraba
7405	TR/0056	TR/0056/S/4	Goggoji Memorial Clinic		Behind Federal Science T. College Jalingo, Taraba State	Jalingo	Taraba
7406	TR/0056	TR/0056/S/5	Goggoji Memorial Clinic		Behind Federal Science T. College Jalingo, Taraba State	Jalingo	Taraba
7407	TR/0058	TR/0058/S/5	General Hospital Takum		Takum, Taraba State	Takum	Taraba
7408	TR/0058	TR/0058/S/4	General Hospital Takum		Takum, Taraba State	Takum	Taraba

7409	TR/0059	TR/0059/S/5	Zahra Clinic		AdjacentMastala Adamawa Street Jalingo, Taraba State.	Jalingo	Taraba
7410	TR/0059	TR/0059/S/4	Zahra Clinic		AdjacentMastala Adamawa Street Jalingo, Taraba State.	Jalingo	Taraba
7411	YB/0002	YB/0002/S/4	General Hospital, Gashua		Gashua,Along Nguru Rd., Gashua	Bade	Yobe
7412	YB/0002	YB/0002/S/5	General Hospital, Gashua		Gashua,Along Nguru Rd., Gashua	Bade	Yobe
7413	YB/0004	YB/0004/S/11	General Sani Abacha Specialist Hospital		Along Gujba Rd., Damaturu	Damaturu	Yobe
7414	YB/0004	YB/0004/S/2	General Sani Abacha Specialist Hospital		Along Gujba Rd., Damaturu	Damaturu	Yobe
7415	YB/0004	YB/0004/S/3	General Sani Abacha Specialist Hospital		Along Gujba Rd., Damaturu	Damaturu	Yobe
7416	YB/0004	YB/0004/S/8	General Sani Abacha Specialist Hospital		Along Gujba Rd., Damaturu	Damaturu	Yobe
7417	YB/0004	YB/0004/S/7	General Sani Abacha Specialist Hospital		Along Gujba Rd., Damaturu	Damaturu	Yobe
7418	YB/0004	YB/0004/S/15	General Sani Abacha Specialist Hospital		Along Gujba Rd., Damaturu	Damaturu	Yobe
7419	YB/0004	YB/0004/S/6	General Sani Abacha Specialist Hospital		Along Gujba Rd., Damaturu	Damaturu	Yobe
7420	YB/0004	YB/0004/S/1	General Sani Abacha Specialist Hospital		Along Gujba Rd., Damaturu	Damaturu	Yobe
7421	YB/0004	YB/0004/S/5	General Sani Abacha Specialist Hospital		Along Gujba Rd., Damaturu	Damaturu	Yobe
7422	YB/0004	YB/0004/S/4	General Sani Abacha Specialist Hospital		Along Gujba Rd., Damaturu	Damaturu	Yobe
7423	YB/0007	YB/0007/S/5	Dr(Mrs) Maryam Abacha Fsp		Gashua Rd., Damaturu	Damaturu	Yobe
7424	YB/0016	YB/0016/S/4	General Hospital Buni-Yadi		Along Biu Road, Buni-Yadi	Gujba	Yobe
7425	YB/0017	YB/0017/S/7	Federal Medical Center, Nguru		Along Gashua Rd.	Nguru	Yobe
7426	YB/0017	YB/0017/S/10	Federal Medical Center, Nguru		Along Gashua Rd.	Nguru	Yobe
7427	YB/0017	YB/0017/S/15	Federal Medical Center, Nguru		Along Gashua Rd.	Nguru	Yobe
7428	YB/0017	YB/0017/S/5	Federal Medical Center, Nguru		Along Gashua Rd.	Nguru	Yobe
7429	YB/0017	YB/0017/S/2	Federal Medical Center, Nguru		Along Gashua Rd.	Nguru	Yobe
7430	YB/0017	YB/0017/S/6	Federal Medical Center, Nguru		Along Gashua Rd.	Nguru	Yobe
7431	YB/0017	YB/0017/S/3	Federal Medical Center, Nguru		Along Gashua Rd.	Nguru	Yobe
7432	YB/0017	YB/0017/S/11	Federal Medical Center, Nguru		Along Gashua Rd.	Nguru	Yobe
7433	YB/0017	YB/0017/S/8	Federal Medical Center, Nguru		Along Gashua Rd.	Nguru	Yobe
7434	YB/0017	YB/0017/S/1	Federal Medical Center, Nguru		Along Gashua Rd.	Nguru	Yobe
7435	YB/0017	YB/0017/S/12	Federal Medical Center, Nguru		Along Gashua Rd.	Nguru	Yobe
7436	YB/0017	YB/0017/S/4	Federal Medical Center, Nguru		Along Gashua Rd.	Nguru	Yobe
7437	YB/0019	YB/0019/S/3	General Hospital Potiskum		Along Hospital Road	Potiskum	Yobe
7438	YB/0019	YB/0019/S/6	General Hospital Potiskum		Along Hospital Road	Potiskum	Yobe
7439	YB/0019	YB/0019/S/2	General Hospital Potiskum		Along Hospital Road	Potiskum	Yobe
7440	YB/0019	YB/0019/S/10	General Hospital Potiskum		Along Hospital Road	Potiskum	Yobe
7441	YB/0019	YB/0019/S/8	General Hospital Potiskum		Along Hospital Road	Potiskum	Yobe
7442	YB/0019	YB/0019/S/4	General Hospital Potiskum		Along Hospital Road	Potiskum	Yobe
7443	YB/0019	YB/0019/S/5	General Hospital Potiskum		Along Hospital Road	Potiskum	Yobe
7444	YB/0019	YB/0019/S/1	General Hospital Potiskum		Along Hospital Road	Potiskum	Yobe
7445	YB/0020	YB/0020/S/7	Ajiko Medical Center		Along Gashua Rd	Damaturu	Yobe
7446	YB/0020	YB/0020/S/4	Ajiko Medical Center		Along Gashua Rd	Damaturu	Yobe
7447	YB/0020	YB/0020/S/5	Ajiko Medical Center		Along Gashua Rd	Damaturu	Yobe
7448	YB/0021	YB/0021/S/4	General Hospital, Geidam		Kaigama Road, Geidam	Geidam	Yobe
7449	YB/0021	YB/0021/S/5	General Hospital, Geidam		Kaigama Road, Geidam	Geidam	Yobe
7450	YB/0022	YB/0022/S/14	Potiskum Medical Clinic		No. 166 Mohammed Idriss Way, Potiskum	Geidam	Yobe
7451	YB/0022	YB/0022/S/3	Potiskum Medical Clinic		No. 166 Mohammed Idriss Way, Potiskum	Geidam	Yobe
7452	YB/0022	YB/0022/S/1	Potiskum Medical Clinic		No. 166 Mohammed Idriss Way, Potiskum	Geidam	Yobe

7453	YB/0040	YB/0040/S/7	City Medical Centre		Zango Ward, Damaturu	Damaturu	Yobe
7454	YB/0040	YB/0040/S/5	City Medical Centre		Zango Ward, Damaturu	Damaturu	Yobe
7455	ZF/0003	ZF/0003/S/5	Freedom Diag. Lab. Centre	Laboratory	Kauran Namoda Road, T/Wada, Gusau	Gusau	Zamfara
7456	ZF/0005	ZF/0005/S/1	Gusau General Hospital - Gusau		Mortage Area, Gusau	Gusau	Zamfara
7457	ZF/0005	ZF/0005/S/6	Gusau General Hospital - Gusau		Mortage Area, Gusau	Gusau	Zamfara
7458	ZF/0005	ZF/0005/S/5	Gusau General Hospital - Gusau		Mortage Area, Gusau	Gusau	Zamfara
7459	ZF/0005	ZF/0005/S/4	Gusau General Hospital - Gusau		Mortage Area, Gusau	Gusau	Zamfara
7460	ZF/0005	ZF/0005/S/14	Gusau General Hospital - Gusau		Mortage Area, Gusau	Gusau	Zamfara
7461	ZF/0005	ZF/0005/S/3	Gusau General Hospital - Gusau		Mortage Area, Gusau	Gusau	Zamfara
7462	ZF/0007	ZF/0007/S/3	Women & Children Specialist Hospital		Gusau	Gusau	Zamfara
7463	ZF/0007	ZF/0007/S/14	Women & Children Specialist Hospital		Gusau	Gusau	Zamfara
7464	ZF/0007	ZF/0007/S/5	Women & Children Specialist Hospital		Gusau	Gusau	Zamfara
7465	ZF/0007	ZF/0007/S/4	Women & Children Specialist Hospital		Gusau	Gusau	Zamfara
7466	ZF/0007	ZF/0007/S/1	Women & Children Specialist Hospital		Gusau	Gusau	Zamfara
7467	ZF/0013	ZF/0013/S/4	Datura Pharmacy Ltd.,	Pharmacy	No.11 K/Namoda Road, Tudun Wada, Gusau	Gusau	Zamfara
7468	ZF/0014	ZF/0014/S/4	Vinna Pharmacy Ltd.	Pharmacy	43, K/Namoda Road, T/Wada, Gusau	Gusau	Zamfara
7469	ZF/0015	ZF/0015/S/4	Ernest Dof Pharmacy Ltd.	Pharmacy	Hospital Road-(PDP Hq House), Gusau	Gusau	Zamfara
7470	ZF/0016	ZF/0016/S/4	U.K. Pharmacy Ltd.	Pharmacy	Hospital Road, (PDP Hq House), Gusau	Gusau	Zamfara
7471	ZF/0021	ZF/0021/S/15	Federal Medical Centre Gusau		Gusau	Gusau	Zamfara
7472	ZF/0021	ZF/0021/S/8	Federal Medical Centre Gusau		Gusau	Gusau	Zamfara
7473	ZF/0021	ZF/0021/S/5	Federal Medical Centre Gusau		Gusau	Gusau	Zamfara
7474	ZF/0021	ZF/0021/S/2	Federal Medical Centre Gusau		Gusau	Gusau	Zamfara
7475	ZF/0021	ZF/0021/S/1	Federal Medical Centre Gusau		Gusau	Gusau	Zamfara
7476	ZF/0021	ZF/0021/S/7	Federal Medical Centre Gusau		Gusau	Gusau	Zamfara
7477	ZF/0021	ZF/0021/S/11	Federal Medical Centre Gusau		Gusau	Gusau	Zamfara
7478	ZF/0021	ZF/0021/S/4	Federal Medical Centre Gusau		Gusau	Gusau	Zamfara
7479	ZF/0021	ZF/0021/S/10	Federal Medical Centre Gusau		Gusau	Gusau	Zamfara
7480	ZF/0021	ZF/0021/S/12	Federal Medical Centre Gusau		Gusau	Gusau	Zamfara
7481	ZF/0021	ZF/0021/S/3	Federal Medical Centre Gusau		Gusau	Gusau	Zamfara
7482	ZF/0021	ZF/0021/S/6	Federal Medical Centre Gusau		Gusau	Gusau	Zamfara
7483	ZF/0022	ZF/0022/S/4	Rayad's Pharmacy Ltd.	Pharmacy	Off K/Namoda Road, Lebin-Lebin, Gusau	Gusau	Zamfara
7484	ZF/0029	ZF/0029/S/6	Yariman Bakura Specialist Hospital		Kawa Namoda Road, Tudun Wada, Gusau, Zamfara State	Gusau	Zamfara
7485	ZF/0029	ZF/0029/S/4	Yariman Bakura Specialist Hospital		Kawa Namoda Road, Tudun Wada, Gusau, Zamfara State	Gusau	Zamfara
7486	ZF/0029	ZF/0029/S/7	Yariman Bakura Specialist Hospital		Kawa Namoda Road, Tudun Wada, Gusau, Zamfara State	Gusau	Zamfara
7487	ZF/0029	ZF/0029/S/8	Yariman Bakura Specialist Hospital		Kawa Namoda Road, Tudun Wada, Gusau, Zamfara State	Gusau	Zamfara
7488	ZF/0029	ZF/0029/S/2	Yariman Bakura Specialist Hospital		Kawa Namoda Road, Tudun Wada, Gusau, Zamfara State	Gusau	Zamfara
7489	ZF/0029	ZF/0029/S/5	Yariman Bakura Specialist Hospital		Kawa Namoda Road, Tudun Wada, Gusau, Zamfara State	Gusau	Zamfara
7490	ZF/0029	ZF/0029/S/15	Yariman Bakura Specialist Hospital		Kawa Namoda Road, Tudun Wada, Gusau, Zamfara State	Gusau	Zamfara
7491	ZF/0029	ZF/0029/S/3	Yariman Bakura Specialist Hospital		Kawa Namoda Road, Tudun Wada, Gusau, Zamfara State	Gusau	Zamfara
7492	ZF/0029	ZF/0029/S/1	Yariman Bakura Specialist Hospital		Kawa Namoda Road, Tudun Wada, Gusau, Zamfara State	Gusau	Zamfara
