# Supplementary material for: Determinants and perception of health insurance participation among healthcare providers in Nigeria: A mixed-methods study
Source: PLoS One. 2021 Aug 4;16(8):e0255206. doi: 10.1371/journal.pone.0255206 (PMC8336839; doi:10.1371/journal.pone.0255206)
Supplement: S3 Appendix — (PDF) [file pone.0255206.s003.pdf]

Healthcare Provider Participation in Insurance Programs in Lagos State: Benefits, Challenges and Expectations

Preliminary information

Date of interview: (DD/MM/YY) (____/____/____)		Health facility code:
Name of interviewer:		
Time Spent	**Start time:** (HR/MIN) (_____/_____)	**End time:** (HR/MIN) (_____/_____)

Questionnaire Overview

This survey questionnaire is about healthcare providers' expectations, challenges and benefits of participating in insurance programs. The questionnaire contains two parts and five sections. Please feel free to discuss and share your ideas, opinions and experiences. All your responses will remain confidential.

PART A: PROVIDER SURVEY QUESTIONNAIRE

SECTION 1: FACILITY CHARACTERISTICS

1. Location
    1. Urban
    2. Peri-urban
    3. Rural

2. Ownership
    1. Mission/Faith-based
    2. NGO/Not-for-profit
    3. Private for-profit
        a. Company
        b. Individual
    4. Government/Public
    5. Others: Specify _____________

3. Level of health facility
    1. Primary
    2. Secondary
    3. Primary and Secondary
    4. Tertiary

4. Type of health facility
    1. Health Clinic/Post
    2. Nursing/Maternity Home
    3. General Hospital/Medical Centre
    4. Specialist Hospital
    5. Teaching Hospital
    6. Others: Specify _____________

5. Bed size
    1. No Beds
    2. Less than 10 beds
    3. 11 – 20 beds
    4. Greater than 20 beds

6. Number of years in operation
    1. Less than 1 year
    2. 1 – 5 years
    3. 5.1 – 10 years
    4. 10.1 – 20 years
    5. Greater than 20 years

7. Staffing/Human resource

Indicate number of staff available in health facility in 2016 disaggregated by full time and part time

Cadre	Full time	Part time	Total
Doctor			
Nurse			
Midwife			
Auxiliary nurse			
Community Health Worker			
Pharmacist			
Pharmacy technician			
Pharmacy assistant			
Laboratory technician			
Laboratory scientist			
Non-medical personnel			
Others			

8. Service Delivery Volume

Indicate volume of services below for 2015 and 2016

S/N	Service	2015	2016
a.	Total number of inpatient admissions		
b.	Total number of deliveries		
c.	Total number of outpatient visits		
d.	Total number of laboratory tests		

9. Insurance Participation
   a. Does health facility accept patients with health insurance?
      1. Yes
      2. No

If answer is No, please skip to Question 9f below. If Yes, continue with Question 9b

   b. If Yes to 9a above, what type of insurance, does health facility accept?
      1. National Health Insurance Scheme (NHIS)
      2. Community Based Health Insurance (CBHI)
      3. Private Health Insurance
      4. Others: Please specify________________

   c. How many years has health facility accepted patients from the following insurers?
      1. National Health Insurance Scheme, Number of years [ ]
      2. Community Based Health Insurance, Number of years [ ]
      3. Private Health Insurance, Number of years [ ]
      4. Others: Please specify______________, Number of years [ ]

   d. What proportion of total patients in 2016 paid via:
      1. National Health Insurance Scheme (NHIS) _________
      2. Community Based Health Insurance (CBHI) ________
      3. Private Health Insurance ________________________

4. Private Out-of-Pocket _________________________
5. Others: Please specify__________________________

e. At the end of 2016, how many HMOs were affiliated with the health facility? __________

f. If No to Question 9a above, did health facility accept patients with insurance in the past?
   1. Yes
   2. No

If Yes to Question 9f, continue with Questions 9g to 9i. If No, skip to Question 10

g. If Yes to Question 9f above, what type of health insurance did health facility accept in the past?
   1. National Health Insurance Scheme
   2. Community Based Health Insurance
   3. Private Health Insurance
   4. Others: Please specify___________

h. How many years did health facility consecutively accept insured patients in the following programs before stopping:
   1. National Health Insurance Scheme, Number of years [ ], End Date: __________
   2. Community Based Health Insurance, Number of years [ ], End Date: _________
   3. Private Health Insurance, Number of years [ ], End Date: __________________
   4. Others: Please specify___________, Number of years [ ], End Date: _________

i. Why was collaboration with insurance(s) discontinued?
   ____
   ____
   ____
   ____
   ____

PART B: PROVIDER INTERVIEW GUIDE

SECTION 2: HEALTH FACILITY MANAGER/ADMINISTRATOR INFORMATION

10. Profession of health administrator/facility manager
    1. Medical doctor
    2. Nurse
    3. Midwife
    4. Pharmacist
    5. Non-medical professional: Please specify______________

11. Highest qualification of health administrator/facility manager
    1. Bachelors
    2. Masters
    3. PhD
    4. Others: Please specify _________________________

12. Number of years spent as administrator/manager in health facility [_______years]

13. Gender of administrator/manager
    1. Male
    2. Female

14. Age of facility manager [______years]

SECTION 3: FACILITY FINANCIAL INFORMATION

15. Revenue

a. What was the total revenue for health facility in 2016?
   1. Less than 1 million Naira
   2. 1 – 5 million Naira
   3. 5.1 – 10 million Naira
   4. 10.1 – 25 million Naira
   5. Greater than 25 million Naira

b. What was the proportion of revenue due to insurance payments in 2016? _________________

c. By what proportion did insurance revenue grow/decline between 2015 and 2016? __________

d. Why did insurance revenue grow/decline between 2015 and 2016?

____

____

16. Expenditure

a. What was the total expenditure for health facility in 2016?
   1. Less than 1 million Naira
   2. 1 – 5 million Naira
   3. 5.1 – 10 million Naira
   4. 10.1 – 25 million Naira
   5. Greater than 25 million Naira

b. By what proportion did the expenditure grow/decline between 2015 and 2016? ____________

c. Why did the expenditure grow/decline between 2015 and 2016?

____

____

17. Profit ☐ Surplus ☐ Loss ☐ ***(Ask facility manager and check the one that applies)***

a. What was the total profit/surplus/loss for health facility in 2016?
   - Less than 1 million Naira
   - 1 – 5 million Naira
   - 5.1 – 10 million Naira
   - 10.1 – 25 million Naira
   - Greater than 25 million Naira

b. By what proportion did the profit/surplus/loss grow or decline between 2015 and 2016? _____

c. Why did the profit/surplus/loss grow or decline between 2015 and 2016?

____
____

SECTION 4: FACILITY INSURANCE PARTICIPATION EXPERIENCE

Please complete Questions 18 to 23 if health facility is accepting or has accepted patients with insurance in the past. If not, skip to question 24

18. What are/were the health facility benefits of participating in insurance?

____
____
____
____
____
____

Probe along the lines of patient volume, cash flow, revenue, profit (surplus), and opportunity to invest in facility/infrastructure upgrade

19. Can you rank the benefits of insurance participation below on order of importance to your health facility, with 1 being the most important?

	Rank
1. Increase in the volume of patients	______
2. Increased cash flow	______
3. Increased revenue	______
4. Increased profit (surplus)	______
5. Opportunity to invest in facility/infrastructure upgrade	______
6. Others ____	______

20. What are/were health facility challenges with participating in insurance?

____
____
____
____
____
____

Probe along the lines of reimbursement fees, paper work, claims processing, patient expectations, attitude of HMOs

21. Can you rank the challenges of insurance participation below on the order of importance to your health facility, with 1 being the most important

	Rank
1. Low reimbursement fees	_______
2. Amount of paper work	_______
3. Inappropriate denial of payment	_______
4. Speed of processing payment	_______
5. Unruly behaviour of patients	_______
6. Unrealistic expectations of benefits and services from patients	_______
7. Poor attitude of HMOs	_______
8. Others____	_______

22. Probe for the manager/administrator's perception on the following:

a. What do you think about the facility's ability to meet the cost of providing services from the capitation fee paid by:
   1. Government Insurance
      ____
      ____
      ____
   2. Private Insurance
      ____
      ____
      ____

b. What do you think about the facility's ability to meet the cost of providing services from the Fee-For-Service paid by:
   1. Government Insurance
      ____
      ____
      ____
   2. Private Insurance
      ____
      ____
      ____

c. For facilities accepting government insurance, what is the extent to which government's insurance has contributed to health facility's bottom line profit or surplus?
   ____
   ____
   ____

d. For facilities accepting private insurance, what is the extent to which private insurance has contributed to health facility's bottom line profit or surplus?
   ____
   ____
   ____

e. How does facility file for claims and reimbursed for services provided to insured patients?
____
____
____
____

f. Is the process in 22e above different for government and private insurance? If yes, how?
____
____
____

g. What is the timeliness of claims processing and reimbursement?
____
____
____

h. What is the type of workforce needed for a health facility participating in insurance, and why?
____
____
____
____
____

i. To what extent is the workforce needed to handle insurance work available and easy to recruit in the labour market?
____
____
____

j. What is the ability and capacity of the health facility to train workforce required to handle the insurance program?
____
____
____

k. Did you change staffing patterns or training as a result of joining an insurance plan?
   - Yes. How and why?
   ____
   ____
   ____
   - No. Why not?
   ____
   ____
   ____

l. What systematic changes did you make in the health facility to serve patients with insurance?
____
____

m. How would you describe the process by which referrals are approved, the timeliness and ease of referring patients to specialist or teaching hospitals?

n. Did you make any efforts to recruit insured patients? If yes, how did you do that?

o. How would you describe insured patients understanding of their benefit plan and expectations when they show up for care?

p. How would you describe insured patients' attitude to care and their level of utilization?

q. What type of accreditation does your health facility have now?

r. What is the process of getting accreditation as a health provider? How easy is it and is it a barrier to insurance participation?

s. How do you interact with HMOs? Do they make inspections or quality checks? What happens after such checks?

____
____
____

t. Did you change quality improvement procedures or clinical protocols as a result of joining an insurance plan?
   - Yes. How and why?
     ____
     ____
     ____
   - No. Why not?
     ____
     ____
     ____

u. How confident is health facility to meet the quality standard? Will it be too costly to achieve?
____
____
____
____
____

v. How does the health facility raise funds for improvements?
____
____
____
____
____

23. Please compare the following aspect of government's insurance with private insurance
(Only applicable to health facilities with experience with both private and government insurance)

**Aspects of government insurance**	**Compared to private insurance**			
a. Reimbursement	More	Less	Much Less	No Difference
b. Amount of paper work	More	Less	Much Less	No Difference
c. Speed of processing payment	More	Less	Much Less	No Difference
d. Inappropriate denial of payments	More	Less	Much Less	No Difference
e. Severity of health needs of patients	More	Less	Much Less	No Difference
f. Likelihood of patients not to show up for appointments	More	Less	Much Less	No Difference
g. Likelihood of non-compliance among patients	More	Less	Much Less	No Difference
h. Level of patients understanding of benefit package and expected services	More	Less	Much Less	No Difference
i. Level of patients utilization of services	More	Less	Much Less	No Difference
j. Desirability of patients	More	Less	Much Less	No Difference
k. Degree to which HMOs respond rapidly to referral requests	More	Less	Much Less	No Difference
l. Ease of accreditation	More	Less	Much Less	No Difference

SECTION 5: PROVIDER PERCEPTION OF LAGOS STATE HEALTH INSURANCE SCHEME

24. Is administrator/manager aware of the Lagos State Health Insurance Scheme's (LSHS) roll out?
    1. Yes
    2. No

25. Has health facility registered and accredited as a provider for the LSHS?
    1. Yes
    2. No

26. If Yes, why did the health facility register as a provider for the LSHS?
    ____
    ____
    ____
    ____

27. If No to Question 25, why has the health facility not registered as a provider for the LSHS?
    ____
    ____
    ____
    ____

28. If No to Question 25, does the health facility intend to register as a provider for the LSHS?
    1. Yes
    2. No

29. If Yes to Question 28, when? __________________________

30. If No to Question 28, why?
    ____
    ____
    ____
    ____

31. If No to Question 28, what would spur or attract management of health facility to register as a provider for the LSHS?
    ____
    ____
    ____
    ____

32. What opportunities does the manager think that LSHS could bring to the health facility?
    a. ____
    b. ____
    c. ____
    d. ____
    e. ____

33. What challenges does the manager foresee with the implementation of the LSHS?
    a. ____
    b. ____
    c. ____
    d. ____
    e. ____

34. If health facility experiences an increased volume of patients, what is the manager's perception of the facility's capacity to accommodate increased demand for services from insured patients? Would the facility need extra resources to participate?

    ____
    ____
    ____
    ____
    ____

35. Given the potential opportunities with accepting patients with government insurance, to what extent is the management of the health facility willing to invest in infrastructure upgrade?
    - Very willing
    - Willing
    - Somewhat willing
    - Not willing
    - Not very willing

36. If health facility is willing to invest in infrastructure upgrade, what part of the health facility system from the list below would be prioritized for investment, and why? Please rank from the highest to lowest priority, with 1 representing highest priority

	Rank
Medical Staff	_____
Non-medical staff	_____
Bed capacity	_____
Administrative infrastructure for claims	_____
Technology systems	_____
Drugs and commodities	_____
Referral system	_____

Why?

____
____
____
____
____

Where does health facility expect to get the funding for this investment?

____
____
____

37. Please carefully select based on how you think your facility ranks on current capacity and ability to obtain the capacity needed for an increased volume of patients:

	**Current Capacity**	**Ability to obtain capacity needed**
**Medical Staff**	Very adequate<br>Adequate<br>Somewhat adequate<br>Inadequate<br>Very inadequate	Very capable<br>Capable<br>Somewhat capable<br>Incapable<br>Very incapable
**Non-medical staff**	Very adequate<br>Adequate<br>Somewhat adequate<br>Inadequate<br>Very inadequate	Very capable<br>Capable<br>Somewhat capable<br>Incapable<br>Very incapable
**Bed capacity**	Very adequate<br>Adequate<br>Somewhat adequate<br>Inadequate<br>Very inadequate	Very capable<br>Capable<br>Somewhat capable<br>Incapable<br>Very incapable
**Administrative infrastructure**	Very adequate<br>Adequate<br>Somewhat adequate<br>Inadequate<br>Very inadequate	Very capable<br>Capable<br>Somewhat capable<br>Incapable<br>Very incapable
**Technology systems**	Very adequate<br>Adequate<br>Somewhat adequate<br>Inadequate<br>Very inadequate	Very capable<br>Capable<br>Somewhat capable<br>Incapable<br>Very incapable
**Availability of drugs and commodities**	Very adequate<br>Adequate<br>Somewhat adequate<br>Inadequate<br>Very inadequate	Very capable<br>Capable<br>Somewhat capable<br>Incapable<br>Very incapable
**Financial resources to invest in infrastructure upgrade if needed**	Very adequate<br>Adequate<br>Somewhat adequate<br>Inadequate<br>Very inadequate	Very capable<br>Capable<br>Somewhat capable<br>Incapable<br>Very incapable
**Referral system if primary health facility**	Very adequate<br>Adequate<br>Somewhat adequate<br>Inadequate<br>Very inadequate	Very capable<br>Capable<br>Somewhat capable<br>Incapable<br>Very incapable
**Quality improvement system**	Very adequate<br>Adequate<br>Somewhat adequate<br>Inadequate<br>Very inadequate	Very capable<br>Capable<br>Somewhat capable<br>Incapable<br>Very incapable

38. What type of support can the Lagos state government provide to address some of the inadequacies identified in Question 35?

____
____
____
____
____

39. What can the Lagos State Health Commission do to address some of the bottlenecks with government insurance?

Low reimbursement fees	
Amount of paper work	
Inappropriate denial of payment	
Speed of processing payment	
Unrealistic expectations of patients with government insurance	
Attitude of HMOs	
Accreditation of health facility	
Others	

40. Are there other issues relevant to the questions asked that you think we have not talked about but would like to discuss?

____
____
____
____
____
